# Whole-Transcriptome RNA Sequencing Reveals Significant Differentially Expressed mRNAs, miRNAs, and lncRNAs and Related Regulating Biological Pathways in the Peripheral Blood of COVID-19 Patients

**DOI:** 10.1155/2021/6635925

**Published:** 2021-04-01

**Authors:** Cai-xia Li, Jian Chen, Shu-kai Lv, Jin-hui Li, Lei-lei Li, Xiao Hu

**Affiliations:** ^1^The Fourth Affiliated Hospital Zhejiang University School of Medicine, Yiwu, Zhejiang, China; ^2^The First Affiliated Hospital Zhejiang University School of Medicine, Hangzhou, Zhejiang, China

## Abstract

Severe acute respiratory syndrome coronavirus 2 (SARS-CoV-2) was initially identified in China and currently worldwide dispersed, resulting in the coronavirus disease 2019 (COVID-19) pandemic. Notably, COVID-19 is characterized by systemic inflammation. However, the potential mechanisms of the “cytokine storm” of COVID-19 are still limited. In this study, fourteen peripheral blood samples from COVID-19 patients (*n* = 10) and healthy donors (*n* = 4) were collected to perform the whole-transcriptome sequencing. Lung tissues of COVID-19 patients (70%) presenting with ground-glass opacity. Also, the leukocytes and lymphocytes were significantly decreased in COVID-19 compared with the control group (*p* < 0.05). In total, 25,482 differentially expressed messenger RNAs (DE mRNA), 23 differentially expressed microRNAs (DE miRNA), and 410 differentially expressed long noncoding RNAs (DE lncRNAs) were identified in the COVID-19 samples compared to the healthy controls. Gene Ontology (GO) analysis showed that the upregulated DE mRNAs were mainly involved in antigen processing and presentation of endogenous antigen, positive regulation of T cell mediated cytotoxicity, and positive regulation of gamma-delta T cell activation. The downregulated DE mRNAs were mainly concentrated in the glycogen biosynthetic process. We also established the protein-protein interaction (PPI) networks of up/downregulated DE mRNAs and identified 4 modules. Functional enrichment analyses indicated that these module targets were associated with positive regulation of cytokine production, cytokine-mediated signaling pathway, leukocyte differentiation, and migration. A total of 6 hub genes were selected in the PPI module networks including AKT1, TNFRSF1B, FCGR2A, CXCL8, STAT3, and TLR2. Moreover, a competing endogenous RNA network showed the interactions between lncRNAs, mRNAs, and miRNAs. Our results highlight the potential pathogenesis of excessive cytokine production such as MSTRG.119845.30/hsa-miR-20a-5p/TNFRSF1B, MSTRG.119845.30/hsa-miR-29b-2-5p/FCGR2A, and MSTRG.106112.2/hsa-miR-6501-5p/STAT3 axis, which may also play an important role in the development of ground-glass opacity in COVID-19 patients. This study gives new insights into inflammation regulatory mechanisms of coding and noncoding RNAs in COVID-19, which may provide novel diagnostic biomarkers and therapeutic avenues for COVID-19 patients.

## 1. Introduction

Severe acute respiratory syndrome coronavirus 2 (SARS-CoV-2) is a novel lineage B betacoronavirus that causes zoonotic diseases and was first reported in Wuhan, China, in December 2019 [1, 2]. The pandemic caused by SARS-CoV-2 was named as coronavirus disease 2019 (COVID-19) by the World Health Organization (WHO) and rapidly spreads around the world [3]. As of February 20, 2021, a total of 111,234,365 coronaviruses confirmed cases and 2,462,703 deaths have been reported all around the world, the USA being on top so far [4].

Previous studies have reported that the COVID-19 patients suffered from asymptomatic or mild respiratory infection to acute respiratory distress syndrome (ARDS) or multiorgan failure, and this disease was more likely to occur in elderly people with a weak immune function [1, 5, 6]. Most patients have a cough, fever, shortness of breath, and fatigue [7]. Also, the majority of patients with COVID-19 have a pleasing prognosis, but there were still ARDS and multiorgan dysfunction that occurred rapidly, resulting in death within a short time [7, 8]. Therefore, understanding the genomic landscape of serum of COVID-19 patients will help in improving the treatment avenues and diagnosis strategies.

Clinically significantly, SARS-CoV-2 infection rapidly results in severe pneumonia symptoms and complications, and the host immune response against SARS-CoV-2 infection remains largely unknown. The viral RNAs are recognized by toll-like receptors (TLRs), NOD-like receptors (NLRs), and RIG-I-like receptors (RLRs), and the innate immune systems activating the T CD8+ cells, natural killer cells, and macrophages to eliminate the viruses [9, 10]. Unfortunately, the SARS-CoV and Middle East respiratory syndrome coronavirus (MERS-CoV) have evolved strategies to avoid immune responses, which usually trigger excessive inflammatory host responses [11]. It has been reported that the dysregulated production of inflammatory cytokines, known as the “cytokine storm,” in serum was associated with pulmonary inflammation and severe lung histopathology changes in SARS-Cov [12] and MERS-CoV infection [13]. Besides, it is noteworthy that the interstitial mononuclear inflammatory infiltrates in lung tissues were remarkably observed in the COVID-19 biopsy samples through clinical pathological analysis [14]. A study by Xiong et al. demonstrated that excessive cytokine release such as CCL2/MCP-1, CXCL10/IP-10, CCL3/MIP-1A, and CCL4/MIP1B is playing critical roles in COVID-19 pathogenesis [10]. However, the underlying mechanisms involved in the abnormal inflammatory responses under SARS-CoV-2 infection remain to be investigated. Hence, it is important to explore the aberrant expression of genes that are associated with the initiation and development of COVID-19.

Noncoding RNAs (ncRNAs) are a class of crucial regulatory potentials both in transcription and posttranscription and are divided into long ncRNAs (lncRNAs) and short ncRNAs [15, 16]. MicroRNAs (miRNAs), a kind of single-stranded RNA of 18~24 nucleotides in length, participate in messenger RNA (mRNAs) degradation or translational inhibition by binding to the 3′ UTR of their target sites [17]. LncRNAs are a special type of ncRNAs with lengths exceeding 200 nucleotides and are also divided into exonic, intronic, intergenic, and overlapping lncRNA based on its locations relative to the corresponding transcripts [18]. A competing endogenous RNA (ceRNA) hypothesis has been firstly proposed by Salmena et al. in 2011 [19] that the lncRNAs can function as a miRNA sponge by complementary base pairing with targeted miRNA using miRNA response elements (MREs) and thus participated in the development and progression of diseases. For the ceRNA patterns, miRNAs also act as the critical communication bridges between the coding and noncoding RNAs. Moreover, recent reports have summarized that miRNAs and lncRNAs have diagnostic and therapeutic potential in inflammatory diseases [20] and infectious diseases [21, 22]. Therefore, it is essential to further investigate the network regulatory crosstalk across coding and noncoding RNAs induced by miRNAs, which may contribute to discovering the promising drug targets for COVID-19 treatment from a holistic attitude.

**Figure 1:**
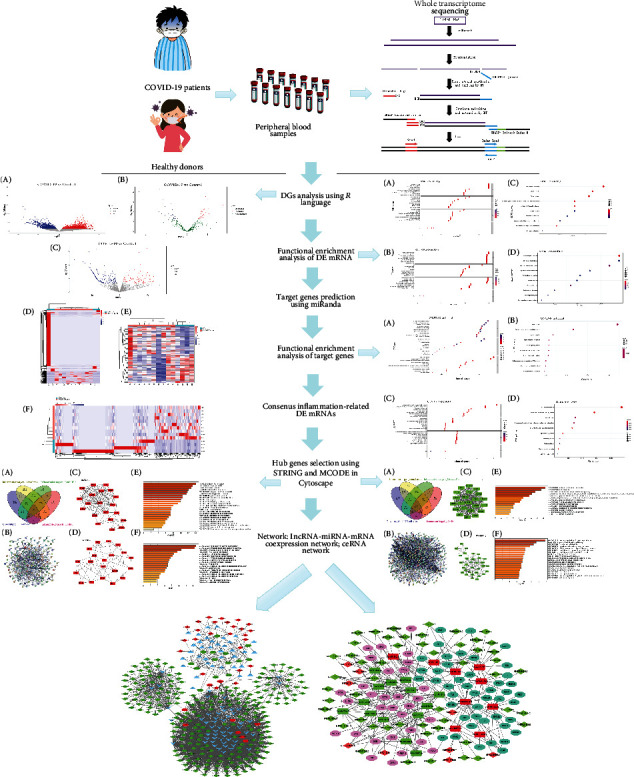
The workflow of the Whole-transcriptome RNA sequencing analysis of peripheral blood samples of COVID-19 patients.

With great advances in high-throughput RNA sequencing (RNAseq) technologies, transcriptomic analyses of peripheral blood samples of patients with virus infection are enable us to analyze the differentially expressed genes (DEGs) that associated with the host immune and/or inflammation response and gene interaction regulatory networks [10, 23]. Interestingly, integrated mRNA, miRNA, and lncRNA sequencing analysis will further increase understanding of the biological functions and the corresponding signaling pathways of DEGs in different diseases. However, relevant researches to discuss the global gene expression profile of COVID-19 are greatly limited. In the current study, whole-transcriptome RNAseq was performed in peripheral blood collected from human COVID-19 patients and healthy donors to illustrate the transcriptomic landscape at mRNAs, miRNAs, and lncRNAs levels. Finally, we successfully constructed a lncRNA-miRNA-mRNA network to research preliminarily the potential molecular mechanisms underlying in response to inflammatory host responses in COVID-19.  Figure 1 shows the workflow of the whole-transcriptome RNA sequencing study of peripheral blood samples of COVID-19 patients.

## 2. Materials and Methods

### 2.1. Patients

The peripheral blood samples were obtained from 10 COVID-19 patients (named as X1~X10, respectively) and 4 healthy donors (named as X11~X14, respectively) at the Fourth Affiliated Hospital, College of Medicine, Zhejiang University from February to March 2020. Besides, the clinical data of these COVID-19 patients was recorded that ranged for various parameters to increase the understanding of COVID-19.

The study was approved by the Ethics Committee of the Fourth Affiliated Hospital, College of Medicine, Zhejiang University, and registered at the Chinese Clinical Trial Registry (ChiCTR2000030305, http://www.medresman.org.cn/login.aspx). All patients provided written informed consent, and the patients tested positive for SARS-CoV-2 RNA in pharyngeal swab specimens by real-time reverse transcription-polymerase chain reaction (RT-PCR).

### 2.2. RNA Extraction, Quality Control, Library Construction, and Sequencing

The whole blood (2 mL) was extracted from all donors by using BD PAXgene blood RNA tubes (BD, cat. no. 762165). The blood was then mixed up and down 8-10 times. After that, the PAXgene tubes were incubated at room temperature for at least 2 h to ensure that the blood cells were completely dissolved. Next, the total RNA was extracted using the TRIzol reagent (Invitrogen, USA) following the manufacturer's protocol. Subsequently, the quantity and quality of total RNA were assessed using a NanoDrop 2000 Spectrophotometer (Thermo Fisher Scientific, USA) and Agilent 2100 bioanalyzer (Agilent Technologies, USA). The sequencing libraries were created with a TruSeq Stranded Total RNA Library Prep Kit (Illumina, USA) according to the manufacturer's protocol. The libraries were sequenced on an Illumina HiSeq X Ten platform at katimesbio CO., LTD (Hangzhou, China) generating 150 bp paired-end reads.

### 2.3. Preprocessing of Raw Reads

The quality of the raw reads was estimated using fastp software [24] to remove the low-quality reads and the adaptors. Also, reads that were shorter than 18 nt and those that mapped to rRNA were discarded. Next, the remaining clean reads were genome-mapped using spliced mapping in the Hisat2 package [25].

### 2.4. Identification of miRNA and lncRNA

miRNAs were initially identified with miRdeep2 software (https://github.com/rajewsky-lab/mirdeep2) [26]. For lncRNAs, we screened transcripts longer than 200 bp containing two or more exons from the alignment and chose the transcripts with noncoding ability using the EggNOG (version 5.0.0), Coding-Non-Coding index (CNCI, version 2.0), Pfam Scan (version 1.6), and Coding Potential Calculator 2.0 (CPC2).

### 2.5. Screening of DE mRNAs, DE miRNAs, and DE lncRNAs

The expression level of each mRNA and lncRNAs was calculated according to the fragments per kilobase of the transcript per million mapped read (FPKM) method. And the expression level of each miRNA was analyzed with the reads per million (RPM) method. Differential expression analysis of mRNA, miRNAs, and lncRNAs was performed with the DESeq R package (version 3.11, https://bioconductor.org/packages/release/bioc/html/DESeq2.html). Transcripts with a *p* value<0.05 and ∣log_2_^foldchange(FC)^ | >1 were considered as DE mRNAs, DE miRNAs, and DE lncRNAs. And the hierarchical clustering analysis was drawn on the DE mRNAs, DE miRNAs, and DE lncRNAs by using the pheatmap R package (http://www.bioconductor.org/packages/release/bioc/html/heatmaps.html).

### 2.6. Target Gene Prediction and Functional Enrichment Analysis

To investigate the function of DE miRNAs and DE lncRNAs, we predicted the target genes of all DE miRNAs, as well as the lncRNA *cis*-target genes. Briefly, the miRanda (http://www.microrna.org/microrna/getDownloads.do) was used to predict the target genes of DE miRNAs. *Cis*-regulation was used to predict lncRNA targets, and then coding genes in 100 kb upstream and downstream were selected in this study. Furthermore, Gene Ontology (GO) and Kyoto Encyclopedia of Genes and Genomes (KEGG) pathway analysis were performed for these up and downregulated DE mRNAs, DE miRNA targets, and DE lncRNA targets by *R* package clusterProfiler (http://bioconductor.org/packages/release/bioc/html/clusterProfiler.html) [27].

### 2.7. Preparation of Inflammation-Related Targets

The putative inflammation-related target genes were collected from the following database. GeneCards (https://www.genecards.org/) provides comprehensive transcriptomic information on the annotated and predicted human genes [28]: The GenCLip 3 (http://ci.smu.edu.cn/genclip3/analysis.php), multifunctional text-mining tools with the integration of Sphinx with MySQL to quickly retrieve function-related genes from more literature sources [29], and Online Mendelian Inheritance in Man® (OMIM®, https://omim.org/), an online catalog of human genes and genetic disorders [30]. Therefore, we retrieved these three databases with the keywords “inflammatory response” to identify the targets associated with inflammation.

### 2.8. Construction of Inflammation-Related DE mRNA PPI Network and Module Selection

The identified inflammation-related targets were integrated with up/downregulated DE mRNA, respectively, to obtain the potential inflammation-related DE mRNAs in COVID-19. To explore the interaction of inflammation-related up/downregulated DE mRNAs, a protein-protein interaction (PPI) network was established according to the Search Tool for the Retrieval of the Interacting Genes (STRING, http://string.embl.de/) database [31]. Species are set as “Homo sapiens,” and the interactions with a combined score >0.4 were selected as significant. Then, the PPI networks of inflammation-related up/downregulated DE mRNAs were visualized by Cytoscape v3.6.1 software (https://cytoscape.org/), respectively. Besides, the Molecular Complex Detection (MCODE) was used to screen modules of PPI networks with degree cutoff = 2, node score cutoff = 0.2, *k* − core = 2, and max.depth = 100 [32]. Furthermore, the GO annotation and KEGG pathway analyses in the modules were performed by using the Metascape online tool (http://metascape.org/gp/index.html#/main/step1) [33].

### 2.9. Coexpression Correlation Analysis

To illustrate the coexpression correlation between DE lncRNAs and module targets in PPI networks, the Pearson correlation coefficients of each DE lncRNAs and module targets were calculated by using their expression matrix data. Pearson′s correlation coefficient > 0.9 and *p* value<0.05 were considered as the cutoff criterion.

### 2.10. Construction of the ceRNA Network

To investigate the possible interaction of module targets, DE miRNAs, and DE lncRNAs, a ceRNA network was constructed in this study. The DE lncRNA-miRNA regulatory relationships of DE miRNAs were predicted using the miRDB (http://mirdb.org/), an online database for miRNA target prediction and functional annotations [34]. Then, the predicted DE lncRNA-miRNA regulatory relationships were integrated with DE miRNAs to acquire the DE lncRNA -DE miRNA regulation relationship. We integrated the DE lncRNA-module target coexpression relationship and the DE miRNA-module target regulatory relationships; then, the lncRNA-miRNA-mRNA coexpression network was constructed by Cytoscape. Based on the mechanism of ceRNA and the lncRNA-miRNA-mRNA coexpression network, we drew attention to screening lncRNAs that can sponge DE miRNAs. Meanwhile, the ceRNA regulatory network was constructed.

### 2.11. Statistical Analysis

Results were expressed as the mean ± standard deviation. Statistical analysis was performed using one-way analysis of variance (ANOVA) and Tukey's post hoc test. A *p* value <0.05 was considered significant.

## 3. Results

### 3.1. Baseline Characteristics of 10 COVID-19 Patients

By 31^st^ March 2020, 10 admitted hospital patients were identified as laboratory-confirmed SARS-CoV-2 infection in Yiwu (Zhejiang, China) in the current study (Table 1). The COVID-19 patients were aged 22~73 years, and 6 patients (60%) were female. Fever (50%), cough (60%), and sputum production (30%) were the most common symptoms. According to chest computed tomography findings, 7 patients (70%) presenting with ground-glass opacity. In blood tests, the leukocytes were significantly decreased in COVID-19 patients, while the lymphocytes were below the levels of healthy donors. Compared to healthy controls, there were no differences in inflammatory indicators of COVID-19 patients in this study.

### 3.2. Identification of Differentially Expressed Genes

**Figure 2:**
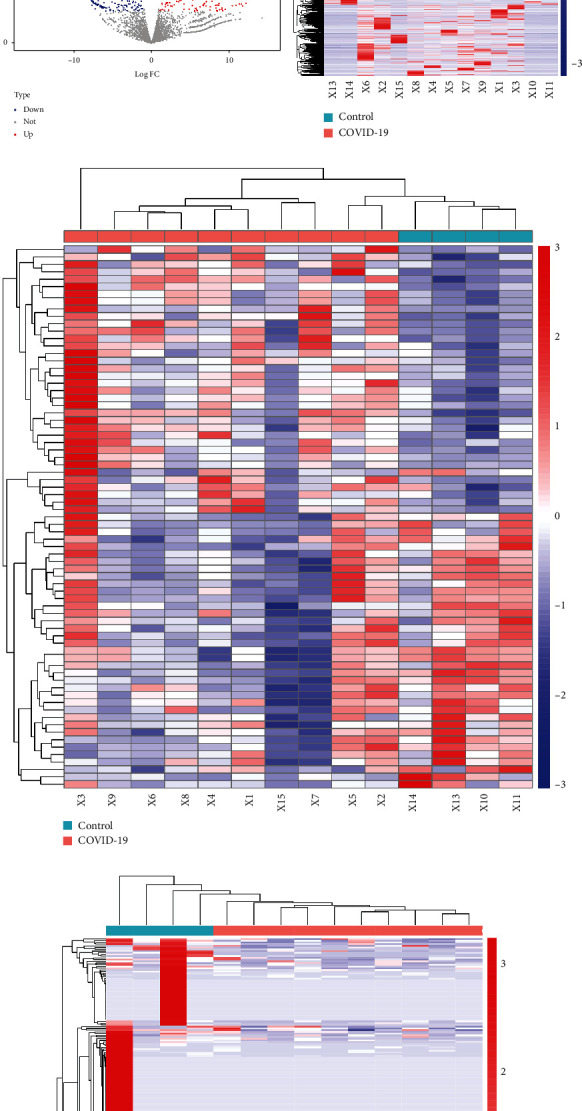
Volcano plots and heatmaps of differentially expressed genes (DEGs). Volcano plots showing significantly different expressions of mRNAs (a), miRNAs (b), and lncRNAs (c) between the COVID-19 and control groups. Red points: upregulated DEGs; blue points: downregulated DEGs; gray and green points: the genes with no obvious difference in expression levels. Heatmaps of DE mRNAs (d), DE miRNAs (e), and DE lncRNAs (f). Red represents upregulation, and blue represents downregulation.

To explore the potential mechanism of “cytokine storm” in COVID-19 patients, we performed the whole transcriptome sequencing to identify the DEGs in the peripheral blood samples. EggNOG, Pfam, CNCI, and CPC2 were used to screen out transcripts with coding potential in this study. The significant differences in DE mRNA, DE miRNA, and DE lncRNA expression between the COVID-19 patients and control groups were represented by volcano plot (Figure 2). Based on the screening criteria, a total of 25,482 DE mRNA were identified, of which 21,340 were downregulated and 4,142 were upregulated (Figure 2(a) and Table S1); 23 DE miRNA were determined to have a fold change ≥ 2.0 and *p* < 0.05, of which 9 were upregulated and 14 were downregulated (Figure 2(b) and Table S2); additionally, we obtained 410 DE lncRNAs including 129 upregulated and 281 downregulated (Figure 2(c) and Table S3). Differentially expressed mRNA, miRNA, and lncRNAs were hierarchically clustered, as shown in Figures 2(d)–(f), from which we found that the COVID-19 samples can be observably separated from the control samples, suggesting that the differential expression analysis of RNA sequencing data was adequately trustworthy.

### 3.3. Functional Analysis of Up/Downregulated DE mRNAs

**Figure 3:**
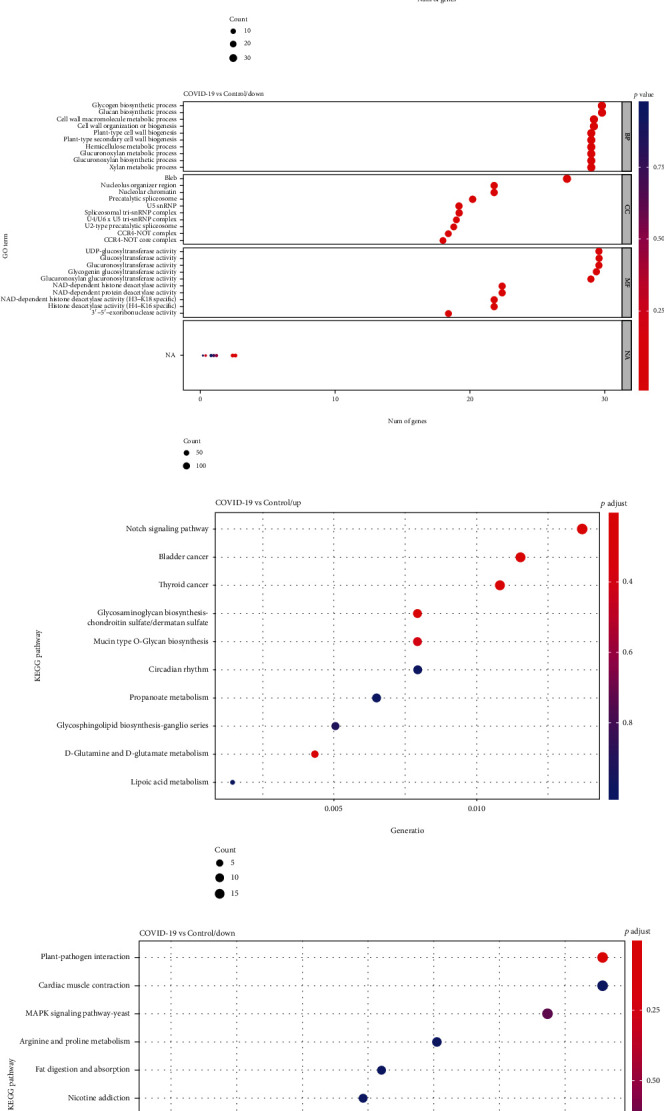
Go terms and KEGG pathway enrichment of up/downregulated DE mRNAs in peripheral blood samples of COVID-19 patients. GO term functional enrichment with 3 categories (BP, MF, CC) for upregulated DE mRNAs (a) and downregulated DE mRNAs (b). Top 10 pathways enriched by upregulated DE mRNAs (c) and downregulated DE mRNAs (d).

We performed functional annotation and KEGG enrichment analysis on the up/downregulated DE mRNAs, respectively.  Figure 3 displays only the top 10 biological processes (BP), cellular component (CC), and molecular function (MF) terms enriched by up/downregulated DE mRNAs. GO enrichment analysis indicated that upregulated DE mRNAs were significantly concentrated in detection of bacterium, detection of external biotic stimulus, antigen processing and presentation of endogenous antigen, positive regulation of T cell mediated cytotoxicity, and positive regulation of gamma-delta T cell activation in BP, azurophil granule membrane and MHC protein complex in CC, telomeric DNA binding, and TAP binding in MF (Figure 3(a)). Also, results of GO analysis showed that the downregulated DE mRNAs were significantly enriched in glycogen biosynthetic process, glucan biosynthetic process, and cell wall macromolecule metabolic process in BP, nucleolus organizer region and nucleolar chromatin in CC, UDP-glucosyltransferase activity, and NAD-dependent histone deacetylase activity in MF (Figure 3(b)). Besides, the results of the pathway analysis showed that the upregulated DE mRNAs were significantly enriched in the Notch signaling pathway (Figure 3(c)). Moreover, the downregulated DE mRNAs were significantly associated with plant-pathogen interaction, cardiac muscle contraction, and MAPK signaling pathways (Figure 3(d)).

### 3.4. GO and KEGG Analysis of miRNA-/lncRNA-Related Target Genes

**Figure 4:**
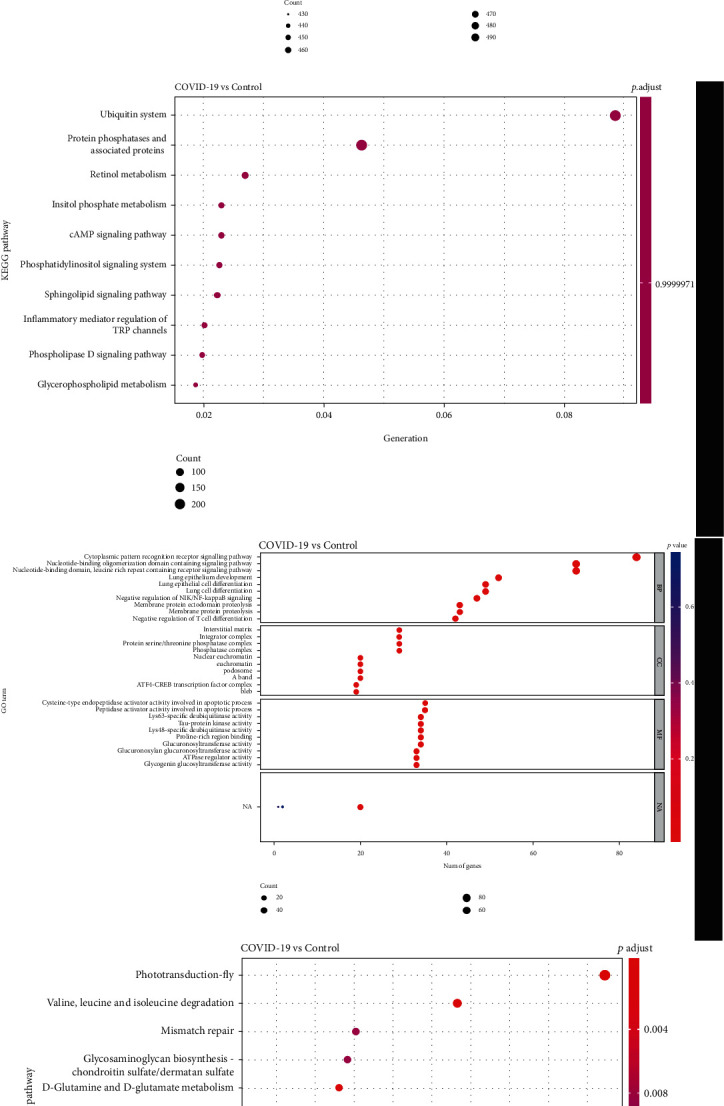
Functional enrichment analyses for target genes of DE miRNA and DE lncRNAs. Bubble plot shows GO (a) and KEGG terms (b) enriched in target genes of DE miRNA. Bubble plot shows GO (c) and KEGG terms (d) enriched in genes regulated by DE lncRNAs.

To investigate the biological significance of miRNA-/lncRNA-related target genes, GO and KEGG enrichment analysis was performed by *R* package clusterProfiler. GO enrichment analysis for miRNA-related genes indicated that they were mainly enriched in aromatic compound catabolic process, regulation of immune effector process in BP, nuclear chromosome part in CC, and Ras GTPase bing in MF (Figure 4(a)). Interestingly, our current study showed found miRNA-related genes are related to the ubiquitin system, inflammatory mediator regulation of TRP channels, etc. (Figure 4(b)). Moreover, the results showed that those genes related to lncRNAs were significantly enriched in the cytoplasmic pattern recognition receptor signaling pathway, lung cell differentiation, negative regulation of NIK/NF-kappaB signaling, and negative regulation of T cell differentiation in BP, protein serine/threonine phosphatase complex in CC, and cysteine-type endopeptidase activator activity involved in the apoptotic process in MF (Figure 4(c)). And the lncRNA-related target genes were associated with phototransduction-fly and valine, leucine, and isoleucine degradation pathways (Figure 4(d)).

### 3.5. PPI Network Analysis

**Figure 5:**
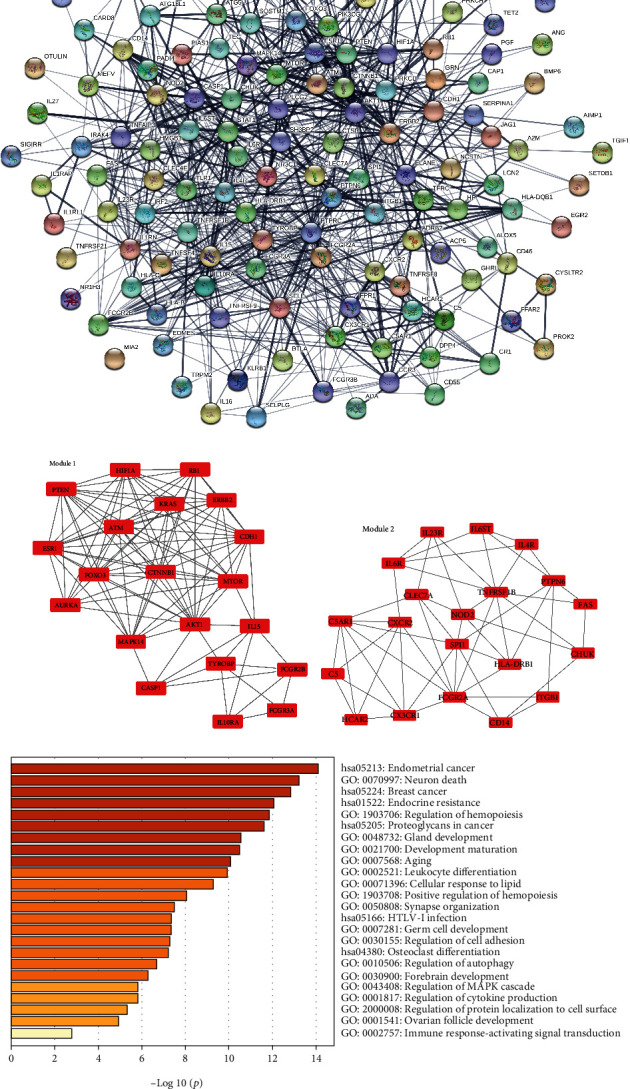
Two modules obtained from upregulated-PPI network and its functional enrichment analysis. (a) 149 upregulated inflammation-related DE mRNAs in COVID-19 were collected with Venn analysis. (b) PPI network of upregulated inflammation-related DE mRNAs. (c, d) Significant clustered modules from the PPI network. Red rectangles represent upregulated genes. The GO-BP terms and KEGG pathways enrichment analysis for module 1 (e) and module 2 (f), respectively, using the Metascape database.

In the current study, the identified inflammation-related targets were collected from the GeneCards, GenCLiP 3, and OMIM databases, respectively. Next, we integrated the upregulated DE mRNAs and the inflammation-related targets, and the results are presented in Figure 5(a) which shown that a total of 149 upregulated inflammation-related DE mRNAs in COVID-19 were obtained. We established the PPI network of the 149 upregulated DE mRNAs associated with the inflammation response according to the interactions predicted by the STRING database. A total of 847 edges and 140 nodes were included (Figure 5(b)). The nodes with a high degree value can be treated as the hub genes of the network. The PPI network of the inflammation-related upregulated DE mRNAs was divided into two modules (Figures 5(c) and 5(d)). AKT serine/threonine kinase 1 (AKT1, degree = 16) was the core of module 1 (score = 10.526), which included 100 edges and 20 nodes. TNF receptor superfamily member 1B (TNFRSF1B, degree = 9) and Fc fragment of IgG receptor IIa (FCGR2A, degree = 9) were the hub genes of module 2 (score = 5.579), which included 53 edges and 20 nodes. Moreover, target genes in module 1 and module 2 were performed for BP and KEGG pathway enrichment analysis. Enrichment analyses for module 1 demonstrated that the BP and pathways are mainly associated with GO 0070997~neuron death, GO: 1903706~regulation of hemopoiesis, GO: 0002521~leukocyte differentiation, GO: 0002757~immune response-activating signal transduction, hsa05213~endometrial cancer, hsa01522~endocrine resistance, and hsa05166~human T cell leukemia virus type I (HTLV-I) infection (Figure 5(e)). Notably, we found that the genes of module 2 were especially involved in GO: 0001817~regulation of cytokine production, hsa04060~cytokine-cytokine receptor interaction, GO:0046649~lymphocyte activation, and GO:0006925~inflammatory cell apoptotic process (Figure 5(f)), indicating that SARS-CoV-2 infection caused active inflammatory responses that resulted in pneumonia in human beings.

**Figure 6:**
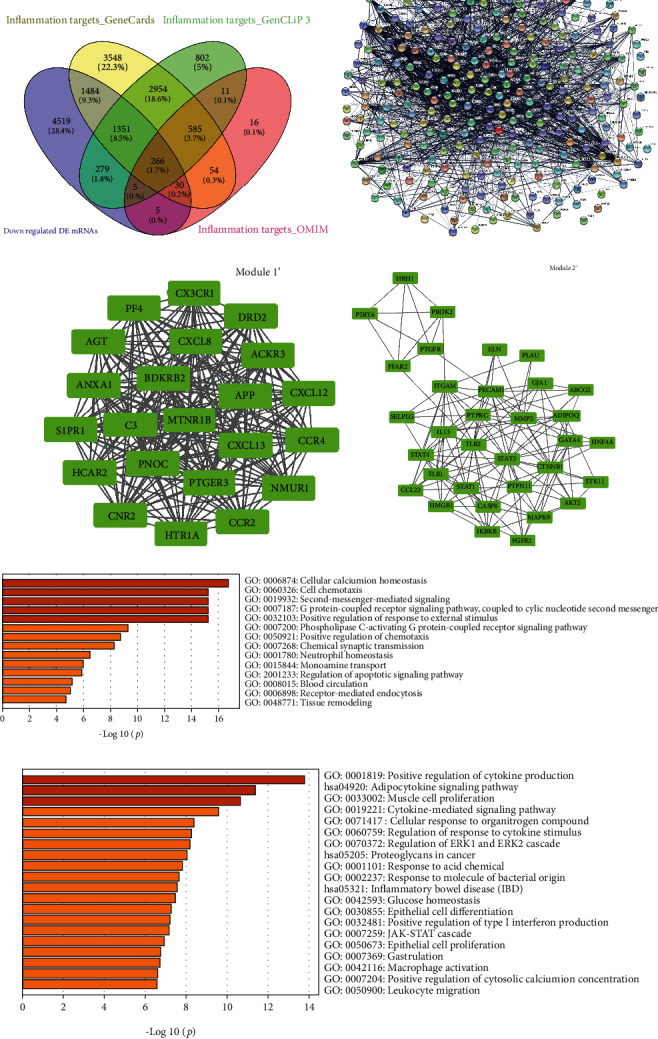
Two modules obtained from the downregulated PPI network and its functional enrichment analysis. (a) 266 downregulated inflammation-related DE mRNAs in COVID-19 were collected with Venn analysis. (b) PPI network of downregulated inflammation-related DE mRNAs. (c, d) Significant clustered modules from the PPI network. Green rectangles represent downregulated genes. The GO-BP terms and KEGG pathways enrichment analysis for module 1′ (e) and module 2′ (f), respectively, using the Metascape database.

Similarly, we also integrated the downregulated DE mRNAs and the inflammation-related targets. Subsequently, the 266 downregulated inflammation-related DE mRNAs were analyzed through the STRING database and Cytoscape software (Figure 6(a)), which formed a PPI network that contains 254 nodes and 1948 edges (Figure 6(b)). The MCODE approach in Cytoscape was used to identify modules in the PPI network of downregulated inflammation-related DE mRNAs. Then, two modules in the downregulated PPI network were established with the *k* − core = 2. Module 1′ (score = 22) containing 22 nodes and 231 edges was identified, and these nodes had the highest node degree (degree = 21) (Figure 6(c)). According to the degree of the total downregulated PPI network, C-X-C motif chemokine ligand 8 (CXCL8) was selected as the core of module 1. Signal transducer and activator of transcription 3 (STAT3) and toll-like receptor 2 (TLR2) were the hub genes of module 2 (score = 10.588), which included 131 edges and 32 nodes (Figure 6(d)). Also, we performed functional enrichment analyses for these dysregulated genes from the module 1′ and module 2, respectively. Many terms of different BP were clustered for module 1′, including GO: 0006874~cellular calcium ion homeostasis, GO: 0060326~cell chemotaxis, and GO: 0019932~second-messenger-mediated signaling (Figure 6(e)). To our great interest, multiple GO and KEGG terms were enriched for module 2 that potentially associated with the cytokine storm in COVID-19, for example, “GO: 0001819~positive regulation of cytokine production,” “GO: 0019221~cytokine-mediated signaling pathway,” “GO: 0042116 ~ macrophage activation”, and “GO: 0050900 ~ leukocyte migration” (Figure 6(f)). All molecular aspects including biological processes and signaling pathways point to the cytokine production and cytokine-mediated signaling pathway, suggesting the worth of the considerable potential value of inflammation-related hub genes in the progression of COVID-19.

### 3.6. Construction of the ceRNA Network

**Figure 7:**
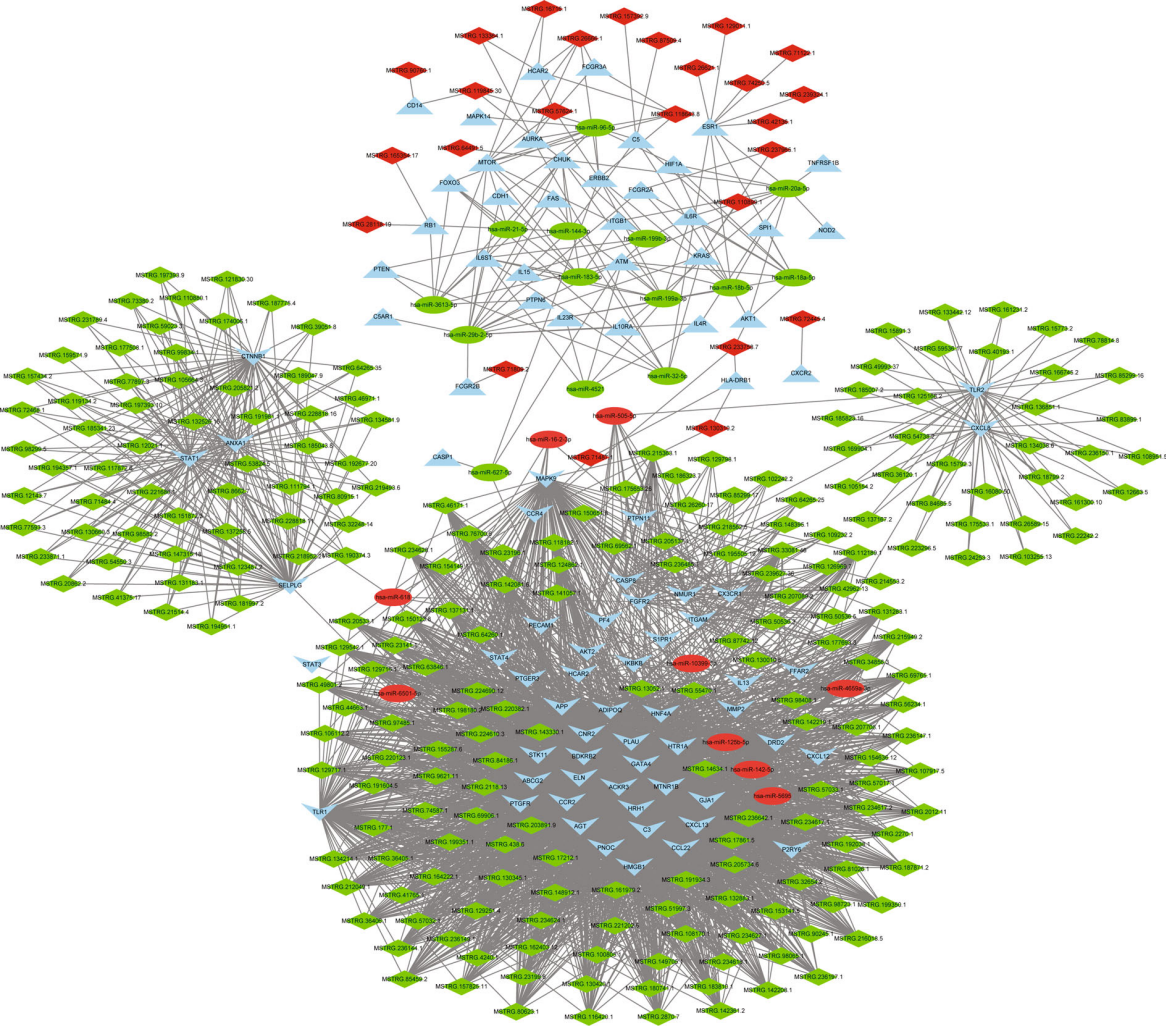
The lncRNA-miRNA-mRNA coexpression network. The downward “v” triangle represents the downregulated DE mRNAs, the upward triangle represents the upregulated DE mRNAs, the red ellipse represents the upregulated DE miRNAs, the green ellipse represents the downregulated DE miRNAs, red diamond indicates upregulated DE lncRNAs, and green diamond indicates downregulated DE lncRNAs.

**Figure 8:**
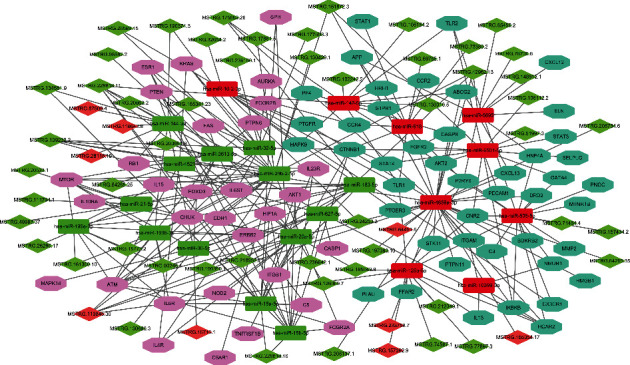
The competing endogenous RNA (ceRNA) network of COVID-19. Pink octagon indicates the upregulated DE mRNAs, light blue octagon indicates the downregulated DE mRNAs, red rectangle indicates upregulated DE miRNAs, green rectangle indicates downregulated DE miRNAs, red diamond indicates upregulated DE lncRNAs, and green diamond indicates downregulated DE lncRNAs.

Based on the DE lncRNA-module target coexpression relationship and the DE miRNA-module target regulatory relationships, the lncRNA-miRNA-mRNA coexpression network was constructed. In total, 3,570 lncRNA-miRNA-mRNA interactions were finally obtained (Figure 7), including 258 DE lncRNAs, 9 upregulated and 14 downregulated DE miRNAs, and 40 upregulated and 55 downregulated module targets. Further, according to the lncRNA-miRNA-mRNA coexpression network and the DE lncRNA-DE miRNA regulation relationship, the ceRNA network was also established (Figure 8). Finally, we found that MSTRG.119845.30/hsa-miR-20a-5p/TNFRSF1B, MSTRG.119845.30/hsa-miR-29b-2-5p/FCGR2A, MSTRG.106112.2/MSTRG.151872.3/MSTRG.51997.3/hsa-miR-6501-5p/STAT3, and MSTRG.106112.2/MSTRG.151872.3/MSTRG.51997.3/hsa-miR-6501-5p/TLR2, MSTRG.105154.2/MSTRG.130010.5/MSTRG.175653.26/MSTRG.228818.11/MSTRG.102242.2/hsa-miR-142-5p/TLR2, MSTRG.157434.2/MSTRG.71484.4/hsa-miR-505-5p/TLR2 axes and a lncRNA could sponge several miRNAs, and a miRNA could also interact with multiple mRNAs. These results highlighted the important role of lncRNAs in pathogenesis of COVID-19 by interacting with miRNAs.

## 4. Discussion

As is widely known, the SARS-CoV-2, a novel coronavirus, is probably originated from zoonotic coronaviruses, which causes serious pneumonia and lung failure to threaten global public health [35]. Currently, numerous diagnostic kits to test for COVID-19 are optional, and several promising antiviral agents against SARS-CoV-2, such as favipiravir, remdesivir, lopinavir, and ritonavir, have shown clinical effectiveness [36]. Although there have been made significant advances in the development of vaccines and drugs against COVID-19, the underlying mechanisms of the pathogenesis of SARS-CoV-2-associated pneumonia in human beings remain incompletely understood. In our study, leukocytes and lymphocytes were significantly decreased in COVID-19 patients. Hence, it is important to explore the mechanisms and identify the biomarkers associated with the development of COVID-19.

Several studies have shown that massive alterations in the host transcriptome have been caused to aid in helping the viral survival and replication by regulating the immune and inflammation response under viral infection [10, 37]. With the development of high throughput sequencing, the intension of the transcriptome has been enlarged to contain many types of transcripts, including mRNAs and ncRNAs. In this study, we simultaneously detected mRNAs, miRNAs, and lncRNAs in peripheral blood from COVID-19 patients and healthy donors by using whole transcriptome sequencing. We found that 25,482 DE mRNA, 23 DE miRNA, and 410 DE lncRNAs when peripheral blood samples of COVID-19 patients were compared to control, respectively. Then, the DE mRNAs were used to be enriched into GO terms and KEGG pathways. As expected, results showed that these DEGs were associated with the detection of bacterium, positive regulation of T cell mediated cytotoxicity, positive regulation of gamma-delta T cell activation, glycogen biosynthetic process, Notch signaling pathway, and MAPK signaling pathways. Additionally, functional analysis on the targets of DE miRNA and DE lncRNAs was also involved in the regulation of immune effector process, inflammatory mediator regulation of TRP channels, lung cell differentiation, negative regulation of NIK/NF-kappaB signaling, and negative regulation of T cell differentiation, which all play significant roles in depressing viral infection.

The inflammatory response has been thought to be a hallmark of SARS and MERS disease with an increased level of IL-6, IL-8, CXCL10, CCL2, and CCL3 [38, 39]. A recent study demonstrated that COVID-19 severe patients had a low level of counts of T cells and a high level of IL-2, IL-6, IFN-*γ*, and IL-10 compared with the mild patients [40], similar to the results in SARS and MERS. Meanwhile, Xu et al. found mild fibrosis and moderate inflammation in the lung biopsy of COVID-19 patients [14]. Therefore, further highlighting of detailed mechanisms and potential targets in this process would be to establish a foundation for future investigation. In this study, we integrated the up/downregulated DE mRNA with inflammation-related target genes, respectively. We performed GO and KEGG pathway analysis to explore the biological functions and potential pathways on module targets of PPI networks. GO analyses indicated that they were enriched in leukocyte differentiation, immune response-activating signal transduction, and positive regulation of cytokine production. KEGG analyses indicated that these genes associated with inflammation in COVID-19 were enriched in cytokine-cytokine receptor interaction and T17 cell differentiation. Taken together, these important biological processes and pathways of module targets played important roles in the inflammatory process of COVID-19, which strengthened our understanding of the underlying mechanism of “cytokine storm” in COVID-19 and may provide novel therapeutic targets for COVID-19.

We also performed the network topology analysis for module genes. Then, a total of 6 hub targets were identified in the modules including AKT1, TNFRSF1B, FCGR2A, CXCL8, STAT3, and TLR2. Recently, a study revealed a prominent alteration of Akt/mTOR/HIF-1 signaling at the proteotranscriptomic levels in SARS-CoV-2 infected Huh7 cells [41]. The activation of the Akt/mTOR pathway might enable SARS-CoV-2 infection by blocking autophagy and apoptosis [42]. Moreover, a high level of IL-6 can activate mTOR in a STAT3-dependent or independent manner [43, 44]. Accumulating evidence indicates that TNF receptor (TNFR) family member, TNFRSF1B (TNFR2), which is sustained T cell responses, plays important role in protective immunity, inflammatory, and autoimmune diseases [45, 46]. Fc gamma receptors (Fc*γ*Rs) have an important function in humoral immune responses. Also, rijkers et al. reported that B lymphocyte growth and differentiation factors, including IL-6, IL-10, and TNFRSF1B are associated with increased susceptibility for pneumonia [47]. CXCL8 (also referred to as IL-8), circulating chemokine, is associated with inflammation and immune cell trafficking in the context of viral infections [48]. TLR2 is well-studied toll-like receptors that identify both structural and nonstructural proteins of the virus, as well as cytokine production and inflammation [49]. And the study by Lester et al. shows TLR agonists can help to initiate an immune response and actively take part in viral clearance [50]. Taken together, the above hub targets were significantly related to the immune and inflammatory process of COVID-19.

Increasing evidence indicates that lncRNAs can influence miRNA activity as endogenous sponges to affect mRNA, act as ceRNAs, to regulate a series of biological processes. In the current study, according to the circRNA-miRNA-mRNA coexpression network and the ceRNA network, we observed that MSTRG.119845.30 was identified to bind hsa-miR-20a-5p; MSTRG.119845.30 was identified to hsa-miR-29b-2-5p; MSTRG.106112.2, MSTRG.151872.3, and MSTRG.51997.3 were identified to bind hsa-miR-6501-5p; MSTRG.105154.2, MSTRG.130010.5, MSTRG.175653.26, MSTRG.228818.11, and MSTRG.102242.2 were identified to bind hsa-miR-142-5p; MSTRG.157434.2 and MSTRG.71484.4 were identified to bind hsa-miR-505-5p competitively with its binding sites. None of these DE lncRNAs and DE miRNAs have been reported to be functional during COVID-19, which provided useful noncoding target candidates for further work on COVID-19 pathogenesis.

## 5. Conclusion

In summary, this study investigated the underlying mechanism of “cytokine storm” in COVID-19 patients using whole-transcriptome sequencing. We identified 95 module targets involved in the positive regulation of cytokine production, including AKT1, TNFRSF1B, FCGR2A, CXCL8, STAT3, and TLR2. In addition, we identified several differentially expressed miRNAs and lncRNAs in peripheral blood samples of COVID-19 patients that were closely related to the regulation of the immune effector process and negative regulation of NIK/NF-kappaB signaling. On the basis of the codifferently expressed lncRNAs and mRNA transcripts, we constructed a ceRNA regulatory network that contained 95 DE mRNAs, 23 DE miRNAs, and 258 DE lncRNAs. Within the network, we suspected that the 6 ceRNA subnetworks (i.e., MSTRG.119845.30/hsa-miR-20a-5p/TNFRSF1B, MSTRG.119845.30/hsa-miR-29b-2-5p/FCGR2A, and MSTRG.106112.2/hsa-miR-6501-5p/STAT3) may play a crucial role in the development of COVID-19. These DEGs contribute to an increased understanding of the genomic landscape of SARS-CoV-2 infection and may be confirmed as promising diagnostic biomarkers and therapeutic strategies for COVID-19 patients. However, there are some limitations to this study. First, the DE mRNAs, DE miRNAs, and DE lncRNAs were identified based on the small sample data. In the future, the precise changes of these DEGs should be confirmed based on large clinical samples. Second, further experimental validation with corresponding COVID-19 model in vitro and in vivo should be made to identify the specific roles of these DEGs.

## Figures and Tables

**Table 1:** Clinical characteristics of subjects with COVID-19 and health donors.

Characteristic	Control (*n* = 4)	Patients (*n* = 10)
Age, y, mean ± SD	34.75 ± 11.84	44.90 ± 19.94
Men, no. (%)	1 (25)	4 (40)
Women, no. (%)	3 (75)	6 (60)
Signs and symptoms		
Fever		5 (50)
Cough		6 (60)
Sputum production		3 (30)
Sore throat		1 (10)
Stuffy nose		2 (10)
Gastrointestinal (GI) symptom		
Abdominal pain		1 (10)
Chest computed tomography		
Right ground-glass opacity		2 (20)
Right lung infiltrates		2 (20)
Left ground-glass opacity		3 (30)
Bilateral lung ground glass opacity		2 (20)
Bilateral lung infiltrates		1 (10)
Blood count, ×10^9^/L, mean ± SD		
Leukocyte	7.40 ± 1.4	5.19 ± 1.7^∗^
Lymphocyte	37.35 ± 4.29	24.02 ± 8.58^∗^
Inflammatory indicators, mean ± SD		
C-reactive protein, mg/L	1.00 ± 0.75	12.61 ± 16.61
Tumor necrosis factor *α* (TNF-*α*), pg/mL	10.35 ± 2.40	44.98 ± 47.67
Interleukin-6, pg/mL	3.24 ± 2.82	15.09 ± 20.81

Abbreviations: COVID-19: coronavirus disease 2019; ^∗^*p* < 0.05.

## Data Availability

All supporting data of this study can be found within the manuscript and its supplementary files and available from the corresponding author upon request.

## Abbreviations

SARS-CoV-2: Severe acute respiratory syndrome coronavirus 2
COVID-19: Coronavirus disease 2019
DE mRNA: Differentially expressed messenger RNAs
DE miRNA: Differentially expressed microRNAs
DE lncRNAs: Differentially expressed long noncoding RNAs
Go: Gene ontology
PPI: Protein-protein interaction
WHO: World Health Organization
ARDS: Acute respiratory distress syndrome
TLRs: Toll-like receptors
NLRs: NOD-like receptors
RLRs: RIG-I-like receptors
MERS-CoV: Middle East respiratory syndrome coronavirus
ncRNAs: Noncoding RNAs
ceRNA: Endogenous RNA
MREs: miRNA response elements
RNAseq: RNA sequencing
DEGs: Differentially expressed genes
CNCI: Coding-noncoding index
CPC2: Coding potential calculator 2.0
FPKM: Fragments per kilobase of transcript per million mapped reads
RPM: Reads per million
KEGG: Kyoto Encyclopedia of Genes and Genomes
STRING: Search Tool for the Retrieval of Interacting Genes
MCODE: Molecular complex detection
BP: Biological process
CC: Cellular component
MF: Molecular function
AKT1: AKT serine/threonine kinase 1
TNFRSF1B: TNF receptor superfamily member 1B
FCGR2A: Fc fragment of IgG receptor IIa
HTLV-I: Human T cell leukemia virus type I
CXCL8: C-X-C motif chemokine ligand 8
STAT3: Signal transducer and activator of transcription 3
TLR2: Toll-like receptor 2
Fc*γ*Rs: Fc gamma receptors.

## Supplementary Materials

Supplementary Materials Table S1: the differentially expressed mRNAs of peripheral blood samples in COVID-19 patients. Table S2: the differentially expressed miRNA of peripheral blood samples in COVID-19 patients. Table S3: the differentially expressed lncRNAs of peripheral blood samples in COVID-19 patients.
