# Supplementary material for: Whole-Transcriptome RNA Sequencing Reveals Significant Differentially Expressed mRNAs, miRNAs, and lncRNAs and Related Regulating Biological Pathways in the Peripheral Blood of COVID-19 Patients
Source: Mediators Inflamm. 2021 Apr 1;2021:6635925. doi: 10.1155/2021/6635925 (PMC8018221; doi:10.1155/2021/6635925)
Supplement: Supplementary Materials — Table S1: the differentially expressed mRNAs of peripheral blood samples in COVID-19 patients. Table S2: the differentially expressed miRNA of peripheral blood samples in COVID-19 patients. Table S3: the differentially expressed lncRNAs of peripheral blood samples in COVID-19 patients. [file 6635925.f1.zip › Table S1 (1).pdf]

Table S1 The differentially expressed mRNAs of peripheral blood samples in COVID-19 patients

Upregulated DE mRNAs

Gene_id	Gene_name	Xloc_id	trans_len	EggNOG	Pfam	CNCI	CPC2
MSTRG.231290.8	ALG13	XLOC_239470	1717	coding	coding	noncoding	noncoding
MSTRG.316.1	CDK11B	XLOC_000115	213	coding	noncoding	noncoding	noncoding
NR_135061.1	EXOSC10-AS1	XLOC_000509	541	coding	noncoding	coding	noncoding
MSTRG.1019.2	MTOR	XLOC_000510	2307	coding	coding	coding	noncoding
MSTRG.1019.3	MTOR-AS1	XLOC_000510	597	coding	coding	noncoding	noncoding
NM_001066.3	TNFRSF1B	XLOC_000569	3687	coding	coding	coding	coding
NM_012231.4	PRDM2	XLOC_000630	7957	coding	coding	coding	coding
MSTRG.1895.4	EIF4G3	XLOC_000981	6285	coding	coding	coding	coding
MSTRG.1895.5	EIF4G3	XLOC_000981	6169	coding	coding	coding	coding
MSTRG.1895.7	EIF4G3	XLOC_000981	6105	coding	coding	coding	coding
MSTRG.2026.4	HNRNPR	XLOC_001077	1866	coding	coding	noncoding	coding
MSTRG.2059.1	ELOA	XLOC_001093	4190	coding	coding	coding	coding
MSTRG.2114.9	SRSF10	XLOC_001111	2885	coding	coding	noncoding	noncoding
NR_159380.1	SRRM1	XLOC_001154	3815	coding	coding	coding	coding
MSTRG.2334.6	UBXN11	XLOC_001244	2145	coding	coding	coding	coding
NM_001039477.2	THEMIS2	XLOC_001351	1240	coding	coding	coding	coding
MSTRG.2514.3	RPA2	XLOC_001357	1523	coding	coding	coding	noncoding
XM_011541260.2	SMPDL3B	XLOC_001359	2636	coding	coding	coding	coding
MSTRG.2525.3	DNAJC8	XLOC_001370	1408	coding	coding	noncoding	noncoding
MSTRG.2963.3	RNF19B	XLOC_001585	2505	coding	coding	coding	noncoding
MSTRG.2963.5	RNF19B	XLOC_001585	1069	coding	noncoding	coding	noncoding
MSTRG.3153.3	SMIM12	XLOC_001693	1991	coding	coding	coding	noncoding
MSTRG.3198.1	ZMYM4	XLOC_001712	314	coding	noncoding	noncoding	noncoding
MSTRG.3360.7	STK40	XLOC_001779	3792	coding	coding	coding	coding
MSTRG.3360.10	STK40	XLOC_001779	4373	coding	coding	coding	coding
MSTRG.3360.15	LSM10	XLOC_001779	1043	coding	coding	coding	noncoding
MSTRG.3398.5	SNIP1	XLOC_001811	1699	coding	noncoding	coding	noncoding
MSTRG.3441.2	FHL3	XLOC_001826	1429	coding	coding	coding	coding
MSTRG.3441.3	FHL3	XLOC_001826	1612	coding	coding	coding	coding
MSTRG.3557.3	PABPC4-AS1	XLOC_001905	2997	coding	coding	coding	noncoding
MSTRG.3557.6	PABPC4-AS1	XLOC_001905	2927	coding	coding	coding	noncoding
MSTRG.3557.7	PABPC4-AS1	XLOC_001905	3102	coding	coding	coding	coding
MSTRG.3557.8	PABPC4-AS1	XLOC_001905	2547	coding	coding	coding	noncoding
MSTRG.3557.9	PABPC4-AS1	XLOC_001905	2736	coding	coding	coding	noncoding
NM_001350483.1	CAP1	XLOC_001937	2755	coding	coding	coding	coding
NM_001355226.2	CCDC30	XLOC_002157	5984	coding	coding	coding	coding
MSTRG.3857.2	P3H1	XLOC_002174	2460	coding	coding	coding	noncoding
NM_001255.3	CDC20	XLOC_002202	1649	coding	coding	coding	coding
MSTRG.4339.1	ZSWIM5	XLOC_002330	1469	coding	noncoding	noncoding	noncoding

MSTRG.4458.9	MKNK1	XLOC_002389	1791	coding	noncoding	coding	noncoding
MSTRG.4522.1	PDZK1IP1	XLOC_002421	907	coding	coding	coding	coding
MSTRG.4585.7	STIL	XLOC_002424	2066	coding	coding	noncoding	noncoding
MSTRG.4943.39	NRDC	XLOC_002565	2639	coding	coding	coding	noncoding
MSTRG.4926.2	TUT4	XLOC_002596	5689	coding	coding	noncoding	noncoding
MSTRG.5032.3	YIPF1	XLOC_002670	1691	coding	coding	noncoding	noncoding
MSTRG.5248.7	USP24	XLOC_002725	8116	coding	coding	coding	coding
MSTRG.5219.1	PLPP3	XLOC_002764	238	coding	noncoding	noncoding	noncoding
MSTRG.5512.1	DAB1	XLOC_002875	320	coding	noncoding	noncoding	noncoding
MSTRG.5365.12	OMA1	XLOC_002988	1969	coding	coding	noncoding	noncoding
MSTRG.5365.11	OMA1	XLOC_002988	3547	coding	coding	noncoding	coding
MSTRG.5547.2	MYSM1	XLOC_002999	7872	coding	coding	coding	noncoding
NM_001017415.1	USP1	XLOC_003148	3509	coding	coding	coding	coding
MSTRG.5847.1	DOCK7	XLOC_003159	270	coding	noncoding	noncoding	noncoding
XM_005271274.4	EFCAB7	XLOC_003199	4170	coding	coding	coding	coding
MSTRG.6112.8	JAK1	XLOC_003256	1088	coding	coding	noncoding	noncoding
MSTRG.6132.5	JAK1	XLOC_003269	4161	coding	noncoding	coding	noncoding
MSTRG.6132.11	JAK1	XLOC_003269	9647	coding	coding	noncoding	noncoding
NM_001077701.3	MIER1	XLOC_003343	5394	coding	coding	coding	coding
MSTRG.6466.5	GNG12-AS1	XLOC_003385	8915	coding	coding	noncoding	noncoding
XM_017001886.1	LRRC7	XLOC_003449	10184	coding	coding	coding	coding
MSTRG.6532.2	HHLA3	XLOC_003467	2126	coding	coding	coding	noncoding
MSTRG.6761.1	NEGR1	XLOC_003535	366	coding	noncoding	noncoding	noncoding
MSTRG.6921.2	PIGK	XLOC_003693	2577	coding	coding	noncoding	noncoding
MSTRG.6945.1		XLOC_003714	497	coding	noncoding	noncoding	noncoding
NM_006417.5	IFI44	XLOC_003750	1682	coding	coding	coding	coding
MSTRG.7474.1	LOC107985037	XLOC_003875	453	coding	noncoding	noncoding	noncoding
MSTRG.7386.21	SPATA1	XLOC_003993	2743	coding	coding	coding	noncoding
MSTRG.7408.2	SSX2IP	XLOC_004000	2307	coding	coding	noncoding	noncoding
MSTRG.7408.3	SSX2IP	XLOC_004000	1844	coding	coding	noncoding	noncoding
MSTRG.7679.1		XLOC_004062	251	coding	noncoding	noncoding	noncoding
MSTRG.7757.4	ODF2L	XLOC_004094	2374	coding	noncoding	coding	noncoding
MSTRG.7757.8	ODF2L	XLOC_004094	1492	coding	noncoding	noncoding	noncoding
MSTRG.8320.16	GBP3	XLOC_004266	3420	coding	coding	noncoding	noncoding
MSTRG.8100.2	ZNF644	XLOC_004388	9023	coding	noncoding	coding	noncoding
XR_001737461.1	CDC7	XLOC_004403	3271	coding	coding	coding	coding
MSTRG.8301.1	C1orf146	XLOC_004433	224	coding	noncoding	noncoding	noncoding
MSTRG.8461.2	EVI5	XLOC_004451	2599	coding	coding	coding	noncoding
NR_146333.1	RPL5	XLOC_004458	1012	coding	coding	coding	coding
MSTRG.8637.1	CCDC18	XLOC_004471	14747	coding	coding	coding	coding
MSTRG.8584.1	BCAR3	XLOC_004507	320	coding	coding	noncoding	noncoding
MSTRG.8597.1	ARHGAP29	XLOC_004530	411	coding	coding	noncoding	noncoding

MSTRG.9377.2	DPYD-AS1	XLOC_004641	1565	coding	coding	noncoding	noncoding
XM_017001504.1	TRMT13	XLOC_004713	4793	coding	coding	noncoding	coding
MSTRG.8972.1	LRRC39	XLOC_004717	272	coding	noncoding	noncoding	noncoding
MSTRG.9064.1	LOC102723784	XLOC_004828	555	coding	noncoding	noncoding	noncoding
MSTRG.9673.2	VAV3	XLOC_004963	2100	coding	coding	coding	noncoding
MSTRG.9651.3	SORT1	XLOC_005045	3331	coding	coding	coding	noncoding
MSTRG.9927.6	LRIF1	XLOC_005136	2555	coding	coding	coding	noncoding
MSTRG.10016.6	CEPT1	XLOC_005153	1943	coding	coding	coding	noncoding
MSTRG.10140.2	ST7L	XLOC_005241	1364	coding	coding	noncoding	noncoding
MSTRG.10140.4	ST7L	XLOC_005241	885	coding	coding	noncoding	noncoding
MSTRG.10312.4	SIKE1	XLOC_005335	5227	coding	coding	coding	noncoding
MSTRG.10434.1	SLC22A15	XLOC_005386	295	coding	noncoding	noncoding	noncoding
MSTRG.10805.12	NOTCH2	XLOC_005582	9567	coding	noncoding	noncoding	noncoding
MSTRG.10801.5	SEC22B	XLOC_005591	961	coding	coding	noncoding	noncoding
MSTRG.11377.1	LOC100996724	XLOC_005606	231	coding	noncoding	coding	noncoding
MSTRG.11448.1	FAM72B	XLOC_005658	342	coding	coding	noncoding	noncoding
MSTRG.11029.1	HIST2H3PS2	XLOC_005752	1931	coding	coding	coding	noncoding
MSTRG.11097.19	LOC100996740	XLOC_005796	18038	coding	noncoding	coding	coding
MSTRG.11194.3	NBPF20	XLOC_005802	434	coding	noncoding	noncoding	noncoding
MSTRG.11217.2	POLR3C	XLOC_005811	3675	coding	coding	coding	coding
MSTRG.11217.3	RNF115	XLOC_005811	1288	coding	coding	noncoding	noncoding
MSTRG.11218.8	PIAS3	XLOC_005812	1388	coding	coding	coding	noncoding
MSTRG.11245.6	NBPF10	XLOC_005836	1112	coding	noncoding	noncoding	noncoding
MSTRG.11245.25	NBPF10	XLOC_005840	20592	coding	coding	coding	coding
MSTRG.11245.36	NOTCH2NLA	XLOC_005840	11906	coding	coding	coding	noncoding
MSTRG.11245.38	NOTCH2NLA	XLOC_005840	2536	coding	coding	coding	noncoding
MSTRG.11286.2	FMO5	XLOC_005878	1977	coding	coding	noncoding	noncoding
MSTRG.11286.3	FMO5	XLOC_005878	463	coding	coding	noncoding	coding
MSTRG.12121.1	NBPF14	XLOC_005922	4581	coding	coding	coding	noncoding
MSTRG.12121.16	NBPF14	XLOC_005922	4704	coding	coding	coding	noncoding
MSTRG.12121.18	NBPF14	XLOC_005922	284	coding	coding	noncoding	noncoding
MSTRG.12121.35	NBPF14	XLOC_005922	2184	coding	coding	noncoding	noncoding
MSTRG.12121.43	NOTCH2NLB	XLOC_005922	13557	coding	coding	coding	noncoding
NR_111930.1	LOC100132057	XLOC_005931	1485	coding	noncoding	noncoding	noncoding
MSTRG.12143.12	LOC100132057	XLOC_005931	5716	coding	coding	coding	coding
MSTRG.12166.1		XLOC_005936	395	coding	noncoding	noncoding	noncoding
MSTRG.12198.2	PDE4DIP	XLOC_005945	14742	coding	coding	noncoding	noncoding
NM_001304723.1	PLEKHO1	XLOC_006033	2068	coding	coding	coding	coding
MSTRG.11622.4	ARNT	XLOC_006069	4537	coding	coding	noncoding	noncoding
MSTRG.11622.6	ARNT	XLOC_006069	4423	coding	coding	noncoding	noncoding
MSTRG.11622.5	ARNT	XLOC_006069	4426	coding	coding	noncoding	noncoding
MSTRG.11695.4	SELENBP1	XLOC_006111	1890	coding	coding	coding	coding

MSTRG.11977.13	CRTC2	XLOC_006269	1866	coding	coding	coding	noncoding
MSTRG.11977.12	CRTC2	XLOC_006269	1881	coding	coding	coding	noncoding
NM_000565.4	IL6R	XLOC_006295	5764	coding	coding	coding	coding
MSTRG.12078.6	SHC1	XLOC_006318	1986	coding	coding	coding	noncoding
MSTRG.12078.7	SHC1	XLOC_006318	1741	coding	coding	noncoding	noncoding
NM_005227.3	EFNA4	XLOC_006330	1254	coding	coding	coding	coding
NM_004428.3	EFNA1	XLOC_006336	1552	coding	coding	coding	coding
NM_001287590.2	SLC50A1	XLOC_006337	1340	coding	coding	coding	coding
MSTRG.12113.2	TRIM46	XLOC_006339	3603	coding	coding	coding	coding
MSTRG.12313.6	CLK2	XLOC_006347	1974	coding	coding	coding	coding
MSTRG.12486.6	ASH1L	XLOC_006352	5074	coding	coding	noncoding	noncoding
MSTRG.12486.24	GON4L	XLOC_006352	5819	coding	noncoding	coding	coding
MSTRG.12486.27	DAP3	XLOC_006352	2597	coding	noncoding	coding	coding
MSTRG.12486.58	GON4L	XLOC_006352	1463	coding	noncoding	noncoding	noncoding
MSTRG.12445.5	GLMP	XLOC_006390	1595	coding	coding	noncoding	coding
MSTRG.12445.4	TMEM79	XLOC_006390	3078	coding	coding	coding	noncoding
MSTRG.12461.2	MRPL24	XLOC_006421	889	coding	coding	noncoding	noncoding
MSTRG.12472.1	PRCC	XLOC_006425	367	coding	noncoding	noncoding	noncoding
XM_017000460.1	LRRC71	XLOC_006430	2307	coding	coding	coding	coding
MSTRG.12564.3	FCRL5	XLOC_006457	1587	coding	coding	noncoding	noncoding
MSTRG.12663.6	SPTA1	XLOC_006504	3215	coding	coding	coding	coding
NM_152501.5	PYHIN1	XLOC_006537	2088	coding	coding	coding	coding
MSTRG.12758.2	TAGLN2	XLOC_006619	1314	coding	coding	coding	noncoding
MSTRG.12887.11	LOC100287049	XLOC_006636	5932	coding	coding	coding	noncoding
XM_005245053.5	NCSTN	XLOC_006638	2021	coding	coding	coding	coding
MSTRG.12986.2	ARHGAP30	XLOC_006679	7085	coding	coding	coding	coding
MSTRG.13072.11	NIT1	XLOC_006685	3888	coding	coding	coding	noncoding
NM_016406.4	UFC1	XLOC_006686	888	coding	coding	coding	coding
MSTRG.13219.3	FCGR3B	XLOC_006730	3875	coding	coding	noncoding	noncoding
MSTRG.13219.6	FCGR3A	XLOC_006730	3771	coding	coding	noncoding	noncoding
MSTRG.13219.10	FCGR3B	XLOC_006730	6049	coding	coding	coding	noncoding
MSTRG.13376.1	RGS5	XLOC_006849	888	coding	noncoding	noncoding	noncoding
XM_024447727.1	POU2F1	XLOC_007003	14055	coding	coding	coding	coding
NM_001322923.1	RCSD1	XLOC_007024	5392	coding	coding	coding	coding
MSTRG.13805.1		XLOC_007102	278	coding	noncoding	noncoding	noncoding
MSTRG.14206.35	C1orf105	XLOC_007293	1432	coding	coding	noncoding	noncoding
MSTRG.14206.37	C1orf105	XLOC_007293	1502	coding	coding	noncoding	noncoding
MSTRG.14368.1	TNFSF4	XLOC_007372	207	coding	coding	noncoding	noncoding
MSTRG.14239.4	RC3H1	XLOC_007443	5556	coding	coding	noncoding	coding
MSTRG.14239.7	RC3H1	XLOC_007443	6354	coding	coding	noncoding	coding
NM_001366447.1	RABGAP1L	XLOC_007455	2790	coding	coding	coding	coding
NM_001330989.2	RABGAP1L	XLOC_007455	7229	coding	noncoding	coding	coding

MSTRG.14702.1	RABGAP1L	XLOC_007482	202	coding	noncoding	noncoding	noncoding
MSTRG.14308.2	KIAA0040	XLOC_007563	6185	coding	noncoding	coding	noncoding
MSTRG.14308.1	KIAA0040	XLOC_007563	6259	coding	noncoding	noncoding	noncoding
MSTRG.14869.17	COP1	XLOC_007604	5532	coding	coding	coding	noncoding
MSTRG.14956.4	TOR1AIP2	XLOC_007740	2368	coding	coding	noncoding	coding
MSTRG.15017.1	LHX4	XLOC_007773	281	coding	noncoding	noncoding	noncoding
MSTRG.15354.1		XLOC_007939	303	coding	noncoding	noncoding	noncoding
MSTRG.15659.10	PRG4	XLOC_008043	7866	coding	coding	coding	coding
XM_024449332.1	RO60	XLOC_008271	9013	coding	coding	coding	coding
XM_011509209.1	NEK7	XLOC_008463	3686	coding	coding	coding	coding
NM_080921.3	PTPRC	XLOC_008513	4946	coding	coding	coding	coding
MSTRG.16509.4	KIF14	XLOC_008587	987	coding	noncoding	noncoding	noncoding
MSTRG.16517.1	DDX59	XLOC_008592	1977	coding	coding	coding	noncoding
MSTRG.16630.1	UBE2T	XLOC_008668	342	coding	noncoding	noncoding	noncoding
MSTRG.17126.1		XLOC_008835	321	coding	noncoding	noncoding	noncoding
MSTRG.17453.1		XLOC_008991	232	coding	noncoding	noncoding	noncoding
MSTRG.17857.1	CD46	XLOC_009059	286	coding	noncoding	noncoding	noncoding
MSTRG.18405.5	RAB3GAP2	XLOC_009550	7289	coding	coding	coding	noncoding
XM_011509903.3	MARC1	XLOC_009566	2670	coding	coding	coding	coding
MSTRG.18547.2	DUSP10	XLOC_009601	1155	coding	coding	coding	coding
MSTRG.18836.4	CNIH3	XLOC_009770	8609	coding	coding	coding	coding
MSTRG.18836.5	CNIH3	XLOC_009770	7131	coding	coding	coding	coding
MSTRG.18836.9	WDR26	XLOC_009770	1854	coding	coding	coding	noncoding
MSTRG.18897.34	LBR	XLOC_009778	3996	coding	coding	noncoding	noncoding
MSTRG.18917.1	DNAH14	XLOC_009786	785	coding	noncoding	noncoding	noncoding
MSTRG.18958.7	TMEM63A	XLOC_009804	4748	coding	coding	coding	coding
MSTRG.19007.2	ITPKB	XLOC_009841	6894	coding	coding	coding	coding
MSTRG.19061.8	SNAP47	XLOC_009866	4384	coding	coding	coding	coding
MSTRG.19219.5	ABCB10	XLOC_009969	2905	coding	coding	noncoding	noncoding
MSTRG.19219.4	ABCB10	XLOC_009969	2271	coding	coding	coding	noncoding
MSTRG.19219.8	ABCB10	XLOC_009969	1953	coding	coding	noncoding	noncoding
MSTRG.19388.3	TTC13	XLOC_010042	3832	coding	coding	coding	noncoding
MSTRG.19388.5	TTC13	XLOC_010042	2761	coding	coding	coding	noncoding
MSTRG.19400.3	C1orf131	XLOC_010056	1285	coding	coding	noncoding	noncoding
NM_001164550.2	DISC1	XLOC_010078	2857	coding	noncoding	coding	coding
MSTRG.19824.3	PCNX2	XLOC_010179	3772	coding	noncoding	coding	noncoding
MSTRG.19631.1	TARBP1	XLOC_010223	5359	coding	noncoding	noncoding	noncoding
NM_001037277.1	GGPS1	XLOC_010269	2747	coding	coding	coding	coding
MSTRG.19888.1	LYST	XLOC_010279	15231	coding	coding	coding	coding
XM_024452537.1	SDCCAG8	XLOC_010488	7971	coding	coding	coding	coding
MSTRG.20414.4	ADSS	XLOC_010532	2416	coding	coding	coding	noncoding
MSTRG.20414.7	ADSS	XLOC_010532	673	coding	coding	noncoding	noncoding

MSTRG.20553.5	AHCTF1	XLOC_010592	7782	coding	noncoding	noncoding	noncoding
MSTRG.20553.8	AHCTF1	XLOC_010592	5580	coding	coding	coding	noncoding
MSTRG.20553.11	AHCTF1	XLOC_010592	927	coding	coding	noncoding	noncoding
MSTRG.20754.2	SH3BP5L	XLOC_010704	2978	coding	coding	coding	noncoding
NM_001353642.2	NADK	XLOC_010848	3296	coding	coding	coding	coding
XM_017001745.1	DNAJC11	XLOC_011043	3146	coding	coding	coding	coding
NM_001561.5	TNFRSF9	XLOC_011096	5993	coding	noncoding	coding	coding
MSTRG.626.5	PARK7	XLOC_011098	815	coding	coding	noncoding	noncoding
NM_020248.3	CTNNBIP1	XLOC_011196	3006	coding	coding	coding	noncoding
MSTRG.1073.2	TNFRSF8	XLOC_011281	1822	coding	noncoding	coding	noncoding
MSTRG.1200.4	DDI2	XLOC_011428	4965	coding	coding	noncoding	noncoding
MSTRG.1523.6	PADI4	XLOC_011518	2994	coding	coding	coding	coding
MSTRG.1601.2	ARHGEF10L	XLOC_011523	1512	coding	coding	coding	noncoding
XM_011541185.3	TMCO4	XLOC_011611	3112	coding	coding	coding	coding
NM_002167.5	ID3	XLOC_011795	950	coding	coding	coding	coding
MSTRG.2051.3	RPL11	XLOC_011801	819	coding	coding	coding	coding
NR_135778.1	RSRP1	XLOC_011890	2367	coding	coding	coding	coding
MSTRG.2241.2	LDLRAP1	XLOC_011897	4309	coding	coding	coding	coding
MSTRG.2347.3	RPS6KA1	XLOC_011946	3868	coding	coding	coding	coding
NM_001164721.1	PTAFR	XLOC_012041	4191	coding	coding	coding	coding
MSTRG.2530.3	PHACTR4	XLOC_012047	1057	coding	noncoding	coding	noncoding
MSTRG.2599.1	GMEB1	XLOC_012060	2771	coding	coding	coding	noncoding
MSTRG.2599.4	GMEB1	XLOC_012060	2276	coding	coding	coding	coding
XM_011542098.2	LAPTM5	XLOC_012127	2072	coding	coding	coding	coding
MSTRG.2778.1	PUM1	XLOC_012144	447	coding	noncoding	noncoding	noncoding
MSTRG.2852.1	TMEM39B	XLOC_012195	1772	coding	coding	coding	coding
NM_001300826.2	RNF19B	XLOC_012239	2677	coding	coding	coding	coding
MSTRG.2988.2	ZNF362	XLOC_012273	1623	coding	coding	coding	coding
MSTRG.3292.5	AGO3	XLOC_012383	1472	coding	coding	noncoding	noncoding
MSTRG.3350.7	THRAP3	XLOC_012398	3668	coding	coding	coding	noncoding
MSTRG.3350.10	THRAP3	XLOC_012398	6062	coding	coding	coding	noncoding
MSTRG.3365.1	STK40	XLOC_012409	283	coding	noncoding	noncoding	noncoding
MSTRG.3666.2	MACF1	XLOC_012563	4786	coding	coding	noncoding	noncoding
MSTRG.3666.18	MACF1	XLOC_012563	24918	coding	coding	coding	coding
MSTRG.3574.4	BMP8B	XLOC_012581	1154	coding	coding	noncoding	noncoding
MSTRG.3608.5	RLF	XLOC_012602	2745	coding	coding	noncoding	noncoding
MSTRG.3701.14	SMAP2	XLOC_012607	10173	coding	noncoding	coding	noncoding
MSTRG.3905.5	CCDC30	XLOC_012731	1206	coding	coding	coding	noncoding
MSTRG.3876.4	ZNF691	XLOC_012762	1525	coding	coding	coding	noncoding
MSTRG.4348.3	AKR1A1	XLOC_012911	1298	coding	coding	noncoding	noncoding
MSTRG.4944.7	OSBPL9	XLOC_013177	1535	coding	coding	noncoding	noncoding
XM_006710599.3	FAM151A	XLOC_013298	2054	coding	coding	coding	coding

MSTRG.5853.1	ANGPTL3	XLOC_013628	321	coding	noncoding	noncoding	noncoding
MSTRG.6082.11	DNAJC6	XLOC_013751	1670	coding	coding	noncoding	noncoding
MSTRG.6167.1	IL23R	XLOC_013875	623	coding	noncoding	noncoding	noncoding
MSTRG.6357.1	IL12RB2	XLOC_013888	228	coding	noncoding	noncoding	noncoding
MSTRG.6648.8	SRSF11	XLOC_014020	7140	coding	noncoding	noncoding	noncoding
MSTRG.7256.1	ST6GALNAC3	XLOC_014179	1013	coding	coding	coding	coding
MSTRG.7256.3	ST6GALNAC3	XLOC_014179	860	coding	coding	noncoding	noncoding
MSTRG.7151.3	MIGA1	XLOC_014249	2012	coding	coding	coding	coding
MSTRG.7229.6	IFI44L	XLOC_014283	1216	coding	noncoding	noncoding	noncoding
MSTRG.7229.13	IFI44	XLOC_014283	2718	coding	noncoding	noncoding	noncoding
MSTRG.7334.3	PRKACB	XLOC_014456	1260	coding	coding	noncoding	noncoding
XM_024446127.1	LPAR3	XLOC_014499	4009	coding	coding	coding	coding
XM_011541742.1	MCOLN3	XLOC_014512	2984	coding	coding	coding	coding
MSTRG.7612.1		XLOC_014519	307	coding	noncoding	noncoding	noncoding
MSTRG.7921.1		XLOC_014658	250	coding	noncoding	noncoding	noncoding
NM_001162536.3	RBMXL1	XLOC_014662	4756	coding	coding	coding	coding
MSTRG.8342.1		XLOC_014683	221	coding	coding	noncoding	noncoding
MSTRG.8387.1		XLOC_014723	288	coding	noncoding	noncoding	noncoding
MSTRG.8407.1		XLOC_014740	631	coding	coding	noncoding	noncoding
MSTRG.8140.3	LRRC8B	XLOC_014770	2887	coding	coding	noncoding	coding
MSTRG.8140.4	LRRC8B	XLOC_014770	6410	coding	coding	noncoding	coding
MSTRG.7995.1	LOC107985744	XLOC_014795	292	coding	noncoding	noncoding	noncoding
MSTRG.8046.1	LRRC8D	XLOC_014800	7510	coding	coding	coding	noncoding
MSTRG.8273.1	BTBD8	XLOC_014917	249	coding	noncoding	noncoding	noncoding
MSTRG.8673.3	ABCD3	XLOC_015060	3410	coding	coding	noncoding	noncoding
MSTRG.8773.12	TLCD4	XLOC_015087	1037	coding	coding	coding	noncoding
MSTRG.8960.3	MFSD14A	XLOC_015356	1764	coding	coding	coding	noncoding
MSTRG.8987.7	RTCA	XLOC_015373	3308	coding	coding	noncoding	noncoding
MSTRG.9151.2	DPH5	XLOC_015401	17591	coding	coding	coding	coding
MSTRG.9569.3	PRPF38B	XLOC_015629	2068	coding	coding	coding	coding
MSTRG.9569.10	PRPF38B	XLOC_015629	1105	coding	coding	noncoding	noncoding
MSTRG.9829.1	KCNC4	XLOC_015718	307	coding	noncoding	noncoding	noncoding
MSTRG.9872.4	RBM15	XLOC_015730	5575	coding	coding	coding	coding
MSTRG.9930.1	LRIF1	XLOC_015772	467	coding	noncoding	noncoding	noncoding
MSTRG.9941.1	LRIF1	XLOC_015780	313	coding	noncoding	noncoding	noncoding
NM_019099.5	INKA2	XLOC_015822	5951	coding	coding	coding	coding
MSTRG.10205.11	HIPK1	XLOC_015910	10317	coding	coding	coding	coding
MSTRG.10293.1		XLOC_015947	473	coding	noncoding	noncoding	noncoding
MSTRG.10419.17	SLC22A15	XLOC_016014	561	coding	noncoding	noncoding	noncoding
MSTRG.10584.4	MAN1A2	XLOC_016091	3825	coding	coding	coding	noncoding
MSTRG.10584.8	MAN1A2	XLOC_016091	1617	coding	coding	noncoding	noncoding
XR_946574.1	SPAG17	XLOC_016119	7082	coding	coding	coding	coding

MSTRG.11366.14	NBPF8	XLOC_016278	4109	coding	coding	noncoding	noncoding
MSTRG.11366.34	NBPF26	XLOC_016278	490	coding	coding	noncoding	noncoding
MSTRG.10857.1		XLOC_016326	733	coding	noncoding	noncoding	noncoding
MSTRG.11014.1	LOC199882	XLOC_016421	592	coding	noncoding	noncoding	noncoding
NM_001364007.1	NOTCH2NLB	XLOC_016662	8334	coding	coding	coding	coding
MSTRG.12230.8	NBPF19	XLOC_016707	14216	coding	noncoding	coding	coding
MSTRG.12230.19	NBPF19	XLOC_016707	20790	coding	coding	coding	coding
MSTRG.12230.20	NBPF19	XLOC_016707	14326	coding	noncoding	coding	coding
MSTRG.12230.24	LOC653513	XLOC_016707	5671	coding	noncoding	coding	noncoding
MSTRG.12230.27	LOC653513	XLOC_016707	4389	coding	noncoding	coding	noncoding
MSTRG.12230.28	LOC653513	XLOC_016707	4009	coding	noncoding	coding	noncoding
MSTRG.12247.11	NBPF19	XLOC_016710	11109	coding	coding	coding	noncoding
MSTRG.12247.15	NOTCH2NLC	XLOC_016710	18155	coding	noncoding	coding	noncoding
MSTRG.12247.34	NBPF19	XLOC_016710	200	coding	coding	noncoding	noncoding
MSTRG.12247.35	NBPF19	XLOC_016710	2498	coding	coding	noncoding	coding
MSTRG.12247.37	NBPF19	XLOC_016710	1949	coding	coding	noncoding	noncoding
MSTRG.12247.41	NBPF19	XLOC_016710	570	coding	coding	noncoding	noncoding
MSTRG.11486.1	LOC102723955	XLOC_016744	453	coding	coding	noncoding	noncoding
MSTRG.11512.3	HIST2H2AB	XLOC_016757	1061	coding	coding	coding	coding
MSTRG.11541.2	VPS45	XLOC_016771	2512	coding	coding	noncoding	noncoding
MSTRG.11548.4	PLEKHO1	XLOC_016773	3181	coding	coding	coding	coding
MSTRG.11565.5	PRPF3	XLOC_016783	2443	coding	coding	coding	coding
NM_182763.2	MCL1	XLOC_016795	3837	coding	noncoding	coding	coding
MSTRG.11631.8	SETDB1	XLOC_016825	4314	coding	coding	coding	coding
MSTRG.11631.9	SETDB1	XLOC_016825	4236	coding	coding	coding	coding
MSTRG.11964.2	ATP8B2	XLOC_017033	5364	coding	coding	coding	coding
XR_001737388.2	THBS3	XLOC_017092	4957	coding	coding	coding	coding
MSTRG.12321.3	RUSC1-AS1	XLOC_017099	1102	coding	coding	coding	noncoding
NM_001198906.2	YY1AP1	XLOC_017126	2694	coding	noncoding	coding	coding
XM_005245587.3	ARHGEF2	XLOC_017140	4253	coding	coding	coding	coding
XM_011509453.2	HDGF	XLOC_017206	2208	coding	noncoding	coding	coding
NM_005240.2	ETV3	XLOC_017231	1617	coding	coding	coding	coding
MSTRG.12827.1	PYHIN1	XLOC_017312	17332	coding	coding	noncoding	noncoding
MSTRG.12827.29	IFI16	XLOC_017312	2522	coding	coding	coding	noncoding
MSTRG.12827.35	LOC105371461	XLOC_017312	7987	coding	coding	coding	noncoding
MSTRG.12827.36	IFI16	XLOC_017312	6972	coding	noncoding	coding	noncoding
MSTRG.12885.2	LOC729867	XLOC_017371	2674	coding	coding	coding	noncoding
XM_011510171.2	CD48	XLOC_017432	1197	coding	coding	coding	coding
MSTRG.13211.1	FCGR2A	XLOC_017527	2882	coding	coding	noncoding	coding
MSTRG.13211.4	FCGR2A	XLOC_017527	3358	coding	coding	noncoding	noncoding
MSTRG.13211.40	FCGR2B	XLOC_017531	1943	coding	coding	noncoding	noncoding
MSTRG.13163.2	ATF6	XLOC_017543	3561	coding	coding	noncoding	noncoding

MSTRG.13163.11	ATF6	XLOC_017543	1718	coding	coding	coding	noncoding
MSTRG.13458.1	MGST3	XLOC_017691	300	coding	noncoding	noncoding	noncoding
MSTRG.13570.1	TADA1	XLOC_017739	8044	coding	coding	coding	coding
MSTRG.13770.2	MPC2	XLOC_017814	4731	coding	coding	coding	noncoding
MSTRG.13900.7	BLZF1	XLOC_017891	1633	coding	noncoding	noncoding	noncoding
MSTRG.13929.1	SELL	XLOC_017916	2474	coding	coding	coding	coding
MSTRG.13957.1	SCYL3	XLOC_017925	668	coding	coding	coding	noncoding
MSTRG.14089.9	PRRC2C	XLOC_017998	8901	coding	coding	coding	coding
MSTRG.14671.23	LOC105371622	XLOC_018138	6209	coding	noncoding	noncoding	noncoding
NM_014656.3	KIAA0040	XLOC_018153	4548	coding	noncoding	coding	coding
MSTRG.14934.1		XLOC_018241	260	coding	noncoding	noncoding	noncoding
MSTRG.14789.4	TOR3A	XLOC_018369	1906	coding	coding	coding	coding
MSTRG.14962.5	TOR1AIP1	XLOC_018396	3101	coding	coding	noncoding	noncoding
MSTRG.14972.2	CEP350	XLOC_018401	5604	coding	noncoding	coding	coding
MSTRG.15300.8	NPL	XLOC_018562	2156	coding	coding	coding	noncoding
MSTRG.15300.6	NPL	XLOC_018562	2540	coding	coding	coding	noncoding
MSTRG.15300.11	NPL	XLOC_018562	2864	coding	coding	noncoding	noncoding
MSTRG.15333.8	SMG7	XLOC_018592	3640	coding	coding	coding	noncoding
MSTRG.15421.3	RNF2	XLOC_018704	2694	coding	coding	noncoding	noncoding
MSTRG.15585.3	SWT1	XLOC_018711	3884	coding	coding	noncoding	noncoding
MSTRG.15585.4	IVNS1ABP	XLOC_018711	4564	coding	coding	noncoding	noncoding
MSTRG.15585.6	SWT1	XLOC_018711	3821	coding	coding	noncoding	noncoding
MSTRG.15585.11	SWT1	XLOC_018711	2698	coding	coding	noncoding	noncoding
MSTRG.16719.1		XLOC_019000	285	coding	noncoding	noncoding	noncoding
MSTRG.16819.1		XLOC_019080	296	coding	noncoding	noncoding	noncoding
XR_001738352.1	LOC107985242	XLOC_019090	1899	coding	noncoding	noncoding	noncoding
MSTRG.16393.1	PTPRC	XLOC_019264	31501	coding	coding	noncoding	noncoding
MSTRG.16393.2	PTPRC	XLOC_019264	11989	coding	coding	coding	noncoding
MSTRG.16393.5	PTPRC	XLOC_019264	12338	coding	coding	noncoding	noncoding
MSTRG.16393.4	PTPRC	XLOC_019264	12319	coding	coding	noncoding	noncoding
MSTRG.16393.7	PTPRC	XLOC_019264	11048	coding	coding	noncoding	noncoding
XM_017001407.1	ADIPOR1	XLOC_019442	2156	coding	coding	coding	coding
MSTRG.17066.17	ZC3H11A	XLOC_019483	1446	coding	coding	noncoding	noncoding
MSTRG.17066.23	ZC3H11A	XLOC_019483	12032	coding	coding	coding	coding
NM_001276320.1	YOD1	XLOC_019688	6291	coding	noncoding	coding	coding
MSTRG.17469.4	CD55	XLOC_019702	7689	coding	coding	noncoding	noncoding
MSTRG.17817.16	CR1	XLOC_019714	6179	coding	coding	noncoding	noncoding
MSTRG.17817.21	CR1	XLOC_019714	8362	coding	coding	noncoding	noncoding
MSTRG.17817.20	CR1	XLOC_019714	4091	coding	coding	noncoding	noncoding
MSTRG.17794.1	RCOR3	XLOC_019853	7471	coding	noncoding	noncoding	noncoding
MSTRG.18007.7	PPP2R5A	XLOC_019911	1231	coding	noncoding	coding	coding
MSTRG.18007.9	PPP2R5A	XLOC_019911	3851	coding	noncoding	noncoding	noncoding

MSTRG.18007.10	PPP2R5A	XLOC_019911	6934	coding	noncoding	noncoding	noncoding
MSTRG.18007.11	PPP2R5A	XLOC_019911	3712	coding	noncoding	noncoding	noncoding
MSTRG.18519.3	MARC1	XLOC_020179	2137	coding	coding	coding	noncoding
MSTRG.18793.1	LOC100287497	XLOC_020317	556	coding	noncoding	noncoding	noncoding
MSTRG.18802.1	FBXO28	XLOC_020323	467	coding	noncoding	noncoding	noncoding
MSTRG.18802.3	DEGS1	XLOC_020323	2051	coding	coding	coding	noncoding
MSTRG.18829.4	CNIH4	XLOC_020336	3035	coding	coding	coding	noncoding
XM_005273125.3	LBR	XLOC_020371	3650	coding	coding	coding	coding
MSTRG.18945.2	H3F3A	XLOC_020406	1167	coding	coding	coding	noncoding
MSTRG.19453.2	SPRTN	XLOC_020685	1636	coding	noncoding	noncoding	noncoding
MSTRG.19736.4	DISC1	XLOC_020725	1850	coding	noncoding	coding	noncoding
MSTRG.19796.1	LOC105373171	XLOC_020733	258	coding	noncoding	noncoding	noncoding
NR_125961.1	COA6-AS1	XLOC_020827	575	coding	noncoding	noncoding	noncoding
NM_000081.3	LYST	XLOC_020896	13497	coding	coding	coding	coding
MSTRG.19931.1	EDARADD	XLOC_020972	245	coding	noncoding	noncoding	noncoding
MSTRG.20077.2	MTR	XLOC_020986	3180	coding	coding	coding	coding
MSTRG.20077.6	MTR	XLOC_020986	3746	coding	coding	coding	coding
MSTRG.20226.1	EXO1	XLOC_021108	248	coding	noncoding	noncoding	noncoding
MSTRG.20227.1	EXO1	XLOC_021109	270	coding	noncoding	noncoding	noncoding
MSTRG.20358.1	SDCCAG8	XLOC_021142	340	coding	noncoding	noncoding	noncoding
MSTRG.20530.5	CNST	XLOC_021367	2421	coding	noncoding	coding	noncoding
MSTRG.20530.10	CNST	XLOC_021367	3723	coding	coding	coding	noncoding
MSTRG.20522.2	SCCPDH	XLOC_021374	1204	coding	coding	coding	noncoding
XR_426916.2	AHCTF1	XLOC_021380	8478	coding	coding	coding	coding
MSTRG.21504.11	RSAD2	XLOC_021725	3116	coding	coding	coding	coding
NM_001039362.2	ATP6V1C2	XLOC_021964	3259	coding	coding	coding	coding
MSTRG.21989.3	NBAS	XLOC_022117	1370	coding	noncoding	noncoding	noncoding
MSTRG.21989.6	NBAS	XLOC_022117	4754	coding	coding	coding	noncoding
MSTRG.22055.4	FAM49A	XLOC_022159	10425	coding	coding	noncoding	noncoding
MSTRG.22055.3	FAM49A	XLOC_022159	23202	coding	coding	coding	noncoding
MSTRG.22055.5	FAM49A	XLOC_022159	23137	coding	coding	coding	noncoding
MSTRG.22055.12	FAM49A	XLOC_022159	25237	coding	noncoding	coding	noncoding
MSTRG.22411.1		XLOC_022287	249	coding	coding	noncoding	noncoding
MSTRG.22444.1		XLOC_022363	348	coding	coding	noncoding	noncoding
MSTRG.23058.1	ATAD2B	XLOC_022485	899	coding	noncoding	noncoding	noncoding
MSTRG.23062.2	UBXN2A	XLOC_022487	15707	coding	coding	coding	coding
MSTRG.23062.6	ATAD2B	XLOC_022487	6505	coding	coding	coding	noncoding
MSTRG.23062.9	ATAD2B	XLOC_022487	4335	coding	coding	noncoding	noncoding
MSTRG.23062.12	ATAD2B	XLOC_022487	2738	coding	coding	coding	noncoding
MSTRG.23062.13	ATAD2B	XLOC_022487	1464	coding	noncoding	coding	noncoding
NM_001281512.1	HADHB	XLOC_022667	2143	coding	coding	coding	coding
MSTRG.23318.2	OTOF	XLOC_022677	1098	coding	coding	coding	noncoding

MSTRG.23318.1	OTOF	XLOC_022677	5144	coding	coding	coding	coding
MSTRG.23369.3	ABHD1	XLOC_022706	3175	coding	coding	coding	coding
MSTRG.23369.2	ABHD1	XLOC_022706	3334	coding	coding	coding	coding
MSTRG.23419.5	EIF2B4	XLOC_022723	1443	coding	coding	coding	noncoding
NM_001329113.1	BABAM2	XLOC_022759	2032	coding	coding	coding	coding
NM_005253.4	FOSL2	XLOC_022813	6713	coding	coding	coding	coding
NM_024692.6	CLIP4	XLOC_022856	4299	coding	coding	coding	coding
XR_002959339.1	CLIP4	XLOC_022856	5364	coding	coding	coding	coding
MSTRG.23831.21	SLC30A6	XLOC_023016	5663	coding	coding	coding	coding
XM_017004558.1	BIRC6	XLOC_023021	15743	coding	coding	coding	coding
MSTRG.24171.1	NDUFAF7	XLOC_023149	4023	coding	noncoding	noncoding	noncoding
MSTRG.24250.1	LOC375196	XLOC_023257	237	coding	noncoding	coding	noncoding
MSTRG.24253.12	SOS1	XLOC_023259	4255	coding	noncoding	coding	noncoding
MSTRG.24758.7	SLC8A1	XLOC_023309	4033	coding	coding	coding	coding
MSTRG.24758.26	SLC8A1	XLOC_023309	15975	coding	coding	noncoding	coding
XR_001738710.1	EML4	XLOC_023378	5597	coding	coding	coding	coding
MSTRG.24859.3	LOC107985877	XLOC_023458	5870	coding	coding	noncoding	noncoding
MSTRG.24859.6	THADA	XLOC_023458	5214	coding	coding	noncoding	noncoding
MSTRG.25102.8	CAMKMT	XLOC_023489	8623	coding	coding	coding	coding
MSTRG.25102.11	CAMKMT	XLOC_023489	3142	coding	coding	coding	coding
MSTRG.24907.7	SRBD1	XLOC_023516	2060	coding	coding	noncoding	noncoding
MSTRG.25027.1	ATP6V1E2	XLOC_023567	307	coding	noncoding	noncoding	noncoding
MSTRG.25133.13	FBXO11	XLOC_023639	4112	coding	coding	noncoding	noncoding
XM_005264283.2	FOXN2	XLOC_023676	5658	coding	coding	coding	coding
XM_006712002.3	FOXN2	XLOC_023676	5354	coding	coding	coding	coding
MSTRG.25641.3	ACYP2	XLOC_023857	8681	coding	coding	coding	coding
MSTRG.25532.16	RTN4	XLOC_023883	8453	coding	noncoding	noncoding	noncoding
NM_002954.5	RPS27A	XLOC_023905	1068	coding	coding	coding	coding
MSTRG.25603.5	CCDC88A	XLOC_023907	3749	coding	noncoding	noncoding	coding
MSTRG.25681.3	PPP4R3B	XLOC_023914	5483	coding	coding	coding	noncoding
MSTRG.25681.6	PPP4R3B	XLOC_023914	1301	coding	coding	noncoding	noncoding
MSTRG.25894.1	REL	XLOC_024160	276	coding	noncoding	noncoding	noncoding
MSTRG.25896.2	PUS10	XLOC_024162	247	coding	noncoding	noncoding	noncoding
MSTRG.26169.3	AHSA2P	XLOC_024171	14289	coding	coding	coding	coding
MSTRG.26200.5	XPO1	XLOC_024174	4407	coding	coding	noncoding	noncoding
MSTRG.26311.3	SERTAD2	XLOC_024322	4620	coding	noncoding	noncoding	coding
MSTRG.26298.1	LINC02245	XLOC_024340	305	coding	noncoding	noncoding	noncoding
NM_001005386.2	ACTR2	XLOC_024375	3934	coding	coding	coding	coding
NM_001364819.1	ARHGAP25	XLOC_024515	2966	coding	coding	coding	coding
NM_001202514.1	MXD1	XLOC_024614	5587	coding	coding	coding	coding
MSTRG.26840.4	PCBP1-AS1	XLOC_024622	841	coding	coding	noncoding	noncoding
MSTRG.26884.3	TIA1	XLOC_024652	4187	coding	coding	noncoding	noncoding

NM_014497.5	ZNF638	XLOC_024708	6487	coding	coding	coding	coding
MSTRG.27195.1	ZNF638	XLOC_024737	205	coding	noncoding	noncoding	noncoding
MSTRG.27154.4	TPRKB	XLOC_024837	663	coding	coding	noncoding	noncoding
MSTRG.27807.6	TRABD2A	XLOC_025137	2762	coding	coding	noncoding	noncoding
MSTRG.27963.10	TGOLN2	XLOC_025160	5855	coding	coding	coding	noncoding
MSTRG.28036.3	ST3GAL5	XLOC_025195	1536	coding	coding	coding	noncoding
MSTRG.28427.5	IGKC	XLOC_025345	1284	coding	coding	coding	coding
MSTRG.28427.20	IGKC	XLOC_025345	879	coding	coding	coding	coding
MSTRG.28427.23	IGKC	XLOC_025345	918	coding	coding	coding	coding
MSTRG.28427.26	IGKC	XLOC_025345	928	coding	coding	coding	coding
MSTRG.28427.37	IGKC	XLOC_025345	1904	coding	coding	coding	coding
MSTRG.28427.42	IGKC	XLOC_025345	1017	coding	coding	coding	coding
NM_001319092.1	ARID5A	XLOC_025473	2549	coding	coding	coding	coding
MSTRG.28647.3	KANSL3	XLOC_025480	346	coding	noncoding	noncoding	noncoding
MSTRG.28716.1	LOC107985920	XLOC_025536	270	coding	coding	noncoding	noncoding
MSTRG.28767.8	ANKRD36B	XLOC_025538	4403	coding	coding	coding	noncoding
MSTRG.28767.13	ANKRD36B	XLOC_025538	1474	coding	coding	coding	noncoding
MSTRG.28985.4	UNC50	XLOC_025598	5941	coding	coding	coding	coding
MSTRG.28985.6	UNC50	XLOC_025598	8877	coding	coding	coding	coding
MSTRG.28985.10	MGAT4A	XLOC_025598	5680	coding	noncoding	noncoding	noncoding
MSTRG.28932.1	MITD1	XLOC_025642	1144	coding	coding	noncoding	noncoding
NM_015904.4	EIF5B	XLOC_025653	5741	coding	coding	coding	coding
MSTRG.29015.6	REV1	XLOC_025653	2446	coding	coding	coding	noncoding
MSTRG.29015.7	REV1	XLOC_025653	2224	coding	coding	coding	noncoding
MSTRG.29184.2	RNF149	XLOC_025708	2967	coding	coding	coding	noncoding
MSTRG.29184.8	CNOT11	XLOC_025708	15853	coding	coding	coding	noncoding
MSTRG.29184.21	RNF149	XLOC_025708	6390	coding	coding	coding	noncoding
MSTRG.30035.10	MIR4435-2HG	XLOC_026171	2624	coding	noncoding	noncoding	noncoding
MSTRG.30035.30	MIR4435-2HG	XLOC_026171	4484	coding	noncoding	coding	noncoding
MSTRG.30024.5	ANAPC1	XLOC_026185	3363	coding	coding	noncoding	noncoding
NM_173842.3	IL1RN	XLOC_026256	1704	coding	coding	coding	coding
MSTRG.30235.1	PAX8	XLOC_026267	380	coding	noncoding	noncoding	noncoding
MSTRG.30551.7	CCDC93	XLOC_026426	4441	coding	coding	coding	noncoding
MSTRG.31093.3	BIN1	XLOC_026684	1850	coding	coding	coding	coding
MSTRG.31361.1	IMP4	XLOC_026813	5315	coding	coding	coding	coding
MSTRG.31893.1	TMEM163	XLOC_026999	205	coding	noncoding	noncoding	noncoding
MSTRG.31679.2	DARS	XLOC_027109	1947	coding	coding	noncoding	noncoding
MSTRG.31813.1		XLOC_027196	442	coding	noncoding	noncoding	noncoding
MSTRG.32185.1		XLOC_027231	294	coding	noncoding	noncoding	noncoding
MSTRG.32239.1		XLOC_027276	507	coding	noncoding	noncoding	noncoding
MSTRG.32689.3	GTDC1	XLOC_027631	2702	coding	coding	noncoding	noncoding
MSTRG.32434.1	ZEB2	XLOC_027633	50789	coding	noncoding	coding	coding

MSTRG.32434.2	ZEB2	XLOC_027633	51595	coding	noncoding	coding	coding
MSTRG.32434.19	ZEB2	XLOC_027633	25107	coding	noncoding	coding	noncoding
MSTRG.32434.20	ZEB2	XLOC_027633	7340	coding	noncoding	noncoding	noncoding
MSTRG.32482.1	EPC2	XLOC_027813	279	coding	coding	noncoding	noncoding
MSTRG.32593.2	LOC101929231	XLOC_027864	1112	coding	coding	noncoding	noncoding
MSTRG.33386.2	CACNB4	XLOC_027953	3099	coding	coding	noncoding	coding
MSTRG.32981.1	LINC01876	XLOC_028078	303	coding	noncoding	noncoding	noncoding
NM_000408.5	GPD2	XLOC_028104	5812	coding	coding	coding	coding
MSTRG.33437.2	ERMN	XLOC_028140	690	coding	noncoding	noncoding	noncoding
MSTRG.34306.4	LOC643072	XLOC_028256	10445	coding	coding	coding	noncoding
MSTRG.34306.10	BAZ2B	XLOC_028256	6709	coding	coding	coding	noncoding
MSTRG.34306.19	LOC643072	XLOC_028256	8437	coding	coding	coding	noncoding
MSTRG.34306.29	LOC643072	XLOC_028256	5021	coding	coding	coding	noncoding
XM_017004719.1	7-Mar	XLOC_028256	3373	coding	coding	coding	coding
XM_017004721.1	7-Mar	XLOC_028256	3311	coding	coding	coding	coding
MSTRG.33939.2	DPP4	XLOC_028361	576	coding	coding	noncoding	noncoding
MSTRG.34174.4	GALNT3	XLOC_028506	2455	coding	coding	noncoding	noncoding
NM_006063.3	KLHL41	XLOC_028641	2460	coding	coding	coding	coding
MSTRG.34358.1	PPIG	XLOC_028647	278	coding	noncoding	noncoding	noncoding
MSTRG.34613.2	METTL8	XLOC_028726	748	coding	noncoding	noncoding	noncoding
MSTRG.34680.1		XLOC_028753	214	coding	noncoding	noncoding	noncoding
MSTRG.35013.11	WIPF1	XLOC_028884	24267	coding	coding	coding	noncoding
MSTRG.35013.12	WIPF1	XLOC_028884	13742	coding	coding	coding	noncoding
MSTRG.35013.15	WIPF1	XLOC_028884	8006	coding	coding	coding	noncoding
MSTRG.35013.29	WIPF1	XLOC_028884	8497	coding	noncoding	coding	noncoding
MSTRG.35099.21	NFE2L2	XLOC_028952	2316	coding	coding	noncoding	noncoding
MSTRG.35897.9	TTN-AS1	XLOC_029016	8446	coding	coding	coding	noncoding
NR_120594.1	LOC101927055	XLOC_029026	1956	coding	coding	noncoding	noncoding
MSTRG.35917.7	TTN	XLOC_029026	6515	coding	coding	coding	coding
MSTRG.35237.2	SESTD1	XLOC_029030	3374	coding	coding	coding	noncoding
XR_001738911.2	ITPRID2	XLOC_029164	5606	coding	coding	coding	coding
MSTRG.36053.1	LOC107985783	XLOC_029286	957	coding	noncoding	noncoding	noncoding
MSTRG.36054.1	LOC107985783	XLOC_029287	202	coding	noncoding	noncoding	noncoding
MSTRG.36093.5	LOC105373785	XLOC_029320	1225	coding	noncoding	noncoding	noncoding
MSTRG.36135.3	ZC3H15	XLOC_029331	1377	coding	coding	noncoding	coding
NM_001353497.1	ASNSD1	XLOC_029449	2417	coding	coding	coding	coding
NM_001042520.1	C2orf88	XLOC_029464	3991	coding	coding	coding	noncoding
MSTRG.36204.1	C2orf88	XLOC_029472	250	coding	noncoding	noncoding	noncoding
MSTRG.36327.11	GLS	XLOC_029538	16631	coding	coding	coding	coding
MSTRG.36327.17	STAT1	XLOC_029538	2536	coding	coding	noncoding	coding
MSTRG.36327.18	STAT1	XLOC_029538	6110	coding	coding	noncoding	noncoding
MSTRG.36605.2	HECW2	XLOC_029768	1698	coding	coding	noncoding	noncoding

MSTRG.36815.9	ANKRD44	XLOC_029810	4828	coding	coding	coding	coding
MSTRG.36815.16	ANKRD44	XLOC_029810	10613	coding	coding	coding	noncoding
MSTRG.36732.4	HSPD1	XLOC_029817	1011	coding	coding	coding	coding
MSTRG.36851.1	FTCDNL1	XLOC_029929	249	coding	coding	noncoding	noncoding
MSTRG.36877.2	TYW5	XLOC_029932	213	coding	noncoding	noncoding	noncoding
MSTRG.36890.1	KCTD18	XLOC_029955	2214	coding	coding	noncoding	noncoding
MSTRG.36998.4	PPIL3	XLOC_029971	1033	coding	coding	noncoding	coding
MSTRG.37114.2	LOC105373835	XLOC_029972	3287	coding	coding	noncoding	noncoding
MSTRG.37114.15	LOC105373835	XLOC_029972	5451	coding	coding	coding	noncoding
NM_001127183.4	CFLAR	XLOC_029972	14480	coding	coding	coding	coding
NM_005759.6	ABI2	XLOC_030110	21925	coding	coding	coding	coding
XM_017003897.1	RUFY4	XLOC_030799	4307	coding	coding	coding	coding
NM_001168298.2	CXCR2	XLOC_030801	2656	coding	coding	coding	coding
MSTRG.38589.7	TMBIM1	XLOC_030811	2064	coding	coding	coding	coding
MSTRG.38607.1	CATIP	XLOC_030817	310	coding	noncoding	noncoding	noncoding
XM_017005249.2	CNOT9	XLOC_030826	1556	coding	coding	noncoding	coding
MSTRG.38725.11	TUBA4A	XLOC_030871	1944	coding	coding	coding	coding
MSTRG.38725.12	TUBA4A	XLOC_030871	1171	coding	coding	coding	noncoding
MSTRG.39463.13	TRIP12	XLOC_031236	1935	coding	noncoding	coding	coding
MSTRG.39435.1	SLC16A14	XLOC_031246	286	coding	noncoding	noncoding	noncoding
MSTRG.39865.2	SP110	XLOC_031254	11744	coding	coding	coding	noncoding
MSTRG.39865.13	SP110	XLOC_031254	16653	coding	coding	coding	noncoding
MSTRG.39515.5	CAB39	XLOC_031296	1156	coding	coding	coding	coding
NM_080678.2	UBE2F	XLOC_031639	2206	coding	coding	coding	coding
MSTRG.40417.1	ANKMY1	XLOC_031747	724	coding	coding	noncoding	noncoding
MSTRG.40467.24	SNED1	XLOC_031787	2940	coding	coding	coding	noncoding
MSTRG.40570.9	SEPTIN2	XLOC_031797	8321	coding	coding	coding	coding
MSTRG.21302.1		XLOC_032210	279	coding	noncoding	noncoding	noncoding
NM_001348743.2	KIDINS220	XLOC_032214	8643	coding	coding	coding	coding
NM_001321265.2	MBOAT2	XLOC_032231	7476	coding	coding	coding	coding
MSTRG.21427.3	ADAM17	XLOC_032266	1171	coding	coding	noncoding	noncoding
MSTRG.21741.1		XLOC_032454	247	coding	noncoding	noncoding	noncoding
XM_024453164.1	FAM49A	XLOC_032547	4653	coding	coding	coding	coding
MSTRG.22907.8	MFSD2B	XLOC_033029	2375	coding	coding	coding	coding
MSTRG.22916.13	FAM228B	XLOC_033033	883	coding	noncoding	noncoding	noncoding
MSTRG.23444.1	ZNF512	XLOC_033241	3916	coding	coding	coding	noncoding
MSTRG.23611.8	BABAM2-AS1	XLOC_033252	1615	coding	coding	coding	coding
MSTRG.23674.2	CLIP4	XLOC_033321	338	coding	noncoding	noncoding	noncoding
MSTRG.23595.4	YPEL5	XLOC_033333	2607	coding	coding	noncoding	noncoding
MSTRG.23818.3	SPAST	XLOC_033415	1849	coding	coding	coding	noncoding
MSTRG.24020.15	BIRC6	XLOC_033420	5405	coding	coding	noncoding	noncoding
MSTRG.24020.18	BIRC6	XLOC_033420	14637	coding	coding	coding	noncoding

MSTRG.23915.1	LTBP1	XLOC_033434	5662	coding	coding	coding	noncoding
MSTRG.24174.2	NDUFAF7	XLOC_033580	1271	coding	noncoding	noncoding	noncoding
MSTRG.24174.5	NDUFAF7	XLOC_033580	2244	coding	coding	coding	noncoding
MSTRG.24470.11	CDC42EP3	XLOC_033602	279	coding	noncoding	noncoding	noncoding
XR_001739415.1	LOC107985872	XLOC_033647	951	coding	noncoding	noncoding	noncoding
XR_001739426.1	LOC107985873	XLOC_033836	914	coding	noncoding	noncoding	coding
MSTRG.24746.6	PPM1B	XLOC_033954	1865	coding	coding	noncoding	noncoding
MSTRG.25179.6	FOXN2	XLOC_034138	3037	coding	coding	coding	noncoding
MSTRG.25888.3	REL	XLOC_034501	10642	coding	coding	noncoding	noncoding
MSTRG.25888.4	REL	XLOC_034501	9587	coding	coding	noncoding	noncoding
XM_005264437.3	PELI1	XLOC_034631	3384	coding	coding	noncoding	coding
MSTRG.26609.10		XLOC_034662	7013	coding	noncoding	coding	noncoding
MSTRG.26609.13	LOC105374768	XLOC_034662	15653	coding	noncoding	noncoding	noncoding
MSTRG.26609.21	LGALSL-DT	XLOC_034662	9627	coding	noncoding	noncoding	noncoding
MSTRG.26609.19	MIR4433A	XLOC_034662	2244	coding	noncoding	noncoding	noncoding
MSTRG.26609.24	LGALSL-DT	XLOC_034662	3876	coding	coding	coding	noncoding
NM_014755.3	SERTAD2	XLOC_034676	5549	coding	coding	coding	coding
NM_001002755.3	NFU1	XLOC_034843	1274	coding	coding	coding	coding
MSTRG.27045.2	MXD1	XLOC_034900	5438	coding	coding	coding	noncoding
MSTRG.26866.1		XLOC_034906	270	coding	noncoding	noncoding	noncoding
MSTRG.27167.20	ZNF638	XLOC_034967	4178	coding	coding	noncoding	coding
MSTRG.27246.1	ALMS1	XLOC_035056	323	coding	noncoding	noncoding	noncoding
MSTRG.27257.1	ALMS1P1	XLOC_035061	267	coding	coding	noncoding	noncoding
MSTRG.27231.5	LOC112268419	XLOC_035073	1470	coding	coding	coding	coding
NM_001317110.2	MOB1A	XLOC_035079	4893	coding	coding	noncoding	coding
MSTRG.27271.2	INO80B-WBP1	XLOC_035090	2719	coding	coding	coding	coding
MSTRG.27271.5	INO80B-WBP1	XLOC_035090	1068	coding	coding	coding	noncoding
MSTRG.27839.1		XLOC_035147	278	coding	noncoding	noncoding	noncoding
MSTRG.27996.3	GGCX	XLOC_035469	9613	coding	coding	coding	coding
MSTRG.28073.1	PTCD3	XLOC_035503	3929	coding	coding	noncoding	noncoding
NR_036454.1	CHMP3	XLOC_035523	2951	coding	coding	coding	coding
MSTRG.28112.1	ANAPC1P1	XLOC_035554	303	coding	coding	noncoding	noncoding
MSTRG.28311.19	LOC730268	XLOC_035593	1809	coding	noncoding	noncoding	noncoding
MSTRG.28710.1		XLOC_035875	270	coding	coding	noncoding	noncoding
MSTRG.28956.9	INPP4A	XLOC_035955	7878	coding	noncoding	coding	noncoding
MSTRG.28930.1	LIPT1	XLOC_036009	1994	coding	noncoding	noncoding	noncoding
MSTRG.28930.6	LIPT1	XLOC_036009	1249	coding	noncoding	noncoding	noncoding
MSTRG.29256.6	IL1R2	XLOC_036144	1845	coding	coding	noncoding	noncoding
MSTRG.29223.1	IL1RL1	XLOC_036161	492	coding	noncoding	noncoding	noncoding
NM_001144013.1	RGPD3	XLOC_036331	5873	coding	coding	coding	coding
MSTRG.29711.5	GCC2	XLOC_036372	4829	coding	coding	coding	coding
MSTRG.29725.1	LIMS1	XLOC_036373	887	coding	coding	coding	noncoding

MSTRG.29959.1	SH3RF3	XLOC_036471	246	coding	noncoding	noncoding	noncoding
MSTRG.30082.3	TMEM87B	XLOC_036606	4838	coding	coding	noncoding	noncoding
XM_024453174.1	ZC3H8	XLOC_036623	1864	coding	coding	coding	coding
MSTRG.30287.4	CBWD2	XLOC_036716	965	coding	coding	noncoding	noncoding
MSTRG.30638.1	STEAP3	XLOC_036896	287	coding	noncoding	noncoding	noncoding
MSTRG.30738.4	RALB	XLOC_036951	2662	coding	coding	noncoding	noncoding
MSTRG.31354.2	MZT2B	XLOC_037291	476	coding	coding	noncoding	noncoding
MSTRG.31584.1	MAP3K19	XLOC_037436	467	coding	noncoding	noncoding	noncoding
MSTRG.31895.3	ZRANB3	XLOC_037438	3047	coding	coding	noncoding	noncoding
MSTRG.31950.3	R3HDM1	XLOC_037445	3172	coding	coding	noncoding	noncoding
MSTRG.31630.3	LOC107985946	XLOC_037446	4447	coding	coding	coding	noncoding
MSTRG.32248.4	SPOPL	XLOC_037550	5636	coding	coding	coding	coding
MSTRG.32248.11	SPOPL	XLOC_037550	4064	coding	coding	coding	noncoding
MSTRG.33081.6	ARHGAP15	XLOC_037652	2624	coding	coding	noncoding	noncoding
MSTRG.33081.7	ARHGAP15	XLOC_037652	23810	coding	noncoding	noncoding	noncoding
MSTRG.33081.22	ARHGAP15	XLOC_037652	9928	coding	coding	noncoding	noncoding
MSTRG.33081.38	ARHGAP15	XLOC_037652	21450	coding	noncoding	noncoding	noncoding
MSTRG.33081.43	LOC105373654	XLOC_037652	35968	coding	noncoding	noncoding	noncoding
NM_001171653.2	ZEB2	XLOC_037718	9193	coding	coding	coding	coding
MSTRG.32429.1		XLOC_037805	236	coding	noncoding	noncoding	noncoding
MSTRG.32525.1	KIF5C	XLOC_037842	308	coding	noncoding	noncoding	noncoding
MSTRG.33041.4	GPD2	XLOC_038131	741	coding	noncoding	noncoding	noncoding
MSTRG.33525.1		XLOC_038165	546	coding	coding	noncoding	noncoding
MSTRG.34308.50	LY75-CD302	XLOC_038251	8989	coding	coding	coding	coding
MSTRG.34308.51	LY75-CD302	XLOC_038251	14058	coding	coding	coding	coding
MSTRG.33792.7	TANK	XLOC_038338	1704	coding	coding	noncoding	noncoding
MSTRG.33776.1	PSMD14	XLOC_038350	786	coding	noncoding	noncoding	noncoding
MSTRG.34072.1	KCNH7	XLOC_038398	231	coding	noncoding	noncoding	noncoding
NM_004482.4	GALNT3	XLOC_038475	3436	coding	coding	coding	coding
MSTRG.34318.4	DHRS9	XLOC_038569	3569	coding	coding	noncoding	noncoding
MSTRG.34373.1	PHOSPHO2-KLHL23	XLOC_038591	2347	coding	coding	noncoding	noncoding
MSTRG.34375.1	PHOSPHO2-KLHL23	XLOC_038593	218	coding	coding	noncoding	noncoding
MSTRG.34473.2	UBR3	XLOC_038605	7503	coding	coding	coding	coding
MSTRG.34626.3	DYNC1I2	XLOC_038685	3255	coding	coding	noncoding	noncoding
MSTRG.34679.1		XLOC_038687	217	coding	coding	noncoding	noncoding
MSTRG.34688.1		XLOC_038691	328	coding	noncoding	noncoding	noncoding
MSTRG.34825.1	PDK1	XLOC_038737	1579	coding	coding	coding	noncoding
MSTRG.34825.7	PDK1	XLOC_038737	926	coding	coding	coding	noncoding
MSTRG.34859.4	SCRN3	XLOC_038794	386	coding	noncoding	noncoding	noncoding
MSTRG.35898.3	TTN	XLOC_038971	21798	coding	coding	coding	coding
MSTRG.35898.27	TTN	XLOC_038971	25874	coding	coding	noncoding	coding
MSTRG.35898.32	TTN	XLOC_038971	1985	coding	noncoding	noncoding	noncoding

MSTRG.35290.4	UBE2E3	XLOC_039062	1356	coding	coding	noncoding	noncoding
MSTRG.35548.1		XLOC_039312	397	coding	coding	noncoding	noncoding
MSTRG.36163.1	LOC105373793	XLOC_039455	6620	coding	coding	coding	noncoding
MSTRG.36236.2	INPP1	XLOC_039492	1546	coding	coding	coding	noncoding
MSTRG.36325.7	GLS	XLOC_039530	6639	coding	coding	coding	noncoding
MSTRG.36325.11	GLS	XLOC_039530	6937	coding	coding	coding	noncoding
NM_007315.3	STAT1	XLOC_039530	4308	coding	coding	coding	coding
NM_139266.2	STAT1	XLOC_039530	2798	coding	coding	coding	coding
MSTRG.36631.5	SLC39A10	XLOC_039694	1946	coding	noncoding	noncoding	noncoding
NM_001195144.2	ANKRD44	XLOC_039737	6087	coding	coding	coding	coding
MSTRG.36733.5	HSPE1	XLOC_039758	275	coding	coding	noncoding	noncoding
NM_001284361.2	BOLL	XLOC_039776	3285	coding	coding	coding	coding
MSTRG.37152.16	CFLAR-AS1	XLOC_039907	7777	coding	coding	coding	noncoding
MSTRG.37172.13	FLACC1	XLOC_039912	6201	coding	coding	coding	coding
MSTRG.37172.12	FLACC1	XLOC_039912	5569	coding	coding	coding	coding
MSTRG.37040.1		XLOC_039951	358	coding	noncoding	noncoding	noncoding
MSTRG.37200.2	FAM117B	XLOC_039964	1970	coding	coding	noncoding	coding
MSTRG.37200.3	FAM117B	XLOC_039964	3294	coding	coding	noncoding	coding
MSTRG.37196.4	CARF	XLOC_039990	912	coding	coding	noncoding	noncoding
MSTRG.37197.1		XLOC_039991	533	coding	noncoding	noncoding	noncoding
NM_213589.3	RAPH1	XLOC_040013	9699	coding	coding	coding	coding
MSTRG.37401.3	EEF1B2	XLOC_040119	841	coding	coding	coding	noncoding
MSTRG.37522.1	MDH1B	XLOC_040145	8537	coding	coding	coding	coding
NM_003709.4	KLF7	XLOC_040163	8365	coding	coding	coding	coding
MSTRG.37913.11	CREB1	XLOC_040211	6739	coding	coding	noncoding	noncoding
MSTRG.37638.5	PIKFYVE	XLOC_040239	1921	coding	coding	noncoding	noncoding
MSTRG.38626.2	TTLL4	XLOC_040660	5999	coding	coding	coding	coding
MSTRG.38621.1		XLOC_040662	288	coding	noncoding	noncoding	noncoding
MSTRG.38726.1	STK16	XLOC_040701	1223	coding	coding	coding	noncoding
MSTRG.38739.5	GMPPA	XLOC_040717	1735	coding	coding	coding	noncoding
MSTRG.39371.10	RHBDD1	XLOC_041021	4455	coding	coding	noncoding	noncoding
MSTRG.39495.1	FBXO36	XLOC_041137	245	coding	coding	noncoding	noncoding
NM_004509.4	SP110	XLOC_041146	4013	coding	coding	coding	coding
MSTRG.39882.21	LOC105373925	XLOC_041161	6039	coding	coding	noncoding	coding
MSTRG.39882.23	LOC105373925	XLOC_041161	1660	coding	coding	noncoding	coding
MSTRG.39905.19	SP100	XLOC_041163	9371	coding	coding	noncoding	noncoding
MSTRG.39653.8	EIF4E2	XLOC_041252	4697	coding	coding	noncoding	noncoding
MSTRG.39989.3	COPS8	XLOC_041411	2386	coding	coding	noncoding	noncoding
MSTRG.39989.4	COPS8	XLOC_041411	321	coding	coding	noncoding	noncoding
MSTRG.40193.7	LRRFIP1	XLOC_041456	5311	coding	coding	coding	noncoding
MSTRG.40193.12	LRRFIP1	XLOC_041456	1256	coding	coding	coding	noncoding
MSTRG.40576.18	SEPTIN2	XLOC_041671	3502	coding	coding	noncoding	noncoding

MSTRG.40576.23	SEPTIN2	XLOC_041671	3880	coding	coding	coding	noncoding
MSTRG.40576.24	SEPTIN2	XLOC_041671	3377	coding	coding	noncoding	noncoding
MSTRG.40513.1	FARP2	XLOC_041682	280	coding	noncoding	noncoding	noncoding
MSTRG.41302.4	SUMF1	XLOC_041880	1360	coding	coding	noncoding	noncoding
MSTRG.41302.8	SUMF1	XLOC_041880	1052	coding	coding	noncoding	noncoding
XM_017006116.2	THUMPD3	XLOC_042093	3597	coding	coding	coding	coding
MSTRG.41152.3	EMC3	XLOC_042126	1548	coding	coding	noncoding	noncoding
MSTRG.41379.2	SEC13	XLOC_042144	1817	coding	coding	coding	coding
MSTRG.41439.1	MKRN2	XLOC_042209	8207	coding	coding	coding	coding
MSTRG.41439.9	RAF1	XLOC_042209	2780	coding	coding	noncoding	noncoding
MSTRG.41423.1	RPL32	XLOC_042211	541	coding	coding	noncoding	coding
MSTRG.41423.3	RPL32	XLOC_042211	528	coding	coding	noncoding	coding
MSTRG.41583.4	NUP210	XLOC_042250	7322	coding	coding	coding	coding
MSTRG.41674.1	METTL6	XLOC_042342	581	coding	coding	noncoding	noncoding
MSTRG.41828.21	ANKRD28	XLOC_042350	434	coding	noncoding	noncoding	noncoding
MSTRG.41905.39	OXNAD1	XLOC_042386	3069	coding	coding	coding	noncoding
NM_001144382.2	PLCL2	XLOC_042399	4161	coding	coding	coding	coding
MSTRG.42429.1	TBC1D5	XLOC_042432	13188	coding	coding	noncoding	noncoding
MSTRG.42050.7	SATB1	XLOC_042449	11739	coding	coding	coding	noncoding
MSTRG.42050.11	SATB1	XLOC_042449	746	coding	coding	noncoding	noncoding
NM_002948.4	RPL15	XLOC_042649	2730	coding	coding	coding	coding
MSTRG.42615.1	NGLY1	XLOC_042693	1922	coding	coding	noncoding	noncoding
MSTRG.42615.3	NGLY1	XLOC_042693	1987	coding	coding	noncoding	noncoding
MSTRG.42695.5	SLC4A7	XLOC_042737	728	coding	coding	coding	noncoding
MSTRG.43186.5	TMPPE	XLOC_042959	10493	coding	coding	coding	noncoding
MSTRG.43204.32	UBP1	XLOC_042966	3933	coding	coding	coding	noncoding
MSTRG.43256.3	CLASP2	XLOC_042970	3967	coding	coding	coding	noncoding
MSTRG.43406.2	TRANK1	XLOC_043047	13260	coding	noncoding	coding	coding
MSTRG.43672.10	LOC112268444	XLOC_043060	3162	coding	noncoding	coding	noncoding
MSTRG.43684.2	LRRFIP2	XLOC_043062	533	coding	noncoding	noncoding	noncoding
XM_005265069.3	GOLGA4	XLOC_043066	7843	coding	coding	coding	coding
NR_144399.1	WDR48	XLOC_043154	3806	coding	coding	coding	coding
NM_001346225.2	WDR48	XLOC_043154	4079	coding	coding	coding	coding
NM_001304288.2	RPSA	XLOC_043175	1155	coding	coding	coding	coding
MSTRG.44122.1	ULK4	XLOC_043348	230	coding	noncoding	noncoding	noncoding
MSTRG.44132.1	ULK4	XLOC_043358	736	coding	coding	noncoding	noncoding
MSTRG.44190.2	ANO10	XLOC_043469	1983	coding	coding	coding	coding
MSTRG.44190.3	ANO10	XLOC_043469	2078	coding	coding	coding	coding
MSTRG.44333.4	LOC107986083	XLOC_043560	1328	coding	coding	noncoding	noncoding
MSTRG.44411.1	LTF	XLOC_043623	5303	coding	coding	coding	coding
MSTRG.44577.6	CCDC12	XLOC_043651	784	coding	coding	noncoding	noncoding
MSTRG.44497.5	SMARCC1	XLOC_043680	320	coding	coding	noncoding	noncoding

MSTRG.44527.4	NME6	XLOC_043696	3108	coding	coding	noncoding	noncoding
MSTRG.44527.5	NME6	XLOC_043696	1554	coding	coding	coding	noncoding
MSTRG.44618.3	UQCRC1	XLOC_043716	1184	coding	coding	coding	noncoding
MSTRG.44640.6	IP6K2	XLOC_043726	3845	coding	coding	noncoding	noncoding
MSTRG.44640.7	IP6K2	XLOC_043726	1912	coding	coding	noncoding	noncoding
MSTRG.44640.9	IP6K2	XLOC_043726	1972	coding	coding	noncoding	noncoding
MSTRG.44688.5	QRICH1	XLOC_043746	2967	coding	coding	noncoding	noncoding
MSTRG.44704.4	USP19	XLOC_043749	689	coding	noncoding	coding	noncoding
MSTRG.44725.4	RHOA	XLOC_043766	2767	coding	coding	noncoding	noncoding
XM_017005503.1	RBM5	XLOC_043802	3113	coding	coding	coding	coding
XR_001740024.1	PPM1M	XLOC_043921	2279	coding	coding	coding	coding
XM_017005730.1	GLYCTK	XLOC_043922	1857	coding	coding	coding	coding
MSTRG.45168.2	PBRM1	XLOC_043940	7868	coding	coding	noncoding	coding
MSTRG.45168.10	PBRM1	XLOC_043940	5392	coding	coding	noncoding	coding
NM_001354679.2	PRKCD	XLOC_043979	2837	coding	coding	coding	coding
MSTRG.45456.1	CCDC66	XLOC_044069	9473	coding	coding	coding	coding
NM_001349489.1	PXK	XLOC_044151	3159	coding	coding	coding	coding
MSTRG.47145.1	FHIT	XLOC_044254	401	coding	noncoding	noncoding	noncoding
MSTRG.45869.2	THOC7	XLOC_044386	1110	coding	coding	coding	noncoding
MSTRG.46181.1	LOC112267879	XLOC_044490	221	coding	noncoding	noncoding	noncoding
MSTRG.46067.1	TAFA1	XLOC_044530	226	coding	noncoding	noncoding	noncoding
MSTRG.45961.2	TMF1	XLOC_044574	5605	coding	coding	coding	coding
MSTRG.45961.5	TMF1	XLOC_044574	3975	coding	coding	coding	noncoding
MSTRG.46031.4	FRMD4B	XLOC_044580	4248	coding	coding	coding	noncoding
MSTRG.47644.24	FOXP1	XLOC_044617	1392	coding	noncoding	coding	noncoding
MSTRG.47644.27	FOXP1	XLOC_044617	1200	coding	coding	noncoding	noncoding
MSTRG.47644.41	EIF4E3	XLOC_044617	15229	coding	coding	coding	noncoding
MSTRG.46354.2	RYBP	XLOC_044663	4670	coding	noncoding	noncoding	noncoding
MSTRG.46512.3	MIR4273	XLOC_044746	1765	coding	coding	coding	noncoding
MSTRG.47333.1	CGGBP1	XLOC_045005	5036	coding	noncoding	coding	noncoding
MSTRG.47333.6	CGGBP1	XLOC_045005	6479	coding	noncoding	coding	noncoding
NM_001320399.1	PCNP	XLOC_045366	2497	coding	coding	coding	coding
MSTRG.48581.10	CD47	XLOC_045606	7608	coding	coding	noncoding	noncoding
MSTRG.48581.16	CD47	XLOC_045606	4874	coding	coding	noncoding	noncoding
MSTRG.48690.1	ZBED2	XLOC_045711	299	coding	coding	noncoding	noncoding
MSTRG.48769.1		XLOC_045782	280	coding	noncoding	noncoding	noncoding
MSTRG.48826.4	LOC107986114	XLOC_045792	5901	coding	coding	coding	noncoding
MSTRG.48928.5	USF3	XLOC_045848	8133	coding	coding	coding	coding
XM_024453388.1	TIGIT	XLOC_045890	22608	coding	coding	coding	coding
MSTRG.49993.7	ZBTB20-AS1	XLOC_045890	4144	coding	coding	coding	coding
MSTRG.49993.10	ZBTB20-AS1	XLOC_045890	4471	coding	coding	coding	coding
MSTRG.49993.11	ZBTB20-AS1	XLOC_045890	2123	coding	coding	coding	coding

MSTRG.49276.3	TMEM39A	XLOC_046037	2896	coding	coding	coding	noncoding
MSTRG.49353.6	GSK3B	XLOC_046057	3645	coding	coding	coding	noncoding
MSTRG.49353.8	GSK3B	XLOC_046057	3177	coding	coding	coding	noncoding
MSTRG.49344.1		XLOC_046089	367	coding	noncoding	noncoding	noncoding
MSTRG.49348.2	RABL3	XLOC_046091	2023	coding	coding	noncoding	noncoding
MSTRG.49470.7	GOLGB1	XLOC_046122	4515	coding	noncoding	coding	noncoding
MSTRG.49526.4	KPNA1	XLOC_046172	2129	coding	coding	coding	noncoding
MSTRG.49577.7	PARP9	XLOC_046176	4006	coding	coding	noncoding	noncoding
MSTRG.49577.12	DTX3L	XLOC_046176	8540	coding	coding	coding	coding
MSTRG.49690.4	MYLK-AS1	XLOC_046229	1421	coding	coding	coding	noncoding
MSTRG.49676.1	CCDC14	XLOC_046244	3842	coding	coding	coding	noncoding
MSTRG.49965.5	ZXDC	XLOC_046345	2654	coding	coding	coding	coding
MSTRG.50603.2	TMCC1	XLOC_046520	5151	coding	noncoding	coding	noncoding
MSTRG.50663.2	PIK3R4	XLOC_046557	3232	coding	coding	noncoding	noncoding
MSTRG.50705.2	LOC105374114	XLOC_046601	1734	coding	coding	noncoding	coding
MSTRG.50922.2	NPHP3-ACAD11	XLOC_046676	3324	coding	coding	noncoding	noncoding
NR_163491.1	SRPRB	XLOC_046715	4087	coding	coding	coding	coding
NM_001353126.1	CEP63	XLOC_046746	5480	coding	coding	coding	coding
MSTRG.51118.8	STAG1	XLOC_046841	796	coding	noncoding	noncoding	noncoding
NM_001190796.3	NCK1	XLOC_046854	4154	coding	coding	coding	coding
MSTRG.51190.1	DZIP1L	XLOC_046899	240	coding	coding	noncoding	noncoding
MSTRG.51191.1		XLOC_046901	275	coding	noncoding	noncoding	noncoding
MSTRG.51205.1		XLOC_046925	387	coding	noncoding	noncoding	noncoding
MSTRG.51275.2	PIK3CB	XLOC_046939	5711	coding	coding	coding	noncoding
MSTRG.51527.4	TFDP2	XLOC_047082	3988	coding	coding	noncoding	noncoding
MSTRG.51527.5	TFDP2	XLOC_047082	4316	coding	coding	noncoding	noncoding
MSTRG.51548.4	GK5	XLOC_047087	2248	coding	coding	coding	noncoding
MSTRG.51993.12		XLOC_047251	294	coding	noncoding	noncoding	noncoding
MSTRG.52019.2	HLTF	XLOC_047267	1099	coding	noncoding	noncoding	noncoding
MSTRG.52223.3	SIAH2	XLOC_047411	2029	coding	coding	coding	noncoding
MSTRG.52223.4	SIAH2	XLOC_047411	1761	coding	coding	coding	noncoding
XM_011512848.2	MBNL1	XLOC_047501	5203	coding	noncoding	coding	coding
MSTRG.52562.1	LOC105374170	XLOC_047602	311	coding	noncoding	noncoding	noncoding
NM_007287.3	MME	XLOC_047640	5665	coding	coding	coding	coding
MSTRG.53154.2	IFT80	XLOC_047844	1762	coding	coding	noncoding	noncoding
MSTRG.53170.3	TRIM59-IFT80	XLOC_047849	1231	coding	coding	noncoding	noncoding
MSTRG.53209.3	LINC02067	XLOC_047921	561	coding	coding	noncoding	noncoding
MSTRG.53706.3	PHC3	XLOC_048145	7746	coding	coding	noncoding	noncoding
MSTRG.53706.11	PHC3	XLOC_048145	3167	coding	coding	coding	coding
MSTRG.53706.14	PHC3	XLOC_048145	3414	coding	coding	coding	noncoding
XM_011512597.2	ATP11B	XLOC_048554	6882	coding	coding	coding	coding
MSTRG.54594.4	KLHL6	XLOC_048618	5717	coding	coding	coding	noncoding

MSTRG.54650.3	ABCC5	XLOC_048643	7423	coding	coding	coding	coding
NM_001025205.1	AP2M1	XLOC_048655	1934	coding	coding	coding	coding
MSTRG.54727.1	EHHADH	XLOC_048752	263	coding	coding	noncoding	noncoding
MSTRG.54805.5	TRA2B	XLOC_048777	2723	coding	coding	coding	noncoding
MSTRG.55625.2	ACAP2	XLOC_049285	8896	coding	coding	coding	noncoding
MSTRG.55625.3	ACAP2	XLOC_049285	3984	coding	coding	coding	noncoding
MSTRG.55728.2	TFRC	XLOC_049330	4775	coding	coding	noncoding	noncoding
MSTRG.55755.2	RNF168	XLOC_049353	2797	coding	coding	coding	noncoding
NM_017861.4	PIGX	XLOC_049367	3038	coding	coding	coding	coding
MSTRG.55905.7	NCBP2-AS1	XLOC_049387	4003	coding	coding	noncoding	noncoding
MSTRG.55999.2	DLG1	XLOC_049402	5729	coding	coding	coding	noncoding
MSTRG.40761.1	CRBN	XLOC_049506	7604	coding	coding	noncoding	noncoding
MSTRG.40761.3	CRBN	XLOC_049506	4670	coding	coding	noncoding	noncoding
XM_011533625.3	SUMF1	XLOC_049546	1278	coding	coding	coding	coding
MSTRG.41346.1	SETMAR	XLOC_049563	925	coding	noncoding	noncoding	noncoding
MSTRG.40785.3	BHLHE40-AS1	XLOC_049578	4744	coding	coding	noncoding	coding
MSTRG.41098.16	SRGAP3	XLOC_049705	7474	coding	coding	coding	coding
MSTRG.41091.2	MTMR14	XLOC_049713	2143	coding	noncoding	coding	noncoding
MSTRG.41371.4	TATDN2	XLOC_049745	3079	coding	coding	coding	coding
MSTRG.41375.2	GHRL	XLOC_049746	3165	coding	noncoding	noncoding	noncoding
MSTRG.41560.2	ATG7	XLOC_049780	3741	coding	coding	noncoding	coding
MSTRG.41560.1	VGLL4	XLOC_049780	6961	coding	coding	coding	coding
MSTRG.41440.3	RAF1	XLOC_049808	6224	coding	coding	coding	coding
MSTRG.41612.4	XPC	XLOC_049901	2121	coding	coding	coding	noncoding
MSTRG.41641.10	GRIP2	XLOC_049910	6060	coding	coding	coding	noncoding
MSTRG.41641.12	GRIP2	XLOC_049910	7735	coding	coding	coding	noncoding
MSTRG.41687.5	CAPN7	XLOC_049928	4854	coding	coding	coding	noncoding
MSTRG.41687.9	SH3BP5	XLOC_049928	9245	coding	coding	coding	noncoding
MSTRG.41844.7	BTD	XLOC_049941	243	coding	noncoding	noncoding	noncoding
MSTRG.41906.3	OXNAD1	XLOC_050022	5712	coding	coding	coding	noncoding
MSTRG.41906.8	RFTN1	XLOC_050022	3937	coding	coding	coding	noncoding
MSTRG.41906.17	RFTN1	XLOC_050022	8096	coding	coding	noncoding	noncoding
MSTRG.41804.4	PLCL2	XLOC_050059	1534	coding	noncoding	coding	noncoding
MSTRG.42134.1		XLOC_050300	215	coding	noncoding	noncoding	noncoding
MSTRG.42149.1		XLOC_050305	256	coding	noncoding	noncoding	noncoding
MSTRG.42192.1		XLOC_050343	349	coding	noncoding	noncoding	noncoding
NM_001330700.1	TOP2B	XLOC_050487	5485	coding	coding	coding	coding
XM_005265510.4	EOMES	XLOC_050565	3530	coding	coding	coding	coding
MSTRG.43180.2	CNOT10	XLOC_050809	2479	coding	noncoding	noncoding	noncoding
MSTRG.43224.1		XLOC_050873	554	coding	noncoding	noncoding	noncoding
MSTRG.43535.4	EXOG	XLOC_051112	1867	coding	coding	noncoding	noncoding
MSTRG.43621.3	GORASP1	XLOC_051128	7346	coding	coding	coding	coding

NM_001171171.1	CX3CR1	XLOC_051141	3256	coding	coding	coding	coding
MSTRG.43777.3	ZNF619	XLOC_051188	3851	coding	coding	coding	noncoding
MSTRG.44061.6	CTNNB1	XLOC_051223	31882	coding	coding	coding	noncoding
MSTRG.43931.2	VIPR1-AS1	XLOC_051239	1474	coding	coding	coding	noncoding
MSTRG.44015.20	SEC22C	XLOC_051241	11259	coding	coding	coding	noncoding
MSTRG.44015.23	SEC22C	XLOC_051241	2015	coding	coding	noncoding	noncoding
MSTRG.44015.26	SEC22C	XLOC_051241	8981	coding	coding	coding	noncoding
MSTRG.44015.29	SEC22C	XLOC_051241	3085	coding	coding	noncoding	noncoding
MSTRG.44164.4	SNRK	XLOC_051273	19980	coding	coding	coding	noncoding
MSTRG.44444.4	CCR3	XLOC_051404	1759	coding	coding	coding	coding
MSTRG.44576.15	NBEAL2	XLOC_051449	9414	coding	coding	coding	coding
MSTRG.44663.6	ARIH2OS	XLOC_051555	1403	coding	coding	coding	noncoding
MSTRG.44699.2	KLHDC8B	XLOC_051573	1534	coding	coding	coding	noncoding
NM_001313943.2	RHOA	XLOC_051584	1944	coding	coding	coding	coding
MSTRG.44760.5	MST1	XLOC_051598	7330	coding	coding	coding	coding
MSTRG.44793.9	RBM6	XLOC_051616	1387	coding	coding	noncoding	noncoding
MSTRG.44920.2	RAD54L2	XLOC_051716	586	coding	noncoding	noncoding	noncoding
MSTRG.44926.3	TEX264	XLOC_051719	1438	coding	noncoding	coding	coding
MSTRG.45151.3	PRKCD	XLOC_051817	2957	coding	coding	coding	noncoding
MSTRG.45441.3	ASB14	XLOC_051992	2412	coding	coding	coding	noncoding
MSTRG.45511.3	SLMAP	XLOC_052012	1803	coding	noncoding	coding	noncoding
XM_017006974.1	PROK2	XLOC_052555	1547	coding	coding	noncoding	noncoding
MSTRG.46974.2	CHMP2B	XLOC_052938	311	coding	noncoding	coding	noncoding
NM_003663.3	CGGBP1	XLOC_052961	4437	coding	noncoding	coding	coding
MSTRG.47460.7	DHFR2	XLOC_053046	4410	coding	coding	noncoding	noncoding
MSTRG.47460.10	NSUN3	XLOC_053046	4304	coding	coding	noncoding	coding
MSTRG.47744.3	DCBLD2	XLOC_053155	1380	coding	coding	noncoding	noncoding
MSTRG.47789.4	TBC1D23	XLOC_053190	1872	coding	coding	noncoding	noncoding
MSTRG.47836.3	ABI3BP	XLOC_053216	8810	coding	coding	coding	coding
MSTRG.48067.3	PCNP	XLOC_053256	2629	coding	coding	noncoding	noncoding
MSTRG.47948.1	NXPE3	XLOC_053266	3617	coding	coding	coding	noncoding
MSTRG.47948.6	NXPE3	XLOC_053266	3013	coding	coding	noncoding	noncoding
MSTRG.47948.8	NXPE3	XLOC_053266	1290	coding	noncoding	noncoding	noncoding
XM_011513257.1	CBLB	XLOC_053373	6600	coding	coding	coding	coding
MSTRG.48684.7	CD96	XLOC_053643	4608	coding	noncoding	noncoding	noncoding
NM_181780.4	BTLA	XLOC_053698	3126	coding	coding	noncoding	coding
MSTRG.48818.6	SLC35A5	XLOC_053718	620	coding	coding	noncoding	noncoding
MSTRG.49992.4	TIGIT	XLOC_053826	1985	coding	coding	noncoding	noncoding
XR_001740197.2	TMEM39A	XLOC_054126	4500	coding	coding	coding	coding
MSTRG.49578.20	PARP9	XLOC_054325	4809	coding	coding	noncoding	noncoding
MSTRG.49581.5	PARP15	XLOC_054326	1683	coding	coding	coding	noncoding
XR_001740267.2	HSPBAP1	XLOC_054333	2034	coding	coding	coding	coding

MSTRG.49699.1	MYLK-AS1	XLOC_054380	480	coding	noncoding	noncoding	noncoding
NM_053032.4	MYLK	XLOC_054382	4775	coding	coding	coding	coding
MSTRG.49821.1	ZNF148	XLOC_054449	294	coding	noncoding	noncoding	noncoding
MSTRG.50377.1		XLOC_054713	326	coding	noncoding	noncoding	noncoding
NR_034179.1	LOC653712	XLOC_054722	1104	coding	noncoding	coding	coding
NM_001199469.2	ISY1	XLOC_054734	3678	coding	coding	coding	coding
MSTRG.50514.2	COPG1	XLOC_054750	3211	coding	coding	coding	noncoding
MSTRG.50770.2	DNAJC13	XLOC_054933	2965	coding	noncoding	noncoding	noncoding
MSTRG.51661.62	CEP63	XLOC_055016	18179	coding	noncoding	coding	noncoding
MSTRG.51661.67	EPHB1	XLOC_055016	3184	coding	noncoding	coding	noncoding
XR_001740924.2	LOC105374121	XLOC_055019	21856	coding	noncoding	noncoding	coding
MSTRG.51036.1	PPP2R3A	XLOC_055039	279	coding	noncoding	noncoding	noncoding
XR_001740929.1	IL20RB-AS1	XLOC_055084	768	coding	coding	noncoding	noncoding
MSTRG.51353.2	SLC25A36	XLOC_055256	1068	coding	coding	noncoding	noncoding
MSTRG.52762.21	MBNL1-AS1	XLOC_055746	12871	coding	noncoding	coding	noncoding
MSTRG.52762.23	MBNL1	XLOC_055746	51800	coding	coding	noncoding	noncoding
XM_017006892.2	CCNL1	XLOC_055931	3861	coding	coding	coding	coding
MSTRG.52898.2	VEPH1	XLOC_055935	637	coding	noncoding	noncoding	noncoding
MSTRG.52916.2	LOC100996447	XLOC_055949	2820	coding	noncoding	noncoding	noncoding
MSTRG.52953.2	GFM1	XLOC_055956	2626	coding	coding	noncoding	noncoding
MSTRG.52976.1	LOC100287290	XLOC_055967	390	coding	noncoding	noncoding	noncoding
MSTRG.53020.1		XLOC_055990	281	coding	coding	noncoding	noncoding
MSTRG.53671.2	SKIL	XLOC_056329	4206	coding	coding	coding	coding
MSTRG.53922.12	FNDC3B	XLOC_056415	3169	coding	coding	coding	noncoding
MSTRG.53787.1	LINC02068	XLOC_056438	213	coding	noncoding	noncoding	noncoding
MSTRG.53880.3	ECT2	XLOC_056450	2914	coding	coding	noncoding	noncoding
MSTRG.54256.7	KCNMB3	XLOC_056642	7499	coding	coding	coding	coding
MSTRG.54294.2	MRPL47	XLOC_056654	2781	coding	coding	noncoding	coding
MSTRG.54373.7	FXR1	XLOC_056698	2026	coding	coding	noncoding	noncoding
MSTRG.54373.9	FXR1	XLOC_056698	1734	coding	coding	noncoding	noncoding
MSTRG.54569.8	MCF2L2	XLOC_056791	6323	coding	coding	coding	noncoding
MSTRG.54875.5	VPS8	XLOC_056855	3343	coding	coding	noncoding	noncoding
MSTRG.54770.2	SENP2	XLOC_056894	2861	coding	coding	noncoding	noncoding
MSTRG.54770.3	SENP2	XLOC_056894	2865	coding	coding	noncoding	noncoding
MSTRG.54940.2	RFC4	XLOC_056953	4621	coding	coding	coding	noncoding
MSTRG.54954.3	ST6GAL1	XLOC_056958	4318	coding	coding	coding	noncoding
MSTRG.54998.1		XLOC_056975	4745	coding	noncoding	noncoding	noncoding
MSTRG.55261.5	IL1RAP	XLOC_057139	1325	coding	coding	noncoding	noncoding
MSTRG.55506.4	OPA1-AS1	XLOC_057227	4181	coding	coding	coding	noncoding
MSTRG.55506.7	OPA1	XLOC_057227	4341	coding	coding	noncoding	noncoding
MSTRG.56080.2	RPL35A	XLOC_057481	570	coding	coding	noncoding	noncoding
MSTRG.56042.1	LOC107986177	XLOC_057488	2391	coding	noncoding	coding	noncoding

MSTRG.56042.17	FAM157A	XLOC_057488	4652	coding	noncoding	coding	coding
MSTRG.56042.18	FAM157A	XLOC_057488	1006	coding	noncoding	coding	noncoding
NM_014392.4	NSG1	XLOC_057788	2418	coding	coding	coding	coding
NM_152293.3	TADA2B	XLOC_057937	4369	coding	coding	coding	coding
NM_000798.5	DRD5	XLOC_058103	2376	coding	coding	coding	coding
MSTRG.57237.1		XLOC_058133	241	coding	noncoding	noncoding	noncoding
MSTRG.58530.1	LOC101929095	XLOC_058398	415	coding	noncoding	noncoding	noncoding
MSTRG.58559.1	LOC101929095	XLOC_058424	250	coding	coding	noncoding	noncoding
MSTRG.58560.1	LOC101929095	XLOC_058425	267	coding	noncoding	noncoding	noncoding
MSTRG.58563.1	LOC101929095	XLOC_058428	2375	coding	noncoding	noncoding	noncoding
MSTRG.58567.2	LOC101929095	XLOC_058431	6957	coding	coding	noncoding	noncoding
XM_011513772.1	C1QTNF7	XLOC_058433	4390	coding	coding	coding	coding
MSTRG.58584.19	CC2D2A	XLOC_058437	4108	coding	noncoding	noncoding	noncoding
MSTRG.58584.27	FAM200B	XLOC_058437	16236	coding	coding	coding	coding
XM_024453999.1	FAM200B	XLOC_058437	3865	coding	noncoding	noncoding	coding
MSTRG.58106.1	ADGRA3	XLOC_058672	320	coding	coding	noncoding	noncoding
XR_002959726.1	ANAPC4	XLOC_058805	2662	coding	coding	coding	coding
MSTRG.59150.1	ARAP2	XLOC_059120	471	coding	noncoding	noncoding	noncoding
MSTRG.59153.2	LOC439933	XLOC_059123	10480	coding	coding	coding	noncoding
MSTRG.59297.2	TLR1	XLOC_059290	11130	coding	coding	noncoding	noncoding
MSTRG.59297.6	TLR1	XLOC_059290	9648	coding	coding	noncoding	coding
MSTRG.59297.9	TLR6	XLOC_059290	11685	coding	coding	noncoding	noncoding
MSTRG.59297.13	TLR1	XLOC_059290	14205	coding	coding	noncoding	noncoding
MSTRG.59297.11	TLR1	XLOC_059290	14529	coding	coding	noncoding	noncoding
MSTRG.59297.29	TLR1	XLOC_059290	12662	coding	coding	noncoding	noncoding
MSTRG.59297.34	TLR1	XLOC_059290	14867	coding	coding	noncoding	noncoding
MSTRG.59297.35	TLR1	XLOC_059290	12587	coding	coding	coding	noncoding
MSTRG.59415.18	RFC1	XLOC_059311	3522	coding	coding	noncoding	coding
XM_011513719.2	N4BP2	XLOC_059344	9496	coding	coding	coding	coding
NM_001278365.2	RHOH	XLOC_059351	4276	coding	coding	coding	coding
MSTRG.59805.4	ATP8A1	XLOC_059457	9177	coding	coding	coding	noncoding
MSTRG.59805.5	ATP8A1	XLOC_059457	5675	coding	coding	coding	noncoding
MSTRG.59950.1		XLOC_059570	1276	coding	noncoding	noncoding	noncoding
MSTRG.59996.6	TXK	XLOC_059617	3188	coding	coding	noncoding	noncoding
MSTRG.59996.12	TEC	XLOC_059617	877	coding	coding	noncoding	noncoding
XM_017008412.1	TMEM165	XLOC_059838	1893	coding	coding	coding	coding
XM_011534397.3	KIAA1211	XLOC_059875	7660	coding	coding	coding	coding
MSTRG.61193.10	YTHDC1	XLOC_060148	3823	coding	coding	coding	noncoding
XM_017008303.1	UGT2B28	XLOC_060175	1321	coding	coding	coding	coding
MSTRG.61260.2	SULT1B1	XLOC_060191	10920	coding	coding	coding	coding
MSTRG.61260.3	SULT1B1	XLOC_060191	8148	coding	coding	coding	coding
MSTRG.61328.1	RUFY3	XLOC_060238	293	coding	noncoding	noncoding	noncoding

XM_011532412.2	MOB1B	XLOC_060242	6966	coding	coding	coding	coding
NM_002993.4	CXCL6	XLOC_060343	1537	coding	coding	coding	coding
MSTRG.61739.12	NAAA	XLOC_060452	1680	coding	coding	coding	noncoding
MSTRG.62013.7	CNOT6L	XLOC_060552	10824	coding	noncoding	noncoding	noncoding
MSTRG.62013.16	CNOT6L	XLOC_060552	1858	coding	coding	noncoding	noncoding
MSTRG.62013.17	CNOT6L	XLOC_060552	1514	coding	coding	noncoding	noncoding
MSTRG.62391.11	SEC31A	XLOC_060761	1774	coding	noncoding	noncoding	noncoding
MSTRG.62424.7	PLAC8	XLOC_060771	4822	coding	coding	coding	coding
MSTRG.62492.3	COQ2	XLOC_060810	1179	coding	coding	noncoding	noncoding
MSTRG.62537.4	HELQ	XLOC_060816	1667	coding	coding	noncoding	noncoding
MSTRG.62538.5	MRPS18C	XLOC_060817	2236	coding	coding	noncoding	noncoding
MSTRG.62538.7	MRPS18C	XLOC_060817	1648	coding	noncoding	noncoding	noncoding
MSTRG.62592.16	WDFY3	XLOC_060848	4364	coding	noncoding	coding	coding
MSTRG.62592.20	WDFY3	XLOC_060848	2035	coding	noncoding	noncoding	noncoding
MSTRG.63032.9	FAM13A	XLOC_061044	602	coding	coding	noncoding	noncoding
NM_001128430.2	SMARCAD1	XLOC_061176	5022	coding	coding	coding	coding
XM_024453949.1	METAP1	XLOC_061312	3036	coding	coding	coding	coding
MSTRG.63755.7	PPP3CA	XLOC_061395	9245	coding	noncoding	noncoding	noncoding
MSTRG.63755.12	PPP3CA	XLOC_061395	5697	coding	noncoding	noncoding	noncoding
MSTRG.63755.14	PPP3CA	XLOC_061395	35937	coding	noncoding	coding	noncoding
NM_001127208.2	TET2	XLOC_061583	9780	coding	coding	coding	coding
XM_006714242.3	TET2	XLOC_061583	9324	coding	coding	coding	coding
MSTRG.64364.7	AIMP1	XLOC_061621	5177	coding	coding	coding	noncoding
MSTRG.64491.9	LEF1-AS1	XLOC_061677	10659	coding	coding	coding	coding
MSTRG.64821.1	ZGRF1	XLOC_061924	233	coding	coding	noncoding	noncoding
MSTRG.65059.6	CAMK2D	XLOC_061945	211	coding	noncoding	noncoding	noncoding
NM_016599.4	MYOZ2	XLOC_062115	2597	coding	coding	coding	coding
MSTRG.65452.1		XLOC_062197	355	coding	noncoding	noncoding	noncoding
MSTRG.65483.1	PRDM5	XLOC_062222	1205	coding	coding	noncoding	noncoding
MSTRG.65483.2	PRDM5	XLOC_062222	1348	coding	coding	noncoding	noncoding
MSTRG.65395.2	ANXA5	XLOC_062266	1569	coding	coding	coding	noncoding
XM_011532327.1	KIAA1109	XLOC_062297	16206	coding	coding	coding	coding
MSTRG.66398.42	SCLT1	XLOC_062542	1086	coding	noncoding	noncoding	noncoding
XM_005263236.3	NAA15	XLOC_062765	5429	coding	coding	coding	coding
NM_172175.3	IL15	XLOC_062874	2336	coding	coding	noncoding	coding
MSTRG.66767.14	GYPB	XLOC_062956	1110	coding	coding	noncoding	noncoding
MSTRG.66767.23	GYPA	XLOC_062956	804	coding	coding	noncoding	noncoding
MSTRG.66767.28	GYPB	XLOC_062956	629	coding	coding	noncoding	noncoding
MSTRG.66813.1		XLOC_062979	250	coding	noncoding	noncoding	noncoding
MSTRG.66806.4	OTUD4	XLOC_062996	4468	coding	coding	coding	noncoding
XR_002959746.1	C4orf51	XLOC_063013	1590	coding	coding	noncoding	coding
MSTRG.67009.6	SLC10A7	XLOC_063034	669	coding	coding	noncoding	noncoding

MSTRG.67228.6	LRBA	XLOC_063137	7964	coding	coding	coding	coding
MSTRG.67228.9	LRBA	XLOC_063137	7859	coding	coding	coding	coding
MSTRG.67331.2	FBXW7-AS1	XLOC_063195	11324	coding	coding	coding	noncoding
MSTRG.68249.1	KLHL2	XLOC_063619	291	coding	noncoding	noncoding	noncoding
MSTRG.68409.1		XLOC_063701	2333	coding	noncoding	noncoding	noncoding
MSTRG.68433.1		XLOC_063712	866	coding	noncoding	noncoding	noncoding
MSTRG.68436.7	DDX60L	XLOC_063715	6879	coding	coding	noncoding	noncoding
MSTRG.68436.8	DDX60L	XLOC_063715	7189	coding	coding	noncoding	noncoding
MSTRG.68337.27	CBR4	XLOC_063716	2358	coding	coding	noncoding	noncoding
MSTRG.68520.4	MFAP3L	XLOC_063765	4156	coding	coding	coding	noncoding
MSTRG.68520.7	MFAP3L	XLOC_063765	4817	coding	coding	coding	noncoding
MSTRG.68657.1	GALNT7	XLOC_063847	582	coding	noncoding	noncoding	noncoding
MSTRG.68665.2	HMGB2	XLOC_063853	1197	coding	coding	coding	coding
MSTRG.69192.3	DCTD	XLOC_064083	2018	coding	coding	noncoding	coding
MSTRG.69192.4	DCTD	XLOC_064083	2097	coding	coding	noncoding	coding
MSTRG.69324.1	RWDD4	XLOC_064132	250	coding	noncoding	noncoding	noncoding
MSTRG.69364.11	IRF2	XLOC_064167	13953	coding	noncoding	coding	noncoding
MSTRG.69669.1	LOC339975	XLOC_064282	363	coding	noncoding	noncoding	noncoding
XR_427651.4	LINC02515	XLOC_064293	1743	coding	noncoding	noncoding	noncoding
MSTRG.56166.4	PIGG	XLOC_064413	2333	coding	coding	coding	coding
MSTRG.56166.7	PIGG	XLOC_064413	2826	coding	coding	coding	coding
MSTRG.56194.2	LOC107986211	XLOC_064428	4929	coding	coding	coding	noncoding
XM_017007764.1	CTBP1	XLOC_064478	8510	coding	coding	coding	coding
XM_005248038.4	TMEM129	XLOC_064509	2709	coding	coding	coding	coding
MSTRG.56388.1	NELFA	XLOC_064528	317	coding	noncoding	noncoding	noncoding
MSTRG.56445.7	FAM193A	XLOC_064552	999	coding	noncoding	noncoding	coding
MSTRG.56474.3	SH3BP2	XLOC_064563	3676	coding	coding	coding	coding
MSTRG.56561.2	HTT	XLOC_064577	10800	coding	coding	coding	coding
NM_016930.4	STX18	XLOC_064638	2158	coding	coding	coding	coding
MSTRG.56827.1	STX18-AS1	XLOC_064663	361	coding	noncoding	noncoding	noncoding
MSTRG.57183.1		XLOC_064912	300	coding	noncoding	noncoding	noncoding
MSTRG.57184.1		XLOC_064913	814	coding	noncoding	noncoding	noncoding
XM_011513832.2	FBXL5	XLOC_065241	2847	coding	coding	coding	coding
MSTRG.58614.5	BST1	XLOC_065242	7270	coding	coding	coding	noncoding
MSTRG.57882.3	FAM184B	XLOC_065324	1983	coding	coding	coding	noncoding
MSTRG.58050.1	LCORL	XLOC_065354	245	coding	noncoding	noncoding	noncoding
XM_011513819.2	SEL1L3	XLOC_065595	5991	coding	coding	coding	coding
MSTRG.58479.2	RBPJ	XLOC_065622	16078	coding	coding	coding	noncoding
MSTRG.59012.7	STIM2	XLOC_065653	6123	coding	coding	coding	coding
MSTRG.59012.10	STIM2	XLOC_065653	6431	coding	coding	coding	coding
MSTRG.59107.4	DTHD1	XLOC_065848	3586	coding	coding	noncoding	noncoding
NM_001085400.2	RELL1	XLOC_065887	3617	coding	coding	coding	coding

MSTRG.59342.4	TBC1D1	XLOC_065914	11057	coding	coding	coding	coding
MSTRG.59342.6	TBC1D1	XLOC_065914	5237	coding	coding	coding	coding
MSTRG.59264.8	KLF3-AS1	XLOC_065934	10940	coding	coding	coding	noncoding
XM_024454198.1	TLR1	XLOC_065945	7209	coding	coding	coding	coding
MSTRG.59484.6	LOC344967	XLOC_066003	3048	coding	coding	coding	noncoding
MSTRG.59614.1	LIMCH1	XLOC_066070	209	coding	noncoding	noncoding	noncoding
MSTRG.59636.1	LOC105374426	XLOC_066096	10296	coding	coding	coding	noncoding
MSTRG.59671.1	BEND4	XLOC_066112	303	coding	noncoding	noncoding	noncoding
MSTRG.59823.1	ATP8A1	XLOC_066136	248	coding	coding	noncoding	noncoding
MSTRG.60032.3	ATP10D	XLOC_066261	4209	coding	coding	coding	coding
MSTRG.59979.4	SLAIN2	XLOC_066327	4125	coding	coding	noncoding	noncoding
MSTRG.60548.8	FIP1L1	XLOC_066490	1754	coding	coding	coding	noncoding
NM_182606.4	TMPRSS11A	XLOC_066921	3313	coding	coding	coding	coding
MSTRG.61213.2	UGT2B11	XLOC_066953	1416	coding	coding	coding	coding
MSTRG.61418.1	SLC4A4	XLOC_067028	451	coding	noncoding	noncoding	noncoding
MSTRG.61560.1	MTHFD2L	XLOC_067147	269	coding	noncoding	noncoding	noncoding
MSTRG.61525.1		XLOC_067174	252	coding	coding	noncoding	noncoding
XR_001741407.1	LOC105377286	XLOC_067240	4585	coding	noncoding	noncoding	noncoding
NR_146464.1	PAQR3	XLOC_067409	3894	coding	coding	coding	coding
NM_031370.3	HNRNPD	XLOC_067546	3068	coding	coding	coding	coding
MSTRG.62404.1	THAP9	XLOC_067579	397	coding	coding	noncoding	noncoding
MSTRG.62505.3	GPAT3	XLOC_067627	2628	coding	coding	noncoding	noncoding
MSTRG.62836.13	AFF1	XLOC_067761	7293	coding	coding	coding	coding
MSTRG.62836.21	AFF1	XLOC_067761	7673	coding	coding	noncoding	noncoding
MSTRG.62871.1		XLOC_067766	288	coding	noncoding	noncoding	noncoding
MSTRG.63016.17	FAM13A-AS1	XLOC_067832	4346	coding	coding	noncoding	noncoding
MSTRG.63195.1	CCSER1	XLOC_067882	252	coding	coding	noncoding	noncoding
MSTRG.63209.1	CCSER1	XLOC_067895	324	coding	noncoding	noncoding	noncoding
MSTRG.63273.1	CCSER1	XLOC_067940	285	coding	coding	noncoding	noncoding
MSTRG.63501.9	RAP1GDS1	XLOC_068117	631	coding	coding	noncoding	noncoding
MSTRG.63536.1	LOC100507053	XLOC_068153	274	coding	noncoding	noncoding	noncoding
NM_001278310.2	DNAJB14	XLOC_068199	5795	coding	coding	coding	coding
XM_024454127.1	PPP3CA	XLOC_068245	4005	coding	coding	coding	coding
XM_024454048.1	MANBA	XLOC_068396	3162	coding	coding	coding	coding
MSTRG.63987.1	MANBA	XLOC_068415	316	coding	noncoding	noncoding	noncoding
NM_181886.3	UBE2D3	XLOC_068428	4169	coding	coding	noncoding	noncoding
NR_047515.1	SLC9B1	XLOC_068432	1489	coding	coding	noncoding	coding
MSTRG.64265.4	TET2-AS1	XLOC_068518	43691	coding	coding	coding	noncoding
MSTRG.64265.7	TET2	XLOC_068518	8132	coding	noncoding	coding	noncoding
MSTRG.64265.12	TET2	XLOC_068518	14908	coding	coding	coding	coding
MSTRG.64265.19	TET2	XLOC_068518	13204	coding	coding	coding	noncoding
MSTRG.64265.21	TET2	XLOC_068518	14400	coding	coding	noncoding	noncoding

MSTRG.64265.26	TET2-AS1	XLOC_068518	9216	coding	noncoding	noncoding	noncoding
MSTRG.64407.5	RPL34	XLOC_068704	682	coding	coding	noncoding	noncoding
MSTRG.64407.6	RPL34	XLOC_068704	1138	coding	coding	noncoding	noncoding
MSTRG.64548.5	SEC24B	XLOC_068744	4254	coding	coding	coding	noncoding
MSTRG.64548.12	SEC24B	XLOC_068744	1678	coding	coding	noncoding	noncoding
MSTRG.64623.3	EGF	XLOC_068778	357	coding	coding	noncoding	noncoding
MSTRG.64773.6	ALPK1	XLOC_068853	7057	coding	noncoding	coding	coding
MSTRG.64773.11	ALPK1	XLOC_068853	6489	coding	noncoding	noncoding	noncoding
MSTRG.65064.1	CAMK2D	XLOC_068886	212	coding	noncoding	noncoding	noncoding
NM_018699.3	PRDM5	XLOC_069153	5349	coding	coding	coding	coding
MSTRG.65636.18	KIAA1109	XLOC_069191	12202	coding	noncoding	coding	noncoding
MSTRG.65636.23	KIAA1109	XLOC_069191	8725	coding	noncoding	noncoding	noncoding
NM_152778.3	MFSD8	XLOC_069329	4597	coding	coding	noncoding	coding
MSTRG.66399.2	JADE1	XLOC_069367	3793	coding	coding	coding	noncoding
MSTRG.66401.4	C4orf33	XLOC_069369	1717	coding	noncoding	noncoding	noncoding
NM_001276457.1	ELF2	XLOC_069561	3005	coding	coding	coding	coding
MSTRG.66603.6	GAB1	XLOC_069832	6186	coding	coding	coding	coding
MSTRG.66874.1		XLOC_069933	222	coding	coding	noncoding	noncoding
MSTRG.67638.3	RNF175	XLOC_070332	3807	coding	noncoding	noncoding	noncoding
MSTRG.67638.17	RNF175	XLOC_070332	22819	coding	coding	coding	coding
MSTRG.67638.16	RNF175	XLOC_070332	23440	coding	coding	coding	coding
MSTRG.68053.7	RAPGEF2	XLOC_070462	3516	coding	coding	coding	noncoding
MSTRG.68112.5	1-Mar	XLOC_070549	6645	coding	coding	coding	coding
MSTRG.68229.7	KLHL2	XLOC_070592	11612	coding	coding	coding	noncoding
XM_011532103.3	DDX60	XLOC_070657	5964	coding	coding	coding	coding
MSTRG.68462.1	DDX60L	XLOC_070694	292	coding	noncoding	noncoding	noncoding
MSTRG.68786.1	CEP44	XLOC_070918	3403	coding	noncoding	noncoding	noncoding
MSTRG.68869.2	SPCS3	XLOC_070974	3983	coding	coding	coding	noncoding
MSTRG.69375.1		XLOC_071265	599	coding	noncoding	noncoding	noncoding
NM_004346.4	CASP3	XLOC_071280	2645	coding	coding	coding	coding
XM_017007888.2	ACSL1	XLOC_071286	3801	coding	coding	coding	coding
MSTRG.69384.1		XLOC_071298	287	coding	coding	noncoding	noncoding
NM_018409.3	LRP2BP	XLOC_071316	5158	coding	coding	coding	coding
MSTRG.69888.2	LPCAT1	XLOC_071552	5931	coding	coding	coding	noncoding
MSTRG.69909.3	SDHAP3	XLOC_071557	4929	coding	coding	coding	noncoding
MSTRG.69923.2	MRPL36	XLOC_071564	501	coding	coding	noncoding	noncoding
MSTRG.70265.2	FASTKD3	XLOC_071763	5318	coding	coding	coding	noncoding
MSTRG.70378.1	CMBL	XLOC_071862	1709	coding	coding	noncoding	noncoding
NM_138348.6	OTULIN	XLOC_071993	7969	coding	coding	coding	coding
MSTRG.70844.1	ZNF622	XLOC_072053	1521	coding	coding	coding	coding
MSTRG.71484.2	GOLPH3	XLOC_072408	5714	coding	coding	coding	noncoding
MSTRG.71538.1	MTMR12	XLOC_072413	5175	coding	coding	coding	noncoding

MSTRG.71538.7	MTMR12	XLOC_072413	207	coding	noncoding	noncoding	noncoding
MSTRG.71558.2	ZFR	XLOC_072418	4063	coding	coding	coding	coding
MSTRG.71804.1	C1QTNF3	XLOC_072492	4092	coding	coding	noncoding	noncoding
MSTRG.71809.5	C1QTNF3	XLOC_072494	1802	coding	coding	noncoding	noncoding
MSTRG.71913.1	LOC105374724	XLOC_072614	319	coding	noncoding	noncoding	noncoding
XM_005248282.5	NIPBL	XLOC_072660	9016	coding	coding	coding	coding
MSTRG.72079.1	CPLANE1	XLOC_072664	221	coding	noncoding	noncoding	noncoding
MSTRG.72087.1	CPLANE1	XLOC_072672	1730	coding	coding	noncoding	noncoding
MSTRG.72181.17	FYB1	XLOC_072736	5574	coding	coding	noncoding	coding
MSTRG.72387.1		XLOC_072855	372	coding	noncoding	noncoding	noncoding
NM_001330707.2	ZNF131	XLOC_072897	3286	coding	coding	coding	coding
MSTRG.72488.2	C5orf34	XLOC_072913	515	coding	coding	noncoding	noncoding
MSTRG.72496.2	PAIP1	XLOC_072916	2779	coding	coding	coding	noncoding
MSTRG.72751.1	EMB	XLOC_073081	2454	coding	noncoding	noncoding	noncoding
MSTRG.72798.1		XLOC_073129	217	coding	coding	noncoding	noncoding
NM_001102575.2	SNX18	XLOC_073270	5223	coding	coding	coding	coding
MSTRG.73161.6	IL6ST	XLOC_073324	6136	coding	coding	noncoding	noncoding
MSTRG.73325.16	SETD9	XLOC_073422	2180	coding	coding	noncoding	noncoding
MSTRG.74805.1	PDE4D	XLOC_073609	246	coding	noncoding	noncoding	noncoding
MSTRG.73508.3	ELOVL7	XLOC_073683	2720	coding	coding	noncoding	noncoding
MSTRG.73600.1		XLOC_073717	446	coding	noncoding	noncoding	noncoding
NM_001098511.2	KIF2A	XLOC_073812	4161	coding	coding	coding	coding
NM_001164442.2	SHISAL2B	XLOC_073862	772	coding	coding	coding	coding
MSTRG.73818.1	ADAMTS6	XLOC_073921	279	coding	noncoding	noncoding	noncoding
NM_001093755.1	TRAPPC13	XLOC_073936	3103	coding	coding	coding	coding
MSTRG.74892.2	ENC1	XLOC_074306	291	coding	noncoding	noncoding	noncoding
MSTRG.74972.4	POLK	XLOC_074338	8620	coding	coding	coding	coding
XR_002956163.1	POLK	XLOC_074338	3730	coding	coding	coding	coding
MSTRG.75138.6	AP3B1	XLOC_074463	2559	coding	coding	coding	coding
MSTRG.75255.2	LHFPL2	XLOC_074485	3021	coding	coding	noncoding	noncoding
NM_001349549.2	TENT2	XLOC_074535	5175	coding	coding	coding	coding
MSTRG.75446.5	SSBP2	XLOC_074677	12747	coding	coding	noncoding	noncoding
MSTRG.75502.3	RPS23	XLOC_074710	3070	coding	coding	coding	noncoding
MSTRG.75598.1		XLOC_074728	247	coding	noncoding	noncoding	noncoding
MSTRG.75869.16	RASA1	XLOC_074816	49707	coding	coding	coding	noncoding
MSTRG.75869.22	RASA1	XLOC_074816	16214	coding	coding	coding	noncoding
MSTRG.75869.23	RASA1	XLOC_074816	10674	coding	coding	coding	noncoding
MSTRG.75888.5	TMEM161B	XLOC_074845	3443	coding	coding	noncoding	noncoding
MSTRG.75985.3	LYSMD3	XLOC_074921	3169	coding	coding	noncoding	noncoding
MSTRG.76257.1	LUCAT1	XLOC_074925	1413	coding	noncoding	noncoding	noncoding
MSTRG.76257.20	LUCAT1	XLOC_074925	4202	coding	noncoding	noncoding	noncoding
MSTRG.77957.5	FAM172A	XLOC_074983	435	coding	noncoding	noncoding	noncoding

MSTRG.77957.8	FAM172A	XLOC_074983	2049	coding	noncoding	noncoding	noncoding
MSTRG.77957.15	FAM172A	XLOC_074983	1539	coding	noncoding	noncoding	noncoding
MSTRG.77957.38	KIAA0825	XLOC_074988	513	coding	coding	noncoding	coding
MSTRG.77957.63	MCTP1	XLOC_074988	4145	coding	coding	noncoding	noncoding
MSTRG.76618.1	LOC107986435	XLOC_075163	287	coding	noncoding	noncoding	noncoding
MSTRG.76698.9	ST8SIA4	XLOC_075218	29139	coding	coding	noncoding	noncoding
MSTRG.76714.1		XLOC_075259	379	coding	noncoding	noncoding	noncoding
MSTRG.76727.1	LOC105379102	XLOC_075268	564	coding	coding	noncoding	noncoding
MSTRG.76952.1	NUDT12	XLOC_075389	216	coding	noncoding	noncoding	noncoding
MSTRG.77279.2	PJA2	XLOC_075573	19341	coding	coding	noncoding	noncoding
NM_016144.4	COMMD10	XLOC_075919	1435	coding	coding	coding	coding
NM_014350.4	TNFAIP8	XLOC_076082	6976	coding	coding	coding	coding
MSTRG.78718.2	CEP120	XLOC_076262	2803	coding	coding	coding	noncoding
MSTRG.78858.1	ZNF608	XLOC_076322	203	coding	noncoding	noncoding	noncoding
NM_001293735.2	LYRM7	XLOC_076553	6137	coding	coding	noncoding	noncoding
MSTRG.79387.7	RAPGEF6	XLOC_076564	4458	coding	coding	coding	noncoding
MSTRG.79530.30	C5orf56	XLOC_076602	5966	coding	noncoding	coding	coding
MSTRG.79530.29	C5orf56	XLOC_076602	6635	coding	noncoding	coding	noncoding
MSTRG.79457.1	KIF3A	XLOC_076621	666	coding	noncoding	noncoding	noncoding
XM_006714682.2	TCF7	XLOC_076690	3401	coding	coding	coding	coding
MSTRG.79668.1	CDKL3	XLOC_076702	217	coding	noncoding	noncoding	noncoding
MSTRG.79920.1		XLOC_076838	220	coding	noncoding	noncoding	noncoding
MSTRG.79950.1	KLHL3	XLOC_076855	242	coding	coding	noncoding	noncoding
XM_024446028.1	PKD2L2	XLOC_076909	2730	coding	coding	coding	coding
MSTRG.80015.19	PKD2L2	XLOC_076909	5248	coding	coding	coding	noncoding
MSTRG.80028.2	BRD8	XLOC_076912	3432	coding	coding	coding	coding
MSTRG.80028.3	BRD8	XLOC_076912	3558	coding	coding	coding	coding
MSTRG.80028.6	BRD8	XLOC_076912	1563	coding	noncoding	coding	coding
NM_005733.3	KIF20A	XLOC_076913	3095	coding	coding	coding	coding
MSTRG.80245.2	PFDN1	XLOC_077016	1222	coding	noncoding	coding	noncoding
MSTRG.80303.5	LOC112267855	XLOC_077046	387	coding	noncoding	noncoding	noncoding
XM_017009617.2	TMCO6	XLOC_077048	2107	coding	noncoding	coding	coding
XM_024446124.1	TMCO6	XLOC_077048	1895	coding	noncoding	coding	coding
NM_032094.2	PCDHGA12	XLOC_077098	2716	coding	coding	coding	coding
XR_944312.2	ARHGAP26	XLOC_077149	3543	coding	coding	coding	coding
MSTRG.80648.3	PRELID2	XLOC_077406	740	coding	coding	coding	noncoding
MSTRG.80659.2	LARS	XLOC_077420	3687	coding	coding	noncoding	noncoding
MSTRG.81351.7	LOC105378230	XLOC_077681	1755	coding	noncoding	noncoding	noncoding
MSTRG.81672.2	TIMD4	XLOC_077865	398	coding	noncoding	coding	noncoding
NM_001099287.1	NIPAL4	XLOC_077919	3279	coding	coding	coding	coding
MSTRG.81944.4	PWWP2A	XLOC_078015	2219	coding	noncoding	coding	noncoding
MSTRG.82589.22	C5orf58	XLOC_078369	16199	coding	coding	noncoding	noncoding

NM_001031711.3	ERGIC1	XLOC_078485	2903	coding	coding	coding	coding
MSTRG.83231.3	LMAN2	XLOC_078705	1641	coding	coding	coding	noncoding
MSTRG.83329.3	DOK3	XLOC_078729	2472	coding	coding	coding	coding
MSTRG.83329.7	DOK3	XLOC_078729	3507	coding	coding	coding	coding
MSTRG.83336.5	FAM193B	XLOC_078731	1796	coding	noncoding	coding	noncoding
MSTRG.83393.5	HNRNPAB	XLOC_078767	3344	coding	coding	coding	coding
MSTRG.83433.4	CLK4	XLOC_078788	2445	coding	coding	noncoding	noncoding
MSTRG.83433.6	CLK4	XLOC_078788	2470	coding	coding	noncoding	noncoding
MSTRG.83444.2	ZNF354A	XLOC_078791	2449	coding	coding	noncoding	noncoding
MSTRG.83663.1	RACK1	XLOC_078909	1161	coding	coding	coding	coding
MSTRG.71087.1		XLOC_079597	291	coding	noncoding	noncoding	noncoding
MSTRG.71113.1	GUSBP1	XLOC_079620	639	coding	noncoding	noncoding	noncoding
MSTRG.71115.1	GUSBP1	XLOC_079622	743	coding	noncoding	noncoding	noncoding
MSTRG.71873.11	IL7R	XLOC_080001	10619	coding	coding	noncoding	noncoding
MSTRG.71931.3	SKP2	XLOC_080013	1625	coding	coding	noncoding	noncoding
MSTRG.72074.9	CPLANE1	XLOC_080046	8955	coding	coding	noncoding	noncoding
MSTRG.72041.2	WDR70	XLOC_080054	1064	coding	coding	noncoding	noncoding
XM_006714464.3	FYB1	XLOC_080104	4806	coding	coding	coding	coding
MSTRG.72218.2	CARD6	XLOC_080191	3474	coding	noncoding	noncoding	noncoding
MSTRG.72312.3	C5orf51	XLOC_080223	5358	coding	coding	noncoding	noncoding
XM_005248333.5	TMEM267	XLOC_080307	3203	coding	noncoding	coding	coding
MSTRG.73006.15	PARP8	XLOC_080409	18457	coding	noncoding	noncoding	noncoding
MSTRG.72997.1		XLOC_080498	388	coding	noncoding	noncoding	noncoding
MSTRG.73326.1	SETD9	XLOC_080709	245	coding	noncoding	noncoding	noncoding
MSTRG.73875.33	PPWD1	XLOC_080959	2670	coding	coding	noncoding	noncoding
MSTRG.73916.1		XLOC_080981	280	coding	coding	noncoding	noncoding
XR_001742418.1	LOC105379623	XLOC_081131	1588	coding	noncoding	coding	noncoding
MSTRG.74408.2	TNPO1	XLOC_081221	8314	coding	coding	noncoding	noncoding
MSTRG.74420.1	TNPO1	XLOC_081223	798	coding	noncoding	noncoding	noncoding
MSTRG.74458.9	FCHO2	XLOC_081230	1140	coding	coding	noncoding	noncoding
MSTRG.74458.12	FCHO2	XLOC_081230	2226	coding	coding	noncoding	noncoding
MSTRG.74973.9	POLK	XLOC_081339	2886	coding	coding	noncoding	noncoding
MSTRG.75165.1	AP3B1	XLOC_081471	409	coding	noncoding	noncoding	noncoding
MSTRG.75415.5	ZFYVE16	XLOC_081595	6431	coding	coding	noncoding	noncoding
MSTRG.75415.7	ZFYVE16	XLOC_081595	7814	coding	coding	coding	noncoding
MSTRG.75834.6	ZCCHC9	XLOC_081618	852	coding	coding	noncoding	noncoding
MSTRG.75651.6	LOC107986431	XLOC_081721	8774	coding	coding	coding	coding
MSTRG.75651.7	VCAN-AS1	XLOC_081721	23306	coding	coding	coding	coding
MSTRG.78039.1	LOC105379087	XLOC_082186	267	coding	noncoding	noncoding	noncoding
MSTRG.78085.1	SLF1	XLOC_082224	1553	coding	coding	noncoding	noncoding
MSTRG.76888.3	ERAP1	XLOC_082343	10518	coding	coding	coding	coding
MSTRG.76888.39	LNPEP	XLOC_082343	7894	coding	coding	coding	noncoding

MSTRG.76904.2	ERAP2	XLOC_082345	3555	coding	coding	noncoding	noncoding
MSTRG.76904.5	ERAP2	XLOC_082345	5107	coding	coding	noncoding	noncoding
MSTRG.77375.2	FER	XLOC_082674	1631	coding	coding	coding	noncoding
MSTRG.77430.1	FER	XLOC_082711	358	coding	noncoding	noncoding	noncoding
MSTRG.77289.2	MAN2A1	XLOC_082752	7565	coding	coding	coding	noncoding
MSTRG.77593.1	CAMK4	XLOC_082825	55716	coding	coding	coding	noncoding
MSTRG.77593.4	CAMK4	XLOC_082825	31190	coding	coding	coding	noncoding
MSTRG.77547.1		XLOC_082855	208	coding	coding	noncoding	noncoding
MSTRG.77751.4	YTHDC2	XLOC_082901	997	coding	coding	coding	noncoding
MSTRG.78522.1		XLOC_083009	357	coding	noncoding	noncoding	noncoding
MSTRG.78247.4	DMXL1	XLOC_083135	696	coding	noncoding	noncoding	noncoding
MSTRG.78269.4	TNFAIP8	XLOC_083139	14302	coding	coding	coding	coding
MSTRG.78269.5	TNFAIP8	XLOC_083139	13672	coding	coding	coding	coding
MSTRG.78741.1		XLOC_083307	265	coding	noncoding	noncoding	noncoding
MSTRG.79375.9	LOC105379173	XLOC_083616	3448	coding	noncoding	noncoding	noncoding
MSTRG.79375.14	LOC105379173	XLOC_083616	2493	coding	noncoding	coding	noncoding
NM_001164390.2	RAPGEF6	XLOC_083619	3107	coding	coding	coding	coding
MSTRG.79474.1	CCNI2	XLOC_083721	396	coding	noncoding	coding	noncoding
MSTRG.79814.6	SMAD5	XLOC_083889	1508	coding	coding	noncoding	noncoding
MSTRG.80128.2	CTNNA1	XLOC_083995	2429	coding	coding	noncoding	coding
MSTRG.80176.3	UBE2D2	XLOC_084035	2949	coding	coding	coding	coding
NM_006051.3	APBB3	XLOC_084082	2158	coding	coding	coding	coding
NM_001174104.1	CD14	XLOC_084092	1648	coding	noncoding	coding	coding
MSTRG.80320.8	HARS	XLOC_084094	5288	coding	coding	coding	coding
MSTRG.80415.1	PCDHGA8	XLOC_084119	334	coding	coding	noncoding	noncoding
MSTRG.80990.10	ARHGAP26	XLOC_084195	8377	coding	coding	coding	noncoding
MSTRG.80990.21	ARHGAP26	XLOC_084195	16713	coding	noncoding	coding	noncoding
NM_001204258.2	NR3C1	XLOC_084212	6778	coding	coding	coding	coding
MSTRG.80692.3	TCERG1	XLOC_084303	1243	coding	coding	coding	noncoding
MSTRG.80933.5	ADRB2	XLOC_084403	2624	coding	coding	coding	noncoding
NR_132366.1	GRPEL2-AS1	XLOC_084425	401	coding	noncoding	noncoding	noncoding
NM_001892.6	CSNK1A1	XLOC_084432	5360	coding	coding	coding	coding
NM_001025158.2	CD74	XLOC_084508	1305	coding	coding	coding	coding
NM_001155.5	ANXA6	XLOC_084555	2889	coding	coding	coding	coding
MSTRG.81354.3	GM2A	XLOC_084562	6216	coding	coding	noncoding	noncoding
MSTRG.81547.14	FAM114A2	XLOC_084667	5112	coding	coding	noncoding	noncoding
MSTRG.81638.8	GALNT10	XLOC_084674	3813	coding	coding	coding	coding
MSTRG.81638.10	GALNT10	XLOC_084674	5253	coding	coding	coding	coding
MSTRG.81638.11	SAP30L-AS1	XLOC_084674	3129	coding	coding	coding	noncoding
MSTRG.81565.8	CNOT8	XLOC_084703	2760	coding	coding	noncoding	noncoding
MSTRG.81565.9	CNOT8	XLOC_084703	2802	coding	coding	noncoding	noncoding
MSTRG.82230.1		XLOC_085092	258	coding	coding	noncoding	noncoding

MSTRG.82766.11	DOCK2	XLOC_085186	3464	coding	coding	noncoding	noncoding
MSTRG.83003.6	CPEB4	XLOC_085380	4985	coding	noncoding	noncoding	noncoding
MSTRG.83104.1	FAM153B	XLOC_085448	322	coding	noncoding	coding	noncoding
MSTRG.83105.1	FAM153B	XLOC_085449	407	coding	noncoding	noncoding	noncoding
MSTRG.83273.1	ZNF346	XLOC_085507	390	coding	noncoding	noncoding	noncoding
NR_109789.1	RAB24	XLOC_085511	1753	coding	coding	coding	coding
MSTRG.83352.2	NHP2	XLOC_085575	3453	coding	coding	coding	noncoding
MSTRG.83471.1		XLOC_085624	213	coding	coding	noncoding	noncoding
MSTRG.83501.3	LOC101928445	XLOC_085634	2233	coding	coding	coding	noncoding
MSTRG.83501.6	LOC101928445	XLOC_085634	1877	coding	coding	coding	noncoding
MSTRG.83495.4	CANX	XLOC_085638	1578	coding	coding	noncoding	noncoding
MSTRG.83515.1	SQSTM1	XLOC_085644	2328	coding	coding	coding	noncoding
MSTRG.83515.2	MRNIP	XLOC_085644	3220	coding	coding	coding	coding
MSTRG.83515.5	MRNIP	XLOC_085644	2571	coding	coding	coding	coding
MSTRG.83515.4	MRNIP	XLOC_085644	2622	coding	coding	coding	coding
MSTRG.83592.2	CNOT6	XLOC_085684	3099	coding	coding	noncoding	coding
MSTRG.83592.5	CNOT6	XLOC_085684	2330	coding	coding	noncoding	coding
MSTRG.83640.2	BTNL8	XLOC_085708	1991	coding	coding	noncoding	coding
MSTRG.83790.1	LOC102723944	XLOC_085793	503	coding	noncoding	noncoding	noncoding
MSTRG.83852.1		XLOC_085854	294	coding	coding	noncoding	noncoding
MSTRG.83887.3	SERPINB6	XLOC_085868	1316	coding	coding	noncoding	noncoding
MSTRG.83900.7	NQO2	XLOC_085871	1612	coding	noncoding	noncoding	noncoding
MSTRG.83900.8	NQO2	XLOC_085871	895	coding	noncoding	coding	noncoding
NR_104463.1	C6orf201	XLOC_085949	3068	coding	coding	coding	noncoding
MSTRG.84047.12	C6orf201	XLOC_085949	829	coding	coding	noncoding	noncoding
MSTRG.84488.5	BMP6	XLOC_086159	2969	coding	coding	coding	noncoding
MSTRG.84488.3	BMP6	XLOC_086159	3214	coding	coding	coding	noncoding
MSTRG.84805.15	NEDD9	XLOC_086280	14415	coding	noncoding	coding	coding
MSTRG.85034.21	NOL7	XLOC_086396	2519	coding	coding	noncoding	noncoding
MSTRG.85445.2	ATXN1	XLOC_086512	10301	coding	coding	coding	noncoding
MSTRG.85270.3	LOC105374952	XLOC_086549	4139	coding	coding	coding	coding
MSTRG.85326.2	KDM1B	XLOC_086560	7955	coding	coding	coding	coding
MSTRG.85534.4	MBOAT1	XLOC_086646	2967	coding	coding	noncoding	noncoding
NM_003512.3	HIST1H2AC	XLOC_086909	546	coding	coding	coding	coding
NM_001145008.2	BTN3A1	XLOC_086949	3232	coding	coding	coding	coding
MSTRG.86163.2	ZNF322	XLOC_086986	4796	coding	coding	coding	noncoding
MSTRG.86398.2	ZKSCAN4	XLOC_087128	2489	coding	coding	coding	coding
MSTRG.86398.3	ZKSCAN4	XLOC_087128	1707	coding	coding	coding	noncoding
NM_001007531.3	NKAPL	XLOC_087129	1662	coding	coding	coding	coding
MSTRG.86471.2	HLA-H	XLOC_087203	2376	coding	coding	coding	coding
MSTRG.86516.2	HLA-C	XLOC_087251	1399	coding	coding	coding	coding
MSTRG.86630.2	HLA-DRB5	XLOC_087324	1139	coding	coding	coding	noncoding

NM_014260.3	PFDN6	XLOC_087365	598	coding	coding	coding	coding
MSTRG.86693.1	TAPBP	XLOC_087367	2611	coding	coding	coding	coding
MSTRG.86698.3	CUTA	XLOC_087371	598	coding	coding	noncoding	noncoding
XM_011514404.2	SCUBE3	XLOC_087452	7476	coding	coding	coding	coding
MSTRG.86940.3	FKBP5	XLOC_087487	4498	coding	coding	coding	coding
MSTRG.86940.7	FKBP5	XLOC_087487	3651	coding	coding	coding	coding
MSTRG.86975.2	SRPK1	XLOC_087499	4300	coding	coding	noncoding	coding
MSTRG.86975.3	SRPK1	XLOC_087499	4094	coding	coding	noncoding	noncoding
XM_011514310.3	MAPK14	XLOC_087511	4068	coding	coding	coding	coding
MSTRG.87357.1	LOC107986593	XLOC_087623	244	coding	noncoding	noncoding	noncoding
MSTRG.87742.2	MED20	XLOC_087906	2126	coding	coding	coding	noncoding
MSTRG.88018.3	YIPF3	XLOC_088024	1393	coding	noncoding	noncoding	noncoding
MSTRG.88020.5	POLR1C	XLOC_088025	5243	coding	coding	coding	coding
MSTRG.88772.1	SUPT3H	XLOC_088091	5975	coding	noncoding	noncoding	noncoding
NM_001015051.3	RUNX2	XLOC_088092	5487	coding	coding	coding	coding
MSTRG.88240.1	TNFRSF21	XLOC_088215	381	coding	noncoding	coding	noncoding
MSTRG.88462.2	MCM3	XLOC_088344	3214	coding	coding	coding	noncoding
MSTRG.88587.6	ELOVL5	XLOC_088394	1723	coding	coding	coding	noncoding
MSTRG.88978.1	DST	XLOC_088503	241	coding	noncoding	noncoding	noncoding
MSTRG.88890.1	PRIM2	XLOC_088549	330	coding	noncoding	noncoding	noncoding
MSTRG.89192.1	KHDRBS2	XLOC_088653	308	coding	noncoding	noncoding	noncoding
XM_006715426.3	PHF3	XLOC_088691	8477	coding	coding	coding	coding
NM_001290260.1	PHF3	XLOC_088691	2969	coding	noncoding	coding	coding
XR_001744208.1	LOC105377862	XLOC_089089	10681	coding	noncoding	noncoding	noncoding
MSTRG.90236.1		XLOC_089298	297	coding	noncoding	noncoding	noncoding
MSTRG.90343.1	UBE3D	XLOC_089339	559	coding	noncoding	noncoding	noncoding
MSTRG.90399.1	ME1	XLOC_089390	252	coding	noncoding	noncoding	noncoding
NM_002526.4	NT5E	XLOC_089479	3562	coding	coding	coding	coding
NM_001168398.1	SLC35A1	XLOC_089562	1729	coding	coding	noncoding	coding
MSTRG.90762.1	LOC105377885	XLOC_089603	1796	coding	noncoding	noncoding	noncoding
MSTRG.90762.5	LOC105377885	XLOC_089603	6476	coding	noncoding	coding	noncoding
MSTRG.90762.6	LOC105377885	XLOC_089603	6261	coding	noncoding	coding	noncoding
MSTRG.90762.7	LOC105377885	XLOC_089603	1930	coding	noncoding	noncoding	noncoding
MSTRG.90762.8	LOC105377885	XLOC_089603	932	coding	noncoding	noncoding	noncoding
MSTRG.91054.3	MAP3K7	XLOC_089685	6107	coding	coding	noncoding	noncoding
MSTRG.91054.4	MAP3K7	XLOC_089685	3026	coding	coding	coding	noncoding
MSTRG.91115.1		XLOC_089753	245	coding	noncoding	noncoding	noncoding
MSTRG.91141.1		XLOC_089778	418	coding	noncoding	noncoding	noncoding
MSTRG.91470.4	FBXL4	XLOC_089954	2799	coding	coding	noncoding	coding
MSTRG.91767.6	ASCC3	XLOC_089997	1248	coding	noncoding	coding	noncoding
MSTRG.91872.4	PREP	XLOC_090085	19874	coding	coding	coding	noncoding
MSTRG.91964.4	ATG5	XLOC_090139	808	coding	coding	noncoding	noncoding

MSTRG.92031.1	CRYBG1	XLOC_090157	679	coding	coding	noncoding	noncoding
MSTRG.92003.2	CD24	XLOC_090190	2102	coding	coding	noncoding	noncoding
MSTRG.92075.5	SEC63	XLOC_090217	1749	coding	coding	coding	coding
MSTRG.92221.1	FOXO3	XLOC_090258	447	coding	noncoding	noncoding	noncoding
NM_032131.6	ARMC2	XLOC_090272	3734	coding	noncoding	coding	coding
XM_011536168.3	ARMC2	XLOC_090272	2906	coding	noncoding	coding	coding
MSTRG.92455.1	AK9	XLOC_090308	450	coding	coding	noncoding	noncoding
MSTRG.92270.1	WASF1	XLOC_090355	286	coding	noncoding	noncoding	noncoding
MSTRG.92877.3	TRAF3IP2-AS1	XLOC_090429	13113	coding	coding	coding	noncoding
MSTRG.92750.16	LOC105377945	XLOC_090439	2538	coding	coding	coding	noncoding
MSTRG.92750.23	LOC105377945	XLOC_090439	2860	coding	coding	coding	coding
MSTRG.92750.25	LOC105377945	XLOC_090439	13952	coding	coding	noncoding	noncoding
NM_001010892.3	RSPH4A	XLOC_090606	2840	coding	coding	coding	coding
MSTRG.92809.2	ZUP1	XLOC_090609	1225	coding	coding	noncoding	coding
MSTRG.93147.3	CEP85L	XLOC_090692	5568	coding	noncoding	noncoding	noncoding
MSTRG.93147.9	CEP85L	XLOC_090692	2678	coding	noncoding	coding	coding
MSTRG.93444.3	MAN1A1	XLOC_090738	1670	coding	coding	coding	coding
MSTRG.93416.2	TBC1D32	XLOC_090790	691	coding	coding	noncoding	noncoding
MSTRG.93827.2	RNF146	XLOC_090969	8880	coding	coding	coding	coding
MSTRG.93853.2	THEMIS	XLOC_091010	2181	coding	coding	noncoding	noncoding
NR_125849.1	LOC101928140	XLOC_091016	1818	coding	noncoding	noncoding	noncoding
MSTRG.93995.1		XLOC_091080	444	coding	noncoding	noncoding	noncoding
MSTRG.93998.2	ARHGAP18	XLOC_091082	1369	coding	coding	noncoding	noncoding
MSTRG.94086.1	LOC107986643	XLOC_091152	1114	coding	noncoding	noncoding	noncoding
MSTRG.94210.1	LOC105378005	XLOC_091195	293	coding	coding	noncoding	noncoding
MSTRG.94233.1		XLOC_091211	300	coding	noncoding	noncoding	noncoding
MSTRG.94274.5	STX7	XLOC_091242	4317	coding	coding	noncoding	noncoding
MSTRG.94333.3	VNN3	XLOC_091251	6616	coding	noncoding	noncoding	noncoding
MSTRG.94305.2	SLC18B1	XLOC_091253	2538	coding	coding	coding	noncoding
MSTRG.94305.6	SLC18B1	XLOC_091253	2303	coding	coding	coding	noncoding
MSTRG.94606.13	BCLAF1	XLOC_091384	5167	coding	coding	noncoding	noncoding
MSTRG.94784.4	MAP3K5	XLOC_091403	5152	coding	coding	coding	noncoding
NM_006290.4	TNFAIP3	XLOC_091453	4617	coding	coding	coding	coding
MSTRG.94835.1	NHSL1	XLOC_091483	421	coding	noncoding	noncoding	noncoding
MSTRG.94889.1	ECT2L	XLOC_091513	279	coding	noncoding	noncoding	noncoding
XM_017011151.1	ABRACL	XLOC_091523	830	coding	coding	noncoding	noncoding
MSTRG.94952.1	TXLNB	XLOC_091536	389	coding	noncoding	noncoding	noncoding
MSTRG.95054.1		XLOC_091602	286	coding	noncoding	noncoding	noncoding
MSTRG.95171.3	LOC107986655	XLOC_091651	12003	coding	coding	coding	coding
MSTRG.95171.7	HIVEP2	XLOC_091651	13519	coding	coding	coding	coding
XM_011535453.1	STXBP5	XLOC_091897	9073	coding	coding	coding	coding
MSTRG.95762.3	NUP43	XLOC_092033	2114	coding	noncoding	noncoding	noncoding

MSTRG.96141.36	OPRM1	XLOC_092164	5730	coding	coding	noncoding	noncoding
MSTRG.96527.1	SERAC1	XLOC_092555	384	coding	noncoding	noncoding	noncoding
MSTRG.96686.4	SYTL3	XLOC_092584	6916	coding	coding	coding	coding
MSTRG.96718.3	RSPH3	XLOC_092608	929	coding	coding	noncoding	noncoding
MSTRG.97017.1		XLOC_092666	7396	coding	noncoding	noncoding	noncoding
MSTRG.97063.3	SOD2	XLOC_092674	983	coding	coding	coding	noncoding
MSTRG.97069.1	SOD2	XLOC_092680	950	coding	noncoding	coding	coding
NM_004906.5	WTAP	XLOC_092685	2124	coding	coding	coding	coding
NM_152857.2	WTAP	XLOC_092685	1716	coding	coding	coding	coding
NM_001270531.2	WTAP	XLOC_092685	2105	coding	coding	coding	coding
MSTRG.97873.1	PRKN	XLOC_092922	312	coding	noncoding	noncoding	noncoding
MSTRG.97379.2	MPC1	XLOC_093114	834	coding	coding	noncoding	noncoding
MSTRG.97646.1	WDR27	XLOC_093233	319	coding	noncoding	noncoding	noncoding
MSTRG.97649.1	WDR27	XLOC_093236	237	coding	noncoding	noncoding	noncoding
MSTRG.83722.2	IRF4	XLOC_093333	5076	coding	coding	coding	noncoding
NM_001347872.2	MYLK4	XLOC_093467	5886	coding	coding	coding	coding
NR_073112.1	SERPINB1	XLOC_093481	2658	coding	coding	coding	coding
MSTRG.84040.4	PRPF4B	XLOC_093546	7532	coding	coding	coding	coding
MSTRG.84067.1	CDYL	XLOC_093572	5845	coding	coding	coding	noncoding
XM_017010865.1	MAK	XLOC_093781	3832	coding	coding	coding	coding
MSTRG.84695.2	SMIM13	XLOC_093800	2639	coding	coding	coding	noncoding
MSTRG.84912.5	HIVEP1	XLOC_093896	8520	coding	coding	coding	coding
MSTRG.85299.13	JARID2	XLOC_094064	4661	coding	noncoding	noncoding	noncoding
MSTRG.85189.5	MYLIP	XLOC_094092	18131	coding	coding	coding	noncoding
MSTRG.85529.2	LOC101928433	XLOC_094170	1509	coding	noncoding	coding	noncoding
MSTRG.85327.3	DEK	XLOC_094240	7681	coding	coding	coding	coding
MSTRG.85351.4	RNF144B	XLOC_094247	4357	coding	noncoding	noncoding	noncoding
MSTRG.85565.9	E2F3	XLOC_094305	4341	coding	coding	noncoding	noncoding
MSTRG.85952.21	RIPOR2	XLOC_094434	9348	coding	coding	coding	coding
MSTRG.86077.1	HIST1H2AC	XLOC_094569	2263	coding	noncoding	noncoding	noncoding
MSTRG.86299.24	BTN2A2	XLOC_094609	2579	coding	coding	coding	coding
MSTRG.86299.27	BTN3A3	XLOC_094609	3012	coding	coding	coding	noncoding
NM_003519.3	HIST1H2BL	XLOC_094714	453	coding	coding	coding	coding
MSTRG.86321.6	OR2B6	XLOC_094719	829	coding	coding	noncoding	coding
MSTRG.86416.1	PGBD1	XLOC_094761	267	coding	coding	noncoding	noncoding
MSTRG.86458.1		XLOC_094831	1237	coding	noncoding	noncoding	noncoding
NM_001363515.1	DHX16	XLOC_094846	4137	coding	coding	coding	coding
XM_006715005.3	DXO	XLOC_094914	1668	coding	coding	coding	coding
NM_001202470.2	RPS10-NUDT3	XLOC_095013	2529	coding	coding	coding	coding
MSTRG.86868.3	ZNF76	XLOC_095075	4520	coding	coding	coding	coding
XR_001743146.1	STK38	XLOC_095168	3467	coding	coding	coding	coding
MSTRG.87359.12	ZFAND3	XLOC_095232	2003	coding	noncoding	noncoding	noncoding

MSTRG.87359.16	ZFAND3	XLOC_095232	4777	coding	noncoding	coding	noncoding
MSTRG.87659.20	OARD1	XLOC_095404	4785	coding	coding	coding	noncoding
XM_017011042.1	TRERF1	XLOC_095510	9026	coding	coding	coding	coding
MSTRG.87855.4	BICRAL	XLOC_095547	2216	coding	noncoding	noncoding	noncoding
MSTRG.88041.4	LOC107986599	XLOC_095646	2797	coding	coding	coding	noncoding
MSTRG.88041.5	TMEM63B	XLOC_095646	1915	coding	coding	coding	noncoding
MSTRG.88260.2	CD2AP	XLOC_095791	2538	coding	coding	coding	coding
MSTRG.89022.11	ZNF451	XLOC_096085	3461	coding	coding	coding	noncoding
MSTRG.89420.2	FAM135A	XLOC_096310	2138	coding	coding	coding	noncoding
MSTRG.89420.4	FAM135A	XLOC_096310	2018	coding	coding	noncoding	noncoding
MSTRG.89495.1		XLOC_096334	415	coding	noncoding	noncoding	noncoding
MSTRG.89779.6	SENP6	XLOC_096483	5410	coding	coding	coding	noncoding
MSTRG.90147.1	BCKDHB	XLOC_096648	294	coding	coding	noncoding	noncoding
MSTRG.90654.4	ZNF292	XLOC_096847	7513	coding	coding	coding	noncoding
MSTRG.90691.1	SMIM8	XLOC_096859	441	coding	coding	noncoding	noncoding
MSTRG.90702.2	C6orf163	XLOC_096865	323	coding	noncoding	noncoding	noncoding
NM_032870.4	PNISR	XLOC_097339	5113	coding	coding	coding	coding
XM_005266912.4	PNISR	XLOC_097339	5190	coding	coding	coding	coding
NM_001346030.2	USP45	XLOC_097347	5207	coding	coding	coding	coding
MSTRG.91716.1	TSTD3	XLOC_097351	335	coding	noncoding	noncoding	noncoding
MSTRG.91718.1	TSTD3	XLOC_097353	448	coding	noncoding	noncoding	noncoding
MSTRG.92025.3	RTN4IP1	XLOC_097595	9539	coding	coding	coding	coding
MSTRG.92176.1	AFG1L	XLOC_097680	309	coding	coding	noncoding	noncoding
MSTRG.92213.3	FOXO3	XLOC_097693	10660	coding	coding	coding	noncoding
MSTRG.92213.5	FOXO3	XLOC_097693	14811	coding	coding	coding	noncoding
MSTRG.92373.3	CDC40	XLOC_097811	3676	coding	coding	noncoding	noncoding
MSTRG.92883.1	TRAF3IP2-AS1	XLOC_097895	318	coding	noncoding	noncoding	noncoding
MSTRG.92983.3	NT5DC1	XLOC_098049	1764	coding	coding	noncoding	noncoding
MSTRG.92983.10	COL10A1	XLOC_098049	11980	coding	noncoding	noncoding	noncoding
MSTRG.93023.1	DSE	XLOC_098059	384	coding	noncoding	coding	noncoding
MSTRG.92915.1	DCBLD1	XLOC_098132	394	coding	noncoding	noncoding	noncoding
MSTRG.92941.1	GOPC	XLOC_098158	322	coding	noncoding	noncoding	noncoding
XM_011535571.3	TBC1D32	XLOC_098319	6010	coding	coding	coding	coding
MSTRG.93398.1	PKIB	XLOC_098382	297	coding	coding	noncoding	noncoding
MSTRG.93723.6	TRMT11	XLOC_098499	845	coding	noncoding	noncoding	noncoding
MSTRG.93648.1		XLOC_098557	269	coding	noncoding	noncoding	noncoding
MSTRG.94121.2	SAMD3	XLOC_098695	2606	coding	coding	noncoding	noncoding
MSTRG.94121.4	SAMD3	XLOC_098695	2572	coding	coding	noncoding	noncoding
MSTRG.94566.1	PDE7B	XLOC_098980	497	coding	coding	coding	noncoding
MSTRG.94645.1	PEX7	XLOC_099069	306	coding	noncoding	noncoding	noncoding
MSTRG.94876.2	ABRACL	XLOC_099186	688	coding	coding	noncoding	noncoding
MSTRG.94933.7	HECA	XLOC_099191	16233	coding	coding	coding	coding

MSTRG.95327.4	PHACTR2	XLOC_099392	8454	coding	coding	coding	noncoding
MSTRG.95458.12	UTRN	XLOC_099423	16641	coding	coding	coding	noncoding
MSTRG.95458.24	UTRN	XLOC_099423	5577	coding	coding	coding	noncoding
NM_001348092.2	FBXO30	XLOC_099463	8962	coding	coding	coding	coding
MSTRG.95985.1		XLOC_099599	412	coding	noncoding	noncoding	noncoding
MSTRG.96013.6	TAB2	XLOC_099614	8006	coding	coding	noncoding	noncoding
MSTRG.96013.7	TAB2	XLOC_099614	7211	coding	coding	noncoding	noncoding
MSTRG.95916.2	ARMT1	XLOC_099741	1831	coding	coding	noncoding	noncoding
MSTRG.96424.1	ESR1	XLOC_099770	252	coding	coding	noncoding	noncoding
MSTRG.96579.9	ARID1B	XLOC_100027	3797	coding	noncoding	noncoding	noncoding
MSTRG.96612.1	ARID1B	XLOC_100031	592	coding	noncoding	noncoding	noncoding
MSTRG.96677.5	TMEM181	XLOC_100131	4230	coding	coding	noncoding	noncoding
XM_011536110.1	EZR	XLOC_100133	2523	coding	coding	coding	coding
MSTRG.96725.1	LOC105378083	XLOC_100146	206	coding	noncoding	noncoding	noncoding
NM_001322820.2	SOD2	XLOC_100198	14169	coding	coding	coding	coding
MSTRG.97061.17	WTAP	XLOC_100198	2751	coding	coding	noncoding	noncoding
MSTRG.97141.3	IGF2R	XLOC_100211	10081	coding	coding	coding	noncoding
MSTRG.97196.1	SLC22A1	XLOC_100232	357	coding	noncoding	noncoding	noncoding
MSTRG.97223.1		XLOC_100269	266	coding	noncoding	noncoding	noncoding
MSTRG.97970.1	FAM120B	XLOC_100636	3787	coding	noncoding	noncoding	noncoding
MSTRG.97970.3	FAM120B	XLOC_100636	6733	coding	noncoding	coding	noncoding
NM_001363655.2	PDCD2	XLOC_100646	1107	coding	coding	coding	coding
MSTRG.97993.2	LOC105375112	XLOC_100652	3778	coding	noncoding	coding	coding
MSTRG.98193.4	C7orf50	XLOC_100703	1238	coding	coding	coding	noncoding
NM_001033518.2	WIPI2	XLOC_100858	4412	coding	noncoding	coding	coding
MSTRG.98642.3	RNF216	XLOC_100890	4596	coding	noncoding	coding	coding
MSTRG.98672.23	AIMP2	XLOC_100902	4289	coding	coding	noncoding	coding
MSTRG.98726.2	DAGLB	XLOC_100935	2633	coding	coding	coding	coding
MSTRG.98837.1	C1GALT1	XLOC_100986	217	coding	noncoding	noncoding	noncoding
MSTRG.99524.1		XLOC_101497	296	coding	noncoding	noncoding	noncoding
MSTRG.99709.6	SNX13	XLOC_101511	2207	coding	coding	noncoding	noncoding
NM_016447.4	MPP6	XLOC_101826	8515	coding	coding	coding	coding
MSTRG.100267.1	LOC107986779	XLOC_101839	709	coding	noncoding	noncoding	noncoding
MSTRG.100270.2	OSBPL3	XLOC_101842	6254	coding	coding	noncoding	coding
MSTRG.100297.4	NFE2L3	XLOC_101897	11178	coding	coding	noncoding	coding
MSTRG.100297.5	NFE2L3	XLOC_101897	9146	coding	coding	noncoding	coding
MSTRG.100297.11	NFE2L3	XLOC_101897	9590	coding	coding	noncoding	coding
NM_001362753.1	SNX10	XLOC_101904	2844	coding	coding	coding	coding
NR_038831.1	HOXA-AS3	XLOC_101972	3992	coding	coding	coding	noncoding
MSTRG.100387.1		XLOC_101985	569	coding	coding	noncoding	noncoding
MSTRG.100511.18	JAZF1-AS1	XLOC_101996	1919	coding	noncoding	coding	noncoding
XR_001744893.2	CREB5	XLOC_102002	7914	coding	coding	coding	coding

MSTRG.100933.4	GARS	XLOC_102145	12950	coding	coding	coding	coding
MSTRG.101060.7	DPY19L1P1	XLOC_102222	1180	coding	coding	noncoding	noncoding
MSTRG.101315.1	EEPD1	XLOC_102371	345	coding	noncoding	noncoding	noncoding
MSTRG.101395.8	AOAH	XLOC_102392	2619	coding	coding	noncoding	noncoding
MSTRG.101395.9	AOAH	XLOC_102392	6426	coding	coding	noncoding	noncoding
MSTRG.101804.1		XLOC_102401	307	coding	noncoding	noncoding	noncoding
XM_017012693.1	STARD3NL	XLOC_102456	1708	coding	coding	coding	coding
MSTRG.101666.48	TRG-AS1	XLOC_102462	280	coding	noncoding	noncoding	noncoding
MSTRG.101642.1		XLOC_102560	226	coding	noncoding	noncoding	noncoding
MSTRG.102190.3	STK17A	XLOC_102660	2618	coding	coding	coding	coding
MSTRG.102190.11	COA1	XLOC_102660	1170	coding	coding	noncoding	noncoding
XM_017012017.2	SPDYE1	XLOC_102678	1904	coding	coding	coding	coding
MSTRG.102239.3	H2AFV	XLOC_102722	3730	coding	coding	noncoding	noncoding
XR_002956418.1	SUMF2	XLOC_103132	2250	coding	coding	coding	coding
MSTRG.103359.6	ZNF117	XLOC_103315	15247	coding	coding	coding	noncoding
MSTRG.103371.1	CCT6P3	XLOC_103323	255	coding	noncoding	noncoding	noncoding
MSTRG.103555.1		XLOC_103394	497	coding	noncoding	noncoding	noncoding
MSTRG.103868.6	NCF1C	XLOC_103653	1322	coding	coding	coding	coding
NM_001346186.1	RHBDD2	XLOC_103703	1955	coding	coding	coding	coding
NR_023383.1	DTX2P1-UPK3BP1-PMS2P11	XLOC_103752	1763	coding	coding	coding	coding
MSTRG.104781.3	FAM185BP	XLOC_103755	1449	coding	noncoding	coding	noncoding
MSTRG.104781.6	FAM185BP	XLOC_103755	1238	coding	noncoding	coding	noncoding
MSTRG.104825.2	GSAP	XLOC_103783	4753	coding	coding	coding	noncoding
MSTRG.104936.3	LOC102723885	XLOC_104094	1029	coding	coding	noncoding	noncoding
MSTRG.105114.1		XLOC_104234	329	coding	noncoding	noncoding	noncoding
MSTRG.105129.1		XLOC_104247	257	coding	noncoding	noncoding	noncoding
MSTRG.105143.1		XLOC_104259	200	coding	noncoding	noncoding	noncoding
XM_017011644.2	AKAP9	XLOC_104272	14780	coding	coding	noncoding	coding
MSTRG.105164.1		XLOC_104277	269	coding	noncoding	noncoding	noncoding
MSTRG.105386.2	HEPACAM2	XLOC_104303	2020	coding	coding	noncoding	noncoding
MSTRG.105664.2	MIR5692A1	XLOC_104452	8548	coding	coding	coding	noncoding
MSTRG.106602.7	LOC100630923	XLOC_104714	1142	coding	noncoding	noncoding	noncoding
MSTRG.106619.10	POLR2J	XLOC_104720	524	coding	coding	noncoding	noncoding
MSTRG.106619.21	POLR2J2	XLOC_104720	1393	coding	noncoding	coding	coding
XM_017012223.1	SPDYE2	XLOC_104725	4899	coding	coding	coding	coding
MSTRG.106645.25	LRRC17	XLOC_104734	1826	coding	coding	noncoding	noncoding
MSTRG.106654.2	FAM185A	XLOC_104739	11771	coding	coding	noncoding	coding
MSTRG.106432.13	KMT2E	XLOC_104806	13694	coding	coding	coding	coding
MSTRG.106202.2	PUS7	XLOC_104815	214	coding	noncoding	noncoding	noncoding
MSTRG.106282.4	ATXN7L1	XLOC_104822	719	coding	coding	noncoding	noncoding
NM_001282427.2	PIK3CG	XLOC_104894	6983	coding	coding	coding	coding

NM_001282426.2	PIK3CG	XLOC_104894	7059	coding	coding	coding	coding
NM_001289751.1	DLD	XLOC_104927	3544	coding	coding	coding	coding
MSTRG.106504.1	NRCAM	XLOC_104970	256	coding	noncoding	noncoding	noncoding
MSTRG.107477.1	IMMP2L	XLOC_105078	828	coding	coding	noncoding	noncoding
MSTRG.107522.1	IMMP2L	XLOC_105107	361	coding	noncoding	noncoding	noncoding
MSTRG.107603.1	CPED1	XLOC_105486	278	coding	noncoding	noncoding	noncoding
MSTRG.108521.15	LINC-PINT	XLOC_105841	4344	coding	noncoding	noncoding	noncoding
MSTRG.108521.24	LINC-PINT	XLOC_105841	12730	coding	noncoding	coding	noncoding
MSTRG.108521.27	LINC-PINT	XLOC_105841	2043	coding	noncoding	noncoding	noncoding
MSTRG.109094.3	KDM7A	XLOC_106384	9112	coding	coding	coding	noncoding
MSTRG.109143.6	BRAF	XLOC_106413	10073	coding	coding	coding	noncoding
NR_126022.1	LOC101928605	XLOC_106605	990	coding	coding	noncoding	noncoding
NM_170686.3	ZNF398	XLOC_106734	5460	coding	coding	coding	coding
MSTRG.109908.6	ZNF746	XLOC_106759	5823	coding	noncoding	noncoding	noncoding
MSTRG.110143.1	ACTR3C	XLOC_106844	1278	coding	noncoding	noncoding	noncoding
MSTRG.110156.1	ACTR3C	XLOC_106857	304	coding	noncoding	noncoding	noncoding
MSTRG.110164.1	ACTR3C	XLOC_106865	249	coding	noncoding	noncoding	noncoding
MSTRG.110171.1	ACTR3C	XLOC_106872	756	coding	noncoding	noncoding	noncoding
MSTRG.110055.3	GIMAP6	XLOC_106922	4097	coding	coding	coding	noncoding
MSTRG.110055.4	GIMAP6	XLOC_106922	5837	coding	coding	coding	noncoding
NM_001199577.1	GIMAP1-GIMAP5	XLOC_106929	2262	coding	coding	coding	coding
MSTRG.109983.1	LOC105375566	XLOC_106940	2801	coding	coding	noncoding	noncoding
MSTRG.109994.3	TMEM176B	XLOC_106942	1104	coding	coding	noncoding	noncoding
MSTRG.109994.4	TMEM176B	XLOC_106942	1202	coding	coding	coding	noncoding
MSTRG.110495.17	KMT2C	XLOC_106999	3709	coding	coding	coding	coding
MSTRG.110733.34	LMBR1	XLOC_107161	224	coding	noncoding	noncoding	noncoding
MSTRG.98198.7	GPR146	XLOC_107302	1991	coding	coding	coding	noncoding
MSTRG.98267.2	EIF3B	XLOC_107372	3152	coding	coding	coding	coding
MSTRG.98299.2	CHST12	XLOC_107373	2117	coding	coding	coding	noncoding
MSTRG.98685.2	USP42	XLOC_107608	1070	coding	coding	noncoding	coding
MSTRG.98877.3	MIOS	XLOC_107690	3841	coding	coding	coding	noncoding
MSTRG.99576.5	RPA3	XLOC_107695	4428	coding	noncoding	coding	noncoding
MSTRG.99381.1	AGMO	XLOC_107934	921	coding	noncoding	noncoding	noncoding
MSTRG.100633.1	HDAC9	XLOC_108124	478	coding	noncoding	noncoding	noncoding
MSTRG.100147.1	MPP6	XLOC_108320	1271	coding	coding	noncoding	noncoding
MSTRG.100512.11	TAX1BP1	XLOC_108520	9653	coding	coding	noncoding	noncoding
MSTRG.100512.12	TAX1BP1	XLOC_108520	2872	coding	coding	noncoding	noncoding
MSTRG.100512.13	TAX1BP1	XLOC_108520	5297	coding	noncoding	noncoding	noncoding
MSTRG.101912.1	ELMO1	XLOC_109032	363	coding	noncoding	noncoding	noncoding
MSTRG.101486.10	GPR141	XLOC_109088	3468	coding	coding	noncoding	noncoding
MSTRG.101486.18	GPR141	XLOC_109088	13545	coding	noncoding	noncoding	noncoding
MSTRG.101486.17	GPR141	XLOC_109088	2249	coding	noncoding	noncoding	noncoding

MSTRG.101486.23	NME8	XLOC_109088	4959	coding	coding	noncoding	noncoding
MSTRG.101497.4	NME8	XLOC_109090	1311	coding	coding	noncoding	noncoding
MSTRG.101668.38	TRGC2	XLOC_109105	4216	coding	coding	coding	coding
MSTRG.101668.49	TRG-AS1	XLOC_109105	592	coding	noncoding	noncoding	noncoding
MSTRG.101668.50	TRGC2	XLOC_109105	4060	coding	coding	coding	noncoding
MSTRG.101668.53	TRGC2	XLOC_109105	4395	coding	coding	coding	noncoding
MSTRG.101668.57	TRG-AS1	XLOC_109105	3953	coding	noncoding	noncoding	noncoding
MSTRG.101668.61	TRGC2	XLOC_109105	1354	coding	noncoding	noncoding	noncoding
MSTRG.101668.63	TRGC2	XLOC_109105	4052	coding	coding	coding	noncoding
MSTRG.102045.1	SUGCT	XLOC_109241	292	coding	noncoding	noncoding	noncoding
MSTRG.102189.4	COA1	XLOC_109371	3187	coding	coding	noncoding	noncoding
MSTRG.102189.5	COA1	XLOC_109371	10555	coding	coding	noncoding	noncoding
XM_024446827.1	COA1	XLOC_109371	10449	coding	coding	coding	noncoding
MSTRG.102321.7	CCM2	XLOC_109466	2065	coding	coding	coding	noncoding
MSTRG.102321.12	CCM2	XLOC_109466	627	coding	noncoding	coding	noncoding
MSTRG.102616.1	ABCA13	XLOC_109593	2341	coding	noncoding	noncoding	noncoding
MSTRG.102622.1	ABCA13	XLOC_109599	484	coding	noncoding	noncoding	noncoding
MSTRG.102626.1	ABCA13	XLOC_109602	1175	coding	noncoding	noncoding	coding
MSTRG.103252.7	ZNF107	XLOC_109936	5071	coding	coding	noncoding	noncoding
MSTRG.103211.1	ZNF138	XLOC_109945	1542	coding	noncoding	noncoding	noncoding
NM_001007253.4	ERV3-1	XLOC_109956	3206	coding	coding	noncoding	coding
MSTRG.103587.2	GTF2IP23	XLOC_110013	498	coding	noncoding	noncoding	noncoding
MSTRG.103491.3	TYW1	XLOC_110023	916	coding	coding	noncoding	coding
MSTRG.103491.4	TYW1	XLOC_110023	1251	coding	coding	noncoding	noncoding
MSTRG.103819.5	NCF1B	XLOC_110358	1459	coding	coding	coding	noncoding
MSTRG.104730.4	SSC4D	XLOC_110487	522	coding	coding	coding	noncoding
MSTRG.105054.1	DBF4	XLOC_110814	577	coding	noncoding	noncoding	coding
MSTRG.105229.3	AKAP9	XLOC_110944	2841	coding	noncoding	noncoding	noncoding
MSTRG.105229.9	AKAP9	XLOC_110944	2028	coding	noncoding	coding	noncoding
MSTRG.105707.2	LMTK2	XLOC_111232	2376	coding	coding	coding	noncoding
MSTRG.105773.3	PDAP1	XLOC_111279	1661	coding	coding	coding	noncoding
NM_024637.5	GAL3ST4	XLOC_111344	2405	coding	coding	coding	coding
MSTRG.106042.17	ZCWPW1	XLOC_111352	959	coding	coding	coding	noncoding
MSTRG.105968.3	SLC12A9	XLOC_111385	2021	coding	coding	coding	coding
MSTRG.105974.1	TRIP6	XLOC_111387	325	coding	noncoding	noncoding	noncoding
MSTRG.106611.2	ORAI2	XLOC_111441	4686	coding	coding	coding	noncoding
MSTRG.106611.3	ORAI2	XLOC_111441	1633	coding	coding	coding	noncoding
XR_927408.3	FBXL13	XLOC_111472	2927	coding	coding	coding	coding
MSTRG.106663.26	ARMC10	XLOC_111472	1178	coding	coding	noncoding	noncoding
MSTRG.106438.12	KMT2E	XLOC_111529	4226	coding	noncoding	coding	noncoding
MSTRG.106438.18	KMT2E	XLOC_111529	2336	coding	coding	noncoding	noncoding
XR_001744796.2	PNPLA8	XLOC_111657	4179	coding	coding	coding	coding

MSTRG.106931.2	ZNF277	XLOC_111853	2185	coding	coding	noncoding	noncoding
MSTRG.106979.6	MDFIC	XLOC_111946	1488	coding	noncoding	coding	noncoding
MSTRG.107997.5	LOC101928451	XLOC_112325	3867	coding	noncoding	coding	noncoding
MSTRG.107997.18	LOC101928451	XLOC_112325	1365	coding	noncoding	noncoding	noncoding
NM_001708.2	OPN1SW	XLOC_112333	1098	coding	coding	coding	coding
MSTRG.108132.3	AHCYL2	XLOC_112366	2503	coding	coding	coding	coding
MSTRG.108169.4	NRF1	XLOC_112381	3003	coding	coding	noncoding	coding
NR_015431.2	LINC-PINT	XLOC_112467	3128	coding	noncoding	noncoding	noncoding
MSTRG.108592.1	MKLN1	XLOC_112500	10382	coding	coding	coding	noncoding
MSTRG.108592.11	MKLN1	XLOC_112500	2701	coding	coding	noncoding	coding
MSTRG.108592.10	MKLN1	XLOC_112500	10460	coding	coding	noncoding	noncoding
MSTRG.108592.17	MKLN1	XLOC_112500	3498	coding	coding	noncoding	noncoding
MSTRG.108354.1		XLOC_112590	238	coding	noncoding	noncoding	noncoding
MSTRG.108355.1		XLOC_112591	326	coding	noncoding	noncoding	noncoding
MSTRG.108747.4	CYREN	XLOC_112629	7515	coding	coding	coding	noncoding
MSTRG.109035.1	LOC105375523	XLOC_112696	304	coding	noncoding	noncoding	noncoding
MSTRG.109036.1	LOC105375523	XLOC_112697	407	coding	noncoding	noncoding	noncoding
MSTRG.109073.2	LOC100129148	XLOC_112829	5739	coding	coding	coding	noncoding
MSTRG.109263.19	TBXAS1	XLOC_112851	2222	coding	coding	coding	coding
XM_017012558.1	BRAF	XLOC_112882	9595	coding	coding	coding	coding
MSTRG.109190.2	AGK	XLOC_112918	838	coding	coding	coding	noncoding
MSTRG.109321.2	MGAM	XLOC_112951	5239	coding	coding	coding	coding
MSTRG.109526.6	TRBV1	XLOC_112979	2126	coding	coding	coding	noncoding
MSTRG.109526.28	TRBV1	XLOC_112979	1734	coding	coding	coding	noncoding
MSTRG.109526.37	TRBV1	XLOC_112979	1185	coding	coding	coding	noncoding
MSTRG.109389.3	CASP2	XLOC_113007	2618	coding	coding	noncoding	noncoding
MSTRG.109921.1		XLOC_113252	241	coding	noncoding	noncoding	noncoding
MSTRG.110082.3	ZNF862	XLOC_113285	1565	coding	coding	noncoding	noncoding
NR_147019.1	ACTR3C	XLOC_113289	8455	coding	coding	noncoding	coding
MSTRG.109950.6	REPIN1	XLOC_113292	2847	coding	coding	coding	coding
MSTRG.110024.2	GIMAP8	XLOC_113306	3657	coding	coding	coding	coding
MSTRG.110060.1		XLOC_113323	501	coding	noncoding	noncoding	noncoding
MSTRG.110062.1	GIMAP2	XLOC_113324	8321	coding	coding	noncoding	noncoding
MSTRG.109993.13	TMEM176A	XLOC_113334	1209	coding	coding	coding	noncoding
MSTRG.109993.14	TMEM176A	XLOC_113334	1331	coding	coding	noncoding	noncoding
NM_031434.4	TMUB1	XLOC_113355	1491	coding	coding	coding	coding
MSTRG.110213.5	SMARCD3	XLOC_113374	5754	coding	coding	coding	coding
MSTRG.110213.7	SMARCD3	XLOC_113374	6470	coding	coding	coding	coding
MSTRG.110244.2	WDR86	XLOC_113376	6468	coding	coding	coding	coding
MSTRG.110245.1	NUB1	XLOC_113377	288	coding	noncoding	noncoding	noncoding
XM_017012277.2	PRKAG2	XLOC_113396	2276	coding	coding	coding	coding
MSTRG.110352.3	ACTR3B	XLOC_113484	411	coding	coding	coding	noncoding

MSTRG.110575.7	RBM33	XLOC_113584	15501	coding	noncoding	coding	noncoding
MSTRG.110637.1	UBE3C	XLOC_113653	4764	coding	coding	coding	coding
MSTRG.110651.2	DNAJB6	XLOC_113656	2350	coding	coding	noncoding	noncoding
MSTRG.111086.1	DEFA1	XLOC_113879	481	coding	coding	noncoding	noncoding
MSTRG.111413.1	CTSB	XLOC_114014	2395	coding	coding	coding	coding
MSTRG.111413.3	CTSB	XLOC_114014	2240	coding	coding	coding	coding
MSTRG.111671.12	ZDHHC2	XLOC_114115	2415	coding	coding	noncoding	noncoding
XR_002956595.1	VPS37A	XLOC_114117	4726	coding	coding	coding	coding
MSTRG.111786.1		XLOC_114169	6965	coding	noncoding	coding	noncoding
MSTRG.111985.3	NUDT18	XLOC_114324	247	coding	coding	noncoding	noncoding
NM_021174.6	CCAR2	XLOC_114363	5019	coding	coding	coding	coding
MSTRG.112161.6	ENTPD4	XLOC_114396	7831	coding	coding	coding	noncoding
MSTRG.112161.8	ENTPD4	XLOC_114396	3052	coding	coding	coding	noncoding
MSTRG.112200.1	NKX3-1	XLOC_114415	3172	coding	coding	noncoding	coding
MSTRG.112223.1		XLOC_114430	313	coding	noncoding	coding	noncoding
XM_024447269.1	HMBOX1	XLOC_114722	15995	coding	coding	coding	coding
MSTRG.112917.2	KIF13B	XLOC_114745	4810	coding	coding	coding	coding
MSTRG.112787.3	GSR	XLOC_114797	1789	coding	coding	coding	noncoding
NM_013962.2	NRG1	XLOC_114838	1987	coding	coding	coding	coding
MSTRG.112953.2	MAK16	XLOC_114884	4149	coding	coding	coding	coding
NM_153692.4	HTRA4	XLOC_115054	2032	coding	coding	coding	coding
NM_016099.2	GOLGA7	XLOC_115138	1904	coding	coding	coding	coding
MSTRG.113576.17	LOC105379393	XLOC_115159	3139	coding	coding	noncoding	noncoding
MSTRG.113576.18	LOC105379393	XLOC_115159	3080	coding	coding	noncoding	noncoding
MSTRG.113617.2	SLC20A2	XLOC_115186	1473	coding	coding	coding	coding
MSTRG.113854.2	PRKDC	XLOC_115270	13111	coding	coding	noncoding	noncoding
MSTRG.114001.8	PCMTD1	XLOC_115385	3367	coding	coding	coding	noncoding
MSTRG.114105.6	TCEA1	XLOC_115438	709	coding	coding	coding	noncoding
MSTRG.114120.4	LYPLA1	XLOC_115444	1472	coding	coding	coding	noncoding
MSTRG.114295.1		XLOC_115534	480	coding	noncoding	noncoding	noncoding
MSTRG.115566.7	ASPH	XLOC_115704	3277	coding	coding	coding	noncoding
MSTRG.115566.11	ASPH	XLOC_115704	2811	coding	coding	coding	noncoding
MSTRG.114832.11	PDE7A	XLOC_115843	5481	coding	coding	noncoding	noncoding
MSTRG.114832.13	PDE7A	XLOC_115843	22839	coding	noncoding	coding	noncoding
MSTRG.114847.1	LOC102724687	XLOC_115871	287	coding	noncoding	noncoding	noncoding
MSTRG.114920.3	VCPIP1	XLOC_115904	7867	coding	coding	coding	noncoding
MSTRG.115076.41	CSPP1	XLOC_115921	13565	coding	coding	coding	noncoding
NM_001287258.2	XKR9	XLOC_116001	3318	coding	coding	coding	coding
MSTRG.115411.7	STAU2	XLOC_116079	2789	coding	coding	coding	noncoding
MSTRG.115411.8	STAU2	XLOC_116079	2014	coding	coding	coding	noncoding
NM_024721.5	ZFHX4	XLOC_116161	13987	coding	coding	noncoding	coding
MSTRG.115808.2	PEX2	XLOC_116179	1849	coding	coding	noncoding	noncoding

MSTRG.115984.4	MRPS28	XLOC_116319	539	coding	coding	noncoding	noncoding
MSTRG.116182.14	PAG1	XLOC_116346	4119	coding	coding	coding	noncoding
MSTRG.116182.15	PAG1	XLOC_116346	1602	coding	coding	coding	noncoding
MSTRG.116273.2	SNX16	XLOC_116388	399	coding	noncoding	noncoding	noncoding
MSTRG.116800.1		XLOC_116594	320	coding	noncoding	noncoding	noncoding
XM_005251092.3	RIPK2	XLOC_116597	2389	coding	coding	coding	coding
MSTRG.116817.1		XLOC_116608	280	coding	noncoding	noncoding	noncoding
MSTRG.117114.14	NDUFAF6	XLOC_116836	8876	coding	coding	noncoding	noncoding
MSTRG.117114.16	NDUFAF6	XLOC_116836	6035	coding	coding	noncoding	noncoding
MSTRG.117114.17	TP53INP1	XLOC_116836	5224	coding	coding	noncoding	noncoding
MSTRG.117582.1	VPS13B	XLOC_117030	3307	coding	coding	coding	coding
MSTRG.117388.3	RNF19A	XLOC_117046	6712	coding	coding	coding	noncoding
MSTRG.117416.2	PABPC1	XLOC_117072	2727	coding	coding	coding	noncoding
MSTRG.117445.6	YWHAZ	XLOC_117081	3267	coding	coding	noncoding	coding
MSTRG.117445.7	YWHAZ	XLOC_117081	7336	coding	coding	coding	coding
MSTRG.117491.1		XLOC_117104	254	coding	coding	noncoding	noncoding
MSTRG.117734.3	NCALD	XLOC_117105	4152	coding	coding	noncoding	coding
MSTRG.117731.11	LOC105375683	XLOC_117114	4457	coding	coding	coding	noncoding
MSTRG.117804.6	AZIN1	XLOC_117131	4201	coding	coding	noncoding	noncoding
MSTRG.117870.1		XLOC_117180	203	coding	noncoding	noncoding	noncoding
MSTRG.118996.1	SAMD12	XLOC_117552	356	coding	noncoding	noncoding	noncoding
MSTRG.119104.1	SAMD12	XLOC_117616	313	coding	coding	noncoding	noncoding
MSTRG.118785.2	TAF2	XLOC_117667	4892	coding	noncoding	noncoding	noncoding
MSTRG.118785.6	TAF2	XLOC_117667	1524	coding	noncoding	noncoding	noncoding
MSTRG.119503.2	LINC00861	XLOC_117931	22372	coding	noncoding	noncoding	noncoding
MSTRG.119845.1	ASAP1	XLOC_118131	6671	coding	coding	noncoding	noncoding
MSTRG.119845.27	ASAP1	XLOC_118131	10020	coding	noncoding	noncoding	noncoding
MSTRG.119735.1		XLOC_118189	202	coding	noncoding	noncoding	noncoding
XM_005251040.4	TG	XLOC_118278	8170	coding	coding	coding	coding
MSTRG.119976.4	ST3GAL1	XLOC_118303	6917	coding	coding	coding	coding
MSTRG.120001.1		XLOC_118320	296	coding	noncoding	noncoding	noncoding
MSTRG.120945.1	LOC105375787	XLOC_118467	8144	coding	coding	coding	coding
MSTRG.120913.1	LY6E-DT	XLOC_118559	351	coding	noncoding	noncoding	noncoding
MSTRG.121225.5	RPL8	XLOC_118679	965	coding	coding	coding	coding
MSTRG.121245.1	ZNF250	XLOC_118687	318	coding	noncoding	noncoding	noncoding
MSTRG.110851.2	FBXO25	XLOC_118711	1442	coding	noncoding	noncoding	noncoding
XM_017013119.2	ERICH1	XLOC_118724	2465	coding	noncoding	coding	coding
MSTRG.111259.1	MSRA	XLOC_118949	237	coding	noncoding	noncoding	noncoding
NM_173683.4	XKR6	XLOC_119041	4327	coding	coding	coding	coding
NR_073395.1	FAM90A25P	XLOC_119097	1896	coding	coding	coding	coding
MSTRG.111710.3	NAT1	XLOC_119239	1147	coding	coding	noncoding	noncoding
MSTRG.111803.1	LPL	XLOC_119305	476	coding	coding	noncoding	noncoding

MSTRG.111996.5	DMTN	XLOC_119357	2777	coding	coding	coding	noncoding
MSTRG.111996.10	DMTN	XLOC_119357	2316	coding	coding	coding	noncoding
MSTRG.111996.12	DMTN	XLOC_119357	2514	coding	coding	coding	noncoding
NM_025232.4	REEP4	XLOC_119363	1666	coding	coding	coding	coding
MSTRG.112030.3	PDLIM2	XLOC_119397	1835	coding	coding	coding	coding
MSTRG.112129.4	TNFRSF10C	XLOC_119427	2518	coding	coding	coding	noncoding
MSTRG.112187.1	SLC25A37	XLOC_119458	11978	coding	coding	coding	noncoding
MSTRG.112187.15	SLC25A37	XLOC_119458	4676	coding	coding	coding	coding
MSTRG.112187.20	SLC25A37	XLOC_119458	5126	coding	coding	coding	noncoding
MSTRG.112268.1	ADAM28	XLOC_119495	3525	coding	coding	noncoding	noncoding
MSTRG.112460.11	KCTD9	XLOC_119520	21789	coding	coding	coding	noncoding
MSTRG.112460.26	DOCK5	XLOC_119520	6624	coding	coding	coding	noncoding
MSTRG.112521.1	EPHX2	XLOC_119605	308	coding	noncoding	noncoding	noncoding
MSTRG.112594.1	ESCO2	XLOC_119623	626	coding	coding	noncoding	noncoding
XM_006716357.4	INTS9	XLOC_119672	2217	coding	coding	coding	coding
MSTRG.112890.3	HMBOX1	XLOC_119679	4633	coding	coding	coding	noncoding
MSTRG.112890.7	HMBOX1	XLOC_119679	2871	coding	coding	noncoding	noncoding
MSTRG.112743.3	LEPROTL1	XLOC_119732	4983	coding	coding	noncoding	noncoding
MSTRG.113298.5	ASH2L	XLOC_120010	2379	coding	coding	coding	coding
MSTRG.113308.2	BAG4	XLOC_120016	3319	coding	coding	noncoding	noncoding
NM_031940.4	TM2D2	XLOC_120056	3628	coding	coding	coding	coding
MSTRG.113513.9	TM2D2	XLOC_120056	2766	coding	coding	noncoding	noncoding
MSTRG.113513.10	ADAM9	XLOC_120056	1657	coding	coding	noncoding	noncoding
MSTRG.113522.2	GOLGA7	XLOC_120115	2328	coding	coding	noncoding	noncoding
MSTRG.113782.4	UBE2V2	XLOC_120257	1148	coding	coding	noncoding	noncoding
MSTRG.114005.2	LOC102724330	XLOC_120360	317	coding	noncoding	noncoding	noncoding
XM_011517647.3	RB1CC1	XLOC_120383	5426	coding	noncoding	coding	coding
MSTRG.114229.3	CHCHD7	XLOC_120502	2588	coding	noncoding	coding	noncoding
MSTRG.114423.12	SDCBP	XLOC_120587	6124	coding	coding	coding	noncoding
NM_002603.4	PDE7A	XLOC_120877	6443	coding	coding	noncoding	coding
MSTRG.114937.2	C8orf44-SGK3	XLOC_120935	4198	coding	coding	noncoding	noncoding
MSTRG.115078.2	CSPP1	XLOC_120950	2573	coding	noncoding	noncoding	noncoding
MSTRG.115078.4	CSPP1	XLOC_120950	1874	coding	noncoding	noncoding	noncoding
MSTRG.115452.10	ELOC	XLOC_121286	1961	coding	noncoding	noncoding	noncoding
MSTRG.115456.2	TMEM70	XLOC_121288	1207	coding	coding	coding	coding
XR_001745986.1	LOC105375624	XLOC_121798	3863	coding	noncoding	noncoding	noncoding
MSTRG.116651.4	DECR1	XLOC_121867	1742	coding	coding	coding	noncoding
MSTRG.116651.5	DECR1	XLOC_121867	1714	coding	coding	noncoding	noncoding
MSTRG.116651.14	DECR1	XLOC_121867	6721	coding	coding	coding	noncoding
XM_011517152.3	PIP4P2	XLOC_121905	921	coding	coding	noncoding	coding
MSTRG.116870.3	OTUD6B	XLOC_121922	2278	coding	coding	noncoding	noncoding
MSTRG.116970.1	TMEM67	XLOC_122008	360	coding	coding	noncoding	noncoding

MSTRG.117060.1	LOC101926977	XLOC_122049	952	coding	noncoding	noncoding	noncoding
MSTRG.117107.2	CCNE2	XLOC_122063	6961	coding	coding	noncoding	coding
MSTRG.117603.32	CPQ	XLOC_122184	2254	coding	noncoding	noncoding	noncoding
MSTRG.117579.15	VPS13B-DT	XLOC_122280	3829	coding	coding	coding	noncoding
MSTRG.117586.1	VPS13B	XLOC_122285	2496	coding	coding	noncoding	coding
XM_017013302.1	RNF19A	XLOC_122316	4209	coding	coding	coding	coding
MSTRG.118151.3	OXR1	XLOC_122648	2826	coding	coding	coding	coding
MSTRG.118195.2	EMC2	XLOC_122714	1115	coding	coding	noncoding	noncoding
MSTRG.119146.1	SNTB1	XLOC_123191	236	coding	noncoding	noncoding	noncoding
MSTRG.119165.9	TBC1D31	XLOC_123272	3766	coding	coding	noncoding	noncoding
MSTRG.119165.10	TBC1D31	XLOC_123272	3417	coding	coding	noncoding	noncoding
MSTRG.120510.1	LOC105375751	XLOC_123570	1010	coding	noncoding	noncoding	noncoding
MSTRG.120520.1	LOC105375751	XLOC_123576	2152	coding	noncoding	noncoding	noncoding
MSTRG.120536.1	PCAT2	XLOC_123590	1221	coding	noncoding	noncoding	noncoding
MSTRG.120538.1	PCAT2	XLOC_123591	265	coding	noncoding	noncoding	noncoding
MSTRG.120574.1	LOC105375752	XLOC_123622	240	coding	noncoding	noncoding	noncoding
MSTRG.119783.2	EFR3A	XLOC_123857	2389	coding	noncoding	noncoding	coding
XM_017013894.2	TRAPPC9	XLOC_124160	5426	coding	coding	coding	coding
MSTRG.120946.17	SLC45A4	XLOC_124233	912	coding	noncoding	coding	noncoding
MSTRG.120918.2	LY6E	XLOC_124314	1160	coding	coding	coding	noncoding
MSTRG.120929.1	LOC107986906	XLOC_124321	650	coding	coding	noncoding	noncoding
MSTRG.121031.3	ZNF623	XLOC_124370	3939	coding	coding	noncoding	noncoding
XM_017013382.1	NRBP2	XLOC_124380	2292	coding	coding	coding	coding
MSTRG.121095.3	GRINA	XLOC_124391	1461	coding	coding	coding	coding
NM_000973.5	RPL8	XLOC_124461	845	coding	coding	coding	coding
MSTRG.121195.1		XLOC_124464	272	coding	noncoding	noncoding	noncoding
MSTRG.121253.1		XLOC_124482	215	coding	noncoding	noncoding	noncoding
XM_011518045.3	DOCK8	XLOC_124507	7213	coding	coding	coding	coding
MSTRG.121403.1		XLOC_124614	437	coding	noncoding	noncoding	noncoding
MSTRG.121831.5	RFX3	XLOC_124642	2298	coding	coding	noncoding	noncoding
MSTRG.121686.3	AK3	XLOC_124761	2133	coding	coding	noncoding	noncoding
NM_004972.3	JAK2	XLOC_124780	5285	coding	coding	coding	coding
NM_001135920.3	RIC1	XLOC_124856	4045	coding	coding	coding	coding
XM_024447459.1	KDM4C	XLOC_124915	6575	coding	coding	coding	coding
MSTRG.122217.1		XLOC_125109	248	coding	noncoding	noncoding	noncoding
MSTRG.122606.2	HAUS6	XLOC_125250	3093	coding	coding	coding	coding
MSTRG.122771.1	MIR31HG	XLOC_125318	369	coding	coding	noncoding	noncoding
MSTRG.123120.17	C9orf72	XLOC_125462	3219	coding	coding	noncoding	noncoding
MSTRG.124172.1	LINGO2	XLOC_125568	242	coding	noncoding	noncoding	noncoding
MSTRG.124334.1	LINGO2	XLOC_125708	903	coding	noncoding	noncoding	noncoding
MSTRG.124365.1	LINGO2	XLOC_125738	443	coding	noncoding	noncoding	noncoding
MSTRG.123336.3	APTX	XLOC_125859	2077	coding	coding	coding	noncoding

MSTRG.123336.5	APTX	XLOC_125859	1871	coding	coding	coding	noncoding
MSTRG.123360.2	SMU1	XLOC_125878	4225	coding	coding	coding	noncoding
MSTRG.123462.2	UBAP2	XLOC_125934	4314	coding	coding	coding	noncoding
MSTRG.123401.1	KIF24	XLOC_125944	551	coding	noncoding	noncoding	noncoding
MSTRG.123468.2	DCTN3	XLOC_125967	915	coding	coding	noncoding	noncoding
MSTRG.123576.2	FAM214B	XLOC_126007	3089	coding	coding	coding	coding
MSTRG.123576.9	FAM214B	XLOC_126007	2959	coding	coding	coding	coding
MSTRG.123621.2	SIT1	XLOC_126036	798	coding	noncoding	noncoding	noncoding
NM_022343.4	GLIPR2	XLOC_126077	1895	coding	coding	coding	coding
MSTRG.124467.1		XLOC_126299	2812	coding	noncoding	noncoding	noncoding
MSTRG.124553.4	CBWD6	XLOC_126358	1244	coding	coding	noncoding	noncoding
MSTRG.124568.1		XLOC_126359	15451	coding	coding	noncoding	noncoding
XM_011518783.3	PGM5	XLOC_126656	3764	coding	coding	coding	coding
MSTRG.125264.4	PTAR1	XLOC_126739	10136	coding	coding	noncoding	noncoding
MSTRG.125402.6	CEMIP2	XLOC_126811	3465	coding	noncoding	noncoding	coding
MSTRG.125491.3	ZFAND5	XLOC_126853	2398	coding	coding	coding	noncoding
MSTRG.125715.1	NMRK1	XLOC_126925	2398	coding	coding	noncoding	noncoding
MSTRG.126059.15	GNAQ	XLOC_127048	6194	coding	coding	coding	noncoding
MSTRG.126059.24	GNAQ	XLOC_127048	1560	coding	noncoding	noncoding	noncoding
MSTRG.126583.1	FRMD3	XLOC_127276	521	coding	coding	noncoding	noncoding
XM_017014722.1	IDNK	XLOC_127292	1138	coding	noncoding	noncoding	coding
MSTRG.126496.25	LOC105376335	XLOC_127298	2411	coding	coding	coding	noncoding
MSTRG.126504.3	RMI1	XLOC_127301	5175	coding	coding	coding	noncoding
MSTRG.126638.5	AGTPBP1	XLOC_127332	4104	coding	coding	coding	noncoding
MSTRG.126766.16	TUT7	XLOC_127354	7335	coding	coding	coding	noncoding
XM_005251757.4	DAPK1	XLOC_127394	3236	coding	coding	coding	coding
MSTRG.126789.3	LOC497256	XLOC_127419	468	coding	noncoding	noncoding	noncoding
MSTRG.127045.34	SECISBP2	XLOC_127471	8291	coding	coding	coding	coding
MSTRG.127120.1	LOC100507103	XLOC_127567	219	coding	noncoding	noncoding	noncoding
MSTRG.127176.3	NOL8	XLOC_127613	4058	coding	coding	noncoding	noncoding
MSTRG.127399.2	FAM120AOS	XLOC_127672	3556	coding	noncoding	coding	coding
MSTRG.127418.1	FAM120A	XLOC_127691	372	coding	noncoding	noncoding	noncoding
MSTRG.127650.7	ZNF782	XLOC_127861	2440	coding	coding	coding	noncoding
MSTRG.127657.1		XLOC_127866	1011	coding	noncoding	noncoding	noncoding
MSTRG.127682.4	MFSD14C	XLOC_127878	1528	coding	noncoding	coding	noncoding
NM_001166116.1	TMOD1	XLOC_127917	3261	coding	coding	coding	coding
NM_001351507.1	NCBP1	XLOC_127918	5317	coding	coding	coding	coding
MSTRG.127841.8	NCBP1	XLOC_127918	1711	coding	coding	coding	coding
MSTRG.127820.3	TRMO	XLOC_127947	290	coding	noncoding	noncoding	noncoding
MSTRG.128198.1		XLOC_128178	233	coding	noncoding	noncoding	noncoding
MSTRG.128745.2	ELP1	XLOC_128418	5051	coding	coding	coding	coding
MSTRG.128895.3	ECPAS	XLOC_128505	8513	coding	coding	coding	noncoding

MSTRG.129007.3	SUSD1	XLOC_128571	3389	coding	coding	coding	noncoding
MSTRG.129014.4	PTBP3	XLOC_128573	1349	coding	coding	coding	noncoding
MSTRG.129014.9	PTBP3	XLOC_128573	10222	coding	coding	noncoding	noncoding
MSTRG.129697.3	CDK5RAP2	XLOC_128925	4886	coding	noncoding	noncoding	coding
MSTRG.129697.4	CDK5RAP2	XLOC_128925	6043	coding	coding	noncoding	coding
MSTRG.129693.2	MEGF9	XLOC_128928	6894	coding	coding	coding	coding
XM_005251679.4	CNTRL	XLOC_128959	7782	coding	coding	coding	coding
MSTRG.129981.1	CNTRL	XLOC_128959	12547	coding	coding	coding	coding
MSTRG.130139.3	LOC112268055	XLOC_129186	3201	coding	coding	noncoding	noncoding
MSTRG.130500.3	ST6GALNAC6	XLOC_129344	4218	coding	coding	coding	coding
MSTRG.130742.10	TOR1B	XLOC_129473	5298	coding	coding	coding	coding
MSTRG.130808.1	FNBP1	XLOC_129479	6351	coding	coding	coding	noncoding
MSTRG.131039.1		XLOC_129643	825	coding	noncoding	noncoding	noncoding
MSTRG.131374.3	ENTR1	XLOC_129806	969	coding	noncoding	coding	coding
MSTRG.121442.6	DOCK8-AS1	XLOC_129952	15418	coding	coding	coding	coding
MSTRG.121466.1	KANK1	XLOC_129967	276	coding	noncoding	noncoding	noncoding
MSTRG.121750.1	INSL6	XLOC_130141	374	coding	noncoding	noncoding	noncoding
MSTRG.121938.2	RIC1	XLOC_130159	5466	coding	coding	noncoding	noncoding
XR_001746382.2	ERMP1	XLOC_130159	5188	coding	coding	coding	coding
MSTRG.121818.8	UHRF2	XLOC_130189	634	coding	coding	noncoding	noncoding
MSTRG.122081.3	KDM4C	XLOC_130204	4709	coding	coding	coding	coding
MSTRG.122618.4	DENND4C	XLOC_130533	8412	coding	coding	coding	noncoding
MSTRG.122610.3	ACER2	XLOC_130534	1982	coding	coding	noncoding	noncoding
MSTRG.122610.4	ACER2	XLOC_130534	1825	coding	noncoding	noncoding	noncoding
NM_002176.4	IFNB1	XLOC_130611	839	coding	coding	coding	coding
MSTRG.122790.2	MTAP	XLOC_130656	2549	coding	coding	coding	noncoding
NM_001363763.2	CDKN2A	XLOC_130669	1302	coding	noncoding	coding	coding
MSTRG.123319.6	LOC105376018	XLOC_131011	2869	coding	coding	noncoding	noncoding
MSTRG.123853.12	ZCCHC7	XLOC_131254	2579	coding	noncoding	coding	noncoding
NR_126046.1	GLIDR	XLOC_131365	1038	coding	noncoding	noncoding	noncoding
MSTRG.124533.5	PGM5P2	XLOC_131401	1650	coding	noncoding	coding	noncoding
MSTRG.124498.1		XLOC_131428	1062	coding	noncoding	noncoding	noncoding
MSTRG.124500.1		XLOC_131429	1234	coding	noncoding	noncoding	noncoding
MSTRG.124618.1		XLOC_131478	2564	coding	coding	noncoding	noncoding
MSTRG.124780.1		XLOC_131544	411	coding	noncoding	noncoding	noncoding
MSTRG.124839.1		XLOC_131573	358	coding	coding	noncoding	noncoding
MSTRG.124840.8	LINC01410	XLOC_131574	4980	coding	noncoding	noncoding	noncoding
MSTRG.124886.3	LINC00537	XLOC_131612	1401	coding	noncoding	noncoding	noncoding
MSTRG.125033.6	LOC105379251	XLOC_131658	4193	coding	noncoding	noncoding	noncoding
MSTRG.125033.11	CBWD5	XLOC_131658	1344	coding	noncoding	noncoding	noncoding
MSTRG.125033.14	CBWD5	XLOC_131658	1203	coding	coding	noncoding	noncoding
MSTRG.124986.1		XLOC_131694	295	coding	noncoding	noncoding	noncoding

MSTRG.125093.2	CBWD3	XLOC_131732	5801	coding	coding	noncoding	noncoding
MSTRG.125093.7	CBWD3	XLOC_131732	1594	coding	coding	noncoding	noncoding
MSTRG.125093.11	CBWD3	XLOC_131732	1450	coding	coding	noncoding	noncoding
MSTRG.125093.12	CBWD3	XLOC_131732	868	coding	coding	noncoding	noncoding
MSTRG.125093.13	CBWD3	XLOC_131732	784	coding	coding	noncoding	noncoding
MSTRG.125318.8	SMC5	XLOC_131828	3024	coding	noncoding	noncoding	noncoding
NM_001102421.3	ZFAND5	XLOC_131920	5704	coding	coding	coding	coding
MSTRG.125674.1		XLOC_132056	205	coding	noncoding	noncoding	noncoding
MSTRG.125681.1		XLOC_132062	251	coding	noncoding	noncoding	noncoding
MSTRG.125923.5	CEP78	XLOC_132256	3356	coding	coding	coding	noncoding
MSTRG.126216.9	TLE4	XLOC_132358	5717	coding	coding	coding	noncoding
MSTRG.126430.1		XLOC_132377	341	coding	noncoding	noncoding	noncoding
MSTRG.126669.1	NAA35	XLOC_132623	408	coding	coding	noncoding	noncoding
XM_011519011.2	TUT7	XLOC_132636	8220	coding	coding	coding	coding
MSTRG.127044.3	SECISBP2	XLOC_132745	3323	coding	coding	noncoding	coding
MSTRG.127044.6	SEMA4D	XLOC_132745	4558	coding	coding	noncoding	noncoding
MSTRG.126969.10	SYK	XLOC_132796	12242	coding	noncoding	coding	noncoding
XM_011519073.2	ZNF484	XLOC_132932	4220	coding	coding	coding	coding
NM_004148.4	NINJ1	XLOC_132949	1237	coding	coding	coding	coding
MSTRG.127398.16	FAM120AOS	XLOC_132960	4433	coding	noncoding	coding	noncoding
MSTRG.127309.1	PTPDC1	XLOC_132976	1052	coding	noncoding	noncoding	noncoding
MSTRG.127310.1	PTPDC1	XLOC_132977	700	coding	noncoding	noncoding	noncoding
MSTRG.127301.1		XLOC_132995	271	coding	noncoding	noncoding	noncoding
MSTRG.127357.1		XLOC_133008	409	coding	noncoding	noncoding	noncoding
MSTRG.127627.1	SUGT1P4-STRA6LP-CCDC180	XLOC_133193	520	coding	noncoding	noncoding	noncoding
MSTRG.127842.3	NCBP1	XLOC_133219	2906	coding	coding	coding	noncoding
XM_005252086.1	HEMGN	XLOC_133235	2213	coding	noncoding	coding	coding
MSTRG.127924.5	TGFBR1	XLOC_133270	26724	coding	coding	coding	noncoding
MSTRG.128203.2	STX17	XLOC_133304	1455	coding	noncoding	noncoding	noncoding
NM_015051.3	ERP44	XLOC_133304	4808	coding	coding	coding	coding
MSTRG.128293.2	FSD1L	XLOC_133466	997	coding	noncoding	noncoding	noncoding
MSTRG.129067.2	C9orf147	XLOC_133829	3515	coding	coding	noncoding	noncoding
MSTRG.129067.9	C9orf147	XLOC_133829	1684	coding	noncoding	coding	noncoding
MSTRG.129096.1	KIAA1958	XLOC_133836	238	coding	noncoding	noncoding	noncoding
MSTRG.129036.2	SNX30	XLOC_133846	7376	coding	coding	coding	noncoding
MSTRG.129036.3	SNX30	XLOC_133846	3656	coding	noncoding	coding	noncoding
MSTRG.129124.5	FKBP15	XLOC_133860	8538	coding	coding	coding	coding
MSTRG.129153.7	RGS3	XLOC_133875	2379	coding	coding	coding	coding
MSTRG.129153.10	RGS3	XLOC_133875	2447	coding	coding	coding	coding
MSTRG.129256.1		XLOC_133947	210	coding	noncoding	noncoding	noncoding
MSTRG.129602.2	TRIM32	XLOC_134047	2294	coding	coding	coding	noncoding

MSTRG.129974.9	C5	XLOC_134181	7984	coding	coding	coding	coding
MSTRG.129974.11	C5	XLOC_134181	994	coding	noncoding	noncoding	noncoding
MSTRG.129918.1	RABGAP1	XLOC_134270	226	coding	noncoding	noncoding	noncoding
MSTRG.130219.1	DENND1A	XLOC_134306	325	coding	noncoding	noncoding	noncoding
MSTRG.130132.13	GAPVD1	XLOC_134419	3099	coding	coding	noncoding	coding
MSTRG.130281.3	PBX3	XLOC_134452	2044	coding	coding	noncoding	noncoding
MSTRG.130334.1	ZBTB34	XLOC_134497	6469	coding	coding	coding	coding
MSTRG.130334.4	ZBTB34	XLOC_134497	6880	coding	coding	noncoding	coding
MSTRG.130532.4	LCN2	XLOC_134587	813	coding	coding	noncoding	noncoding
MSTRG.130669.3	LOC101929331	XLOC_134651	2041	coding	noncoding	coding	noncoding
MSTRG.130719.2	LINC00963	XLOC_134673	1586	coding	noncoding	coding	noncoding
MSTRG.130719.5	LINC00963	XLOC_134673	2454	coding	noncoding	coding	noncoding
XM_017014492.2	FNBP1	XLOC_134693	2988	coding	coding	coding	coding
XM_017015413.2	LOC100996574	XLOC_134840	764	coding	noncoding	coding	coding
MSTRG.131097.2	RPL7A	XLOC_134854	1123	coding	coding	coding	noncoding
MSTRG.131612.3	EHMT1	XLOC_135124	3647	coding	coding	coding	coding
MSTRG.131598.10	FAM157B	XLOC_135141	1103	coding	noncoding	noncoding	noncoding
MSTRG.131895.1	KLF6	XLOC_135296	9378	coding	noncoding	coding	coding
MSTRG.132073.1		XLOC_135389	212	coding	noncoding	noncoding	noncoding
MSTRG.132177.1	LOC105376387	XLOC_135511	290	coding	noncoding	noncoding	noncoding
MSTRG.132378.1		XLOC_135639	289	coding	noncoding	noncoding	noncoding
MSTRG.132389.1		XLOC_135648	373	coding	noncoding	noncoding	noncoding
MSTRG.132447.1		XLOC_135683	1317	coding	noncoding	noncoding	noncoding
XM_017015562.1	CELF2	XLOC_135717	9301	coding	coding	coding	coding
XM_011519297.1	CELF2	XLOC_135717	9384	coding	coding	coding	coding
NM_001326348.1	CELF2	XLOC_135717	2955	coding	coding	coding	coding
MSTRG.132616.5	UPF2	XLOC_135795	4939	coding	coding	noncoding	noncoding
MSTRG.132616.6	UPF2	XLOC_135795	963	coding	coding	coding	noncoding
MSTRG.132616.9	UPF2	XLOC_135795	2329	coding	noncoding	noncoding	noncoding
MSTRG.132634.5	SEC61A2	XLOC_135800	6000	coding	coding	coding	coding
MSTRG.132832.9	FAM107B	XLOC_135964	4093	coding	coding	noncoding	noncoding
XM_006717503.3	SUV39H2	XLOC_135977	2617	coding	coding	coding	coding
MSTRG.133069.1		XLOC_136006	491	coding	noncoding	noncoding	noncoding
MSTRG.133070.2	MINDY3	XLOC_136007	1918	coding	noncoding	coding	noncoding
MSTRG.133171.2	TRDMT1	XLOC_136057	981	coding	noncoding	noncoding	noncoding
MSTRG.133171.8	VIM	XLOC_136057	1908	coding	coding	coding	noncoding
MSTRG.133366.3	NSUN6	XLOC_136140	1438	coding	coding	noncoding	noncoding
MSTRG.133573.2	PIP4K2A	XLOC_136306	5273	coding	coding	coding	noncoding
MSTRG.133573.4	PIP4K2A	XLOC_136306	4754	coding	coding	coding	noncoding
MSTRG.133708.3	ARHGAP21	XLOC_136370	4813	coding	coding	noncoding	noncoding
MSTRG.133824.19	ABI1	XLOC_136418	3146	coding	coding	noncoding	noncoding
MSTRG.133824.21	ABI1	XLOC_136418	1932	coding	coding	noncoding	noncoding

MSTRG.134074.1		XLOC_136517	290	coding	noncoding	noncoding	noncoding
MSTRG.134248.6	ZNF438	XLOC_136622	2899	coding	noncoding	coding	noncoding
MSTRG.134248.12	ZNF438	XLOC_136622	9468	coding	noncoding	noncoding	noncoding
MSTRG.134380.1	LOC102031319	XLOC_136686	6783	coding	coding	coding	noncoding
MSTRG.134380.10	LOC102031319	XLOC_136686	16306	coding	noncoding	coding	noncoding
MSTRG.134793.3	ITGB1	XLOC_136709	8650	coding	coding	noncoding	noncoding
MSTRG.134564.6		XLOC_136872	1646	coding	coding	noncoding	noncoding
MSTRG.134669.1	ZNF248	XLOC_136901	243	coding	coding	coding	noncoding
MSTRG.134698.3	ZNF25	XLOC_136925	2610	coding	coding	noncoding	noncoding
MSTRG.134698.4	ZNF25	XLOC_136925	2820	coding	coding	noncoding	noncoding
NM_001278175.2	ZNF33A	XLOC_136928	6180	coding	coding	coding	coding
MSTRG.135053.1	ZNF239	XLOC_137058	866	coding	noncoding	noncoding	noncoding
MSTRG.135501.1		XLOC_137157	9341	coding	noncoding	noncoding	noncoding
MSTRG.135362.1	FAM170B-AS1	XLOC_137329	412	coding	noncoding	noncoding	noncoding
MSTRG.135349.1		XLOC_137344	249	coding	noncoding	noncoding	noncoding
MSTRG.135903.1		XLOC_137549	329	coding	noncoding	noncoding	noncoding
MSTRG.136390.3	ANK3	XLOC_137616	4346	coding	coding	coding	noncoding
MSTRG.136391.1	ANK3	XLOC_137617	1468	coding	noncoding	noncoding	noncoding
MSTRG.136193.1	RTKN2	XLOC_137711	234	coding	coding	noncoding	noncoding
MSTRG.136256.1	EGR2	XLOC_137734	304	coding	noncoding	noncoding	noncoding
MSTRG.136634.3	JMJD1C	XLOC_137743	9803	coding	coding	coding	coding
MSTRG.136566.3	HERC4	XLOC_137851	3405	coding	coding	noncoding	noncoding
MSTRG.136566.8	HERC4	XLOC_137851	389	coding	coding	noncoding	noncoding
NM_004728.4	DDX21	XLOC_137886	4664	coding	coding	coding	coding
MSTRG.136806.2	TYSND1	XLOC_137955	966	coding	coding	coding	noncoding
MSTRG.137105.6	P4HA1	XLOC_138073	627	coding	noncoding	noncoding	noncoding
XM_011539740.2	FAM149B1	XLOC_138073	1447	coding	coding	noncoding	coding
MSTRG.137056.2	ANXA7	XLOC_138087	2299	coding	coding	noncoding	noncoding
MSTRG.137160.4	BMS1P4-AGAP5	XLOC_138105	1978	coding	coding	coding	coding
MSTRG.137248.6	AP3M1	XLOC_138120	467	coding	noncoding	noncoding	noncoding
MSTRG.137337.1	DLG5	XLOC_138304	244	coding	noncoding	noncoding	noncoding
MSTRG.137392.2	POLR3A	XLOC_138308	4652	coding	coding	coding	coding
MSTRG.137569.5	LOC642361	XLOC_138391	2254	coding	noncoding	coding	noncoding
MSTRG.137569.9	LOC642361	XLOC_138391	410	coding	noncoding	noncoding	noncoding
XM_011540192.3	TMEM254	XLOC_138414	955	coding	coding	noncoding	coding
MSTRG.137986.1		XLOC_138626	849	coding	noncoding	coding	noncoding
MSTRG.137952.6	ATAD1	XLOC_138646	1210	coding	coding	noncoding	noncoding
NM_001304718.2	PTEN	XLOC_138651	8629	coding	coding	coding	coding
MSTRG.138125.4	LOC105378415	XLOC_138666	20022	coding	noncoding	coding	noncoding
MSTRG.138125.7	LOC105378415	XLOC_138666	18478	coding	noncoding	coding	noncoding
MSTRG.138125.9	LOC105378415	XLOC_138666	15074	coding	noncoding	coding	noncoding
MSTRG.138125.20	LOC105378415	XLOC_138666	12611	coding	noncoding	coding	noncoding

XM_024447826.1	LIPJ	XLOC_138750	3402	coding	coding	noncoding	coding
NM_001198829.2	LIPF	XLOC_138751	1472	coding	coding	coding	coding
NR_135314.2	FAS	XLOC_138780	3783	coding	coding	coding	noncoding
NM_001270929.2	IFIT1	XLOC_138831	4411	coding	coding	coding	coding
MSTRG.138423.1	FLJ37201	XLOC_138851	1041	coding	coding	noncoding	noncoding
NM_001257101.1	PCGF5	XLOC_138909	7039	coding	coding	noncoding	coding
XM_017016880.2	BTAF1	XLOC_138970	8003	coding	coding	coding	coding
MSTRG.138913.1		XLOC_139101	380	coding	noncoding	noncoding	noncoding
MSTRG.139178.1	ENTPD1-AS1	XLOC_139187	949	coding	noncoding	noncoding	noncoding
XR_002956980.1	CC2D2B	XLOC_139189	3059	coding	coding	coding	coding
MSTRG.139246.1	ENTPD1-AS1	XLOC_139222	322	coding	noncoding	noncoding	noncoding
MSTRG.139045.2		XLOC_139295	3326	coding	noncoding	noncoding	noncoding
MSTRG.139311.3	RRP12	XLOC_139297	773	coding	noncoding	coding	noncoding
MSTRG.139423.5	HPS1	XLOC_139363	2557	coding	noncoding	coding	noncoding
MSTRG.139558.1	SEMA4G	XLOC_139455	3500	coding	coding	coding	coding
MSTRG.139619.6	POLL	XLOC_139489	2698	coding	coding	coding	coding
MSTRG.139674.4	OGA	XLOC_139498	6043	coding	coding	coding	noncoding
MSTRG.139674.12	OGA	XLOC_139498	8428	coding	coding	coding	noncoding
MSTRG.139653.2	PPRC1	XLOC_139514	3319	coding	coding	coding	coding
XM_011539864.3	SUFU	XLOC_139539	1883	coding	coding	coding	coding
MSTRG.139911.24	CNNM2	XLOC_139570	3448	coding	coding	noncoding	noncoding
MSTRG.139911.23	CNNM2	XLOC_139570	4054	coding	coding	noncoding	noncoding
MSTRG.139911.29	CNNM2	XLOC_139570	5520	coding	coding	coding	noncoding
MSTRG.139911.39	NT5C2	XLOC_139570	459	coding	noncoding	noncoding	noncoding
MSTRG.139826.5	CALHM2	XLOC_139585	1866	coding	coding	coding	coding
NM_001304743.2	SLK	XLOC_139622	7734	coding	coding	coding	coding
XM_011539273.2	GSTO2	XLOC_139637	6504	coding	coding	coding	coding
MSTRG.140029.17	ITPRIP	XLOC_139637	10307	coding	coding	coding	noncoding
MSTRG.140622.9	ZDHHC6	XLOC_139874	2879	coding	noncoding	noncoding	noncoding
MSTRG.140596.3	CCDC186	XLOC_140014	3765	coding	noncoding	noncoding	noncoding
MSTRG.140596.4	CCDC186	XLOC_140014	4613	coding	noncoding	noncoding	noncoding
MSTRG.140814.6	ABLIM1	XLOC_140029	5612	coding	coding	noncoding	noncoding
MSTRG.140814.4	ABLIM1	XLOC_140029	7355	coding	coding	coding	coding
MSTRG.140814.8	ABLIM1	XLOC_140029	8790	coding	coding	noncoding	noncoding
MSTRG.141251.2	ATE1	XLOC_140279	4573	coding	coding	coding	noncoding
MSTRG.141388.15	CHST15	XLOC_140375	6567	coding	coding	coding	noncoding
MSTRG.141388.17	CHST15	XLOC_140375	8495	coding	coding	coding	noncoding
MSTRG.141532.1	LOC105378539	XLOC_140391	9842	coding	coding	coding	noncoding
MSTRG.141629.7	CTBP2	XLOC_140405	2749	coding	coding	coding	coding
NM_130435.5	PTPRE	XLOC_140534	4915	coding	coding	coding	coding
XM_024447980.1	IDI1	XLOC_140816	1847	coding	coding	noncoding	coding
MSTRG.132015.1	NET1	XLOC_140977	232	coding	noncoding	noncoding	noncoding

MSTRG.132116.4	LOC105376384	XLOC_141001	2797	coding	coding	coding	coding
NM_001282645.1	PRKCQ	XLOC_141020	3224	coding	coding	coding	coding
MSTRG.132684.1	CELF2-AS1	XLOC_141185	16538	coding	coding	noncoding	noncoding
NM_001321648.1	NUDT5	XLOC_141240	3545	coding	coding	coding	coding
MSTRG.132876.10	CAMK1D	XLOC_141249	4486	coding	coding	coding	coding
MSTRG.132908.5	CAMK1D	XLOC_141255	2631	coding	noncoding	noncoding	noncoding
MSTRG.132997.4	RPP38-DT	XLOC_141347	1069	coding	coding	coding	noncoding
MSTRG.132997.5	RPP38-DT	XLOC_141347	1119	coding	coding	noncoding	noncoding
MSTRG.132997.6	RPP38	XLOC_141347	1119	coding	coding	noncoding	noncoding
MSTRG.133090.4	PTER	XLOC_141405	2411	coding	coding	noncoding	noncoding
MSTRG.133090.7	PTER	XLOC_141405	1846	coding	coding	noncoding	noncoding
MSTRG.133177.5	VIM-AS1	XLOC_141447	2174	coding	coding	coding	noncoding
NM_001270383.1	ENKUR	XLOC_141716	3295	coding	coding	coding	coding
MSTRG.133804.6	APBB1IP	XLOC_141752	1988	coding	coding	coding	noncoding
MSTRG.133804.11	APBB1IP	XLOC_141752	10370	coding	noncoding	coding	noncoding
NM_014263.4	YME1L1	XLOC_141785	4191	coding	coding	coding	coding
XM_017016106.1	MKX	XLOC_141822	3867	coding	coding	coding	coding
MSTRG.134077.3	WAC-AS1	XLOC_141870	4079	coding	coding	coding	noncoding
MSTRG.134330.12	ZEB1	XLOC_142023	4987	coding	coding	noncoding	coding
MSTRG.134330.11	ZEB1	XLOC_142023	4984	coding	coding	noncoding	coding
MSTRG.134330.29	LOC100505502	XLOC_142023	15500	coding	coding	coding	coding
MSTRG.134727.10	CCDC7	XLOC_142097	1322	coding	noncoding	coding	noncoding
MSTRG.134784.1	CCDC7	XLOC_142138	312	coding	coding	noncoding	noncoding
MSTRG.134840.6	ZNF37A	XLOC_142287	3387	coding	coding	noncoding	noncoding
MSTRG.134869.1	HSD17B7P2	XLOC_142300	256	coding	noncoding	noncoding	noncoding
MSTRG.134994.8	CSGALNACT2	XLOC_142374	3582	coding	coding	noncoding	noncoding
MSTRG.135058.2	ZNF485	XLOC_142407	1787	coding	coding	noncoding	noncoding
MSTRG.135109.1		XLOC_142439	298	coding	noncoding	noncoding	noncoding
MSTRG.135173.2	ALOX5	XLOC_142461	2481	coding	coding	coding	coding
MSTRG.135176.10	8-Mar	XLOC_142464	6143	coding	coding	coding	coding
MSTRG.135473.5	AGAP7P	XLOC_142477	2175	coding	coding	coding	coding
MSTRG.135473.6	WASHC2C	XLOC_142477	248	coding	coding	noncoding	noncoding
MSTRG.135478.3	WASHC2C	XLOC_142478	4705	coding	coding	coding	noncoding
MSTRG.135488.1	NCOA4	XLOC_142481	3428	coding	coding	coding	coding
NM_001145260.1	NCOA4	XLOC_142481	3738	coding	coding	coding	coding
MSTRG.135491.9	LOC107984026	XLOC_142483	1165	coding	noncoding	coding	noncoding
MSTRG.135566.4	WASHC2A	XLOC_142611	2542	coding	coding	coding	noncoding
MSTRG.135944.2	TFAM	XLOC_142867	1710	coding	coding	noncoding	noncoding
MSTRG.136625.3	NRBF2	XLOC_143074	1681	coding	coding	noncoding	noncoding
MSTRG.136625.4	NRBF2	XLOC_143074	1535	coding	coding	noncoding	noncoding
NM_001322258.2	JMJD1C	XLOC_143078	8322	coding	coding	coding	coding
NM_001318154.2	JMJD1C	XLOC_143078	8428	coding	coding	coding	coding

NM_022079.3	HERC4	XLOC_143177	4448	coding	coding	coding	coding
MSTRG.136790.1		XLOC_143294	214	coding	noncoding	noncoding	noncoding
MSTRG.136999.40	ASCC1	XLOC_143397	1658	coding	coding	noncoding	noncoding
MSTRG.137033.2	MCU	XLOC_143425	2812	coding	coding	noncoding	noncoding
NR_160427.1	BMS1P4-AGAP5	XLOC_143489	3796	coding	coding	coding	coding
MSTRG.137167.4	SEC24C	XLOC_143494	4418	coding	coding	coding	noncoding
MSTRG.137231.8	SAMD8	XLOC_143553	1767	coding	coding	noncoding	noncoding
MSTRG.138042.2	LRMDA	XLOC_143564	832	coding	coding	coding	noncoding
MSTRG.137659.4	TSPAN14	XLOC_143725	1395	coding	coding	coding	coding
MSTRG.137659.11	TSPAN14	XLOC_143725	6599	coding	noncoding	noncoding	noncoding
MSTRG.137804.3	CCSER2	XLOC_143816	3044	coding	noncoding	coding	noncoding
MSTRG.137804.5	CCSER2	XLOC_143816	2757	coding	noncoding	coding	noncoding
XR_002957094.1	LOC112268064	XLOC_143903	2198	coding	noncoding	noncoding	noncoding
XM_011540303.3	ATAD1	XLOC_143928	1706	coding	coding	coding	coding
MSTRG.138017.6	KLLN	XLOC_143932	11876	coding	coding	coding	noncoding
MSTRG.138396.5	LOC107984251	XLOC_144036	12644	coding	coding	coding	noncoding
MSTRG.138560.1		XLOC_144141	367	coding	coding	noncoding	noncoding
NM_014391.2	ANKRD1	XLOC_144142	1974	coding	coding	coding	coding
MSTRG.138662.4	BTAF1	XLOC_144173	3210	coding	coding	coding	noncoding
MSTRG.138662.5	BTAF1	XLOC_144173	8021	coding	coding	coding	noncoding
MSTRG.138781.1	EXOC6	XLOC_144198	2556	coding	coding	noncoding	noncoding
MSTRG.138859.1		XLOC_144244	1048	coding	noncoding	noncoding	noncoding
MSTRG.139075.6	LOC102723665	XLOC_144419	10355	coding	coding	coding	noncoding
XM_017016494.1	AVPI1	XLOC_144457	1566	coding	coding	coding	coding
MSTRG.139264.1	ZFYVE27	XLOC_144461	1421	coding	coding	coding	noncoding
NM_001278.5	CHUK	XLOC_144569	3600	coding	coding	coding	coding
MSTRG.139595.1	BTRC	XLOC_144623	686	coding	noncoding	noncoding	noncoding
MSTRG.139616.40	DPCD	XLOC_144624	802	coding	coding	noncoding	noncoding
MSTRG.139665.2	NFKB2	XLOC_144675	2803	coding	coding	coding	noncoding
MSTRG.139725.4	RPARP-AS1	XLOC_144683	443	coding	noncoding	noncoding	noncoding
MSTRG.139824.3	PDCD11	XLOC_144756	2596	coding	coding	noncoding	noncoding
MSTRG.140210.1		XLOC_144973	343	coding	noncoding	noncoding	noncoding
XM_006717846.3	ABLIM1	XLOC_145207	7808	coding	coding	coding	coding
MSTRG.141031.9	FAM45A	XLOC_145377	2535	coding	coding	noncoding	noncoding
NM_001256379.1	MCMBP	XLOC_145415	4102	coding	coding	coding	coding
MSTRG.141089.4	SEC23IP	XLOC_145421	3729	coding	coding	noncoding	noncoding
MSTRG.141419.1		XLOC_145561	601	coding	noncoding	noncoding	noncoding
MSTRG.141467.1		XLOC_145604	306	coding	noncoding	noncoding	noncoding
MSTRG.141490.1		XLOC_145624	910	coding	noncoding	noncoding	noncoding
MSTRG.141628.10	CTBP2	XLOC_145721	6435	coding	coding	noncoding	noncoding
MSTRG.141628.11	CTBP2	XLOC_145721	8402	coding	coding	coding	coding
MSTRG.141618.5	EDRF1-AS1	XLOC_145739	4452	coding	noncoding	coding	coding

MSTRG.142328.7	RNH1	XLOC_146130	1695	coding	coding	coding	coding
XM_011520236.3	PHRF1	XLOC_146136	5557	coding	coding	coding	coding
MSTRG.142339.11	IRF7	XLOC_146136	1821	coding	coding	coding	noncoding
NM_001242932.1	LSP1	XLOC_146204	2016	coding	coding	coding	coding
NM_001256235.1	PGAP2	XLOC_146325	1815	coding	coding	coding	coding
XM_011519997.1	PGAP2	XLOC_146325	2392	coding	coding	coding	coding
MSTRG.142724.1		XLOC_146361	246	coding	noncoding	noncoding	noncoding
MSTRG.142837.1		XLOC_146367	399	coding	coding	coding	noncoding
NM_001004754.2	OR51I2	XLOC_146434	1070	coding	coding	coding	coding
MSTRG.143136.20	ILK	XLOC_146551	1524	coding	coding	coding	noncoding
MSTRG.143271.3	TRIM66	XLOC_146658	1364	coding	noncoding	noncoding	noncoding
MSTRG.143325.6	DENND5A	XLOC_146688	4915	coding	coding	coding	coding
MSTRG.144081.3	COPB1	XLOC_146921	3276	coding	coding	noncoding	noncoding
MSTRG.143960.12	SOX6	XLOC_146994	3695	coding	coding	noncoding	noncoding
MSTRG.144130.2	HPS5	XLOC_147079	4455	coding	noncoding	noncoding	noncoding
MSTRG.144130.5	GTF2H1	XLOC_147079	4731	coding	noncoding	coding	noncoding
MSTRG.144191.7	LDHAL6A	XLOC_147086	3113	coding	coding	coding	coding
XM_005252729.3	HIPK3	XLOC_147557	7497	coding	coding	coding	coding
XM_017017076.1	HIPK3	XLOC_147557	7107	coding	coding	coding	coding
MSTRG.145109.4	LMO2	XLOC_147587	1807	coding	coding	coding	noncoding
MSTRG.145109.5	LMO2	XLOC_147587	1958	coding	coding	coding	coding
MSTRG.145596.1		XLOC_147876	311	coding	noncoding	noncoding	noncoding
MSTRG.146052.10	PHF21A	XLOC_148073	4189	coding	coding	coding	coding
MSTRG.146052.11	PHF21A	XLOC_148073	12780	coding	coding	coding	coding
MSTRG.146052.20	PHF21A	XLOC_148073	2586	coding	noncoding	noncoding	noncoding
NM_001251935.1	NR1H3	XLOC_148120	1920	coding	coding	coding	coding
MSTRG.146160.4	SPI1	XLOC_148130	3759	coding	coding	coding	coding
MSTRG.146201.2	FNBP4	XLOC_148151	5322	coding	coding	coding	coding
MSTRG.146469.4	SLC43A3	XLOC_148336	2556	coding	coding	noncoding	noncoding
MSTRG.146479.2	TIMM10	XLOC_148344	1414	coding	coding	coding	coding
MSTRG.146545.1		XLOC_148385	248	coding	noncoding	noncoding	noncoding
NM_001369455.1	FAM111A	XLOC_148456	3907	coding	coding	coding	coding
MSTRG.146697.1	DTX4	XLOC_148466	9237	coding	coding	coding	coding
MSTRG.146828.2	MS4A4E	XLOC_148522	747	coding	noncoding	noncoding	noncoding
NM_206938.1	MS4A7	XLOC_148527	2874	coding	coding	coding	coding
MSTRG.146931.1	CPSF7	XLOC_148602	3660	coding	coding	coding	coding
MSTRG.147043.13	AHNAK	XLOC_148658	11914	coding	noncoding	coding	coding
MSTRG.147043.27	AHNAK	XLOC_148658	14802	coding	noncoding	coding	coding
NM_001265591.2	RTN3	XLOC_148721	2273	coding	coding	coding	coding
NM_001265589.2	RTN3	XLOC_148721	4917	coding	coding	coding	coding
MSTRG.147187.1	C11orf95	XLOC_148725	423	coding	coding	noncoding	noncoding
XM_005273783.3	SPINDOC	XLOC_148727	1988	coding	coding	coding	coding

MSTRG.147308.4	RASGRP2	XLOC_148785	2330	coding	coding	coding	noncoding
MSTRG.147315.12	SF1	XLOC_148789	5704	coding	coding	coding	noncoding
MSTRG.147297.2	MAP4K2	XLOC_148792	714	coding	coding	noncoding	noncoding
MSTRG.147424.3	NEAT1	XLOC_148840	13107	coding	noncoding	noncoding	noncoding
MSTRG.147424.2	NEAT1	XLOC_148840	21520	coding	noncoding	coding	noncoding
NR_002819.4	MALAT1	XLOC_148861	8779	coding	noncoding	noncoding	noncoding
XM_024448620.1	SCYL1	XLOC_148864	2780	coding	coding	coding	coding
MSTRG.147472.1		XLOC_148869	274	coding	noncoding	noncoding	noncoding
MSTRG.147502.6	SNX32	XLOC_148888	1173	coding	coding	coding	coding
MSTRG.147656.8	RBM4	XLOC_148981	2297	coding	coding	coding	coding
MSTRG.147805.1	ACY3	XLOC_149064	293	coding	coding	noncoding	noncoding
MSTRG.147901.1	MRPL21	XLOC_149144	700	coding	coding	noncoding	coding
MSTRG.148156.37	LRTOMT	XLOC_149253	1419	coding	coding	coding	coding
MSTRG.148315.13	KCNE3	XLOC_149332	2385	coding	coding	noncoding	noncoding
MSTRG.148678.4	PAK1	XLOC_149501	4983	coding	coding	noncoding	noncoding
MSTRG.149026.6	KCTD21-AS1	XLOC_149528	2447	coding	coding	coding	coding
MSTRG.149030.16	GAB2	XLOC_149529	10285	coding	noncoding	coding	coding
MSTRG.149143.5	CREBZF	XLOC_149802	6784	coding	noncoding	coding	noncoding
MSTRG.149140.1	CCDC89	XLOC_149809	434	coding	noncoding	noncoding	noncoding
MSTRG.150042.22	CEP295	XLOC_150098	4975	coding	coding	noncoding	coding
MSTRG.149936.1		XLOC_150124	304	coding	coding	noncoding	noncoding
MSTRG.149976.4	MRE11	XLOC_150146	4428	coding	coding	noncoding	noncoding
XR_947911.2	LINC02700	XLOC_150159	11925	coding	coding	coding	coding
MSTRG.150651.6	MAML2	XLOC_150264	9543	coding	noncoding	noncoding	noncoding
MSTRG.150651.15	MIR1260B	XLOC_150264	19840	coding	coding	noncoding	noncoding
MSTRG.150509.1	MMP8	XLOC_150444	2962	coding	coding	coding	noncoding
MSTRG.150509.3	MMP8	XLOC_150444	1611	coding	coding	coding	noncoding
MSTRG.150791.1	PDGFD	XLOC_150491	1446	coding	noncoding	noncoding	noncoding
MSTRG.150916.12	CASP1	XLOC_150526	2568	coding	coding	noncoding	noncoding
MSTRG.151254.1	ATM	XLOC_150643	21847	coding	coding	coding	coding
XM_011542844.3	ATM	XLOC_150643	10076	coding	coding	coding	coding
XM_011542845.2	ATM	XLOC_150643	8588	coding	coding	coding	coding
MSTRG.151321.1	NKAPD1	XLOC_150813	4202	coding	coding	coding	coding
MSTRG.151548.2	USP28	XLOC_150892	4487	coding	coding	coding	noncoding
MSTRG.151548.3	USP28	XLOC_150892	4346	coding	coding	coding	noncoding
MSTRG.151748.2	ZPR1	XLOC_150990	2616	coding	coding	noncoding	noncoding
NM_001558.3	IL10RA	XLOC_151079	3656	coding	coding	coding	coding
MSTRG.151948.2	JAML	XLOC_151094	1750	coding	coding	noncoding	coding
NM_032873.5	UBASH3B	XLOC_151310	6865	coding	coding	coding	coding
XM_024448623.1	GRAMD1B	XLOC_151348	1861	coding	noncoding	coding	coding
XR_001748011.2	TBRG1	XLOC_151399	5357	coding	coding	coding	coding
XM_017018286.1	FAM118B	XLOC_151486	2445	coding	coding	coding	coding

NM_002017.5	FLI1	XLOC_151567	3825	coding	coding	coding	coding
MSTRG.152891.1		XLOC_151678	390	coding	noncoding	noncoding	noncoding
MSTRG.152923.1		XLOC_151703	260	coding	noncoding	noncoding	noncoding
MSTRG.152935.1		XLOC_151714	404	coding	noncoding	noncoding	noncoding
MSTRG.142373.1	LOC105376504	XLOC_151894	3424	coding	noncoding	noncoding	coding
XM_005253046.1	SIGIRR	XLOC_151900	2016	coding	coding	coding	coding
MSTRG.142425.2	CD151	XLOC_151940	1472	coding	coding	coding	coding
MSTRG.142536.6	C11orf21	XLOC_152007	736	coding	coding	coding	noncoding
MSTRG.142722.1		XLOC_152111	273	coding	coding	noncoding	noncoding
MSTRG.142830.1	TRIM21	XLOC_152112	12702	coding	coding	coding	coding
MSTRG.142830.6	OR52K2	XLOC_152112	28075	coding	coding	coding	noncoding
MSTRG.142830.13	OR52K2	XLOC_152112	14229	coding	coding	noncoding	noncoding
MSTRG.142830.18		XLOC_152112	30155	coding	coding	coding	coding
MSTRG.142830.20		XLOC_152112	12275	coding	coding	noncoding	noncoding
XM_017018462.2	TRIM5	XLOC_152169	1595	coding	coding	coding	coding
NM_001242854.1	ARFIP2	XLOC_152230	3846	coding	coding	coding	coding
MSTRG.143123.1	RRP8	XLOC_152254	292	coding	noncoding	noncoding	noncoding
MSTRG.143137.1	TAF10	XLOC_152258	2811	coding	coding	coding	noncoding
MSTRG.143195.2	PPFIBP2	XLOC_152304	3759	coding	coding	coding	noncoding
MSTRG.143239.2	TMEM9B	XLOC_152363	1982	coding	coding	coding	noncoding
MSTRG.143239.6	TMEM9B	XLOC_152363	1275	coding	noncoding	noncoding	noncoding
MSTRG.143346.3	ZNF143	XLOC_152409	3631	coding	coding	coding	coding
MSTRG.143346.4	ZNF143	XLOC_152409	2778	coding	coding	coding	coding
MSTRG.143433.4	AMPD3	XLOC_152504	3667	coding	coding	coding	coding
MSTRG.143442.1	MRVI1	XLOC_152507	5261	coding	coding	coding	coding
MSTRG.143726.2	FAR1	XLOC_152621	5741	coding	coding	noncoding	noncoding
MSTRG.144118.7	CYP2R1	XLOC_152683	4052	coding	coding	coding	coding
MSTRG.144118.10	PDE3B	XLOC_152683	15918	coding	coding	coding	noncoding
MSTRG.143835.1		XLOC_152697	275	coding	noncoding	noncoding	noncoding
MSTRG.144014.1	NCR3LG1	XLOC_152805	997	coding	coding	noncoding	noncoding
MSTRG.144418.2	HTATIP2	XLOC_152954	1683	coding	coding	noncoding	noncoding
MSTRG.144446.1	ANO5	XLOC_153006	250	coding	noncoding	noncoding	noncoding
MSTRG.144454.1	ANO5	XLOC_153013	246	coding	coding	noncoding	noncoding
MSTRG.144571.1	GAS2	XLOC_153031	343	coding	coding	coding	noncoding
NM_148893.3	SVIP	XLOC_153042	4479	coding	coding	noncoding	noncoding
MSTRG.145013.2	LOC105376615	XLOC_153363	7339	coding	coding	coding	coding
MSTRG.145077.9	HIPK3	XLOC_153393	1584	coding	noncoding	noncoding	noncoding
MSTRG.146134.1	ZNF408	XLOC_153843	737	coding	noncoding	noncoding	noncoding
MSTRG.146833.2	MS4A4A	XLOC_154194	1639	coding	coding	noncoding	coding
MSTRG.146794.5	MS4A7	XLOC_154198	1067	coding	coding	noncoding	noncoding
MSTRG.146911.13	TMEM138	XLOC_154241	625	coding	coding	noncoding	noncoding
MSTRG.146985.5	LOC107984334	XLOC_154276	1952	coding	coding	coding	noncoding

MSTRG.147024.1	ASRGL1	XLOC_154309	286	coding	coding	noncoding	noncoding
MSTRG.147148.4	TTC9C	XLOC_154341	1294	coding	noncoding	coding	noncoding
NM_018093.3	WDR74	XLOC_154352	1373	coding	coding	coding	coding
MSTRG.147131.1		XLOC_154391	378	coding	noncoding	noncoding	noncoding
MSTRG.147232.1	TRPT1	XLOC_154421	3498	coding	coding	coding	coding
MSTRG.147235.4	NUDT22	XLOC_154422	1175	coding	noncoding	coding	noncoding
MSTRG.147235.5	DNAJC4	XLOC_154422	1156	coding	coding	coding	noncoding
NM_138689.3	PPP1R14B	XLOC_154425	989	coding	coding	coding	coding
MSTRG.147435.6	NEAT1	XLOC_154510	19411	coding	noncoding	noncoding	coding
MSTRG.147435.8	NEAT1	XLOC_154510	11585	coding	noncoding	coding	noncoding
MSTRG.147435.17	NEAT1	XLOC_154510	3518	coding	noncoding	noncoding	coding
MSTRG.147419.11	TALAM1	XLOC_154517	6403	coding	noncoding	noncoding	noncoding
MSTRG.147419.12	TALAM1	XLOC_154517	6139	coding	noncoding	noncoding	noncoding
MSTRG.147756.4	TBC1D10C	XLOC_154664	2244	coding	coding	coding	coding
XR_001747983.2	TMEM134	XLOC_154674	1107	coding	coding	coding	coding
XR_001748282.2	LOC107984344	XLOC_154758	907	coding	noncoding	coding	noncoding
MSTRG.148207.2	P2RY2	XLOC_154960	1176	coding	coding	coding	noncoding
MSTRG.148439.4	XRRA1	XLOC_155056	14995	coding	coding	coding	noncoding
MSTRG.148439.6	XRRA1	XLOC_155056	16330	coding	coding	coding	noncoding
MSTRG.148635.2	ACER3	XLOC_155144	1277	coding	coding	noncoding	noncoding
MSTRG.148655.1	GDPD4	XLOC_155154	357	coding	noncoding	noncoding	noncoding
XM_017017847.2	PAK1	XLOC_155157	3095	coding	coding	coding	coding
XM_017018388.2	PICALM	XLOC_155530	3451	coding	coding	coding	coding
MSTRG.149369.4	HIKESHI	XLOC_155568	937	coding	coding	noncoding	noncoding
MSTRG.149679.1	LOC101929174	XLOC_155666	273	coding	noncoding	noncoding	noncoding
MSTRG.150060.3	MED17	XLOC_155845	1145	coding	noncoding	coding	noncoding
MSTRG.149945.1		XLOC_155858	542	coding	noncoding	noncoding	noncoding
MSTRG.149985.5	ANKRD49	XLOC_155878	3821	coding	coding	noncoding	noncoding
MSTRG.150125.3	SESN3	XLOC_155913	9771	coding	coding	coding	coding
MSTRG.150185.15	CEP57	XLOC_155926	3411	coding	coding	noncoding	noncoding
MSTRG.150727.1	MAML2	XLOC_156006	714	coding	noncoding	noncoding	noncoding
MSTRG.150226.2	JRKL	XLOC_156044	2876	coding	coding	noncoding	noncoding
XM_011542735.3	ANGPTL5	XLOC_156191	2693	coding	coding	noncoding	coding
NM_001257119.3	CASP1	XLOC_156312	1933	coding	coding	coding	coding
MSTRG.150991.1	LOC105369477	XLOC_156378	291	coding	noncoding	noncoding	noncoding
MSTRG.151245.2	ACAT1	XLOC_156417	660	coding	coding	noncoding	noncoding
MSTRG.151257.1	C11orf65	XLOC_156421	10964	coding	coding	coding	noncoding
MSTRG.151257.20	C11orf65	XLOC_156421	11320	coding	coding	noncoding	noncoding
MSTRG.151257.19	C11orf65	XLOC_156421	11306	coding	coding	noncoding	noncoding
MSTRG.151257.28	C11orf65	XLOC_156421	3177	coding	coding	noncoding	noncoding
MSTRG.151097.1	ZC3H12C	XLOC_156453	2400	coding	noncoding	coding	noncoding
MSTRG.151118.1		XLOC_156470	290	coding	noncoding	noncoding	noncoding

XM_024448598.1	PPP2R1B	XLOC_156531	5995	coding	coding	coding	coding
MSTRG.151296.1		XLOC_156562	243	coding	noncoding	noncoding	noncoding
MSTRG.151404.1		XLOC_156569	949	coding	noncoding	noncoding	noncoding
XR_001748023.2	ZPR1	XLOC_156798	3073	coding	coding	coding	coding
XM_011542726.1	SIK3	XLOC_156803	2588	coding	coding	coding	coding
MSTRG.151755.2	PAFAH1B2	XLOC_156857	3904	coding	coding	coding	noncoding
NM_001164837.2	FXYD6	XLOC_156882	2132	coding	coding	coding	noncoding
MSTRG.151967.3	LOC100131626	XLOC_156912	5865	coding	coding	noncoding	noncoding
MSTRG.151967.8	LOC100131626	XLOC_156912	8712	coding	coding	noncoding	noncoding
MSTRG.152018.3	LOC101929089	XLOC_156914	17279	coding	coding	coding	noncoding
XM_005271422.3	DPAGT1	XLOC_156947	2021	coding	coding	coding	coding
MSTRG.152291.1		XLOC_157100	259	coding	noncoding	noncoding	noncoding
MSTRG.152526.6	SIAE	XLOC_157210	3433	coding	coding	noncoding	noncoding
MSTRG.152796.3	ST3GAL4	XLOC_157274	1562	coding	coding	coding	coding
NM_001161707.1	KIRREL3	XLOC_157276	2534	coding	coding	coding	coding
MSTRG.152871.11	FLI1	XLOC_157365	5042	coding	coding	coding	coding
MSTRG.152871.16	SENCR	XLOC_157365	7424	coding	coding	coding	coding
MSTRG.153082.5	APLP2	XLOC_157433	2259	coding	coding	coding	coding
MSTRG.153398.9	CCDC77	XLOC_157605	5472	coding	coding	coding	coding
MSTRG.153374.2	LOC101929432	XLOC_157617	1008	coding	noncoding	noncoding	noncoding
MSTRG.153431.1	FBXL14	XLOC_157661	2783	coding	coding	coding	coding
MSTRG.153756.10	PARP11	XLOC_157728	3863	coding	coding	coding	noncoding
MSTRG.153756.13	PARP11	XLOC_157728	1556	coding	coding	noncoding	noncoding
MSTRG.153849.16	TAPBPL	XLOC_157833	5144	coding	coding	coding	coding
MSTRG.153887.5	ZNF384	XLOC_157849	2845	coding	coding	noncoding	noncoding
XM_024449106.1	PTPN6	XLOC_157877	2152	coding	coding	coding	coding
NM_002831.6	PTPN6	XLOC_157877	2161	coding	coding	coding	coding
MSTRG.153977.1		XLOC_157896	269	coding	noncoding	noncoding	noncoding
MSTRG.154117.1	LOC107987171	XLOC_157947	326	coding	noncoding	noncoding	noncoding
MSTRG.154129.1		XLOC_157956	234	coding	noncoding	noncoding	noncoding
MSTRG.154136.1	LINC00937	XLOC_157962	302	coding	noncoding	noncoding	noncoding
MSTRG.154142.1	LINC00937	XLOC_157967	340	coding	noncoding	noncoding	noncoding
MSTRG.154175.8	CLEC4E	XLOC_157984	2890	coding	coding	noncoding	noncoding
MSTRG.154380.6	PHC1	XLOC_157998	7860	coding	coding	coding	coding
MSTRG.154380.15	PHC1	XLOC_157998	2325	coding	coding	noncoding	noncoding
MSTRG.154238.1		XLOC_158123	280	coding	noncoding	coding	noncoding
MSTRG.154245.5	CLEC7A	XLOC_158126	3935	coding	coding	noncoding	noncoding
MSTRG.154245.7	CLEC7A	XLOC_158126	3643	coding	coding	noncoding	noncoding
MSTRG.154242.1	OLR1	XLOC_158129	641	coding	coding	noncoding	noncoding
MSTRG.154488.1	LOC101928100	XLOC_158150	14181	coding	coding	coding	coding
MSTRG.154488.5	LOC101928100	XLOC_158150	14494	coding	coding	coding	noncoding
MSTRG.154488.11	LOC101928100	XLOC_158150	2876	coding	coding	noncoding	coding

MSTRG.154488.19	KLRC4-KLRK1	XLOC_158150	8528	coding	coding	noncoding	noncoding
MSTRG.154438.1		XLOC_158152	232	coding	coding	noncoding	noncoding
MSTRG.154461.1		XLOC_158171	519	coding	noncoding	noncoding	noncoding
MSTRG.154562.1	PRH1-PRR4	XLOC_158188	235	coding	noncoding	noncoding	noncoding
MSTRG.154557.4	DUSP16	XLOC_158307	1036	coding	noncoding	noncoding	noncoding
MSTRG.154716.1	GPRC5D	XLOC_158350	424	coding	noncoding	noncoding	noncoding
MSTRG.154878.2	ERP27	XLOC_158452	2472	coding	coding	noncoding	noncoding
MSTRG.154929.1	EPS8	XLOC_158486	315	coding	coding	noncoding	noncoding
MSTRG.155389.11	GOLT1B	XLOC_158643	3330	coding	coding	noncoding	noncoding
MSTRG.155507.4	C2CD5	XLOC_158698	625	coding	noncoding	noncoding	noncoding
MSTRG.155712.6	ETFRF1	XLOC_158845	6341	coding	coding	noncoding	noncoding
MSTRG.155712.8	KRAS	XLOC_158845	4762	coding	noncoding	noncoding	noncoding
MSTRG.155905.10	ITPR2	XLOC_158887	3548	coding	coding	coding	noncoding
NM_001171887.2	FGFR1OP2	XLOC_158900	2818	coding	coding	coding	coding
NM_004264.5	MED21	XLOC_158901	2669	coding	coding	coding	coding
XM_024448893.1	STK38L	XLOC_158914	5458	coding	coding	coding	coding
MSTRG.155796.1		XLOC_158957	318	coding	noncoding	noncoding	noncoding
NM_001352086.2	CCDC91	XLOC_158961	2786	coding	noncoding	coding	coding
MSTRG.156158.1	CCDC91	XLOC_158977	325	coding	noncoding	noncoding	noncoding
MSTRG.156200.12	ERGIC2	XLOC_159015	3848	coding	coding	noncoding	noncoding
MSTRG.156262.2	CAPRIN2	XLOC_159140	4015	coding	coding	noncoding	noncoding
XM_011520558.2	FGD4	XLOC_159204	7297	coding	coding	coding	coding
MSTRG.156789.2	YAF2	XLOC_159422	1441	coding	coding	coding	noncoding
NM_001364833.2	PPHLN1	XLOC_159426	2483	coding	noncoding	coding	coding
XM_024449008.1	IRAK4	XLOC_159476	1643	coding	noncoding	noncoding	coding
MSTRG.157091.9	SCAF11	XLOC_159582	5202	coding	noncoding	noncoding	noncoding
MSTRG.157393.29	PCED1B	XLOC_159668	1259	coding	noncoding	noncoding	noncoding
MSTRG.157332.1	LINC02416	XLOC_159685	461	coding	noncoding	noncoding	noncoding
MSTRG.157410.2	RPAP3	XLOC_159720	3140	coding	coding	noncoding	noncoding
MSTRG.157440.3	PFKM	XLOC_159739	8616	coding	coding	coding	coding
MSTRG.157440.2	PFKM	XLOC_159739	8259	coding	coding	coding	coding
NM_001354741.1	PFKM	XLOC_159739	3310	coding	coding	coding	coding
MSTRG.157500.2	ZNF641	XLOC_159753	14264	coding	coding	noncoding	noncoding
MSTRG.157500.6	ZNF641	XLOC_159753	2701	coding	coding	coding	noncoding
MSTRG.157804.5	KCNH3	XLOC_159818	5899	coding	coding	coding	coding
NM_001098576.1	TMBIM6	XLOC_159821	3254	coding	coding	coding	coding
MSTRG.157938.2	SLC11A2	XLOC_159903	2109	coding	coding	noncoding	coding
MSTRG.157938.5	SLC11A2	XLOC_159903	1571	coding	coding	noncoding	coding
NM_001136264.1	DAZAP2	XLOC_159915	2100	coding	coding	coding	coding
MSTRG.157992.1		XLOC_159928	290	coding	coding	noncoding	noncoding
MSTRG.158351.1		XLOC_160085	214	coding	noncoding	noncoding	noncoding
MSTRG.158411.1	TESPA1	XLOC_160089	4173	coding	coding	noncoding	noncoding

MSTRG.158432.9	LOC105369779	XLOC_160128	2489	coding	coding	coding	noncoding
MSTRG.158445.5	SARNP	XLOC_160129	543	coding	coding	noncoding	noncoding
MSTRG.158582.2	PRIM1	XLOC_160197	2138	coding	coding	noncoding	coding
MSTRG.158648.6	R3HDM2	XLOC_160232	3203	coding	coding	coding	noncoding
MSTRG.158648.7	R3HDM2	XLOC_160232	2194	coding	coding	noncoding	noncoding
MSTRG.158992.15	MON2	XLOC_160399	307	coding	noncoding	noncoding	noncoding
NM_001251922.2	RAP1B	XLOC_160822	13237	coding	coding	coding	noncoding
XM_006719400.4	MDM2	XLOC_160839	7270	coding	coding	coding	coding
MSTRG.159602.8	MDM2	XLOC_160839	637	coding	coding	noncoding	noncoding
MSTRG.160077.1	LOC105369842	XLOC_161071	557	coding	noncoding	noncoding	noncoding
MSTRG.161249.2	DUSP6	XLOC_161492	5548	coding	coding	coding	coding
MSTRG.161249.12	POC1B-GALNT4	XLOC_161492	2573	coding	coding	noncoding	noncoding
MSTRG.161249.18	POC1B	XLOC_161492	975	coding	noncoding	noncoding	noncoding
XR_945216.2	LOC105369908	XLOC_161652	2188	coding	noncoding	noncoding	noncoding
MSTRG.161190.2	NUDT4	XLOC_161673	12129	coding	coding	coding	coding
MSTRG.161565.2	CEP83	XLOC_161745	986	coding	noncoding	noncoding	noncoding
MSTRG.161565.3	CEP83	XLOC_161745	912	coding	noncoding	noncoding	noncoding
MSTRG.161401.2	NR2C1	XLOC_161812	2417	coding	coding	coding	noncoding
MSTRG.161695.14	CDK17	XLOC_161867	1018	coding	noncoding	coding	noncoding
MSTRG.161695.15	CDK17	XLOC_161867	4348	coding	noncoding	noncoding	noncoding
MSTRG.161771.5	UHRF1BP1L	XLOC_161990	1986	coding	coding	noncoding	coding
MSTRG.161771.4	UHRF1BP1L	XLOC_161990	1931	coding	coding	noncoding	coding
MSTRG.161771.6	UHRF1BP1L	XLOC_161990	1879	coding	coding	noncoding	coding
MSTRG.161771.7	UHRF1BP1L	XLOC_161990	600	coding	noncoding	coding	noncoding
XR_001748816.1	CHPT1	XLOC_162048	2788	coding	coding	coding	coding
MSTRG.161928.6	WASHC3	XLOC_162063	1531	coding	coding	noncoding	noncoding
XM_017019369.1	CHST11	XLOC_162204	5339	coding	coding	coding	coding
MSTRG.162214.1		XLOC_162337	458	coding	coding	noncoding	coding
MSTRG.162620.4	SELPLG	XLOC_162483	4356	coding	noncoding	coding	coding
MSTRG.162620.3	SELPLG	XLOC_162483	4420	coding	noncoding	coding	coding
MSTRG.162624.4	LOC105369968	XLOC_162484	8114	coding	coding	coding	coding
XM_017020025.2	TCHP	XLOC_162532	3294	coding	coding	coding	coding
MSTRG.162793.5	GIT2	XLOC_162541	6887	coding	coding	coding	noncoding
MSTRG.162793.4	GIT2	XLOC_162541	5547	coding	coding	coding	noncoding
XM_005253981.3	ANKRD13A	XLOC_162542	3910	coding	coding	coding	coding
MSTRG.162853.1	VPS29	XLOC_162567	2586	coding	coding	noncoding	noncoding
MSTRG.162903.9	TCTN1	XLOC_162571	3392	coding	coding	coding	coding
MSTRG.162926.2	BRAP	XLOC_162608	3708	coding	coding	coding	noncoding
MSTRG.162926.4	BRAP	XLOC_162608	1506	coding	coding	noncoding	noncoding
MSTRG.162998.2	NAA25	XLOC_162632	5144	coding	coding	noncoding	noncoding
MSTRG.163017.3	HECTD4	XLOC_162639	12461	coding	noncoding	coding	coding
MSTRG.163017.5	HECTD4	XLOC_162639	9338	coding	noncoding	coding	coding

MSTRG.163017.11	HECTD4	XLOC_162639	3314	coding	noncoding	coding	noncoding
MSTRG.162959.2	RPL6	XLOC_162642	1500	coding	coding	coding	noncoding
MSTRG.162959.6	RPL6	XLOC_162642	705	coding	coding	noncoding	noncoding
NM_002534.3	OAS1	XLOC_162651	1627	coding	coding	coding	coding
MSTRG.163270.14	MED13L	XLOC_162741	2713	coding	noncoding	noncoding	noncoding
MSTRG.163280.2	MED13L	XLOC_162744	40080	coding	noncoding	noncoding	noncoding
NM_001085481.3	MAP1LC3B2	XLOC_162756	818	coding	coding	coding	coding
XR_001748653.1	FBXW8	XLOC_162765	6320	coding	coding	coding	coding
MSTRG.163354.1	FBXW8	XLOC_162777	231	coding	noncoding	noncoding	noncoding
MSTRG.163299.2	TESC	XLOC_162783	950	coding	noncoding	coding	noncoding
MSTRG.163453.14	TAOK3	XLOC_162813	2532	coding	coding	noncoding	noncoding
NR_033684.1	GATC	XLOC_162879	4346	coding	noncoding	coding	coding
XM_024448851.1	TMEM120B	XLOC_162938	1564	coding	coding	coding	coding
MSTRG.163725.1	CLIP1	XLOC_162958	5816	coding	coding	coding	coding
MSTRG.163734.8	HCAR2	XLOC_162967	2112	coding	coding	coding	coding
MSTRG.163734.12	HCAR2	XLOC_162967	3953	coding	coding	coding	coding
MSTRG.163803.8	MPHOSPH9	XLOC_162991	3824	coding	noncoding	noncoding	noncoding
MSTRG.163814.2	CDK2AP1	XLOC_162996	1287	coding	coding	noncoding	noncoding
MSTRG.163834.5	SNRNP35	XLOC_163006	1703	coding	noncoding	noncoding	noncoding
MSTRG.164334.3	GLT1D1	XLOC_163211	4349	coding	coding	coding	coding
MSTRG.164498.3	ANKLE2	XLOC_163356	4318	coding	coding	coding	noncoding
MSTRG.164498.4	ANKLE2	XLOC_163356	3148	coding	coding	coding	noncoding
MSTRG.164510.2	ZNF605	XLOC_163369	1158	coding	coding	noncoding	noncoding
MSTRG.153660.2	RAD52	XLOC_163445	13351	coding	coding	coding	coding
NM_152441.3	FBXL14	XLOC_163455	2527	coding	coding	coding	coding
NM_006422.3	AKAP3	XLOC_163583	3318	coding	coding	coding	coding
MSTRG.153848.6	TAPBPL	XLOC_163662	1413	coding	coding	coding	coding
NM_004054.4	C3AR1	XLOC_163767	3434	coding	coding	coding	coding
MSTRG.154090.1	LOC112268090	XLOC_163801	243	coding	coding	noncoding	noncoding
MSTRG.154383.7	A2M	XLOC_163854	33068	coding	coding	coding	noncoding
MSTRG.154383.16	A2M	XLOC_163854	61915	coding	coding	coding	noncoding
MSTRG.154383.27	A2M	XLOC_163854	22081	coding	coding	coding	coding
MSTRG.154196.1		XLOC_163857	297	coding	noncoding	noncoding	noncoding
MSTRG.154274.1		XLOC_163872	601	coding	noncoding	noncoding	noncoding
MSTRG.154275.1	OVOS	XLOC_163873	595	coding	coding	noncoding	noncoding
MSTRG.154278.1	OVOS	XLOC_163875	230	coding	noncoding	noncoding	noncoding
MSTRG.154279.1	OVOS	XLOC_163876	374	coding	coding	noncoding	noncoding
MSTRG.154281.1	OVOS	XLOC_163878	306	coding	noncoding	noncoding	noncoding
MSTRG.154291.1	KLRB1	XLOC_163885	1254	coding	coding	noncoding	coding
MSTRG.154297.15	CLEC2D	XLOC_163888	28852	coding	coding	coding	noncoding
MSTRG.154297.18	LOC374443	XLOC_163888	2094	coding	noncoding	noncoding	noncoding
MSTRG.154297.20		XLOC_163888	6194	coding	noncoding	coding	noncoding

MSTRG.154341.5	CLEC1B	XLOC_163911	3869	coding	coding	noncoding	noncoding
MSTRG.154341.4	CLEC1B	XLOC_163911	4208	coding	coding	noncoding	noncoding
MSTRG.154341.11	CLEC1B	XLOC_163911	5863	coding	coding	noncoding	noncoding
MSTRG.154341.12	CLEC1B	XLOC_163911	6112	coding	coding	noncoding	noncoding
MSTRG.154341.15	CLEC1B	XLOC_163911	12534	coding	coding	noncoding	noncoding
MSTRG.154341.17	CLEC1B	XLOC_163911	5571	coding	coding	noncoding	noncoding
MSTRG.154344.1	CLEC12A	XLOC_163912	1458	coding	coding	noncoding	noncoding
MSTRG.154354.1	LOC102724020	XLOC_163918	2072	coding	coding	noncoding	noncoding
NR_120484.1	LOC102724020	XLOC_163918	670	coding	noncoding	noncoding	noncoding
MSTRG.154247.11	CLEC7A	XLOC_163927	1633	coding	coding	noncoding	coding
MSTRG.154981.5	ATF7IP	XLOC_164119	10392	coding	coding	noncoding	coding
MSTRG.154981.7	ATF7IP	XLOC_164119	2586	coding	coding	noncoding	coding
MSTRG.154981.9	ATF7IP	XLOC_164119	8835	coding	noncoding	noncoding	noncoding
MSTRG.154981.11	ATF7IP	XLOC_164119	5254	coding	noncoding	noncoding	coding
MSTRG.155091.1	MGST1	XLOC_164221	869	coding	coding	noncoding	noncoding
MSTRG.155197.1		XLOC_164338	223	coding	noncoding	noncoding	noncoding
MSTRG.155246.1		XLOC_164386	202	coding	noncoding	noncoding	noncoding
MSTRG.155483.3	ETNK1	XLOC_164440	17982	coding	coding	coding	noncoding
MSTRG.155483.5	ETNK1	XLOC_164440	4020	coding	noncoding	coding	noncoding
MSTRG.155483.6	ETNK1	XLOC_164440	1760	coding	coding	coding	noncoding
NM_004985.5	KRAS	XLOC_164506	5306	coding	coding	coding	coding
MSTRG.155732.2	MED21	XLOC_164643	3960	coding	coding	noncoding	noncoding
MSTRG.155766.3	STK38L	XLOC_164650	3947	coding	coding	noncoding	noncoding
MSTRG.155833.2	MRPS35	XLOC_164685	1750	coding	coding	noncoding	noncoding
MSTRG.156140.7	LOC729291	XLOC_164695	1369	coding	noncoding	noncoding	coding
NM_001278412.1	AMN1	XLOC_164841	2319	coding	coding	noncoding	coding
MSTRG.156401.3	FGD4	XLOC_164887	3867	coding	coding	noncoding	noncoding
MSTRG.156460.3	DNM1L	XLOC_164891	2052	coding	coding	noncoding	noncoding
MSTRG.156455.1		XLOC_164908	223	coding	noncoding	noncoding	noncoding
MSTRG.156723.1	C12orf40	XLOC_165004	536	coding	noncoding	noncoding	noncoding
MSTRG.156898.13	LRRK2	XLOC_165040	4050	coding	coding	noncoding	noncoding
MSTRG.156898.14	LRRK2	XLOC_165040	10407	coding	coding	coding	coding
MSTRG.156866.9	PPHLN1	XLOC_165099	3203	coding	coding	noncoding	noncoding
MSTRG.156866.10	PPHLN1	XLOC_165099	851	coding	noncoding	noncoding	coding
MSTRG.157111.21	IRAK4	XLOC_165128	3752	coding	coding	coding	noncoding
MSTRG.157111.22	IRAK4	XLOC_165128	6026	coding	coding	coding	noncoding
MSTRG.156943.4	PLEKHA8P1	XLOC_165310	5950	coding	coding	coding	noncoding
NM_004719.3	SCAF11	XLOC_165329	7552	coding	noncoding	coding	coding
MSTRG.157678.1		XLOC_165428	333	coding	noncoding	noncoding	noncoding
MSTRG.157392.5	AMIGO2	XLOC_165506	5134	coding	coding	coding	coding
MSTRG.157392.13	PCED1B-AS1	XLOC_165506	2286	coding	noncoding	coding	coding
XM_011538481.1	HDAC7	XLOC_165550	4135	coding	coding	coding	coding

XM_017019239.2	SENP1	XLOC_165563	4935	coding	coding	coding	coding
MSTRG.157791.1	TUBA1A	XLOC_165666	3336	coding	coding	coding	coding
NM_001331069.2	CERS5	XLOC_165744	2801	coding	coding	coding	coding
MSTRG.157942.6	LARP4	XLOC_165769	1781	coding	coding	noncoding	noncoding
MSTRG.157942.9	LARP4	XLOC_165769	1157	coding	noncoding	noncoding	noncoding
XM_005268607.1	GALNT6	XLOC_165798	4958	coding	coding	coding	coding
MSTRG.158094.2	ATG101	XLOC_165868	397	coding	coding	noncoding	noncoding
MSTRG.158185.1	IGFBP6	XLOC_165937	478	coding	coding	noncoding	noncoding
MSTRG.158220.3	SP1	XLOC_165949	8299	coding	coding	coding	noncoding
MSTRG.158312.3	COPZ1	XLOC_166000	2647	coding	coding	coding	noncoding
MSTRG.158312.9	COPZ1	XLOC_166000	2415	coding	coding	coding	noncoding
MSTRG.158459.2	DNAJC14	XLOC_166081	695	coding	coding	noncoding	noncoding
MSTRG.158465.5	RAB5B	XLOC_166091	2336	coding	coding	noncoding	noncoding
MSTRG.158534.2	CNPY2	XLOC_166117	794	coding	coding	noncoding	noncoding
MSTRG.158556.1	SPRYD4	XLOC_166128	1719	coding	coding	noncoding	noncoding
NM_005594.6	NACA	XLOC_166145	1099	coding	coding	coding	coding
MSTRG.159436.32	USP15	XLOC_166306	5400	coding	coding	noncoding	noncoding
MSTRG.159470.1		XLOC_166307	299	coding	coding	noncoding	noncoding
MSTRG.158991.2	MON2	XLOC_166308	7316	coding	coding	coding	noncoding
MSTRG.159094.2	XPOT	XLOC_166373	1286	coding	coding	noncoding	noncoding
MSTRG.159098.3	TBK1	XLOC_166374	1688	coding	coding	noncoding	noncoding
MSTRG.159348.3	HELB	XLOC_166449	3428	coding	coding	coding	noncoding
MSTRG.159348.5	HELB	XLOC_166449	2950	coding	coding	coding	noncoding
MSTRG.159614.4	RAP1B	XLOC_166542	4941	coding	noncoding	coding	noncoding
XM_011537961.1	BEST3	XLOC_166601	3996	coding	coding	coding	coding
MSTRG.159855.10	LINC01481	XLOC_166621	2240	coding	coding	coding	noncoding
MSTRG.160134.2	KRR1	XLOC_166817	7972	coding	coding	coding	noncoding
MSTRG.161189.1	NUDT4	XLOC_167404	2473	coding	coding	coding	noncoding
MSTRG.161189.7	NUDT4	XLOC_167404	4261	coding	coding	coding	noncoding
MSTRG.161485.4	VEZT	XLOC_167488	2679	coding	coding	noncoding	noncoding
MSTRG.161485.5	VEZT	XLOC_167488	2819	coding	coding	noncoding	noncoding
MSTRG.161504.2	METAP2	XLOC_167500	3070	coding	coding	noncoding	noncoding
MSTRG.161845.4	ANKS1B	XLOC_167631	11917	coding	coding	coding	coding
MSTRG.162010.7	CHPT1	XLOC_167678	2304	coding	coding	coding	noncoding
MSTRG.162112.3	HSP90B1	XLOC_167768	2770	coding	coding	noncoding	noncoding
MSTRG.162403.4	CHST11	XLOC_167793	5786	coding	coding	coding	noncoding
MSTRG.162403.20	CHST11	XLOC_167793	6736	coding	noncoding	coding	noncoding
MSTRG.162403.22	CHST11	XLOC_167793	11031	coding	coding	coding	noncoding
MSTRG.162403.30	CHST11	XLOC_167793	7088	coding	coding	coding	noncoding
MSTRG.162403.31	CHST11	XLOC_167793	4415	coding	coding	coding	noncoding
MSTRG.162320.10	WASHC4	XLOC_167859	5225	coding	coding	noncoding	noncoding
MSTRG.162527.2	POLR3B	XLOC_167926	2459	coding	coding	noncoding	noncoding

MSTRG.163037.7	OAS1	XLOC_168219	11239	coding	coding	coding	noncoding
MSTRG.163037.6	OAS3	XLOC_168219	16528	coding	coding	coding	coding
MSTRG.163037.11	OAS3	XLOC_168219	8968	coding	coding	coding	coding
MSTRG.163037.10	OAS3	XLOC_168219	8921	coding	coding	coding	coding
MSTRG.163037.17	OAS2	XLOC_168219	6305	coding	coding	coding	noncoding
MSTRG.163342.3	LOC100506551	XLOC_168376	1169	coding	noncoding	noncoding	noncoding
MSTRG.163357.1	FBXW8	XLOC_168377	1571	coding	noncoding	noncoding	noncoding
XM_011537779.2	RAB35	XLOC_168520	2875	coding	coding	coding	coding
NM_001002.4	RPLP0	XLOC_168530	1105	coding	coding	coding	coding
XM_011538867.3	KDM2B	XLOC_168600	5647	coding	coding	coding	coding
MSTRG.163692.3	HPD	XLOC_168616	294	coding	noncoding	noncoding	noncoding
MSTRG.163895.21	ZNF664	XLOC_168714	3021	coding	coding	noncoding	noncoding
NM_145648.4	SLC15A4	XLOC_168871	2751	coding	coding	coding	coding
MSTRG.164337.7	GLT1D1	XLOC_168881	1919	coding	coding	coding	noncoding
MSTRG.164323.4	SFSWAP	XLOC_168982	2093	coding	coding	coding	noncoding
MSTRG.164575.1	ZNF10	XLOC_169101	253	coding	noncoding	noncoding	noncoding
XM_024449407.1	ZMYM2	XLOC_169138	3809	coding	coding	coding	coding
MSTRG.165326.6	GTF3A	XLOC_169442	1513	coding	coding	noncoding	noncoding
MSTRG.165578.3	HMGB1	XLOC_169612	4502	coding	coding	noncoding	coding
MSTRG.165578.5	HMGB1	XLOC_169612	4986	coding	coding	noncoding	noncoding
MSTRG.165588.1	HMGB1	XLOC_169620	284	coding	noncoding	noncoding	noncoding
MSTRG.166221.1		XLOC_169691	418	coding	noncoding	noncoding	noncoding
MSTRG.165798.3	N4BP2L1	XLOC_169812	2476	coding	coding	coding	noncoding
MSTRG.165800.3	N4BP2L2	XLOC_169813	4070	coding	coding	noncoding	noncoding
MSTRG.166055.3	SPART	XLOC_169972	2778	coding	coding	coding	noncoding
MSTRG.166177.8	EXOSC8	XLOC_169994	2180	coding	noncoding	coding	noncoding
MSTRG.166628.1		XLOC_170207	284	coding	noncoding	noncoding	noncoding
MSTRG.166629.1		XLOC_170208	348	coding	coding	noncoding	noncoding
MSTRG.166706.7	ELF1	XLOC_170232	4099	coding	coding	noncoding	noncoding
MSTRG.166706.9	ELF1	XLOC_170232	3683	coding	coding	noncoding	noncoding
MSTRG.166706.18	ELF1	XLOC_170232	2422	coding	noncoding	noncoding	noncoding
XR_941899.2	LOC105370173	XLOC_170251	1124	coding	noncoding	noncoding	noncoding
XM_017020381.2	AKAP11	XLOC_170283	9948	coding	noncoding	coding	coding
MSTRG.166883.8	EPSTI1	XLOC_170314	7754	coding	noncoding	noncoding	noncoding
MSTRG.166883.10	EPSTI1	XLOC_170314	1566	coding	noncoding	noncoding	noncoding
MSTRG.166883.15	EPSTI1	XLOC_170314	6103	coding	coding	noncoding	noncoding
XR_941910.2	LOC105370178	XLOC_170314	907	coding	noncoding	noncoding	noncoding
MSTRG.166917.1	CCDC122	XLOC_170341	214	coding	noncoding	noncoding	noncoding
MSTRG.167057.4	TSC22D1-AS1	XLOC_170368	3731	coding	noncoding	coding	coding
MSTRG.167143.2	SLC25A30-AS1	XLOC_170420	3700	coding	coding	noncoding	noncoding
MSTRG.167151.5	ZC3H13	XLOC_170442	2326	coding	coding	noncoding	noncoding
MSTRG.167183.1	LRRC63	XLOC_170451	2333	coding	coding	noncoding	coding

MSTRG.167192.1	LOC105370194	XLOC_170455	270	coding	noncoding	noncoding	noncoding
MSTRG.167310.2	SUCLA2	XLOC_170525	356	coding	coding	noncoding	noncoding
MSTRG.167350.1	ITM2B	XLOC_170546	10225	coding	coding	coding	coding
MSTRG.167493.8	RCBTB2	XLOC_170591	2089	coding	coding	noncoding	noncoding
XM_017020440.2	FNDC3A	XLOC_170616	5985	coding	coding	coding	coding
XM_017020443.1	FNDC3A	XLOC_170616	4311	coding	coding	coding	coding
MSTRG.167444.1	FNDC3A	XLOC_170642	310	coding	noncoding	noncoding	noncoding
MSTRG.167410.1	LOC105370203	XLOC_170655	270	coding	noncoding	noncoding	noncoding
NM_001007278.3	TRIM13	XLOC_170686	6919	coding	coding	coding	coding
MSTRG.167640.1	ATP7B	XLOC_170773	216	coding	noncoding	noncoding	noncoding
MSTRG.168730.7	DIS3	XLOC_171331	3673	coding	coding	coding	noncoding
MSTRG.169029.4	KLF12	XLOC_171380	5799	coding	coding	coding	noncoding
XM_017020725.1	UCHL3	XLOC_171458	1207	coding	coding	coding	coding
MSTRG.169454.22	MYCBP2	XLOC_171584	11393	coding	coding	coding	coding
MSTRG.169454.25	MYCBP2	XLOC_171584	968	coding	coding	noncoding	noncoding
MSTRG.170235.15	LOC102724149	XLOC_171993	2755	coding	coding	coding	coding
MSTRG.170311.1	UGGT2	XLOC_172028	313	coding	noncoding	noncoding	noncoding
XM_024449300.1	MBNL2	XLOC_172063	5798	coding	noncoding	coding	coding
MSTRG.170546.3	DOCK9	XLOC_172127	2856	coding	coding	coding	noncoding
MSTRG.170526.2	GPR183	XLOC_172154	3590	coding	coding	noncoding	noncoding
XM_017020612.1	PCCA	XLOC_172210	5689	coding	coding	coding	coding
MSTRG.170771.1	POGLUT2	XLOC_172281	208	coding	noncoding	noncoding	noncoding
MSTRG.171009.1		XLOC_172423	337	coding	noncoding	noncoding	noncoding
MSTRG.171209.3	ANKRD10	XLOC_172552	3315	coding	coding	coding	coding
NM_001363660.2	PSPC1	XLOC_172737	4418	coding	coding	coding	coding
MSTRG.164786.10	ZMYM2	XLOC_172745	4079	coding	coding	noncoding	noncoding
MSTRG.164786.12	ZMYM2	XLOC_172745	1182	coding	coding	noncoding	noncoding
MSTRG.164655.1	IFT88	XLOC_172769	206	coding	noncoding	noncoding	noncoding
XM_017020706.2	XPO4	XLOC_172785	8448	coding	coding	coding	coding
MSTRG.164699.2	SAP18	XLOC_172805	1438	coding	coding	noncoding	noncoding
XM_017020892.2	LOC101928764	XLOC_172809	2351	coding	noncoding	coding	coding
MSTRG.164952.5	SPATA13	XLOC_173026	2353	coding	coding	coding	noncoding
MSTRG.164952.4	SPATA13	XLOC_173026	8156	coding	coding	coding	noncoding
MSTRG.164952.6	SPATA13	XLOC_173026	2762	coding	coding	coding	noncoding
MSTRG.164952.11	SPATA13	XLOC_173026	6153	coding	coding	coding	coding
MSTRG.165303.3	RPL21	XLOC_173129	685	coding	coding	noncoding	noncoding
MSTRG.165354.5	LNX2	XLOC_173148	2660	coding	noncoding	coding	coding
MSTRG.165354.12	LNX2	XLOC_173148	4714	coding	noncoding	coding	noncoding
MSTRG.165414.14	PAN3-AS1	XLOC_173164	8131	coding	coding	coding	noncoding
MSTRG.165406.1		XLOC_173182	323	coding	coding	noncoding	noncoding
MSTRG.165821.3	PDS5B	XLOC_173398	5271	coding	noncoding	noncoding	noncoding
MSTRG.165821.2	PDS5B	XLOC_173398	8106	coding	noncoding	noncoding	noncoding

MSTRG.165821.5	PDS5B	XLOC_173398	950	coding	noncoding	noncoding	noncoding
MSTRG.165777.1	KL	XLOC_173414	523	coding	coding	noncoding	noncoding
MSTRG.166107.1	NBEA	XLOC_173515	256	coding	noncoding	noncoding	noncoding
MSTRG.166120.1	NBEA	XLOC_173523	264	coding	noncoding	noncoding	noncoding
NM_001370331.1	ELF1	XLOC_173773	3631	coding	coding	coding	coding
MSTRG.166725.3	WBP4	XLOC_173789	569	coding	coding	noncoding	noncoding
MSTRG.166769.5	NAA16	XLOC_173803	3653	coding	coding	noncoding	noncoding
XM_005266374.2	LCP1	XLOC_174038	3675	coding	coding	coding	coding
MSTRG.167287.2	LRCH1	XLOC_174072	2866	coding	coding	coding	noncoding
NM_014166.4	MED4	XLOC_174114	2254	coding	coding	coding	coding
MSTRG.167357.2	ITM2B	XLOC_174122	16066	coding	coding	coding	noncoding
MSTRG.167457.9	RB1	XLOC_174125	3373	coding	coding	noncoding	coding
MSTRG.167395.1	CYSLTR2	XLOC_174140	4787	coding	coding	noncoding	noncoding
MSTRG.167417.2	FNDC3A	XLOC_174157	4861	coding	coding	coding	coding
MSTRG.168862.1	DLEU1	XLOC_174270	511	coding	noncoding	noncoding	noncoding
MSTRG.167849.2	WDFY2	XLOC_174413	4174	coding	coding	coding	noncoding
MSTRG.167681.1	OLFM4	XLOC_174463	2317	coding	coding	noncoding	noncoding
MSTRG.168746.3	PIBF1	XLOC_174873	1144	coding	noncoding	noncoding	noncoding
MSTRG.169141.2	UCHL3	XLOC_175001	564	coding	coding	noncoding	noncoding
MSTRG.169128.1		XLOC_175053	260	coding	noncoding	noncoding	noncoding
XM_017020538.2	FBXL3	XLOC_175057	1337	coding	coding	coding	coding
XM_005266337.3	FBXL3	XLOC_175057	3162	coding	noncoding	coding	coding
MSTRG.169905.1	LINC00373	XLOC_175463	278	coding	noncoding	noncoding	noncoding
MSTRG.170889.2	PCCA	XLOC_175818	1399	coding	coding	noncoding	noncoding
MSTRG.171466.6	ATP11A	XLOC_176242	4224	coding	coding	coding	noncoding
MSTRG.171486.12	TMCO3	XLOC_176281	2295	coding	coding	coding	noncoding
MSTRG.171496.9	TFDP1	XLOC_176284	2320	coding	coding	coding	noncoding
NM_001145.4	ANG	XLOC_176422	1222	coding	coding	coding	coding
MSTRG.171915.13	HAUS4	XLOC_176525	2908	coding	coding	coding	noncoding
MSTRG.171941.11	LOC107984665	XLOC_176535	3531	coding	coding	coding	coding
MSTRG.171941.10	LOC107984665	XLOC_176535	3684	coding	coding	coding	coding
NR_104265.1	GMPR2	XLOC_176600	1996	coding	coding	coding	coding
MSTRG.172088.12	KHNYN	XLOC_176611	1558	coding	coding	noncoding	noncoding
MSTRG.172542.4	HECTD1	XLOC_176770	9105	coding	coding	coding	coding
MSTRG.172464.2	HEATR5A	XLOC_176772	7429	coding	noncoding	coding	coding
MSTRG.172479.1	LOC101927124	XLOC_176777	521	coding	noncoding	noncoding	noncoding
MSTRG.172773.1	LOC102724945	XLOC_176871	384	coding	noncoding	noncoding	noncoding
MSTRG.173056.3	RALGAPA1	XLOC_176918	3775	coding	noncoding	coding	noncoding
MSTRG.173056.5	RALGAPA1	XLOC_176918	3375	coding	noncoding	coding	noncoding
NM_003616.2	GEMIN2	XLOC_177056	1359	coding	coding	noncoding	coding
MSTRG.173123.4	TRAPPC6B	XLOC_177063	463	coding	noncoding	noncoding	noncoding
MSTRG.173485.2	KLHL28	XLOC_177191	5997	coding	coding	coding	noncoding

MSTRG.173462.3	MIS18BP1	XLOC_177203	2624	coding	coding	noncoding	noncoding
MSTRG.173614.1	RN7SL1	XLOC_177292	279	coding	noncoding	coding	noncoding
MSTRG.173678.1	NEMF	XLOC_177306	321	coding	noncoding	coding	noncoding
MSTRG.173678.27	LOC105378179	XLOC_177306	282	coding	noncoding	noncoding	noncoding
MSTRG.173678.29	LOC105378179	XLOC_177306	1015	coding	noncoding	noncoding	noncoding
MSTRG.173721.2	SAV1	XLOC_177334	2509	coding	coding	noncoding	noncoding
MSTRG.173789.3	LOC105370489	XLOC_177337	16919	coding	noncoding	coding	coding
MSTRG.173789.1	LOC105370489	XLOC_177337	12500	coding	noncoding	coding	noncoding
MSTRG.173789.10	NIN	XLOC_177337	11516	coding	noncoding	noncoding	coding
MSTRG.173789.18	NIN	XLOC_177337	8570	coding	noncoding	coding	noncoding
MSTRG.173789.24	NIN	XLOC_177337	3823	coding	noncoding	noncoding	noncoding
MSTRG.174172.4	DDHD1	XLOC_177474	3494	coding	coding	coding	coding
MSTRG.174172.5	DDHD1	XLOC_177474	3078	coding	coding	coding	coding
MSTRG.174105.4	GCH1	XLOC_177538	2409	coding	coding	coding	noncoding
MSTRG.174620.1	PSMA3	XLOC_177741	11824	coding	coding	coding	coding
MSTRG.174620.12	PSMA3-AS1	XLOC_177741	2124	coding	noncoding	noncoding	noncoding
XM_005267964.2	ARID4A	XLOC_177743	5951	coding	coding	coding	coding
XM_017021458.1	PRKCH	XLOC_177889	3194	coding	coding	coding	coding
NM_001243084.1	HIF1A	XLOC_177965	3956	coding	coding	coding	coding
XM_011536628.3	PLEKHG3	XLOC_178053	7931	coding	coding	coding	coding
MSTRG.175127.4	SPTB	XLOC_178056	4251	coding	coding	coding	coding
MSTRG.175217.1		XLOC_178156	280	coding	noncoding	noncoding	noncoding
MSTRG.175721.1		XLOC_178213	253	coding	coding	noncoding	noncoding
XM_011536503.2	FAM71D	XLOC_178268	2724	coding	coding	coding	coding
MSTRG.175794.12	ATP6V1D	XLOC_178271	1768	coding	coding	noncoding	noncoding
MSTRG.175358.2	ZFP36L1	XLOC_178365	2297	coding	coding	coding	coding
XM_017021096.1	PCNX1	XLOC_178519	12562	coding	coding	coding	coding
XM_005267425.4	PCNX1	XLOC_178519	12586	coding	coding	coding	coding
XM_005267422.4	PCNX1	XLOC_178519	12889	coding	coding	coding	coding
NM_001308160.2	PCNX1	XLOC_178519	12532	coding	coding	coding	coding
MSTRG.175700.1	PCNX1	XLOC_178562	296	coding	noncoding	noncoding	noncoding
MSTRG.176027.7	PAPLN	XLOC_178703	9615	coding	coding	coding	coding
NM_025057.3	BBOF1	XLOC_178733	3113	coding	coding	coding	coding
MSTRG.176265.3	NEK9	XLOC_178798	1343	coding	coding	coding	noncoding
MSTRG.176480.5	TMED8	XLOC_178905	4034	coding	coding	coding	noncoding
MSTRG.176495.4	VIPAS39	XLOC_178911	2415	coding	coding	coding	noncoding
MSTRG.176504.2	SLIRP	XLOC_178917	2526	coding	coding	noncoding	coding
MSTRG.176998.1	CEP128	XLOC_178992	441	coding	noncoding	noncoding	noncoding
MSTRG.177055.1	CEP128	XLOC_179039	374	coding	noncoding	noncoding	noncoding
MSTRG.177072.1	CEP128	XLOC_179055	514	coding	noncoding	noncoding	noncoding
MSTRG.176896.1		XLOC_179261	985	coding	noncoding	noncoding	noncoding
NM_024824.5	ZC3H14	XLOC_179288	18153	coding	coding	coding	coding

MSTRG.177185.1	EML5	XLOC_179292	624	coding	coding	noncoding	noncoding
MSTRG.177199.1	EML5	XLOC_179306	308	coding	noncoding	noncoding	noncoding
NM_001102366.2	DGLUCY	XLOC_179405	3182	coding	coding	coding	coding
MSTRG.177439.8	TC2N	XLOC_179435	3724	coding	noncoding	noncoding	noncoding
MSTRG.177439.10	TC2N	XLOC_179435	1466	coding	noncoding	noncoding	noncoding
MSTRG.177447.3	TRIP11	XLOC_179438	6489	coding	noncoding	noncoding	coding
XM_011536440.2	SLC24A4	XLOC_179455	9688	coding	coding	coding	coding
MSTRG.177559.1	ASB2	XLOC_179518	265	coding	coding	noncoding	noncoding
MSTRG.177768.1	DICER1-AS1	XLOC_179560	12628	coding	coding	coding	noncoding
MSTRG.177830.5	ATG2B	XLOC_179608	1763	coding	noncoding	noncoding	noncoding
NM_001271905.1	GSKIP	XLOC_179609	2212	coding	coding	coding	coding
MSTRG.178576.16	CINP	XLOC_180089	1639	coding	noncoding	coding	noncoding
NM_145725.2	TRAF3	XLOC_180115	7776	coding	coding	coding	coding
MSTRG.178784.5	LOC102723342	XLOC_180218	1776	coding	coding	coding	coding
MSTRG.178995.4	IGHA2	XLOC_180279	1159	coding	coding	coding	noncoding
MSTRG.178995.6	IGHA2	XLOC_180279	1629	coding	coding	coding	noncoding
MSTRG.171811.2	ZNF219	XLOC_180445	5183	coding	coding	coding	coding
XM_011536710.2	HNRNPC	XLOC_180453	3199	coding	coding	coding	coding
XM_024449556.1	HNRNPC	XLOC_180453	3692	coding	coding	coding	coding
XM_024449558.1	HNRNPC	XLOC_180453	2978	coding	coding	coding	coding
XM_006720125.3	HNRNPC	XLOC_180453	2485	coding	coding	coding	coding
MSTRG.171824.3	RPGRIP1	XLOC_180466	321	coding	noncoding	noncoding	noncoding
MSTRG.172221.1	TRAV1-1	XLOC_180514	635	coding	coding	noncoding	noncoding
MSTRG.172250.22	TRAV1-1	XLOC_180531	2538	coding	coding	noncoding	noncoding
MSTRG.172250.43	TRAV1-1	XLOC_180531	10437	coding	coding	coding	noncoding
MSTRG.171864.2	ABHD4	XLOC_180559	3168	coding	coding	noncoding	noncoding
XM_011537245.3	ZFHX2	XLOC_180644	9463	coding	coding	noncoding	coding
NR_023921.2	DHRS4-AS1	XLOC_180654	3052	coding	noncoding	coding	noncoding
NR_023922.2	DHRS4-AS1	XLOC_180654	2793	coding	noncoding	noncoding	noncoding
NM_006405.7	TM9SF1	XLOC_180677	2200	coding	coding	coding	coding
XM_011536659.3	CIDEB	XLOC_180689	2043	coding	coding	coding	coding
MSTRG.172091.2	SDR39U1	XLOC_180696	6609	coding	coding	coding	coding
MSTRG.172435.6	G2E3	XLOC_180816	1514	coding	coding	noncoding	noncoding
MSTRG.172598.1	NUBPL	XLOC_180897	262	coding	coding	coding	noncoding
MSTRG.172599.1	NUBPL	XLOC_180898	386	coding	noncoding	noncoding	noncoding
MSTRG.172636.1	NUBPL	XLOC_180933	321	coding	noncoding	noncoding	noncoding
MSTRG.172558.3	ARHGAP5-AS1	XLOC_180959	7646	coding	coding	coding	noncoding
MSTRG.172859.6	FAM177A1	XLOC_181060	1804	coding	coding	coding	noncoding
MSTRG.173010.1		XLOC_181179	283	coding	noncoding	noncoding	noncoding
MSTRG.173127.2	PNN	XLOC_181202	5051	coding	coding	coding	noncoding
MSTRG.173127.3	PNN	XLOC_181202	2408	coding	coding	coding	coding
MSTRG.173318.1	LOC100288846	XLOC_181205	2285	coding	noncoding	noncoding	noncoding

MSTRG.173318.3	LOC100288846	XLOC_181205	2609	coding	noncoding	noncoding	noncoding
MSTRG.173318.6	MIA2	XLOC_181205	4656	coding	noncoding	noncoding	coding
MSTRG.173329.1	MIA2	XLOC_181206	282	coding	noncoding	noncoding	noncoding
MSTRG.173504.6	PRPF39	XLOC_181382	2766	coding	noncoding	noncoding	noncoding
MSTRG.173612.1	LRR1	XLOC_181466	211	coding	noncoding	noncoding	noncoding
MSTRG.173612.2	LOC107987206	XLOC_181466	286	coding	noncoding	noncoding	noncoding
MSTRG.173622.3	MGAT2	XLOC_181469	1890	coding	coding	coding	coding
XM_024449554.1	ERO1A	XLOC_181667	5414	coding	coding	coding	coding
MSTRG.173971.3	PSMC6	XLOC_181674	428	coding	coding	noncoding	noncoding
MSTRG.173993.2	GNPNAT1	XLOC_181676	8222	coding	coding	noncoding	noncoding
MSTRG.174051.2	CGRRF1	XLOC_181834	335	coding	noncoding	noncoding	noncoding
MSTRG.174153.6	FBXO34	XLOC_181894	3299	coding	noncoding	noncoding	noncoding
MSTRG.174327.2	KTN1	XLOC_181900	4565	coding	noncoding	noncoding	coding
MSTRG.174327.4	KTN1	XLOC_181900	4645	coding	noncoding	noncoding	coding
MSTRG.174327.7	KTN1	XLOC_181900	4426	coding	noncoding	noncoding	coding
MSTRG.174327.11	KTN1	XLOC_181900	2908	coding	coding	noncoding	coding
MSTRG.174511.4	PELI2	XLOC_181924	10524	coding	coding	coding	coding
MSTRG.174369.1		XLOC_181957	296	coding	coding	noncoding	noncoding
MSTRG.174433.1		XLOC_181986	470	coding	noncoding	noncoding	noncoding
MSTRG.174666.2	KIAA0586	XLOC_182033	2409	coding	coding	noncoding	noncoding
MSTRG.174866.1	SLC38A6	XLOC_182136	290	coding	noncoding	noncoding	noncoding
MSTRG.174882.2	SNAPC1	XLOC_182174	1144	coding	coding	noncoding	noncoding
MSTRG.175271.6	SYNE2	XLOC_182235	21734	coding	coding	coding	coding
MSTRG.175271.11	SYNE2	XLOC_182235	7409	coding	coding	noncoding	noncoding
MSTRG.175271.12	SYNE2	XLOC_182235	21780	coding	coding	coding	coding
MSTRG.175110.26	ZBTB25	XLOC_182242	2746	coding	coding	coding	noncoding
MSTRG.175155.6	CHURC1	XLOC_182255	1978	coding	coding	noncoding	noncoding
MSTRG.175155.9	CHURC1	XLOC_182255	482	coding	coding	noncoding	noncoding
MSTRG.175952.2	RAD51B	XLOC_182347	1188	coding	coding	noncoding	noncoding
MSTRG.175447.3	SLC39A9	XLOC_182415	5532	coding	coding	coding	noncoding
MSTRG.175461.2	SUSD6	XLOC_182423	5902	coding	coding	coding	noncoding
MSTRG.175461.4	SUSD6	XLOC_182423	6724	coding	noncoding	noncoding	noncoding
MSTRG.175461.7	SUSD6	XLOC_182423	6429	coding	noncoding	noncoding	noncoding
MSTRG.175461.11	SUSD6	XLOC_182423	5043	coding	noncoding	noncoding	noncoding
MSTRG.175653.9	PCNX1	XLOC_182493	11880	coding	coding	coding	noncoding
MSTRG.175653.18	PCNX1	XLOC_182493	7888	coding	coding	coding	coding
MSTRG.176045.29	LOC102724015	XLOC_182505	9031	coding	coding	coding	noncoding
MSTRG.176045.28	LOC102724015	XLOC_182505	9028	coding	coding	coding	noncoding
MSTRG.176045.32	LOC102724015	XLOC_182505	7905	coding	coding	coding	noncoding
MSTRG.176045.35	LOC102724015	XLOC_182505	5733	coding	coding	coding	noncoding
MSTRG.175918.3	PSEN1	XLOC_182540	6771	coding	coding	coding	noncoding
MSTRG.175918.11	PSEN1	XLOC_182540	5922	coding	coding	coding	noncoding

MSTRG.175918.12	PSEN1	XLOC_182540	6051	coding	coding	coding	noncoding
MSTRG.175942.1	ACOT1	XLOC_182557	245	coding	noncoding	noncoding	noncoding
MSTRG.176154.9	ALDH6A1	XLOC_182582	1830	coding	coding	noncoding	noncoding
NM_002632.5	PGF	XLOC_182626	1911	coding	coding	coding	coding
MSTRG.177176.2	TTC8	XLOC_183076	279	coding	noncoding	noncoding	noncoding
MSTRG.177293.2	NRDE2	XLOC_183158	1670	coding	coding	noncoding	noncoding
MSTRG.177505.6	DGLUCY	XLOC_183212	5322	coding	coding	noncoding	coding
MSTRG.177572.9	IFI27	XLOC_183306	695	coding	coding	coding	coding
NM_001127703.2	SERPINA1	XLOC_183313	3438	coding	coding	coding	coding
NM_001080451.2	SERPINA11	XLOC_183321	1486	coding	coding	coding	coding
MSTRG.177885.5	VRK1	XLOC_183458	1112	coding	coding	noncoding	noncoding
MSTRG.177885.7	VRK1	XLOC_183458	2036	coding	coding	noncoding	noncoding
MSTRG.177885.8	VRK1	XLOC_183458	1242	coding	coding	noncoding	noncoding
NM_004184.4	WARS	XLOC_183682	2639	coding	coding	coding	coding
MSTRG.178580.6	TECPR2	XLOC_183767	3526	coding	coding	coding	coding
MSTRG.178580.9	ANKRD9	XLOC_183767	7242	coding	noncoding	coding	coding
MSTRG.178673.4	MARK3	XLOC_183805	3613	coding	coding	coding	noncoding
MSTRG.178730.2	TDRD9	XLOC_183838	1057	coding	coding	noncoding	noncoding
NM_005163.2	AKT1	XLOC_183872	2989	coding	coding	coding	coding
MSTRG.178912.1	HERC2P3	XLOC_183956	741	coding	coding	noncoding	noncoding
MSTRG.178916.4	HERC2P3	XLOC_183960	2863	coding	coding	noncoding	noncoding
MSTRG.179388.35	UBE3A	XLOC_184024	5419	coding	coding	noncoding	noncoding
MSTRG.179291.24	FAN1	XLOC_184229	4270	coding	coding	noncoding	noncoding
MSTRG.179291.25	FAN1	XLOC_184229	4236	coding	coding	coding	noncoding
MSTRG.179600.4	EMC4	XLOC_184337	8598	coding	coding	coding	coding
MSTRG.179600.16	SLC12A6	XLOC_184337	6934	coding	coding	coding	noncoding
MSTRG.179600.15	SLC12A6	XLOC_184337	7565	coding	coding	coding	noncoding
MSTRG.179608.4	GOLGA8A	XLOC_184342	2691	coding	noncoding	noncoding	coding
MSTRG.179608.12	GOLGA8B	XLOC_184342	1527	coding	noncoding	coding	noncoding
MSTRG.179756.1	DPH6	XLOC_184392	262	coding	coding	noncoding	noncoding
MSTRG.180106.2	BMF	XLOC_184593	4773	coding	coding	coding	noncoding
MSTRG.180106.4	BMF	XLOC_184593	3099	coding	coding	noncoding	noncoding
MSTRG.180169.5	PLCB2	XLOC_184603	4496	coding	coding	coding	coding
MSTRG.180440.2	LOC105370795	XLOC_184717	7352	coding	coding	coding	noncoding
MSTRG.180440.4	VPS39	XLOC_184717	4927	coding	coding	coding	noncoding
MSTRG.180440.12	LOC105370795	XLOC_184717	2839	coding	coding	noncoding	noncoding
NM_130798.2	SNAP23	XLOC_184732	2482	coding	noncoding	coding	coding
MSTRG.180480.9	SNAP23	XLOC_184732	1022	coding	coding	noncoding	noncoding
MSTRG.180420.2	UBR1	XLOC_184752	7733	coding	coding	noncoding	noncoding
MSTRG.180432.52	CCNDBP1	XLOC_184754	3445	coding	coding	coding	coding
MSTRG.180550.1		XLOC_184785	596	coding	noncoding	noncoding	noncoding
NM_205850.3	SLC24A5	XLOC_184965	1879	coding	coding	coding	coding

MSTRG.180982.18	SLC12A1	XLOC_184977	2232	coding	coding	noncoding	noncoding
MSTRG.180920.2	SECISBP2L	XLOC_185023	8379	coding	coding	noncoding	coding
MSTRG.180920.3	SECISBP2L	XLOC_185023	5403	coding	coding	noncoding	coding
MSTRG.181060.1		XLOC_185032	231	coding	noncoding	noncoding	noncoding
MSTRG.180943.1		XLOC_185080	215	coding	coding	noncoding	noncoding
MSTRG.181033.7	ATP8B4	XLOC_185089	3652	coding	coding	coding	noncoding
NM_001165257.1	SCG3	XLOC_185157	3257	coding	coding	coding	coding
MSTRG.181186.4	LYSMD2	XLOC_185157	1623	coding	coding	coding	noncoding
MSTRG.181456.2	MYO5A	XLOC_185198	9147	coding	coding	coding	coding
MSTRG.181502.15	CCPG1	XLOC_185269	1770	coding	noncoding	coding	noncoding
MSTRG.181511.1	DNAAF4-CCPG1	XLOC_185274	277	coding	noncoding	noncoding	noncoding
MSTRG.181513.1	DNAAF4-CCPG1	XLOC_185276	274	coding	coding	noncoding	noncoding
MSTRG.181728.1	ADAM10	XLOC_185473	13262	coding	coding	noncoding	noncoding
MSTRG.181728.4	ADAM10	XLOC_185473	10409	coding	coding	noncoding	noncoding
MSTRG.181728.13	ADAM10	XLOC_185473	2096	coding	coding	noncoding	noncoding
MSTRG.181767.3	SLTM	XLOC_185484	3787	coding	coding	coding	noncoding
MSTRG.181767.8	SLTM	XLOC_185484	3235	coding	coding	coding	noncoding
MSTRG.181767.9	SLTM	XLOC_185484	3091	coding	noncoding	noncoding	noncoding
MSTRG.182184.8	RORA-AS1	XLOC_185541	9194	coding	coding	noncoding	noncoding
MSTRG.182123.5	VPS13C	XLOC_185593	7875	coding	noncoding	noncoding	noncoding
XM_011522105.3	APH1B	XLOC_185635	1148	coding	coding	coding	coding
NR_046342.1	USP3	XLOC_185650	5722	coding	coding	coding	coding
NR_046341.1	USP3	XLOC_185650	5759	coding	coding	coding	coding
NM_006537.4	USP3	XLOC_185650	5517	coding	coding	coding	coding
MSTRG.182336.3	HERC1	XLOC_185658	2222	coding	coding	coding	coding
MSTRG.182336.6	HERC1	XLOC_185658	12142	coding	coding	coding	coding
MSTRG.182336.11	HERC1	XLOC_185658	9566	coding	coding	coding	coding
MSTRG.182336.15	HERC1	XLOC_185658	6804	coding	coding	coding	coding
XM_005254677.3	SNX22	XLOC_185669	832	coding	noncoding	coding	coding
MSTRG.182439.3	CSNK1G1	XLOC_185670	7487	coding	coding	noncoding	noncoding
MSTRG.182493.3	LOC112268147	XLOC_185736	1502	coding	coding	coding	coding
MSTRG.182504.4	DPP8	XLOC_185749	7290	coding	coding	coding	noncoding
MSTRG.182504.5	DPP8	XLOC_185749	3796	coding	coding	coding	noncoding
MSTRG.182697.2	AAGAB	XLOC_185825	1876	coding	coding	noncoding	noncoding
MSTRG.182795.11	CALML4	XLOC_185874	1215	coding	noncoding	coding	noncoding
MSTRG.182981.4	TLE3	XLOC_185950	14304	coding	coding	coding	coding
MSTRG.183109.4	MYO9A	XLOC_186011	2153	coding	coding	noncoding	noncoding
MSTRG.183060.4	PARP6	XLOC_186021	1235	coding	noncoding	noncoding	noncoding
MSTRG.183060.7	PARP6	XLOC_186021	3311	coding	coding	noncoding	noncoding
MSTRG.183069.1	HEXA-AS1	XLOC_186027	2045	coding	coding	coding	noncoding
MSTRG.183262.1	REC114	XLOC_186087	678	coding	noncoding	noncoding	noncoding
MSTRG.183526.2	SIN3A	XLOC_186199	4929	coding	coding	noncoding	noncoding

MSTRG.183729.4	PEAK1	XLOC_186248	3320	coding	noncoding	coding	coding
MSTRG.183729.6	PEAK1	XLOC_186248	2525	coding	noncoding	coding	noncoding
NM_001330376.1	TMED3	XLOC_186345	3092	coding	coding	coding	coding
MSTRG.183984.5	MTHFS	XLOC_186396	1152	coding	coding	noncoding	noncoding
NM_004513.6	IL16	XLOC_186440	7315	coding	coding	coding	coding
MSTRG.184143.2	EFL1	XLOC_186489	2704	coding	coding	coding	noncoding
NM_001321976.2	MRPS11	XLOC_186758	1700	coding	noncoding	coding	noncoding
NM_152924.5	ABHD2	XLOC_186784	8424	coding	coding	coding	coding
MSTRG.184720.8	AP3S2	XLOC_186824	694	coding	coding	noncoding	noncoding
NM_001130926.2	MEF2A	XLOC_187357	5530	coding	coding	coding	coding
MSTRG.185687.9	LINS1	XLOC_187463	2389	coding	coding	noncoding	noncoding
XM_011522015.3	LRRK1	XLOC_187480	9190	coding	coding	coding	coding
MSTRG.178955.1	HERC2P2	XLOC_187586	385	coding	noncoding	noncoding	noncoding
MSTRG.179272.3	LOC105376707	XLOC_187795	5686	coding	coding	coding	coding
MSTRG.179277.1	KLF13	XLOC_187798	271	coding	noncoding	noncoding	noncoding
MSTRG.179932.1		XLOC_188088	612	coding	noncoding	coding	noncoding
MSTRG.179992.1		XLOC_188114	601	coding	coding	noncoding	noncoding
MSTRG.180243.5	SPINT1-AS1	XLOC_188234	2203	coding	coding	coding	coding
MSTRG.180319.2	RTF1	XLOC_188262	4462	coding	coding	coding	noncoding
MSTRG.180554.2	PDIA3	XLOC_188395	1869	coding	coding	coding	noncoding
MSTRG.180591.3	CTDSPL2	XLOC_188422	2105	coding	noncoding	noncoding	noncoding
MSTRG.180698.2	SPATA5L1	XLOC_188462	2349	coding	coding	coding	noncoding
MSTRG.180769.9	BLOC1S6	XLOC_188472	1453	coding	coding	noncoding	noncoding
MSTRG.181009.2	DUT	XLOC_188549	1037	coding	coding	coding	noncoding
MSTRG.181018.12	USP50	XLOC_188644	3728	coding	coding	noncoding	noncoding
NM_001306195.1	ARPP19	XLOC_188757	5344	coding	coding	noncoding	noncoding
MSTRG.181753.2	MINDY2	XLOC_189067	1671	coding	coding	coding	noncoding
MSTRG.182100.2	LACTB	XLOC_189277	1881	coding	coding	coding	noncoding
MSTRG.182796.3	PIAS1	XLOC_189531	7253	coding	coding	coding	noncoding
MSTRG.182796.7	PIAS1	XLOC_189531	8093	coding	coding	coding	noncoding
MSTRG.182737.1		XLOC_189558	374	coding	noncoding	noncoding	noncoding
MSTRG.183226.1	NEO1	XLOC_189827	256	coding	noncoding	noncoding	noncoding
NM_017455.4	NPTN	XLOC_189844	2072	coding	coding	coding	coding
NM_001286742.1	UBL7	XLOC_189889	1548	coding	coding	coding	coding
MSTRG.183362.3	ARID3B	XLOC_189891	4671	coding	coding	coding	coding
NM_001145357.2	SIN3A	XLOC_189974	6636	coding	coding	coding	coding
NM_004390.4	CTSH	XLOC_190155	1505	coding	coding	coding	coding
MSTRG.183855.1	TMED3	XLOC_190198	1075	coding	noncoding	noncoding	noncoding
MSTRG.183932.6	ZFAND6	XLOC_190258	6523	coding	coding	noncoding	noncoding
MSTRG.183932.7	ZFAND6	XLOC_190258	5590	coding	coding	noncoding	noncoding
MSTRG.183932.9	ZFAND6	XLOC_190258	6486	coding	coding	noncoding	noncoding
MSTRG.184100.4	STARD5	XLOC_190328	9761	coding	coding	coding	noncoding

MSTRG.184355.4	ZNF592	XLOC_190465	9000	coding	coding	coding	coding
MSTRG.184546.4	AKAP13	XLOC_190487	4628	coding	coding	coding	coding
XM_011521637.3	NTRK3	XLOC_190544	4458	coding	coding	coding	coding
MSTRG.184592.3	MRPS11	XLOC_190566	788	coding	coding	coding	coding
MSTRG.184802.5	FES	XLOC_190692	2807	coding	coding	coding	coding
MSTRG.184840.2	UNC45A	XLOC_190697	3161	coding	coding	coding	coding
MSTRG.184840.4	UNC45A	XLOC_190697	1204	coding	noncoding	coding	noncoding
MSTRG.185043.1	SLCO3A1	XLOC_190735	14928	coding	noncoding	noncoding	noncoding
MSTRG.185132.19	CHD2	XLOC_190763	6519	coding	coding	noncoding	coding
MSTRG.185341.7	MCTP2	XLOC_190802	8556	coding	coding	coding	noncoding
MSTRG.185341.13	MCTP2	XLOC_190802	8853	coding	coding	noncoding	noncoding
MSTRG.185520.5	IRAIN	XLOC_190913	1185	coding	coding	coding	coding
MSTRG.185440.3	LRRC28	XLOC_190939	1305	coding	coding	noncoding	noncoding
MSTRG.185456.11	MEF2A	XLOC_190949	1405	coding	noncoding	noncoding	noncoding
NM_152334.3	TARSL2	XLOC_191059	3481	coding	coding	coding	coding
NM_001321551.1	LOC100128108	XLOC_191068	522	coding	noncoding	noncoding	coding
MSTRG.185805.7	NPRL3	XLOC_191096	2560	coding	coding	coding	coding
MSTRG.185816.2	HBA1	XLOC_191103	1060	coding	coding	coding	coding
MSTRG.185816.7	HBA1	XLOC_191103	990	coding	coding	coding	coding
MSTRG.185816.11	HBA1	XLOC_191103	873	coding	coding	coding	coding
MSTRG.185811.6	LUC7L	XLOC_191106	1378	coding	coding	coding	noncoding
NM_005861.4	STUB1	XLOC_191142	1340	coding	coding	coding	coding
MSTRG.185963.1	PRR25	XLOC_191159	476	coding	noncoding	noncoding	noncoding
MSTRG.186032.2	C16orf91	XLOC_191200	898	coding	coding	coding	noncoding
MSTRG.186136.2	RPS2	XLOC_191250	911	coding	coding	noncoding	noncoding
XM_011522450.2	RAB26	XLOC_191278	2441	coding	coding	coding	coding
MSTRG.186330.1	MEFV	XLOC_191367	3293	coding	coding	coding	coding
MSTRG.186760.9	USP7	XLOC_191560	4215	coding	coding	coding	noncoding
XR_933075.2	LOC105371082	XLOC_191640	1838	coding	noncoding	coding	noncoding
MSTRG.186889.6	LITAF	XLOC_191669	3589	coding	coding	noncoding	noncoding
MSTRG.186903.7	TXNDC11	XLOC_191674	1880	coding	coding	coding	noncoding
MSTRG.186960.1	RSL1D1	XLOC_191678	2267	coding	coding	coding	noncoding
NM_001145204.3	SHISA9	XLOC_191724	6749	coding	coding	coding	coding
MSTRG.187155.3	PARN	XLOC_191779	813	coding	coding	noncoding	noncoding
MSTRG.187212.3	MARF1	XLOC_191806	8096	coding	coding	coding	noncoding
MSTRG.187212.7	MARF1	XLOC_191806	7966	coding	coding	coding	noncoding
MSTRG.187212.9	MARF1	XLOC_191806	7647	coding	coding	coding	noncoding
MSTRG.187212.10	MARF1	XLOC_191806	7647	coding	coding	coding	noncoding
MSTRG.187322.5	NOMO2	XLOC_191865	4701	coding	coding	coding	coding
MSTRG.187384.3	SMG1	XLOC_191871	9427	coding	coding	coding	coding
MSTRG.187523.3	THUMPD1	XLOC_191955	4045	coding	coding	noncoding	noncoding
MSTRG.187528.1	ACSM3	XLOC_191960	311	coding	noncoding	noncoding	noncoding

MSTRG.187580.1	NPIPB3	XLOC_191985	1508	coding	coding	noncoding	noncoding
MSTRG.187588.2	SMG1P3	XLOC_191990	2250	coding	coding	noncoding	noncoding
MSTRG.187784.1	ZKSCAN2	XLOC_192115	399	coding	coding	noncoding	noncoding
MSTRG.188051.1	IL27	XLOC_192217	312	coding	noncoding	coding	noncoding
NM_138414.3	SGF29	XLOC_192222	1159	coding	coding	coding	coding
MSTRG.188258.15	SMG1P6	XLOC_192287	2097	coding	coding	noncoding	coding
MSTRG.188258.20	SMG1P2	XLOC_192287	1253	coding	noncoding	noncoding	noncoding
MSTRG.188258.22	SMG1P2	XLOC_192287	958	coding	noncoding	noncoding	coding
MSTRG.188258.27		XLOC_192287	2749	coding	coding	noncoding	noncoding
MSTRG.188258.26		XLOC_192287	2837	coding	coding	noncoding	noncoding
MSTRG.188188.4		XLOC_192345	2975	coding	coding	noncoding	coding
MSTRG.188332.1	BCL7C	XLOC_192391	278	coding	noncoding	noncoding	noncoding
MSTRG.188678.2	VPS35	XLOC_192582	6165	coding	coding	noncoding	noncoding
MSTRG.188884.4	ITFG1	XLOC_192615	1271	coding	coding	coding	noncoding
MSTRG.188885.1	ITFG1-AS1	XLOC_192616	403	coding	coding	noncoding	noncoding
MSTRG.188914.1	PHKB	XLOC_192638	312	coding	noncoding	noncoding	noncoding
MSTRG.188759.8	N4BP1	XLOC_192657	3054	coding	coding	noncoding	coding
MSTRG.188944.3	LOC101927272	XLOC_192725	5861	coding	noncoding	coding	noncoding
MSTRG.188944.8	SNX20	XLOC_192725	2310	coding	noncoding	noncoding	noncoding
NM_022162.3	NOD2	XLOC_192729	4675	coding	coding	coding	coding
XR_001751995.2	CHD9	XLOC_192796	11755	coding	coding	coding	coding
MSTRG.189304.4	LOC643802	XLOC_192824	1463	coding	coding	noncoding	coding
NM_017839.5	LPCAT2	XLOC_192912	5313	coding	coding	coding	coding
MSTRG.189441.13	FAM192A	XLOC_193019	2209	coding	coding	noncoding	noncoding
XM_011522949.2	ADGRG5	XLOC_193046	4350	coding	coding	coding	coding
MSTRG.189521.3	USB1	XLOC_193072	1230	coding	noncoding	coding	noncoding
NM_001126129.2	GINS3	XLOC_193081	2317	coding	coding	coding	coding
NM_001160305.2	SETD6	XLOC_193087	2985	coding	coding	coding	coding
MSTRG.189605.21	CNOT1	XLOC_193087	4757	coding	coding	coding	coding
MSTRG.190093.3	TK2	XLOC_193391	3569	coding	coding	coding	noncoding
MSTRG.190113.3	DYNC1LI2	XLOC_193418	1934	coding	noncoding	noncoding	noncoding
NM_025187.5	C16orf70	XLOC_193444	2917	coding	coding	coding	coding
MSTRG.190170.2	LRRC29	XLOC_193460	367	coding	noncoding	noncoding	noncoding
MSTRG.190196.2	ZDHHC1	XLOC_193479	1267	coding	coding	coding	coding
XM_011522874.1	CARMIL2	XLOC_193492	4428	coding	coding	coding	coding
MSTRG.190272.3	RANBP10	XLOC_193499	5240	coding	coding	coding	noncoding
NM_001322041.2	NUTF2	XLOC_193505	2442	coding	coding	coding	coding
MSTRG.190295.5	DPEP2	XLOC_193528	3174	coding	coding	coding	coding
XM_011522979.2	PLA2G15	XLOC_193550	2796	coding	coding	coding	coding
MSTRG.190404.2	TERF2	XLOC_193585	1452	coding	coding	noncoding	noncoding
MSTRG.190446.4	PDXDC2P-NPIPB14P	XLOC_193631	1888	coding	coding	noncoding	noncoding
MSTRG.190561.2	AARS	XLOC_193646	3114	coding	coding	coding	coding

MSTRG.190572.3	ST3GAL2	XLOC_193650	4227	coding	coding	coding	coding
MSTRG.190534.4	CMTR2	XLOC_193679	3259	coding	coding	noncoding	noncoding
MSTRG.190595.6	PHLPP2	XLOC_193695	3074	coding	coding	coding	noncoding
MSTRG.190595.10	PHLPP2	XLOC_193695	2618	coding	coding	coding	noncoding
NM_020995.3	HPR	XLOC_193723	1242	coding	coding	coding	coding
MSTRG.190720.1	LOC283922	XLOC_193872	1950	coding	coding	coding	noncoding
MSTRG.190730.3	GLG1	XLOC_193888	5189	coding	coding	coding	coding
NM_001100624.2	CENPN	XLOC_194147	4644	coding	coding	coding	coding
NM_198390.2	CMIP	XLOC_194168	4357	coding	noncoding	coding	coding
MSTRG.191294.1		XLOC_194241	336	coding	noncoding	noncoding	noncoding
MSTRG.191603.2	ZDHHC7	XLOC_194355	5087	coding	coding	coding	noncoding
NM_001318794.2	COX4I1	XLOC_194405	979	coding	coding	coding	coding
MSTRG.185749.1	DDX11L10	XLOC_194686	1159	coding	coding	noncoding	coding
MSTRG.185815.2	HBA1	XLOC_194697	1089	coding	coding	coding	noncoding
MSTRG.185815.10	HBA1	XLOC_194697	995	coding	coding	coding	noncoding
MSTRG.185815.26	HBA1	XLOC_194697	1438	coding	coding	coding	noncoding
MSTRG.186059.1	TELO2	XLOC_194799	275	coding	noncoding	noncoding	noncoding
MSTRG.186596.26	GLYR1	XLOC_195042	13314	coding	coding	coding	coding
MSTRG.186596.31	PPL	XLOC_195042	12687	coding	coding	coding	coding
MSTRG.186841.1	ATF7IP2	XLOC_195196	2493	coding	coding	noncoding	coding
MSTRG.186869.10	CLEC16A	XLOC_195209	3438	coding	coding	coding	coding
MSTRG.186869.13	CLEC16A	XLOC_195209	3399	coding	coding	coding	coding
MSTRG.186869.17	CLEC16A	XLOC_195209	2682	coding	noncoding	coding	coding
NR_024320.1	LITAF	XLOC_195242	2535	coding	coding	coding	coding
XM_011522754.3	LITAF	XLOC_195242	2680	coding	coding	coding	coding
MSTRG.187076.2	ERCC4	XLOC_195334	782	coding	noncoding	noncoding	noncoding
MSTRG.187215.3	MYH11	XLOC_195388	3343	coding	coding	coding	coding
MSTRG.187383.1		XLOC_195449	286	coding	noncoding	noncoding	noncoding
MSTRG.187350.1		XLOC_195469	305	coding	noncoding	noncoding	noncoding
MSTRG.187365.1	SYT17	XLOC_195481	277	coding	coding	noncoding	noncoding
MSTRG.187404.2	CCP110	XLOC_195495	4802	coding	coding	coding	noncoding
MSTRG.187495.1	ACSM1	XLOC_195526	232	coding	noncoding	noncoding	noncoding
MSTRG.187496.1	ACSM1	XLOC_195527	260	coding	noncoding	noncoding	noncoding
MSTRG.187511.1		XLOC_195540	622	coding	noncoding	noncoding	noncoding
MSTRG.187548.6	LYRM1	XLOC_195553	1267	coding	coding	coding	noncoding
MSTRG.187548.7	LYRM1	XLOC_195553	674	coding	coding	noncoding	noncoding
MSTRG.187631.3	RRN3P3	XLOC_195604	6097	coding	coding	coding	noncoding
MSTRG.187631.5	RRN3P3	XLOC_195604	1766	coding	coding	coding	noncoding
MSTRG.187631.9	RRN3P3	XLOC_195604	4649	coding	coding	coding	noncoding
MSTRG.187684.3	DCTN5	XLOC_195635	2047	coding	coding	noncoding	noncoding
MSTRG.187741.2	TNRC6A	XLOC_195658	1828	coding	noncoding	noncoding	coding
MSTRG.187879.2	IL4R	XLOC_195726	5717	coding	coding	coding	coding

MSTRG.187879.4	IL4R	XLOC_195726	4538	coding	coding	coding	noncoding
MSTRG.187978.2	KIAA0556	XLOC_195734	776	coding	noncoding	noncoding	noncoding
XM_024450212.1	XPO6	XLOC_195757	4672	coding	coding	coding	coding
NM_001042432.1	CLN3	XLOC_195801	1913	coding	coding	coding	coding
XM_024450408.1	SULT1A1	XLOC_195811	1799	coding	coding	coding	coding
MSTRG.188206.3	RRN3P2	XLOC_195839	2144	coding	coding	noncoding	noncoding
MSTRG.188206.4	RRN3P2	XLOC_195839	788	coding	coding	noncoding	noncoding
NM_001031827.2	BOLA2	XLOC_195864	1017	coding	coding	coding	coding
MSTRG.188259.16	BOLA2-SMG1P6	XLOC_195864	1582	coding	coding	coding	coding
MSTRG.188143.4	PPP4C	XLOC_195909	1304	coding	coding	coding	noncoding
XR_001751850.1	SEPTIN1	XLOC_195936	1641	coding	coding	coding	coding
NM_002773.5	PRSS8	XLOC_195991	1837	coding	coding	coding	coding
MSTRG.188585.10	ZNF267	XLOC_196043	7048	coding	coding	coding	noncoding
MSTRG.188888.1	ITFG1	XLOC_196175	369	coding	noncoding	noncoding	noncoding
NM_003031.4	SIAH1	XLOC_196199	2110	coding	coding	coding	coding
MSTRG.188878.2	TENT4B	XLOC_196273	3453	coding	coding	coding	noncoding
MSTRG.188970.5	BRD7	XLOC_196275	13724	coding	coding	coding	coding
MSTRG.189047.5	NOD2	XLOC_196294	4143	coding	coding	coding	coding
MSTRG.189052.7	LOC102724907	XLOC_196295	1790	coding	coding	noncoding	noncoding
MSTRG.189052.12	LOC102724907	XLOC_196295	8414	coding	coding	noncoding	noncoding
MSTRG.189052.16	LOC102724907	XLOC_196295	2824	coding	coding	noncoding	coding
MSTRG.189052.18	LOC102724907	XLOC_196295	8341	coding	coding	noncoding	noncoding
MSTRG.189273.3	CHD9	XLOC_196352	6740	coding	coding	noncoding	coding
MSTRG.189273.5	CHD9	XLOC_196352	4252	coding	coding	coding	coding
MSTRG.189273.7	CHD9	XLOC_196352	11029	coding	coding	noncoding	coding
MSTRG.189273.10	CHD9	XLOC_196352	4288	coding	coding	coding	coding
MSTRG.189273.15	CHD9	XLOC_196352	5315	coding	coding	noncoding	noncoding
MSTRG.189317.9	AKTIP	XLOC_196361	6298	coding	coding	coding	coding
MSTRG.190163.2	E2F4	XLOC_196831	2198	coding	coding	coding	coding
MSTRG.190358.2	SLC7A6OS	XLOC_196902	5671	coding	coding	coding	noncoding
MSTRG.190345.1	CDH1	XLOC_196922	2045	coding	coding	coding	noncoding
MSTRG.190420.12	NFAT5	XLOC_196945	9458	coding	noncoding	noncoding	noncoding
MSTRG.190495.3	SF3B3	XLOC_196997	4082	coding	coding	noncoding	coding
NM_001201553.1	ZNF821	XLOC_197043	1985	coding	noncoding	coding	coding
MSTRG.190635.2	HP	XLOC_197054	1109	coding	coding	coding	noncoding
MSTRG.190945.1	ZNRF1	XLOC_197174	286	coding	noncoding	noncoding	noncoding
MSTRG.192386.1	WWOX	XLOC_197330	235	coding	noncoding	noncoding	noncoding
MSTRG.191375.11	PLCG2	XLOC_197535	3247	coding	coding	coding	noncoding
MSTRG.191479.1	OSGIN1	XLOC_197615	262	coding	noncoding	noncoding	noncoding
MSTRG.191579.2	KLHL36	XLOC_197660	3984	coding	coding	coding	coding
MSTRG.191596.1	CRISPLD2	XLOC_197669	4562	coding	coding	coding	coding
NM_001282683.2	FBXO31	XLOC_197746	5732	coding	noncoding	coding	coding

MSTRG.192193.18	FAM157C	XLOC_197936	4285	coding	noncoding	coding	coding
MSTRG.192276.3	PITPNA	XLOC_198003	1250	coding	coding	noncoding	coding
MSTRG.192764.3	ZZEF1	XLOC_198122	11227	coding	coding	coding	coding
NM_182538.5	SPNS3	XLOC_198129	1877	coding	coding	coding	coding
MSTRG.192862.3	ENO3	XLOC_198173	1548	coding	coding	coding	noncoding
MSTRG.193023.1	KIAA0753	XLOC_198236	3593	coding	coding	noncoding	coding
NM_001102614.1	SLC35G6	XLOC_198318	1195	coding	noncoding	coding	coding
MSTRG.193169.3	SAT2	XLOC_198331	789	coding	coding	coding	noncoding
MSTRG.193535.5	GAS7	XLOC_198483	3970	coding	coding	coding	coding
MSTRG.193893.4	COX10-AS1	XLOC_198683	1123	coding	noncoding	noncoding	noncoding
MSTRG.194031.11	TTC19	XLOC_198776	15431	coding	coding	coding	noncoding
NR_045022.1	SNHG29	XLOC_198791	1067	coding	noncoding	noncoding	noncoding
MSTRG.194174.6	CCDC144B	XLOC_198907	923	coding	coding	coding	noncoding
MSTRG.194293.1	AKAP10	XLOC_198963	5217	coding	coding	coding	noncoding
MSTRG.194293.3	AKAP10	XLOC_198963	4357	coding	coding	coding	noncoding
MSTRG.194694.1	RAB34	XLOC_199193	1272	coding	coding	coding	noncoding
MSTRG.194923.2	SSH2	XLOC_199271	10481	coding	coding	coding	coding
MSTRG.195188.2	EVI2A	XLOC_199426	2883	coding	coding	noncoding	noncoding
MSTRG.195188.6	EVI2A	XLOC_199426	9596	coding	coding	noncoding	noncoding
XR_002958063.1	ZNF207	XLOC_199473	9430	coding	noncoding	coding	coding
MSTRG.195457.4	SLFN11	XLOC_199635	4476	coding	coding	noncoding	noncoding
MSTRG.195452.1		XLOC_199639	228	coding	noncoding	noncoding	noncoding
MSTRG.195463.1	SLFN12	XLOC_199643	5379	coding	coding	noncoding	noncoding
MSTRG.195505.11	SLFN12L	XLOC_199644	3554	coding	coding	noncoding	noncoding
MSTRG.195527.4	LOC107985033	XLOC_199647	5807	coding	coding	noncoding	noncoding
NR_135479.1	SNHG30	XLOC_199648	825	coding	noncoding	noncoding	noncoding
MSTRG.195643.7	SYNRG	XLOC_199710	7291	coding	noncoding	coding	coding
MSTRG.195643.8	SYNRG	XLOC_199710	5414	coding	noncoding	coding	coding
MSTRG.195643.12	SYNRG	XLOC_199710	4274	coding	noncoding	coding	coding
XM_006722258.4	LOC101929950	XLOC_199721	916	coding	coding	coding	coding
MSTRG.195763.11	ERBB2	XLOC_199784	1376	coding	coding	noncoding	noncoding
MSTRG.195777.6	IKZF3	XLOC_199786	5551	coding	noncoding	coding	coding
MSTRG.195777.7	IKZF3	XLOC_199786	3982	coding	coding	coding	coding
MSTRG.195777.8	IKZF3	XLOC_199786	3197	coding	noncoding	coding	coding
XM_005257163.2	CASC3	XLOC_199803	3906	coding	coding	coding	coding
MSTRG.195838.1	TOP2A	XLOC_199815	206	coding	coding	noncoding	noncoding
MSTRG.195888.2	SMARCE1	XLOC_199835	3851	coding	coding	noncoding	noncoding
MSTRG.195909.5	LOC107985072	XLOC_199843	1329	coding	coding	coding	noncoding
MSTRG.195973.5	ACLY	XLOC_199878	3852	coding	coding	coding	coding
MSTRG.195965.2	DHX58	XLOC_199884	2532	coding	coding	coding	coding
MSTRG.195965.3	DHX58	XLOC_199884	1466	coding	coding	coding	noncoding
MSTRG.196031.5	RETREG3	XLOC_199912	3784	coding	noncoding	coding	noncoding

MSTRG.196257.5	UBTF	XLOC_200039	1130	coding	coding	coding	noncoding
NM_002087.3	GRN	XLOC_200046	2346	coding	coding	coding	coding
MSTRG.196401.2	KANSL1	XLOC_200115	5331	coding	coding	coding	noncoding
MSTRG.196478.1	ARL17B	XLOC_200121	870	coding	noncoding	noncoding	noncoding
NM_002476.2	MYL4	XLOC_200140	855	coding	noncoding	coding	coding
MSTRG.196439.1	MRPL45P2	XLOC_200148	1382	coding	noncoding	noncoding	noncoding
XM_011524697.2	SNX11	XLOC_200189	2506	coding	coding	coding	coding
MSTRG.196689.16	SKAP1	XLOC_200192	21085	coding	noncoding	coding	coding
XM_005257429.2	ABI3	XLOC_200241	1775	coding	coding	coding	coding
MSTRG.196652.3	ZNF652	XLOC_200247	5186	coding	coding	coding	coding
MSTRG.196675.3	SPOP	XLOC_200260	2874	coding	coding	noncoding	noncoding
MSTRG.196675.5	SPOP	XLOC_200260	2765	coding	coding	coding	noncoding
MSTRG.196675.6	SPOP	XLOC_200260	1283	coding	coding	noncoding	noncoding
NM_001199900.1	PDK2	XLOC_200279	980	coding	coding	coding	coding
XR_002958056.1	SGCA	XLOC_200287	1990	coding	coding	coding	coding
NM_138962.4	MSI2	XLOC_200513	6379	coding	coding	coding	coding
MSTRG.197355.13	SRSF1	XLOC_200540	9642	coding	coding	noncoding	noncoding
XM_011525093.2	RAD51C	XLOC_200599	1574	coding	coding	coding	coding
MSTRG.197527.4	TRIM37	XLOC_200614	1450	coding	noncoding	noncoding	noncoding
NM_001329399.2	VMP1	XLOC_200640	3595	coding	noncoding	coding	coding
MSTRG.197793.20	USP32	XLOC_200661	2377	coding	coding	noncoding	noncoding
MSTRG.197541.2	APPBP2	XLOC_200667	4742	coding	coding	noncoding	noncoding
MSTRG.197597.1	MED13	XLOC_200747	11215	coding	coding	coding	noncoding
NM_002401.5	MAP3K3	XLOC_200816	4752	coding	coding	coding	coding
MSTRG.197833.3	STRADA	XLOC_200820	1931	coding	coding	coding	coding
MSTRG.197891.2	CCDC47	XLOC_200822	3247	coding	coding	coding	coding
NM_001191029.2	PRR29	XLOC_200839	3055	coding	coding	coding	coding
MSTRG.197996.16	MILR1	XLOC_200850	7518	coding	coding	coding	noncoding
MSTRG.197996.22	DDX5	XLOC_200850	6082	coding	coding	coding	noncoding
MSTRG.197996.26	CEP95	XLOC_200850	4627	coding	coding	noncoding	noncoding
MSTRG.197996.25	CEP95	XLOC_200850	5746	coding	coding	noncoding	noncoding
MSTRG.198178.2	PSMD12	XLOC_200984	3569	coding	coding	coding	noncoding
MSTRG.198283.5	SLC16A6	XLOC_201047	2612	coding	coding	coding	coding
MSTRG.198283.26	ARSG	XLOC_201047	3836	coding	coding	noncoding	noncoding
NM_001278433.1	PRKAR1A	XLOC_201048	4307	coding	coding	coding	coding
MSTRG.198621.1	SLC39A11	XLOC_201191	469	coding	noncoding	noncoding	noncoding
MSTRG.198569.1	LINC00469	XLOC_201235	208	coding	coding	noncoding	noncoding
MSTRG.198668.7	LOC107985074	XLOC_201271	1959	coding	coding	coding	noncoding
NM_001545.3	MRPL58	XLOC_201304	894	coding	coding	coding	coding
XR_002958068.1	ARMC7	XLOC_201310	2291	coding	coding	coding	noncoding
MSTRG.198772.5	GGA3	XLOC_201316	2001	coding	coding	coding	noncoding
MSTRG.198855.3	ZACN	XLOC_201364	4797	coding	coding	coding	coding

MSTRG.198855.5	ZACN	XLOC_201364	2969	coding	coding	coding	coding
MSTRG.198904.7	RHBDF2	XLOC_201384	3169	coding	coding	coding	coding
NR_038109.1	SNHG16	XLOC_201396	2534	coding	noncoding	noncoding	noncoding
MSTRG.198964.29	MFSD11	XLOC_201412	4445	coding	coding	coding	coding
MSTRG.199092.1	AFMID	XLOC_201495	533	coding	coding	noncoding	coding
XM_005257546.4	RNF213	XLOC_201588	17680	coding	coding	noncoding	coding
MSTRG.199508.2	MAFG	XLOC_201699	1488	coding	coding	coding	coding
MSTRG.199564.3	CD7	XLOC_201743	1267	coding	coding	coding	coding
NM_001004431.3	METRNL	XLOC_201775	1690	coding	noncoding	coding	coding
MSTRG.192624.4	PAFAH1B1	XLOC_201869	5327	coding	coding	coding	noncoding
MSTRG.192859.4	SLC25A11	XLOC_201988	3409	coding	coding	coding	coding
MSTRG.192859.10	SLC25A11	XLOC_201988	2826	coding	coding	coding	coding
MSTRG.192968.1	NUP88	XLOC_202010	9859	coding	coding	coding	coding
MSTRG.193034.1	ALOX15P1	XLOC_202072	510	coding	coding	noncoding	noncoding
NM_177550.4	SLC13A5	XLOC_202078	3289	coding	coding	coding	coding
MSTRG.192999.3	XAF1	XLOC_202081	4629	coding	coding	coding	coding
MSTRG.192999.8	XAF1	XLOC_202081	2698	coding	coding	coding	coding
MSTRG.193084.1	ASGR2	XLOC_202117	248	coding	noncoding	noncoding	noncoding
MSTRG.193106.2	LOC105371512	XLOC_202122	4755	coding	noncoding	coding	noncoding
MSTRG.193118.19	DLG4	XLOC_202124	2597	coding	coding	coding	coding
MSTRG.193147.2	POLR2A	XLOC_202142	2614	coding	coding	coding	coding
MSTRG.193155.2	TNFSF13	XLOC_202145	2056	coding	coding	noncoding	coding
MSTRG.193260.4	CHD3	XLOC_202175	1954	coding	coding	coding	coding
XR_002958073.1	CTC1	XLOC_202208	7300	coding	coding	coding	coding
MSTRG.193898.4	COX10	XLOC_202425	300	coding	noncoding	noncoding	noncoding
MSTRG.194013.2	NT5M	XLOC_202541	1466	coding	coding	coding	noncoding
MSTRG.194271.3	MFAP4	XLOC_202679	513	coding	coding	coding	coding
NM_152908.4	SLC47A2	XLOC_202694	2232	coding	coding	coding	coding
MSTRG.194384.2	DHRS7B	XLOC_202761	1234	coding	coding	noncoding	noncoding
MSTRG.194598.2	KSR1	XLOC_202846	664	coding	coding	coding	noncoding
NM_005148.3	UNC119	XLOC_202897	1384	coding	coding	coding	coding
NM_001033561.2	PHF12	XLOC_202931	4506	coding	coding	coding	coding
MSTRG.194840.4	TAOK1	XLOC_202968	10966	coding	coding	coding	noncoding
MSTRG.194840.9	TAOK1	XLOC_202968	11726	coding	noncoding	noncoding	noncoding
MSTRG.194840.10	TAOK1	XLOC_202968	5867	coding	noncoding	noncoding	noncoding
MSTRG.195117.2	SUZ12P1	XLOC_203069	817	coding	noncoding	noncoding	noncoding
MSTRG.195140.2	ADAP2	XLOC_203077	2398	coding	coding	coding	noncoding
MSTRG.195156.1	LOC107984974	XLOC_203087	272	coding	noncoding	noncoding	noncoding
MSTRG.195441.1		XLOC_203244	251	coding	coding	noncoding	noncoding
MSTRG.195538.7	AP2B1	XLOC_203284	5842	coding	coding	coding	coding
MSTRG.195620.1	TBC1D3D	XLOC_203347	254	coding	noncoding	coding	noncoding
MSTRG.195748.7	CDK12	XLOC_203412	4854	coding	coding	coding	coding

NM_021724.5	NR1D1	XLOC_203453	2630	coding	coding	coding	coding
MSTRG.196002.5	ATP6V0A1	XLOC_203617	421	coding	coding	noncoding	noncoding
MSTRG.196035.4	PSMC3IP	XLOC_203622	2263	coding	coding	noncoding	noncoding
MSTRG.196155.3	NBR2	XLOC_203664	1239	coding	coding	noncoding	noncoding
MSTRG.196485.5	NSF	XLOC_203828	4709	coding	coding	coding	coding
MSTRG.196485.9	NSF	XLOC_203828	1029	coding	coding	noncoding	noncoding
MSTRG.196431.1	EFCAB13	XLOC_203863	256	coding	noncoding	noncoding	noncoding
MSTRG.196536.2	PNPO	XLOC_203896	1465	coding	coding	coding	coding
MSTRG.196554.4	NFE2L1	XLOC_203902	3340	coding	coding	coding	coding
MSTRG.196554.3	NFE2L1	XLOC_203902	4654	coding	coding	coding	coding
MSTRG.196554.5	NFE2L1	XLOC_203902	3228	coding	coding	coding	coding
MSTRG.196631.4	UBE2Z	XLOC_204029	2534	coding	noncoding	noncoding	noncoding
MSTRG.196843.1	PPP1R9B	XLOC_204100	444	coding	noncoding	noncoding	noncoding
NM_001243877.1	TOB1	XLOC_204143	2224	coding	coding	coding	coding
MSTRG.197242.5	DGKE	XLOC_204347	639	coding	coding	noncoding	noncoding
MSTRG.197426.5	SUPT4H1	XLOC_204433	1756	coding	noncoding	coding	noncoding
MSTRG.197457.2	PRR11	XLOC_204463	1194	coding	noncoding	noncoding	noncoding
MSTRG.197794.3	CA4	XLOC_204502	1048	coding	coding	coding	noncoding
MSTRG.197680.5	BCAS3	XLOC_204541	2838	coding	coding	coding	noncoding
XR_001752965.1	LOC105371857	XLOC_204610	1149	coding	noncoding	noncoding	noncoding
MSTRG.197673.3	DCAF7	XLOC_204625	1434	coding	noncoding	noncoding	coding
MSTRG.197997.2	MILR1	XLOC_204673	1485	coding	coding	noncoding	noncoding
MSTRG.198232.11	BPTF	XLOC_204800	9150	coding	coding	coding	noncoding
MSTRG.198232.10	BPTF	XLOC_204800	10142	coding	coding	coding	noncoding
MSTRG.198255.4	LINC00674	XLOC_204805	6287	coding	coding	coding	coding
MSTRG.198525.1	FAM104A	XLOC_204958	4693	coding	coding	coding	coding
XM_005257182.2	CDC42EP4	XLOC_204960	3416	coding	coding	coding	coding
MSTRG.198770.19	MIF4GD	XLOC_205048	2767	coding	coding	coding	coding
XM_005257822.4	RECQL5	XLOC_205086	3089	coding	coding	coding	coding
MSTRG.199026.14	SEC14L1	XLOC_205186	5320	coding	coding	coding	coding
MSTRG.199026.18	SEC14L1	XLOC_205186	4954	coding	coding	coding	coding
MSTRG.199026.21	SEC14L1	XLOC_205186	4840	coding	coding	coding	coding
MSTRG.199026.20	SEC14L1	XLOC_205186	4944	coding	coding	coding	coding
MSTRG.199026.24	SEC14L1	XLOC_205186	4773	coding	coding	coding	coding
MSTRG.199026.23	SEC14L1	XLOC_205186	4812	coding	coding	coding	coding
MSTRG.199094.2	AFMID	XLOC_205236	474	coding	noncoding	noncoding	noncoding
MSTRG.199330.4	LOC100294362	XLOC_205342	18824	coding	coding	coding	coding
MSTRG.199533.4	GPS1	XLOC_205454	1828	coding	coding	coding	coding
MSTRG.199533.5	GPS1	XLOC_205454	1796	coding	coding	coding	coding
NM_022156.5	DUS1L	XLOC_205454	2233	coding	coding	coding	coding
MSTRG.199570.4	CSNK1D	XLOC_205462	1777	coding	coding	coding	coding
NR_110578.2	CSNK1D	XLOC_205462	3572	coding	noncoding	coding	coding

XR_933922.3	CSNK1D	XLOC_205462	4014	coding	coding	coding	coding
XR_002957961.1	CSNK1D	XLOC_205462	4259	coding	coding	coding	coding
XM_017025073.2	CYBC1	XLOC_205474	3102	coding	coding	coding	coding
XM_024451242.1	TYMS	XLOC_205535	1176	coding	coding	coding	coding
MSTRG.199836.7	LPIN2	XLOC_205602	2085	coding	coding	coding	noncoding
XM_017025958.1	TGIF1	XLOC_205635	1940	coding	coding	coding	coding
MSTRG.200305.8	PPP4R1	XLOC_205893	4012	coding	coding	coding	noncoding
MSTRG.200612.1	CEP76	XLOC_206047	554	coding	noncoding	coding	noncoding
MSTRG.200618.1		XLOC_206065	308	coding	noncoding	noncoding	noncoding
MSTRG.200870.2	ROCK1	XLOC_206211	5262	coding	coding	coding	noncoding
MSTRG.200870.3	ROCK1	XLOC_206211	5375	coding	coding	coding	noncoding
MSTRG.200884.2	ROCK1	XLOC_206212	6387	coding	noncoding	noncoding	noncoding
MSTRG.200953.2	ABHD3	XLOC_206230	3191	coding	noncoding	noncoding	noncoding
MSTRG.201111.12	NPC1	XLOC_206317	4170	coding	coding	coding	coding
MSTRG.201534.2	DSC2	XLOC_206563	3950	coding	coding	noncoding	noncoding
MSTRG.201570.4	TRAPPC8	XLOC_206597	5041	coding	coding	coding	coding
MSTRG.201776.6	LOC107985139	XLOC_206726	9171	coding	coding	noncoding	noncoding
XR_002958176.1	PIK3C3	XLOC_206912	6400	coding	coding	coding	coding
MSTRG.202127.1		XLOC_206959	325	coding	noncoding	noncoding	noncoding
XM_024451149.1	SETBP1	XLOC_206999	10177	coding	noncoding	coding	coding
MSTRG.202413.5	EPG5	XLOC_207070	2769	coding	noncoding	noncoding	noncoding
MSTRG.202605.4	PIAS2	XLOC_207136	723	coding	coding	noncoding	noncoding
MSTRG.202668.4	SMAD2	XLOC_207160	3494	coding	coding	noncoding	noncoding
MSTRG.202730.3	RPL17-C18orf32	XLOC_207200	937	coding	coding	noncoding	noncoding
MSTRG.202730.6	RPL17-C18orf32	XLOC_207200	665	coding	coding	noncoding	noncoding
MSTRG.203140.4	TCF4	XLOC_207368	2274	coding	noncoding	noncoding	noncoding
MSTRG.203503.2	RNF152	XLOC_207604	589	coding	coding	coding	noncoding
MSTRG.203891.8	BCL2	XLOC_207685	5002	coding	coding	coding	noncoding
MSTRG.204090.9	CD226	XLOC_207868	4057	coding	coding	noncoding	noncoding
MSTRG.204564.6	ZNF407	XLOC_208043	4339	coding	noncoding	coding	noncoding
MSTRG.204527.6	ZNF516	XLOC_208180	5346	coding	coding	coding	coding
MSTRG.204527.7	ZNF516	XLOC_208180	5238	coding	coding	coding	coding
MSTRG.204734.11	MBP	XLOC_208218	41498	coding	coding	noncoding	coding
MSTRG.204734.15	MBP	XLOC_208218	6230	coding	noncoding	coding	noncoding
MSTRG.204798.3	KCNG2	XLOC_208293	8929	coding	coding	coding	coding
MSTRG.204811.1	SLC66A2	XLOC_208294	408	coding	noncoding	noncoding	noncoding
MSTRG.204825.1	TXNL4A	XLOC_208304	411	coding	coding	noncoding	noncoding
MSTRG.199824.4	SMCHD1	XLOC_208402	7423	coding	coding	coding	noncoding
MSTRG.199824.2	SMCHD1	XLOC_208402	11031	coding	coding	coding	coding
MSTRG.199824.7	SMCHD1	XLOC_208402	6127	coding	coding	coding	noncoding
MSTRG.199931.3	LINC00667	XLOC_208502	1197	coding	coding	noncoding	noncoding
MSTRG.200337.2	PTPRM	XLOC_208593	1962	coding	coding	coding	noncoding

NR_052003.1	PPP4R1	XLOC_208633	3941	coding	coding	coding	coding
MSTRG.200388.6	VAPA	XLOC_208653	4363	coding	coding	coding	noncoding
NM_023075.6	MPPE1	XLOC_208726	3330	coding	coding	coding	coding
MSTRG.200631.9	CEP192	XLOC_208771	8000	coding	noncoding	coding	noncoding
MSTRG.200918.1	LDLRAD4	XLOC_208799	1008	coding	noncoding	noncoding	coding
MSTRG.200804.1		XLOC_208885	289	coding	coding	noncoding	noncoding
MSTRG.201041.4	RBBP8	XLOC_208974	2929	coding	coding	noncoding	noncoding
MSTRG.201796.1	ZSCAN30	XLOC_209306	1796	coding	coding	noncoding	noncoding
MSTRG.202315.1	KIAA1328	XLOC_209383	317	coding	noncoding	noncoding	noncoding
MSTRG.202188.2	PIK3C3	XLOC_209518	10176	coding	coding	noncoding	noncoding
MSTRG.202222.1		XLOC_209533	226	coding	coding	noncoding	noncoding
MSTRG.202246.1	LINC00907	XLOC_209554	236	coding	noncoding	noncoding	noncoding
NM_001199344.2	RPL17	XLOC_209830	796	coding	coding	coding	coding
NM_001199341.2	RPL17	XLOC_209830	813	coding	coding	coding	coding
MSTRG.203217.2	WDR7	XLOC_210041	4725	coding	coding	coding	noncoding
XM_017025614.2	FECH	XLOC_210065	2406	coding	coding	coding	coding
NM_052947.4	ALPK2	XLOC_210100	7437	coding	coding	coding	coding
MSTRG.203406.3	PMAIP1	XLOC_210145	769	coding	coding	coding	coding
MSTRG.203636.4	RELCH	XLOC_210210	4347	coding	noncoding	coding	noncoding
MSTRG.203636.12	RELCH	XLOC_210210	1038	coding	noncoding	noncoding	noncoding
MSTRG.203553.1		XLOC_210220	305	coding	noncoding	noncoding	noncoding
MSTRG.203560.8	ZCCHC2	XLOC_210223	8894	coding	coding	coding	noncoding
MSTRG.203986.1	CCDC102B	XLOC_210463	317	coding	noncoding	noncoding	noncoding
MSTRG.204030.1	DOK6	XLOC_210479	452	coding	coding	noncoding	noncoding
MSTRG.204138.1	GTSCR1	XLOC_210553	330	coding	noncoding	noncoding	noncoding
MSTRG.204392.3	CNDP2	XLOC_210664	2882	coding	coding	coding	coding
MSTRG.204454.2	TSHZ1	XLOC_210689	1006	coding	noncoding	coding	noncoding
MSTRG.204693.4	ZNF236	XLOC_210773	2599	coding	coding	coding	noncoding
MSTRG.204844.2	ADNP2	XLOC_210889	986	coding	noncoding	noncoding	noncoding
NM_001303501.2	CNN2	XLOC_210980	2212	coding	coding	coding	coding
NM_001687.5	ATP5F1D	XLOC_210991	995	coding	coding	coding	coding
NM_001300829.2	CIRBP	XLOC_210997	3346	coding	coding	coding	coding
MSTRG.205314.1	GNA15	XLOC_211138	386	coding	noncoding	noncoding	noncoding
MSTRG.205851.1	CLEC4G	XLOC_211438	508	coding	coding	coding	noncoding
MSTRG.206201.1		XLOC_211621	238	coding	noncoding	noncoding	noncoding
MSTRG.206207.2	ATG4D	XLOC_211633	4686	coding	coding	coding	coding
MSTRG.206334.2	ACP5	XLOC_211697	1481	coding	coding	coding	noncoding
MSTRG.206475.1	ZNF564	XLOC_211768	1369	coding	coding	noncoding	noncoding
MSTRG.206492.2	MAN2B1	XLOC_211777	3067	coding	coding	coding	coding
MSTRG.206497.3	TNPO2	XLOC_211782	2333	coding	coding	coding	coding
MSTRG.206701.49	ZNF333	XLOC_211871	7400	coding	coding	coding	noncoding
XM_024451681.1	TPM4	XLOC_211942	2286	coding	coding	coding	coding

MSTRG.206955.1	ANO8	XLOC_212031	271	coding	noncoding	noncoding	noncoding
MSTRG.207065.2	LSM4	XLOC_212085	1194	coding	coding	coding	noncoding
MSTRG.207649.5	ZNF91	XLOC_212374	4768	coding	coding	noncoding	noncoding
MSTRG.207617.1		XLOC_212411	404	coding	noncoding	noncoding	noncoding
MSTRG.207802.5	LOC101927151	XLOC_212441	3081	coding	noncoding	coding	noncoding
MSTRG.208221.1	SCGB1B2P	XLOC_212713	378	coding	noncoding	noncoding	noncoding
NM_001242597.2	TMEM147	XLOC_212810	939	coding	coding	coding	coding
MSTRG.208386.4	U2AF1L4	XLOC_212822	994	coding	coding	noncoding	noncoding
NM_001302632.2	CAPNS1	XLOC_212849	1533	coding	coding	coding	coding
MSTRG.208474.2	ZNF529-AS1	XLOC_212870	3783	coding	coding	noncoding	noncoding
MSTRG.208585.1	ZNF829	XLOC_212900	665	coding	coding	noncoding	noncoding
NM_021913.5	AXL	XLOC_213143	4717	coding	coding	coding	coding
MSTRG.209065.1	BCKDHA	XLOC_213162	3672	coding	coding	coding	coding
MSTRG.209239.1	CEACAM8	XLOC_213274	1427	coding	coding	noncoding	noncoding
MSTRG.209239.2	CEACAM8	XLOC_213274	1027	coding	coding	noncoding	noncoding
MSTRG.209358.6	PLAUR	XLOC_213326	1403	coding	coding	coding	coding
NM_001294.4	CLPTM1	XLOC_213432	2470	coding	coding	coding	coding
NM_001329921.1	CALM3	XLOC_213513	2179	coding	coding	coding	coding
XM_005259190.5	C5AR1	XLOC_213553	2263	coding	coding	coding	coding
MSTRG.209963.4	CARD8-AS1	XLOC_213614	7064	coding	coding	coding	noncoding
MSTRG.209963.11	CARD8-AS1	XLOC_213614	4449	coding	coding	coding	coding
MSTRG.209963.14	CARD8-AS1	XLOC_213614	6397	coding	coding	coding	noncoding
MSTRG.209963.17	CARD8-AS1	XLOC_213614	4225	coding	coding	coding	noncoding
NM_001204502.2	FLT3LG	XLOC_213705	1101	coding	coding	coding	coding
MSTRG.210114.7	BCL2L12	XLOC_213726	2300	coding	coding	coding	coding
MSTRG.210140.3	MIR4751	XLOC_213748	3377	coding	coding	coding	coding
MSTRG.210264.1	CTU1	XLOC_213810	223	coding	noncoding	noncoding	noncoding
MSTRG.210362.3	FPR1	XLOC_213861	3521	coding	coding	coding	coding
MSTRG.210382.3	ZNF350	XLOC_213873	2179	coding	coding	noncoding	noncoding
MSTRG.210558.1	ZNF611	XLOC_213924	3536	coding	coding	noncoding	noncoding
NM_031895.6	CACNG8	XLOC_214039	8850	coding	coding	coding	coding
NM_001278428.3	LILRB4	XLOC_214071	3902	coding	coding	coding	coding
MSTRG.210654.2	TNNT1	XLOC_214087	325	coding	noncoding	noncoding	noncoding
MSTRG.210672.3	TMEM86B	XLOC_214093	1029	coding	coding	coding	coding
MSTRG.210699.2	ISOC2	XLOC_214110	671	coding	coding	coding	noncoding
MSTRG.211000.2	ZNF671	XLOC_214280	2584	coding	coding	coding	coding
MSTRG.204975.4	PLPPR3	XLOC_214420	3547	coding	coding	coding	coding
MSTRG.204972.1	ELANE	XLOC_214422	836	coding	coding	coding	noncoding
MSTRG.205049.2	POLR2E	XLOC_214439	7036	coding	coding	coding	coding
XM_011527804.3	SBNO2	XLOC_214441	4891	coding	coding	coding	coding
MSTRG.205005.2	MIDN	XLOC_214451	3496	coding	noncoding	coding	coding
MSTRG.205030.3	RPS15	XLOC_214464	637	coding	coding	coding	noncoding

MSTRG.205266.2	ZNF554	XLOC_214568	991	coding	coding	noncoding	noncoding
XR_936248.2	LOC101928631	XLOC_214573	588	coding	noncoding	noncoding	noncoding
MSTRG.205371.2	FZR1	XLOC_214616	702	coding	noncoding	coding	noncoding
MSTRG.205474.3	MPND	XLOC_214670	289	coding	coding	noncoding	noncoding
MSTRG.205789.1	LOC105372256	XLOC_214820	4533	coding	coding	coding	coding
MSTRG.205799.3	ZNF557	XLOC_214829	2813	coding	coding	noncoding	noncoding
MSTRG.205819.5	ARHGEF18	XLOC_214838	1505	coding	noncoding	noncoding	noncoding
MSTRG.205915.5	CERS4	XLOC_214893	1663	coding	coding	coding	noncoding
MSTRG.205944.3	2-Mar	XLOC_214904	1424	coding	coding	coding	noncoding
MSTRG.205951.1	HNRNPM	XLOC_214905	2355	coding	coding	coding	coding
MSTRG.205951.3	HNRNPM	XLOC_214905	2468	coding	coding	coding	coding
MSTRG.205990.1	MYO1F	XLOC_214915	338	coding	noncoding	noncoding	noncoding
NM_016581.5	ECSIT	XLOC_215091	1647	coding	coding	coding	coding
MSTRG.206422.1	ZNF433-AS1	XLOC_215136	358	coding	noncoding	coding	noncoding
MSTRG.206369.1	ZNF788P	XLOC_215149	776	coding	coding	noncoding	noncoding
MSTRG.206586.6	NFIX	XLOC_215215	2444	coding	coding	coding	noncoding
NM_003765.3	STX10	XLOC_215217	1304	coding	coding	coding	coding
MSTRG.206639.9	DDX39A	XLOC_215257	6195	coding	coding	coding	coding
MSTRG.206639.13	DDX39A	XLOC_215257	6355	coding	coding	coding	coding
NM_001300914.1	DNAJB1	XLOC_215266	2260	coding	coding	coding	coding
MSTRG.206812.2	FAM32A	XLOC_215363	1470	coding	noncoding	coding	coding
MSTRG.206911.3	SIN3B	XLOC_215405	2658	coding	coding	coding	coding
MSTRG.206941.3	BABAM1	XLOC_215424	1434	coding	noncoding	coding	coding
MSTRG.206941.4	BABAM1	XLOC_215424	1386	coding	noncoding	coding	coding
XM_005259739.4	LRRC25	XLOC_215502	1967	coding	noncoding	coding	coding
MSTRG.207123.2	REX1BD	XLOC_215515	698	coding	coding	coding	noncoding
MSTRG.207249.3	ZNF101	XLOC_215575	2089	coding	coding	noncoding	coding
XR_001754067.1	LOC105372310	XLOC_215616	4416	coding	coding	noncoding	noncoding
MSTRG.207556.1	ZNF431	XLOC_215651	2731	coding	coding	noncoding	noncoding
MSTRG.207582.12	ZNF493	XLOC_215666	1381	coding	coding	noncoding	noncoding
MSTRG.207436.4	ZNF429	XLOC_215683	1792	coding	coding	noncoding	noncoding
MSTRG.207524.1	ZNF492	XLOC_215734	258	coding	noncoding	noncoding	noncoding
MSTRG.207704.7	ZNF254	XLOC_215781	2827	coding	coding	noncoding	noncoding
MSTRG.208056.3	DPY19L3	XLOC_215960	2588	coding	coding	noncoding	noncoding
MSTRG.208111.2	GPATCH1	XLOC_215990	684	coding	noncoding	noncoding	noncoding
MSTRG.208111.3	GPATCH1	XLOC_215990	759	coding	noncoding	noncoding	noncoding
MSTRG.208174.4	LSM14A	XLOC_216021	839	coding	coding	coding	noncoding
MSTRG.208185.2	KIAA0355	XLOC_216023	3239	coding	coding	coding	coding
MSTRG.208185.3	KIAA0355	XLOC_216023	795	coding	coding	noncoding	noncoding
MSTRG.208197.4	UBA2	XLOC_216027	1695	coding	coding	noncoding	noncoding
MSTRG.208338.8	FFAR2	XLOC_216085	1575	coding	coding	coding	noncoding
NR_033390.1	TYROBP	XLOC_216125	551	coding	noncoding	noncoding	noncoding

MSTRG.208444.11	LOC107985304	XLOC_216147	3527	coding	coding	noncoding	noncoding
MSTRG.208769.1	MRPS12	XLOC_216327	802	coding	coding	coding	noncoding
MSTRG.208900.1	ZNF546	XLOC_216393	364	coding	noncoding	noncoding	noncoding
MSTRG.208940.2	PLD3	XLOC_216412	2362	coding	coding	coding	coding
MSTRG.208940.4	PLD3	XLOC_216412	2203	coding	coding	coding	coding
MSTRG.209030.6	LOC105372401	XLOC_216464	5428	coding	coding	coding	coding
MSTRG.209093.2	RPS19	XLOC_216501	748	coding	coding	coding	coding
NM_002659.4	PLAUR	XLOC_216617	1368	coding	coding	coding	coding
MSTRG.209621.4	VASP	XLOC_216750	2481	coding	coding	coding	coding
MSTRG.209621.3	VASP	XLOC_216750	2674	coding	coding	coding	coding
XR_001753637.2	CARD8	XLOC_216904	3046	coding	coding	coding	coding
MSTRG.210125.2	PRMT1	XLOC_217017	1369	coding	coding	coding	coding
XM_011527347.1	FUZ	XLOC_217023	1643	coding	noncoding	coding	coding
MSTRG.210287.4	LOC101928517	XLOC_217111	1346	coding	coding	noncoding	noncoding
MSTRG.210287.3	LOC101928517	XLOC_217111	1394	coding	coding	coding	noncoding
XM_017026246.1	CLDND2	XLOC_217124	2124	coding	noncoding	coding	coding
NR_108100.1	SPACA6P-AS	XLOC_217152	3962	coding	noncoding	coding	noncoding
MSTRG.210365.7	FPR1	XLOC_217161	4578	coding	coding	coding	coding
MSTRG.210365.6	FPR1	XLOC_217161	4734	coding	coding	coding	coding
MSTRG.210365.5	FPR1	XLOC_217161	4798	coding	coding	coding	coding
MSTRG.210365.4	FPR1	XLOC_217161	5818	coding	coding	coding	coding
MSTRG.210534.6	ZNF701	XLOC_217208	1608	coding	coding	noncoding	noncoding
MSTRG.210591.8	ZNF765-ZNF761	XLOC_217251	682	coding	coding	noncoding	noncoding
MSTRG.210599.5	ZNF813	XLOC_217253	2937	coding	coding	coding	noncoding
MSTRG.210516.10	MYADM	XLOC_217262	2244	coding	coding	coding	coding
NM_001289026.2	LAIR1	XLOC_217287	2766	coding	coding	coding	coding
MSTRG.210645.8	LILRA2	XLOC_217295	4464	coding	coding	coding	noncoding
MSTRG.210645.16	LILRA1	XLOC_217295	1615	coding	coding	noncoding	coding
NM_001002836.4	ZNF787	XLOC_217375	1940	coding	coding	coding	coding
NM_001351682.2	ZNF787	XLOC_217375	1702	coding	noncoding	coding	noncoding
MSTRG.211049.1	ZNF274	XLOC_217510	4754	coding	coding	coding	coding
MSTRG.211107.1	ZNF544	XLOC_217512	386	coding	noncoding	noncoding	noncoding
MSTRG.211090.2	ZNF497	XLOC_217521	3626	coding	coding	coding	coding
MSTRG.211237.4	CSNK2A1	XLOC_217588	3773	coding	coding	noncoding	noncoding
MSTRG.211336.1		XLOC_217639	308	coding	noncoding	noncoding	noncoding
MSTRG.211508.3	NSFL1C	XLOC_217667	7718	coding	coding	noncoding	noncoding
MSTRG.211530.4	SIRPB1	XLOC_217669	3170	coding	coding	noncoding	noncoding
MSTRG.211530.9	LOC105372499	XLOC_217669	4695	coding	coding	coding	noncoding
MSTRG.211530.26	LOC107985409	XLOC_217669	9534	coding	coding	coding	noncoding
NR_109859.1	LINC01730	XLOC_217823	706	coding	noncoding	noncoding	noncoding
MSTRG.211836.23	PANK2	XLOC_217828	12547	coding	coding	coding	noncoding
MSTRG.211836.32	PANK2	XLOC_217828	8118	coding	coding	noncoding	noncoding

MSTRG.211789.2	SLC23A2	XLOC_217859	7175	coding	coding	coding	coding
MSTRG.211886.3	GPCPD1	XLOC_217895	11025	coding	coding	coding	noncoding
MSTRG.211886.6	GPCPD1	XLOC_217895	7892	coding	coding	coding	noncoding
MSTRG.211886.14	GPCPD1	XLOC_217895	2892	coding	coding	coding	noncoding
MSTRG.212302.1	JAG1	XLOC_218126	228	coding	noncoding	noncoding	noncoding
NM_018327.4	SPTLC3	XLOC_218201	6191	coding	coding	coding	coding
NM_001317960.1	XRN2	XLOC_218498	3615	coding	coding	coding	coding
MSTRG.213355.16	GZF1	XLOC_218580	11465	coding	coding	coding	noncoding
MSTRG.213355.20	NAPB	XLOC_218580	1233	coding	coding	noncoding	noncoding
MSTRG.213419.1		XLOC_218631	214	coding	noncoding	noncoding	noncoding
XR_001754418.1	ITCH	XLOC_218902	2335	coding	coding	coding	coding
NM_014183.3	DYNLRB1	XLOC_218913	700	coding	coding	coding	noncoding
MSTRG.214008.3	MYH7B	XLOC_218926	9389	coding	coding	coding	coding
MSTRG.214096.2	CPNE1	XLOC_218971	1966	coding	coding	noncoding	noncoding
MSTRG.214096.6	CPNE1	XLOC_218971	1762	coding	coding	noncoding	noncoding
MSTRG.214139.2	SCAND1	XLOC_218990	1843	coding	coding	coding	noncoding
MSTRG.214363.3	TTI1	XLOC_219105	3550	coding	noncoding	noncoding	noncoding
MSTRG.214785.1	ZHX3	XLOC_219277	308	coding	noncoding	noncoding	noncoding
MSTRG.215014.3	CHD6	XLOC_219290	4822	coding	coding	coding	coding
MSTRG.214914.1	TOX2	XLOC_219356	263	coding	noncoding	noncoding	noncoding
MSTRG.215009.2	ADA	XLOC_219408	1356	coding	coding	coding	coding
XM_017028029.2	STK4	XLOC_219423	5470	coding	coding	coding	coding
MSTRG.215375.15	SULF2	XLOC_219565	4012	coding	coding	coding	coding
MSTRG.215485.2	B4GALT5	XLOC_219635	4710	coding	coding	coding	coding
MSTRG.215503.2	B4GALT5	XLOC_219636	1958	coding	noncoding	noncoding	noncoding
MSTRG.215681.4	ADNP-AS1	XLOC_219713	4998	coding	coding	noncoding	noncoding
MSTRG.215681.5	ADNP	XLOC_219713	4830	coding	coding	noncoding	noncoding
MSTRG.215681.8	ADNP	XLOC_219713	5486	coding	coding	noncoding	noncoding
MSTRG.215692.3	DPM1	XLOC_219714	1174	coding	coding	noncoding	noncoding
MSTRG.215780.3	NFATC2	XLOC_219736	7437	coding	coding	coding	noncoding
MSTRG.216009.1	AURKA	XLOC_219886	895	coding	coding	noncoding	noncoding
MSTRG.216009.6	AURKA	XLOC_219886	426	coding	noncoding	noncoding	coding
NM_020356.4	CASS4	XLOC_219889	4195	coding	coding	coding	coding
NM_001291780.2	RBM38	XLOC_219934	2489	coding	coding	coding	coding
MSTRG.216218.8	PRELID3B	XLOC_220016	2220	coding	coding	noncoding	noncoding
MSTRG.216267.1	ZNF831	XLOC_220025	214	coding	noncoding	noncoding	noncoding
MSTRG.216294.1	SYCP2	XLOC_220064	472	coding	noncoding	noncoding	noncoding
XR_936990.3	LOC105372717	XLOC_220235	1656	coding	noncoding	coding	noncoding
MSTRG.216751.3	ARFRP1	XLOC_220284	890	coding	coding	coding	noncoding
MSTRG.211341.2	PSMF1	XLOC_220407	3335	coding	coding	coding	coding
NR_037661.1	FKBP1A-SDCBP2	XLOC_220436	1689	coding	coding	coding	coding
MSTRG.211535.1	LOC105372499	XLOC_220485	234	coding	coding	noncoding	noncoding

XR_001754121.1	SIRPB1	XLOC_220504	1462	coding	coding	coding	coding
MSTRG.211645.2	LOC102724545	XLOC_220552	6854	coding	coding	coding	coding
NM_001258430.2	C20orf27	XLOC_220625	1706	coding	coding	coding	coding
MSTRG.211726.2	CDC25B	XLOC_220628	2852	coding	coding	coding	coding
MSTRG.211758.4	SMOX	XLOC_220640	2292	coding	coding	coding	noncoding
MSTRG.211769.5	PRNP	XLOC_220650	2664	coding	coding	coding	coding
MSTRG.211805.5	CDS2	XLOC_220667	3383	coding	coding	coding	noncoding
MSTRG.211881.6	SHLD1	XLOC_220722	585	coding	coding	noncoding	noncoding
MSTRG.212397.10	PLCB1	XLOC_220791	11982	coding	noncoding	coding	noncoding
MSTRG.212262.5	SLX4IP	XLOC_220837	3616	coding	coding	noncoding	noncoding
MSTRG.212997.1	SLC24A3	XLOC_221215	665	coding	noncoding	noncoding	noncoding
MSTRG.213655.1	FRG1BP	XLOC_221702	439	coding	coding	noncoding	noncoding
MSTRG.213683.1	HM13	XLOC_221725	3783	coding	coding	coding	coding
MSTRG.213683.2	HM13	XLOC_221725	2306	coding	coding	coding	coding
NM_002657.3	PLAGL2	XLOC_221756	5656	coding	coding	coding	coding
MSTRG.213836.3	ASXL1	XLOC_221764	6735	coding	coding	noncoding	coding
MSTRG.213800.2	MAPRE1	XLOC_221779	7845	coding	coding	noncoding	noncoding
MSTRG.213949.4	DYNLRB1	XLOC_221851	664	coding	coding	noncoding	noncoding
MSTRG.214003.2	ACSS2	XLOC_221880	2856	coding	coding	coding	noncoding
NM_001242600.2	RBM39	XLOC_221948	4976	coding	coding	coding	coding
XM_017028141.2	RBM39	XLOC_221948	4339	coding	coding	coding	coding
MSTRG.214204.1	DLGAP4	XLOC_221974	3026	coding	coding	coding	noncoding
MSTRG.214250.3	MROH8	XLOC_222008	4128	coding	coding	coding	coding
MSTRG.214368.2	RPRD1B	XLOC_222045	1471	coding	coding	coding	noncoding
MSTRG.215116.3	YWHAB	XLOC_222434	2970	coding	coding	coding	noncoding
MSTRG.215142.2	STK4	XLOC_222454	7437	coding	coding	coding	noncoding
MSTRG.215142.5	STK4	XLOC_222454	21131	coding	coding	noncoding	noncoding
MSTRG.215142.19	STK4	XLOC_222454	6841	coding	coding	coding	noncoding
MSTRG.215053.12	LINC02597	XLOC_222466	3768	coding	noncoding	noncoding	noncoding
MSTRG.215180.2	ZSWIM1	XLOC_222513	2094	coding	coding	coding	noncoding
NM_182764.2	ELMO2	XLOC_222537	4003	coding	coding	coding	coding
NM_001282578.3	UBE2V1	XLOC_222743	2299	coding	coding	coding	noncoding
NR_047554.1	UBE2V1	XLOC_222743	2009	coding	coding	noncoding	noncoding
MSTRG.216018.2	CASS4	XLOC_222967	4172	coding	coding	coding	noncoding
MSTRG.216092.5	RAB22A	XLOC_223042	2413	coding	noncoding	noncoding	noncoding
MSTRG.216127.3	VAPB	XLOC_223046	2339	coding	coding	coding	noncoding
MSTRG.216136.5	STX16	XLOC_223060	3856	coding	coding	coding	noncoding
MSTRG.216136.4	STX16	XLOC_223060	4685	coding	coding	noncoding	noncoding
MSTRG.216136.10	STX16	XLOC_223060	8587	coding	coding	noncoding	noncoding
MSTRG.216261.7	ZNF831	XLOC_223075	6774	coding	noncoding	coding	noncoding
MSTRG.216510.2	LOC105372707	XLOC_223252	1384	coding	coding	coding	coding
MSTRG.216510.3	LOC105372707	XLOC_223252	1231	coding	coding	coding	coding

MSTRG.216696.13	GID8	XLOC_223286	1562	coding	coding	coding	coding
MSTRG.216804.1		XLOC_223386	325	coding	noncoding	noncoding	noncoding
MSTRG.216971.2	RNA45SN1	XLOC_223443	2422	coding	noncoding	coding	noncoding
MSTRG.217062.1		XLOC_223534	483	coding	noncoding	noncoding	noncoding
MSTRG.217036.1		XLOC_223555	285	coding	noncoding	noncoding	noncoding
MSTRG.217040.2	ANKRD20A11P	XLOC_223558	434	coding	noncoding	noncoding	noncoding
MSTRG.217514.2	CHODL	XLOC_223708	2879	coding	coding	noncoding	noncoding
MSTRG.217793.4	GABPA	XLOC_223871	5151	coding	coding	coding	coding
MSTRG.217793.7	GABPA	XLOC_223871	4776	coding	coding	coding	coding
MSTRG.218244.1		XLOC_223964	243	coding	noncoding	noncoding	noncoding
MSTRG.218332.1	N6AMT1	XLOC_224039	711	coding	noncoding	noncoding	noncoding
MSTRG.218384.1	N6AMT1	XLOC_224085	1784	coding	coding	noncoding	coding
MSTRG.217938.8	LTN1	XLOC_224111	2275	coding	noncoding	noncoding	noncoding
NM_001032410.1	USP16	XLOC_224117	3038	coding	coding	coding	coding
XM_017028420.1	EVA1C	XLOC_224298	1981	coding	coding	coding	coding
MSTRG.218512.7	PAXBP1-AS1	XLOC_224347	4252	coding	coding	coding	noncoding
MSTRG.218708.1	ITSN1	XLOC_224466	363	coding	noncoding	noncoding	noncoding
MSTRG.218952.12	RUNX1	XLOC_224522	2035	coding	noncoding	coding	noncoding
MSTRG.218952.22	RUNX1	XLOC_224522	18732	coding	noncoding	coding	noncoding
NM_001320446.1	MORC3	XLOC_224574	4332	coding	coding	coding	coding
MSTRG.219090.1	LOC105372795	XLOC_224606	425	coding	noncoding	noncoding	noncoding
NM_001320704.2	TTC3	XLOC_224608	7896	coding	coding	coding	coding
NM_001320703.2	TTC3	XLOC_224608	7906	coding	coding	coding	coding
NM_005239.6	ETS2	XLOC_224700	3668	coding	coding	coding	coding
NM_058186.4	FAM3B	XLOC_224779	1317	coding	coding	coding	coding
MSTRG.219624.4	U2AF1	XLOC_224901	1706	coding	coding	noncoding	noncoding
MSTRG.219807.2	UBE2G2	XLOC_225010	3815	coding	coding	coding	noncoding
MSTRG.219818.2	PTTG1IP	XLOC_225014	2357	coding	noncoding	coding	noncoding
XM_011529451.1	COL6A2	XLOC_225094	3730	coding	coding	coding	coding
MSTRG.216824.1	LOC101928576	XLOC_225144	282	coding	noncoding	noncoding	noncoding
MSTRG.216965.11	LOC107987293	XLOC_225181	1133	coding	noncoding	coding	noncoding
MSTRG.216965.16	LOC105379508	XLOC_225181	4329	coding	noncoding	coding	noncoding
MSTRG.216965.19	LOC105379508	XLOC_225181	3156	coding	noncoding	coding	noncoding
MSTRG.216965.30	LOC105379508	XLOC_225181	3870	coding	noncoding	coding	noncoding
MSTRG.216965.32	LOC105379508	XLOC_225181	2709	coding	noncoding	coding	noncoding
MSTRG.216986.1	TPTE	XLOC_225228	275	coding	noncoding	noncoding	noncoding
MSTRG.217117.2	ABCC13	XLOC_225288	1737	coding	coding	coding	coding
MSTRG.217117.4	ABCC13	XLOC_225288	2131	coding	coding	coding	noncoding
MSTRG.217117.5	ABCC13	XLOC_225288	2330	coding	coding	coding	noncoding
MSTRG.217326.12	USP25	XLOC_225364	1822	coding	coding	coding	noncoding
MSTRG.217792.12	GABPA	XLOC_225673	2067	coding	coding	noncoding	noncoding
MSTRG.217952.3	USP16	XLOC_225804	2726	coding	coding	noncoding	noncoding

MSTRG.218562.14	BACH1	XLOC_225815	6382	coding	coding	coding	coding
MSTRG.218562.18	BACH1	XLOC_225815	5039	coding	coding	coding	coding
MSTRG.218541.1		XLOC_226012	407	coding	noncoding	noncoding	noncoding
MSTRG.218671.7	LOC107985481	XLOC_226022	10151	coding	coding	coding	coding
MSTRG.218671.5	LOC107985481	XLOC_226022	9223	coding	coding	coding	coding
MSTRG.218704.5	ITSN1	XLOC_226037	1516	coding	coding	noncoding	noncoding
MSTRG.218941.6	MORC3	XLOC_226216	4319	coding	coding	noncoding	noncoding
MSTRG.219094.3	TTC3-AS1	XLOC_226244	7706	coding	coding	noncoding	coding
MSTRG.219229.1	LOC105372801	XLOC_226289	32407	coding	coding	coding	coding
MSTRG.219229.12	LOC105372801	XLOC_226289	32568	coding	noncoding	coding	coding
MSTRG.219229.19	LOC105372801	XLOC_226289	37333	coding	coding	coding	coding
MSTRG.219229.22	LOC105372801	XLOC_226289	29538	coding	noncoding	coding	coding
MSTRG.219229.21	LOC105372801	XLOC_226289	29738	coding	noncoding	coding	coding
MSTRG.219396.1	FAM3B	XLOC_226420	819	coding	coding	noncoding	noncoding
MSTRG.219426.2	MX1	XLOC_226424	3008	coding	coding	coding	noncoding
MSTRG.219493.3	ABCG1	XLOC_226465	3907	coding	coding	coding	noncoding
MSTRG.219493.4	ABCG1	XLOC_226465	4722	coding	coding	coding	noncoding
MSTRG.219509.3	UBASH3A	XLOC_226473	2252	coding	coding	coding	coding
MSTRG.219753.1	TRPM2	XLOC_226599	284	coding	noncoding	noncoding	noncoding
MSTRG.219974.1	PCBP3	XLOC_226709	255	coding	noncoding	noncoding	noncoding
MSTRG.220193.7	MCM3AP	XLOC_226766	409	coding	coding	noncoding	noncoding
MSTRG.220219.5	DIP2A	XLOC_226782	1663	coding	coding	coding	noncoding
MSTRG.220571.2	KLHL22	XLOC_227041	2471	coding	coding	coding	coding
NR_028436.2	UBE2L3	XLOC_227080	3137	coding	coding	coding	noncoding
XM_005261448.4	PPIL2	XLOC_227090	3058	coding	coding	coding	coding
MSTRG.220671.5	MAPK1	XLOC_227096	8144	coding	coding	coding	noncoding
NM_002073.4	GNAZ	XLOC_227155	3170	coding	coding	coding	coding
MSTRG.221042.3	HPS4	XLOC_227329	1388	coding	noncoding	coding	coding
NM_001318371.2	SLC35E4	XLOC_227591	2775	coding	coding	coding	coding
MSTRG.221629.7	SLC35E4	XLOC_227591	1221	coding	coding	coding	noncoding
MSTRG.221697.2	PATZ1	XLOC_227623	2541	coding	coding	coding	coding
MSTRG.221744.4	EIF4ENIF1	XLOC_227632	2404	coding	coding	noncoding	noncoding
MSTRG.221707.1	PRR14L	XLOC_227634	10886	coding	coding	coding	noncoding
MSTRG.221707.5	PRR14L	XLOC_227634	11403	coding	coding	noncoding	noncoding
XM_011530392.3	APOL6	XLOC_227804	10314	coding	coding	coding	coding
MSTRG.222202.2	RAC2	XLOC_227890	1474	coding	coding	coding	noncoding
MSTRG.222328.3	PLA2G6	XLOC_227952	590	coding	noncoding	noncoding	noncoding
NM_001161572.1	MAFF	XLOC_227954	2466	coding	coding	coding	coding
MSTRG.222458.3	RPL3	XLOC_228013	2270	coding	coding	coding	coding
NM_182810.2	ATF4	XLOC_228026	1420	coding	coding	coding	coding
MSTRG.222766.1	CENPM	XLOC_228195	349	coding	coding	noncoding	noncoding
MSTRG.222767.1	CENPM	XLOC_228196	256	coding	coding	noncoding	noncoding

MSTRG.222778.5	WBP2NL	XLOC_228199	8258	coding	coding	coding	coding
XM_005261312.1	NUP50	XLOC_228348	5275	coding	coding	coding	coding
MSTRG.223296.4	CERK	XLOC_228438	1219	coding	noncoding	noncoding	noncoding
MSTRG.223334.1	LINC01644	XLOC_228504	398	coding	noncoding	noncoding	noncoding
NM_014838.3	ZBED4	XLOC_228584	6893	coding	coding	coding	coding
XR_001755232.1	NCAPH2	XLOC_228637	3431	coding	coding	coding	coding
MSTRG.223632.14	TYMP	XLOC_228637	1729	coding	coding	coding	noncoding
MSTRG.223632.18	TYMP	XLOC_228637	1838	coding	coding	coding	coding
MSTRG.220313.1	USP18	XLOC_228785	1933	coding	coding	noncoding	coding
MSTRG.220543.1	ZNF74	XLOC_228911	213	coding	noncoding	noncoding	noncoding
MSTRG.220641.1	AIFM3	XLOC_228945	399	coding	noncoding	noncoding	noncoding
MSTRG.220652.2	UBE2L3	XLOC_228968	2861	coding	coding	coding	noncoding
MSTRG.220966.3	IGLVI-70	XLOC_229023	675	coding	coding	noncoding	noncoding
MSTRG.220966.22	IGLVI-70	XLOC_229023	869	coding	coding	noncoding	noncoding
MSTRG.220966.36	LOC102724638	XLOC_229023	879	coding	coding	noncoding	noncoding
MSTRG.220966.39	IGLL5	XLOC_229023	898	coding	coding	coding	noncoding
MSTRG.220890.3	ADORA2A-AS1	XLOC_229092	2085	coding	coding	coding	coding
MSTRG.221474.16	EWSR1	XLOC_229390	2341	coding	coding	coding	noncoding
MSTRG.221532.7	MTMR3	XLOC_229425	3115	coding	noncoding	coding	noncoding
MSTRG.221532.9	MTMR3	XLOC_229425	1016	coding	coding	coding	noncoding
MSTRG.221600.3	SEC14L3	XLOC_229464	2237	coding	noncoding	coding	noncoding
MSTRG.221600.2	SEC14L3	XLOC_229464	1991	coding	noncoding	coding	noncoding
MSTRG.221679.2	RNF185	XLOC_229495	3062	coding	coding	noncoding	noncoding
MSTRG.221992.2	TOM1	XLOC_229646	217	coding	coding	noncoding	noncoding
MSTRG.222026.2	APOL6	XLOC_229668	3338	coding	noncoding	noncoding	noncoding
MSTRG.222149.1		XLOC_229739	354	coding	noncoding	noncoding	noncoding
MSTRG.222277.2	TRIOBP	XLOC_229798	2680	coding	coding	coding	coding
MSTRG.222445.3	APOBEC3A	XLOC_229876	1574	coding	coding	noncoding	noncoding
MSTRG.222445.10	APOBEC3B-AS1	XLOC_229876	1859	coding	coding	coding	coding
MSTRG.222491.1	MIEF1	XLOC_229910	6584	coding	coding	coding	coding
NR_130151.1	RPS19BP1	XLOC_229912	1018	coding	coding	coding	noncoding
MSTRG.222620.19	TNRC6B	XLOC_229943	16251	coding	coding	noncoding	noncoding
MSTRG.222600.6	EP300	XLOC_229995	8526	coding	coding	coding	coding
MSTRG.223159.3	ATXN10	XLOC_230248	1469	coding	noncoding	noncoding	noncoding
MSTRG.223452.5	ZBED4	XLOC_230443	8986	coding	coding	coding	coding
MSTRG.223595.3	TRABD	XLOC_230464	2276	coding	coding	coding	coding
MSTRG.223622.4	PPP6R2	XLOC_230478	3743	coding	coding	coding	coding
MSTRG.223737.2	ZBED1	XLOC_230553	4375	coding	coding	coding	coding
NM_001139466.1	TBL1X	XLOC_230728	5596	coding	coding	coding	coding
XM_017029404.2	AMELX	XLOC_230831	6915	coding	coding	coding	coding
MSTRG.224511.1	GLRA2	XLOC_230939	315	coding	coding	noncoding	noncoding
XM_005274625.3	REPS2	XLOC_231035	7731	coding	coding	coding	coding

MSTRG.224827.1	MAP3K15	XLOC_231145	246	coding	coding	noncoding	noncoding
MSTRG.224929.6	RPS6KA3	XLOC_231167	4777	coding	coding	coding	noncoding
NM_006406.2	PRDX4	XLOC_231245	956	coding	coding	coding	coding
MSTRG.225267.2	PCYT1B	XLOC_231286	2199	coding	coding	coding	noncoding
MSTRG.225587.1		XLOC_231505	271	coding	noncoding	noncoding	noncoding
MSTRG.225852.2	RPGR	XLOC_231607	2472	coding	coding	coding	noncoding
MSTRG.225852.4	RPGR	XLOC_231607	2499	coding	coding	coding	noncoding
MSTRG.225852.3	RPGR	XLOC_231607	2166	coding	coding	coding	noncoding
MSTRG.226093.2	CXorf38	XLOC_231733	4032	coding	coding	coding	noncoding
NR_024616.1	INE1	XLOC_231995	1014	coding	noncoding	noncoding	noncoding
MSTRG.227096.1		XLOC_232218	255	coding	noncoding	noncoding	noncoding
MSTRG.227211.7	KDM5C	XLOC_232339	3124	coding	coding	coding	coding
MSTRG.227194.2	HSD17B10	XLOC_232357	1038	coding	coding	noncoding	coding
MSTRG.227340.14	HUWE1	XLOC_232361	6700	coding	coding	coding	coding
MSTRG.227388.7	APEX2	XLOC_232424	2006	coding	coding	coding	coding
MSTRG.227481.1		XLOC_232471	226	coding	noncoding	noncoding	noncoding
MSTRG.227745.1		XLOC_232632	357	coding	noncoding	noncoding	noncoding
MSTRG.227998.1	MTMR8	XLOC_232703	1192	coding	noncoding	noncoding	noncoding
MSTRG.228002.1	MTMR8	XLOC_232706	1310	coding	noncoding	noncoding	noncoding
MSTRG.228591.2	LAS1L	XLOC_232759	2265	coding	coding	noncoding	noncoding
XM_017029546.1	MSN	XLOC_232760	4010	coding	coding	coding	coding
MSTRG.228184.1		XLOC_232784	303	coding	noncoding	noncoding	noncoding
MSTRG.228120.1	OPHN1	XLOC_232860	359	coding	coding	noncoding	noncoding
MSTRG.228434.1	TEX11	XLOC_232947	348	coding	noncoding	noncoding	noncoding
XM_006724663.4	NLGN3	XLOC_232970	3328	coding	coding	coding	coding
MSTRG.228609.2	ZMYM3	XLOC_232985	1121	coding	noncoding	coding	coding
NR_104396.2	TAF1	XLOC_232992	1545	coding	noncoding	coding	coding
MSTRG.228803.4	HDAC8	XLOC_233049	619	coding	coding	coding	noncoding
MSTRG.228826.1	MAP2K4P1	XLOC_233075	922	coding	coding	noncoding	noncoding
MSTRG.229009.1	JPX	XLOC_233123	232	coding	noncoding	noncoding	noncoding
MSTRG.229017.1	FTX	XLOC_233125	231	coding	noncoding	noncoding	noncoding
MSTRG.229067.1	FTX	XLOC_233167	296	coding	noncoding	noncoding	noncoding
MSTRG.229077.3	FTX	XLOC_233176	2897	coding	noncoding	coding	noncoding
MSTRG.229077.9	FTX	XLOC_233176	1877	coding	noncoding	coding	noncoding
MSTRG.229077.20	FTX	XLOC_233176	25635	coding	coding	coding	noncoding
MSTRG.229077.22	FTX	XLOC_233176	52304	coding	noncoding	coding	noncoding
MSTRG.229077.27	FTX	XLOC_233176	3556	coding	noncoding	noncoding	noncoding
MSTRG.229134.3	ATRX	XLOC_233289	8797	coding	coding	noncoding	coding
MSTRG.229134.7	ATRX	XLOC_233289	4962	coding	coding	noncoding	coding
MSTRG.229134.10	ATRX	XLOC_233289	1304	coding	noncoding	noncoding	noncoding
MSTRG.229128.1	MAGT1	XLOC_233295	5419	coding	coding	noncoding	noncoding
MSTRG.229612.1		XLOC_233461	473	coding	noncoding	noncoding	noncoding

MSTRG.230782.1	MORC4	XLOC_234083	321	coding	noncoding	noncoding	noncoding
MSTRG.230819.5	RBM41	XLOC_234101	599	coding	coding	noncoding	noncoding
XM_017029855.1	ALG13	XLOC_234271	3561	coding	noncoding	coding	coding
MSTRG.231249.1		XLOC_234400	387	coding	noncoding	noncoding	noncoding
MSTRG.231396.1	KLHL13	XLOC_234428	314	coding	coding	noncoding	noncoding
XM_005262369.5	DOCK11	XLOC_234456	6603	coding	coding	coding	coding
MSTRG.231552.2	NKAP	XLOC_234517	948	coding	coding	noncoding	coding
MSTRG.231604.1		XLOC_234546	616	coding	noncoding	noncoding	noncoding
MSTRG.231611.1	NKAPP1	XLOC_234552	716	coding	noncoding	coding	noncoding
MSTRG.231619.3	LAMP2	XLOC_234559	8036	coding	coding	coding	noncoding
MSTRG.231627.1	CUL4B	XLOC_234565	8295	coding	coding	coding	noncoding
MSTRG.231636.2	C1GALT1C1	XLOC_234567	1291	coding	noncoding	noncoding	noncoding
NM_012084.4	GLUD2	XLOC_234568	2485	coding	coding	coding	coding
XM_006724728.4	STAG2	XLOC_234654	5041	coding	coding	coding	coding
NM_001042453.2	STK26	XLOC_234894	3020	coding	coding	coding	coding
MSTRG.232374.7	MBNL3	XLOC_234922	8712	coding	noncoding	coding	noncoding
MSTRG.232432.2	ZNF75D	XLOC_234981	5738	coding	coding	coding	noncoding
MSTRG.232531.2	ARHGEF6	XLOC_235044	5069	coding	coding	noncoding	noncoding
MSTRG.233434.2	BCAP31	XLOC_235525	1465	coding	coding	coding	coding
MSTRG.233532.2	SLC10A3	XLOC_235573	2012	coding	coding	coding	coding
MSTRG.233827.12	TMLHE	XLOC_235625	1538	coding	coding	coding	noncoding
MSTRG.223693.3	CSF2RA	XLOC_235638	2103	coding	coding	coding	noncoding
MSTRG.224614.1	CLTRN	XLOC_236052	4937	coding	coding	coding	coding
MSTRG.225067.1	CNKSR2	XLOC_236336	597	coding	coding	noncoding	noncoding
NM_153270.3	KLHL34	XLOC_236370	3641	coding	coding	coding	coding
MSTRG.225222.1		XLOC_236441	382	coding	noncoding	noncoding	noncoding
MSTRG.225248.8	ZFX-AS1	XLOC_236446	7673	coding	coding	noncoding	noncoding
MSTRG.225248.10	ZFX	XLOC_236446	3015	coding	noncoding	noncoding	noncoding
MSTRG.225248.11	ZFX	XLOC_236446	6915	coding	coding	coding	noncoding
MSTRG.225257.2	PDK3	XLOC_236465	2867	coding	coding	coding	noncoding
MSTRG.225386.5	POLA1	XLOC_236469	1452	coding	coding	coding	noncoding
MSTRG.225545.12	GK	XLOC_236586	682	coding	coding	noncoding	noncoding
MSTRG.225545.20	GK	XLOC_236586	2867	coding	coding	noncoding	noncoding
MSTRG.225795.4	CYBB	XLOC_236716	7576	coding	coding	coding	coding
MSTRG.225894.1		XLOC_236778	718	coding	noncoding	noncoding	noncoding
NM_144970.3	CXorf38	XLOC_236860	4244	coding	coding	coding	coding
MSTRG.226428.11	KDM6A	XLOC_237010	4816	coding	coding	coding	noncoding
MSTRG.226630.1	ZNF674	XLOC_237115	246	coding	noncoding	noncoding	noncoding
MSTRG.226655.1	UBA1	XLOC_237154	3510	coding	coding	coding	coding
MSTRG.226699.3	ZNF81	XLOC_237186	11478	coding	coding	coding	noncoding
MSTRG.226704.1		XLOC_237187	376	coding	noncoding	noncoding	noncoding
MSTRG.227599.1	KLF8	XLOC_237572	871	coding	coding	noncoding	noncoding

MSTRG.227639.1	KLF8	XLOC_237605	404	coding	noncoding	noncoding	noncoding
XR_001755692.1	SPIN2B	XLOC_237666	1454	coding	coding	noncoding	coding
MSTRG.227810.1	FAAH2	XLOC_237671	1493	coding	noncoding	noncoding	noncoding
MSTRG.227824.1	FAAH2	XLOC_237681	1281	coding	coding	coding	coding
MSTRG.227856.1	FAAH2	XLOC_237710	269	coding	noncoding	noncoding	noncoding
MSTRG.227859.1	FAAH2	XLOC_237712	388	coding	noncoding	noncoding	noncoding
MSTRG.227887.1	FAAH2	XLOC_237730	215	coding	noncoding	noncoding	noncoding
MSTRG.228516.1	ZC3H12B	XLOC_237892	267	coding	noncoding	noncoding	noncoding
MSTRG.228518.1	ZC3H12B	XLOC_237894	645	coding	coding	noncoding	noncoding
MSTRG.228531.1	ZC3H12B	XLOC_237902	202	coding	noncoding	noncoding	noncoding
MSTRG.228544.1	ZC3H12B	XLOC_237913	327	coding	noncoding	noncoding	noncoding
MSTRG.228547.1	ZC3H12B	XLOC_237916	293	coding	noncoding	noncoding	noncoding
MSTRG.228561.1	ZC3H12B	XLOC_237927	212	coding	coding	noncoding	noncoding
MSTRG.228339.1	EDA	XLOC_238108	976	coding	noncoding	noncoding	noncoding
MSTRG.228345.1	EDA	XLOC_238114	984	coding	noncoding	noncoding	noncoding
MSTRG.228385.1	EDA	XLOC_238147	207	coding	noncoding	noncoding	noncoding
MSTRG.228386.1	EDA	XLOC_238148	288	coding	coding	noncoding	noncoding
MSTRG.228393.1	EDA	XLOC_238153	449	coding	noncoding	noncoding	noncoding
MSTRG.228732.10	NHSL2	XLOC_238268	9628	coding	coding	coding	noncoding
MSTRG.229229.1	LOC646127	XLOC_238438	247	coding	noncoding	noncoding	noncoding
MSTRG.229233.1	LOC646127	XLOC_238442	302	coding	noncoding	noncoding	noncoding
XM_006724668.3	ATRX	XLOC_238526	6054	coding	coding	coding	coding
MSTRG.229405.2	LOC107985690	XLOC_238580	9076	coding	coding	noncoding	noncoding
MSTRG.229405.5	LOC107985690	XLOC_238580	8848	coding	coding	noncoding	noncoding
MSTRG.229411.1	LOC107985690	XLOC_238581	587	coding	noncoding	noncoding	noncoding
MSTRG.229385.1		XLOC_238656	567	coding	noncoding	noncoding	noncoding
MSTRG.230469.3	DIAPH2	XLOC_238977	3723	coding	coding	coding	coding
MSTRG.230469.5	DIAPH2	XLOC_238977	2053	coding	coding	coding	noncoding
MSTRG.230307.1	ARMCX4	XLOC_239076	683	coding	noncoding	noncoding	noncoding
MSTRG.230688.1	ARMCX5-GPRASP2	XLOC_239143	287	coding	noncoding	noncoding	noncoding
MSTRG.230700.1	ARMCX5-GPRASP2	XLOC_239153	1136	coding	coding	noncoding	noncoding
MSTRG.230735.1	ARMCX5-GPRASP2	XLOC_239184	503	coding	noncoding	noncoding	noncoding
MSTRG.230958.2	NXT2	XLOC_239422	1742	coding	coding	noncoding	noncoding
MSTRG.231290.9	ALG13	XLOC_239470	711	coding	coding	noncoding	noncoding
MSTRG.231290.12	ALG13	XLOC_239470	699	coding	noncoding	noncoding	noncoding
MSTRG.231388.1		XLOC_239617	282	coding	noncoding	noncoding	noncoding
MSTRG.231864.8	STAG2	XLOC_239804	5840	coding	coding	noncoding	noncoding
MSTRG.232178.1		XLOC_239999	211	coding	coding	noncoding	noncoding
XM_005262441.3	MBNL3	XLOC_240044	8843	coding	noncoding	coding	coding
MSTRG.232413.2	FAM122C	XLOC_240107	705	coding	noncoding	noncoding	noncoding
NM_007131.5	ZNF75D	XLOC_240130	5611	coding	coding	coding	coding
MSTRG.232463.5	INTS6L	XLOC_240141	3571	coding	coding	noncoding	noncoding

MSTRG.233089.5	AFF2	XLOC_240480	1119	coding	coding	noncoding	noncoding
NM_001166550.4	IDS	XLOC_240499	7536	coding	coding	coding	coding
XM_024452426.1	PNMA6E	XLOC_240658	3279	coding	coding	coding	coding
MSTRG.233427.2	SLC6A8	XLOC_240669	3466	coding	coding	coding	coding
XM_005274648.1	GAB3	XLOC_240740	2289	coding	coding	coding	coding
MSTRG.233836.1	TMLHE-AS1	XLOC_240801	302	coding	noncoding	noncoding	noncoding
MSTRG.233681.4	ZBED1	XLOC_240884	4375	coding	coding	coding	coding
MSTRG.234036.14	UTY	XLOC_240989	10218	coding	coding	coding	coding
MSTRG.233966.1	TTTY14	XLOC_241008	619	coding	noncoding	noncoding	noncoding
MSTRG.233990.1	TTTY14	XLOC_241031	325	coding	noncoding	noncoding	noncoding
MSTRG.234004.1	TTTY14	XLOC_241043	236	coding	coding	noncoding	noncoding
MSTRG.233758.2	LINC00278	XLOC_241235	13838	coding	coding	noncoding	noncoding
MSTRG.233758.10	LINC00278	XLOC_241235	12613	coding	noncoding	coding	noncoding
MSTRG.233890.2	USP9Y	XLOC_241311	2297	coding	noncoding	noncoding	noncoding
MSTRG.234179.1		XLOC_241503	256	coding	noncoding	noncoding	noncoding
MSTRG.234321.2	HLA-A	XLOC_241651	1062	coding	coding	coding	coding
MSTRG.234384.6	HLA-B	XLOC_241699	1291	coding	coding	coding	coding
MSTRG.234319.1	HLA-H	XLOC_241858	746	coding	coding	coding	noncoding
MSTRG.234319.2	HLA-A	XLOC_241858	958	coding	coding	noncoding	coding
MSTRG.234319.6	HLA-A	XLOC_241858	935	coding	coding	coding	noncoding
MSTRG.234319.7	HLA-A	XLOC_241858	8036	coding	coding	coding	coding
MSTRG.234453.1	RNF5	XLOC_241953	770	coding	noncoding	noncoding	noncoding
NR_003287.4	RNA28SN5	XLOC_242059	5070	coding	noncoding	coding	noncoding
MSTRG.234635.11	RNA45SN5	XLOC_242059	2874	coding	noncoding	coding	noncoding
MSTRG.234635.13	RNA45SN5	XLOC_242059	3775	coding	noncoding	coding	coding
MSTRG.234635.18	RNA45SN5	XLOC_242059	4995	coding	noncoding	coding	noncoding
MSTRG.234635.19	RNA45SN5	XLOC_242059	4623	coding	noncoding	coding	noncoding
MSTRG.234634.13	LOC100507412	XLOC_242063	2757	coding	noncoding	coding	noncoding
MSTRG.234634.21	LOC112268313	XLOC_242063	3360	coding	noncoding	coding	noncoding
MSTRG.234645.2	HLA-A	XLOC_242242	714	coding	coding	noncoding	noncoding
MSTRG.234645.4	HLA-A	XLOC_242242	907	coding	coding	coding	noncoding
MSTRG.235008.1	HLA-C	XLOC_242734	1568	coding	coding	noncoding	coding
MSTRG.235008.9	HLA-C	XLOC_242734	913	coding	coding	coding	noncoding
NR_073072.1_4	NRM	XLOC_242878	1224	coding	noncoding	coding	coding
MSTRG.235179.1	HLA-C	XLOC_243033	1828	coding	coding	coding	coding
MSTRG.235179.3	HLA-C	XLOC_243033	2090	coding	coding	coding	coding
MSTRG.235179.4	HLA-C	XLOC_243033	1546	coding	coding	coding	coding
MSTRG.235179.7	HLA-C	XLOC_243033	1551	coding	coding	coding	coding
MSTRG.235179.12	HLA-B	XLOC_243033	1670	coding	coding	coding	coding
NR_073072.1_5	NRM	XLOC_243196	1224	coding	noncoding	coding	coding
MSTRG.235182.1	HLA-C	XLOC_243213	2092	coding	coding	coding	coding
MSTRG.235174.1	MICB	XLOC_243219	345	coding	coding	noncoding	noncoding

MSTRG.235411.2	HLA-C	XLOC_243395	895	coding	coding	coding	noncoding
MSTRG.235411.4	HLA-C	XLOC_243395	1488	coding	coding	coding	coding
MSTRG.235442.1	LY6G5C	XLOC_243426	380	coding	noncoding	noncoding	noncoding
NM_001363515.1_6	DHX16	XLOC_243560	4137	coding	coding	coding	coding
MSTRG.235607.1	TRIM10	XLOC_243721	1748	coding	noncoding	noncoding	noncoding
NM_001161376.2_6	C6orf136	XLOC_243741	1882	coding	coding	coding	coding
MSTRG.235656.1	HLA-C	XLOC_243759	1555	coding	coding	coding	noncoding
MSTRG.235656.2	HLA-C	XLOC_243759	1449	coding	coding	coding	coding
MSTRG.235656.5	HLA-B	XLOC_243759	526	coding	noncoding	noncoding	noncoding
MSTRG.235690.1	LY6G5C	XLOC_243793	380	coding	noncoding	noncoding	noncoding
MSTRG.235749.2	HLA-DRB1	XLOC_243823	1171	coding	coding	coding	noncoding
MSTRG.235749.7	HLA-DRB1	XLOC_243823	794	coding	coding	coding	noncoding
MSTRG.235598.2	HLA-H	XLOC_243903	524	coding	coding	noncoding	noncoding
MSTRG.235598.4	HLA-A	XLOC_243903	1741	coding	coding	noncoding	noncoding
MSTRG.235657.3	HLA-C	XLOC_243934	603	coding	coding	coding	noncoding
MSTRG.235724.1	SKIV2L	XLOC_243975	230	coding	noncoding	noncoding	noncoding
NM_001243962.1_5	HLA-DQB1	XLOC_243995	1641	coding	coding	coding	coding
MSTRG.235825.7	YTHDC1	XLOC_244027	3796	coding	coding	coding	noncoding
MSTRG.235825.8	YTHDC1	XLOC_244027	3874	coding	coding	coding	noncoding
MSTRG.236074.5	LOC105379549	XLOC_244298	6296	coding	noncoding	coding	noncoding
MSTRG.236074.12	RNA45SN4	XLOC_244298	4659	coding	noncoding	coding	noncoding
MSTRG.236074.9	LOC105379549	XLOC_244298	3056	coding	noncoding	coding	noncoding
MSTRG.236361.2	CEP170	XLOC_244582	5162	coding	coding	noncoding	coding
MSTRG.236361.3	CEP170	XLOC_244582	7975	coding	coding	noncoding	noncoding
MSTRG.236329.1		XLOC_244725	348	coding	noncoding	noncoding	noncoding
MSTRG.236498.1	FAM120B	XLOC_244873	209	coding	noncoding	noncoding	noncoding
MSTRG.236512.4	THEMIS	XLOC_244901	4430	coding	coding	noncoding	noncoding
NR_125849.1_1	LOC101928140	XLOC_244906	1818	coding	noncoding	noncoding	noncoding
MSTRG.236740.52	TRBV1	XLOC_245023	355	coding	coding	coding	noncoding
MSTRG.236740.69	TRBV1	XLOC_245023	530	coding	noncoding	noncoding	noncoding
MSTRG.236617.6	MGAM	XLOC_245105	4212	coding	coding	coding	coding
MSTRG.236856.22	TRBV1	XLOC_245152	744	coding	coding	noncoding	noncoding
MSTRG.236667.2	TSTA3	XLOC_245215	909	coding	coding	coding	coding
NM_003313.4_1	TSTA3	XLOC_245232	1345	coding	coding	coding	coding
MSTRG.236687.3	LY6E	XLOC_245254	1131	coding	coding	coding	noncoding
MSTRG.236908.7	NAP1L4	XLOC_245388	2682	coding	coding	noncoding	coding
MSTRG.236908.11	NAP1L4	XLOC_245388	2429	coding	coding	noncoding	noncoding
MSTRG.236906.1	SLC22A18AS	XLOC_245397	3018	coding	coding	coding	coding
MSTRG.236918.1	RNH1	XLOC_245409	1925	coding	coding	coding	coding
MSTRG.237073.8	IGHA2	XLOC_245514	1262	coding	coding	coding	coding
MSTRG.237106.2	IGHA2	XLOC_245539	523	coding	coding	noncoding	noncoding
MSTRG.237021.1	ASB2	XLOC_245583	265	coding	coding	noncoding	noncoding

MSTRG.237039.4	SERPINA1	XLOC_245595	621	coding	coding	coding	noncoding
MSTRG.237029.1	IFI27	XLOC_245613	518	coding	coding	coding	coding
MSTRG.237029.5	IFI27	XLOC_245613	401	coding	coding	noncoding	noncoding
NM_001127701.1_1	SERPINA1	XLOC_245620	3532	coding	coding	coding	coding
MSTRG.237230.3	MARF1	XLOC_245741	8688	coding	coding	coding	noncoding
MSTRG.237230.11	MARF1	XLOC_245741	4075	coding	coding	coding	noncoding
MSTRG.237230.14	MARF1	XLOC_245741	3148	coding	coding	coding	noncoding
MSTRG.237184.3	NTAN1	XLOC_245778	1016	coding	coding	noncoding	noncoding
NM_001004431.3_1	METRNL	XLOC_245867	1689	coding	noncoding	coding	coding
NM_001281433.1_1	ZNHIT3	XLOC_245909	925	coding	coding	coding	noncoding
MSTRG.237495.1	ELANE	XLOC_246101	828	coding	coding	coding	noncoding
MSTRG.237660.3	NAIP	XLOC_246393	882	coding	coding	noncoding	noncoding
MSTRG.237618.5	LOC105379623	XLOC_246412	5522	coding	coding	coding	noncoding
MSTRG.237638.1	SMN2	XLOC_246414	910	coding	coding	noncoding	coding
MSTRG.237699.3	LOC105375112	XLOC_246442	10805	coding	noncoding	coding	coding
MSTRG.237699.4	LOC105375112	XLOC_246442	11280	coding	noncoding	noncoding	coding
MSTRG.237699.12	LOC105375112	XLOC_246442	6897	coding	noncoding	coding	coding
MSTRG.237742.1	PRH1-PRR4	XLOC_246480	235	coding	noncoding	noncoding	noncoding
NM_176888.2_2	TAS2R19	XLOC_246516	1002	coding	coding	coding	coding
NM_014967.5_2	FAN1	XLOC_246587	4849	coding	coding	coding	coding
NM_015443.3_2	KANSL1	XLOC_246756	5051	coding	coding	coding	coding
MSTRG.237935.1		XLOC_246785	291	coding	noncoding	noncoding	noncoding
MSTRG.237966.4	LOC101927752	XLOC_246919	1061	coding	noncoding	coding	noncoding
MSTRG.238020.9	LILRB2	XLOC_247049	2185	coding	coding	coding	noncoding
NM_001289026.2_4	LAIR1	XLOC_247099	2766	coding	coding	coding	coding
MSTRG.238045.3	LILRA2	XLOC_247108	3236	coding	coding	coding	noncoding
MSTRG.238045.12	LILRA2	XLOC_247108	2161	coding	coding	noncoding	noncoding
MSTRG.238049.6	LILRA1	XLOC_247110	4341	coding	coding	coding	coding
MSTRG.238056.2	SLC27A3	XLOC_247132	1675	coding	coding	coding	coding
MSTRG.238123.6	NAIP	XLOC_247265	5356	coding	coding	noncoding	noncoding
MSTRG.238256.2	8-Mar	XLOC_247349	5482	coding	coding	coding	coding
MSTRG.238256.3	8-Mar	XLOC_247349	9928	coding	coding	coding	coding
MSTRG.238327.1		XLOC_247469	291	coding	noncoding	noncoding	noncoding
MSTRG.238346.1		XLOC_247489	528	coding	noncoding	noncoding	noncoding
MSTRG.238349.3	LSM14A	XLOC_247496	1754	coding	coding	coding	noncoding
MSTRG.238349.5	LSM14A	XLOC_247496	5701	coding	coding	coding	noncoding
MSTRG.238354.2	KIAA0355	XLOC_247498	1160	coding	noncoding	noncoding	noncoding
NR_027873.1_1	PAXBP1	XLOC_247518	671	coding	noncoding	coding	noncoding
NM_001329128.1_1	IFNGR2	XLOC_247527	1560	coding	coding	coding	coding
MSTRG.238397.5	APOBEC3A_B	XLOC_247554	1162	coding	coding	noncoding	noncoding
MSTRG.238397.8	APOBEC3A_B	XLOC_247554	1727	coding	coding	coding	noncoding
MSTRG.238454.1	PRH1-PRR4	XLOC_247581	235	coding	noncoding	noncoding	noncoding

MSTRG.238471.2	PRH1-PRR4	XLOC_247597	842	coding	noncoding	noncoding	noncoding
MSTRG.238494.1	PRH1-PRR4	XLOC_247608	518	coding	coding	noncoding	noncoding
NM_001278428.3_1	LILRB4	XLOC_247659	3902	coding	coding	coding	coding
MSTRG.238505.4	LILRA2	XLOC_247694	2909	coding	coding	coding	coding
MSTRG.238505.8	LILRA2	XLOC_247694	3090	coding	coding	coding	noncoding
MSTRG.238505.12	LILRA2	XLOC_247694	4579	coding	coding	coding	coding
MSTRG.238505.15	LILRA2	XLOC_247694	4278	coding	coding	coding	noncoding
MSTRG.238528.1	LAIR1	XLOC_247712	858	coding	coding	noncoding	noncoding
NM_001289026.2_1	LAIR1	XLOC_247740	2766	coding	coding	coding	coding
MSTRG.238525.1	FCAR	XLOC_247741	341	coding	noncoding	noncoding	noncoding
NM_024316.3_6	LENG1	XLOC_247868	1381	coding	coding	coding	coding
MSTRG.238613.1	LAIR1	XLOC_247892	2563	coding	coding	noncoding	noncoding
NM_001278428.3_2	LILRB4	XLOC_247902	3902	coding	coding	coding	coding
NM_001289026.2_2	LAIR1	XLOC_247934	2677	coding	coding	coding	coding
MSTRG.238624.2	LILRA2	XLOC_247942	4291	coding	coding	coding	coding
MSTRG.238624.7	LILRA2	XLOC_247942	3022	coding	coding	coding	coding
MSTRG.238624.8	LILRA2	XLOC_247942	3141	coding	coding	coding	coding
MSTRG.238624.15	LILRA2	XLOC_247942	4213	coding	coding	noncoding	coding
MSTRG.238652.2	LAIR1	XLOC_247966	2682	coding	coding	noncoding	noncoding
NM_001289026.2_3	LAIR1	XLOC_247995	2766	coding	coding	coding	coding
MSTRG.238741.2	RPL7A	XLOC_248155	1123	coding	coding	coding	noncoding
MSTRG.238765.2	CCDC84	XLOC_248173	1824	coding	coding	coding	coding
MSTRG.238790.2	PSMC4	XLOC_248207	692	coding	coding	coding	noncoding
MSTRG.238797.1	ZNF546	XLOC_248211	364	coding	noncoding	noncoding	noncoding
MSTRG.238835.3	PADI4	XLOC_248264	1923	coding	coding	coding	coding
MSTRG.238852.3	ATG16L1	XLOC_248286	8317	coding	coding	coding	noncoding
MSTRG.238990.7	FAN1	XLOC_248431	3778	coding	coding	coding	noncoding
MSTRG.238943.1		XLOC_248467	253	coding	noncoding	noncoding	noncoding
MSTRG.239028.1	IGKV7-3	XLOC_248545	362	coding	coding	noncoding	noncoding
IGKV3-20_1	IGKV3-20	XLOC_248551	348	coding	coding	coding	noncoding
MSTRG.239109.1		XLOC_248646	297	coding	noncoding	noncoding	noncoding
MSTRG.239114.2	ATAD1	XLOC_248660	995	coding	coding	noncoding	noncoding
MSTRG.239238.1	MRPS12	XLOC_248801	802	coding	coding	coding	noncoding
MSTRG.239245.1		XLOC_248825	329	coding	noncoding	noncoding	noncoding
MSTRG.239276.2	NDUFS1	XLOC_248865	439	coding	noncoding	noncoding	noncoding
MSTRG.239303.4	GUSBP1	XLOC_248909	978	coding	coding	coding	noncoding
MSTRG.239316.1	GUSBP1	XLOC_248921	795	coding	noncoding	noncoding	noncoding
MSTRG.239345.6	HNRNPH1	XLOC_248945	1903	coding	coding	noncoding	noncoding
MSTRG.239333.4	CANX	XLOC_248969	4911	coding	coding	noncoding	noncoding
MSTRG.239353.2	SQSTM1	XLOC_248973	1995	coding	coding	coding	coding
NM_001102614.1_1	SLC35G6	XLOC_248986	1195	coding	noncoding	coding	coding
MSTRG.239406.5	PLCL2	XLOC_249163	1550	coding	noncoding	coding	noncoding

MSTRG.239537.3	SMG1P3	XLOC_249287	2369	coding	coding	coding	noncoding
MSTRG.239537.7	SMG1P1	XLOC_249287	3948	coding	coding	coding	noncoding
MSTRG.239537.6	SMG1P1	XLOC_249287	1010	coding	coding	coding	coding
NR_047581.1_1	POLR3E	XLOC_249344	3681	coding	coding	coding	coding
MSTRG.239627.2	RRN3P1	XLOC_249351	4750	coding	coding	coding	coding
MSTRG.239627.1	RRN3P1	XLOC_249351	4272	coding	coding	coding	noncoding
MSTRG.239627.7	RRN3P1	XLOC_249351	7299	coding	coding	coding	noncoding
MSTRG.239627.22	LOC112268174	XLOC_249351	6466	coding	coding	noncoding	noncoding
MSTRG.239627.46		XLOC_249351	6431	coding	coding	coding	noncoding
MSTRG.239705.1		XLOC_249452	267	coding	noncoding	noncoding	noncoding
NR_126022.1_1	LOC101928605	XLOC_249527	990	coding	coding	noncoding	noncoding
MSTRG.239818.3	DEFA1	XLOC_249629	750	coding	coding	coding	coding
MSTRG.239822.1	DEFA3	XLOC_249630	574	coding	coding	noncoding	noncoding
MSTRG.239879.3	FDFT1	XLOC_249654	1869	coding	coding	noncoding	noncoding
MSTRG.239863.1		XLOC_249846	243	coding	noncoding	noncoding	noncoding
MSTRG.240109.1	LOC100506990	XLOC_249996	379	coding	coding	noncoding	noncoding
MSTRG.240227.16	GMPR2	XLOC_250083	4476	coding	coding	coding	coding
NR_023921.2_1	DHRS4-AS1	XLOC_250095	3052	coding	noncoding	coding	noncoding
MSTRG.240205.11	DCAF11	XLOC_250103	3282	coding	coding	coding	coding
MSTRG.240212.5	IRF9	XLOC_250109	775	coding	coding	noncoding	noncoding
MSTRG.240212.7	IRF9	XLOC_250109	1648	coding	coding	noncoding	noncoding
NM_006405.7_1	TM9SF1	XLOC_250111	2200	coding	coding	coding	coding
MSTRG.240285.8	FNBP4	XLOC_250176	2442	coding	coding	coding	noncoding
MSTRG.240383.1	HSD17B7P2	XLOC_250301	454	coding	noncoding	noncoding	noncoding
NM_001242932.1_1	LSP1	XLOC_250322	2016	coding	coding	coding	coding
MSTRG.240481.1		XLOC_250410	371	coding	noncoding	noncoding	noncoding
NM_001286742.1_1	UBL7	XLOC_250445	1548	coding	coding	coding	coding
NM_001143760.1_1	EIF5A	XLOC_250626	1256	coding	coding	coding	coding
MSTRG.240742.8	RNA45SN1	XLOC_250701	3719	coding	noncoding	coding	noncoding
MSTRG.240737.2	C1GALT1C1	XLOC_250717	1291	coding	noncoding	noncoding	noncoding
NM_012084.4_1	GLUD2	XLOC_250718	2485	coding	coding	coding	coding

Downregulated DE mRNAs

Gene_id	Gene_name	Xloc_id	trans_len	EggNOG	Pfam	CNCI	CPC2
MSTRG.156.1	LOC729737	XLOC_000011	7220	coding	coding	coding	noncoding
MSTRG.169.1	LOC112268260	XLOC_000020	478	coding	coding	noncoding	noncoding
MSTRG.185.1		XLOC_000023	636	coding	coding	noncoding	noncoding
MSTRG.194.1	LOC107984841	XLOC_000026	5412	coding	coding	coding	noncoding
XR_002958526.1	LOC284600	XLOC_000039	3858	coding	noncoding	coding	noncoding
MSTRG.22.7	KLHL17	XLOC_000043	269	coding	coding	coding	noncoding
MSTRG.15.1		XLOC_000047	238	coding	noncoding	noncoding	noncoding
MSTRG.21.1	ISG15	XLOC_000049	381	coding	noncoding	noncoding	noncoding
MSTRG.46.1	C1orf159	XLOC_000056	216	coding	noncoding	noncoding	noncoding

MSTRG.36.1		XLOC_000058	391	coding	noncoding	noncoding	noncoding
MSTRG.38.1	LINC01342	XLOC_000060	268	coding	noncoding	noncoding	noncoding
MSTRG.52.1	TTLL10-AS1	XLOC_000068	234	coding	noncoding	noncoding	noncoding
MSTRG.71.1	LINC01786	XLOC_000080	228	coding	noncoding	noncoding	noncoding
MSTRG.85.1	LOC105378585	XLOC_000099	255	coding	noncoding	noncoding	noncoding
NM_001039211.3	ATAD3C	XLOC_000103	3864	coding	coding	coding	coding
NM_001317238.2	ATAD3B	XLOC_000104	4491	coding	coding	coding	coding
MSTRG.121.1	ATAD3B	XLOC_000106	400	coding	noncoding	noncoding	noncoding
MSTRG.319.6	SLC35E2B	XLOC_000116	696	coding	noncoding	noncoding	noncoding
MSTRG.129.1		XLOC_000121	275	coding	noncoding	noncoding	noncoding
XM_017001793.1	PRKCZ	XLOC_000129	3212	coding	coding	coding	coding
MSTRG.234.1	FAAP20	XLOC_000135	252	coding	noncoding	noncoding	noncoding
NM_001303013.1	PLCH2	XLOC_000153	5811	coding	coding	coding	coding
MSTRG.278.2	PANK4	XLOC_000155	2393	coding	coding	coding	coding
MSTRG.247.1	HES5	XLOC_000156	305	coding	coding	noncoding	noncoding
NM_022114.4	PRDM16	XLOC_000182	8698	coding	coding	coding	coding
XM_011541786.2	AJAP1	XLOC_000243	12138	coding	coding	coding	coding
MSTRG.443.1		XLOC_000249	681	coding	noncoding	noncoding	noncoding
MSTRG.464.1		XLOC_000257	276	coding	noncoding	noncoding	noncoding
MSTRG.512.1	CHD5	XLOC_000292	443	coding	noncoding	coding	noncoding
MSTRG.513.1	CHD5	XLOC_000293	273	coding	noncoding	noncoding	noncoding
XM_011542236.2	ESPN	XLOC_000304	3501	coding	coding	coding	coding
MSTRG.591.1	TAS1R1	XLOC_000312	324	coding	coding	noncoding	noncoding
MSTRG.593.1	LOC107984912	XLOC_000313	268	coding	noncoding	noncoding	noncoding
MSTRG.640.1	CAMTA1	XLOC_000326	253	coding	coding	noncoding	noncoding
MSTRG.654.1	CAMTA1	XLOC_000331	229	coding	noncoding	noncoding	noncoding
MSTRG.655.1	CAMTA1	XLOC_000332	218	coding	noncoding	noncoding	noncoding
MSTRG.664.1	CAMTA1	XLOC_000337	454	coding	noncoding	noncoding	noncoding
MSTRG.700.1		XLOC_000368	855	coding	coding	noncoding	noncoding
MSTRG.698.1		XLOC_000372	298	coding	noncoding	noncoding	noncoding
MSTRG.709.1	SLC45A1	XLOC_000375	325	coding	noncoding	noncoding	noncoding
MSTRG.958.1	RERE	XLOC_000378	8748	coding	coding	coding	coding
MSTRG.958.3	RERE	XLOC_000378	3978	coding	coding	coding	coding
MSTRG.741.1	SLC2A7	XLOC_000401	255	coding	noncoding	noncoding	noncoding
MSTRG.742.1	SLC2A7	XLOC_000402	365	coding	noncoding	noncoding	noncoding
MSTRG.744.1		XLOC_000407	221	coding	coding	noncoding	noncoding
MSTRG.772.1	LNCTAM34A	XLOC_000415	272	coding	noncoding	noncoding	noncoding
XM_017002865.2	H6PD	XLOC_000420	11394	coding	coding	coding	coding
MSTRG.777.1		XLOC_000425	286	coding	noncoding	noncoding	noncoding
MSTRG.789.1		XLOC_000426	264	coding	noncoding	noncoding	noncoding
MSTRG.791.1		XLOC_000427	224	coding	noncoding	noncoding	noncoding
MSTRG.819.1		XLOC_000446	276	coding	noncoding	noncoding	noncoding
MSTRG.851.1	UBE4B	XLOC_000451	261	coding	noncoding	noncoding	noncoding

XM_005263470.5	PEX14	XLOC_000476	5077	coding	coding	coding	coding
MSTRG.930.1	PEX14	XLOC_000477	1008	coding	noncoding	noncoding	noncoding
MSTRG.931.1	PEX14	XLOC_000478	228	coding	noncoding	noncoding	noncoding
MSTRG.860.1		XLOC_000498	288	coding	noncoding	noncoding	noncoding
MSTRG.862.1		XLOC_000500	307	coding	noncoding	noncoding	noncoding
MSTRG.864.1		XLOC_000502	309	coding	noncoding	noncoding	noncoding
MSTRG.1019.5	MTOR	XLOC_000510	218	coding	noncoding	noncoding	noncoding
NM_021146.4	ANGPTL7	XLOC_000511	2224	coding	coding	coding	coding
NM_001330350.1	UBIAD1	XLOC_000513	5563	coding	coding	coding	coding
MSTRG.1025.1	UBIAD1	XLOC_000515	290	coding	noncoding	noncoding	noncoding
NM_020780.2	DISP3	XLOC_000523	5265	coding	coding	coding	coding
MSTRG.952.1	DISP3	XLOC_000524	266	coding	noncoding	noncoding	noncoding
NM_001256959.1	CLCN6	XLOC_000543	5632	coding	coding	coding	coding
MSTRG.1056.1	NPPA	XLOC_000545	233	coding	noncoding	noncoding	noncoding
MSTRG.1035.1		XLOC_000550	224	coding	noncoding	noncoding	noncoding
MSTRG.1040.1	PLOD1	XLOC_000552	229	coding	noncoding	noncoding	noncoding
MSTRG.1063.1	MFN2	XLOC_000555	304	coding	noncoding	noncoding	noncoding
MSTRG.1064.1	MFN2	XLOC_000556	229	coding	noncoding	noncoding	noncoding
XM_017002211.1	TNFRSF1B	XLOC_000569	2411	coding	coding	coding	coding
XM_017002215.1	TNFRSF1B	XLOC_000569	3632	coding	coding	coding	coding
XM_011542060.2	TNFRSF1B	XLOC_000569	3801	coding	coding	coding	coding
MSTRG.1079.1		XLOC_000597	243	coding	coding	noncoding	noncoding
NM_001080830.4	PRAMEF12	XLOC_000602	1779	coding	noncoding	coding	coding
MSTRG.1116.1	LOC107984919	XLOC_000626	272	coding	noncoding	noncoding	noncoding
XM_017002255.1	PRDM2	XLOC_000630	7189	coding	coding	coding	coding
MSTRG.1264.1	KAZN	XLOC_000654	287	coding	noncoding	noncoding	noncoding
MSTRG.1276.1	KAZN	XLOC_000660	216	coding	noncoding	noncoding	noncoding
MSTRG.1301.1	KAZN-AS1	XLOC_000669	307	coding	noncoding	noncoding	noncoding
MSTRG.1314.1	KAZN	XLOC_000674	215	coding	noncoding	noncoding	noncoding
MSTRG.1315.1	KAZN	XLOC_000675	295	coding	noncoding	noncoding	noncoding
MSTRG.1324.1	LOC105376759	XLOC_000678	759	coding	noncoding	noncoding	noncoding
MSTRG.1346.1	KAZN	XLOC_000684	288	coding	noncoding	noncoding	noncoding
MSTRG.1347.1	KAZN	XLOC_000685	225	coding	noncoding	noncoding	noncoding
MSTRG.1363.1	TMEM51-AS1	XLOC_000689	271	coding	noncoding	noncoding	noncoding
MSTRG.1366.1	TMEM51	XLOC_000691	227	coding	noncoding	noncoding	noncoding
XM_011540595.2	FHAD1	XLOC_000695	4488	coding	noncoding	coding	coding
MSTRG.1175.1	FHAD1	XLOC_000698	250	coding	noncoding	noncoding	noncoding
MSTRG.1148.1	CTRC	XLOC_000713	235	coding	noncoding	noncoding	noncoding
MSTRG.1162.1		XLOC_000725	267	coding	noncoding	noncoding	noncoding
XM_011541292.2	SLC25A34	XLOC_000736	4373	coding	coding	coding	coding
MSTRG.1372.1		XLOC_000750	211	coding	noncoding	noncoding	noncoding
MSTRG.1383.1		XLOC_000760	809	coding	noncoding	noncoding	noncoding
NM_001114600.3	SZRD1	XLOC_000772	3480	coding	coding	coding	noncoding

MSTRG.1419.1	SPATA21	XLOC_000773	205	coding	noncoding	noncoding	noncoding
MSTRG.1420.1	SPATA21	XLOC_000774	263	coding	noncoding	noncoding	noncoding
MSTRG.1423.1		XLOC_000783	225	coding	noncoding	noncoding	noncoding
MSTRG.1426.1	LINC01783	XLOC_000784	266	coding	noncoding	noncoding	noncoding
MSTRG.1448.1		XLOC_000799	405	coding	noncoding	noncoding	noncoding
MSTRG.1502.1	ATP13A2	XLOC_000815	200	coding	noncoding	noncoding	noncoding
MSTRG.1556.1	IGSF21	XLOC_000852	317	coding	noncoding	noncoding	noncoding
MSTRG.1548.1		XLOC_000860	229	coding	noncoding	noncoding	noncoding
NM_152375.2	KLHDC7A	XLOC_000861	5070	coding	coding	coding	coding
NM_001135254.2	PAX7	XLOC_000863	6213	coding	coding	coding	coding
MSTRG.1580.1	PAX7	XLOC_000864	236	coding	noncoding	noncoding	noncoding
MSTRG.1774.1	LOC105376815	XLOC_000883	285	coding	noncoding	noncoding	noncoding
NM_001204086.2	NBL1	XLOC_000909	2160	coding	coding	coding	coding
MSTRG.1640.1	MICOS10	XLOC_000913	280	coding	noncoding	noncoding	noncoding
MSTRG.1682.1		XLOC_000937	214	coding	noncoding	noncoding	noncoding
MSTRG.1677.1		XLOC_000938	322	coding	noncoding	noncoding	noncoding
MSTRG.1693.1	PLA2G5	XLOC_000942	285	coding	noncoding	noncoding	noncoding
NM_022819.4	PLA2G2F	XLOC_000946	2721	coding	coding	coding	coding
MSTRG.1752.1	LINC01141	XLOC_000956	291	coding	noncoding	noncoding	noncoding
MSTRG.1743.1		XLOC_000960	329	coding	noncoding	noncoding	noncoding
MSTRG.1756.1		XLOC_000969	302	coding	noncoding	noncoding	noncoding
MSTRG.1802.1	KIF17	XLOC_000974	367	coding	noncoding	noncoding	noncoding
MSTRG.1804.1	KIF17	XLOC_000975	270	coding	noncoding	noncoding	noncoding
MSTRG.1895.8	EIF4G3	XLOC_000981	5477	coding	coding	coding	coding
MSTRG.1824.1	ECE1	XLOC_000983	5735	coding	coding	coding	coding
MSTRG.1815.1		XLOC_000984	215	coding	noncoding	noncoding	noncoding
MSTRG.1816.1		XLOC_000985	261	coding	noncoding	noncoding	noncoding
XM_024450190.1	NBPF3	XLOC_000988	5209	coding	coding	coding	coding
MSTRG.1855.1	ALPL	XLOC_000992	276	coding	noncoding	noncoding	noncoding
MSTRG.1857.1	ALPL	XLOC_000994	223	coding	noncoding	noncoding	noncoding
MSTRG.1835.1		XLOC_000996	326	coding	noncoding	noncoding	noncoding
MSTRG.1925.8	USP48	XLOC_001004	1443	coding	coding	coding	noncoding
MSTRG.1876.1	HSPG2	XLOC_001007	242	coding	coding	noncoding	noncoding
MSTRG.1971.1		XLOC_001024	447	coding	noncoding	noncoding	noncoding
MSTRG.2004.1	ZBTB40	XLOC_001040	229	coding	noncoding	noncoding	noncoding
MSTRG.1936.1	LACTBL1	XLOC_001063	216	coding	noncoding	noncoding	noncoding
MSTRG.2039.1		XLOC_001087	272	coding	noncoding	noncoding	noncoding
MSTRG.2041.1		XLOC_001088	297	coding	noncoding	noncoding	noncoding
MSTRG.2057.1		XLOC_001092	268	coding	noncoding	noncoding	noncoding
MSTRG.2062.1	ELOA-AS1	XLOC_001095	230	coding	noncoding	noncoding	noncoding
MSTRG.2063.1	ELOA-AS1	XLOC_001096	260	coding	noncoding	noncoding	noncoding
MSTRG.2081.3	FUCA1	XLOC_001106	779	coding	coding	noncoding	noncoding
MSTRG.2103.1		XLOC_001124	268	coding	noncoding	noncoding	noncoding

XM_011541870.2	GRHL3	XLOC_001127	2671	coding	coding	coding	coding
NM_001010980.5	NCMAP	XLOC_001149	3980	coding	noncoding	coding	noncoding
MSTRG.2196.1	NCMAP	XLOC_001150	230	coding	noncoding	noncoding	noncoding
MSTRG.2201.1	NCMAP	XLOC_001152	270	coding	noncoding	noncoding	noncoding
NM_001366585.1	SRRM1	XLOC_001154	5843	coding	coding	coding	coding
NR_159383.1	SRRM1	XLOC_001154	3946	coding	coding	coding	coding
MSTRG.2174.1		XLOC_001171	242	coding	noncoding	noncoding	noncoding
MSTRG.2193.1		XLOC_001184	233	coding	noncoding	noncoding	noncoding
MSTRG.2247.10	RSRP1	XLOC_001188	5516	coding	noncoding	coding	coding
MSTRG.2254.1	TMEM50A	XLOC_001193	262	coding	noncoding	noncoding	noncoding
MSTRG.2268.1		XLOC_001222	305	coding	noncoding	noncoding	noncoding
MSTRG.2280.1		XLOC_001228	474	coding	noncoding	noncoding	noncoding
MSTRG.2310.1	CATSPER4	XLOC_001238	247	coding	noncoding	noncoding	noncoding
MSTRG.2334.7	UBXN11	XLOC_001244	710	coding	coding	coding	coding
MSTRG.2342.2	HMGN2	XLOC_001252	1046	coding	coding	noncoding	noncoding
NM_001202554.1	PIGV	XLOC_001271	2730	coding	coding	coding	coding
MSTRG.2354.1	ZDHHC18	XLOC_001276	274	coding	noncoding	noncoding	noncoding
MSTRG.2357.1		XLOC_001283	264	coding	noncoding	noncoding	noncoding
MSTRG.2399.1		XLOC_001295	586	coding	coding	noncoding	noncoding
MSTRG.2401.1		XLOC_001296	346	coding	noncoding	noncoding	noncoding
MSTRG.2402.1		XLOC_001297	227	coding	noncoding	noncoding	noncoding
MSTRG.2421.1	WDTC1	XLOC_001300	295	coding	noncoding	noncoding	noncoding
MSTRG.2441.1		XLOC_001318	208	coding	noncoding	noncoding	noncoding
MSTRG.2475.1		XLOC_001335	297	coding	noncoding	noncoding	noncoding
MSTRG.2478.1		XLOC_001337	279	coding	noncoding	noncoding	noncoding
XM_017000543.2	FAM76A	XLOC_001339	2139	coding	coding	coding	coding
MSTRG.2491.1	FAM76A	XLOC_001340	288	coding	noncoding	noncoding	noncoding
MSTRG.2496.1	STX12	XLOC_001342	260	coding	noncoding	noncoding	noncoding
MSTRG.2504.1		XLOC_001348	278	coding	noncoding	noncoding	noncoding
MSTRG.2506.1		XLOC_001358	200	coding	noncoding	noncoding	noncoding
MSTRG.2522.1	PTAFR	XLOC_001369	239	coding	noncoding	noncoding	noncoding
MSTRG.2532.1	PHACTR4	XLOC_001376	299	coding	noncoding	noncoding	noncoding
MSTRG.2535.1	PHACTR4	XLOC_001379	301	coding	noncoding	noncoding	noncoding
MSTRG.2578.1	RCC1	XLOC_001386	222	coding	noncoding	noncoding	noncoding
MSTRG.2582.1	TRNAU1AP	XLOC_001389	274	coding	noncoding	noncoding	noncoding
MSTRG.2583.1	TRNAU1AP	XLOC_001390	226	coding	noncoding	noncoding	noncoding
MSTRG.2623.1	OPRD1	XLOC_001402	315	coding	noncoding	noncoding	noncoding
NM_203343.3	EPB41	XLOC_001403	5675	coding	coding	coding	coding
MSTRG.2626.22	EPB41	XLOC_001403	3906	coding	coding	coding	coding
MSTRG.2606.1		XLOC_001404	261	coding	noncoding	noncoding	noncoding
MSTRG.2610.1		XLOC_001410	223	coding	noncoding	noncoding	noncoding
MSTRG.2668.1		XLOC_001427	274	coding	noncoding	noncoding	noncoding
MSTRG.2702.1		XLOC_001456	289	coding	noncoding	noncoding	noncoding

MSTRG.2750.1		XLOC_001480	270	coding	noncoding	noncoding	noncoding
MSTRG.2751.1		XLOC_001481	309	coding	noncoding	noncoding	noncoding
MSTRG.2752.1		XLOC_001482	443	coding	noncoding	noncoding	noncoding
MSTRG.2753.1		XLOC_001483	370	coding	noncoding	noncoding	noncoding
MSTRG.2762.1	NKAIN1	XLOC_001488	278	coding	noncoding	noncoding	noncoding
MSTRG.2764.1	NKAIN1	XLOC_001489	222	coding	noncoding	noncoding	noncoding
MSTRG.2821.1	ZCCHC17	XLOC_001499	281	coding	noncoding	noncoding	noncoding
MSTRG.2822.1	FABP3	XLOC_001500	226	coding	noncoding	noncoding	noncoding
MSTRG.2782.1	LOC107985470	XLOC_001503	247	coding	noncoding	noncoding	noncoding
MSTRG.2789.1	LOC105379772	XLOC_001508	216	coding	noncoding	noncoding	noncoding
MSTRG.2802.1		XLOC_001514	305	coding	noncoding	noncoding	noncoding
NM_022164.3	TINAGL1	XLOC_001515	2207	coding	coding	coding	coding
MSTRG.2840.9	HCRTR1	XLOC_001518	1321	coding	coding	noncoding	noncoding
MSTRG.2824.1		XLOC_001523	273	coding	noncoding	noncoding	noncoding
MSTRG.2825.1		XLOC_001525	201	coding	noncoding	noncoding	noncoding
MSTRG.2835.1		XLOC_001532	336	coding	noncoding	noncoding	noncoding
MSTRG.2869.1		XLOC_001538	280	coding	noncoding	noncoding	noncoding
NM_001301011.2	CCDC28B	XLOC_001544	1453	coding	coding	coding	coding
XM_017002306.2	CCDC28B	XLOC_001544	898	coding	coding	coding	coding
MSTRG.2894.1		XLOC_001555	282	coding	noncoding	noncoding	noncoding
MSTRG.2897.1	ZBTB8B	XLOC_001557	244	coding	noncoding	noncoding	noncoding
MSTRG.2898.1	ZBTB8B	XLOC_001558	209	coding	noncoding	noncoding	noncoding
MSTRG.2902.1	ZBTB8A	XLOC_001560	291	coding	noncoding	noncoding	noncoding
MSTRG.2927.1	RBBP4	XLOC_001566	302	coding	noncoding	noncoding	noncoding
MSTRG.2915.1		XLOC_001568	270	coding	noncoding	noncoding	noncoding
MSTRG.2917.1		XLOC_001569	244	coding	noncoding	noncoding	noncoding
MSTRG.2977.1		XLOC_001616	318	coding	noncoding	noncoding	noncoding
MSTRG.2982.1		XLOC_001618	300	coding	noncoding	noncoding	noncoding
MSTRG.3060.1	ZSCAN20	XLOC_001635	249	coding	noncoding	noncoding	noncoding
MSTRG.3055.1		XLOC_001636	264	coding	noncoding	noncoding	noncoding
MSTRG.3079.1		XLOC_001659	229	coding	noncoding	noncoding	noncoding
MSTRG.3095.1	LOC105378641	XLOC_001667	467	coding	coding	noncoding	noncoding
MSTRG.3102.1		XLOC_001679	241	coding	noncoding	noncoding	noncoding
MSTRG.3179.1	ZMYM1	XLOC_001705	430	coding	noncoding	noncoding	noncoding
MSTRG.3160.1		XLOC_001706	247	coding	noncoding	noncoding	noncoding
MSTRG.3202.1	ZMYM4	XLOC_001716	217	coding	noncoding	noncoding	noncoding
MSTRG.3191.1		XLOC_001738	337	coding	noncoding	noncoding	noncoding
MSTRG.3274.1		XLOC_001742	316	coding	noncoding	noncoding	noncoding
MSTRG.3275.1		XLOC_001743	306	coding	noncoding	noncoding	noncoding
XR_001737019.1	AGO4	XLOC_001744	5104	coding	coding	coding	coding
MSTRG.3283.1	AGO4	XLOC_001749	469	coding	noncoding	noncoding	noncoding
MSTRG.3284.1	AGO4	XLOC_001750	231	coding	noncoding	noncoding	noncoding
MSTRG.3290.1	AGO1	XLOC_001754	277	coding	noncoding	noncoding	noncoding

MSTRG.3293.1	AGO3	XLOC_001756	296	coding	noncoding	noncoding	noncoding
MSTRG.3250.3	ADPRHL2	XLOC_001764	244	coding	noncoding	noncoding	noncoding
MSTRG.3249.1		XLOC_001766	266	coding	noncoding	noncoding	noncoding
MSTRG.3259.1	TRAPPC3	XLOC_001770	222	coding	noncoding	noncoding	noncoding
MSTRG.3352.1	THRAP3	XLOC_001776	316	coding	noncoding	noncoding	noncoding
MSTRG.3360.2	STK40	XLOC_001779	3648	coding	coding	coding	coding
MSTRG.3360.6	STK40	XLOC_001779	3144	coding	coding	coding	noncoding
MSTRG.3307.1		XLOC_001787	248	coding	noncoding	noncoding	noncoding
MSTRG.3421.1	EPHA10	XLOC_001819	279	coding	noncoding	noncoding	noncoding
MSTRG.3527.1	GJA9-MYCBP	XLOC_001860	258	coding	noncoding	noncoding	noncoding
MSTRG.3534.1	RHBDL2	XLOC_001864	309	coding	noncoding	noncoding	noncoding
MSTRG.3507.1		XLOC_001865	303	coding	noncoding	noncoding	noncoding
MSTRG.3515.1	AKIRIN1	XLOC_001868	325	coding	noncoding	noncoding	noncoding
NM_001184979.1	NDUFS5	XLOC_001869	567	coding	coding	coding	noncoding
MSTRG.3652.1	MACF1	XLOC_001872	306	coding	noncoding	noncoding	noncoding
MSTRG.3665.1	MACF1	XLOC_001877	278	coding	noncoding	noncoding	noncoding
MSTRG.3674.1	MACF1	XLOC_001885	292	coding	noncoding	noncoding	noncoding
MSTRG.3681.1	MACF1	XLOC_001892	291	coding	noncoding	noncoding	noncoding
MSTRG.3685.1	MACF1	XLOC_001896	244	coding	noncoding	noncoding	noncoding
NM_181809.4	BMP8A	XLOC_001897	5636	coding	coding	coding	coding
MSTRG.3557.5	PABPC4-AS1	XLOC_001905	3259	coding	coding	coding	noncoding
MSTRG.3549.1		XLOC_001906	458	coding	noncoding	noncoding	noncoding
MSTRG.3575.1	PPIE	XLOC_001912	302	coding	noncoding	noncoding	noncoding
MSTRG.3569.1	LINC02811	XLOC_001918	264	coding	noncoding	noncoding	noncoding
MSTRG.3607.1		XLOC_001939	272	coding	noncoding	noncoding	noncoding
MSTRG.3727.1		XLOC_001980	265	coding	noncoding	noncoding	noncoding
MSTRG.3764.1	RIMS3	XLOC_001992	222	coding	noncoding	noncoding	noncoding
MSTRG.3745.1		XLOC_002001	342	coding	noncoding	noncoding	noncoding
MSTRG.3748.1		XLOC_002004	238	coding	noncoding	noncoding	noncoding
MSTRG.4155.1	HIVEP3	XLOC_002103	681	coding	noncoding	noncoding	noncoding
MSTRG.3823.1		XLOC_002143	279	coding	coding	noncoding	noncoding
MSTRG.3818.1		XLOC_002144	262	coding	noncoding	noncoding	noncoding
MSTRG.3828.1		XLOC_002148	222	coding	noncoding	noncoding	noncoding
MSTRG.3907.1	ZMYND12	XLOC_002156	427	coding	noncoding	noncoding	noncoding
MSTRG.3942.1	PPIH	XLOC_002172	224	coding	noncoding	noncoding	noncoding
NR_104171.1	LOC339539	XLOC_002181	2365	coding	coding	coding	coding
MSTRG.3883.1		XLOC_002191	248	coding	noncoding	noncoding	noncoding
MSTRG.3894.1		XLOC_002195	418	coding	noncoding	noncoding	noncoding
XM_005270467.4	TMEM125	XLOC_002198	2375	coding	coding	coding	coding
XM_006710869.1	TIE1	XLOC_002199	4114	coding	coding	coding	coding
MSTRG.3952.7	TIE1	XLOC_002199	458	coding	coding	coding	noncoding
XM_011541107.2	SZT2	XLOC_002208	10660	coding	noncoding	noncoding	coding
MSTRG.3990.1	SZT2	XLOC_002209	213	coding	noncoding	noncoding	noncoding

MSTRG.3963.1		XLOC_002214	323	coding	noncoding	noncoding	noncoding
MSTRG.3964.1		XLOC_002215	273	coding	noncoding	noncoding	noncoding
XM_005271079.3	PTPRF	XLOC_002216	7788	coding	coding	coding	coding
MSTRG.3965.1		XLOC_002219	277	coding	noncoding	noncoding	noncoding
MSTRG.4021.6	B4GALT2	XLOC_002242	417	coding	coding	noncoding	noncoding
MSTRG.4018.1	KLF17	XLOC_002250	302	coding	noncoding	noncoding	noncoding
MSTRG.4315.1	ZSWIM5	XLOC_002307	396	coding	noncoding	coding	coding
MSTRG.4340.1		XLOC_002336	280	coding	noncoding	noncoding	noncoding
MSTRG.4358.1	CCDC17	XLOC_002345	302	coding	noncoding	noncoding	noncoding
MSTRG.4372.4	GPBP1L1	XLOC_002347	3337	coding	coding	coding	noncoding
MSTRG.4375.1	TMEM69	XLOC_002349	235	coding	noncoding	noncoding	noncoding
XM_005270655.3	MAST2	XLOC_002355	5458	coding	coding	coding	coding
MSTRG.4383.1		XLOC_002374	503	coding	noncoding	noncoding	noncoding
MSTRG.4437.1		XLOC_002380	292	coding	noncoding	noncoding	noncoding
MSTRG.4430.1		XLOC_002383	274	coding	noncoding	noncoding	noncoding
NM_172225.2	DMBX1	XLOC_002387	5050	coding	coding	coding	coding
MSTRG.4458.6	MKNK1-AS1	XLOC_002389	2361	coding	coding	noncoding	coding
MSTRG.4483.1	CYP4Z2P	XLOC_002405	276	coding	noncoding	noncoding	noncoding
MSTRG.4497.1		XLOC_002410	265	coding	noncoding	noncoding	noncoding
MSTRG.4501.1	CYP4X1	XLOC_002412	325	coding	noncoding	noncoding	noncoding
MSTRG.4585.5	STIL	XLOC_002424	2970	coding	coding	noncoding	coding
MSTRG.4531.1		XLOC_002436	288	coding	noncoding	noncoding	noncoding
MSTRG.4605.1	SKINT1L	XLOC_002459	560	coding	noncoding	noncoding	noncoding
MSTRG.4729.1	AGBL4	XLOC_002515	295	coding	noncoding	noncoding	noncoding
MSTRG.4738.1	AGBL4	XLOC_002519	381	coding	noncoding	noncoding	noncoding
MSTRG.4747.1	AGBL4	XLOC_002524	304	coding	noncoding	noncoding	noncoding
MSTRG.4649.1	ELAVL4	XLOC_002532	276	coding	noncoding	noncoding	noncoding
MSTRG.4807.1	LOC105378720	XLOC_002563	309	coding	noncoding	noncoding	noncoding
MSTRG.4812.1	RAB3B	XLOC_002566	246	coding	noncoding	noncoding	noncoding
MSTRG.4815.1		XLOC_002567	425	coding	noncoding	noncoding	noncoding
XM_017002845.1	ZFYVE9	XLOC_002577	4324	coding	coding	coding	coding
MSTRG.4864.1	ZFYVE9	XLOC_002579	281	coding	noncoding	noncoding	noncoding
MSTRG.4869.1	ZFYVE9	XLOC_002581	281	coding	coding	noncoding	noncoding
MSTRG.4873.1	ZFYVE9	XLOC_002582	289	coding	noncoding	noncoding	noncoding
MSTRG.4875.1	ZFYVE9	XLOC_002583	253	coding	noncoding	noncoding	noncoding
MSTRG.4926.9	TUT4	XLOC_002596	3160	coding	coding	noncoding	noncoding
MSTRG.4887.1		XLOC_002600	244	coding	coding	noncoding	noncoding
MSTRG.4907.1	SHISAL2A	XLOC_002611	253	coding	noncoding	noncoding	noncoding
MSTRG.4912.1		XLOC_002613	298	coding	noncoding	noncoding	noncoding
MSTRG.4961.1	ZYG11B	XLOC_002620	237	coding	noncoding	noncoding	noncoding
MSTRG.4962.1	ZYG11B	XLOC_002621	311	coding	noncoding	noncoding	noncoding
XM_017000294.2	PODN	XLOC_002632	3648	coding	coding	coding	coding
MSTRG.5015.1	LRP8	XLOC_002645	201	coding	coding	noncoding	noncoding

NR_037639.2	MROH7-TTC4	XLOC_002699	6486	coding	noncoding	coding	coding
MSTRG.5109.1		XLOC_002716	309	coding	noncoding	noncoding	noncoding
MSTRG.5110.1		XLOC_002717	240	coding	noncoding	noncoding	noncoding
MSTRG.5114.1		XLOC_002722	381	coding	coding	noncoding	noncoding
MSTRG.5118.1		XLOC_002723	307	coding	noncoding	noncoding	noncoding
MSTRG.5248.9	USP24	XLOC_002725	3177	coding	coding	coding	noncoding
MSTRG.5174.1		XLOC_002728	232	coding	noncoding	noncoding	noncoding
MSTRG.5177.1		XLOC_002731	288	coding	noncoding	noncoding	noncoding
MSTRG.5200.1	LOC105378741	XLOC_002751	309	coding	coding	noncoding	noncoding
MSTRG.5210.1		XLOC_002762	249	coding	noncoding	noncoding	noncoding
MSTRG.5387.1	DAB1	XLOC_002775	257	coding	noncoding	noncoding	noncoding
MSTRG.5428.1	DAB1	XLOC_002805	213	coding	noncoding	noncoding	noncoding
MSTRG.5234.1		XLOC_002905	526	coding	noncoding	noncoding	noncoding
MSTRG.5365.7	OMA1	XLOC_002988	514	coding	coding	noncoding	noncoding
MSTRG.5365.9	OMA1	XLOC_002988	2426	coding	coding	noncoding	noncoding
MSTRG.5547.6	MYSM1	XLOC_002999	4157	coding	coding	noncoding	noncoding
MSTRG.5373.1	LOC112268263	XLOC_003002	237	coding	noncoding	noncoding	noncoding
MSTRG.5374.1	LOC112268263	XLOC_003003	296	coding	noncoding	noncoding	noncoding
MSTRG.5559.1		XLOC_003030	383	coding	noncoding	noncoding	noncoding
XM_017001656.1	FGGY	XLOC_003033	2122	coding	coding	coding	coding
MSTRG.6041.1	FGGY	XLOC_003036	256	coding	noncoding	noncoding	noncoding
MSTRG.6067.1	FGGY	XLOC_003054	250	coding	noncoding	noncoding	noncoding
MSTRG.5651.1		XLOC_003062	285	coding	noncoding	noncoding	noncoding
MSTRG.5710.1		XLOC_003085	302	coding	noncoding	noncoding	noncoding
MSTRG.5717.1		XLOC_003088	248	coding	noncoding	noncoding	noncoding
MSTRG.5718.1		XLOC_003105	218	coding	noncoding	noncoding	noncoding
MSTRG.5721.1		XLOC_003108	306	coding	noncoding	noncoding	noncoding
MSTRG.5727.1		XLOC_003112	233	coding	noncoding	noncoding	noncoding
XM_005270347.2	PATJ	XLOC_003124	4274	coding	coding	coding	coding
MSTRG.5804.1	PATJ	XLOC_003134	271	coding	noncoding	noncoding	noncoding
MSTRG.5809.1	PATJ	XLOC_003138	231	coding	noncoding	noncoding	noncoding
MSTRG.5754.1		XLOC_003143	303	coding	noncoding	noncoding	noncoding
MSTRG.5787.1		XLOC_003162	237	coding	noncoding	noncoding	noncoding
MSTRG.5816.1	LINC00466	XLOC_003182	250	coding	noncoding	noncoding	noncoding
MSTRG.5821.1	LINC00466	XLOC_003184	304	coding	noncoding	noncoding	noncoding
MSTRG.5984.1	ALG6	XLOC_003187	300	coding	noncoding	noncoding	noncoding
XM_011542301.2	EFCAB7	XLOC_003199	3092	coding	coding	coding	coding
MSTRG.5904.1	UBE2U	XLOC_003235	256	coding	noncoding	noncoding	noncoding
MSTRG.5889.1		XLOC_003242	538	coding	noncoding	noncoding	noncoding
MSTRG.5892.1		XLOC_003245	317	coding	coding	noncoding	noncoding
MSTRG.5897.1		XLOC_003247	420	coding	noncoding	noncoding	noncoding
MSTRG.5898.1		XLOC_003248	219	coding	noncoding	noncoding	noncoding
MSTRG.6023.1		XLOC_003270	242	coding	noncoding	noncoding	noncoding

MSTRG.6032.1	AK4	XLOC_003275	204	coding	noncoding	noncoding	noncoding
NM_001256865.1	DNAJC6	XLOC_003277	5916	coding	coding	coding	coding
NM_014787.4	DNAJC6	XLOC_003277	5750	coding	coding	coding	coding
NM_017526.5	LEPROT	XLOC_003279	4541	coding	coding	coding	coding
MSTRG.6103.1	LEPR	XLOC_003284	415	coding	noncoding	noncoding	noncoding
MSTRG.6106.1	LEPR	XLOC_003287	260	coding	noncoding	noncoding	noncoding
MSTRG.6094.1		XLOC_003296	263	coding	noncoding	noncoding	noncoding
MSTRG.6281.1	PDE4B	XLOC_003303	258	coding	noncoding	noncoding	noncoding
MSTRG.6303.1	PDE4B	XLOC_003305	239	coding	noncoding	noncoding	noncoding
NM_001350217.2	SGIP1	XLOC_003329	10749	coding	coding	coding	coding
MSTRG.6180.1	WDR78	XLOC_003340	250	coding	noncoding	noncoding	noncoding
MSTRG.6181.1	WDR78	XLOC_003341	290	coding	noncoding	noncoding	noncoding
NM_001146110.2	MIER1	XLOC_003343	5533	coding	coding	coding	coding
MSTRG.6172.1	IL23R	XLOC_003365	261	coding	noncoding	noncoding	noncoding
MSTRG.6366.1		XLOC_003371	493	coding	noncoding	noncoding	noncoding
MSTRG.6368.7	SERBP1	XLOC_003373	2510	coding	coding	noncoding	noncoding
MSTRG.6349.1		XLOC_003375	223	coding	noncoding	noncoding	noncoding
MSTRG.6466.6	WLS	XLOC_003385	3399	coding	coding	coding	coding
MSTRG.6468.1	GNG12-AS1	XLOC_003386	262	coding	noncoding	noncoding	noncoding
MSTRG.6471.1	GNG12-AS1	XLOC_003387	260	coding	noncoding	noncoding	noncoding
MSTRG.6375.1		XLOC_003397	271	coding	noncoding	noncoding	noncoding
MSTRG.6388.1		XLOC_003409	295	coding	coding	noncoding	noncoding
MSTRG.6401.1		XLOC_003413	493	coding	noncoding	noncoding	noncoding
MSTRG.6406.1		XLOC_003415	239	coding	noncoding	noncoding	noncoding
MSTRG.6431.1	LINC01707	XLOC_003429	211	coding	noncoding	noncoding	noncoding
MSTRG.6434.1	LINC01707	XLOC_003432	278	coding	noncoding	noncoding	noncoding
MSTRG.6446.1		XLOC_003438	298	coding	noncoding	noncoding	noncoding
MSTRG.6603.1	LRRC7	XLOC_003451	228	coding	noncoding	noncoding	noncoding
MSTRG.6507.1		XLOC_003468	221	coding	noncoding	noncoding	noncoding
XM_005270509.3	CTH	XLOC_003469	4258	coding	coding	coding	coding
MSTRG.6510.1		XLOC_003473	287	coding	noncoding	noncoding	noncoding
MSTRG.6517.1		XLOC_003476	292	coding	noncoding	noncoding	noncoding
MSTRG.6730.1	NEGR1	XLOC_003511	244	coding	noncoding	noncoding	noncoding
MSTRG.6774.1	NEGR1	XLOC_003547	305	coding	noncoding	noncoding	noncoding
MSTRG.6654.1		XLOC_003559	254	coding	noncoding	noncoding	noncoding
MSTRG.6668.1		XLOC_003564	248	coding	noncoding	noncoding	noncoding
MSTRG.6677.1		XLOC_003577	243	coding	noncoding	noncoding	noncoding
MSTRG.6702.1	LINC02238	XLOC_003582	268	coding	noncoding	noncoding	noncoding
NR_121670.1	ERICH3-AS1	XLOC_003601	4967	coding	noncoding	noncoding	noncoding
MSTRG.6834.1		XLOC_003609	288	coding	noncoding	noncoding	noncoding
MSTRG.6839.1		XLOC_003612	296	coding	noncoding	noncoding	noncoding
MSTRG.6872.1	ACADM	XLOC_003626	256	coding	noncoding	noncoding	noncoding
MSTRG.6888.1	MSH4	XLOC_003635	285	coding	noncoding	noncoding	noncoding

MSTRG.6904.1		XLOC_003683	263	coding	noncoding	noncoding	noncoding
MSTRG.6915.1	ST6GALNAC5	XLOC_003688	262	coding	noncoding	noncoding	noncoding
MSTRG.6964.1	AK5	XLOC_003704	318	coding	noncoding	noncoding	noncoding
MSTRG.7161.9	NEXN	XLOC_003722	10154	coding	coding	coding	coding
MSTRG.7171.1	DNAJB4	XLOC_003725	282	coding	noncoding	noncoding	noncoding
MSTRG.7176.1	DNAJB4	XLOC_003726	200	coding	noncoding	noncoding	noncoding
MSTRG.6994.1		XLOC_003728	238	coding	noncoding	noncoding	noncoding
MSTRG.6999.1	GIPC2	XLOC_003730	253	coding	noncoding	noncoding	noncoding
MSTRG.6995.1		XLOC_003731	210	coding	noncoding	noncoding	noncoding
NM_001039585.2	PTGFR	XLOC_003739	5500	coding	coding	noncoding	coding
MSTRG.7065.1		XLOC_003772	260	coding	noncoding	noncoding	noncoding
MSTRG.7077.1		XLOC_003777	258	coding	coding	noncoding	noncoding
NR_125940.1	LINC01781	XLOC_003782	591	coding	noncoding	noncoding	noncoding
MSTRG.7110.1		XLOC_003785	269	coding	noncoding	noncoding	noncoding
XM_006710485.3	ADGRL2	XLOC_003794	6439	coding	coding	coding	coding
MSTRG.7419.1	LINC01362	XLOC_003832	235	coding	coding	noncoding	noncoding
MSTRG.7422.1	LINC01362	XLOC_003833	639	coding	noncoding	noncoding	noncoding
MSTRG.7572.1	TTLL7	XLOC_003952	278	coding	noncoding	noncoding	noncoding
MSTRG.7241.1		XLOC_003956	283	coding	noncoding	noncoding	noncoding
XM_005271020.2	PRKACB	XLOC_003961	1818	coding	coding	coding	coding
MSTRG.7386.19	SPATA1	XLOC_003993	2530	coding	coding	coding	noncoding
MSTRG.7658.1	DDAH1	XLOC_004044	212	coding	noncoding	noncoding	noncoding
MSTRG.7659.1	DDAH1	XLOC_004045	335	coding	noncoding	noncoding	noncoding
MSTRG.7665.1	DDAH1	XLOC_004047	295	coding	noncoding	noncoding	noncoding
MSTRG.7734.1		XLOC_004101	244	coding	noncoding	noncoding	noncoding
MSTRG.7738.1		XLOC_004104	269	coding	noncoding	noncoding	noncoding
MSTRG.7771.1	CLCA4	XLOC_004106	248	coding	noncoding	noncoding	noncoding
MSTRG.7775.1	CLCA4-AS1	XLOC_004109	316	coding	noncoding	noncoding	noncoding
MSTRG.7777.1	CLCA4-AS1	XLOC_004111	306	coding	noncoding	noncoding	noncoding
MSTRG.7779.1	CLCA4-AS1	XLOC_004113	353	coding	noncoding	noncoding	noncoding
MSTRG.7800.1		XLOC_004159	286	coding	noncoding	noncoding	noncoding
MSTRG.7886.1		XLOC_004187	402	coding	coding	noncoding	noncoding
MSTRG.7908.1		XLOC_004202	257	coding	noncoding	noncoding	noncoding
MSTRG.7929.1	PKN2-AS1	XLOC_004210	285	coding	coding	noncoding	noncoding
MSTRG.7930.1	PKN2-AS1	XLOC_004211	290	coding	coding	noncoding	noncoding
NM_001320707.1	PKN2	XLOC_004220	6055	coding	coding	coding	coding
NM_001320709.2	PKN2	XLOC_004220	6022	coding	coding	coding	coding
MSTRG.7968.1	PKN2	XLOC_004247	914	coding	noncoding	noncoding	noncoding
MSTRG.8354.25	LOC112268267	XLOC_004270	5105	coding	coding	noncoding	noncoding
MSTRG.8370.1	GBP7	XLOC_004272	285	coding	noncoding	noncoding	noncoding
MSTRG.8431.1		XLOC_004282	523	coding	noncoding	noncoding	noncoding
XM_011542283.2	LRRC8C	XLOC_004292	8759	coding	coding	coding	coding
MSTRG.8006.1		XLOC_004363	268	coding	noncoding	noncoding	noncoding

MSTRG.8022.1	LOC105378849	XLOC_004367	260	coding	noncoding	noncoding	noncoding
MSTRG.8023.1	LOC105378849	XLOC_004368	265	coding	noncoding	noncoding	noncoding
MSTRG.8018.1		XLOC_004369	250	coding	noncoding	noncoding	noncoding
MSTRG.8036.1		XLOC_004375	253	coding	noncoding	noncoding	noncoding
MSTRG.8095.1		XLOC_004392	284	coding	noncoding	noncoding	noncoding
MSTRG.8131.1	HFM1	XLOC_004393	287	coding	noncoding	noncoding	noncoding
MSTRG.8137.1	HFM1	XLOC_004397	323	coding	noncoding	noncoding	noncoding
MSTRG.8202.1		XLOC_004417	277	coding	noncoding	noncoding	noncoding
MSTRG.8271.1	BTBD8	XLOC_004429	267	coding	noncoding	noncoding	noncoding
MSTRG.8303.5	GLMN	XLOC_004434	989	coding	coding	noncoding	coding
XR_246295.3	RPAP2	XLOC_004435	3087	coding	coding	coding	coding
MSTRG.8293.1		XLOC_004449	320	coding	noncoding	noncoding	noncoding
NM_000969.5	RPL5	XLOC_004458	1028	coding	coding	coding	coding
MSTRG.8637.5	CCDC18	XLOC_004471	12972	coding	coding	coding	coding
XM_011541625.2	FNBP1L	XLOC_004476	2327	coding	coding	coding	coding
MSTRG.8546.1	FNBP1L	XLOC_004481	201	coding	noncoding	noncoding	noncoding
MSTRG.8551.1	BCAR3	XLOC_004484	239	coding	noncoding	noncoding	noncoding
MSTRG.8582.1	BCAR3	XLOC_004505	315	coding	noncoding	noncoding	noncoding
MSTRG.8515.1		XLOC_004521	645	coding	noncoding	noncoding	noncoding
MSTRG.8618.1	LOC105378861	XLOC_004552	298	coding	noncoding	noncoding	noncoding
XM_011540635.3	SLC44A3	XLOC_004563	2320	coding	coding	coding	coding
MSTRG.8768.1		XLOC_004575	565	coding	noncoding	noncoding	noncoding
NM_001199691.1	TLCD4-RWDD3	XLOC_004576	1491	coding	coding	noncoding	coding
MSTRG.8770.4	TLCD4-RWDD3	XLOC_004576	628	coding	coding	noncoding	coding
MSTRG.8695.1		XLOC_004591	277	coding	noncoding	noncoding	noncoding
MSTRG.8739.1	LOC105378866	XLOC_004616	263	coding	noncoding	noncoding	noncoding
MSTRG.8781.1		XLOC_004635	535	coding	noncoding	noncoding	noncoding
MSTRG.8871.1	LOC100129620	XLOC_004678	248	coding	noncoding	noncoding	noncoding
MSTRG.8877.1		XLOC_004684	252	coding	noncoding	noncoding	noncoding
MSTRG.8897.1		XLOC_004695	418	coding	noncoding	noncoding	noncoding
XM_005270557.2	AGL	XLOC_004703	7057	coding	coding	coding	coding
MSTRG.8915.8	AGL	XLOC_004703	415	coding	coding	noncoding	noncoding
MSTRG.8912.1		XLOC_004705	249	coding	noncoding	noncoding	noncoding
MSTRG.8974.4	DBT	XLOC_004719	1784	coding	coding	noncoding	noncoding
MSTRG.9008.1	CDC14A	XLOC_004734	367	coding	noncoding	noncoding	noncoding
MSTRG.9012.1	CDC14A	XLOC_004738	287	coding	coding	noncoding	noncoding
MSTRG.8949.1		XLOC_004780	319	coding	coding	noncoding	noncoding
MSTRG.9089.1	OLFM3	XLOC_004836	352	coding	coding	noncoding	noncoding
MSTRG.9097.1	OLFM3	XLOC_004843	257	coding	noncoding	noncoding	noncoding
MSTRG.9106.1		XLOC_004849	252	coding	noncoding	noncoding	noncoding
MSTRG.9117.1		XLOC_004857	316	coding	coding	noncoding	noncoding
MSTRG.9132.1	COL11A1	XLOC_004863	430	coding	noncoding	noncoding	noncoding
MSTRG.9226.1	LOC101928436	XLOC_004868	311	coding	coding	noncoding	noncoding

MSTRG.9234.3	RNPC3	XLOC_004874	308	coding	noncoding	coding	noncoding
MSTRG.9177.1		XLOC_004883	200	coding	noncoding	noncoding	noncoding
MSTRG.9192.1		XLOC_004891	224	coding	noncoding	noncoding	noncoding
MSTRG.9204.1		XLOC_004897	249	coding	noncoding	noncoding	noncoding
MSTRG.9205.1		XLOC_004898	318	coding	noncoding	noncoding	noncoding
MSTRG.9215.1		XLOC_004903	312	coding	noncoding	noncoding	noncoding
MSTRG.9220.1		XLOC_004906	286	coding	noncoding	noncoding	noncoding
MSTRG.9260.1		XLOC_004915	289	coding	noncoding	noncoding	noncoding
MSTRG.9261.1		XLOC_004916	302	coding	noncoding	noncoding	noncoding
MSTRG.9264.1		XLOC_004919	281	coding	noncoding	noncoding	noncoding
MSTRG.9282.1		XLOC_004928	294	coding	noncoding	noncoding	noncoding
MSTRG.9283.1		XLOC_004929	270	coding	noncoding	noncoding	noncoding
MSTRG.9290.1		XLOC_004932	242	coding	noncoding	noncoding	noncoding
MSTRG.9294.1		XLOC_004935	233	coding	noncoding	noncoding	noncoding
MSTRG.9298.1		XLOC_004938	295	coding	noncoding	noncoding	noncoding
MSTRG.9319.1		XLOC_004948	257	coding	noncoding	noncoding	noncoding
MSTRG.9370.1		XLOC_004977	223	coding	noncoding	noncoding	noncoding
NM_001349761.2	PRPF38B	XLOC_004999	4894	coding	coding	coding	coding
MSTRG.9622.1	C1orf194	XLOC_005026	264	coding	noncoding	noncoding	noncoding
MSTRG.9625.1	KIAA1324	XLOC_005029	230	coding	noncoding	noncoding	noncoding
MSTRG.9627.1	KIAA1324	XLOC_005031	278	coding	noncoding	noncoding	noncoding
MSTRG.9616.1		XLOC_005034	217	coding	noncoding	noncoding	noncoding
MSTRG.9647.1	MYBPHL	XLOC_005041	209	coding	noncoding	noncoding	noncoding
MSTRG.9782.1	GNAI3	XLOC_005061	275	coding	noncoding	noncoding	noncoding
MSTRG.9796.1		XLOC_005080	261	coding	noncoding	noncoding	noncoding
NM_001010898.4	SLC6A17	XLOC_005090	6419	coding	coding	coding	coding
MSTRG.9902.4	KCNA3	XLOC_005125	6760	coding	coding	coding	coding
NM_001040033.1	CD53	XLOC_005134	1572	coding	coding	coding	coding
MSTRG.10016.11	DRAM2	XLOC_005153	1224	coding	coding	noncoding	noncoding
XM_024450080.1	CEPT1	XLOC_005153	2439	coding	coding	noncoding	coding
MSTRG.10035.1	TMIGD3	XLOC_005183	272	coding	noncoding	noncoding	noncoding
MSTRG.10037.1	RAP1A	XLOC_005185	277	coding	noncoding	noncoding	noncoding
MSTRG.10057.1	RAP1A	XLOC_005200	245	coding	noncoding	noncoding	noncoding
MSTRG.9989.1		XLOC_005226	304	coding	coding	noncoding	noncoding
XM_017001806.1	CTTNBP2NL	XLOC_005234	5704	coding	coding	coding	coding
MSTRG.10132.1	WNT2B	XLOC_005237	271	coding	noncoding	noncoding	noncoding
XM_017002424.2	CAPZA1	XLOC_005242	2511	coding	coding	coding	coding
NM_001286072.1	MOV10	XLOC_005245	4498	coding	coding	coding	coding
MSTRG.10144.18	MOV10	XLOC_005245	1039	coding	coding	coding	noncoding
MSTRG.10091.1		XLOC_005249	374	coding	noncoding	noncoding	noncoding
MSTRG.10097.1	LINC01356	XLOC_005250	244	coding	noncoding	noncoding	noncoding
MSTRG.10108.1		XLOC_005261	289	coding	coding	noncoding	noncoding
MSTRG.10114.1		XLOC_005264	230	coding	noncoding	noncoding	noncoding

MSTRG.10160.1	MAGI3	XLOC_005280	463	coding	coding	noncoding	noncoding
MSTRG.10170.1	MAGI3	XLOC_005281	285	coding	noncoding	noncoding	noncoding
XM_006710443.4	HIPK1	XLOC_005289	7792	coding	coding	coding	coding
MSTRG.10206.10	HIPK1	XLOC_005289	2980	coding	coding	coding	coding
MSTRG.10178.1		XLOC_005296	232	coding	noncoding	noncoding	noncoding
MSTRG.10184.1	SYT6	XLOC_005297	298	coding	noncoding	noncoding	noncoding
MSTRG.10215.1		XLOC_005318	303	coding	noncoding	noncoding	noncoding
MSTRG.10254.1	AMPD1	XLOC_005331	230	coding	noncoding	noncoding	noncoding
MSTRG.10312.1	SIKE1	XLOC_005335	5425	coding	coding	coding	noncoding
MSTRG.10295.1		XLOC_005336	287	coding	noncoding	noncoding	noncoding
MSTRG.10323.1	SYCP1	XLOC_005339	234	coding	noncoding	noncoding	noncoding
MSTRG.10327.1	SYCP1	XLOC_005341	231	coding	noncoding	noncoding	noncoding
MSTRG.10378.1	VANGL1	XLOC_005365	304	coding	noncoding	noncoding	noncoding
MSTRG.10371.1	CASQ2	XLOC_005366	243	coding	noncoding	noncoding	noncoding
MSTRG.10429.1	SLC22A15	XLOC_005381	241	coding	noncoding	noncoding	noncoding
NM_001160233.1	ATP1A1	XLOC_005407	3765	coding	coding	coding	coding
MSTRG.10553.1	TTF2	XLOC_005467	226	coding	noncoding	noncoding	noncoding
MSTRG.10576.1		XLOC_005490	249	coding	coding	noncoding	noncoding
MSTRG.10600.1		XLOC_005516	276	coding	noncoding	noncoding	noncoding
MSTRG.10614.1		XLOC_005523	319	coding	noncoding	noncoding	noncoding
MSTRG.10620.1		XLOC_005526	296	coding	noncoding	noncoding	noncoding
MSTRG.10623.1		XLOC_005527	303	coding	noncoding	noncoding	noncoding
XM_011541228.2	PHGDH	XLOC_005575	7269	coding	coding	coding	coding
MSTRG.10734.1	PHGDH	XLOC_005577	260	coding	noncoding	noncoding	noncoding
MSTRG.10801.2	SEC22B	XLOC_005591	5474	coding	coding	noncoding	noncoding
MSTRG.11360.1		XLOC_005597	258	coding	noncoding	noncoding	noncoding
MSTRG.11412.1		XLOC_005635	240	coding	noncoding	noncoding	noncoding
MSTRG.11426.1	LINC00623	XLOC_005646	222	coding	noncoding	noncoding	noncoding
NR_160533.1	LOC105369199	XLOC_005650	1313	coding	noncoding	noncoding	noncoding
MSTRG.11461.1	SRGAP2-AS1	XLOC_005669	273	coding	noncoding	noncoding	noncoding
XR_001737699.1	LOC101929805	XLOC_005791	3657	coding	noncoding	coding	coding
MSTRG.11097.16	LINC01145	XLOC_005796	1755	coding	noncoding	noncoding	noncoding
MSTRG.11097.20	LOC100996740	XLOC_005796	5308	coding	noncoding	coding	noncoding
NR_104075.1	RNVU1-14	XLOC_005798	164	coding	noncoding	noncoding	noncoding
XM_011509910.3	GPR89A	XLOC_005808	902	coding	coding	coding	coding
MSTRG.11114.1	ANKRD35	XLOC_005815	297	coding	noncoding	noncoding	noncoding
MSTRG.11122.1		XLOC_005826	241	coding	noncoding	noncoding	noncoding
MSTRG.11144.1		XLOC_005857	436	coding	noncoding	noncoding	noncoding
NR_145686.1	CHD1L	XLOC_005873	3124	coding	coding	coding	coding
MSTRG.11238.1	LINC00624	XLOC_005886	246	coding	noncoding	noncoding	noncoding
MSTRG.11268.1	LOC105371230	XLOC_005904	244	coding	coding	noncoding	noncoding
MSTRG.11263.1		XLOC_005908	230	coding	noncoding	noncoding	noncoding
NM_001350182.1	GPR89B	XLOC_005910	2252	coding	coding	coding	coding

MSTRG.12143.10	LOC100132057	XLOC_005931	3522	coding	noncoding	noncoding	noncoding
MSTRG.12143.14	LINC01138	XLOC_005931	852	coding	coding	coding	coding
MSTRG.12143.17	LOC105371224	XLOC_005931	4600	coding	noncoding	noncoding	noncoding
MSTRG.11551.1	LOC105371433	XLOC_006036	230	coding	noncoding	noncoding	noncoding
MSTRG.11530.4	ANP32E	XLOC_006039	1658	coding	noncoding	noncoding	noncoding
NR_073514.1	TARS2	XLOC_006052	2664	coding	coding	coding	coding
MSTRG.11574.1	ECM1	XLOC_006056	294	coding	noncoding	noncoding	noncoding
MSTRG.11592.17	ADAMTSL4	XLOC_006058	3318	coding	coding	coding	coding
MSTRG.11635.1	SETDB1	XLOC_006075	316	coding	noncoding	noncoding	noncoding
MSTRG.11636.1	SETDB1	XLOC_006076	321	coding	noncoding	noncoding	noncoding
MSTRG.11639.1	SETDB1	XLOC_006079	284	coding	noncoding	noncoding	noncoding
NM_021222.3	PRUNE1	XLOC_006084	3025	coding	coding	coding	coding
MSTRG.11676.1	MLLT11	XLOC_006092	263	coding	noncoding	noncoding	noncoding
NM_001323912.2	GABPB2	XLOC_006093	8644	coding	coding	coding	coding
MSTRG.11646.1	GABPB2	XLOC_006095	256	coding	noncoding	noncoding	noncoding
MSTRG.11647.1	GABPB2	XLOC_006096	320	coding	noncoding	noncoding	noncoding
MSTRG.11677.1		XLOC_006105	239	coding	noncoding	noncoding	noncoding
MSTRG.11693.1		XLOC_006112	250	coding	noncoding	noncoding	noncoding
MSTRG.11703.1		XLOC_006115	233	coding	noncoding	noncoding	noncoding
MSTRG.11706.1	CGN	XLOC_006118	275	coding	noncoding	noncoding	noncoding
MSTRG.11709.1		XLOC_006121	234	coding	noncoding	noncoding	noncoding
MSTRG.11795.1	LOC105371441	XLOC_006161	259	coding	noncoding	noncoding	noncoding
XR_002958366.1	LOC112268240	XLOC_006174	1960	coding	noncoding	coding	noncoding
MSTRG.11835.1	FLG	XLOC_006178	418	coding	coding	noncoding	noncoding
MSTRG.11843.1		XLOC_006181	222	coding	coding	noncoding	noncoding
MSTRG.11845.1		XLOC_006183	287	coding	noncoding	noncoding	noncoding
MSTRG.11951.1	S100A13	XLOC_006256	243	coding	noncoding	noncoding	noncoding
NM_001324475.2	INTS3	XLOC_006264	4911	coding	coding	coding	coding
MSTRG.11982.2	SLC39A1	XLOC_006270	1852	coding	coding	coding	coding
MSTRG.12003.1	TPM3	XLOC_006278	297	coding	noncoding	noncoding	noncoding
MSTRG.12041.1	IL6R	XLOC_006297	276	coding	noncoding	noncoding	noncoding
MSTRG.12042.1	IL6R	XLOC_006298	212	coding	noncoding	noncoding	noncoding
MSTRG.12043.1	SHE	XLOC_006299	339	coding	noncoding	noncoding	noncoding
MSTRG.12045.1	SHE	XLOC_006301	255	coding	noncoding	noncoding	noncoding
MSTRG.12051.4	CHRNB2	XLOC_006304	6608	coding	coding	coding	coding
MSTRG.12051.3	CHRNB2	XLOC_006304	6607	coding	coding	coding	coding
MSTRG.12051.2	CHRNB2	XLOC_006304	6606	coding	coding	coding	coding
MSTRG.12069.1	LOC105371449	XLOC_006312	241	coding	noncoding	noncoding	noncoding
MSTRG.12091.1		XLOC_006335	256	coding	noncoding	noncoding	noncoding
MSTRG.12486.14	ASH1L-AS1	XLOC_006352	6189	coding	noncoding	coding	coding
NR_147963.1	ASH1L-AS1	XLOC_006352	1209	coding	noncoding	coding	noncoding
MSTRG.12486.20	GON4L	XLOC_006352	4743	coding	noncoding	coding	coding
XM_024448043.1	MSTO1	XLOC_006358	2697	coding	coding	coding	coding

MSTRG.12361.1	LOC107985210	XLOC_006372	220	coding	noncoding	noncoding	noncoding
MSTRG.12365.1	LMNA	XLOC_006378	462	coding	noncoding	noncoding	noncoding
NM_001370571.1	SEMA4A	XLOC_006379	3463	coding	coding	coding	coding
MSTRG.12426.1		XLOC_006382	283	coding	coding	noncoding	noncoding
MSTRG.12378.1		XLOC_006392	304	coding	noncoding	noncoding	noncoding
MSTRG.12384.1	C1orf61	XLOC_006400	285	coding	coding	noncoding	noncoding
MSTRG.12404.1	IQGAP3	XLOC_006408	236	coding	noncoding	noncoding	noncoding
MSTRG.12543.1		XLOC_006458	325	coding	noncoding	noncoding	noncoding
MSTRG.12556.1		XLOC_006460	321	coding	noncoding	noncoding	noncoding
MSTRG.12595.1	KIRREL1	XLOC_006480	304	coding	noncoding	noncoding	noncoding
MSTRG.12604.1		XLOC_006489	285	coding	noncoding	noncoding	noncoding
MSTRG.12619.1	CD1B	XLOC_006496	255	coding	noncoding	noncoding	noncoding
XM_011510134.3	CD1E	XLOC_006497	2551	coding	coding	coding	coding
MSTRG.12622.1		XLOC_006499	232	coding	noncoding	noncoding	noncoding
MSTRG.12628.1		XLOC_006502	283	coding	coding	noncoding	noncoding
MSTRG.12698.1	OR6N1	XLOC_006531	284	coding	noncoding	noncoding	noncoding
XM_017000463.1	PYHIN1	XLOC_006537	2554	coding	coding	coding	coding
XM_017001150.2	IFI16	XLOC_006548	3548	coding	coding	coding	coding
MSTRG.12737.1		XLOC_006579	275	coding	noncoding	noncoding	noncoding
MSTRG.12762.1	SLAMF9	XLOC_006621	257	coding	noncoding	noncoding	noncoding
MSTRG.12826.1	ATP1A4	XLOC_006634	401	coding	noncoding	noncoding	noncoding
MSTRG.12867.4	SLAMF6	XLOC_006646	2377	coding	noncoding	noncoding	noncoding
MSTRG.12867.5	SLAMF6	XLOC_006646	2944	coding	coding	noncoding	noncoding
NM_001282593.1	SLAMF7	XLOC_006662	2352	coding	noncoding	coding	noncoding
NM_001282589.1	SLAMF7	XLOC_006662	2504	coding	noncoding	coding	coding
NM_001282590.2	SLAMF7	XLOC_006662	2386	coding	noncoding	coding	coding
MSTRG.12919.1	LOC105371470	XLOC_006670	315	coding	coding	noncoding	noncoding
MSTRG.12986.1	ARHGAP30	XLOC_006679	4373	coding	coding	coding	coding
XM_017001451.1	UFC1	XLOC_006686	1231	coding	coding	coding	coding
MSTRG.13000.1		XLOC_006698	271	coding	noncoding	noncoding	noncoding
MSTRG.13134.1	NOS1AP	XLOC_006795	318	coding	noncoding	noncoding	noncoding
MSTRG.13136.1	LOC105371475	XLOC_006796	269	coding	noncoding	noncoding	noncoding
NM_001135240.2	C1orf226	XLOC_006804	4156	coding	coding	coding	coding
MSTRG.13123.1		XLOC_006807	241	coding	noncoding	noncoding	noncoding
MSTRG.13231.1	UAP1	XLOC_006814	299	coding	noncoding	noncoding	noncoding
MSTRG.13279.1	HSD17B7	XLOC_006826	256	coding	noncoding	noncoding	noncoding
MSTRG.13287.1		XLOC_006834	330	coding	noncoding	noncoding	noncoding
MSTRG.13306.1		XLOC_006883	358	coding	noncoding	noncoding	noncoding
MSTRG.13314.1		XLOC_006885	220	coding	noncoding	coding	noncoding
MSTRG.13310.1		XLOC_006890	324	coding	noncoding	noncoding	noncoding
MSTRG.13325.1		XLOC_006893	239	coding	noncoding	noncoding	noncoding
MSTRG.13329.1		XLOC_006895	327	coding	noncoding	noncoding	noncoding
MSTRG.13343.1		XLOC_006900	205	coding	noncoding	noncoding	noncoding

MSTRG.13438.1	PBX1	XLOC_006906	225	coding	noncoding	noncoding	noncoding
MSTRG.13443.1	LRRC52-AS1	XLOC_006927	306	coding	noncoding	noncoding	noncoding
MSTRG.13544.1		XLOC_006976	267	coding	noncoding	noncoding	noncoding
MSTRG.13565.1		XLOC_006997	286	coding	noncoding	noncoding	noncoding
MSTRG.13577.1		XLOC_007000	301	coding	noncoding	noncoding	noncoding
MSTRG.13637.1	MPZL1	XLOC_007046	257	coding	noncoding	noncoding	noncoding
MSTRG.13751.1	ADCY10	XLOC_007058	321	coding	noncoding	noncoding	noncoding
MSTRG.13754.1	ADCY10	XLOC_007060	258	coding	noncoding	noncoding	noncoding
MSTRG.13721.1		XLOC_007090	281	coding	noncoding	noncoding	noncoding
MSTRG.13800.1		XLOC_007110	319	coding	noncoding	noncoding	noncoding
MSTRG.13813.1		XLOC_007112	276	coding	noncoding	noncoding	noncoding
MSTRG.13791.1		XLOC_007115	511	coding	noncoding	noncoding	noncoding
MSTRG.13844.1		XLOC_007188	277	coding	noncoding	noncoding	noncoding
MSTRG.13930.1		XLOC_007190	304	coding	noncoding	noncoding	noncoding
MSTRG.13981.1	GORAB-AS1	XLOC_007205	249	coding	noncoding	noncoding	noncoding
MSTRG.13982.1	GORAB-AS1	XLOC_007206	266	coding	noncoding	noncoding	noncoding
MSTRG.13983.1	GORAB-AS1	XLOC_007207	279	coding	noncoding	noncoding	noncoding
MSTRG.14054.1		XLOC_007254	230	coding	noncoding	noncoding	noncoding
MSTRG.14060.1		XLOC_007256	270	coding	noncoding	noncoding	noncoding
MSTRG.14115.1		XLOC_007297	744	coding	noncoding	noncoding	noncoding
NM_001282751.1	SUCO	XLOC_007298	5497	coding	coding	coding	coding
NM_004905.3	PRDX6	XLOC_007421	1690	coding	coding	coding	coding
MSTRG.14219.1	SLC9C2	XLOC_007422	302	coding	noncoding	noncoding	noncoding
MSTRG.14265.1	KLHL20	XLOC_007429	247	coding	noncoding	noncoding	noncoding
MSTRG.14236.1		XLOC_007451	576	coding	noncoding	noncoding	noncoding
NM_001366456.1	RABGAP1L	XLOC_007455	6817	coding	noncoding	coding	coding
NM_001243765.2	RABGAP1L	XLOC_007455	7015	coding	noncoding	coding	coding
MSTRG.14746.1	RABGAP1L	XLOC_007523	318	coding	noncoding	noncoding	noncoding
MSTRG.14756.1	RABGAP1L	XLOC_007533	288	coding	noncoding	noncoding	noncoding
MSTRG.14774.1	RABGAP1L	XLOC_007551	223	coding	noncoding	noncoding	noncoding
MSTRG.14306.1		XLOC_007565	216	coding	noncoding	noncoding	noncoding
MSTRG.14869.18	COP1	XLOC_007604	1791	coding	coding	noncoding	noncoding
MSTRG.14935.1		XLOC_007609	453	coding	noncoding	noncoding	noncoding
XM_017002024.1	PAPPA2	XLOC_007612	9832	coding	coding	coding	coding
MSTRG.14509.1	PAPPA2	XLOC_007617	260	coding	noncoding	noncoding	noncoding
MSTRG.14546.1	ASTN1	XLOC_007625	249	coding	noncoding	noncoding	noncoding
MSTRG.14549.1		XLOC_007643	270	coding	noncoding	noncoding	noncoding
MSTRG.14558.1		XLOC_007644	229	coding	noncoding	noncoding	noncoding
MSTRG.14562.1		XLOC_007659	243	coding	noncoding	noncoding	noncoding
XM_017002849.1	RASAL2	XLOC_007660	12905	coding	coding	coding	coding
MSTRG.14586.1	RASAL2	XLOC_007662	314	coding	noncoding	noncoding	noncoding
MSTRG.14807.1	RALGPS2	XLOC_007694	227	coding	noncoding	coding	noncoding
MSTRG.14824.1	RALGPS2	XLOC_007710	290	coding	noncoding	noncoding	noncoding

XM_017003001.2	FAM20B	XLOC_007715	5900	coding	coding	coding	coding
NM_003101.6	SOAT1	XLOC_007724	6840	coding	coding	coding	coding
MSTRG.14839.1	AXDND1	XLOC_007729	270	coding	noncoding	noncoding	noncoding
MSTRG.14841.1	AXDND1	XLOC_007730	268	coding	noncoding	noncoding	noncoding
NM_001199085.3	TDRD5	XLOC_007733	3745	coding	coding	coding	coding
MSTRG.14860.1	FAM163A	XLOC_007738	320	coding	noncoding	noncoding	noncoding
MSTRG.14956.3	TOR1AIP2	XLOC_007740	2822	coding	coding	noncoding	coding
MSTRG.14975.1	CEP350	XLOC_007750	250	coding	noncoding	noncoding	noncoding
XM_017002755.1	LHX4	XLOC_007770	6160	coding	coding	coding	coding
MSTRG.15003.1	XPR1	XLOC_007788	311	coding	noncoding	noncoding	noncoding
MSTRG.15043.1		XLOC_007804	290	coding	noncoding	noncoding	noncoding
MSTRG.15044.1		XLOC_007805	260	coding	noncoding	noncoding	noncoding
MSTRG.15083.1	MR1	XLOC_007807	281	coding	noncoding	noncoding	noncoding
MSTRG.15079.1		XLOC_007821	412	coding	coding	noncoding	noncoding
XM_017002244.1	CACNA1E	XLOC_007823	17053	coding	coding	coding	coding
MSTRG.15100.1		XLOC_007838	279	coding	noncoding	noncoding	noncoding
MSTRG.15103.1		XLOC_007839	278	coding	noncoding	noncoding	noncoding
MSTRG.15108.1		XLOC_007842	869	coding	coding	noncoding	noncoding
MSTRG.15121.1		XLOC_007850	694	coding	noncoding	noncoding	noncoding
MSTRG.15126.1		XLOC_007853	256	coding	noncoding	noncoding	noncoding
MSTRG.15137.1	LINC01344	XLOC_007856	276	coding	noncoding	noncoding	noncoding
MSTRG.15138.1	LINC01344	XLOC_007857	301	coding	noncoding	noncoding	noncoding
MSTRG.15242.1		XLOC_007911	252	coding	noncoding	noncoding	noncoding
MSTRG.15247.1	SHCBP1L	XLOC_007913	349	coding	noncoding	noncoding	noncoding
MSTRG.15280.1	NMNAT2	XLOC_007925	234	coding	noncoding	noncoding	noncoding
XM_011510208.3	SMG7	XLOC_007929	5753	coding	coding	coding	coding
MSTRG.15373.1	RGL1	XLOC_007949	267	coding	noncoding	noncoding	noncoding
MSTRG.15416.1	LINC01633	XLOC_007999	615	coding	coding	coding	noncoding
MSTRG.15426.1	RNF2	XLOC_008004	304	coding	noncoding	noncoding	noncoding
MSTRG.15584.20	SWT1	XLOC_008011	589	coding	noncoding	coding	noncoding
NM_031935.3	HMCN1	XLOC_008030	18368	coding	coding	noncoding	coding
MSTRG.15674.1	PDC	XLOC_008047	300	coding	noncoding	noncoding	noncoding
MSTRG.15656.1		XLOC_008065	254	coding	noncoding	noncoding	noncoding
MSTRG.15709.1		XLOC_008091	306	coding	noncoding	noncoding	noncoding
MSTRG.15721.1		XLOC_008097	326	coding	noncoding	noncoding	noncoding
MSTRG.15724.1		XLOC_008098	245	coding	noncoding	noncoding	noncoding
MSTRG.15747.1	LOC105371657	XLOC_008107	338	coding	coding	noncoding	noncoding
MSTRG.15749.1	LOC105371657	XLOC_008109	266	coding	noncoding	noncoding	noncoding
MSTRG.15751.1	LOC105371657	XLOC_008110	250	coding	noncoding	noncoding	noncoding
MSTRG.15744.1		XLOC_008116	269	coding	noncoding	noncoding	noncoding
MSTRG.15844.1		XLOC_008143	292	coding	noncoding	noncoding	noncoding
MSTRG.15889.1		XLOC_008164	266	coding	noncoding	noncoding	noncoding
MSTRG.15978.3	CDC73	XLOC_008279	368	coding	coding	noncoding	noncoding

MSTRG.16121.1		XLOC_008371	357	coding	noncoding	noncoding	noncoding
MSTRG.16129.1		XLOC_008373	243	coding	noncoding	noncoding	noncoding
MSTRG.16164.1	LOC105371671	XLOC_008386	290	coding	noncoding	noncoding	noncoding
MSTRG.16169.1	LOC105371671	XLOC_008389	238	coding	noncoding	noncoding	noncoding
MSTRG.16151.1		XLOC_008396	291	coding	noncoding	noncoding	noncoding
MSTRG.16159.1		XLOC_008403	287	coding	noncoding	noncoding	noncoding
MSTRG.16176.1		XLOC_008404	359	coding	noncoding	noncoding	noncoding
MSTRG.16209.1	KCNT2	XLOC_008419	248	coding	noncoding	noncoding	noncoding
XM_017000344.1	NEK7	XLOC_008463	4136	coding	coding	coding	coding
NM_002838.5	PTPRC	XLOC_008513	5357	coding	coding	coding	coding
MSTRG.16351.1	LINC02789	XLOC_008545	306	coding	noncoding	noncoding	noncoding
MSTRG.16338.1		XLOC_008549	312	coding	coding	noncoding	noncoding
MSTRG.16354.1		XLOC_008552	296	coding	noncoding	noncoding	noncoding
MSTRG.16387.1	NR5A2	XLOC_008556	240	coding	noncoding	noncoding	noncoding
MSTRG.16400.1		XLOC_008581	354	coding	noncoding	noncoding	noncoding
MSTRG.16404.1		XLOC_008584	549	coding	noncoding	noncoding	noncoding
MSTRG.16509.3	KIF14	XLOC_008587	1514	coding	coding	noncoding	noncoding
MSTRG.16573.1		XLOC_008622	299	coding	noncoding	noncoding	noncoding
MSTRG.16670.1	IPO9	XLOC_008642	604	coding	noncoding	noncoding	noncoding
MSTRG.16604.1	TIMM17A	XLOC_008653	300	coding	noncoding	noncoding	noncoding
MSTRG.16626.2	PTPN7	XLOC_008660	1184	coding	coding	noncoding	coding
MSTRG.16625.1	PTPRVP	XLOC_008662	273	coding	noncoding	noncoding	noncoding
XM_011509842.2	LGR6	XLOC_008663	4807	coding	coding	coding	coding
MSTRG.16870.1		XLOC_008710	288	coding	noncoding	noncoding	noncoding
MSTRG.16883.3	RABIF	XLOC_008713	1617	coding	coding	coding	noncoding
MSTRG.16940.1		XLOC_008743	292	coding	noncoding	noncoding	noncoding
NM_001365784.1	ATP2B4	XLOC_008756	4326	coding	coding	coding	coding
XM_017002958.1	ZC3H11A	XLOC_008768	6026	coding	coding	coding	coding
MSTRG.17023.1		XLOC_008775	301	coding	noncoding	noncoding	noncoding
MSTRG.17032.1		XLOC_008778	244	coding	noncoding	noncoding	noncoding
MSTRG.17038.1	LINC00303	XLOC_008781	252	coding	noncoding	noncoding	noncoding
MSTRG.17035.1		XLOC_008782	213	coding	noncoding	noncoding	noncoding
MSTRG.17081.1	PLEKHA6	XLOC_008795	260	coding	noncoding	noncoding	noncoding
MSTRG.17085.1	PLEKHA6	XLOC_008798	262	coding	noncoding	noncoding	noncoding
MSTRG.17177.1		XLOC_008843	306	coding	noncoding	noncoding	noncoding
MSTRG.17186.1		XLOC_008850	354	coding	noncoding	noncoding	noncoding
MSTRG.17208.1	LEMD1	XLOC_008855	242	coding	coding	noncoding	noncoding
MSTRG.17230.1	SLC45A3	XLOC_008872	282	coding	noncoding	noncoding	noncoding
MSTRG.17272.1	LOC284581	XLOC_008894	225	coding	noncoding	noncoding	noncoding
MSTRG.17291.3	CTSE	XLOC_008899	1642	coding	coding	noncoding	coding
MSTRG.17403.1	SRGAP2	XLOC_008920	259	coding	noncoding	noncoding	noncoding
NM_001004023.2	DYRK3	XLOC_008939	8239	coding	coding	coding	coding
MSTRG.17350.1	IL19	XLOC_008952	266	coding	noncoding	noncoding	noncoding

MSTRG.17375.1	FCAMR	XLOC_008961	227	coding	noncoding	noncoding	noncoding
NM_006212.2	PFKFB2	XLOC_008963	7073	coding	coding	coding	coding
MSTRG.17413.1		XLOC_008973	265	coding	noncoding	noncoding	noncoding
MSTRG.17845.1	CR1L	XLOC_009048	226	coding	noncoding	noncoding	noncoding
MSTRG.17846.1	CR1L	XLOC_009049	249	coding	noncoding	noncoding	noncoding
NM_153826.4	CD46	XLOC_009057	3227	coding	coding	coding	coding
MSTRG.17861.8	MIR29B2CHG	XLOC_009063	4704	coding	noncoding	coding	noncoding
MSTRG.17534.1	PLXNA2	XLOC_009074	380	coding	coding	noncoding	noncoding
MSTRG.17507.1		XLOC_009115	395	coding	noncoding	noncoding	noncoding
MSTRG.17600.1	LOC105372892	XLOC_009135	260	coding	noncoding	noncoding	noncoding
MSTRG.17618.1	LOC107985255	XLOC_009142	257	coding	noncoding	noncoding	noncoding
NM_001146264.3	SYT14	XLOC_009199	12184	coding	coding	coding	coding
XM_017001740.1	HHAT	XLOC_009206	2048	coding	coding	coding	coding
MSTRG.17738.1	KCNH1	XLOC_009215	258	coding	noncoding	noncoding	noncoding
MSTRG.17741.1	KCNH1	XLOC_009216	221	coding	noncoding	noncoding	noncoding
MSTRG.17743.1	KCNH1	XLOC_009218	315	coding	noncoding	noncoding	noncoding
MSTRG.17744.1	KCNH1	XLOC_009219	235	coding	noncoding	noncoding	noncoding
MSTRG.17745.1	KCNH1	XLOC_009220	266	coding	noncoding	noncoding	noncoding
XM_017001765.2	RCOR3	XLOC_009228	3871	coding	coding	coding	coding
MSTRG.17753.1		XLOC_009251	240	coding	noncoding	noncoding	noncoding
MSTRG.17765.1	LINC01693	XLOC_009261	204	coding	noncoding	noncoding	noncoding
MSTRG.17773.1		XLOC_009264	286	coding	noncoding	noncoding	noncoding
MSTRG.17895.1		XLOC_009279	294	coding	noncoding	noncoding	noncoding
MSTRG.17912.1		XLOC_009303	225	coding	noncoding	noncoding	noncoding
XM_024453283.1	TATDN3	XLOC_009328	2285	coding	coding	coding	coding
MSTRG.18099.1	RPS6KC1	XLOC_009351	255	coding	noncoding	noncoding	noncoding
MSTRG.18113.1	RPS6KC1	XLOC_009360	320	coding	noncoding	noncoding	noncoding
MSTRG.18060.1	LOC105372912	XLOC_009365	294	coding	noncoding	noncoding	noncoding
XM_011509772.2	PROX1	XLOC_009376	8147	coding	coding	coding	coding
MSTRG.18048.1		XLOC_009381	366	coding	noncoding	noncoding	noncoding
MSTRG.18143.1	SMYD2	XLOC_009386	643	coding	noncoding	noncoding	noncoding
MSTRG.18155.1	PTPN14	XLOC_009393	302	coding	noncoding	noncoding	noncoding
MSTRG.18129.1		XLOC_009403	281	coding	noncoding	noncoding	noncoding
MSTRG.18135.1		XLOC_009410	222	coding	noncoding	noncoding	noncoding
MSTRG.18193.1	USH2A	XLOC_009424	293	coding	noncoding	noncoding	noncoding
MSTRG.18198.1	USH2A	XLOC_009427	275	coding	noncoding	noncoding	noncoding
MSTRG.18205.1	USH2A	XLOC_009433	288	coding	noncoding	noncoding	noncoding
MSTRG.18230.1	ESRRG	XLOC_009441	296	coding	noncoding	noncoding	noncoding
MSTRG.18214.1		XLOC_009452	238	coding	noncoding	noncoding	noncoding
NM_001135599.3	TGFB2	XLOC_009473	6004	coding	coding	coding	coding
MSTRG.18293.1		XLOC_009483	248	coding	noncoding	noncoding	noncoding
MSTRG.18299.1		XLOC_009487	251	coding	noncoding	noncoding	noncoding
MSTRG.18447.1		XLOC_009490	315	coding	noncoding	noncoding	noncoding

MSTRG.18451.1		XLOC_009492	270	coding	noncoding	noncoding	noncoding
MSTRG.18328.1		XLOC_009535	318	coding	noncoding	noncoding	noncoding
MSTRG.18374.1		XLOC_009557	215	coding	noncoding	noncoding	noncoding
MSTRG.18376.1		XLOC_009558	261	coding	noncoding	noncoding	noncoding
NM_001286129.2	MARK1	XLOC_009559	2776	coding	noncoding	coding	noncoding
MSTRG.18399.1		XLOC_009581	211	coding	noncoding	noncoding	noncoding
MSTRG.18416.1		XLOC_009588	251	coding	noncoding	noncoding	noncoding
MSTRG.18431.1		XLOC_009596	235	coding	noncoding	noncoding	noncoding
MSTRG.18442.1		XLOC_009598	275	coding	noncoding	coding	noncoding
MSTRG.18444.1		XLOC_009599	298	coding	noncoding	noncoding	noncoding
MSTRG.18604.1		XLOC_009634	224	coding	noncoding	noncoding	noncoding
MSTRG.18610.1	LOC105372956	XLOC_009638	623	coding	coding	noncoding	noncoding
MSTRG.18619.1	HHIPL2	XLOC_009642	214	coding	noncoding	noncoding	noncoding
MSTRG.18703.1	DISP1	XLOC_009667	296	coding	noncoding	noncoding	noncoding
MSTRG.18651.1		XLOC_009681	247	coding	noncoding	noncoding	noncoding
MSTRG.18762.1		XLOC_009723	279	coding	noncoding	noncoding	noncoding
MSTRG.18733.1	LOC105373061	XLOC_009745	610	coding	noncoding	noncoding	noncoding
MSTRG.18731.1		XLOC_009746	278	coding	noncoding	noncoding	noncoding
XM_017002648.2	DEGS1	XLOC_009754	1826	coding	coding	noncoding	coding
MSTRG.18806.1	DEGS1	XLOC_009755	367	coding	noncoding	noncoding	noncoding
MSTRG.18760.1		XLOC_009757	260	coding	noncoding	noncoding	noncoding
MSTRG.18761.1		XLOC_009758	222	coding	noncoding	noncoding	noncoding
MSTRG.18808.1		XLOC_009760	281	coding	noncoding	noncoding	noncoding
NM_001277199.2	CNIH4	XLOC_009764	4374	coding	coding	noncoding	noncoding
MSTRG.18825.1		XLOC_009775	285	coding	noncoding	noncoding	noncoding
MSTRG.18826.1		XLOC_009776	309	coding	coding	noncoding	noncoding
XM_011544071.2	DNAH14	XLOC_009778	14796	coding	coding	coding	coding
MSTRG.18901.1	DNAH14	XLOC_009779	285	coding	coding	noncoding	noncoding
MSTRG.18859.1		XLOC_009791	254	coding	noncoding	noncoding	noncoding
MSTRG.18861.1		XLOC_009796	234	coding	noncoding	noncoding	noncoding
MSTRG.18958.1	EPHX1	XLOC_009804	7969	coding	coding	coding	coding
MSTRG.18888.2	PYCR2	XLOC_009810	879	coding	coding	noncoding	noncoding
MSTRG.18891.1		XLOC_009813	269	coding	noncoding	noncoding	noncoding
MSTRG.18927.1	LINC01703	XLOC_009818	452	coding	noncoding	noncoding	noncoding
MSTRG.18963.1		XLOC_009820	520	coding	noncoding	noncoding	noncoding
MSTRG.18950.1		XLOC_009823	262	coding	noncoding	noncoding	noncoding
MSTRG.18987.1		XLOC_009843	232	coding	noncoding	noncoding	noncoding
MSTRG.19044.5	CDC42BPA	XLOC_009854	653	coding	coding	noncoding	noncoding
MSTRG.19000.1		XLOC_009857	207	coding	noncoding	noncoding	noncoding
NM_178549.4	ZNF678	XLOC_009861	8515	coding	coding	noncoding	coding
MSTRG.19077.1	ZNF678	XLOC_009865	204	coding	noncoding	noncoding	noncoding
NM_183062.2	PRSS38	XLOC_009870	1243	coding	coding	coding	coding
MSTRG.19083.1	WNT3A	XLOC_009879	308	coding	noncoding	noncoding	noncoding

MSTRG.19186.1		XLOC_009918	656	coding	noncoding	noncoding	noncoding
MSTRG.19206.1	ACTA1	XLOC_009964	387	coding	coding	noncoding	noncoding
MSTRG.19219.6	ABCB10	XLOC_009969	2391	coding	coding	noncoding	noncoding
MSTRG.19219.12	ABCB10	XLOC_009969	1887	coding	coding	noncoding	noncoding
MSTRG.19219.11	ABCB10	XLOC_009969	2199	coding	coding	coding	noncoding
MSTRG.19209.1		XLOC_009970	272	coding	noncoding	noncoding	noncoding
XM_017000964.2	GALNT2	XLOC_009987	2778	coding	coding	coding	coding
MSTRG.19351.1	COG2	XLOC_010028	284	coding	noncoding	noncoding	noncoding
MSTRG.19388.7	TTC13	XLOC_010042	2047	coding	coding	coding	noncoding
MSTRG.19437.1		XLOC_010072	253	coding	noncoding	noncoding	noncoding
NM_001164554.2	DISC1	XLOC_010078	1832	coding	noncoding	coding	coding
MSTRG.19754.1	LOC105373170	XLOC_010103	421	coding	noncoding	noncoding	noncoding
MSTRG.19775.1	TSNAX-DISC1	XLOC_010123	209	coding	noncoding	noncoding	noncoding
MSTRG.19487.1		XLOC_010139	309	coding	noncoding	noncoding	noncoding
MSTRG.19498.1		XLOC_010150	343	coding	noncoding	noncoding	noncoding
MSTRG.19507.1	LINC01744	XLOC_010155	259	coding	noncoding	noncoding	noncoding
MSTRG.19546.1		XLOC_010197	283	coding	noncoding	noncoding	noncoding
MSTRG.19549.1		XLOC_010198	283	coding	noncoding	noncoding	noncoding
MSTRG.19563.1		XLOC_010206	301	coding	coding	noncoding	noncoding
MSTRG.19564.1		XLOC_010207	270	coding	noncoding	noncoding	noncoding
MSTRG.19584.1	SLC35F3	XLOC_010211	357	coding	coding	noncoding	noncoding
MSTRG.19585.1	SLC35F3	XLOC_010212	405	coding	noncoding	noncoding	noncoding
MSTRG.19661.1		XLOC_010261	331	coding	noncoding	noncoding	noncoding
MSTRG.19663.1		XLOC_010263	617	coding	noncoding	noncoding	noncoding
MSTRG.19693.1	GNG4	XLOC_010275	229	coding	noncoding	noncoding	noncoding
MSTRG.19867.1		XLOC_010289	285	coding	noncoding	noncoding	noncoding
MSTRG.19883.1		XLOC_010298	215	coding	noncoding	noncoding	noncoding
MSTRG.19884.1		XLOC_010299	316	coding	noncoding	noncoding	noncoding
MSTRG.19936.1	EDARADD	XLOC_010301	247	coding	noncoding	noncoding	noncoding
MSTRG.19941.1		XLOC_010303	291	coding	noncoding	noncoding	noncoding
MSTRG.19964.1		XLOC_010308	201	coding	noncoding	noncoding	noncoding
MSTRG.19968.1	ACTN2	XLOC_010311	364	coding	noncoding	noncoding	noncoding
MSTRG.19976.1	LOC107985368	XLOC_010332	268	coding	noncoding	noncoding	noncoding
MSTRG.20007.1	LOC105373220	XLOC_010362	230	coding	noncoding	noncoding	noncoding
MSTRG.20035.1		XLOC_010382	234	coding	noncoding	noncoding	noncoding
XM_017000152.2	CHRM3	XLOC_010388	12109	coding	coding	coding	coding
MSTRG.20137.1	LOC105373225	XLOC_010395	200	coding	noncoding	noncoding	noncoding
MSTRG.20113.1	FMN2	XLOC_010406	361	coding	noncoding	noncoding	noncoding
MSTRG.20115.1	FMN2	XLOC_010407	303	coding	noncoding	noncoding	noncoding
MSTRG.20121.1	FMN2	XLOC_010412	285	coding	noncoding	noncoding	noncoding
MSTRG.20122.1	FMN2	XLOC_010413	293	coding	noncoding	noncoding	noncoding
MSTRG.20175.1	LOC105373230	XLOC_010448	276	coding	noncoding	noncoding	noncoding
MSTRG.20282.1	WDR64	XLOC_010455	230	coding	noncoding	noncoding	noncoding

MSTRG.20284.1	WDR64	XLOC_010457	271	coding	noncoding	noncoding	noncoding
MSTRG.20216.1		XLOC_010461	300	coding	noncoding	coding	noncoding
MSTRG.20218.1		XLOC_010463	318	coding	noncoding	noncoding	noncoding
MSTRG.20248.1	PLD5	XLOC_010466	277	coding	coding	noncoding	noncoding
MSTRG.20233.1		XLOC_010479	287	coding	noncoding	noncoding	noncoding
MSTRG.20239.1		XLOC_010481	277	coding	noncoding	noncoding	noncoding
MSTRG.20291.1		XLOC_010493	238	coding	noncoding	noncoding	noncoding
MSTRG.20408.1	C1orf100	XLOC_010527	250	coding	noncoding	noncoding	noncoding
MSTRG.20414.5	ADSS	XLOC_010532	2298	coding	coding	coding	noncoding
MSTRG.20434.1	CATSPERE	XLOC_010535	220	coding	noncoding	noncoding	noncoding
XM_011544205.3	DESI2	XLOC_010539	4747	coding	coding	coding	coding
MSTRG.20350.1	DESI2	XLOC_010541	267	coding	noncoding	noncoding	noncoding
MSTRG.20345.1		XLOC_010542	276	coding	noncoding	noncoding	noncoding
MSTRG.20464.1		XLOC_010548	248	coding	noncoding	noncoding	noncoding
XM_011544300.2	EFCAB2	XLOC_010554	3792	coding	noncoding	coding	coding
MSTRG.20489.1	EFCAB2	XLOC_010558	305	coding	noncoding	noncoding	noncoding
MSTRG.20467.1	KIF26B	XLOC_010562	281	coding	noncoding	noncoding	noncoding
MSTRG.20469.1	KIF26B-AS1	XLOC_010563	297	coding	noncoding	noncoding	noncoding
MSTRG.20512.1		XLOC_010581	221	coding	noncoding	noncoding	noncoding
XM_005273083.5	CNST	XLOC_010583	4936	coding	coding	coding	coding
MSTRG.20540.1	ZNF670-ZNF695	XLOC_010594	243	coding	coding	noncoding	noncoding
MSTRG.20549.1	ZNF670	XLOC_010602	288	coding	noncoding	noncoding	noncoding
MSTRG.20579.1	LOC112268259	XLOC_010623	1676	coding	coding	noncoding	noncoding
MSTRG.20579.2	LOC112268259	XLOC_010623	1643	coding	coding	noncoding	noncoding
MSTRG.20620.1	LOC107985115	XLOC_010629	277	coding	noncoding	noncoding	noncoding
MSTRG.20660.3	TRIM58	XLOC_010646	2764	coding	noncoding	noncoding	noncoding
NM_001004686.2	OR2L2	XLOC_010656	1134	coding	coding	coding	coding
MSTRG.20784.1		XLOC_010717	398	coding	noncoding	noncoding	noncoding
NR_024540.1	WASH7P	XLOC_010720	1769	coding	coding	coding	coding
NR_039983.2	LOC729737	XLOC_010724	5474	coding	coding	coding	coding
MSTRG.180.1	LOC105378947	XLOC_010738	264	coding	noncoding	noncoding	noncoding
NR_027055.1	FAM41C	XLOC_010770	1706	coding	noncoding	noncoding	noncoding
MSTRG.88.10	ACAP3	XLOC_010802	310	coding	noncoding	noncoding	noncoding
XR_002957684.1	CCNL2	XLOC_010813	4399	coding	coding	coding	coding
MSTRG.114.1	ATAD3B	XLOC_010821	366	coding	noncoding	noncoding	noncoding
MSTRG.118.1	ATAD3B	XLOC_010825	255	coding	noncoding	noncoding	noncoding
MSTRG.105.1		XLOC_010832	314	coding	noncoding	noncoding	noncoding
MSTRG.310.1	MIB2	XLOC_010840	259	coding	noncoding	coding	noncoding
XM_017002145.2	NADK	XLOC_010848	3477	coding	coding	coding	coding
MSTRG.336.1	GNB1	XLOC_010859	281	coding	noncoding	noncoding	noncoding
MSTRG.338.1	GNB1	XLOC_010861	384	coding	noncoding	noncoding	noncoding
MSTRG.130.1		XLOC_010864	254	coding	noncoding	noncoding	noncoding
MSTRG.139.1	CFAP74	XLOC_010867	228	coding	noncoding	noncoding	noncoding

MSTRG.227.3	PRKCZ	XLOC_010872	885	coding	coding	noncoding	noncoding
NM_001282670.1	FAAP20	XLOC_010872	3576	coding	coding	coding	coding
MSTRG.140.1		XLOC_010874	221	coding	noncoding	noncoding	noncoding
XR_946873.2	LOC105378604	XLOC_010914	11969	coding	noncoding	coding	noncoding
MSTRG.398.1	TP73	XLOC_010932	299	coding	noncoding	coding	noncoding
MSTRG.409.1	LRRC47	XLOC_010938	284	coding	noncoding	noncoding	noncoding
MSTRG.413.1		XLOC_010952	233	coding	noncoding	noncoding	noncoding
MSTRG.509.1		XLOC_010992	272	coding	noncoding	noncoding	noncoding
NM_012405.4	ICMT	XLOC_011016	4795	coding	coding	coding	coding
MSTRG.533.1	ICMT	XLOC_011017	265	coding	noncoding	noncoding	noncoding
MSTRG.536.1	ACOT7	XLOC_011021	289	coding	noncoding	noncoding	noncoding
MSTRG.537.1	ACOT7	XLOC_011022	265	coding	noncoding	noncoding	noncoding
MSTRG.539.1	ACOT7	XLOC_011024	248	coding	noncoding	noncoding	noncoding
MSTRG.548.2	ESPN	XLOC_011030	1650	coding	coding	coding	noncoding
MSTRG.552.1	ESPN	XLOC_011032	246	coding	noncoding	noncoding	noncoding
MSTRG.601.1		XLOC_011049	270	coding	noncoding	noncoding	noncoding
MSTRG.665.1	CAMTA1	XLOC_011065	261	coding	noncoding	noncoding	noncoding
MSTRG.620.1	TNFRSF9	XLOC_011097	254	coding	noncoding	noncoding	noncoding
MSTRG.626.4	PARK7	XLOC_011098	854	coding	coding	noncoding	noncoding
MSTRG.632.1		XLOC_011104	286	coding	noncoding	noncoding	noncoding
MSTRG.634.1		XLOC_011105	266	coding	coding	noncoding	noncoding
MSTRG.705.1		XLOC_011107	307	coding	noncoding	noncoding	noncoding
MSTRG.703.1		XLOC_011108	299	coding	noncoding	noncoding	noncoding
MSTRG.702.1		XLOC_011110	221	coding	noncoding	noncoding	noncoding
MSTRG.977.1	RERE	XLOC_011131	365	coding	noncoding	noncoding	noncoding
MSTRG.991.1		XLOC_011142	353	coding	noncoding	noncoding	noncoding
XM_011540824.2	SLC2A7	XLOC_011149	1739	coding	coding	coding	coding
MSTRG.740.1	SLC2A7	XLOC_011150	293	coding	noncoding	noncoding	noncoding
MSTRG.771.1	LNCTAM34A	XLOC_011159	272	coding	noncoding	noncoding	noncoding
NR_104622.2	LINC02606	XLOC_011174	2363	coding	noncoding	coding	noncoding
MSTRG.790.1		XLOC_011177	235	coding	noncoding	noncoding	noncoding
MSTRG.834.8	PIK3CD	XLOC_011186	5138	coding	coding	coding	coding
NR_027045.1	PIK3CD-AS1	XLOC_011188	1823	coding	noncoding	coding	coding
MSTRG.821.1	LZIC	XLOC_011198	259	coding	noncoding	noncoding	noncoding
MSTRG.824.2	NMNAT1	XLOC_011200	1038	coding	coding	noncoding	noncoding
MSTRG.848.7	UBE4B	XLOC_011204	2159	coding	noncoding	noncoding	noncoding
MSTRG.859.1		XLOC_011222	288	coding	noncoding	noncoding	noncoding
MSTRG.937.1		XLOC_011239	439	coding	noncoding	noncoding	noncoding
NM_012168.6	FBXO2	XLOC_011249	1287	coding	coding	coding	coding
XM_005263460.5	MTHFR	XLOC_011260	3245	coding	coding	coding	coding
MSTRG.1027.1		XLOC_011270	301	coding	noncoding	noncoding	noncoding
MSTRG.1032.1		XLOC_011271	281	coding	noncoding	noncoding	noncoding
MSTRG.1067.1	TNFRSF8	XLOC_011278	433	coding	noncoding	noncoding	noncoding

MSTRG.1069.1	TNFRSF8	XLOC_011279	269	coding	noncoding	noncoding	noncoding
MSTRG.1081.1	AADACL4	XLOC_011298	304	coding	coding	noncoding	noncoding
MSTRG.1099.1		XLOC_011319	271	coding	noncoding	noncoding	noncoding
MSTRG.1104.1		XLOC_011324	238	coding	noncoding	noncoding	noncoding
MSTRG.1111.1		XLOC_011325	285	coding	noncoding	noncoding	noncoding
MSTRG.1112.1		XLOC_011326	266	coding	noncoding	noncoding	noncoding
MSTRG.1113.1		XLOC_011327	284	coding	noncoding	noncoding	noncoding
MSTRG.1285.1	KAZN	XLOC_011349	314	coding	noncoding	noncoding	noncoding
XM_017002222.2	C1orf195	XLOC_011401	1366	coding	noncoding	noncoding	noncoding
MSTRG.1174.1	FHAD1	XLOC_011409	250	coding	noncoding	noncoding	noncoding
MSTRG.1178.1	FHAD1	XLOC_011411	361	coding	noncoding	noncoding	noncoding
MSTRG.1164.4	DNAJC16	XLOC_011424	421	coding	coding	noncoding	noncoding
MSTRG.1159.1		XLOC_011425	228	coding	noncoding	noncoding	noncoding
MSTRG.1161.1		XLOC_011427	267	coding	noncoding	noncoding	noncoding
MSTRG.1242.1		XLOC_011433	241	coding	coding	coding	noncoding
MSTRG.1243.1	UQCRHL	XLOC_011434	255	coding	noncoding	noncoding	noncoding
MSTRG.1373.1		XLOC_011437	303	coding	noncoding	noncoding	noncoding
XR_946991.2	ANO7L1	XLOC_011445	1466	coding	noncoding	coding	noncoding
MSTRG.1413.1	FBXO42	XLOC_011451	214	coding	noncoding	noncoding	noncoding
MSTRG.1396.1		XLOC_011455	303	coding	noncoding	noncoding	noncoding
NR_023386.1	CROCCP3	XLOC_011458	5368	coding	coding	coding	coding
NR_135764.1	LINC01783	XLOC_011465	1546	coding	noncoding	coding	noncoding
MSTRG.1460.1	LOC105376794	XLOC_011471	711	coding	noncoding	noncoding	noncoding
MSTRG.1463.1		XLOC_011473	321	coding	noncoding	noncoding	noncoding
MSTRG.1464.1		XLOC_011474	289	coding	noncoding	noncoding	noncoding
MSTRG.1468.1	MST1P2	XLOC_011477	399	coding	noncoding	noncoding	noncoding
MSTRG.1506.1	SDHB	XLOC_011505	219	coding	noncoding	noncoding	noncoding
MSTRG.1510.1	SDHB	XLOC_011509	291	coding	noncoding	noncoding	noncoding
MSTRG.1520.1	PADI2	XLOC_011514	310	coding	noncoding	noncoding	noncoding
MSTRG.1534.1	ACTL8	XLOC_011531	210	coding	noncoding	noncoding	noncoding
MSTRG.1559.1	IGSF21	XLOC_011539	494	coding	noncoding	noncoding	noncoding
MSTRG.1553.1		XLOC_011546	234	coding	noncoding	noncoding	noncoding
MSTRG.1575.1	ALDH4A1	XLOC_011554	258	coding	noncoding	noncoding	noncoding
MSTRG.1615.1	IFFO2	XLOC_011560	245	coding	noncoding	noncoding	noncoding
MSTRG.1775.1	LOC105376815	XLOC_011563	285	coding	noncoding	noncoding	noncoding
XM_024450096.1	CAPZB	XLOC_011582	1782	coding	coding	coding	coding
MSTRG.1618.1		XLOC_011601	239	coding	noncoding	noncoding	noncoding
MSTRG.1628.1	HTR6	XLOC_011609	675	coding	noncoding	noncoding	noncoding
MSTRG.1738.1	TMCO4	XLOC_011617	267	coding	noncoding	noncoding	noncoding
MSTRG.1694.1	PLA2G5	XLOC_011632	285	coding	noncoding	noncoding	noncoding
NM_012400.4	PLA2G2D	XLOC_011636	2651	coding	coding	coding	coding
MSTRG.1681.1		XLOC_011637	380	coding	noncoding	noncoding	noncoding
MSTRG.1702.1	PLA2G2F	XLOC_011638	288	coding	noncoding	noncoding	noncoding

MSTRG.1705.1		XLOC_011644	213	coding	noncoding	noncoding	noncoding
MSTRG.1744.1		XLOC_011655	263	coding	noncoding	noncoding	noncoding
MSTRG.1747.1		XLOC_011656	316	coding	noncoding	noncoding	noncoding
MSTRG.1766.3	PINK1-AS	XLOC_011660	2013	coding	coding	coding	coding
MSTRG.1803.1	KIF17	XLOC_011662	270	coding	noncoding	noncoding	noncoding
XR_001737206.2	HP1BP3	XLOC_011667	4589	coding	coding	coding	coding
MSTRG.1916.1	EIF4G3	XLOC_011690	284	coding	noncoding	noncoding	noncoding
MSTRG.1827.1	ECE1	XLOC_011697	500	coding	noncoding	noncoding	noncoding
MSTRG.1830.1	ECE1	XLOC_011700	296	coding	noncoding	noncoding	noncoding
MSTRG.1817.1		XLOC_011704	261	coding	noncoding	noncoding	noncoding
MSTRG.1930.1	USP48	XLOC_011733	387	coding	noncoding	noncoding	noncoding
MSTRG.1866.1		XLOC_011742	267	coding	noncoding	noncoding	noncoding
MSTRG.1869.1	CELA3B	XLOC_011744	276	coding	noncoding	noncoding	noncoding
MSTRG.1962.5	CDC42	XLOC_011746	3344	coding	coding	noncoding	noncoding
MSTRG.1933.1		XLOC_011768	284	coding	noncoding	noncoding	noncoding
MSTRG.2040.1		XLOC_011798	272	coding	noncoding	noncoding	noncoding
MSTRG.2046.1		XLOC_011800	225	coding	noncoding	noncoding	noncoding
MSTRG.2051.1	RPL11	XLOC_011801	621	coding	coding	noncoding	coding
MSTRG.2055.1		XLOC_011807	266	coding	noncoding	noncoding	noncoding
MSTRG.2056.1		XLOC_011808	268	coding	noncoding	noncoding	noncoding
MSTRG.2088.1	CNR2	XLOC_011823	297	coding	noncoding	noncoding	noncoding
MSTRG.2115.2	SRSF10	XLOC_011824	13754	coding	coding	noncoding	noncoding
MSTRG.2115.6	SRSF10	XLOC_011824	7762	coding	coding	noncoding	noncoding
MSTRG.2090.1		XLOC_011826	243	coding	noncoding	noncoding	noncoding
MSTRG.2129.1	GRHL3	XLOC_011840	232	coding	noncoding	noncoding	noncoding
MSTRG.2118.12	RCAN3	XLOC_011847	2771	coding	coding	noncoding	coding
MSTRG.2200.1	NCMAP	XLOC_011851	270	coding	noncoding	noncoding	noncoding
MSTRG.2150.1		XLOC_011854	236	coding	noncoding	noncoding	noncoding
MSTRG.2152.1		XLOC_011856	239	coding	noncoding	noncoding	noncoding
MSTRG.2175.1		XLOC_011878	242	coding	noncoding	noncoding	noncoding
MSTRG.2191.1		XLOC_011884	238	coding	noncoding	noncoding	noncoding
NR_135784.2	RSRP1	XLOC_011890	2829	coding	coding	coding	coding
NR_135793.1	RSRP1	XLOC_011890	3356	coding	coding	coding	noncoding
MSTRG.2234.4	MACO1	XLOC_011894	1864	coding	coding	noncoding	coding
MSTRG.2243.1	LDLRAP1	XLOC_011899	238	coding	noncoding	noncoding	noncoding
MSTRG.2313.2	MAN1C1	XLOC_011905	2371	coding	coding	coding	noncoding
NM_001145454.3	STMN1	XLOC_011914	2162	coding	coding	coding	coding
MSTRG.2267.1		XLOC_011917	305	coding	noncoding	noncoding	noncoding
MSTRG.2297.1	C1orf232	XLOC_011932	253	coding	noncoding	noncoding	noncoding
MSTRG.2311.1	CATSPER4	XLOC_011935	298	coding	noncoding	noncoding	noncoding
MSTRG.2349.1		XLOC_011954	255	coding	noncoding	noncoding	noncoding
MSTRG.2391.1	GPN2	XLOC_011956	258	coding	noncoding	noncoding	noncoding
MSTRG.2413.1		XLOC_011969	275	coding	noncoding	noncoding	noncoding

XR_946795.3	MAP3K6	XLOC_011981	5526	coding	coding	coding	coding
MSTRG.2440.1		XLOC_011993	208	coding	noncoding	noncoding	noncoding
MSTRG.2476.1		XLOC_012008	297	coding	noncoding	noncoding	noncoding
MSTRG.2545.4	XKR8	XLOC_012016	1934	coding	coding	coding	coding
MSTRG.2563.1	EYA3	XLOC_012030	302	coding	noncoding	noncoding	noncoding
MSTRG.2568.1		XLOC_012035	263	coding	noncoding	noncoding	noncoding
MSTRG.2569.1		XLOC_012036	274	coding	noncoding	noncoding	noncoding
MSTRG.2588.1		XLOC_012058	235	coding	noncoding	noncoding	noncoding
MSTRG.2589.1		XLOC_012059	266	coding	noncoding	noncoding	noncoding
MSTRG.2624.1		XLOC_012065	385	coding	noncoding	noncoding	noncoding
MSTRG.2611.1		XLOC_012067	289	coding	noncoding	noncoding	noncoding
MSTRG.2669.1		XLOC_012086	257	coding	noncoding	noncoding	noncoding
MSTRG.2701.1		XLOC_012112	289	coding	noncoding	noncoding	noncoding
MSTRG.2708.1	LOC105378617	XLOC_012114	306	coding	noncoding	noncoding	noncoding
MSTRG.2717.1	LOC105378618	XLOC_012116	237	coding	noncoding	noncoding	noncoding
MSTRG.2726.1		XLOC_012123	227	coding	noncoding	noncoding	noncoding
XM_011542464.2	SDC3	XLOC_012133	5326	coding	coding	coding	coding
MSTRG.2755.1		XLOC_012147	488	coding	noncoding	noncoding	noncoding
MSTRG.2763.1	NKAIN1	XLOC_012149	222	coding	noncoding	noncoding	noncoding
MSTRG.2813.1	SNRNP40	XLOC_012152	259	coding	noncoding	noncoding	noncoding
MSTRG.2783.1		XLOC_012156	247	coding	noncoding	noncoding	noncoding
MSTRG.2787.1		XLOC_012161	261	coding	noncoding	noncoding	noncoding
MSTRG.2803.1		XLOC_012169	324	coding	noncoding	noncoding	noncoding
MSTRG.2805.1		XLOC_012171	227	coding	noncoding	noncoding	noncoding
MSTRG.2847.1	COL16A1	XLOC_012176	285	coding	noncoding	noncoding	noncoding
NM_144569.6	SPOCD1	XLOC_012182	3962	coding	coding	coding	coding
MSTRG.2839.1	SPOCD1	XLOC_012184	286	coding	noncoding	noncoding	noncoding
MSTRG.2870.1	KPNA6	XLOC_012196	7787	coding	coding	coding	noncoding
MSTRG.2870.5	KPNA6	XLOC_012196	7054	coding	coding	coding	noncoding
MSTRG.2890.1	MTMR9LP	XLOC_012204	261	coding	noncoding	noncoding	noncoding
MSTRG.2895.1		XLOC_012215	224	coding	noncoding	noncoding	noncoding
MSTRG.2899.1	ZBTB8B	XLOC_012216	209	coding	noncoding	noncoding	noncoding
XM_011541327.3	ZBTB8OS	XLOC_012219	1506	coding	coding	coding	coding
MSTRG.2914.1		XLOC_012223	270	coding	noncoding	noncoding	noncoding
MSTRG.2918.1		XLOC_012224	244	coding	noncoding	noncoding	noncoding
MSTRG.2945.1	KIAA1522	XLOC_012227	335	coding	noncoding	noncoding	noncoding
MSTRG.2947.9	YARS	XLOC_012229	1288	coding	coding	noncoding	noncoding
MSTRG.2928.1		XLOC_012237	294	coding	noncoding	noncoding	noncoding
MSTRG.2966.1		XLOC_012246	255	coding	noncoding	noncoding	noncoding
MSTRG.2976.1		XLOC_012267	318	coding	noncoding	noncoding	noncoding
MSTRG.2981.1		XLOC_012270	312	coding	noncoding	noncoding	noncoding
MSTRG.3142.1	CSMD2	XLOC_012309	308	coding	noncoding	noncoding	noncoding
MSTRG.3103.1		XLOC_012329	241	coding	noncoding	noncoding	noncoding

MSTRG.3176.3	ZMYM1	XLOC_012339	1969	coding	coding	noncoding	coding
MSTRG.3157.1		XLOC_012340	291	coding	coding	noncoding	noncoding
MSTRG.3158.1	LOC105378644	XLOC_012341	253	coding	noncoding	noncoding	noncoding
XM_017002053.2	SFPQ	XLOC_012344	8892	coding	coding	coding	coding
MSTRG.3220.1	KIAA0319L	XLOC_012355	258	coding	noncoding	noncoding	noncoding
MSTRG.3236.1	TFAP2E	XLOC_012365	240	coding	noncoding	noncoding	noncoding
MSTRG.3246.1	PSMB2	XLOC_012372	245	coding	noncoding	noncoding	noncoding
MSTRG.3247.1	PSMB2	XLOC_012373	297	coding	noncoding	noncoding	noncoding
MSTRG.3192.1		XLOC_012376	337	coding	noncoding	noncoding	noncoding
NM_001348691.1	C1orf216	XLOC_012377	2862	coding	coding	coding	coding
MSTRG.3292.2	AGO3	XLOC_012383	3234	coding	coding	noncoding	coding
MSTRG.3350.6	THRAP3	XLOC_012398	4306	coding	coding	coding	noncoding
MSTRG.3356.1	SH3D21	XLOC_012401	286	coding	noncoding	noncoding	noncoding
MSTRG.3271.1		XLOC_012417	316	coding	noncoding	noncoding	noncoding
MSTRG.3273.1		XLOC_012419	273	coding	noncoding	noncoding	noncoding
NR_073091.1	MEAF6	XLOC_012460	4846	coding	coding	coding	coding
NR_073090.1	MEAF6	XLOC_012460	4876	coding	coding	coding	coding
MSTRG.3406.1	DNALI1	XLOC_012466	215	coding	noncoding	noncoding	noncoding
MSTRG.3415.1	CDCA8	XLOC_012474	411	coding	coding	coding	noncoding
MSTRG.3438.1	INPP5B	XLOC_012486	598	coding	noncoding	noncoding	noncoding
MSTRG.3489.1	LOC105378657	XLOC_012513	209	coding	noncoding	noncoding	noncoding
MSTRG.3476.1		XLOC_012518	293	coding	noncoding	noncoding	noncoding
MSTRG.3477.1		XLOC_012519	313	coding	noncoding	noncoding	noncoding
MSTRG.3499.1		XLOC_012523	284	coding	noncoding	noncoding	noncoding
MSTRG.3528.1	GJA9-MYCBP	XLOC_012535	258	coding	noncoding	noncoding	noncoding
MSTRG.3535.1	RHBDL2	XLOC_012538	309	coding	noncoding	noncoding	noncoding
MSTRG.3506.1		XLOC_012539	302	coding	noncoding	noncoding	noncoding
MSTRG.3508.1		XLOC_012540	257	coding	noncoding	noncoding	noncoding
MSTRG.3509.1		XLOC_012541	282	coding	noncoding	noncoding	noncoding
MSTRG.3644.1		XLOC_012548	273	coding	noncoding	noncoding	noncoding
MSTRG.3666.8	MACF1	XLOC_012563	17653	coding	coding	noncoding	coding
MSTRG.3666.11	MACF1	XLOC_012563	10941	coding	coding	coding	noncoding
MSTRG.3666.17	MACF1	XLOC_012563	3689	coding	coding	noncoding	noncoding
MSTRG.3666.21	MACF1	XLOC_012563	23075	coding	coding	coding	coding
MSTRG.3539.1	BMP8A	XLOC_012567	246	coding	noncoding	noncoding	noncoding
MSTRG.3553.1		XLOC_012575	247	coding	noncoding	noncoding	noncoding
MSTRG.3561.1		XLOC_012580	303	coding	noncoding	noncoding	noncoding
XM_005270954.2	TRIT1	XLOC_012589	2353	coding	coding	coding	coding
MSTRG.3600.1	TRIT1	XLOC_012593	305	coding	noncoding	noncoding	noncoding
MSTRG.3628.9	PPT1	XLOC_012601	2578	coding	coding	coding	coding
MSTRG.3633.3	ZMPSTE24	XLOC_012604	2855	coding	coding	noncoding	noncoding
MSTRG.3726.1		XLOC_012628	265	coding	noncoding	noncoding	noncoding
NR_135092.1	SCMH1	XLOC_012651	3401	coding	coding	coding	coding

MSTRG.3797.1	SCMH1	XLOC_012661	443	coding	noncoding	noncoding	noncoding
MSTRG.3804.1	SCMH1	XLOC_012663	228	coding	noncoding	noncoding	noncoding
MSTRG.4156.1	HIVEP3	XLOC_012680	249	coding	noncoding	noncoding	noncoding
MSTRG.4195.1	HIVEP3	XLOC_012688	242	coding	noncoding	noncoding	noncoding
MSTRG.3859.2	C1orf50	XLOC_012756	732	coding	coding	noncoding	noncoding
NM_199342.4	SVBP	XLOC_012761	807	coding	coding	noncoding	noncoding
MSTRG.3874.2	SVBP	XLOC_012761	3979	coding	coding	coding	noncoding
MSTRG.3865.1		XLOC_012767	211	coding	noncoding	noncoding	noncoding
MSTRG.3882.1		XLOC_012768	383	coding	noncoding	noncoding	noncoding
MSTRG.3895.1		XLOC_012772	254	coding	noncoding	noncoding	noncoding
MSTRG.3951.1	CFAP57	XLOC_012780	292	coding	noncoding	noncoding	noncoding
MSTRG.3903.1	TMEM125	XLOC_012781	284	coding	coding	noncoding	noncoding
MSTRG.3904.1		XLOC_012784	370	coding	noncoding	noncoding	noncoding
MSTRG.3954.1	TIE1	XLOC_012786	245	coding	coding	noncoding	noncoding
MSTRG.3945.1		XLOC_012787	266	coding	noncoding	noncoding	noncoding
MSTRG.3973.1	PTPRF	XLOC_012809	561	coding	coding	coding	noncoding
MSTRG.3966.1		XLOC_012813	293	coding	noncoding	noncoding	noncoding
MSTRG.4029.2	LOC107984948	XLOC_012832	915	coding	noncoding	noncoding	noncoding
MSTRG.4244.5	LOC107984950	XLOC_012857	1916	coding	coding	coding	coding
MSTRG.4272.3	PTCH2	XLOC_012873	313	coding	noncoding	noncoding	noncoding
MSTRG.4274.1		XLOC_012875	249	coding	noncoding	noncoding	noncoding
MSTRG.4310.1	ZSWIM5	XLOC_012885	258	coding	noncoding	noncoding	noncoding
MSTRG.4289.1		XLOC_012889	462	coding	noncoding	noncoding	noncoding
MSTRG.4293.1		XLOC_012893	253	coding	noncoding	noncoding	noncoding
MSTRG.4297.1		XLOC_012896	269	coding	noncoding	noncoding	noncoding
MSTRG.4368.1	TESK2	XLOC_012906	240	coding	noncoding	noncoding	noncoding
XM_017000457.1	CCDC17	XLOC_012914	2293	coding	noncoding	coding	coding
MSTRG.4371.10	GPBP1L1	XLOC_012915	3422	coding	coding	coding	coding
MSTRG.4380.1	IPP	XLOC_012917	296	coding	noncoding	noncoding	noncoding
MSTRG.4369.1		XLOC_012918	241	coding	noncoding	noncoding	noncoding
MSTRG.4406.1	PIK3R3	XLOC_012933	239	coding	noncoding	noncoding	noncoding
MSTRG.4407.1	PIK3R3	XLOC_012934	227	coding	noncoding	noncoding	noncoding
MSTRG.4384.1		XLOC_012942	239	coding	noncoding	noncoding	noncoding
MSTRG.4435.1		XLOC_012948	264	coding	noncoding	noncoding	noncoding
MSTRG.4454.1	DMBX1	XLOC_012958	257	coding	noncoding	noncoding	noncoding
XM_024450514.1	MKNK1	XLOC_012961	2930	coding	coding	coding	coding
MSTRG.4476.1		XLOC_012976	279	coding	noncoding	noncoding	noncoding
MSTRG.4477.1	CYP4Z2P	XLOC_012977	407	coding	noncoding	noncoding	noncoding
MSTRG.4500.1	CYP4X1	XLOC_012984	243	coding	noncoding	noncoding	noncoding
MSTRG.4504.1	CYP4X1	XLOC_012987	242	coding	noncoding	noncoding	noncoding
MSTRG.4511.1		XLOC_012990	393	coding	coding	noncoding	noncoding
MSTRG.4515.1		XLOC_012993	226	coding	noncoding	noncoding	noncoding
XR_001737370.1	STIL	XLOC_013002	5138	coding	coding	coding	coding

MSTRG.4587.1	STIL	XLOC_013004	220	coding	noncoding	noncoding	noncoding
MSTRG.4588.1		XLOC_013005	277	coding	noncoding	noncoding	noncoding
MSTRG.4535.1		XLOC_013017	401	coding	noncoding	noncoding	noncoding
MSTRG.4562.1		XLOC_013025	267	coding	noncoding	noncoding	noncoding
MSTRG.4568.1		XLOC_013034	287	coding	noncoding	noncoding	noncoding
MSTRG.4662.1	SPATA6	XLOC_013050	251	coding	noncoding	noncoding	noncoding
MSTRG.4665.1	SPATA6	XLOC_013053	464	coding	noncoding	noncoding	noncoding
MSTRG.4671.4	SPATA6	XLOC_013058	1206	coding	coding	noncoding	noncoding
XM_011542310.2	AGBL4	XLOC_013067	2427	coding	coding	coding	coding
MSTRG.4716.1	AGBL4	XLOC_013077	295	coding	noncoding	noncoding	noncoding
MSTRG.4727.1	LOC107984954	XLOC_013083	307	coding	coding	noncoding	noncoding
MSTRG.4730.1	AGBL4	XLOC_013084	295	coding	noncoding	coding	noncoding
MSTRG.4650.1	ELAVL4	XLOC_013100	276	coding	noncoding	noncoding	noncoding
MSTRG.4848.1	FAF1	XLOC_013124	223	coding	noncoding	noncoding	noncoding
MSTRG.4753.1		XLOC_013136	303	coding	noncoding	noncoding	noncoding
MSTRG.4759.1	C1orf185	XLOC_013139	270	coding	noncoding	noncoding	noncoding
NR_147076.1	LINC01562	XLOC_013142	2366	coding	noncoding	noncoding	noncoding
NM_001981.3	EPS15	XLOC_013151	5163	coding	coding	coding	coding
XM_005270618.3	EPS15	XLOC_013151	4937	coding	coding	coding	coding
MSTRG.4800.1	EPS15	XLOC_013167	282	coding	noncoding	noncoding	noncoding
MSTRG.4944.13	MIR761	XLOC_013177	1012	coding	coding	coding	coding
NM_002867.4	RAB3B	XLOC_013179	12780	coding	coding	coding	coding
MSTRG.4814.1	RAB3B	XLOC_013181	414	coding	noncoding	noncoding	noncoding
MSTRG.4816.1		XLOC_013182	248	coding	noncoding	noncoding	noncoding
XM_017001389.2	ORC1	XLOC_013203	3202	coding	coding	coding	coding
MSTRG.4889.1		XLOC_013215	269	coding	noncoding	noncoding	noncoding
MSTRG.4911.1		XLOC_013222	3119	coding	noncoding	noncoding	noncoding
MSTRG.4914.1		XLOC_013227	235	coding	noncoding	noncoding	noncoding
MSTRG.4939.1	ZYG11A	XLOC_013228	237	coding	noncoding	noncoding	noncoding
XM_011542002.2	SLC1A7	XLOC_013235	2372	coding	coding	coding	coding
XR_001738058.1	LINC02812	XLOC_013249	3768	coding	noncoding	coding	noncoding
MSTRG.5068.1		XLOC_013292	244	coding	noncoding	noncoding	noncoding
MSTRG.5091.1	MROH7	XLOC_013303	286	coding	noncoding	noncoding	noncoding
MSTRG.5092.1	MROH7-TTC4	XLOC_013304	385	coding	noncoding	noncoding	noncoding
MSTRG.5251.1	USP24	XLOC_013336	306	coding	noncoding	noncoding	noncoding
MSTRG.5152.1		XLOC_013363	245	coding	noncoding	noncoding	noncoding
MSTRG.5195.1	LOC105378737	XLOC_013367	258	coding	noncoding	noncoding	noncoding
XR_001738063.1	LOC105378741	XLOC_013368	6829	coding	noncoding	noncoding	noncoding
MSTRG.5199.1	LOC105378741	XLOC_013371	274	coding	noncoding	noncoding	noncoding
MSTRG.5159.1		XLOC_013374	256	coding	noncoding	noncoding	noncoding
MSTRG.5211.1		XLOC_013381	368	coding	noncoding	noncoding	noncoding
MSTRG.5216.1	PLPP3	XLOC_013384	386	coding	noncoding	noncoding	noncoding
MSTRG.5212.1		XLOC_013389	251	coding	noncoding	noncoding	noncoding

MSTRG.5225.1	PRKAA2	XLOC_013392	279	coding	noncoding	noncoding	noncoding
MSTRG.5403.1	DAB1	XLOC_013408	267	coding	noncoding	noncoding	noncoding
MSTRG.5497.1	DAB1	XLOC_013425	316	coding	noncoding	noncoding	noncoding
MSTRG.5335.1		XLOC_013436	269	coding	noncoding	noncoding	noncoding
MSTRG.5355.1		XLOC_013445	213	coding	noncoding	noncoding	noncoding
MSTRG.5564.1		XLOC_013525	257	coding	noncoding	noncoding	noncoding
NM_152377.3	C1orf87	XLOC_013563	2028	coding	coding	coding	coding
MSTRG.5675.1	C1orf87	XLOC_013564	246	coding	noncoding	noncoding	noncoding
MSTRG.5687.1		XLOC_013571	276	coding	noncoding	noncoding	noncoding
MSTRG.5689.1		XLOC_013572	211	coding	noncoding	noncoding	noncoding
MSTRG.5703.1	LOC101926964	XLOC_013579	268	coding	noncoding	noncoding	noncoding
MSTRG.5764.6	NFIA	XLOC_013591	1956	coding	coding	noncoding	noncoding
MSTRG.5738.1		XLOC_013598	284	coding	noncoding	noncoding	noncoding
MSTRG.5753.1		XLOC_013615	317	coding	noncoding	noncoding	noncoding
MSTRG.5756.1		XLOC_013616	405	coding	noncoding	noncoding	noncoding
MSTRG.5837.1	DOCK7	XLOC_013623	510	coding	noncoding	noncoding	noncoding
MSTRG.5786.1		XLOC_013631	237	coding	noncoding	noncoding	noncoding
MSTRG.5789.1		XLOC_013633	213	coding	noncoding	noncoding	noncoding
MSTRG.5814.1		XLOC_013650	288	coding	coding	noncoding	noncoding
MSTRG.5939.1	ROR1	XLOC_013686	260	coding	noncoding	noncoding	noncoding
XR_947459.2	LOC105378773	XLOC_013695	5783	coding	coding	coding	coding
MSTRG.5901.1	UBE2U	XLOC_013696	230	coding	noncoding	noncoding	noncoding
MSTRG.5903.1	UBE2U	XLOC_013697	256	coding	noncoding	noncoding	noncoding
MSTRG.5890.1		XLOC_013706	299	coding	noncoding	noncoding	noncoding
MSTRG.6114.1	RAVER2	XLOC_013733	314	coding	noncoding	noncoding	noncoding
MSTRG.6024.1		XLOC_013745	264	coding	noncoding	noncoding	noncoding
MSTRG.6085.1		XLOC_013755	216	coding	coding	noncoding	noncoding
MSTRG.6300.1	PDE4B	XLOC_013829	249	coding	noncoding	noncoding	noncoding
MSTRG.6324.1	PDE4B	XLOC_013846	240	coding	noncoding	noncoding	noncoding
MSTRG.6222.1		XLOC_013899	304	coding	noncoding	noncoding	noncoding
MSTRG.6219.1	LINC01702	XLOC_013904	310	coding	noncoding	noncoding	noncoding
MSTRG.6215.1		XLOC_013908	262	coding	noncoding	noncoding	noncoding
MSTRG.6467.1	GNG12-AS1	XLOC_013914	262	coding	noncoding	noncoding	noncoding
NM_001193334.1	WLS	XLOC_013920	2468	coding	coding	coding	coding
MSTRG.6374.1		XLOC_013943	273	coding	noncoding	noncoding	noncoding
MSTRG.6378.1		XLOC_013944	251	coding	noncoding	noncoding	noncoding
MSTRG.6402.1		XLOC_013955	364	coding	noncoding	coding	noncoding
MSTRG.6404.1		XLOC_013956	354	coding	noncoding	noncoding	noncoding
MSTRG.6428.1		XLOC_013968	249	coding	noncoding	noncoding	noncoding
MSTRG.6437.1		XLOC_013971	325	coding	noncoding	noncoding	noncoding
MSTRG.6447.1		XLOC_013975	248	coding	coding	noncoding	noncoding
MSTRG.6604.1	LRRC7	XLOC_013986	228	coding	noncoding	noncoding	noncoding
MSTRG.6506.1	ANKRD13C	XLOC_014021	283	coding	noncoding	noncoding	noncoding

MSTRG.6509.1		XLOC_014041	221	coding	noncoding	noncoding	noncoding
NM_198719.2	PTGER3	XLOC_014051	2360	coding	coding	coding	coding
XM_011541200.3	NEGR1	XLOC_014073	2659	coding	coding	coding	coding
MSTRG.6731.1	NEGR1	XLOC_014085	218	coding	noncoding	noncoding	noncoding
MSTRG.6790.1	LOC105378797	XLOC_014101	420	coding	coding	noncoding	noncoding
MSTRG.6658.1	LOC105378798	XLOC_014105	235	coding	noncoding	noncoding	noncoding
MSTRG.6693.1		XLOC_014126	265	coding	coding	noncoding	noncoding
XM_017000274.1	ERICH3	XLOC_014147	7816	coding	coding	coding	coding
MSTRG.6858.1	SLC44A5	XLOC_014161	308	coding	noncoding	noncoding	noncoding
MSTRG.6859.1	SLC44A5	XLOC_014162	270	coding	coding	noncoding	noncoding
MSTRG.6866.1	SLC44A5	XLOC_014166	238	coding	noncoding	noncoding	noncoding
MSTRG.6869.3	ACADM	XLOC_014168	1186	coding	coding	noncoding	noncoding
MSTRG.6911.1	ST6GALNAC5	XLOC_014195	240	coding	noncoding	noncoding	noncoding
MSTRG.6922.2	PIGK	XLOC_014201	431	coding	coding	noncoding	noncoding
MSTRG.6944.1		XLOC_014240	298	coding	noncoding	noncoding	noncoding
XM_017000723.2	USP33	XLOC_014241	4207	coding	coding	coding	coding
MSTRG.6947.14	USP33	XLOC_014241	2858	coding	coding	coding	coding
MSTRG.7178.1		XLOC_014266	478	coding	noncoding	noncoding	noncoding
MSTRG.6993.1		XLOC_014267	238	coding	noncoding	noncoding	noncoding
MSTRG.6998.1	GIPC2	XLOC_014268	253	coding	noncoding	noncoding	noncoding
MSTRG.7001.1		XLOC_014271	230	coding	noncoding	noncoding	noncoding
MSTRG.7013.1	MGC27382	XLOC_014275	297	coding	noncoding	noncoding	noncoding
MSTRG.7017.1		XLOC_014280	226	coding	noncoding	noncoding	noncoding
MSTRG.7036.1	LOC107984998	XLOC_014292	269	coding	noncoding	noncoding	noncoding
MSTRG.7044.1		XLOC_014300	308	coding	noncoding	noncoding	noncoding
MSTRG.7059.1		XLOC_014302	353	coding	noncoding	noncoding	noncoding
MSTRG.7069.1		XLOC_014308	247	coding	noncoding	noncoding	noncoding
MSTRG.7070.1		XLOC_014309	264	coding	coding	noncoding	noncoding
MSTRG.7076.1		XLOC_014312	265	coding	noncoding	noncoding	noncoding
MSTRG.7085.1		XLOC_014319	206	coding	noncoding	noncoding	noncoding
MSTRG.7088.1		XLOC_014321	265	coding	noncoding	noncoding	noncoding
MSTRG.7101.1	LINC01781	XLOC_014326	274	coding	noncoding	noncoding	noncoding
MSTRG.7109.1		XLOC_014337	269	coding	noncoding	noncoding	noncoding
MSTRG.7198.1	ADGRL2	XLOC_014359	268	coding	noncoding	noncoding	noncoding
MSTRG.7205.1	ADGRL2	XLOC_014363	430	coding	noncoding	noncoding	noncoding
MSTRG.7206.1	ADGRL2	XLOC_014364	274	coding	noncoding	noncoding	noncoding
MSTRG.7460.1	LOC107985037	XLOC_014398	244	coding	noncoding	noncoding	noncoding
MSTRG.7476.1	LOC107985037	XLOC_014401	219	coding	noncoding	noncoding	noncoding
MSTRG.7334.4	PRKACB	XLOC_014456	1486	coding	coding	noncoding	noncoding
MSTRG.7253.1		XLOC_014468	205	coding	noncoding	noncoding	noncoding
MSTRG.7630.1	SYDE2	XLOC_014527	264	coding	noncoding	noncoding	noncoding
MSTRG.7685.1	ZNHIT6	XLOC_014554	284	coding	noncoding	noncoding	noncoding
MSTRG.7709.1	COL24A1	XLOC_014558	260	coding	coding	noncoding	noncoding

MSTRG.7697.1	LINC02795	XLOC_014569	224	coding	noncoding	noncoding	noncoding
MSTRG.7765.1	ODF2L	XLOC_014584	241	coding	noncoding	noncoding	noncoding
MSTRG.7728.1		XLOC_014589	261	coding	noncoding	noncoding	noncoding
MSTRG.7735.1		XLOC_014590	244	coding	noncoding	noncoding	noncoding
MSTRG.7770.1	CLCA4	XLOC_014592	248	coding	noncoding	noncoding	noncoding
MSTRG.7743.1		XLOC_014597	233	coding	noncoding	noncoding	noncoding
MSTRG.7805.1		XLOC_014609	233	coding	noncoding	noncoding	noncoding
MSTRG.7887.1		XLOC_014643	256	coding	noncoding	noncoding	noncoding
MSTRG.8324.1	KYAT3	XLOC_014665	230	coding	noncoding	noncoding	noncoding
MSTRG.8356.1		XLOC_014693	303	coding	noncoding	noncoding	noncoding
MSTRG.8385.1		XLOC_014721	285	coding	coding	noncoding	noncoding
MSTRG.8388.1		XLOC_014724	422	coding	noncoding	noncoding	noncoding
MSTRG.7985.1	LOC105378844	XLOC_014766	304	coding	noncoding	noncoding	noncoding
MSTRG.8092.1		XLOC_014868	254	coding	noncoding	noncoding	noncoding
MSTRG.8094.1		XLOC_014870	284	coding	noncoding	noncoding	noncoding
MSTRG.8215.5	CDC7	XLOC_014877	245	coding	coding	noncoding	noncoding
MSTRG.8203.1		XLOC_014886	277	coding	noncoding	noncoding	noncoding
MSTRG.8207.1		XLOC_014889	235	coding	noncoding	noncoding	noncoding
MSTRG.8249.1	TGFBR3	XLOC_014891	231	coding	noncoding	noncoding	noncoding
MSTRG.8258.1	TGFBR3	XLOC_014900	368	coding	noncoding	noncoding	noncoding
MSTRG.8260.1	TGFBR3	XLOC_014902	263	coding	noncoding	noncoding	noncoding
MSTRG.8474.1	EVI5	XLOC_014951	284	coding	coding	noncoding	noncoding
MSTRG.8484.1	EVI5	XLOC_014961	265	coding	noncoding	noncoding	noncoding
MSTRG.8440.1		XLOC_014969	385	coding	noncoding	noncoding	noncoding
MSTRG.8441.1		XLOC_014970	300	coding	noncoding	noncoding	noncoding
MSTRG.8640.8	CCDC18	XLOC_014978	1261	coding	noncoding	noncoding	noncoding
MSTRG.8662.6	DR1	XLOC_014997	4554	coding	noncoding	noncoding	noncoding
NM_001308253.1	GCLM	XLOC_015030	4985	coding	noncoding	coding	coding
MSTRG.8528.1	ABCA4	XLOC_015043	227	coding	noncoding	noncoding	noncoding
NR_160780.1	SLC44A3-AS1	XLOC_015066	3373	coding	noncoding	noncoding	noncoding
XM_017000245.2	CNN3	XLOC_015076	4265	coding	coding	coding	coding
MSTRG.8773.4	TLCD4	XLOC_015087	1831	coding	coding	coding	coding
MSTRG.8773.17	TLCD4	XLOC_015087	1202	coding	coding	coding	coding
MSTRG.8702.1		XLOC_015101	257	coding	noncoding	noncoding	noncoding
MSTRG.8729.1	LINC01787	XLOC_015110	255	coding	coding	noncoding	noncoding
MSTRG.8738.1	LOC105378866	XLOC_015116	263	coding	noncoding	noncoding	noncoding
MSTRG.8742.1		XLOC_015119	226	coding	coding	noncoding	noncoding
MSTRG.8743.1		XLOC_015120	271	coding	noncoding	noncoding	noncoding
MSTRG.8789.6	PTBP2	XLOC_015124	473	coding	coding	noncoding	noncoding
MSTRG.9380.7	DPYD	XLOC_015129	4105	coding	coding	coding	coding
MSTRG.9509.1	DPYD	XLOC_015253	262	coding	noncoding	noncoding	noncoding
MSTRG.8807.1		XLOC_015293	222	coding	noncoding	noncoding	noncoding
MSTRG.8827.1		XLOC_015303	426	coding	noncoding	noncoding	noncoding

MSTRG.8834.1		XLOC_015308	286	coding	noncoding	noncoding	noncoding
MSTRG.8844.1	SNX7	XLOC_015310	325	coding	noncoding	coding	noncoding
MSTRG.8840.1		XLOC_015315	301	coding	noncoding	noncoding	noncoding
MSTRG.8851.1		XLOC_015324	227	coding	noncoding	noncoding	noncoding
MSTRG.8874.1		XLOC_015327	247	coding	noncoding	noncoding	noncoding
MSTRG.8875.1		XLOC_015330	285	coding	noncoding	noncoding	noncoding
MSTRG.8878.1		XLOC_015332	430	coding	noncoding	noncoding	noncoding
MSTRG.8883.1		XLOC_015335	256	coding	noncoding	noncoding	noncoding
MSTRG.8901.1		XLOC_015345	243	coding	noncoding	noncoding	noncoding
MSTRG.9005.4	CDC14A	XLOC_015377	3916	coding	coding	coding	noncoding
MSTRG.8929.1		XLOC_015387	267	coding	noncoding	coding	noncoding
MSTRG.8936.1		XLOC_015392	305	coding	noncoding	noncoding	noncoding
MSTRG.8947.1		XLOC_015397	306	coding	noncoding	noncoding	noncoding
MSTRG.8948.1		XLOC_015398	372	coding	coding	noncoding	noncoding
MSTRG.9054.3	S1PR1	XLOC_015409	2014	coding	coding	coding	noncoding
MSTRG.9059.1		XLOC_015411	290	coding	noncoding	noncoding	noncoding
MSTRG.9078.1	LINC01709	XLOC_015423	263	coding	noncoding	noncoding	noncoding
MSTRG.9080.1		XLOC_015424	298	coding	noncoding	noncoding	noncoding
XM_017000240.1	OLFM3	XLOC_015425	5909	coding	coding	coding	coding
MSTRG.9087.1		XLOC_015434	311	coding	noncoding	noncoding	noncoding
MSTRG.9107.1		XLOC_015438	252	coding	noncoding	noncoding	noncoding
MSTRG.9123.1	LOC107985096	XLOC_015445	248	coding	coding	coding	noncoding
MSTRG.9180.1		XLOC_015464	209	coding	noncoding	noncoding	noncoding
MSTRG.9196.1		XLOC_015468	355	coding	noncoding	noncoding	noncoding
MSTRG.9223.1		XLOC_015483	298	coding	noncoding	noncoding	noncoding
MSTRG.9249.1		XLOC_015488	354	coding	noncoding	noncoding	noncoding
MSTRG.9271.1	LINC01677	XLOC_015499	310	coding	noncoding	noncoding	noncoding
MSTRG.9293.1	LOC105378887	XLOC_015508	410	coding	noncoding	noncoding	noncoding
MSTRG.9309.1		XLOC_015515	352	coding	noncoding	noncoding	noncoding
MSTRG.9320.1		XLOC_015521	257	coding	noncoding	noncoding	noncoding
MSTRG.9322.1		XLOC_015523	262	coding	noncoding	noncoding	noncoding
MSTRG.9637.1	MYBPHL	XLOC_015660	283	coding	noncoding	noncoding	noncoding
XR_947695.3	LOC105378897	XLOC_015712	2734	coding	coding	noncoding	noncoding
MSTRG.9899.1		XLOC_015768	277	coding	noncoding	noncoding	noncoding
MSTRG.10044.8	LOC107985184	XLOC_015819	4773	coding	coding	coding	noncoding
XM_017001245.2	KCND3	XLOC_015825	7130	coding	coding	coding	coding
MSTRG.9988.1		XLOC_015831	270	coding	noncoding	noncoding	noncoding
MSTRG.9996.1		XLOC_015836	462	coding	coding	noncoding	noncoding
XM_011541628.2	ST7L	XLOC_015848	2616	coding	coding	noncoding	coding
MSTRG.10145.4	MOV10	XLOC_015850	758	coding	coding	noncoding	noncoding
MSTRG.10089.1		XLOC_015854	264	coding	noncoding	noncoding	noncoding
MSTRG.10092.1		XLOC_015855	221	coding	noncoding	noncoding	noncoding
XR_001738191.2	LOC105378911	XLOC_015858	1739	coding	noncoding	coding	noncoding

MSTRG.10167.1	MAGI3	XLOC_015882	304	coding	noncoding	noncoding	noncoding
MSTRG.10169.1	MAGI3	XLOC_015884	285	coding	noncoding	noncoding	noncoding
NM_001308297.1	PTPN22	XLOC_015896	3582	coding	coding	coding	coding
XM_017001004.1	PTPN22	XLOC_015896	2483	coding	coding	coding	coding
MSTRG.10235.1	AP4B1-AS1	XLOC_015897	262	coding	noncoding	noncoding	noncoding
MSTRG.10244.1	AP4B1-AS1	XLOC_015904	245	coding	noncoding	noncoding	noncoding
MSTRG.10186.1		XLOC_015916	219	coding	noncoding	noncoding	noncoding
MSTRG.10187.1		XLOC_015918	290	coding	coding	noncoding	noncoding
MSTRG.10282.1	TRIM33	XLOC_015937	318	coding	noncoding	noncoding	noncoding
MSTRG.10218.1		XLOC_015951	216	coding	noncoding	noncoding	noncoding
MSTRG.10260.1	DENND2C	XLOC_015955	243	coding	noncoding	noncoding	noncoding
MSTRG.10265.1	DENND2C	XLOC_015958	203	coding	noncoding	noncoding	noncoding
NM_001130523.3	CSDE1	XLOC_015962	4151	coding	coding	coding	coding
MSTRG.10294.1		XLOC_015969	287	coding	noncoding	noncoding	noncoding
MSTRG.10326.1	SYCP1	XLOC_015977	270	coding	noncoding	noncoding	noncoding
MSTRG.10369.1		XLOC_016010	241	coding	coding	noncoding	noncoding
MSTRG.10390.1		XLOC_016033	254	coding	noncoding	noncoding	noncoding
MSTRG.10545.1	LOC101929099	XLOC_016084	259	coding	noncoding	noncoding	noncoding
MSTRG.10577.1		XLOC_016104	249	coding	coding	noncoding	noncoding
MSTRG.10613.1		XLOC_016134	225	coding	noncoding	noncoding	noncoding
MSTRG.10644.1		XLOC_016203	296	coding	coding	noncoding	noncoding
MSTRG.10728.1	LOC105378937	XLOC_016215	308	coding	noncoding	noncoding	noncoding
MSTRG.10729.1	LOC105378937	XLOC_016216	371	coding	noncoding	noncoding	noncoding
NM_001159353.2	REG4	XLOC_016222	2209	coding	noncoding	noncoding	noncoding
XM_017001186.1	NBPF7	XLOC_016224	3338	coding	coding	coding	coding
MSTRG.10849.1		XLOC_016317	289	coding	noncoding	noncoding	noncoding
MSTRG.10947.1		XLOC_016395	273	coding	noncoding	noncoding	noncoding
MSTRG.11086.1	FAM72D	XLOC_016465	262	coding	noncoding	noncoding	noncoding
MSTRG.11212.7	GPR89A	XLOC_016493	302	coding	coding	noncoding	noncoding
NM_144698.5	ANKRD35	XLOC_016501	3359	coding	coding	coding	coding
MSTRG.11133.1	LIX1L-AS1	XLOC_016508	313	coding	noncoding	noncoding	noncoding
MSTRG.11169.1	LOC112268274	XLOC_016540	457	coding	noncoding	noncoding	noncoding
MSTRG.11143.1		XLOC_016548	277	coding	noncoding	noncoding	noncoding
MSTRG.11145.1		XLOC_016549	333	coding	noncoding	noncoding	noncoding
MSTRG.11228.2	NBPF12	XLOC_016553	1573	coding	coding	coding	noncoding
MSTRG.11183.1		XLOC_016561	255	coding	noncoding	noncoding	noncoding
MSTRG.11187.1		XLOC_016565	209	coding	noncoding	noncoding	noncoding
MSTRG.11293.2	CHD1L	XLOC_016576	1891	coding	coding	coding	coding
MSTRG.11239.1	LINC00624	XLOC_016580	246	coding	noncoding	noncoding	noncoding
MSTRG.11331.1	ACP6	XLOC_016603	299	coding	coding	noncoding	noncoding
MSTRG.11267.1	LOC102723321	XLOC_016606	244	coding	coding	noncoding	noncoding
MSTRG.11264.1		XLOC_016609	228	coding	noncoding	noncoding	noncoding
MSTRG.12131.1	LOC101927468	XLOC_016629	279	coding	noncoding	noncoding	noncoding

MSTRG.12200.1	PDE4DIP	XLOC_016682	255	coding	noncoding	noncoding	noncoding
MSTRG.11512.1	HIST2H2AA3	XLOC_016757	732	coding	coding	coding	noncoding
MSTRG.11536.1	LOC105371427	XLOC_016769	366	coding	coding	noncoding	noncoding
MSTRG.11528.1		XLOC_016774	288	coding	noncoding	noncoding	noncoding
XM_005245513.4	ANP32E	XLOC_016775	3480	coding	coding	coding	coding
MSTRG.11529.1		XLOC_016777	322	coding	noncoding	noncoding	noncoding
MSTRG.11580.1	ECM1	XLOC_016786	281	coding	noncoding	noncoding	noncoding
MSTRG.11573.1		XLOC_016787	294	coding	noncoding	noncoding	noncoding
MSTRG.11604.1	ADAMTSL4-AS1	XLOC_016799	276	coding	noncoding	noncoding	noncoding
MSTRG.11658.1	ANXA9	XLOC_016830	267	coding	noncoding	noncoding	noncoding
MSTRG.11659.1	ANXA9	XLOC_016831	213	coding	coding	noncoding	noncoding
MSTRG.11679.1	LYSMD1	XLOC_016845	437	coding	noncoding	noncoding	noncoding
NM_005997.3	VPS72	XLOC_016846	1511	coding	coding	coding	coding
XM_017001486.1	PI4KB	XLOC_016851	4307	coding	coding	coding	coding
NM_001258288.2	SELENBP1	XLOC_016853	1536	coding	coding	coding	coding
MSTRG.11694.1		XLOC_016854	256	coding	noncoding	noncoding	noncoding
XM_017000746.1	POGZ	XLOC_016856	6526	coding	coding	coding	coding
MSTRG.11707.1	CGN	XLOC_016860	231	coding	noncoding	noncoding	noncoding
MSTRG.11718.1	TUFT1	XLOC_016867	225	coding	noncoding	noncoding	noncoding
MSTRG.11708.1		XLOC_016870	439	coding	noncoding	noncoding	noncoding
MSTRG.11729.1		XLOC_016872	279	coding	noncoding	noncoding	noncoding
MSTRG.11794.1	LOC105371441	XLOC_016902	259	coding	noncoding	noncoding	noncoding
NM_007113.3	TCHH	XLOC_016913	6995	coding	coding	coding	coding
MSTRG.11826.1		XLOC_016922	276	coding	noncoding	noncoding	noncoding
MSTRG.11828.1		XLOC_016923	238	coding	coding	noncoding	noncoding
MSTRG.11864.1		XLOC_016953	275	coding	noncoding	noncoding	noncoding
MSTRG.11906.1		XLOC_016983	261	coding	coding	coding	noncoding
MSTRG.11907.1		XLOC_016984	293	coding	noncoding	noncoding	noncoding
MSTRG.11929.1	NPR1	XLOC_016998	307	coding	noncoding	noncoding	noncoding
MSTRG.11969.1	GATAD2B	XLOC_017003	280	coding	noncoding	noncoding	noncoding
MSTRG.11971.1	GATAD2B	XLOC_017005	222	coding	noncoding	noncoding	noncoding
XM_011510220.2	DENND4B	XLOC_017011	5508	coding	coding	coding	coding
MSTRG.11994.1	RAB13	XLOC_017024	269	coding	noncoding	noncoding	noncoding
MSTRG.12008.18	UBAP2L	XLOC_017030	3802	coding	coding	coding	coding
MSTRG.11960.1		XLOC_017034	278	coding	noncoding	noncoding	noncoding
MSTRG.12038.5	SHE	XLOC_017036	5374	coding	coding	coding	coding
MSTRG.12050.1	TDRD10	XLOC_017039	225	coding	noncoding	noncoding	noncoding
NM_001025107.3	ADAR	XLOC_017043	6532	coding	coding	coding	coding
MSTRG.12057.1	ADAR	XLOC_017045	290	coding	noncoding	noncoding	noncoding
MSTRG.12004.1		XLOC_017050	222	coding	noncoding	noncoding	noncoding
MSTRG.12036.1		XLOC_017061	236	coding	noncoding	noncoding	noncoding
MSTRG.12073.1	PMVK	XLOC_017064	272	coding	noncoding	noncoding	noncoding
XM_011509189.2	DCST2	XLOC_017072	2285	coding	coding	coding	coding

MSTRG.12089.1		XLOC_017080	270	coding	noncoding	noncoding	noncoding
MSTRG.12090.1		XLOC_017081	256	coding	noncoding	noncoding	noncoding
MSTRG.12092.1		XLOC_017085	250	coding	noncoding	noncoding	noncoding
MSTRG.12497.1	ASH1L	XLOC_017108	254	coding	noncoding	noncoding	noncoding
MSTRG.12510.1		XLOC_017119	203	coding	noncoding	noncoding	noncoding
MSTRG.12514.1		XLOC_017121	270	coding	noncoding	noncoding	noncoding
MSTRG.12520.17	DAP3	XLOC_017126	1443	coding	coding	noncoding	noncoding
MSTRG.12526.1	GON4L	XLOC_017132	256	coding	noncoding	noncoding	noncoding
NM_001256820.2	RIT1	XLOC_017134	3241	coding	coding	coding	coding
MSTRG.12350.1		XLOC_017139	300	coding	noncoding	noncoding	noncoding
MSTRG.12355.1	ARHGEF2	XLOC_017142	232	coding	noncoding	noncoding	noncoding
MSTRG.12343.1	LOC105371729	XLOC_017149	275	coding	noncoding	noncoding	noncoding
MSTRG.12381.1	CCT3	XLOC_017176	313	coding	noncoding	coding	noncoding
XM_005245170.3	MEF2D	XLOC_017183	5986	coding	coding	coding	coding
MSTRG.12396.1	MEF2D	XLOC_017184	281	coding	noncoding	noncoding	noncoding
MSTRG.12397.1	MEF2D	XLOC_017185	298	coding	noncoding	noncoding	noncoding
XR_922175.3	LOC105371453	XLOC_017197	9468	coding	coding	coding	noncoding
XM_005245518.4	ISG20L2	XLOC_017204	2238	coding	noncoding	coding	coding
NM_004494.3	HDGF	XLOC_017206	2309	coding	coding	coding	coding
MSTRG.12577.1	ARHGEF11	XLOC_017226	289	coding	noncoding	noncoding	noncoding
MSTRG.12555.1		XLOC_017246	321	coding	noncoding	noncoding	noncoding
MSTRG.12644.1		XLOC_017255	498	coding	noncoding	noncoding	noncoding
XM_011509125.1	FCRL1	XLOC_017258	5222	coding	coding	coding	coding
MSTRG.12605.1		XLOC_017276	259	coding	noncoding	noncoding	noncoding
NM_001004477.1	OR10X1	XLOC_017293	981	coding	coding	coding	coding
MSTRG.12666.1	SPTA1	XLOC_017296	271	coding	noncoding	noncoding	noncoding
NM_001005327.2	OR6K3	XLOC_017299	948	coding	coding	coding	coding
MSTRG.12669.1		XLOC_017311	202	coding	noncoding	noncoding	noncoding
MSTRG.12827.10	PYHIN1	XLOC_017312	1531	coding	coding	coding	coding
MSTRG.12827.12	PYHIN1	XLOC_017312	4972	coding	coding	noncoding	noncoding
MSTRG.12827.8	IFI16	XLOC_017312	5414	coding	coding	coding	noncoding
MSTRG.12827.11	IFI16	XLOC_017312	5246	coding	coding	coding	noncoding
MSTRG.12827.9	IFI16	XLOC_017312	6205	coding	coding	coding	noncoding
MSTRG.12827.7	LOC105371461	XLOC_017312	8458	coding	coding	coding	noncoding
MSTRG.12704.1		XLOC_017319	229	coding	noncoding	noncoding	noncoding
MSTRG.12733.1		XLOC_017332	295	coding	noncoding	noncoding	noncoding
MSTRG.12775.8	LOC105371464	XLOC_017343	2415	coding	coding	noncoding	noncoding
MSTRG.12761.1	SLAMF9	XLOC_017362	257	coding	noncoding	noncoding	noncoding
MSTRG.12822.1	ATP1A2	XLOC_017370	271	coding	noncoding	noncoding	noncoding
MSTRG.12900.1	LOC107985219	XLOC_017385	251	coding	noncoding	noncoding	noncoding
NM_001184715.2	SLAMF6	XLOC_017402	2583	coding	noncoding	coding	coding
NM_003874.4	CD84	XLOC_017414	8204	coding	noncoding	coding	coding
NM_001184879.2	CD84	XLOC_017414	8255	coding	noncoding	coding	coding

MSTRG.12866.1		XLOC_017416	351	coding	noncoding	noncoding	noncoding
XM_005245625.1	CD48	XLOC_017432	830	coding	coding	coding	coding
XM_017002867.2	CD48	XLOC_017432	808	coding	coding	noncoding	coding
MSTRG.12918.1	LOC105371470	XLOC_017443	278	coding	noncoding	noncoding	noncoding
MSTRG.12970.1	LOC101928372	XLOC_017450	200	coding	noncoding	noncoding	noncoding
MSTRG.13069.1	PFDN2	XLOC_017464	228	coding	noncoding	noncoding	noncoding
NM_001199873.1	B4GALT3	XLOC_017473	2415	coding	coding	coding	coding
XR_001737549.2	ADAMTS4	XLOC_017473	5866	coding	coding	coding	coding
MSTRG.12999.1		XLOC_017482	272	coding	noncoding	noncoding	noncoding
MSTRG.13003.1	PCP4L1	XLOC_017483	275	coding	noncoding	noncoding	noncoding
MSTRG.13005.1	PCP4L1	XLOC_017484	251	coding	noncoding	noncoding	noncoding
XM_017001321.2	MPZ	XLOC_017487	1457	coding	coding	coding	coding
MSTRG.13211.14	FCGR2A	XLOC_017527	3347	coding	coding	noncoding	noncoding
MSTRG.13211.18	FCGR3B	XLOC_017530	2995	coding	coding	coding	coding
MSTRG.13094.4	FCRLA	XLOC_017536	831	coding	coding	noncoding	noncoding
MSTRG.13122.1		XLOC_017578	241	coding	noncoding	noncoding	noncoding
MSTRG.13126.1		XLOC_017580	243	coding	coding	noncoding	noncoding
MSTRG.13247.1	DDR2	XLOC_017595	275	coding	noncoding	noncoding	noncoding
MSTRG.13257.1	DDR2	XLOC_017602	391	coding	noncoding	noncoding	noncoding
MSTRG.13307.1		XLOC_017641	305	coding	noncoding	noncoding	noncoding
MSTRG.13308.1		XLOC_017642	281	coding	noncoding	noncoding	noncoding
MSTRG.13328.1		XLOC_017652	281	coding	noncoding	noncoding	noncoding
MSTRG.13339.1		XLOC_017659	240	coding	noncoding	noncoding	noncoding
MSTRG.13345.1		XLOC_017662	244	coding	noncoding	noncoding	noncoding
NM_177398.4	LMX1A	XLOC_017678	3545	coding	coding	coding	coding
MSTRG.13453.1	LRRC52-AS1	XLOC_017689	229	coding	noncoding	noncoding	noncoding
MSTRG.13479.1	TMCO1	XLOC_017701	478	coding	noncoding	noncoding	noncoding
MSTRG.13480.1	TMCO1	XLOC_017702	275	coding	noncoding	noncoding	noncoding
MSTRG.13489.1		XLOC_017712	277	coding	noncoding	noncoding	noncoding
MSTRG.13490.1		XLOC_017713	270	coding	coding	noncoding	noncoding
MSTRG.13498.1		XLOC_017726	230	coding	coding	noncoding	noncoding
MSTRG.13570.6	POGK	XLOC_017739	3591	coding	coding	coding	coding
MSTRG.13566.1		XLOC_017751	235	coding	noncoding	noncoding	noncoding
MSTRG.13576.1		XLOC_017754	301	coding	noncoding	noncoding	noncoding
MSTRG.13598.1		XLOC_017787	276	coding	noncoding	noncoding	noncoding
MSTRG.13750.1	ADCY10	XLOC_017805	321	coding	noncoding	noncoding	noncoding
MSTRG.13652.1	GPR161	XLOC_017817	260	coding	noncoding	noncoding	noncoding
MSTRG.13653.1	GPR161	XLOC_017818	278	coding	noncoding	noncoding	noncoding
MSTRG.13786.1		XLOC_017864	260	coding	noncoding	noncoding	noncoding
MSTRG.13798.1		XLOC_017866	226	coding	coding	noncoding	noncoding
MSTRG.13812.1		XLOC_017872	277	coding	noncoding	noncoding	noncoding
NM_000130.4	F5	XLOC_017902	9179	coding	coding	coding	coding
MSTRG.13931.1		XLOC_017933	263	coding	noncoding	noncoding	noncoding

NM_001204514.1	KIFAP3	XLOC_017934	3161	coding	coding	coding	coding
MSTRG.13941.1	LINC01681	XLOC_017956	294	coding	noncoding	noncoding	noncoding
MSTRG.14048.1	LOC105371611	XLOC_017981	216	coding	noncoding	noncoding	noncoding
MSTRG.14055.1		XLOC_017994	403	coding	noncoding	noncoding	noncoding
MSTRG.14061.1		XLOC_017997	270	coding	noncoding	noncoding	noncoding
MSTRG.14149.1		XLOC_018027	264	coding	noncoding	noncoding	noncoding
MSTRG.14152.1		XLOC_018029	274	coding	noncoding	noncoding	noncoding
MSTRG.14170.1		XLOC_018038	380	coding	noncoding	noncoding	noncoding
MSTRG.14349.1		XLOC_018050	267	coding	noncoding	noncoding	noncoding
XR_001737395.2	TNFSF4	XLOC_018052	4369	coding	noncoding	noncoding	noncoding
MSTRG.14444.1	TNFSF4	XLOC_018083	271	coding	noncoding	noncoding	noncoding
XM_011509470.2	ANKRD45	XLOC_018091	2122	coding	coding	coding	coding
MSTRG.14272.5	DARS2	XLOC_018100	1599	coding	coding	coding	noncoding
MSTRG.14237.1		XLOC_018136	576	coding	noncoding	noncoding	noncoding
MSTRG.14671.12	RABGAP1L	XLOC_018138	2123	coding	coding	noncoding	noncoding
MSTRG.14303.1	TNN	XLOC_018152	320	coding	coding	noncoding	noncoding
NM_001162894.2	KIAA0040	XLOC_018153	4539	coding	noncoding	coding	coding
MSTRG.14482.1		XLOC_018253	276	coding	noncoding	noncoding	noncoding
MSTRG.14513.1	PAPPA2	XLOC_018266	228	coding	noncoding	noncoding	noncoding
NM_001364856.1	ASTN1	XLOC_018271	7165	coding	coding	coding	coding
XR_922299.3	LOC105371625	XLOC_018284	1332	coding	noncoding	noncoding	noncoding
MSTRG.14550.1		XLOC_018294	206	coding	noncoding	noncoding	noncoding
MSTRG.14608.1	RASAL2	XLOC_018321	270	coding	noncoding	noncoding	noncoding
MSTRG.14644.1		XLOC_018347	371	coding	noncoding	noncoding	noncoding
MSTRG.14794.7	RALGPS2	XLOC_018359	5109	coding	noncoding	noncoding	noncoding
MSTRG.14661.1		XLOC_018366	477	coding	noncoding	noncoding	noncoding
MSTRG.14835.1	ABL2	XLOC_018373	276	coding	noncoding	noncoding	noncoding
MSTRG.14840.1	AXDND1	XLOC_018377	270	coding	noncoding	noncoding	noncoding
MSTRG.14842.1	AXDND1	XLOC_018378	268	coding	noncoding	noncoding	noncoding
MSTRG.14843.1	AXDND1	XLOC_018379	306	coding	noncoding	noncoding	noncoding
MSTRG.14845.1	AXDND1	XLOC_018381	262	coding	noncoding	noncoding	noncoding
MSTRG.14850.1	NPHS2	XLOC_018385	263	coding	noncoding	noncoding	noncoding
MSTRG.14968.1	LOC101928933	XLOC_018400	295	coding	noncoding	noncoding	noncoding
MSTRG.15040.1	ACBD6	XLOC_018429	405	coding	noncoding	noncoding	noncoding
MSTRG.14952.1		XLOC_018434	243	coding	noncoding	noncoding	noncoding
MSTRG.15101.1		XLOC_018507	238	coding	noncoding	noncoding	noncoding
MSTRG.15102.1		XLOC_018508	278	coding	noncoding	noncoding	noncoding
MSTRG.15146.1		XLOC_018533	225	coding	noncoding	noncoding	noncoding
MSTRG.15176.1	RGS8	XLOC_018552	218	coding	noncoding	noncoding	noncoding
MSTRG.15189.1	RGS8	XLOC_018558	254	coding	noncoding	noncoding	noncoding
MSTRG.15241.1		XLOC_018565	252	coding	noncoding	noncoding	noncoding
MSTRG.15272.1	LAMC2	XLOC_018584	281	coding	noncoding	noncoding	noncoding
MSTRG.15278.1	NMNAT2	XLOC_018589	252	coding	noncoding	noncoding	noncoding

MSTRG.15350.1		XLOC_018631	259	coding	noncoding	noncoding	noncoding
MSTRG.15347.1		XLOC_018634	278	coding	noncoding	noncoding	noncoding
NR_135118.1	EDEM3	XLOC_018645	6699	coding	coding	coding	coding
XM_005245499.2	EDEM3	XLOC_018645	6623	coding	coding	coding	coding
MSTRG.15476.4	NIBAN1	XLOC_018665	3678	coding	noncoding	coding	coding
MSTRG.15417.1	LINC01633	XLOC_018703	467	coding	coding	noncoding	noncoding
MSTRG.15419.1		XLOC_018706	221	coding	noncoding	noncoding	noncoding
MSTRG.15585.10	SWT1	XLOC_018711	3106	coding	coding	noncoding	noncoding
MSTRG.15585.13	SWT1	XLOC_018711	1808	coding	noncoding	noncoding	noncoding
MSTRG.15538.1	LOC107985239	XLOC_018751	299	coding	noncoding	noncoding	noncoding
MSTRG.15536.1		XLOC_018773	263	coding	noncoding	noncoding	noncoding
MSTRG.15668.1	ODR4	XLOC_018782	2402	coding	coding	noncoding	noncoding
MSTRG.15639.1		XLOC_018812	297	coding	noncoding	noncoding	noncoding
MSTRG.15646.1		XLOC_018819	268	coding	noncoding	noncoding	noncoding
MSTRG.15657.1		XLOC_018823	254	coding	noncoding	noncoding	noncoding
MSTRG.15694.1		XLOC_018851	214	coding	noncoding	noncoding	noncoding
MSTRG.15710.1		XLOC_018861	306	coding	noncoding	noncoding	noncoding
MSTRG.15711.1		XLOC_018862	260	coding	noncoding	noncoding	noncoding
MSTRG.15714.1		XLOC_018864	236	coding	noncoding	noncoding	noncoding
MSTRG.15740.1		XLOC_018877	300	coding	coding	noncoding	noncoding
MSTRG.15812.1		XLOC_018893	279	coding	noncoding	noncoding	noncoding
MSTRG.15824.1		XLOC_018899	201	coding	noncoding	noncoding	noncoding
MSTRG.15865.1		XLOC_018918	465	coding	noncoding	noncoding	noncoding
MSTRG.15875.1		XLOC_018925	301	coding	noncoding	noncoding	noncoding
MSTRG.15944.1		XLOC_018947	622	coding	coding	noncoding	noncoding
MSTRG.15959.1		XLOC_018950	218	coding	noncoding	noncoding	noncoding
MSTRG.16868.1	LOC107985242	XLOC_019111	278	coding	noncoding	noncoding	noncoding
MSTRG.16094.1		XLOC_019113	278	coding	noncoding	noncoding	noncoding
MSTRG.16127.1		XLOC_019130	433	coding	noncoding	noncoding	noncoding
MSTRG.16140.1		XLOC_019136	464	coding	noncoding	noncoding	noncoding
XR_922393.2	LOC105371672	XLOC_019145	5864	coding	noncoding	noncoding	noncoding
MSTRG.16153.1		XLOC_019147	300	coding	noncoding	noncoding	noncoding
MSTRG.16177.1		XLOC_019148	299	coding	noncoding	noncoding	noncoding
MSTRG.16181.1		XLOC_019151	316	coding	noncoding	noncoding	noncoding
XM_017001179.2	KCNT2	XLOC_019159	6550	coding	coding	coding	coding
MSTRG.16206.1	KCNT2	XLOC_019164	258	coding	noncoding	noncoding	noncoding
MSTRG.16222.1	CFHR3	XLOC_019170	442	coding	noncoding	noncoding	noncoding
MSTRG.16211.1		XLOC_019171	315	coding	noncoding	noncoding	noncoding
XR_922396.2	LOC105371675	XLOC_019172	2594	coding	noncoding	noncoding	noncoding
MSTRG.16224.1	F13B	XLOC_019175	434	coding	noncoding	noncoding	noncoding
MSTRG.16444.1	CRB1	XLOC_019207	269	coding	noncoding	noncoding	noncoding
MSTRG.16453.1		XLOC_019213	314	coding	noncoding	noncoding	noncoding
MSTRG.16470.1	DENND1B	XLOC_019229	225	coding	noncoding	noncoding	noncoding

MSTRG.16243.1		XLOC_019243	294	coding	noncoding	noncoding	noncoding
MSTRG.16258.1		XLOC_019251	279	coding	noncoding	noncoding	noncoding
MSTRG.16260.1		XLOC_019253	263	coding	noncoding	noncoding	noncoding
MSTRG.16281.7	NEK7	XLOC_019256	3933	coding	noncoding	noncoding	noncoding
MSTRG.16393.10	PTPRC	XLOC_019264	1681	coding	coding	noncoding	noncoding
MSTRG.16355.1		XLOC_019284	276	coding	noncoding	noncoding	noncoding
MSTRG.16502.1		XLOC_019295	283	coding	noncoding	noncoding	noncoding
MSTRG.16405.1		XLOC_019300	298	coding	noncoding	noncoding	noncoding
XM_011510236.1	KIF14	XLOC_019302	7160	coding	coding	coding	coding
MSTRG.16481.1		XLOC_019304	260	coding	noncoding	noncoding	noncoding
MSTRG.16539.1	CAMSAP2	XLOC_019323	252	coding	noncoding	noncoding	noncoding
XM_017000732.1	KIF21B	XLOC_019334	4375	coding	coding	coding	coding
NM_000069.3	CACNA1S	XLOC_019340	6028	coding	coding	coding	coding
NM_003281.4	TNNI1	XLOC_019358	6110	coding	coding	coding	coding
MSTRG.16623.1	ARL8A	XLOC_019398	269	coding	noncoding	noncoding	noncoding
MSTRG.16879.1	KDM5B	XLOC_019432	201	coding	noncoding	noncoding	noncoding
MSTRG.16888.1	KLHL12	XLOC_019439	276	coding	noncoding	noncoding	noncoding
MSTRG.16899.1	LOC100506747	XLOC_019447	220	coding	noncoding	noncoding	noncoding
MSTRG.16922.1		XLOC_019462	252	coding	noncoding	noncoding	noncoding
MSTRG.17000.1	LOC102723529	XLOC_019466	759	coding	noncoding	noncoding	noncoding
MSTRG.17001.1		XLOC_019475	241	coding	noncoding	noncoding	noncoding
MSTRG.17008.3	ATP2B4	XLOC_019480	6385	coding	coding	coding	coding
MSTRG.17066.19	ZC3H11A	XLOC_019483	4774	coding	coding	coding	coding
MSTRG.17022.1		XLOC_019489	301	coding	noncoding	noncoding	noncoding
MSTRG.17024.1		XLOC_019490	277	coding	noncoding	noncoding	noncoding
NR_027902.1	LINC00303	XLOC_019492	1514	coding	noncoding	noncoding	noncoding
MSTRG.17037.1	LINC00303	XLOC_019493	252	coding	noncoding	noncoding	noncoding
MSTRG.17034.1		XLOC_019494	316	coding	noncoding	noncoding	noncoding
XM_011509301.3	PLEKHA6	XLOC_019514	11723	coding	coding	coding	coding
MSTRG.17076.1	PLEKHA6	XLOC_019515	258	coding	noncoding	noncoding	noncoding
NM_002646.3	PIK3C2B	XLOC_019524	7606	coding	coding	coding	coding
XM_017001474.1	PIK3C2B	XLOC_019524	7794	coding	coding	coding	coding
XM_017001473.1	PIK3C2B	XLOC_019524	8591	coding	coding	coding	coding
MSTRG.17101.1		XLOC_019545	256	coding	noncoding	noncoding	noncoding
MSTRG.17115.1	NFASC	XLOC_019549	348	coding	noncoding	noncoding	noncoding
MSTRG.17122.1	NFASC	XLOC_019553	206	coding	noncoding	noncoding	noncoding
MSTRG.17176.1		XLOC_019574	306	coding	noncoding	noncoding	noncoding
MSTRG.17210.1	LEMD1	XLOC_019586	285	coding	noncoding	noncoding	noncoding
MSTRG.17217.1	LEMD1-DT	XLOC_019591	276	coding	noncoding	noncoding	noncoding
MSTRG.17226.1		XLOC_019605	622	coding	noncoding	noncoding	noncoding
XM_005245453.1	NUCKS1	XLOC_019608	6063	coding	noncoding	coding	coding
MSTRG.17258.1	NUCKS1	XLOC_019609	201	coding	noncoding	noncoding	noncoding
MSTRG.17253.1		XLOC_019613	360	coding	noncoding	noncoding	noncoding

MSTRG.17270.1	LOC284581	XLOC_019621	280	coding	noncoding	noncoding	noncoding
MSTRG.17271.1	LOC284581	XLOC_019622	225	coding	noncoding	noncoding	noncoding
NM_052934.4	SLC26A9	XLOC_019624	4791	coding	coding	coding	coding
MSTRG.17288.1	RAB7B	XLOC_019628	226	coding	noncoding	noncoding	noncoding
MSTRG.17310.2	IKBKE	XLOC_019640	345	coding	coding	noncoding	noncoding
MSTRG.17341.5	EIF2D	XLOC_019651	10751	coding	coding	coding	coding
MSTRG.17349.1	IL19	XLOC_019669	236	coding	noncoding	noncoding	noncoding
MSTRG.17418.1		XLOC_019696	241	coding	noncoding	noncoding	noncoding
MSTRG.17817.14	CR1	XLOC_019714	6268	coding	coding	noncoding	noncoding
MSTRG.17516.1	LOC105372889	XLOC_019755	289	coding	noncoding	noncoding	noncoding
MSTRG.17508.1		XLOC_019761	294	coding	noncoding	noncoding	noncoding
MSTRG.17622.1	LOC107985255	XLOC_019784	263	coding	noncoding	noncoding	noncoding
MSTRG.17602.1		XLOC_019791	230	coding	noncoding	noncoding	noncoding
MSTRG.17687.7	C1orf74	XLOC_019812	8990	coding	coding	coding	noncoding
NM_006147.4	IRF6	XLOC_019814	4478	coding	coding	coding	coding
MSTRG.17652.1	SYT14	XLOC_019820	334	coding	coding	noncoding	noncoding
MSTRG.17653.1	SYT14	XLOC_019821	292	coding	noncoding	noncoding	noncoding
MSTRG.17746.1	KCNH1	XLOC_019850	266	coding	noncoding	noncoding	noncoding
MSTRG.17707.1		XLOC_019852	264	coding	noncoding	noncoding	noncoding
MSTRG.17794.10	RCOR3	XLOC_019853	3262	coding	coding	coding	noncoding
MSTRG.17956.1	LPGAT1-AS1	XLOC_019886	281	coding	noncoding	noncoding	noncoding
MSTRG.17960.1		XLOC_019890	262	coding	noncoding	noncoding	noncoding
MSTRG.17896.1		XLOC_019903	294	coding	noncoding	noncoding	noncoding
MSTRG.17893.1		XLOC_019904	241	coding	noncoding	noncoding	noncoding
MSTRG.18007.8	PPP2R5A	XLOC_019911	1982	coding	coding	coding	coding
MSTRG.18007.12	PPP2R5A	XLOC_019911	637	coding	coding	noncoding	noncoding
MSTRG.18013.1	PACC1	XLOC_019913	224	coding	noncoding	noncoding	noncoding
MSTRG.17926.1	ATF3	XLOC_019937	252	coding	noncoding	noncoding	noncoding
XR_921869.2	BATF3	XLOC_019944	1232	coding	noncoding	coding	noncoding
MSTRG.18047.1		XLOC_019985	260	coding	noncoding	noncoding	noncoding
MSTRG.18154.1	PTPN14	XLOC_019992	275	coding	noncoding	noncoding	noncoding
MSTRG.18165.1		XLOC_020007	248	coding	noncoding	noncoding	noncoding
MSTRG.18163.1	KCNK2	XLOC_020011	292	coding	noncoding	noncoding	noncoding
MSTRG.18137.1		XLOC_020016	271	coding	coding	noncoding	noncoding
MSTRG.18134.1		XLOC_020018	375	coding	noncoding	noncoding	noncoding
MSTRG.18118.1		XLOC_020019	617	coding	coding	noncoding	noncoding
XM_017000621.2	ESRRG	XLOC_020035	9490	coding	coding	coding	coding
MSTRG.18224.1	ESRRG	XLOC_020038	346	coding	noncoding	noncoding	noncoding
MSTRG.18215.1		XLOC_020051	238	coding	noncoding	noncoding	noncoding
MSTRG.18256.1	SPATA17	XLOC_020073	280	coding	noncoding	noncoding	noncoding
MSTRG.18257.1	SPATA17	XLOC_020074	275	coding	noncoding	noncoding	noncoding
MSTRG.18263.1	SPATA17	XLOC_020078	222	coding	noncoding	noncoding	noncoding
MSTRG.18285.1	TGFB2	XLOC_020090	335	coding	coding	noncoding	noncoding

MSTRG.18448.1		XLOC_020105	315	coding	noncoding	noncoding	noncoding
MSTRG.18452.1		XLOC_020107	270	coding	noncoding	noncoding	noncoding
MSTRG.18329.1		XLOC_020133	554	coding	noncoding	noncoding	noncoding
MSTRG.18350.1	SLC30A10	XLOC_020145	312	coding	noncoding	noncoding	noncoding
MSTRG.18352.1	LOC107985281	XLOC_020146	283	coding	noncoding	noncoding	noncoding
MSTRG.18354.1	BPNT1	XLOC_020152	534	coding	noncoding	noncoding	noncoding
MSTRG.18388.1	MARK1	XLOC_020168	288	coding	noncoding	noncoding	noncoding
MSTRG.18377.1		XLOC_020171	219	coding	noncoding	noncoding	noncoding
MSTRG.18403.1		XLOC_020188	316	coding	noncoding	noncoding	noncoding
MSTRG.18438.1	LOC105372932	XLOC_020203	302	coding	coding	noncoding	noncoding
MSTRG.18546.1		XLOC_020212	263	coding	noncoding	noncoding	noncoding
MSTRG.18556.1	LINC01655	XLOC_020222	353	coding	noncoding	noncoding	noncoding
MSTRG.18580.1	LINC02257	XLOC_020229	395	coding	noncoding	noncoding	noncoding
MSTRG.18608.1		XLOC_020247	304	coding	noncoding	noncoding	noncoding
MSTRG.18612.1	LOC105372956	XLOC_020249	279	coding	noncoding	noncoding	noncoding
MSTRG.18644.1		XLOC_020270	279	coding	noncoding	noncoding	noncoding
XM_011509687.1	SUSD4	XLOC_020273	3106	coding	coding	coding	coding
MSTRG.18736.1		XLOC_020321	260	coding	noncoding	noncoding	noncoding
MSTRG.18759.1		XLOC_020325	280	coding	noncoding	noncoding	noncoding
MSTRG.18817.1	NVL	XLOC_020331	248	coding	noncoding	noncoding	noncoding
MSTRG.18807.1		XLOC_020335	439	coding	noncoding	noncoding	noncoding
MSTRG.18827.1		XLOC_020357	309	coding	coding	noncoding	noncoding
MSTRG.18900.1	DNAH14	XLOC_020361	293	coding	noncoding	noncoding	noncoding
MSTRG.18902.1	DNAH14	XLOC_020362	293	coding	noncoding	noncoding	noncoding
MSTRG.18909.1	DNAH14	XLOC_020369	340	coding	noncoding	noncoding	noncoding
XM_011544185.3	LBR	XLOC_020371	4237	coding	coding	coding	coding
MSTRG.18948.1		XLOC_020408	287	coding	noncoding	noncoding	noncoding
MSTRG.18928.1	LINC01703	XLOC_020410	544	coding	noncoding	noncoding	noncoding
MSTRG.18934.1		XLOC_020411	307	coding	noncoding	noncoding	noncoding
MSTRG.18932.1	ACBD3	XLOC_020415	293	coding	noncoding	noncoding	noncoding
MSTRG.18924.1	LOC101927247	XLOC_020417	220	coding	noncoding	noncoding	noncoding
MSTRG.18949.1		XLOC_020418	306	coding	noncoding	noncoding	noncoding
MSTRG.18957.1		XLOC_020421	217	coding	noncoding	noncoding	noncoding
MSTRG.18970.1		XLOC_020426	270	coding	noncoding	noncoding	noncoding
XM_017002582.2	CDC42BPA	XLOC_020467	6122	coding	coding	coding	coding
MSTRG.19041.1	CDC42BPA	XLOC_020468	234	coding	noncoding	noncoding	noncoding
MSTRG.18999.1		XLOC_020473	264	coding	noncoding	noncoding	noncoding
MSTRG.19067.1		XLOC_020475	291	coding	noncoding	noncoding	noncoding
MSTRG.19084.1	WNT3A	XLOC_020495	308	coding	noncoding	noncoding	noncoding
MSTRG.19128.1		XLOC_020521	225	coding	coding	noncoding	noncoding
MSTRG.19161.1	OBSCN	XLOC_020534	2080	coding	coding	coding	coding
MSTRG.19178.1	OBSCN	XLOC_020549	223	coding	coding	noncoding	noncoding
XR_949242.2	LINC02814	XLOC_020587	12763	coding	noncoding	coding	noncoding

MSTRG.19208.1		XLOC_020599	238	coding	noncoding	noncoding	noncoding
MSTRG.19216.1		XLOC_020600	253	coding	noncoding	noncoding	noncoding
MSTRG.19412.1	GALNT2	XLOC_020624	245	coding	noncoding	noncoding	noncoding
NM_000029.4	AGT	XLOC_020641	2116	coding	coding	coding	coding
MSTRG.19331.1	LOC105373166	XLOC_020643	234	coding	noncoding	noncoding	noncoding
MSTRG.19346.1	C1orf198	XLOC_020650	211	coding	noncoding	noncoding	noncoding
MSTRG.19377.1	TRIM67	XLOC_020673	276	coding	noncoding	noncoding	noncoding
MSTRG.19464.1	EGLN1	XLOC_020695	267	coding	noncoding	noncoding	noncoding
MSTRG.19443.1		XLOC_020720	269	coding	noncoding	noncoding	noncoding
MSTRG.19492.1		XLOC_020739	426	coding	noncoding	noncoding	noncoding
MSTRG.19503.1		XLOC_020756	286	coding	noncoding	noncoding	noncoding
NM_001328607.2	PCNX2	XLOC_020769	904	coding	noncoding	noncoding	noncoding
MSTRG.19830.1	PCNX2	XLOC_020775	311	coding	noncoding	noncoding	noncoding
MSTRG.19843.1	PCNX2	XLOC_020780	208	coding	noncoding	noncoding	noncoding
MSTRG.19849.1	PCNX2	XLOC_020784	278	coding	noncoding	noncoding	noncoding
MSTRG.19534.1		XLOC_020790	307	coding	noncoding	noncoding	noncoding
MSTRG.19561.1		XLOC_020800	308	coding	noncoding	noncoding	noncoding
MSTRG.19586.1	SLC35F3	XLOC_020808	608	coding	noncoding	noncoding	noncoding
MSTRG.19587.1	SLC35F3	XLOC_020809	348	coding	noncoding	noncoding	noncoding
MSTRG.19680.1		XLOC_020858	347	coding	noncoding	noncoding	noncoding
MSTRG.19657.1		XLOC_020864	235	coding	noncoding	noncoding	noncoding
MSTRG.19690.1		XLOC_020889	232	coding	noncoding	noncoding	noncoding
XM_017000394.1	B3GALNT2	XLOC_020890	4848	coding	coding	coding	coding
MSTRG.19695.1	GNG4	XLOC_020893	269	coding	noncoding	noncoding	noncoding
MSTRG.19697.1	GNG4	XLOC_020894	306	coding	noncoding	noncoding	noncoding
MSTRG.19862.1	LINC02768	XLOC_020945	240	coding	noncoding	noncoding	noncoding
MSTRG.19866.1		XLOC_020952	305	coding	noncoding	noncoding	noncoding
MSTRG.19868.1		XLOC_020953	622	coding	noncoding	noncoding	noncoding
MSTRG.19885.1		XLOC_020971	368	coding	noncoding	noncoding	noncoding
MSTRG.19980.4	HEATR1	XLOC_020981	5882	coding	coding	noncoding	noncoding
MSTRG.19960.1		XLOC_020982	283	coding	noncoding	noncoding	noncoding
MSTRG.19971.1		XLOC_020995	313	coding	noncoding	coding	noncoding
MSTRG.20064.1	RYR2	XLOC_021002	343	coding	noncoding	noncoding	noncoding
MSTRG.20068.1	RYR2	XLOC_021006	350	coding	noncoding	noncoding	noncoding
MSTRG.19987.1		XLOC_021014	230	coding	coding	noncoding	noncoding
MSTRG.20006.1	LOC105373220	XLOC_021019	318	coding	noncoding	noncoding	noncoding
MSTRG.20040.1		XLOC_021034	310	coding	noncoding	noncoding	noncoding
MSTRG.20104.1	FMN2	XLOC_021067	313	coding	noncoding	coding	noncoding
MSTRG.20110.1	FMN2	XLOC_021070	203	coding	noncoding	noncoding	noncoding
XM_017002013.1	RGS7	XLOC_021080	3865	coding	coding	coding	coding
XM_011544132.2	FH	XLOC_021089	2245	coding	coding	coding	coding
MSTRG.20285.1	WDR64	XLOC_021101	271	coding	noncoding	noncoding	noncoding
MSTRG.20213.1		XLOC_021105	354	coding	noncoding	noncoding	noncoding

MSTRG.20215.1		XLOC_021112	300	coding	noncoding	noncoding	noncoding
MSTRG.20221.1		XLOC_021117	266	coding	noncoding	noncoding	noncoding
XM_017000569.1	PLD5	XLOC_021120	8321	coding	coding	coding	coding
MSTRG.20250.1	PLD5	XLOC_021121	251	coding	noncoding	noncoding	noncoding
MSTRG.20245.1		XLOC_021133	281	coding	noncoding	noncoding	noncoding
MSTRG.20292.1		XLOC_021188	238	coding	noncoding	noncoding	noncoding
MSTRG.20407.1	C1orf100	XLOC_021210	293	coding	noncoding	noncoding	noncoding
MSTRG.20409.1	C1orf100	XLOC_021211	250	coding	noncoding	noncoding	noncoding
MSTRG.20415.1		XLOC_021212	280	coding	noncoding	noncoding	noncoding
MSTRG.20344.1		XLOC_021247	276	coding	noncoding	noncoding	noncoding
MSTRG.20811.1	SMYD3	XLOC_021287	377	coding	noncoding	noncoding	noncoding
MSTRG.20530.12	CNST	XLOC_021367	1320	coding	noncoding	noncoding	noncoding
XR_949136.3	AHCTF1	XLOC_021380	8747	coding	coding	coding	coding
NM_001323342.2	AHCTF1	XLOC_021380	8887	coding	coding	coding	coding
MSTRG.20539.1	ZNF670-ZNF695	XLOC_021401	256	coding	noncoding	noncoding	noncoding
MSTRG.20611.1	LOC107985375	XLOC_021423	271	coding	noncoding	noncoding	noncoding
MSTRG.20652.1		XLOC_021445	313	coding	noncoding	noncoding	noncoding
MSTRG.20659.2	TRIM58	XLOC_021448	2786	coding	coding	coding	noncoding
MSTRG.20661.1		XLOC_021449	910	coding	noncoding	noncoding	noncoding
MSTRG.20662.1		XLOC_021450	293	coding	noncoding	noncoding	noncoding
MSTRG.20653.1		XLOC_021452	548	coding	noncoding	noncoding	noncoding
MSTRG.20682.1	OR2L13	XLOC_021460	246	coding	coding	noncoding	noncoding
MSTRG.20727.1	OR2T10	XLOC_021494	289	coding	noncoding	noncoding	noncoding
MSTRG.20774.1	PGBD2	XLOC_021511	298	coding	noncoding	noncoding	noncoding
MSTRG.20792.1		XLOC_021535	252	coding	noncoding	noncoding	noncoding
MSTRG.20900.1		XLOC_021542	297	coding	noncoding	noncoding	noncoding
MSTRG.20913.1		XLOC_021544	227	coding	noncoding	noncoding	noncoding
MSTRG.21049.1	MYT1L	XLOC_021583	286	coding	noncoding	noncoding	noncoding
MSTRG.20970.1		XLOC_021601	248	coding	noncoding	noncoding	noncoding
MSTRG.21120.1		XLOC_021646	282	coding	noncoding	noncoding	noncoding
MSTRG.21140.1	DCDC2C	XLOC_021652	285	coding	noncoding	noncoding	noncoding
MSTRG.21129.1		XLOC_021654	386	coding	noncoding	noncoding	noncoding
MSTRG.21134.1		XLOC_021657	299	coding	noncoding	noncoding	noncoding
MSTRG.21152.1		XLOC_021666	302	coding	noncoding	noncoding	noncoding
MSTRG.21154.1		XLOC_021668	278	coding	noncoding	noncoding	noncoding
MSTRG.21161.1		XLOC_021671	277	coding	noncoding	noncoding	noncoding
MSTRG.21172.1		XLOC_021678	254	coding	noncoding	noncoding	noncoding
MSTRG.21185.1		XLOC_021685	264	coding	noncoding	noncoding	noncoding
MSTRG.21208.1		XLOC_021697	243	coding	noncoding	noncoding	noncoding
NR_026832.1	SILC1	XLOC_021698	11868	coding	noncoding	noncoding	noncoding
MSTRG.21215.1	SILC1	XLOC_021699	286	coding	noncoding	noncoding	noncoding
MSTRG.21227.1		XLOC_021706	272	coding	noncoding	noncoding	noncoding
MSTRG.21228.1		XLOC_021707	241	coding	noncoding	noncoding	noncoding

MSTRG.21257.1		XLOC_021754	248	coding	noncoding	noncoding	noncoding
MSTRG.21275.1	LOC105373408	XLOC_021766	290	coding	noncoding	noncoding	noncoding
MSTRG.21286.1	LOC101929551	XLOC_021769	244	coding	noncoding	noncoding	noncoding
MSTRG.21340.21	MBOAT2	XLOC_021892	586	coding	noncoding	coding	noncoding
MSTRG.21337.1		XLOC_021899	325	coding	noncoding	noncoding	noncoding
MSTRG.21339.1		XLOC_021900	249	coding	noncoding	noncoding	noncoding
XM_011510409.1	ASAP2	XLOC_021901	5366	coding	coding	coding	coding
XR_001739279.1	LOC107985851	XLOC_021902	3533	coding	noncoding	noncoding	noncoding
MSTRG.21415.1	ASAP2	XLOC_021905	253	coding	noncoding	noncoding	noncoding
XR_922777.2	LOC101929643	XLOC_021915	1716	coding	noncoding	noncoding	noncoding
MSTRG.21383.1	TAF1B	XLOC_021933	265	coding	noncoding	noncoding	noncoding
MSTRG.21392.1		XLOC_021939	230	coding	noncoding	noncoding	noncoding
MSTRG.21402.1	RRM2	XLOC_021941	238	coding	noncoding	noncoding	noncoding
MSTRG.21461.1		XLOC_021957	262	coding	noncoding	noncoding	noncoding
MSTRG.21464.1	LOC105373426	XLOC_021959	296	coding	noncoding	noncoding	noncoding
MSTRG.21465.1	LOC105373426	XLOC_021960	296	coding	noncoding	noncoding	noncoding
XR_002959251.1	ATP6V1C2	XLOC_021964	2858	coding	coding	coding	coding
MSTRG.21620.1	C2orf50	XLOC_021973	283	coding	noncoding	noncoding	noncoding
NM_001282710.1	SLC66A3	XLOC_021974	1788	coding	coding	coding	coding
MSTRG.21648.1		XLOC_021975	338	coding	noncoding	noncoding	noncoding
MSTRG.21654.1	LOC105373429	XLOC_021978	278	coding	noncoding	noncoding	noncoding
XM_024453256.1	GREB1	XLOC_021990	10768	coding	coding	coding	coding
MSTRG.21676.1	GREB1	XLOC_021991	253	coding	noncoding	noncoding	noncoding
MSTRG.21669.1		XLOC_021992	206	coding	noncoding	noncoding	noncoding
MSTRG.21671.1		XLOC_021993	286	coding	noncoding	noncoding	noncoding
XM_011510335.3	LPIN1	XLOC_021994	6129	coding	coding	coding	coding
MSTRG.21747.1	LOC105373436	XLOC_022057	230	coding	noncoding	noncoding	noncoding
MSTRG.21757.1	LOC105373436	XLOC_022061	271	coding	noncoding	noncoding	noncoding
MSTRG.21740.1		XLOC_022066	201	coding	noncoding	noncoding	noncoding
MSTRG.21769.1	LOC105373438	XLOC_022071	246	coding	noncoding	noncoding	noncoding
MSTRG.21795.1	LINC00276	XLOC_022090	218	coding	noncoding	noncoding	noncoding
MSTRG.21817.1		XLOC_022103	275	coding	noncoding	noncoding	noncoding
MSTRG.21989.5	NBAS	XLOC_022117	4884	coding	coding	coding	noncoding
MSTRG.21935.1	DDX1	XLOC_022121	279	coding	noncoding	noncoding	noncoding
MSTRG.21900.1		XLOC_022125	447	coding	noncoding	noncoding	noncoding
MSTRG.21910.1		XLOC_022129	397	coding	noncoding	noncoding	noncoding
MSTRG.21915.1	MYCNUT	XLOC_022133	245	coding	noncoding	noncoding	noncoding
MSTRG.21922.1		XLOC_022136	284	coding	noncoding	noncoding	noncoding
MSTRG.21925.1		XLOC_022138	223	coding	noncoding	noncoding	noncoding
MSTRG.21931.1		XLOC_022141	279	coding	noncoding	noncoding	noncoding
MSTRG.22055.13	FAM49A	XLOC_022159	11559	coding	noncoding	coding	noncoding
MSTRG.22017.1		XLOC_022164	286	coding	noncoding	noncoding	noncoding
MSTRG.22036.1		XLOC_022171	252	coding	noncoding	noncoding	noncoding

MSTRG.22127.1	VSNL1	XLOC_022188	268	coding	noncoding	noncoding	noncoding
MSTRG.22131.1	VSNL1	XLOC_022190	246	coding	noncoding	noncoding	noncoding
MSTRG.22099.1		XLOC_022201	311	coding	noncoding	noncoding	noncoding
MSTRG.22108.1		XLOC_022207	210	coding	noncoding	noncoding	noncoding
MSTRG.22116.1		XLOC_022212	223	coding	noncoding	noncoding	noncoding
MSTRG.22119.1	LOC105373454	XLOC_022215	290	coding	noncoding	noncoding	noncoding
MSTRG.22121.1	LOC105373454	XLOC_022216	252	coding	noncoding	noncoding	noncoding
MSTRG.22367.1		XLOC_022252	274	coding	noncoding	noncoding	noncoding
MSTRG.22369.1		XLOC_022253	394	coding	noncoding	noncoding	noncoding
MSTRG.22373.1		XLOC_022255	225	coding	noncoding	noncoding	noncoding
MSTRG.22317.1	LOC101928196	XLOC_022306	259	coding	noncoding	noncoding	noncoding
MSTRG.22450.1	SDC1	XLOC_022371	247	coding	noncoding	noncoding	noncoding
MSTRG.22583.4	PUM2	XLOC_022373	6192	coding	coding	coding	noncoding
MSTRG.22470.1		XLOC_022388	264	coding	noncoding	noncoding	noncoding
MSTRG.22545.1		XLOC_022407	279	coding	noncoding	noncoding	noncoding
MSTRG.22571.1		XLOC_022411	278	coding	noncoding	noncoding	noncoding
MSTRG.22607.1	APOB	XLOC_022417	308	coding	coding	noncoding	noncoding
MSTRG.22618.1		XLOC_022420	310	coding	noncoding	noncoding	noncoding
MSTRG.22635.1	LOC100507562	XLOC_022428	305	coding	noncoding	noncoding	noncoding
MSTRG.22641.1		XLOC_022432	257	coding	noncoding	noncoding	noncoding
MSTRG.22658.1		XLOC_022437	244	coding	noncoding	coding	noncoding
MSTRG.22662.1		XLOC_022441	330	coding	noncoding	noncoding	noncoding
NM_052920.2	KLHL29	XLOC_022467	5325	coding	coding	coding	coding
MSTRG.23036.1	KLHL29	XLOC_022472	293	coding	noncoding	noncoding	noncoding
NR_136538.1	FKBP1B	XLOC_022491	1099	coding	coding	coding	noncoding
NR_136536.1	FKBP1B	XLOC_022491	1167	coding	coding	coding	noncoding
MSTRG.22928.1		XLOC_022503	249	coding	noncoding	noncoding	noncoding
NM_001362952.1	NCOA1	XLOC_022509	7401	coding	coding	coding	coding
MSTRG.23087.1	EFR3B	XLOC_022609	469	coding	noncoding	noncoding	noncoding
MSTRG.23092.1		XLOC_022627	283	coding	noncoding	noncoding	noncoding
MSTRG.23110.1		XLOC_022636	260	coding	noncoding	noncoding	noncoding
MSTRG.23111.1		XLOC_022637	232	coding	noncoding	noncoding	noncoding
MSTRG.23125.1	KIF3C	XLOC_022646	295	coding	noncoding	coding	noncoding
MSTRG.23117.1		XLOC_022653	295	coding	noncoding	noncoding	noncoding
MSTRG.23121.1		XLOC_022655	218	coding	noncoding	noncoding	noncoding
MSTRG.23187.1	RAB10	XLOC_022664	267	coding	noncoding	noncoding	noncoding
MSTRG.23300.1	CIB4	XLOC_022684	462	coding	noncoding	noncoding	noncoding
NM_001809.4	CENPA	XLOC_022690	1389	coding	coding	coding	noncoding
XR_001738852.2	DPYSL5	XLOC_022691	5538	coding	coding	coding	coding
MSTRG.23334.1		XLOC_022692	227	coding	noncoding	noncoding	noncoding
MSTRG.23364.1	CGREF1	XLOC_022704	266	coding	noncoding	noncoding	noncoding
XM_011533138.3	ABHD1	XLOC_022706	1604	coding	coding	coding	coding
NM_175769.3	TCF23	XLOC_022707	4882	coding	coding	coding	noncoding

NM_080592.3	ATRAID	XLOC_022713	1258 coding	noncoding	coding	coding
MSTRG.23373.1	SLC30A3	XLOC_022716	256 coding	noncoding	noncoding	noncoding
NM_001267060.1	SNX17	XLOC_022724	2400 coding	coding	coding	coding
MSTRG.23447.1	ZNF512	XLOC_022745	316 coding	noncoding	noncoding	noncoding
MSTRG.23429.1		XLOC_022751	281 coding	noncoding	noncoding	noncoding
MSTRG.23622.1	BABAM2	XLOC_022764	254 coding	noncoding	noncoding	noncoding
MSTRG.23655.1	BABAM2	XLOC_022794	241 coding	noncoding	noncoding	noncoding
MSTRG.23667.1	BABAM2	XLOC_022805	496 coding	noncoding	noncoding	noncoding
MSTRG.23462.1		XLOC_022819	262 coding	noncoding	noncoding	noncoding
MSTRG.23501.1	PLB1	XLOC_022826	296 coding	noncoding	noncoding	noncoding
MSTRG.23518.1	PPP1CB	XLOC_022833	265 coding	noncoding	noncoding	noncoding
MSTRG.23483.1		XLOC_022840	387 coding	noncoding	noncoding	noncoding
XM_011532628.2	TOGARAM2	XLOC_022852	3090 coding	coding	coding	coding
NM_199280.3	TOGARAM2	XLOC_022852	12691 coding	coding	coding	coding
XM_017004318.1	YPEL5	XLOC_022886	2855 coding	coding	coding	coding
NM_001127399.2	YPEL5	XLOC_022886	2197 coding	coding	coding	noncoding
NM_182551.5	LCLAT1	XLOC_022904	5071 coding	coding	coding	coding
MSTRG.23792.2	GALNT14	XLOC_022944	1056 coding	coding	coding	noncoding
MSTRG.23758.1		XLOC_022972	222 coding	noncoding	noncoding	noncoding
MSTRG.23775.1	LINC01946	XLOC_022982	351 coding	coding	noncoding	noncoding
MSTRG.23785.1		XLOC_022991	411 coding	noncoding	noncoding	noncoding
MSTRG.23821.1	SPAST	XLOC_023007	306 coding	noncoding	noncoding	noncoding
MSTRG.23823.1	SPAST	XLOC_023009	232 coding	noncoding	noncoding	noncoding
MSTRG.24023.1		XLOC_023019	321 coding	noncoding	noncoding	noncoding
XM_006712054.3	BIRC6	XLOC_023021	15866 coding	coding	coding	coding
MSTRG.23904.1	LINC00486	XLOC_023063	378 coding	noncoding	noncoding	noncoding
XM_024452888.1	LTBP1	XLOC_023065	5679 coding	coding	coding	coding
MSTRG.23916.1	LTBP1	XLOC_023068	344 coding	noncoding	noncoding	noncoding
MSTRG.23970.1		XLOC_023078	298 coding	noncoding	noncoding	noncoding
MSTRG.23930.1	LINC01317	XLOC_023090	261 coding	noncoding	noncoding	noncoding
MSTRG.23938.1	LINC01317	XLOC_023093	243 coding	noncoding	noncoding	noncoding
MSTRG.23958.1		XLOC_023100	320 coding	noncoding	noncoding	noncoding
MSTRG.23959.1		XLOC_023101	295 coding	noncoding	noncoding	noncoding
MSTRG.23991.1		XLOC_023105	284 coding	noncoding	noncoding	noncoding
MSTRG.24086.1	SULT6B1	XLOC_023146	250 coding	noncoding	noncoding	noncoding
MSTRG.24457.1	LOC105374464	XLOC_023157	310 coding	noncoding	noncoding	noncoding
MSTRG.24185.1	LINC02613	XLOC_023226	349 coding	noncoding	noncoding	noncoding
MSTRG.24202.1		XLOC_023241	223 coding	coding	noncoding	noncoding
MSTRG.24219.1		XLOC_023249	238 coding	noncoding	noncoding	noncoding
MSTRG.24307.1	CDKL4	XLOC_023262	270 coding	noncoding	noncoding	noncoding
MSTRG.24312.2	MAP4K3	XLOC_023265	229 coding	coding	noncoding	noncoding
MSTRG.24303.1	THUMPD2	XLOC_023303	305 coding	noncoding	noncoding	noncoding
MSTRG.24279.1		XLOC_023307	278 coding	noncoding	noncoding	noncoding

MSTRG.24758.24	SLC8A1	XLOC_023309	1708 coding	noncoding	noncoding	noncoding
MSTRG.24758.27	SLC8A1	XLOC_023309	3936 coding	noncoding	noncoding	noncoding
MSTRG.24758.30	SLC8A1	XLOC_023309	21291 coding	noncoding	noncoding	noncoding
MSTRG.24766.1	SLC8A1	XLOC_023312	275 coding	noncoding	noncoding	noncoding
MSTRG.24775.1	LOC101929667	XLOC_023321	277 coding	noncoding	noncoding	noncoding
MSTRG.24374.1	LOC105374497	XLOC_023331	225 coding	noncoding	noncoding	noncoding
MSTRG.24383.1	LOC105374497	XLOC_023336	314 coding	noncoding	noncoding	noncoding
MSTRG.24441.1	LOC105374506	XLOC_023355	306 coding	noncoding	noncoding	noncoding
MSTRG.24391.1		XLOC_023360	305 coding	noncoding	noncoding	noncoding
MSTRG.24409.1		XLOC_023371	234 coding	noncoding	noncoding	noncoding
MSTRG.24641.1	COX7A2L	XLOC_023385	401 coding	noncoding	noncoding	noncoding
MSTRG.24643.2	COX7A2L	XLOC_023387	571 coding	coding	noncoding	noncoding
MSTRG.24452.1	KCNG3	XLOC_023388	216 coding	noncoding	noncoding	noncoding
MSTRG.24653.1	MTA3	XLOC_023392	287 coding	noncoding	noncoding	noncoding
MSTRG.24661.1	MTA3	XLOC_023395	474 coding	noncoding	noncoding	noncoding
MSTRG.24669.1	MTA3	XLOC_023400	295 coding	noncoding	noncoding	noncoding
MSTRG.24670.1	MTA3	XLOC_023401	215 coding	noncoding	noncoding	noncoding
MSTRG.24683.1	MTA3	XLOC_023407	248 coding	noncoding	noncoding	noncoding
MSTRG.24612.1		XLOC_023412	246 coding	noncoding	noncoding	noncoding
MSTRG.24613.1		XLOC_023413	246 coding	noncoding	noncoding	noncoding
MSTRG.24616.1	LOC112268413	XLOC_023416	272 coding	noncoding	noncoding	noncoding
MSTRG.24841.1		XLOC_023448	270 coding	noncoding	noncoding	noncoding
MSTRG.24731.2	LRPPRC	XLOC_023472	5127 coding	coding	coding	noncoding
XM_017004395.2	PPM1B	XLOC_023476	2654 coding	coding	coding	coding
MSTRG.24749.1	PPM1B	XLOC_023478	218 coding	noncoding	noncoding	noncoding
MSTRG.24788.1	SIX2	XLOC_023494	525 coding	noncoding	noncoding	noncoding
XM_006712050.3	PRKCE	XLOC_023520	4880 coding	coding	coding	coding
MSTRG.25273.1	PRKCE	XLOC_023524	213 coding	noncoding	noncoding	noncoding
MSTRG.24957.1	EPAS1	XLOC_023555	303 coding	coding	noncoding	noncoding
MSTRG.25030.12	RHOQ	XLOC_023570	540 coding	noncoding	noncoding	noncoding
MSTRG.24992.1	SOCS5	XLOC_023578	231 coding	noncoding	noncoding	noncoding
MSTRG.25113.1	TTC7A	XLOC_023586	9652 coding	coding	coding	coding
MSTRG.25003.1	EPCAM-DT	XLOC_023601	239 coding	noncoding	noncoding	noncoding
MSTRG.25004.1	EPCAM-DT	XLOC_023602	219 coding	noncoding	noncoding	noncoding
MSTRG.25008.1	EPCAM-DT	XLOC_023605	297 coding	noncoding	noncoding	noncoding
MSTRG.25015.1	EPCAM-DT	XLOC_023609	227 coding	noncoding	noncoding	noncoding
MSTRG.25017.1	EPCAM-DT	XLOC_023610	318 coding	noncoding	noncoding	noncoding
XM_005264332.4	MSH2	XLOC_023613	7145 coding	coding	coding	coding
MSTRG.25065.1	MSH2	XLOC_023614	232 coding	noncoding	noncoding	noncoding
MSTRG.25068.1	MSH2	XLOC_023617	290 coding	noncoding	noncoding	noncoding
MSTRG.25071.1	MSH2	XLOC_023620	426 coding	noncoding	noncoding	noncoding
MSTRG.25130.1	LOC105374589	XLOC_023636	249 coding	noncoding	noncoding	noncoding
MSTRG.25132.1		XLOC_023638	297 coding	noncoding	noncoding	noncoding

MSTRG.25165.1		XLOC_023663	290 coding	noncoding	noncoding	noncoding
MSTRG.25176.1	LOC102724008	XLOC_023669	312 coding	noncoding	noncoding	noncoding
MSTRG.25217.1		XLOC_023712	242 coding	noncoding	noncoding	noncoding
MSTRG.25224.1		XLOC_023713	260 coding	noncoding	noncoding	noncoding
MSTRG.25225.1		XLOC_023714	385 coding	noncoding	noncoding	noncoding
MSTRG.25232.1		XLOC_023718	311 coding	noncoding	noncoding	noncoding
MSTRG.25414.1	NRXN1	XLOC_023725	302 coding	noncoding	noncoding	noncoding
MSTRG.25434.1	NRXN1	XLOC_023735	260 coding	noncoding	noncoding	noncoding
MSTRG.25320.1	LOC730100	XLOC_023789	287 coding	noncoding	noncoding	noncoding
MSTRG.25324.1	LOC730100	XLOC_023791	290 coding	noncoding	noncoding	noncoding
MSTRG.25328.1	LOC730100	XLOC_023792	283 coding	noncoding	noncoding	noncoding
MSTRG.25336.1	LOC730100	XLOC_023797	279 coding	noncoding	noncoding	noncoding
MSTRG.25302.1		XLOC_023803	324 coding	noncoding	noncoding	noncoding
MSTRG.25301.1		XLOC_023804	206 coding	noncoding	noncoding	noncoding
MSTRG.25308.1		XLOC_023809	244 coding	noncoding	noncoding	noncoding
MSTRG.25356.1		XLOC_023832	249 coding	coding	noncoding	noncoding
MSTRG.25358.1		XLOC_023833	230 coding	noncoding	noncoding	noncoding
NM_001100396.2	C2orf73	XLOC_023859	1975 coding	coding	noncoding	coding
MSTRG.25498.1	C2orf73	XLOC_023860	310 coding	coding	noncoding	noncoding
MSTRG.25514.1	SPTBN1	XLOC_023871	268 coding	noncoding	noncoding	noncoding
MSTRG.25600.1		XLOC_023906	261 coding	noncoding	noncoding	noncoding
MSTRG.25603.8	CCDC88A	XLOC_023907	537 coding	noncoding	noncoding	noncoding
MSTRG.25729.1		XLOC_023940	281 coding	noncoding	noncoding	noncoding
MSTRG.26023.1	LOC105377632	XLOC_023964	242 coding	noncoding	noncoding	noncoding
MSTRG.25743.1		XLOC_024094	271 coding	noncoding	noncoding	noncoding
MSTRG.25846.1		XLOC_024144	225 coding	noncoding	noncoding	noncoding
MSTRG.25855.1		XLOC_024148	253 coding	noncoding	noncoding	noncoding
MSTRG.25856.1		XLOC_024149	241 coding	noncoding	noncoding	noncoding
MSTRG.25857.1		XLOC_024150	423 coding	noncoding	noncoding	noncoding
MSTRG.25891.1	REL	XLOC_024157	244 coding	noncoding	noncoding	noncoding
MSTRG.25899.1	PEX13	XLOC_024164	332 coding	coding	noncoding	noncoding
MSTRG.25913.1		XLOC_024176	228 coding	noncoding	noncoding	noncoding
MSTRG.25919.1		XLOC_024178	257 coding	noncoding	noncoding	noncoding
MSTRG.25954.1	COMMD1	XLOC_024185	227 coding	noncoding	noncoding	noncoding
MSTRG.25958.1	COMMD1	XLOC_024189	295 coding	noncoding	noncoding	noncoding
MSTRG.25943.1	B3GNT2	XLOC_024200	213 coding	noncoding	noncoding	noncoding
MSTRG.25985.1		XLOC_024208	221 coding	noncoding	noncoding	noncoding
MSTRG.25975.1		XLOC_024217	232 coding	noncoding	noncoding	noncoding
MSTRG.25996.1		XLOC_024218	395 coding	noncoding	noncoding	noncoding
MSTRG.26217.1	EHBP1	XLOC_024223	260 coding	noncoding	noncoding	noncoding
MSTRG.26222.1	EHBP1	XLOC_024228	289 coding	noncoding	noncoding	noncoding
MSTRG.26618.1		XLOC_024298	534 coding	noncoding	noncoding	noncoding
MSTRG.26627.1	LGALSL-DT	XLOC_024305	308 coding	noncoding	noncoding	noncoding

MSTRG.26276.1		XLOC_024336	251	coding	noncoding	noncoding	noncoding
MSTRG.26300.1	LINC02245	XLOC_024342	240	coding	noncoding	noncoding	noncoding
MSTRG.26306.1		XLOC_024367	276	coding	noncoding	noncoding	noncoding
MSTRG.26337.3	ACTR2	XLOC_024375	1581	coding	coding	coding	coding
MSTRG.26342.1		XLOC_024398	401	coding	noncoding	noncoding	noncoding
MSTRG.26343.1		XLOC_024399	318	coding	noncoding	noncoding	noncoding
MSTRG.26353.1		XLOC_024402	305	coding	noncoding	noncoding	noncoding
MSTRG.26382.1	LOC105369167	XLOC_024407	251	coding	noncoding	noncoding	noncoding
MSTRG.26436.1	LOC105369168	XLOC_024417	360	coding	noncoding	noncoding	noncoding
MSTRG.26476.1	LINC01829	XLOC_024444	263	coding	noncoding	coding	noncoding
MSTRG.26502.1		XLOC_024452	298	coding	noncoding	noncoding	noncoding
MSTRG.26518.1	LOC105374786	XLOC_024465	309	coding	noncoding	noncoding	noncoding
MSTRG.26710.1		XLOC_024494	221	coding	noncoding	noncoding	noncoding
MSTRG.26868.1		XLOC_024656	324	coding	noncoding	noncoding	noncoding
NM_001330425.3	NAGK	XLOC_024689	2286	coding	coding	coding	coding
MSTRG.27085.1		XLOC_024783	268	coding	noncoding	noncoding	noncoding
NM_003124.5	SPR	XLOC_024785	1432	coding	coding	coding	coding
MSTRG.27093.1		XLOC_024794	236	coding	noncoding	noncoding	noncoding
MSTRG.27094.1		XLOC_024795	294	coding	noncoding	noncoding	noncoding
MSTRG.27096.1		XLOC_024797	473	coding	noncoding	noncoding	noncoding
MSTRG.27098.1		XLOC_024799	246	coding	noncoding	noncoding	noncoding
MSTRG.27108.1	EGR4	XLOC_024812	239	coding	noncoding	noncoding	noncoding
MSTRG.27244.1	ALMS1	XLOC_024822	254	coding	noncoding	noncoding	noncoding
MSTRG.27148.1		XLOC_024833	390	coding	noncoding	noncoding	noncoding
NR_134896.1	DGUOK	XLOC_024849	749	coding	coding	coding	noncoding
NM_080918.3	DGUOK	XLOC_024849	811	coding	coding	coding	coding
MSTRG.27258.1		XLOC_024894	261	coding	noncoding	noncoding	noncoding
NM_001320824.2	WDR54	XLOC_024899	1180	coding	noncoding	coding	coding
XM_017004709.1	TTC31	XLOC_024910	3042	coding	coding	coding	coding
XR_001738589.2	SEMA4F	XLOC_024926	3389	coding	coding	coding	coding
MSTRG.27384.1	LOC102724482	XLOC_024930	216	coding	noncoding	noncoding	noncoding
MSTRG.27813.3	POLE4	XLOC_024935	3029	coding	coding	noncoding	noncoding
MSTRG.27430.1		XLOC_024978	217	coding	noncoding	noncoding	noncoding
MSTRG.27502.1	LRRTM4	XLOC_025011	281	coding	noncoding	noncoding	noncoding
MSTRG.27503.1	LRRTM4	XLOC_025012	321	coding	noncoding	noncoding	noncoding
MSTRG.27529.1	LOC101927967	XLOC_025019	240	coding	coding	noncoding	noncoding
MSTRG.27553.1		XLOC_025036	206	coding	noncoding	noncoding	noncoding
MSTRG.27557.1		XLOC_025039	225	coding	noncoding	noncoding	noncoding
MSTRG.27605.1	CTNNA2	XLOC_025048	289	coding	noncoding	noncoding	noncoding
MSTRG.27608.1	CTNNA2	XLOC_025050	246	coding	noncoding	noncoding	noncoding
MSTRG.27609.1	CTNNA2	XLOC_025051	245	coding	noncoding	noncoding	noncoding
MSTRG.27610.1	CTNNA2	XLOC_025052	308	coding	noncoding	noncoding	noncoding
MSTRG.27618.1	CTNNA2	XLOC_025055	282	coding	noncoding	noncoding	noncoding

MSTRG.27636.1	CTNNA2	XLOC_025062	305 coding	noncoding	noncoding	noncoding
MSTRG.27587.1		XLOC_025075	246 coding	noncoding	noncoding	noncoding
MSTRG.27653.1	LOC102724542	XLOC_025079	286 coding	noncoding	noncoding	noncoding
MSTRG.27671.1		XLOC_025089	310 coding	noncoding	noncoding	noncoding
MSTRG.27679.1		XLOC_025093	220 coding	noncoding	noncoding	noncoding
MSTRG.27686.1		XLOC_025102	286 coding	noncoding	noncoding	noncoding
MSTRG.27695.1		XLOC_025104	327 coding	noncoding	noncoding	noncoding
MSTRG.27709.1		XLOC_025111	241 coding	noncoding	noncoding	noncoding
MSTRG.27724.1		XLOC_025120	274 coding	noncoding	noncoding	noncoding
MSTRG.27741.1	LOC107985905	XLOC_025128	237 coding	noncoding	noncoding	noncoding
MSTRG.27733.1		XLOC_025129	224 coding	noncoding	noncoding	noncoding
MSTRG.27765.1	KCMF1	XLOC_025147	278 coding	noncoding	noncoding	noncoding
MSTRG.27748.1		XLOC_025153	235 coding	noncoding	noncoding	noncoding
MSTRG.27954.1	TCF7L1	XLOC_025155	334 coding	noncoding	noncoding	noncoding
MSTRG.27962.1	TCF7L1	XLOC_025159	254 coding	noncoding	noncoding	noncoding
MSTRG.27963.1	TGOLN2	XLOC_025160	6315 coding	coding	coding	coding
NM_001329791.2	ELMOD3	XLOC_025161	2662 coding	coding	coding	coding
XM_006711922.2	USP39	XLOC_025185	2144 coding	coding	coding	coding
XR_001739003.1	ATOH8	XLOC_025191	10861 coding	coding	coding	coding
XR_002959408.1	LOC112268420	XLOC_025198	8636 coding	noncoding	noncoding	noncoding
XM_024452994.1	KDM3A	XLOC_025219	4774 coding	coding	coding	coding
MSTRG.28161.1	RNF103-CHMP3	XLOC_025245	225 coding	noncoding	noncoding	noncoding
MSTRG.28175.1	RMND5A	XLOC_025255	274 coding	noncoding	noncoding	noncoding
MSTRG.28116.1		XLOC_025271	254 coding	noncoding	noncoding	noncoding
MSTRG.28225.1		XLOC_025326	207 coding	noncoding	noncoding	noncoding
MSTRG.28237.1		XLOC_025329	260 coding	noncoding	noncoding	noncoding
MSTRG.28243.1		XLOC_025330	260 coding	noncoding	noncoding	noncoding
MSTRG.28254.1		XLOC_025334	246 coding	noncoding	noncoding	noncoding
MSTRG.28427.15	IGKC	XLOC_025345	631 coding	coding	coding	coding
MSTRG.28427.43	IGKC	XLOC_025345	695 coding	coding	coding	coding
MSTRG.28445.1	IGKV4-1	XLOC_025356	246 coding	noncoding	noncoding	noncoding
MSTRG.28286.1		XLOC_025364	266 coding	noncoding	noncoding	noncoding
XR_001739589.1	LOC101926969	XLOC_025365	2720 coding	noncoding	noncoding	noncoding
MSTRG.28299.1	LSP1P4	XLOC_025368	717 coding	noncoding	noncoding	noncoding
MSTRG.28301.1		XLOC_025370	297 coding	noncoding	noncoding	noncoding
MSTRG.28390.1	LOC107985915	XLOC_025391	270 coding	noncoding	noncoding	noncoding
XM_017005468.2	LOC100509620	XLOC_025396	2279 coding	coding	coding	coding
NM_001286559.2	TEKT4	XLOC_025401	2215 coding	coding	coding	coding
MSTRG.28502.1		XLOC_025424	216 coding	noncoding	noncoding	noncoding
MSTRG.28520.1		XLOC_025436	205 coding	noncoding	noncoding	noncoding
MSTRG.28534.1		XLOC_025441	212 coding	noncoding	noncoding	noncoding
MSTRG.28541.1		XLOC_025442	326 coding	coding	noncoding	noncoding
MSTRG.28582.8	ANKRD36C	XLOC_025443	1225 coding	coding	coding	noncoding

MSTRG.28582.7	ANKRD36C	XLOC_025443	1225 coding	coding	coding	noncoding
MSTRG.28551.1	FAHD2CP	XLOC_025449	243 coding	noncoding	noncoding	noncoding
MSTRG.28552.1	FAHD2CP	XLOC_025450	231 coding	noncoding	coding	noncoding
MSTRG.28564.1		XLOC_025456	227 coding	noncoding	noncoding	noncoding
MSTRG.28628.4	TMEM127	XLOC_025461	6055 coding	noncoding	coding	noncoding
MSTRG.28628.3	TMEM127	XLOC_025461	3823 coding	noncoding	coding	noncoding
MSTRG.28628.7	TMEM127	XLOC_025461	2751 coding	noncoding	coding	noncoding
MSTRG.28618.1	NCAPH	XLOC_025465	271 coding	noncoding	noncoding	noncoding
MSTRG.28612.1		XLOC_025466	224 coding	noncoding	noncoding	noncoding
XM_011512124.2	FER1L5	XLOC_025481	6097 coding	coding	coding	coding
MSTRG.28651.1	FER1L5	XLOC_025482	219 coding	noncoding	noncoding	noncoding
MSTRG.28652.1	FER1L5	XLOC_025483	216 coding	noncoding	noncoding	noncoding
XM_011510955.3	CNNM4	XLOC_025487	2360 coding	coding	coding	coding
MSTRG.28689.9	CNNM3	XLOC_025488	5551 coding	coding	coding	coding
MSTRG.28682.1	FAM178B	XLOC_025505	261 coding	noncoding	noncoding	noncoding
MSTRG.28746.1	ANKRD36	XLOC_025515	297 coding	noncoding	noncoding	noncoding
MSTRG.28722.1		XLOC_025539	253 coding	noncoding	noncoding	noncoding
MSTRG.28817.5	TMEM131	XLOC_025556	1351 coding	coding	noncoding	noncoding
MSTRG.28789.1	VWA3B	XLOC_025564	262 coding	noncoding	noncoding	noncoding
XM_011510554.2	CNGA3	XLOC_025570	4623 coding	coding	coding	coding
NM_001134225.2	INPP4A	XLOC_025572	10096 coding	noncoding	coding	coding
MSTRG.28985.9	UNC50	XLOC_025598	7651 coding	coding	coding	noncoding
MSTRG.28860.1		XLOC_025603	244 coding	noncoding	noncoding	noncoding
MSTRG.28906.1	TSGA10	XLOC_025624	260 coding	noncoding	noncoding	noncoding
NM_145198.3	LIPT1	XLOC_025640	1508 coding	noncoding	coding	coding
MSTRG.28932.2	MITD1	XLOC_025642	914 coding	coding	noncoding	noncoding
MSTRG.28938.1		XLOC_025644	240 coding	noncoding	noncoding	noncoding
MSTRG.28953.1		XLOC_025655	297 coding	noncoding	noncoding	noncoding
MSTRG.29663.1	AFF3	XLOC_025659	1985 coding	coding	coding	noncoding
MSTRG.29664.4	AFF3	XLOC_025660	1027 coding	coding	coding	noncoding
MSTRG.29020.1		XLOC_025670	241 coding	noncoding	noncoding	noncoding
MSTRG.29022.1		XLOC_025672	268 coding	noncoding	noncoding	noncoding
MSTRG.29184.15	CNOT11	XLOC_025708	6452 coding	coding	coding	noncoding
MSTRG.29082.1	CREG2	XLOC_025711	282 coding	noncoding	noncoding	noncoding
MSTRG.29088.1		XLOC_025723	420 coding	noncoding	noncoding	noncoding
MSTRG.29093.1		XLOC_025724	235 coding	noncoding	noncoding	noncoding
MSTRG.29117.1		XLOC_025726	296 coding	noncoding	noncoding	noncoding
MSTRG.29305.1	IL18RAP	XLOC_025837	979 coding	noncoding	noncoding	noncoding
MSTRG.29333.1		XLOC_025871	245 coding	noncoding	noncoding	noncoding
MSTRG.29366.1		XLOC_025888	322 coding	noncoding	noncoding	noncoding
XM_017005103.1	NCK2	XLOC_025942	2775 coding	coding	coding	coding
MSTRG.29593.1		XLOC_025971	259 coding	noncoding	noncoding	noncoding
MSTRG.29523.2	UXS1	XLOC_025980	2010 coding	coding	noncoding	noncoding

MSTRG.29508.1		XLOC_025981	242 coding	noncoding	noncoding	noncoding
MSTRG.29510.1		XLOC_025982	307 coding	noncoding	noncoding	noncoding
MSTRG.29512.1		XLOC_025983	250 coding	noncoding	noncoding	noncoding
MSTRG.29551.1	RGPD3	XLOC_025992	276 coding	noncoding	noncoding	noncoding
MSTRG.29552.1	RGPD3	XLOC_025993	279 coding	noncoding	noncoding	noncoding
MSTRG.29542.1		XLOC_026000	243 coding	noncoding	noncoding	noncoding
MSTRG.29602.1		XLOC_026004	314 coding	noncoding	noncoding	noncoding
MSTRG.29604.1		XLOC_026008	243 coding	noncoding	noncoding	noncoding
MSTRG.29621.1	LINC01885	XLOC_026013	248 coding	noncoding	noncoding	noncoding
MSTRG.29631.1	GACAT1	XLOC_026023	288 coding	noncoding	noncoding	noncoding
MSTRG.29643.1	RGPD4	XLOC_026026	417 coding	noncoding	noncoding	noncoding
NM_006588.4	SULT1C4	XLOC_026037	2850 coding	coding	coding	coding
MSTRG.29729.1	LIMS1	XLOC_026054	227 coding	noncoding	noncoding	noncoding
XM_017003492.1	CCDC138	XLOC_026064	4743 coding	coding	coding	coding
MSTRG.29658.1	CCDC138	XLOC_026066	300 coding	noncoding	noncoding	noncoding
MSTRG.29916.1	SH3RF3	XLOC_026086	228 coding	noncoding	noncoding	noncoding
XR_002959478.1	LOC112268438	XLOC_026116	3465 coding	noncoding	coding	noncoding
MSTRG.29792.1	LOC105373548	XLOC_026117	248 coding	noncoding	noncoding	noncoding
MSTRG.29816.1	NPHP1	XLOC_026124	218 coding	noncoding	noncoding	noncoding
MSTRG.29988.1	ACOXL	XLOC_026163	264 coding	noncoding	noncoding	noncoding
NM_138624.3	BCL2L11	XLOC_026166	5132 coding	coding	noncoding	coding
MSTRG.30020.1	MERTK	XLOC_026189	266 coding	noncoding	noncoding	noncoding
MSTRG.30089.1		XLOC_026204	268 coding	noncoding	noncoding	noncoding
MSTRG.30133.1		XLOC_026211	228 coding	noncoding	noncoding	noncoding
MSTRG.30118.1		XLOC_026216	323 coding	noncoding	noncoding	noncoding
MSTRG.30120.1		XLOC_026218	364 coding	noncoding	noncoding	noncoding
NM_153712.5	TTL	XLOC_026220	14267 coding	coding	coding	coding
MSTRG.30149.1	TTL	XLOC_026222	401 coding	noncoding	noncoding	coding
MSTRG.30184.1		XLOC_026246	352 coding	noncoding	noncoding	noncoding
MSTRG.30186.1		XLOC_026249	388 coding	noncoding	noncoding	noncoding
MSTRG.30293.1	CBWD2	XLOC_026284	264 coding	coding	noncoding	noncoding
MSTRG.30301.1		XLOC_026304	261 coding	noncoding	noncoding	noncoding
MSTRG.30391.1	ACTR3-AS1	XLOC_026318	277 coding	noncoding	noncoding	noncoding
NM_001178037.2	DPP10	XLOC_026361	5971 coding	coding	coding	coding
MSTRG.30483.1	DPP10	XLOC_026370	287 coding	coding	noncoding	noncoding
MSTRG.30495.1	DPP10	XLOC_026376	228 coding	noncoding	noncoding	noncoding
MSTRG.30349.1		XLOC_026389	221 coding	noncoding	noncoding	noncoding
MSTRG.30353.1		XLOC_026391	350 coding	noncoding	noncoding	noncoding
MSTRG.30377.1		XLOC_026403	304 coding	noncoding	noncoding	noncoding
MSTRG.30458.1		XLOC_026417	251 coding	noncoding	noncoding	noncoding
MSTRG.30518.2	DDX18	XLOC_026418	358 coding	coding	noncoding	noncoding
MSTRG.30588.1		XLOC_026451	293 coding	noncoding	noncoding	noncoding
MSTRG.30592.1	LOC105373579	XLOC_026453	231 coding	noncoding	noncoding	noncoding

MSTRG.30615.1		XLOC_026468	272	coding	noncoding	noncoding	noncoding
MSTRG.30668.1	C2orf76	XLOC_026481	295	coding	noncoding	noncoding	noncoding
NM_001352432.1	DBI	XLOC_026486	904	coding	coding	noncoding	noncoding
MSTRG.30676.1	CFAP221	XLOC_026492	224	coding	noncoding	noncoding	noncoding
MSTRG.30798.1	LOC105373582	XLOC_026532	676	coding	noncoding	noncoding	noncoding
MSTRG.30677.1		XLOC_026533	263	coding	noncoding	noncoding	noncoding
NM_001369400.1	RALB	XLOC_026535	2250	coding	coding	coding	coding
MSTRG.30731.1	LOC105373585	XLOC_026542	286	coding	noncoding	noncoding	noncoding
MSTRG.30732.1		XLOC_026543	296	coding	noncoding	noncoding	noncoding
MSTRG.30748.1		XLOC_026558	236	coding	noncoding	noncoding	noncoding
MSTRG.30810.1		XLOC_026577	279	coding	coding	noncoding	noncoding
MSTRG.30815.1	LOC105373590	XLOC_026579	221	coding	noncoding	noncoding	noncoding
MSTRG.30842.1	LOC105373592	XLOC_026586	231	coding	noncoding	noncoding	noncoding
MSTRG.30850.1	LOC105373592	XLOC_026588	298	coding	noncoding	noncoding	noncoding
MSTRG.30826.1		XLOC_026596	205	coding	noncoding	noncoding	noncoding
MSTRG.30883.1		XLOC_026606	276	coding	noncoding	noncoding	noncoding
MSTRG.30895.1		XLOC_026612	244	coding	noncoding	noncoding	noncoding
MSTRG.30909.1		XLOC_026617	251	coding	noncoding	noncoding	noncoding
MSTRG.30965.1	CNTNAP5	XLOC_026632	533	coding	noncoding	noncoding	noncoding
MSTRG.30915.1		XLOC_026640	325	coding	noncoding	noncoding	noncoding
MSTRG.30924.1		XLOC_026645	280	coding	noncoding	noncoding	noncoding
MSTRG.31179.1		XLOC_026726	251	coding	noncoding	noncoding	noncoding
MSTRG.31285.1	UGGT1	XLOC_026736	297	coding	noncoding	noncoding	noncoding
MSTRG.31235.2	LOC105373611	XLOC_026752	283	coding	noncoding	noncoding	noncoding
MSTRG.31230.1		XLOC_026765	206	coding	noncoding	noncoding	noncoding
MSTRG.31233.1		XLOC_026768	258	coding	noncoding	noncoding	noncoding
MSTRG.31264.1		XLOC_026773	260	coding	noncoding	noncoding	noncoding
MSTRG.31307.1		XLOC_026784	370	coding	noncoding	noncoding	noncoding
NR_110285.1	LINC01856	XLOC_026795	2398	coding	noncoding	noncoding	noncoding
MSTRG.31329.1		XLOC_026798	370	coding	noncoding	noncoding	noncoding
MSTRG.31339.1		XLOC_026808	248	coding	noncoding	noncoding	noncoding
MSTRG.31361.3	CCDC115	XLOC_026813	1532	coding	noncoding	coding	noncoding
XM_011510793.2	AMER3	XLOC_026835	7278	coding	coding	coding	coding
MSTRG.31403.1		XLOC_026839	253	coding	noncoding	noncoding	noncoding
MSTRG.31421.1		XLOC_026849	273	coding	coding	noncoding	noncoding
NR_033885.3	POTEKP	XLOC_026860	1530	coding	coding	coding	coding
MSTRG.31505.1	GPR39	XLOC_026880	287	coding	noncoding	noncoding	noncoding
MSTRG.31541.1	NCKAP5	XLOC_026900	236	coding	noncoding	noncoding	noncoding
MSTRG.31559.1	NCKAP5	XLOC_026909	262	coding	noncoding	noncoding	noncoding
MSTRG.31575.1	NCKAP5	XLOC_026919	241	coding	coding	noncoding	noncoding
MSTRG.31576.1	NCKAP5	XLOC_026920	264	coding	noncoding	noncoding	noncoding
MSTRG.31578.1	NCKAP5	XLOC_026921	308	coding	noncoding	noncoding	noncoding
MSTRG.31481.1		XLOC_026925	387	coding	noncoding	noncoding	noncoding

MSTRG.31492.1	LOC105373628	XLOC_026932	422 coding	noncoding	noncoding	noncoding
MSTRG.31833.1	MGAT5	XLOC_026943	226 coding	noncoding	noncoding	noncoding
NR_037649.1	CCNT2	XLOC_027025	6909 coding	coding	coding	coding
MSTRG.31587.1	MAP3K19	XLOC_027033	307 coding	noncoding	noncoding	noncoding
MSTRG.31588.1	MAP3K19	XLOC_027034	259 coding	noncoding	noncoding	noncoding
MSTRG.31911.1	ZRANB3	XLOC_027050	271 coding	noncoding	noncoding	noncoding
MSTRG.31928.1	ZRANB3	XLOC_027065	219 coding	noncoding	noncoding	noncoding
MSTRG.31960.1	R3HDM1	XLOC_027093	202 coding	noncoding	noncoding	noncoding
MSTRG.31690.1		XLOC_027122	400 coding	noncoding	noncoding	noncoding
MSTRG.31693.1		XLOC_027123	316 coding	noncoding	noncoding	noncoding
MSTRG.31713.1		XLOC_027131	324 coding	noncoding	noncoding	noncoding
MSTRG.31760.1	THSD7B	XLOC_027150	255 coding	noncoding	noncoding	noncoding
MSTRG.31722.1		XLOC_027157	277 coding	noncoding	noncoding	noncoding
MSTRG.31773.1	LOC105373636	XLOC_027169	267 coding	noncoding	noncoding	noncoding
MSTRG.31815.1		XLOC_027197	428 coding	noncoding	noncoding	noncoding
XM_005263655.5	SPOPL	XLOC_027283	5614 coding	coding	coding	coding
MSTRG.31980.1		XLOC_027320	425 coding	noncoding	noncoding	noncoding
MSTRG.31996.1	LOC105373644	XLOC_027336	281 coding	noncoding	noncoding	noncoding
MSTRG.32081.1	LRP1B	XLOC_027353	274 coding	noncoding	noncoding	noncoding
MSTRG.32115.1	LRP1B	XLOC_027371	273 coding	noncoding	noncoding	noncoding
XR_001739132.2	LOC107985779	XLOC_027376	9126 coding	noncoding	noncoding	noncoding
XR_923383.3	LOC105373648	XLOC_027379	7129 coding	noncoding	noncoding	noncoding
MSTRG.32151.1	LRP1B	XLOC_027388	233 coding	noncoding	noncoding	noncoding
MSTRG.32032.1	LOC105373651	XLOC_027391	233 coding	noncoding	noncoding	noncoding
MSTRG.32050.1		XLOC_027398	279 coding	noncoding	noncoding	noncoding
MSTRG.32689.7	GTDC1	XLOC_027631	1332 coding	noncoding	noncoding	noncoding
MSTRG.32689.13	GTDC1	XLOC_027631	748 coding	coding	noncoding	noncoding
MSTRG.32689.14	GTDC1	XLOC_027631	1045 coding	coding	noncoding	noncoding
MSTRG.32689.16	GTDC1	XLOC_027631	1036 coding	coding	noncoding	noncoding
MSTRG.32434.25	ZEB2-AS1	XLOC_027633	3753 coding	noncoding	coding	noncoding
MSTRG.32316.1	TEX41	XLOC_027641	289 coding	noncoding	noncoding	noncoding
MSTRG.32397.1		XLOC_027697	291 coding	noncoding	noncoding	noncoding
MSTRG.32418.1		XLOC_027704	282 coding	noncoding	noncoding	noncoding
MSTRG.33279.6	ORC4	XLOC_027715	398 coding	noncoding	noncoding	noncoding
MSTRG.33334.1	MBD5	XLOC_027765	266 coding	noncoding	noncoding	noncoding
MSTRG.32463.1		XLOC_027801	221 coding	noncoding	noncoding	noncoding
MSTRG.32467.1		XLOC_027837	249 coding	noncoding	noncoding	noncoding
XR_244868.1	LOC101928526	XLOC_027838	3063 coding	noncoding	coding	noncoding
MSTRG.32541.1	LYPD6	XLOC_027860	309 coding	noncoding	noncoding	noncoding
MSTRG.32532.1		XLOC_027861	374 coding	noncoding	noncoding	noncoding
MSTRG.32570.1		XLOC_027865	298 coding	noncoding	noncoding	noncoding
MSTRG.32577.1		XLOC_027869	266 coding	noncoding	noncoding	noncoding
MSTRG.32619.1	LINC01818	XLOC_027876	272 coding	noncoding	noncoding	noncoding

MSTRG.32664.1		XLOC_027898	283	coding	noncoding	noncoding	noncoding
MSTRG.32679.1	LOC101929319	XLOC_027900	321	coding	noncoding	noncoding	noncoding
MSTRG.32673.1		XLOC_027902	268	coding	noncoding	noncoding	noncoding
NM_018151.5	RIF1	XLOC_027904	14662	coding	coding	coding	coding
MSTRG.33473.1	RIF1	XLOC_027906	278	coding	noncoding	noncoding	noncoding
MSTRG.33493.1	NEB	XLOC_027919	313	coding	noncoding	noncoding	noncoding
MSTRG.33495.1	NEB	XLOC_027921	853	coding	noncoding	noncoding	noncoding
MSTRG.32748.1		XLOC_027930	210	coding	noncoding	noncoding	noncoding
MSTRG.33441.16	ARL6IP6	XLOC_027972	10797	coding	coding	coding	coding
MSTRG.33441.21	PRPF40A	XLOC_027972	2497	coding	coding	coding	coding
MSTRG.32804.1		XLOC_027983	296	coding	noncoding	noncoding	noncoding
MSTRG.32826.1		XLOC_027993	267	coding	noncoding	noncoding	noncoding
MSTRG.32831.1		XLOC_027995	374	coding	coding	noncoding	noncoding
MSTRG.32837.1		XLOC_027998	250	coding	coding	noncoding	noncoding
MSTRG.32864.1	GALNT13	XLOC_028003	265	coding	noncoding	noncoding	noncoding
MSTRG.32875.1	GALNT13	XLOC_028008	275	coding	noncoding	noncoding	noncoding
MSTRG.32877.1	GALNT13	XLOC_028009	244	coding	noncoding	noncoding	noncoding
MSTRG.32878.1	GALNT13	XLOC_028010	247	coding	noncoding	noncoding	noncoding
MSTRG.32851.1	LOC105373693	XLOC_028016	307	coding	coding	noncoding	noncoding
NM_002239.4	KCNJ3	XLOC_028019	4628	coding	coding	coding	coding
MSTRG.32890.1		XLOC_028028	216	coding	noncoding	noncoding	noncoding
MSTRG.32900.1		XLOC_028037	285	coding	noncoding	noncoding	noncoding
XM_017003830.1	GPD2	XLOC_028104	5636	coding	coding	coding	coding
MSTRG.33011.1		XLOC_028125	249	coding	noncoding	noncoding	noncoding
XM_017003237.2	GALNT5	XLOC_028139	10441	coding	coding	coding	coding
MSTRG.33705.1	CCDC148	XLOC_028205	221	coding	noncoding	noncoding	noncoding
MSTRG.33714.1	PKP4	XLOC_028212	253	coding	noncoding	noncoding	noncoding
MSTRG.33723.1	PKP4	XLOC_028220	322	coding	noncoding	noncoding	noncoding
MSTRG.33736.1	PKP4-AS1	XLOC_028232	207	coding	coding	noncoding	noncoding
XM_005246774.1	7-Mar	XLOC_028256	3484	coding	coding	coding	coding
MSTRG.33677.1	ITGB6	XLOC_028274	259	coding	coding	noncoding	noncoding
MSTRG.33754.1		XLOC_028293	279	coding	noncoding	noncoding	noncoding
MSTRG.33772.1		XLOC_028336	200	coding	noncoding	noncoding	noncoding
XM_011511513.2	SLC4A10	XLOC_028344	5952	coding	coding	coding	coding
MSTRG.33835.1	FAP	XLOC_028365	254	coding	noncoding	noncoding	noncoding
NM_001330270.2	GCA	XLOC_028372	3736	coding	coding	coding	coding
MSTRG.34074.1	KCNH7	XLOC_028397	247	coding	noncoding	noncoding	noncoding
MSTRG.34083.1	KCNH7	XLOC_028400	306	coding	noncoding	noncoding	noncoding
MSTRG.33957.1		XLOC_028410	271	coding	noncoding	noncoding	noncoding
MSTRG.33964.1		XLOC_028414	260	coding	noncoding	noncoding	noncoding
XM_017004658.1	SCN2A	XLOC_028484	8974	coding	coding	coding	coding
MSTRG.34189.1		XLOC_028557	342	coding	noncoding	noncoding	noncoding
MSTRG.34191.1		XLOC_028559	205	coding	noncoding	noncoding	noncoding

XM_017003309.1	XIRP2	XLOC_028561	12170 coding	coding	noncoding	coding
MSTRG.34340.1	LRP2	XLOC_028637	234 coding	noncoding	noncoding	noncoding
MSTRG.34316.1		XLOC_028638	306 coding	noncoding	noncoding	noncoding
MSTRG.34367.1	CCDC173	XLOC_028656	277 coding	coding	noncoding	noncoding
MSTRG.34368.1	CCDC173	XLOC_028657	263 coding	noncoding	noncoding	noncoding
MSTRG.34478.1	UBR3	XLOC_028668	286 coding	noncoding	noncoding	noncoding
XM_011510658.3	MYO3B	XLOC_028691	5067 coding	coding	coding	coding
MSTRG.34525.1	MYO3B	XLOC_028692	283 coding	noncoding	noncoding	noncoding
MSTRG.34529.1	MYO3B	XLOC_028695	273 coding	noncoding	noncoding	noncoding
XM_011511159.2	SP5	XLOC_028703	3110 coding	coding	coding	coding
MSTRG.34511.1	LOC101926913	XLOC_028705	239 coding	noncoding	noncoding	noncoding
MSTRG.34590.1	TLK1	XLOC_028718	232 coding	noncoding	noncoding	noncoding
MSTRG.34613.4	DCAF17	XLOC_028726	5527 coding	coding	coding	coding
MSTRG.34621.1	DCAF17	XLOC_028734	290 coding	noncoding	noncoding	noncoding
MSTRG.34635.1		XLOC_028762	291 coding	noncoding	noncoding	noncoding
MSTRG.34651.1	METAP1D	XLOC_028765	271 coding	noncoding	noncoding	noncoding
NM_001316306.2	ITGA6	XLOC_028781	5551 coding	coding	coding	coding
MSTRG.34831.1	RAPGEF4	XLOC_028802	216 coding	noncoding	noncoding	noncoding
MSTRG.34832.1	RAPGEF4	XLOC_028803	295 coding	noncoding	noncoding	noncoding
MSTRG.34739.1	MAP3K20	XLOC_028818	261 coding	coding	noncoding	noncoding
MSTRG.34726.1		XLOC_028833	315 coding	noncoding	noncoding	noncoding
MSTRG.34732.1		XLOC_028839	264 coding	noncoding	noncoding	noncoding
MSTRG.34762.1		XLOC_028865	261 coding	noncoding	noncoding	noncoding
MSTRG.34860.3	CIR1	XLOC_028868	1724 coding	coding	noncoding	noncoding
MSTRG.34937.1	CHN1	XLOC_028889	313 coding	noncoding	noncoding	noncoding
MSTRG.34941.7	ATF2	XLOC_028892	2138 coding	coding	coding	noncoding
MSTRG.34877.1		XLOC_028897	239 coding	noncoding	noncoding	noncoding
MSTRG.34881.1	LOC105373752	XLOC_028900	265 coding	noncoding	noncoding	noncoding
MSTRG.34893.1	LOC107985962	XLOC_028904	256 coding	coding	noncoding	noncoding
MSTRG.34901.1	LOC107985830	XLOC_028910	222 coding	noncoding	noncoding	noncoding
XM_011511068.2	HOXD13	XLOC_028912	2638 coding	coding	coding	coding
XM_011511065.3	HOXD3	XLOC_028921	4374 coding	coding	coding	coding
MSTRG.35099.20	NFE2L2	XLOC_028952	2561 coding	coding	noncoding	noncoding
MSTRG.35044.1	PDE11A	XLOC_028988	252 coding	noncoding	noncoding	noncoding
MSTRG.35053.1	PDE11A	XLOC_028990	276 coding	noncoding	noncoding	noncoding
MSTRG.35078.1	OSBPL6	XLOC_029003	278 coding	noncoding	noncoding	noncoding
MSTRG.35160.1		XLOC_029032	225 coding	noncoding	noncoding	noncoding
MSTRG.35162.1		XLOC_029034	258 coding	noncoding	noncoding	noncoding
MSTRG.35186.1	ZNF385B	XLOC_029042	325 coding	noncoding	noncoding	noncoding
MSTRG.35198.1		XLOC_029059	294 coding	noncoding	noncoding	noncoding
MSTRG.35214.1		XLOC_029069	261 coding	noncoding	noncoding	noncoding
MSTRG.35225.1		XLOC_029073	356 coding	noncoding	noncoding	noncoding
MSTRG.35229.1		XLOC_029075	266 coding	noncoding	noncoding	noncoding

MSTRG.35260.1	SCHLAP1	XLOC_029081	252 coding	noncoding	noncoding	noncoding
XM_005246244.2	UBE2E3	XLOC_029085	1538 coding	coding	coding	coding
MSTRG.35287.1		XLOC_029105	279 coding	noncoding	noncoding	noncoding
MSTRG.35319.1		XLOC_029158	237 coding	coding	noncoding	noncoding
MSTRG.35358.1	PDE1A	XLOC_029177	330 coding	coding	noncoding	noncoding
MSTRG.35418.1		XLOC_029223	291 coding	noncoding	noncoding	noncoding
MSTRG.35443.1		XLOC_029231	322 coding	noncoding	noncoding	noncoding
MSTRG.35450.1	LOC105373777	XLOC_029232	303 coding	noncoding	noncoding	noncoding
MSTRG.35464.1	LOC102724340	XLOC_029241	270 coding	coding	noncoding	noncoding
MSTRG.35525.1		XLOC_029255	225 coding	noncoding	noncoding	noncoding
MSTRG.35729.1		XLOC_029353	292 coding	noncoding	noncoding	noncoding
MSTRG.35731.1		XLOC_029354	473 coding	noncoding	noncoding	noncoding
MSTRG.35745.1		XLOC_029359	395 coding	noncoding	noncoding	noncoding
MSTRG.35743.1		XLOC_029360	303 coding	noncoding	noncoding	noncoding
MSTRG.35794.1	CALCRL	XLOC_029367	344 coding	coding	noncoding	noncoding
MSTRG.35800.2	TFPI	XLOC_029371	851 coding	coding	noncoding	noncoding
MSTRG.35765.1		XLOC_029379	248 coding	noncoding	noncoding	noncoding
MSTRG.35767.1		XLOC_029381	206 coding	noncoding	noncoding	noncoding
XR_002959305.1	GULP1	XLOC_029386	5485 coding	coding	noncoding	coding
MSTRG.35824.1	GULP1	XLOC_029389	239 coding	noncoding	noncoding	noncoding
MSTRG.35814.1	LOC105373790	XLOC_029399	277 coding	noncoding	noncoding	noncoding
NM_000090.3	COL3A1	XLOC_029403	5490 coding	coding	coding	coding
MSTRG.35854.1		XLOC_029418	255 coding	noncoding	noncoding	noncoding
MSTRG.35884.1		XLOC_029448	600 coding	noncoding	noncoding	noncoding
XM_011511356.3	PMS1	XLOC_029452	3901 coding	coding	coding	coding
MSTRG.36175.1	ANKAR	XLOC_029460	386 coding	noncoding	noncoding	noncoding
MSTRG.36193.1	C2orf88	XLOC_029466	262 coding	noncoding	noncoding	noncoding
MSTRG.35965.1	NAB1	XLOC_029514	338 coding	noncoding	noncoding	noncoding
MSTRG.35941.1		XLOC_029526	272 coding	noncoding	noncoding	noncoding
MSTRG.36327.2	GLS	XLOC_029538	12583 coding	coding	coding	coding
MSTRG.36152.1		XLOC_029553	265 coding	noncoding	noncoding	noncoding
MSTRG.36250.1		XLOC_029561	318 coding	noncoding	noncoding	noncoding
MSTRG.36332.1	LOC107985969	XLOC_029605	432 coding	noncoding	noncoding	noncoding
MSTRG.36400.1		XLOC_029611	895 coding	coding	noncoding	noncoding
MSTRG.36413.1		XLOC_029614	382 coding	coding	noncoding	noncoding
MSTRG.36444.1	LINC01821	XLOC_029624	225 coding	noncoding	noncoding	noncoding
NM_001127257.2	SLC39A10	XLOC_029726	5219 coding	coding	coding	coding
XM_005246689.4	SLC39A10	XLOC_029726	5328 coding	coding	coding	coding
MSTRG.36648.1	DNAH7	XLOC_029743	213 coding	noncoding	noncoding	noncoding
MSTRG.36657.1	DNAH7	XLOC_029752	258 coding	coding	noncoding	noncoding
MSTRG.36658.1	DNAH7	XLOC_029753	227 coding	noncoding	noncoding	noncoding
MSTRG.36659.1	DNAH7	XLOC_029754	325 coding	coding	noncoding	noncoding
MSTRG.36615.1	HECW2	XLOC_029773	283 coding	noncoding	noncoding	noncoding

MSTRG.36815.8	ANKRD44	XLOC_029810	6043	coding	coding	coding	coding
MSTRG.36815.13	ANKRD44	XLOC_029810	7759	coding	noncoding	coding	coding
MSTRG.36815.23	ANKRD44	XLOC_029810	5317	coding	noncoding	coding	noncoding
MSTRG.36705.2	SF3B1	XLOC_029814	8742	coding	coding	noncoding	noncoding
MSTRG.36720.1	RFTN2	XLOC_029822	212	coding	noncoding	noncoding	noncoding
MSTRG.36721.1	RFTN2	XLOC_029823	232	coding	noncoding	noncoding	noncoding
MSTRG.36713.1		XLOC_029826	277	coding	noncoding	noncoding	noncoding
MSTRG.36714.1		XLOC_029827	282	coding	coding	noncoding	noncoding
MSTRG.36743.1	BOLL	XLOC_029831	363	coding	noncoding	noncoding	noncoding
MSTRG.36746.1	BOLL	XLOC_029833	236	coding	noncoding	noncoding	noncoding
MSTRG.36747.1	BOLL	XLOC_029834	209	coding	noncoding	noncoding	noncoding
MSTRG.36772.1	LINC01923	XLOC_029885	216	coding	noncoding	noncoding	noncoding
MSTRG.36776.1		XLOC_029887	267	coding	noncoding	noncoding	noncoding
MSTRG.36760.1		XLOC_029893	288	coding	noncoding	noncoding	noncoding
MSTRG.36860.1		XLOC_029936	273	coding	noncoding	noncoding	noncoding
MSTRG.36863.1		XLOC_029937	323	coding	noncoding	noncoding	noncoding
MSTRG.36961.1	SGO2	XLOC_029959	301	coding	noncoding	noncoding	noncoding
MSTRG.36951.1	AOX1	XLOC_029961	573	coding	coding	noncoding	noncoding
MSTRG.36952.1	AOX1	XLOC_029962	249	coding	noncoding	noncoding	noncoding
MSTRG.36980.1		XLOC_029967	240	coding	noncoding	coding	noncoding
MSTRG.36995.1	CLK1	XLOC_029969	2432	coding	coding	noncoding	noncoding
MSTRG.37114.6	FAM126B	XLOC_029972	8621	coding	noncoding	noncoding	noncoding
MSTRG.37114.17	LOC105373835	XLOC_029972	3696	coding	coding	noncoding	noncoding
MSTRG.37114.19	LOC105373835	XLOC_029972	3586	coding	coding	noncoding	noncoding
XM_017005191.2	CFLAR	XLOC_029972	8634	coding	coding	coding	coding
NM_001308042.3	CFLAR	XLOC_029972	11686	coding	coding	coding	coding
MSTRG.37146.5	NDUFB3	XLOC_029975	777	coding	coding	coding	noncoding
MSTRG.37179.1	CASP8	XLOC_029994	263	coding	noncoding	noncoding	noncoding
XM_017004438.1	STRADB	XLOC_030000	2128	coding	coding	coding	coding
MSTRG.37003.1	C2CD6	XLOC_030002	305	coding	noncoding	noncoding	noncoding
XM_011511655.2	CDK15	XLOC_030023	3680	coding	coding	coding	coding
MSTRG.37020.1		XLOC_030034	445	coding	noncoding	noncoding	noncoding
MSTRG.37043.1	KIAA2012	XLOC_030037	259	coding	noncoding	noncoding	noncoding
MSTRG.37036.1		XLOC_030045	272	coding	noncoding	noncoding	noncoding
MSTRG.37078.1		XLOC_030046	256	coding	noncoding	noncoding	noncoding
MSTRG.37090.1		XLOC_030055	511	coding	noncoding	noncoding	noncoding
MSTRG.37109.1	BMPR2	XLOC_030062	239	coding	noncoding	noncoding	noncoding
MSTRG.37242.1	ICA1L	XLOC_030093	269	coding	noncoding	noncoding	noncoding
MSTRG.37253.1	NBEAL1	XLOC_030105	287	coding	noncoding	noncoding	noncoding
NM_177538.2	CYP20A1	XLOC_030107	10943	coding	coding	coding	coding
XM_005246701.4	CYP20A1	XLOC_030107	1925	coding	coding	coding	coding
MSTRG.37323.1	ABI2	XLOC_030112	234	coding	noncoding	noncoding	noncoding
MSTRG.37269.1		XLOC_030124	261	coding	noncoding	noncoding	noncoding

MSTRG.37274.1		XLOC_030130	260 coding	coding	noncoding	noncoding
MSTRG.37440.1	PARD3B	XLOC_030167	253 coding	noncoding	noncoding	noncoding
MSTRG.37357.1		XLOC_030183	411 coding	noncoding	noncoding	noncoding
MSTRG.37393.1		XLOC_030203	254 coding	noncoding	noncoding	noncoding
MSTRG.37506.1	ADAM23	XLOC_030211	395 coding	coding	noncoding	noncoding
MSTRG.37494.1	DYTN	XLOC_030217	245 coding	coding	noncoding	noncoding
MSTRG.37540.1	CPO	XLOC_030225	615 coding	noncoding	noncoding	noncoding
MSTRG.37544.1		XLOC_030260	274 coding	coding	coding	noncoding
MSTRG.37547.1		XLOC_030261	304 coding	noncoding	noncoding	noncoding
MSTRG.37669.1	PTH2R	XLOC_030299	257 coding	noncoding	noncoding	noncoding
MSTRG.37693.1	LOC101927960	XLOC_030309	249 coding	noncoding	noncoding	noncoding
MSTRG.37697.1	LOC105373856	XLOC_030310	374 coding	noncoding	noncoding	noncoding
MSTRG.37603.1		XLOC_030327	261 coding	noncoding	noncoding	noncoding
MSTRG.37608.1		XLOC_030330	357 coding	noncoding	noncoding	noncoding
XM_017004112.2	MAP2	XLOC_030331	9944 coding	coding	coding	coding
MSTRG.37624.1	MAP2	XLOC_030336	324 coding	noncoding	noncoding	noncoding
XM_017003891.1	UNC80	XLOC_030340	13820 coding	coding	noncoding	coding
MSTRG.37632.1	UNC80	XLOC_030341	286 coding	noncoding	noncoding	noncoding
MSTRG.37707.1	ACADL	XLOC_030347	238 coding	noncoding	noncoding	noncoding
MSTRG.37742.1	CPS1	XLOC_030354	288 coding	noncoding	noncoding	noncoding
MSTRG.37724.1		XLOC_030358	366 coding	coding	coding	noncoding
MSTRG.37746.1		XLOC_030360	261 coding	noncoding	noncoding	noncoding
MSTRG.37789.1	ERBB4	XLOC_030372	314 coding	noncoding	noncoding	noncoding
MSTRG.37761.1		XLOC_030391	288 coding	coding	noncoding	noncoding
XM_017004899.2	SPAG16	XLOC_030484	3461 coding	noncoding	coding	coding
MSTRG.37988.1	SPAG16	XLOC_030492	302 coding	noncoding	noncoding	noncoding
MSTRG.37993.1	SPAG16	XLOC_030493	324 coding	coding	noncoding	noncoding
MSTRG.38015.1	SPAG16	XLOC_030504	255 coding	noncoding	noncoding	noncoding
MSTRG.37970.1		XLOC_030568	247 coding	noncoding	noncoding	noncoding
MSTRG.38075.1	SNHG31	XLOC_030580	260 coding	noncoding	noncoding	noncoding
MSTRG.38154.1	FN1	XLOC_030599	283 coding	noncoding	noncoding	noncoding
MSTRG.38164.1	FN1	XLOC_030607	326 coding	coding	noncoding	noncoding
MSTRG.38112.1		XLOC_030629	329 coding	noncoding	noncoding	noncoding
MSTRG.38359.1	TMEM169	XLOC_030645	265 coding	noncoding	noncoding	noncoding
NM_000998.5	RPL37A	XLOC_030676	2992 coding	coding	coding	noncoding
MSTRG.38374.1	LOC101928278	XLOC_030689	281 coding	noncoding	noncoding	noncoding
MSTRG.38385.1	LOC101928278	XLOC_030695	314 coding	noncoding	noncoding	noncoding
MSTRG.38387.1	LOC101928278	XLOC_030696	202 coding	noncoding	noncoding	noncoding
MSTRG.38393.5	TNS1	XLOC_030790	6158 coding	coding	coding	coding
XM_017003115.2	ARPC2	XLOC_030807	1397 coding	coding	coding	coding
XM_017003771.1	PNKD	XLOC_030810	2934 coding	coding	coding	coding
MSTRG.38589.1	TMBIM1	XLOC_030811	2383 coding	coding	coding	coding
MSTRG.38594.1	LOC105373881	XLOC_030812	277 coding	noncoding	noncoding	noncoding

MSTRG.38603.1	CATIP-AS2	XLOC_030814	303	coding	noncoding	noncoding	noncoding
MSTRG.38612.1		XLOC_030819	265	coding	noncoding	noncoding	noncoding
MSTRG.38615.1	SLC11A1	XLOC_030821	215	coding	noncoding	noncoding	noncoding
MSTRG.38564.3	USP37	XLOC_030825	3458	coding	coding	coding	noncoding
XM_011512138.3	CNOT9	XLOC_030826	3551	coding	coding	coding	coding
MSTRG.38577.1	CNOT9	XLOC_030828	389	coding	noncoding	noncoding	noncoding
MSTRG.38624.1		XLOC_030841	230	coding	noncoding	noncoding	noncoding
MSTRG.38639.1		XLOC_030848	257	coding	coding	coding	noncoding
MSTRG.38691.1		XLOC_030874	249	coding	noncoding	noncoding	noncoding
MSTRG.38707.1		XLOC_030891	281	coding	noncoding	noncoding	noncoding
MSTRG.38777.1		XLOC_030897	271	coding	noncoding	noncoding	noncoding
MSTRG.38764.1	LOC105373893	XLOC_030918	254	coding	noncoding	noncoding	noncoding
MSTRG.38728.1		XLOC_030924	270	coding	noncoding	noncoding	noncoding
MSTRG.38808.1		XLOC_030939	239	coding	noncoding	noncoding	noncoding
MSTRG.38844.1		XLOC_030947	272	coding	noncoding	noncoding	noncoding
MSTRG.38905.1	LOC105373903	XLOC_030976	363	coding	noncoding	noncoding	noncoding
MSTRG.38907.1	LOC105373903	XLOC_030977	237	coding	noncoding	noncoding	noncoding
MSTRG.38909.1		XLOC_030980	281	coding	noncoding	noncoding	noncoding
MSTRG.38966.1	ACSL3	XLOC_030984	294	coding	noncoding	noncoding	noncoding
MSTRG.38972.1		XLOC_030989	258	coding	noncoding	noncoding	noncoding
MSTRG.38939.1		XLOC_030996	235	coding	noncoding	coding	noncoding
MSTRG.38955.1		XLOC_031007	310	coding	noncoding	noncoding	noncoding
MSTRG.38989.1	LOC105373908	XLOC_031040	280	coding	noncoding	noncoding	noncoding
MSTRG.38990.1		XLOC_031042	243	coding	noncoding	noncoding	noncoding
MSTRG.39051.4	CUL3	XLOC_031052	808	coding	coding	noncoding	noncoding
MSTRG.39047.1		XLOC_031061	258	coding	noncoding	noncoding	noncoding
MSTRG.39101.1		XLOC_031080	313	coding	noncoding	noncoding	noncoding
MSTRG.39173.1		XLOC_031115	239	coding	noncoding	noncoding	noncoding
XR_001738982.2	RHBDD1	XLOC_031126	5611	coding	coding	coding	coding
XM_017005088.1	RHBDD1	XLOC_031126	4929	coding	coding	coding	coding
MSTRG.39403.1	COL4A4	XLOC_031152	241	coding	noncoding	noncoding	noncoding
MSTRG.39422.1	LOC654841	XLOC_031160	291	coding	noncoding	noncoding	noncoding
XM_005246685.4	MFF	XLOC_031162	2033	coding	coding	coding	coding
MSTRG.39192.1		XLOC_031167	446	coding	noncoding	noncoding	noncoding
MSTRG.39304.1	AGFG1	XLOC_031180	211	coding	noncoding	noncoding	noncoding
MSTRG.39316.1		XLOC_031200	264	coding	noncoding	noncoding	noncoding
MSTRG.39325.1		XLOC_031203	304	coding	noncoding	noncoding	noncoding
MSTRG.39340.1		XLOC_031212	291	coding	noncoding	noncoding	noncoding
MSTRG.39368.1	DNER	XLOC_031230	308	coding	noncoding	noncoding	noncoding
MSTRG.39354.1		XLOC_031232	277	coding	noncoding	noncoding	noncoding
MSTRG.39483.1	FBXO36	XLOC_031239	230	coding	noncoding	noncoding	noncoding
MSTRG.39489.1	FBXO36	XLOC_031241	284	coding	noncoding	noncoding	noncoding
MSTRG.39490.1	FBXO36	XLOC_031242	226	coding	noncoding	noncoding	noncoding

MSTRG.39865.15	SP140	XLOC_031254	8395 coding	coding	noncoding	noncoding
MSTRG.39499.1	LOC112268431	XLOC_031287	255 coding	noncoding	noncoding	noncoding
MSTRG.39497.1		XLOC_031289	311 coding	noncoding	noncoding	noncoding
XM_011511914.2	ARMC9	XLOC_031321	2494 coding	noncoding	coding	coding
NM_001271466.4	ARMC9	XLOC_031321	8122 coding	noncoding	coding	coding
MSTRG.39574.1		XLOC_031336	231 coding	noncoding	noncoding	noncoding
MSTRG.39603.1		XLOC_031350	487 coding	noncoding	noncoding	noncoding
MSTRG.39608.1		XLOC_031351	729 coding	noncoding	noncoding	noncoding
MSTRG.39660.1	DIS3L2	XLOC_031364	255 coding	noncoding	noncoding	noncoding
MSTRG.39667.1	DIS3L2	XLOC_031371	205 coding	noncoding	noncoding	noncoding
MSTRG.39673.1	DIS3L2	XLOC_031377	571 coding	noncoding	noncoding	noncoding
MSTRG.39709.1	GIGYF2	XLOC_031401	285 coding	noncoding	noncoding	noncoding
XM_017004083.2	SNORC	XLOC_031403	3572 coding	coding	coding	coding
MSTRG.39720.1	NGEF	XLOC_031405	287 coding	noncoding	noncoding	noncoding
MSTRG.39722.1	NGEF	XLOC_031407	283 coding	noncoding	noncoding	noncoding
MSTRG.39655.1		XLOC_031410	472 coding	noncoding	noncoding	noncoding
NM_005383.2	NEU2	XLOC_031411	1143 coding	coding	coding	coding
MSTRG.39727.1		XLOC_031437	244 coding	coding	noncoding	noncoding
MSTRG.39798.1		XLOC_031463	307 coding	noncoding	noncoding	noncoding
MSTRG.39822.1		XLOC_031477	229 coding	noncoding	noncoding	noncoding
MSTRG.39829.1		XLOC_031488	306 coding	noncoding	noncoding	noncoding
MSTRG.39835.1		XLOC_031493	252 coding	noncoding	noncoding	noncoding
MSTRG.39839.1		XLOC_031496	295 coding	noncoding	noncoding	noncoding
MSTRG.40028.1	AGAP1	XLOC_031499	264 coding	noncoding	noncoding	noncoding
MSTRG.40038.1	AGAP1	XLOC_031502	300 coding	noncoding	noncoding	noncoding
MSTRG.40041.1	AGAP1	XLOC_031504	317 coding	noncoding	noncoding	noncoding
MSTRG.40050.1	AGAP1	XLOC_031508	220 coding	noncoding	noncoding	noncoding
MSTRG.40063.1	AGAP1	XLOC_031520	286 coding	noncoding	noncoding	noncoding
MSTRG.40094.1	AGAP1	XLOC_031527	213 coding	noncoding	noncoding	noncoding
MSTRG.39928.1		XLOC_031528	290 coding	noncoding	noncoding	noncoding
MSTRG.39921.1		XLOC_031529	407 coding	noncoding	noncoding	noncoding
NM_020311.3	ACKR3	XLOC_031536	2015 coding	coding	coding	coding
MSTRG.40014.1		XLOC_031586	251 coding	noncoding	noncoding	noncoding
MSTRG.40108.1	MLPH	XLOC_031603	253 coding	noncoding	noncoding	noncoding
MSTRG.40110.1	MLPH	XLOC_031604	317 coding	noncoding	noncoding	noncoding
MSTRG.40127.1		XLOC_031606	376 coding	noncoding	noncoding	noncoding
XM_017003153.2	RAMP1	XLOC_031635	1299 coding	coding	coding	coding
NR_103500.1	UBE2F	XLOC_031639	2305 coding	coding	noncoding	noncoding
NM_194312.4	ESPNL	XLOC_031648	4653 coding	coding	coding	coding
XM_011510947.2	TRAF3IP1	XLOC_031671	4320 coding	coding	coding	coding
MSTRG.40268.1		XLOC_031677	317 coding	noncoding	noncoding	noncoding
MSTRG.40291.1		XLOC_031695	238 coding	noncoding	noncoding	noncoding
MSTRG.40320.1		XLOC_031729	260 coding	noncoding	noncoding	noncoding

XM_024453115.1	CROCC2	XLOC_031783	7329 coding	coding	coding	coding
MSTRG.40570.15	LOC101927509	XLOC_031797	1610 coding	coding	coding	coding
NM_001321035.1	SEPTIN2	XLOC_031797	3416 coding	coding	coding	coding
NM_001282973.1	SEPTIN2	XLOC_031797	3673 coding	coding	coding	coding
MSTRG.40491.1	BOK-AS1	XLOC_031805	309 coding	noncoding	noncoding	noncoding
NM_032329.6	ING5	XLOC_031815	5197 coding	coding	coding	coding
MSTRG.40613.1	ING5	XLOC_031817	242 coding	noncoding	noncoding	noncoding
MSTRG.20899.1		XLOC_031860	331 coding	noncoding	noncoding	noncoding
MSTRG.20948.1	PXDN	XLOC_031917	228 coding	noncoding	noncoding	noncoding
XM_011510320.3	MYT1L	XLOC_031920	7552 coding	coding	coding	coding
MSTRG.20971.1		XLOC_031936	299 coding	noncoding	noncoding	noncoding
NR_148534.1	RNASEH1	XLOC_031963	3006 coding	coding	coding	coding
MSTRG.21121.1		XLOC_031968	282 coding	noncoding	noncoding	noncoding
MSTRG.21131.1		XLOC_031976	273 coding	noncoding	noncoding	noncoding
MSTRG.21153.1		XLOC_031983	302 coding	noncoding	noncoding	noncoding
MSTRG.21160.1		XLOC_031986	277 coding	noncoding	noncoding	noncoding
MSTRG.21179.1		XLOC_031993	294 coding	noncoding	noncoding	noncoding
MSTRG.21184.1		XLOC_031996	264 coding	noncoding	noncoding	noncoding
MSTRG.21197.1		XLOC_032004	271 coding	noncoding	noncoding	noncoding
MSTRG.21499.1	LINC00487	XLOC_032037	284 coding	noncoding	noncoding	noncoding
MSTRG.21294.1		XLOC_032213	305 coding	noncoding	noncoding	noncoding
NM_001348738.2	KIDINS220	XLOC_032214	8742 coding	coding	coding	coding
MSTRG.21320.1	KIDINS220	XLOC_032228	319 coding	noncoding	noncoding	noncoding
NR_135605.2	MBOAT2	XLOC_032231	7450 coding	coding	coding	coding
NR_135598.1	MBOAT2	XLOC_032231	7740 coding	coding	coding	coding
MSTRG.21336.1		XLOC_032252	413 coding	noncoding	noncoding	noncoding
MSTRG.21414.1	ASAP2	XLOC_032261	253 coding	noncoding	noncoding	noncoding
MSTRG.21363.1		XLOC_032280	294 coding	noncoding	noncoding	noncoding
MSTRG.21375.1		XLOC_032289	300 coding	noncoding	noncoding	noncoding
MSTRG.21395.1	LOC112268412	XLOC_032299	258 coding	noncoding	noncoding	noncoding
XR_001738957.1	NOL10	XLOC_032330	2732 coding	coding	coding	coding
MSTRG.21492.1	NOL10	XLOC_032334	397 coding	noncoding	noncoding	noncoding
MSTRG.21649.1		XLOC_032374	288 coding	noncoding	noncoding	noncoding
MSTRG.21655.1	LOC105373429	XLOC_032376	278 coding	noncoding	noncoding	noncoding
MSTRG.21673.1	GREB1	XLOC_032380	266 coding	noncoding	noncoding	noncoding
MSTRG.21679.1	GREB1	XLOC_032385	259 coding	coding	noncoding	noncoding
MSTRG.21700.1		XLOC_032396	279 coding	noncoding	noncoding	noncoding
MSTRG.21844.1	MIR3681HG	XLOC_032405	281 coding	noncoding	noncoding	noncoding
MSTRG.21864.1	MIR3681HG	XLOC_032417	315 coding	noncoding	coding	noncoding
MSTRG.21746.1	LOC105373436	XLOC_032442	305 coding	noncoding	noncoding	noncoding
MSTRG.21772.1	LOC105373438	XLOC_032458	356 coding	noncoding	noncoding	noncoding
MSTRG.21798.1	LINC00276	XLOC_032468	253 coding	coding	noncoding	noncoding
MSTRG.21802.1		XLOC_032474	280 coding	noncoding	noncoding	noncoding

MSTRG.21983.1	NBAS	XLOC_032484	214 coding	noncoding	noncoding	noncoding
MSTRG.21896.1		XLOC_032513	246 coding	noncoding	noncoding	noncoding
MSTRG.21920.1		XLOC_032532	205 coding	noncoding	noncoding	noncoding
MSTRG.21921.1		XLOC_032533	469 coding	noncoding	noncoding	noncoding
MSTRG.22098.1		XLOC_032619	311 coding	noncoding	noncoding	noncoding
MSTRG.22120.1	LOC105373454	XLOC_032627	252 coding	noncoding	noncoding	noncoding
MSTRG.22374.1		XLOC_032817	225 coding	noncoding	noncoding	noncoding
MSTRG.22537.1	WDR35	XLOC_032852	236 coding	noncoding	noncoding	noncoding
MSTRG.22433.1		XLOC_032865	244 coding	noncoding	noncoding	noncoding
NM_001006946.1	SDC1	XLOC_032869	3293 coding	coding	coding	coding
NM_001282791.2	PUM2	XLOC_032870	6003 coding	coding	coding	coding
NM_001282790.2	PUM2	XLOC_032870	5878 coding	coding	coding	coding
NM_001282752.2	PUM2	XLOC_032870	6252 coding	coding	coding	coding
MSTRG.22572.1		XLOC_032913	431 coding	noncoding	noncoding	noncoding
NM_000384.3	APOB	XLOC_032920	14121 coding	coding	noncoding	coding
MSTRG.22619.1		XLOC_032923	310 coding	noncoding	noncoding	noncoding
MSTRG.22615.1		XLOC_032925	526 coding	noncoding	noncoding	noncoding
MSTRG.22629.1	LOC105374318	XLOC_032933	256 coding	noncoding	noncoding	noncoding
MSTRG.22642.1		XLOC_032946	257 coding	noncoding	noncoding	noncoding
MSTRG.23076.1	ATAD2B	XLOC_033019	290 coding	noncoding	noncoding	noncoding
MSTRG.23082.1	UBXN2A	XLOC_033024	275 coding	noncoding	noncoding	noncoding
MSTRG.22905.1		XLOC_033028	705 coding	noncoding	noncoding	noncoding
MSTRG.22914.1		XLOC_033039	288 coding	noncoding	coding	noncoding
MSTRG.22933.1	LOC105374329	XLOC_033064	223 coding	noncoding	noncoding	noncoding
MSTRG.23291.1		XLOC_033071	352 coding	noncoding	noncoding	noncoding
MSTRG.22962.1		XLOC_033088	314 coding	noncoding	noncoding	noncoding
MSTRG.22964.1		XLOC_033090	246 coding	noncoding	noncoding	noncoding
MSTRG.22999.1	DNAJC27	XLOC_033092	291 coding	noncoding	noncoding	noncoding
NM_001035256.2	POMC	XLOC_033103	1456 coding	coding	coding	coding
MSTRG.23163.1	DTNB	XLOC_033119	234 coding	noncoding	noncoding	noncoding
MSTRG.23165.1	DTNB	XLOC_033120	243 coding	noncoding	noncoding	noncoding
MSTRG.23171.1	DTNB	XLOC_033126	281 coding	noncoding	noncoding	noncoding
MSTRG.23112.1		XLOC_033133	232 coding	noncoding	noncoding	noncoding
MSTRG.23114.1		XLOC_033148	304 coding	noncoding	noncoding	noncoding
MSTRG.23120.1		XLOC_033153	218 coding	noncoding	noncoding	noncoding
MSTRG.23194.1		XLOC_033161	259 coding	noncoding	noncoding	noncoding
XM_017003331.1	CIB4	XLOC_033179	950 coding	noncoding	coding	coding
MSTRG.23296.1		XLOC_033182	213 coding	noncoding	coding	noncoding
MSTRG.23333.1		XLOC_033189	288 coding	noncoding	noncoding	noncoding
NM_001134693.2	OST4	XLOC_033203	442 coding	coding	coding	noncoding
MSTRG.23363.1	KHK	XLOC_033207	208 coding	noncoding	coding	noncoding
XM_011533102.2	SLC30A3	XLOC_033220	2090 coding	coding	coding	coding
MSTRG.23414.1	GTF3C2	XLOC_033226	359 coding	noncoding	noncoding	noncoding

MSTRG.23416.1	GTF3C2	XLOC_033228	294	coding	noncoding	noncoding	noncoding
NM_144631.6	ZNF513	XLOC_033232	2144	coding	coding	coding	coding
NR_002201.1	FTH1P3	XLOC_033234	954	coding	coding	coding	noncoding
MSTRG.23444.3	ZNF512	XLOC_033241	3441	coding	coding	noncoding	noncoding
MSTRG.23428.1		XLOC_033247	281	coding	noncoding	noncoding	noncoding
MSTRG.23611.5	BABAM2-AS1	XLOC_033252	3141	coding	coding	coding	noncoding
MSTRG.23497.1	PLB1	XLOC_033277	254	coding	noncoding	noncoding	noncoding
MSTRG.23502.1	PLB1	XLOC_033280	296	coding	noncoding	noncoding	noncoding
MSTRG.23482.1		XLOC_033286	261	coding	noncoding	noncoding	noncoding
XR_939886.2	LOC112267877	XLOC_033288	3732	coding	noncoding	coding	noncoding
MSTRG.23509.1		XLOC_033302	300	coding	noncoding	noncoding	noncoding
MSTRG.23572.2	LBH	XLOC_033339	5172	coding	coding	coding	noncoding
MSTRG.23604.1		XLOC_033344	416	coding	noncoding	coding	noncoding
XR_001739074.1	CAPN13	XLOC_033349	4085	coding	coding	coding	coding
MSTRG.23797.1	GALNT14	XLOC_033356	308	coding	noncoding	noncoding	noncoding
MSTRG.23747.1	CAPN14	XLOC_033364	202	coding	noncoding	noncoding	noncoding
MSTRG.23738.1		XLOC_033368	290	coding	noncoding	noncoding	noncoding
XM_011533069.2	SRD5A2	XLOC_033374	5261	coding	coding	coding	coding
MSTRG.23853.1	MEMO1	XLOC_033393	282	coding	noncoding	noncoding	noncoding
MSTRG.23855.1	MEMO1	XLOC_033395	301	coding	noncoding	noncoding	noncoding
MSTRG.23856.1	MEMO1	XLOC_033396	568	coding	noncoding	noncoding	noncoding
MSTRG.23866.1	MEMO1	XLOC_033406	303	coding	noncoding	noncoding	noncoding
MSTRG.23868.1	MEMO1	XLOC_033408	280	coding	noncoding	noncoding	noncoding
NM_001321209.2	DPY30	XLOC_033412	688	coding	coding	noncoding	coding
MSTRG.23877.1	DPY30	XLOC_033413	279	coding	noncoding	noncoding	noncoding
MSTRG.24020.8	BIRC6	XLOC_033420	15513	coding	coding	coding	coding
MSTRG.23903.1	LINC00486	XLOC_033427	429	coding	noncoding	noncoding	noncoding
MSTRG.23908.1	LINC00486	XLOC_033430	483	coding	noncoding	noncoding	noncoding
MSTRG.23915.4	LTBP1	XLOC_033434	4554	coding	coding	coding	noncoding
MSTRG.23965.1	RASGRP3	XLOC_033436	260	coding	noncoding	noncoding	noncoding
MSTRG.23968.3	RASGRP3	XLOC_033438	2243	coding	coding	noncoding	noncoding
MSTRG.23950.1		XLOC_033465	246	coding	noncoding	noncoding	noncoding
MSTRG.23952.1		XLOC_033469	225	coding	noncoding	noncoding	noncoding
MSTRG.23957.1		XLOC_033475	301	coding	noncoding	noncoding	noncoding
MSTRG.23980.1		XLOC_033480	259	coding	noncoding	noncoding	noncoding
MSTRG.23983.1		XLOC_033489	310	coding	noncoding	noncoding	noncoding
MSTRG.23998.1		XLOC_033496	258	coding	noncoding	noncoding	noncoding
MSTRG.24062.1		XLOC_033545	282	coding	noncoding	noncoding	noncoding
MSTRG.24157.1	HEATR5B	XLOC_033564	258	coding	noncoding	noncoding	noncoding
MSTRG.24158.1	HEATR5B	XLOC_033565	282	coding	noncoding	noncoding	noncoding
MSTRG.24458.1	LOC105374464	XLOC_033593	310	coding	noncoding	noncoding	noncoding
MSTRG.24552.1	LINC00211	XLOC_033640	315	coding	noncoding	noncoding	noncoding
MSTRG.24180.1		XLOC_033681	235	coding	noncoding	noncoding	noncoding

MSTRG.24205.1		XLOC_033702	215 coding	noncoding	noncoding	noncoding
MSTRG.24236.1	DHX57	XLOC_033707	243 coding	noncoding	noncoding	noncoding
MSTRG.24308.1	CDKL4	XLOC_033736	232 coding	noncoding	noncoding	noncoding
MSTRG.24323.1	MAP4K3	XLOC_033738	242 coding	noncoding	noncoding	noncoding
MSTRG.24335.1	MAP4K3	XLOC_033748	226 coding	noncoding	noncoding	noncoding
MSTRG.24275.1		XLOC_033769	293 coding	coding	noncoding	noncoding
MSTRG.24278.1		XLOC_033771	278 coding	noncoding	noncoding	noncoding
MSTRG.24761.1	SLC8A1-AS1	XLOC_033776	204 coding	noncoding	noncoding	noncoding
MSTRG.24762.1	SLC8A1-AS1	XLOC_033777	217 coding	noncoding	noncoding	noncoding
MSTRG.24360.1		XLOC_033788	319 coding	noncoding	noncoding	noncoding
XR_939995.2	LOC105374497	XLOC_033789	2076 coding	noncoding	noncoding	noncoding
MSTRG.24373.1	LOC105374497	XLOC_033790	225 coding	noncoding	noncoding	noncoding
XR_939997.2	LOC105374506	XLOC_033807	11601 coding	noncoding	coding	noncoding
MSTRG.24398.1		XLOC_033818	311 coding	noncoding	noncoding	noncoding
MSTRG.24408.1		XLOC_033825	234 coding	noncoding	noncoding	noncoding
MSTRG.24656.1	MTA3	XLOC_033845	235 coding	noncoding	noncoding	noncoding
MSTRG.24658.1	MTA3	XLOC_033847	306 coding	noncoding	noncoding	noncoding
MSTRG.24662.1	MTA3	XLOC_033849	220 coding	noncoding	noncoding	noncoding
MSTRG.24863.1	THADA	XLOC_033899	281 coding	noncoding	noncoding	noncoding
MSTRG.24879.1	THADA	XLOC_033915	280 coding	noncoding	noncoding	noncoding
MSTRG.24706.1	PLEKHH2	XLOC_033930	281 coding	noncoding	noncoding	noncoding
MSTRG.24739.1	LRPPRC	XLOC_033946	235 coding	noncoding	noncoding	noncoding
MSTRG.24722.1		XLOC_033950	267 coding	noncoding	noncoding	noncoding
MSTRG.25103.1	SLC3A1	XLOC_033956	227 coding	noncoding	coding	noncoding
XR_002959310.1	SRBD1	XLOC_033981	3655 coding	coding	coding	coding
MSTRG.24895.1		XLOC_034023	225 coding	noncoding	noncoding	noncoding
MSTRG.25031.9	RHOQ	XLOC_034052	9605 coding	noncoding	coding	noncoding
MSTRG.25047.1	STPG4	XLOC_034077	287 coding	noncoding	noncoding	noncoding
MSTRG.25010.1	EPCAM-DT	XLOC_034085	312 coding	noncoding	noncoding	noncoding
MSTRG.25016.1	EPCAM-DT	XLOC_034087	441 coding	noncoding	noncoding	noncoding
MSTRG.25001.1		XLOC_034088	249 coding	noncoding	noncoding	noncoding
MSTRG.25022.1		XLOC_034091	314 coding	noncoding	noncoding	noncoding
MSTRG.25166.1		XLOC_034133	273 coding	noncoding	noncoding	noncoding
MSTRG.25179.8	FOXN2	XLOC_034138	10798 coding	coding	noncoding	noncoding
XM_011532736.2	FSHR	XLOC_034157	12527 coding	coding	coding	coding
MSTRG.25428.1	NRXN1	XLOC_034185	303 coding	noncoding	noncoding	noncoding
MSTRG.25435.1	NRXN1	XLOC_034188	260 coding	noncoding	noncoding	noncoding
MSTRG.25318.1	LOC730100	XLOC_034201	220 coding	noncoding	noncoding	noncoding
MSTRG.25323.1	LOC730100	XLOC_034204	373 coding	noncoding	noncoding	noncoding
MSTRG.25309.1		XLOC_034222	244 coding	noncoding	noncoding	noncoding
MSTRG.25633.1	ASB3	XLOC_034258	221 coding	noncoding	noncoding	noncoding
MSTRG.25547.1	EML6	XLOC_034306	248 coding	noncoding	noncoding	noncoding
NM_001353781.2	CLHC1	XLOC_034336	5630 coding	coding	noncoding	coding

MSTRG.25592.1	CLHC1	XLOC_034339	214 coding	noncoding	noncoding	noncoding
MSTRG.25613.1		XLOC_034360	211 coding	noncoding	noncoding	noncoding
NR_125368.1	LOC100129434	XLOC_034378	2863 coding	coding	coding	noncoding
MSTRG.25722.1		XLOC_034384	252 coding	noncoding	noncoding	noncoding
MSTRG.25728.1		XLOC_034387	263 coding	noncoding	noncoding	noncoding
MSTRG.25725.1		XLOC_034392	240 coding	noncoding	noncoding	noncoding
XR_001739483.1	LOC105377632	XLOC_034394	11548 coding	noncoding	noncoding	noncoding
MSTRG.26004.1	LOC105377632	XLOC_034397	292 coding	noncoding	noncoding	noncoding
MSTRG.26024.1	LOC105377632	XLOC_034403	223 coding	noncoding	noncoding	noncoding
MSTRG.26041.1	LOC105377632	XLOC_034407	306 coding	noncoding	noncoding	noncoding
MSTRG.26116.1	VRK2	XLOC_034416	302 coding	noncoding	noncoding	noncoding
MSTRG.25764.1	LINC01122	XLOC_034431	647 coding	noncoding	noncoding	noncoding
MSTRG.25790.1	LOC105374754	XLOC_034444	259 coding	noncoding	noncoding	noncoding
MSTRG.25776.1		XLOC_034446	251 coding	noncoding	noncoding	noncoding
MSTRG.25811.1		XLOC_034462	319 coding	noncoding	noncoding	noncoding
MSTRG.25818.1		XLOC_034465	263 coding	noncoding	noncoding	noncoding
MSTRG.25847.1		XLOC_034491	225 coding	noncoding	noncoding	noncoding
MSTRG.25854.1		XLOC_034495	253 coding	noncoding	noncoding	noncoding
MSTRG.25859.1		XLOC_034496	273 coding	noncoding	noncoding	noncoding
MSTRG.25873.1		XLOC_034498	235 coding	noncoding	noncoding	noncoding
MSTRG.26170.6	USP34	XLOC_034517	481 coding	noncoding	noncoding	noncoding
MSTRG.25912.1		XLOC_034547	277 coding	noncoding	noncoding	noncoding
MSTRG.25916.1		XLOC_034549	230 coding	noncoding	noncoding	noncoding
MSTRG.25917.1		XLOC_034550	312 coding	noncoding	noncoding	noncoding
MSTRG.25925.1	LOC105374760	XLOC_034554	215 coding	noncoding	noncoding	noncoding
MSTRG.25946.1	LOC105374761	XLOC_034572	273 coding	noncoding	noncoding	noncoding
MSTRG.25982.1		XLOC_034577	273 coding	noncoding	noncoding	noncoding
MSTRG.25972.1		XLOC_034587	298 coding	coding	noncoding	noncoding
MSTRG.25974.1		XLOC_034588	300 coding	noncoding	noncoding	noncoding
MSTRG.26584.1		XLOC_034654	542 coding	noncoding	noncoding	noncoding
MSTRG.26609.26		XLOC_034662	4422 coding	noncoding	coding	noncoding
MSTRG.26307.1		XLOC_034709	276 coding	noncoding	noncoding	noncoding
NM_181784.3	SPRED2	XLOC_034716	4519 coding	coding	coding	coding
XR_940187.3	LOC105374780	XLOC_034718	3637 coding	noncoding	coding	noncoding
MSTRG.26341.1	LOC105374781	XLOC_034719	245 coding	noncoding	noncoding	noncoding
MSTRG.26348.1		XLOC_034723	227 coding	noncoding	noncoding	noncoding
MSTRG.26349.1		XLOC_034724	601 coding	noncoding	noncoding	noncoding
MSTRG.26350.1		XLOC_034725	501 coding	noncoding	noncoding	noncoding
MSTRG.26390.1		XLOC_034737	325 coding	noncoding	noncoding	noncoding
MSTRG.26451.1		XLOC_034754	239 coding	noncoding	noncoding	noncoding
MSTRG.26477.1	LINC01828	XLOC_034763	263 coding	noncoding	noncoding	noncoding
MSTRG.26514.1		XLOC_034779	255 coding	coding	noncoding	noncoding
MSTRG.26543.1		XLOC_034792	248 coding	noncoding	noncoding	noncoding

MSTRG.26773.1	GFPT1	XLOC_034841	321 coding	noncoding	noncoding	noncoding
XM_017003808.2	NFU1	XLOC_034843	1549 coding	coding	coding	coding
MSTRG.26970.1	NFU1	XLOC_034844	265 coding	noncoding	noncoding	noncoding
MSTRG.26978.1	AAK1	XLOC_034849	260 coding	noncoding	noncoding	noncoding
MSTRG.27001.1	AAK1	XLOC_034870	559 coding	noncoding	noncoding	noncoding
NM_001351518.1	TIA1	XLOC_034914	1160 coding	coding	coding	coding
NM_001351519.2	TIA1	XLOC_034914	802 coding	coding	coding	coding
MSTRG.26867.1		XLOC_034921	337 coding	noncoding	noncoding	noncoding
MSTRG.26874.1		XLOC_034922	321 coding	noncoding	noncoding	noncoding
MSTRG.26879.1		XLOC_034924	261 coding	noncoding	noncoding	noncoding
MSTRG.26907.1	TGFA	XLOC_034928	315 coding	noncoding	noncoding	noncoding
MSTRG.26930.1		XLOC_034955	217 coding	coding	noncoding	noncoding
MSTRG.26936.1	OR7E91P	XLOC_034956	269 coding	coding	noncoding	noncoding
MSTRG.26940.1		XLOC_034958	223 coding	noncoding	noncoding	noncoding
MSTRG.27059.1		XLOC_034982	326 coding	noncoding	noncoding	noncoding
MSTRG.27092.1		XLOC_035039	236 coding	noncoding	noncoding	noncoding
NM_001965.4	EGR4	XLOC_035047	2220 coding	coding	coding	coding
MSTRG.27151.1	C2orf78	XLOC_035072	360 coding	noncoding	noncoding	noncoding
NM_001035505.2	BOLA3	XLOC_035076	466 coding	noncoding	noncoding	noncoding
NM_001317112.2	MOB1A	XLOC_035079	4732 coding	coding	noncoding	coding
MSTRG.27326.1		XLOC_035083	240 coding	noncoding	noncoding	noncoding
MSTRG.27271.4	INO80B-WBP1	XLOC_035090	3265 coding	coding	coding	coding
MSTRG.27271.7	INO80B-WBP1	XLOC_035090	948 coding	coding	coding	coding
MSTRG.27370.3	TTC31	XLOC_035094	546 coding	noncoding	noncoding	noncoding
MSTRG.27376.3	PCGF1	XLOC_035097	253 coding	noncoding	noncoding	noncoding
MSTRG.27382.1	LOC102724497	XLOC_035117	262 coding	noncoding	noncoding	noncoding
MSTRG.27385.1	LOC102724482	XLOC_035119	595 coding	noncoding	noncoding	noncoding
MSTRG.27387.1		XLOC_035124	319 coding	noncoding	noncoding	noncoding
MSTRG.27860.1	TACR1	XLOC_035168	639 coding	noncoding	noncoding	noncoding
MSTRG.27440.1		XLOC_035237	269 coding	noncoding	noncoding	noncoding
MSTRG.27450.1		XLOC_035242	204 coding	noncoding	noncoding	noncoding
MSTRG.27460.1		XLOC_035248	261 coding	noncoding	noncoding	noncoding
MSTRG.27536.1	LOC101927967	XLOC_035273	252 coding	noncoding	noncoding	noncoding
MSTRG.27540.1	LOC101927967	XLOC_035276	257 coding	noncoding	noncoding	noncoding
MSTRG.27541.1	LOC101927967	XLOC_035277	385 coding	noncoding	noncoding	noncoding
MSTRG.27547.1	LOC101927967	XLOC_035281	419 coding	noncoding	noncoding	noncoding
MSTRG.27555.1		XLOC_035296	252 coding	coding	noncoding	noncoding
MSTRG.27614.1	CTNNA2	XLOC_035314	268 coding	noncoding	noncoding	noncoding
MSTRG.27628.1	CTNNA2	XLOC_035323	715 coding	noncoding	noncoding	noncoding
MSTRG.27584.1		XLOC_035338	216 coding	coding	noncoding	noncoding
MSTRG.27585.1		XLOC_035339	331 coding	noncoding	noncoding	noncoding
MSTRG.27649.1	LOC102724542	XLOC_035345	242 coding	noncoding	noncoding	noncoding
MSTRG.27597.1		XLOC_035357	272 coding	noncoding	noncoding	noncoding

MSTRG.27667.1		XLOC_035362	316	coding	noncoding	noncoding	noncoding
MSTRG.27670.1		XLOC_035367	310	coding	noncoding	noncoding	noncoding
MSTRG.27680.1		XLOC_035373	228	coding	noncoding	noncoding	noncoding
MSTRG.27696.1		XLOC_035381	327	coding	noncoding	noncoding	noncoding
MSTRG.27714.1		XLOC_035389	232	coding	coding	noncoding	noncoding
MSTRG.27715.1		XLOC_035390	310	coding	noncoding	noncoding	noncoding
MSTRG.27716.1		XLOC_035392	242	coding	noncoding	noncoding	noncoding
MSTRG.27739.1	LOC107985905	XLOC_035400	229	coding	noncoding	noncoding	noncoding
MSTRG.27734.1		XLOC_035401	224	coding	noncoding	noncoding	noncoding
MSTRG.27755.1	LOC105374838	XLOC_035440	317	coding	noncoding	noncoding	noncoding
MSTRG.27759.5	KCMF1	XLOC_035444	1833	coding	coding	noncoding	noncoding
MSTRG.27747.1		XLOC_035447	235	coding	noncoding	noncoding	noncoding
MSTRG.27955.1	TCF7L1	XLOC_035449	263	coding	noncoding	noncoding	noncoding
NM_017750.4	RETSAT	XLOC_035457	3141	coding	coding	coding	coding
MSTRG.27979.3	ELMOD3	XLOC_035460	210	coding	coding	noncoding	noncoding
MSTRG.28011.1		XLOC_035484	250	coding	noncoding	noncoding	noncoding
MSTRG.28037.1	ST3GAL5	XLOC_035489	234	coding	noncoding	noncoding	noncoding
MSTRG.28073.4	PTCD3	XLOC_035503	1308	coding	coding	noncoding	noncoding
MSTRG.28083.1	IMMT	XLOC_035511	252	coding	noncoding	noncoding	noncoding
MSTRG.28056.1	REEP1	XLOC_035515	218	coding	noncoding	noncoding	noncoding
MSTRG.28048.1		XLOC_035516	304	coding	noncoding	noncoding	noncoding
MSTRG.28162.1	RNF103-CHMP3	XLOC_035535	273	coding	noncoding	noncoding	noncoding
MSTRG.28163.1	RNF103-CHMP3	XLOC_035536	260	coding	noncoding	noncoding	noncoding
MSTRG.28113.1	ANAPC1P1	XLOC_035555	325	coding	noncoding	noncoding	noncoding
MSTRG.28221.1		XLOC_035607	576	coding	noncoding	noncoding	noncoding
MSTRG.28249.1	LOC101928347	XLOC_035612	285	coding	noncoding	noncoding	noncoding
MSTRG.28246.1		XLOC_035625	293	coding	noncoding	noncoding	noncoding
NM_001313915.1	EIF2AK3	XLOC_035626	4223	coding	coding	coding	coding
MSTRG.28280.3	ANKRD36BP2	XLOC_035640	3099	coding	coding	noncoding	noncoding
NR_160763.1	LOC107985911	XLOC_035647	4457	coding	noncoding	coding	noncoding
MSTRG.28460.1	IGKV4-1	XLOC_035662	295	coding	noncoding	noncoding	noncoding
MSTRG.28463.1	IGKV4-1	XLOC_035664	366	coding	coding	noncoding	noncoding
MSTRG.28288.1		XLOC_035675	292	coding	coding	noncoding	noncoding
MSTRG.28298.1	LSP1P4	XLOC_035680	438	coding	noncoding	noncoding	noncoding
MSTRG.28361.1		XLOC_035690	255	coding	noncoding	noncoding	noncoding
MSTRG.28369.1		XLOC_035691	239	coding	noncoding	noncoding	noncoding
MSTRG.28393.1	LOC107985915	XLOC_035702	249	coding	noncoding	noncoding	noncoding
MSTRG.28395.1	LOC107985915	XLOC_035703	424	coding	noncoding	noncoding	noncoding
MSTRG.28406.1	LOC100509620	XLOC_035713	316	coding	noncoding	noncoding	noncoding
MSTRG.28408.1	LOC100509620	XLOC_035715	259	coding	noncoding	noncoding	noncoding
MSTRG.28400.1		XLOC_035716	327	coding	noncoding	noncoding	noncoding
MSTRG.28419.1	TEKT4	XLOC_035720	238	coding	coding	coding	noncoding
MSTRG.28484.1	MRPS5	XLOC_035729	253	coding	noncoding	noncoding	noncoding

MSTRG.28501.1		XLOC_035740	234	coding	noncoding	noncoding	noncoding
XR_923077.2	LOC105373490	XLOC_035761	4565	coding	noncoding	noncoding	noncoding
MSTRG.28535.1		XLOC_035766	627	coding	noncoding	noncoding	noncoding
MSTRG.28594.1	ANKRD36C	XLOC_035778	372	coding	noncoding	noncoding	noncoding
MSTRG.28606.1	ANKRD36C	XLOC_035787	340	coding	noncoding	noncoding	noncoding
MSTRG.28545.1		XLOC_035799	285	coding	noncoding	noncoding	noncoding
XM_011511206.2	ASTL	XLOC_035802	3367	coding	coding	coding	coding
MSTRG.28572.1	STARD7-AS1	XLOC_035810	643	coding	coding	noncoding	noncoding
MSTRG.28632.1		XLOC_035821	313	coding	noncoding	noncoding	noncoding
MSTRG.28610.1		XLOC_035831	300	coding	noncoding	noncoding	noncoding
MSTRG.28654.1	FER1L5	XLOC_035840	294	coding	noncoding	noncoding	noncoding
MSTRG.28661.1	LMAN2L	XLOC_035845	271	coding	noncoding	noncoding	noncoding
MSTRG.28685.1	FAM178B	XLOC_035862	421	coding	noncoding	noncoding	noncoding
XM_011510746.2	FAHD2B	XLOC_035866	3176	coding	coding	coding	coding
MSTRG.28775.1	ANKRD36B	XLOC_035887	251	coding	noncoding	noncoding	noncoding
MSTRG.28721.1		XLOC_035890	253	coding	noncoding	noncoding	noncoding
MSTRG.28849.1	TMEM131	XLOC_035940	252	coding	noncoding	noncoding	noncoding
MSTRG.28956.10	INPP4A	XLOC_035955	10187	coding	noncoding	coding	coding
NM_001008215.3	COA5	XLOC_035958	1770	coding	coding	noncoding	noncoding
MSTRG.28905.1	TSGA10	XLOC_036001	260	coding	noncoding	noncoding	noncoding
MSTRG.28930.22	MRPL30	XLOC_036009	1342	coding	coding	noncoding	noncoding
MSTRG.29016.3	EIF5B	XLOC_036032	2538	coding	coding	coding	noncoding
NR_135650.1	REV1	XLOC_036032	5223	coding	coding	coding	coding
MSTRG.28952.1		XLOC_036034	286	coding	noncoding	noncoding	noncoding
MSTRG.28950.1		XLOC_036035	271	coding	noncoding	noncoding	noncoding
MSTRG.29666.1	AFF3	XLOC_036040	246	coding	noncoding	noncoding	noncoding
MSTRG.29684.1	AFF3	XLOC_036055	296	coding	noncoding	noncoding	noncoding
MSTRG.29019.1		XLOC_036075	241	coding	noncoding	noncoding	noncoding
MSTRG.29041.1		XLOC_036090	464	coding	noncoding	noncoding	noncoding
MSTRG.29056.1		XLOC_036093	230	coding	noncoding	noncoding	noncoding
MSTRG.29059.1		XLOC_036097	258	coding	noncoding	noncoding	noncoding
XR_001739612.1	LOC105375310	XLOC_036099	9341	coding	noncoding	noncoding	coding
MSTRG.29175.1	TBC1D8	XLOC_036112	210	coding	noncoding	noncoding	noncoding
MSTRG.29182.1		XLOC_036115	261	coding	noncoding	noncoding	noncoding
XM_011510990.2	RNF149	XLOC_036117	4659	coding	coding	coding	coding
MSTRG.29083.1	CREG2	XLOC_036120	282	coding	noncoding	noncoding	noncoding
MSTRG.29084.1	CREG2	XLOC_036121	292	coding	noncoding	noncoding	noncoding
XM_011511777.2	RFX8	XLOC_036124	2389	coding	noncoding	coding	coding
MSTRG.29089.1		XLOC_036127	318	coding	noncoding	noncoding	noncoding
MSTRG.29091.1		XLOC_036128	289	coding	noncoding	noncoding	noncoding
MSTRG.29092.1		XLOC_036129	235	coding	noncoding	noncoding	noncoding
MSTRG.29118.14	MAP4K4	XLOC_036131	4441	coding	coding	coding	coding
MSTRG.29121.1	MAP4K4	XLOC_036132	459	coding	noncoding	noncoding	noncoding

MSTRG.29114.1		XLOC_036149	333	coding	noncoding	noncoding	noncoding
MSTRG.29247.1	SLC9A2	XLOC_036187	280	coding	noncoding	noncoding	noncoding
MSTRG.29329.1		XLOC_036226	344	coding	noncoding	noncoding	noncoding
MSTRG.29332.1		XLOC_036227	302	coding	noncoding	noncoding	noncoding
MSTRG.29371.1		XLOC_036253	217	coding	noncoding	noncoding	noncoding
MSTRG.29383.1	PANTR1	XLOC_036258	361	coding	noncoding	noncoding	noncoding
MSTRG.29426.1		XLOC_036272	280	coding	noncoding	noncoding	noncoding
MSTRG.29428.1		XLOC_036275	287	coding	noncoding	noncoding	noncoding
NM_001318894.1	FHL2	XLOC_036284	1767	coding	coding	coding	coding
MSTRG.29487.1	FHL2	XLOC_036285	299	coding	noncoding	noncoding	noncoding
XM_011511904.3	UXS1	XLOC_036313	2166	coding	coding	coding	coding
NM_025076.4	UXS1	XLOC_036313	2084	coding	coding	coding	coding
MSTRG.29511.1		XLOC_036324	250	coding	noncoding	noncoding	noncoding
MSTRG.29513.1		XLOC_036325	603	coding	noncoding	noncoding	noncoding
MSTRG.29518.1		XLOC_036330	283	coding	noncoding	noncoding	noncoding
XM_017004739.2	RGPD3	XLOC_036331	5892	coding	coding	coding	coding
MSTRG.29545.5	RGPD3	XLOC_036331	455	coding	noncoding	noncoding	noncoding
MSTRG.29557.1		XLOC_036342	445	coding	noncoding	noncoding	noncoding
MSTRG.29556.1		XLOC_036343	214	coding	noncoding	noncoding	noncoding
MSTRG.29607.1	LINC01789	XLOC_036346	241	coding	noncoding	noncoding	noncoding
MSTRG.29657.1		XLOC_036376	215	coding	noncoding	noncoding	noncoding
MSTRG.29747.1		XLOC_036389	264	coding	noncoding	noncoding	noncoding
MSTRG.29925.1	SH3RF3	XLOC_036446	221	coding	noncoding	noncoding	noncoding
MSTRG.29773.1		XLOC_036482	250	coding	noncoding	noncoding	noncoding
MSTRG.29774.1		XLOC_036483	259	coding	noncoding	noncoding	noncoding
MSTRG.29775.1	LOC100506563	XLOC_036484	349	coding	noncoding	noncoding	noncoding
NM_000272.4	NPHP1	XLOC_036505	3727	coding	coding	coding	coding
MSTRG.29811.1	NPHP1	XLOC_036506	458	coding	noncoding	noncoding	noncoding
MSTRG.29817.1	NPHP1	XLOC_036508	424	coding	noncoding	noncoding	noncoding
MSTRG.29820.1	NPHP1	XLOC_036509	266	coding	noncoding	noncoding	noncoding
MSTRG.29826.1		XLOC_036513	261	coding	coding	noncoding	noncoding
MSTRG.29843.1	LOC102724875	XLOC_036518	277	coding	noncoding	noncoding	noncoding
MSTRG.29969.1	ACOXL	XLOC_036529	226	coding	noncoding	noncoding	noncoding
MSTRG.29986.1	ACOXL	XLOC_036542	294	coding	noncoding	noncoding	noncoding
MSTRG.30043.1	LOC101927283	XLOC_036554	308	coding	noncoding	noncoding	noncoding
MSTRG.30001.1		XLOC_036590	447	coding	noncoding	noncoding	noncoding
XM_017004715.1	ANAPC1	XLOC_036592	5185	coding	coding	coding	coding
MSTRG.30029.1	ANAPC1	XLOC_036597	226	coding	noncoding	noncoding	noncoding
MSTRG.30155.1		XLOC_036643	276	coding	noncoding	noncoding	noncoding
MSTRG.30142.1		XLOC_036652	243	coding	noncoding	noncoding	noncoding
MSTRG.30143.1		XLOC_036653	287	coding	noncoding	noncoding	noncoding
MSTRG.30192.1		XLOC_036661	231	coding	noncoding	noncoding	noncoding
MSTRG.30209.1	IL36B	XLOC_036676	289	coding	noncoding	noncoding	noncoding

MSTRG.30210.1		XLOC_036679	224 coding	noncoding	noncoding	noncoding
MSTRG.30302.1		XLOC_036733	261 coding	noncoding	noncoding	noncoding
MSTRG.30392.1	ACTR3-AS1	XLOC_036749	225 coding	noncoding	noncoding	noncoding
MSTRG.30470.1	DPP10	XLOC_036776	287 coding	noncoding	noncoding	noncoding
MSTRG.30471.1	DPP10	XLOC_036777	289 coding	noncoding	noncoding	noncoding
MSTRG.30474.1	DPP10	XLOC_036780	242 coding	noncoding	noncoding	noncoding
MSTRG.30478.1	DPP10	XLOC_036782	266 coding	noncoding	noncoding	noncoding
MSTRG.30511.1	DPP10	XLOC_036798	229 coding	noncoding	noncoding	noncoding
MSTRG.30354.1		XLOC_036803	215 coding	coding	noncoding	noncoding
MSTRG.30376.1		XLOC_036814	486 coding	noncoding	noncoding	noncoding
MSTRG.30445.1		XLOC_036821	249 coding	noncoding	noncoding	noncoding
NM_019044.5	CCDC93	XLOC_036831	6833 coding	coding	coding	coding
MSTRG.30587.1		XLOC_036864	293 coding	noncoding	noncoding	noncoding
MSTRG.30607.1		XLOC_036876	259 coding	noncoding	noncoding	noncoding
MSTRG.30616.1		XLOC_036885	272 coding	noncoding	noncoding	noncoding
MSTRG.30655.1	C2orf76	XLOC_036899	396 coding	noncoding	noncoding	noncoding
MSTRG.30649.1		XLOC_036915	314 coding	noncoding	noncoding	noncoding
MSTRG.30693.14	LOC101927764	XLOC_036926	5190 coding	coding	coding	coding
MSTRG.30804.1		XLOC_036942	236 coding	noncoding	noncoding	noncoding
MSTRG.30806.1		XLOC_036944	230 coding	coding	noncoding	noncoding
MSTRG.30723.1		XLOC_036957	205 coding	noncoding	noncoding	noncoding
MSTRG.30769.1		XLOC_036982	279 coding	noncoding	noncoding	noncoding
XM_017003679.2	CLASP1	XLOC_036983	8798 coding	coding	coding	coding
MSTRG.30812.1		XLOC_037029	255 coding	noncoding	noncoding	noncoding
MSTRG.30816.1	LOC105373590	XLOC_037032	221 coding	noncoding	noncoding	noncoding
MSTRG.30836.1	LOC105373592	XLOC_037036	211 coding	coding	noncoding	noncoding
MSTRG.30841.1	LOC105373592	XLOC_037039	328 coding	noncoding	noncoding	noncoding
MSTRG.30875.1		XLOC_037067	404 coding	noncoding	noncoding	noncoding
MSTRG.30884.1		XLOC_037075	276 coding	noncoding	noncoding	noncoding
MSTRG.30890.1		XLOC_037078	313 coding	noncoding	noncoding	noncoding
MSTRG.30896.1		XLOC_037081	244 coding	noncoding	noncoding	noncoding
MSTRG.30911.1		XLOC_037088	296 coding	noncoding	noncoding	noncoding
MSTRG.30961.1	CNTNAP5	XLOC_037100	250 coding	noncoding	noncoding	noncoding
MSTRG.30967.1	CNTNAP5	XLOC_037103	211 coding	noncoding	noncoding	noncoding
MSTRG.30978.1	CNTNAP5	XLOC_037110	310 coding	noncoding	noncoding	noncoding
MSTRG.30938.1		XLOC_037123	276 coding	noncoding	noncoding	noncoding
MSTRG.30939.1		XLOC_037124	214 coding	noncoding	noncoding	noncoding
NM_001367502.1	CYP27C1	XLOC_037158	5082 coding	coding	coding	coding
MSTRG.31176.1	POLR2D	XLOC_037207	365 coding	noncoding	noncoding	noncoding
MSTRG.31180.1		XLOC_037212	251 coding	noncoding	noncoding	noncoding
MSTRG.31178.1		XLOC_037213	242 coding	noncoding	noncoding	noncoding
MSTRG.31182.1		XLOC_037215	242 coding	noncoding	noncoding	noncoding
MSTRG.31203.1	SAP130	XLOC_037219	294 coding	noncoding	noncoding	noncoding

MSTRG.31205.1	SAP130	XLOC_037221	252 coding	noncoding	noncoding	noncoding
MSTRG.31206.1	SAP130	XLOC_037222	314 coding	noncoding	noncoding	noncoding
MSTRG.31254.1	LOC105373611	XLOC_037245	283 coding	noncoding	noncoding	noncoding
MSTRG.31260.1		XLOC_037251	308 coding	noncoding	noncoding	noncoding
MSTRG.31315.1		XLOC_037265	300 coding	coding	noncoding	noncoding
MSTRG.31321.1		XLOC_037281	305 coding	noncoding	coding	noncoding
NR_026758.2	FAR2P1	XLOC_037284	4526 coding	coding	coding	noncoding
MSTRG.31338.1		XLOC_037295	349 coding	noncoding	noncoding	noncoding
MSTRG.31343.1		XLOC_037298	322 coding	coding	noncoding	noncoding
NR_046258.1	FAR2P2	XLOC_037313	2858 coding	noncoding	noncoding	noncoding
MSTRG.31455.1	LOC150776	XLOC_037350	443 coding	noncoding	noncoding	noncoding
MSTRG.31460.1	LOC105373622	XLOC_037368	246 coding	noncoding	noncoding	noncoding
MSTRG.31501.1	GPR39	XLOC_037372	324 coding	coding	noncoding	noncoding
MSTRG.31506.1	GPR39	XLOC_037374	287 coding	noncoding	noncoding	noncoding
XM_011511102.2	NCKAP5	XLOC_037377	11571 coding	coding	coding	coding
MSTRG.31521.1	NCKAP5	XLOC_037382	371 coding	noncoding	noncoding	noncoding
MSTRG.31523.1	NCKAP5	XLOC_037384	360 coding	coding	noncoding	noncoding
MSTRG.31555.1	NCKAP5	XLOC_037396	248 coding	noncoding	noncoding	noncoding
MSTRG.31558.1	NCKAP5	XLOC_037398	262 coding	noncoding	noncoding	noncoding
MSTRG.31577.1	NCKAP5	XLOC_037405	264 coding	noncoding	noncoding	noncoding
MSTRG.31473.1		XLOC_037409	259 coding	noncoding	noncoding	noncoding
MSTRG.31493.1		XLOC_037418	471 coding	noncoding	noncoding	noncoding
NM_030923.5	TMEM163	XLOC_037419	1892 coding	noncoding	coding	coding
MSTRG.31938.1	ZRANB3	XLOC_037443	562 coding	noncoding	noncoding	noncoding
MSTRG.31939.1	ZRANB3	XLOC_037444	461 coding	noncoding	noncoding	noncoding
MSTRG.31769.1	THSD7B	XLOC_037519	248 coding	noncoding	noncoding	noncoding
MSTRG.32154.1		XLOC_037537	266 coding	coding	noncoding	noncoding
MSTRG.31990.1		XLOC_037558	315 coding	noncoding	noncoding	noncoding
MSTRG.31981.1		XLOC_037562	272 coding	noncoding	noncoding	noncoding
MSTRG.31999.1	LOC105373643	XLOC_037572	320 coding	noncoding	noncoding	noncoding
MSTRG.32018.1		XLOC_037581	226 coding	noncoding	noncoding	noncoding
MSTRG.32027.1		XLOC_037586	299 coding	noncoding	noncoding	noncoding
MSTRG.32028.1		XLOC_037587	239 coding	noncoding	noncoding	noncoding
MSTRG.32086.1	LRP1B	XLOC_037591	216 coding	noncoding	noncoding	noncoding
MSTRG.32116.1	LOC105373649	XLOC_037606	366 coding	noncoding	noncoding	noncoding
MSTRG.32147.1	LRP1B	XLOC_037621	283 coding	noncoding	noncoding	noncoding
MSTRG.32152.1	LRP1B	XLOC_037625	233 coding	noncoding	noncoding	noncoding
MSTRG.32051.1		XLOC_037638	279 coding	noncoding	noncoding	noncoding
MSTRG.33081.27	ARHGAP15	XLOC_037652	2058 coding	coding	noncoding	noncoding
MSTRG.33081.31	ARHGAP15	XLOC_037652	11119 coding	noncoding	noncoding	noncoding
MSTRG.33081.36	ARHGAP15	XLOC_037652	6556 coding	noncoding	noncoding	noncoding
MSTRG.33081.35	ARHGAP15	XLOC_037652	30252 coding	noncoding	coding	noncoding
MSTRG.33081.40	ARHGAP15	XLOC_037652	12382 coding	coding	coding	noncoding

MSTRG.32323.1	TEX41	XLOC_037750	522	coding	noncoding	noncoding	noncoding
MSTRG.32327.1	TEX41	XLOC_037751	291	coding	noncoding	noncoding	noncoding
MSTRG.32345.1		XLOC_037766	257	coding	noncoding	noncoding	noncoding
MSTRG.32351.1		XLOC_037770	272	coding	noncoding	noncoding	noncoding
MSTRG.32355.1	LOC105373667	XLOC_037773	302	coding	coding	noncoding	noncoding
MSTRG.32364.1	LOC105373667	XLOC_037779	309	coding	noncoding	noncoding	noncoding
MSTRG.32374.1		XLOC_037781	313	coding	noncoding	noncoding	noncoding
MSTRG.32390.1		XLOC_037787	240	coding	noncoding	noncoding	noncoding
MSTRG.32423.1		XLOC_037802	323	coding	noncoding	noncoding	noncoding
MSTRG.32428.1		XLOC_037804	391	coding	noncoding	noncoding	noncoding
MSTRG.32464.1		XLOC_037827	221	coding	noncoding	noncoding	noncoding
MSTRG.32562.1	LYPD6B	XLOC_037856	216	coding	noncoding	noncoding	noncoding
MSTRG.32537.1		XLOC_037872	289	coding	noncoding	noncoding	noncoding
MSTRG.32607.1	LOC101929231	XLOC_037887	428	coding	noncoding	noncoding	noncoding
MSTRG.32636.1		XLOC_037921	255	coding	noncoding	noncoding	noncoding
MSTRG.32649.1		XLOC_037934	313	coding	noncoding	noncoding	noncoding
MSTRG.33470.6	RIF1	XLOC_037952	7507	coding	coding	coding	coding
XR_001739736.2	LOC101929356	XLOC_037964	7055	coding	noncoding	noncoding	noncoding
MSTRG.32776.1	ARL5A	XLOC_037969	251	coding	noncoding	noncoding	noncoding
MSTRG.32777.1		XLOC_038003	285	coding	noncoding	noncoding	noncoding
MSTRG.32783.1		XLOC_038007	304	coding	noncoding	noncoding	noncoding
MSTRG.33455.1	PRPF40A	XLOC_038023	202	coding	noncoding	noncoding	noncoding
MSTRG.32807.1		XLOC_038046	330	coding	noncoding	noncoding	noncoding
MSTRG.32825.1		XLOC_038057	314	coding	noncoding	noncoding	noncoding
MSTRG.32829.1		XLOC_038060	309	coding	coding	noncoding	noncoding
MSTRG.32832.1		XLOC_038061	303	coding	coding	noncoding	noncoding
MSTRG.32876.1	GALNT13	XLOC_038074	341	coding	coding	noncoding	noncoding
MSTRG.32891.1		XLOC_038091	226	coding	noncoding	noncoding	noncoding
MSTRG.32889.1		XLOC_038092	232	coding	noncoding	noncoding	noncoding
MSTRG.32916.1	LOC105373703	XLOC_038102	275	coding	noncoding	noncoding	noncoding
MSTRG.32949.1	LINC01876	XLOC_038107	290	coding	noncoding	noncoding	noncoding
MSTRG.32951.1	LINC01876	XLOC_038108	276	coding	coding	noncoding	noncoding
MSTRG.33608.1	ACVR1	XLOC_038197	248	coding	noncoding	noncoding	noncoding
MSTRG.33623.1	LOC105373714	XLOC_038210	300	coding	noncoding	noncoding	noncoding
MSTRG.33581.1		XLOC_038213	274	coding	noncoding	noncoding	noncoding
MSTRG.33586.1	UPP2	XLOC_038215	228	coding	noncoding	noncoding	noncoding
MSTRG.33589.1	UPP2	XLOC_038217	201	coding	noncoding	noncoding	noncoding
MSTRG.33630.1		XLOC_038230	238	coding	coding	noncoding	noncoding
MSTRG.33645.1	TANC1	XLOC_038238	285	coding	noncoding	noncoding	noncoding
MSTRG.33740.1		XLOC_038325	225	coding	noncoding	noncoding	noncoding
MSTRG.33767.1		XLOC_038348	322	coding	noncoding	noncoding	noncoding
XM_011510797.3	FAP	XLOC_038382	6007	coding	coding	coding	coding
MSTRG.34079.1	KCNH7	XLOC_038404	251	coding	noncoding	noncoding	noncoding

MSTRG.34084.1	KCNH7	XLOC_038406	306 coding	noncoding	noncoding	noncoding
MSTRG.33965.1		XLOC_038418	260 coding	noncoding	noncoding	noncoding
MSTRG.33969.1		XLOC_038421	448 coding	noncoding	noncoding	noncoding
XM_017004419.1	FIGN	XLOC_038422	19473 coding	coding	coding	coding
MSTRG.34000.1		XLOC_038434	582 coding	coding	noncoding	noncoding
NM_004490.3	GRB14	XLOC_038436	2415 coding	coding	coding	coding
MSTRG.34025.1	SLC38A11	XLOC_038452	247 coding	noncoding	noncoding	noncoding
MSTRG.34154.1	CSRNP3	XLOC_038469	281 coding	noncoding	noncoding	noncoding
MSTRG.34211.1	LOC102724058	XLOC_038491	292 coding	noncoding	noncoding	noncoding
MSTRG.34208.1	XIRP2	XLOC_038514	243 coding	coding	noncoding	noncoding
MSTRG.34246.1	B3GALT1	XLOC_038519	265 coding	noncoding	noncoding	noncoding
MSTRG.34299.1		XLOC_038550	286 coding	noncoding	noncoding	noncoding
MSTRG.34335.1	LRP2	XLOC_038575	234 coding	noncoding	noncoding	noncoding
MSTRG.34333.1		XLOC_038580	246 coding	noncoding	noncoding	noncoding
MSTRG.34352.1	FASTKD1	XLOC_038586	282 coding	noncoding	noncoding	noncoding
MSTRG.34390.1		XLOC_038611	230 coding	noncoding	noncoding	noncoding
MSTRG.34391.1		XLOC_038612	258 coding	coding	noncoding	noncoding
MSTRG.34535.1	MYO3B	XLOC_038622	788 coding	coding	coding	noncoding
MSTRG.34544.1	MYO3B	XLOC_038627	232 coding	noncoding	noncoding	noncoding
MSTRG.34505.1		XLOC_038646	642 coding	noncoding	noncoding	noncoding
MSTRG.34695.1	SLC25A12	XLOC_038696	299 coding	noncoding	noncoding	noncoding
MSTRG.34675.1	LOC107985960	XLOC_038727	244 coding	noncoding	noncoding	noncoding
MSTRG.34735.3	MAP3K20	XLOC_038751	429 coding	noncoding	noncoding	noncoding
MSTRG.34705.1		XLOC_038753	265 coding	noncoding	noncoding	noncoding
MSTRG.34714.1		XLOC_038758	283 coding	noncoding	noncoding	noncoding
MSTRG.34722.1		XLOC_038761	264 coding	noncoding	noncoding	noncoding
MSTRG.34763.1		XLOC_038790	262 coding	noncoding	noncoding	noncoding
MSTRG.34764.1		XLOC_038791	284 coding	noncoding	noncoding	noncoding
MSTRG.34858.1	CIR1	XLOC_038793	239 coding	noncoding	noncoding	noncoding
MSTRG.35010.1		XLOC_038800	640 coding	noncoding	noncoding	noncoding
MSTRG.35021.1	WIPF1	XLOC_038808	237 coding	noncoding	noncoding	noncoding
MSTRG.34883.1		XLOC_038856	282 coding	noncoding	noncoding	noncoding
MSTRG.34906.1	HOXD12	XLOC_038873	352 coding	coding	coding	noncoding
NR_110458.1	HAGLR	XLOC_038881	4086 coding	noncoding	coding	noncoding
MSTRG.34919.1		XLOC_038883	279 coding	noncoding	noncoding	noncoding
MSTRG.34932.1		XLOC_038889	259 coding	noncoding	noncoding	noncoding
MSTRG.34934.1		XLOC_038890	221 coding	noncoding	noncoding	noncoding
MSTRG.34965.1		XLOC_038891	317 coding	coding	noncoding	noncoding
MSTRG.34977.1		XLOC_038899	232 coding	noncoding	noncoding	noncoding
MSTRG.35000.1		XLOC_038909	298 coding	noncoding	noncoding	noncoding
MSTRG.35100.1		XLOC_038913	300 coding	coding	noncoding	noncoding
MSTRG.35102.1	HNRNPA3	XLOC_038915	7061 coding	coding	noncoding	noncoding
MSTRG.35156.1		XLOC_038938	300 coding	noncoding	noncoding	noncoding

NM_016953.4	PDE11A	XLOC_038942	9305 coding	coding	coding	coding
MSTRG.35054.1	PDE11A	XLOC_038951	249 coding	noncoding	noncoding	noncoding
MSTRG.35071.1	OSBPL6	XLOC_038957	236 coding	noncoding	noncoding	noncoding
MSTRG.35065.1		XLOC_038963	326 coding	noncoding	noncoding	noncoding
MSTRG.35898.7	TTN	XLOC_038971	3743 coding	coding	coding	coding
MSTRG.35898.28	TTN	XLOC_038971	15143 coding	coding	noncoding	coding
MSTRG.35928.1	CCDC141	XLOC_038989	336 coding	noncoding	noncoding	noncoding
MSTRG.35234.1	SESTD1	XLOC_038991	245 coding	coding	noncoding	noncoding
MSTRG.35168.1		XLOC_039019	229 coding	noncoding	noncoding	noncoding
MSTRG.35272.1		XLOC_039023	258 coding	noncoding	noncoding	noncoding
MSTRG.35277.3	CWC22	XLOC_039025	229 coding	noncoding	noncoding	noncoding
MSTRG.35197.1		XLOC_039034	294 coding	noncoding	noncoding	noncoding
MSTRG.35228.1		XLOC_039051	266 coding	noncoding	noncoding	noncoding
MSTRG.35261.1	SCHLAP1	XLOC_039058	252 coding	noncoding	noncoding	noncoding
MSTRG.35338.1	PPP1R1C	XLOC_039089	303 coding	noncoding	noncoding	noncoding
MSTRG.35369.1	PDE1A	XLOC_039101	269 coding	noncoding	noncoding	noncoding
MSTRG.35378.5	DNAJC10	XLOC_039104	1320 coding	coding	noncoding	noncoding
NM_205842.3	NCKAP1	XLOC_039115	20350 coding	coding	coding	coding
MSTRG.35499.1		XLOC_039142	225 coding	noncoding	noncoding	noncoding
MSTRG.35439.1		XLOC_039174	293 coding	noncoding	noncoding	noncoding
MSTRG.36065.1	LOC107985783	XLOC_039342	313 coding	coding	noncoding	noncoding
MSTRG.36134.2	ZC3H15	XLOC_039378	2027 coding	coding	noncoding	noncoding
MSTRG.35724.1		XLOC_039381	267 coding	noncoding	noncoding	noncoding
MSTRG.35730.1		XLOC_039384	292 coding	noncoding	noncoding	noncoding
MSTRG.35787.1	LOC105373786	XLOC_039398	298 coding	noncoding	noncoding	noncoding
XM_024453059.1	TFPI	XLOC_039403	2079 coding	coding	coding	coding
MSTRG.35846.1	COL5A2	XLOC_039441	353 coding	noncoding	noncoding	noncoding
MSTRG.35875.1	LOC101927252	XLOC_039448	259 coding	noncoding	noncoding	noncoding
MSTRG.35888.1	SLC40A1	XLOC_039453	263 coding	noncoding	noncoding	noncoding
NM_001128150.1	ORMDL1	XLOC_039463	2072 coding	coding	noncoding	coding
MSTRG.35934.1		XLOC_039494	275 coding	noncoding	coding	noncoding
MSTRG.35940.1		XLOC_039523	287 coding	coding	coding	noncoding
XM_017004783.2	STAT1	XLOC_039530	4360 coding	coding	coding	coding
MSTRG.36288.1	STAT4	XLOC_039555	729 coding	noncoding	noncoding	noncoding
MSTRG.36330.1		XLOC_039618	290 coding	noncoding	coding	noncoding
MSTRG.36403.1		XLOC_039628	322 coding	noncoding	noncoding	noncoding
MSTRG.36387.1		XLOC_039633	286 coding	noncoding	noncoding	noncoding
MSTRG.36435.1		XLOC_039656	287 coding	noncoding	noncoding	noncoding
MSTRG.36436.1		XLOC_039657	211 coding	noncoding	noncoding	noncoding
MSTRG.36445.1	LINC01821	XLOC_039661	265 coding	noncoding	noncoding	noncoding
MSTRG.36451.1		XLOC_039665	219 coding	noncoding	noncoding	noncoding
MSTRG.36484.1	LOC105376755	XLOC_039668	261 coding	noncoding	noncoding	noncoding
MSTRG.36602.1		XLOC_039728	213 coding	noncoding	noncoding	noncoding

MSTRG.36688.1	PGAP1	XLOC_039732	280 coding	noncoding	noncoding	noncoding
XM_011512148.2	ANKRD44	XLOC_039737	9307 coding	coding	coding	coding
MSTRG.36715.1		XLOC_039765	292 coding	coding	noncoding	noncoding
MSTRG.36752.1		XLOC_039791	261 coding	noncoding	noncoding	noncoding
MSTRG.36756.1		XLOC_039793	419 coding	noncoding	noncoding	noncoding
MSTRG.36797.1		XLOC_039821	222 coding	noncoding	noncoding	noncoding
XR_002959289.1	FTCDNL1	XLOC_039823	6811 coding	coding	noncoding	noncoding
XM_024452854.1	FTCDNL1	XLOC_039823	1631 coding	coding	noncoding	noncoding
MSTRG.36864.1		XLOC_039848	323 coding	noncoding	noncoding	noncoding
MSTRG.36979.1		XLOC_039870	233 coding	coding	noncoding	noncoding
NM_001162407.1	CLK1	XLOC_039871	2213 coding	coding	coding	coding
MSTRG.37172.17	CASP8	XLOC_039912	2020 coding	coding	coding	coding
MSTRG.37172.16	CASP8	XLOC_039912	2065 coding	coding	coding	coding
XM_017003362.2	FLACC1	XLOC_039912	1527 coding	noncoding	coding	coding
MSTRG.37005.1	C2CD6	XLOC_039922	268 coding	noncoding	noncoding	noncoding
MSTRG.37067.1	ALS2	XLOC_039928	289 coding	noncoding	noncoding	noncoding
MSTRG.37074.1	ALS2	XLOC_039929	273 coding	noncoding	noncoding	noncoding
MSTRG.37045.1	KIAA2012	XLOC_039943	253 coding	noncoding	noncoding	noncoding
MSTRG.37092.1		XLOC_039957	369 coding	noncoding	noncoding	noncoding
MSTRG.37100.1		XLOC_039961	255 coding	noncoding	noncoding	noncoding
MSTRG.37102.1		XLOC_039962	257 coding	noncoding	noncoding	noncoding
MSTRG.37312.1	CYP20A1	XLOC_040005	900 coding	noncoding	noncoding	noncoding
MSTRG.37319.1		XLOC_040011	239 coding	noncoding	noncoding	noncoding
MSTRG.37272.1		XLOC_040018	223 coding	noncoding	noncoding	noncoding
MSTRG.37343.1		XLOC_040050	283 coding	noncoding	noncoding	noncoding
MSTRG.37441.1	PARD3B	XLOC_040074	253 coding	noncoding	noncoding	noncoding
MSTRG.37455.1	PARD3B	XLOC_040080	318 coding	coding	noncoding	noncoding
MSTRG.37457.1	PARD3B	XLOC_040082	243 coding	coding	noncoding	noncoding
MSTRG.37358.1		XLOC_040097	283 coding	noncoding	noncoding	noncoding
MSTRG.37371.1		XLOC_040112	305 coding	noncoding	noncoding	noncoding
MSTRG.37483.1		XLOC_040125	252 coding	noncoding	noncoding	noncoding
MSTRG.37484.1		XLOC_040126	371 coding	noncoding	noncoding	noncoding
NM_001270943.2	KLF7	XLOC_040163	7990 coding	coding	coding	coding
MSTRG.37913.4	CREB1	XLOC_040211	10281 coding	coding	coding	noncoding
MSTRG.37566.1	LOC100507443	XLOC_040233	279 coding	noncoding	noncoding	noncoding
MSTRG.37678.1		XLOC_040257	269 coding	noncoding	noncoding	noncoding
MSTRG.37584.1		XLOC_040272	236 coding	noncoding	noncoding	noncoding
MSTRG.37633.1	UNC80	XLOC_040293	301 coding	noncoding	noncoding	noncoding
MSTRG.37636.1	UNC80	XLOC_040295	274 coding	noncoding	noncoding	noncoding
MSTRG.37819.9	KANSL1L	XLOC_040296	5849 coding	coding	noncoding	noncoding
XM_005246517.4	ACADL	XLOC_040299	2944 coding	coding	coding	coding
MSTRG.37758.1		XLOC_040332	231 coding	noncoding	noncoding	noncoding
MSTRG.37783.1	ERBB4	XLOC_040336	323 coding	noncoding	noncoding	noncoding

MSTRG.37788.1	ERBB4	XLOC_040340	314	coding	noncoding	coding	noncoding
MSTRG.37800.1	ERBB4	XLOC_040344	505	coding	noncoding	noncoding	noncoding
MSTRG.37767.1		XLOC_040357	223	coding	noncoding	noncoding	noncoding
MSTRG.38211.1		XLOC_040359	764	coding	noncoding	noncoding	noncoding
XM_005246384.5	IKZF2	XLOC_040377	9528	coding	coding	coding	coding
NR_146974.1	LINC01953	XLOC_040409	694	coding	noncoding	noncoding	noncoding
MSTRG.37999.1	SPAG16	XLOC_040427	256	coding	coding	noncoding	noncoding
MSTRG.38004.1	SPAG16	XLOC_040429	302	coding	noncoding	noncoding	noncoding
MSTRG.38006.1	SPAG16	XLOC_040431	216	coding	noncoding	noncoding	noncoding
MSTRG.38014.1	SPAG16	XLOC_040435	255	coding	noncoding	coding	noncoding
MSTRG.37971.1		XLOC_040448	247	coding	noncoding	noncoding	noncoding
MSTRG.38090.1	ABCA12	XLOC_040475	296	coding	noncoding	noncoding	noncoding
MSTRG.38153.1	FN1	XLOC_040485	439	coding	noncoding	noncoding	noncoding
MSTRG.38165.1	FN1	XLOC_040488	223	coding	noncoding	noncoding	noncoding
MSTRG.38108.1		XLOC_040512	239	coding	noncoding	noncoding	noncoding
MSTRG.38113.1		XLOC_040514	329	coding	noncoding	noncoding	noncoding
XM_024452999.1	PECR	XLOC_040520	4669	coding	coding	coding	coding
MSTRG.38367.1		XLOC_040538	240	coding	noncoding	noncoding	noncoding
MSTRG.38336.1		XLOC_040552	263	coding	noncoding	noncoding	noncoding
MSTRG.38412.1	TNS1	XLOC_040589	337	coding	noncoding	noncoding	noncoding
MSTRG.38595.1	PNKD	XLOC_040624	276	coding	noncoding	noncoding	noncoding
MSTRG.38617.3	CTDSP1	XLOC_040636	2536	coding	coding	coding	coding
MSTRG.38570.1	USP37	XLOC_040642	294	coding	noncoding	noncoding	noncoding
MSTRG.38572.1	USP37	XLOC_040644	237	coding	noncoding	noncoding	noncoding
MSTRG.38563.1		XLOC_040647	228	coding	noncoding	noncoding	noncoding
MSTRG.38661.1	ZNF142	XLOC_040652	243	coding	noncoding	noncoding	noncoding
MSTRG.38667.1	RNF25	XLOC_040656	279	coding	noncoding	noncoding	noncoding
MSTRG.38626.6	TTLL4	XLOC_040660	1104	coding	noncoding	noncoding	coding
MSTRG.38640.1		XLOC_040671	257	coding	coding	noncoding	noncoding
MSTRG.38646.1	LINC01494	XLOC_040678	259	coding	noncoding	noncoding	noncoding
MSTRG.38690.1	RETREG2	XLOC_040694	4544	coding	noncoding	coding	noncoding
MSTRG.38676.1		XLOC_040696	241	coding	noncoding	noncoding	noncoding
MSTRG.38696.1	LOC105373883	XLOC_040710	304	coding	noncoding	noncoding	noncoding
MSTRG.38733.1	SPEG	XLOC_040714	240	coding	noncoding	noncoding	noncoding
XM_017003696.2	OBSL1	XLOC_040720	6582	coding	coding	coding	coding
MSTRG.38708.1		XLOC_040722	250	coding	noncoding	noncoding	noncoding
MSTRG.38765.1	LOC105373893	XLOC_040748	217	coding	coding	noncoding	noncoding
MSTRG.38767.1	LOC105373893	XLOC_040749	264	coding	noncoding	noncoding	noncoding
MSTRG.38828.1	LOC107985988	XLOC_040761	278	coding	noncoding	noncoding	noncoding
MSTRG.38876.1	EPHA4	XLOC_040778	252	coding	noncoding	noncoding	noncoding
MSTRG.38879.1	EPHA4	XLOC_040781	294	coding	noncoding	noncoding	noncoding
MSTRG.38866.1	PAX3	XLOC_040796	320	coding	noncoding	noncoding	noncoding
MSTRG.38867.1	PAX3	XLOC_040797	286	coding	noncoding	noncoding	noncoding

MSTRG.38908.1	LOC105373903	XLOC_040823	237	coding	noncoding	noncoding	noncoding
MSTRG.38962.3	ACSL3	XLOC_040825	2798	coding	coding	coding	noncoding
MSTRG.38934.1		XLOC_040837	229	coding	noncoding	noncoding	noncoding
MSTRG.39029.1		XLOC_040871	430	coding	noncoding	noncoding	noncoding
MSTRG.39036.1		XLOC_040872	291	coding	noncoding	noncoding	noncoding
MSTRG.39059.1	CUL3	XLOC_040880	218	coding	coding	noncoding	noncoding
MSTRG.39040.1		XLOC_040908	307	coding	noncoding	noncoding	noncoding
MSTRG.39046.1		XLOC_040913	258	coding	noncoding	noncoding	noncoding
NM_001363762.1	DOCK10	XLOC_040914	7290	coding	coding	coding	coding
MSTRG.39128.1	NYAP2	XLOC_040991	276	coding	noncoding	noncoding	noncoding
MSTRG.39109.1		XLOC_040996	271	coding	noncoding	noncoding	noncoding
MSTRG.39371.18	RHBDD1	XLOC_041021	1349	coding	coding	noncoding	noncoding
NR_102371.1	LOC654841	XLOC_041033	2502	coding	noncoding	coding	noncoding
MSTRG.39421.1	COL4A3	XLOC_041038	291	coding	noncoding	noncoding	noncoding
MSTRG.39425.1	COL4A3	XLOC_041040	230	coding	noncoding	noncoding	noncoding
NR_149019.1	LOC729968	XLOC_041056	1412	coding	noncoding	coding	coding
MSTRG.39275.1		XLOC_041058	265	coding	noncoding	noncoding	noncoding
MSTRG.39286.1		XLOC_041064	253	coding	noncoding	noncoding	noncoding
XR_001739909.1	LOC107985993	XLOC_041068	6081	coding	coding	noncoding	noncoding
MSTRG.39331.1		XLOC_041077	240	coding	noncoding	noncoding	noncoding
NM_001348324.2	TRIP12	XLOC_041113	10196	coding	coding	coding	coding
NM_001348315.2	TRIP12	XLOC_041113	10408	coding	coding	coding	coding
NM_001348323.1	TRIP12	XLOC_041113	10100	coding	coding	coding	coding
NM_001348328.1	TRIP12	XLOC_041113	10103	coding	coding	coding	coding
XM_005246961.4	TRIP12	XLOC_041113	7432	coding	coding	coding	coding
MSTRG.39484.1	FBXO36	XLOC_041130	271	coding	noncoding	noncoding	noncoding
MSTRG.39494.1	FBXO36	XLOC_041136	221	coding	noncoding	noncoding	noncoding
XM_005246525.4	SP110	XLOC_041146	6069	coding	coding	coding	coding
XM_024452851.1	SP110	XLOC_041146	1809	coding	coding	coding	coding
NM_004510.4	SP110	XLOC_041146	1952	coding	coding	coding	coding
MSTRG.39905.8	SP100	XLOC_041163	4454	coding	coding	noncoding	noncoding
MSTRG.39496.1		XLOC_041167	311	coding	noncoding	noncoding	noncoding
MSTRG.39501.1		XLOC_041168	237	coding	noncoding	noncoding	noncoding
MSTRG.39511.1		XLOC_041172	247	coding	noncoding	noncoding	noncoding
MSTRG.39512.1		XLOC_041174	281	coding	noncoding	noncoding	noncoding
MSTRG.39554.1	ARMC9	XLOC_041184	321	coding	noncoding	noncoding	noncoding
MSTRG.39538.1		XLOC_041194	208	coding	noncoding	noncoding	noncoding
MSTRG.39541.1		XLOC_041197	341	coding	noncoding	noncoding	noncoding
XM_006712195.3	NMUR1	XLOC_041207	1676	coding	coding	coding	coding
MSTRG.39586.1	LOC105376787	XLOC_041208	263	coding	noncoding	noncoding	noncoding
MSTRG.39604.1		XLOC_041215	263	coding	noncoding	noncoding	noncoding
MSTRG.39607.1		XLOC_041218	258	coding	noncoding	noncoding	noncoding
XM_011511245.3	NPPC	XLOC_041233	2700	coding	coding	coding	noncoding

MSTRG.39645.1	EFHD1	XLOC_041255	272	coding	noncoding	noncoding	noncoding
MSTRG.39649.1	EFHD1	XLOC_041258	294	coding	noncoding	noncoding	noncoding
MSTRG.39732.1	UGT1A8	XLOC_041284	263	coding	noncoding	noncoding	noncoding
MSTRG.39786.1		XLOC_041304	260	coding	noncoding	noncoding	noncoding
MSTRG.39821.1		XLOC_041318	229	coding	noncoding	noncoding	noncoding
MSTRG.39824.1	LOC101927896	XLOC_041320	272	coding	noncoding	noncoding	noncoding
MSTRG.39853.1	SH3BP4	XLOC_041329	241	coding	noncoding	coding	noncoding
MSTRG.40036.1	AGAP1	XLOC_041343	491	coding	noncoding	noncoding	noncoding
MSTRG.40037.1	AGAP1	XLOC_041344	300	coding	noncoding	noncoding	noncoding
MSTRG.40096.1	AGAP1	XLOC_041377	256	coding	coding	coding	noncoding
MSTRG.39934.1	IQCA1	XLOC_041392	317	coding	noncoding	noncoding	noncoding
NM_004369.3	COL6A3	XLOC_041431	10581	coding	coding	noncoding	coding
MSTRG.40126.1	COL6A3	XLOC_041435	322	coding	noncoding	noncoding	noncoding
MSTRG.40020.1		XLOC_041436	268	coding	noncoding	noncoding	noncoding
MSTRG.40128.1		XLOC_041443	376	coding	noncoding	noncoding	noncoding
MSTRG.40131.1		XLOC_041472	223	coding	noncoding	noncoding	noncoding
MSTRG.40176.1	ILKAP	XLOC_041486	356	coding	noncoding	noncoding	noncoding
NR_026925.1	LINC02610	XLOC_041488	1959	coding	noncoding	coding	coding
MSTRG.40231.1	PER2	XLOC_041492	277	coding	noncoding	noncoding	noncoding
MSTRG.40271.1		XLOC_041522	249	coding	noncoding	noncoding	noncoding
MSTRG.40279.1		XLOC_041525	421	coding	noncoding	noncoding	noncoding
MSTRG.40289.1		XLOC_041531	248	coding	noncoding	noncoding	noncoding
XM_011512230.1	HDAC4	XLOC_041533	7862	coding	coding	coding	coding
MSTRG.40540.1	HDAC4	XLOC_041539	206	coding	noncoding	noncoding	noncoding
MSTRG.40550.1	HDAC4	XLOC_041547	296	coding	noncoding	noncoding	noncoding
NM_001005853.1	OR6B2	XLOC_041587	939	coding	coding	coding	coding
MSTRG.40445.2	RNPEPL1	XLOC_041614	3654	coding	coding	coding	noncoding
NM_001085437.3	MAB21L4	XLOC_041634	2525	coding	coding	coding	coding
MSTRG.40407.1	CROCC2	XLOC_041640	280	coding	noncoding	noncoding	noncoding
MSTRG.40456.1	PASK	XLOC_041664	226	coding	noncoding	noncoding	noncoding
MSTRG.40517.1	FARP2	XLOC_041686	248	coding	noncoding	noncoding	noncoding
MSTRG.40488.1		XLOC_041696	281	coding	noncoding	noncoding	noncoding
XR_001739970.2	LOC105373975	XLOC_041699	865	coding	noncoding	noncoding	noncoding
XR_001739971.2	LOC105373975	XLOC_041699	2822	coding	noncoding	noncoding	noncoding
MSTRG.40602.8	ATG4B	XLOC_041701	928	coding	coding	coding	coding
MSTRG.40638.1	LOC107986057	XLOC_041742	316	coding	noncoding	noncoding	noncoding
MSTRG.40644.1		XLOC_041758	594	coding	noncoding	noncoding	noncoding
XR_001740584.1	LOC107986058	XLOC_041759	8296	coding	coding	coding	noncoding
MSTRG.40692.1	CNTN6	XLOC_041770	222	coding	noncoding	noncoding	noncoding
MSTRG.40696.1	CNTN6	XLOC_041773	325	coding	noncoding	noncoding	noncoding
MSTRG.40676.1		XLOC_041776	225	coding	noncoding	noncoding	noncoding
XM_011533428.2	CNTN4	XLOC_041779	6482	coding	coding	coding	coding
MSTRG.40736.1	CNTN4	XLOC_041786	415	coding	noncoding	noncoding	noncoding

MSTRG.40743.1	CNTN4	XLOC_041790	258	coding	noncoding	noncoding	noncoding
MSTRG.40748.1	CNTN4	XLOC_041794	318	coding	noncoding	noncoding	noncoding
MSTRG.40786.1	BHLHE40-AS1	XLOC_041953	527	coding	noncoding	noncoding	noncoding
MSTRG.40779.1	LOC105376934	XLOC_041967	268	coding	noncoding	noncoding	noncoding
MSTRG.40780.1	LOC105376934	XLOC_041968	303	coding	noncoding	noncoding	noncoding
MSTRG.40808.1		XLOC_041979	261	coding	noncoding	noncoding	noncoding
MSTRG.40897.1		XLOC_042007	221	coding	noncoding	noncoding	noncoding
XR_001740415.1	LOC105376944	XLOC_042009	2379	coding	noncoding	noncoding	noncoding
MSTRG.40911.1		XLOC_042039	269	coding	noncoding	noncoding	noncoding
MSTRG.40923.1		XLOC_042050	325	coding	coding	noncoding	noncoding
MSTRG.41125.1	SETD5	XLOC_042099	210	coding	noncoding	noncoding	noncoding
MSTRG.41084.1	LHFPL4	XLOC_042102	218	coding	noncoding	noncoding	noncoding
MSTRG.41086.1	LOC105376949	XLOC_042103	276	coding	noncoding	noncoding	noncoding
MSTRG.41089.1	CPNE9	XLOC_042110	214	coding	noncoding	noncoding	noncoding
MSTRG.41172.1	RPUSD3	XLOC_042114	258	coding	noncoding	noncoding	noncoding
MSTRG.41160.1	IRAK2	XLOC_042137	256	coding	noncoding	noncoding	noncoding
MSTRG.41190.1		XLOC_042158	259	coding	noncoding	noncoding	noncoding
XM_011534033.2	SLC6A11	XLOC_042159	3250	coding	coding	coding	coding
NM_001317406.2	SLC6A11	XLOC_042159	11069	coding	coding	coding	coding
NM_014229.3	SLC6A11	XLOC_042159	4249	coding	coding	coding	coding
MSTRG.41224.1		XLOC_042166	249	coding	noncoding	noncoding	noncoding
MSTRG.41364.1	LOC102723663	XLOC_042174	243	coding	noncoding	noncoding	noncoding
MSTRG.41367.1	LOC102723663	XLOC_042176	232	coding	noncoding	noncoding	noncoding
NM_001349233.2	ATG7	XLOC_042178	5087	coding	coding	coding	coding
XM_006712933.3	ATG7	XLOC_042178	2615	coding	coding	coding	coding
MSTRG.41396.1	TAMM41	XLOC_042181	240	coding	noncoding	noncoding	noncoding
MSTRG.41388.1	SYN2	XLOC_042194	239	coding	noncoding	noncoding	noncoding
MSTRG.41384.1		XLOC_042200	270	coding	noncoding	noncoding	noncoding
MSTRG.41419.1		XLOC_042203	340	coding	noncoding	noncoding	noncoding
XM_024453784.1	TSEN2	XLOC_042204	2003	coding	coding	coding	coding
XR_940599.2	LOC105376956	XLOC_042213	5188	coding	noncoding	coding	noncoding
MSTRG.41482.9	IQSEC1	XLOC_042213	3963	coding	coding	coding	noncoding
MSTRG.41446.1		XLOC_042243	280	coding	noncoding	noncoding	noncoding
MSTRG.41448.1		XLOC_042244	249	coding	noncoding	noncoding	noncoding
MSTRG.41450.1		XLOC_042252	316	coding	noncoding	noncoding	noncoding
NM_001165035.2	FBLN2	XLOC_042256	4563	coding	coding	coding	coding
MSTRG.41553.1	TPRXL	XLOC_042272	429	coding	noncoding	coding	noncoding
NR_103507.2	SLC6A6	XLOC_042288	6405	coding	coding	coding	coding
MSTRG.41651.1	GRIP2	XLOC_042296	272	coding	noncoding	noncoding	noncoding
MSTRG.41828.16	BTD	XLOC_042350	253	coding	noncoding	noncoding	noncoding
MSTRG.41828.18	BTD	XLOC_042350	265	coding	noncoding	noncoding	noncoding
MSTRG.41828.25	ANKRD28	XLOC_042350	867	coding	noncoding	noncoding	noncoding
MSTRG.41904.3	DPH3	XLOC_042385	2908	coding	coding	noncoding	noncoding

MSTRG.42365.5	TBC1D5	XLOC_042429	6386 coding	coding	noncoding	noncoding
MSTRG.42365.9	TBC1D5	XLOC_042429	867 coding	noncoding	noncoding	noncoding
MSTRG.41889.1	LOC339862	XLOC_042445	230 coding	noncoding	noncoding	noncoding
MSTRG.41974.1		XLOC_042464	641 coding	noncoding	noncoding	noncoding
MSTRG.41999.1		XLOC_042490	254 coding	noncoding	noncoding	noncoding
MSTRG.42067.1	RAB5A	XLOC_042501	321 coding	noncoding	noncoding	noncoding
MSTRG.42073.1	PP2D1	XLOC_042506	300 coding	coding	noncoding	noncoding
XR_001740351.1	KAT2B	XLOC_042508	4319 coding	coding	coding	coding
XM_005265528.4	KAT2B	XLOC_042508	4361 coding	coding	coding	coding
MSTRG.42189.1		XLOC_042535	348 coding	coding	noncoding	noncoding
MSTRG.42217.1	LOC105376988	XLOC_042543	295 coding	noncoding	noncoding	noncoding
MSTRG.42219.1	LOC105376988	XLOC_042544	248 coding	noncoding	noncoding	noncoding
MSTRG.42294.1	ZNF385D	XLOC_042562	276 coding	noncoding	noncoding	noncoding
MSTRG.42233.1		XLOC_042574	238 coding	noncoding	noncoding	noncoding
MSTRG.42245.1		XLOC_042579	218 coding	noncoding	noncoding	noncoding
MSTRG.42258.1		XLOC_042588	216 coding	coding	noncoding	noncoding
MSTRG.42260.1		XLOC_042589	260 coding	noncoding	noncoding	noncoding
NM_001370226.1	UBE2E2	XLOC_042594	3222 coding	coding	coding	coding
MSTRG.42730.1	UBE2E2	XLOC_042601	223 coding	noncoding	noncoding	noncoding
XR_940652.3	LOC105376993	XLOC_042627	3238 coding	noncoding	noncoding	noncoding
MSTRG.42320.1		XLOC_042630	262 coding	noncoding	noncoding	noncoding
MSTRG.42345.1	UBE2E1	XLOC_042639	325 coding	noncoding	noncoding	noncoding
MSTRG.42331.1		XLOC_042652	288 coding	noncoding	noncoding	noncoding
MSTRG.42338.1		XLOC_042654	256 coding	noncoding	noncoding	noncoding
MSTRG.42561.14	TOP2B	XLOC_042687	5503 coding	coding	coding	coding
MSTRG.42561.15	TOP2B	XLOC_042687	5281 coding	coding	noncoding	coding
MSTRG.42529.1		XLOC_042698	303 coding	noncoding	noncoding	noncoding
MSTRG.42550.1		XLOC_042709	262 coding	noncoding	noncoding	noncoding
MSTRG.42602.1		XLOC_042715	235 coding	coding	noncoding	noncoding
XM_017005675.1	LRRC3B	XLOC_042719	7522 coding	coding	coding	coding
MSTRG.42660.1		XLOC_042739	231 coding	noncoding	noncoding	noncoding
MSTRG.42663.1		XLOC_042741	253 coding	noncoding	noncoding	noncoding
MSTRG.42832.1	CMC1	XLOC_042759	255 coding	noncoding	noncoding	noncoding
MSTRG.42756.1		XLOC_042772	209 coding	noncoding	noncoding	noncoding
MSTRG.42932.1	GADL1	XLOC_042829	306 coding	noncoding	noncoding	noncoding
MSTRG.42937.1		XLOC_042834	356 coding	noncoding	noncoding	noncoding
MSTRG.42940.1		XLOC_042836	238 coding	noncoding	noncoding	noncoding
MSTRG.42952.1		XLOC_042844	355 coding	coding	noncoding	noncoding
MSTRG.42998.1		XLOC_042902	257 coding	coding	coding	noncoding
MSTRG.43112.1		XLOC_042948	298 coding	noncoding	noncoding	noncoding
MSTRG.43181.1	CNOT10	XLOC_042951	242 coding	noncoding	noncoding	noncoding
MSTRG.43163.1		XLOC_042954	232 coding	noncoding	noncoding	noncoding
NM_001039111.3	TRIM71	XLOC_042955	8704 coding	coding	coding	coding

MSTRG.43169.1	TRIM71	XLOC_042956	267 coding	coding	noncoding	noncoding
NM_005508.5	CCR4	XLOC_042957	3025 coding	coding	coding	coding
MSTRG.43198.1		XLOC_042962	500 coding	noncoding	noncoding	noncoding
MSTRG.43202.1		XLOC_042968	300 coding	noncoding	noncoding	noncoding
MSTRG.43227.1		XLOC_042978	290 coding	noncoding	noncoding	noncoding
MSTRG.43237.1		XLOC_042984	278 coding	noncoding	noncoding	noncoding
MSTRG.43250.1		XLOC_043000	217 coding	noncoding	noncoding	noncoding
MSTRG.43304.1		XLOC_043004	317 coding	noncoding	noncoding	noncoding
XM_017005575.2	ARPP21	XLOC_043020	15101 coding	coding	coding	coding
MSTRG.43376.1	ARPP21	XLOC_043022	281 coding	noncoding	noncoding	noncoding
MSTRG.43368.1		XLOC_043032	239 coding	noncoding	noncoding	noncoding
MSTRG.43398.1		XLOC_043042	535 coding	coding	noncoding	noncoding
MSTRG.43401.1		XLOC_043052	229 coding	noncoding	noncoding	noncoding
MSTRG.43721.1	C3orf35	XLOC_043081	244 coding	noncoding	noncoding	noncoding
MSTRG.43726.1	C3orf35	XLOC_043083	242 coding	noncoding	noncoding	noncoding
MSTRG.43738.1	C3orf35	XLOC_043084	284 coding	coding	noncoding	noncoding
MSTRG.43444.1	ITGA9	XLOC_043093	292 coding	noncoding	noncoding	noncoding
MSTRG.43470.1	CTDSPL	XLOC_043105	283 coding	noncoding	noncoding	noncoding
MSTRG.43474.1		XLOC_043112	286 coding	noncoding	noncoding	noncoding
MSTRG.43499.1		XLOC_043126	277 coding	noncoding	noncoding	noncoding
MSTRG.43506.1		XLOC_043132	265 coding	noncoding	noncoding	noncoding
XR_001740393.2	XYLB	XLOC_043134	1390 coding	coding	coding	coding
MSTRG.43547.1	XYLB	XLOC_043135	274 coding	noncoding	noncoding	noncoding
MSTRG.43553.1	XYLB	XLOC_043136	270 coding	noncoding	noncoding	noncoding
MSTRG.43567.1	XYLB	XLOC_043138	227 coding	noncoding	noncoding	noncoding
MSTRG.43570.1	XYLB	XLOC_043140	305 coding	noncoding	noncoding	noncoding
MSTRG.43616.1	SCN11A	XLOC_043152	377 coding	noncoding	noncoding	noncoding
NM_001346228.1	WDR48	XLOC_043154	4224 coding	coding	coding	coding
MSTRG.43598.6	CX3CR1	XLOC_043165	3260 coding	coding	coding	coding
MSTRG.43604.1		XLOC_043174	309 coding	noncoding	noncoding	noncoding
MSTRG.43835.1	MYRIP	XLOC_043187	268 coding	noncoding	noncoding	noncoding
MSTRG.43753.1	ENTPD3-AS1	XLOC_043251	212 coding	noncoding	noncoding	noncoding
MSTRG.43819.1	LOC105377043	XLOC_043277	235 coding	noncoding	noncoding	noncoding
MSTRG.43804.1		XLOC_043285	309 coding	noncoding	noncoding	noncoding
XM_024453356.1	CTNNB1	XLOC_043287	3594 coding	coding	coding	coding
MSTRG.43940.1	TRAK1	XLOC_043373	253 coding	noncoding	noncoding	noncoding
MSTRG.43911.1		XLOC_043383	282 coding	noncoding	noncoding	noncoding
MSTRG.43919.1		XLOC_043387	291 coding	noncoding	noncoding	noncoding
MSTRG.43921.1		XLOC_043388	575 coding	noncoding	noncoding	noncoding
MSTRG.43922.1		XLOC_043389	297 coding	noncoding	noncoding	noncoding
MSTRG.43972.1	HHATL	XLOC_043400	450 coding	noncoding	noncoding	noncoding
MSTRG.43985.1	CCDC13	XLOC_043411	246 coding	noncoding	noncoding	noncoding
MSTRG.43969.1		XLOC_043430	245 coding	noncoding	noncoding	noncoding

MSTRG.43993.1		XLOC_043441	255 coding	noncoding	noncoding	noncoding
NM_001355186.2	ABHD5	XLOC_043482	1456 coding	coding	coding	coding
XM_011533694.2	TOPAZ1	XLOC_043501	5637 coding	coding	coding	coding
NM_001258280.2	ZNF501	XLOC_043519	3353 coding	coding	noncoding	coding
NM_144638.3	TMEM42	XLOC_043523	977 coding	noncoding	coding	coding
MSTRG.44256.1	CDCP1	XLOC_043530	704 coding	noncoding	noncoding	noncoding
MSTRG.44282.1	LARS2	XLOC_043545	282 coding	noncoding	noncoding	noncoding
MSTRG.44467.1	ALS2CL	XLOC_043637	214 coding	noncoding	noncoding	noncoding
NM_001370524.1	TMIE	XLOC_043641	2417 coding	coding	coding	noncoding
MSTRG.44459.1		XLOC_043644	327 coding	noncoding	noncoding	noncoding
MSTRG.44577.5	CCDC12	XLOC_043651	795 coding	coding	noncoding	noncoding
MSTRG.44600.1	KIF9-AS1	XLOC_043660	314 coding	noncoding	noncoding	noncoding
MSTRG.44493.1		XLOC_043687	217 coding	noncoding	noncoding	noncoding
MSTRG.44517.1		XLOC_043694	246 coding	noncoding	noncoding	noncoding
MSTRG.44541.1		XLOC_043704	355 coding	noncoding	noncoding	noncoding
MSTRG.44635.1	NCKIPSD	XLOC_043722	670 coding	noncoding	noncoding	noncoding
MSTRG.44639.1		XLOC_043728	294 coding	noncoding	noncoding	noncoding
MSTRG.44680.1	P4HTM	XLOC_043742	325 coding	noncoding	noncoding	noncoding
MSTRG.44672.1		XLOC_043751	412 coding	noncoding	noncoding	noncoding
MSTRG.44702.1	C3orf84	XLOC_043758	389 coding	noncoding	noncoding	noncoding
XM_011533672.2	CCDC36	XLOC_043759	2683 coding	coding	coding	coding
MSTRG.44717.1	CCDC36	XLOC_043760	253 coding	noncoding	noncoding	noncoding
MSTRG.44718.1	CCDC36	XLOC_043761	273 coding	noncoding	noncoding	noncoding
MSTRG.44719.1		XLOC_043762	308 coding	noncoding	noncoding	noncoding
MSTRG.44721.1		XLOC_043764	256 coding	noncoding	noncoding	noncoding
MSTRG.44714.1	DAG1	XLOC_043774	367 coding	noncoding	noncoding	noncoding
MSTRG.44708.1		XLOC_043775	222 coding	noncoding	noncoding	noncoding
MSTRG.44751.1	BSN	XLOC_043777	270 coding	noncoding	noncoding	noncoding
MSTRG.44796.1	RBM6	XLOC_043795	287 coding	noncoding	noncoding	noncoding
MSTRG.44801.1	RBM6	XLOC_043799	237 coding	noncoding	noncoding	noncoding
MSTRG.44804.1	RBM6	XLOC_043801	325 coding	noncoding	noncoding	noncoding
NM_144499.3	GNAT1	XLOC_043807	3599 coding	coding	coding	coding
XM_006712954.3	SLC38A3	XLOC_043808	3012 coding	coding	coding	coding
MSTRG.44771.1		XLOC_043809	321 coding	noncoding	noncoding	noncoding
NM_001290060.2	SEMA3B	XLOC_043815	3184 coding	coding	coding	coding
NR_111913.2	CYB561D2	XLOC_043822	932 coding	coding	noncoding	noncoding
MSTRG.44829.1	HEMK1	XLOC_043843	295 coding	noncoding	noncoding	noncoding
MSTRG.44970.1	DOCK3	XLOC_043853	290 coding	noncoding	noncoding	noncoding
MSTRG.44972.1	DOCK3	XLOC_043855	271 coding	noncoding	noncoding	noncoding
MSTRG.44978.1	DOCK3	XLOC_043857	270 coding	noncoding	noncoding	noncoding
MSTRG.44990.1	DOCK3	XLOC_043864	230 coding	noncoding	noncoding	noncoding
MSTRG.45006.1	DOCK3	XLOC_043870	423 coding	coding	noncoding	noncoding
MSTRG.45007.1	DOCK3	XLOC_043871	317 coding	noncoding	noncoding	noncoding

MSTRG.45010.1	DOCK3	XLOC_043873	307 coding	noncoding	noncoding	noncoding
MSTRG.44899.1	LOC105377085	XLOC_043881	259 coding	noncoding	noncoding	noncoding
XM_011533635.1	GRM2	XLOC_043885	4937 coding	coding	coding	coding
MSTRG.44962.1	POC1A	XLOC_043912	253 coding	noncoding	noncoding	noncoding
MSTRG.44952.1		XLOC_043915	229 coding	noncoding	noncoding	noncoding
NR_026699.1	GLYCTK	XLOC_043922	3800 coding	coding	coding	coding
MSTRG.45088.1	DNAH1	XLOC_043924	217 coding	noncoding	noncoding	noncoding
XM_024453589.1	PHF7	XLOC_043927	2739 coding	coding	coding	coding
MSTRG.45032.1	PHF7	XLOC_043929	242 coding	noncoding	noncoding	noncoding
MSTRG.45043.1	NISCH	XLOC_043934	289 coding	noncoding	noncoding	noncoding
MSTRG.45168.7	PBRM1	XLOC_043940	8538 coding	coding	noncoding	coding
MSTRG.45053.5	ITIH1	XLOC_043948	203 coding	noncoding	noncoding	noncoding
MSTRG.45157.1		XLOC_043988	248 coding	noncoding	noncoding	noncoding
MSTRG.45232.1	CACNA1D	XLOC_043998	319 coding	noncoding	noncoding	noncoding
MSTRG.45193.2	IL17RB	XLOC_044004	2822 coding	coding	coding	coding
MSTRG.45182.1		XLOC_044008	254 coding	noncoding	noncoding	noncoding
MSTRG.45289.1	CACNA2D3	XLOC_044017	270 coding	noncoding	noncoding	noncoding
MSTRG.45302.1	CACNA2D3	XLOC_044022	241 coding	noncoding	noncoding	noncoding
MSTRG.45365.1	ERC2	XLOC_044057	269 coding	noncoding	noncoding	noncoding
MSTRG.45456.60	TASOR	XLOC_044069	5164 coding	coding	coding	coding
MSTRG.45453.1	DNAH12	XLOC_044099	245 coding	noncoding	noncoding	noncoding
MSTRG.45462.1	DENND6A-DT	XLOC_044115	268 coding	noncoding	noncoding	noncoding
MSTRG.45463.1		XLOC_044116	225 coding	noncoding	noncoding	noncoding
MSTRG.45464.1		XLOC_044117	233 coding	noncoding	noncoding	noncoding
MSTRG.45512.1	SLMAP	XLOC_044119	238 coding	noncoding	noncoding	noncoding
MSTRG.45499.1	LOC105377650	XLOC_044143	254 coding	noncoding	noncoding	noncoding
MSTRG.45504.1	DNASE1L3	XLOC_044145	260 coding	noncoding	noncoding	noncoding
XM_005265334.4	ABHD6	XLOC_044148	2463 coding	coding	coding	coding
NM_001348712.1	HTD2	XLOC_044150	3402 coding	coding	coding	coding
NR_146194.1	PXK	XLOC_044151	3041 coding	coding	coding	coding
MSTRG.45543.1		XLOC_044154	301 coding	noncoding	noncoding	noncoding
MSTRG.45547.1		XLOC_044155	350 coding	noncoding	noncoding	noncoding
MSTRG.45553.1	LOC107986092	XLOC_044157	235 coding	noncoding	noncoding	noncoding
MSTRG.45570.1	ACOX2	XLOC_044161	231 coding	coding	noncoding	noncoding
MSTRG.45579.1	FAM3D	XLOC_044166	212 coding	noncoding	noncoding	noncoding
MSTRG.45588.1	LOC105377109	XLOC_044170	244 coding	noncoding	noncoding	noncoding
MSTRG.45593.1	C3orf67	XLOC_044173	269 coding	noncoding	noncoding	noncoding
MSTRG.45594.1	C3orf67	XLOC_044174	293 coding	noncoding	noncoding	noncoding
MSTRG.45601.1	C3orf67	XLOC_044180	283 coding	noncoding	noncoding	noncoding
MSTRG.45604.1	C3orf67	XLOC_044183	256 coding	noncoding	noncoding	noncoding
MSTRG.47105.1	FHIT	XLOC_044222	216 coding	noncoding	noncoding	noncoding
MSTRG.45616.1	LOC105377114	XLOC_044314	230 coding	noncoding	noncoding	noncoding
MSTRG.45631.1	PTPRG	XLOC_044318	319 coding	noncoding	noncoding	noncoding

MSTRG.45647.1	PTPRG	XLOC_044325	314	coding	noncoding	noncoding	noncoding
MSTRG.45652.1	PTPRG	XLOC_044327	266	coding	noncoding	noncoding	noncoding
MSTRG.45667.1	PTPRG	XLOC_044335	287	coding	noncoding	noncoding	noncoding
MSTRG.45672.1	PTPRG-AS1	XLOC_044337	277	coding	noncoding	noncoding	noncoding
MSTRG.45677.1		XLOC_044352	294	coding	noncoding	noncoding	noncoding
MSTRG.45692.1	SYNPR	XLOC_044355	201	coding	noncoding	noncoding	noncoding
MSTRG.45696.1	SYNPR	XLOC_044359	220	coding	noncoding	noncoding	noncoding
MSTRG.45697.1	SYNPR	XLOC_044360	216	coding	noncoding	noncoding	noncoding
MSTRG.45714.1	SYNPR-AS1	XLOC_044368	289	coding	noncoding	noncoding	noncoding
MSTRG.45779.1	PRICKLE2	XLOC_044408	207	coding	noncoding	noncoding	noncoding
MSTRG.45786.1		XLOC_044427	280	coding	noncoding	noncoding	noncoding
MSTRG.45819.1	MAGI1	XLOC_044431	296	coding	noncoding	noncoding	noncoding
MSTRG.45823.1	MAGI1	XLOC_044434	302	coding	noncoding	noncoding	noncoding
MSTRG.45825.1	MAGI1	XLOC_044436	571	coding	noncoding	noncoding	noncoding
MSTRG.45835.1	MAGI1	XLOC_044441	369	coding	noncoding	noncoding	noncoding
MSTRG.45844.1	MAGI1	XLOC_044445	234	coding	noncoding	noncoding	noncoding
MSTRG.45880.1		XLOC_044467	394	coding	noncoding	coding	noncoding
MSTRG.45907.1		XLOC_044479	325	coding	coding	noncoding	noncoding
MSTRG.45908.1		XLOC_044480	200	coding	noncoding	noncoding	noncoding
MSTRG.46273.1	SUCLG2-AS1	XLOC_044520	332	coding	noncoding	noncoding	noncoding
MSTRG.46122.1	TAFA1	XLOC_044561	274	coding	noncoding	noncoding	noncoding
MSTRG.45988.1		XLOC_044584	216	coding	noncoding	noncoding	noncoding
MSTRG.46019.1	MITF	XLOC_044591	282	coding	noncoding	noncoding	noncoding
MSTRG.46052.1		XLOC_044611	245	coding	noncoding	noncoding	noncoding
MSTRG.46050.1		XLOC_044612	298	coding	noncoding	noncoding	noncoding
MSTRG.47644.9	FOXP1	XLOC_044617	2982	coding	coding	coding	noncoding
MSTRG.47644.48	EIF4E3	XLOC_044617	3368	coding	coding	noncoding	noncoding
MSTRG.46291.1		XLOC_044653	256	coding	noncoding	noncoding	noncoding
NM_001080393.2	GXYLT2	XLOC_044671	3276	coding	coding	coding	coding
MSTRG.46434.1	PDZRN3	XLOC_044688	291	coding	noncoding	noncoding	noncoding
MSTRG.46405.1		XLOC_044699	263	coding	noncoding	noncoding	noncoding
MSTRG.46415.1	LINC02047	XLOC_044705	279	coding	noncoding	noncoding	noncoding
MSTRG.46466.1	CNTN3	XLOC_044711	246	coding	noncoding	noncoding	noncoding
MSTRG.46450.1		XLOC_044720	240	coding	noncoding	noncoding	noncoding
MSTRG.46480.1	LOC105377167	XLOC_044725	289	coding	coding	noncoding	noncoding
MSTRG.46490.1		XLOC_044733	235	coding	noncoding	noncoding	noncoding
XM_017005489.2	FRG2C	XLOC_044739	2979	coding	coding	coding	coding
MSTRG.46584.1	ROBO2	XLOC_044759	286	coding	noncoding	noncoding	noncoding
MSTRG.46590.1	ROBO2	XLOC_044763	265	coding	noncoding	noncoding	noncoding
MSTRG.46595.1	ROBO2	XLOC_044765	272	coding	noncoding	noncoding	noncoding
MSTRG.46550.1		XLOC_044809	255	coding	noncoding	noncoding	noncoding
MSTRG.46553.1		XLOC_044810	273	coding	coding	noncoding	noncoding
MSTRG.46555.1		XLOC_044811	302	coding	noncoding	noncoding	noncoding

MSTRG.46728.1	ROBO1	XLOC_044854	274 coding	noncoding	noncoding	noncoding
MSTRG.46731.1	ROBO1	XLOC_044855	221 coding	noncoding	noncoding	noncoding
MSTRG.46732.1	ROBO1	XLOC_044856	310 coding	noncoding	noncoding	noncoding
MSTRG.46660.1		XLOC_044865	239 coding	noncoding	noncoding	noncoding
MSTRG.46667.1		XLOC_044868	271 coding	noncoding	noncoding	noncoding
MSTRG.46674.1		XLOC_044873	219 coding	noncoding	noncoding	noncoding
MSTRG.46750.1	LOC105377177	XLOC_044875	258 coding	noncoding	noncoding	noncoding
MSTRG.46772.1	LINC02027	XLOC_044888	321 coding	noncoding	noncoding	noncoding
MSTRG.46789.1		XLOC_044895	308 coding	noncoding	noncoding	noncoding
MSTRG.46814.1	LINC02008	XLOC_044910	233 coding	noncoding	noncoding	noncoding
MSTRG.46800.1		XLOC_044914	212 coding	noncoding	noncoding	noncoding
MSTRG.46830.1		XLOC_044923	213 coding	noncoding	noncoding	noncoding
MSTRG.46834.1	LOC101927439	XLOC_044926	341 coding	coding	noncoding	noncoding
MSTRG.46849.1		XLOC_044937	263 coding	noncoding	noncoding	noncoding
MSTRG.46911.1	CADM2	XLOC_044955	247 coding	noncoding	noncoding	noncoding
MSTRG.46918.1	CADM2	XLOC_044959	288 coding	noncoding	noncoding	noncoding
MSTRG.46872.1		XLOC_044966	252 coding	noncoding	noncoding	noncoding
MSTRG.46874.1		XLOC_044967	295 coding	noncoding	noncoding	noncoding
MSTRG.46882.1	LOC102723364	XLOC_044969	227 coding	noncoding	noncoding	noncoding
MSTRG.46883.1	LOC102723364	XLOC_044970	293 coding	noncoding	noncoding	noncoding
MSTRG.46962.1	LINC00506	XLOC_044985	284 coding	noncoding	noncoding	noncoding
MSTRG.46990.1	LOC105377198	XLOC_044999	310 coding	noncoding	noncoding	noncoding
XM_005264751.3	HTR1F	XLOC_045001	4412 coding	coding	coding	coding
MSTRG.47005.1	HTR1F	XLOC_045003	233 coding	noncoding	noncoding	noncoding
XM_011534340.3	ZNF654	XLOC_045006	3926 coding	noncoding	noncoding	coding
NM_001350136.2	ZNF654	XLOC_045006	6324 coding	coding	coding	coding
XM_005264745.5	C3orf38	XLOC_045027	2356 coding	coding	coding	coding
MSTRG.47240.1		XLOC_045029	210 coding	noncoding	noncoding	noncoding
MSTRG.47262.1		XLOC_045040	207 coding	noncoding	noncoding	noncoding
MSTRG.47269.1		XLOC_045044	257 coding	noncoding	noncoding	noncoding
MSTRG.47273.1		XLOC_045047	303 coding	noncoding	noncoding	noncoding
MSTRG.47283.1		XLOC_045051	220 coding	noncoding	noncoding	noncoding
MSTRG.47310.1	EPHA3	XLOC_045061	263 coding	noncoding	noncoding	noncoding
MSTRG.47294.1		XLOC_045067	355 coding	noncoding	noncoding	noncoding
MSTRG.47318.1		XLOC_045069	271 coding	noncoding	noncoding	noncoding
MSTRG.47327.1		XLOC_045073	273 coding	noncoding	noncoding	noncoding
MSTRG.47328.1		XLOC_045074	254 coding	noncoding	noncoding	noncoding
MSTRG.47362.1		XLOC_045076	231 coding	noncoding	noncoding	noncoding
MSTRG.47376.1		XLOC_045086	253 coding	noncoding	noncoding	noncoding
MSTRG.47383.1		XLOC_045090	250 coding	noncoding	noncoding	noncoding
XM_011512532.2	ARL13B	XLOC_045093	3618 coding	coding	coding	coding
MSTRG.47411.1		XLOC_045122	210 coding	coding	noncoding	noncoding
MSTRG.47436.1		XLOC_045132	381 coding	noncoding	noncoding	noncoding

MSTRG.47479.1		XLOC_045142	276 coding	noncoding	noncoding	noncoding
MSTRG.47546.1	GABRR3	XLOC_045180	302 coding	coding	noncoding	noncoding
MSTRG.47558.1		XLOC_045190	283 coding	coding	noncoding	noncoding
MSTRG.47608.1		XLOC_045214	224 coding	noncoding	noncoding	noncoding
NM_006100.3	ST3GAL6	XLOC_045219	3571 coding	coding	coding	coding
MSTRG.47629.1		XLOC_045251	251 coding	noncoding	coding	noncoding
MSTRG.47630.1		XLOC_045252	374 coding	noncoding	noncoding	noncoding
MSTRG.47633.1		XLOC_045254	291 coding	noncoding	noncoding	noncoding
MSTRG.47716.1	LOC105374007	XLOC_045262	314 coding	noncoding	noncoding	noncoding
MSTRG.47872.1	FILIP1L	XLOC_045286	261 coding	noncoding	noncoding	noncoding
MSTRG.47773.1		XLOC_045299	314 coding	coding	noncoding	noncoding
MSTRG.47819.1	LNP1	XLOC_045313	249 coding	noncoding	noncoding	noncoding
MSTRG.47846.1	ABI3BP	XLOC_045332	569 coding	noncoding	noncoding	noncoding
MSTRG.47822.1		XLOC_045336	279 coding	noncoding	noncoding	noncoding
MSTRG.47825.1		XLOC_045338	277 coding	noncoding	noncoding	noncoding
MSTRG.47831.1		XLOC_045341	318 coding	noncoding	noncoding	noncoding
MSTRG.47896.1		XLOC_045381	271 coding	noncoding	noncoding	noncoding
MSTRG.47950.1	NXPE3	XLOC_045389	211 coding	noncoding	noncoding	noncoding
MSTRG.47916.1		XLOC_045396	290 coding	noncoding	noncoding	noncoding
MSTRG.47917.1		XLOC_045397	225 coding	noncoding	noncoding	noncoding
MSTRG.47976.1		XLOC_045414	298 coding	coding	noncoding	noncoding
MSTRG.47987.1		XLOC_045427	269 coding	noncoding	noncoding	noncoding
MSTRG.47989.1		XLOC_045428	302 coding	noncoding	coding	noncoding
MSTRG.48168.1		XLOC_045503	289 coding	noncoding	noncoding	noncoding
MSTRG.48215.1	LOC101929485	XLOC_045511	247 coding	noncoding	noncoding	noncoding
MSTRG.48222.1		XLOC_045516	252 coding	noncoding	noncoding	noncoding
MSTRG.48226.1		XLOC_045520	235 coding	noncoding	noncoding	noncoding
MSTRG.48236.1	LINC00882	XLOC_045524	291 coding	noncoding	noncoding	noncoding
XM_024453651.1	BBX	XLOC_045541	3983 coding	coding	coding	coding
MSTRG.48262.1	LINC00635	XLOC_045597	551 coding	noncoding	noncoding	noncoding
MSTRG.48581.4	CD47	XLOC_045606	8888 coding	coding	coding	noncoding
MSTRG.48332.1		XLOC_045616	236 coding	noncoding	noncoding	noncoding
MSTRG.48340.1		XLOC_045640	217 coding	noncoding	noncoding	noncoding
MSTRG.48379.1	LINC01205	XLOC_045664	301 coding	noncoding	noncoding	noncoding
MSTRG.48420.1		XLOC_045665	294 coding	noncoding	noncoding	noncoding
MSTRG.48426.1		XLOC_045668	844 coding	noncoding	noncoding	noncoding
MSTRG.48419.1		XLOC_045673	236 coding	noncoding	coding	noncoding
MSTRG.48462.1		XLOC_045685	370 coding	noncoding	noncoding	noncoding
MSTRG.48466.1		XLOC_045687	255 coding	noncoding	noncoding	noncoding
XM_017005521.1	CD96	XLOC_045706	4140 coding	coding	coding	coding
MSTRG.48716.1	PLCXD2	XLOC_045733	244 coding	noncoding	noncoding	noncoding
MSTRG.48745.1	LOC105374040	XLOC_045742	277 coding	noncoding	noncoding	noncoding
MSTRG.48750.1	PHLDB2	XLOC_045745	262 coding	noncoding	noncoding	noncoding

MSTRG.48660.1	CCDC80	XLOC_045769	275 coding	coding	noncoding	noncoding
MSTRG.48671.1		XLOC_045780	457 coding	noncoding	noncoding	noncoding
NM_014170.3	GTPBP8	XLOC_045792	1913 coding	coding	coding	coding
NM_001301867.2	BOC	XLOC_045799	2181 coding	coding	coding	coding
MSTRG.48897.2	ATP6V1A	XLOC_045852	3187 coding	coding	coding	coding
MSTRG.48848.1		XLOC_045857	354 coding	noncoding	noncoding	noncoding
MSTRG.48933.1	GRAMD1C	XLOC_045859	492 coding	noncoding	noncoding	noncoding
MSTRG.48938.1	GRAMD1C	XLOC_045863	620 coding	noncoding	noncoding	noncoding
MSTRG.48939.1	GRAMD1C	XLOC_045864	265 coding	noncoding	noncoding	noncoding
MSTRG.48950.1	DRD3	XLOC_045886	294 coding	noncoding	noncoding	noncoding
MSTRG.49000.1	LOC101926886	XLOC_045899	233 coding	noncoding	noncoding	noncoding
MSTRG.49040.1		XLOC_045930	253 coding	noncoding	noncoding	noncoding
MSTRG.49049.1		XLOC_045936	294 coding	noncoding	noncoding	noncoding
MSTRG.49115.1		XLOC_045968	441 coding	coding	noncoding	noncoding
MSTRG.49177.1	LOC107983969	XLOC_045991	566 coding	noncoding	noncoding	noncoding
MSTRG.49189.1		XLOC_046013	239 coding	noncoding	noncoding	noncoding
MSTRG.49255.1	B4GALT4-AS1	XLOC_046022	292 coding	noncoding	noncoding	noncoding
XM_006713714.3	ARHGAP31	XLOC_046024	6349 coding	coding	coding	coding
MSTRG.49266.1	ARHGAP31	XLOC_046029	371 coding	noncoding	noncoding	noncoding
MSTRG.49268.1	ARHGAP31	XLOC_046031	293 coding	noncoding	noncoding	noncoding
XR_001740212.2	POGLUT1	XLOC_046038	3654 coding	coding	coding	coding
MSTRG.49245.1	MAATS1	XLOC_046055	247 coding	noncoding	noncoding	noncoding
MSTRG.49299.1	GPR156	XLOC_046068	222 coding	noncoding	noncoding	noncoding
XM_017007535.2	STXBP5L	XLOC_046103	6823 coding	coding	noncoding	coding
MSTRG.49433.1	STXBP5L	XLOC_046106	202 coding	noncoding	noncoding	noncoding
MSTRG.49512.1	CCDC58	XLOC_046164	250 coding	noncoding	noncoding	noncoding
XR_924405.3	LOC105374071	XLOC_046176	5347 coding	noncoding	noncoding	noncoding
MSTRG.49636.1	SLC49A4	XLOC_046196	239 coding	noncoding	noncoding	noncoding
MSTRG.49601.1	PDIA5	XLOC_046209	229 coding	noncoding	noncoding	noncoding
MSTRG.49700.1	MYLK-AS1	XLOC_046233	255 coding	noncoding	noncoding	noncoding
MSTRG.49715.1	MYLK	XLOC_046238	289 coding	noncoding	noncoding	noncoding
MSTRG.49676.4	CCDC14	XLOC_046244	3940 coding	coding	noncoding	noncoding
MSTRG.49720.1		XLOC_046263	230 coding	noncoding	noncoding	noncoding
MSTRG.49763.1	MUC13	XLOC_046268	291 coding	noncoding	noncoding	noncoding
MSTRG.49860.4	OSBPL11	XLOC_046294	528 coding	coding	noncoding	noncoding
MSTRG.49851.1		XLOC_046296	248 coding	noncoding	noncoding	noncoding
MSTRG.49854.1		XLOC_046298	220 coding	noncoding	noncoding	noncoding
MSTRG.49935.1	LOC107986044	XLOC_046339	202 coding	noncoding	noncoding	noncoding
MSTRG.50301.1	LOC107986129	XLOC_046405	246 coding	noncoding	noncoding	noncoding
MSTRG.50322.1		XLOC_046414	287 coding	noncoding	noncoding	noncoding
MSTRG.50384.1	RUVBL1	XLOC_046416	1403 coding	coding	coding	coding
MSTRG.50384.2	RUVBL1	XLOC_046416	1320 coding	coding	coding	coding
NR_125398.1	GATA2-AS1	XLOC_046435	2358 coding	noncoding	coding	noncoding

MSTRG.50338.1		XLOC_046445	229	coding	noncoding	noncoding	noncoding
MSTRG.50364.1	RAB7A	XLOC_046449	294	coding	noncoding	noncoding	noncoding
MSTRG.50485.1	ACAD9	XLOC_046467	296	coding	noncoding	noncoding	noncoding
XR_001740270.2	EFCC1	XLOC_046473	1319	coding	coding	coding	coding
XM_005247781.4	EFCC1	XLOC_046473	2778	coding	coding	coding	coding
MSTRG.50467.2	ISY1-RAB43	XLOC_046484	1936	coding	coding	noncoding	noncoding
MSTRG.50473.1	CNBP	XLOC_046487	1651	coding	coding	coding	coding
MSTRG.50516.1	COPG1	XLOC_046493	279	coding	noncoding	noncoding	noncoding
NM_007117.5	TRH	XLOC_046527	1560	coding	coding	coding	coding
NR_027898.1	COL6A4P2	XLOC_046539	9161	coding	coding	coding	coding
NR_022012.2	COL6A5	XLOC_046541	9228	coding	coding	noncoding	coding
NM_001199182.2	ATP2C1	XLOC_046562	3526	coding	coding	coding	coding
NM_014382.4	ATP2C1	XLOC_046562	4832	coding	coding	coding	coding
MSTRG.50826.1	NEK11	XLOC_046568	290	coding	noncoding	noncoding	noncoding
MSTRG.50672.1		XLOC_046595	258	coding	noncoding	noncoding	noncoding
MSTRG.50719.1	CPNE4	XLOC_046605	247	coding	noncoding	noncoding	noncoding
MSTRG.50749.1	CPNE4	XLOC_046625	243	coding	noncoding	noncoding	noncoding
MSTRG.50682.1	ACPP	XLOC_046638	283	coding	noncoding	noncoding	noncoding
MSTRG.50878.1	TMEM108	XLOC_046683	288	coding	noncoding	noncoding	noncoding
MSTRG.50866.1		XLOC_046704	558	coding	noncoding	noncoding	noncoding
MSTRG.50867.1		XLOC_046705	215	coding	noncoding	noncoding	noncoding
NM_001354703.2	TF	XLOC_046715	21134	coding	coding	coding	coding
MSTRG.50992.3	RYK	XLOC_046737	3485	coding	coding	coding	noncoding
NR_148353.1	CEP63	XLOC_046746	5546	coding	coding	coding	coding
MSTRG.51660.61	CEP63	XLOC_046746	3107	coding	coding	noncoding	noncoding
MSTRG.51660.62	CEP63	XLOC_046746	587	coding	noncoding	noncoding	noncoding
MSTRG.51016.1		XLOC_046823	471	coding	noncoding	noncoding	noncoding
MSTRG.51027.1		XLOC_046830	446	coding	noncoding	noncoding	noncoding
XM_017006787.2	PPP2R3A	XLOC_046831	8252	coding	coding	coding	coding
MSTRG.51029.1		XLOC_046833	251	coding	noncoding	noncoding	noncoding
MSTRG.51111.1		XLOC_046835	270	coding	noncoding	noncoding	noncoding
MSTRG.51048.1		XLOC_046844	300	coding	noncoding	noncoding	noncoding
MSTRG.51053.1		XLOC_046847	264	coding	noncoding	noncoding	noncoding
NM_025246.3	SLC35G2	XLOC_046848	1949	coding	coding	coding	coding
MSTRG.51178.1	IL20RB	XLOC_046866	358	coding	noncoding	noncoding	noncoding
MSTRG.51086.1		XLOC_046880	311	coding	noncoding	noncoding	noncoding
MSTRG.51097.1	LOC105374126	XLOC_046885	285	coding	noncoding	noncoding	noncoding
MSTRG.51228.1	NME9	XLOC_046916	226	coding	noncoding	noncoding	noncoding
MSTRG.51275.7	PIK3CB	XLOC_046939	595	coding	coding	noncoding	noncoding
MSTRG.51260.1		XLOC_046946	259	coding	coding	noncoding	noncoding
MSTRG.51264.1		XLOC_046947	279	coding	noncoding	noncoding	noncoding
MSTRG.51399.1	LOC100507291	XLOC_046954	253	coding	noncoding	noncoding	noncoding
MSTRG.51317.1	CLSTN2	XLOC_046971	245	coding	noncoding	noncoding	noncoding

MSTRG.51299.1	LOC102724068	XLOC_046984	252 coding	noncoding	noncoding	noncoding
MSTRG.51308.1		XLOC_046990	279 coding	noncoding	noncoding	noncoding
NM_080862.3	SPSB4	XLOC_047002	2963 coding	coding	coding	coding
XM_017007509.2	SPSB4	XLOC_047002	14504 coding	coding	coding	coding
MSTRG.51350.1	SPSB4	XLOC_047003	282 coding	noncoding	noncoding	noncoding
MSTRG.51462.1	GRK7	XLOC_047066	325 coding	noncoding	noncoding	noncoding
MSTRG.51474.1	ATP1B3	XLOC_047075	237 coding	noncoding	noncoding	noncoding
MSTRG.51555.1	LOC105374136	XLOC_047100	206 coding	noncoding	noncoding	noncoding
MSTRG.51565.1	PLS1	XLOC_047101	430 coding	noncoding	noncoding	noncoding
MSTRG.51572.1	PCOLCE2	XLOC_047106	257 coding	noncoding	noncoding	noncoding
MSTRG.51573.1	PCOLCE2	XLOC_047107	257 coding	noncoding	noncoding	noncoding
MSTRG.52401.1		XLOC_047131	268 coding	noncoding	noncoding	noncoding
MSTRG.51648.1		XLOC_047141	225 coding	noncoding	noncoding	noncoding
MSTRG.51827.1		XLOC_047148	275 coding	noncoding	noncoding	noncoding
MSTRG.51871.1	LOC105374140	XLOC_047160	245 coding	noncoding	noncoding	noncoding
MSTRG.51742.1		XLOC_047167	276 coding	noncoding	noncoding	noncoding
MSTRG.51746.1		XLOC_047170	359 coding	noncoding	noncoding	noncoding
MSTRG.51771.1		XLOC_047184	203 coding	noncoding	noncoding	noncoding
MSTRG.51779.1		XLOC_047188	297 coding	noncoding	noncoding	noncoding
MSTRG.51801.1		XLOC_047203	238 coding	noncoding	noncoding	noncoding
MSTRG.51884.1		XLOC_047207	270 coding	noncoding	noncoding	noncoding
MSTRG.51899.1	PLSCR4	XLOC_047211	298 coding	noncoding	noncoding	noncoding
MSTRG.51891.1		XLOC_047212	260 coding	noncoding	noncoding	noncoding
MSTRG.51904.1		XLOC_047221	281 coding	noncoding	noncoding	noncoding
MSTRG.51913.1		XLOC_047228	263 coding	noncoding	noncoding	noncoding
NM_003412.4	ZIC1	XLOC_047234	5260 coding	coding	coding	coding
MSTRG.51937.1		XLOC_047241	217 coding	noncoding	noncoding	noncoding
MSTRG.52059.1	CP	XLOC_047275	340 coding	coding	noncoding	noncoding
MSTRG.52060.1	CP	XLOC_047276	237 coding	noncoding	noncoding	noncoding
MSTRG.52026.1		XLOC_047279	251 coding	noncoding	noncoding	noncoding
MSTRG.52028.1		XLOC_047280	294 coding	noncoding	noncoding	noncoding
MSTRG.52045.1	WWTR1	XLOC_047289	286 coding	noncoding	noncoding	noncoding
MSTRG.52068.1		XLOC_047307	356 coding	noncoding	noncoding	noncoding
NM_183383.2	RNF13	XLOC_047308	2743 coding	coding	coding	coding
XM_017005658.1	RNF13	XLOC_047308	2950 coding	coding	coding	coding
MSTRG.52090.1		XLOC_047347	238 coding	noncoding	noncoding	noncoding
XM_011513337.3	TSC22D2	XLOC_047369	2792 coding	noncoding	coding	coding
MSTRG.52187.1		XLOC_047404	285 coding	noncoding	noncoding	noncoding
MSTRG.52250.1	CLRN1	XLOC_047415	217 coding	noncoding	noncoding	noncoding
MSTRG.52286.1	GPR87	XLOC_047430	412 coding	coding	noncoding	noncoding
MSTRG.52312.1	IGSF10	XLOC_047443	254 coding	noncoding	noncoding	noncoding
MSTRG.52314.1	IGSF10	XLOC_047444	279 coding	coding	noncoding	noncoding
NM_207365.4	AADACL2	XLOC_047456	5060 coding	coding	coding	coding

XM_017006433.2	MBNL1	XLOC_047501	4869 coding	noncoding	coding	coding
XM_017006426.1	MBNL1	XLOC_047501	5641 coding	noncoding	coding	coding
XR_924590.2	LOC102724289	XLOC_047525	3843 coding	coding	coding	noncoding
MSTRG.52351.1		XLOC_047527	288 coding	noncoding	noncoding	noncoding
MSTRG.52361.1		XLOC_047533	290 coding	noncoding	noncoding	noncoding
MSTRG.52516.1	ARHGEF26-AS1	XLOC_047560	257 coding	noncoding	noncoding	noncoding
MSTRG.52491.1	GPR149	XLOC_047574	247 coding	noncoding	noncoding	noncoding
MSTRG.52487.1		XLOC_047577	316 coding	coding	noncoding	noncoding
MSTRG.52502.1		XLOC_047581	503 coding	noncoding	noncoding	noncoding
MSTRG.52503.1		XLOC_047582	253 coding	coding	noncoding	noncoding
MSTRG.52549.1		XLOC_047590	244 coding	noncoding	noncoding	noncoding
MSTRG.52600.1		XLOC_047635	231 coding	noncoding	noncoding	noncoding
MSTRG.52610.1		XLOC_047647	310 coding	noncoding	noncoding	noncoding
MSTRG.52617.1		XLOC_047652	271 coding	noncoding	noncoding	noncoding
MSTRG.52640.1	PLCH1	XLOC_047654	452 coding	noncoding	coding	noncoding
MSTRG.52629.1		XLOC_047666	294 coding	noncoding	noncoding	noncoding
MSTRG.52671.1	GMPS	XLOC_047677	771 coding	noncoding	noncoding	noncoding
MSTRG.52665.1		XLOC_047680	276 coding	noncoding	noncoding	noncoding
MSTRG.52667.1		XLOC_047681	302 coding	noncoding	noncoding	noncoding
MSTRG.52792.1	TIPARP	XLOC_047692	254 coding	noncoding	noncoding	noncoding
NM_016625.3	RSRC1	XLOC_047748	1727 coding	noncoding	coding	coding
XM_006713730.2	MFSD1	XLOC_047799	1443 coding	coding	coding	coding
MSTRG.53093.1	IQCJ-SCHIP1	XLOC_047825	296 coding	noncoding	noncoding	noncoding
MSTRG.53099.1	IQCJ-SCHIP1	XLOC_047826	247 coding	noncoding	noncoding	noncoding
MSTRG.53050.1	IL12A-AS1	XLOC_047832	309 coding	noncoding	noncoding	noncoding
MSTRG.53051.1	IL12A-AS1	XLOC_047833	233 coding	noncoding	noncoding	noncoding
MSTRG.53171.1	TRIM59-IFT80	XLOC_047850	264 coding	coding	noncoding	noncoding
MSTRG.53173.1	TRIM59-IFT80	XLOC_047852	386 coding	noncoding	noncoding	noncoding
MSTRG.53142.1		XLOC_047857	256 coding	noncoding	noncoding	noncoding
NR_134243.1	PPM1L	XLOC_047870	10853 coding	coding	coding	noncoding
MSTRG.53444.1	PPM1L	XLOC_047894	298 coding	noncoding	noncoding	noncoding
MSTRG.53217.1		XLOC_047926	844 coding	noncoding	noncoding	noncoding
MSTRG.53219.1		XLOC_047928	324 coding	coding	noncoding	noncoding
MSTRG.53236.1		XLOC_047936	305 coding	noncoding	noncoding	noncoding
MSTRG.53257.1		XLOC_047948	265 coding	noncoding	noncoding	noncoding
MSTRG.53270.1		XLOC_047952	201 coding	noncoding	noncoding	noncoding
MSTRG.53278.1		XLOC_047956	314 coding	noncoding	noncoding	noncoding
MSTRG.53283.1		XLOC_047960	236 coding	noncoding	noncoding	noncoding
MSTRG.53299.1		XLOC_047966	264 coding	noncoding	noncoding	noncoding
MSTRG.53303.1		XLOC_047976	319 coding	noncoding	noncoding	noncoding
MSTRG.53337.1		XLOC_047989	273 coding	noncoding	noncoding	noncoding
MSTRG.53339.1		XLOC_047990	261 coding	noncoding	noncoding	noncoding
MSTRG.53351.1		XLOC_047991	272 coding	noncoding	noncoding	noncoding

MSTRG.53379.1		XLOC_048005	316	coding	noncoding	noncoding	noncoding
MSTRG.53384.1		XLOC_048008	303	coding	noncoding	noncoding	noncoding
MSTRG.53387.1		XLOC_048010	256	coding	noncoding	noncoding	noncoding
MSTRG.53397.1	LOC105374193	XLOC_048012	279	coding	noncoding	noncoding	noncoding
MSTRG.53409.1	ZBBX	XLOC_048019	250	coding	noncoding	noncoding	noncoding
MSTRG.53404.1		XLOC_048024	256	coding	coding	noncoding	noncoding
MSTRG.53405.1		XLOC_048025	281	coding	noncoding	noncoding	noncoding
MSTRG.53467.1	WDR49	XLOC_048034	317	coding	noncoding	noncoding	noncoding
MSTRG.53505.1		XLOC_048079	292	coding	noncoding	noncoding	noncoding
MSTRG.53614.1	MECOM	XLOC_048100	257	coding	noncoding	noncoding	noncoding
MSTRG.53617.1	MECOM	XLOC_048102	302	coding	noncoding	noncoding	noncoding
MSTRG.53622.1	MECOM	XLOC_048105	301	coding	noncoding	noncoding	noncoding
MSTRG.53599.1		XLOC_048117	450	coding	coding	noncoding	noncoding
MSTRG.53600.1		XLOC_048118	287	coding	noncoding	noncoding	noncoding
MSTRG.53601.1		XLOC_048119	275	coding	noncoding	noncoding	noncoding
MSTRG.53603.1		XLOC_048120	282	coding	noncoding	noncoding	noncoding
MSTRG.53608.1	ACTRT3	XLOC_048122	257	coding	coding	noncoding	noncoding
MSTRG.53641.1		XLOC_048131	362	coding	noncoding	noncoding	noncoding
MSTRG.53643.1		XLOC_048138	306	coding	noncoding	noncoding	noncoding
MSTRG.53661.1		XLOC_048147	240	coding	noncoding	noncoding	noncoding
MSTRG.53666.1	PRKCI	XLOC_048149	450	coding	noncoding	noncoding	noncoding
MSTRG.53663.1		XLOC_048150	260	coding	noncoding	noncoding	noncoding
NM_005602.6	CLDN11	XLOC_048153	2758	coding	coding	coding	coding
MSTRG.53668.1		XLOC_048155	294	coding	noncoding	noncoding	noncoding
MSTRG.53669.1		XLOC_048156	277	coding	noncoding	noncoding	noncoding
MSTRG.53691.1	SLC7A14-AS1	XLOC_048163	290	coding	noncoding	noncoding	noncoding
MSTRG.53693.1	SLC7A14-AS1	XLOC_048164	211	coding	noncoding	noncoding	noncoding
MSTRG.53673.1		XLOC_048168	296	coding	noncoding	noncoding	noncoding
MSTRG.53709.1	SLC2A2	XLOC_048178	317	coding	noncoding	noncoding	noncoding
MSTRG.53873.1	SPATA16	XLOC_048254	273	coding	noncoding	noncoding	noncoding
MSTRG.53877.1	SPATA16	XLOC_048255	254	coding	noncoding	noncoding	noncoding
MSTRG.53863.1	LOC105374224	XLOC_048259	306	coding	noncoding	noncoding	noncoding
XM_017005895.2	NLGN1	XLOC_048262	19459	coding	coding	coding	coding
MSTRG.53896.1	NLGN1	XLOC_048263	363	coding	noncoding	noncoding	noncoding
MSTRG.53912.1	NLGN1	XLOC_048268	402	coding	noncoding	noncoding	noncoding
MSTRG.54007.1	NAALADL2	XLOC_048283	304	coding	noncoding	noncoding	noncoding
MSTRG.53977.1		XLOC_048326	225	coding	noncoding	noncoding	noncoding
MSTRG.53983.1		XLOC_048329	280	coding	coding	noncoding	noncoding
MSTRG.54122.7	TBL1XR1	XLOC_048337	3229	coding	coding	coding	noncoding
MSTRG.54073.1	LINC00501	XLOC_048344	288	coding	noncoding	noncoding	noncoding
MSTRG.54114.1	LINC00578	XLOC_048358	265	coding	noncoding	noncoding	noncoding
MSTRG.54095.1	LINC02015	XLOC_048362	284	coding	noncoding	noncoding	noncoding
MSTRG.54187.1	KCNMB2-AS1	XLOC_048378	262	coding	coding	noncoding	noncoding

MSTRG.54188.1	KCNMB2-AS1	XLOC_048379	264	coding	noncoding	noncoding	noncoding
MSTRG.54195.1	KCNMB2-AS1	XLOC_048385	242	coding	noncoding	noncoding	noncoding
MSTRG.54210.1		XLOC_048398	685	coding	noncoding	noncoding	noncoding
MSTRG.54252.1	LOC101928739	XLOC_048418	210	coding	noncoding	noncoding	noncoding
MSTRG.54239.1		XLOC_048448	392	coding	noncoding	noncoding	noncoding
XM_017006550.1	ZNF639	XLOC_048450	3176	coding	noncoding	coding	coding
MSTRG.54246.1	ZNF639	XLOC_048451	340	coding	noncoding	noncoding	noncoding
MSTRG.54287.1	LOC107986157	XLOC_048456	342	coding	noncoding	noncoding	noncoding
MSTRG.54311.1	USP13	XLOC_048463	238	coding	noncoding	noncoding	noncoding
MSTRG.54322.1		XLOC_048472	275	coding	noncoding	noncoding	noncoding
MSTRG.54329.1		XLOC_048475	328	coding	coding	noncoding	noncoding
MSTRG.54330.1		XLOC_048476	287	coding	noncoding	noncoding	noncoding
MSTRG.54353.1	LOC101928882	XLOC_048489	257	coding	noncoding	noncoding	noncoding
NM_005087.4	FXR1	XLOC_048495	8343	coding	coding	coding	coding
MSTRG.54473.1	SOX2-OT	XLOC_048515	258	coding	noncoding	noncoding	noncoding
MSTRG.54385.1		XLOC_048520	313	coding	noncoding	noncoding	noncoding
MSTRG.54413.1		XLOC_048532	313	coding	coding	noncoding	noncoding
MSTRG.54520.1	LOC105374245	XLOC_048552	249	coding	noncoding	noncoding	noncoding
XM_017006008.1	ATP11B	XLOC_048554	6879	coding	coding	coding	coding
MSTRG.54535.1	ATP11B	XLOC_048566	285	coding	noncoding	noncoding	noncoding
MSTRG.54550.6	DCUN1D1	XLOC_048578	2675	coding	coding	noncoding	noncoding
MSTRG.54507.1		XLOC_048593	218	coding	noncoding	noncoding	noncoding
MSTRG.54614.1	YEATS2	XLOC_048630	303	coding	noncoding	noncoding	noncoding
MSTRG.54615.1	YEATS2	XLOC_048631	324	coding	noncoding	noncoding	noncoding
MSTRG.54611.1	MIR4448	XLOC_048642	235	coding	noncoding	coding	noncoding
MSTRG.54650.5	ABCC5	XLOC_048643	1779	coding	noncoding	coding	noncoding
MSTRG.54634.1		XLOC_048646	272	coding	noncoding	noncoding	noncoding
MSTRG.54635.1		XLOC_048648	219	coding	noncoding	noncoding	noncoding
MSTRG.54643.1	LOC105374249	XLOC_048651	281	coding	noncoding	noncoding	noncoding
NM_001311198.1	AP2M1	XLOC_048655	2015	coding	coding	coding	coding
NM_032331.3	EEF1AKMT4	XLOC_048660	990	coding	coding	coding	coding
MSTRG.54676.1	LINC02054	XLOC_048671	304	coding	noncoding	noncoding	noncoding
NM_004443.4	EPHB3	XLOC_048673	4234	coding	coding	coding	coding
MSTRG.54682.1		XLOC_048675	271	coding	noncoding	noncoding	noncoding
MSTRG.54693.1	LINC02069	XLOC_048680	275	coding	noncoding	noncoding	noncoding
MSTRG.54695.1	LINC02069	XLOC_048682	215	coding	noncoding	noncoding	noncoding
MSTRG.54922.1	VPS8	XLOC_048731	254	coding	noncoding	noncoding	noncoding
MSTRG.54934.1		XLOC_048742	254	coding	noncoding	noncoding	noncoding
MSTRG.54714.1	C3orf70	XLOC_048745	310	coding	noncoding	noncoding	noncoding
MSTRG.54719.1	EHHADH-AS1	XLOC_048747	306	coding	noncoding	noncoding	noncoding
XM_011513310.2	MAP3K13	XLOC_048754	3820	coding	coding	coding	coding
MSTRG.54742.1	MAP3K13	XLOC_048755	301	coding	noncoding	noncoding	noncoding
MSTRG.54748.1	MAP3K13	XLOC_048757	541	coding	noncoding	noncoding	noncoding

MSTRG.54759.1	LOC101929018	XLOC_048761	360	coding	noncoding	noncoding	noncoding
MSTRG.54735.1		XLOC_048769	324	coding	noncoding	noncoding	noncoding
MSTRG.54772.1	SENP2	XLOC_048771	230	coding	noncoding	noncoding	noncoding
MSTRG.54778.1		XLOC_048775	279	coding	noncoding	noncoding	noncoding
MSTRG.54799.1		XLOC_048783	260	coding	noncoding	noncoding	noncoding
MSTRG.54816.1		XLOC_048791	274	coding	noncoding	noncoding	noncoding
MSTRG.54831.1	LOC105374258	XLOC_048807	216	coding	coding	noncoding	noncoding
MSTRG.54834.1	KNG1	XLOC_048809	249	coding	noncoding	noncoding	noncoding
MSTRG.54837.1	LOC105374259	XLOC_048812	498	coding	noncoding	noncoding	noncoding
NM_001177800.2	ADIPOQ	XLOC_048821	4593	coding	coding	coding	coding
MSTRG.54959.1	ST6GAL1	XLOC_048827	238	coding	noncoding	noncoding	noncoding
MSTRG.55006.1		XLOC_048867	306	coding	noncoding	noncoding	noncoding
MSTRG.55891.1	LPP	XLOC_049000	272	coding	noncoding	noncoding	noncoding
MSTRG.55080.1		XLOC_049004	295	coding	noncoding	noncoding	noncoding
MSTRG.55223.1	TPRG1	XLOC_049016	414	coding	noncoding	noncoding	noncoding
MSTRG.55099.1	LOC107986169	XLOC_049024	258	coding	coding	noncoding	noncoding
MSTRG.55122.1	TP63	XLOC_049032	290	coding	noncoding	noncoding	noncoding
MSTRG.55124.1	TP63	XLOC_049033	263	coding	noncoding	noncoding	noncoding
MSTRG.55145.1	P3H2	XLOC_049040	257	coding	noncoding	noncoding	noncoding
MSTRG.55131.1		XLOC_049054	286	coding	noncoding	noncoding	noncoding
MSTRG.55134.1		XLOC_049056	317	coding	noncoding	noncoding	noncoding
MSTRG.55137.1		XLOC_049061	292	coding	noncoding	noncoding	noncoding
MSTRG.55171.1	TMEM207	XLOC_049066	247	coding	noncoding	noncoding	noncoding
MSTRG.55245.1		XLOC_049094	266	coding	noncoding	noncoding	noncoding
MSTRG.55289.1		XLOC_049119	279	coding	noncoding	noncoding	noncoding
MSTRG.55295.1	LINCR-0002	XLOC_049123	313	coding	noncoding	noncoding	noncoding
MSTRG.55296.1	LINCR-0002	XLOC_049124	264	coding	noncoding	noncoding	noncoding
MSTRG.55317.1		XLOC_049136	313	coding	noncoding	noncoding	noncoding
MSTRG.55361.1	FGF12	XLOC_049143	285	coding	noncoding	noncoding	noncoding
MSTRG.55364.1	FGF12	XLOC_049145	228	coding	noncoding	noncoding	noncoding
MSTRG.55378.1	FGF12	XLOC_049152	291	coding	noncoding	noncoding	noncoding
MSTRG.55388.1		XLOC_049160	312	coding	noncoding	noncoding	noncoding
MSTRG.55392.1		XLOC_049162	258	coding	noncoding	noncoding	noncoding
MSTRG.55400.1		XLOC_049166	296	coding	noncoding	noncoding	noncoding
MSTRG.55412.1	PLAAT1	XLOC_049169	723	coding	noncoding	noncoding	noncoding
MSTRG.55418.1	ATP13A5	XLOC_049173	277	coding	noncoding	noncoding	noncoding
MSTRG.55408.1		XLOC_049174	305	coding	noncoding	noncoding	noncoding
MSTRG.55484.1	ATP13A4	XLOC_049181	238	coding	noncoding	noncoding	noncoding
MSTRG.55505.17	OPA1	XLOC_049195	1594	coding	noncoding	noncoding	noncoding
MSTRG.55505.22	OPA1	XLOC_049195	1914	coding	noncoding	coding	coding
MSTRG.55425.1		XLOC_049206	805	coding	noncoding	noncoding	noncoding
MSTRG.55429.1	LOC105374287	XLOC_049208	252	coding	noncoding	noncoding	noncoding
MSTRG.55453.1	LINC02028	XLOC_049214	291	coding	coding	noncoding	noncoding

MSTRG.55455.1	LINC02028	XLOC_049215	223 coding	noncoding	noncoding	noncoding
MSTRG.55441.1	LINC02036	XLOC_049220	298 coding	noncoding	noncoding	noncoding
MSTRG.55442.1	LINC02036	XLOC_049221	224 coding	noncoding	noncoding	noncoding
MSTRG.55473.1		XLOC_049233	275 coding	noncoding	noncoding	noncoding
MSTRG.55534.1	TMEM44	XLOC_049237	283 coding	noncoding	noncoding	noncoding
MSTRG.55557.1	LINC01972	XLOC_049247	309 coding	noncoding	noncoding	noncoding
MSTRG.55570.1		XLOC_049256	220 coding	noncoding	noncoding	noncoding
MSTRG.55623.1		XLOC_049286	276 coding	noncoding	noncoding	noncoding
MSTRG.55675.1		XLOC_049305	296 coding	noncoding	noncoding	noncoding
MSTRG.55678.1		XLOC_049308	309 coding	noncoding	noncoding	noncoding
MSTRG.55720.1	LINC00885	XLOC_049335	239 coding	noncoding	noncoding	noncoding
MSTRG.55718.1		XLOC_049337	268 coding	noncoding	noncoding	noncoding
MSTRG.55741.1	TCTEX1D2	XLOC_049344	369 coding	noncoding	noncoding	noncoding
MSTRG.55754.1	UBXN7	XLOC_049352	257 coding	noncoding	noncoding	noncoding
MSTRG.55929.1		XLOC_049364	274 coding	noncoding	noncoding	noncoding
MSTRG.55944.1	PAK2	XLOC_049376	362 coding	noncoding	noncoding	noncoding
MSTRG.55949.1	PAK2	XLOC_049381	279 coding	noncoding	noncoding	noncoding
MSTRG.55776.1		XLOC_049385	285 coding	noncoding	noncoding	noncoding
MSTRG.55777.1		XLOC_049386	252 coding	noncoding	noncoding	noncoding
NM_001308045.1	SENP5	XLOC_049387	6170 coding	coding	coding	coding
MSTRG.55908.1	NCBP2AS2	XLOC_049389	300 coding	noncoding	noncoding	noncoding
MSTRG.55959.1		XLOC_049406	514 coding	noncoding	noncoding	noncoding
MSTRG.55961.1		XLOC_049407	306 coding	noncoding	noncoding	noncoding
MSTRG.55968.1		XLOC_049410	241 coding	noncoding	noncoding	noncoding
MSTRG.56036.1	FYTTD1	XLOC_049433	372 coding	noncoding	noncoding	noncoding
MSTRG.56062.1	LOC105374310	XLOC_049441	529 coding	coding	noncoding	noncoding
MSTRG.56037.1		XLOC_049456	293 coding	noncoding	noncoding	noncoding
MSTRG.40648.1	LINC01266	XLOC_049473	246 coding	noncoding	noncoding	noncoding
MSTRG.40669.1		XLOC_049480	309 coding	noncoding	noncoding	noncoding
MSTRG.40677.1		XLOC_049486	254 coding	coding	noncoding	noncoding
MSTRG.40727.1	CNTN4	XLOC_049493	296 coding	noncoding	noncoding	noncoding
MSTRG.40759.1	IL5RA	XLOC_049505	342 coding	noncoding	noncoding	noncoding
MSTRG.40698.1		XLOC_049507	231 coding	noncoding	noncoding	noncoding
MSTRG.40886.1		XLOC_049641	278 coding	noncoding	noncoding	noncoding
MSTRG.40894.1		XLOC_049645	232 coding	noncoding	noncoding	noncoding
MSTRG.40898.1		XLOC_049647	221 coding	noncoding	noncoding	noncoding
MSTRG.40939.1	GRM7	XLOC_049656	243 coding	noncoding	noncoding	noncoding
MSTRG.40910.1		XLOC_049672	269 coding	noncoding	noncoding	noncoding
MSTRG.40921.1		XLOC_049679	246 coding	noncoding	noncoding	noncoding
XM_017006535.1	SSUH2	XLOC_049693	6564 coding	noncoding	coding	coding
MSTRG.41082.1		XLOC_049710	225 coding	noncoding	noncoding	noncoding
MSTRG.41170.3	TTLL3	XLOC_049718	678 coding	noncoding	noncoding	noncoding
MSTRG.41153.1	EMC3	XLOC_049731	870 coding	noncoding	noncoding	noncoding

NM_018447.3	EMC3	XLOC_049732	2642	coding	coding	coding	coding
XM_011533330.2	FANCD2OS	XLOC_049736	1510	coding	coding	coding	coding
MSTRG.41181.1		XLOC_049743	293	coding	noncoding	noncoding	noncoding
MSTRG.41368.1	HRH1	XLOC_049779	331	coding	noncoding	noncoding	noncoding
MSTRG.41385.1		XLOC_049795	235	coding	noncoding	noncoding	noncoding
XM_017006966.1	RAF1	XLOC_049808	2880	coding	coding	coding	coding
MSTRG.41421.1		XLOC_049810	254	coding	noncoding	noncoding	noncoding
NM_014869.7	IQSEC1	XLOC_049818	5518	coding	coding	coding	coding
MSTRG.41502.1	IQSEC1	XLOC_049833	437	coding	noncoding	noncoding	noncoding
XM_024453855.1	LOC112267878	XLOC_049846	5057	coding	noncoding	coding	coding
MSTRG.41575.1	LOC112267878	XLOC_049853	284	coding	noncoding	noncoding	noncoding
MSTRG.41580.1		XLOC_049857	264	coding	noncoding	noncoding	noncoding
MSTRG.41449.1		XLOC_049868	318	coding	noncoding	noncoding	noncoding
MSTRG.41452.1		XLOC_049869	303	coding	noncoding	noncoding	noncoding
NM_004625.4	WNT7A	XLOC_049884	3991	coding	coding	coding	coding
MSTRG.41554.1	TPRXL	XLOC_049893	267	coding	noncoding	noncoding	noncoding
XR_002959614.1	LOC112268445	XLOC_049897	8815	coding	coding	noncoding	coding
MSTRG.41622.1	FGD5	XLOC_049919	200	coding	noncoding	noncoding	noncoding
MSTRG.41669.16	MRPS25	XLOC_049923	11765	coding	coding	coding	coding
MSTRG.41657.1		XLOC_049924	213	coding	noncoding	noncoding	noncoding
MSTRG.41659.1		XLOC_049926	259	coding	noncoding	noncoding	noncoding
MSTRG.41844.14	BTD	XLOC_049941	239	coding	noncoding	noncoding	noncoding
MSTRG.41844.16	BTD	XLOC_049941	214	coding	noncoding	noncoding	noncoding
MSTRG.41726.1		XLOC_049970	503	coding	noncoding	noncoding	noncoding
MSTRG.41906.1	RFTN1	XLOC_050022	2645	coding	coding	coding	noncoding
MSTRG.41906.12	OXNAD1	XLOC_050022	3031	coding	coding	noncoding	noncoding
MSTRG.41906.13	OXNAD1	XLOC_050022	1491	coding	coding	noncoding	noncoding
NM_001134381.1	TBC1D5	XLOC_050065	6519	coding	coding	coding	coding
XM_017007556.1	TBC1D5	XLOC_050065	6762	coding	coding	coding	coding
MSTRG.42396.1	TBC1D5	XLOC_050094	265	coding	noncoding	noncoding	noncoding
MSTRG.42434.1	TBC1D5	XLOC_050131	386	coding	noncoding	noncoding	noncoding
MSTRG.42435.1	TBC1D5	XLOC_050132	283	coding	noncoding	noncoding	noncoding
MSTRG.42438.1	TBC1D5	XLOC_050134	223	coding	noncoding	noncoding	noncoding
MSTRG.41972.1		XLOC_050202	251	coding	noncoding	noncoding	noncoding
MSTRG.41981.1	LOC107986066	XLOC_050205	221	coding	noncoding	noncoding	noncoding
MSTRG.41988.1		XLOC_050246	285	coding	coding	noncoding	noncoding
MSTRG.42049.1		XLOC_050257	273	coding	noncoding	noncoding	noncoding
MSTRG.42220.1	LOC105376988	XLOC_050361	248	coding	noncoding	noncoding	noncoding
MSTRG.42214.1		XLOC_050362	358	coding	noncoding	noncoding	noncoding
NM_024697.3	ZNF385D	XLOC_050365	10478	coding	coding	coding	coding
XM_011534122.2	ZNF385D	XLOC_050365	9216	coding	coding	coding	coding
MSTRG.42293.1	ZNF385D	XLOC_050382	276	coding	noncoding	noncoding	noncoding
MSTRG.42230.1		XLOC_050393	206	coding	noncoding	noncoding	noncoding

MSTRG.42259.1		XLOC_050405	260	coding	noncoding	noncoding	noncoding
MSTRG.42261.1		XLOC_050406	267	coding	noncoding	noncoding	noncoding
MSTRG.42317.1	LOC105376993	XLOC_050412	272	coding	noncoding	noncoding	noncoding
MSTRG.42321.1		XLOC_050414	262	coding	noncoding	noncoding	noncoding
MSTRG.42339.5	UBE2E1	XLOC_050416	1946	coding	coding	noncoding	noncoding
MSTRG.42328.1		XLOC_050420	225	coding	noncoding	noncoding	noncoding
MSTRG.42334.2	NR1D2	XLOC_050421	2799	coding	coding	noncoding	coding
MSTRG.42332.1		XLOC_050425	289	coding	noncoding	noncoding	noncoding
MSTRG.42562.1	RARB	XLOC_050457	277	coding	noncoding	noncoding	noncoding
MSTRG.42578.1	RARB	XLOC_050473	218	coding	noncoding	noncoding	noncoding
MSTRG.42579.1	RARB	XLOC_050474	274	coding	noncoding	noncoding	noncoding
MSTRG.42590.1	RARB	XLOC_050485	305	coding	noncoding	noncoding	noncoding
MSTRG.42531.1		XLOC_050503	240	coding	noncoding	noncoding	noncoding
MSTRG.42551.1		XLOC_050514	262	coding	noncoding	noncoding	noncoding
MSTRG.42601.1		XLOC_050521	236	coding	noncoding	noncoding	noncoding
MSTRG.42605.1		XLOC_050525	294	coding	coding	noncoding	noncoding
MSTRG.42624.1		XLOC_050534	269	coding	noncoding	noncoding	noncoding
MSTRG.42628.1		XLOC_050537	279	coding	noncoding	noncoding	noncoding
MSTRG.42638.1		XLOC_050539	281	coding	noncoding	noncoding	noncoding
MSTRG.42641.1		XLOC_050540	216	coding	noncoding	noncoding	noncoding
MSTRG.42655.1	NEK10	XLOC_050546	305	coding	noncoding	noncoding	noncoding
MSTRG.42855.1	ZCWPW2	XLOC_050608	291	coding	noncoding	noncoding	noncoding
MSTRG.42763.1		XLOC_050621	259	coding	noncoding	noncoding	noncoding
MSTRG.42782.1		XLOC_050625	248	coding	noncoding	noncoding	noncoding
MSTRG.42930.1	GADL1	XLOC_050701	338	coding	noncoding	noncoding	noncoding
MSTRG.42931.1	GADL1	XLOC_050702	342	coding	noncoding	noncoding	noncoding
MSTRG.42936.1		XLOC_050709	291	coding	noncoding	noncoding	noncoding
MSTRG.42939.1		XLOC_050712	342	coding	noncoding	noncoding	noncoding
MSTRG.42953.1		XLOC_050718	436	coding	coding	noncoding	noncoding
MSTRG.43059.1	OSBPL10	XLOC_050734	268	coding	noncoding	noncoding	noncoding
MSTRG.43062.1	OSBPL10	XLOC_050737	206	coding	noncoding	noncoding	noncoding
MSTRG.43081.1	OSBPL10	XLOC_050741	297	coding	noncoding	noncoding	noncoding
MSTRG.43005.1		XLOC_050762	456	coding	noncoding	noncoding	noncoding
MSTRG.43113.1		XLOC_050805	276	coding	noncoding	noncoding	noncoding
MSTRG.43116.1		XLOC_050807	249	coding	noncoding	noncoding	noncoding
MSTRG.43162.1		XLOC_050812	232	coding	noncoding	noncoding	noncoding
MSTRG.43167.1		XLOC_050818	268	coding	noncoding	noncoding	noncoding
MSTRG.43187.1	GLB1	XLOC_050825	310	coding	noncoding	noncoding	noncoding
MSTRG.43216.1		XLOC_050846	261	coding	noncoding	noncoding	noncoding
MSTRG.43367.1		XLOC_050946	239	coding	noncoding	noncoding	noncoding
MSTRG.43393.1	STAC	XLOC_050949	730	coding	coding	noncoding	coding
MSTRG.43399.1		XLOC_050951	305	coding	coding	noncoding	noncoding
MSTRG.43420.1	TRANK1	XLOC_050971	290	coding	noncoding	noncoding	noncoding

MSTRG.43679.1	LRRFIP2	XLOC_050988	262 coding	noncoding	noncoding	noncoding
MSTRG.43467.4	CTDSPL	XLOC_051053	1251 coding	coding	noncoding	noncoding
MSTRG.43546.1	XYLB	XLOC_051081	274 coding	noncoding	noncoding	noncoding
MSTRG.43548.1	XYLB	XLOC_051082	280 coding	noncoding	noncoding	noncoding
MSTRG.43554.1	XYLB	XLOC_051087	270 coding	noncoding	noncoding	noncoding
MSTRG.43614.1	SCN11A	XLOC_051125	322 coding	noncoding	noncoding	noncoding
MSTRG.43617.1	SCN11A	XLOC_051126	314 coding	noncoding	noncoding	noncoding
MSTRG.43627.1	TTC21A	XLOC_051132	638 coding	noncoding	noncoding	noncoding
MSTRG.43592.1		XLOC_051145	262 coding	noncoding	noncoding	noncoding
MSTRG.43657.1		XLOC_051160	300 coding	noncoding	noncoding	noncoding
MSTRG.43834.1	MYRIP	XLOC_051166	268 coding	noncoding	noncoding	noncoding
MSTRG.43853.1	MYRIP	XLOC_051172	282 coding	noncoding	noncoding	noncoding
MSTRG.43748.1		XLOC_051177	297 coding	noncoding	noncoding	noncoding
MSTRG.43789.1		XLOC_051194	243 coding	coding	noncoding	noncoding
MSTRG.43818.1	LOC105377043	XLOC_051208	235 coding	noncoding	noncoding	noncoding
MSTRG.43905.1		XLOC_051225	252 coding	noncoding	noncoding	noncoding
MSTRG.43918.1		XLOC_051235	291 coding	noncoding	noncoding	noncoding
MSTRG.43923.1		XLOC_051237	241 coding	noncoding	noncoding	noncoding
XR_002959565.1	HHATL	XLOC_051245	2136 coding	noncoding	coding	coding
NM_004391.3	CYP8B1	XLOC_051256	3688 coding	coding	coding	coding
MSTRG.43952.1	CYP8B1	XLOC_051256	546 coding	noncoding	noncoding	noncoding
MSTRG.43964.1		XLOC_051260	280 coding	noncoding	noncoding	noncoding
MSTRG.44164.8	SNRK	XLOC_051273	6132 coding	coding	noncoding	noncoding
NM_001204831.2	ANO10	XLOC_051275	2746 coding	coding	coding	coding
NM_181489.6	ZNF445	XLOC_051310	18314 coding	coding	coding	coding
NM_020696.4	KIAA1143	XLOC_051327	5079 coding	coding	coding	coding
XM_017006562.1	ZDHHC3	XLOC_051329	4067 coding	coding	coding	coding
XM_011534024.1	CDCP1	XLOC_051342	5965 coding	noncoding	coding	coding
MSTRG.44238.1	LOC101928636	XLOC_051346	250 coding	noncoding	noncoding	noncoding
NR_075080.2	LZTFL1	XLOC_051366	3901 coding	coding	coding	noncoding
XM_006713333.3	FYCO1	XLOC_051370	9679 coding	coding	coding	coding
MSTRG.44368.3	CCR2	XLOC_051406	3705 coding	coding	noncoding	noncoding
MSTRG.44377.1	LOC102724297	XLOC_051408	413 coding	noncoding	noncoding	noncoding
XR_940406.2	ALS2CL	XLOC_051434	6609 coding	coding	coding	coding
MSTRG.44457.1		XLOC_051443	315 coding	noncoding	noncoding	noncoding
MSTRG.44463.1		XLOC_051446	311 coding	coding	noncoding	noncoding
XR_002959493.1	CCDC12	XLOC_051449	7770 coding	coding	coding	coding
MSTRG.44576.13	CCDC12	XLOC_051449	13463 coding	coding	coding	coding
XM_005264967.2	SCAP	XLOC_051470	4207 coding	coding	coding	coding
XM_011533852.3	ELP6	XLOC_051473	2934 coding	coding	coding	coding
MSTRG.44500.1	SMARCC1	XLOC_051477	310 coding	noncoding	noncoding	noncoding
MSTRG.44507.1	SMARCC1	XLOC_051484	295 coding	noncoding	noncoding	noncoding
MSTRG.44558.1	MAP4	XLOC_051498	246 coding	noncoding	noncoding	noncoding

MSTRG.44494.1		XLOC_051504	254 coding	noncoding	noncoding	noncoding
XR_940457.2	PLXNB1	XLOC_051518	11147 coding	coding	coding	coding
MSTRG.44617.3	UQCRC1	XLOC_051530	255 coding	noncoding	noncoding	noncoding
MSTRG.44609.1		XLOC_051533	218 coding	noncoding	noncoding	noncoding
MSTRG.44634.1	LINC02585	XLOC_051538	243 coding	noncoding	noncoding	noncoding
MSTRG.44642.1	IP6K2	XLOC_051540	283 coding	noncoding	noncoding	noncoding
MSTRG.44643.1	IP6K2	XLOC_051541	291 coding	noncoding	noncoding	noncoding
MSTRG.44644.1	IP6K2	XLOC_051542	252 coding	noncoding	noncoding	noncoding
MSTRG.44651.1	PRKAR2A	XLOC_051547	282 coding	noncoding	noncoding	noncoding
MSTRG.44653.1	PRKAR2A	XLOC_051549	287 coding	noncoding	noncoding	noncoding
MSTRG.44724.1	C3orf62	XLOC_051577	229 coding	noncoding	noncoding	noncoding
NM_001313946.2	RHOA	XLOC_051584	1553 coding	coding	coding	noncoding
MSTRG.44709.1		XLOC_051589	222 coding	noncoding	noncoding	noncoding
MSTRG.44752.1	BSN	XLOC_051593	270 coding	noncoding	noncoding	noncoding
MSTRG.44753.1	BSN	XLOC_051594	225 coding	noncoding	noncoding	noncoding
MSTRG.44754.1	BSN	XLOC_051595	239 coding	noncoding	noncoding	noncoding
MSTRG.44757.1	BSN	XLOC_051596	402 coding	noncoding	coding	noncoding
MSTRG.44760.2	MST1	XLOC_051598	10522 coding	coding	coding	coding
MSTRG.44769.1	IP6K1	XLOC_051601	380 coding	noncoding	noncoding	noncoding
MSTRG.44783.1	SEMA3F-AS1	XLOC_051622	268 coding	noncoding	noncoding	noncoding
MSTRG.44773.1	GNAT1	XLOC_051623	584 coding	noncoding	noncoding	coding
MSTRG.44821.1	HYAL3	XLOC_051633	246 coding	noncoding	noncoding	noncoding
NR_047690.1	HYAL1	XLOC_051635	2547 coding	coding	coding	coding
XR_001739982.1	NPRL2	XLOC_051641	1435 coding	coding	coding	coding
MSTRG.44828.1		XLOC_051660	297 coding	noncoding	noncoding	noncoding
MSTRG.44895.1		XLOC_051673	223 coding	noncoding	noncoding	noncoding
MSTRG.45009.1	DOCK3	XLOC_051699	308 coding	noncoding	noncoding	noncoding
MSTRG.44908.1	GRM2	XLOC_051720	319 coding	coding	noncoding	noncoding
MSTRG.44903.1		XLOC_051724	309 coding	noncoding	noncoding	noncoding
MSTRG.44911.1		XLOC_051726	234 coding	coding	noncoding	noncoding
MSTRG.44912.1		XLOC_051729	224 coding	noncoding	noncoding	noncoding
MSTRG.44961.1	POC1A	XLOC_051750	253 coding	noncoding	noncoding	noncoding
MSTRG.45131.1	SFMBT1	XLOC_051809	317 coding	noncoding	coding	noncoding
MSTRG.45158.1		XLOC_051826	248 coding	noncoding	noncoding	noncoding
MSTRG.45183.1		XLOC_051869	254 coding	noncoding	noncoding	noncoding
MSTRG.45189.1	LOC105377095	XLOC_051873	303 coding	noncoding	noncoding	noncoding
MSTRG.45190.1	LOC105377095	XLOC_051874	265 coding	noncoding	noncoding	noncoding
MSTRG.45286.1	CACNA2D3	XLOC_051886	300 coding	coding	noncoding	noncoding
MSTRG.45292.1	CACNA2D3	XLOC_051891	233 coding	noncoding	noncoding	noncoding
MSTRG.45253.1		XLOC_051907	280 coding	noncoding	noncoding	noncoding
MSTRG.45254.1		XLOC_051908	237 coding	noncoding	noncoding	noncoding
MSTRG.45267.1		XLOC_051915	245 coding	noncoding	noncoding	noncoding
MSTRG.45356.1	ERC2	XLOC_051938	261 coding	noncoding	noncoding	noncoding

MSTRG.45358.1	ERC2	XLOC_051939	201 coding	noncoding	noncoding	noncoding
MSTRG.45364.1	ERC2	XLOC_051942	391 coding	noncoding	noncoding	noncoding
MSTRG.45382.1		XLOC_051952	333 coding	noncoding	noncoding	noncoding
MSTRG.45419.1	ARHGEF3	XLOC_051976	246 coding	noncoding	noncoding	noncoding
MSTRG.45424.1	ARHGEF3	XLOC_051981	307 coding	noncoding	noncoding	noncoding
MSTRG.45425.1	ARHGEF3	XLOC_051982	291 coding	noncoding	noncoding	noncoding
MSTRG.45426.1	ARHGEF3	XLOC_051983	242 coding	noncoding	noncoding	noncoding
MSTRG.45432.1	ARHGEF3	XLOC_051986	227 coding	noncoding	noncoding	noncoding
XM_005265238.4	IL17RD	XLOC_051988	8955 coding	coding	coding	coding
XM_005265526.4	HESX1	XLOC_051991	2375 coding	coding	noncoding	coding
MSTRG.45451.1	DNAH12	XLOC_051995	329 coding	noncoding	noncoding	noncoding
XM_006713020.2	DENND6A	XLOC_052008	4890 coding	coding	coding	coding
MSTRG.45495.1		XLOC_052017	293 coding	noncoding	noncoding	noncoding
MSTRG.45505.1	DNASE1L3	XLOC_052023	309 coding	noncoding	noncoding	noncoding
MSTRG.45544.1		XLOC_052036	633 coding	noncoding	noncoding	noncoding
MSTRG.45546.1		XLOC_052038	350 coding	noncoding	noncoding	noncoding
MSTRG.45581.1	LOC105377109	XLOC_052053	286 coding	noncoding	noncoding	noncoding
MSTRG.47058.1	FHIT	XLOC_052100	284 coding	noncoding	noncoding	noncoding
MSTRG.47096.1	FHIT	XLOC_052121	317 coding	noncoding	noncoding	noncoding
MSTRG.47196.1	FHIT	XLOC_052135	356 coding	noncoding	noncoding	noncoding
MSTRG.47209.1	FHIT	XLOC_052144	374 coding	noncoding	noncoding	noncoding
MSTRG.47228.1	FHIT	XLOC_052158	251 coding	noncoding	noncoding	noncoding
MSTRG.47231.1	FHIT	XLOC_052159	267 coding	noncoding	noncoding	noncoding
MSTRG.45611.1		XLOC_052163	299 coding	noncoding	noncoding	noncoding
MSTRG.45630.1	PTPRG	XLOC_052164	319 coding	noncoding	noncoding	noncoding
MSTRG.45640.1	PTPRG	XLOC_052169	325 coding	coding	coding	noncoding
MSTRG.45668.1	PTPRG	XLOC_052184	287 coding	noncoding	noncoding	noncoding
NR_038281.1	PTPRG-AS1	XLOC_052185	2682 coding	noncoding	noncoding	noncoding
MSTRG.45740.1	CADPS	XLOC_052198	237 coding	noncoding	noncoding	noncoding
MSTRG.45625.1		XLOC_052205	312 coding	noncoding	noncoding	noncoding
MSTRG.45676.1		XLOC_052208	247 coding	noncoding	noncoding	noncoding
MSTRG.45698.1	SYNPR	XLOC_052210	216 coding	noncoding	noncoding	noncoding
MSTRG.45710.1	SYNPR	XLOC_052216	208 coding	noncoding	noncoding	noncoding
MSTRG.45711.1	SYNPR	XLOC_052217	306 coding	noncoding	noncoding	noncoding
MSTRG.45715.1	SYNPR	XLOC_052219	289 coding	noncoding	noncoding	noncoding
MSTRG.45679.1		XLOC_052225	264 coding	noncoding	noncoding	noncoding
XR_001740729.1	LOC100130345	XLOC_052230	7230 coding	noncoding	coding	noncoding
NM_001271779.1	PSMD6	XLOC_052236	1635 coding	coding	coding	coding
MSTRG.45757.1	PRICKLE2-AS1	XLOC_052241	277 coding	noncoding	noncoding	noncoding
MSTRG.45778.1	PRICKLE2	XLOC_052252	207 coding	noncoding	noncoding	noncoding
MSTRG.45791.1	ADAMTS9	XLOC_052257	202 coding	noncoding	noncoding	noncoding
MSTRG.45754.1		XLOC_052262	295 coding	noncoding	noncoding	noncoding
NM_001365903.2	MAGI1	XLOC_052274	8030 coding	coding	coding	coding

MSTRG.45836.1	MAGI1	XLOC_052282	254	coding	noncoding	noncoding	noncoding
MSTRG.45859.1	MAGI1	XLOC_052292	327	coding	noncoding	noncoding	noncoding
MSTRG.45885.1	LOC105377141	XLOC_052312	287	coding	noncoding	noncoding	noncoding
MSTRG.46280.1	SUCLG2-AS1	XLOC_052396	294	coding	noncoding	noncoding	noncoding
MSTRG.45944.1		XLOC_052448	268	coding	noncoding	noncoding	noncoding
MSTRG.45964.1	TMF1	XLOC_052461	270	coding	noncoding	noncoding	noncoding
MSTRG.46036.1	FRMD4B	XLOC_052483	266	coding	noncoding	noncoding	noncoding
MSTRG.46009.1		XLOC_052503	210	coding	noncoding	noncoding	noncoding
MSTRG.47678.1		XLOC_052538	377	coding	noncoding	noncoding	noncoding
MSTRG.47702.2	PROK2	XLOC_052555	2322	coding	coding	noncoding	noncoding
MSTRG.46146.1		XLOC_052560	217	coding	noncoding	noncoding	noncoding
MSTRG.46330.1	LINC00877	XLOC_052596	235	coding	noncoding	noncoding	noncoding
MSTRG.46300.1		XLOC_052605	274	coding	noncoding	noncoding	noncoding
MSTRG.46360.1		XLOC_052643	288	coding	noncoding	noncoding	noncoding
MSTRG.46373.1	LOC107986098	XLOC_052650	276	coding	noncoding	noncoding	noncoding
MSTRG.46408.1	LINC02005	XLOC_052673	301	coding	noncoding	noncoding	noncoding
MSTRG.46449.1		XLOC_052688	240	coding	noncoding	noncoding	noncoding
MSTRG.46502.1		XLOC_052711	257	coding	noncoding	noncoding	noncoding
MSTRG.46506.1		XLOC_052714	235	coding	noncoding	noncoding	noncoding
MSTRG.46616.1	ROBO2	XLOC_052737	249	coding	noncoding	noncoding	noncoding
MSTRG.46549.1		XLOC_052780	255	coding	noncoding	noncoding	noncoding
MSTRG.46551.1		XLOC_052782	203	coding	noncoding	noncoding	noncoding
MSTRG.46552.1		XLOC_052783	246	coding	noncoding	noncoding	noncoding
MSTRG.46719.1	ROBO1	XLOC_052800	230	coding	noncoding	noncoding	noncoding
MSTRG.46733.1	ROBO1	XLOC_052806	310	coding	noncoding	coding	noncoding
MSTRG.46663.1		XLOC_052815	289	coding	noncoding	noncoding	noncoding
MSTRG.46749.1	LOC105377177	XLOC_052821	270	coding	noncoding	noncoding	noncoding
MSTRG.46759.1	LOC105377177	XLOC_052824	273	coding	noncoding	noncoding	noncoding
MSTRG.46760.1	LOC105377177	XLOC_052825	312	coding	noncoding	noncoding	noncoding
MSTRG.46773.1	LINC02027	XLOC_052833	321	coding	noncoding	noncoding	noncoding
MSTRG.46788.1		XLOC_052843	308	coding	noncoding	noncoding	noncoding
MSTRG.46808.1	LINC02008	XLOC_052860	273	coding	noncoding	noncoding	noncoding
MSTRG.46839.1		XLOC_052878	211	coding	noncoding	noncoding	noncoding
MSTRG.46848.1		XLOC_052886	263	coding	noncoding	noncoding	noncoding
MSTRG.46899.1	CADM2	XLOC_052897	221	coding	noncoding	noncoding	noncoding
MSTRG.46910.1	CADM2	XLOC_052903	247	coding	noncoding	noncoding	noncoding
MSTRG.46920.1	CADM2	XLOC_052908	298	coding	noncoding	noncoding	noncoding
MSTRG.46871.1		XLOC_052911	284	coding	noncoding	noncoding	noncoding
MSTRG.46873.1		XLOC_052912	365	coding	noncoding	noncoding	noncoding
MSTRG.46881.1	LOC102723364	XLOC_052914	227	coding	noncoding	noncoding	noncoding
MSTRG.46890.1		XLOC_052920	366	coding	coding	noncoding	noncoding
MSTRG.46933.1		XLOC_052925	214	coding	noncoding	noncoding	noncoding
MSTRG.46985.1		XLOC_052945	253	coding	coding	noncoding	noncoding

MSTRG.46988.1		XLOC_052947	269	coding	noncoding	noncoding	noncoding
MSTRG.47272.1		XLOC_052986	233	coding	noncoding	noncoding	noncoding
MSTRG.47275.1		XLOC_052988	277	coding	coding	noncoding	noncoding
MSTRG.47308.1	EPHA3	XLOC_052998	277	coding	noncoding	noncoding	noncoding
MSTRG.47311.1	EPHA3	XLOC_052999	263	coding	noncoding	noncoding	noncoding
MSTRG.47293.1		XLOC_053008	218	coding	noncoding	noncoding	noncoding
MSTRG.47390.1	PROS1	XLOC_053034	391	coding	noncoding	noncoding	noncoding
MSTRG.47460.3	DHFR2	XLOC_053046	8863	coding	coding	coding	coding
MSTRG.47397.1		XLOC_053051	224	coding	noncoding	noncoding	noncoding
MSTRG.47416.1		XLOC_053060	230	coding	noncoding	noncoding	noncoding
MSTRG.47431.1		XLOC_053070	292	coding	coding	noncoding	noncoding
MSTRG.47480.1		XLOC_053076	288	coding	noncoding	coding	noncoding
NM_001105580.2	GABRR3	XLOC_053120	1521	coding	coding	coding	coding
MSTRG.47559.1	LOC101929320	XLOC_053128	287	coding	noncoding	noncoding	noncoding
MSTRG.47566.1		XLOC_053133	204	coding	noncoding	noncoding	noncoding
MSTRG.47569.1		XLOC_053136	278	coding	noncoding	noncoding	noncoding
MSTRG.47577.1		XLOC_053140	249	coding	noncoding	noncoding	noncoding
MSTRG.47607.1		XLOC_053148	302	coding	noncoding	noncoding	noncoding
MSTRG.47609.1		XLOC_053149	224	coding	noncoding	noncoding	noncoding
MSTRG.47789.9	TBC1D23	XLOC_053190	1080	coding	coding	noncoding	noncoding
MSTRG.47784.1	ADGRG7	XLOC_053214	254	coding	noncoding	noncoding	noncoding
MSTRG.47826.1		XLOC_053218	277	coding	noncoding	noncoding	noncoding
NM_016247.4	IMPG2	XLOC_053222	8352	coding	coding	coding	coding
MSTRG.47963.1		XLOC_053306	276	coding	noncoding	noncoding	noncoding
MSTRG.47988.1		XLOC_053322	269	coding	noncoding	noncoding	noncoding
MSTRG.48073.1		XLOC_053328	282	coding	noncoding	noncoding	noncoding
MSTRG.48091.1		XLOC_053334	255	coding	noncoding	noncoding	noncoding
MSTRG.48081.1		XLOC_053335	451	coding	noncoding	noncoding	noncoding
MSTRG.48083.1		XLOC_053339	213	coding	noncoding	noncoding	noncoding
MSTRG.48085.1		XLOC_053342	276	coding	noncoding	noncoding	noncoding
MSTRG.48101.1		XLOC_053349	262	coding	coding	noncoding	noncoding
MSTRG.48107.1	LOC105374019	XLOC_053352	282	coding	noncoding	noncoding	noncoding
XR_924302.2	LOC105374021	XLOC_053358	3841	coding	noncoding	noncoding	noncoding
MSTRG.48167.1		XLOC_053434	289	coding	noncoding	noncoding	noncoding
MSTRG.48196.1		XLOC_053446	217	coding	noncoding	noncoding	noncoding
MSTRG.48214.1	LOC101929485	XLOC_053458	212	coding	noncoding	noncoding	noncoding
MSTRG.48216.1	LOC101929485	XLOC_053459	247	coding	noncoding	noncoding	noncoding
MSTRG.48217.1	LOC101929485	XLOC_053460	381	coding	noncoding	noncoding	noncoding
MSTRG.48220.1	LOC101929485	XLOC_053461	621	coding	noncoding	noncoding	noncoding
MSTRG.48208.1		XLOC_053464	268	coding	noncoding	noncoding	noncoding
MSTRG.48221.1		XLOC_053466	401	coding	coding	noncoding	noncoding
MSTRG.48227.1		XLOC_053468	375	coding	noncoding	noncoding	noncoding
MSTRG.48249.1		XLOC_053480	202	coding	noncoding	noncoding	noncoding

MSTRG.48624.1		XLOC_053524	686	coding	noncoding	noncoding	noncoding
MSTRG.48331.1		XLOC_053533	236	coding	noncoding	noncoding	noncoding
XM_011512691.1	MORC1	XLOC_053562	3812	coding	coding	coding	coding
XM_011512444.3	DPPA2	XLOC_053568	1402	coding	coding	noncoding	coding
MSTRG.48382.1	LINC01205	XLOC_053573	324	coding	noncoding	noncoding	noncoding
MSTRG.48384.1		XLOC_053574	206	coding	noncoding	noncoding	noncoding
MSTRG.48436.1		XLOC_053585	202	coding	noncoding	noncoding	noncoding
MSTRG.48448.1		XLOC_053595	301	coding	noncoding	coding	noncoding
MSTRG.48461.1		XLOC_053609	270	coding	noncoding	noncoding	noncoding
MSTRG.48548.1		XLOC_053618	303	coding	noncoding	noncoding	noncoding
MSTRG.48555.1		XLOC_053640	298	coding	coding	noncoding	noncoding
MSTRG.48776.1		XLOC_053735	317	coding	coding	noncoding	noncoding
MSTRG.48888.4	SPICE1	XLOC_053766	777	coding	coding	coding	noncoding
MSTRG.48843.1		XLOC_053777	220	coding	noncoding	noncoding	noncoding
MSTRG.48932.1	GRAMD1C	XLOC_053789	492	coding	noncoding	noncoding	noncoding
MSTRG.50085.1	ZBTB20	XLOC_053916	268	coding	noncoding	noncoding	noncoding
NM_001318915.2	LSAMP	XLOC_054037	9485	coding	coding	coding	coding
MSTRG.49039.1		XLOC_054055	222	coding	noncoding	noncoding	noncoding
MSTRG.49086.1		XLOC_054063	271	coding	coding	noncoding	noncoding
MSTRG.49136.1		XLOC_054084	281	coding	noncoding	noncoding	noncoding
MSTRG.49150.1		XLOC_054091	229	coding	coding	noncoding	noncoding
MSTRG.49154.1		XLOC_054106	282	coding	noncoding	noncoding	noncoding
MSTRG.49209.1	IGSF11	XLOC_054114	254	coding	noncoding	noncoding	noncoding
MSTRG.49301.1	GPR156	XLOC_054219	219	coding	noncoding	noncoding	noncoding
MSTRG.49302.1	GPR156	XLOC_054220	277	coding	noncoding	noncoding	noncoding
MSTRG.49429.1	STXBP5L	XLOC_054249	308	coding	noncoding	noncoding	noncoding
MSTRG.49432.1	STXBP5L	XLOC_054251	395	coding	noncoding	noncoding	noncoding
MSTRG.49434.1	STXBP5L	XLOC_054252	258	coding	noncoding	noncoding	noncoding
MSTRG.49437.1	STXBP5L	XLOC_054253	280	coding	coding	noncoding	noncoding
MSTRG.49439.1	STXBP5L	XLOC_054255	205	coding	noncoding	noncoding	noncoding
MSTRG.49448.1	ARGFX	XLOC_054261	288	coding	noncoding	noncoding	noncoding
MSTRG.49463.1	FBXO40	XLOC_054263	225	coding	noncoding	noncoding	noncoding
XM_017005697.2	CCDC58	XLOC_054316	487	coding	coding	noncoding	coding
MSTRG.49606.3	PARP14	XLOC_054328	7885	coding	coding	coding	noncoding
MSTRG.49756.3	UMPS	XLOC_054415	1472	coding	coding	noncoding	noncoding
MSTRG.49768.1	ITGB5	XLOC_054422	230	coding	noncoding	noncoding	noncoding
MSTRG.49762.1	MUC13	XLOC_054427	291	coding	noncoding	noncoding	noncoding
NM_024628.5	SLC12A8	XLOC_054432	3510	coding	coding	coding	coding
MSTRG.49803.1	SNX4	XLOC_054476	306	coding	noncoding	noncoding	noncoding
MSTRG.49848.1		XLOC_054489	255	coding	noncoding	noncoding	noncoding
MSTRG.49855.1		XLOC_054492	220	coding	noncoding	noncoding	noncoding
MSTRG.49859.1		XLOC_054495	284	coding	noncoding	noncoding	noncoding
MSTRG.49870.1		XLOC_054496	296	coding	coding	noncoding	noncoding

XR_001740875.2	LOC102723696	XLOC_054499	1723 coding	noncoding	noncoding	noncoding
MSTRG.50226.1	CHCHD6	XLOC_054549	255 coding	noncoding	noncoding	noncoding
MSTRG.50228.1	CHCHD6	XLOC_054551	278 coding	coding	noncoding	noncoding
MSTRG.50184.1	PLXNA1	XLOC_054588	245 coding	coding	noncoding	noncoding
MSTRG.50395.1	RUVBL1	XLOC_054651	220 coding	noncoding	noncoding	noncoding
MSTRG.50339.1		XLOC_054701	256 coding	noncoding	noncoding	noncoding
MSTRG.50351.1	RPN1	XLOC_054708	317 coding	noncoding	noncoding	noncoding
MSTRG.50352.1		XLOC_054711	281 coding	noncoding	noncoding	noncoding
MSTRG.50452.1		XLOC_054733	248 coding	noncoding	noncoding	noncoding
MSTRG.50471.1	CNBP	XLOC_054736	492 coding	noncoding	noncoding	noncoding
MSTRG.50503.1		XLOC_054752	279 coding	noncoding	noncoding	noncoding
MSTRG.50529.1		XLOC_054768	390 coding	noncoding	noncoding	noncoding
MSTRG.50602.1	TMCC1	XLOC_054772	225 coding	noncoding	noncoding	noncoding
MSTRG.50604.1	TMCC1	XLOC_054773	366 coding	noncoding	noncoding	noncoding
MSTRG.50583.1		XLOC_054839	345 coding	noncoding	noncoding	noncoding
MSTRG.50593.1	COL6A5	XLOC_054841	206 coding	coding	noncoding	noncoding
MSTRG.50847.1	LOC339874	XLOC_054886	296 coding	noncoding	noncoding	noncoding
NM_130808.2	CPNE4	XLOC_054903	4186 coding	coding	coding	coding
MSTRG.50718.1	CPNE4	XLOC_054908	247 coding	noncoding	noncoding	noncoding
MSTRG.50721.1	CPNE4	XLOC_054909	218 coding	noncoding	noncoding	noncoding
MSTRG.50751.1	CPNE4	XLOC_054919	264 coding	noncoding	noncoding	noncoding
MSTRG.50928.1	NPHP3-AS1	XLOC_054951	219 coding	noncoding	noncoding	noncoding
MSTRG.50879.1	TMEM108	XLOC_054957	288 coding	noncoding	noncoding	noncoding
MSTRG.50931.6	CDV3	XLOC_054967	2561 coding	coding	coding	noncoding
MSTRG.50931.10	CDV3	XLOC_054967	2540 coding	coding	coding	noncoding
MSTRG.50960.1	TOPBP1	XLOC_054971	352 coding	noncoding	noncoding	noncoding
MSTRG.50948.1		XLOC_054991	269 coding	noncoding	noncoding	noncoding
MSTRG.51661.40	KY	XLOC_055016	8034 coding	noncoding	noncoding	noncoding
XR_001739978.1	STAG1	XLOC_055052	2816 coding	coding	coding	coding
MSTRG.51129.1	STAG1	XLOC_055062	248 coding	noncoding	noncoding	noncoding
MSTRG.51138.1	STAG1	XLOC_055070	260 coding	noncoding	noncoding	noncoding
MSTRG.51049.1		XLOC_055076	300 coding	noncoding	noncoding	noncoding
MSTRG.51069.1		XLOC_055100	365 coding	noncoding	noncoding	noncoding
MSTRG.51083.1		XLOC_055110	315 coding	noncoding	noncoding	noncoding
MSTRG.51096.1	LOC105374126	XLOC_055115	340 coding	noncoding	noncoding	noncoding
MSTRG.51104.1		XLOC_055120	216 coding	noncoding	noncoding	noncoding
XR_001740930.1	LOC105374128	XLOC_055125	4212 coding	noncoding	noncoding	noncoding
MSTRG.51215.5	ARMC8	XLOC_055132	2739 coding	coding	coding	coding
MSTRG.51207.1		XLOC_055142	232 coding	noncoding	noncoding	noncoding
MSTRG.51273.1		XLOC_055150	249 coding	noncoding	noncoding	noncoding
MSTRG.51285.1	PIK3CB	XLOC_055160	321 coding	coding	noncoding	noncoding
MSTRG.51244.1		XLOC_055161	241 coding	noncoding	noncoding	noncoding
MSTRG.51250.1		XLOC_055169	355 coding	noncoding	noncoding	noncoding

MSTRG.51252.1		XLOC_055173	287	coding	noncoding	noncoding	noncoding
MSTRG.51262.1		XLOC_055178	276	coding	noncoding	noncoding	noncoding
MSTRG.51263.1		XLOC_055179	279	coding	noncoding	noncoding	noncoding
XM_024453831.1	COPB2	XLOC_055183	4924	coding	coding	coding	coding
MSTRG.51398.1	LOC100507291	XLOC_055188	253	coding	noncoding	noncoding	noncoding
MSTRG.51289.1		XLOC_055230	282	coding	noncoding	noncoding	noncoding
MSTRG.51319.1	CLSTN2	XLOC_055238	267	coding	noncoding	noncoding	noncoding
MSTRG.51342.1		XLOC_055254	285	coding	noncoding	noncoding	noncoding
MSTRG.51349.1	SPSB4	XLOC_055259	282	coding	noncoding	noncoding	noncoding
MSTRG.51448.1	LINC02618	XLOC_055275	313	coding	noncoding	noncoding	noncoding
XM_024453725.1	TFDP2	XLOC_055286	9730	coding	coding	coding	coding
MSTRG.51528.1	TFDP2	XLOC_055287	232	coding	noncoding	noncoding	noncoding
MSTRG.51529.1	TFDP2	XLOC_055288	204	coding	noncoding	noncoding	noncoding
MSTRG.51619.1	XRN1	XLOC_055306	239	coding	noncoding	noncoding	noncoding
MSTRG.51643.1	ATR	XLOC_055328	229	coding	noncoding	noncoding	noncoding
MSTRG.51570.1		XLOC_055360	215	coding	noncoding	noncoding	noncoding
NM_198504.3	PAQR9	XLOC_055363	2437	coding	coding	coding	coding
MSTRG.51600.1	LOC105374138	XLOC_055374	301	coding	noncoding	noncoding	noncoding
MSTRG.51649.1		XLOC_055428	291	coding	noncoding	noncoding	noncoding
MSTRG.51870.1	LOC105374140	XLOC_055485	245	coding	noncoding	noncoding	noncoding
MSTRG.51741.1		XLOC_055495	422	coding	coding	noncoding	noncoding
MSTRG.51753.1		XLOC_055500	278	coding	coding	noncoding	noncoding
MSTRG.51755.1		XLOC_055502	307	coding	coding	noncoding	noncoding
MSTRG.51756.1		XLOC_055503	309	coding	noncoding	noncoding	noncoding
MSTRG.51800.1		XLOC_055520	275	coding	noncoding	noncoding	noncoding
XR_001740953.1	LOC105374145	XLOC_055524	7278	coding	noncoding	noncoding	noncoding
XM_017006629.1	PLSCR1	XLOC_055538	2272	coding	coding	noncoding	coding
MSTRG.51923.1	ZIC1	XLOC_055557	266	coding	noncoding	noncoding	noncoding
MSTRG.52024.1	HLTF-AS1	XLOC_055604	280	coding	noncoding	noncoding	noncoding
MSTRG.52027.1		XLOC_055608	251	coding	noncoding	noncoding	noncoding
MSTRG.52029.1		XLOC_055610	286	coding	noncoding	noncoding	noncoding
MSTRG.52089.1		XLOC_055638	238	coding	noncoding	noncoding	noncoding
NM_005067.7	SIAH2	XLOC_055668	2311	coding	coding	coding	coding
MSTRG.52227.1		XLOC_055670	209	coding	noncoding	noncoding	noncoding
MSTRG.52228.1		XLOC_055671	356	coding	noncoding	noncoding	noncoding
NM_001256819.2	CLRN1	XLOC_055685	2259	coding	noncoding	coding	coding
MSTRG.52267.1	MED12L	XLOC_055700	260	coding	noncoding	noncoding	noncoding
XM_005247922.3	P2RY14	XLOC_055702	2469	coding	coding	coding	coding
XM_011513340.3	P2RY14	XLOC_055702	3107	coding	coding	coding	coding
MSTRG.52284.1	MED12L	XLOC_055710	356	coding	coding	noncoding	noncoding
MSTRG.52287.1	P2RY13	XLOC_055712	2315	coding	coding	coding	coding
MSTRG.52295.1	MED12L	XLOC_055717	389	coding	noncoding	noncoding	noncoding
MSTRG.52313.1	IGSF10	XLOC_055725	254	coding	noncoding	noncoding	noncoding

MSTRG.52316.1	IGSF10	XLOC_055726	233 coding	noncoding	noncoding	noncoding
MSTRG.52322.1	IGSF10	XLOC_055728	245 coding	noncoding	noncoding	noncoding
MSTRG.52762.26	TMEM14EP	XLOC_055746	15069 coding	coding	noncoding	noncoding
MSTRG.52336.1		XLOC_055752	248 coding	noncoding	noncoding	noncoding
MSTRG.52345.1		XLOC_055758	233 coding	noncoding	noncoding	noncoding
MSTRG.52350.1		XLOC_055761	288 coding	noncoding	noncoding	noncoding
MSTRG.52362.1		XLOC_055766	276 coding	noncoding	noncoding	noncoding
MSTRG.52364.1		XLOC_055768	271 coding	noncoding	noncoding	noncoding
MSTRG.52469.1	LINC02006	XLOC_055785	244 coding	noncoding	noncoding	noncoding
MSTRG.52474.1	C3orf79	XLOC_055787	308 coding	noncoding	noncoding	noncoding
MSTRG.52515.1	ARHGEF26-AS1	XLOC_055797	268 coding	noncoding	noncoding	noncoding
NM_001038705.3	GPR149	XLOC_055810	5527 coding	noncoding	coding	coding
MSTRG.52498.1		XLOC_055815	287 coding	noncoding	noncoding	noncoding
MSTRG.52501.1		XLOC_055817	217 coding	noncoding	noncoding	noncoding
XM_011512562.3	PLCH1	XLOC_055843	7254 coding	coding	coding	coding
MSTRG.52641.1	PLCH1	XLOC_055844	276 coding	noncoding	noncoding	noncoding
MSTRG.52653.1	PLCH1	XLOC_055847	234 coding	noncoding	noncoding	noncoding
MSTRG.52656.1		XLOC_055854	278 coding	noncoding	noncoding	noncoding
MSTRG.52666.1		XLOC_055862	300 coding	noncoding	noncoding	noncoding
MSTRG.52704.1	SSR3	XLOC_055881	266 coding	noncoding	noncoding	noncoding
MSTRG.52841.1	LEKR1	XLOC_055904	448 coding	noncoding	noncoding	noncoding
MSTRG.53070.1	IQCJ	XLOC_056021	387 coding	noncoding	noncoding	noncoding
MSTRG.53091.1	SCHIP1	XLOC_056034	320 coding	coding	noncoding	noncoding
MSTRG.53098.1	SCHIP1	XLOC_056040	244 coding	noncoding	noncoding	noncoding
MSTRG.53100.1	SCHIP1	XLOC_056041	321 coding	noncoding	noncoding	noncoding
MSTRG.53121.1		XLOC_056091	294 coding	noncoding	noncoding	noncoding
MSTRG.53145.1		XLOC_056093	293 coding	noncoding	noncoding	noncoding
MSTRG.53218.1		XLOC_056122	322 coding	noncoding	noncoding	noncoding
MSTRG.53223.1		XLOC_056123	269 coding	noncoding	noncoding	noncoding
MSTRG.53226.1		XLOC_056125	614 coding	noncoding	noncoding	noncoding
MSTRG.53227.1		XLOC_056126	258 coding	noncoding	noncoding	noncoding
MSTRG.53254.1	LINC01192	XLOC_056140	274 coding	coding	noncoding	noncoding
MSTRG.53259.1	LOC105374188	XLOC_056142	291 coding	noncoding	noncoding	noncoding
MSTRG.53269.1		XLOC_056149	309 coding	noncoding	noncoding	noncoding
MSTRG.53293.1		XLOC_056162	309 coding	coding	noncoding	noncoding
MSTRG.53296.1	LOC105374189	XLOC_056163	253 coding	noncoding	noncoding	noncoding
MSTRG.53326.1		XLOC_056184	248 coding	noncoding	noncoding	noncoding
MSTRG.53327.1		XLOC_056185	324 coding	noncoding	noncoding	noncoding
MSTRG.53352.1		XLOC_056196	272 coding	noncoding	noncoding	noncoding
MSTRG.53359.1		XLOC_056202	470 coding	noncoding	noncoding	noncoding
MSTRG.53367.1		XLOC_056207	229 coding	noncoding	noncoding	noncoding
MSTRG.53370.1		XLOC_056208	313 coding	noncoding	noncoding	noncoding
MSTRG.53378.1		XLOC_056209	236 coding	noncoding	noncoding	noncoding

MSTRG.53380.1		XLOC_056210	231 coding	noncoding	noncoding	noncoding
MSTRG.53466.1	WDR49	XLOC_056232	528 coding	noncoding	noncoding	noncoding
MSTRG.53484.1	WDR49	XLOC_056238	302 coding	noncoding	noncoding	noncoding
MSTRG.53496.1	WDR49	XLOC_056242	497 coding	noncoding	noncoding	noncoding
MSTRG.53504.1	LRRC77P	XLOC_056277	371 coding	noncoding	noncoding	noncoding
MSTRG.53506.1		XLOC_056286	292 coding	noncoding	noncoding	noncoding
XR_001741014.1	LOC105374200	XLOC_056296	11183 coding	noncoding	noncoding	noncoding
MSTRG.53613.1	MECOM	XLOC_056304	257 coding	noncoding	noncoding	noncoding
MSTRG.53624.1	MECOM	XLOC_056308	307 coding	coding	noncoding	noncoding
MSTRG.53602.1		XLOC_056314	232 coding	noncoding	noncoding	noncoding
MSTRG.53656.8	LOC100128164	XLOC_056323	4667 coding	coding	noncoding	noncoding
MSTRG.53644.1		XLOC_056325	306 coding	noncoding	noncoding	noncoding
MSTRG.53667.1		XLOC_056330	236 coding	noncoding	noncoding	noncoding
NM_020949.3	SLC7A14	XLOC_056331	10140 coding	coding	coding	coding
MSTRG.53686.1	SLC7A14-AS1	XLOC_056334	272 coding	noncoding	noncoding	noncoding
MSTRG.53675.1	LOC105374211	XLOC_056338	265 coding	noncoding	noncoding	noncoding
MSTRG.53716.1		XLOC_056342	363 coding	noncoding	noncoding	noncoding
MSTRG.53922.8	FNDC3B	XLOC_056415	1585 coding	coding	noncoding	noncoding
MSTRG.53760.1		XLOC_056420	281 coding	noncoding	noncoding	noncoding
MSTRG.53874.1	SPATA16	XLOC_056458	519 coding	noncoding	noncoding	noncoding
MSTRG.53908.1	NLGN1	XLOC_056472	378 coding	noncoding	noncoding	noncoding
MSTRG.54054.1	NAALADL2	XLOC_056510	238 coding	noncoding	noncoding	noncoding
MSTRG.53982.1		XLOC_056533	760 coding	coding	noncoding	noncoding
MSTRG.53987.1		XLOC_056536	273 coding	noncoding	noncoding	noncoding
MSTRG.53988.1		XLOC_056537	240 coding	noncoding	noncoding	noncoding
MSTRG.54068.1		XLOC_056544	286 coding	noncoding	noncoding	noncoding
MSTRG.54069.1		XLOC_056545	276 coding	noncoding	noncoding	noncoding
MSTRG.54103.1	LINC00578	XLOC_056554	293 coding	noncoding	noncoding	noncoding
MSTRG.54115.1	LINC00578	XLOC_056560	265 coding	noncoding	noncoding	noncoding
MSTRG.54096.1	LINC02015	XLOC_056570	284 coding	noncoding	noncoding	noncoding
XR_924740.2	LOC105374235	XLOC_056616	11782 coding	coding	coding	noncoding
NR_126560.1	KCNMB2-AS1	XLOC_056620	2055 coding	noncoding	noncoding	noncoding
MSTRG.54177.1		XLOC_056625	257 coding	noncoding	noncoding	noncoding
MSTRG.54201.1		XLOC_056626	200 coding	noncoding	noncoding	noncoding
XM_011513073.2	ZMAT3	XLOC_056633	9005 coding	coding	coding	coding
MSTRG.54321.1		XLOC_056679	275 coding	noncoding	noncoding	noncoding
MSTRG.54328.1		XLOC_056683	281 coding	noncoding	noncoding	noncoding
MSTRG.54333.1		XLOC_056685	258 coding	noncoding	noncoding	noncoding
MSTRG.54354.1	LOC101928882	XLOC_056693	298 coding	noncoding	noncoding	noncoding
MSTRG.54355.1	LOC101928882	XLOC_056694	242 coding	noncoding	noncoding	noncoding
MSTRG.54373.11	FXR1	XLOC_056698	2298 coding	coding	noncoding	noncoding
MSTRG.54431.1	SOX2-OT	XLOC_056702	275 coding	noncoding	noncoding	noncoding
MSTRG.54472.1	SOX2-OT	XLOC_056733	258 coding	noncoding	noncoding	noncoding

MSTRG.54390.1		XLOC_056741	264 coding	noncoding	noncoding	noncoding
MSTRG.54402.1		XLOC_056747	222 coding	noncoding	noncoding	noncoding
MSTRG.54424.1	LINC01994	XLOC_056761	275 coding	noncoding	noncoding	noncoding
MSTRG.54496.1		XLOC_056767	257 coding	noncoding	noncoding	noncoding
MSTRG.54519.1	LOC105374245	XLOC_056772	249 coding	noncoding	noncoding	noncoding
MSTRG.54512.1	LAMP3	XLOC_056787	249 coding	noncoding	noncoding	noncoding
MSTRG.54515.1	LAMP3	XLOC_056788	234 coding	noncoding	noncoding	noncoding
MSTRG.54508.1		XLOC_056790	218 coding	noncoding	noncoding	noncoding
XM_011513274.3	KLHL6	XLOC_056796	2635 coding	coding	coding	coding
MSTRG.54568.1		XLOC_056802	294 coding	noncoding	noncoding	noncoding
MSTRG.54602.1		XLOC_056805	286 coding	noncoding	noncoding	noncoding
XM_024453628.1	PARL	XLOC_056809	1515 coding	coding	coding	coding
MSTRG.54625.18	PARL	XLOC_056809	530 coding	noncoding	noncoding	noncoding
MSTRG.54653.1	ABCC5	XLOC_056817	266 coding	noncoding	noncoding	noncoding
MSTRG.54633.1		XLOC_056818	272 coding	noncoding	noncoding	noncoding
MSTRG.54644.1	LOC105374249	XLOC_056824	228 coding	noncoding	noncoding	noncoding
MSTRG.54669.1	EEF1AKMT4-ECE2	XLOC_056834	228 coding	noncoding	noncoding	noncoding
MSTRG.54706.1	CLCN2	XLOC_056840	262 coding	noncoding	noncoding	noncoding
MSTRG.54675.1	LINC02054	XLOC_056844	514 coding	noncoding	noncoding	noncoding
MSTRG.54681.1		XLOC_056848	271 coding	noncoding	noncoding	noncoding
MSTRG.54720.1	EHHADH-AS1	XLOC_056863	306 coding	noncoding	noncoding	noncoding
MSTRG.54753.1	MAP3K13	XLOC_056879	293 coding	coding	coding	noncoding
MSTRG.54760.1	MAP3K13	XLOC_056884	288 coding	noncoding	noncoding	noncoding
MSTRG.54729.1		XLOC_056891	281 coding	noncoding	noncoding	noncoding
MSTRG.54736.1		XLOC_056893	324 coding	noncoding	noncoding	noncoding
XM_017005563.1	IGF2BP2	XLOC_056895	2383 coding	coding	coding	coding
MSTRG.54786.1	IGF2BP2	XLOC_056900	300 coding	noncoding	noncoding	noncoding
MSTRG.54779.1		XLOC_056906	450 coding	noncoding	noncoding	noncoding
MSTRG.54797.1		XLOC_056917	270 coding	noncoding	noncoding	noncoding
MSTRG.54856.1	DGKG	XLOC_056924	246 coding	noncoding	noncoding	noncoding
MSTRG.54815.1		XLOC_056938	274 coding	noncoding	noncoding	noncoding
MSTRG.54822.1	AHSG	XLOC_056949	519 coding	noncoding	noncoding	noncoding
MSTRG.54830.1	LOC105374258	XLOC_056951	216 coding	coding	noncoding	noncoding
MSTRG.54833.1	KNG1	XLOC_056952	249 coding	noncoding	noncoding	noncoding
XM_017006870.2	MASP1	XLOC_056966	4373 coding	coding	coding	coding
MSTRG.54982.1		XLOC_056971	314 coding	noncoding	noncoding	noncoding
MSTRG.55007.1		XLOC_056981	306 coding	noncoding	noncoding	noncoding
MSTRG.55051.1	LOC107986166	XLOC_057024	404 coding	noncoding	noncoding	noncoding
MSTRG.55078.1		XLOC_057043	316 coding	noncoding	noncoding	noncoding
MSTRG.55160.1	P3H2	XLOC_057128	271 coding	noncoding	noncoding	noncoding
MSTRG.55132.1		XLOC_057131	274 coding	noncoding	noncoding	noncoding
MSTRG.55135.1		XLOC_057132	261 coding	noncoding	noncoding	noncoding
NM_021101.5	CLDN1	XLOC_057134	3446 coding	coding	coding	coding

MSTRG.55244.1		XLOC_057149	267	coding	noncoding	noncoding	noncoding
MSTRG.55260.1		XLOC_057155	394	coding	coding	noncoding	noncoding
MSTRG.55300.1		XLOC_057168	267	coding	noncoding	noncoding	noncoding
MSTRG.55303.1		XLOC_057171	249	coding	noncoding	noncoding	noncoding
MSTRG.55316.1		XLOC_057176	313	coding	noncoding	noncoding	noncoding
MSTRG.55319.1		XLOC_057177	315	coding	noncoding	noncoding	noncoding
NM_021032.4	FGF12	XLOC_057182	6174	coding	coding	coding	coding
MSTRG.55362.1	FGF12	XLOC_057185	396	coding	noncoding	noncoding	noncoding
MSTRG.55367.1	FGF12	XLOC_057187	351	coding	noncoding	noncoding	noncoding
MSTRG.55377.1	FGF12	XLOC_057192	291	coding	noncoding	noncoding	noncoding
MSTRG.55379.1	FGF12	XLOC_057193	302	coding	noncoding	noncoding	noncoding
MSTRG.55386.1		XLOC_057206	280	coding	noncoding	noncoding	noncoding
MSTRG.55397.1		XLOC_057211	273	coding	noncoding	noncoding	noncoding
MSTRG.55399.1		XLOC_057212	296	coding	noncoding	noncoding	noncoding
MSTRG.55480.1	ATP13A4	XLOC_057219	255	coding	noncoding	noncoding	noncoding
MSTRG.55494.1	ATP13A4	XLOC_057222	611	coding	noncoding	noncoding	noncoding
MSTRG.55499.1	ATP13A4	XLOC_057225	435	coding	noncoding	noncoding	noncoding
MSTRG.55506.15	OPA1	XLOC_057227	3944	coding	noncoding	noncoding	noncoding
NR_027764.1	DPPA2P3	XLOC_057244	1146	coding	coding	noncoding	noncoding
MSTRG.55454.1	LINC02028	XLOC_057251	223	coding	noncoding	noncoding	noncoding
XR_001740368.1	TMEM44	XLOC_057278	4775	coding	noncoding	coding	coding
XM_017006796.1	LSG1	XLOC_057279	2900	coding	coding	coding	coding
MSTRG.55561.1	LOC105374292	XLOC_057290	267	coding	noncoding	noncoding	noncoding
MSTRG.55571.1		XLOC_057298	220	coding	noncoding	noncoding	noncoding
MSTRG.55617.1	XXYLT1	XLOC_057317	237	coding	noncoding	noncoding	noncoding
MSTRG.55620.1	XXYLT1	XLOC_057320	211	coding	noncoding	noncoding	noncoding
MSTRG.55640.1	ACAP2	XLOC_057335	238	coding	noncoding	noncoding	noncoding
MSTRG.55622.1		XLOC_057350	276	coding	noncoding	noncoding	noncoding
NM_001316325.2	PPP1R2	XLOC_057352	3531	coding	coding	coding	coding
XM_011512317.3	TNK2	XLOC_057361	4887	coding	coding	coding	coding
MSTRG.55683.1		XLOC_057366	226	coding	noncoding	noncoding	noncoding
MSTRG.55727.1	TFRC	XLOC_057372	369	coding	noncoding	noncoding	noncoding
MSTRG.55721.1	LINC00885	XLOC_057374	239	coding	noncoding	noncoding	noncoding
MSTRG.55752.1	UBXN7	XLOC_057385	256	coding	noncoding	noncoding	noncoding
MSTRG.55932.7	PIGX	XLOC_057408	860	coding	coding	noncoding	noncoding
MSTRG.55775.1		XLOC_057415	285	coding	noncoding	noncoding	noncoding
MSTRG.55904.1		XLOC_057419	309	coding	noncoding	noncoding	noncoding
MSTRG.55960.1		XLOC_057449	514	coding	noncoding	noncoding	noncoding
MSTRG.55967.1		XLOC_057453	219	coding	noncoding	noncoding	noncoding
MSTRG.55985.1		XLOC_057458	755	coding	noncoding	noncoding	noncoding
MSTRG.55986.1		XLOC_057459	229	coding	noncoding	noncoding	noncoding
MSTRG.55989.1		XLOC_057460	264	coding	noncoding	noncoding	noncoding
MSTRG.56042.11	FAM157A	XLOC_057488	1509	coding	noncoding	noncoding	noncoding

MSTRG.56042.22	FAM157A	XLOC_057488	555	coding	noncoding	noncoding	noncoding
MSTRG.56044.1		XLOC_057489	399	coding	noncoding	noncoding	noncoding
MSTRG.56103.1	ZNF141	XLOC_057510	267	coding	noncoding	noncoding	noncoding
MSTRG.56163.1	ZNF721	XLOC_057526	581	coding	noncoding	noncoding	noncoding
MSTRG.56139.1		XLOC_057534	329	coding	coding	noncoding	noncoding
MSTRG.56253.1	TMEM175	XLOC_057558	258	coding	noncoding	noncoding	noncoding
XR_924947.1	IDUA	XLOC_057566	2222	coding	coding	coding	coding
MSTRG.56261.1		XLOC_057594	215	coding	noncoding	noncoding	noncoding
XR_001741093.1	MAEA	XLOC_057596	2158	coding	coding	coding	coding
XR_001741298.1	UVSSA	XLOC_057599	3621	coding	coding	coding	coding
MSTRG.56302.1	FAM53A	XLOC_057604	237	coding	noncoding	noncoding	noncoding
MSTRG.56306.1		XLOC_057611	249	coding	noncoding	noncoding	noncoding
MSTRG.56371.1	NSD2	XLOC_057619	242	coding	noncoding	noncoding	noncoding
MSTRG.56372.1	NSD2	XLOC_057620	235	coding	noncoding	noncoding	noncoding
MSTRG.56360.1	POLN	XLOC_057636	247	coding	noncoding	noncoding	noncoding
MSTRG.56362.2	POLN	XLOC_057638	326	coding	noncoding	noncoding	noncoding
MSTRG.56441.1	CFAP99	XLOC_057676	333	coding	noncoding	noncoding	noncoding
MSTRG.56447.1	RNF4	XLOC_057679	200	coding	noncoding	noncoding	noncoding
MSTRG.56448.1		XLOC_057680	279	coding	noncoding	noncoding	noncoding
MSTRG.56451.1	FAM193A	XLOC_057683	266	coding	noncoding	noncoding	noncoding
NM_001354758.1	ADD1	XLOC_057695	4086	coding	coding	coding	coding
XM_005247934.2	ADD1	XLOC_057695	4216	coding	coding	coding	coding
MSTRG.56502.1	GRK4	XLOC_057705	265	coding	coding	noncoding	noncoding
MSTRG.56563.1	HTT	XLOC_057709	287	coding	noncoding	noncoding	noncoding
MSTRG.56564.1	HTT	XLOC_057710	282	coding	noncoding	noncoding	noncoding
MSTRG.56471.1		XLOC_057721	240	coding	noncoding	noncoding	noncoding
MSTRG.56473.1		XLOC_057733	279	coding	noncoding	noncoding	noncoding
MSTRG.56557.1		XLOC_057750	207	coding	noncoding	noncoding	noncoding
MSTRG.56620.1	ZBTB49	XLOC_057780	261	coding	noncoding	noncoding	noncoding
MSTRG.56606.1		XLOC_057783	278	coding	noncoding	noncoding	noncoding
MSTRG.56609.1		XLOC_057784	323	coding	noncoding	noncoding	noncoding
MSTRG.56607.1		XLOC_057785	225	coding	noncoding	noncoding	noncoding
MSTRG.56610.1	LOC112268462	XLOC_057787	239	coding	noncoding	noncoding	noncoding
MSTRG.56643.1		XLOC_057810	269	coding	noncoding	noncoding	noncoding
MSTRG.56644.1		XLOC_057812	250	coding	noncoding	noncoding	noncoding
MSTRG.56667.1	STK32B	XLOC_057819	240	coding	noncoding	noncoding	noncoding
MSTRG.56674.1	STK32B	XLOC_057823	211	coding	noncoding	noncoding	noncoding
MSTRG.56682.1	STK32B	XLOC_057826	277	coding	noncoding	noncoding	noncoding
MSTRG.56708.5	EVC	XLOC_057845	7022	coding	noncoding	coding	coding
MSTRG.56717.1	CRMP1	XLOC_057848	286	coding	noncoding	noncoding	noncoding
MSTRG.56725.1	C4orf50	XLOC_057852	368	coding	coding	noncoding	noncoding
MSTRG.56768.2	JAKMIP1	XLOC_057856	256	coding	coding	noncoding	noncoding
MSTRG.56728.1		XLOC_057866	250	coding	noncoding	noncoding	noncoding

NM_006005.3	WFS1	XLOC_057867	3640	coding	noncoding	coding	coding
MSTRG.56741.1	PPP2R2C	XLOC_057872	234	coding	noncoding	noncoding	noncoding
MSTRG.56756.1	PPP2R2C	XLOC_057879	276	coding	noncoding	noncoding	noncoding
MSTRG.56792.1	MAN2B2	XLOC_057885	248	coding	noncoding	noncoding	noncoding
NR_158178.1	LOC93622	XLOC_057888	1618	coding	coding	coding	noncoding
MSTRG.56952.1	TBC1D14	XLOC_057925	417	coding	noncoding	noncoding	noncoding
MSTRG.56942.1	LINC02447	XLOC_057944	276	coding	noncoding	noncoding	noncoding
MSTRG.56931.1		XLOC_057945	261	coding	noncoding	noncoding	noncoding
MSTRG.56933.1		XLOC_057946	281	coding	noncoding	noncoding	noncoding
XM_017008481.1	SORCS2	XLOC_057948	5032	coding	coding	coding	coding
MSTRG.57085.1		XLOC_058036	330	coding	noncoding	noncoding	noncoding
MSTRG.57116.1	LOC105369250	XLOC_058056	263	coding	coding	noncoding	noncoding
MSTRG.57123.1	LOC105369250	XLOC_058062	374	coding	noncoding	noncoding	noncoding
NM_001358418.1	FAM90A26	XLOC_058066	1395	coding	coding	coding	coding
NM_001256854.1	USP17L11	XLOC_058069	1593	coding	coding	coding	coding
NM_001256853.1	USP17L12	XLOC_058070	1593	coding	coding	coding	coding
NM_001256859.1	USP17L18	XLOC_058074	1593	coding	coding	coding	coding
NM_001256861.1	USP17L20	XLOC_058076	1593	coding	coding	coding	coding
NM_001256863.1	USP17L22	XLOC_058078	1593	coding	coding	coding	coding
MSTRG.57165.1		XLOC_058090	280	coding	noncoding	noncoding	noncoding
MSTRG.57193.1		XLOC_058096	251	coding	coding	coding	noncoding
MSTRG.57195.1		XLOC_058097	244	coding	noncoding	noncoding	noncoding
MSTRG.57173.1		XLOC_058100	240	coding	noncoding	noncoding	noncoding
MSTRG.57257.1		XLOC_058156	255	coding	noncoding	noncoding	noncoding
MSTRG.57451.1	LINC02360	XLOC_058183	308	coding	noncoding	noncoding	noncoding
MSTRG.57406.1	LOC105374483	XLOC_058186	200	coding	noncoding	noncoding	noncoding
MSTRG.57465.1		XLOC_058203	297	coding	noncoding	noncoding	noncoding
MSTRG.57615.1	LOC105374493	XLOC_058220	291	coding	noncoding	coding	noncoding
MSTRG.57489.1		XLOC_058288	254	coding	noncoding	noncoding	noncoding
MSTRG.57532.11	BOD1L1	XLOC_058291	12656	coding	coding	coding	coding
MSTRG.57503.1	LOC107986183	XLOC_058297	236	coding	noncoding	noncoding	noncoding
MSTRG.57506.1	LOC107986184	XLOC_058298	273	coding	noncoding	noncoding	noncoding
MSTRG.57524.1	LOC107986182	XLOC_058309	227	coding	noncoding	noncoding	noncoding
MSTRG.57536.1		XLOC_058321	307	coding	noncoding	noncoding	noncoding
MSTRG.57537.1		XLOC_058322	271	coding	coding	noncoding	noncoding
MSTRG.57597.1	LINC00504	XLOC_058346	221	coding	noncoding	noncoding	noncoding
MSTRG.58509.1	LOC101929095	XLOC_058382	283	coding	noncoding	noncoding	noncoding
MSTRG.58562.1	LOC101929095	XLOC_058427	321	coding	noncoding	noncoding	noncoding
MSTRG.58584.5	CC2D2A	XLOC_058437	12087	coding	coding	coding	coding
MSTRG.58617.1	BST1	XLOC_058443	245	coding	noncoding	noncoding	noncoding
MSTRG.57738.1	PROM1	XLOC_058460	307	coding	noncoding	noncoding	noncoding
MSTRG.57739.1		XLOC_058478	279	coding	noncoding	noncoding	noncoding
MSTRG.57743.1		XLOC_058520	264	coding	noncoding	noncoding	noncoding

MSTRG.57746.1		XLOC_058521	239 coding	noncoding	noncoding	noncoding
MSTRG.57782.1		XLOC_058524	294 coding	noncoding	noncoding	noncoding
MSTRG.57891.1	FAM184B	XLOC_058551	276 coding	noncoding	noncoding	noncoding
MSTRG.57905.1		XLOC_058564	265 coding	coding	noncoding	noncoding
MSTRG.57943.1	LOC105374510	XLOC_058578	317 coding	noncoding	noncoding	noncoding
MSTRG.57974.1	LINC02438	XLOC_058593	230 coding	noncoding	noncoding	noncoding
MSTRG.57994.1		XLOC_058604	298 coding	noncoding	noncoding	noncoding
MSTRG.57997.1		XLOC_058606	287 coding	noncoding	noncoding	noncoding
MSTRG.58009.1	SLIT2	XLOC_058610	274 coding	noncoding	noncoding	noncoding
MSTRG.58011.1	SLIT2	XLOC_058612	312 coding	noncoding	noncoding	noncoding
MSTRG.58013.1	SLIT2	XLOC_058613	229 coding	noncoding	noncoding	noncoding
MSTRG.58022.1	SLIT2	XLOC_058620	231 coding	noncoding	noncoding	noncoding
MSTRG.58023.1	SLIT2	XLOC_058621	284 coding	noncoding	noncoding	noncoding
MSTRG.58179.1	KCNIP4	XLOC_058630	235 coding	noncoding	noncoding	noncoding
MSTRG.58182.1	KCNIP4	XLOC_058633	214 coding	noncoding	noncoding	noncoding
MSTRG.58199.1	KCNIP4	XLOC_058644	265 coding	noncoding	noncoding	noncoding
MSTRG.58200.1	KCNIP4	XLOC_058645	313 coding	noncoding	noncoding	noncoding
MSTRG.58211.1	KCNIP4	XLOC_058652	217 coding	noncoding	noncoding	noncoding
MSTRG.58063.1		XLOC_058655	260 coding	noncoding	noncoding	noncoding
MSTRG.58073.1		XLOC_058660	289 coding	noncoding	noncoding	noncoding
MSTRG.58100.1	ADGRA3	XLOC_058667	295 coding	noncoding	noncoding	noncoding
MSTRG.58092.1		XLOC_058675	242 coding	noncoding	noncoding	noncoding
MSTRG.58151.1	LOC105374523	XLOC_058691	263 coding	noncoding	noncoding	noncoding
MSTRG.58130.1		XLOC_058694	285 coding	noncoding	noncoding	noncoding
MSTRG.58229.1	PPARGC1A	XLOC_058706	316 coding	noncoding	noncoding	noncoding
MSTRG.58328.1	LOC102723675	XLOC_058769	279 coding	noncoding	noncoding	noncoding
MSTRG.58375.1	PI4K2B	XLOC_058795	268 coding	noncoding	noncoding	noncoding
MSTRG.58388.1		XLOC_058814	387 coding	noncoding	coding	noncoding
MSTRG.58395.1	LOC101929161	XLOC_058817	260 coding	noncoding	noncoding	noncoding
MSTRG.58396.1		XLOC_058818	302 coding	noncoding	noncoding	noncoding
MSTRG.58399.1		XLOC_058823	259 coding	noncoding	noncoding	noncoding
XM_017008174.2	RBPJ	XLOC_058839	1987 coding	coding	coding	coding
XM_017008363.1	TBC1D19	XLOC_058856	1831 coding	coding	noncoding	coding
MSTRG.58982.1	TBC1D19	XLOC_058859	303 coding	noncoding	noncoding	noncoding
MSTRG.58631.1		XLOC_058906	246 coding	noncoding	noncoding	noncoding
MSTRG.58632.1		XLOC_058908	210 coding	noncoding	noncoding	noncoding
MSTRG.58673.1	LOC105374553	XLOC_058928	268 coding	noncoding	noncoding	noncoding
MSTRG.58691.1	LOC105374557	XLOC_058932	241 coding	noncoding	noncoding	noncoding
MSTRG.58727.1		XLOC_058962	255 coding	noncoding	noncoding	noncoding
NM_032456.3	PCDH7	XLOC_058985	4967 coding	coding	coding	coding
MSTRG.58829.1	LOC105374566	XLOC_059019	367 coding	noncoding	noncoding	noncoding
MSTRG.58831.1		XLOC_059020	271 coding	noncoding	noncoding	noncoding
MSTRG.58832.1		XLOC_059021	324 coding	noncoding	noncoding	noncoding

MSTRG.58882.1	LINC02506	XLOC_059032	251 coding	noncoding	noncoding	noncoding
MSTRG.58884.1		XLOC_059034	251 coding	noncoding	noncoding	noncoding
MSTRG.58870.1		XLOC_059057	253 coding	noncoding	noncoding	noncoding
MSTRG.58899.1		XLOC_059064	276 coding	noncoding	noncoding	noncoding
MSTRG.58907.1	LOC101928622	XLOC_059068	393 coding	noncoding	noncoding	noncoding
MSTRG.58914.1	LINC02484	XLOC_059071	282 coding	noncoding	noncoding	noncoding
MSTRG.58916.1	LINC02484	XLOC_059072	324 coding	noncoding	noncoding	noncoding
MSTRG.58918.1	LINC02484	XLOC_059073	278 coding	noncoding	noncoding	noncoding
MSTRG.58919.1	LINC02484	XLOC_059074	282 coding	noncoding	noncoding	noncoding
XM_011513696.2	DTHD1	XLOC_059126	5981 coding	coding	coding	coding
MSTRG.59077.1	LINC02505	XLOC_059144	292 coding	noncoding	noncoding	noncoding
MSTRG.59085.1	LINC02505	XLOC_059147	475 coding	noncoding	noncoding	noncoding
MSTRG.59097.1		XLOC_059157	291 coding	noncoding	noncoding	noncoding
MSTRG.59100.1		XLOC_059160	237 coding	noncoding	noncoding	noncoding
MSTRG.59126.1	LOC105374402	XLOC_059164	220 coding	noncoding	noncoding	noncoding
MSTRG.59344.1	TBC1D1	XLOC_059198	313 coding	noncoding	noncoding	noncoding
MSTRG.59373.1	TBC1D1	XLOC_059225	288 coding	noncoding	noncoding	noncoding
MSTRG.59230.1		XLOC_059248	217 coding	noncoding	noncoding	noncoding
MSTRG.59235.1		XLOC_059250	381 coding	noncoding	noncoding	noncoding
MSTRG.59297.10	TLR1	XLOC_059290	15000 coding	coding	coding	noncoding
NM_001007075.2	KLHL5	XLOC_059298	7657 coding	coding	noncoding	coding
MSTRG.59316.1		XLOC_059310	314 coding	noncoding	noncoding	noncoding
MSTRG.59439.1	LOC105374419	XLOC_059323	238 coding	noncoding	noncoding	noncoding
MSTRG.59440.1	LOC105374419	XLOC_059324	303 coding	noncoding	noncoding	noncoding
MSTRG.59448.1	UBE2K	XLOC_059330	326 coding	noncoding	noncoding	noncoding
MSTRG.59453.1	UBE2K	XLOC_059335	307 coding	noncoding	noncoding	noncoding
MSTRG.59455.1	UBE2K	XLOC_059337	437 coding	coding	noncoding	noncoding
MSTRG.59420.1		XLOC_059339	244 coding	noncoding	noncoding	noncoding
MSTRG.59457.5	PDS5A	XLOC_059341	692 coding	noncoding	noncoding	noncoding
MSTRG.59425.1		XLOC_059343	313 coding	noncoding	noncoding	noncoding
MSTRG.59486.1	N4BP2	XLOC_059345	306 coding	noncoding	noncoding	noncoding
NM_001278359.1	RHOH	XLOC_059351	2268 coding	coding	coding	coding
MSTRG.59522.1	RHOH	XLOC_059366	415 coding	noncoding	noncoding	noncoding
MSTRG.59473.1		XLOC_059367	437 coding	noncoding	noncoding	noncoding
MSTRG.59479.1		XLOC_059371	306 coding	noncoding	noncoding	noncoding
MSTRG.59482.1		XLOC_059372	276 coding	noncoding	noncoding	noncoding
MSTRG.59536.11	RBM47	XLOC_059373	4619 coding	coding	coding	coding
MSTRG.59536.12	RBM47	XLOC_059373	2296 coding	coding	coding	coding
MSTRG.59568.1	NSUN7	XLOC_059384	540 coding	noncoding	noncoding	noncoding
MSTRG.59576.1	APBB2	XLOC_059389	239 coding	noncoding	noncoding	noncoding
XM_011513651.2	LIMCH1	XLOC_059405	8762 coding	coding	coding	coding
MSTRG.59624.1	LIMCH1	XLOC_059408	254 coding	noncoding	noncoding	noncoding
MSTRG.59602.1	LOC105374425	XLOC_059412	310 coding	noncoding	noncoding	noncoding

MSTRG.59604.1	LOC105374425	XLOC_059414	273 coding	noncoding	noncoding	noncoding
MSTRG.59695.1		XLOC_059440	264 coding	noncoding	noncoding	noncoding
MSTRG.59673.1		XLOC_059449	243 coding	noncoding	noncoding	noncoding
MSTRG.59677.1	LOC105374428	XLOC_059451	294 coding	noncoding	noncoding	noncoding
MSTRG.59678.1	LOC105374428	XLOC_059452	217 coding	noncoding	noncoding	noncoding
MSTRG.59699.1	LOC105374430	XLOC_059460	227 coding	noncoding	noncoding	noncoding
MSTRG.59708.1		XLOC_059467	274 coding	coding	noncoding	noncoding
MSTRG.59714.1		XLOC_059468	210 coding	noncoding	noncoding	noncoding
MSTRG.59719.1		XLOC_059470	400 coding	noncoding	noncoding	noncoding
MSTRG.59736.1		XLOC_059479	256 coding	noncoding	noncoding	noncoding
MSTRG.59737.1		XLOC_059480	292 coding	noncoding	noncoding	noncoding
MSTRG.59741.1		XLOC_059482	269 coding	noncoding	coding	noncoding
NM_001345867.1	GUF1	XLOC_059500	4233 coding	coding	coding	coding
MSTRG.59789.1		XLOC_059512	253 coding	noncoding	noncoding	noncoding
MSTRG.59799.1		XLOC_059517	283 coding	noncoding	noncoding	noncoding
MSTRG.59801.1		XLOC_059519	306 coding	noncoding	noncoding	noncoding
MSTRG.59869.1		XLOC_059522	246 coding	coding	noncoding	noncoding
MSTRG.59856.1		XLOC_059523	292 coding	noncoding	noncoding	noncoding
MSTRG.59854.1		XLOC_059524	300 coding	noncoding	noncoding	noncoding
MSTRG.59872.1		XLOC_059526	242 coding	noncoding	noncoding	noncoding
MSTRG.59874.1		XLOC_059528	214 coding	noncoding	noncoding	noncoding
MSTRG.59873.1		XLOC_059530	306 coding	noncoding	noncoding	noncoding
MSTRG.59885.1		XLOC_059536	304 coding	noncoding	noncoding	noncoding
MSTRG.59906.1	COX7B2	XLOC_059544	294 coding	noncoding	noncoding	noncoding
MSTRG.59924.1	GABRB1	XLOC_059551	261 coding	noncoding	noncoding	noncoding
MSTRG.59929.1	GABRB1	XLOC_059555	287 coding	noncoding	noncoding	noncoding
MSTRG.59935.1	GABRB1	XLOC_059559	258 coding	noncoding	noncoding	noncoding
MSTRG.59936.1	GABRB1	XLOC_059560	287 coding	noncoding	noncoding	noncoding
MSTRG.60041.1	CORIN	XLOC_059585	285 coding	coding	noncoding	noncoding
MSTRG.60049.1	CORIN	XLOC_059593	242 coding	noncoding	noncoding	noncoding
MSTRG.60050.1	CORIN	XLOC_059594	278 coding	noncoding	noncoding	noncoding
MSTRG.59996.4	TXK	XLOC_059617	2455 coding	noncoding	noncoding	noncoding
MSTRG.59959.1		XLOC_059621	212 coding	noncoding	noncoding	noncoding
MSTRG.60102.1		XLOC_059648	324 coding	noncoding	noncoding	noncoding
MSTRG.60123.1		XLOC_059654	256 coding	noncoding	noncoding	noncoding
MSTRG.60200.1	LOC107986279	XLOC_059698	264 coding	coding	noncoding	noncoding
MSTRG.60546.1	LOC105377654	XLOC_059725	295 coding	noncoding	noncoding	noncoding
MSTRG.60562.1	LNX1	XLOC_059740	249 coding	coding	noncoding	noncoding
MSTRG.60567.1	LNX1	XLOC_059745	316 coding	noncoding	noncoding	noncoding
MSTRG.60572.1	LNX1	XLOC_059750	252 coding	noncoding	noncoding	noncoding
MSTRG.60305.1		XLOC_059758	319 coding	noncoding	noncoding	noncoding
MSTRG.60387.5	CHIC2	XLOC_059793	1260 coding	coding	coding	coding
MSTRG.60348.1		XLOC_059799	297 coding	noncoding	noncoding	noncoding

MSTRG.60349.1		XLOC_059800	227 coding	noncoding	noncoding	noncoding
MSTRG.60352.1		XLOC_059801	257 coding	noncoding	noncoding	noncoding
NM_001347830.1	PDGFRA	XLOC_059802	6796 coding	coding	coding	coding
MSTRG.60353.1		XLOC_059805	272 coding	noncoding	noncoding	noncoding
MSTRG.60424.1	LOC105377657	XLOC_059818	317 coding	coding	noncoding	noncoding
MSTRG.60446.1		XLOC_059826	284 coding	noncoding	noncoding	noncoding
MSTRG.60451.1		XLOC_059830	357 coding	coding	noncoding	noncoding
MSTRG.60452.1		XLOC_059831	312 coding	noncoding	noncoding	noncoding
MSTRG.60462.1		XLOC_059834	213 coding	noncoding	noncoding	noncoding
NM_018475.5	TMEM165	XLOC_059838	1931 coding	coding	coding	coding
MSTRG.60476.1	PDCL2	XLOC_059849	274 coding	noncoding	noncoding	noncoding
XM_005265752.2	KIAA1211	XLOC_059875	6918 coding	coding	coding	coding
MSTRG.60498.1	KIAA1211	XLOC_059877	259 coding	noncoding	noncoding	noncoding
NM_001256475.2	THEGL	XLOC_059892	1653 coding	coding	noncoding	coding
MSTRG.60606.1	THEGL	XLOC_059893	208 coding	noncoding	noncoding	noncoding
MSTRG.60615.2	HOPX	XLOC_059898	996 coding	coding	noncoding	noncoding
MSTRG.60630.1		XLOC_059906	313 coding	noncoding	noncoding	noncoding
MSTRG.60637.1		XLOC_059909	242 coding	noncoding	noncoding	noncoding
MSTRG.60643.1		XLOC_059918	365 coding	noncoding	noncoding	noncoding
MSTRG.60688.1		XLOC_059924	246 coding	noncoding	noncoding	noncoding
MSTRG.60696.1		XLOC_059934	245 coding	noncoding	noncoding	noncoding
MSTRG.60710.1		XLOC_059942	271 coding	noncoding	noncoding	noncoding
MSTRG.60709.1		XLOC_059943	230 coding	noncoding	noncoding	noncoding
MSTRG.60739.1		XLOC_059955	386 coding	noncoding	noncoding	noncoding
MSTRG.60786.1		XLOC_059975	200 coding	noncoding	noncoding	noncoding
MSTRG.60800.1		XLOC_059982	279 coding	coding	noncoding	noncoding
MSTRG.60814.1		XLOC_059988	281 coding	noncoding	noncoding	noncoding
MSTRG.60861.1	ADGRL3	XLOC_060001	218 coding	noncoding	noncoding	noncoding
MSTRG.60886.1	ADGRL3-AS1	XLOC_060015	241 coding	noncoding	noncoding	noncoding
MSTRG.60851.1		XLOC_060026	406 coding	noncoding	noncoding	noncoding
MSTRG.60854.1		XLOC_060029	255 coding	noncoding	noncoding	noncoding
MSTRG.60891.1		XLOC_060033	223 coding	noncoding	noncoding	noncoding
MSTRG.60902.1		XLOC_060042	297 coding	noncoding	noncoding	noncoding
MSTRG.60916.1		XLOC_060043	261 coding	noncoding	noncoding	noncoding
MSTRG.60918.1		XLOC_060045	257 coding	noncoding	noncoding	noncoding
MSTRG.60920.1		XLOC_060046	308 coding	noncoding	noncoding	noncoding
MSTRG.60971.1		XLOC_060078	288 coding	noncoding	noncoding	noncoding
MSTRG.60972.1		XLOC_060079	358 coding	coding	noncoding	noncoding
MSTRG.61000.1		XLOC_060091	208 coding	noncoding	noncoding	noncoding
MSTRG.61008.1		XLOC_060094	253 coding	noncoding	noncoding	noncoding
MSTRG.61035.1		XLOC_060107	303 coding	noncoding	noncoding	noncoding
MSTRG.61037.1		XLOC_060108	265 coding	noncoding	noncoding	noncoding
MSTRG.61048.1	STAP1	XLOC_060117	307 coding	noncoding	noncoding	noncoding

MSTRG.61116.1	TMPRSS11F	XLOC_060141	246 coding	noncoding	noncoding	noncoding
MSTRG.61260.7	SULT1B1	XLOC_060191	6456 coding	coding	coding	coding
MSTRG.61260.8	SULT1B1	XLOC_060191	2812 coding	coding	noncoding	noncoding
MSTRG.61259.1		XLOC_060198	322 coding	noncoding	noncoding	noncoding
MSTRG.61280.1	AMTN	XLOC_060219	265 coding	noncoding	noncoding	noncoding
MSTRG.61278.1		XLOC_060220	226 coding	noncoding	noncoding	noncoding
MSTRG.61283.1		XLOC_060222	311 coding	noncoding	noncoding	noncoding
MSTRG.61293.2	JCHAIN	XLOC_060228	1229 coding	coding	noncoding	noncoding
MSTRG.61325.1	RUFY3	XLOC_060235	249 coding	noncoding	noncoding	noncoding
MSTRG.61327.1	RUFY3	XLOC_060237	295 coding	coding	noncoding	noncoding
MSTRG.61317.1		XLOC_060248	332 coding	coding	noncoding	noncoding
XM_024454267.1	SLC4A4	XLOC_060251	9576 coding	coding	coding	coding
MSTRG.61400.1	SLC4A4	XLOC_060255	299 coding	coding	noncoding	noncoding
MSTRG.61434.1	SLC4A4	XLOC_060272	262 coding	coding	noncoding	noncoding
MSTRG.61344.1		XLOC_060276	233 coding	noncoding	noncoding	noncoding
MSTRG.61345.1		XLOC_060277	308 coding	noncoding	noncoding	noncoding
MSTRG.61371.1	ADAMTS3	XLOC_060286	316 coding	noncoding	noncoding	noncoding
MSTRG.61360.1		XLOC_060292	289 coding	noncoding	noncoding	noncoding
NM_001354840.2	CXCL8	XLOC_060340	672 coding	coding	noncoding	noncoding
NM_002620.4	PF4V1	XLOC_060345	743 coding	coding	noncoding	noncoding
MSTRG.61490.1		XLOC_060356	277 coding	noncoding	noncoding	noncoding
NM_001270989.2	EPGN	XLOC_060373	2681 coding	noncoding	coding	coding
NM_001432.3	EREG	XLOC_060378	4615 coding	noncoding	noncoding	coding
MSTRG.61526.1		XLOC_060390	244 coding	noncoding	noncoding	noncoding
MSTRG.61591.1		XLOC_060415	253 coding	coding	noncoding	noncoding
MSTRG.61685.1	CDKL2	XLOC_060434	293 coding	noncoding	noncoding	noncoding
MSTRG.61708.1	LOC105377284	XLOC_060448	473 coding	noncoding	noncoding	noncoding
MSTRG.61711.1	PPEF2	XLOC_060449	285 coding	noncoding	noncoding	noncoding
MSTRG.61739.5	NAAA	XLOC_060452	1138 coding	coding	coding	noncoding
MSTRG.61739.10	NAAA	XLOC_060452	1947 coding	coding	coding	noncoding
MSTRG.61733.1	ART3	XLOC_060457	270 coding	coding	noncoding	noncoding
NM_001242939.1	FAM47E-STBD1	XLOC_060464	3060 coding	coding	coding	coding
MSTRG.61811.1	LOC105377289	XLOC_060477	224 coding	noncoding	noncoding	noncoding
MSTRG.61784.1	SOWAHB	XLOC_060481	289 coding	noncoding	noncoding	noncoding
MSTRG.61942.1	CXCL13	XLOC_060531	204 coding	noncoding	noncoding	noncoding
MSTRG.61943.1	CXCL13	XLOC_060532	249 coding	coding	noncoding	noncoding
MSTRG.62013.4	CNOT6L	XLOC_060552	12727 coding	coding	coding	noncoding
MSTRG.61963.1		XLOC_060556	228 coding	noncoding	noncoding	noncoding
MSTRG.61978.1	MRPL1	XLOC_060560	269 coding	noncoding	noncoding	noncoding
MSTRG.61971.1		XLOC_060567	279 coding	noncoding	noncoding	noncoding
NM_001166133.1	FRAS1	XLOC_060570	7217 coding	coding	coding	coding
MSTRG.61989.1	FRAS1	XLOC_060573	260 coding	coding	noncoding	noncoding
MSTRG.61994.1	FRAS1	XLOC_060577	305 coding	coding	noncoding	noncoding

MSTRG.62083.1	LINC01088	XLOC_060637	278 coding	noncoding	noncoding	noncoding
MSTRG.62091.1	LINC01088	XLOC_060641	259 coding	noncoding	noncoding	noncoding
MSTRG.62098.1	NAA11	XLOC_060643	249 coding	noncoding	noncoding	noncoding
MSTRG.62101.1	NAA11	XLOC_060645	294 coding	coding	noncoding	noncoding
XR_001741747.1	LOC107986294	XLOC_060653	14647 coding	noncoding	noncoding	noncoding
MSTRG.62106.1	LINC02469	XLOC_060657	308 coding	noncoding	noncoding	noncoding
MSTRG.62109.1	LOC105377302	XLOC_060658	269 coding	noncoding	noncoding	noncoding
XM_011531816.2	CFAP299	XLOC_060680	2311 coding	coding	coding	coding
MSTRG.62212.1	CFAP299	XLOC_060689	261 coding	noncoding	noncoding	noncoding
MSTRG.62265.1		XLOC_060708	284 coding	noncoding	noncoding	noncoding
MSTRG.62263.1		XLOC_060710	234 coding	noncoding	noncoding	noncoding
MSTRG.62333.1	LOC107986215	XLOC_060732	267 coding	noncoding	noncoding	noncoding
MSTRG.62372.4	HNRNPDL	XLOC_060753	2789 coding	coding	coding	coding
MSTRG.62411.4	LIN54	XLOC_060764	3778 coding	coding	coding	noncoding
MSTRG.62424.8	PLAC8	XLOC_060771	2531 coding	noncoding	noncoding	noncoding
MSTRG.62421.1		XLOC_060772	254 coding	noncoding	noncoding	noncoding
MSTRG.62435.1		XLOC_060813	249 coding	noncoding	noncoding	noncoding
MSTRG.62528.1	LOC101928978	XLOC_060831	263 coding	noncoding	noncoding	noncoding
MSTRG.62592.15	WDFY3	XLOC_060848	4597 coding	noncoding	coding	noncoding
MSTRG.62592.21	WDFY3	XLOC_060848	561 coding	noncoding	noncoding	noncoding
MSTRG.62561.1		XLOC_060855	331 coding	noncoding	noncoding	noncoding
NM_031305.3	ARHGAP24	XLOC_060862	4827 coding	coding	coding	coding
MSTRG.62789.1	ARHGAP24	XLOC_060878	297 coding	coding	noncoding	noncoding
MSTRG.62816.1	ARHGAP24	XLOC_060901	336 coding	noncoding	noncoding	noncoding
MSTRG.62822.1	ARHGAP24	XLOC_060907	249 coding	noncoding	noncoding	noncoding
MSTRG.62654.1	MAPK10	XLOC_060917	305 coding	noncoding	noncoding	noncoding
MSTRG.62667.1	MAPK10	XLOC_060926	384 coding	coding	noncoding	noncoding
MSTRG.62574.1		XLOC_060938	229 coding	noncoding	noncoding	noncoding
MSTRG.62829.1		XLOC_060941	509 coding	noncoding	noncoding	noncoding
MSTRG.62833.1	AFF1-AS1	XLOC_060944	236 coding	noncoding	noncoding	noncoding
MSTRG.62843.1	AFF1	XLOC_060952	474 coding	coding	coding	noncoding
MSTRG.62868.1	AFF1	XLOC_060974	251 coding	noncoding	noncoding	noncoding
MSTRG.62710.1		XLOC_060990	275 coding	noncoding	noncoding	noncoding
MSTRG.62729.1		XLOC_060998	290 coding	noncoding	noncoding	noncoding
MSTRG.62731.1		XLOC_060999	233 coding	noncoding	noncoding	noncoding
MSTRG.62757.1		XLOC_061003	315 coding	noncoding	noncoding	noncoding
XM_011532029.1	PKD2	XLOC_061011	4369 coding	coding	coding	coding
MSTRG.62878.1	PKD2	XLOC_061012	225 coding	noncoding	noncoding	noncoding
MSTRG.62886.1	ABCG2	XLOC_061015	227 coding	noncoding	noncoding	noncoding
MSTRG.62892.1	PPM1K-DT	XLOC_061019	277 coding	noncoding	noncoding	noncoding
XM_017008808.1	HERC3	XLOC_061032	4637 coding	coding	coding	coding
MSTRG.62920.1		XLOC_061055	236 coding	noncoding	noncoding	noncoding
MSTRG.62929.1		XLOC_061062	319 coding	noncoding	noncoding	noncoding

MSTRG.62933.1	LOC105377328	XLOC_061063	342 coding	coding	noncoding	noncoding
XM_011531941.2	CCSER1	XLOC_061085	14387 coding	coding	coding	coding
MSTRG.63193.1	CCSER1	XLOC_061086	236 coding	noncoding	noncoding	noncoding
MSTRG.63251.1	CCSER1	XLOC_061098	276 coding	noncoding	noncoding	noncoding
MSTRG.63260.1	CCSER1	XLOC_061102	206 coding	noncoding	noncoding	noncoding
MSTRG.63297.1	CCSER1	XLOC_061113	281 coding	noncoding	noncoding	noncoding
MSTRG.63331.1	CCSER1	XLOC_061118	271 coding	noncoding	noncoding	noncoding
XR_002959799.1	LOC112268468	XLOC_061126	551 coding	noncoding	noncoding	noncoding
MSTRG.63000.1		XLOC_061128	278 coding	noncoding	noncoding	noncoding
XM_017008120.2	GRID2	XLOC_061129	12797 coding	coding	coding	coding
MSTRG.63069.1	GRID2	XLOC_061141	305 coding	noncoding	noncoding	noncoding
MSTRG.63099.1	GRID2	XLOC_061159	272 coding	coding	noncoding	noncoding
MSTRG.63106.1	GRID2	XLOC_061162	239 coding	noncoding	noncoding	noncoding
MSTRG.63380.1	PDLIM5	XLOC_061197	227 coding	noncoding	noncoding	noncoding
MSTRG.63385.1	PDLIM5	XLOC_061202	211 coding	noncoding	noncoding	noncoding
MSTRG.63391.1	PDLIM5	XLOC_061208	296 coding	noncoding	noncoding	noncoding
MSTRG.63118.1	BMPR1B	XLOC_061215	259 coding	noncoding	noncoding	noncoding
MSTRG.63131.1	UNC5C	XLOC_061218	210 coding	noncoding	noncoding	noncoding
NM_005390.5	PDHA2	XLOC_061226	1372 coding	coding	coding	coding
MSTRG.63151.1		XLOC_061229	284 coding	noncoding	noncoding	noncoding
MSTRG.63177.1	LINC02267	XLOC_061234	289 coding	noncoding	noncoding	noncoding
MSTRG.63153.1		XLOC_061237	282 coding	noncoding	noncoding	noncoding
MSTRG.63160.1		XLOC_061242	280 coding	noncoding	noncoding	noncoding
MSTRG.63539.1	LOC100507053	XLOC_061325	264 coding	noncoding	noncoding	noncoding
MSTRG.63552.1	ADH6	XLOC_061326	309 coding	noncoding	noncoding	noncoding
MSTRG.63556.1	LOC100507053	XLOC_061327	241 coding	noncoding	noncoding	noncoding
NM_000253.3	MTTP	XLOC_061336	4095 coding	coding	coding	coding
MSTRG.63590.1		XLOC_061373	251 coding	noncoding	noncoding	noncoding
MSTRG.63594.1		XLOC_061375	314 coding	noncoding	noncoding	noncoding
MSTRG.63625.1		XLOC_061392	515 coding	noncoding	noncoding	noncoding
MSTRG.63755.9	PPP3CA	XLOC_061395	6986 coding	noncoding	noncoding	noncoding
MSTRG.63692.1		XLOC_061401	307 coding	noncoding	noncoding	noncoding
MSTRG.63698.1		XLOC_061404	387 coding	coding	noncoding	noncoding
MSTRG.63968.2	MANBA	XLOC_061478	3253 coding	coding	coding	noncoding
MSTRG.64081.1	CISD2	XLOC_061493	283 coding	noncoding	noncoding	noncoding
MSTRG.64132.1	CENPE	XLOC_061528	258 coding	noncoding	noncoding	noncoding
MSTRG.63875.1		XLOC_061532	252 coding	noncoding	noncoding	noncoding
MSTRG.63877.1		XLOC_061533	258 coding	noncoding	noncoding	noncoding
MSTRG.63912.1		XLOC_061552	259 coding	noncoding	noncoding	noncoding
MSTRG.64058.1		XLOC_061573	296 coding	noncoding	noncoding	noncoding
MSTRG.64136.1		XLOC_061603	263 coding	noncoding	noncoding	noncoding
MSTRG.64193.1		XLOC_061623	281 coding	noncoding	noncoding	noncoding
MSTRG.64209.1		XLOC_061631	712 coding	noncoding	noncoding	noncoding

MSTRG.64211.1		XLOC_061632	317 coding	coding	noncoding	noncoding
MSTRG.64223.1		XLOC_061640	209 coding	noncoding	noncoding	noncoding
MSTRG.64232.1		XLOC_061644	297 coding	noncoding	noncoding	noncoding
MSTRG.64252.1	LOC105377358	XLOC_061656	248 coding	noncoding	noncoding	noncoding
MSTRG.64256.1	LOC105377357	XLOC_061659	248 coding	noncoding	noncoding	noncoding
XR_002959727.1	HADH	XLOC_061674	2162 coding	coding	coding	coding
MSTRG.64369.1		XLOC_061721	329 coding	noncoding	noncoding	noncoding
MSTRG.64415.1		XLOC_061727	252 coding	noncoding	noncoding	noncoding
MSTRG.64418.1		XLOC_061729	315 coding	coding	noncoding	noncoding
MSTRG.64444.1	COL25A1	XLOC_061734	298 coding	noncoding	noncoding	noncoding
MSTRG.64468.1	COL25A1	XLOC_061744	317 coding	noncoding	noncoding	noncoding
MSTRG.64547.13	SEC24B	XLOC_061762	310 coding	noncoding	noncoding	noncoding
MSTRG.64590.1	MCUB	XLOC_061782	261 coding	noncoding	noncoding	noncoding
XM_017007845.1	EGF	XLOC_061801	5632 coding	coding	coding	coding
MSTRG.64631.1		XLOC_061808	263 coding	noncoding	noncoding	noncoding
MSTRG.64655.1		XLOC_061813	296 coding	noncoding	noncoding	noncoding
MSTRG.64649.1		XLOC_061821	305 coding	coding	noncoding	noncoding
MSTRG.64651.1	PANCR	XLOC_061822	309 coding	noncoding	noncoding	noncoding
MSTRG.64684.1		XLOC_061832	361 coding	noncoding	noncoding	noncoding
MSTRG.64691.1		XLOC_061836	256 coding	noncoding	noncoding	noncoding
MSTRG.64699.1		XLOC_061841	276 coding	noncoding	noncoding	noncoding
MSTRG.64716.1		XLOC_061853	259 coding	noncoding	noncoding	noncoding
MSTRG.64722.1		XLOC_061860	362 coding	noncoding	noncoding	noncoding
MSTRG.64733.1		XLOC_061861	299 coding	noncoding	noncoding	noncoding
MSTRG.64737.1		XLOC_061863	257 coding	noncoding	noncoding	noncoding
MSTRG.64740.1		XLOC_061865	284 coding	noncoding	noncoding	noncoding
MSTRG.64777.1	ALPK1	XLOC_061882	355 coding	coding	noncoding	noncoding
MSTRG.64772.1		XLOC_061928	244 coding	noncoding	noncoding	noncoding
MSTRG.64838.1	ANK2	XLOC_061930	281 coding	noncoding	noncoding	noncoding
MSTRG.64858.1	ANK2	XLOC_061939	276 coding	noncoding	noncoding	noncoding
MSTRG.64862.1		XLOC_061947	263 coding	noncoding	noncoding	noncoding
MSTRG.64922.1		XLOC_061977	321 coding	noncoding	noncoding	noncoding
MSTRG.64923.1		XLOC_061978	244 coding	noncoding	noncoding	noncoding
MSTRG.64947.1		XLOC_061985	259 coding	noncoding	noncoding	noncoding
MSTRG.64956.1		XLOC_061991	251 coding	noncoding	noncoding	noncoding
MSTRG.64957.1		XLOC_061992	251 coding	noncoding	noncoding	noncoding
MSTRG.64958.1		XLOC_061993	200 coding	noncoding	noncoding	noncoding
MSTRG.64985.1		XLOC_062004	304 coding	noncoding	noncoding	noncoding
MSTRG.64992.1		XLOC_062008	252 coding	noncoding	noncoding	noncoding
MSTRG.65001.1	LOC107986306	XLOC_062010	254 coding	noncoding	noncoding	noncoding
MSTRG.64994.1		XLOC_062012	279 coding	noncoding	noncoding	noncoding
MSTRG.65015.1		XLOC_062020	261 coding	noncoding	noncoding	noncoding
MSTRG.65133.1	NDST3	XLOC_062076	278 coding	noncoding	noncoding	noncoding

MSTRG.65140.1	NDST3	XLOC_062080	340	coding	coding	noncoding	noncoding
MSTRG.65161.1		XLOC_062092	267	coding	noncoding	noncoding	noncoding
MSTRG.65166.1		XLOC_062094	362	coding	noncoding	noncoding	noncoding
MSTRG.65177.1	LOC729218	XLOC_062097	588	coding	noncoding	noncoding	coding
NM_001128933.3	SYNPO2	XLOC_062105	15417	coding	coding	coding	coding
MSTRG.65189.1		XLOC_062109	215	coding	noncoding	noncoding	noncoding
MSTRG.65191.1		XLOC_062110	233	coding	coding	noncoding	noncoding
MSTRG.65193.1		XLOC_062111	207	coding	noncoding	noncoding	noncoding
MSTRG.65253.1	LINC01061	XLOC_062139	650	coding	coding	coding	noncoding
MSTRG.65276.1		XLOC_062161	442	coding	noncoding	noncoding	noncoding
MSTRG.65312.1		XLOC_062175	268	coding	noncoding	noncoding	noncoding
MSTRG.65314.1		XLOC_062176	213	coding	noncoding	noncoding	noncoding
MSTRG.65345.1		XLOC_062232	316	coding	noncoding	noncoding	noncoding
MSTRG.65380.1		XLOC_062259	304	coding	noncoding	noncoding	noncoding
MSTRG.65390.1		XLOC_062267	258	coding	noncoding	noncoding	noncoding
MSTRG.65392.1		XLOC_062269	283	coding	noncoding	noncoding	noncoding
MSTRG.65406.1		XLOC_062293	315	coding	noncoding	noncoding	noncoding
XM_017008696.1	KIAA1109	XLOC_062297	15803	coding	coding	coding	coding
XM_005262743.3	ADAD1	XLOC_062330	1931	coding	coding	coding	coding
MSTRG.65511.1	ADAD1	XLOC_062331	280	coding	noncoding	noncoding	noncoding
MSTRG.65515.1		XLOC_062335	312	coding	noncoding	noncoding	noncoding
MSTRG.65533.1		XLOC_062343	353	coding	noncoding	noncoding	noncoding
XM_017007829.1	SPATA5	XLOC_062353	4037	coding	coding	coding	coding
MSTRG.65835.1	SPATA5	XLOC_062368	224	coding	noncoding	noncoding	noncoding
MSTRG.65843.1	SPATA5	XLOC_062376	355	coding	coding	noncoding	noncoding
MSTRG.65558.1	LINC01091	XLOC_062388	296	coding	noncoding	noncoding	noncoding
MSTRG.65547.1		XLOC_062393	305	coding	noncoding	noncoding	noncoding
MSTRG.65575.1	LOC105377407	XLOC_062397	501	coding	noncoding	noncoding	noncoding
MSTRG.65566.1		XLOC_062404	280	coding	noncoding	noncoding	noncoding
MSTRG.65607.1		XLOC_062424	273	coding	noncoding	noncoding	noncoding
MSTRG.65621.1		XLOC_062435	837	coding	noncoding	noncoding	noncoding
MSTRG.65708.1		XLOC_062449	278	coding	noncoding	noncoding	noncoding
MSTRG.65693.1		XLOC_062450	299	coding	noncoding	noncoding	noncoding
MSTRG.65702.1		XLOC_062454	372	coding	noncoding	noncoding	noncoding
MSTRG.65715.1		XLOC_062458	250	coding	noncoding	noncoding	noncoding
MSTRG.65718.1		XLOC_062460	313	coding	noncoding	noncoding	noncoding
MSTRG.65725.1		XLOC_062464	349	coding	noncoding	noncoding	noncoding
MSTRG.65743.1		XLOC_062474	293	coding	noncoding	noncoding	noncoding
MSTRG.65746.1		XLOC_062483	295	coding	coding	noncoding	noncoding
MSTRG.65784.1		XLOC_062503	251	coding	noncoding	noncoding	noncoding
MSTRG.65876.1	LARP1B	XLOC_062522	218	coding	noncoding	noncoding	noncoding
MSTRG.65878.1	LARP1B	XLOC_062523	321	coding	noncoding	noncoding	noncoding
MSTRG.65867.1		XLOC_062525	225	coding	noncoding	noncoding	noncoding

MSTRG.65895.2	PGRMC2	XLOC_062526	1528	coding	coding	noncoding	noncoding
MSTRG.65911.1	LINC02615	XLOC_062534	255	coding	noncoding	noncoding	noncoding
MSTRG.65913.1		XLOC_062540	212	coding	noncoding	noncoding	noncoding
MSTRG.65922.1		XLOC_062546	283	coding	noncoding	noncoding	noncoding
MSTRG.65938.1		XLOC_062556	273	coding	noncoding	noncoding	noncoding
MSTRG.65948.1		XLOC_062560	378	coding	coding	noncoding	noncoding
MSTRG.65954.1		XLOC_062563	203	coding	noncoding	noncoding	noncoding
MSTRG.65955.1		XLOC_062564	262	coding	noncoding	noncoding	noncoding
MSTRG.65957.1		XLOC_062565	346	coding	noncoding	noncoding	noncoding
MSTRG.65959.1		XLOC_062566	288	coding	noncoding	noncoding	noncoding
MSTRG.65968.1		XLOC_062571	333	coding	noncoding	noncoding	noncoding
MSTRG.65981.1		XLOC_062577	235	coding	noncoding	noncoding	noncoding
MSTRG.65990.1		XLOC_062584	302	coding	noncoding	noncoding	noncoding
MSTRG.65997.1		XLOC_062587	224	coding	noncoding	noncoding	noncoding
MSTRG.66087.1		XLOC_062640	222	coding	noncoding	noncoding	noncoding
MSTRG.66104.1	PABPC4L	XLOC_062644	277	coding	noncoding	noncoding	noncoding
MSTRG.66108.1	PABPC4L	XLOC_062646	297	coding	noncoding	noncoding	noncoding
MSTRG.66123.1		XLOC_062659	650	coding	noncoding	noncoding	noncoding
MSTRG.66139.1		XLOC_062664	293	coding	noncoding	noncoding	noncoding
MSTRG.66161.1		XLOC_062673	251	coding	noncoding	noncoding	noncoding
MSTRG.66172.1		XLOC_062680	220	coding	noncoding	noncoding	noncoding
MSTRG.66206.1	LINC02511	XLOC_062691	331	coding	noncoding	noncoding	noncoding
MSTRG.66217.1		XLOC_062706	264	coding	noncoding	noncoding	noncoding
MSTRG.66233.1		XLOC_062712	316	coding	noncoding	noncoding	noncoding
MSTRG.66261.1	SLC7A11-AS1	XLOC_062717	289	coding	noncoding	noncoding	noncoding
MSTRG.66235.1		XLOC_062724	252	coding	noncoding	noncoding	noncoding
MSTRG.66325.2	ELF2	XLOC_062757	2512	coding	coding	coding	coding
MSTRG.66279.1	MGARP	XLOC_062763	253	coding	noncoding	noncoding	noncoding
MSTRG.66281.1		XLOC_062779	462	coding	noncoding	noncoding	noncoding
MSTRG.66456.1	RNF150	XLOC_062823	303	coding	noncoding	noncoding	noncoding
MSTRG.66462.1	RNF150	XLOC_062828	303	coding	noncoding	noncoding	noncoding
MSTRG.66564.1		XLOC_062851	234	coding	noncoding	noncoding	noncoding
MSTRG.66583.1	LINC02276	XLOC_062869	286	coding	noncoding	noncoding	noncoding
MSTRG.66489.1		XLOC_062886	397	coding	noncoding	noncoding	noncoding
MSTRG.66879.9	INPP4B	XLOC_062893	1096	coding	noncoding	noncoding	noncoding
MSTRG.66500.1		XLOC_062898	215	coding	noncoding	noncoding	noncoding
MSTRG.66508.1	LOC107986194	XLOC_062903	246	coding	noncoding	noncoding	noncoding
MSTRG.66509.1	LOC107986194	XLOC_062904	222	coding	noncoding	noncoding	noncoding
MSTRG.66519.1		XLOC_062926	310	coding	noncoding	noncoding	noncoding
NM_002039.4	GAB1	XLOC_062930	7774	coding	coding	coding	coding
MSTRG.66767.13	GYPA	XLOC_062956	1280	coding	coding	noncoding	noncoding
MSTRG.66767.29	GYPB	XLOC_062956	1240	coding	coding	noncoding	noncoding
MSTRG.66715.1		XLOC_062960	301	coding	noncoding	noncoding	noncoding

MSTRG.66726.1	LOC105377462	XLOC_062963	307 coding	noncoding	noncoding	noncoding
NM_022475.3	HHIP	XLOC_062965	9937 coding	coding	coding	coding
MSTRG.66806.2	OTUD4	XLOC_062996	5284 coding	coding	coding	noncoding
MSTRG.66790.1		XLOC_063000	306 coding	noncoding	noncoding	noncoding
MSTRG.66800.1		XLOC_063001	228 coding	noncoding	noncoding	noncoding
NM_001354811.1	SMAD1	XLOC_063002	3303 coding	coding	coding	coding
XM_011531685.2	MMAA	XLOC_063010	4768 coding	coding	coding	coding
MSTRG.66837.1		XLOC_063012	217 coding	noncoding	noncoding	noncoding
MSTRG.67009.5	SLC10A7	XLOC_063034	707 coding	coding	noncoding	noncoding
MSTRG.66976.1		XLOC_063048	225 coding	noncoding	noncoding	noncoding
MSTRG.66998.1	EDNRA	XLOC_063051	222 coding	noncoding	noncoding	noncoding
MSTRG.67034.1		XLOC_063062	289 coding	noncoding	noncoding	noncoding
MSTRG.67422.1	ARHGAP10	XLOC_063065	220 coding	noncoding	noncoding	noncoding
MSTRG.67424.1	ARHGAP10	XLOC_063067	257 coding	noncoding	noncoding	noncoding
MSTRG.67430.1	ARHGAP10	XLOC_063073	270 coding	coding	noncoding	noncoding
MSTRG.67505.1	LOC107986195	XLOC_063090	276 coding	noncoding	noncoding	noncoding
MSTRG.67532.1	LOC107986195	XLOC_063094	250 coding	noncoding	noncoding	noncoding
MSTRG.67555.1	LOC107986195	XLOC_063102	424 coding	noncoding	noncoding	noncoding
MSTRG.67067.1	IQCM	XLOC_063110	237 coding	noncoding	noncoding	noncoding
MSTRG.67078.1	IQCM	XLOC_063118	304 coding	noncoding	noncoding	noncoding
MSTRG.67083.1	IQCM	XLOC_063122	207 coding	noncoding	noncoding	noncoding
MSTRG.67098.1	DCLK2	XLOC_063136	291 coding	noncoding	noncoding	noncoding
MSTRG.67100.1		XLOC_063141	243 coding	noncoding	noncoding	noncoding
NM_001267699.1	RPS3A	XLOC_063146	1581 coding	coding	noncoding	noncoding
MSTRG.67135.1	SH3D19	XLOC_063151	335 coding	noncoding	noncoding	noncoding
MSTRG.67136.1	SH3D19	XLOC_063152	223 coding	noncoding	noncoding	noncoding
MSTRG.67120.1		XLOC_063158	264 coding	noncoding	noncoding	noncoding
MSTRG.67130.1		XLOC_063162	266 coding	noncoding	noncoding	noncoding
MSTRG.67194.1	FAM160A1	XLOC_063167	314 coding	noncoding	noncoding	noncoding
MSTRG.67331.4	FBXW7-AS1	XLOC_063195	4872 coding	coding	coding	noncoding
MSTRG.67331.7	FBXW7-AS1	XLOC_063195	3623 coding	coding	noncoding	noncoding
MSTRG.67311.1		XLOC_063240	257 coding	noncoding	noncoding	noncoding
MSTRG.67343.1	TRIM2	XLOC_063245	261 coding	noncoding	noncoding	noncoding
MSTRG.67351.1	LOC105377496	XLOC_063247	235 coding	noncoding	noncoding	noncoding
MSTRG.67320.1		XLOC_063257	273 coding	noncoding	noncoding	noncoding
NM_003264.5	TLR2	XLOC_063298	3581 coding	coding	coding	coding
MSTRG.67661.1		XLOC_063322	266 coding	noncoding	noncoding	noncoding
XR_939389.2	LOC105377507	XLOC_063341	4820 coding	noncoding	noncoding	noncoding
MSTRG.67715.1		XLOC_063353	253 coding	noncoding	noncoding	noncoding
MSTRG.67809.1	TMEM144	XLOC_063383	260 coding	noncoding	noncoding	noncoding
MSTRG.67793.1	LOC105377510	XLOC_063387	324 coding	noncoding	noncoding	noncoding
MSTRG.67791.1		XLOC_063389	280 coding	noncoding	coding	noncoding
MSTRG.67813.1		XLOC_063404	235 coding	noncoding	noncoding	noncoding

MSTRG.67814.1		XLOC_063405	315	coding	noncoding	noncoding	noncoding
MSTRG.67835.1	FNIP2	XLOC_063408	313	coding	noncoding	noncoding	noncoding
NM_001351725.1	RAPGEF2	XLOC_063413	7780	coding	coding	coding	coding
MSTRG.67868.1		XLOC_063477	231	coding	noncoding	noncoding	noncoding
MSTRG.67875.1		XLOC_063479	298	coding	coding	noncoding	noncoding
MSTRG.67881.1		XLOC_063483	317	coding	noncoding	noncoding	noncoding
MSTRG.67886.1		XLOC_063485	215	coding	noncoding	noncoding	noncoding
MSTRG.67924.1	FSTL5	XLOC_063497	277	coding	noncoding	noncoding	noncoding
MSTRG.67929.1	FSTL5	XLOC_063498	272	coding	noncoding	noncoding	noncoding
MSTRG.67938.1	FSTL5	XLOC_063504	314	coding	noncoding	noncoding	noncoding
MSTRG.67956.1		XLOC_063522	284	coding	noncoding	noncoding	noncoding
MSTRG.68131.1	1-Mar	XLOC_063556	214	coding	coding	noncoding	noncoding
MSTRG.68013.1		XLOC_063568	272	coding	noncoding	noncoding	noncoding
MSTRG.68015.1		XLOC_063570	281	coding	noncoding	noncoding	noncoding
MSTRG.68028.1		XLOC_063580	248	coding	noncoding	noncoding	noncoding
MSTRG.68031.1		XLOC_063582	250	coding	noncoding	noncoding	noncoding
MSTRG.68047.1		XLOC_063592	238	coding	noncoding	noncoding	noncoding
MSTRG.68223.1		XLOC_063594	290	coding	coding	noncoding	noncoding
XM_017007674.1	KLHL2	XLOC_063600	3078	coding	coding	coding	coding
NM_001873.4	CPE	XLOC_063634	2582	coding	coding	coding	coding
MSTRG.68159.1		XLOC_063638	254	coding	noncoding	noncoding	noncoding
MSTRG.68176.1	LOC101928131	XLOC_063646	268	coding	noncoding	noncoding	noncoding
XM_024454195.1	TLL1	XLOC_063651	9033	coding	coding	coding	coding
MSTRG.68190.1	TLL1	XLOC_063652	325	coding	noncoding	noncoding	noncoding
MSTRG.68200.1	TLL1	XLOC_063656	288	coding	noncoding	noncoding	noncoding
MSTRG.68211.1		XLOC_063665	271	coding	noncoding	noncoding	noncoding
MSTRG.68285.1	SPOCK3	XLOC_063671	303	coding	noncoding	noncoding	noncoding
MSTRG.68286.1	SPOCK3	XLOC_063672	213	coding	noncoding	noncoding	noncoding
XM_017007911.1	PALLD	XLOC_063716	4146	coding	coding	coding	coding
XM_024453939.1	PALLD	XLOC_063716	6138	coding	coding	coding	coding
MSTRG.68337.28	CBR4	XLOC_063716	244	coding	coding	noncoding	noncoding
MSTRG.68340.1	PALLD	XLOC_063717	263	coding	noncoding	noncoding	noncoding
MSTRG.68347.1	PALLD	XLOC_063720	217	coding	noncoding	noncoding	noncoding
MSTRG.68315.1	LOC105377529	XLOC_063744	385	coding	noncoding	noncoding	noncoding
MSTRG.68320.1		XLOC_063746	239	coding	coding	noncoding	noncoding
XM_011531586.2	CLCN3	XLOC_063751	4151	coding	coding	coding	coding
MSTRG.68492.1	CLCN3	XLOC_063752	224	coding	noncoding	noncoding	noncoding
MSTRG.68520.6	MFAP3L	XLOC_063765	4535	coding	coding	coding	noncoding
MSTRG.68520.5	MFAP3L	XLOC_063765	4737	coding	coding	coding	noncoding
MSTRG.68517.1		XLOC_063767	316	coding	coding	noncoding	noncoding
MSTRG.68532.1		XLOC_063777	301	coding	noncoding	noncoding	noncoding
MSTRG.68536.1		XLOC_063780	282	coding	coding	noncoding	noncoding
MSTRG.68538.1		XLOC_063782	226	coding	noncoding	noncoding	noncoding

MSTRG.68545.1		XLOC_063785	249 coding	noncoding	noncoding	noncoding
MSTRG.68554.1		XLOC_063793	244 coding	noncoding	noncoding	noncoding
MSTRG.68565.1		XLOC_063798	270 coding	noncoding	noncoding	noncoding
XM_017008243.2	GALNTL6	XLOC_063799	2739 coding	coding	coding	coding
MSTRG.68596.1	GALNTL6	XLOC_063803	307 coding	noncoding	noncoding	noncoding
MSTRG.68604.1	GALNTL6	XLOC_063808	283 coding	noncoding	noncoding	noncoding
MSTRG.68626.1	GALNTL6	XLOC_063816	278 coding	noncoding	noncoding	noncoding
MSTRG.68715.1	SCRG1	XLOC_063866	216 coding	noncoding	noncoding	noncoding
MSTRG.68669.1		XLOC_063875	477 coding	coding	noncoding	noncoding
MSTRG.68672.1		XLOC_063877	301 coding	noncoding	noncoding	noncoding
MSTRG.68741.1		XLOC_063891	266 coding	noncoding	noncoding	noncoding
MSTRG.68752.1		XLOC_063899	212 coding	noncoding	noncoding	noncoding
MSTRG.68756.1		XLOC_063904	289 coding	noncoding	noncoding	noncoding
MSTRG.68820.1	GPM6A	XLOC_063925	288 coding	noncoding	noncoding	noncoding
MSTRG.68890.1		XLOC_063961	299 coding	noncoding	noncoding	noncoding
MSTRG.68911.1	NEIL3	XLOC_063971	294 coding	noncoding	noncoding	noncoding
MSTRG.68952.1		XLOC_063985	272 coding	noncoding	noncoding	noncoding
MSTRG.69004.1		XLOC_064003	275 coding	noncoding	noncoding	noncoding
MSTRG.69030.1		XLOC_064013	271 coding	noncoding	noncoding	noncoding
MSTRG.69037.1		XLOC_064019	363 coding	noncoding	noncoding	noncoding
MSTRG.69060.1	LOC105377567	XLOC_064024	322 coding	noncoding	noncoding	noncoding
MSTRG.69046.1		XLOC_064035	312 coding	noncoding	noncoding	noncoding
MSTRG.69082.1		XLOC_064040	286 coding	noncoding	noncoding	noncoding
MSTRG.69131.1	TEMN3-AS1	XLOC_064053	410 coding	noncoding	noncoding	noncoding
MSTRG.69144.1	TENM3	XLOC_064059	292 coding	noncoding	noncoding	noncoding
MSTRG.69151.1	TENM3	XLOC_064062	254 coding	noncoding	noncoding	noncoding
MSTRG.69156.1	TENM3	XLOC_064065	323 coding	noncoding	noncoding	noncoding
MSTRG.69172.1	TENM3	XLOC_064070	226 coding	noncoding	noncoding	noncoding
MSTRG.69184.1	TENM3	XLOC_064075	614 coding	noncoding	noncoding	noncoding
MSTRG.69101.1		XLOC_064089	535 coding	noncoding	noncoding	noncoding
MSTRG.69112.1	LOC105377578	XLOC_064094	217 coding	noncoding	noncoding	noncoding
MSTRG.69242.1	WWC2	XLOC_064100	291 coding	noncoding	noncoding	noncoding
MSTRG.69247.1	WWC2	XLOC_064102	200 coding	noncoding	noncoding	noncoding
MSTRG.69205.1		XLOC_064109	220 coding	noncoding	noncoding	noncoding
MSTRG.69317.1	STOX2	XLOC_064148	270 coding	noncoding	noncoding	noncoding
MSTRG.69297.1	ENPP6	XLOC_064153	263 coding	noncoding	noncoding	noncoding
MSTRG.69402.1	PRIMPOL	XLOC_064181	309 coding	noncoding	noncoding	noncoding
MSTRG.69410.1		XLOC_064195	236 coding	noncoding	noncoding	noncoding
MSTRG.69416.1		XLOC_064200	225 coding	noncoding	noncoding	noncoding
MSTRG.69425.1		XLOC_064203	313 coding	noncoding	noncoding	noncoding
MSTRG.69438.1		XLOC_064217	299 coding	noncoding	noncoding	noncoding
MSTRG.69486.1	SORBS2	XLOC_064233	307 coding	noncoding	noncoding	noncoding
NM_001367768.2	FAM149A	XLOC_064240	5968 coding	coding	coding	coding

MSTRG.69576.1	KLKB1	XLOC_064249	315	coding	noncoding	noncoding	noncoding
MSTRG.69544.1	LOC105377596	XLOC_064256	247	coding	noncoding	noncoding	noncoding
MSTRG.69629.1		XLOC_064321	302	coding	noncoding	noncoding	noncoding
MSTRG.69637.1		XLOC_064325	277	coding	noncoding	noncoding	noncoding
MSTRG.69638.1		XLOC_064327	294	coding	noncoding	noncoding	noncoding
MSTRG.69646.1		XLOC_064332	242	coding	noncoding	noncoding	noncoding
MSTRG.69659.1	LINC01060	XLOC_064337	309	coding	noncoding	noncoding	noncoding
MSTRG.69715.1		XLOC_064344	301	coding	noncoding	noncoding	noncoding
MSTRG.69731.1		XLOC_064352	288	coding	noncoding	noncoding	noncoding
MSTRG.69742.1		XLOC_064357	280	coding	noncoding	noncoding	noncoding
MSTRG.69745.1		XLOC_064358	264	coding	noncoding	noncoding	noncoding
MSTRG.56110.1	ZNF718	XLOC_064377	445	coding	coding	noncoding	noncoding
MSTRG.56110.7	ZNF718	XLOC_064377	448	coding	coding	noncoding	noncoding
MSTRG.56090.1		XLOC_064399	390	coding	noncoding	noncoding	noncoding
MSTRG.56095.1		XLOC_064401	736	coding	noncoding	noncoding	noncoding
MSTRG.56151.1	ABCA11P	XLOC_064411	309	coding	noncoding	noncoding	noncoding
MSTRG.56146.1	LOC107986245	XLOC_064427	227	coding	noncoding	noncoding	noncoding
MSTRG.56194.8	LOC107986211	XLOC_064428	3312	coding	coding	noncoding	noncoding
MSTRG.56201.1		XLOC_064430	352	coding	noncoding	noncoding	noncoding
MSTRG.56203.1	LOC100129917	XLOC_064431	241	coding	noncoding	noncoding	noncoding
MSTRG.56189.1		XLOC_064434	322	coding	noncoding	noncoding	noncoding
NM_001128325.2	SPON2	XLOC_064468	1807	coding	coding	coding	coding
MSTRG.56295.1	NKX1-1	XLOC_064501	249	coding	noncoding	noncoding	noncoding
MSTRG.56303.1	FAM53A	XLOC_064507	237	coding	noncoding	noncoding	noncoding
MSTRG.56346.1	LETM1	XLOC_064513	227	coding	noncoding	noncoding	noncoding
MSTRG.56368.6	NSD2	XLOC_064518	927	coding	coding	noncoding	coding
MSTRG.56390.1	NELFA	XLOC_064530	301	coding	noncoding	noncoding	noncoding
MSTRG.56359.1	POLN	XLOC_064539	247	coding	noncoding	noncoding	noncoding
MSTRG.56444.1	LOC105374353	XLOC_064551	286	coding	noncoding	noncoding	noncoding
MSTRG.56445.2	FAM193A	XLOC_064552	2443	coding	coding	coding	coding
MSTRG.56437.1		XLOC_064562	271	coding	noncoding	noncoding	noncoding
MSTRG.56485.2	ADD1	XLOC_064567	3340	coding	coding	coding	coding
MSTRG.56501.1	GRK4	XLOC_064574	265	coding	coding	noncoding	noncoding
MSTRG.56582.1		XLOC_064619	311	coding	noncoding	noncoding	noncoding
MSTRG.56586.1		XLOC_064621	293	coding	noncoding	noncoding	noncoding
MSTRG.56612.1	LYAR	XLOC_064625	219	coding	noncoding	noncoding	noncoding
XM_011513505.1	LYAR	XLOC_064626	1871	coding	coding	coding	coding
MSTRG.56618.3	ZBTB49	XLOC_064629	519	coding	coding	noncoding	noncoding
MSTRG.56608.1		XLOC_064636	570	coding	noncoding	noncoding	noncoding
MSTRG.56642.1		XLOC_064710	455	coding	noncoding	noncoding	noncoding
MSTRG.56649.1	LOC101928306	XLOC_064713	283	coding	noncoding	noncoding	noncoding
MSTRG.56653.1		XLOC_064719	303	coding	noncoding	noncoding	noncoding
MSTRG.56693.1	STK32B	XLOC_064737	321	coding	noncoding	noncoding	noncoding

MSTRG.56713.1	EVC	XLOC_064745	357 coding	noncoding	noncoding	noncoding
MSTRG.56742.1	PPP2R2C	XLOC_064774	272 coding	noncoding	noncoding	noncoding
MSTRG.56875.1		XLOC_064790	481 coding	coding	coding	noncoding
MSTRG.56900.1		XLOC_064808	369 coding	noncoding	noncoding	noncoding
MSTRG.56930.1		XLOC_064817	261 coding	noncoding	noncoding	noncoding
MSTRG.56932.1		XLOC_064818	281 coding	noncoding	noncoding	noncoding
MSTRG.56945.1		XLOC_064821	303 coding	noncoding	noncoding	noncoding
MSTRG.56972.1	SORCS2	XLOC_064822	251 coding	noncoding	noncoding	noncoding
NM_001085382.2	PSAPL1	XLOC_064828	4646 coding	coding	coding	coding
MSTRG.57035.1		XLOC_064858	272 coding	noncoding	noncoding	noncoding
MSTRG.57148.1	ACOX3	XLOC_064873	260 coding	noncoding	noncoding	noncoding
MSTRG.57082.1		XLOC_064885	281 coding	noncoding	noncoding	noncoding
MSTRG.57092.1		XLOC_064891	266 coding	noncoding	noncoding	noncoding
MSTRG.57111.1	LOC105369250	XLOC_064896	328 coding	noncoding	noncoding	noncoding
MSTRG.57100.1		XLOC_064898	250 coding	noncoding	noncoding	noncoding
MSTRG.57164.1		XLOC_064902	280 coding	noncoding	noncoding	noncoding
MSTRG.57163.1		XLOC_064903	242 coding	noncoding	noncoding	noncoding
XM_024454265.1	ZNF518B	XLOC_064982	9777 coding	noncoding	coding	coding
MSTRG.57264.1	LOC105374481	XLOC_064985	253 coding	noncoding	noncoding	noncoding
MSTRG.57265.1	LOC105374481	XLOC_064986	321 coding	noncoding	noncoding	noncoding
MSTRG.57247.1	LINC02498	XLOC_064992	270 coding	noncoding	noncoding	noncoding
MSTRG.57405.1	LOC105374483	XLOC_065079	200 coding	noncoding	noncoding	noncoding
MSTRG.57408.1		XLOC_065082	205 coding	noncoding	noncoding	noncoding
MSTRG.57410.1		XLOC_065084	363 coding	noncoding	noncoding	noncoding
MSTRG.57418.1		XLOC_065089	291 coding	noncoding	noncoding	noncoding
MSTRG.57466.1		XLOC_065095	297 coding	noncoding	noncoding	noncoding
MSTRG.57488.1		XLOC_065104	320 coding	noncoding	noncoding	noncoding
MSTRG.57505.1	LINC01182	XLOC_065146	280 coding	noncoding	noncoding	noncoding
MSTRG.57507.1	LINC01182	XLOC_065147	273 coding	noncoding	noncoding	noncoding
MSTRG.57523.1	LOC107986182	XLOC_065153	227 coding	noncoding	noncoding	noncoding
MSTRG.57538.1		XLOC_065172	222 coding	noncoding	noncoding	noncoding
NR_126435.1	LINC00504	XLOC_065175	4672 coding	coding	coding	coding
XM_006713959.3	FBXL5	XLOC_065241	3014 coding	coding	coding	coding
MSTRG.57745.1		XLOC_065302	239 coding	noncoding	noncoding	noncoding
MSTRG.57805.1		XLOC_065319	235 coding	noncoding	noncoding	noncoding
MSTRG.58049.1	LCORL	XLOC_065353	620 coding	noncoding	noncoding	noncoding
MSTRG.57916.1	LOC107986262	XLOC_065373	258 coding	noncoding	noncoding	noncoding
MSTRG.57962.1		XLOC_065399	275 coding	noncoding	noncoding	noncoding
MSTRG.58006.1	LOC105374515	XLOC_065428	388 coding	noncoding	noncoding	noncoding
MSTRG.58007.1		XLOC_065429	322 coding	noncoding	noncoding	noncoding
MSTRG.58207.1	KCNIP4	XLOC_065458	586 coding	noncoding	noncoding	noncoding
MSTRG.58074.1		XLOC_065464	289 coding	noncoding	noncoding	noncoding
MSTRG.58076.1		XLOC_065466	241 coding	noncoding	noncoding	noncoding

MSTRG.58077.1		XLOC_065467	324	coding	noncoding	noncoding	noncoding
MSTRG.58091.1		XLOC_065479	261	coding	noncoding	noncoding	noncoding
MSTRG.58152.1	LOC105374524	XLOC_065501	263	coding	noncoding	noncoding	noncoding
MSTRG.58230.1	PPARGC1A	XLOC_065519	316	coding	noncoding	noncoding	noncoding
MSTRG.58390.1		XLOC_065590	256	coding	noncoding	noncoding	noncoding
MSTRG.58421.1		XLOC_065610	259	coding	noncoding	noncoding	noncoding
MSTRG.58467.1	RBPJ	XLOC_065618	289	coding	noncoding	noncoding	noncoding
MSTRG.58476.1	RBPJ	XLOC_065621	355	coding	noncoding	noncoding	noncoding
MSTRG.58633.1		XLOC_065661	210	coding	noncoding	noncoding	noncoding
MSTRG.58644.1		XLOC_065667	333	coding	noncoding	noncoding	noncoding
MSTRG.58649.1	LOC105374548	XLOC_065670	308	coding	noncoding	noncoding	noncoding
MSTRG.58801.1	PCDH7	XLOC_065728	291	coding	coding	noncoding	noncoding
MSTRG.58773.1		XLOC_065739	287	coding	noncoding	noncoding	noncoding
MSTRG.58778.1		XLOC_065742	288	coding	noncoding	noncoding	noncoding
MSTRG.58798.1	LINC02501	XLOC_065749	280	coding	noncoding	noncoding	noncoding
MSTRG.58841.1		XLOC_065760	256	coding	noncoding	noncoding	noncoding
MSTRG.58860.1		XLOC_065767	307	coding	noncoding	noncoding	noncoding
MSTRG.58900.1		XLOC_065779	233	coding	noncoding	noncoding	noncoding
MSTRG.58915.1	LINC02484	XLOC_065785	282	coding	noncoding	noncoding	noncoding
MSTRG.58917.1	LINC02484	XLOC_065786	324	coding	noncoding	noncoding	noncoding
MSTRG.58920.1	LINC02484	XLOC_065787	283	coding	noncoding	noncoding	noncoding
MSTRG.59107.8	DTHD1	XLOC_065848	6113	coding	coding	coding	noncoding
MSTRG.59069.1		XLOC_065856	330	coding	noncoding	noncoding	noncoding
MSTRG.59124.1	LOC101928721	XLOC_065873	459	coding	noncoding	noncoding	noncoding
MSTRG.59122.1		XLOC_065875	219	coding	noncoding	noncoding	noncoding
MSTRG.59207.1	RELL1	XLOC_065890	251	coding	noncoding	noncoding	noncoding
MSTRG.59229.1		XLOC_065924	297	coding	noncoding	noncoding	noncoding
MSTRG.59236.1	LINC01258	XLOC_065928	289	coding	noncoding	noncoding	noncoding
MSTRG.59264.1	KLF3-AS1	XLOC_065934	11427	coding	coding	coding	noncoding
MSTRG.59253.1		XLOC_065935	377	coding	noncoding	noncoding	noncoding
MSTRG.59391.1	KLHL5	XLOC_065964	1274	coding	noncoding	noncoding	noncoding
MSTRG.59428.1	UGDH	XLOC_065978	274	coding	noncoding	noncoding	noncoding
NM_001317897.2	SMIM14	XLOC_065983	6263	coding	coding	coding	noncoding
MSTRG.59438.6	UBE2K	XLOC_065984	677	coding	coding	noncoding	noncoding
MSTRG.59438.10	UBE2K	XLOC_065984	2094	coding	coding	noncoding	noncoding
MSTRG.59470.1	PDS5A	XLOC_066001	248	coding	noncoding	noncoding	noncoding
MSTRG.59499.1	N4BP2	XLOC_066011	299	coding	noncoding	noncoding	noncoding
MSTRG.59506.2	RHOH	XLOC_066016	2406	coding	coding	coding	coding
MSTRG.59472.1		XLOC_066021	437	coding	noncoding	noncoding	noncoding
MSTRG.59474.1		XLOC_066022	211	coding	noncoding	noncoding	noncoding
MSTRG.59483.1		XLOC_066027	276	coding	noncoding	noncoding	noncoding
XM_005248103.4	RBM47	XLOC_066028	4884	coding	coding	coding	coding
XM_017008304.2	RBM47	XLOC_066028	6019	coding	coding	coding	coding

XM_011513708.2	RBM47	XLOC_066028	3701 coding	coding	coding	coding
MSTRG.59525.1		XLOC_066050	280 coding	noncoding	noncoding	noncoding
XR_001741664.1	LOC107986273	XLOC_066058	4566 coding	noncoding	noncoding	noncoding
MSTRG.59594.1	APBB2	XLOC_066065	215 coding	coding	noncoding	noncoding
MSTRG.59630.1	LIMCH1	XLOC_066083	246 coding	noncoding	noncoding	noncoding
MSTRG.59605.1	LOC105374425	XLOC_066089	395 coding	noncoding	noncoding	noncoding
MSTRG.59675.1		XLOC_066118	228 coding	noncoding	noncoding	noncoding
MSTRG.59816.1	ATP8A1	XLOC_066129	300 coding	noncoding	noncoding	noncoding
MSTRG.59707.1		XLOC_066168	253 coding	noncoding	noncoding	noncoding
MSTRG.59709.1		XLOC_066171	275 coding	noncoding	noncoding	noncoding
MSTRG.59735.1		XLOC_066184	256 coding	noncoding	noncoding	noncoding
MSTRG.59738.1		XLOC_066185	292 coding	noncoding	noncoding	noncoding
MSTRG.59752.1	LOC105374438	XLOC_066192	278 coding	noncoding	noncoding	noncoding
XM_011513690.3	KCTD8	XLOC_066197	3662 coding	coding	coding	coding
MSTRG.59758.1		XLOC_066204	336 coding	noncoding	noncoding	noncoding
XM_011513679.2	YIPF7	XLOC_066207	9037 coding	coding	coding	coding
MSTRG.59855.1		XLOC_066225	267 coding	noncoding	noncoding	noncoding
MSTRG.59857.1		XLOC_066227	299 coding	noncoding	noncoding	noncoding
MSTRG.59868.1		XLOC_066232	284 coding	noncoding	noncoding	noncoding
MSTRG.60094.1	CNGA1	XLOC_066287	241 coding	noncoding	noncoding	noncoding
MSTRG.60016.1	TXK	XLOC_066311	355 coding	noncoding	noncoding	noncoding
MSTRG.60021.1	TEC	XLOC_066316	289 coding	noncoding	noncoding	noncoding
MSTRG.59973.1		XLOC_066338	362 coding	noncoding	noncoding	noncoding
XM_011513682.3	FRYL	XLOC_066343	11747 coding	coding	coding	coding
XM_011513681.3	FRYL	XLOC_066343	11793 coding	coding	coding	coding
MSTRG.60249.1	FRYL	XLOC_066354	255 coding	noncoding	noncoding	noncoding
MSTRG.60189.3	DCUN1D4	XLOC_066424	1634 coding	coding	noncoding	noncoding
MSTRG.60520.1	SCFD2	XLOC_066464	280 coding	noncoding	noncoding	noncoding
MSTRG.60544.1	SCFD2	XLOC_066488	377 coding	coding	noncoding	noncoding
MSTRG.60548.5	FIP1L1	XLOC_066490	1951 coding	coding	coding	coding
MSTRG.60304.1		XLOC_066499	319 coding	noncoding	noncoding	noncoding
MSTRG.60307.1		XLOC_066500	229 coding	noncoding	noncoding	noncoding
MSTRG.60339.1		XLOC_066506	250 coding	noncoding	noncoding	noncoding
MSTRG.60346.1		XLOC_066537	272 coding	noncoding	noncoding	noncoding
MSTRG.60351.1		XLOC_066539	257 coding	noncoding	noncoding	noncoding
MSTRG.60360.1	PDGFRA	XLOC_066540	268 coding	noncoding	noncoding	noncoding
MSTRG.60361.1	PDGFRA	XLOC_066541	586 coding	coding	noncoding	noncoding
MSTRG.60357.1		XLOC_066546	348 coding	noncoding	noncoding	noncoding
MSTRG.60432.1	KIT	XLOC_066557	219 coding	noncoding	noncoding	noncoding
MSTRG.60423.1	LOC105377657	XLOC_066567	259 coding	noncoding	noncoding	noncoding
MSTRG.60444.1		XLOC_066574	322 coding	noncoding	noncoding	noncoding
NM_002253.3	KDR	XLOC_066576	5833 coding	coding	coding	coding
MSTRG.60467.2	SRD5A3	XLOC_066586	327 coding	noncoding	coding	noncoding

MSTRG.60482.7	CLOCK	XLOC_066590	7571 coding	coding	coding	coding
MSTRG.60475.1	PDCL2	XLOC_066593	361 coding	noncoding	noncoding	noncoding
MSTRG.60478.1		XLOC_066595	293 coding	coding	noncoding	noncoding
MSTRG.60506.1	KIAA1211	XLOC_066609	252 coding	noncoding	noncoding	noncoding
XM_017007740.2	AASDH	XLOC_066612	4620 coding	coding	coding	coding
MSTRG.60665.1	LOC100506514	XLOC_066617	286 coding	noncoding	noncoding	noncoding
NM_001145460.1	HOPX	XLOC_066625	1673 coding	noncoding	coding	coding
MSTRG.60622.1		XLOC_066634	213 coding	noncoding	noncoding	noncoding
MSTRG.60628.1		XLOC_066635	293 coding	noncoding	noncoding	noncoding
MSTRG.60629.1		XLOC_066636	283 coding	noncoding	noncoding	noncoding
MSTRG.60649.1		XLOC_066651	215 coding	coding	noncoding	noncoding
MSTRG.60687.1		XLOC_066656	478 coding	noncoding	noncoding	noncoding
MSTRG.60695.1		XLOC_066665	297 coding	noncoding	noncoding	noncoding
MSTRG.60699.1		XLOC_066666	228 coding	noncoding	noncoding	noncoding
MSTRG.60735.1		XLOC_066681	269 coding	noncoding	noncoding	noncoding
MSTRG.60740.1		XLOC_066684	386 coding	noncoding	noncoding	noncoding
MSTRG.60749.1		XLOC_066689	283 coding	noncoding	noncoding	noncoding
MSTRG.60767.1		XLOC_066699	306 coding	noncoding	noncoding	noncoding
MSTRG.60785.1		XLOC_066710	265 coding	noncoding	noncoding	noncoding
MSTRG.60801.1		XLOC_066718	251 coding	noncoding	coding	noncoding
MSTRG.60802.1		XLOC_066719	289 coding	noncoding	noncoding	noncoding
MSTRG.60804.1	LINC02496	XLOC_066720	302 coding	noncoding	noncoding	noncoding
MSTRG.60807.1		XLOC_066722	294 coding	noncoding	coding	noncoding
MSTRG.60813.1		XLOC_066726	281 coding	noncoding	coding	noncoding
MSTRG.60887.1	ADGRL3-AS1	XLOC_066740	241 coding	noncoding	noncoding	noncoding
MSTRG.60826.1		XLOC_066742	309 coding	noncoding	noncoding	noncoding
MSTRG.60829.1		XLOC_066744	319 coding	noncoding	noncoding	noncoding
MSTRG.60832.1		XLOC_066747	306 coding	noncoding	noncoding	noncoding
MSTRG.60855.1		XLOC_066759	255 coding	noncoding	noncoding	noncoding
XR_938815.2	LOC105377253	XLOC_066761	4617 coding	noncoding	noncoding	noncoding
MSTRG.60897.1		XLOC_066764	232 coding	noncoding	noncoding	noncoding
MSTRG.60892.1		XLOC_066767	301 coding	coding	noncoding	noncoding
MSTRG.60914.1		XLOC_066774	338 coding	noncoding	noncoding	noncoding
MSTRG.60915.1		XLOC_066775	261 coding	noncoding	noncoding	noncoding
MSTRG.60919.1		XLOC_066776	257 coding	noncoding	noncoding	noncoding
MSTRG.60923.1		XLOC_066778	244 coding	noncoding	noncoding	noncoding
MSTRG.60926.1		XLOC_066779	293 coding	noncoding	noncoding	noncoding
MSTRG.60948.1	LOC107986284	XLOC_066791	288 coding	noncoding	noncoding	noncoding
MSTRG.60950.1	LOC107986284	XLOC_066793	235 coding	noncoding	noncoding	noncoding
NR_033976.1	LINC02232	XLOC_066795	4079 coding	noncoding	noncoding	noncoding
MSTRG.60967.1		XLOC_066807	286 coding	noncoding	noncoding	noncoding
MSTRG.60970.1		XLOC_066809	288 coding	noncoding	noncoding	noncoding
MSTRG.61021.1	LOC105377262	XLOC_066827	269 coding	noncoding	noncoding	noncoding

MSTRG.61019.1		XLOC_066834	346	coding	noncoding	noncoding	noncoding
MSTRG.61034.1		XLOC_066835	224	coding	noncoding	noncoding	noncoding
MSTRG.61036.1		XLOC_066836	265	coding	noncoding	noncoding	noncoding
MSTRG.61064.1	CENPC	XLOC_066854	430	coding	noncoding	noncoding	noncoding
MSTRG.61162.1		XLOC_066891	283	coding	coding	noncoding	noncoding
NM_001031732.4	YTHDC1	XLOC_066938	6233	coding	coding	coding	coding
XR_001741714.1	LOC105377265	XLOC_066949	4109	coding	coding	coding	noncoding
MSTRG.61208.1		XLOC_066962	244	coding	noncoding	noncoding	noncoding
MSTRG.61209.1		XLOC_066963	253	coding	noncoding	noncoding	noncoding
MSTRG.61274.1	SMR3B	XLOC_066987	232	coding	noncoding	noncoding	noncoding
MSTRG.61302.1		XLOC_067004	255	coding	noncoding	noncoding	noncoding
MSTRG.61395.1	SLC4A4	XLOC_067014	266	coding	noncoding	noncoding	noncoding
MSTRG.61372.1	ADAMTS3	XLOC_067053	316	coding	noncoding	noncoding	noncoding
MSTRG.61375.1	ADAMTS3	XLOC_067055	259	coding	noncoding	noncoding	noncoding
MSTRG.61359.1		XLOC_067060	289	coding	noncoding	noncoding	noncoding
MSTRG.61362.1		XLOC_067062	244	coding	noncoding	noncoding	noncoding
MSTRG.61384.1		XLOC_067066	667	coding	noncoding	noncoding	noncoding
MSTRG.61668.1	LOC102724832	XLOC_067099	553	coding	noncoding	noncoding	noncoding
XM_017007838.1	RASSF6	XLOC_067107	3323	coding	noncoding	coding	coding
MSTRG.61455.1		XLOC_067108	402	coding	noncoding	noncoding	noncoding
MSTRG.61483.1		XLOC_067114	250	coding	noncoding	noncoding	noncoding
NM_001363352.1	PF4	XLOC_067118	1061	coding	coding	noncoding	noncoding
MSTRG.61489.1		XLOC_067126	277	coding	noncoding	noncoding	noncoding
MSTRG.61516.1		XLOC_067168	393	coding	noncoding	noncoding	noncoding
NM_001729.4	BTC	XLOC_067175	2653	coding	noncoding	noncoding	coding
MSTRG.61535.1	LOC105377282	XLOC_067180	238	coding	noncoding	noncoding	noncoding
NM_001330724.2	CDKL2	XLOC_067207	4935	coding	coding	coding	coding
MSTRG.61715.11	G3BP2	XLOC_067208	2780	coding	coding	coding	coding
MSTRG.61723.4	USO1	XLOC_067213	3137	coding	coding	noncoding	coding
MSTRG.61713.1	LOC105377285	XLOC_067218	321	coding	noncoding	noncoding	noncoding
MSTRG.61746.1	SDAD1	XLOC_067224	272	coding	noncoding	noncoding	noncoding
NM_001204255.2	SCARB2	XLOC_067235	4265	coding	coding	coding	coding
MSTRG.61765.1	FAM47E	XLOC_067239	271	coding	noncoding	noncoding	noncoding
XM_011531910.3	CCDC158	XLOC_067244	4381	coding	coding	coding	coding
MSTRG.61777.1	CCDC158	XLOC_067246	237	coding	noncoding	noncoding	noncoding
MSTRG.61786.1	SHROOM3	XLOC_067248	220	coding	noncoding	noncoding	noncoding
MSTRG.61803.1	SHROOM3	XLOC_067259	290	coding	noncoding	noncoding	noncoding
MSTRG.61806.1	SHROOM3	XLOC_067262	230	coding	noncoding	noncoding	noncoding
MSTRG.61808.1	SHROOM3	XLOC_067264	242	coding	noncoding	noncoding	noncoding
MSTRG.61810.1	LOC105377289	XLOC_067266	224	coding	noncoding	noncoding	noncoding
NM_001029870.3	SOWAHB	XLOC_067268	3993	coding	coding	coding	coding
MSTRG.61894.2	CCNI	XLOC_067277	2156	coding	coding	coding	coding
NM_001348133.1	CCNI	XLOC_067277	1505	coding	coding	coding	coding

MSTRG.61820.1	LOC107986292	XLOC_067286	311	coding	noncoding	noncoding	noncoding
MSTRG.61953.1		XLOC_067354	294	coding	noncoding	noncoding	noncoding
MSTRG.62041.3	ANXA3	XLOC_067394	878	coding	coding	noncoding	noncoding
XM_017008779.1	NAA11	XLOC_067417	2518	coding	coding	coding	coding
MSTRG.62112.1		XLOC_067429	217	coding	noncoding	noncoding	noncoding
MSTRG.62110.1	LOC105377302	XLOC_067435	269	coding	noncoding	noncoding	noncoding
MSTRG.62192.1	CFAP299	XLOC_067485	253	coding	coding	noncoding	noncoding
MSTRG.62264.1		XLOC_067512	275	coding	noncoding	noncoding	noncoding
MSTRG.62303.1	RASGEF1B	XLOC_067515	215	coding	noncoding	noncoding	noncoding
MSTRG.62306.1	RASGEF1B	XLOC_067518	244	coding	noncoding	noncoding	noncoding
MSTRG.62299.1		XLOC_067524	255	coding	noncoding	noncoding	noncoding
MSTRG.62332.1	LOC107986215	XLOC_067531	258	coding	noncoding	noncoding	noncoding
MSTRG.62316.1		XLOC_067538	269	coding	noncoding	noncoding	noncoding
MSTRG.62361.1		XLOC_067542	272	coding	noncoding	noncoding	noncoding
NM_002138.3	HNRNPD	XLOC_067546	2110	coding	coding	coding	coding
MSTRG.62349.1		XLOC_067552	267	coding	noncoding	noncoding	noncoding
MSTRG.62378.1		XLOC_067556	289	coding	noncoding	noncoding	noncoding
MSTRG.62356.1	TMEM150C	XLOC_067561	228	coding	noncoding	noncoding	noncoding
NM_001288996.2	LIN54	XLOC_067584	5854	coding	coding	coding	coding
MSTRG.62413.1	LIN54	XLOC_067586	274	coding	noncoding	noncoding	noncoding
MSTRG.62415.1	LIN54	XLOC_067588	301	coding	noncoding	noncoding	noncoding
MSTRG.62388.1		XLOC_067591	209	coding	noncoding	noncoding	noncoding
NM_001297758.1	HELQ	XLOC_067623	2456	coding	noncoding	coding	coding
MSTRG.62539.3	ABRAXAS1	XLOC_067624	4195	coding	coding	noncoding	coding
MSTRG.62508.1		XLOC_067643	241	coding	coding	noncoding	noncoding
MSTRG.62655.1	MAPK10	XLOC_067728	332	coding	noncoding	noncoding	noncoding
MSTRG.62579.1		XLOC_067756	247	coding	noncoding	noncoding	noncoding
MSTRG.62827.1		XLOC_067759	201	coding	noncoding	noncoding	noncoding
MSTRG.62836.1	AFF1	XLOC_067761	12726	coding	coding	coding	coding
MSTRG.62836.10	AFF1	XLOC_067761	7698	coding	coding	coding	coding
MSTRG.62836.17	AFF1	XLOC_067761	6183	coding	noncoding	noncoding	noncoding
MSTRG.62711.1		XLOC_067786	229	coding	noncoding	noncoding	noncoding
NM_001291976.1	SPARCL1	XLOC_067791	3019	coding	coding	coding	coding
MSTRG.62730.1		XLOC_067797	233	coding	noncoding	noncoding	noncoding
MSTRG.62744.1		XLOC_067799	257	coding	noncoding	noncoding	noncoding
MSTRG.62745.1		XLOC_067800	359	coding	noncoding	noncoding	noncoding
MSTRG.62756.1		XLOC_067802	295	coding	noncoding	noncoding	noncoding
MSTRG.62761.1	MEPE	XLOC_067805	210	coding	noncoding	noncoding	noncoding
MSTRG.62882.1	ABCG2	XLOC_067813	249	coding	noncoding	noncoding	noncoding
MSTRG.62884.1	ABCG2	XLOC_067815	241	coding	noncoding	noncoding	noncoding
MSTRG.62885.1	ABCG2	XLOC_067816	227	coding	noncoding	noncoding	noncoding
MSTRG.62893.1	PPM1K-DT	XLOC_067820	277	coding	noncoding	noncoding	noncoding
NM_001042616.2	PIGY	XLOC_067829	1356	coding	coding	coding	noncoding

MSTRG.62900.1		XLOC_067830	251	coding	noncoding	noncoding	noncoding
MSTRG.62921.1		XLOC_067842	236	coding	noncoding	noncoding	noncoding
XM_017008044.1	GPRIN3	XLOC_067844	12762	coding	coding	coding	coding
MSTRG.62943.1	LOC105377329	XLOC_067866	314	coding	noncoding	noncoding	noncoding
MSTRG.63292.1	CCSER1	XLOC_067953	214	coding	noncoding	noncoding	noncoding
MSTRG.63066.1	GRID2	XLOC_068019	201	coding	noncoding	noncoding	noncoding
MSTRG.63100.1	GRID2	XLOC_068033	272	coding	coding	noncoding	noncoding
MSTRG.63103.1	GRID2	XLOC_068035	223	coding	noncoding	noncoding	noncoding
MSTRG.63107.1	GRID2	XLOC_068037	239	coding	noncoding	noncoding	noncoding
MSTRG.63119.1	BMPR1B	XLOC_068064	259	coding	noncoding	noncoding	noncoding
MSTRG.63121.1	BMPR1B	XLOC_068066	282	coding	noncoding	noncoding	noncoding
MSTRG.63167.1	LINC02267	XLOC_068086	397	coding	noncoding	noncoding	noncoding
MSTRG.63175.1	LINC02267	XLOC_068092	224	coding	noncoding	noncoding	noncoding
MSTRG.63159.1		XLOC_068098	280	coding	noncoding	noncoding	noncoding
MSTRG.63417.1	TSPAN5	XLOC_068125	321	coding	noncoding	noncoding	noncoding
MSTRG.63407.1	LOC105377343	XLOC_068136	259	coding	noncoding	noncoding	noncoding
MSTRG.63411.1	LOC105377343	XLOC_068138	313	coding	noncoding	noncoding	noncoding
MSTRG.63526.1		XLOC_068182	276	coding	noncoding	noncoding	noncoding
XM_373030.11	C4orf54	XLOC_068189	10526	coding	coding	coding	coding
MSTRG.63591.1		XLOC_068226	261	coding	noncoding	noncoding	noncoding
MSTRG.63626.1		XLOC_068243	400	coding	noncoding	noncoding	noncoding
MSTRG.63684.1		XLOC_068360	265	coding	noncoding	noncoding	noncoding
MSTRG.63712.1		XLOC_068382	263	coding	coding	noncoding	noncoding
XM_017008205.2	MANBA	XLOC_068396	5264	coding	coding	coding	coding
MSTRG.63974.1	MANBA	XLOC_068402	271	coding	noncoding	noncoding	noncoding
MSTRG.63995.1	MANBA	XLOC_068423	278	coding	noncoding	noncoding	noncoding
NM_181892.3	UBE2D3	XLOC_068428	4201	coding	coding	noncoding	coding
NM_178833.7	SLC9B2	XLOC_068432	6351	coding	coding	coding	coding
MSTRG.64094.1	SLC9B1	XLOC_068437	252	coding	noncoding	noncoding	noncoding
MSTRG.64098.1	SLC9B1	XLOC_068439	317	coding	coding	noncoding	noncoding
MSTRG.64105.1	SLC9B1	XLOC_068440	459	coding	noncoding	noncoding	noncoding
MSTRG.64116.1	SLC9B2	XLOC_068443	319	coding	noncoding	noncoding	noncoding
MSTRG.63876.1		XLOC_068449	278	coding	coding	noncoding	noncoding
MSTRG.63886.1		XLOC_068454	221	coding	noncoding	noncoding	noncoding
MSTRG.63892.1	TACR3	XLOC_068457	535	coding	noncoding	noncoding	noncoding
MSTRG.64028.1	CXXC4-AS1	XLOC_068480	295	coding	coding	noncoding	noncoding
MSTRG.64035.1		XLOC_068487	293	coding	noncoding	noncoding	noncoding
MSTRG.64048.1		XLOC_068495	277	coding	noncoding	noncoding	noncoding
MSTRG.64265.9	TET2	XLOC_068518	12800	coding	coding	coding	noncoding
MSTRG.64265.15	TET2	XLOC_068518	9834	coding	coding	coding	noncoding
MSTRG.64265.29	TET2-AS1	XLOC_068518	1220	coding	noncoding	noncoding	noncoding
MSTRG.64265.32	TET2	XLOC_068518	10049	coding	coding	noncoding	noncoding
MSTRG.64190.1		XLOC_068582	303	coding	noncoding	noncoding	noncoding

MSTRG.64192.1	LINC02173	XLOC_068583	290	coding	noncoding	noncoding	noncoding
MSTRG.64194.1		XLOC_068584	429	coding	noncoding	noncoding	noncoding
MSTRG.64237.1		XLOC_068604	264	coding	coding	noncoding	noncoding
MSTRG.64246.1		XLOC_068607	260	coding	noncoding	noncoding	noncoding
MSTRG.64255.1	LOC105377357	XLOC_068610	303	coding	noncoding	noncoding	noncoding
MSTRG.64306.1		XLOC_068626	323	coding	noncoding	noncoding	noncoding
MSTRG.64309.1		XLOC_068628	243	coding	noncoding	noncoding	noncoding
MSTRG.64312.1		XLOC_068630	275	coding	noncoding	noncoding	noncoding
MSTRG.64401.1	RPL34-AS1	XLOC_068702	264	coding	noncoding	noncoding	noncoding
MSTRG.64462.1	COL25A1	XLOC_068732	276	coding	coding	noncoding	noncoding
MSTRG.64580.1	CFI	XLOC_068765	267	coding	noncoding	noncoding	noncoding
MSTRG.64642.1	ELOVL6	XLOC_068783	282	coding	noncoding	noncoding	noncoding
MSTRG.64643.1	ELOVL6	XLOC_068784	405	coding	noncoding	noncoding	noncoding
MSTRG.64646.1	ELOVL6	XLOC_068787	286	coding	noncoding	noncoding	noncoding
MSTRG.64635.1		XLOC_068793	260	coding	noncoding	noncoding	noncoding
MSTRG.64666.1	ENPEP	XLOC_068800	237	coding	noncoding	noncoding	noncoding
MSTRG.64648.1		XLOC_068801	257	coding	noncoding	noncoding	noncoding
MSTRG.64685.1		XLOC_068814	248	coding	noncoding	noncoding	noncoding
MSTRG.64687.1		XLOC_068815	224	coding	noncoding	noncoding	noncoding
MSTRG.64734.1		XLOC_068835	500	coding	noncoding	noncoding	noncoding
MSTRG.64744.1		XLOC_068839	223	coding	noncoding	noncoding	noncoding
MSTRG.64773.21	ALPK1	XLOC_068853	4450	coding	noncoding	noncoding	noncoding
NM_024019.4	NEUROG2	XLOC_068857	2295	coding	coding	coding	coding
MSTRG.64836.1	ANK2	XLOC_068865	374	coding	noncoding	noncoding	noncoding
MSTRG.64842.1	ANK2	XLOC_068869	209	coding	noncoding	noncoding	noncoding
MSTRG.64834.1	LOC105377374	XLOC_068881	296	coding	noncoding	noncoding	noncoding
MSTRG.64865.1		XLOC_068931	317	coding	noncoding	noncoding	noncoding
MSTRG.64861.1		XLOC_068932	294	coding	coding	noncoding	noncoding
MSTRG.64879.1		XLOC_068938	307	coding	noncoding	noncoding	noncoding
MSTRG.64888.1		XLOC_068943	310	coding	noncoding	noncoding	noncoding
MSTRG.64891.1	LOC107986235	XLOC_068945	225	coding	noncoding	noncoding	noncoding
MSTRG.64900.1		XLOC_068950	232	coding	noncoding	noncoding	noncoding
MSTRG.64918.1		XLOC_068964	270	coding	noncoding	noncoding	noncoding
MSTRG.64925.1		XLOC_068977	306	coding	noncoding	noncoding	noncoding
MSTRG.64959.1		XLOC_068982	295	coding	noncoding	noncoding	noncoding
MSTRG.64960.1		XLOC_068983	493	coding	noncoding	noncoding	noncoding
MSTRG.64963.1		XLOC_068985	251	coding	noncoding	noncoding	noncoding
MSTRG.64987.1		XLOC_069000	218	coding	noncoding	noncoding	noncoding
MSTRG.64990.1		XLOC_069002	253	coding	noncoding	noncoding	noncoding
MSTRG.64991.1		XLOC_069003	322	coding	noncoding	noncoding	noncoding
MSTRG.65011.1		XLOC_069013	258	coding	noncoding	noncoding	noncoding
MSTRG.65012.1		XLOC_069014	213	coding	noncoding	noncoding	noncoding
MSTRG.65053.1	LINC02264	XLOC_069027	275	coding	coding	noncoding	noncoding

MSTRG.65141.1	NDST3	XLOC_069034	294	coding	coding	noncoding	noncoding
MSTRG.65148.1	NDST3	XLOC_069040	319	coding	noncoding	noncoding	noncoding
MSTRG.65225.1	SEC24D	XLOC_069069	224	coding	noncoding	noncoding	noncoding
MSTRG.65227.1	SEC24D	XLOC_069071	438	coding	noncoding	noncoding	noncoding
MSTRG.65203.1	SYNPO2	XLOC_069083	255	coding	noncoding	noncoding	noncoding
MSTRG.65190.1		XLOC_069087	233	coding	coding	noncoding	noncoding
MSTRG.65277.1		XLOC_069117	295	coding	noncoding	noncoding	noncoding
MSTRG.65280.1		XLOC_069118	267	coding	noncoding	noncoding	noncoding
MSTRG.65282.1		XLOC_069120	383	coding	noncoding	noncoding	noncoding
XM_024454212.1	NDNF	XLOC_069165	8926	coding	coding	coding	coding
MSTRG.65389.1		XLOC_069180	258	coding	noncoding	noncoding	noncoding
MSTRG.65407.1		XLOC_069188	322	coding	coding	noncoding	noncoding
MSTRG.65512.1	ADAD1	XLOC_069196	280	coding	noncoding	noncoding	noncoding
MSTRG.65532.1		XLOC_069209	282	coding	noncoding	noncoding	noncoding
MSTRG.65537.1		XLOC_069212	324	coding	noncoding	noncoding	noncoding
MSTRG.65585.1	LOC105377406	XLOC_069241	314	coding	noncoding	noncoding	noncoding
MSTRG.65632.1		XLOC_069263	228	coding	noncoding	noncoding	noncoding
MSTRG.65711.1		XLOC_069286	514	coding	noncoding	noncoding	noncoding
MSTRG.65716.1		XLOC_069288	250	coding	noncoding	noncoding	noncoding
MSTRG.65717.1		XLOC_069289	313	coding	noncoding	noncoding	noncoding
MSTRG.65724.1		XLOC_069293	307	coding	noncoding	noncoding	noncoding
MSTRG.65762.1	LOC102724210	XLOC_069308	282	coding	noncoding	noncoding	noncoding
MSTRG.65747.1		XLOC_069312	286	coding	noncoding	noncoding	noncoding
MSTRG.65773.1	SLC25A31	XLOC_069319	345	coding	noncoding	noncoding	noncoding
MSTRG.65799.1		XLOC_069325	227	coding	coding	noncoding	noncoding
MSTRG.65869.4	LARP1B	XLOC_069335	665	coding	noncoding	noncoding	noncoding
MSTRG.66399.4	JADE1	XLOC_069367	2203	coding	coding	coding	noncoding
MSTRG.65947.1		XLOC_069392	240	coding	noncoding	noncoding	noncoding
MSTRG.65951.1		XLOC_069394	309	coding	noncoding	noncoding	noncoding
MSTRG.65953.1	LINC02479	XLOC_069395	281	coding	noncoding	noncoding	noncoding
MSTRG.65956.1		XLOC_069396	262	coding	noncoding	noncoding	noncoding
MSTRG.66000.1		XLOC_069417	321	coding	noncoding	noncoding	noncoding
MSTRG.66013.1		XLOC_069427	238	coding	noncoding	noncoding	noncoding
MSTRG.66015.1		XLOC_069429	275	coding	noncoding	noncoding	noncoding
MSTRG.66048.1		XLOC_069434	312	coding	noncoding	noncoding	noncoding
MSTRG.66057.1	LOC101927359	XLOC_069440	320	coding	noncoding	noncoding	noncoding
MSTRG.66079.1		XLOC_069452	318	coding	noncoding	noncoding	noncoding
NM_001363585.1	PABPC4L	XLOC_069457	5040	coding	coding	coding	coding
MSTRG.66109.1	PABPC4L	XLOC_069463	297	coding	noncoding	noncoding	noncoding
MSTRG.66110.1	PABPC4L	XLOC_069464	249	coding	noncoding	noncoding	noncoding
MSTRG.66097.1		XLOC_069467	271	coding	noncoding	noncoding	noncoding
MSTRG.66122.1		XLOC_069472	481	coding	noncoding	noncoding	noncoding
MSTRG.66125.1		XLOC_069474	253	coding	noncoding	noncoding	noncoding

MSTRG.66140.1		XLOC_069484	415 coding	noncoding	noncoding	noncoding
MSTRG.66147.1		XLOC_069488	300 coding	noncoding	noncoding	noncoding
MSTRG.66173.1		XLOC_069501	276 coding	coding	noncoding	noncoding
MSTRG.66194.1	LINC02511	XLOC_069506	300 coding	noncoding	noncoding	noncoding
MSTRG.66201.1	LINC02511	XLOC_069511	225 coding	noncoding	noncoding	noncoding
XM_017008311.1	PCDH18	XLOC_069523	5958 coding	coding	coding	coding
MSTRG.66268.1	SLC7A11	XLOC_069540	258 coding	noncoding	noncoding	noncoding
MSTRG.66308.1	LOC105377448	XLOC_069553	286 coding	noncoding	noncoding	noncoding
MSTRG.66310.1	LOC105377448	XLOC_069554	245 coding	noncoding	noncoding	noncoding
MSTRG.66324.1	LOC105377448	XLOC_069555	273 coding	noncoding	noncoding	noncoding
MSTRG.66274.1	LOC105379412	XLOC_069594	205 coding	noncoding	noncoding	noncoding
MSTRG.66278.1	MGARP	XLOC_069597	253 coding	noncoding	noncoding	noncoding
MSTRG.66280.1	MGARP	XLOC_069598	296 coding	noncoding	noncoding	noncoding
MSTRG.66394.1		XLOC_069614	361 coding	noncoding	noncoding	noncoding
MSTRG.66623.1	LOC101927490	XLOC_069625	266 coding	noncoding	noncoding	noncoding
MSTRG.66637.1	MAML3	XLOC_069634	260 coding	noncoding	noncoding	noncoding
MSTRG.66410.1		XLOC_069704	346 coding	noncoding	noncoding	noncoding
MSTRG.66412.1		XLOC_069705	292 coding	noncoding	noncoding	noncoding
MSTRG.66483.1	TBC1D9	XLOC_069724	249 coding	noncoding	noncoding	noncoding
MSTRG.66467.1	RNF150	XLOC_069735	284 coding	noncoding	noncoding	noncoding
MSTRG.66510.1	LOC107986194	XLOC_069824	222 coding	noncoding	noncoding	noncoding
MSTRG.66755.1	FREM3	XLOC_069843	264 coding	coding	noncoding	noncoding
XM_011531903.2	GYPB	XLOC_069853	552 coding	coding	noncoding	coding
XM_011531904.3	GYPB	XLOC_069853	3057 coding	coding	noncoding	noncoding
MSTRG.66720.1	LOC105377462	XLOC_069863	275 coding	noncoding	noncoding	noncoding
MSTRG.66815.27	ABCE1	XLOC_069875	1034 coding	coding	noncoding	noncoding
MSTRG.66789.1		XLOC_069887	306 coding	noncoding	noncoding	noncoding
MSTRG.66793.1		XLOC_069890	422 coding	noncoding	noncoding	noncoding
MSTRG.66799.1		XLOC_069897	228 coding	noncoding	noncoding	noncoding
MSTRG.66801.1		XLOC_069898	317 coding	noncoding	noncoding	noncoding
MSTRG.66947.1	C4orf51	XLOC_069909	295 coding	noncoding	noncoding	noncoding
MSTRG.66854.1	LOC105377470	XLOC_069925	285 coding	noncoding	noncoding	noncoding
MSTRG.66857.1		XLOC_069938	243 coding	noncoding	noncoding	noncoding
MSTRG.67015.1	SLC10A7	XLOC_069944	303 coding	noncoding	noncoding	noncoding
MSTRG.66978.1	TTC29	XLOC_069967	259 coding	noncoding	noncoding	noncoding
MSTRG.66979.1	TTC29	XLOC_069968	246 coding	noncoding	noncoding	noncoding
MSTRG.66974.1		XLOC_069976	237 coding	noncoding	noncoding	noncoding
MSTRG.66975.1		XLOC_069977	226 coding	noncoding	noncoding	noncoding
MSTRG.67005.1	LINC02507	XLOC_069988	365 coding	noncoding	noncoding	noncoding
MSTRG.67417.1		XLOC_069990	274 coding	noncoding	noncoding	noncoding
MSTRG.67420.4	ARHGAP10	XLOC_069992	2386 coding	coding	noncoding	coding
MSTRG.67450.1	NR3C2	XLOC_070000	305 coding	coding	noncoding	noncoding
MSTRG.67462.1	NR3C2	XLOC_070011	243 coding	noncoding	noncoding	noncoding

MSTRG.67056.1	LINC02355	XLOC_070102	251	coding	noncoding	noncoding	noncoding
MSTRG.67099.1	DCLK2	XLOC_070119	291	coding	noncoding	noncoding	noncoding
MSTRG.67244.1	LRBA	XLOC_070136	234	coding	noncoding	noncoding	noncoding
MSTRG.67269.1	LRBA	XLOC_070160	201	coding	noncoding	noncoding	noncoding
MSTRG.67272.1	LRBA	XLOC_070163	276	coding	noncoding	noncoding	noncoding
MSTRG.67283.1	LRBA	XLOC_070174	221	coding	noncoding	noncoding	noncoding
MSTRG.67284.1	LRBA	XLOC_070175	298	coding	noncoding	noncoding	noncoding
MSTRG.67101.1		XLOC_070178	243	coding	noncoding	noncoding	noncoding
MSTRG.67102.1		XLOC_070179	269	coding	coding	noncoding	noncoding
MSTRG.67103.1		XLOC_070180	255	coding	noncoding	noncoding	noncoding
MSTRG.67107.1		XLOC_070182	256	coding	noncoding	noncoding	noncoding
MSTRG.67109.1		XLOC_070184	251	coding	noncoding	noncoding	noncoding
MSTRG.67119.1		XLOC_070196	264	coding	noncoding	noncoding	noncoding
MSTRG.67202.1	FAM160A1	XLOC_070216	436	coding	noncoding	noncoding	noncoding
MSTRG.67205.1	FAM160A1	XLOC_070219	284	coding	noncoding	noncoding	noncoding
MSTRG.67224.1	GATB	XLOC_070236	209	coding	noncoding	noncoding	noncoding
MSTRG.67173.1		XLOC_070254	300	coding	noncoding	noncoding	noncoding
MSTRG.67178.1	LOC105377490	XLOC_070257	305	coding	noncoding	noncoding	noncoding
NM_152680.3	TMEM154	XLOC_070269	10534	coding	coding	coding	coding
MSTRG.67319.1		XLOC_070292	266	coding	noncoding	noncoding	noncoding
MSTRG.67344.1	TRIM2	XLOC_070294	261	coding	noncoding	noncoding	noncoding
MSTRG.67352.1	TRIM2	XLOC_070300	235	coding	noncoding	noncoding	noncoding
MSTRG.67637.1	DCHS2	XLOC_070345	288	coding	noncoding	noncoding	noncoding
MSTRG.67674.1		XLOC_070368	356	coding	coding	noncoding	noncoding
MSTRG.67676.1	LOC105377505	XLOC_070369	241	coding	noncoding	noncoding	noncoding
MSTRG.67706.1		XLOC_070383	264	coding	noncoding	noncoding	noncoding
MSTRG.67730.1	GLRB	XLOC_070402	230	coding	noncoding	noncoding	noncoding
MSTRG.67733.1		XLOC_070409	237	coding	noncoding	noncoding	noncoding
MSTRG.67734.1		XLOC_070410	249	coding	noncoding	noncoding	noncoding
MSTRG.67756.1	LOC105377509	XLOC_070419	216	coding	noncoding	noncoding	noncoding
MSTRG.67794.1	LOC105377510	XLOC_070445	268	coding	noncoding	noncoding	noncoding
MSTRG.67799.1		XLOC_070448	253	coding	noncoding	noncoding	noncoding
XM_017007810.1	C4orf45	XLOC_070455	1824	coding	coding	noncoding	coding
MSTRG.67845.1	C4orf45	XLOC_070460	393	coding	noncoding	noncoding	noncoding
MSTRG.67870.1		XLOC_070481	272	coding	noncoding	noncoding	noncoding
MSTRG.67874.1		XLOC_070484	298	coding	coding	noncoding	noncoding
MSTRG.67923.1	FSTL5	XLOC_070503	277	coding	noncoding	noncoding	noncoding
MSTRG.67917.1		XLOC_070521	200	coding	noncoding	noncoding	noncoding
MSTRG.67970.1		XLOC_070531	234	coding	noncoding	noncoding	noncoding
MSTRG.67980.1		XLOC_070546	317	coding	noncoding	noncoding	noncoding
MSTRG.68132.1	1-Mar	XLOC_070561	214	coding	coding	noncoding	noncoding
MSTRG.68137.1	1-Mar	XLOC_070563	292	coding	noncoding	noncoding	noncoding
MSTRG.68029.1		XLOC_070579	248	coding	noncoding	noncoding	noncoding

MSTRG.68042.1		XLOC_070585	320 coding	noncoding	noncoding	noncoding
MSTRG.68044.1		XLOC_070588	214 coding	noncoding	noncoding	noncoding
MSTRG.68046.1		XLOC_070589	238 coding	noncoding	noncoding	noncoding
MSTRG.68048.1		XLOC_070590	311 coding	noncoding	noncoding	noncoding
MSTRG.68195.1	TLL1	XLOC_070617	239 coding	noncoding	noncoding	noncoding
MSTRG.68202.1		XLOC_070622	275 coding	noncoding	noncoding	noncoding
MSTRG.68207.1		XLOC_070625	235 coding	noncoding	noncoding	noncoding
MSTRG.68277.1	SPOCK3	XLOC_070631	301 coding	coding	noncoding	noncoding
MSTRG.68293.1		XLOC_070643	322 coding	noncoding	noncoding	noncoding
MSTRG.68426.1		XLOC_070667	284 coding	noncoding	noncoding	noncoding
XM_011532406.1	DDX60L	XLOC_070669	6679 coding	coding	coding	coding
MSTRG.68359.1	PALLD	XLOC_070723	214 coding	noncoding	noncoding	noncoding
MSTRG.68361.1	PALLD	XLOC_070724	217 coding	noncoding	noncoding	noncoding
MSTRG.68312.1		XLOC_070753	226 coding	noncoding	noncoding	noncoding
MSTRG.68325.1	SH3RF1	XLOC_070755	212 coding	noncoding	noncoding	noncoding
MSTRG.68321.1		XLOC_070766	235 coding	noncoding	noncoding	noncoding
NR_037878.1	LINC02275	XLOC_070787	4365 coding	noncoding	coding	noncoding
MSTRG.68539.1		XLOC_070801	206 coding	noncoding	noncoding	noncoding
MSTRG.68540.1		XLOC_070802	286 coding	coding	noncoding	noncoding
XR_001741531.1	LINC02504	XLOC_070809	4931 coding	noncoding	noncoding	noncoding
MSTRG.68593.1	GALNTL6	XLOC_070828	299 coding	noncoding	noncoding	noncoding
MSTRG.68595.1	GALNTL6	XLOC_070830	307 coding	noncoding	noncoding	noncoding
MSTRG.68597.1	GALNTL6	XLOC_070831	222 coding	noncoding	noncoding	noncoding
MSTRG.68617.1	GALNTL6	XLOC_070840	262 coding	noncoding	noncoding	noncoding
MSTRG.68622.1	GALNTL6	XLOC_070845	305 coding	coding	noncoding	noncoding
MSTRG.68573.1		XLOC_070851	290 coding	noncoding	noncoding	noncoding
MSTRG.68671.1		XLOC_070907	215 coding	noncoding	noncoding	noncoding
MSTRG.68676.1	LINC02268	XLOC_070912	339 coding	noncoding	noncoding	noncoding
MSTRG.68785.2	FBXO8	XLOC_070917	3932 coding	coding	coding	coding
MSTRG.68760.1	HPGD	XLOC_070934	426 coding	noncoding	noncoding	noncoding
XM_017008630.1	GLRA3	XLOC_070942	2888 coding	coding	coding	coding
MSTRG.68807.1		XLOC_070951	283 coding	noncoding	noncoding	noncoding
MSTRG.68824.1	GPM6A	XLOC_070957	252 coding	noncoding	noncoding	noncoding
MSTRG.68845.1	ASB5	XLOC_070972	350 coding	noncoding	noncoding	noncoding
MSTRG.68886.1	LOC105377555	XLOC_070987	260 coding	noncoding	noncoding	noncoding
MSTRG.68896.1		XLOC_070992	257 coding	noncoding	noncoding	noncoding
MSTRG.68973.1	LOC105377561	XLOC_071050	343 coding	noncoding	noncoding	noncoding
MSTRG.68984.1		XLOC_071055	295 coding	coding	noncoding	noncoding
MSTRG.69006.1		XLOC_071067	263 coding	noncoding	noncoding	noncoding
MSTRG.69011.1		XLOC_071071	263 coding	noncoding	noncoding	noncoding
MSTRG.69017.1		XLOC_071074	257 coding	coding	noncoding	noncoding
MSTRG.69029.1		XLOC_071081	271 coding	noncoding	noncoding	noncoding
MSTRG.69058.1	LOC105377567	XLOC_071088	227 coding	noncoding	noncoding	noncoding

MSTRG.69045.1		XLOC_071098	312	coding	noncoding	noncoding	noncoding
MSTRG.69138.1	TENM3	XLOC_071123	274	coding	noncoding	noncoding	noncoding
MSTRG.69152.1	TENM3	XLOC_071131	254	coding	noncoding	noncoding	noncoding
MSTRG.69157.1	TENM3	XLOC_071133	709	coding	noncoding	noncoding	noncoding
MSTRG.69164.1	TENM3	XLOC_071138	225	coding	noncoding	noncoding	noncoding
MSTRG.69174.1	TENM3	XLOC_071145	228	coding	noncoding	noncoding	noncoding
MSTRG.69185.1	TENM3	XLOC_071151	280	coding	noncoding	noncoding	noncoding
MSTRG.69088.1		XLOC_071152	269	coding	noncoding	noncoding	noncoding
MSTRG.69100.1	LOC107986327	XLOC_071161	314	coding	noncoding	noncoding	noncoding
MSTRG.69110.1	LOC105377578	XLOC_071167	268	coding	noncoding	noncoding	noncoding
MSTRG.69204.1		XLOC_071207	257	coding	noncoding	noncoding	noncoding
MSTRG.69208.1		XLOC_071209	319	coding	noncoding	noncoding	noncoding
MSTRG.69209.1		XLOC_071210	264	coding	noncoding	noncoding	noncoding
MSTRG.69256.1		XLOC_071225	302	coding	noncoding	noncoding	noncoding
XR_002959716.1	RWDD4	XLOC_071230	2768	coding	coding	coding	coding
MSTRG.69302.1	STOX2	XLOC_071237	256	coding	noncoding	noncoding	noncoding
MSTRG.69393.1		XLOC_071276	293	coding	noncoding	noncoding	noncoding
NM_001286710.1	ACSL1	XLOC_071286	3921	coding	coding	coding	coding
NM_001286712.1	ACSL1	XLOC_071286	4104	coding	coding	coding	coding
MSTRG.69422.1	LINC02436	XLOC_071308	255	coding	noncoding	noncoding	noncoding
MSTRG.69424.1		XLOC_071310	384	coding	noncoding	noncoding	noncoding
MSTRG.69487.1	SNX25	XLOC_071311	236	coding	noncoding	noncoding	noncoding
MSTRG.69439.1		XLOC_071353	294	coding	noncoding	noncoding	noncoding
MSTRG.69481.1	LOC101928929	XLOC_071369	206	coding	noncoding	noncoding	noncoding
MSTRG.69485.1	SORBS2	XLOC_071371	228	coding	noncoding	noncoding	noncoding
MSTRG.69446.1		XLOC_071372	312	coding	noncoding	noncoding	noncoding
MSTRG.69452.1		XLOC_071378	292	coding	noncoding	noncoding	noncoding
MSTRG.69555.1	F11-AS1	XLOC_071389	388	coding	noncoding	noncoding	noncoding
MSTRG.69557.1	F11-AS1	XLOC_071390	272	coding	noncoding	noncoding	noncoding
MSTRG.69542.1		XLOC_071393	256	coding	noncoding	noncoding	noncoding
MSTRG.69546.1		XLOC_071394	238	coding	noncoding	noncoding	noncoding
XM_005262834.3	FAT1	XLOC_071395	14967	coding	coding	noncoding	coding
MSTRG.69607.1	LOC102723906	XLOC_071410	230	coding	noncoding	noncoding	noncoding
MSTRG.69615.1	LOC107986335	XLOC_071416	247	coding	noncoding	noncoding	noncoding
MSTRG.69636.1		XLOC_071438	277	coding	noncoding	noncoding	noncoding
MSTRG.69648.1		XLOC_071444	240	coding	noncoding	noncoding	noncoding
MSTRG.69729.1		XLOC_071458	262	coding	noncoding	noncoding	noncoding
MSTRG.69730.1		XLOC_071459	330	coding	noncoding	noncoding	noncoding
MSTRG.69762.1	LOC107986338	XLOC_071483	240	coding	noncoding	noncoding	noncoding
MSTRG.69770.1	PLEKHG4B	XLOC_071486	206	coding	noncoding	noncoding	noncoding
NM_001166260.2	TRIP13	XLOC_071533	1452	coding	coding	coding	coding
NM_004237.4	TRIP13	XLOC_071533	2431	coding	coding	coding	coding
MSTRG.69811.1		XLOC_071534	437	coding	noncoding	noncoding	noncoding

MSTRG.69820.1	SLC12A7	XLOC_071538	376 coding	noncoding	noncoding	noncoding
MSTRG.69816.1		XLOC_071549	280 coding	noncoding	noncoding	noncoding
MSTRG.69834.1	TERT	XLOC_071550	867 coding	noncoding	coding	coding
MSTRG.69896.1		XLOC_071558	318 coding	noncoding	noncoding	noncoding
XR_925685.2	LOC105374616	XLOC_071570	2467 coding	noncoding	noncoding	noncoding
MSTRG.69933.1	LOC112267947	XLOC_071572	247 coding	noncoding	noncoding	noncoding
XR_002956203.1	LOC105374618	XLOC_071573	8837 coding	noncoding	noncoding	noncoding
MSTRG.69947.1		XLOC_071578	301 coding	noncoding	noncoding	noncoding
MSTRG.69983.1		XLOC_071603	251 coding	noncoding	noncoding	noncoding
MSTRG.70021.1		XLOC_071625	317 coding	noncoding	noncoding	noncoding
MSTRG.70026.1		XLOC_071628	286 coding	noncoding	noncoding	noncoding
MSTRG.70057.1		XLOC_071672	259 coding	noncoding	noncoding	noncoding
MSTRG.70070.1		XLOC_071679	293 coding	noncoding	noncoding	noncoding
MSTRG.70077.1		XLOC_071683	243 coding	noncoding	noncoding	noncoding
XR_001742591.1	LOC102724943	XLOC_071724	8189 coding	noncoding	noncoding	noncoding
MSTRG.70173.1	LOC102724943	XLOC_071725	211 coding	noncoding	noncoding	noncoding
MSTRG.70175.1		XLOC_071727	258 coding	noncoding	noncoding	noncoding
MSTRG.70194.1		XLOC_071736	311 coding	noncoding	noncoding	noncoding
MSTRG.70202.1		XLOC_071741	293 coding	noncoding	noncoding	noncoding
NM_001364441.2	MTRR	XLOC_071762	3748 coding	coding	coding	coding
MSTRG.70252.1		XLOC_071771	221 coding	noncoding	noncoding	noncoding
MSTRG.70253.1		XLOC_071772	330 coding	noncoding	noncoding	noncoding
MSTRG.70257.1		XLOC_071773	303 coding	noncoding	noncoding	noncoding
MSTRG.70302.1		XLOC_071797	226 coding	noncoding	noncoding	noncoding
MSTRG.70320.1	LINC02112	XLOC_071831	270 coding	noncoding	noncoding	noncoding
MSTRG.70329.1	LINC02112	XLOC_071837	323 coding	noncoding	noncoding	noncoding
MSTRG.70382.1		XLOC_071847	247 coding	noncoding	noncoding	noncoding
NM_001306153.1	CCT5	XLOC_071861	3275 coding	coding	coding	coding
MSTRG.70408.1	ANKRD33B	XLOC_071888	221 coding	noncoding	noncoding	noncoding
MSTRG.70456.1		XLOC_071898	610 coding	noncoding	noncoding	noncoding
MSTRG.70513.1	CTNND2	XLOC_071906	309 coding	noncoding	noncoding	noncoding
MSTRG.70525.1	CTNND2	XLOC_071912	301 coding	noncoding	noncoding	noncoding
MSTRG.70586.1		XLOC_071947	319 coding	noncoding	noncoding	noncoding
MSTRG.70610.1	DNAH5	XLOC_071954	238 coding	coding	noncoding	noncoding
MSTRG.70603.1		XLOC_071958	391 coding	noncoding	noncoding	noncoding
MSTRG.70758.1	TRIO	XLOC_071968	278 coding	noncoding	noncoding	noncoding
MSTRG.70619.1		XLOC_071981	311 coding	noncoding	noncoding	noncoding
MSTRG.70640.1		XLOC_072002	304 coding	noncoding	noncoding	noncoding
MSTRG.70643.1		XLOC_072004	301 coding	noncoding	noncoding	noncoding
MSTRG.70674.1		XLOC_072020	210 coding	noncoding	noncoding	noncoding
MSTRG.70727.1	FBXL7	XLOC_072032	236 coding	noncoding	noncoding	noncoding
MSTRG.70731.1	FBXL7	XLOC_072035	224 coding	noncoding	noncoding	noncoding
MSTRG.70743.1	11-Mar	XLOC_072043	305 coding	noncoding	noncoding	noncoding

MSTRG.70745.1		XLOC_072047	202	coding	noncoding	noncoding	noncoding
MSTRG.70779.1		XLOC_072066	300	coding	noncoding	noncoding	noncoding
MSTRG.70811.1	BASP1-AS1	XLOC_072068	283	coding	noncoding	noncoding	noncoding
MSTRG.70816.1	BASP1-AS1	XLOC_072071	231	coding	noncoding	noncoding	noncoding
MSTRG.70960.1		XLOC_072140	309	coding	noncoding	noncoding	noncoding
MSTRG.70968.1		XLOC_072145	234	coding	noncoding	noncoding	noncoding
MSTRG.71004.1		XLOC_072150	319	coding	noncoding	noncoding	noncoding
MSTRG.71025.1	CDH18	XLOC_072160	281	coding	noncoding	noncoding	noncoding
MSTRG.71033.1	CDH18	XLOC_072164	293	coding	noncoding	noncoding	noncoding
MSTRG.71048.1	CDH18	XLOC_072172	226	coding	noncoding	noncoding	noncoding
MSTRG.71056.1	CDH18	XLOC_072178	271	coding	noncoding	noncoding	noncoding
MSTRG.71075.1	LINC02241	XLOC_072183	304	coding	noncoding	noncoding	noncoding
MSTRG.71095.1		XLOC_072190	224	coding	noncoding	noncoding	noncoding
MSTRG.71128.1		XLOC_072214	310	coding	noncoding	noncoding	noncoding
MSTRG.71147.1		XLOC_072226	318	coding	noncoding	noncoding	noncoding
MSTRG.71151.1		XLOC_072229	244	coding	noncoding	noncoding	noncoding
MSTRG.71154.1		XLOC_072231	252	coding	noncoding	noncoding	noncoding
MSTRG.71199.1		XLOC_072235	287	coding	noncoding	noncoding	noncoding
MSTRG.71226.1	C5orf17	XLOC_072243	247	coding	noncoding	noncoding	noncoding
MSTRG.71233.1		XLOC_072247	297	coding	noncoding	noncoding	noncoding
MSTRG.71236.1		XLOC_072252	221	coding	noncoding	noncoding	noncoding
MSTRG.71254.1		XLOC_072260	266	coding	noncoding	noncoding	noncoding
MSTRG.71264.1	LINC02228	XLOC_072262	276	coding	noncoding	noncoding	noncoding
MSTRG.71260.1	LINC02211	XLOC_072269	287	coding	noncoding	noncoding	noncoding
MSTRG.71307.1		XLOC_072288	215	coding	noncoding	noncoding	noncoding
MSTRG.71317.1		XLOC_072293	341	coding	noncoding	noncoding	noncoding
MSTRG.71323.1		XLOC_072295	297	coding	coding	noncoding	noncoding
MSTRG.71324.1		XLOC_072296	260	coding	noncoding	noncoding	noncoding
MSTRG.71351.1		XLOC_072311	316	coding	coding	noncoding	noncoding
MSTRG.71385.1		XLOC_072331	278	coding	noncoding	noncoding	noncoding
MSTRG.71393.1		XLOC_072338	347	coding	noncoding	noncoding	noncoding
MSTRG.71402.1		XLOC_072343	228	coding	noncoding	noncoding	noncoding
MSTRG.71413.1		XLOC_072347	331	coding	noncoding	noncoding	noncoding
MSTRG.71432.1		XLOC_072360	288	coding	noncoding	noncoding	noncoding
MSTRG.71453.1		XLOC_072367	266	coding	noncoding	noncoding	noncoding
MSTRG.71467.1	CDH6	XLOC_072378	269	coding	noncoding	noncoding	noncoding
MSTRG.71536.1	C5orf22	XLOC_072383	244	coding	noncoding	noncoding	noncoding
MSTRG.71498.1	PDZD2	XLOC_072391	285	coding	noncoding	noncoding	noncoding
MSTRG.71501.1	PDZD2	XLOC_072394	302	coding	noncoding	noncoding	noncoding
MSTRG.71504.1	PDZD2	XLOC_072395	295	coding	noncoding	noncoding	noncoding
MSTRG.71505.1	PDZD2	XLOC_072396	261	coding	noncoding	noncoding	noncoding
MSTRG.71506.1	PDZD2	XLOC_072397	344	coding	coding	coding	noncoding
MSTRG.71511.1	PDZD2	XLOC_072398	279	coding	noncoding	noncoding	noncoding

MSTRG.71515.1	PDZD2	XLOC_072399	320 coding	noncoding	noncoding	noncoding
MSTRG.71479.1		XLOC_072410	438 coding	noncoding	noncoding	noncoding
MSTRG.71538.4	MTMR12	XLOC_072413	3648 coding	coding	coding	noncoding
MSTRG.71551.1		XLOC_072420	247 coding	noncoding	noncoding	noncoding
MSTRG.71557.1		XLOC_072423	260 coding	noncoding	noncoding	noncoding
MSTRG.71577.1	SUB1	XLOC_072427	208 coding	noncoding	noncoding	noncoding
MSTRG.71606.1		XLOC_072438	306 coding	noncoding	noncoding	noncoding
MSTRG.71613.1		XLOC_072441	328 coding	coding	noncoding	noncoding
NM_016568.3	RXFP3	XLOC_072465	2533 coding	coding	coding	coding
MSTRG.71768.1	SLC45A2	XLOC_072467	287 coding	noncoding	noncoding	noncoding
MSTRG.71679.1		XLOC_072527	432 coding	noncoding	noncoding	noncoding
MSTRG.71681.1		XLOC_072528	219 coding	noncoding	noncoding	noncoding
MSTRG.71717.1		XLOC_072551	214 coding	noncoding	noncoding	noncoding
MSTRG.71762.1		XLOC_072569	213 coding	noncoding	noncoding	noncoding
MSTRG.71864.1		XLOC_072631	286 coding	noncoding	noncoding	noncoding
MSTRG.71991.1		XLOC_072709	315 coding	noncoding	noncoding	noncoding
MSTRG.72108.4	OSMR	XLOC_072730	21637 coding	coding	coding	coding
MSTRG.72181.25	FYB1	XLOC_072736	13846 coding	noncoding	noncoding	coding
MSTRG.72105.3	DAB2	XLOC_072740	2837 coding	coding	noncoding	noncoding
MSTRG.72107.1		XLOC_072745	227 coding	noncoding	noncoding	noncoding
MSTRG.72144.1		XLOC_072760	259 coding	noncoding	noncoding	noncoding
MSTRG.72266.1	RPL37	XLOC_072793	444 coding	coding	noncoding	noncoding
MSTRG.72226.1	C7	XLOC_072799	209 coding	noncoding	noncoding	noncoding
MSTRG.72229.1	MROH2B	XLOC_072801	205 coding	noncoding	noncoding	noncoding
MSTRG.72287.1	C6	XLOC_072804	257 coding	noncoding	noncoding	noncoding
MSTRG.72289.1	C6	XLOC_072805	245 coding	noncoding	noncoding	noncoding
MSTRG.72317.1	FBXO4	XLOC_072826	266 coding	noncoding	noncoding	noncoding
MSTRG.72307.1		XLOC_072832	241 coding	noncoding	noncoding	noncoding
NM_001330714.2	ZNF131	XLOC_072897	3340 coding	coding	coding	coding
MSTRG.72426.1	ZNF131	XLOC_072900	293 coding	noncoding	noncoding	noncoding
MSTRG.72545.1	NNT-AS1	XLOC_072926	252 coding	noncoding	noncoding	noncoding
MSTRG.72559.1	NNT	XLOC_072940	247 coding	noncoding	noncoding	noncoding
MSTRG.72501.1		XLOC_072944	395 coding	coding	noncoding	noncoding
MSTRG.72524.1		XLOC_072952	233 coding	noncoding	noncoding	noncoding
MSTRG.72578.1		XLOC_072975	219 coding	coding	noncoding	noncoding
MSTRG.72582.1		XLOC_072977	223 coding	noncoding	noncoding	noncoding
MSTRG.72585.1		XLOC_072979	317 coding	noncoding	noncoding	noncoding
MSTRG.72613.1	HCN1	XLOC_072983	239 coding	coding	noncoding	noncoding
XM_011543631.3	PARP8	XLOC_073154	7090 coding	coding	coding	coding
XM_011543636.2	PARP8	XLOC_073154	7071 coding	coding	coding	coding
MSTRG.72834.1		XLOC_073168	279 coding	noncoding	noncoding	noncoding
MSTRG.72837.1		XLOC_073170	246 coding	noncoding	noncoding	noncoding
MSTRG.72845.1		XLOC_073174	212 coding	noncoding	noncoding	noncoding

MSTRG.72850.1		XLOC_073176	293 coding	noncoding	noncoding	noncoding
MSTRG.72873.1		XLOC_073186	272 coding	noncoding	noncoding	noncoding
MSTRG.72884.1		XLOC_073193	310 coding	coding	noncoding	noncoding
MSTRG.72890.1		XLOC_073196	271 coding	coding	noncoding	noncoding
MSTRG.72910.1	LOC105378964	XLOC_073204	260 coding	noncoding	noncoding	noncoding
MSTRG.72967.1	ITGA1	XLOC_073218	272 coding	noncoding	noncoding	noncoding
MSTRG.72923.1		XLOC_073227	299 coding	noncoding	noncoding	noncoding
MSTRG.72929.1		XLOC_073228	273 coding	noncoding	noncoding	noncoding
MSTRG.72947.1		XLOC_073231	300 coding	noncoding	noncoding	noncoding
MSTRG.72982.1	NDUFS4	XLOC_073238	237 coding	coding	noncoding	noncoding
MSTRG.72971.1		XLOC_073247	242 coding	noncoding	noncoding	noncoding
MSTRG.72974.1		XLOC_073248	453 coding	noncoding	noncoding	noncoding
XM_017008997.1	SNX18	XLOC_073270	2695 coding	coding	coding	coding
MSTRG.73093.1		XLOC_073298	263 coding	noncoding	noncoding	noncoding
MSTRG.73124.1		XLOC_073311	267 coding	noncoding	noncoding	noncoding
MSTRG.73140.1	DDX4	XLOC_073320	232 coding	noncoding	noncoding	noncoding
XM_017009485.1	MAP3K1	XLOC_073408	5295 coding	coding	coding	coding
MSTRG.73322.1		XLOC_073428	268 coding	noncoding	noncoding	noncoding
NM_001203246.1	GPBP1	XLOC_073429	4503 coding	coding	coding	coding
MSTRG.73449.1		XLOC_073454	283 coding	noncoding	noncoding	noncoding
NM_001317915.2	RAB3C	XLOC_073469	8966 coding	coding	coding	coding
MSTRG.73508.2	ELOVL7	XLOC_073683	2778 coding	coding	noncoding	noncoding
MSTRG.73585.1	NDUFAF2	XLOC_073702	209 coding	noncoding	noncoding	noncoding
MSTRG.73753.1	IPO11	XLOC_073830	214 coding	noncoding	noncoding	noncoding
MSTRG.73637.1		XLOC_073834	260 coding	coding	noncoding	noncoding
MSTRG.73638.1		XLOC_073835	272 coding	coding	noncoding	noncoding
MSTRG.73652.1	LOC107986418	XLOC_073839	201 coding	noncoding	noncoding	noncoding
MSTRG.73662.1		XLOC_073846	463 coding	noncoding	noncoding	noncoding
MSTRG.73666.1		XLOC_073848	291 coding	coding	noncoding	noncoding
MSTRG.73676.1		XLOC_073851	261 coding	noncoding	noncoding	noncoding
MSTRG.73765.1	RNF180	XLOC_073855	235 coding	noncoding	noncoding	noncoding
MSTRG.73864.1	CWC27	XLOC_073891	506 coding	noncoding	noncoding	noncoding
MSTRG.73780.1		XLOC_073893	306 coding	noncoding	noncoding	noncoding
MSTRG.73866.1		XLOC_073940	244 coding	noncoding	noncoding	noncoding
NM_139168.3	SREK1	XLOC_073951	6935 coding	coding	noncoding	coding
MSTRG.73920.1		XLOC_073965	357 coding	noncoding	noncoding	noncoding
MSTRG.73945.1	LOC105379003	XLOC_073968	217 coding	noncoding	noncoding	noncoding
MSTRG.74123.1	MAST4	XLOC_073982	359 coding	noncoding	noncoding	noncoding
MSTRG.73999.1		XLOC_074017	276 coding	noncoding	noncoding	noncoding
MSTRG.74018.1		XLOC_074025	305 coding	noncoding	noncoding	noncoding
MSTRG.74100.1	PIK3R1	XLOC_074053	218 coding	noncoding	noncoding	noncoding
NM_002538.4	OCLN	XLOC_074092	6183 coding	coding	coding	coding
MSTRG.74252.1		XLOC_074099	2027 coding	noncoding	coding	noncoding

NM_001178087.1	SERF1B	XLOC_074102	699 coding	coding	coding	noncoding
NR_073404.1	LOC441081	XLOC_074126	5287 coding	coding	coding	coding
NM_022968.1	SERF1A	XLOC_074140	707 coding	coding	coding	noncoding
MSTRG.74239.1	LOC107986382	XLOC_074180	245 coding	noncoding	noncoding	noncoding
MSTRG.74245.1		XLOC_074181	233 coding	noncoding	noncoding	noncoding
MSTRG.74336.1	LOC105379028	XLOC_074184	267 coding	noncoding	noncoding	noncoding
MSTRG.74361.1	PTCD2	XLOC_074193	280 coding	noncoding	noncoding	noncoding
MSTRG.74381.1	ZNF366	XLOC_074200	667 coding	coding	noncoding	coding
MSTRG.74374.1		XLOC_074216	279 coding	noncoding	noncoding	noncoding
MSTRG.74473.1	FCHO2	XLOC_074244	417 coding	noncoding	noncoding	noncoding
MSTRG.74434.1		XLOC_074250	456 coding	noncoding	noncoding	noncoding
MSTRG.74555.1	ARHGEF28	XLOC_074275	255 coding	noncoding	noncoding	noncoding
MSTRG.74507.1		XLOC_074302	292 coding	noncoding	noncoding	noncoding
MSTRG.74571.1	LOC105379039	XLOC_074310	309 coding	noncoding	noncoding	noncoding
XM_011543358.1	HMGCR	XLOC_074334	4620 coding	coding	coding	coding
MSTRG.74908.1		XLOC_074345	245 coding	coding	noncoding	noncoding
MSTRG.74910.1		XLOC_074346	270 coding	noncoding	noncoding	noncoding
XM_011543281.3	SV2C	XLOC_074347	11160 coding	coding	coding	coding
MSTRG.74934.1	SV2C	XLOC_074356	238 coding	coding	noncoding	noncoding
MSTRG.74913.1		XLOC_074362	454 coding	noncoding	noncoding	noncoding
MSTRG.74943.1		XLOC_074419	252 coding	noncoding	noncoding	noncoding
MSTRG.74944.1		XLOC_074420	274 coding	noncoding	noncoding	noncoding
MSTRG.74962.1	S100Z	XLOC_074427	293 coding	coding	noncoding	noncoding
MSTRG.75088.1		XLOC_074453	200 coding	noncoding	noncoding	noncoding
MSTRG.75129.1		XLOC_074458	368 coding	noncoding	noncoding	noncoding
MSTRG.75247.1	SCAMP1	XLOC_074478	222 coding	noncoding	noncoding	noncoding
MSTRG.75214.1		XLOC_074506	226 coding	noncoding	noncoding	noncoding
MSTRG.75215.1		XLOC_074507	322 coding	noncoding	noncoding	noncoding
MSTRG.75228.1	JMY	XLOC_074517	218 coding	noncoding	noncoding	noncoding
MSTRG.75193.1		XLOC_074534	386 coding	noncoding	noncoding	noncoding
XM_011543227.2	TENT2	XLOC_074535	3476 coding	coding	coding	coding
XM_017009151.2	TENT2	XLOC_074535	3725 coding	coding	coding	coding
MSTRG.75309.1	TENT2	XLOC_074540	287 coding	noncoding	noncoding	noncoding
MSTRG.75333.1	CMYA5	XLOC_074558	247 coding	noncoding	noncoding	noncoding
MSTRG.75297.1		XLOC_074593	436 coding	noncoding	noncoding	noncoding
NM_032567.4	SPZ1	XLOC_074594	1841 coding	noncoding	coding	coding
MSTRG.75422.1	ZFYVE16	XLOC_074604	248 coding	noncoding	noncoding	noncoding
XM_011543237.2	FAM151B	XLOC_074607	1640 coding	coding	noncoding	coding
MSTRG.75428.1	FAM151B	XLOC_074610	253 coding	noncoding	noncoding	noncoding
MSTRG.75828.1	RASGRF2	XLOC_074638	267 coding	noncoding	noncoding	noncoding
MSTRG.75552.1	ATG10	XLOC_074685	245 coding	coding	noncoding	noncoding
MSTRG.75569.1	ATG10	XLOC_074697	205 coding	noncoding	noncoding	noncoding
MSTRG.75521.1		XLOC_074715	361 coding	noncoding	noncoding	noncoding

MSTRG.75544.1		XLOC_074734	295 coding	coding	noncoding	noncoding
MSTRG.75581.1		XLOC_074744	265 coding	noncoding	noncoding	noncoding
MSTRG.75620.1	EDIL3	XLOC_074750	207 coding	noncoding	noncoding	noncoding
MSTRG.75627.1	EDIL3	XLOC_074756	280 coding	noncoding	noncoding	noncoding
XR_001742782.1	LOC105379057	XLOC_074758	8261 coding	noncoding	noncoding	noncoding
MSTRG.75609.1		XLOC_074765	254 coding	noncoding	noncoding	noncoding
MSTRG.75615.1		XLOC_074768	263 coding	noncoding	noncoding	noncoding
MSTRG.75641.1		XLOC_074771	268 coding	noncoding	noncoding	noncoding
MSTRG.75643.1		XLOC_074772	221 coding	coding	noncoding	noncoding
MSTRG.75657.1		XLOC_074777	275 coding	noncoding	noncoding	noncoding
MSTRG.75686.1		XLOC_074788	255 coding	noncoding	noncoding	noncoding
MSTRG.75696.1		XLOC_074792	287 coding	noncoding	noncoding	noncoding
NM_001867.3	COX7C	XLOC_074793	629 coding	coding	noncoding	noncoding
MSTRG.75700.1		XLOC_074796	281 coding	noncoding	noncoding	noncoding
MSTRG.75749.1	LOC101929380	XLOC_074810	289 coding	coding	noncoding	noncoding
MSTRG.75869.14	CCNH	XLOC_074816	1394 coding	noncoding	noncoding	noncoding
MSTRG.75869.15	RASA1	XLOC_074816	662 coding	coding	noncoding	noncoding
MSTRG.75869.21	RASA1	XLOC_074816	2134 coding	noncoding	noncoding	noncoding
MSTRG.75869.20	RASA1	XLOC_074816	4888 coding	coding	coding	noncoding
MSTRG.75763.1		XLOC_074826	306 coding	noncoding	noncoding	noncoding
MSTRG.75768.1		XLOC_074830	220 coding	noncoding	noncoding	noncoding
MSTRG.75993.1	LINC00461	XLOC_074854	218 coding	noncoding	noncoding	noncoding
MSTRG.76064.1	MEF2C-AS1	XLOC_074874	258 coding	noncoding	noncoding	noncoding
MSTRG.75921.1		XLOC_074894	267 coding	noncoding	noncoding	noncoding
MSTRG.75936.1	LINC02161	XLOC_074898	309 coding	noncoding	noncoding	noncoding
MSTRG.75961.1	LINC01339	XLOC_074911	248 coding	noncoding	noncoding	noncoding
MSTRG.76257.13	LUCAT1	XLOC_074925	6482 coding	noncoding	noncoding	noncoding
MSTRG.76257.16	LUCAT1	XLOC_074925	2395 coding	noncoding	noncoding	noncoding
MSTRG.76257.19	LUCAT1	XLOC_074925	4608 coding	noncoding	coding	noncoding
MSTRG.76123.1		XLOC_074940	418 coding	noncoding	noncoding	noncoding
MSTRG.76156.1	LOC105379080	XLOC_074958	290 coding	noncoding	noncoding	noncoding
MSTRG.76182.1		XLOC_074974	248 coding	noncoding	noncoding	noncoding
MSTRG.76190.1		XLOC_074980	258 coding	coding	noncoding	noncoding
MSTRG.77957.19	FAM172A	XLOC_074983	471 coding	noncoding	noncoding	noncoding
MSTRG.76295.1		XLOC_075010	261 coding	noncoding	noncoding	noncoding
MSTRG.76299.1	LOC105379089	XLOC_075013	341 coding	coding	noncoding	noncoding
MSTRG.76818.1	LOC101929710	XLOC_075076	311 coding	noncoding	noncoding	noncoding
MSTRG.76849.1	LOC101929710	XLOC_075082	314 coding	noncoding	noncoding	noncoding
MSTRG.76505.1		XLOC_075129	269 coding	noncoding	noncoding	noncoding
MSTRG.76391.1		XLOC_075138	223 coding	noncoding	noncoding	noncoding
MSTRG.76414.1		XLOC_075144	230 coding	coding	noncoding	noncoding
MSTRG.76413.1		XLOC_075145	212 coding	noncoding	noncoding	noncoding
MSTRG.76420.1		XLOC_075147	243 coding	noncoding	noncoding	noncoding

MSTRG.76566.1		XLOC_075192	257 coding	coding	noncoding	noncoding
MSTRG.76568.1		XLOC_075194	301 coding	noncoding	noncoding	noncoding
MSTRG.76698.15	ST8SIA4	XLOC_075218	11230 coding	noncoding	noncoding	noncoding
MSTRG.76675.1		XLOC_075242	314 coding	noncoding	noncoding	noncoding
MSTRG.76687.1		XLOC_075252	288 coding	noncoding	noncoding	noncoding
MSTRG.76705.1		XLOC_075295	223 coding	noncoding	noncoding	noncoding
MSTRG.77035.1	PAM	XLOC_075337	250 coding	noncoding	noncoding	noncoding
MSTRG.76926.1		XLOC_075379	245 coding	coding	noncoding	noncoding
MSTRG.76986.1	LOC105379107	XLOC_075393	298 coding	noncoding	noncoding	noncoding
MSTRG.77002.1		XLOC_075400	356 coding	noncoding	noncoding	noncoding
MSTRG.77072.1		XLOC_075404	291 coding	noncoding	noncoding	noncoding
MSTRG.77071.1		XLOC_075409	286 coding	noncoding	noncoding	noncoding
MSTRG.77088.1		XLOC_075421	262 coding	noncoding	noncoding	noncoding
MSTRG.77094.1		XLOC_075423	222 coding	noncoding	noncoding	noncoding
MSTRG.77114.1		XLOC_075435	245 coding	coding	noncoding	noncoding
MSTRG.77133.1		XLOC_075445	310 coding	noncoding	noncoding	noncoding
MSTRG.77158.1		XLOC_075497	331 coding	noncoding	noncoding	noncoding
MSTRG.77217.1		XLOC_075506	242 coding	noncoding	noncoding	noncoding
MSTRG.77222.1		XLOC_075507	276 coding	noncoding	noncoding	noncoding
MSTRG.77376.1	FER	XLOC_075510	222 coding	noncoding	noncoding	noncoding
XM_017009472.1	MAN2A1	XLOC_075579	3659 coding	coding	coding	coding
MSTRG.77288.1		XLOC_075611	221 coding	noncoding	noncoding	noncoding
MSTRG.77346.1		XLOC_075615	251 coding	coding	noncoding	noncoding
MSTRG.77372.1		XLOC_075623	229 coding	noncoding	noncoding	noncoding
NM_138773.4	SLC25A46	XLOC_075637	4745 coding	coding	coding	coding
MSTRG.77657.1	NREP	XLOC_075711	261 coding	noncoding	noncoding	noncoding
MSTRG.77522.1		XLOC_075717	222 coding	noncoding	noncoding	noncoding
MSTRG.77524.1		XLOC_075718	294 coding	noncoding	noncoding	noncoding
MSTRG.77683.1	APC	XLOC_075775	231 coding	noncoding	noncoding	noncoding
XR_001742031.1	DCP2	XLOC_075782	8845 coding	coding	coding	coding
XM_017009163.1	DCP2	XLOC_075782	9065 coding	coding	coding	coding
NM_001242377.2	DCP2	XLOC_075782	10005 coding	coding	coding	coding
MSTRG.77815.1	MCC	XLOC_075800	760 coding	noncoding	noncoding	noncoding
MSTRG.77851.1	MCC	XLOC_075827	275 coding	noncoding	noncoding	noncoding
MSTRG.77720.1	KCNN2	XLOC_075841	303 coding	coding	noncoding	noncoding
MSTRG.77747.1	LOC101927078	XLOC_075855	435 coding	noncoding	noncoding	noncoding
MSTRG.77703.1		XLOC_075857	286 coding	noncoding	noncoding	noncoding
MSTRG.77707.1		XLOC_075859	235 coding	noncoding	noncoding	noncoding
MSTRG.77710.1		XLOC_075860	219 coding	noncoding	noncoding	noncoding
MSTRG.77712.1		XLOC_075862	426 coding	noncoding	noncoding	noncoding
MSTRG.77865.1		XLOC_075882	290 coding	noncoding	noncoding	noncoding
MSTRG.77861.1		XLOC_075884	278 coding	noncoding	noncoding	noncoding
MSTRG.77862.1		XLOC_075885	228 coding	noncoding	noncoding	noncoding

MSTRG.77907.1		XLOC_075896	401	coding	noncoding	noncoding	noncoding
MSTRG.77939.1	LVRN	XLOC_075907	318	coding	noncoding	noncoding	noncoding
MSTRG.78519.1		XLOC_075958	252	coding	noncoding	noncoding	noncoding
MSTRG.78100.1		XLOC_075975	225	coding	noncoding	noncoding	noncoding
MSTRG.78120.1		XLOC_075990	225	coding	noncoding	noncoding	noncoding
MSTRG.78136.1	LINC00992	XLOC_076000	307	coding	coding	noncoding	noncoding
MSTRG.78141.1		XLOC_076020	302	coding	noncoding	noncoding	noncoding
MSTRG.78245.1	LOC105379143	XLOC_076061	215	coding	noncoding	noncoding	noncoding
MSTRG.78250.1	DMXL1	XLOC_076065	261	coding	noncoding	noncoding	noncoding
MSTRG.78253.1	DMXL1	XLOC_076068	221	coding	noncoding	noncoding	noncoding
MSTRG.78258.1	DMXL1	XLOC_076073	307	coding	noncoding	noncoding	noncoding
MSTRG.78235.1		XLOC_076138	286	coding	noncoding	noncoding	noncoding
MSTRG.78337.1		XLOC_076157	241	coding	noncoding	noncoding	noncoding
MSTRG.78418.1		XLOC_076191	208	coding	noncoding	noncoding	noncoding
MSTRG.78432.1		XLOC_076195	322	coding	noncoding	noncoding	noncoding
MSTRG.78439.1		XLOC_076199	226	coding	noncoding	noncoding	noncoding
XM_011543741.2	SNCAIP	XLOC_076226	3659	coding	coding	coding	coding
MSTRG.78649.1	SNCAIP	XLOC_076227	293	coding	noncoding	noncoding	noncoding
MSTRG.78469.1		XLOC_076228	254	coding	noncoding	noncoding	noncoding
MSTRG.78643.1		XLOC_076234	259	coding	noncoding	noncoding	noncoding
MSTRG.78684.1	LOC105379154	XLOC_076240	234	coding	noncoding	noncoding	noncoding
MSTRG.78699.1	SNX24	XLOC_076250	278	coding	noncoding	noncoding	noncoding
MSTRG.78712.1		XLOC_076258	284	coding	coding	noncoding	noncoding
NM_001364150.2	CSNK1G3	XLOC_076267	4525	coding	coding	coding	coding
XM_017009062.1	CSNK1G3	XLOC_076267	4096	coding	coding	coding	coding
MSTRG.78817.1	CSNK1G3	XLOC_076270	291	coding	noncoding	noncoding	noncoding
MSTRG.78743.1		XLOC_076286	244	coding	noncoding	noncoding	noncoding
MSTRG.78760.1		XLOC_076292	282	coding	noncoding	noncoding	noncoding
MSTRG.78790.1	LINC01170	XLOC_076299	264	coding	noncoding	noncoding	noncoding
MSTRG.78837.1		XLOC_076343	286	coding	noncoding	noncoding	noncoding
MSTRG.78892.1		XLOC_076352	304	coding	coding	noncoding	noncoding
MSTRG.78896.1		XLOC_076355	312	coding	noncoding	noncoding	noncoding
MSTRG.78898.1		XLOC_076357	264	coding	noncoding	noncoding	noncoding
MSTRG.78900.1		XLOC_076358	318	coding	noncoding	noncoding	noncoding
MSTRG.78943.1	ALDH7A1	XLOC_076392	274	coding	noncoding	noncoding	noncoding
MSTRG.78949.1	PHAX	XLOC_076396	242	coding	noncoding	noncoding	noncoding
MSTRG.78937.1		XLOC_076398	357	coding	noncoding	noncoding	noncoding
MSTRG.78939.1		XLOC_076399	228	coding	noncoding	noncoding	noncoding
MSTRG.79049.1	LOC105379163	XLOC_076409	234	coding	noncoding	noncoding	noncoding
MSTRG.79067.1		XLOC_076493	239	coding	noncoding	noncoding	noncoding
MSTRG.79264.1		XLOC_076536	353	coding	noncoding	noncoding	noncoding
MSTRG.79269.1		XLOC_076538	292	coding	noncoding	noncoding	noncoding
MSTRG.79272.1		XLOC_076540	277	coding	noncoding	noncoding	noncoding

MSTRG.79277.1		XLOC_076542	317 coding	noncoding	noncoding	noncoding
MSTRG.79287.1		XLOC_076547	237 coding	noncoding	noncoding	noncoding
MSTRG.79291.1		XLOC_076549	288 coding	noncoding	noncoding	noncoding
MSTRG.79292.1		XLOC_076550	269 coding	noncoding	noncoding	noncoding
MSTRG.79294.2	HINT1	XLOC_076552	586 coding	coding	noncoding	noncoding
NM_001038702.1	CDC42SE2	XLOC_076557	3488 coding	noncoding	noncoding	noncoding
XM_017009649.2	CDC42SE2	XLOC_076557	3515 coding	noncoding	noncoding	noncoding
XM_017009648.2	CDC42SE2	XLOC_076557	3665 coding	noncoding	noncoding	noncoding
MSTRG.79530.2	SLC22A4	XLOC_076602	2164 coding	coding	coding	coding
NM_001354992.2	IL13	XLOC_076614	1454 coding	coding	coding	noncoding
MSTRG.79487.1		XLOC_076642	257 coding	noncoding	noncoding	noncoding
MSTRG.79490.1		XLOC_076643	282 coding	noncoding	noncoding	noncoding
MSTRG.79522.1	HSPA4	XLOC_076648	299 coding	noncoding	noncoding	noncoding
MSTRG.79515.1		XLOC_076651	282 coding	noncoding	noncoding	noncoding
MSTRG.79593.1	FSTL4	XLOC_076666	615 coding	noncoding	noncoding	noncoding
MSTRG.79648.2	PPP2CA	XLOC_076699	2278 coding	coding	noncoding	noncoding
MSTRG.79778.6	C5orf66	XLOC_076745	1904 coding	coding	coding	coding
XR_001742007.1	SLC25A48	XLOC_076756	7323 coding	coding	coding	coding
MSTRG.79756.1		XLOC_076760	278 coding	noncoding	noncoding	noncoding
MSTRG.79834.1	TRPC7	XLOC_076785	280 coding	noncoding	noncoding	noncoding
MSTRG.79825.1		XLOC_076789	321 coding	noncoding	noncoding	noncoding
MSTRG.79842.1		XLOC_076791	289 coding	noncoding	noncoding	noncoding
MSTRG.80009.1	WNT8A	XLOC_076911	216 coding	noncoding	noncoding	noncoding
NR_146628.1	LOC100128966	XLOC_076915	551 coding	noncoding	noncoding	coding
XM_011543488.2	KDM3B	XLOC_076919	6528 coding	coding	coding	coding
MSTRG.80056.1	KDM3B	XLOC_076920	245 coding	noncoding	noncoding	noncoding
MSTRG.80061.1	KDM3B	XLOC_076925	355 coding	noncoding	noncoding	noncoding
MSTRG.80130.1	CTNNA1	XLOC_076942	286 coding	noncoding	noncoding	noncoding
MSTRG.80082.1		XLOC_076961	271 coding	noncoding	noncoding	noncoding
NM_001194955.1	MATR3	XLOC_076962	5282 coding	noncoding	coding	coding
XM_017009522.1	PAIP2	XLOC_076969	2886 coding	coding	coding	coding
MSTRG.80107.1		XLOC_076979	216 coding	noncoding	noncoding	noncoding
MSTRG.80111.1		XLOC_076982	279 coding	noncoding	noncoding	noncoding
MSTRG.80178.1	UBE2D2	XLOC_076986	491 coding	noncoding	noncoding	noncoding
MSTRG.80171.1	PSD2	XLOC_076995	316 coding	noncoding	noncoding	noncoding
MSTRG.80196.1	NRG2	XLOC_076996	297 coding	coding	noncoding	noncoding
MSTRG.80205.1	NRG2	XLOC_077001	289 coding	noncoding	noncoding	noncoding
MSTRG.80297.2	APBB3	XLOC_077042	2401 coding	coding	coding	coding
XM_011537668.2	TMCO6	XLOC_077048	1644 coding	coding	coding	coding
XM_005268469.3	WDR55	XLOC_077056	4121 coding	coding	coding	coding
NM_031861.2	PCDHA11	XLOC_077062	4088 coding	coding	coding	coding
NM_031864.3	PCDHA12	XLOC_077062	2582 coding	coding	coding	coding
MSTRG.80259.1	PCDHA11	XLOC_077064	447 coding	noncoding	noncoding	noncoding

NM_018937.4	PCDHB3	XLOC_077069	3586 coding	coding	coding	coding
NM_019120.5	PCDHB8	XLOC_077075	2750 coding	coding	coding	coding
NM_019119.5	PCDHB9	XLOC_077077	4381 coding	coding	coding	coding
NM_032053.3	PCDHGA4	XLOC_077098	2713 coding	coding	coding	coding
MSTRG.80424.6	DIAPH1	XLOC_077100	3161 coding	coding	coding	coding
MSTRG.80334.1		XLOC_077114	438 coding	noncoding	noncoding	noncoding
MSTRG.80364.1		XLOC_077137	315 coding	noncoding	noncoding	noncoding
MSTRG.80371.1	SPRY4-AS1	XLOC_077142	225 coding	noncoding	noncoding	noncoding
MSTRG.81085.1	ARHGAP26	XLOC_077241	282 coding	noncoding	noncoding	noncoding
MSTRG.81160.1		XLOC_077316	266 coding	noncoding	noncoding	noncoding
MSTRG.80512.1		XLOC_077365	236 coding	noncoding	noncoding	noncoding
MSTRG.80555.1		XLOC_077376	230 coding	noncoding	noncoding	noncoding
MSTRG.80643.1	PRELID2	XLOC_077401	229 coding	noncoding	noncoding	noncoding
XM_017008982.2	TCERG1	XLOC_077434	4250 coding	coding	coding	coding
MSTRG.80682.1		XLOC_077444	310 coding	noncoding	noncoding	noncoding
MSTRG.80715.1	STK32A	XLOC_077460	206 coding	noncoding	noncoding	noncoding
MSTRG.80736.1		XLOC_077491	245 coding	noncoding	noncoding	noncoding
XM_017009899.1	FBXO38	XLOC_077523	4561 coding	coding	coding	coding
NM_205836.3	FBXO38	XLOC_077523	4401 coding	coding	coding	coding
MSTRG.80878.12	FBXO38	XLOC_077523	613 coding	noncoding	coding	coding
MSTRG.80932.1		XLOC_077552	281 coding	noncoding	noncoding	noncoding
MSTRG.80846.1		XLOC_077568	205 coding	coding	noncoding	noncoding
MSTRG.80910.1	SH3TC2-DT	XLOC_077575	287 coding	noncoding	noncoding	noncoding
MSTRG.80927.1	AFAP1L1	XLOC_077585	265 coding	noncoding	noncoding	noncoding
MSTRG.80970.5	CSNK1A1	XLOC_077595	1815 coding	coding	noncoding	noncoding
NM_001172699.1	PPARGC1B	XLOC_077602	10541 coding	coding	coding	coding
MSTRG.81199.1	PDE6A	XLOC_077610	220 coding	noncoding	noncoding	noncoding
MSTRG.80988.1		XLOC_077612	253 coding	noncoding	noncoding	noncoding
MSTRG.81221.1	CAMK2A	XLOC_077628	303 coding	noncoding	noncoding	noncoding
MSTRG.81217.1		XLOC_077631	234 coding	noncoding	noncoding	noncoding
MSTRG.81252.1	TCOF1	XLOC_077634	254 coding	noncoding	noncoding	noncoding
MSTRG.81264.1	NDST1-AS1	XLOC_077638	305 coding	noncoding	noncoding	noncoding
MSTRG.81256.1		XLOC_077644	449 coding	noncoding	noncoding	noncoding
MSTRG.81323.8	TNIP1	XLOC_077672	2283 coding	noncoding	coding	coding
MSTRG.81340.1		XLOC_077684	318 coding	noncoding	noncoding	noncoding
MSTRG.81384.1	LOC105378234	XLOC_077685	270 coding	noncoding	noncoding	noncoding
MSTRG.81410.1	FAT2	XLOC_077690	225 coding	noncoding	noncoding	noncoding
NM_198395.2	G3BP1	XLOC_077703	10196 coding	coding	coding	coding
MSTRG.81422.1		XLOC_077708	249 coding	noncoding	noncoding	noncoding
MSTRG.81428.1	GLRA1	XLOC_077710	475 coding	noncoding	noncoding	noncoding
MSTRG.81451.1	LINC01933	XLOC_077718	221 coding	noncoding	noncoding	noncoding
MSTRG.81472.1	LINC01933	XLOC_077725	246 coding	noncoding	noncoding	noncoding
MSTRG.81436.1		XLOC_077729	439 coding	noncoding	noncoding	noncoding

MSTRG.81479.1	LINC01470	XLOC_077731	446 coding	coding	noncoding	noncoding
MSTRG.81482.1	LINC01470	XLOC_077733	432 coding	noncoding	noncoding	noncoding
MSTRG.81483.1	LINC01470	XLOC_077734	252 coding	noncoding	noncoding	noncoding
MSTRG.81486.1	LINC01470	XLOC_077736	289 coding	noncoding	noncoding	noncoding
MSTRG.81477.1		XLOC_077739	305 coding	noncoding	noncoding	noncoding
MSTRG.81541.1		XLOC_077773	323 coding	noncoding	noncoding	noncoding
MSTRG.81553.1		XLOC_077789	399 coding	coding	noncoding	noncoding
XM_011537616.1	LARP1	XLOC_077792	6491 coding	coding	coding	coding
MSTRG.81583.1	LARP1	XLOC_077794	265 coding	noncoding	noncoding	noncoding
MSTRG.81588.1	LARP1	XLOC_077796	274 coding	noncoding	noncoding	noncoding
MSTRG.81599.6	FAXDC2	XLOC_077802	1472 coding	coding	coding	noncoding
MSTRG.81628.1		XLOC_077825	222 coding	noncoding	noncoding	noncoding
MSTRG.81636.1		XLOC_077829	397 coding	noncoding	noncoding	noncoding
XM_017009443.1	ITK	XLOC_077887	4065 coding	coding	coding	coding
MSTRG.81728.1		XLOC_077931	231 coding	noncoding	noncoding	noncoding
MSTRG.81733.1		XLOC_077939	252 coding	noncoding	noncoding	noncoding
MSTRG.81741.1	LOC107986466	XLOC_077942	262 coding	noncoding	noncoding	noncoding
MSTRG.81771.1		XLOC_077955	283 coding	noncoding	noncoding	noncoding
MSTRG.81830.1		XLOC_077975	213 coding	noncoding	noncoding	noncoding
XM_011534437.2	ADRA1B	XLOC_077997	3798 coding	coding	coding	coding
MSTRG.81929.1	PWWP2A	XLOC_078004	254 coding	noncoding	noncoding	noncoding
MSTRG.82000.4	SLU7	XLOC_078037	2521 coding	coding	noncoding	noncoding
MSTRG.81992.1		XLOC_078040	282 coding	noncoding	noncoding	noncoding
MSTRG.82066.1	ATP10B	XLOC_078051	259 coding	noncoding	noncoding	noncoding
MSTRG.82027.1	GABRB2	XLOC_078061	208 coding	noncoding	noncoding	noncoding
MSTRG.82035.1	GABRB2	XLOC_078065	247 coding	noncoding	noncoding	noncoding
MSTRG.82084.1		XLOC_078075	257 coding	noncoding	noncoding	noncoding
MSTRG.82093.1		XLOC_078081	210 coding	noncoding	noncoding	noncoding
MSTRG.82114.1		XLOC_078092	240 coding	noncoding	noncoding	noncoding
MSTRG.82213.1	LOC105377703	XLOC_078138	345 coding	noncoding	noncoding	noncoding
MSTRG.82197.1		XLOC_078144	206 coding	noncoding	noncoding	noncoding
MSTRG.82233.1		XLOC_078152	266 coding	noncoding	noncoding	noncoding
MSTRG.82235.1		XLOC_078154	293 coding	noncoding	noncoding	noncoding
MSTRG.82242.1		XLOC_078159	292 coding	noncoding	noncoding	noncoding
MSTRG.82364.1		XLOC_078170	245 coding	noncoding	noncoding	noncoding
MSTRG.82370.1		XLOC_078173	240 coding	noncoding	noncoding	noncoding
XM_017009660.1	TENM2	XLOC_078178	10321 coding	coding	noncoding	coding
MSTRG.82412.1	TENM2	XLOC_078182	272 coding	noncoding	noncoding	noncoding
MSTRG.82416.1	TENM2	XLOC_078184	256 coding	noncoding	noncoding	noncoding
MSTRG.82419.1	TENM2	XLOC_078186	325 coding	noncoding	noncoding	noncoding
MSTRG.82443.1	TENM2	XLOC_078196	268 coding	noncoding	noncoding	noncoding
MSTRG.82451.1	TENM2	XLOC_078202	258 coding	noncoding	noncoding	noncoding
MSTRG.82453.1	TENM2	XLOC_078203	235 coding	noncoding	noncoding	noncoding

MSTRG.82383.1		XLOC_078206	279	coding	noncoding	noncoding	noncoding
MSTRG.82392.1	WWC1	XLOC_078211	221	coding	noncoding	noncoding	noncoding
MSTRG.82393.1	WWC1	XLOC_078212	271	coding	noncoding	noncoding	noncoding
MSTRG.82493.1	SLIT3	XLOC_078235	297	coding	noncoding	noncoding	noncoding
MSTRG.82465.1		XLOC_078242	361	coding	coding	noncoding	noncoding
MSTRG.82466.1		XLOC_078243	269	coding	noncoding	noncoding	noncoding
MSTRG.82467.1		XLOC_078244	297	coding	noncoding	noncoding	noncoding
MSTRG.82470.1		XLOC_078251	816	coding	coding	noncoding	coding
MSTRG.82518.1		XLOC_078357	280	coding	noncoding	noncoding	noncoding
MSTRG.82584.1	RANBP17	XLOC_078396	228	coding	noncoding	noncoding	noncoding
MSTRG.82570.1		XLOC_078401	252	coding	noncoding	noncoding	noncoding
NM_001355006.1	NPM1	XLOC_078402	1339	coding	coding	coding	coding
MSTRG.82719.1	LOC105377729	XLOC_078469	464	coding	noncoding	noncoding	noncoding
MSTRG.82751.1	LOC101928093	XLOC_078477	270	coding	noncoding	noncoding	noncoding
MSTRG.82888.1	ERGIC1	XLOC_078489	288	coding	noncoding	noncoding	noncoding
MSTRG.82874.1		XLOC_078499	322	coding	noncoding	noncoding	noncoding
MSTRG.82914.1	NKX2-5	XLOC_078507	210	coding	noncoding	noncoding	noncoding
MSTRG.82932.1		XLOC_078526	239	coding	noncoding	noncoding	noncoding
MSTRG.82937.1		XLOC_078529	287	coding	noncoding	noncoding	noncoding
NM_015980.5	NSG2	XLOC_078557	2350	coding	coding	coding	coding
MSTRG.83026.1	LINC01411	XLOC_078569	362	coding	noncoding	noncoding	noncoding
MSTRG.83028.1	LOC105377739	XLOC_078570	266	coding	noncoding	noncoding	noncoding
MSTRG.83033.1		XLOC_078573	238	coding	noncoding	noncoding	noncoding
MSTRG.83034.1		XLOC_078574	262	coding	noncoding	noncoding	noncoding
MSTRG.83037.1		XLOC_078575	261	coding	noncoding	noncoding	noncoding
MSTRG.83039.1		XLOC_078576	220	coding	noncoding	noncoding	noncoding
MSTRG.83064.1		XLOC_078591	580	coding	noncoding	noncoding	noncoding
NM_006650.4	CPLX2	XLOC_078612	4801	coding	coding	coding	coding
MSTRG.83097.1	LOC100996385	XLOC_078653	261	coding	noncoding	coding	noncoding
NR_038402.1	LOC100507387	XLOC_078658	967	coding	noncoding	coding	noncoding
NM_001317948.1	ARL10	XLOC_078661	1631	coding	coding	coding	coding
NM_173664.6	ARL10	XLOC_078661	10826	coding	coding	coding	coding
MSTRG.83164.1		XLOC_078666	259	coding	noncoding	noncoding	noncoding
MSTRG.83165.1		XLOC_078667	237	coding	noncoding	noncoding	noncoding
MSTRG.83211.2	RNF44	XLOC_078671	5669	coding	coding	coding	coding
XM_017009298.1	ZNF346	XLOC_078689	1832	coding	coding	coding	coding
MSTRG.83270.1	ZNF346	XLOC_078695	236	coding	noncoding	noncoding	noncoding
MSTRG.83271.1	ZNF346	XLOC_078696	277	coding	noncoding	noncoding	noncoding
MSTRG.83272.1	ZNF346	XLOC_078697	238	coding	noncoding	noncoding	noncoding
MSTRG.83229.1		XLOC_078707	266	coding	noncoding	noncoding	noncoding
XR_941112.2	SLC34A1	XLOC_078712	3969	coding	coding	coding	coding
MSTRG.83274.8	SLC34A1	XLOC_078712	237	coding	noncoding	noncoding	noncoding
MSTRG.83336.7	FAM193B	XLOC_078731	612	coding	noncoding	noncoding	noncoding

MSTRG.83336.8	FAM193B	XLOC_078731	598 coding	noncoding	coding	noncoding
NR_159407.1	FAM153CP	XLOC_078758	1210 coding	coding	coding	coding
NM_022762.5	RMND5B	XLOC_078763	3911 coding	coding	coding	coding
MSTRG.83417.1	COL23A1	XLOC_078779	203 coding	noncoding	noncoding	noncoding
MSTRG.83440.1	AACSP1	XLOC_078793	324 coding	noncoding	noncoding	noncoding
MSTRG.83480.1		XLOC_078808	266 coding	noncoding	noncoding	noncoding
MSTRG.83487.1		XLOC_078811	338 coding	noncoding	coding	noncoding
XR_245277.3	RUFY1	XLOC_078813	2219 coding	coding	coding	coding
XR_001742278.1	RUFY1	XLOC_078813	2784 coding	coding	coding	coding
MSTRG.83507.1		XLOC_078829	217 coding	noncoding	noncoding	noncoding
MSTRG.83518.1		XLOC_078842	241 coding	noncoding	noncoding	noncoding
MSTRG.83572.1		XLOC_078855	277 coding	noncoding	noncoding	noncoding
MSTRG.83587.1		XLOC_078865	248 coding	noncoding	noncoding	noncoding
MSTRG.83588.1		XLOC_078869	325 coding	noncoding	noncoding	noncoding
MSTRG.83599.1		XLOC_078873	282 coding	noncoding	noncoding	noncoding
MSTRG.83631.2	ZFP62	XLOC_078885	1151 coding	noncoding	coding	noncoding
MSTRG.83631.4	ZFP62	XLOC_078885	2041 coding	coding	noncoding	coding
MSTRG.83655.1		XLOC_078899	460 coding	noncoding	noncoding	noncoding
MSTRG.83656.1		XLOC_078900	213 coding	noncoding	noncoding	noncoding
MSTRG.83633.1		XLOC_078904	253 coding	noncoding	noncoding	noncoding
MSTRG.83663.2	RACK1	XLOC_078909	1148 coding	coding	noncoding	coding
NM_001005224.1	OR4F3	XLOC_078926	939 coding	coding	coding	coding
MSTRG.69766.1		XLOC_078928	308 coding	noncoding	noncoding	noncoding
NM_145265.3	CCDC127	XLOC_078930	9283 coding	noncoding	coding	coding
MSTRG.69805.5	PDCD6	XLOC_078939	1198 coding	coding	noncoding	noncoding
MSTRG.69777.3	EXOC3	XLOC_078942	2610 coding	coding	coding	noncoding
XM_017010120.1	ZDHHC11B	XLOC_078963	6103 coding	coding	coding	coding
XM_017010114.2	ZDHHC11B	XLOC_078963	5293 coding	coding	coding	coding
XM_017009874.2	ZDHHC11	XLOC_078965	3556 coding	noncoding	coding	coding
XR_002956181.1	ZDHHC11	XLOC_078965	3444 coding	coding	coding	coding
MSTRG.69879.1	TRIP13	XLOC_078970	297 coding	noncoding	noncoding	noncoding
NR_104614.1	LOC100506688	XLOC_078972	3517 coding	noncoding	coding	coding
MSTRG.69880.1		XLOC_078995	225 coding	noncoding	noncoding	noncoding
MSTRG.69913.1	LOC728613	XLOC_079000	252 coding	noncoding	noncoding	noncoding
MSTRG.69914.1	LOC728613	XLOC_079001	218 coding	coding	noncoding	noncoding
NM_001278635.1	IRX4	XLOC_079017	2403 coding	coding	coding	coding
MSTRG.69932.1	LOC112267947	XLOC_079021	245 coding	noncoding	noncoding	noncoding
MSTRG.69945.1	LOC100506858	XLOC_079028	577 coding	noncoding	noncoding	noncoding
MSTRG.69967.1		XLOC_079040	244 coding	noncoding	noncoding	noncoding
MSTRG.69976.1		XLOC_079043	412 coding	noncoding	noncoding	noncoding
MSTRG.70032.1		XLOC_079078	255 coding	noncoding	noncoding	noncoding
MSTRG.70099.1	ADAMTS16	XLOC_079082	230 coding	noncoding	noncoding	noncoding
MSTRG.70107.1	ADAMTS16	XLOC_079085	263 coding	noncoding	noncoding	noncoding

MSTRG.70054.1		XLOC_079093	235	coding	noncoding	noncoding	noncoding
MSTRG.70058.1		XLOC_079094	259	coding	noncoding	noncoding	noncoding
MSTRG.70069.1		XLOC_079100	293	coding	noncoding	noncoding	noncoding
MSTRG.70084.1		XLOC_079106	220	coding	noncoding	noncoding	noncoding
MSTRG.70155.1	LINC02102	XLOC_079125	238	coding	noncoding	noncoding	noncoding
MSTRG.70201.1		XLOC_079139	241	coding	noncoding	noncoding	noncoding
MSTRG.70256.1		XLOC_079174	303	coding	noncoding	noncoding	noncoding
MSTRG.70276.1	LINC02226	XLOC_079180	266	coding	coding	noncoding	noncoding
MSTRG.70288.1	LOC101929307	XLOC_079187	331	coding	noncoding	noncoding	noncoding
MSTRG.70321.1	LINC02112	XLOC_079210	255	coding	noncoding	noncoding	noncoding
MSTRG.70403.3	CCT5	XLOC_079223	1711	coding	coding	coding	noncoding
MSTRG.70374.1	LOC101929977	XLOC_079227	239	coding	noncoding	noncoding	noncoding
MSTRG.70452.1		XLOC_079247	205	coding	noncoding	noncoding	noncoding
MSTRG.70479.1	DAP	XLOC_079263	386	coding	noncoding	noncoding	noncoding
MSTRG.70506.1	CTNND2	XLOC_079283	247	coding	noncoding	noncoding	noncoding
MSTRG.70509.1	CTNND2	XLOC_079285	323	coding	noncoding	noncoding	noncoding
MSTRG.70512.1	CTNND2	XLOC_079286	309	coding	noncoding	noncoding	noncoding
MSTRG.70527.1	CTNND2	XLOC_079293	278	coding	noncoding	coding	noncoding
MSTRG.70559.1	LINC01194	XLOC_079320	456	coding	noncoding	noncoding	noncoding
MSTRG.70569.1	LINC02220	XLOC_079323	685	coding	noncoding	noncoding	noncoding
MSTRG.70575.1	LINC02220	XLOC_079327	240	coding	noncoding	noncoding	noncoding
MSTRG.70566.1		XLOC_079329	298	coding	noncoding	coding	noncoding
XM_024454388.1	DNAH5	XLOC_079338	18329	coding	coding	noncoding	coding
MSTRG.70615.1	DNAH5	XLOC_079343	224	coding	noncoding	noncoding	noncoding
MSTRG.70604.1		XLOC_079346	391	coding	noncoding	noncoding	noncoding
NM_054027.6	ANKH	XLOC_079364	8207	coding	coding	coding	coding
MSTRG.70639.1		XLOC_079381	290	coding	noncoding	noncoding	noncoding
MSTRG.70722.1	FBXL7	XLOC_079403	219	coding	noncoding	noncoding	noncoding
MSTRG.70742.1	11-Mar	XLOC_079415	305	coding	noncoding	noncoding	noncoding
XR_001742613.1	LOC105379640	XLOC_079416	2397	coding	noncoding	noncoding	noncoding
MSTRG.70746.1		XLOC_079418	300	coding	noncoding	noncoding	noncoding
MSTRG.70777.1		XLOC_079456	264	coding	noncoding	noncoding	noncoding
MSTRG.70778.1		XLOC_079457	248	coding	noncoding	noncoding	noncoding
MSTRG.70809.1		XLOC_079460	271	coding	noncoding	noncoding	noncoding
MSTRG.70800.1		XLOC_079475	249	coding	noncoding	noncoding	noncoding
MSTRG.70806.1	LINC02111	XLOC_079479	226	coding	noncoding	noncoding	noncoding
MSTRG.70890.1	LOC105374666	XLOC_079506	268	coding	noncoding	noncoding	noncoding
MSTRG.70898.1	LINC02223	XLOC_079515	641	coding	noncoding	noncoding	noncoding
MSTRG.70899.1	LINC02223	XLOC_079516	726	coding	noncoding	noncoding	noncoding
MSTRG.70907.1		XLOC_079520	290	coding	noncoding	noncoding	noncoding
MSTRG.70947.1		XLOC_079527	231	coding	noncoding	noncoding	noncoding
MSTRG.71034.1	CDH18	XLOC_079562	383	coding	noncoding	noncoding	noncoding
MSTRG.71040.1	CDH18	XLOC_079565	400	coding	noncoding	noncoding	noncoding

MSTRG.71052.1	CDH18	XLOC_079569	299	coding	coding	noncoding	noncoding
MSTRG.71089.1		XLOC_079599	243	coding	noncoding	noncoding	noncoding
MSTRG.71177.1	CDH12	XLOC_079645	277	coding	noncoding	noncoding	noncoding
MSTRG.71203.1	LOC107986377	XLOC_079668	269	coding	noncoding	noncoding	noncoding
MSTRG.71216.1	C5orf17	XLOC_079674	253	coding	noncoding	noncoding	noncoding
MSTRG.71232.1		XLOC_079688	297	coding	noncoding	coding	noncoding
MSTRG.71241.1	CDH10	XLOC_079692	252	coding	noncoding	noncoding	noncoding
MSTRG.71253.1		XLOC_079697	266	coding	noncoding	noncoding	noncoding
MSTRG.71263.1	LINC02228	XLOC_079700	276	coding	noncoding	noncoding	noncoding
MSTRG.71291.1		XLOC_079719	389	coding	noncoding	noncoding	noncoding
MSTRG.71308.1		XLOC_079723	214	coding	noncoding	noncoding	noncoding
MSTRG.71314.1		XLOC_079726	292	coding	noncoding	noncoding	noncoding
MSTRG.71325.1		XLOC_079732	260	coding	noncoding	noncoding	noncoding
MSTRG.71343.1	CDH9	XLOC_079738	274	coding	noncoding	noncoding	noncoding
MSTRG.71344.1	CDH9	XLOC_079739	211	coding	noncoding	noncoding	noncoding
MSTRG.71357.1		XLOC_079750	445	coding	noncoding	noncoding	noncoding
MSTRG.71368.1		XLOC_079755	228	coding	noncoding	noncoding	noncoding
MSTRG.71429.1		XLOC_079791	262	coding	noncoding	noncoding	noncoding
MSTRG.71444.1		XLOC_079800	275	coding	noncoding	noncoding	noncoding
MSTRG.71449.1		XLOC_079804	215	coding	noncoding	noncoding	noncoding
MSTRG.71466.1	CDH6	XLOC_079808	269	coding	noncoding	noncoding	noncoding
MSTRG.71471.1	CDH6	XLOC_079811	318	coding	noncoding	noncoding	noncoding
MSTRG.71492.1	PDZD2	XLOC_079819	450	coding	noncoding	noncoding	noncoding
MSTRG.71497.1	PDZD2	XLOC_079823	227	coding	noncoding	noncoding	noncoding
MSTRG.71509.1	PDZD2	XLOC_079828	240	coding	noncoding	noncoding	noncoding
MSTRG.71512.1	PDZD2	XLOC_079830	356	coding	noncoding	noncoding	noncoding
MSTRG.71514.1	PDZD2	XLOC_079832	286	coding	noncoding	noncoding	noncoding
MSTRG.71483.1		XLOC_079843	281	coding	noncoding	noncoding	noncoding
MSTRG.71525.1		XLOC_079850	270	coding	noncoding	noncoding	noncoding
MSTRG.71564.1	ZFR	XLOC_079856	236	coding	noncoding	noncoding	noncoding
MSTRG.71552.1		XLOC_079861	311	coding	noncoding	noncoding	noncoding
MSTRG.71586.1	LINC02061	XLOC_079877	251	coding	noncoding	noncoding	noncoding
MSTRG.71605.1		XLOC_079889	306	coding	noncoding	noncoding	noncoding
MSTRG.71607.1		XLOC_079890	268	coding	noncoding	noncoding	noncoding
MSTRG.71629.1		XLOC_079902	216	coding	noncoding	noncoding	noncoding
NM_001012509.4	SLC45A2	XLOC_079922	2775	coding	coding	coding	coding
MSTRG.71678.1		XLOC_079954	432	coding	noncoding	coding	noncoding
MSTRG.71680.1		XLOC_079955	270	coding	noncoding	noncoding	noncoding
MSTRG.71693.1	RAI14	XLOC_079961	263	coding	noncoding	noncoding	noncoding
MSTRG.71709.1		XLOC_079968	293	coding	noncoding	noncoding	noncoding
MSTRG.71757.1		XLOC_079990	225	coding	noncoding	noncoding	noncoding
MSTRG.71950.1	SLC1A3	XLOC_080028	248	coding	noncoding	noncoding	noncoding
MSTRG.71919.1		XLOC_080039	275	coding	noncoding	noncoding	noncoding

MSTRG.72074.4	CPLANE1	XLOC_080046	27802	coding	coding	coding	coding
MSTRG.71994.1		XLOC_080063	312	coding	noncoding	noncoding	noncoding
NM_001364297.1	LIFR	XLOC_080076	10570	coding	coding	coding	coding
XM_017009312.1	RICTOR	XLOC_080095	9470	coding	coding	coding	coding
MSTRG.72096.1	C9	XLOC_080139	322	coding	noncoding	noncoding	noncoding
MSTRG.72126.1		XLOC_080145	300	coding	noncoding	noncoding	noncoding
MSTRG.72143.1		XLOC_080154	700	coding	noncoding	coding	noncoding
MSTRG.72159.1		XLOC_080161	279	coding	noncoding	noncoding	noncoding
MSTRG.72269.1		XLOC_080188	270	coding	noncoding	noncoding	noncoding
XM_011514117.3	C6	XLOC_080196	3873	coding	coding	coding	coding
MSTRG.72288.1	C6	XLOC_080197	245	coding	noncoding	noncoding	noncoding
MSTRG.72292.1	C6	XLOC_080199	277	coding	noncoding	noncoding	noncoding
NM_001005473.3	PLCXD3	XLOC_080202	7706	coding	noncoding	coding	coding
MSTRG.72350.1	OXCT1	XLOC_080217	416	coding	noncoding	noncoding	noncoding
MSTRG.72315.2	FBXO4	XLOC_080224	578	coding	coding	noncoding	noncoding
MSTRG.72302.1		XLOC_080229	311	coding	noncoding	noncoding	noncoding
MSTRG.72323.1	GHR	XLOC_080236	277	coding	noncoding	noncoding	noncoding
MSTRG.72332.1	GHR	XLOC_080240	280	coding	noncoding	noncoding	noncoding
MSTRG.72337.1	GHR	XLOC_080243	287	coding	noncoding	noncoding	noncoding
MSTRG.72321.1		XLOC_080247	334	coding	noncoding	noncoding	noncoding
MSTRG.72375.1		XLOC_080262	291	coding	noncoding	noncoding	noncoding
MSTRG.72383.1		XLOC_080265	235	coding	noncoding	noncoding	noncoding
MSTRG.72429.1		XLOC_080303	293	coding	coding	noncoding	noncoding
MSTRG.72482.1	CCL28	XLOC_080306	706	coding	noncoding	noncoding	noncoding
MSTRG.72539.6	NNT	XLOC_080318	670	coding	coding	noncoding	noncoding
MSTRG.72512.1		XLOC_080333	318	coding	noncoding	coding	noncoding
MSTRG.72516.1		XLOC_080335	275	coding	noncoding	noncoding	noncoding
MSTRG.72522.1		XLOC_080340	228	coding	noncoding	noncoding	noncoding
MSTRG.72523.1		XLOC_080341	233	coding	noncoding	noncoding	noncoding
MSTRG.72581.1		XLOC_080354	267	coding	coding	noncoding	noncoding
NM_021072.4	HCN1	XLOC_080358	9933	coding	coding	coding	coding
MSTRG.72839.1		XLOC_080415	314	coding	coding	noncoding	noncoding
MSTRG.72849.1		XLOC_080422	298	coding	noncoding	noncoding	noncoding
MSTRG.72860.1		XLOC_080430	278	coding	noncoding	noncoding	noncoding
MSTRG.72881.1		XLOC_080443	203	coding	noncoding	noncoding	noncoding
MSTRG.72886.1	LOC105378961	XLOC_080444	250	coding	noncoding	noncoding	noncoding
MSTRG.72900.1		XLOC_080452	266	coding	coding	noncoding	noncoding
MSTRG.72901.1		XLOC_080453	232	coding	noncoding	noncoding	noncoding
MSTRG.72907.1	LOC105378963	XLOC_080457	280	coding	noncoding	noncoding	noncoding
MSTRG.72924.1		XLOC_080481	299	coding	noncoding	noncoding	noncoding
MSTRG.72927.1		XLOC_080484	258	coding	noncoding	noncoding	noncoding
MSTRG.72948.1		XLOC_080489	300	coding	noncoding	noncoding	noncoding
MSTRG.72973.1		XLOC_080502	453	coding	noncoding	noncoding	noncoding

MSTRG.73402.1	LINC01033	XLOC_080563	305	coding	noncoding	noncoding	noncoding
MSTRG.73403.1	LINC01033	XLOC_080564	262	coding	noncoding	noncoding	noncoding
MSTRG.73019.1		XLOC_080565	246	coding	noncoding	noncoding	noncoding
MSTRG.73021.1		XLOC_080567	292	coding	noncoding	noncoding	noncoding
MSTRG.73022.1		XLOC_080568	237	coding	noncoding	noncoding	noncoding
MSTRG.73041.3	SNX18	XLOC_080569	4976	coding	coding	coding	noncoding
MSTRG.73115.1	CDC20B	XLOC_080625	293	coding	noncoding	noncoding	noncoding
MSTRG.73159.1		XLOC_080666	253	coding	noncoding	noncoding	noncoding
MSTRG.73179.1		XLOC_080681	290	coding	noncoding	noncoding	noncoding
MSTRG.73305.3	MAP3K1	XLOC_080707	4972	coding	coding	coding	noncoding
MSTRG.73321.1		XLOC_080718	268	coding	noncoding	noncoding	noncoding
MSTRG.73323.1		XLOC_080719	239	coding	noncoding	noncoding	noncoding
MSTRG.73419.1		XLOC_080721	434	coding	noncoding	noncoding	noncoding
MSTRG.73406.1		XLOC_080726	207	coding	coding	noncoding	noncoding
MSTRG.73410.1		XLOC_080728	304	coding	noncoding	noncoding	noncoding
MSTRG.73411.1		XLOC_080729	289	coding	noncoding	noncoding	noncoding
NM_001017992.4	ACTBL2	XLOC_080731	2794	coding	coding	coding	coding
MSTRG.73471.1		XLOC_080755	316	coding	noncoding	noncoding	noncoding
MSTRG.74670.1	PDE4D	XLOC_080810	266	coding	noncoding	noncoding	noncoding
MSTRG.74782.1	PDE4D	XLOC_080824	282	coding	noncoding	noncoding	noncoding
MSTRG.74860.1	PDE4D	XLOC_080834	220	coding	noncoding	noncoding	noncoding
NM_001104558.1	ELOVL7	XLOC_080845	3830	coding	coding	coding	coding
MSTRG.73567.1	ERCC8	XLOC_080848	245	coding	noncoding	noncoding	noncoding
MSTRG.73538.1	LOC105378998	XLOC_080885	288	coding	noncoding	noncoding	noncoding
MSTRG.73542.1	LOC105378998	XLOC_080887	269	coding	noncoding	noncoding	noncoding
MSTRG.73543.1	LOC105378998	XLOC_080888	297	coding	noncoding	noncoding	noncoding
MSTRG.73650.1	LOC107986418	XLOC_080908	254	coding	noncoding	noncoding	noncoding
MSTRG.73647.1		XLOC_080914	301	coding	noncoding	noncoding	noncoding
XR_001742672.1	LOC107986416	XLOC_080916	1970	coding	noncoding	noncoding	noncoding
MSTRG.73665.1		XLOC_080918	296	coding	coding	noncoding	noncoding
MSTRG.73673.1		XLOC_080924	252	coding	noncoding	noncoding	noncoding
NM_000524.3	HTR1A	XLOC_080925	2245	coding	coding	coding	coding
MSTRG.73762.1	RNF180	XLOC_080931	246	coding	noncoding	noncoding	noncoding
MSTRG.73764.1	RNF180	XLOC_080933	235	coding	noncoding	noncoding	noncoding
MSTRG.73766.1	RNF180	XLOC_080934	309	coding	noncoding	noncoding	noncoding
MSTRG.73768.1		XLOC_080936	255	coding	noncoding	noncoding	noncoding
MSTRG.73773.1	RGS7BP	XLOC_080938	338	coding	noncoding	noncoding	noncoding
MSTRG.73790.1	ADAMTS6	XLOC_080949	390	coding	noncoding	noncoding	noncoding
MSTRG.73826.1	ADAMTS6	XLOC_080956	302	coding	noncoding	noncoding	noncoding
MSTRG.73878.6	TRAPPC13	XLOC_080961	903	coding	coding	noncoding	noncoding
MSTRG.73865.1		XLOC_080970	235	coding	noncoding	noncoding	noncoding
MSTRG.73922.1		XLOC_080996	305	coding	coding	noncoding	noncoding
MSTRG.73956.1		XLOC_081010	308	coding	noncoding	noncoding	noncoding

MSTRG.73987.1		XLOC_081059	208 coding	noncoding	noncoding	noncoding
MSTRG.74000.1		XLOC_081067	276 coding	noncoding	noncoding	noncoding
MSTRG.74004.1		XLOC_081070	225 coding	noncoding	noncoding	noncoding
MSTRG.74012.1		XLOC_081075	261 coding	noncoding	noncoding	noncoding
MSTRG.74026.1	LINC02219	XLOC_081081	268 coding	noncoding	noncoding	noncoding
MSTRG.74076.3	PIK3R1	XLOC_081084	3991 coding	coding	noncoding	noncoding
XR_002956214.1	LOC105379011	XLOC_081093	8128 coding	noncoding	noncoding	noncoding
MSTRG.74056.1		XLOC_081100	261 coding	noncoding	noncoding	noncoding
MSTRG.74070.1	LINC02198	XLOC_081106	315 coding	noncoding	noncoding	noncoding
MSTRG.74163.2	SLC30A5	XLOC_081110	2591 coding	coding	noncoding	noncoding
MSTRG.74161.1		XLOC_081111	362 coding	noncoding	noncoding	noncoding
MSTRG.74190.2		XLOC_081127	1876 coding	noncoding	noncoding	noncoding
MSTRG.74183.1	GUSBP3	XLOC_081128	4976 coding	coding	coding	noncoding
NM_001346870.1	NAIP	XLOC_081161	6523 coding	coding	coding	coding
MSTRG.74238.1	LOC107986382	XLOC_081181	219 coding	noncoding	noncoding	noncoding
XR_001742729.2	LOC105379028	XLOC_081186	7996 coding	noncoding	coding	noncoding
MSTRG.74337.1	LOC105379028	XLOC_081187	267 coding	noncoding	noncoding	noncoding
MSTRG.74341.1	MAP1B	XLOC_081188	452 coding	noncoding	noncoding	noncoding
MSTRG.74401.1		XLOC_081220	325 coding	noncoding	noncoding	noncoding
MSTRG.74458.5	FCHO2	XLOC_081230	1892 coding	coding	noncoding	noncoding
MSTRG.74458.6	FCHO2	XLOC_081230	2346 coding	coding	noncoding	noncoding
NM_004472.3	FOXD1	XLOC_081243	2512 coding	coding	coding	coding
MSTRG.74450.1	LINC01386	XLOC_081245	259 coding	noncoding	noncoding	noncoding
MSTRG.74456.1		XLOC_081248	282 coding	noncoding	noncoding	noncoding
MSTRG.74564.1	ARHGEF28	XLOC_081281	257 coding	noncoding	noncoding	noncoding
MSTRG.74572.1	LOC105379039	XLOC_081316	309 coding	noncoding	noncoding	noncoding
MSTRG.74569.1		XLOC_081317	280 coding	noncoding	noncoding	noncoding
MSTRG.74574.1		XLOC_081318	244 coding	noncoding	noncoding	noncoding
MSTRG.74575.1		XLOC_081319	489 coding	noncoding	noncoding	noncoding
MSTRG.74609.1	ANKRD31	XLOC_081332	257 coding	noncoding	noncoding	noncoding
MSTRG.74909.1		XLOC_081355	270 coding	noncoding	noncoding	noncoding
MSTRG.74918.1	SV2C	XLOC_081357	267 coding	noncoding	noncoding	noncoding
MSTRG.75025.3	IQGAP2	XLOC_081371	5428 coding	coding	coding	noncoding
MSTRG.74950.1		XLOC_081381	251 coding	noncoding	noncoding	noncoding
MSTRG.75110.1	PDE8B	XLOC_081408	322 coding	noncoding	noncoding	noncoding
MSTRG.75085.1		XLOC_081436	236 coding	coding	noncoding	noncoding
MSTRG.75087.1		XLOC_081437	286 coding	noncoding	noncoding	noncoding
MSTRG.75167.1	AP3B1	XLOC_081473	266 coding	noncoding	noncoding	noncoding
MSTRG.75172.1	AP3B1	XLOC_081478	244 coding	noncoding	noncoding	noncoding
MSTRG.75175.1	AP3B1	XLOC_081481	263 coding	noncoding	noncoding	noncoding
XR_001742065.2	ARSB	XLOC_081506	4917 coding	coding	coding	coding
MSTRG.75290.1	ARSB	XLOC_081512	244 coding	noncoding	noncoding	noncoding
MSTRG.75294.1	ARSB	XLOC_081516	264 coding	noncoding	noncoding	noncoding

MSTRG.75185.1	DMGDH	XLOC_081518	207 coding	noncoding	noncoding	noncoding
MSTRG.75226.3	JMY	XLOC_081522	1743 coding	noncoding	coding	coding
MSTRG.75208.1	HOMER1	XLOC_081532	249 coding	noncoding	noncoding	noncoding
MSTRG.75191.1		XLOC_081533	405 coding	noncoding	noncoding	noncoding
MSTRG.75194.1		XLOC_081534	386 coding	noncoding	noncoding	noncoding
MSTRG.75344.1	CMYA5	XLOC_081550	306 coding	noncoding	coding	noncoding
MSTRG.75345.1	CMYA5	XLOC_081551	225 coding	noncoding	noncoding	noncoding
MSTRG.75352.1	LOC105379048	XLOC_081556	539 coding	noncoding	noncoding	noncoding
MSTRG.75400.1	SERINC5	XLOC_081576	232 coding	noncoding	noncoding	noncoding
MSTRG.75406.1	SERINC5	XLOC_081582	295 coding	noncoding	noncoding	noncoding
NR_003665.1	CRSP8P	XLOC_081593	1362 coding	coding	coding	coding
MSTRG.75302.1		XLOC_081594	288 coding	noncoding	noncoding	noncoding
MSTRG.75415.12	ZFYVE16	XLOC_081595	11944 coding	coding	noncoding	noncoding
MSTRG.75787.1		XLOC_081599	241 coding	noncoding	noncoding	noncoding
MSTRG.75788.1		XLOC_081600	400 coding	noncoding	noncoding	noncoding
MSTRG.75794.8	MSH3	XLOC_081604	895 coding	coding	noncoding	noncoding
MSTRG.75794.9	MSH3	XLOC_081604	1956 coding	coding	noncoding	noncoding
NM_012446.4	SSBP2	XLOC_081622	8994 coding	coding	coding	coding
NM_001345886.1	SSBP2	XLOC_081622	8919 coding	coding	coding	coding
MSTRG.75478.1	SSBP2	XLOC_081653	273 coding	noncoding	noncoding	noncoding
NM_001025.5	RPS23	XLOC_081686	3252 coding	coding	coding	noncoding
MSTRG.75513.1		XLOC_081694	219 coding	noncoding	noncoding	noncoding
MSTRG.75522.1		XLOC_081700	282 coding	noncoding	noncoding	noncoding
MSTRG.75614.1		XLOC_081741	263 coding	noncoding	noncoding	noncoding
MSTRG.75642.1		XLOC_081744	464 coding	noncoding	noncoding	noncoding
MSTRG.75658.1		XLOC_081748	275 coding	noncoding	noncoding	noncoding
MSTRG.75659.1		XLOC_081749	279 coding	coding	noncoding	noncoding
MSTRG.75667.1		XLOC_081752	309 coding	noncoding	noncoding	noncoding
MSTRG.75679.1		XLOC_081760	239 coding	noncoding	noncoding	noncoding
MSTRG.75684.1		XLOC_081765	206 coding	noncoding	noncoding	noncoding
MSTRG.75687.1		XLOC_081767	255 coding	noncoding	noncoding	noncoding
MSTRG.75697.1		XLOC_081774	287 coding	noncoding	noncoding	noncoding
MSTRG.75699.1		XLOC_081779	281 coding	noncoding	noncoding	noncoding
MSTRG.75737.1	LOC645261	XLOC_081804	259 coding	noncoding	noncoding	noncoding
MSTRG.75773.1		XLOC_081823	309 coding	noncoding	noncoding	noncoding
MSTRG.75899.1	TMEM161B-AS1	XLOC_081835	314 coding	coding	noncoding	noncoding
NM_002397.5	MEF2C	XLOC_081850	7281 coding	coding	coding	coding
NM_001364329.2	MEF2C	XLOC_081850	7470 coding	coding	coding	coding
MSTRG.76082.1	MEF2C-AS1	XLOC_081912	293 coding	noncoding	noncoding	noncoding
MSTRG.75935.1	LINC02161	XLOC_081952	309 coding	noncoding	noncoding	noncoding
MSTRG.75925.1		XLOC_081959	370 coding	noncoding	noncoding	noncoding
MSTRG.75962.1	LINC01339	XLOC_081968	248 coding	noncoding	noncoding	noncoding
MSTRG.76243.1	ADGRV1	XLOC_081988	281 coding	coding	noncoding	noncoding

MSTRG.76245.1	ADGRV1	XLOC_081990	275	coding	noncoding	noncoding	noncoding
MSTRG.76249.1	ADGRV1	XLOC_081994	244	coding	noncoding	noncoding	noncoding
MSTRG.76193.1	NR2F1-AS1	XLOC_082107	302	coding	coding	noncoding	noncoding
MSTRG.77961.1	FAM172A	XLOC_082112	297	coding	noncoding	noncoding	noncoding
MSTRG.78038.1	LOC105379087	XLOC_082185	270	coding	noncoding	noncoding	noncoding
MSTRG.78068.1	KIAA0825	XLOC_082209	319	coding	noncoding	noncoding	noncoding
MSTRG.76884.1	LOC101929710	XLOC_082341	311	coding	noncoding	noncoding	noncoding
MSTRG.76888.37	ERAP1	XLOC_082343	13613	coding	coding	coding	coding
MSTRG.76445.1		XLOC_082349	671	coding	noncoding	noncoding	noncoding
MSTRG.76415.1		XLOC_082441	230	coding	coding	noncoding	noncoding
MSTRG.76427.1		XLOC_082448	238	coding	noncoding	noncoding	noncoding
MSTRG.76428.1		XLOC_082449	210	coding	noncoding	noncoding	noncoding
MSTRG.76430.1		XLOC_082450	254	coding	noncoding	noncoding	noncoding
MSTRG.76546.1		XLOC_082476	304	coding	noncoding	coding	noncoding
MSTRG.76557.1		XLOC_082483	425	coding	noncoding	noncoding	noncoding
MSTRG.76760.1		XLOC_082544	394	coding	noncoding	noncoding	noncoding
MSTRG.77008.5	PAM	XLOC_082558	449	coding	coding	noncoding	noncoding
MSTRG.76927.1		XLOC_082574	221	coding	noncoding	noncoding	noncoding
MSTRG.77000.1		XLOC_082601	236	coding	noncoding	noncoding	noncoding
MSTRG.77001.1		XLOC_082602	288	coding	noncoding	noncoding	noncoding
MSTRG.77083.1		XLOC_082611	284	coding	noncoding	noncoding	noncoding
MSTRG.77087.1		XLOC_082615	262	coding	noncoding	noncoding	noncoding
MSTRG.77100.1		XLOC_082621	389	coding	noncoding	noncoding	noncoding
MSTRG.77128.1	LINC01950	XLOC_082634	268	coding	noncoding	noncoding	noncoding
MSTRG.77210.1		XLOC_082655	382	coding	noncoding	noncoding	noncoding
MSTRG.77289.12	MAN2A1	XLOC_082752	2810	coding	coding	coding	noncoding
MSTRG.77358.1		XLOC_082783	211	coding	noncoding	noncoding	noncoding
MSTRG.77364.1		XLOC_082787	255	coding	noncoding	noncoding	noncoding
MSTRG.77484.1	TMEM232	XLOC_082803	274	coding	noncoding	noncoding	noncoding
MSTRG.77488.1	TMEM232	XLOC_082805	248	coding	noncoding	noncoding	noncoding
MSTRG.77492.1	TMEM232	XLOC_082807	244	coding	coding	noncoding	noncoding
MSTRG.77493.1	TMEM232	XLOC_082808	308	coding	coding	noncoding	noncoding
MSTRG.77467.1		XLOC_082814	200	coding	noncoding	noncoding	noncoding
XM_017009041.2	STARD4	XLOC_082824	9115	coding	coding	noncoding	noncoding
MSTRG.77593.2	CAMK4	XLOC_082825	2365	coding	noncoding	noncoding	noncoding
MSTRG.77650.1	NREP	XLOC_082839	283	coding	noncoding	noncoding	noncoding
MSTRG.77658.1	NREP-AS1	XLOC_082843	261	coding	noncoding	noncoding	noncoding
MSTRG.77525.1		XLOC_082846	267	coding	noncoding	noncoding	noncoding
NM_001347887.2	EPB41L4A	XLOC_082849	3698	coding	coding	coding	coding
NM_032028.4	TSSK1B	XLOC_082898	2437	coding	coding	coding	coding
MSTRG.77691.1	LOC107986441	XLOC_082908	262	coding	noncoding	noncoding	noncoding
MSTRG.77728.1	KCNN2	XLOC_082926	228	coding	noncoding	noncoding	noncoding
MSTRG.77749.1	LOC101927078	XLOC_082936	285	coding	noncoding	noncoding	noncoding

MSTRG.77706.1		XLOC_082938	225	coding	coding	noncoding	noncoding
MSTRG.77708.1		XLOC_082939	235	coding	noncoding	noncoding	noncoding
MSTRG.77767.1	TRIM36	XLOC_082945	286	coding	noncoding	noncoding	noncoding
MSTRG.77860.1		XLOC_082960	278	coding	noncoding	noncoding	noncoding
MSTRG.78113.1		XLOC_083084	283	coding	noncoding	noncoding	noncoding
MSTRG.78130.1		XLOC_083091	251	coding	noncoding	noncoding	noncoding
MSTRG.78167.1	LINC02147	XLOC_083098	267	coding	noncoding	noncoding	noncoding
MSTRG.78174.1	LINC02147	XLOC_083102	323	coding	noncoding	noncoding	noncoding
MSTRG.78140.1		XLOC_083121	302	coding	noncoding	noncoding	noncoding
MSTRG.78237.1		XLOC_083143	385	coding	noncoding	noncoding	noncoding
MSTRG.78329.1		XLOC_083148	277	coding	noncoding	noncoding	noncoding
MSTRG.78335.1		XLOC_083152	234	coding	noncoding	noncoding	noncoding
MSTRG.78338.1		XLOC_083153	241	coding	noncoding	noncoding	noncoding
MSTRG.78343.1	LOC105379144	XLOC_083154	287	coding	noncoding	noncoding	noncoding
MSTRG.78408.1	PRR16	XLOC_083181	468	coding	noncoding	noncoding	noncoding
MSTRG.78431.1		XLOC_083199	234	coding	noncoding	noncoding	noncoding
MSTRG.78650.1	SNCAIP	XLOC_083236	245	coding	noncoding	noncoding	noncoding
MSTRG.78651.1	SNCAIP	XLOC_083237	214	coding	noncoding	noncoding	noncoding
MSTRG.78653.1	SNCAIP	XLOC_083239	254	coding	noncoding	noncoding	noncoding
MSTRG.78639.1	LINC02201	XLOC_083247	310	coding	noncoding	noncoding	noncoding
MSTRG.78660.1		XLOC_083269	459	coding	noncoding	noncoding	noncoding
MSTRG.78713.1		XLOC_083273	268	coding	noncoding	noncoding	noncoding
XM_011543186.2	CEP120	XLOC_083275	4319	coding	coding	coding	coding
MSTRG.78673.1		XLOC_083300	317	coding	noncoding	noncoding	noncoding
MSTRG.78745.1		XLOC_083310	246	coding	noncoding	noncoding	noncoding
MSTRG.78753.1		XLOC_083313	280	coding	noncoding	noncoding	noncoding
MSTRG.78785.1	LINC01170	XLOC_083330	248	coding	noncoding	noncoding	noncoding
MSTRG.78789.1	LINC01170	XLOC_083332	264	coding	noncoding	noncoding	noncoding
MSTRG.78771.1		XLOC_083337	289	coding	coding	noncoding	noncoding
MSTRG.78775.1		XLOC_083340	219	coding	noncoding	noncoding	noncoding
XM_005272039.3	ZNF608	XLOC_083341	6177	coding	noncoding	coding	coding
MSTRG.78838.1		XLOC_083362	239	coding	noncoding	noncoding	noncoding
MSTRG.78881.1	LOC101927421	XLOC_083366	258	coding	noncoding	noncoding	noncoding
MSTRG.78945.1	ALDH7A1	XLOC_083398	300	coding	noncoding	noncoding	noncoding
MSTRG.78935.1		XLOC_083401	359	coding	noncoding	noncoding	noncoding
MSTRG.78936.1		XLOC_083403	281	coding	noncoding	noncoding	noncoding
MSTRG.78973.1		XLOC_083444	203	coding	noncoding	noncoding	noncoding
MSTRG.79140.1	CCDC192	XLOC_083466	259	coding	noncoding	noncoding	noncoding
NR_152814.1	LINC01184	XLOC_083469	1806	coding	noncoding	coding	noncoding
MSTRG.79152.1	LINC01184	XLOC_083470	256	coding	noncoding	noncoding	noncoding
MSTRG.79073.1	LOC105379168	XLOC_083505	284	coding	noncoding	noncoding	noncoding
MSTRG.79125.1		XLOC_083563	288	coding	noncoding	noncoding	noncoding
MSTRG.79128.1		XLOC_083571	255	coding	noncoding	noncoding	noncoding

MSTRG.79301.1		XLOC_083572	304 coding	noncoding	noncoding	noncoding
MSTRG.79282.1		XLOC_083596	265 coding	noncoding	noncoding	noncoding
MSTRG.79286.1		XLOC_083598	266 coding	coding	noncoding	noncoding
MSTRG.79290.1		XLOC_083599	288 coding	noncoding	noncoding	noncoding
MSTRG.79296.1		XLOC_083603	265 coding	noncoding	noncoding	noncoding
MSTRG.79371.1		XLOC_083612	281 coding	noncoding	noncoding	noncoding
MSTRG.79407.1	RAPGEF6	XLOC_083638	324 coding	noncoding	noncoding	noncoding
MSTRG.79439.1	FNIP1	XLOC_083666	306 coding	noncoding	noncoding	noncoding
MSTRG.79331.1	MEIKIN	XLOC_083672	323 coding	noncoding	noncoding	noncoding
MSTRG.79340.1	MEIKIN	XLOC_083675	271 coding	noncoding	noncoding	noncoding
NM_001205250.1	ACSL6	XLOC_083679	6536 coding	coding	coding	coding
MSTRG.79352.1		XLOC_083681	254 coding	noncoding	noncoding	noncoding
MSTRG.79543.1		XLOC_083696	296 coding	noncoding	noncoding	noncoding
XM_011543308.3	GDF9	XLOC_083728	3634 coding	coding	coding	coding
MSTRG.79488.1		XLOC_083743	309 coding	noncoding	noncoding	noncoding
MSTRG.79489.1		XLOC_083744	282 coding	noncoding	noncoding	noncoding
MSTRG.79594.1	FSTL4	XLOC_083766	270 coding	noncoding	noncoding	noncoding
MSTRG.79627.1	VDAC1	XLOC_083779	316 coding	noncoding	noncoding	noncoding
MSTRG.79632.1	VDAC1	XLOC_083781	247 coding	noncoding	noncoding	noncoding
MSTRG.79614.1	LOC105379185	XLOC_083785	524 coding	noncoding	noncoding	noncoding
NM_006930.3	SKP1	XLOC_083788	2699 coding	coding	coding	coding
MSTRG.79640.1	TCF7	XLOC_083789	9018 coding	noncoding	coding	noncoding
MSTRG.79644.1	TCF7	XLOC_083792	2347 coding	noncoding	coding	coding
NR_149151.1	PPP2CA	XLOC_083793	2705 coding	coding	coding	coding
MSTRG.79683.1		XLOC_083812	246 coding	noncoding	noncoding	noncoding
MSTRG.79783.1	C5orf66	XLOC_083850	211 coding	noncoding	noncoding	noncoding
MSTRG.79814.1	SMAD5-AS1	XLOC_083889	7495 coding	coding	coding	noncoding
NM_020389.2	TRPC7	XLOC_083895	2935 coding	coding	coding	coding
MSTRG.79828.1		XLOC_083899	235 coding	coding	noncoding	noncoding
MSTRG.79853.1		XLOC_083911	280 coding	coding	noncoding	noncoding
MSTRG.79904.1	SPOCK1	XLOC_083928	413 coding	noncoding	noncoding	noncoding
MSTRG.79924.1		XLOC_083939	240 coding	noncoding	noncoding	noncoding
MSTRG.79968.1		XLOC_083942	461 coding	noncoding	noncoding	noncoding
MSTRG.80003.1	MYOT	XLOC_083950	301 coding	noncoding	noncoding	noncoding
MSTRG.80007.1		XLOC_083962	306 coding	noncoding	noncoding	noncoding
XM_024446227.1	NME5	XLOC_083963	2201 coding	coding	noncoding	coding
MSTRG.80011.1	NME5	XLOC_083964	385 coding	noncoding	noncoding	noncoding
MSTRG.80032.1	CDC23	XLOC_083969	294 coding	noncoding	noncoding	noncoding
MSTRG.80013.1		XLOC_083972	260 coding	noncoding	noncoding	noncoding
MSTRG.80036.1	REEP2	XLOC_083976	219 coding	noncoding	noncoding	noncoding
MSTRG.80043.1		XLOC_083985	239 coding	noncoding	noncoding	noncoding
MSTRG.80076.1	HSPA9	XLOC_083988	291 coding	noncoding	noncoding	noncoding
MSTRG.80128.3	CTNNA1	XLOC_083995	1562 coding	coding	noncoding	noncoding

MSTRG.80081.1		XLOC_084017	281	coding	noncoding	noncoding	noncoding
MSTRG.80114.1		XLOC_084034	267	coding	noncoding	noncoding	noncoding
MSTRG.80176.5	UBE2D2	XLOC_084035	527	coding	coding	noncoding	noncoding
MSTRG.80170.1	PSD2	XLOC_084046	266	coding	noncoding	noncoding	noncoding
MSTRG.80206.1	NRG2	XLOC_084056	508	coding	noncoding	noncoding	noncoding
MSTRG.80213.1	NRG2	XLOC_084058	610	coding	noncoding	noncoding	noncoding
MSTRG.80233.1		XLOC_084078	403	coding	noncoding	noncoding	noncoding
MSTRG.80278.9	ANKHD1-EIF4EBP3	XLOC_084080	8227	coding	coding	coding	noncoding
MSTRG.80278.8	ANKHD1-EIF4EBP3	XLOC_084080	8284	coding	coding	coding	noncoding
MSTRG.80314.1	TMCO6	XLOC_084090	299	coding	noncoding	noncoding	noncoding
MSTRG.80320.21	ZMAT2	XLOC_084094	1528	coding	coding	noncoding	noncoding
XM_024446283.1	LOC112267934	XLOC_084095	10200	coding	coding	coding	coding
MSTRG.80253.1		XLOC_084099	219	coding	noncoding	noncoding	noncoding
MSTRG.80418.1	PCDHGB8P	XLOC_084122	231	coding	noncoding	noncoding	noncoding
MSTRG.80437.1	DIAPH1	XLOC_084137	303	coding	noncoding	noncoding	noncoding
XM_017009910.2	SPRY4	XLOC_084182	5977	coding	coding	coding	coding
MSTRG.80396.1	FGF1	XLOC_084191	265	coding	noncoding	noncoding	noncoding
MSTRG.80990.23	ARHGAP26	XLOC_084195	3096	coding	coding	noncoding	noncoding
MSTRG.80990.35	ARHGAP26	XLOC_084195	9710	coding	noncoding	coding	noncoding
MSTRG.81018.2	ARHGAP26	XLOC_084196	10715	coding	noncoding	coding	noncoding
NM_001364184.2	NR3C1	XLOC_084212	6412	coding	coding	coding	coding
MSTRG.80491.1		XLOC_084237	255	coding	noncoding	noncoding	noncoding
MSTRG.80496.1		XLOC_084241	304	coding	noncoding	noncoding	noncoding
MSTRG.80511.1		XLOC_084259	288	coding	coding	noncoding	noncoding
MSTRG.80556.1		XLOC_084267	230	coding	noncoding	noncoding	noncoding
XM_017009128.1	PRELID2	XLOC_084275	2826	coding	coding	coding	coding
MSTRG.80574.1	GRXCR2	XLOC_084282	242	coding	noncoding	noncoding	noncoding
MSTRG.80666.1		XLOC_084299	220	coding	noncoding	noncoding	noncoding
MSTRG.80692.5	TCERG1	XLOC_084303	1180	coding	coding	noncoding	noncoding
MSTRG.80686.1		XLOC_084340	324	coding	noncoding	noncoding	noncoding
MSTRG.80709.1	STK32A-AS1	XLOC_084348	213	coding	noncoding	noncoding	noncoding
NM_001197294.2	DPYSL3	XLOC_084354	5492	coding	coding	coding	coding
MSTRG.80797.1	JAKMIP2-AS1	XLOC_084356	346	coding	noncoding	noncoding	noncoding
NM_001354966.1	SPINK1	XLOC_084372	643	coding	coding	noncoding	noncoding
MSTRG.80735.1		XLOC_084378	245	coding	noncoding	noncoding	noncoding
MSTRG.80756.1		XLOC_084385	227	coding	noncoding	noncoding	noncoding
NM_024577.3	SH3TC2	XLOC_084410	26580	coding	coding	coding	coding
MSTRG.80926.1	AFAP1L1	XLOC_084423	265	coding	noncoding	noncoding	noncoding
MSTRG.80955.1		XLOC_084429	354	coding	noncoding	noncoding	noncoding
MSTRG.80982.1	ARHGEF37	XLOC_084440	292	coding	noncoding	noncoding	noncoding
MSTRG.80989.1		XLOC_084478	253	coding	noncoding	noncoding	noncoding
NM_001355017.2	PDGFRB	XLOC_084484	5734	coding	coding	coding	coding
MSTRG.81216.1		XLOC_084490	234	coding	noncoding	noncoding	noncoding

MSTRG.81251.1	TCOF1	XLOC_084506	451 coding	noncoding	noncoding	noncoding
MSTRG.81236.4	RPS14	XLOC_084510	961 coding	coding	coding	noncoding
MSTRG.81263.3	NDST1-AS1	XLOC_084512	3969 coding	coding	coding	coding
MSTRG.81352.1		XLOC_084560	841 coding	noncoding	noncoding	noncoding
MSTRG.81354.1	GM2A	XLOC_084562	3720 coding	coding	noncoding	noncoding
MSTRG.81385.1	LOC105378234	XLOC_084569	284 coding	noncoding	noncoding	noncoding
MSTRG.81359.1		XLOC_084593	323 coding	noncoding	noncoding	noncoding
MSTRG.81438.1		XLOC_084640	220 coding	noncoding	noncoding	noncoding
MSTRG.81487.1	LINC01470	XLOC_084644	301 coding	noncoding	noncoding	noncoding
MSTRG.81473.1		XLOC_084645	293 coding	noncoding	noncoding	noncoding
MSTRG.81476.1		XLOC_084647	305 coding	noncoding	noncoding	noncoding
MSTRG.81540.1		XLOC_084673	547 coding	noncoding	noncoding	noncoding
MSTRG.81552.1		XLOC_084684	309 coding	coding	noncoding	noncoding
MSTRG.81559.1		XLOC_084689	285 coding	noncoding	noncoding	noncoding
MSTRG.81582.1	LARP1	XLOC_084692	265 coding	noncoding	noncoding	noncoding
MSTRG.81589.1	LARP1	XLOC_084696	216 coding	noncoding	noncoding	noncoding
MSTRG.81563.1		XLOC_084705	312 coding	coding	noncoding	noncoding
MSTRG.81573.1	GEMIN5	XLOC_084707	257 coding	noncoding	noncoding	noncoding
MSTRG.81624.1		XLOC_084724	440 coding	noncoding	noncoding	noncoding
MSTRG.81627.1		XLOC_084726	222 coding	noncoding	noncoding	noncoding
MSTRG.82316.1	SGCD	XLOC_084778	287 coding	noncoding	noncoding	noncoding
MSTRG.81679.1		XLOC_084800	249 coding	noncoding	noncoding	noncoding
MSTRG.81687.1	HAVCR1	XLOC_084802	240 coding	noncoding	noncoding	noncoding
MSTRG.81688.1	HAVCR1	XLOC_084803	386 coding	noncoding	noncoding	noncoding
MSTRG.81671.1		XLOC_084807	300 coding	noncoding	noncoding	noncoding
MSTRG.81705.1		XLOC_084834	977 coding	noncoding	noncoding	noncoding
XM_017010087.2	CLINT1	XLOC_084842	3438 coding	coding	coding	coding
MSTRG.81734.1		XLOC_084851	436 coding	noncoding	noncoding	noncoding
MSTRG.81735.1		XLOC_084852	286 coding	noncoding	noncoding	noncoding
MSTRG.81736.1		XLOC_084853	265 coding	coding	coding	noncoding
MSTRG.81773.1		XLOC_084863	276 coding	noncoding	noncoding	noncoding
MSTRG.81822.1		XLOC_084887	295 coding	noncoding	noncoding	noncoding
MSTRG.81924.2	TTC1	XLOC_084947	1216 coding	noncoding	noncoding	noncoding
XM_011534425.2	C1QTNF2	XLOC_084963	2739 coding	coding	coding	coding
MSTRG.82003.1	SLU7	XLOC_084967	299 coding	noncoding	noncoding	noncoding
MSTRG.82067.1	ATP10B	XLOC_084985	273 coding	coding	noncoding	noncoding
MSTRG.82068.1	ATP10B	XLOC_084986	272 coding	noncoding	noncoding	noncoding
MSTRG.82013.1		XLOC_084993	295 coding	noncoding	noncoding	noncoding
MSTRG.82034.1	GABRB2	XLOC_085000	247 coding	noncoding	noncoding	noncoding
MSTRG.82041.1		XLOC_085011	396 coding	coding	noncoding	noncoding
MSTRG.82082.1		XLOC_085013	232 coding	noncoding	noncoding	noncoding
MSTRG.82094.1		XLOC_085023	299 coding	noncoding	noncoding	noncoding
MSTRG.82098.1	LOC105377697	XLOC_085025	286 coding	noncoding	coding	noncoding

MSTRG.82118.1		XLOC_085035	293	coding	noncoding	noncoding	noncoding
MSTRG.82146.1	LOC105377700	XLOC_085046	322	coding	noncoding	noncoding	noncoding
MSTRG.82164.1		XLOC_085059	532	coding	noncoding	noncoding	noncoding
MSTRG.82189.1		XLOC_085071	305	coding	noncoding	noncoding	noncoding
MSTRG.82220.1		XLOC_085088	287	coding	noncoding	noncoding	noncoding
MSTRG.82234.1		XLOC_085091	293	coding	noncoding	noncoding	noncoding
MSTRG.82239.1		XLOC_085094	317	coding	noncoding	noncoding	noncoding
MSTRG.82348.1		XLOC_085097	224	coding	noncoding	noncoding	noncoding
MSTRG.82377.1		XLOC_085117	215	coding	noncoding	noncoding	noncoding
MSTRG.82379.1		XLOC_085119	242	coding	noncoding	noncoding	noncoding
MSTRG.82411.1	TENM2	XLOC_085131	368	coding	coding	noncoding	noncoding
MSTRG.82420.1	TENM2	XLOC_085135	325	coding	noncoding	noncoding	noncoding
MSTRG.82387.1	WWC1	XLOC_085153	461	coding	noncoding	noncoding	noncoding
MSTRG.82390.1	WWC1	XLOC_085154	239	coding	noncoding	noncoding	noncoding
MSTRG.82509.1	LOC105377715	XLOC_085180	250	coding	noncoding	noncoding	noncoding
MSTRG.82513.1	LOC105377714	XLOC_085183	238	coding	noncoding	noncoding	noncoding
MSTRG.82766.4	INSYN2B	XLOC_085186	19449	coding	coding	coding	coding
MSTRG.82766.8	DOCK2	XLOC_085186	6775	coding	coding	coding	noncoding
MSTRG.82519.1		XLOC_085189	280	coding	noncoding	noncoding	noncoding
MSTRG.82580.1	RANBP17	XLOC_085241	292	coding	noncoding	noncoding	noncoding
MSTRG.82585.1	RANBP17	XLOC_085244	228	coding	noncoding	noncoding	noncoding
MSTRG.82569.1		XLOC_085247	227	coding	noncoding	noncoding	noncoding
MSTRG.82685.1	LOC100288254	XLOC_085305	290	coding	noncoding	noncoding	noncoding
MSTRG.82903.1	CREBRF	XLOC_085336	338	coding	noncoding	noncoding	noncoding
MSTRG.82906.1		XLOC_085338	258	coding	noncoding	noncoding	noncoding
MSTRG.82911.1		XLOC_085342	313	coding	noncoding	noncoding	noncoding
MSTRG.82958.1		XLOC_085379	306	coding	noncoding	noncoding	noncoding
MSTRG.82998.1	NSG2	XLOC_085390	232	coding	noncoding	noncoding	noncoding
MSTRG.83001.1		XLOC_085396	244	coding	noncoding	noncoding	noncoding
MSTRG.83024.1	LINC01411	XLOC_085397	491	coding	noncoding	noncoding	noncoding
MSTRG.83031.1	LOC105377740	XLOC_085401	282	coding	noncoding	noncoding	noncoding
MSTRG.83036.1		XLOC_085403	261	coding	noncoding	noncoding	noncoding
MSTRG.83065.1		XLOC_085418	580	coding	noncoding	noncoding	noncoding
XM_017009652.1	KIAA1191	XLOC_085461	2261	coding	coding	coding	coding
MSTRG.83174.6	NOP16	XLOC_085469	1782	coding	coding	coding	coding
MSTRG.83166.1		XLOC_085473	378	coding	noncoding	noncoding	noncoding
MSTRG.83205.1		XLOC_085487	204	coding	noncoding	noncoding	noncoding
XM_017009578.1	UIMC1	XLOC_085494	1818	coding	coding	coding	coding
MSTRG.83249.1	UIMC1	XLOC_085495	288	coding	noncoding	noncoding	noncoding
MSTRG.83288.2	MXD3	XLOC_085512	1160	coding	coding	coding	noncoding
NR_038915.1	PRR7-AS1	XLOC_085523	1600	coding	noncoding	coding	noncoding
MSTRG.83294.1	LOC105377750	XLOC_085539	347	coding	noncoding	noncoding	noncoding
MSTRG.83298.1		XLOC_085543	242	coding	noncoding	noncoding	noncoding

NM_006261.4	PROP1	XLOC_085567	1464	coding	coding	coding	coding
MSTRG.83345.1		XLOC_085570	236	coding	noncoding	noncoding	noncoding
XM_017010139.1	LOC102724657	XLOC_085592	1789	coding	noncoding	coding	coding
NM_005649.3	ZNF354A	XLOC_085593	2521	coding	coding	coding	coding
MSTRG.83423.1		XLOC_085598	307	coding	noncoding	noncoding	noncoding
NM_000843.4	GRM6	XLOC_085613	6025	coding	coding	coding	coding
MSTRG.83465.1		XLOC_085618	313	coding	noncoding	noncoding	noncoding
MSTRG.83479.1	ADAMTS2	XLOC_085626	246	coding	noncoding	noncoding	noncoding
NM_001364244.2	HNRNPH1	XLOC_085634	2210	coding	coding	coding	coding
NM_001364253.1	HNRNPH1	XLOC_085634	2692	coding	coding	coding	coding
MSTRG.83495.1	CANX	XLOC_085638	2641	coding	coding	noncoding	noncoding
MSTRG.83535.5	RNF130	XLOC_085646	622	coding	coding	coding	noncoding
MSTRG.83519.1		XLOC_085661	241	coding	noncoding	noncoding	noncoding
MSTRG.83529.1	RASGEF1C	XLOC_085668	259	coding	noncoding	noncoding	noncoding
MSTRG.83580.1	MAPK9	XLOC_085672	258	coding	noncoding	noncoding	noncoding
MSTRG.83570.1		XLOC_085681	322	coding	noncoding	noncoding	noncoding
MSTRG.83573.1		XLOC_085683	277	coding	noncoding	noncoding	noncoding
MSTRG.83592.7	CNOT6	XLOC_085684	2067	coding	coding	noncoding	noncoding
MSTRG.83586.1		XLOC_085686	325	coding	noncoding	noncoding	noncoding
MSTRG.83608.1	FLT4	XLOC_085691	312	coding	noncoding	noncoding	noncoding
XM_017009719.2	ZFP62	XLOC_085705	4022	coding	coding	coding	coding
MSTRG.83621.1		XLOC_085707	233	coding	noncoding	noncoding	noncoding
MSTRG.83634.1		XLOC_085715	228	coding	noncoding	noncoding	noncoding
NM_032765.4	TRIM52	XLOC_085736	3491	coding	coding	coding	coding
MSTRG.83754.1		XLOC_085772	270	coding	noncoding	noncoding	noncoding
MSTRG.83764.1		XLOC_085779	276	coding	noncoding	noncoding	noncoding
MSTRG.83777.1		XLOC_085785	286	coding	noncoding	noncoding	noncoding
MSTRG.83780.1		XLOC_085790	366	coding	noncoding	noncoding	noncoding
MSTRG.83793.1	LOC102723944	XLOC_085796	259	coding	noncoding	noncoding	noncoding
MSTRG.84461.1	GMDS-DT	XLOC_085834	263	coding	noncoding	noncoding	noncoding
NM_130395.3	WRNIP1	XLOC_085848	3823	coding	coding	coding	coding
MSTRG.83916.1	BPHL	XLOC_085881	241	coding	noncoding	noncoding	noncoding
XM_011514591.3	PSMG4	XLOC_085888	1389	coding	noncoding	noncoding	coding
MSTRG.83929.1		XLOC_085909	274	coding	noncoding	noncoding	noncoding
MSTRG.83935.1	LOC100507336	XLOC_085911	219	coding	noncoding	noncoding	noncoding
MSTRG.83956.1		XLOC_085919	277	coding	noncoding	noncoding	noncoding
MSTRG.83957.1		XLOC_085920	290	coding	noncoding	noncoding	noncoding
XM_017011420.2	PRPF4B	XLOC_085944	6212	coding	coding	coding	coding
MSTRG.84103.1		XLOC_086039	246	coding	noncoding	noncoding	noncoding
MSTRG.84107.1		XLOC_086047	252	coding	noncoding	noncoding	noncoding
MSTRG.84204.1		XLOC_086113	379	coding	noncoding	noncoding	noncoding
MSTRG.84285.1	CAGE1	XLOC_086136	237	coding	noncoding	noncoding	noncoding
MSTRG.84314.1		XLOC_086158	278	coding	noncoding	noncoding	noncoding

MSTRG.84530.1		XLOC_086211	319 coding	noncoding	noncoding	noncoding
MSTRG.84532.1		XLOC_086212	256 coding	noncoding	noncoding	noncoding
MSTRG.84538.1		XLOC_086215	242 coding	noncoding	noncoding	noncoding
MSTRG.84605.1		XLOC_086246	245 coding	noncoding	noncoding	noncoding
XM_017010732.2	GCNT2	XLOC_086254	1713 coding	coding	coding	coding
MSTRG.84666.1	LOC101928191	XLOC_086269	318 coding	noncoding	noncoding	noncoding
MSTRG.84680.1		XLOC_086279	402 coding	noncoding	noncoding	noncoding
MSTRG.84723.1		XLOC_086302	254 coding	noncoding	noncoding	noncoding
XM_011514547.2	HIVEP1	XLOC_086321	9076 coding	coding	coding	coding
MSTRG.84946.1	HIVEP1	XLOC_086351	270 coding	noncoding	noncoding	noncoding
MSTRG.84770.1		XLOC_086355	369 coding	noncoding	noncoding	noncoding
MSTRG.84773.1		XLOC_086359	269 coding	noncoding	noncoding	noncoding
MSTRG.84787.1		XLOC_086362	302 coding	noncoding	noncoding	noncoding
MSTRG.84788.1		XLOC_086363	204 coding	noncoding	noncoding	noncoding
MSTRG.84800.1		XLOC_086386	307 coding	noncoding	noncoding	noncoding
MSTRG.84865.1		XLOC_086392	212 coding	noncoding	noncoding	noncoding
MSTRG.84980.1		XLOC_086402	293 coding	noncoding	noncoding	noncoding
MSTRG.85050.1		XLOC_086416	263 coding	noncoding	noncoding	noncoding
MSTRG.85084.1	LOC105374945	XLOC_086469	273 coding	noncoding	noncoding	noncoding
MSTRG.85058.1		XLOC_086476	656 coding	noncoding	noncoding	noncoding
MSTRG.85066.1		XLOC_086478	281 coding	noncoding	noncoding	noncoding
MSTRG.85067.1		XLOC_086479	236 coding	noncoding	noncoding	noncoding
MSTRG.85092.1		XLOC_086480	290 coding	noncoding	noncoding	noncoding
MSTRG.85094.1		XLOC_086481	263 coding	noncoding	noncoding	noncoding
MSTRG.85164.1		XLOC_086489	269 coding	noncoding	noncoding	noncoding
XM_017010789.1	MYLIP	XLOC_086496	1627 coding	coding	coding	coding
MSTRG.85200.1		XLOC_086522	251 coding	noncoding	noncoding	noncoding
MSTRG.85235.1		XLOC_086534	202 coding	noncoding	noncoding	noncoding
MSTRG.85250.1	CAP2	XLOC_086541	291 coding	noncoding	noncoding	noncoding
MSTRG.85252.1	CAP2	XLOC_086543	259 coding	noncoding	noncoding	noncoding
MSTRG.85267.1		XLOC_086550	356 coding	noncoding	coding	noncoding
MSTRG.85276.1		XLOC_086553	254 coding	noncoding	noncoding	noncoding
MSTRG.85334.1	LOC105374955	XLOC_086595	248 coding	noncoding	noncoding	noncoding
MSTRG.85336.1		XLOC_086600	228 coding	noncoding	noncoding	noncoding
MSTRG.85390.1		XLOC_086609	252 coding	noncoding	noncoding	noncoding
MSTRG.85404.1		XLOC_086614	256 coding	noncoding	noncoding	noncoding
MSTRG.85406.1		XLOC_086616	219 coding	noncoding	noncoding	noncoding
MSTRG.85410.1	LOC105374959	XLOC_086617	261 coding	noncoding	noncoding	noncoding
MSTRG.85429.1		XLOC_086633	223 coding	noncoding	noncoding	noncoding
MSTRG.85554.1	LOC101928573	XLOC_086650	275 coding	noncoding	noncoding	noncoding
MSTRG.85562.1		XLOC_086671	229 coding	noncoding	noncoding	noncoding
MSTRG.85563.1		XLOC_086672	241 coding	noncoding	noncoding	noncoding
MSTRG.85815.1	CDKAL1	XLOC_086681	258 coding	noncoding	noncoding	noncoding

MSTRG.85821.1	CDKAL1	XLOC_086686	271 coding	noncoding	noncoding	noncoding
MSTRG.85837.1	CDKAL1	XLOC_086700	232 coding	noncoding	noncoding	noncoding
MSTRG.85598.1		XLOC_086723	333 coding	noncoding	noncoding	noncoding
MSTRG.85601.1		XLOC_086724	279 coding	noncoding	noncoding	noncoding
MSTRG.85610.1		XLOC_086729	312 coding	noncoding	noncoding	noncoding
MSTRG.85635.1	CASC15	XLOC_086736	252 coding	noncoding	noncoding	noncoding
MSTRG.85655.1	CASC15	XLOC_086742	294 coding	noncoding	noncoding	noncoding
MSTRG.85673.1	LOC105374971	XLOC_086753	204 coding	noncoding	noncoding	noncoding
MSTRG.85700.1	LOC105374974	XLOC_086767	249 coding	coding	noncoding	noncoding
MSTRG.85703.1	LOC105374974	XLOC_086769	211 coding	coding	noncoding	noncoding
MSTRG.85726.1		XLOC_086786	258 coding	noncoding	noncoding	noncoding
MSTRG.85760.1	GPLD1	XLOC_086799	271 coding	noncoding	noncoding	noncoding
MSTRG.85764.1		XLOC_086824	311 coding	noncoding	noncoding	noncoding
XM_005249159.2	GMNN	XLOC_086825	1388 coding	coding	coding	coding
MSTRG.85864.1		XLOC_086834	269 coding	noncoding	noncoding	noncoding
NM_017640.6	CARMIL1	XLOC_086845	5485 coding	coding	coding	coding
NM_003542.3	HIST1H4C	XLOC_086907	390 coding	coding	coding	coding
MSTRG.86132.1		XLOC_086937	262 coding	noncoding	noncoding	noncoding
MSTRG.86133.1		XLOC_086942	257 coding	noncoding	noncoding	noncoding
XM_017010211.1	BTN3A2	XLOC_086943	3793 coding	coding	coding	coding
XM_005248831.4	BTN3A2	XLOC_086943	3577 coding	coding	coding	coding
XM_017010213.2	BTN3A2	XLOC_086943	1428 coding	coding	coding	coding
XM_005248833.2	BTN3A1	XLOC_086949	3381 coding	coding	coding	coding
NM_001145009.2	BTN3A1	XLOC_086949	4145 coding	coding	coding	coding
MSTRG.86134.1	HCG11	XLOC_086964	295 coding	noncoding	noncoding	noncoding
MSTRG.86181.1	LINC00240	XLOC_087015	237 coding	noncoding	noncoding	noncoding
MSTRG.86191.1	TRS-GCT2-1	XLOC_087039	312 coding	noncoding	noncoding	noncoding
MSTRG.86195.1	TRS-GCT2-1	XLOC_087040	223 coding	noncoding	noncoding	noncoding
MSTRG.86196.1		XLOC_087041	280 coding	noncoding	noncoding	noncoding
MSTRG.86247.1	ZNF391	XLOC_087051	288 coding	noncoding	noncoding	noncoding
MSTRG.86258.1		XLOC_087063	300 coding	noncoding	noncoding	noncoding
MSTRG.86263.1		XLOC_087068	249 coding	noncoding	noncoding	noncoding
MSTRG.86280.1		XLOC_087073	292 coding	noncoding	noncoding	noncoding
MSTRG.86281.1		XLOC_087075	255 coding	noncoding	noncoding	noncoding
MSTRG.86339.1		XLOC_087098	704 coding	noncoding	noncoding	noncoding
MSTRG.86314.1		XLOC_087104	265 coding	noncoding	noncoding	noncoding
XM_011514944.2	PGBD1	XLOC_087133	4502 coding	coding	coding	coding
NR_103864.1	ZNRD1	XLOC_087206	661 coding	coding	noncoding	noncoding
NM_001190724.3	ATAT1	XLOC_087226	2861 coding	coding	coding	coding
MSTRG.86505.1	IER3	XLOC_087235	272 coding	noncoding	noncoding	noncoding
XM_011514914.2	LST1	XLOC_087266	758 coding	coding	noncoding	noncoding
NM_004638.4	PRRC2A	XLOC_087269	6903 coding	coding	coding	coding
MSTRG.86636.2		XLOC_087327	2888 coding	noncoding	noncoding	noncoding

MSTRG.86647.2	TAP2	XLOC_087334	3877 coding	coding	noncoding	coding
NM_022551.3	RPS18	XLOC_087363	549 coding	coding	coding	coding
MSTRG.86699.1		XLOC_087376	240 coding	noncoding	noncoding	noncoding
XR_002956337.1	LOC105375027	XLOC_087399	10339 coding	noncoding	coding	noncoding
MSTRG.86731.1		XLOC_087403	244 coding	noncoding	noncoding	noncoding
MSTRG.86798.1	RPS10-NUDT3	XLOC_087413	362 coding	noncoding	noncoding	noncoding
MSTRG.86777.1		XLOC_087415	257 coding	noncoding	noncoding	noncoding
MSTRG.86802.1		XLOC_087424	236 coding	noncoding	noncoding	noncoding
MSTRG.86836.1	TCP11	XLOC_087450	287 coding	noncoding	noncoding	noncoding
MSTRG.86845.1		XLOC_087469	301 coding	coding	noncoding	noncoding
MSTRG.86890.1	PPARD	XLOC_087471	298 coding	noncoding	noncoding	noncoding
MSTRG.86850.1		XLOC_087479	254 coding	noncoding	noncoding	noncoding
MSTRG.86886.1	TULP1	XLOC_087485	235 coding	noncoding	noncoding	noncoding
MSTRG.86940.4	FKBP5	XLOC_087487	3937 coding	coding	coding	coding
MSTRG.86940.10	FKBP5	XLOC_087487	1059 coding	coding	noncoding	coding
MSTRG.86938.1		XLOC_087496	244 coding	noncoding	noncoding	noncoding
MSTRG.87017.1	MAPK14	XLOC_087521	235 coding	noncoding	noncoding	noncoding
MSTRG.87064.1		XLOC_087565	290 coding	noncoding	noncoding	noncoding
MSTRG.87115.1	CPNE5	XLOC_087569	270 coding	noncoding	noncoding	noncoding
MSTRG.87127.1		XLOC_087582	207 coding	noncoding	noncoding	noncoding
MSTRG.87133.1		XLOC_087586	264 coding	noncoding	noncoding	noncoding
MSTRG.87149.1	TBC1D22B	XLOC_087594	300 coding	noncoding	noncoding	noncoding
MSTRG.87141.1	LOC105375040	XLOC_087599	222 coding	noncoding	noncoding	noncoding
MSTRG.87374.1	ZFAND3	XLOC_087637	229 coding	noncoding	coding	noncoding
NM_001206927.2	DNAH8	XLOC_087739	14663 coding	coding	noncoding	coding
MSTRG.87213.1	DNAH8	XLOC_087740	330 coding	noncoding	noncoding	noncoding
MSTRG.87204.1		XLOC_087756	201 coding	noncoding	noncoding	noncoding
XM_006715045.3	DAAM2	XLOC_087769	7438 coding	coding	coding	coding
MSTRG.87259.1	LOC102723789	XLOC_087775	227 coding	noncoding	noncoding	noncoding
MSTRG.87255.1		XLOC_087777	295 coding	noncoding	noncoding	noncoding
MSTRG.87321.1	LOC105375053	XLOC_087819	217 coding	coding	noncoding	noncoding
MSTRG.87561.1		XLOC_087862	299 coding	noncoding	noncoding	noncoding
MSTRG.87579.1		XLOC_087876	309 coding	noncoding	noncoding	noncoding
MSTRG.87581.1		XLOC_087877	301 coding	noncoding	noncoding	noncoding
MSTRG.87600.1	PGC	XLOC_087884	213 coding	noncoding	noncoding	noncoding
MSTRG.87731.1	USP49	XLOC_087896	218 coding	noncoding	noncoding	noncoding
XR_926799.2	LOC105375059	XLOC_087906	1172 coding	noncoding	noncoding	noncoding
MSTRG.87626.1	TAF8	XLOC_087913	205 coding	noncoding	noncoding	noncoding
NM_001319061.1	GUCA1A	XLOC_087914	2331 coding	coding	coding	coding
MSTRG.87637.1		XLOC_087926	289 coding	noncoding	noncoding	noncoding
MSTRG.87821.1		XLOC_087932	292 coding	noncoding	noncoding	noncoding
MSTRG.87843.1	UBR2	XLOC_087952	331 coding	noncoding	noncoding	noncoding
XM_024446389.1	BICRAL	XLOC_087961	6407 coding	coding	coding	coding

NR_134562.1	RPL7L1	XLOC_087972	3922	coding	coding	coding	noncoding
MSTRG.87908.1	CUL7	XLOC_087989	245	coding	noncoding	noncoding	noncoding
MSTRG.87935.1	CUL9	XLOC_088004	297	coding	noncoding	noncoding	noncoding
MSTRG.87930.1		XLOC_088015	247	coding	noncoding	noncoding	noncoding
XR_002956312.1	ABCC10	XLOC_088018	5425	coding	coding	coding	coding
MSTRG.87965.1	TJAP1	XLOC_088023	233	coding	noncoding	noncoding	noncoding
MSTRG.88034.1	MAD2L1BP	XLOC_088028	254	coding	noncoding	noncoding	noncoding
NR_160955.1	C6orf223	XLOC_088040	5293	coding	coding	coding	coding
MSTRG.88064.1		XLOC_088060	266	coding	noncoding	noncoding	noncoding
NM_001271970.1	HSP90AB1	XLOC_088062	2644	coding	coding	coding	coding
NM_001137560.2	TMEM151B	XLOC_088064	4911	coding	coding	coding	coding
MSTRG.88087.1	CDC5L	XLOC_088078	248	coding	noncoding	noncoding	noncoding
MSTRG.88790.1	RUNX2	XLOC_088096	321	coding	noncoding	noncoding	noncoding
MSTRG.88807.1	RUNX2	XLOC_088111	265	coding	noncoding	noncoding	noncoding
MSTRG.88208.1	CYP39A1	XLOC_088185	252	coding	noncoding	noncoding	noncoding
MSTRG.88223.1		XLOC_088217	272	coding	noncoding	noncoding	noncoding
MSTRG.88316.1		XLOC_088266	268	coding	noncoding	noncoding	noncoding
MSTRG.88319.1		XLOC_088268	418	coding	noncoding	noncoding	noncoding
MSTRG.88359.1	CRISP1	XLOC_088290	252	coding	noncoding	noncoding	noncoding
MSTRG.88381.1		XLOC_088303	218	coding	noncoding	noncoding	noncoding
XM_017011233.1	TFAP2B	XLOC_088309	6457	coding	coding	coding	coding
MSTRG.88446.1	PKHD1	XLOC_088329	286	coding	noncoding	noncoding	noncoding
MSTRG.88427.1	LOC102724327	XLOC_088342	345	coding	noncoding	noncoding	noncoding
MSTRG.88469.1		XLOC_088365	263	coding	noncoding	noncoding	noncoding
MSTRG.88481.1		XLOC_088367	373	coding	noncoding	noncoding	noncoding
MSTRG.88541.1		XLOC_088400	265	coding	noncoding	noncoding	noncoding
MSTRG.88557.1		XLOC_088410	266	coding	noncoding	noncoding	noncoding
MSTRG.88555.1		XLOC_088411	296	coding	noncoding	noncoding	noncoding
NR_125842.1	LOC101927189	XLOC_088417	3168	coding	noncoding	noncoding	noncoding
MSTRG.88665.1	LOC105375097	XLOC_088428	260	coding	noncoding	noncoding	noncoding
MSTRG.88675.1	LOC107986606	XLOC_088442	413	coding	noncoding	noncoding	noncoding
NM_001010872.3	FAM83B	XLOC_088445	6244	coding	coding	coding	coding
MSTRG.88682.1		XLOC_088446	254	coding	noncoding	noncoding	noncoding
MSTRG.88689.1		XLOC_088449	284	coding	noncoding	noncoding	noncoding
MSTRG.88699.1	HCRTR2	XLOC_088455	290	coding	noncoding	noncoding	noncoding
MSTRG.88700.1	HCRTR2	XLOC_088456	290	coding	noncoding	noncoding	noncoding
MSTRG.88703.1	HCRTR2	XLOC_088458	338	coding	noncoding	noncoding	noncoding
MSTRG.88697.1		XLOC_088460	262	coding	noncoding	noncoding	noncoding
MSTRG.89010.1	DST	XLOC_088526	257	coding	noncoding	noncoding	noncoding
NM_001318539.1	BEND6	XLOC_088530	3650	coding	noncoding	coding	noncoding
MSTRG.89020.1	KIAA1586	XLOC_088531	491	coding	noncoding	coding	coding
MSTRG.88869.1		XLOC_088563	320	coding	noncoding	noncoding	noncoding
MSTRG.88942.1		XLOC_088602	320	coding	noncoding	noncoding	noncoding

MSTRG.88946.1		XLOC_088604	309	coding	noncoding	noncoding	noncoding
MSTRG.88953.1		XLOC_088606	337	coding	noncoding	noncoding	noncoding
MSTRG.89055.1		XLOC_088658	243	coding	noncoding	noncoding	noncoding
MSTRG.89066.1		XLOC_088667	296	coding	coding	noncoding	noncoding
MSTRG.89071.1		XLOC_088669	293	coding	noncoding	noncoding	noncoding
XM_011536112.1	PTP4A1	XLOC_088687	4976	coding	coding	coding	coding
MSTRG.89382.1	EYS	XLOC_088735	279	coding	noncoding	noncoding	noncoding
MSTRG.89399.1	EYS	XLOC_088752	304	coding	noncoding	noncoding	noncoding
MSTRG.89413.1	EYS	XLOC_088764	242	coding	noncoding	noncoding	noncoding
MSTRG.89111.1		XLOC_088771	232	coding	noncoding	noncoding	noncoding
MSTRG.89213.1		XLOC_088788	295	coding	coding	noncoding	noncoding
MSTRG.89226.1		XLOC_088792	233	coding	noncoding	noncoding	noncoding
MSTRG.89227.1		XLOC_088794	314	coding	noncoding	noncoding	noncoding
MSTRG.89229.1	LOC102723883	XLOC_088797	296	coding	noncoding	noncoding	noncoding
MSTRG.89232.1		XLOC_088800	203	coding	noncoding	noncoding	noncoding
MSTRG.89233.1		XLOC_088801	296	coding	noncoding	noncoding	noncoding
MSTRG.89289.1	ADGRB3	XLOC_088817	291	coding	noncoding	noncoding	noncoding
MSTRG.89297.1	ADGRB3	XLOC_088821	318	coding	noncoding	noncoding	noncoding
MSTRG.89244.1		XLOC_088825	273	coding	noncoding	noncoding	noncoding
MSTRG.89247.1		XLOC_088826	282	coding	noncoding	noncoding	noncoding
MSTRG.89459.1	COL19A1	XLOC_088850	270	coding	noncoding	noncoding	noncoding
MSTRG.89329.1		XLOC_088868	267	coding	noncoding	coding	noncoding
MSTRG.89330.1		XLOC_088869	294	coding	noncoding	noncoding	noncoding
MSTRG.89525.1	SMAP1	XLOC_088872	245	coding	noncoding	noncoding	noncoding
MSTRG.89528.1	SMAP1	XLOC_088874	255	coding	noncoding	noncoding	noncoding
MSTRG.89506.1	LINC01626	XLOC_088903	241	coding	noncoding	noncoding	noncoding
MSTRG.89508.1		XLOC_088906	324	coding	noncoding	noncoding	noncoding
NM_001350413.1	RIMS1	XLOC_088911	2576	coding	noncoding	coding	coding
MSTRG.89566.1	RIMS1	XLOC_088917	216	coding	noncoding	noncoding	noncoding
MSTRG.89552.1		XLOC_088924	244	coding	noncoding	noncoding	noncoding
MSTRG.90286.1	KCNQ5	XLOC_088962	237	coding	noncoding	noncoding	noncoding
MSTRG.90311.1	KCNQ5	XLOC_088967	300	coding	noncoding	noncoding	noncoding
MSTRG.90313.1	KCNQ5	XLOC_088968	247	coding	noncoding	noncoding	noncoding
MSTRG.89588.1	KHDC1	XLOC_088973	245	coding	noncoding	noncoding	noncoding
MSTRG.89576.1		XLOC_088976	279	coding	noncoding	noncoding	noncoding
MSTRG.89641.1	CD109	XLOC_088992	204	coding	noncoding	noncoding	noncoding
MSTRG.89643.1	CD109	XLOC_088994	234	coding	noncoding	noncoding	noncoding
MSTRG.89622.1		XLOC_088999	232	coding	noncoding	noncoding	noncoding
MSTRG.89628.1		XLOC_089004	276	coding	coding	noncoding	noncoding
MSTRG.89673.1	LOC105377858	XLOC_089015	264	coding	noncoding	noncoding	noncoding
MSTRG.89680.1	LOC105377858	XLOC_089019	209	coding	noncoding	noncoding	noncoding
MSTRG.89682.1		XLOC_089023	278	coding	noncoding	noncoding	noncoding
MSTRG.89718.1	FILIP1	XLOC_089032	259	coding	noncoding	noncoding	noncoding

MSTRG.89805.1	SENP6	XLOC_089064	288 coding	coding	noncoding	noncoding
MSTRG.89806.1	SENP6	XLOC_089065	253 coding	noncoding	noncoding	noncoding
MSTRG.89746.1		XLOC_089080	347 coding	coding	noncoding	noncoding
MSTRG.89813.1		XLOC_089123	324 coding	coding	noncoding	noncoding
MSTRG.89826.1	MEI4	XLOC_089131	264 coding	coding	noncoding	noncoding
MSTRG.89902.1		XLOC_089153	273 coding	noncoding	noncoding	noncoding
MSTRG.89910.1		XLOC_089160	307 coding	noncoding	noncoding	noncoding
MSTRG.89937.1		XLOC_089172	326 coding	noncoding	noncoding	noncoding
MSTRG.89938.1		XLOC_089173	305 coding	noncoding	noncoding	noncoding
MSTRG.90108.1	BCKDHB	XLOC_089189	213 coding	noncoding	noncoding	noncoding
MSTRG.90027.1		XLOC_089214	311 coding	noncoding	noncoding	noncoding
MSTRG.90045.1		XLOC_089223	248 coding	noncoding	noncoding	noncoding
MSTRG.90046.1		XLOC_089224	273 coding	noncoding	noncoding	noncoding
MSTRG.90094.1	LINC02542	XLOC_089288	289 coding	noncoding	noncoding	noncoding
MSTRG.90096.1	LINC02542	XLOC_089290	340 coding	noncoding	noncoding	noncoding
MSTRG.90087.1		XLOC_089295	272 coding	noncoding	noncoding	noncoding
MSTRG.90175.1		XLOC_089304	498 coding	coding	noncoding	noncoding
MSTRG.90221.1	LOC105377876	XLOC_089306	276 coding	coding	noncoding	noncoding
MSTRG.90393.1	ME1	XLOC_089384	286 coding	noncoding	noncoding	noncoding
MSTRG.90371.1		XLOC_089394	644 coding	noncoding	noncoding	noncoding
MSTRG.90375.1		XLOC_089396	283 coding	noncoding	noncoding	noncoding
MSTRG.90377.1		XLOC_089397	310 coding	noncoding	noncoding	noncoding
MSTRG.90378.1		XLOC_089398	237 coding	noncoding	noncoding	noncoding
MSTRG.90414.1	SNAP91	XLOC_089401	312 coding	noncoding	noncoding	noncoding
MSTRG.90406.1	LOC105377879	XLOC_089406	337 coding	noncoding	noncoding	noncoding
MSTRG.90469.1	CYB5R4	XLOC_089417	302 coding	noncoding	noncoding	noncoding
XM_017010220.1	MRAP2	XLOC_089448	2781 coding	coding	coding	coding
MSTRG.90510.1		XLOC_089470	297 coding	coding	noncoding	noncoding
MSTRG.90557.1	LOC107986622	XLOC_089493	261 coding	noncoding	noncoding	noncoding
MSTRG.90561.1	LOC107986622	XLOC_089494	271 coding	noncoding	noncoding	noncoding
MSTRG.90567.1		XLOC_089498	290 coding	noncoding	noncoding	noncoding
MSTRG.90575.1	LOC101928842	XLOC_089500	238 coding	noncoding	noncoding	noncoding
MSTRG.90569.1		XLOC_089501	258 coding	noncoding	noncoding	noncoding
MSTRG.90570.1		XLOC_089502	235 coding	noncoding	noncoding	noncoding
MSTRG.90580.1		XLOC_089506	296 coding	noncoding	noncoding	noncoding
MSTRG.90585.1		XLOC_089509	286 coding	noncoding	noncoding	noncoding
MSTRG.90589.1		XLOC_089513	297 coding	noncoding	noncoding	noncoding
MSTRG.90593.1		XLOC_089515	235 coding	noncoding	noncoding	noncoding
MSTRG.90595.1		XLOC_089517	324 coding	noncoding	noncoding	noncoding
MSTRG.90604.1	HTR1E	XLOC_089522	462 coding	noncoding	noncoding	noncoding
XM_011535625.2	ZNF292	XLOC_089527	10404 coding	coding	coding	coding
MSTRG.90791.1	ORC3	XLOC_089565	301 coding	noncoding	noncoding	noncoding
MSTRG.90651.1	LOC101928911	XLOC_089577	397 coding	noncoding	noncoding	noncoding

MSTRG.90749.1		XLOC_089607	278 coding	noncoding	noncoding	noncoding
MSTRG.90822.1	PM20D2	XLOC_089620	241 coding	noncoding	noncoding	noncoding
MSTRG.90982.1	ANKRD6	XLOC_089652	311 coding	noncoding	noncoding	noncoding
MSTRG.90987.1	ANKRD6	XLOC_089653	219 coding	noncoding	noncoding	noncoding
MSTRG.90988.1	ANKRD6	XLOC_089654	243 coding	noncoding	noncoding	noncoding
MSTRG.91038.1		XLOC_089706	293 coding	noncoding	noncoding	noncoding
MSTRG.91039.1		XLOC_089713	298 coding	noncoding	noncoding	noncoding
MSTRG.91142.1		XLOC_089779	358 coding	noncoding	noncoding	noncoding
MSTRG.91230.1		XLOC_089838	224 coding	noncoding	noncoding	noncoding
MSTRG.91234.1		XLOC_089840	292 coding	noncoding	noncoding	noncoding
MSTRG.91246.1		XLOC_089845	219 coding	noncoding	noncoding	noncoding
MSTRG.91266.1		XLOC_089860	292 coding	noncoding	noncoding	noncoding
MSTRG.91294.1	FUT9	XLOC_089871	243 coding	noncoding	noncoding	noncoding
MSTRG.91298.1		XLOC_089895	282 coding	coding	noncoding	noncoding
MSTRG.91451.1	KLHL32	XLOC_089908	285 coding	noncoding	noncoding	noncoding
MSTRG.91340.1		XLOC_089912	236 coding	noncoding	noncoding	noncoding
MSTRG.91360.1	LOC101927314	XLOC_089917	245 coding	noncoding	noncoding	noncoding
MSTRG.91365.1	LOC101927314	XLOC_089919	310 coding	coding	noncoding	noncoding
MSTRG.91378.1		XLOC_089933	258 coding	noncoding	noncoding	noncoding
MSTRG.91383.1		XLOC_089936	251 coding	noncoding	noncoding	noncoding
MSTRG.91388.1		XLOC_089941	249 coding	noncoding	noncoding	noncoding
XR_001743062.2	TSTD3	XLOC_089966	1487 coding	coding	noncoding	noncoding
MSTRG.91519.1	SIM1	XLOC_089990	308 coding	noncoding	noncoding	noncoding
MSTRG.91767.5	ASCC3	XLOC_089997	1388 coding	coding	coding	noncoding
MSTRG.91823.1	LOC107984041	XLOC_090006	830 coding	noncoding	noncoding	noncoding
MSTRG.91648.1		XLOC_090030	232 coding	noncoding	noncoding	noncoding
MSTRG.91664.1		XLOC_090035	285 coding	noncoding	noncoding	noncoding
MSTRG.91668.1		XLOC_090039	275 coding	coding	noncoding	noncoding
MSTRG.91669.1		XLOC_090040	289 coding	noncoding	noncoding	noncoding
MSTRG.91688.1		XLOC_090051	228 coding	noncoding	noncoding	noncoding
MSTRG.91689.1		XLOC_090052	284 coding	noncoding	noncoding	noncoding
MSTRG.91746.1		XLOC_090075	269 coding	coding	noncoding	noncoding
NR_037157.1	BVES-AS1	XLOC_090080	1716 coding	noncoding	noncoding	noncoding
MSTRG.91760.1	BVES-AS1	XLOC_090081	278 coding	noncoding	noncoding	noncoding
MSTRG.91946.1	PRDM1	XLOC_090129	304 coding	noncoding	noncoding	noncoding
MSTRG.92016.1	CRYBG1	XLOC_090146	608 coding	noncoding	noncoding	noncoding
MSTRG.92024.1	CRYBG1	XLOC_090151	301 coding	noncoding	noncoding	noncoding
MSTRG.91985.1		XLOC_090186	245 coding	noncoding	noncoding	noncoding
MSTRG.91996.1		XLOC_090188	268 coding	noncoding	noncoding	noncoding
MSTRG.92000.1		XLOC_090191	251 coding	noncoding	noncoding	noncoding
MSTRG.92001.1		XLOC_090192	298 coding	noncoding	noncoding	noncoding
MSTRG.92006.1	C6orf203	XLOC_090194	269 coding	noncoding	noncoding	noncoding
MSTRG.92069.1		XLOC_090218	201 coding	noncoding	noncoding	noncoding

MSTRG.92070.1		XLOC_090219	259 coding	noncoding	noncoding	noncoding
MSTRG.92083.1		XLOC_090230	256 coding	noncoding	noncoding	noncoding
MSTRG.92172.1	LOC105377932	XLOC_090244	302 coding	noncoding	noncoding	noncoding
MSTRG.92187.1	AFG1L	XLOC_090249	321 coding	noncoding	noncoding	noncoding
MSTRG.92190.1	AFG1L	XLOC_090250	294 coding	coding	noncoding	noncoding
MSTRG.92202.1		XLOC_090266	249 coding	noncoding	noncoding	noncoding
MSTRG.92297.1		XLOC_090271	290 coding	noncoding	noncoding	noncoding
MSTRG.92300.1	ARMC2	XLOC_090273	247 coding	noncoding	noncoding	noncoding
MSTRG.92303.1	ARMC2	XLOC_090276	248 coding	noncoding	noncoding	noncoding
MSTRG.92307.1	ARMC2	XLOC_090277	235 coding	noncoding	noncoding	noncoding
MSTRG.92317.1	ARMC2	XLOC_090281	229 coding	noncoding	noncoding	noncoding
NM_001083535.2	CEP57L1	XLOC_090282	6647 coding	coding	coding	coding
XM_011536281.3	FIG4	XLOC_090321	3638 coding	coding	coding	coding
MSTRG.92259.1		XLOC_090352	222 coding	coding	noncoding	noncoding
NM_001287216.1	AMD1	XLOC_090408	3064 coding	coding	coding	coding
MSTRG.92430.1		XLOC_090416	230 coding	noncoding	noncoding	noncoding
MSTRG.92863.1	SLC16A10	XLOC_090425	268 coding	noncoding	noncoding	noncoding
MSTRG.92750.17	LOC105377945	XLOC_090439	8231 coding	coding	coding	noncoding
MSTRG.92750.32	LOC105377945	XLOC_090439	2032 coding	coding	coding	noncoding
MSTRG.92548.1	LOC105377947	XLOC_090444	473 coding	noncoding	noncoding	noncoding
MSTRG.92579.1	LAMA4	XLOC_090453	523 coding	noncoding	noncoding	noncoding
MSTRG.92563.1		XLOC_090457	338 coding	coding	coding	noncoding
MSTRG.92590.1		XLOC_090461	456 coding	noncoding	noncoding	noncoding
MSTRG.92615.1		XLOC_090476	293 coding	noncoding	noncoding	noncoding
MSTRG.92620.1		XLOC_090480	216 coding	noncoding	noncoding	noncoding
MSTRG.92628.1		XLOC_090484	264 coding	noncoding	noncoding	noncoding
MSTRG.92630.1		XLOC_090486	274 coding	noncoding	noncoding	noncoding
MSTRG.92733.1		XLOC_090540	464 coding	noncoding	noncoding	noncoding
NM_001322940.2	DSE	XLOC_090580	11177 coding	coding	coding	coding
MSTRG.93059.1	DSE	XLOC_090590	300 coding	noncoding	noncoding	noncoding
NM_173560.4	RFX6	XLOC_090620	3476 coding	coding	coding	coding
MSTRG.92826.1	RFX6	XLOC_090621	314 coding	noncoding	noncoding	noncoding
MSTRG.92952.1	NUS1	XLOC_090639	407 coding	noncoding	noncoding	noncoding
MSTRG.92944.1		XLOC_090643	317 coding	noncoding	noncoding	noncoding
MSTRG.93071.1		XLOC_090663	281 coding	noncoding	noncoding	noncoding
MSTRG.93144.1	FAM184A	XLOC_090734	278 coding	noncoding	noncoding	noncoding
MSTRG.93204.1		XLOC_090764	303 coding	noncoding	noncoding	noncoding
MSTRG.93262.1		XLOC_090777	268 coding	noncoding	noncoding	noncoding
MSTRG.93263.1		XLOC_090778	263 coding	noncoding	noncoding	noncoding
MSTRG.93426.3	TBC1D32	XLOC_090796	891 coding	coding	noncoding	noncoding
NM_000165.5	GJA1	XLOC_090799	3083 coding	coding	coding	coding
MSTRG.93288.1		XLOC_090801	229 coding	noncoding	noncoding	noncoding
MSTRG.93296.1		XLOC_090803	200 coding	noncoding	noncoding	noncoding

MSTRG.93295.1		XLOC_090804	268 coding	noncoding	noncoding	noncoding
MSTRG.93301.1		XLOC_090806	661 coding	noncoding	noncoding	noncoding
MSTRG.93328.1	LOC105377979	XLOC_090813	358 coding	noncoding	noncoding	noncoding
MSTRG.93305.1		XLOC_090816	394 coding	noncoding	noncoding	noncoding
MSTRG.93306.1		XLOC_090817	255 coding	noncoding	noncoding	noncoding
MSTRG.93311.1		XLOC_090819	293 coding	noncoding	noncoding	noncoding
MSTRG.93334.1		XLOC_090835	285 coding	noncoding	noncoding	noncoding
MSTRG.93342.1		XLOC_090839	261 coding	noncoding	noncoding	noncoding
MSTRG.93498.1	NKAIN2	XLOC_090856	257 coding	noncoding	noncoding	noncoding
MSTRG.93535.1	NKAIN2	XLOC_090869	227 coding	noncoding	noncoding	noncoding
NR_136734.1	RNF217	XLOC_090881	11447 coding	coding	coding	coding
MSTRG.93570.1		XLOC_090908	276 coding	noncoding	noncoding	noncoding
XM_024446331.1	NCOA7	XLOC_090917	5559 coding	coding	coding	coding
MSTRG.93637.1	NCOA7	XLOC_090921	228 coding	noncoding	noncoding	noncoding
MSTRG.93674.1		XLOC_090956	281 coding	noncoding	noncoding	noncoding
MSTRG.93696.1		XLOC_090963	283 coding	noncoding	noncoding	noncoding
MSTRG.93701.1		XLOC_090964	423 coding	coding	noncoding	noncoding
NM_001242850.1	RNF146	XLOC_090969	2145 coding	coding	coding	coding
MSTRG.93906.1	PTPRK	XLOC_091033	260 coding	noncoding	noncoding	noncoding
XM_017010852.1	LAMA2	XLOC_091040	11234 coding	coding	coding	coding
MSTRG.93944.1	LAMA2	XLOC_091049	286 coding	noncoding	noncoding	noncoding
MSTRG.93952.1	LAMA2	XLOC_091051	396 coding	noncoding	noncoding	noncoding
MSTRG.93909.1		XLOC_091083	281 coding	noncoding	noncoding	noncoding
XR_002956397.1	LOC112267974	XLOC_091096	3671 coding	noncoding	noncoding	noncoding
MSTRG.94008.1		XLOC_091102	297 coding	noncoding	noncoding	noncoding
MSTRG.94017.1		XLOC_091108	509 coding	noncoding	noncoding	noncoding
MSTRG.94022.1		XLOC_091111	200 coding	noncoding	noncoding	noncoding
MSTRG.94055.1	LOC102723445	XLOC_091122	296 coding	noncoding	coding	noncoding
MSTRG.94242.2	MED23	XLOC_091217	5371 coding	coding	coding	coding
MSTRG.94109.1		XLOC_091235	248 coding	noncoding	noncoding	noncoding
MSTRG.94110.1		XLOC_091237	228 coding	noncoding	noncoding	noncoding
MSTRG.94305.4	SLC18B1	XLOC_091253	2440 coding	coding	coding	noncoding
MSTRG.94301.1		XLOC_091258	295 coding	coding	noncoding	noncoding
MSTRG.94319.1		XLOC_091266	325 coding	noncoding	noncoding	noncoding
MSTRG.94374.1		XLOC_091289	247 coding	noncoding	noncoding	noncoding
MSTRG.94443.9	SGK1	XLOC_091291	3101 coding	noncoding	noncoding	coding
MSTRG.94413.1	CT69	XLOC_091312	274 coding	noncoding	noncoding	noncoding
MSTRG.94425.1	LOC101928277	XLOC_091319	521 coding	noncoding	noncoding	noncoding
MSTRG.94430.1	LOC101928277	XLOC_091322	299 coding	noncoding	noncoding	noncoding
MSTRG.94463.1		XLOC_091345	261 coding	noncoding	noncoding	noncoding
MSTRG.94597.1	PDE7B	XLOC_091380	295 coding	noncoding	noncoding	noncoding
MSTRG.94604.1	MTFR2	XLOC_091383	405 coding	coding	noncoding	noncoding
MSTRG.94606.17	BCLAF1	XLOC_091384	4052 coding	coding	noncoding	noncoding

MSTRG.94606.21	BCLAF1	XLOC_091384	5218	coding	coding	noncoding	noncoding
MSTRG.94541.1		XLOC_091385	281	coding	noncoding	noncoding	noncoding
MSTRG.94548.1		XLOC_091389	231	coding	noncoding	noncoding	noncoding
MSTRG.94784.5	MAP3K5	XLOC_091403	4427	coding	coding	coding	coding
MSTRG.94625.1		XLOC_091412	603	coding	noncoding	noncoding	noncoding
MSTRG.94637.1	IL22RA2	XLOC_091415	402	coding	noncoding	noncoding	noncoding
MSTRG.94662.1		XLOC_091417	255	coding	noncoding	noncoding	noncoding
MSTRG.94678.1	LOC102723633	XLOC_091425	277	coding	noncoding	noncoding	noncoding
MSTRG.94683.1		XLOC_091428	302	coding	noncoding	noncoding	noncoding
MSTRG.94686.1		XLOC_091429	368	coding	noncoding	noncoding	noncoding
MSTRG.94689.1		XLOC_091431	225	coding	noncoding	noncoding	noncoding
MSTRG.94708.1		XLOC_091462	265	coding	noncoding	noncoding	noncoding
MSTRG.94710.1		XLOC_091464	224	coding	noncoding	noncoding	noncoding
MSTRG.94753.1		XLOC_091476	314	coding	noncoding	noncoding	noncoding
MSTRG.94850.1	NHSL1	XLOC_091495	271	coding	noncoding	noncoding	noncoding
MSTRG.94900.2	REPS1	XLOC_091521	2726	coding	coding	noncoding	coding
MSTRG.94921.1		XLOC_091550	280	coding	noncoding	noncoding	noncoding
MSTRG.94973.1	FILNC1	XLOC_091556	209	coding	noncoding	noncoding	noncoding
MSTRG.95022.1	LOC105378027	XLOC_091577	272	coding	noncoding	noncoding	noncoding
MSTRG.95003.1		XLOC_091579	270	coding	noncoding	noncoding	noncoding
MSTRG.95056.1		XLOC_091604	303	coding	noncoding	noncoding	noncoding
MSTRG.95083.1	LOC105378031	XLOC_091620	304	coding	noncoding	noncoding	noncoding
MSTRG.95076.1		XLOC_091631	267	coding	noncoding	noncoding	noncoding
MSTRG.95116.1	LOC153910	XLOC_091645	282	coding	noncoding	noncoding	noncoding
MSTRG.95171.14	HIVEP2	XLOC_091651	4309	coding	coding	coding	coding
MSTRG.95171.19	HIVEP2	XLOC_091651	5533	coding	noncoding	coding	noncoding
MSTRG.95167.1	AIG1	XLOC_091674	287	coding	noncoding	noncoding	noncoding
MSTRG.95284.1	ZC2HC1B	XLOC_091718	256	coding	noncoding	noncoding	noncoding
MSTRG.95293.1		XLOC_091726	233	coding	noncoding	noncoding	noncoding
MSTRG.95463.1		XLOC_091740	301	coding	noncoding	noncoding	noncoding
MSTRG.95464.1		XLOC_091741	241	coding	noncoding	noncoding	noncoding
XM_024446536.1	UTRN	XLOC_091742	12372	coding	coding	noncoding	coding
MSTRG.95350.1		XLOC_091827	315	coding	noncoding	noncoding	noncoding
MSTRG.95363.1		XLOC_091832	231	coding	noncoding	noncoding	noncoding
MSTRG.95366.1		XLOC_091833	212	coding	coding	noncoding	noncoding
MSTRG.95367.1	TRQ-TTG4-1	XLOC_091835	222	coding	noncoding	noncoding	noncoding
MSTRG.95585.3	EPM2A	XLOC_091840	383	coding	noncoding	noncoding	noncoding
NM_001278065.2	GRM1	XLOC_091851	6917	coding	coding	coding	coding
MSTRG.95396.1		XLOC_091860	236	coding	noncoding	noncoding	noncoding
MSTRG.95399.1		XLOC_091861	268	coding	noncoding	noncoding	noncoding
NM_024694.4	ADGB	XLOC_091862	5318	coding	coding	coding	coding
MSTRG.95418.1	ADGB	XLOC_091870	236	coding	noncoding	noncoding	noncoding
MSTRG.95425.1		XLOC_091925	300	coding	noncoding	noncoding	noncoding

MSTRG.95447.1		XLOC_091935	225 coding	noncoding	noncoding	noncoding
XM_024446385.1	SASH1	XLOC_091936	8682 coding	coding	coding	coding
MSTRG.95562.1	SASH1	XLOC_091939	485 coding	noncoding	noncoding	noncoding
XR_001743088.2	UST	XLOC_091949	1694 coding	coding	coding	coding
MSTRG.95759.1		XLOC_092031	225 coding	noncoding	noncoding	noncoding
MSTRG.95763.9	PCMT1	XLOC_092034	465 coding	coding	coding	noncoding
MSTRG.95765.1	PCMT1	XLOC_092035	347 coding	noncoding	noncoding	noncoding
MSTRG.95735.1	RAET1E	XLOC_092042	266 coding	noncoding	noncoding	noncoding
MSTRG.95736.1	RAET1E	XLOC_092043	287 coding	noncoding	noncoding	noncoding
MSTRG.95739.1	RAET1G	XLOC_092045	247 coding	noncoding	noncoding	noncoding
MSTRG.95740.1	RAET1G	XLOC_092046	284 coding	noncoding	noncoding	noncoding
MSTRG.95722.1		XLOC_092047	290 coding	noncoding	noncoding	noncoding
MSTRG.95730.1		XLOC_092056	245 coding	noncoding	noncoding	noncoding
MSTRG.95797.1	PPP1R14C	XLOC_092063	291 coding	noncoding	noncoding	noncoding
NM_001164694.2	IYD	XLOC_092066	8734 coding	coding	coding	coding
MSTRG.95775.1		XLOC_092069	267 coding	noncoding	noncoding	noncoding
MSTRG.95779.1		XLOC_092073	276 coding	noncoding	noncoding	noncoding
NM_001329801.2	PLEKHG1	XLOC_092074	7127 coding	coding	coding	coding
MSTRG.95858.1	MTHFD1L	XLOC_092081	274 coding	noncoding	noncoding	noncoding
MSTRG.95814.1		XLOC_092083	263 coding	noncoding	noncoding	noncoding
XM_017011517.2	AKAP12	XLOC_092085	8562 coding	coding	coding	coding
MSTRG.95887.1	AKAP12	XLOC_092088	267 coding	noncoding	noncoding	noncoding
MSTRG.96409.39	SYNE1-AS1	XLOC_092103	17075 coding	coding	coding	coding
MSTRG.96439.1	ESR1	XLOC_092107	262 coding	noncoding	noncoding	noncoding
MSTRG.96037.1		XLOC_092124	284 coding	noncoding	noncoding	noncoding
MSTRG.96109.1	RGS17	XLOC_092139	280 coding	noncoding	noncoding	noncoding
MSTRG.96110.1	RGS17	XLOC_092140	211 coding	noncoding	noncoding	noncoding
MSTRG.96071.1	LOC105378066	XLOC_092152	208 coding	noncoding	noncoding	noncoding
MSTRG.96073.1		XLOC_092156	251 coding	noncoding	noncoding	noncoding
MSTRG.96121.1		XLOC_092175	287 coding	noncoding	noncoding	noncoding
MSTRG.96136.1		XLOC_092179	237 coding	noncoding	noncoding	noncoding
NM_001286189.1	SCAF8	XLOC_092184	4908 coding	coding	coding	coding
MSTRG.96269.1		XLOC_092213	230 coding	noncoding	noncoding	noncoding
MSTRG.96177.1		XLOC_092217	277 coding	noncoding	noncoding	noncoding
MSTRG.96187.1	LOC105378068	XLOC_092220	293 coding	noncoding	noncoding	noncoding
MSTRG.96199.1		XLOC_092428	212 coding	noncoding	noncoding	noncoding
MSTRG.96209.1		XLOC_092433	279 coding	noncoding	noncoding	noncoding
MSTRG.96219.1		XLOC_092437	307 coding	noncoding	noncoding	noncoding
MSTRG.96301.1		XLOC_092531	276 coding	noncoding	noncoding	noncoding
MSTRG.96319.1	SNX9	XLOC_092539	252 coding	coding	noncoding	noncoding
MSTRG.96686.24	EZR-AS1	XLOC_092584	3190 coding	coding	coding	coding
MSTRG.96562.1		XLOC_092590	317 coding	noncoding	noncoding	noncoding
NM_032532.3	FNDC1	XLOC_092625	6548 coding	coding	coding	coding

MSTRG.96666.1	FNDC1	XLOC_092628	280 coding	noncoding	noncoding	noncoding
MSTRG.96654.1		XLOC_092631	247 coding	noncoding	noncoding	noncoding
XM_024446597.1	WTAP	XLOC_092685	1942 coding	coding	coding	coding
MSTRG.97092.1	SLC22A2	XLOC_092743	228 coding	noncoding	noncoding	noncoding
MSTRG.97134.1		XLOC_092761	236 coding	noncoding	noncoding	noncoding
MSTRG.97112.1		XLOC_092763	215 coding	noncoding	noncoding	noncoding
NM_001291958.1	MAP3K4	XLOC_092767	5645 coding	coding	coding	coding
MSTRG.97209.1	MAP3K4	XLOC_092773	361 coding	noncoding	noncoding	noncoding
XM_006715344.4	PACRG	XLOC_092837	7362 coding	coding	coding	coding
MSTRG.97784.1	PRKN	XLOC_092851	269 coding	noncoding	noncoding	noncoding
MSTRG.97960.1	PACRG-AS1	XLOC_092980	264 coding	noncoding	noncoding	noncoding
MSTRG.97298.1	LOC107986667	XLOC_093076	293 coding	noncoding	noncoding	noncoding
MSTRG.97309.1		XLOC_093080	276 coding	noncoding	noncoding	noncoding
MSTRG.97324.1		XLOC_093087	306 coding	noncoding	noncoding	noncoding
MSTRG.97461.1		XLOC_093144	289 coding	noncoding	noncoding	noncoding
MSTRG.97476.1		XLOC_093147	733 coding	coding	noncoding	noncoding
XM_011535907.2	UNC93A	XLOC_093148	2741 coding	coding	coding	coding
MSTRG.97484.1		XLOC_093154	259 coding	noncoding	noncoding	noncoding
MSTRG.97520.1	KIF25	XLOC_093187	237 coding	noncoding	noncoding	noncoding
MSTRG.97576.1		XLOC_093227	248 coding	noncoding	noncoding	noncoding
NM_001286380.1	FAM120B	XLOC_093300	5207 coding	noncoding	coding	coding
MSTRG.83729.1	LOC102723922	XLOC_093324	225 coding	noncoding	noncoding	noncoding
MSTRG.83747.1	LOC105374873	XLOC_093361	256 coding	noncoding	noncoding	noncoding
MSTRG.83776.1		XLOC_093377	286 coding	noncoding	noncoding	noncoding
XR_926373.2	LOC105374880	XLOC_093381	2137 coding	noncoding	coding	noncoding
MSTRG.83787.1	LOC102723944	XLOC_093384	240 coding	noncoding	noncoding	noncoding
MSTRG.84424.1	GMDS	XLOC_093423	292 coding	noncoding	noncoding	noncoding
MSTRG.83829.1		XLOC_093464	251 coding	noncoding	noncoding	noncoding
MSTRG.83838.13	MYLK4	XLOC_093467	2436 coding	coding	noncoding	noncoding
MSTRG.83871.1		XLOC_093495	218 coding	noncoding	noncoding	noncoding
MSTRG.83923.1		XLOC_093514	254 coding	noncoding	noncoding	noncoding
MSTRG.83928.1		XLOC_093520	274 coding	noncoding	noncoding	noncoding
MSTRG.83938.1	LOC100507336	XLOC_093525	255 coding	noncoding	noncoding	noncoding
MSTRG.83939.1	LOC100507336	XLOC_093526	288 coding	noncoding	noncoding	noncoding
MSTRG.83948.1		XLOC_093532	318 coding	noncoding	noncoding	noncoding
MSTRG.84025.1		XLOC_093565	292 coding	noncoding	noncoding	noncoding
MSTRG.84067.3	CDYL	XLOC_093572	1637 coding	coding	coding	coding
MSTRG.84145.1	F13A1	XLOC_093631	3891 coding	coding	coding	coding
MSTRG.84145.3	F13A1	XLOC_093631	841 coding	coding	coding	noncoding
MSTRG.84189.1		XLOC_093657	242 coding	noncoding	noncoding	noncoding
MSTRG.84193.1	LOC105374903	XLOC_093659	270 coding	noncoding	noncoding	noncoding
MSTRG.84203.1		XLOC_093667	266 coding	noncoding	noncoding	noncoding
MSTRG.84270.1		XLOC_093686	295 coding	noncoding	noncoding	noncoding

MSTRG.84297.1	DSP	XLOC_093691	209	coding	noncoding	noncoding	noncoding
MSTRG.84313.1		XLOC_093698	278	coding	noncoding	noncoding	noncoding
MSTRG.84378.1	LOC105374912	XLOC_093705	280	coding	noncoding	noncoding	noncoding
MSTRG.84515.1		XLOC_093726	288	coding	noncoding	noncoding	noncoding
MSTRG.84531.1		XLOC_093732	256	coding	noncoding	noncoding	noncoding
MSTRG.84537.1		XLOC_093736	280	coding	noncoding	noncoding	noncoding
MSTRG.84539.1		XLOC_093737	266	coding	noncoding	noncoding	noncoding
MSTRG.84566.1	LOC112267856	XLOC_093749	273	coding	noncoding	noncoding	noncoding
MSTRG.84615.1	OFCC1	XLOC_093753	310	coding	noncoding	noncoding	noncoding
MSTRG.84623.1	LOC105374918	XLOC_093757	245	coding	noncoding	noncoding	noncoding
MSTRG.84567.1		XLOC_093760	423	coding	noncoding	coding	noncoding
NR_027793.1	LINC00518	XLOC_093768	3018	coding	noncoding	noncoding	coding
NM_004752.3	GCM2	XLOC_093788	2365	coding	coding	coding	coding
MSTRG.84847.1	LOC105374925	XLOC_093853	749	coding	noncoding	noncoding	noncoding
MSTRG.84719.1	LOC105374928	XLOC_093875	286	coding	noncoding	noncoding	noncoding
MSTRG.84724.1		XLOC_093879	302	coding	noncoding	noncoding	noncoding
MSTRG.84786.1		XLOC_093923	362	coding	noncoding	noncoding	noncoding
MSTRG.84790.1		XLOC_093924	278	coding	noncoding	noncoding	noncoding
NR_149140.1	LINC02530	XLOC_093928	1041	coding	noncoding	noncoding	noncoding
MSTRG.84794.1		XLOC_093929	264	coding	noncoding	noncoding	noncoding
MSTRG.84909.1	TBC1D7-LOC100130357	XLOC_093953	308	coding	noncoding	noncoding	noncoding
MSTRG.84801.1		XLOC_093955	288	coding	noncoding	noncoding	noncoding
MSTRG.84866.1		XLOC_093967	212	coding	noncoding	noncoding	noncoding
MSTRG.84974.1		XLOC_093971	225	coding	noncoding	noncoding	noncoding
MSTRG.84977.1	MCUR1	XLOC_093973	273	coding	noncoding	noncoding	noncoding
MSTRG.84979.1		XLOC_093977	225	coding	noncoding	noncoding	noncoding
MSTRG.84990.7	RNF182	XLOC_093980	3608	coding	noncoding	coding	coding
MSTRG.84996.1		XLOC_093982	347	coding	noncoding	noncoding	noncoding
MSTRG.85124.1	LOC101928354	XLOC_094036	270	coding	noncoding	noncoding	noncoding
MSTRG.85083.1	LOC105374945	XLOC_094049	270	coding	noncoding	noncoding	noncoding
MSTRG.85059.1		XLOC_094052	219	coding	noncoding	noncoding	noncoding
MSTRG.85060.1		XLOC_094053	277	coding	noncoding	noncoding	noncoding
MSTRG.85063.1		XLOC_094055	247	coding	noncoding	noncoding	noncoding
MSTRG.85070.1	LOC105374946	XLOC_094059	242	coding	noncoding	noncoding	noncoding
MSTRG.85068.1		XLOC_094061	236	coding	noncoding	noncoding	noncoding
MSTRG.85093.1		XLOC_094062	290	coding	noncoding	noncoding	noncoding
MSTRG.85177.1		XLOC_094085	237	coding	noncoding	noncoding	noncoding
MSTRG.85185.1		XLOC_094093	345	coding	noncoding	noncoding	noncoding
MSTRG.85203.1		XLOC_094172	248	coding	noncoding	noncoding	noncoding
MSTRG.85220.1		XLOC_094187	503	coding	noncoding	noncoding	noncoding
MSTRG.85221.1		XLOC_094188	264	coding	noncoding	noncoding	noncoding
MSTRG.85236.1		XLOC_094195	202	coding	noncoding	noncoding	noncoding

MSTRG.85237.1		XLOC_094196	216	coding	noncoding	noncoding	noncoding
MSTRG.85253.1	CAP2	XLOC_094202	259	coding	noncoding	noncoding	noncoding
MSTRG.85256.1	CAP2	XLOC_094204	229	coding	noncoding	noncoding	noncoding
MSTRG.85268.1		XLOC_094217	258	coding	noncoding	noncoding	noncoding
MSTRG.85310.10	KIF13A	XLOC_094218	692	coding	noncoding	noncoding	noncoding
MSTRG.85322.1	KIF13A	XLOC_094230	697	coding	noncoding	noncoding	noncoding
MSTRG.85278.1		XLOC_094234	251	coding	noncoding	noncoding	noncoding
MSTRG.85293.1		XLOC_094245	238	coding	noncoding	noncoding	noncoding
MSTRG.85338.1		XLOC_094263	283	coding	noncoding	noncoding	noncoding
MSTRG.85386.1		XLOC_094271	232	coding	noncoding	noncoding	noncoding
MSTRG.85408.1		XLOC_094277	642	coding	coding	noncoding	noncoding
MSTRG.85411.1	LOC105374959	XLOC_094279	261	coding	noncoding	noncoding	noncoding
NM_001080480.3	MBOAT1	XLOC_094284	4325	coding	coding	coding	coding
MSTRG.85565.3	E2F3	XLOC_094305	597	coding	coding	noncoding	noncoding
MSTRG.85808.3	CDKAL1	XLOC_094309	1567	coding	coding	noncoding	noncoding
MSTRG.85607.1		XLOC_094331	205	coding	noncoding	noncoding	noncoding
MSTRG.85638.1	CASC15	XLOC_094339	254	coding	noncoding	noncoding	noncoding
MSTRG.85656.1	CASC15	XLOC_094352	294	coding	noncoding	noncoding	noncoding
MSTRG.85631.1		XLOC_094372	240	coding	noncoding	noncoding	noncoding
MSTRG.85676.1		XLOC_094376	241	coding	noncoding	noncoding	noncoding
MSTRG.85686.1		XLOC_094377	295	coding	noncoding	noncoding	noncoding
MSTRG.85689.1		XLOC_094378	293	coding	noncoding	noncoding	noncoding
MSTRG.85695.1		XLOC_094380	395	coding	noncoding	noncoding	noncoding
MSTRG.85696.1		XLOC_094383	234	coding	coding	noncoding	noncoding
MSTRG.85697.1		XLOC_094384	285	coding	noncoding	noncoding	noncoding
MSTRG.85711.1		XLOC_094397	303	coding	coding	noncoding	noncoding
MSTRG.85713.1		XLOC_094399	273	coding	noncoding	noncoding	noncoding
MSTRG.85715.1		XLOC_094401	278	coding	noncoding	noncoding	noncoding
MSTRG.85721.1		XLOC_094405	303	coding	noncoding	noncoding	noncoding
XM_005249433.4	C6orf62	XLOC_094428	1987	coding	coding	coding	coding
MSTRG.85886.1		XLOC_094477	205	coding	noncoding	noncoding	noncoding
MSTRG.85893.1	SCGN	XLOC_094535	238	coding	noncoding	noncoding	noncoding
MSTRG.86299.3	BTN3A2	XLOC_094609	3874	coding	coding	noncoding	noncoding
MSTRG.86138.1	BTN1A1	XLOC_094612	304	coding	noncoding	noncoding	noncoding
MSTRG.86148.3	HMGN4	XLOC_094616	2709	coding	coding	noncoding	noncoding
MSTRG.86186.1		XLOC_094657	321	coding	coding	noncoding	noncoding
MSTRG.86190.1		XLOC_094661	312	coding	noncoding	noncoding	noncoding
MSTRG.86194.1		XLOC_094662	223	coding	noncoding	noncoding	noncoding
MSTRG.86197.1		XLOC_094663	280	coding	noncoding	noncoding	noncoding
MSTRG.86198.1		XLOC_094667	241	coding	noncoding	noncoding	noncoding
MSTRG.86264.1		XLOC_094695	249	coding	noncoding	noncoding	noncoding
MSTRG.86279.1		XLOC_094704	292	coding	noncoding	noncoding	noncoding
MSTRG.86350.1		XLOC_094743	254	coding	coding	noncoding	noncoding

MSTRG.86442.1	ZBED9	XLOC_094782	293 coding	noncoding	noncoding	noncoding
NM_007243.2	NRM	XLOC_094848	1755 coding	noncoding	coding	coding
NR_037853.1	ATP6V1G2-DDX39B	XLOC_094879	2305 coding	coding	coding	coding
MSTRG.86589.3	SNHG32	XLOC_094905	485 coding	coding	noncoding	noncoding
XR_001744085.1	LOC107986589	XLOC_094935	3380 coding	noncoding	noncoding	noncoding
NM_006120.4	HLA-DMA	XLOC_094948	1093 coding	coding	coding	coding
MSTRG.86683.1	RPS18	XLOC_094959	934 coding	coding	noncoding	noncoding
MSTRG.86706.1		XLOC_094975	273 coding	noncoding	noncoding	noncoding
MSTRG.86765.1	LEMD2	XLOC_094989	223 coding	noncoding	noncoding	noncoding
MSTRG.86730.1		XLOC_094997	244 coding	noncoding	noncoding	noncoding
NM_000841.4	GRM4	XLOC_095001	7368 coding	coding	coding	coding
MSTRG.86735.1		XLOC_095006	247 coding	noncoding	noncoding	noncoding
MSTRG.86745.1		XLOC_095009	310 coding	noncoding	noncoding	noncoding
MSTRG.86793.1	RPS10-NUDT3	XLOC_095020	254 coding	noncoding	noncoding	noncoding
MSTRG.86799.1	RPS10-NUDT3	XLOC_095025	274 coding	noncoding	noncoding	noncoding
MSTRG.86781.1	PACSIN1	XLOC_095028	245 coding	noncoding	noncoding	noncoding
MSTRG.86805.1		XLOC_095044	351 coding	noncoding	noncoding	noncoding
MSTRG.86833.1		XLOC_095068	299 coding	noncoding	noncoding	noncoding
MSTRG.86883.1	TEAD3	XLOC_095096	237 coding	noncoding	noncoding	noncoding
MSTRG.86941.5	FKBP5	XLOC_095101	814 coding	coding	coding	noncoding
MSTRG.86964.1	FKBP5	XLOC_095122	299 coding	noncoding	noncoding	noncoding
MSTRG.86971.1	ARMC12	XLOC_095125	321 coding	noncoding	noncoding	noncoding
MSTRG.86972.1		XLOC_095127	244 coding	noncoding	coding	noncoding
MSTRG.86984.1		XLOC_095136	206 coding	noncoding	noncoding	noncoding
MSTRG.87057.3	BRPF3	XLOC_095154	2396 coding	coding	coding	coding
XM_006714988.4	STK38	XLOC_095168	3403 coding	coding	coding	coding
MSTRG.87069.5	SRSF3	XLOC_095169	5303 coding	coding	noncoding	noncoding
NR_144384.1	DINOL	XLOC_095174	951 coding	noncoding	noncoding	noncoding
MSTRG.87118.1	C6orf89	XLOC_095188	267 coding	noncoding	noncoding	noncoding
MSTRG.87123.3	C6orf89	XLOC_095191	4060 coding	noncoding	coding	noncoding
MSTRG.87132.1		XLOC_095203	263 coding	noncoding	noncoding	noncoding
MSTRG.87359.6	ZFAND3	XLOC_095232	3169 coding	noncoding	noncoding	noncoding
MSTRG.87498.1	BTBD9	XLOC_095260	259 coding	noncoding	noncoding	noncoding
MSTRG.87501.1	BTBD9	XLOC_095263	291 coding	noncoding	noncoding	noncoding
MSTRG.87503.1	BTBD9	XLOC_095265	585 coding	noncoding	noncoding	noncoding
MSTRG.87230.1	DNAH8	XLOC_095329	212 coding	noncoding	noncoding	noncoding
NM_003740.4	KCNK5	XLOC_095334	3783 coding	coding	coding	coding
MSTRG.87199.1	KCNK5	XLOC_095335	307 coding	noncoding	noncoding	noncoding
NM_145027.6	KIF6	XLOC_095343	9085 coding	coding	coding	coding
MSTRG.87252.1	KIF6	XLOC_095348	312 coding	noncoding	noncoding	noncoding
MSTRG.87273.1		XLOC_095370	298 coding	noncoding	noncoding	noncoding
MSTRG.87304.1		XLOC_095378	280 coding	noncoding	noncoding	noncoding
XR_926281.2	LRFN2	XLOC_095380	13980 coding	noncoding	coding	coding

MSTRG.87322.1	LOC105375053	XLOC_095393	217	coding	coding	noncoding	noncoding
MSTRG.87705.1		XLOC_095425	227	coding	noncoding	noncoding	noncoding
MSTRG.87560.1		XLOC_095436	300	coding	noncoding	noncoding	noncoding
MSTRG.87580.1		XLOC_095454	301	coding	noncoding	noncoding	noncoding
MSTRG.87746.2	BYSL	XLOC_095475	416	coding	coding	coding	noncoding
MSTRG.87762.1	CCND3	XLOC_095489	585	coding	noncoding	noncoding	noncoding
NM_001164446.3	C6orf132	XLOC_095505	6385	coding	noncoding	coding	coding
XM_005249206.3	MRPS10	XLOC_095508	2094	coding	coding	noncoding	coding
XM_011514725.3	MRPS10	XLOC_095508	2142	coding	coding	noncoding	coding
NM_001008739.2	C6orf226	XLOC_095549	557	coding	noncoding	noncoding	noncoding
MSTRG.87872.1	CNPY3-GNMT	XLOC_095557	201	coding	noncoding	noncoding	noncoding
MSTRG.87878.1	PEX6	XLOC_095560	240	coding	noncoding	noncoding	noncoding
MSTRG.87896.1	PTK7	XLOC_095574	262	coding	noncoding	noncoding	noncoding
MSTRG.87900.1	PTK7	XLOC_095578	313	coding	noncoding	noncoding	noncoding
MSTRG.87963.1	TJAP1	XLOC_095605	452	coding	noncoding	noncoding	noncoding
MSTRG.87974.1		XLOC_095629	250	coding	noncoding	noncoding	noncoding
MSTRG.87992.1	LOC107986598	XLOC_095633	281	coding	noncoding	noncoding	noncoding
MSTRG.88074.1		XLOC_095671	324	coding	noncoding	noncoding	noncoding
MSTRG.88081.1		XLOC_095677	290	coding	noncoding	coding	noncoding
MSTRG.88181.1		XLOC_095767	255	coding	noncoding	noncoding	noncoding
NM_153840.4	ADGRF1	XLOC_095775	5432	coding	coding	coding	coding
MSTRG.88222.1		XLOC_095786	224	coding	noncoding	noncoding	noncoding
XM_017010896.1	PTCHD4	XLOC_095797	4671	coding	coding	coding	coding
MSTRG.88283.1	PTCHD4	XLOC_095802	312	coding	noncoding	noncoding	noncoding
MSTRG.88258.1		XLOC_095807	213	coding	noncoding	noncoding	noncoding
MSTRG.88288.1		XLOC_095809	476	coding	coding	noncoding	noncoding
MSTRG.88307.1		XLOC_095815	293	coding	noncoding	noncoding	noncoding
MSTRG.88349.1		XLOC_095837	288	coding	noncoding	noncoding	noncoding
MSTRG.88379.1		XLOC_095855	200	coding	noncoding	noncoding	noncoding
MSTRG.88396.1		XLOC_095863	269	coding	noncoding	noncoding	noncoding
MSTRG.88403.1		XLOC_095867	260	coding	noncoding	noncoding	noncoding
XM_011514535.3	GSTA4	XLOC_095936	1376	coding	coding	coding	coding
MSTRG.88564.1	GCM1	XLOC_095949	262	coding	noncoding	noncoding	noncoding
MSTRG.88566.1	GCM1	XLOC_095950	258	coding	noncoding	noncoding	noncoding
MSTRG.88576.1	GCM1	XLOC_095953	238	coding	noncoding	noncoding	noncoding
XM_017010749.1	GCLC	XLOC_095971	3262	coding	coding	coding	coding
MSTRG.88556.1		XLOC_095978	450	coding	coding	noncoding	noncoding
XR_001744176.2	LOC107986606	XLOC_096020	3220	coding	coding	noncoding	noncoding
MSTRG.88678.1	LOC107986606	XLOC_096022	382	coding	noncoding	noncoding	noncoding
MSTRG.88687.1	FAM83B	XLOC_096028	223	coding	noncoding	noncoding	noncoding
MSTRG.88681.1		XLOC_096030	254	coding	noncoding	noncoding	noncoding
MSTRG.88683.1		XLOC_096031	210	coding	noncoding	noncoding	noncoding
XM_011514817.3	BMP5	XLOC_096043	7603	coding	coding	coding	coding

MSTRG.88962.1	DST	XLOC_096066	369	coding	noncoding	noncoding	noncoding
MSTRG.88870.1		XLOC_096098	272	coding	noncoding	noncoding	noncoding
MSTRG.88923.1	LINC00680-GUSBP4	XLOC_096106	291	coding	noncoding	noncoding	noncoding
MSTRG.88941.1		XLOC_096122	313	coding	noncoding	noncoding	noncoding
MSTRG.88947.1		XLOC_096125	294	coding	noncoding	noncoding	noncoding
MSTRG.89051.1		XLOC_096154	201	coding	noncoding	noncoding	noncoding
MSTRG.89044.1		XLOC_096164	316	coding	noncoding	noncoding	noncoding
MSTRG.89059.1		XLOC_096165	251	coding	coding	noncoding	noncoding
MSTRG.89063.1		XLOC_096167	276	coding	noncoding	noncoding	noncoding
MSTRG.89065.1		XLOC_096169	313	coding	noncoding	noncoding	noncoding
MSTRG.89211.1		XLOC_096211	263	coding	noncoding	noncoding	noncoding
MSTRG.89215.1	LOC107986540	XLOC_096212	270	coding	noncoding	noncoding	noncoding
MSTRG.89216.1		XLOC_096214	259	coding	noncoding	noncoding	noncoding
MSTRG.89225.1		XLOC_096221	282	coding	coding	noncoding	noncoding
MSTRG.89290.1	ADGRB3	XLOC_096251	291	coding	noncoding	noncoding	noncoding
MSTRG.89248.1		XLOC_096258	282	coding	noncoding	noncoding	noncoding
MSTRG.89420.3	FAM135A	XLOC_096310	2360	coding	coding	noncoding	coding
MSTRG.89487.1		XLOC_096326	292	coding	noncoding	noncoding	noncoding
MSTRG.89561.1	RIMS1	XLOC_096354	327	coding	coding	noncoding	noncoding
MSTRG.89562.1	RIMS1	XLOC_096355	270	coding	noncoding	noncoding	noncoding
XR_002956358.1	LOC112267961	XLOC_096396	2276	coding	noncoding	coding	noncoding
MSTRG.89593.1	KHDC1	XLOC_096404	271	coding	noncoding	noncoding	noncoding
MSTRG.89578.1	KHDC3L	XLOC_096407	323	coding	coding	noncoding	noncoding
MSTRG.89603.1		XLOC_096412	317	coding	noncoding	noncoding	noncoding
MSTRG.89607.1	CGAS	XLOC_096414	230	coding	noncoding	noncoding	noncoding
MSTRG.89610.3	EEF1A1	XLOC_096416	2303	coding	coding	coding	coding
MSTRG.89617.1	SLC17A5	XLOC_096421	282	coding	noncoding	noncoding	noncoding
MSTRG.89664.1	LOC101928516	XLOC_096440	336	coding	noncoding	noncoding	noncoding
MSTRG.89630.1		XLOC_096449	582	coding	coding	noncoding	noncoding
MSTRG.89683.1		XLOC_096451	278	coding	noncoding	noncoding	noncoding
NM_001289987.3	FILIP1	XLOC_096468	4802	coding	coding	coding	coding
MSTRG.89719.1	LOC101928540	XLOC_096476	259	coding	noncoding	noncoding	noncoding
MSTRG.89687.1		XLOC_096480	217	coding	noncoding	noncoding	noncoding
MSTRG.89779.14	SENP6	XLOC_096483	3219	coding	coding	noncoding	noncoding
MSTRG.89730.1		XLOC_096485	246	coding	noncoding	noncoding	noncoding
MSTRG.89749.1		XLOC_096500	254	coding	noncoding	coding	noncoding
MSTRG.89754.1	LINC02540	XLOC_096502	211	coding	noncoding	noncoding	noncoding
MSTRG.89764.1	LOC105377861	XLOC_096507	235	coding	noncoding	noncoding	noncoding
MSTRG.89765.1	LOC105377861	XLOC_096508	255	coding	noncoding	noncoding	noncoding
MSTRG.89853.1	LOC105377862	XLOC_096511	292	coding	noncoding	noncoding	noncoding
MSTRG.89854.1	LOC105377862	XLOC_096512	282	coding	noncoding	noncoding	noncoding
MSTRG.89865.1	LOC105377862	XLOC_096516	328	coding	noncoding	noncoding	noncoding
NM_000863.2	HTR1B	XLOC_096524	3175	coding	coding	coding	coding

MSTRG.89821.1	MEI4	XLOC_096527	258 coding	noncoding	noncoding	noncoding
MSTRG.89825.1	MEI4	XLOC_096528	279 coding	coding	noncoding	noncoding
MSTRG.89827.1	MEI4	XLOC_096529	264 coding	coding	coding	noncoding
MSTRG.89834.1	MEI4	XLOC_096533	265 coding	coding	noncoding	noncoding
MSTRG.89840.1	LOC105377865	XLOC_096536	259 coding	noncoding	noncoding	noncoding
MSTRG.89842.1	LOC105377865	XLOC_096537	343 coding	noncoding	noncoding	noncoding
MSTRG.89893.1		XLOC_096546	270 coding	noncoding	noncoding	noncoding
MSTRG.89975.1	LOC107986613	XLOC_096550	266 coding	coding	noncoding	noncoding
MSTRG.89981.1	IRAK1BP1	XLOC_096554	337 coding	noncoding	noncoding	noncoding
MSTRG.89914.1		XLOC_096602	271 coding	noncoding	noncoding	noncoding
MSTRG.89934.1		XLOC_096614	231 coding	noncoding	noncoding	noncoding
MSTRG.89951.1		XLOC_096621	277 coding	noncoding	noncoding	noncoding
MSTRG.89964.1		XLOC_096624	219 coding	noncoding	noncoding	noncoding
MSTRG.90047.1		XLOC_096687	277 coding	noncoding	noncoding	noncoding
MSTRG.90049.1		XLOC_096688	228 coding	noncoding	noncoding	noncoding
XR_001744224.1	LOC105377872	XLOC_096700	12153 coding	noncoding	noncoding	noncoding
MSTRG.90091.1	LINC02542	XLOC_096709	660 coding	coding	noncoding	noncoding
MSTRG.90103.1	LOC107986617	XLOC_096713	247 coding	noncoding	noncoding	noncoding
MSTRG.90173.1		XLOC_096719	476 coding	noncoding	noncoding	noncoding
MSTRG.90402.1	ME1	XLOC_096737	224 coding	noncoding	noncoding	noncoding
MSTRG.90372.1		XLOC_096739	644 coding	noncoding	noncoding	noncoding
MSTRG.90413.1	SNAP91	XLOC_096744	315 coding	noncoding	noncoding	noncoding
MSTRG.90409.1		XLOC_096748	315 coding	noncoding	noncoding	noncoding
MSTRG.90411.1		XLOC_096749	259 coding	noncoding	noncoding	noncoding
MSTRG.90542.1	MRAP2	XLOC_096767	248 coding	noncoding	noncoding	noncoding
MSTRG.90437.1		XLOC_096772	264 coding	noncoding	noncoding	noncoding
MSTRG.90531.1		XLOC_096792	220 coding	noncoding	noncoding	noncoding
MSTRG.90532.1		XLOC_096793	235 coding	noncoding	noncoding	noncoding
NM_001350553.1	SNX14	XLOC_096796	3464 coding	coding	coding	coding
NR_146777.1	SNX14	XLOC_096796	4000 coding	coding	coding	coding
MSTRG.90629.1	SNX14	XLOC_096807	481 coding	noncoding	coding	noncoding
NM_001159676.1	SYNCRIP	XLOC_096814	6796 coding	coding	coding	coding
MSTRG.90559.1	LOC107986622	XLOC_096824	277 coding	noncoding	noncoding	noncoding
MSTRG.90568.1		XLOC_096830	290 coding	noncoding	noncoding	noncoding
MSTRG.90574.1	LOC101928842	XLOC_096832	238 coding	noncoding	noncoding	noncoding
MSTRG.90579.1		XLOC_096834	296 coding	noncoding	noncoding	noncoding
MSTRG.90584.1		XLOC_096836	286 coding	noncoding	noncoding	noncoding
MSTRG.90596.1		XLOC_096839	432 coding	noncoding	coding	noncoding
MSTRG.90720.1	CFAP206	XLOC_096880	243 coding	noncoding	noncoding	noncoding
MSTRG.90790.2	ORC3	XLOC_096896	2553 coding	coding	noncoding	noncoding
MSTRG.90727.1	SPACA1	XLOC_096911	234 coding	noncoding	noncoding	noncoding
MSTRG.90890.1	RNGTT	XLOC_096942	241 coding	noncoding	noncoding	noncoding
MSTRG.90909.1	RNGTT	XLOC_096960	225 coding	noncoding	noncoding	noncoding

MSTRG.90833.1	PM20D2	XLOC_096977	320 coding	noncoding	noncoding	noncoding
MSTRG.90837.1	PM20D2	XLOC_096981	327 coding	noncoding	noncoding	noncoding
NM_145332.3	MAP3K7	XLOC_097123	4816 coding	coding	coding	coding
MSTRG.91031.1		XLOC_097146	270 coding	noncoding	noncoding	noncoding
MSTRG.91037.1		XLOC_097152	293 coding	noncoding	noncoding	noncoding
MSTRG.91029.1		XLOC_097155	603 coding	noncoding	noncoding	noncoding
MSTRG.91236.1		XLOC_097209	203 coding	noncoding	noncoding	noncoding
MSTRG.91237.1		XLOC_097210	223 coding	noncoding	noncoding	noncoding
MSTRG.91321.1	UFL1-AS1	XLOC_097238	278 coding	coding	noncoding	noncoding
MSTRG.91327.1	UFL1-AS1	XLOC_097240	207 coding	coding	noncoding	noncoding
MSTRG.91297.1		XLOC_097245	274 coding	coding	noncoding	noncoding
XR_942376.1	MMS22L	XLOC_097285	8456 coding	coding	coding	coding
MSTRG.91359.1	LOC101927314	XLOC_097298	245 coding	noncoding	noncoding	noncoding
MSTRG.91364.1	LOC101927314	XLOC_097301	310 coding	coding	noncoding	noncoding
MSTRG.91377.1		XLOC_097313	258 coding	noncoding	noncoding	noncoding
NM_001322406.2	PNISR	XLOC_097339	5225 coding	coding	coding	coding
MSTRG.91821.1	LOC107984041	XLOC_097442	265 coding	noncoding	noncoding	noncoding
MSTRG.91632.1	GRIK2	XLOC_097454	351 coding	noncoding	noncoding	noncoding
MSTRG.91612.1		XLOC_097459	265 coding	noncoding	noncoding	noncoding
MSTRG.91637.1		XLOC_097466	309 coding	noncoding	noncoding	noncoding
MSTRG.91642.1		XLOC_097469	635 coding	noncoding	coding	noncoding
MSTRG.91670.1		XLOC_097486	221 coding	noncoding	noncoding	noncoding
MSTRG.91677.1		XLOC_097491	225 coding	noncoding	noncoding	noncoding
MSTRG.91850.1	HACE1	XLOC_097509	272 coding	noncoding	noncoding	noncoding
MSTRG.91853.1	HACE1	XLOC_097512	239 coding	noncoding	noncoding	noncoding
MSTRG.91753.1	LIN28B	XLOC_097515	222 coding	noncoding	noncoding	noncoding
MSTRG.91857.1		XLOC_097537	318 coding	noncoding	noncoding	noncoding
MSTRG.91919.1	LOC105377923	XLOC_097550	220 coding	noncoding	noncoding	noncoding
MSTRG.91927.1	LOC105377923	XLOC_097553	215 coding	noncoding	noncoding	noncoding
MSTRG.91945.1	PRDM1	XLOC_097563	304 coding	noncoding	noncoding	noncoding
MSTRG.91942.1		XLOC_097567	320 coding	noncoding	noncoding	noncoding
MSTRG.92010.1	CRYBG1	XLOC_097587	294 coding	noncoding	noncoding	noncoding
MSTRG.92019.1	CRYBG1	XLOC_097592	267 coding	noncoding	noncoding	noncoding
MSTRG.91998.1		XLOC_097606	399 coding	noncoding	noncoding	noncoding
MSTRG.91999.1		XLOC_097607	276 coding	noncoding	noncoding	noncoding
MSTRG.92121.1		XLOC_097614	247 coding	noncoding	noncoding	noncoding
MSTRG.92127.1	PDSS2	XLOC_097617	277 coding	noncoding	noncoding	noncoding
MSTRG.92129.1	PDSS2	XLOC_097619	219 coding	noncoding	noncoding	noncoding
MSTRG.92087.1	OSTM1-AS1	XLOC_097667	236 coding	noncoding	noncoding	noncoding
MSTRG.92090.1	NR2E1	XLOC_097668	536 coding	noncoding	coding	noncoding
MSTRG.92173.1	AFG1L	XLOC_097678	302 coding	noncoding	noncoding	noncoding
MSTRG.92335.1	SESN1	XLOC_097745	218 coding	noncoding	noncoding	noncoding
MSTRG.92348.1	CCDC162P	XLOC_097757	236 coding	noncoding	noncoding	noncoding

NM_022765.4	MICAL1	XLOC_097774	3430 coding	coding	coding	coding
MSTRG.92458.1	AK9	XLOC_097782	276 coding	noncoding	noncoding	noncoding
MSTRG.92288.1		XLOC_097833	248 coding	noncoding	noncoding	noncoding
MSTRG.92508.1	CDK19	XLOC_097841	275 coding	noncoding	noncoding	noncoding
MSTRG.92532.1	CDK19	XLOC_097864	264 coding	noncoding	noncoding	noncoding
XR_001743550.2	REV3L	XLOC_097890	10562 coding	coding	coding	coding
MSTRG.92564.1		XLOC_097962	314 coding	coding	noncoding	coding
MSTRG.92619.1		XLOC_097987	216 coding	noncoding	noncoding	noncoding
MSTRG.92627.1		XLOC_097992	275 coding	noncoding	noncoding	noncoding
MSTRG.92645.1		XLOC_097998	336 coding	noncoding	noncoding	noncoding
MSTRG.92714.1	HDAC2-AS2	XLOC_098016	270 coding	noncoding	noncoding	noncoding
MSTRG.92667.1	LOC105377959	XLOC_098022	242 coding	noncoding	noncoding	noncoding
MSTRG.92670.1		XLOC_098025	224 coding	noncoding	noncoding	noncoding
MSTRG.92675.1		XLOC_098028	293 coding	coding	noncoding	noncoding
MSTRG.92679.1		XLOC_098031	301 coding	noncoding	noncoding	noncoding
MSTRG.92683.1		XLOC_098034	346 coding	coding	noncoding	noncoding
MSTRG.92728.1		XLOC_098041	307 coding	noncoding	noncoding	noncoding
MSTRG.92723.1		XLOC_098042	244 coding	coding	noncoding	noncoding
MSTRG.92732.1		XLOC_098044	464 coding	noncoding	coding	noncoding
MSTRG.92735.1		XLOC_098045	288 coding	noncoding	noncoding	noncoding
MSTRG.92781.2	CALHM6	XLOC_098094	586 coding	coding	coding	noncoding
MSTRG.92827.1	RFX6	XLOC_098123	286 coding	noncoding	noncoding	noncoding
MSTRG.92945.1		XLOC_098170	313 coding	noncoding	noncoding	noncoding
MSTRG.93102.1		XLOC_098182	373 coding	noncoding	noncoding	noncoding
MSTRG.93169.1	LOC107986524	XLOC_098205	243 coding	noncoding	noncoding	noncoding
MSTRG.93218.1	MCM9	XLOC_098234	246 coding	noncoding	noncoding	noncoding
MSTRG.93219.1	MCM9	XLOC_098235	264 coding	noncoding	noncoding	noncoding
MSTRG.93229.1		XLOC_098244	316 coding	noncoding	noncoding	noncoding
XM_005266986.4	MAN1A1	XLOC_098262	4880 coding	coding	coding	coding
MSTRG.93241.1		XLOC_098297	222 coding	noncoding	noncoding	noncoding
MSTRG.93243.1		XLOC_098299	422 coding	noncoding	noncoding	noncoding
MSTRG.93255.1		XLOC_098306	270 coding	noncoding	noncoding	noncoding
MSTRG.93264.1		XLOC_098310	263 coding	noncoding	noncoding	noncoding
MSTRG.93265.1		XLOC_098311	227 coding	noncoding	noncoding	noncoding
MSTRG.93435.1	TBC1D32	XLOC_098332	273 coding	noncoding	noncoding	noncoding
MSTRG.93284.1		XLOC_098335	222 coding	noncoding	noncoding	noncoding
MSTRG.93287.1		XLOC_098337	254 coding	noncoding	noncoding	noncoding
MSTRG.93321.1	LOC105377979	XLOC_098350	230 coding	coding	noncoding	noncoding
MSTRG.93326.1	LOC105377979	XLOC_098353	301 coding	noncoding	noncoding	noncoding
MSTRG.93304.1		XLOC_098354	291 coding	noncoding	noncoding	noncoding
MSTRG.93307.1		XLOC_098355	255 coding	noncoding	noncoding	noncoding
MSTRG.93309.1		XLOC_098357	308 coding	noncoding	noncoding	noncoding
MSTRG.93312.1		XLOC_098358	293 coding	noncoding	noncoding	noncoding

MSTRG.93333.1		XLOC_098391	285	coding	noncoding	noncoding	noncoding
MSTRG.93337.1		XLOC_098393	274	coding	noncoding	noncoding	noncoding
MSTRG.93340.1		XLOC_098395	284	coding	noncoding	noncoding	noncoding
MSTRG.93351.1		XLOC_098399	248	coding	noncoding	noncoding	noncoding
MSTRG.93503.1	NKAIN2	XLOC_098416	249	coding	noncoding	noncoding	noncoding
MSTRG.93514.1	NKAIN2	XLOC_098425	237	coding	coding	noncoding	noncoding
MSTRG.93523.1	NKAIN2	XLOC_098432	206	coding	noncoding	noncoding	noncoding
MSTRG.93558.1	TPD52L1	XLOC_098471	256	coding	noncoding	noncoding	noncoding
MSTRG.93561.1		XLOC_098478	248	coding	noncoding	noncoding	noncoding
MSTRG.93693.1		XLOC_098590	297	coding	noncoding	noncoding	noncoding
XR_002956389.1	LOC105377989	XLOC_098591	7422	coding	noncoding	noncoding	noncoding
MSTRG.93697.1		XLOC_098593	283	coding	noncoding	noncoding	noncoding
MSTRG.93710.1		XLOC_098598	257	coding	noncoding	noncoding	noncoding
MSTRG.93900.1	PTPRK	XLOC_098644	237	coding	noncoding	noncoding	noncoding
MSTRG.93942.1	LAMA2	XLOC_098653	244	coding	noncoding	noncoding	noncoding
MSTRG.93945.1	LAMA2	XLOC_098655	293	coding	noncoding	noncoding	noncoding
MSTRG.93961.1	LAMA2	XLOC_098666	359	coding	coding	noncoding	noncoding
MSTRG.93964.1	LAMA2	XLOC_098668	561	coding	noncoding	noncoding	noncoding
MSTRG.94003.1	ARHGAP18	XLOC_098680	322	coding	noncoding	noncoding	noncoding
NM_001017373.4	SAMD3	XLOC_098695	2093	coding	coding	coding	coding
MSTRG.94018.1		XLOC_098742	242	coding	noncoding	noncoding	noncoding
MSTRG.94057.1	LOC107986643	XLOC_098758	3400	coding	coding	noncoding	noncoding
MSTRG.94100.1	ENPP1	XLOC_098796	282	coding	noncoding	noncoding	noncoding
MSTRG.94106.1		XLOC_098800	291	coding	noncoding	noncoding	noncoding
NM_138327.2	TAAR1	XLOC_098841	1129	coding	coding	coding	coding
MSTRG.94307.1	SLC18B1	XLOC_098861	237	coding	noncoding	noncoding	noncoding
MSTRG.94311.1	LOC105378008	XLOC_098868	294	coding	noncoding	noncoding	noncoding
MSTRG.94360.1	TARID	XLOC_098881	252	coding	noncoding	noncoding	noncoding
MSTRG.94370.1		XLOC_098888	264	coding	noncoding	noncoding	noncoding
MSTRG.94385.1		XLOC_098898	265	coding	noncoding	noncoding	noncoding
MSTRG.94404.1		XLOC_098902	411	coding	noncoding	noncoding	noncoding
NR_125852.1	CT69	XLOC_098903	1188	coding	noncoding	noncoding	noncoding
MSTRG.94426.1	LOC101928277	XLOC_098914	239	coding	noncoding	noncoding	noncoding
MSTRG.94489.1	HBS1L	XLOC_098939	319	coding	noncoding	noncoding	noncoding
MSTRG.94458.1	LOC105378010	XLOC_098943	274	coding	noncoding	noncoding	noncoding
MSTRG.94491.2	LOC105378011	XLOC_098945	451	coding	coding	noncoding	noncoding
MSTRG.94462.1		XLOC_098946	264	coding	noncoding	noncoding	noncoding
MSTRG.94536.1	LINC00271	XLOC_098958	289	coding	noncoding	noncoding	noncoding
XR_001743770.2	BCLAF1	XLOC_099017	5470	coding	coding	coding	coding
XR_001743771.1	BCLAF1	XLOC_099017	6152	coding	coding	coding	coding
MSTRG.94540.1		XLOC_099023	318	coding	noncoding	noncoding	noncoding
MSTRG.94547.1		XLOC_099025	231	coding	noncoding	noncoding	noncoding
MSTRG.94770.1	MAP7	XLOC_099028	261	coding	noncoding	noncoding	noncoding

MSTRG.94774.1	MAP7	XLOC_099029	298 coding	coding	noncoding	noncoding
MSTRG.94805.1	MAP3K5	XLOC_099051	274 coding	noncoding	noncoding	noncoding
MSTRG.94807.1	MAP3K5	XLOC_099053	296 coding	noncoding	noncoding	noncoding
MSTRG.94631.1		XLOC_099096	265 coding	coding	noncoding	noncoding
MSTRG.94636.1	IL22RA2	XLOC_099100	298 coding	coding	noncoding	noncoding
MSTRG.94691.1	LOC102723649	XLOC_099116	310 coding	noncoding	noncoding	noncoding
MSTRG.94713.1	LOC105378021	XLOC_099134	225 coding	noncoding	noncoding	noncoding
MSTRG.94722.1		XLOC_099139	273 coding	noncoding	noncoding	noncoding
MSTRG.94851.1	NHSL1	XLOC_099152	271 coding	noncoding	noncoding	noncoding
MSTRG.94855.1	NHSL1	XLOC_099155	308 coding	noncoding	noncoding	noncoding
MSTRG.94859.1	NHSL1	XLOC_099157	244 coding	noncoding	noncoding	noncoding
MSTRG.94820.1		XLOC_099159	282 coding	coding	coding	noncoding
NR_161203.1	GVQW2	XLOC_099160	2732 coding	noncoding	noncoding	noncoding
MSTRG.94884.1	ECT2L	XLOC_099169	284 coding	noncoding	noncoding	noncoding
MSTRG.94887.1	ECT2L	XLOC_099171	272 coding	noncoding	noncoding	noncoding
MSTRG.94870.1		XLOC_099188	213 coding	noncoding	noncoding	noncoding
MSTRG.94941.1	TXLNB	XLOC_099197	218 coding	noncoding	noncoding	noncoding
MSTRG.94922.1	LOC105378024	XLOC_099213	300 coding	noncoding	noncoding	noncoding
MSTRG.94929.1		XLOC_099217	379 coding	noncoding	noncoding	noncoding
MSTRG.94967.1		XLOC_099223	300 coding	noncoding	noncoding	noncoding
MSTRG.94972.1	FILNC1	XLOC_099224	262 coding	noncoding	noncoding	noncoding
MSTRG.94991.1		XLOC_099236	268 coding	coding	noncoding	noncoding
MSTRG.94992.1		XLOC_099237	248 coding	coding	noncoding	noncoding
MSTRG.94995.1		XLOC_099238	262 coding	noncoding	noncoding	noncoding
MSTRG.95023.1	LOC105378027	XLOC_099246	263 coding	noncoding	noncoding	noncoding
MSTRG.95002.1	LOC102723724	XLOC_099248	360 coding	noncoding	noncoding	noncoding
MSTRG.95009.1		XLOC_099251	535 coding	noncoding	coding	noncoding
MSTRG.95026.1		XLOC_099255	306 coding	noncoding	noncoding	noncoding
MSTRG.95082.1	LOC105378031	XLOC_099271	304 coding	noncoding	noncoding	noncoding
MSTRG.95092.1	LOC105378031	XLOC_099272	267 coding	noncoding	noncoding	noncoding
MSTRG.95077.1	NMBR	XLOC_099274	267 coding	noncoding	noncoding	noncoding
MSTRG.95093.1		XLOC_099278	294 coding	noncoding	noncoding	noncoding
MSTRG.95118.1	LOC153910	XLOC_099290	659 coding	noncoding	noncoding	noncoding
MSTRG.95281.2	LTV1	XLOC_099397	1652 coding	coding	noncoding	noncoding
MSTRG.95291.6	STX11	XLOC_099421	1807 coding	coding	coding	noncoding
MSTRG.95349.1		XLOC_099432	243 coding	noncoding	noncoding	noncoding
MSTRG.95351.1		XLOC_099433	278 coding	coding	noncoding	noncoding
MSTRG.95355.1		XLOC_099436	380 coding	noncoding	noncoding	noncoding
MSTRG.95372.1		XLOC_099445	256 coding	noncoding	noncoding	noncoding
MSTRG.95583.1	EPM2A	XLOC_099449	424 coding	noncoding	noncoding	noncoding
MSTRG.95375.1		XLOC_099499	310 coding	coding	noncoding	noncoding
MSTRG.95383.1	GRM1	XLOC_099503	317 coding	noncoding	noncoding	noncoding
MSTRG.95392.1	GRM1	XLOC_099509	227 coding	coding	noncoding	noncoding

MSTRG.95400.1	LOC101928661	XLOC_099513	268	coding	noncoding	noncoding	noncoding
MSTRG.95405.1	ADGB	XLOC_099516	312	coding	noncoding	noncoding	noncoding
MSTRG.95438.1	SAMD5	XLOC_099537	277	coding	noncoding	noncoding	noncoding
MSTRG.95559.1	SASH1	XLOC_099551	283	coding	noncoding	noncoding	noncoding
MSTRG.95657.1	PPIL4	XLOC_099626	306	coding	noncoding	noncoding	noncoding
NM_004690.4	LATS1	XLOC_099633	7362	coding	coding	coding	coding
NM_198887.3	NUP43	XLOC_099639	3831	coding	noncoding	coding	coding
MSTRG.95734.1	RAET1E-AS1	XLOC_099644	267	coding	noncoding	noncoding	noncoding
XM_017010831.2	RAET1G	XLOC_099645	2158	coding	coding	coding	coding
MSTRG.95728.1		XLOC_099646	320	coding	noncoding	noncoding	noncoding
MSTRG.95746.1		XLOC_099650	240	coding	noncoding	noncoding	noncoding
MSTRG.95803.1	LOC105378054	XLOC_099660	220	coding	noncoding	noncoding	noncoding
MSTRG.95788.1		XLOC_099662	223	coding	noncoding	noncoding	noncoding
MSTRG.95781.1		XLOC_099664	226	coding	noncoding	noncoding	noncoding
MSTRG.95776.1		XLOC_099669	267	coding	noncoding	noncoding	noncoding
MSTRG.95773.1		XLOC_099670	281	coding	noncoding	noncoding	noncoding
MSTRG.95780.1		XLOC_099671	276	coding	noncoding	noncoding	noncoding
MSTRG.95813.1		XLOC_099715	268	coding	noncoding	noncoding	noncoding
XM_017011137.1	ZBTB2	XLOC_099725	3504	coding	coding	coding	coding
MSTRG.96418.1	ESR1	XLOC_099765	250	coding	noncoding	noncoding	noncoding
XM_006715423.2	SYNE1	XLOC_099807	27714	coding	coding	noncoding	coding
MSTRG.96047.3	MYCT1	XLOC_099863	366	coding	noncoding	noncoding	noncoding
MSTRG.96115.1	RGS17	XLOC_099878	240	coding	noncoding	noncoding	noncoding
MSTRG.96058.1		XLOC_099882	205	coding	noncoding	noncoding	noncoding
MSTRG.96070.1	LOC105378066	XLOC_099885	214	coding	noncoding	noncoding	noncoding
MSTRG.96074.1		XLOC_099888	242	coding	noncoding	noncoding	noncoding
MSTRG.96084.1		XLOC_099893	209	coding	noncoding	noncoding	noncoding
MSTRG.96089.1		XLOC_099896	284	coding	noncoding	noncoding	noncoding
NM_173515.4	CNKSR3	XLOC_099929	21078	coding	coding	coding	coding
MSTRG.96120.1		XLOC_099930	287	coding	noncoding	noncoding	noncoding
MSTRG.96268.1		XLOC_099951	314	coding	noncoding	noncoding	noncoding
MSTRG.96271.1		XLOC_099953	239	coding	noncoding	noncoding	noncoding
MSTRG.96273.1		XLOC_099954	1033	coding	noncoding	noncoding	noncoding
MSTRG.96176.1		XLOC_099962	241	coding	noncoding	noncoding	noncoding
MSTRG.96803.1	LOC101928923	XLOC_099980	279	coding	noncoding	noncoding	noncoding
MSTRG.96890.1	LOC101928923	XLOC_099995	282	coding	noncoding	noncoding	noncoding
MSTRG.96916.1	LOC101928923	XLOC_099998	225	coding	noncoding	noncoding	noncoding
MSTRG.96208.1		XLOC_100018	279	coding	noncoding	noncoding	noncoding
MSTRG.96222.1		XLOC_100026	260	coding	noncoding	noncoding	noncoding
MSTRG.96291.1		XLOC_100047	236	coding	noncoding	noncoding	noncoding
MSTRG.96310.1	SNX9	XLOC_100083	308	coding	noncoding	noncoding	noncoding
MSTRG.96306.1		XLOC_100090	214	coding	noncoding	noncoding	noncoding
MSTRG.96353.1	SYNJ2	XLOC_100105	294	coding	noncoding	noncoding	noncoding

MSTRG.96408.1		XLOC_100122	254 coding	noncoding	noncoding	noncoding
MSTRG.96525.1		XLOC_100129	284 coding	noncoding	noncoding	noncoding
MSTRG.96677.3	TMEM181	XLOC_100131	6221 coding	coding	noncoding	noncoding
NM_001291602.2	DYNLT1	XLOC_100132	688 coding	coding	coding	noncoding
MSTRG.97036.1		XLOC_100195	342 coding	noncoding	noncoding	noncoding
MSTRG.97026.1		XLOC_100197	432 coding	noncoding	noncoding	noncoding
MSTRG.97041.1		XLOC_100208	301 coding	noncoding	noncoding	noncoding
MSTRG.97056.1		XLOC_100236	288 coding	noncoding	noncoding	noncoding
NM_003058.4	SLC22A2	XLOC_100239	2409 coding	coding	coding	coding
NM_005577.3	LPA	XLOC_100252	6414 coding	coding	coding	coding
MSTRG.97139.1	LPA	XLOC_100255	324 coding	noncoding	noncoding	noncoding
MSTRG.97113.1		XLOC_100256	224 coding	noncoding	noncoding	noncoding
MSTRG.97119.1	LOC105378093	XLOC_100262	253 coding	noncoding	noncoding	noncoding
MSTRG.97236.1	AGPAT4	XLOC_100279	736 coding	noncoding	noncoding	noncoding
MSTRG.97707.1	PRKN	XLOC_100298	512 coding	noncoding	noncoding	noncoding
MSTRG.97775.1	PRKN	XLOC_100313	282 coding	noncoding	noncoding	noncoding
MSTRG.97801.1	PRKN	XLOC_100322	316 coding	noncoding	noncoding	noncoding
MSTRG.97860.1	PRKN	XLOC_100333	231 coding	noncoding	noncoding	noncoding
MSTRG.97892.1	PRKN	XLOC_100335	268 coding	noncoding	noncoding	noncoding
MSTRG.97251.1		XLOC_100365	273 coding	noncoding	noncoding	noncoding
MSTRG.97264.1		XLOC_100378	287 coding	noncoding	noncoding	noncoding
MSTRG.97301.1	LOC107986667	XLOC_100399	303 coding	noncoding	noncoding	noncoding
MSTRG.97302.1	LOC107986667	XLOC_100400	312 coding	noncoding	noncoding	noncoding
MSTRG.97310.1		XLOC_100403	276 coding	noncoding	noncoding	noncoding
MSTRG.97321.1		XLOC_100408	308 coding	noncoding	noncoding	noncoding
MSTRG.97322.1		XLOC_100409	206 coding	noncoding	noncoding	noncoding
MSTRG.97323.1		XLOC_100410	263 coding	noncoding	noncoding	noncoding
XM_017010197.2	PDE10A	XLOC_100413	6375 coding	coding	coding	coding
MSTRG.97352.1	PDE10A	XLOC_100416	304 coding	noncoding	noncoding	noncoding
MSTRG.97356.1	PDE10A	XLOC_100418	351 coding	noncoding	noncoding	noncoding
NM_003181.3	TBXT	XLOC_100430	2486 coding	coding	coding	coding
XM_017011399.1	RNASET2	XLOC_100482	1172 coding	coding	coding	coding
MSTRG.97498.1		XLOC_100531	259 coding	noncoding	noncoding	noncoding
NM_024919.5	FRMD1	XLOC_100557	5699 coding	coding	coding	coding
MSTRG.97529.1		XLOC_100565	213 coding	noncoding	noncoding	noncoding
XM_011535684.3	WDR27	XLOC_100599	4150 coding	coding	coding	coding
MSTRG.97676.1	WDR27	XLOC_100603	256 coding	noncoding	noncoding	noncoding
MSTRG.97624.1		XLOC_100624	295 coding	noncoding	noncoding	noncoding
MSTRG.98009.1		XLOC_100670	324 coding	noncoding	noncoding	noncoding
MSTRG.98049.1	DNAAF5	XLOC_100685	246 coding	noncoding	noncoding	noncoding
MSTRG.98147.1	SUN1	XLOC_100689	267 coding	noncoding	noncoding	noncoding
MSTRG.98165.1		XLOC_100714	430 coding	noncoding	noncoding	coding
MSTRG.98170.1	UNCX	XLOC_100717	260 coding	noncoding	noncoding	noncoding

MSTRG.98216.1	INTS1	XLOC_100727	3746 coding	coding	coding	noncoding
MSTRG.98223.1		XLOC_100736	280 coding	noncoding	noncoding	noncoding
MSTRG.98234.1		XLOC_100739	228 coding	noncoding	noncoding	noncoding
XR_108730.4	LOC100127955	XLOC_100746	8450 coding	noncoding	coding	coding
MSTRG.98264.1	SNX8	XLOC_100756	249 coding	noncoding	noncoding	noncoding
MSTRG.98369.2	CARD11	XLOC_100780	1624 coding	noncoding	coding	noncoding
MSTRG.98358.1		XLOC_100795	377 coding	coding	noncoding	noncoding
MSTRG.98360.1	LOC100129603	XLOC_100796	237 coding	noncoding	noncoding	noncoding
XM_024446683.1	SDK1	XLOC_100798	10437 coding	coding	coding	coding
MSTRG.98565.1	SDK1	XLOC_100813	296 coding	noncoding	noncoding	noncoding
MSTRG.98388.1		XLOC_100820	242 coding	noncoding	noncoding	noncoding
MSTRG.98397.1		XLOC_100828	236 coding	noncoding	noncoding	noncoding
MSTRG.98421.1	MMD2	XLOC_100839	219 coding	noncoding	noncoding	noncoding
MSTRG.98451.1	RBAK	XLOC_100848	252 coding	noncoding	noncoding	noncoding
MSTRG.98446.1		XLOC_100857	318 coding	noncoding	noncoding	noncoding
NM_001033520.1	WIPI2	XLOC_100858	4295 coding	noncoding	coding	coding
XM_011515200.2	SLC29A4	XLOC_100860	2906 coding	coding	coding	coding
MSTRG.98603.1	SLC29A4	XLOC_100861	305 coding	noncoding	noncoding	noncoding
MSTRG.98611.1	TNRC18	XLOC_100867	313 coding	noncoding	noncoding	noncoding
MSTRG.98613.1	TNRC18	XLOC_100869	7141 coding	noncoding	noncoding	coding
MSTRG.98587.1		XLOC_100875	267 coding	noncoding	noncoding	noncoding
MSTRG.98589.1	FBXL18	XLOC_100876	235 coding	noncoding	noncoding	noncoding
MSTRG.98640.1	FSCN1	XLOC_100889	305 coding	noncoding	noncoding	noncoding
MSTRG.98632.1	OCM	XLOC_100896	292 coding	noncoding	noncoding	noncoding
MSTRG.98672.3	AIMP2	XLOC_100902	4159 coding	coding	coding	coding
MSTRG.98635.1		XLOC_100905	274 coding	noncoding	noncoding	noncoding
MSTRG.98684.1	USP42	XLOC_100907	264 coding	noncoding	noncoding	noncoding
MSTRG.98695.1	CYTH3	XLOC_100913	577 coding	noncoding	noncoding	noncoding
MSTRG.98696.1	CYTH3	XLOC_100914	244 coding	noncoding	noncoding	noncoding
MSTRG.98701.1	CYTH3	XLOC_100919	250 coding	noncoding	noncoding	noncoding
MSTRG.98660.1		XLOC_100930	282 coding	noncoding	noncoding	noncoding
MSTRG.98722.1		XLOC_100932	218 coding	noncoding	noncoding	noncoding
MSTRG.98678.1		XLOC_100936	257 coding	noncoding	noncoding	noncoding
XR_927004.2	LOC101927325	XLOC_100940	1556 coding	noncoding	noncoding	noncoding
MSTRG.98718.1	GRID2IP	XLOC_100941	278 coding	noncoding	noncoding	noncoding
MSTRG.98719.1	GRID2IP	XLOC_100942	284 coding	noncoding	noncoding	noncoding
MSTRG.98720.1	GRID2IP	XLOC_100943	240 coding	noncoding	noncoding	noncoding
MSTRG.98755.1		XLOC_100957	225 coding	noncoding	noncoding	noncoding
MSTRG.98764.1		XLOC_100961	235 coding	noncoding	noncoding	noncoding
MSTRG.98769.1		XLOC_100963	281 coding	coding	noncoding	noncoding
MSTRG.98771.1		XLOC_100964	275 coding	noncoding	noncoding	noncoding
MSTRG.98793.1	LOC100131257	XLOC_100971	230 coding	noncoding	noncoding	noncoding
MSTRG.98901.1	LOC105375144	XLOC_101140	220 coding	noncoding	noncoding	noncoding

MSTRG.98892.1		XLOC_101145	219 coding	noncoding	noncoding	noncoding
MSTRG.98912.1		XLOC_101147	276 coding	noncoding	noncoding	noncoding
MSTRG.98923.1		XLOC_101151	237 coding	coding	noncoding	noncoding
MSTRG.98932.1		XLOC_101157	267 coding	noncoding	noncoding	noncoding
MSTRG.98939.1		XLOC_101168	251 coding	noncoding	noncoding	noncoding
MSTRG.98940.1		XLOC_101169	432 coding	noncoding	noncoding	noncoding
MSTRG.99003.1	LOC105375149	XLOC_101172	362 coding	noncoding	noncoding	noncoding
MSTRG.99005.1	LOC105375149	XLOC_101173	209 coding	noncoding	noncoding	noncoding
MSTRG.99006.1	LOC105375149	XLOC_101174	396 coding	noncoding	noncoding	noncoding
MSTRG.99020.1	MGC4859	XLOC_101189	275 coding	noncoding	noncoding	noncoding
NM_001007157.2	PHF14	XLOC_101202	3427 coding	coding	coding	coding
MSTRG.99045.1		XLOC_101226	333 coding	noncoding	noncoding	noncoding
MSTRG.99050.1		XLOC_101228	243 coding	noncoding	noncoding	noncoding
MSTRG.99077.1	LOC105375153	XLOC_101230	406 coding	noncoding	noncoding	noncoding
MSTRG.99181.1		XLOC_101290	231 coding	noncoding	noncoding	noncoding
MSTRG.99187.1	LOC102725191	XLOC_101294	247 coding	noncoding	noncoding	noncoding
MSTRG.99225.1		XLOC_101304	303 coding	noncoding	coding	noncoding
MSTRG.99244.1	LOC105375158	XLOC_101314	287 coding	noncoding	noncoding	noncoding
MSTRG.99258.1	LOC107986770	XLOC_101324	683 coding	noncoding	noncoding	noncoding
MSTRG.99272.1	LOC107986770	XLOC_101329	202 coding	noncoding	noncoding	noncoding
MSTRG.99277.1		XLOC_101336	260 coding	coding	noncoding	noncoding
MSTRG.99289.1	DGKB	XLOC_101338	250 coding	noncoding	noncoding	noncoding
MSTRG.99305.1	DGKB	XLOC_101348	317 coding	noncoding	noncoding	noncoding
MSTRG.99378.1	AGMO	XLOC_101353	320 coding	noncoding	noncoding	noncoding
MSTRG.99394.1	AGMO	XLOC_101366	794 coding	noncoding	noncoding	noncoding
MSTRG.99321.1	LOC105375167	XLOC_101390	246 coding	noncoding	noncoding	noncoding
MSTRG.99336.1		XLOC_101406	346 coding	noncoding	noncoding	noncoding
MSTRG.99370.1		XLOC_101420	227 coding	noncoding	noncoding	noncoding
MSTRG.99372.1		XLOC_101422	290 coding	noncoding	noncoding	noncoding
MSTRG.99483.1		XLOC_101471	217 coding	noncoding	noncoding	noncoding
MSTRG.99709.4	SNX13	XLOC_101511	4876 coding	coding	noncoding	noncoding
MSTRG.99709.15	SNX13	XLOC_101511	486 coding	noncoding	noncoding	noncoding
MSTRG.99571.1		XLOC_101512	313 coding	coding	noncoding	noncoding
MSTRG.99574.1		XLOC_101514	413 coding	noncoding	noncoding	noncoding
MSTRG.99743.1		XLOC_101591	307 coding	noncoding	noncoding	noncoding
MSTRG.99775.1		XLOC_101611	249 coding	noncoding	noncoding	noncoding
MSTRG.99790.1		XLOC_101614	277 coding	noncoding	noncoding	noncoding
MSTRG.99807.1	LOC101927668	XLOC_101622	240 coding	noncoding	noncoding	noncoding
XM_011515393.2	ITGB8	XLOC_101635	8575 coding	coding	coding	coding
MSTRG.99869.6	ABCB5	XLOC_101657	257 coding	noncoding	noncoding	noncoding
MSTRG.99883.1		XLOC_101670	217 coding	noncoding	noncoding	noncoding
MSTRG.99889.1		XLOC_101686	276 coding	noncoding	noncoding	noncoding
MSTRG.100053.3	FAM126A	XLOC_101774	3536 coding	coding	noncoding	noncoding

MSTRG.100083.1	KLHL7	XLOC_101784	214	coding	noncoding	noncoding	noncoding
MSTRG.100066.1		XLOC_101787	292	coding	noncoding	noncoding	noncoding
MSTRG.100069.1	GPNMB	XLOC_101790	316	coding	noncoding	noncoding	noncoding
MSTRG.100092.1	IGF2BP3	XLOC_101793	217	coding	noncoding	noncoding	noncoding
MSTRG.100094.1		XLOC_101800	488	coding	noncoding	noncoding	noncoding
MSTRG.100095.1		XLOC_101801	280	coding	noncoding	noncoding	noncoding
MSTRG.100126.1	STK31	XLOC_101808	239	coding	noncoding	noncoding	noncoding
MSTRG.100128.1	STK31	XLOC_101810	291	coding	noncoding	noncoding	noncoding
MSTRG.100131.1	STK31	XLOC_101811	219	coding	noncoding	noncoding	noncoding
MSTRG.100119.1		XLOC_101816	292	coding	coding	noncoding	noncoding
XM_005249775.5	MPP6	XLOC_101826	8127	coding	coding	coding	coding
MSTRG.100170.1	GSDME	XLOC_101835	229	coding	noncoding	noncoding	noncoding
MSTRG.100174.1		XLOC_101844	305	coding	noncoding	noncoding	noncoding
MSTRG.100176.1		XLOC_101845	283	coding	noncoding	noncoding	noncoding
MSTRG.100209.1	C7orf31	XLOC_101853	267	coding	noncoding	noncoding	noncoding
MSTRG.100191.1	LOC105375191	XLOC_101857	275	coding	noncoding	noncoding	noncoding
MSTRG.100216.1		XLOC_101869	262	coding	noncoding	noncoding	noncoding
MSTRG.100243.1	LOC646588	XLOC_101885	287	coding	noncoding	noncoding	noncoding
MSTRG.100254.1	LOC105375199	XLOC_101892	215	coding	noncoding	noncoding	noncoding
MSTRG.100255.1	LOC105375199	XLOC_101893	297	coding	noncoding	noncoding	noncoding
MSTRG.100263.1	LOC105375199	XLOC_101895	250	coding	noncoding	noncoding	noncoding
MSTRG.100295.1		XLOC_101903	261	coding	noncoding	noncoding	noncoding
XM_017012086.1	SNX10	XLOC_101904	3232	coding	coding	coding	coding
XM_006715712.2	SNX10	XLOC_101904	2778	coding	coding	coding	coding
XM_017012809.1	CREB5	XLOC_102002	8298	coding	coding	coding	coding
XM_017012807.1	CREB5	XLOC_102002	9385	coding	coding	coding	coding
MSTRG.100680.1	CREB5	XLOC_102003	283	coding	noncoding	noncoding	noncoding
MSTRG.100685.1	CREB5	XLOC_102005	296	coding	noncoding	noncoding	noncoding
MSTRG.100863.1		XLOC_102132	259	coding	coding	noncoding	noncoding
MSTRG.100867.1		XLOC_102134	220	coding	noncoding	noncoding	noncoding
MSTRG.100906.1	INMT-MINDY4	XLOC_102158	585	coding	noncoding	noncoding	noncoding
MSTRG.100922.1	LOC107986781	XLOC_102171	228	coding	coding	noncoding	noncoding
MSTRG.100923.1	LOC107986781	XLOC_102172	273	coding	noncoding	noncoding	noncoding
MSTRG.100925.1		XLOC_102175	253	coding	noncoding	noncoding	noncoding
MSTRG.100926.1		XLOC_102176	284	coding	noncoding	noncoding	noncoding
MSTRG.100961.1	ITPRID1	XLOC_102180	301	coding	noncoding	noncoding	noncoding
MSTRG.101013.1		XLOC_102241	223	coding	noncoding	noncoding	noncoding
MSTRG.101335.1	BBS9	XLOC_102249	332	coding	coding	noncoding	noncoding
MSTRG.101348.1	BBS9	XLOC_102261	249	coding	noncoding	noncoding	noncoding
MSTRG.101353.1	BBS9	XLOC_102266	226	coding	noncoding	noncoding	noncoding
MSTRG.101166.1	BMPER	XLOC_102282	283	coding	noncoding	noncoding	noncoding
MSTRG.101191.1	NPSR1-AS1	XLOC_102291	281	coding	noncoding	noncoding	noncoding
MSTRG.101204.1	TBX20	XLOC_102307	320	coding	noncoding	noncoding	noncoding

MSTRG.101211.1	LOC401324	XLOC_102313	249	coding	noncoding	noncoding	noncoding
MSTRG.101214.1		XLOC_102316	390	coding	noncoding	noncoding	noncoding
MSTRG.101254.1	SEPTIN7-AS1	XLOC_102341	257	coding	noncoding	noncoding	noncoding
MSTRG.101297.10	SEPTIN7	XLOC_102350	4331	coding	coding	coding	coding
MSTRG.101293.1		XLOC_102356	281	coding	noncoding	noncoding	noncoding
MSTRG.101380.1	KIAA0895	XLOC_102384	254	coding	noncoding	noncoding	noncoding
MSTRG.101389.1	ANLN	XLOC_102389	255	coding	noncoding	noncoding	noncoding
MSTRG.101835.5	ELMO1-AS1	XLOC_102427	3550	coding	coding	coding	noncoding
MSTRG.101468.1		XLOC_102454	733	coding	noncoding	noncoding	noncoding
MSTRG.101666.9	TRGC2	XLOC_102459	1513	coding	coding	noncoding	noncoding
MSTRG.101666.10	TRGC2	XLOC_102459	3345	coding	coding	noncoding	noncoding
MSTRG.101666.17	TRGC2	XLOC_102459	1458	coding	coding	noncoding	noncoding
MSTRG.101666.21	TARP	XLOC_102459	1286	coding	coding	noncoding	noncoding
MSTRG.101666.23	TRGC2	XLOC_102459	1274	coding	coding	noncoding	noncoding
MSTRG.101666.24	TRGC2	XLOC_102459	1489	coding	coding	noncoding	noncoding
MSTRG.101666.26	TRGC2	XLOC_102459	2205	coding	coding	noncoding	noncoding
MSTRG.101519.1	POU6F2	XLOC_102500	473	coding	noncoding	coding	noncoding
MSTRG.101522.1	POU6F2	XLOC_102501	237	coding	noncoding	noncoding	noncoding
MSTRG.101504.1		XLOC_102506	215	coding	noncoding	noncoding	noncoding
MSTRG.101542.1		XLOC_102515	248	coding	noncoding	noncoding	noncoding
MSTRG.101586.1		XLOC_102521	301	coding	noncoding	noncoding	noncoding
MSTRG.101626.1	CDK13	XLOC_102549	339	coding	noncoding	coding	noncoding
MSTRG.101634.1		XLOC_102556	308	coding	noncoding	noncoding	noncoding
MSTRG.102055.1	SUGCT	XLOC_102574	226	coding	noncoding	noncoding	noncoding
MSTRG.102075.1	SUGCT	XLOC_102577	650	coding	noncoding	noncoding	noncoding
MSTRG.101652.1		XLOC_102582	253	coding	noncoding	noncoding	noncoding
MSTRG.101710.1		XLOC_102593	237	coding	noncoding	noncoding	noncoding
MSTRG.101735.1	LOC107986788	XLOC_102605	361	coding	noncoding	noncoding	noncoding
XM_011515224.2	HECW1	XLOC_102652	9496	coding	coding	coding	coding
MSTRG.102121.1	HECW1	XLOC_102658	216	coding	noncoding	noncoding	noncoding
MSTRG.102190.12	COA1	XLOC_102660	840	coding	coding	noncoding	noncoding
MSTRG.102129.1	LOC102724946	XLOC_102663	561	coding	noncoding	noncoding	noncoding
MSTRG.102123.1		XLOC_102665	652	coding	noncoding	noncoding	noncoding
MSTRG.102144.1	URGCP	XLOC_102668	494	coding	coding	noncoding	noncoding
MSTRG.102153.1	POLR2J4	XLOC_102675	280	coding	noncoding	noncoding	noncoding
NM_001129.5	AEBP1	XLOC_102685	4097	coding	coding	coding	coding
MSTRG.102181.2	POLD2	XLOC_102687	1602	coding	coding	coding	noncoding
MSTRG.102197.1	CAMK2B	XLOC_102695	230	coding	noncoding	noncoding	noncoding
MSTRG.102186.1		XLOC_102697	260	coding	noncoding	noncoding	noncoding
MSTRG.102252.1	OGDH	XLOC_102712	234	coding	noncoding	noncoding	noncoding
MSTRG.102230.1		XLOC_102719	287	coding	noncoding	noncoding	noncoding
MSTRG.102289.1		XLOC_102752	313	coding	noncoding	noncoding	noncoding
MSTRG.102296.1		XLOC_102756	250	coding	noncoding	noncoding	noncoding

MSTRG.102299.1		XLOC_102757	225 coding	noncoding	noncoding	noncoding
MSTRG.102415.1		XLOC_102785	247 coding	noncoding	noncoding	noncoding
MSTRG.102420.1		XLOC_102787	273 coding	noncoding	noncoding	noncoding
MSTRG.102447.1		XLOC_102801	502 coding	noncoding	noncoding	noncoding
MSTRG.102511.2	TNS3	XLOC_102814	369 coding	noncoding	noncoding	noncoding
NM_001123065.1	C7orf65	XLOC_102820	2911 coding	noncoding	coding	noncoding
MSTRG.102476.1	PKD1L1	XLOC_102827	299 coding	noncoding	noncoding	noncoding
MSTRG.102478.1	PKD1L1	XLOC_102829	227 coding	noncoding	noncoding	noncoding
MSTRG.102481.1	PKD1L1	XLOC_102832	245 coding	noncoding	noncoding	noncoding
MSTRG.102472.1		XLOC_102848	250 coding	noncoding	noncoding	noncoding
MSTRG.102520.1		XLOC_102852	457 coding	noncoding	noncoding	noncoding
MSTRG.102521.1		XLOC_102853	278 coding	coding	noncoding	noncoding
MSTRG.102530.1		XLOC_102870	263 coding	noncoding	noncoding	noncoding
MSTRG.102554.1		XLOC_102884	279 coding	noncoding	noncoding	noncoding
NM_198570.5	VWC2	XLOC_102904	11322 coding	coding	coding	coding
MSTRG.102607.1	SPATA48	XLOC_102908	257 coding	noncoding	noncoding	noncoding
MSTRG.102591.1		XLOC_102913	289 coding	noncoding	noncoding	noncoding
MSTRG.102592.1		XLOC_102914	323 coding	noncoding	noncoding	noncoding
MSTRG.102671.1	DDC	XLOC_102965	223 coding	noncoding	noncoding	noncoding
MSTRG.102714.1	COBL	XLOC_103008	221 coding	noncoding	noncoding	noncoding
MSTRG.102739.1		XLOC_103027	235 coding	noncoding	noncoding	noncoding
MSTRG.102819.1		XLOC_103038	289 coding	noncoding	noncoding	noncoding
MSTRG.102815.1		XLOC_103042	240 coding	noncoding	noncoding	noncoding
MSTRG.102822.1		XLOC_103051	230 coding	coding	noncoding	noncoding
MSTRG.102826.1		XLOC_103053	287 coding	noncoding	noncoding	noncoding
MSTRG.102838.1	LINC01446	XLOC_103057	273 coding	noncoding	noncoding	noncoding
MSTRG.102842.1		XLOC_103062	339 coding	noncoding	noncoding	noncoding
MSTRG.102843.1		XLOC_103063	323 coding	noncoding	noncoding	noncoding
XM_017011841.1	VSTM2A	XLOC_103068	9245 coding	coding	coding	coding
MSTRG.102864.1		XLOC_103082	271 coding	noncoding	noncoding	noncoding
MSTRG.102887.1		XLOC_103094	287 coding	noncoding	noncoding	noncoding
MSTRG.102952.1		XLOC_103111	242 coding	noncoding	noncoding	noncoding
MSTRG.102954.1		XLOC_103112	214 coding	noncoding	noncoding	noncoding
MSTRG.102956.1	LOC101060341	XLOC_103113	338 coding	coding	noncoding	noncoding
MSTRG.102959.1		XLOC_103115	312 coding	noncoding	noncoding	noncoding
MSTRG.102962.1	SEPTIN14	XLOC_103118	269 coding	noncoding	noncoding	noncoding
NM_182633.3	ZNF713	XLOC_103119	4358 coding	coding	coding	coding
MSTRG.102971.3	NIPSNAP2	XLOC_103121	536 coding	coding	noncoding	noncoding
NM_001366648.2	SUMF2	XLOC_103132	2108 coding	coding	coding	coding
NM_001146333.2	SUMF2	XLOC_103132	1835 coding	coding	coding	coding
MSTRG.103040.1	PHKG1	XLOC_103134	255 coding	noncoding	noncoding	noncoding
MSTRG.103013.1		XLOC_103137	385 coding	noncoding	noncoding	noncoding
MSTRG.103021.1	LOC650226	XLOC_103140	325 coding	coding	noncoding	noncoding

MSTRG.103022.1	LOC650226	XLOC_103141	287 coding	noncoding	noncoding	noncoding
MSTRG.103041.1		XLOC_103149	259 coding	noncoding	noncoding	noncoding
MSTRG.103052.1		XLOC_103150	318 coding	coding	noncoding	noncoding
MSTRG.103053.1		XLOC_103152	239 coding	noncoding	noncoding	noncoding
MSTRG.103059.1	ZNF479	XLOC_103155	324 coding	noncoding	noncoding	noncoding
MSTRG.103062.1	GUSBP10	XLOC_103157	281 coding	noncoding	coding	noncoding
MSTRG.103065.1		XLOC_103161	279 coding	noncoding	noncoding	noncoding
NR_120505.1	LOC100653233	XLOC_103163	1569 coding	noncoding	noncoding	noncoding
MSTRG.103089.1		XLOC_103175	234 coding	noncoding	noncoding	noncoding
MSTRG.103109.1		XLOC_103187	294 coding	noncoding	noncoding	noncoding
MSTRG.103158.1		XLOC_103213	318 coding	noncoding	noncoding	noncoding
MSTRG.103164.1		XLOC_103215	360 coding	noncoding	noncoding	noncoding
MSTRG.103171.1		XLOC_103220	261 coding	noncoding	noncoding	noncoding
MSTRG.103172.1		XLOC_103221	278 coding	noncoding	noncoding	noncoding
MSTRG.103175.1		XLOC_103223	242 coding	noncoding	noncoding	noncoding
MSTRG.103182.1		XLOC_103231	251 coding	noncoding	noncoding	noncoding
MSTRG.103186.1		XLOC_103236	297 coding	noncoding	noncoding	noncoding
MSTRG.103244.1		XLOC_103245	275 coding	noncoding	noncoding	noncoding
MSTRG.103267.1		XLOC_103264	265 coding	noncoding	noncoding	noncoding
NR_077227.1	LOC100128885	XLOC_103280	4369 coding	noncoding	noncoding	coding
MSTRG.103204.1		XLOC_103293	215 coding	noncoding	noncoding	noncoding
MSTRG.103214.1	ZNF138	XLOC_103298	284 coding	noncoding	noncoding	noncoding
MSTRG.103217.1	ZNF138	XLOC_103301	312 coding	noncoding	noncoding	noncoding
MSTRG.103206.1	ZNF138	XLOC_103303	222 coding	noncoding	noncoding	noncoding
MSTRG.103359.5	ZNF117	XLOC_103315	14335 coding	coding	coding	noncoding
MSTRG.103359.9	ZNF117	XLOC_103315	7374 coding	coding	noncoding	noncoding
MSTRG.103383.1		XLOC_103334	218 coding	noncoding	noncoding	noncoding
MSTRG.103385.1	LOC105375334	XLOC_103336	255 coding	noncoding	noncoding	noncoding
MSTRG.103324.1	VKORC1L1	XLOC_103361	231 coding	noncoding	noncoding	noncoding
MSTRG.103338.1	CRCP	XLOC_103367	481 coding	noncoding	noncoding	noncoding
MSTRG.103331.1		XLOC_103372	206 coding	noncoding	noncoding	noncoding
NM_001367755.1	RABGEF1	XLOC_103402	4781 coding	coding	coding	coding
MSTRG.103590.1	GTF2IP23	XLOC_103405	255 coding	noncoding	noncoding	noncoding
MSTRG.103492.1	TYW1	XLOC_103412	259 coding	noncoding	noncoding	noncoding
MSTRG.103399.1		XLOC_103436	238 coding	noncoding	noncoding	noncoding
MSTRG.103400.1		XLOC_103437	249 coding	noncoding	noncoding	noncoding
MSTRG.103420.1		XLOC_103448	240 coding	noncoding	noncoding	noncoding
MSTRG.103422.1		XLOC_103449	206 coding	noncoding	noncoding	noncoding
MSTRG.103426.1		XLOC_103451	295 coding	noncoding	noncoding	noncoding
MSTRG.103430.1		XLOC_103452	265 coding	noncoding	noncoding	noncoding
MSTRG.103432.1		XLOC_103457	307 coding	noncoding	noncoding	noncoding
MSTRG.103444.1		XLOC_103460	217 coding	noncoding	noncoding	noncoding
MSTRG.103454.1		XLOC_103466	223 coding	noncoding	noncoding	noncoding

MSTRG.103458.1		XLOC_103467	237	coding	noncoding	noncoding	noncoding
MSTRG.103476.1		XLOC_103475	229	coding	noncoding	noncoding	noncoding
MSTRG.103478.1		XLOC_103476	566	coding	noncoding	noncoding	noncoding
MSTRG.103480.1		XLOC_103477	262	coding	noncoding	noncoding	noncoding
MSTRG.103483.1	LOC105375346	XLOC_103479	255	coding	noncoding	noncoding	noncoding
XM_017011951.2	AUTS2	XLOC_103484	6684	coding	coding	coding	coding
MSTRG.104011.1	AUTS2	XLOC_103486	224	coding	noncoding	noncoding	noncoding
MSTRG.104046.1	AUTS2	XLOC_103493	225	coding	noncoding	noncoding	noncoding
MSTRG.104062.1	AUTS2	XLOC_103498	277	coding	noncoding	noncoding	noncoding
MSTRG.104083.1	AUTS2	XLOC_103501	250	coding	noncoding	noncoding	noncoding
MSTRG.104133.1	AUTS2	XLOC_103505	328	coding	noncoding	noncoding	noncoding
MSTRG.103597.1		XLOC_103516	466	coding	noncoding	noncoding	noncoding
MSTRG.103599.1		XLOC_103517	429	coding	noncoding	noncoding	noncoding
MSTRG.103601.1		XLOC_103518	217	coding	noncoding	noncoding	noncoding
MSTRG.103606.1		XLOC_103521	273	coding	noncoding	noncoding	noncoding
NM_022479.3	GALNT17	XLOC_103523	3909	coding	coding	coding	coding
MSTRG.103616.1	GALNT17	XLOC_103524	648	coding	noncoding	noncoding	noncoding
MSTRG.103629.1	GALNT17	XLOC_103527	260	coding	noncoding	noncoding	noncoding
MSTRG.103609.1		XLOC_103530	295	coding	noncoding	noncoding	noncoding
MSTRG.103640.1	CALN1	XLOC_103531	290	coding	noncoding	noncoding	noncoding
MSTRG.103643.1	CALN1	XLOC_103533	342	coding	noncoding	noncoding	noncoding
MSTRG.103653.1	CALN1	XLOC_103538	441	coding	noncoding	noncoding	noncoding
MSTRG.103659.1	CALN1	XLOC_103543	261	coding	noncoding	noncoding	noncoding
MSTRG.103611.1		XLOC_103546	301	coding	noncoding	noncoding	noncoding
MSTRG.103667.1	SBDSP1	XLOC_103556	269	coding	noncoding	noncoding	noncoding
MSTRG.103664.1		XLOC_103557	248	coding	noncoding	noncoding	noncoding
MSTRG.103811.1	TRIM74	XLOC_103564	221	coding	noncoding	noncoding	noncoding
MSTRG.103690.1		XLOC_103571	228	coding	noncoding	noncoding	noncoding
MSTRG.103695.1		XLOC_103576	224	coding	noncoding	noncoding	noncoding
MSTRG.103705.1		XLOC_103577	283	coding	noncoding	noncoding	noncoding
MSTRG.103739.1	STX1A	XLOC_103589	266	coding	noncoding	noncoding	noncoding
MSTRG.103717.1	CLDN3	XLOC_103595	216	coding	coding	noncoding	noncoding
MSTRG.103719.1		XLOC_103596	433	coding	noncoding	noncoding	noncoding
MSTRG.103721.1		XLOC_103601	237	coding	noncoding	noncoding	noncoding
MSTRG.103728.1		XLOC_103602	296	coding	noncoding	noncoding	noncoding
MSTRG.103730.1		XLOC_103603	287	coding	noncoding	noncoding	noncoding
MSTRG.103752.1		XLOC_103604	225	coding	noncoding	coding	noncoding
MSTRG.103754.1		XLOC_103606	295	coding	noncoding	noncoding	noncoding
MSTRG.103747.1		XLOC_103607	288	coding	noncoding	noncoding	noncoding
MSTRG.103766.1		XLOC_103615	261	coding	noncoding	noncoding	noncoding
NM_032421.3	CLIP2	XLOC_103617	5518	coding	coding	coding	coding
MSTRG.103791.1	GTF2IRD1	XLOC_103620	238	coding	noncoding	coding	noncoding
MSTRG.103796.1	GTF2IRD1	XLOC_103622	346	coding	noncoding	noncoding	noncoding

MSTRG.103843.1		XLOC_103643	282	coding	noncoding	noncoding	noncoding
NM_001145064.3	CASTOR2	XLOC_103646	8100	coding	coding	coding	coding
MSTRG.103856.1	CASTOR2	XLOC_103648	241	coding	noncoding	noncoding	noncoding
MSTRG.103920.1	HIP1	XLOC_103678	238	coding	noncoding	noncoding	noncoding
MSTRG.103933.1	HIP1	XLOC_103688	247	coding	noncoding	noncoding	noncoding
MSTRG.103945.1	HIP1	XLOC_103698	356	coding	noncoding	noncoding	noncoding
MSTRG.103905.1		XLOC_103699	284	coding	noncoding	noncoding	noncoding
MSTRG.103909.1		XLOC_103702	494	coding	noncoding	noncoding	noncoding
XM_024446844.1	RHBDD2	XLOC_103703	2621	coding	coding	coding	coding
MSTRG.103948.1		XLOC_103716	219	coding	noncoding	noncoding	noncoding
MSTRG.103954.1		XLOC_103717	212	coding	noncoding	noncoding	noncoding
MSTRG.103959.1	SRRM3	XLOC_103721	347	coding	noncoding	noncoding	noncoding
MSTRG.103962.1	SRRM3	XLOC_103723	333	coding	noncoding	noncoding	noncoding
MSTRG.103963.1	SRRM3	XLOC_103724	233	coding	noncoding	noncoding	noncoding
XM_017011729.2	DTX2	XLOC_103730	2064	coding	coding	coding	coding
MSTRG.104736.1	DTX2	XLOC_103731	291	coding	noncoding	noncoding	noncoding
MSTRG.104754.1	LOC100133091	XLOC_103745	270	coding	noncoding	noncoding	noncoding
MSTRG.104766.1		XLOC_103749	392	coding	noncoding	noncoding	noncoding
MSTRG.104768.1		XLOC_103750	253	coding	noncoding	noncoding	noncoding
NM_001127360.1	PHTF2	XLOC_103823	2625	coding	coding	noncoding	coding
MSTRG.104361.1	MAGI2	XLOC_103839	308	coding	noncoding	noncoding	noncoding
MSTRG.104367.1	MAGI2	XLOC_103843	294	coding	noncoding	noncoding	noncoding
MSTRG.104382.1	MAGI2	XLOC_103851	279	coding	noncoding	coding	noncoding
MSTRG.104305.1		XLOC_103869	285	coding	noncoding	noncoding	noncoding
MSTRG.104430.1		XLOC_103900	350	coding	noncoding	noncoding	noncoding
MSTRG.104439.1		XLOC_103904	460	coding	noncoding	coding	coding
MSTRG.104526.1	CACNA2D1	XLOC_103928	445	coding	noncoding	noncoding	noncoding
MSTRG.104530.1	CACNA2D1	XLOC_103930	261	coding	noncoding	noncoding	noncoding
MSTRG.104500.1		XLOC_103933	288	coding	noncoding	noncoding	noncoding
MSTRG.104508.1		XLOC_103938	277	coding	coding	noncoding	noncoding
MSTRG.104575.1		XLOC_103971	268	coding	coding	noncoding	noncoding
MSTRG.104607.1		XLOC_103980	343	coding	noncoding	noncoding	noncoding
MSTRG.104629.1		XLOC_103986	315	coding	noncoding	noncoding	noncoding
XM_017012865.2	DMTF1	XLOC_104030	5077	coding	coding	coding	coding
MSTRG.105038.1	ABCB4	XLOC_104076	391	coding	coding	noncoding	noncoding
XM_006716028.4	ADAM22	XLOC_104084	6906	coding	coding	coding	coding
MSTRG.104856.1		XLOC_104095	288	coding	noncoding	noncoding	noncoding
MSTRG.104885.1		XLOC_104109	231	coding	noncoding	noncoding	noncoding
MSTRG.104951.1	ZNF804B	XLOC_104119	223	coding	noncoding	noncoding	noncoding
MSTRG.104954.1	ZNF804B	XLOC_104121	281	coding	noncoding	noncoding	noncoding
MSTRG.104956.1	ZNF804B	XLOC_104123	253	coding	coding	noncoding	noncoding
MSTRG.104960.1		XLOC_104126	215	coding	noncoding	noncoding	noncoding
MSTRG.104968.1		XLOC_104130	222	coding	noncoding	noncoding	noncoding

MSTRG.104962.1		XLOC_104131	209 coding	noncoding	noncoding	noncoding
MSTRG.104975.1	STEAP2-AS1	XLOC_104132	310 coding	noncoding	noncoding	noncoding
MSTRG.105075.1		XLOC_104155	359 coding	noncoding	noncoding	noncoding
MSTRG.104997.1		XLOC_104159	200 coding	noncoding	noncoding	noncoding
MSTRG.105002.1		XLOC_104160	316 coding	noncoding	noncoding	noncoding
NM_012395.3	CDK14	XLOC_104163	4954 coding	coding	coding	coding
NM_003505.2	FZD1	XLOC_104216	6894 coding	coding	coding	coding
MSTRG.105107.1		XLOC_104228	237 coding	noncoding	noncoding	noncoding
MSTRG.105234.1	LRRD1	XLOC_104274	323 coding	noncoding	noncoding	noncoding
MSTRG.105224.1		XLOC_104282	285 coding	noncoding	noncoding	noncoding
MSTRG.105227.1	TMBIM7P	XLOC_104283	401 coding	noncoding	noncoding	noncoding
MSTRG.105275.1		XLOC_104294	295 coding	noncoding	noncoding	noncoding
MSTRG.105276.1		XLOC_104295	259 coding	noncoding	noncoding	noncoding
MSTRG.105360.1		XLOC_104330	239 coding	noncoding	noncoding	noncoding
MSTRG.105468.1	LOC105375404	XLOC_104337	296 coding	noncoding	noncoding	noncoding
MSTRG.105398.1		XLOC_104361	295 coding	noncoding	noncoding	noncoding
XM_011516383.1	PPP1R9A	XLOC_104362	10625 coding	coding	coding	coding
MSTRG.105421.1	PPP1R9A	XLOC_104364	365 coding	noncoding	noncoding	noncoding
MSTRG.105422.1	PPP1R9A	XLOC_104365	233 coding	noncoding	noncoding	noncoding
MSTRG.105433.1	PPP1R9A	XLOC_104366	304 coding	noncoding	noncoding	noncoding
MSTRG.105439.1	LOC105375405	XLOC_104367	312 coding	noncoding	noncoding	noncoding
MSTRG.105411.1		XLOC_104374	242 coding	noncoding	noncoding	noncoding
MSTRG.105412.1		XLOC_104375	254 coding	noncoding	noncoding	noncoding
MSTRG.105414.1		XLOC_104376	257 coding	noncoding	noncoding	noncoding
NM_016116.3	ASB4	XLOC_104379	3784 coding	coding	coding	coding
MSTRG.105531.1		XLOC_104411	211 coding	noncoding	noncoding	noncoding
MSTRG.105537.1		XLOC_104413	239 coding	noncoding	noncoding	noncoding
MSTRG.105543.1		XLOC_104416	229 coding	noncoding	noncoding	noncoding
MSTRG.105552.1		XLOC_104424	276 coding	noncoding	noncoding	noncoding
MSTRG.105553.1		XLOC_104425	323 coding	noncoding	noncoding	noncoding
MSTRG.105563.1		XLOC_104433	264 coding	noncoding	noncoding	noncoding
MSTRG.105564.1		XLOC_104434	563 coding	noncoding	noncoding	noncoding
MSTRG.105573.1		XLOC_104435	288 coding	noncoding	noncoding	noncoding
MSTRG.105575.1		XLOC_104436	265 coding	noncoding	noncoding	noncoding
MSTRG.105576.1		XLOC_104437	287 coding	noncoding	noncoding	noncoding
MSTRG.105587.1	LOC105375416	XLOC_104441	227 coding	noncoding	noncoding	noncoding
MSTRG.105593.1	LOC105375416	XLOC_104444	281 coding	noncoding	noncoding	noncoding
MSTRG.105582.1		XLOC_104446	251 coding	noncoding	noncoding	noncoding
MSTRG.105597.1		XLOC_104448	300 coding	coding	noncoding	noncoding
MSTRG.105708.1	LMTK2	XLOC_104454	285 coding	noncoding	noncoding	noncoding
MSTRG.105723.1	TECPR1	XLOC_104469	2934 coding	noncoding	coding	noncoding
MSTRG.105729.1		XLOC_104473	297 coding	noncoding	noncoding	noncoding
MSTRG.105742.1	BAIAP2L1	XLOC_104479	875 coding	coding	noncoding	coding

MSTRG.105677.1		XLOC_104480	315 coding	noncoding	noncoding	noncoding
MSTRG.105681.1		XLOC_104481	397 coding	noncoding	noncoding	noncoding
MSTRG.105685.1		XLOC_104483	277 coding	noncoding	noncoding	noncoding
MSTRG.105694.1		XLOC_104490	260 coding	noncoding	noncoding	noncoding
MSTRG.105697.1		XLOC_104492	290 coding	noncoding	noncoding	noncoding
MSTRG.105786.1	TRRAP	XLOC_104498	255 coding	noncoding	noncoding	noncoding
XM_006715826.2	ARPC1B	XLOC_104511	1687 coding	coding	coding	coding
NM_001318162.1	CPSF4	XLOC_104513	1730 coding	coding	coding	coding
MSTRG.105782.2	ATP5MF-PTCD1	XLOC_104514	453 coding	coding	noncoding	noncoding
MSTRG.105766.1	ZKSCAN5	XLOC_104519	275 coding	noncoding	noncoding	noncoding
MSTRG.105841.1	CYP3A7-CYP3A51P	XLOC_104539	249 coding	noncoding	noncoding	noncoding
MSTRG.105829.1	CYP3A43	XLOC_104545	254 coding	noncoding	noncoding	noncoding
MSTRG.105858.1		XLOC_104557	251 coding	noncoding	noncoding	noncoding
MSTRG.105891.1	ZKSCAN1	XLOC_104562	301 coding	noncoding	noncoding	noncoding
NM_004722.4	AP4M1	XLOC_104567	3591 coding	coding	coding	coding
MSTRG.105903.2	CNPY4	XLOC_104568	510 coding	coding	noncoding	noncoding
MSTRG.105873.1		XLOC_104571	305 coding	noncoding	noncoding	noncoding
MSTRG.106043.1	STAG3L5P-PVRIG2P-PILRB	XLOC_104585	293 coding	noncoding	noncoding	noncoding
MSTRG.105912.1	LOC107986829	XLOC_104603	312 coding	noncoding	noncoding	noncoding
MSTRG.105914.1	LOC107986829	XLOC_104604	288 coding	noncoding	noncoding	noncoding
MSTRG.105907.1		XLOC_104605	229 coding	noncoding	noncoding	noncoding
MSTRG.105920.1	AGFG2	XLOC_104607	284 coding	noncoding	noncoding	noncoding
MSTRG.105944.1	PCOLCE-AS1	XLOC_104610	200 coding	noncoding	noncoding	noncoding
MSTRG.105933.1	LOC105375429	XLOC_104618	239 coding	noncoding	noncoding	noncoding
NM_005273.4	GNB2	XLOC_104619	1664 coding	coding	coding	coding
MSTRG.105953.1		XLOC_104625	232 coding	noncoding	noncoding	noncoding
NR_111917.1	ZAN	XLOC_104626	8734 coding	coding	noncoding	coding
MSTRG.105965.1	EPHB4	XLOC_104630	459 coding	coding	coding	noncoding
XM_006716055.3	SLC12A9	XLOC_104631	3020 coding	coding	coding	coding
MSTRG.105969.1	SLC12A9-AS1	XLOC_104632	228 coding	noncoding	noncoding	noncoding
MSTRG.105983.2	MUC12	XLOC_104643	435 coding	noncoding	noncoding	noncoding
NM_001040105.2	MUC17	XLOC_104645	14352 coding	coding	noncoding	coding
MSTRG.105999.1		XLOC_104646	253 coding	noncoding	noncoding	noncoding
MSTRG.105998.1		XLOC_104647	227 coding	noncoding	noncoding	noncoding
MSTRG.106004.1		XLOC_104651	361 coding	noncoding	noncoding	noncoding
MSTRG.106017.1		XLOC_104656	293 coding	noncoding	noncoding	noncoding
MSTRG.106026.1		XLOC_104663	440 coding	noncoding	noncoding	noncoding
MSTRG.106078.1	COL26A1	XLOC_104670	396 coding	noncoding	noncoding	noncoding
MSTRG.106084.1	COL26A1	XLOC_104674	259 coding	noncoding	noncoding	noncoding
MSTRG.106060.1		XLOC_104681	227 coding	noncoding	noncoding	noncoding
MSTRG.106061.1		XLOC_104684	303 coding	noncoding	noncoding	noncoding
MSTRG.106098.1	CUX1	XLOC_104691	215 coding	noncoding	noncoding	noncoding

MSTRG.106110.1	CUX1	XLOC_104703	260 coding	noncoding	noncoding	noncoding
MSTRG.106111.1	CUX1	XLOC_104704	232 coding	noncoding	noncoding	noncoding
MSTRG.106113.1	CUX1	XLOC_104705	253 coding	noncoding	noncoding	noncoding
MSTRG.106600.1	LOC107986717	XLOC_104713	294 coding	noncoding	noncoding	noncoding
MSTRG.106629.1	POLR2J3	XLOC_104724	648 coding	noncoding	noncoding	noncoding
MSTRG.106173.1	SLC26A5	XLOC_104765	254 coding	noncoding	noncoding	noncoding
MSTRG.106175.1	SLC26A5	XLOC_104767	345 coding	noncoding	noncoding	noncoding
MSTRG.106177.1	SLC26A5	XLOC_104769	265 coding	noncoding	noncoding	noncoding
MSTRG.106148.1	LHFPL3	XLOC_104795	270 coding	noncoding	noncoding	noncoding
MSTRG.106153.1	LHFPL3	XLOC_104797	267 coding	noncoding	noncoding	noncoding
MSTRG.106156.1	LHFPL3	XLOC_104798	203 coding	noncoding	noncoding	noncoding
MSTRG.106167.1	LHFPL3-AS2	XLOC_104805	282 coding	noncoding	noncoding	noncoding
XM_011516400.2	KMT2E	XLOC_104806	7985 coding	coding	coding	coding
MSTRG.106432.19	SRPK2	XLOC_104806	3115 coding	coding	noncoding	coding
MSTRG.106441.1	SRPK2	XLOC_104810	2973 coding	noncoding	noncoding	noncoding
MSTRG.106225.1		XLOC_104842	281 coding	noncoding	noncoding	noncoding
MSTRG.106229.1		XLOC_104845	298 coding	noncoding	noncoding	noncoding
MSTRG.106257.1		XLOC_104893	247 coding	noncoding	noncoding	noncoding
NM_002649.3	PIK3CG	XLOC_104894	7210 coding	coding	coding	coding
XM_011516056.3	GPR22	XLOC_104910	3587 coding	coding	noncoding	coding
MSTRG.106430.1	LAMB1	XLOC_104936	221 coding	noncoding	noncoding	noncoding
MSTRG.106469.1	LOC107986836	XLOC_104989	375 coding	noncoding	noncoding	noncoding
MSTRG.106472.1		XLOC_104990	281 coding	noncoding	noncoding	noncoding
MSTRG.106542.1		XLOC_104992	214 coding	noncoding	noncoding	noncoding
MSTRG.106584.1	LOC105375451	XLOC_105014	231 coding	noncoding	noncoding	noncoding
MSTRG.106591.1		XLOC_105127	292 coding	noncoding	noncoding	noncoding
MSTRG.106699.1		XLOC_105128	217 coding	noncoding	noncoding	noncoding
MSTRG.106730.1	LOC101928012	XLOC_105179	287 coding	noncoding	noncoding	noncoding
MSTRG.106776.1		XLOC_105197	234 coding	noncoding	noncoding	noncoding
MSTRG.106794.1		XLOC_105205	241 coding	coding	noncoding	noncoding
MSTRG.106853.1	FOXP2	XLOC_105216	263 coding	noncoding	noncoding	noncoding
MSTRG.106854.1	FOXP2	XLOC_105217	253 coding	noncoding	noncoding	noncoding
MSTRG.106830.1		XLOC_105231	309 coding	noncoding	noncoding	noncoding
MSTRG.106836.1		XLOC_105234	213 coding	noncoding	noncoding	noncoding
MSTRG.106837.1		XLOC_105235	260 coding	noncoding	noncoding	noncoding
MSTRG.106840.1		XLOC_105236	290 coding	noncoding	noncoding	noncoding
MSTRG.106950.1		XLOC_105264	324 coding	coding	noncoding	noncoding
MSTRG.106972.1		XLOC_105278	298 coding	noncoding	noncoding	noncoding
MSTRG.107009.1		XLOC_105282	255 coding	noncoding	noncoding	noncoding
MSTRG.107050.1	LOC105375463	XLOC_105317	323 coding	noncoding	noncoding	noncoding
NM_001206748.2	CAV2	XLOC_105325	2973 coding	coding	coding	noncoding
MSTRG.107092.1	MET	XLOC_105331	389 coding	noncoding	noncoding	noncoding
MSTRG.107214.1	ST7	XLOC_105354	307 coding	noncoding	noncoding	noncoding

MSTRG.107218.1	ST7	XLOC_105357	237	coding	noncoding	noncoding	noncoding
MSTRG.107136.1	CFTR-AS1	XLOC_105374	212	coding	noncoding	noncoding	noncoding
MSTRG.107192.1	LOC102724495	XLOC_105404	216	coding	noncoding	noncoding	noncoding
MSTRG.107195.1		XLOC_105406	223	coding	noncoding	noncoding	noncoding
XR_001745344.1	LOC105375472	XLOC_105408	7135	coding	noncoding	noncoding	noncoding
MSTRG.107239.1		XLOC_105418	241	coding	noncoding	noncoding	noncoding
MSTRG.107225.1		XLOC_105420	247	coding	noncoding	noncoding	noncoding
MSTRG.107247.1		XLOC_105426	305	coding	noncoding	coding	noncoding
MSTRG.107301.1	LINC02476	XLOC_105448	297	coding	noncoding	noncoding	noncoding
MSTRG.107271.1		XLOC_105452	292	coding	noncoding	noncoding	noncoding
MSTRG.107274.1		XLOC_105453	269	coding	noncoding	noncoding	noncoding
MSTRG.107279.1		XLOC_105456	204	coding	noncoding	noncoding	noncoding
XM_011516165.3	KCND2	XLOC_105461	5957	coding	coding	coding	coding
MSTRG.107311.1		XLOC_105473	262	coding	noncoding	noncoding	noncoding
MSTRG.107346.1		XLOC_105555	217	coding	noncoding	noncoding	noncoding
MSTRG.107382.1		XLOC_105571	322	coding	noncoding	noncoding	noncoding
NR_036484.1	FEZF1-AS1	XLOC_105575	2653	coding	noncoding	coding	noncoding
MSTRG.107692.1	CADPS2	XLOC_105583	231	coding	noncoding	noncoding	noncoding
MSTRG.107674.1		XLOC_105590	310	coding	coding	noncoding	noncoding
MSTRG.107710.1		XLOC_105594	226	coding	noncoding	noncoding	noncoding
MSTRG.107739.1	HYAL4	XLOC_105615	250	coding	noncoding	noncoding	noncoding
MSTRG.107743.1	HYAL4	XLOC_105617	272	coding	noncoding	noncoding	noncoding
NR_133947.1	LOC105375483	XLOC_105620	1741	coding	noncoding	noncoding	noncoding
MSTRG.107779.1		XLOC_105636	291	coding	noncoding	noncoding	noncoding
MSTRG.107877.1	GRM8	XLOC_105667	254	coding	noncoding	noncoding	noncoding
MSTRG.107881.1	GRM8	XLOC_105669	258	coding	noncoding	noncoding	noncoding
MSTRG.107847.1		XLOC_105685	206	coding	noncoding	noncoding	noncoding
MSTRG.107917.6	ZNF800	XLOC_105688	2262	coding	coding	coding	coding
MSTRG.107938.1		XLOC_105701	236	coding	noncoding	noncoding	noncoding
MSTRG.108058.4	LRRC4	XLOC_105702	2643	coding	coding	coding	coding
MSTRG.107973.1		XLOC_105726	291	coding	noncoding	noncoding	noncoding
MSTRG.108026.1		XLOC_105757	282	coding	noncoding	noncoding	noncoding
MSTRG.108029.1		XLOC_105759	214	coding	noncoding	noncoding	noncoding
NM_178562.5	TSPAN33	XLOC_105763	2951	coding	coding	coding	coding
XM_017011907.1	AHCYL2	XLOC_105766	4767	coding	coding	coding	coding
MSTRG.108146.1	AHCYL2	XLOC_105778	259	coding	noncoding	noncoding	noncoding
MSTRG.108150.1	AHCYL2	XLOC_105782	450	coding	noncoding	noncoding	noncoding
MSTRG.108158.1		XLOC_105788	288	coding	noncoding	noncoding	noncoding
MSTRG.108159.1		XLOC_105789	226	coding	noncoding	noncoding	noncoding
MSTRG.108161.1		XLOC_105790	656	coding	noncoding	noncoding	noncoding
MSTRG.108111.1		XLOC_105823	246	coding	noncoding	noncoding	noncoding
MSTRG.108112.1		XLOC_105824	233	coding	noncoding	noncoding	noncoding
MSTRG.108114.1	CPA2	XLOC_105826	245	coding	noncoding	noncoding	noncoding

MSTRG.108521.5	LINC-PINT	XLOC_105841	3105	coding	noncoding	noncoding	noncoding
MSTRG.108521.14	LINC-PINT	XLOC_105841	4322	coding	noncoding	coding	noncoding
MSTRG.108641.1	PODXL	XLOC_105896	330	coding	noncoding	noncoding	noncoding
MSTRG.108212.1		XLOC_105904	223	coding	coding	noncoding	noncoding
MSTRG.108314.1		XLOC_105979	255	coding	noncoding	noncoding	noncoding
MSTRG.108490.1	EXOC4	XLOC_106056	306	coding	coding	noncoding	noncoding
MSTRG.108387.1	AKR1B10	XLOC_106086	276	coding	noncoding	noncoding	noncoding
XR_927537.3	CALD1	XLOC_106105	5025	coding	coding	coding	coding
NM_001130929.2	STMP1	XLOC_106179	2461	coding	coding	coding	coding
MSTRG.108662.1	FAM180A	XLOC_106182	239	coding	noncoding	noncoding	noncoding
MSTRG.108663.1	FAM180A	XLOC_106183	235	coding	coding	noncoding	noncoding
MSTRG.108665.1	FAM180A	XLOC_106184	304	coding	noncoding	noncoding	noncoding
MSTRG.108784.1		XLOC_106215	229	coding	noncoding	noncoding	noncoding
MSTRG.108787.1		XLOC_106218	306	coding	noncoding	noncoding	noncoding
NM_001006628.2	CHRM2	XLOC_106221	5680	coding	coding	coding	coding
MSTRG.108794.1		XLOC_106229	649	coding	noncoding	noncoding	noncoding
MSTRG.108824.1	PTN	XLOC_106235	262	coding	noncoding	noncoding	noncoding
MSTRG.108841.1	DGKI	XLOC_106242	238	coding	noncoding	noncoding	noncoding
MSTRG.108854.1	DGKI	XLOC_106249	284	coding	noncoding	noncoding	noncoding
MSTRG.108870.1		XLOC_106265	285	coding	noncoding	noncoding	noncoding
MSTRG.108875.1	AKR1D1	XLOC_106267	237	coding	noncoding	noncoding	noncoding
MSTRG.108878.1		XLOC_106271	273	coding	noncoding	noncoding	noncoding
MSTRG.108882.1		XLOC_106273	526	coding	noncoding	noncoding	noncoding
MSTRG.108886.1		XLOC_106275	251	coding	noncoding	noncoding	noncoding
MSTRG.108890.1		XLOC_106277	267	coding	noncoding	noncoding	noncoding
MSTRG.108892.1		XLOC_106278	309	coding	noncoding	noncoding	noncoding
MSTRG.108940.1	TRIM24	XLOC_106281	445	coding	noncoding	noncoding	noncoding
MSTRG.108903.1		XLOC_106293	312	coding	noncoding	noncoding	noncoding
XM_005250167.3	TMEM213	XLOC_106295	4629	coding	coding	coding	coding
NM_001318333.2	TTC26	XLOC_106312	4460	coding	coding	coding	coding
XM_011516003.2	UBN2	XLOC_106313	6930	coding	coding	coding	coding
MSTRG.109064.1		XLOC_106327	323	coding	coding	noncoding	noncoding
MSTRG.109095.1		XLOC_106391	239	coding	noncoding	noncoding	noncoding
MSTRG.109103.1		XLOC_106397	533	coding	noncoding	noncoding	noncoding
MSTRG.109111.1	DENND2A	XLOC_106398	281	coding	noncoding	noncoding	noncoding
MSTRG.109120.1	LOC105375536	XLOC_106416	315	coding	noncoding	noncoding	noncoding
XM_011515705.2	TMEM178B	XLOC_106422	2972	coding	coding	coding	coding
NM_001195278.2	TMEM178B	XLOC_106422	10726	coding	coding	coding	coding
MSTRG.109164.1	TMEM178B	XLOC_106423	207	coding	noncoding	noncoding	noncoding
MSTRG.109170.1	TMEM178B	XLOC_106426	269	coding	noncoding	noncoding	noncoding
MSTRG.109176.1	TMEM178B	XLOC_106430	239	coding	noncoding	noncoding	noncoding
MSTRG.109231.1		XLOC_106447	313	coding	noncoding	noncoding	noncoding
MSTRG.109483.70	TRBJ1-2	XLOC_106467	765	coding	coding	coding	noncoding

NM_001143680.1	GSTK1	XLOC_106555	1022	coding	coding	coding	coding
MSTRG.109593.1		XLOC_106625	261	coding	noncoding	noncoding	noncoding
MSTRG.109603.1		XLOC_106628	303	coding	coding	noncoding	noncoding
MSTRG.109615.1		XLOC_106635	242	coding	noncoding	noncoding	noncoding
MSTRG.109668.1	CNTNAP2	XLOC_106654	499	coding	coding	noncoding	noncoding
MSTRG.109674.1	CNTNAP2	XLOC_106658	265	coding	coding	noncoding	noncoding
MSTRG.109682.1	CNTNAP2	XLOC_106662	573	coding	noncoding	noncoding	noncoding
MSTRG.109703.1	CNTNAP2	XLOC_106670	243	coding	noncoding	noncoding	noncoding
MSTRG.109632.1		XLOC_106684	220	coding	noncoding	noncoding	noncoding
MSTRG.109760.1	CUL1	XLOC_106698	288	coding	noncoding	noncoding	noncoding
MSTRG.109720.1		XLOC_106714	224	coding	noncoding	noncoding	noncoding
XM_011516439.2	ZNF398	XLOC_106734	5306	coding	coding	coding	coding
MSTRG.109908.5	ZNF767P	XLOC_106759	832	coding	coding	coding	noncoding
MSTRG.109901.4	ZNF467	XLOC_106785	1811	coding	coding	coding	coding
NM_138434.2	ZBED6CL	XLOC_106891	2873	coding	noncoding	coding	coding
XM_017012081.2	REPIN1	XLOC_106897	3718	coding	coding	coding	coding
MSTRG.110055.2	GIMAP6	XLOC_106922	5453	coding	coding	coding	coding
MSTRG.110055.5	GIMAP6	XLOC_106922	4366	coding	coding	coding	noncoding
XM_017011945.1	AOC1	XLOC_106943	2729	coding	coding	coding	coding
MSTRG.109991.1	LOC105375567	XLOC_106944	204	coding	noncoding	noncoding	noncoding
MSTRG.110003.1	KCNH2	XLOC_106952	284	coding	noncoding	coding	noncoding
MSTRG.110231.1		XLOC_106987	276	coding	noncoding	noncoding	noncoding
MSTRG.110495.4	KMT2C	XLOC_106999	17287	coding	coding	coding	coding
MSTRG.110268.1		XLOC_107004	398	coding	noncoding	noncoding	noncoding
MSTRG.110270.1		XLOC_107005	307	coding	noncoding	noncoding	noncoding
MSTRG.110283.1	XRCC2	XLOC_107010	258	coding	noncoding	noncoding	noncoding
MSTRG.110354.1	ACTR3B	XLOC_107014	270	coding	noncoding	noncoding	noncoding
MSTRG.110368.1	ACTR3B	XLOC_107018	279	coding	noncoding	noncoding	noncoding
MSTRG.110376.1	ACTR3B	XLOC_107020	252	coding	noncoding	noncoding	noncoding
MSTRG.110314.1		XLOC_107026	633	coding	noncoding	noncoding	noncoding
MSTRG.110341.1		XLOC_107036	272	coding	noncoding	noncoding	noncoding
NM_130797.4	DPP6	XLOC_107042	4826	coding	coding	coding	coding
MSTRG.110390.1	DPP6	XLOC_107047	300	coding	noncoding	noncoding	noncoding
MSTRG.110398.1	DPP6	XLOC_107049	258	coding	noncoding	noncoding	noncoding
MSTRG.110403.1	LOC105375582	XLOC_107052	294	coding	noncoding	noncoding	noncoding
MSTRG.110408.1	DPP6	XLOC_107055	259	coding	noncoding	noncoding	noncoding
MSTRG.110414.1		XLOC_107061	302	coding	noncoding	noncoding	noncoding
MSTRG.110424.1		XLOC_107064	283	coding	noncoding	noncoding	noncoding
NM_001346592.1	INSIG1	XLOC_107073	3082	coding	coding	coding	coding
MSTRG.110549.1		XLOC_107137	245	coding	noncoding	noncoding	noncoding
MSTRG.110559.1		XLOC_107146	216	coding	noncoding	noncoding	noncoding
MSTRG.110543.1		XLOC_107149	370	coding	noncoding	noncoding	noncoding
XM_017012818.1	UBE3C	XLOC_107174	5198	coding	coding	coding	coding

MSTRG.110652.1	DNAJB6	XLOC_107186	260 coding	noncoding	noncoding	noncoding
MSTRG.110628.1		XLOC_107193	308 coding	noncoding	noncoding	noncoding
MSTRG.110629.1		XLOC_107194	276 coding	noncoding	noncoding	noncoding
MSTRG.110632.1	LOC101927914	XLOC_107197	282 coding	noncoding	noncoding	noncoding
MSTRG.110786.1	WDR60	XLOC_107236	324 coding	noncoding	noncoding	noncoding
MSTRG.110804.1	VIPR2	XLOC_107245	307 coding	noncoding	noncoding	noncoding
MSTRG.110813.1		XLOC_107253	236 coding	noncoding	noncoding	noncoding
MSTRG.98008.1	PRKAR1B	XLOC_107270	285 coding	noncoding	noncoding	noncoding
MSTRG.98210.1	LOC102723758	XLOC_107309	244 coding	noncoding	noncoding	noncoding
MSTRG.98166.1		XLOC_107312	430 coding	noncoding	noncoding	noncoding
MSTRG.98224.1		XLOC_107329	201 coding	noncoding	noncoding	noncoding
XR_001745051.1	LNCRI	XLOC_107333	4542 coding	noncoding	coding	coding
MSTRG.98248.1	ELFN1	XLOC_107338	260 coding	noncoding	noncoding	noncoding
MSTRG.98235.1		XLOC_107339	243 coding	noncoding	noncoding	noncoding
MSTRG.98236.1		XLOC_107340	262 coding	noncoding	noncoding	noncoding
MSTRG.98291.1	MAD1L1	XLOC_107358	203 coding	noncoding	noncoding	noncoding
MSTRG.98263.1	SNX8	XLOC_107370	334 coding	noncoding	noncoding	noncoding
MSTRG.98265.1	SNX8	XLOC_107371	220 coding	noncoding	noncoding	noncoding
MSTRG.98309.2	LFNG	XLOC_107384	3485 coding	coding	coding	coding
MSTRG.98315.1		XLOC_107397	401 coding	noncoding	noncoding	noncoding
MSTRG.98344.1	GNA12	XLOC_107401	225 coding	noncoding	noncoding	noncoding
MSTRG.98330.1		XLOC_107403	290 coding	noncoding	noncoding	noncoding
MSTRG.98361.1		XLOC_107425	293 coding	noncoding	noncoding	noncoding
MSTRG.98362.1		XLOC_107426	287 coding	noncoding	noncoding	noncoding
MSTRG.98468.1	LOC105375132	XLOC_107430	305 coding	noncoding	noncoding	noncoding
MSTRG.98409.1	FOXK1	XLOC_107529	606 coding	noncoding	noncoding	noncoding
NM_018059.5	RADIL	XLOC_107537	5736 coding	coding	coding	coding
MSTRG.98463.1	ZNF890P	XLOC_107556	407 coding	noncoding	noncoding	noncoding
MSTRG.98445.1		XLOC_107557	431 coding	noncoding	noncoding	noncoding
MSTRG.98606.1	TNRC18	XLOC_107560	246 coding	noncoding	noncoding	noncoding
MSTRG.98614.1	TNRC18	XLOC_107561	229 coding	noncoding	noncoding	noncoding
MSTRG.98616.1	TNRC18	XLOC_107562	467 coding	noncoding	noncoding	noncoding
MSTRG.98639.1	FSCN1	XLOC_107577	305 coding	noncoding	noncoding	noncoding
MSTRG.98657.1	RNF216	XLOC_107593	231 coding	noncoding	noncoding	noncoding
MSTRG.98665.2	LOC107986694	XLOC_107601	1621 coding	coding	noncoding	noncoding
MSTRG.98673.2	EIF2AK1	XLOC_107604	2271 coding	coding	coding	noncoding
MSTRG.98636.1		XLOC_107605	274 coding	noncoding	noncoding	noncoding
MSTRG.98683.1	USP42	XLOC_107607	264 coding	noncoding	noncoding	noncoding
MSTRG.98729.1	DAGLB	XLOC_107619	301 coding	noncoding	noncoding	noncoding
MSTRG.98679.1		XLOC_107621	298 coding	noncoding	noncoding	noncoding
MSTRG.98680.1		XLOC_107622	224 coding	noncoding	noncoding	noncoding
MSTRG.98676.1		XLOC_107625	298 coding	noncoding	noncoding	noncoding
MSTRG.98748.1	ZNF316	XLOC_107633	1527 coding	coding	coding	noncoding

MSTRG.98754.1		XLOC_107641	225 coding	noncoding	noncoding	noncoding
MSTRG.98765.1		XLOC_107646	235 coding	noncoding	noncoding	noncoding
MSTRG.98768.1		XLOC_107648	281 coding	coding	noncoding	noncoding
MSTRG.98772.1		XLOC_107650	275 coding	noncoding	noncoding	noncoding
XR_002956536.1	LOC112267992	XLOC_107651	4423 coding	coding	coding	coding
NR_034022.1	LOC100131257	XLOC_107657	21017 coding	coding	coding	coding
MSTRG.98779.1		XLOC_107658	260 coding	noncoding	noncoding	noncoding
MSTRG.99672.3	GLCCI1	XLOC_107738	315 coding	coding	noncoding	noncoding
MSTRG.98913.1		XLOC_107764	276 coding	noncoding	noncoding	noncoding
MSTRG.98915.1		XLOC_107766	347 coding	noncoding	noncoding	noncoding
MSTRG.99004.1	LOC105375149	XLOC_107789	297 coding	noncoding	noncoding	noncoding
XR_001745089.1	LOC105375150	XLOC_107792	5824 coding	noncoding	noncoding	noncoding
MSTRG.99014.1	MGC4859	XLOC_107797	278 coding	noncoding	noncoding	noncoding
MSTRG.99030.1		XLOC_107799	256 coding	noncoding	noncoding	noncoding
MSTRG.99036.1	LOC107986767	XLOC_107803	326 coding	noncoding	noncoding	noncoding
MSTRG.99049.1		XLOC_107829	243 coding	noncoding	noncoding	noncoding
MSTRG.99088.1	THSD7A	XLOC_107837	300 coding	noncoding	noncoding	noncoding
MSTRG.99188.1	LOC102725191	XLOC_107872	260 coding	noncoding	noncoding	noncoding
MSTRG.99193.1	SCIN	XLOC_107875	223 coding	coding	noncoding	noncoding
MSTRG.99198.1	SCIN	XLOC_107878	316 coding	noncoding	noncoding	noncoding
MSTRG.99219.3	ARL4A	XLOC_107880	2606 coding	coding	noncoding	noncoding
MSTRG.99236.1		XLOC_107892	255 coding	noncoding	noncoding	noncoding
MSTRG.99259.1	LOC107986770	XLOC_107899	289 coding	noncoding	noncoding	noncoding
MSTRG.99268.1	LOC107986770	XLOC_107905	316 coding	noncoding	noncoding	noncoding
MSTRG.99273.1	LOC107986770	XLOC_107908	294 coding	noncoding	noncoding	noncoding
MSTRG.99308.1	DGKB	XLOC_107924	418 coding	noncoding	noncoding	noncoding
MSTRG.99379.1	AGMO	XLOC_107933	348 coding	noncoding	noncoding	noncoding
MSTRG.99318.1	LOC105375167	XLOC_107978	320 coding	noncoding	noncoding	noncoding
MSTRG.99322.1		XLOC_107979	316 coding	noncoding	noncoding	noncoding
MSTRG.99329.1		XLOC_107984	263 coding	noncoding	noncoding	noncoding
MSTRG.99330.1		XLOC_107985	275 coding	noncoding	noncoding	noncoding
MSTRG.99358.1	CRPPA	XLOC_107989	268 coding	noncoding	noncoding	noncoding
MSTRG.99359.1	CRPPA	XLOC_107990	273 coding	noncoding	noncoding	noncoding
MSTRG.99463.3	BZW2	XLOC_108005	1704 coding	coding	coding	noncoding
MSTRG.99504.1	KCCAT333	XLOC_108038	430 coding	coding	noncoding	noncoding
XR_001745108.1	LOC105375172	XLOC_108039	7094 coding	noncoding	noncoding	noncoding
XM_024446696.1	SNX13	XLOC_108053	7804 coding	coding	coding	coding
MSTRG.99788.1	LOC105379720	XLOC_108162	261 coding	noncoding	noncoding	noncoding
MSTRG.99782.1		XLOC_108163	278 coding	coding	noncoding	noncoding
MSTRG.99791.1		XLOC_108164	201 coding	noncoding	noncoding	noncoding
MSTRG.99792.1		XLOC_108173	322 coding	noncoding	noncoding	noncoding
MSTRG.99871.1	ABCB5	XLOC_108188	250 coding	noncoding	noncoding	noncoding
MSTRG.99861.1	LINC01162	XLOC_108194	477 coding	noncoding	noncoding	noncoding

MSTRG.99885.1		XLOC_108206	308	coding	noncoding	noncoding	noncoding
MSTRG.99890.1		XLOC_108214	276	coding	noncoding	noncoding	noncoding
MSTRG.99931.1	DNAH11	XLOC_108221	259	coding	noncoding	noncoding	noncoding
XR_927091.3	LOC102724143	XLOC_108229	4507	coding	noncoding	coding	noncoding
MSTRG.99959.1	RAPGEF5	XLOC_108236	290	coding	noncoding	noncoding	noncoding
NM_032581.4	FAM126A	XLOC_108261	13178	coding	coding	noncoding	coding
MSTRG.100035.1		XLOC_108273	231	coding	noncoding	noncoding	noncoding
MSTRG.100037.1		XLOC_108274	237	coding	noncoding	noncoding	noncoding
MSTRG.100039.1	LOC105375186	XLOC_108276	264	coding	noncoding	noncoding	noncoding
MSTRG.100071.1	NUP42	XLOC_108280	263	coding	noncoding	noncoding	noncoding
MSTRG.100067.1		XLOC_108287	319	coding	noncoding	noncoding	noncoding
MSTRG.100120.1		XLOC_108312	292	coding	coding	noncoding	noncoding
MSTRG.100190.1		XLOC_108368	275	coding	noncoding	noncoding	noncoding
MSTRG.100192.1		XLOC_108369	228	coding	noncoding	noncoding	noncoding
MSTRG.100217.1		XLOC_108377	262	coding	noncoding	noncoding	noncoding
MSTRG.100256.1	LOC105375199	XLOC_108391	281	coding	noncoding	noncoding	noncoding
NM_002137.4	HNRNPA2B1	XLOC_108399	3628	coding	coding	coding	coding
MSTRG.100477.1	SKAP2	XLOC_108456	283	coding	noncoding	noncoding	noncoding
MSTRG.100499.1		XLOC_108475	279	coding	noncoding	noncoding	noncoding
XM_011515343.3	HOXA3	XLOC_108483	7765	coding	coding	coding	coding
NM_000522.5	HOXA13	XLOC_108493	5015	coding	coding	coding	coding
MSTRG.100382.1	LOC107986733	XLOC_108497	210	coding	noncoding	noncoding	noncoding
MSTRG.100392.1		XLOC_108502	316	coding	noncoding	noncoding	noncoding
MSTRG.100681.1	CREB5	XLOC_108528	283	coding	noncoding	noncoding	noncoding
MSTRG.100683.1	CREB5	XLOC_108529	239	coding	noncoding	noncoding	noncoding
MSTRG.100686.1	CREB5	XLOC_108531	368	coding	noncoding	noncoding	noncoding
NM_014817.4	TRIL	XLOC_108539	4973	coding	coding	coding	coding
MSTRG.100506.1		XLOC_108540	208	coding	noncoding	noncoding	noncoding
NM_001145513.1	SCRN1	XLOC_108558	5276	coding	coding	coding	coding
MSTRG.100760.10	MTURN	XLOC_108570	5915	coding	coding	coding	noncoding
MSTRG.100927.1		XLOC_108667	284	coding	noncoding	noncoding	noncoding
MSTRG.100929.1		XLOC_108668	330	coding	noncoding	noncoding	noncoding
MSTRG.100962.1	ITPRID1	XLOC_108674	238	coding	noncoding	noncoding	noncoding
XR_001744805.1	PDE1C	XLOC_108675	9944	coding	coding	coding	coding
MSTRG.100973.1	PDE1C	XLOC_108681	308	coding	noncoding	noncoding	noncoding
MSTRG.100977.1	PDE1C	XLOC_108683	321	coding	noncoding	noncoding	noncoding
MSTRG.100982.1	PDE1C	XLOC_108687	279	coding	noncoding	noncoding	noncoding
MSTRG.100996.1	RP9P	XLOC_108722	238	coding	noncoding	noncoding	noncoding
MSTRG.100991.1		XLOC_108723	251	coding	noncoding	noncoding	noncoding
MSTRG.101331.10	BBS9	XLOC_108737	904	coding	coding	coding	noncoding
MSTRG.101165.1	BMPER	XLOC_108809	283	coding	noncoding	noncoding	noncoding
MSTRG.101186.1	NPSR1-AS1	XLOC_108825	304	coding	noncoding	noncoding	noncoding
MSTRG.101200.1		XLOC_108844	297	coding	noncoding	noncoding	noncoding

MSTRG.101207.1		XLOC_108848	274 coding	noncoding	noncoding	noncoding
XR_001744846.2	HERPUD2	XLOC_108858	3225 coding	coding	coding	coding
XM_017012106.1	AOAH	XLOC_108889	1871 coding	coding	coding	coding
XR_001744894.2	ELMO1	XLOC_108963	3611 coding	coding	coding	coding
MSTRG.101962.1		XLOC_109079	293 coding	noncoding	noncoding	noncoding
MSTRG.101465.1		XLOC_109100	251 coding	noncoding	noncoding	noncoding
MSTRG.101668.66	TRG-AS1	XLOC_109105	2203 coding	noncoding	noncoding	noncoding
MSTRG.101507.1	POU6F2	XLOC_109148	306 coding	noncoding	noncoding	noncoding
MSTRG.101543.1		XLOC_109171	263 coding	noncoding	noncoding	noncoding
MSTRG.101594.1		XLOC_109178	294 coding	noncoding	noncoding	noncoding
MSTRG.101643.1	MPLKIP	XLOC_109189	731 coding	noncoding	noncoding	noncoding
MSTRG.101986.1	SUGCT	XLOC_109191	257 coding	noncoding	noncoding	noncoding
MSTRG.101987.1	SUGCT	XLOC_109192	404 coding	noncoding	noncoding	noncoding
MSTRG.101708.1	LOC105375246	XLOC_109303	246 coding	noncoding	noncoding	noncoding
MSTRG.101738.1	LOC107986788	XLOC_109315	278 coding	noncoding	noncoding	noncoding
NM_000168.6	GLI3	XLOC_109316	8405 coding	coding	coding	coding
NR_110833.1	LINC01448	XLOC_109343	1171 coding	noncoding	noncoding	noncoding
NM_001363436.1	C7orf25	XLOC_109352	2025 coding	coding	coding	coding
MSTRG.101968.1		XLOC_109359	283 coding	noncoding	noncoding	noncoding
MSTRG.102119.1	HECW1	XLOC_109369	307 coding	noncoding	noncoding	noncoding
NR_146941.2	COA1	XLOC_109371	4404 coding	coding	coding	noncoding
MSTRG.102124.1		XLOC_109382	427 coding	coding	noncoding	noncoding
MSTRG.102141.1	URGCP	XLOC_109385	249 coding	noncoding	noncoding	noncoding
MSTRG.102179.1	AEBP1	XLOC_109398	244 coding	coding	noncoding	noncoding
MSTRG.102187.1		XLOC_109407	260 coding	noncoding	noncoding	noncoding
MSTRG.102188.1		XLOC_109408	252 coding	noncoding	noncoding	noncoding
MSTRG.102229.1		XLOC_109436	287 coding	noncoding	noncoding	noncoding
MSTRG.102237.1		XLOC_109442	246 coding	noncoding	noncoding	noncoding
MSTRG.102241.1		XLOC_109445	269 coding	noncoding	noncoding	noncoding
MSTRG.102321.5	CCM2	XLOC_109466	3432 coding	noncoding	coding	coding
XR_002956415.1	NACAD	XLOC_109468	5432 coding	coding	coding	coding
MSTRG.102288.1		XLOC_109480	313 coding	noncoding	noncoding	noncoding
MSTRG.102302.1		XLOC_109487	250 coding	noncoding	noncoding	noncoding
MSTRG.102303.1		XLOC_109488	245 coding	noncoding	noncoding	noncoding
MSTRG.102348.1	ADCY1	XLOC_109492	234 coding	noncoding	noncoding	noncoding
MSTRG.102337.1		XLOC_109497	323 coding	noncoding	noncoding	noncoding
MSTRG.102409.1		XLOC_109541	203 coding	noncoding	noncoding	noncoding
MSTRG.102448.1		XLOC_109563	306 coding	noncoding	noncoding	noncoding
MSTRG.102449.1		XLOC_109564	236 coding	noncoding	noncoding	noncoding
XM_011515476.2	TNS3	XLOC_109569	8499 coding	coding	coding	coding
MSTRG.102519.1	TNS3	XLOC_109575	483 coding	coding	noncoding	noncoding
MSTRG.102465.1		XLOC_109581	254 coding	noncoding	noncoding	noncoding
MSTRG.102529.1		XLOC_109625	263 coding	noncoding	noncoding	noncoding

MSTRG.102542.1		XLOC_109630	304	coding	noncoding	noncoding	noncoding
MSTRG.102544.1		XLOC_109632	224	coding	noncoding	noncoding	noncoding
MSTRG.102602.1	ZPBP	XLOC_109651	403	coding	noncoding	noncoding	noncoding
MSTRG.102603.1	ZPBP	XLOC_109652	243	coding	noncoding	noncoding	noncoding
MSTRG.102606.1	SPATA48	XLOC_109654	250	coding	noncoding	noncoding	noncoding
MSTRG.102589.1		XLOC_109659	227	coding	noncoding	noncoding	noncoding
MSTRG.102672.1	DDC	XLOC_109670	395	coding	noncoding	noncoding	noncoding
XM_017012060.2	GRB10	XLOC_109673	7855	coding	coding	coding	coding
XM_017012029.2	GRB10	XLOC_109673	6043	coding	coding	coding	coding
NM_001287438.3	COBL	XLOC_109686	3830	coding	coding	coding	coding
MSTRG.102719.1	COBL	XLOC_109692	223	coding	noncoding	noncoding	noncoding
MSTRG.102692.1		XLOC_109697	313	coding	noncoding	noncoding	noncoding
MSTRG.102728.1		XLOC_109707	249	coding	noncoding	noncoding	noncoding
MSTRG.102740.1		XLOC_109715	235	coding	noncoding	noncoding	noncoding
MSTRG.102759.1		XLOC_109725	277	coding	coding	noncoding	noncoding
MSTRG.102846.1		XLOC_109748	325	coding	noncoding	noncoding	noncoding
MSTRG.102847.1		XLOC_109749	251	coding	noncoding	noncoding	noncoding
MSTRG.102853.1		XLOC_109751	285	coding	noncoding	noncoding	noncoding
MSTRG.102863.1		XLOC_109756	265	coding	noncoding	noncoding	noncoding
NR_047551.1	EGFR-AS1	XLOC_109771	2821	coding	coding	coding	noncoding
NM_001284284.1	VOPP1	XLOC_109778	2928	coding	noncoding	coding	coding
MSTRG.103003.1	VOPP1	XLOC_109795	601	coding	noncoding	noncoding	noncoding
MSTRG.102948.1		XLOC_109805	354	coding	noncoding	noncoding	noncoding
MSTRG.103010.1		XLOC_109824	257	coding	noncoding	noncoding	noncoding
XM_017012928.2	LOC107986800	XLOC_109829	2978	coding	coding	coding	coding
NR_029420.1	LOC650226	XLOC_109832	1667	coding	coding	noncoding	noncoding
MSTRG.103025.1		XLOC_109835	287	coding	noncoding	noncoding	noncoding
MSTRG.103051.1		XLOC_109839	301	coding	noncoding	noncoding	noncoding
MSTRG.103042.1		XLOC_109841	465	coding	noncoding	noncoding	noncoding
NR_130727.1	LOC401357	XLOC_109842	3613	coding	coding	coding	coding
MSTRG.103054.1		XLOC_109847	285	coding	noncoding	noncoding	noncoding
MSTRG.103057.1		XLOC_109849	260	coding	noncoding	noncoding	noncoding
XM_011515610.2	ZNF479	XLOC_109850	2379	coding	coding	coding	coding
MSTRG.103064.1		XLOC_109853	369	coding	noncoding	noncoding	noncoding
MSTRG.103068.1		XLOC_109856	325	coding	noncoding	noncoding	noncoding
MSTRG.103070.1		XLOC_109858	299	coding	noncoding	noncoding	noncoding
MSTRG.103081.1		XLOC_109866	268	coding	noncoding	noncoding	noncoding
MSTRG.103083.1	LOC105375302	XLOC_109867	263	coding	noncoding	noncoding	noncoding
MSTRG.103170.1		XLOC_109906	218	coding	noncoding	noncoding	noncoding
MSTRG.103181.1		XLOC_109914	291	coding	noncoding	noncoding	noncoding
MSTRG.103183.1		XLOC_109915	251	coding	noncoding	noncoding	noncoding
MSTRG.103185.1		XLOC_109917	231	coding	noncoding	noncoding	noncoding
XR_001744922.1	LOC105375322	XLOC_109938	2678	coding	noncoding	coding	noncoding

XM_024446742.1	ZNF680	XLOC_109939	2926 coding	coding	coding	coding
MSTRG.103316.1		XLOC_109970	267 coding	noncoding	noncoding	noncoding
MSTRG.103335.1	CRCP	XLOC_109980	2310 coding	coding	coding	noncoding
MSTRG.103558.1		XLOC_109992	255 coding	noncoding	noncoding	noncoding
MSTRG.103564.2	GS1-124K5.4	XLOC_109995	420 coding	noncoding	noncoding	noncoding
MSTRG.103565.1		XLOC_109996	448 coding	noncoding	noncoding	noncoding
MSTRG.103566.1		XLOC_109997	256 coding	noncoding	noncoding	noncoding
MSTRG.103571.1		XLOC_110000	272 coding	noncoding	noncoding	noncoding
MSTRG.103577.1	RABGEF1	XLOC_110004	258 coding	noncoding	noncoding	noncoding
MSTRG.103578.1	RABGEF1	XLOC_110005	269 coding	noncoding	noncoding	noncoding
MSTRG.103491.1	TYW1	XLOC_110023	1503 coding	coding	noncoding	coding
MSTRG.103516.1		XLOC_110028	252 coding	noncoding	noncoding	noncoding
MSTRG.103518.1	PMS2P4	XLOC_110030	251 coding	noncoding	noncoding	noncoding
MSTRG.103520.3	STAG3L4	XLOC_110031	277 coding	noncoding	noncoding	noncoding
MSTRG.103421.1		XLOC_110049	240 coding	noncoding	noncoding	noncoding
MSTRG.103423.1		XLOC_110050	285 coding	noncoding	noncoding	noncoding
MSTRG.103427.1		XLOC_110052	244 coding	noncoding	noncoding	noncoding
MSTRG.103429.1		XLOC_110054	265 coding	noncoding	noncoding	noncoding
MSTRG.103435.1	LOC105375341	XLOC_110057	213 coding	noncoding	noncoding	noncoding
MSTRG.103437.1	LOC105375341	XLOC_110059	273 coding	noncoding	noncoding	noncoding
MSTRG.103455.1		XLOC_110067	223 coding	noncoding	noncoding	noncoding
MSTRG.103457.1		XLOC_110069	237 coding	noncoding	noncoding	noncoding
MSTRG.103472.1	LOC105375345	XLOC_110078	201 coding	noncoding	noncoding	noncoding
MSTRG.103470.1		XLOC_110079	412 coding	coding	noncoding	noncoding
MSTRG.103475.1		XLOC_110080	266 coding	noncoding	noncoding	noncoding
MSTRG.103479.1		XLOC_110082	293 coding	noncoding	noncoding	noncoding
MSTRG.103484.1	LOC105375346	XLOC_110083	255 coding	noncoding	noncoding	noncoding
MSTRG.103598.1		XLOC_110300	411 coding	noncoding	noncoding	noncoding
MSTRG.103605.1		XLOC_110303	273 coding	noncoding	noncoding	noncoding
MSTRG.103630.1	GALNT17	XLOC_110315	260 coding	noncoding	noncoding	noncoding
XM_011516596.2	CALN1	XLOC_110318	9956 coding	coding	coding	coding
MSTRG.103638.1	CALN1	XLOC_110321	255 coding	noncoding	noncoding	noncoding
MSTRG.103639.1	CALN1	XLOC_110322	383 coding	noncoding	noncoding	noncoding
MSTRG.103642.1	CALN1	XLOC_110323	342 coding	noncoding	noncoding	noncoding
MSTRG.103660.1	CALN1	XLOC_110330	261 coding	noncoding	noncoding	noncoding
MSTRG.103675.1		XLOC_110337	318 coding	noncoding	noncoding	noncoding
MSTRG.103681.1	TYW1B	XLOC_110340	253 coding	noncoding	noncoding	noncoding
MSTRG.103685.1	TYW1B	XLOC_110342	786 coding	noncoding	noncoding	noncoding
MSTRG.103686.1	TYW1B	XLOC_110343	264 coding	noncoding	noncoding	noncoding
MSTRG.103688.1	TYW1B	XLOC_110345	751 coding	noncoding	noncoding	noncoding
MSTRG.103810.2	TRIM74	XLOC_110352	908 coding	coding	coding	noncoding
XR_002956448.1	TRIM74	XLOC_110352	1590 coding	noncoding	coding	noncoding
XM_011516277.1	MLXIPL	XLOC_110371	3448 coding	coding	coding	coding

MSTRG.103707.1		XLOC_110372	227 coding	noncoding	noncoding	noncoding
MSTRG.103740.1	STX1A	XLOC_110376	266 coding	noncoding	noncoding	noncoding
NM_001306.4	CLDN3	XLOC_110379	1274 coding	coding	coding	coding
MSTRG.103725.1	METTL27	XLOC_110383	226 coding	noncoding	noncoding	noncoding
MSTRG.103720.1		XLOC_110384	257 coding	noncoding	noncoding	noncoding
MSTRG.103729.1		XLOC_110385	225 coding	noncoding	noncoding	noncoding
MSTRG.103731.1		XLOC_110386	287 coding	noncoding	noncoding	noncoding
MSTRG.103753.1		XLOC_110388	225 coding	noncoding	noncoding	noncoding
MSTRG.103758.1	ELN	XLOC_110393	252 coding	noncoding	noncoding	noncoding
MSTRG.103755.1		XLOC_110395	262 coding	noncoding	noncoding	noncoding
MSTRG.103767.1		XLOC_110400	305 coding	noncoding	noncoding	noncoding
MSTRG.103769.1		XLOC_110401	339 coding	noncoding	noncoding	noncoding
MSTRG.103779.1	CLIP2	XLOC_110404	214 coding	noncoding	noncoding	noncoding
MSTRG.103775.1		XLOC_110412	391 coding	noncoding	noncoding	noncoding
MSTRG.103792.1	GTF2IRD1	XLOC_110414	238 coding	noncoding	noncoding	noncoding
MSTRG.103794.1	GTF2IRD1	XLOC_110416	305 coding	noncoding	noncoding	noncoding
MSTRG.103788.1		XLOC_110419	258 coding	noncoding	noncoding	noncoding
NM_001368300.1	GTF2IRD2	XLOC_110424	4387 coding	coding	coding	coding
MSTRG.103838.1	GTF2IRD2	XLOC_110425	226 coding	noncoding	noncoding	noncoding
XR_001745245.1	LOC107986811	XLOC_110429	1660 coding	noncoding	noncoding	noncoding
NM_001363447.2	RCC1L	XLOC_110431	2543 coding	coding	coding	coding
MSTRG.103912.1	HIP1	XLOC_110453	227 coding	noncoding	noncoding	noncoding
MSTRG.103915.1	HIP1	XLOC_110455	282 coding	noncoding	noncoding	noncoding
MSTRG.103917.1	HIP1	XLOC_110457	524 coding	noncoding	noncoding	noncoding
MSTRG.103934.1	HIP1	XLOC_110461	259 coding	noncoding	noncoding	noncoding
MSTRG.103980.1	STYXL1	XLOC_110470	238 coding	noncoding	noncoding	noncoding
MSTRG.104729.1	SSC4D	XLOC_110486	227 coding	noncoding	noncoding	noncoding
MSTRG.104767.1		XLOC_110502	253 coding	noncoding	noncoding	noncoding
NM_001351348.1	SPDYE18	XLOC_110510	1242 coding	coding	coding	coding
XM_011516327.2	GSAP	XLOC_110531	3218 coding	coding	coding	coding
MSTRG.104834.1	GSAP	XLOC_110538	291 coding	noncoding	noncoding	noncoding
MSTRG.104237.1		XLOC_110557	241 coding	noncoding	noncoding	noncoding
MSTRG.104239.1		XLOC_110558	226 coding	noncoding	noncoding	noncoding
MSTRG.104267.9	APTR	XLOC_110566	585 coding	noncoding	coding	noncoding
MSTRG.104267.11	APTR	XLOC_110566	724 coding	noncoding	noncoding	noncoding
MSTRG.104313.4	PHTF2	XLOC_110570	2098 coding	coding	noncoding	noncoding
MSTRG.104360.1	MAGI2	XLOC_110587	325 coding	noncoding	noncoding	noncoding
MSTRG.104294.1		XLOC_110611	274 coding	noncoding	noncoding	noncoding
MSTRG.104304.1		XLOC_110618	285 coding	noncoding	noncoding	noncoding
MSTRG.104436.1		XLOC_110663	307 coding	noncoding	noncoding	noncoding
MSTRG.104437.1		XLOC_110664	227 coding	noncoding	noncoding	noncoding
MSTRG.104438.1		XLOC_110665	383 coding	noncoding	noncoding	noncoding
MSTRG.104457.1		XLOC_110677	303 coding	noncoding	noncoding	noncoding

XM_006716119.3	CACNA2D1	XLOC_110688	9520 coding	coding	coding	coding
MSTRG.104531.1	CACNA2D1	XLOC_110697	261 coding	noncoding	noncoding	noncoding
MSTRG.104541.1	PCLO	XLOC_110714	266 coding	noncoding	noncoding	noncoding
MSTRG.104557.1		XLOC_110724	269 coding	noncoding	noncoding	noncoding
MSTRG.104599.1	SEMA3A	XLOC_110737	423 coding	noncoding	noncoding	noncoding
MSTRG.104604.1	SEMA3A	XLOC_110740	281 coding	noncoding	noncoding	noncoding
MSTRG.104569.1		XLOC_110743	237 coding	noncoding	noncoding	noncoding
MSTRG.104570.1		XLOC_110744	513 coding	noncoding	noncoding	noncoding
MSTRG.104580.1		XLOC_110747	251 coding	noncoding	noncoding	noncoding
XM_017011873.1	SEMA3D	XLOC_110751	7121 coding	coding	coding	coding
MSTRG.104608.1		XLOC_110756	288 coding	noncoding	noncoding	noncoding
MSTRG.104611.1		XLOC_110758	322 coding	noncoding	noncoding	noncoding
MSTRG.104660.1	GRM3	XLOC_110775	250 coding	noncoding	noncoding	noncoding
MSTRG.105037.1	ABCB4	XLOC_110804	249 coding	noncoding	noncoding	noncoding
MSTRG.104880.1		XLOC_110867	239 coding	noncoding	noncoding	noncoding
MSTRG.104958.1	ZNF804B	XLOC_110877	260 coding	coding	noncoding	noncoding
MSTRG.104976.1	STEAP2-AS1	XLOC_110885	230 coding	noncoding	noncoding	noncoding
MSTRG.104979.1	STEAP2-AS1	XLOC_110888	295 coding	noncoding	noncoding	noncoding
MSTRG.105003.1		XLOC_110909	316 coding	noncoding	noncoding	noncoding
MSTRG.105004.1		XLOC_110910	249 coding	noncoding	noncoding	noncoding
MSTRG.105078.1		XLOC_110915	225 coding	noncoding	noncoding	noncoding
MSTRG.105123.1		XLOC_110933	311 coding	noncoding	noncoding	noncoding
XM_011516004.3	FAM133B	XLOC_110973	2360 coding	noncoding	coding	coding
MSTRG.105305.1	CDK6	XLOC_110998	258 coding	noncoding	noncoding	noncoding
MSTRG.105274.1		XLOC_111003	295 coding	noncoding	noncoding	noncoding
MSTRG.105277.1		XLOC_111004	259 coding	noncoding	noncoding	noncoding
NM_001303500.3	SAMD9L	XLOC_111009	6378 coding	noncoding	coding	coding
MSTRG.105335.1		XLOC_111032	383 coding	noncoding	noncoding	noncoding
MSTRG.105337.1		XLOC_111033	230 coding	noncoding	noncoding	noncoding
MSTRG.105340.1		XLOC_111035	308 coding	noncoding	noncoding	noncoding
MSTRG.105359.1		XLOC_111050	239 coding	noncoding	noncoding	noncoding
MSTRG.105403.1		XLOC_111082	380 coding	noncoding	noncoding	noncoding
MSTRG.105394.1		XLOC_111084	244 coding	noncoding	noncoding	noncoding
MSTRG.105397.1		XLOC_111090	295 coding	noncoding	noncoding	noncoding
MSTRG.105434.1	PPP1R9A	XLOC_111104	304 coding	noncoding	noncoding	noncoding
MSTRG.105413.1		XLOC_111122	257 coding	noncoding	noncoding	noncoding
MSTRG.105529.1	LOC105375410	XLOC_111162	241 coding	noncoding	noncoding	noncoding
MSTRG.105544.1		XLOC_111185	227 coding	noncoding	noncoding	noncoding
MSTRG.105559.1	DLX6-AS1	XLOC_111191	258 coding	noncoding	noncoding	noncoding
MSTRG.105562.1		XLOC_111196	264 coding	noncoding	noncoding	noncoding
MSTRG.105574.1		XLOC_111197	288 coding	noncoding	noncoding	noncoding
MSTRG.105577.1		XLOC_111198	277 coding	noncoding	noncoding	noncoding
MSTRG.105592.1	LOC105375416	XLOC_111206	281 coding	noncoding	noncoding	noncoding

MSTRG.105600.1		XLOC_111211	297	coding	noncoding	noncoding	noncoding
MSTRG.105665.1	CZ1P-ASNS	XLOC_111220	274	coding	noncoding	noncoding	noncoding
MSTRG.105738.1	BRI3	XLOC_111240	290	coding	noncoding	noncoding	noncoding
MSTRG.105743.1	BAIAP2L1	XLOC_111242	364	coding	noncoding	noncoding	noncoding
MSTRG.105744.1	BAIAP2L1	XLOC_111243	427	coding	noncoding	noncoding	noncoding
MSTRG.105745.1	BAIAP2L1	XLOC_111244	242	coding	coding	noncoding	noncoding
MSTRG.105683.1		XLOC_111245	255	coding	noncoding	noncoding	noncoding
MSTRG.105678.1		XLOC_111246	315	coding	noncoding	noncoding	noncoding
MSTRG.105682.1		XLOC_111247	570	coding	noncoding	noncoding	noncoding
MSTRG.105676.1		XLOC_111249	263	coding	noncoding	noncoding	noncoding
MSTRG.105684.1		XLOC_111252	277	coding	noncoding	noncoding	noncoding
MSTRG.105698.1		XLOC_111257	290	coding	noncoding	noncoding	noncoding
MSTRG.105696.1		XLOC_111258	277	coding	noncoding	noncoding	noncoding
MSTRG.105701.1		XLOC_111260	300	coding	noncoding	noncoding	noncoding
MSTRG.105704.1	TMEM130	XLOC_111262	250	coding	noncoding	noncoding	noncoding
MSTRG.105748.1		XLOC_111272	317	coding	noncoding	noncoding	noncoding
MSTRG.105751.1		XLOC_111274	283	coding	noncoding	noncoding	noncoding
MSTRG.105769.1	MYH16	XLOC_111276	245	coding	noncoding	noncoding	noncoding
MSTRG.105773.8	PDAP1	XLOC_111279	1607	coding	coding	coding	noncoding
NM_001198879.2	ATP5MF-PTCD1	XLOC_111279	5504	coding	coding	coding	coding
MSTRG.105775.3	ARPC1A	XLOC_111280	1245	coding	noncoding	coding	noncoding
MSTRG.105798.10	FAM200A	XLOC_111291	2625	coding	coding	noncoding	noncoding
MSTRG.105798.16	FAM200A	XLOC_111291	1699	coding	coding	coding	noncoding
MSTRG.105811.1	TMEM225B	XLOC_111294	211	coding	noncoding	noncoding	noncoding
NM_001318135.2	ZNF3	XLOC_111333	2793	coding	coding	coding	coding
MSTRG.105887.1	GAL3ST4	XLOC_111345	254	coding	noncoding	noncoding	noncoding
XR_002956434.1	CASTOR3	XLOC_111347	5377	coding	coding	coding	coding
MSTRG.106040.1		XLOC_111350	213	coding	noncoding	noncoding	noncoding
MSTRG.106041.1		XLOC_111351	215	coding	noncoding	noncoding	noncoding
MSTRG.105913.1	LOC107986829	XLOC_111359	312	coding	noncoding	noncoding	noncoding
MSTRG.105906.1		XLOC_111360	229	coding	noncoding	noncoding	noncoding
MSTRG.105923.1	SAP25	XLOC_111364	279	coding	noncoding	noncoding	noncoding
MSTRG.105945.1	FBXO24	XLOC_111369	263	coding	noncoding	noncoding	noncoding
MSTRG.105927.1	ACTL6B	XLOC_111375	292	coding	noncoding	noncoding	noncoding
MSTRG.105931.1	LOC105375429	XLOC_111377	233	coding	noncoding	noncoding	noncoding
MSTRG.105932.1	LOC105375429	XLOC_111378	239	coding	noncoding	noncoding	noncoding
MSTRG.105951.1		XLOC_111381	237	coding	noncoding	noncoding	noncoding
MSTRG.105954.1		XLOC_111382	348	coding	noncoding	noncoding	noncoding
MSTRG.105967.1	SLC12A9	XLOC_111384	272	coding	noncoding	noncoding	noncoding
MSTRG.105978.1		XLOC_111394	244	coding	noncoding	noncoding	noncoding
MSTRG.105984.1	MUC12	XLOC_111396	323	coding	noncoding	noncoding	noncoding
MSTRG.105993.1	MUC17	XLOC_111398	349	coding	noncoding	noncoding	noncoding
MSTRG.105994.1	MUC17	XLOC_111399	270	coding	noncoding	noncoding	noncoding

MSTRG.106000.1		XLOC_111400	253 coding	noncoding	noncoding	noncoding
MSTRG.106001.1		XLOC_111403	254 coding	noncoding	noncoding	noncoding
XM_005250561.5	VGF	XLOC_111407	2731 coding	noncoding	coding	coding
MSTRG.106018.1		XLOC_111413	323 coding	noncoding	noncoding	noncoding
NM_001287525.2	IFT22	XLOC_111417	4954 coding	coding	coding	noncoding
MSTRG.106031.1		XLOC_111418	215 coding	noncoding	noncoding	noncoding
MSTRG.106075.1	COL26A1	XLOC_111419	306 coding	noncoding	noncoding	noncoding
MSTRG.106082.1	COL26A1	XLOC_111421	539 coding	noncoding	noncoding	noncoding
MSTRG.106068.1		XLOC_111430	255 coding	noncoding	noncoding	noncoding
MSTRG.106063.1		XLOC_111431	215 coding	noncoding	noncoding	noncoding
MSTRG.106603.4	LOC100630923	XLOC_111437	529 coding	coding	coding	noncoding
MSTRG.106613.1	ORAI2	XLOC_111443	593 coding	noncoding	noncoding	noncoding
MSTRG.106620.1	LRWD1	XLOC_111448	454 coding	coding	noncoding	noncoding
MSTRG.106627.1	UPK3BL2	XLOC_111452	230 coding	noncoding	noncoding	noncoding
MSTRG.106633.1	POLR2J2	XLOC_111455	230 coding	noncoding	noncoding	noncoding
MSTRG.106190.1	RELN	XLOC_111492	245 coding	noncoding	noncoding	noncoding
MSTRG.106197.1	RELN	XLOC_111495	316 coding	noncoding	noncoding	noncoding
MSTRG.106133.1	ORC5	XLOC_111502	263 coding	noncoding	noncoding	noncoding
MSTRG.106122.1		XLOC_111506	261 coding	noncoding	noncoding	noncoding
MSTRG.106152.1	LHFPL3	XLOC_111517	363 coding	noncoding	noncoding	noncoding
MSTRG.106168.1	LHFPL3	XLOC_111524	282 coding	noncoding	noncoding	noncoding
MSTRG.106438.19	SRPK2	XLOC_111529	12766 coding	coding	coding	coding
MSTRG.106203.1	PUS7	XLOC_111531	220 coding	noncoding	noncoding	noncoding
MSTRG.106204.1	PUS7	XLOC_111532	240 coding	noncoding	noncoding	noncoding
MSTRG.106206.1		XLOC_111535	273 coding	noncoding	noncoding	noncoding
XM_006715901.3	ATXN7L1	XLOC_111536	6354 coding	coding	coding	coding
MSTRG.106219.1		XLOC_111572	209 coding	noncoding	noncoding	noncoding
MSTRG.106255.1	CTB-30L5.1	XLOC_111603	238 coding	noncoding	noncoding	noncoding
MSTRG.106410.1	SLC26A3	XLOC_111631	273 coding	noncoding	noncoding	noncoding
MSTRG.106397.1		XLOC_111637	313 coding	noncoding	noncoding	noncoding
MSTRG.106461.1	PNPLA8	XLOC_111660	245 coding	noncoding	noncoding	noncoding
MSTRG.106456.1		XLOC_111663	301 coding	noncoding	noncoding	noncoding
MSTRG.106466.1		XLOC_111673	314 coding	noncoding	noncoding	noncoding
MSTRG.106471.1		XLOC_111676	281 coding	noncoding	noncoding	noncoding
MSTRG.106543.1		XLOC_111682	214 coding	noncoding	noncoding	noncoding
MSTRG.106745.8	IFRD1	XLOC_111859	1016 coding	coding	noncoding	noncoding
MSTRG.106722.1		XLOC_111870	272 coding	noncoding	noncoding	noncoding
MSTRG.106729.1	LOC101928012	XLOC_111872	287 coding	noncoding	noncoding	noncoding
MSTRG.106779.1		XLOC_111906	213 coding	noncoding	noncoding	noncoding
MSTRG.106787.1		XLOC_111910	285 coding	noncoding	noncoding	noncoding
MSTRG.106865.1	FOXP2	XLOC_111933	401 coding	noncoding	noncoding	noncoding
MSTRG.106839.1		XLOC_111942	290 coding	noncoding	noncoding	noncoding
MSTRG.106838.1		XLOC_111943	271 coding	coding	noncoding	noncoding

MSTRG.106979.3	MDFIC	XLOC_111946	2368 coding	coding	coding	noncoding
MSTRG.107008.1	LINC01392	XLOC_111951	290 coding	noncoding	noncoding	noncoding
MSTRG.106949.1		XLOC_111954	583 coding	coding	coding	noncoding
MSTRG.106968.1		XLOC_111962	243 coding	noncoding	noncoding	noncoding
MSTRG.106973.1		XLOC_111967	226 coding	noncoding	noncoding	noncoding
NR_130921.2	LOC102724434	XLOC_111984	1997 coding	noncoding	noncoding	noncoding
MSTRG.107045.1		XLOC_111985	238 coding	noncoding	noncoding	noncoding
MSTRG.107086.1		XLOC_112003	302 coding	noncoding	noncoding	noncoding
MSTRG.107141.1		XLOC_112035	366 coding	noncoding	noncoding	noncoding
MSTRG.107150.1		XLOC_112039	415 coding	noncoding	noncoding	noncoding
MSTRG.107196.1		XLOC_112051	223 coding	noncoding	noncoding	noncoding
MSTRG.107205.1	LOC105375473	XLOC_112054	632 coding	noncoding	noncoding	noncoding
MSTRG.107246.1		XLOC_112066	305 coding	noncoding	noncoding	noncoding
MSTRG.107303.1	LINC02476	XLOC_112086	249 coding	noncoding	noncoding	noncoding
MSTRG.107272.1		XLOC_112089	292 coding	noncoding	noncoding	noncoding
MSTRG.107275.1		XLOC_112091	280 coding	noncoding	noncoding	noncoding
MSTRG.107350.1		XLOC_112125	284 coding	noncoding	noncoding	noncoding
MSTRG.107380.1	PTPRZ1	XLOC_112137	265 coding	noncoding	noncoding	noncoding
MSTRG.107383.1		XLOC_112140	346 coding	noncoding	noncoding	noncoding
MSTRG.107699.1	CADPS2	XLOC_112153	220 coding	noncoding	noncoding	noncoding
MSTRG.107741.1	HYAL4	XLOC_112192	234 coding	noncoding	noncoding	noncoding
MSTRG.107751.1		XLOC_112198	312 coding	coding	noncoding	noncoding
MSTRG.107778.1		XLOC_112213	291 coding	noncoding	noncoding	noncoding
MSTRG.107822.1	LOC101928283	XLOC_112248	221 coding	noncoding	noncoding	noncoding
MSTRG.107829.1		XLOC_112252	253 coding	noncoding	noncoding	noncoding
MSTRG.107843.1		XLOC_112260	360 coding	noncoding	noncoding	noncoding
MSTRG.107880.1	GRM8	XLOC_112266	343 coding	noncoding	noncoding	noncoding
MSTRG.107884.1	GRM8	XLOC_112269	229 coding	noncoding	noncoding	noncoding
MSTRG.107892.1	GRM8	XLOC_112272	249 coding	noncoding	noncoding	noncoding
MSTRG.107913.1		XLOC_112291	347 coding	noncoding	noncoding	noncoding
MSTRG.107930.1	LOC105375490	XLOC_112294	312 coding	noncoding	noncoding	noncoding
MSTRG.108069.1		XLOC_112307	453 coding	noncoding	noncoding	noncoding
MSTRG.107947.1		XLOC_112310	408 coding	noncoding	noncoding	noncoding
MSTRG.107948.1		XLOC_112311	308 coding	noncoding	noncoding	noncoding
MSTRG.107974.1		XLOC_112319	222 coding	noncoding	noncoding	noncoding
MSTRG.108050.1	TNPO3	XLOC_112356	210 coding	noncoding	noncoding	noncoding
MSTRG.108051.1	TNPO3	XLOC_112357	276 coding	noncoding	noncoding	noncoding
MSTRG.108025.1		XLOC_112359	282 coding	noncoding	noncoding	noncoding
MSTRG.108027.1		XLOC_112360	247 coding	noncoding	noncoding	noncoding
MSTRG.108156.1		XLOC_112377	289 coding	noncoding	noncoding	noncoding
MSTRG.108157.1		XLOC_112378	288 coding	noncoding	noncoding	noncoding
MSTRG.108160.1		XLOC_112379	964 coding	noncoding	noncoding	noncoding
MSTRG.108169.1	NRF1	XLOC_112381	1834 coding	coding	noncoding	coding

NM_182697.3	UBE2H	XLOC_112391	5069 coding	coding	coding	coding
MSTRG.108091.1	UBE2H	XLOC_112395	224 coding	noncoding	noncoding	noncoding
MSTRG.108100.1	ZC3HC1	XLOC_112404	243 coding	noncoding	noncoding	noncoding
MSTRG.108101.1	ZC3HC1	XLOC_112405	223 coding	noncoding	noncoding	noncoding
MSTRG.108516.1	LOC105375508	XLOC_112434	273 coding	noncoding	noncoding	noncoding
NR_109853.1	LINC-PINT	XLOC_112467	2298 coding	noncoding	noncoding	noncoding
NR_109852.1	LINC-PINT	XLOC_112467	3230 coding	noncoding	noncoding	noncoding
MSTRG.108592.12	MKLN1	XLOC_112500	3448 coding	coding	noncoding	noncoding
MSTRG.108592.18	MKLN1	XLOC_112500	1233 coding	coding	noncoding	noncoding
MSTRG.108218.1		XLOC_112516	249 coding	noncoding	noncoding	noncoding
XM_006716171.4	PLXNA4	XLOC_112519	13417 coding	coding	coding	coding
XM_011516676.2	PLXNA4	XLOC_112519	4587 coding	coding	coding	coding
MSTRG.108330.1	CHCHD3	XLOC_112549	325 coding	noncoding	noncoding	noncoding
MSTRG.108233.1		XLOC_112573	247 coding	noncoding	noncoding	noncoding
MSTRG.108366.1	LRGUK	XLOC_112599	255 coding	noncoding	noncoding	noncoding
XM_024446695.1	AKR1B1	XLOC_112606	2098 coding	coding	coding	coding
MSTRG.108379.1		XLOC_112609	248 coding	noncoding	noncoding	noncoding
MSTRG.108381.1		XLOC_112610	238 coding	noncoding	noncoding	noncoding
XR_928005.2	LOC105375520	XLOC_112611	12249 coding	coding	coding	noncoding
MSTRG.108670.1		XLOC_112662	466 coding	noncoding	noncoding	noncoding
MSTRG.109059.1	LOC105375523	XLOC_112718	314 coding	noncoding	noncoding	noncoding
MSTRG.108788.1		XLOC_112724	261 coding	noncoding	noncoding	noncoding
MSTRG.108818.1	PTN	XLOC_112739	246 coding	noncoding	noncoding	noncoding
MSTRG.108819.1	PTN	XLOC_112740	292 coding	noncoding	noncoding	noncoding
MSTRG.108814.1		XLOC_112743	222 coding	noncoding	noncoding	noncoding
NM_001321710.2	DGKI	XLOC_112744	13232 coding	coding	coding	coding
MSTRG.108844.1	DGKI	XLOC_112747	207 coding	noncoding	noncoding	noncoding
MSTRG.108860.1	DGKI	XLOC_112755	229 coding	noncoding	noncoding	noncoding
MSTRG.108901.1	CREB3L2	XLOC_112767	275 coding	noncoding	noncoding	noncoding
MSTRG.108881.1		XLOC_112775	306 coding	noncoding	noncoding	noncoding
MSTRG.108891.1		XLOC_112780	267 coding	noncoding	noncoding	noncoding
MSTRG.108893.1		XLOC_112781	309 coding	noncoding	noncoding	noncoding
MSTRG.108905.1	SVOPL	XLOC_112785	328 coding	noncoding	noncoding	noncoding
XM_011516442.2	KIAA1549	XLOC_112789	12658 coding	coding	coding	coding
MSTRG.108924.1	KIAA1549	XLOC_112794	314 coding	noncoding	noncoding	noncoding
MSTRG.108953.1	ZC3HAV1	XLOC_112801	389 coding	noncoding	noncoding	noncoding
MSTRG.108966.1		XLOC_112819	247 coding	noncoding	noncoding	noncoding
MSTRG.108986.1	UBN2	XLOC_112824	259 coding	noncoding	noncoding	noncoding
MSTRG.108987.1	UBN2	XLOC_112825	204 coding	noncoding	noncoding	noncoding
MSTRG.109065.1		XLOC_112830	255 coding	noncoding	noncoding	noncoding
MSTRG.109248.1	HIPK2	XLOC_112840	302 coding	noncoding	noncoding	noncoding
MSTRG.109263.2	TBXAS1	XLOC_112851	3502 coding	coding	coding	coding
MSTRG.109093.3	KDM7A	XLOC_112857	4090 coding	coding	coding	coding

MSTRG.109078.1		XLOC_112858	325 coding	noncoding	noncoding	noncoding
MSTRG.109102.1	SLC37A3	XLOC_112869	268 coding	noncoding	noncoding	noncoding
MSTRG.109144.8	BRAF	XLOC_112882	1825 coding	coding	coding	coding
MSTRG.109154.1	BRAF	XLOC_112892	244 coding	noncoding	noncoding	noncoding
MSTRG.109171.1	TMEM178B	XLOC_112908	269 coding	noncoding	noncoding	noncoding
MSTRG.109526.13	TRBV1	XLOC_112979	3905 coding	coding	coding	coding
MSTRG.109526.29	TRBV1	XLOC_112979	936 coding	coding	noncoding	noncoding
MSTRG.109526.39	TRBV1	XLOC_112979	996 coding	coding	coding	noncoding
MSTRG.109423.9	EPHA1	XLOC_113011	3382 coding	coding	coding	coding
MSTRG.109583.1		XLOC_113106	306 coding	noncoding	noncoding	noncoding
MSTRG.109664.1	CNTNAP2	XLOC_113135	384 coding	noncoding	noncoding	noncoding
MSTRG.109673.1	CNTNAP2	XLOC_113138	265 coding	coding	noncoding	noncoding
MSTRG.109621.1		XLOC_113164	228 coding	noncoding	noncoding	noncoding
MSTRG.109624.1		XLOC_113166	264 coding	noncoding	noncoding	noncoding
MSTRG.109625.1		XLOC_113167	214 coding	noncoding	noncoding	noncoding
MSTRG.109629.1		XLOC_113168	299 coding	noncoding	noncoding	noncoding
MSTRG.109630.1		XLOC_113169	277 coding	noncoding	noncoding	noncoding
MSTRG.109634.1		XLOC_113175	256 coding	noncoding	noncoding	noncoding
MSTRG.109721.1		XLOC_113189	281 coding	noncoding	noncoding	noncoding
MSTRG.109733.1	PDIA4	XLOC_113195	222 coding	noncoding	noncoding	noncoding
MSTRG.109734.1	PDIA4	XLOC_113196	402 coding	noncoding	noncoding	noncoding
MSTRG.109786.1		XLOC_113223	276 coding	noncoding	noncoding	noncoding
MSTRG.109888.1	KRBA1	XLOC_113279	438 coding	coding	noncoding	noncoding
MSTRG.110049.2	GIMAP4	XLOC_113314	2238 coding	coding	noncoding	noncoding
MSTRG.110062.3	GIMAP2	XLOC_113324	4701 coding	coding	noncoding	noncoding
MSTRG.109986.1		XLOC_113338	257 coding	noncoding	noncoding	noncoding
NM_004935.4	CDK5	XLOC_113351	1122 coding	coding	coding	coding
NM_001164410.3	CDK5	XLOC_113351	1026 coding	coding	coding	coding
MSTRG.110244.6	WDR86	XLOC_113376	6326 coding	coding	coding	coding
NM_001284260.2	WDR86	XLOC_113376	2102 coding	coding	coding	coding
MSTRG.110230.1		XLOC_113395	276 coding	noncoding	noncoding	noncoding
XM_011516286.2	PRKAG2	XLOC_113396	5520 coding	coding	coding	coding
MSTRG.110300.1	PRKAG2	XLOC_113408	355 coding	noncoding	noncoding	noncoding
MSTRG.110259.1		XLOC_113417	318 coding	noncoding	noncoding	noncoding
MSTRG.110264.1	GALNTL5	XLOC_113421	232 coding	noncoding	noncoding	noncoding
MSTRG.110269.1		XLOC_113473	290 coding	noncoding	noncoding	noncoding
MSTRG.110274.1		XLOC_113476	267 coding	noncoding	noncoding	noncoding
MSTRG.110275.1		XLOC_113480	393 coding	noncoding	noncoding	noncoding
MSTRG.110309.1		XLOC_113504	247 coding	coding	noncoding	noncoding
MSTRG.110312.1		XLOC_113506	286 coding	noncoding	noncoding	noncoding
MSTRG.110382.1	DPP6	XLOC_113530	366 coding	noncoding	noncoding	noncoding
MSTRG.110383.1	DPP6	XLOC_113531	310 coding	noncoding	noncoding	noncoding
MSTRG.110416.1		XLOC_113552	203 coding	noncoding	noncoding	noncoding

MSTRG.110417.1		XLOC_113553	240	coding	noncoding	noncoding	noncoding
NR_163165.1	CNPY1	XLOC_113575	3746	coding	coding	coding	noncoding
MSTRG.110575.1	LOC100506302	XLOC_113584	17446	coding	noncoding	coding	noncoding
MSTRG.110548.1		XLOC_113595	245	coding	noncoding	noncoding	noncoding
MSTRG.110553.1		XLOC_113602	287	coding	noncoding	noncoding	noncoding
MSTRG.110544.1		XLOC_113603	370	coding	noncoding	noncoding	noncoding
MSTRG.110607.1		XLOC_113636	205	coding	noncoding	noncoding	noncoding
MSTRG.110613.1		XLOC_113652	280	coding	noncoding	noncoding	noncoding
XM_011516447.2	PTPRN2	XLOC_113661	11774	coding	coding	coding	coding
XR_002956471.1	ESYT2	XLOC_113690	7518	coding	coding	coding	coding
MSTRG.110782.1		XLOC_113721	279	coding	noncoding	noncoding	noncoding
MSTRG.110817.1		XLOC_113723	345	coding	noncoding	noncoding	noncoding
MSTRG.110920.1		XLOC_113775	215	coding	noncoding	noncoding	noncoding
MSTRG.110942.1		XLOC_113784	225	coding	noncoding	noncoding	noncoding
MSTRG.111027.1	CSMD1	XLOC_113794	234	coding	noncoding	noncoding	noncoding
MSTRG.111030.1	CSMD1	XLOC_113796	375	coding	noncoding	noncoding	noncoding
MSTRG.111040.1	CSMD1	XLOC_113801	313	coding	noncoding	coding	noncoding
MSTRG.111058.1	CSMD1	XLOC_113810	299	coding	noncoding	noncoding	noncoding
MSTRG.110979.1	LOC105377793	XLOC_113832	238	coding	noncoding	noncoding	noncoding
MSTRG.110988.1		XLOC_113837	303	coding	noncoding	noncoding	noncoding
MSTRG.110993.1		XLOC_113839	265	coding	noncoding	noncoding	noncoding
MSTRG.111003.1		XLOC_113844	309	coding	noncoding	noncoding	noncoding
MSTRG.111132.1		XLOC_113848	275	coding	noncoding	noncoding	noncoding
XM_017013829.2	MCPH1	XLOC_113852	3853	coding	coding	coding	coding
MSTRG.111118.1		XLOC_113916	238	coding	noncoding	noncoding	noncoding
MSTRG.111339.1	FAM167A-AS1	XLOC_114001	304	coding	noncoding	noncoding	noncoding
MSTRG.111413.7	CTSB	XLOC_114014	1781	coding	coding	coding	coding
MSTRG.111459.1	LOC100506990	XLOC_114027	227	coding	noncoding	noncoding	noncoding
MSTRG.111498.1	DLC1	XLOC_114047	295	coding	noncoding	noncoding	noncoding
MSTRG.111505.1	DLC1	XLOC_114053	615	coding	noncoding	noncoding	noncoding
MSTRG.111481.1		XLOC_114063	304	coding	noncoding	noncoding	noncoding
MSTRG.111519.1	SGCZ	XLOC_114064	309	coding	noncoding	noncoding	noncoding
MSTRG.111512.1		XLOC_114082	231	coding	noncoding	noncoding	noncoding
MSTRG.111564.1		XLOC_114092	377	coding	noncoding	noncoding	noncoding
MSTRG.111569.1		XLOC_114095	271	coding	noncoding	noncoding	noncoding
XR_001745519.1	MICU3	XLOC_114106	11463	coding	coding	coding	coding
MSTRG.111683.1	VPS37A	XLOC_114126	529	coding	noncoding	noncoding	noncoding
MSTRG.111643.1	PDGFRL	XLOC_114136	587	coding	noncoding	noncoding	noncoding
MSTRG.111644.1	PDGFRL	XLOC_114137	251	coding	noncoding	noncoding	noncoding
MSTRG.111663.1	MTUS1	XLOC_114142	257	coding	noncoding	noncoding	noncoding
MSTRG.111667.1	MTUS1	XLOC_114144	249	coding	noncoding	noncoding	noncoding
MSTRG.111648.1		XLOC_114148	254	coding	noncoding	noncoding	noncoding
NM_001160174.2	NAT1	XLOC_114175	2022	coding	coding	coding	coding

MSTRG.111712.1		XLOC_114186	305	coding	noncoding	noncoding	noncoding
MSTRG.111736.1	PSD3	XLOC_114193	236	coding	noncoding	noncoding	noncoding
MSTRG.111761.1	PSD3	XLOC_114212	271	coding	noncoding	noncoding	noncoding
MSTRG.111715.1	LOC105379301	XLOC_114214	274	coding	noncoding	noncoding	noncoding
MSTRG.111720.1	LOC100128993	XLOC_114218	308	coding	noncoding	noncoding	noncoding
MSTRG.111934.4	CSGALNACT1	XLOC_114237	5185	coding	coding	coding	coding
MSTRG.111821.1	INTS10	XLOC_114244	238	coding	noncoding	noncoding	noncoding
MSTRG.111816.1		XLOC_114263	270	coding	noncoding	noncoding	noncoding
MSTRG.111841.1	LOC105379314	XLOC_114274	301	coding	noncoding	noncoding	noncoding
MSTRG.111988.1	LGI3	XLOC_114326	320	coding	coding	coding	noncoding
MSTRG.112007.1	PHYHIP	XLOC_114330	301	coding	noncoding	noncoding	noncoding
MSTRG.112018.1	PIWIL2	XLOC_114333	258	coding	noncoding	noncoding	noncoding
MSTRG.112021.1	PIWIL2	XLOC_114334	273	coding	noncoding	noncoding	noncoding
XM_011544604.2	CCAR2	XLOC_114363	3582	coding	coding	coding	coding
NM_003841.4	TNFRSF10C	XLOC_114381	1494	coding	coding	coding	coding
XM_017013964.1	CHMP7	XLOC_114388	4560	coding	coding	coding	coding
NM_016612.4	SLC25A37	XLOC_114407	4672	coding	coding	coding	coding
MSTRG.112186.15	SLC25A37	XLOC_114407	1403	coding	coding	coding	noncoding
XM_011544550.2	SLC25A37	XLOC_114407	1176	coding	coding	coding	coding
MSTRG.112198.1	LOC107986930	XLOC_114414	265	coding	noncoding	noncoding	noncoding
MSTRG.112202.1	LOC107986930	XLOC_114417	249	coding	noncoding	noncoding	noncoding
MSTRG.112210.1	LOC107986930	XLOC_114421	288	coding	noncoding	noncoding	noncoding
MSTRG.112211.1	LOC107986930	XLOC_114422	267	coding	noncoding	noncoding	noncoding
MSTRG.112310.1	LOC101929294	XLOC_114476	243	coding	noncoding	noncoding	noncoding
MSTRG.112264.1		XLOC_114501	240	coding	noncoding	noncoding	noncoding
MSTRG.112320.1		XLOC_114556	325	coding	noncoding	noncoding	noncoding
MSTRG.112339.1	LOC107986933	XLOC_114564	410	coding	noncoding	noncoding	noncoding
XM_005273559.1	PPP2R2A	XLOC_114572	2358	coding	noncoding	coding	coding
XM_011544566.1	PPP2R2A	XLOC_114572	2380	coding	noncoding	coding	coding
MSTRG.112418.1	PPP2R2A	XLOC_114576	205	coding	noncoding	noncoding	noncoding
MSTRG.112458.1		XLOC_114604	227	coding	noncoding	noncoding	noncoding
MSTRG.112387.1	DPYSL2	XLOC_114611	393	coding	noncoding	noncoding	noncoding
MSTRG.112388.1	DPYSL2	XLOC_114612	259	coding	coding	noncoding	noncoding
XM_005273447.4	PTK2B	XLOC_114632	4079	coding	coding	coding	coding
MSTRG.112577.1	CHRNA2	XLOC_114655	433	coding	coding	noncoding	noncoding
MSTRG.112593.1	ESCO2	XLOC_114667	258	coding	noncoding	noncoding	noncoding
MSTRG.112632.1	ELP3	XLOC_114689	261	coding	noncoding	noncoding	noncoding
MSTRG.112638.1		XLOC_114708	278	coding	noncoding	noncoding	noncoding
MSTRG.112860.1	EXTL3	XLOC_114713	210	coding	noncoding	noncoding	noncoding
MSTRG.112672.1		XLOC_114755	287	coding	noncoding	noncoding	noncoding
MSTRG.112707.1	LINC02099	XLOC_114769	265	coding	noncoding	noncoding	noncoding
MSTRG.112741.1		XLOC_114780	312	coding	noncoding	noncoding	noncoding
MSTRG.112748.1	MBOAT4	XLOC_114785	281	coding	noncoding	noncoding	noncoding

MSTRG.112725.1		XLOC_114791	329 coding	noncoding	noncoding	noncoding
MSTRG.112734.1		XLOC_114794	258 coding	noncoding	noncoding	noncoding
MSTRG.112787.4	GSR	XLOC_114797	1693 coding	coding	coding	noncoding
MSTRG.112755.1	UBXN8	XLOC_114801	221 coding	noncoding	noncoding	noncoding
MSTRG.112761.1		XLOC_114807	220 coding	noncoding	noncoding	noncoding
MSTRG.112775.1	TEX15	XLOC_114810	281 coding	noncoding	noncoding	noncoding
MSTRG.112771.1		XLOC_114813	291 coding	noncoding	noncoding	noncoding
MSTRG.112824.1	WRN	XLOC_114825	492 coding	noncoding	noncoding	noncoding
MSTRG.112797.1		XLOC_114827	218 coding	noncoding	noncoding	noncoding
MSTRG.112805.1	LOC101929492	XLOC_114833	312 coding	noncoding	noncoding	noncoding
NM_013958.3	NRG1	XLOC_114838	1728 coding	coding	coding	coding
MSTRG.113096.1	NRG1	XLOC_114852	247 coding	noncoding	noncoding	noncoding
MSTRG.113101.1	NRG1	XLOC_114855	239 coding	noncoding	noncoding	noncoding
MSTRG.112851.1	LOC105379362	XLOC_114866	204 coding	noncoding	noncoding	noncoding
MSTRG.112856.1		XLOC_114871	274 coding	noncoding	noncoding	noncoding
MSTRG.112941.1	FUT10	XLOC_114879	518 coding	coding	noncoding	noncoding
MSTRG.112986.1	LOC105379364	XLOC_114894	370 coding	noncoding	noncoding	noncoding
MSTRG.112996.1	LOC105379364	XLOC_114897	240 coding	noncoding	noncoding	noncoding
MSTRG.112976.1		XLOC_114898	249 coding	coding	noncoding	noncoding
MSTRG.113008.1		XLOC_114908	265 coding	noncoding	noncoding	noncoding
MSTRG.113015.1		XLOC_114910	311 coding	noncoding	noncoding	noncoding
MSTRG.113146.1	UNC5D	XLOC_114922	233 coding	noncoding	noncoding	noncoding
MSTRG.113123.1		XLOC_114937	235 coding	noncoding	noncoding	noncoding
MSTRG.113160.1		XLOC_114946	293 coding	noncoding	noncoding	noncoding
NR_134267.1	KCNU1	XLOC_114952	3804 coding	coding	coding	coding
MSTRG.113179.1		XLOC_114958	311 coding	noncoding	noncoding	noncoding
MSTRG.113223.1		XLOC_114978	266 coding	noncoding	noncoding	noncoding
MSTRG.113269.1		XLOC_115001	287 coding	noncoding	noncoding	noncoding
MSTRG.113271.1		XLOC_115002	253 coding	noncoding	noncoding	noncoding
MSTRG.113325.24	NSD3	XLOC_115016	1143 coding	coding	noncoding	noncoding
MSTRG.113350.1	FGFR1	XLOC_115019	301 coding	noncoding	noncoding	noncoding
MSTRG.113317.1	LOC105379384	XLOC_115022	242 coding	noncoding	noncoding	noncoding
MSTRG.113499.1		XLOC_115043	261 coding	noncoding	noncoding	noncoding
MSTRG.113516.1	ADAM9	XLOC_115057	243 coding	noncoding	noncoding	noncoding
MSTRG.113369.1	ADAM32	XLOC_115063	527 coding	noncoding	noncoding	noncoding
MSTRG.113356.1	ADAM18	XLOC_115068	252 coding	noncoding	noncoding	noncoding
XR_949694.3	LOC105379385	XLOC_115079	1938 coding	noncoding	noncoding	noncoding
MSTRG.113399.1	LOC105379385	XLOC_115084	257 coding	noncoding	noncoding	noncoding
NM_020130.5	TCIM	XLOC_115085	1829 coding	coding	coding	noncoding
MSTRG.113406.1		XLOC_115091	271 coding	noncoding	noncoding	noncoding
MSTRG.113437.1	ZMAT4	XLOC_115095	257 coding	noncoding	noncoding	noncoding
MSTRG.113448.1	ZMAT4	XLOC_115106	200 coding	noncoding	noncoding	noncoding
NM_032336.3	GINS4	XLOC_115139	3770 coding	coding	coding	coding

MSTRG.113576.6	LOC105379393	XLOC_115159	3870	coding	coding	coding	noncoding
MSTRG.113576.8	LOC105379393	XLOC_115159	6337	coding	coding	coding	noncoding
MSTRG.113561.1		XLOC_115171	257	coding	noncoding	noncoding	noncoding
XM_005273491.5	IKBKB	XLOC_115172	3992	coding	coding	coding	coding
XR_949402.3	IKBKB	XLOC_115172	4346	coding	coding	coding	coding
MSTRG.113632.1	IKBKB	XLOC_115174	316	coding	noncoding	noncoding	noncoding
MSTRG.113617.5	SMIM19	XLOC_115186	706	coding	coding	coding	noncoding
NM_138436.4	SMIM19	XLOC_115186	3036	coding	coding	noncoding	noncoding
MSTRG.113596.1		XLOC_115192	274	coding	noncoding	noncoding	noncoding
MSTRG.113598.1		XLOC_115193	241	coding	noncoding	noncoding	noncoding
MSTRG.113682.1	LOC105379397	XLOC_115227	206	coding	noncoding	noncoding	noncoding
MSTRG.113679.1		XLOC_115228	281	coding	coding	noncoding	noncoding
MSTRG.113742.1		XLOC_115254	231	coding	noncoding	noncoding	noncoding
MSTRG.113960.2	SPIDR	XLOC_115266	5413	coding	coding	coding	coding
XR_001745508.2	SPIDR	XLOC_115266	3664	coding	coding	coding	coding
MSTRG.113765.1	MCM4	XLOC_115274	278	coding	noncoding	noncoding	noncoding
MSTRG.113783.1	UBE2V2	XLOC_115277	345	coding	noncoding	noncoding	noncoding
MSTRG.113767.1		XLOC_115279	304	coding	noncoding	noncoding	noncoding
MSTRG.113769.1		XLOC_115281	251	coding	noncoding	noncoding	noncoding
MSTRG.113770.1		XLOC_115282	220	coding	noncoding	noncoding	noncoding
MSTRG.113772.1		XLOC_115284	385	coding	noncoding	noncoding	noncoding
MSTRG.113777.1		XLOC_115286	309	coding	noncoding	noncoding	noncoding
MSTRG.113785.1		XLOC_115287	212	coding	noncoding	noncoding	noncoding
MSTRG.113796.1	LOC105375821	XLOC_115293	270	coding	noncoding	noncoding	noncoding
MSTRG.113792.1		XLOC_115298	240	coding	noncoding	noncoding	noncoding
MSTRG.113820.1	LOC105375825	XLOC_115314	331	coding	noncoding	noncoding	noncoding
XR_001745895.2	LOC107986942	XLOC_115317	3543	coding	noncoding	noncoding	noncoding
MSTRG.113838.1		XLOC_115320	228	coding	noncoding	noncoding	noncoding
MSTRG.113935.1	SNTG1	XLOC_115346	210	coding	noncoding	noncoding	noncoding
MSTRG.113952.1	SNTG1	XLOC_115352	224	coding	noncoding	noncoding	noncoding
MSTRG.113906.1		XLOC_115356	237	coding	noncoding	coding	noncoding
MSTRG.113922.1		XLOC_115364	318	coding	noncoding	noncoding	noncoding
MSTRG.114001.1	PXDNL	XLOC_115385	787	coding	coding	noncoding	noncoding
MSTRG.114039.1		XLOC_115406	304	coding	noncoding	noncoding	noncoding
MSTRG.114040.1		XLOC_115407	247	coding	noncoding	noncoding	noncoding
MSTRG.114044.1		XLOC_115410	248	coding	noncoding	noncoding	noncoding
MSTRG.114048.1		XLOC_115415	307	coding	noncoding	noncoding	noncoding
MSTRG.114067.1		XLOC_115425	327	coding	coding	noncoding	noncoding
MSTRG.114113.1		XLOC_115447	294	coding	noncoding	noncoding	noncoding
MSTRG.114123.1		XLOC_115448	359	coding	noncoding	noncoding	noncoding
NM_052898.2	XKR4	XLOC_115467	20241	coding	coding	coding	coding
MSTRG.114180.1		XLOC_115480	299	coding	noncoding	noncoding	noncoding
MSTRG.114257.1		XLOC_115526	269	coding	coding	noncoding	noncoding

MSTRG.114276.1		XLOC_115528	234	coding	noncoding	noncoding	noncoding
MSTRG.114277.1		XLOC_115529	288	coding	noncoding	noncoding	noncoding
MSTRG.114322.1		XLOC_115554	306	coding	noncoding	noncoding	noncoding
MSTRG.114324.1		XLOC_115555	255	coding	noncoding	noncoding	noncoding
MSTRG.114345.1	FAM110B	XLOC_115561	208	coding	noncoding	noncoding	noncoding
MSTRG.114355.1		XLOC_115567	304	coding	noncoding	noncoding	noncoding
NM_001007069.1	SDCBP	XLOC_115584	2164	coding	coding	coding	coding
MSTRG.114417.1		XLOC_115596	245	coding	coding	noncoding	noncoding
MSTRG.114449.1		XLOC_115607	278	coding	noncoding	noncoding	noncoding
MSTRG.114498.1	CA8	XLOC_115628	368	coding	coding	noncoding	noncoding
MSTRG.114486.1		XLOC_115638	559	coding	noncoding	coding	noncoding
MSTRG.114525.1	RAB2A	XLOC_115653	301	coding	noncoding	noncoding	noncoding
MSTRG.114505.1		XLOC_115662	238	coding	noncoding	coding	noncoding
MSTRG.115566.12	ASPH	XLOC_115704	714	coding	coding	coding	noncoding
MSTRG.114606.1	LINC02842	XLOC_115719	286	coding	noncoding	noncoding	noncoding
MSTRG.114579.1		XLOC_115722	319	coding	noncoding	noncoding	noncoding
XM_011517512.2	NKAIN3	XLOC_115724	10026	coding	coding	coding	coding
MSTRG.114632.1	NKAIN3	XLOC_115735	268	coding	noncoding	noncoding	noncoding
NR_027378.1	NKAIN3-IT1	XLOC_115738	7041	coding	noncoding	coding	coding
MSTRG.114644.1		XLOC_115755	246	coding	noncoding	noncoding	noncoding
MSTRG.114649.1		XLOC_115757	280	coding	noncoding	noncoding	noncoding
MSTRG.114651.1		XLOC_115758	200	coding	noncoding	noncoding	noncoding
MSTRG.114660.1		XLOC_115766	259	coding	noncoding	noncoding	noncoding
MSTRG.114703.1	LINC01414	XLOC_115776	302	coding	noncoding	noncoding	noncoding
MSTRG.114707.1	LINC01414	XLOC_115778	248	coding	noncoding	noncoding	noncoding
NM_152414.5	BHLHE22	XLOC_115787	3263	coding	coding	coding	coding
MSTRG.114721.1		XLOC_115819	257	coding	noncoding	noncoding	noncoding
MSTRG.114745.1		XLOC_115833	278	coding	noncoding	noncoding	noncoding
MSTRG.114832.5	PDE7A	XLOC_115843	3398	coding	coding	noncoding	noncoding
MSTRG.114803.1		XLOC_115856	219	coding	noncoding	noncoding	noncoding
MSTRG.114825.1	TRIM55	XLOC_115862	240	coding	noncoding	noncoding	noncoding
MSTRG.114920.2	VCPIP1	XLOC_115904	4432	coding	coding	coding	noncoding
MSTRG.114939.1	PTTG3P	XLOC_115910	228	coding	coding	noncoding	noncoding
MSTRG.114940.1	C8orf44-SGK3	XLOC_115911	264	coding	noncoding	noncoding	noncoding
MSTRG.114898.1		XLOC_115917	758	coding	noncoding	noncoding	noncoding
MSTRG.115073.2	COPS5	XLOC_115920	1278	coding	coding	noncoding	noncoding
MSTRG.115121.1	CPA6	XLOC_115935	291	coding	noncoding	noncoding	noncoding
NM_024870.4	PREX2	XLOC_115941	10824	coding	coding	coding	coding
MSTRG.114914.1	PREX2	XLOC_115943	216	coding	noncoding	noncoding	noncoding
MSTRG.114968.1	C8orf34	XLOC_115949	216	coding	noncoding	noncoding	noncoding
MSTRG.114955.1		XLOC_115950	291	coding	noncoding	noncoding	noncoding
MSTRG.114980.1		XLOC_115959	273	coding	coding	noncoding	noncoding
MSTRG.115009.1	SLCO5A1	XLOC_115972	602	coding	noncoding	noncoding	noncoding

MSTRG.115011.1	SLCO5A1	XLOC_115974	251	coding	noncoding	noncoding	noncoding
MSTRG.115017.1		XLOC_115979	269	coding	noncoding	noncoding	noncoding
MSTRG.115026.1		XLOC_115989	224	coding	noncoding	noncoding	noncoding
MSTRG.115034.1		XLOC_115990	215	coding	noncoding	noncoding	noncoding
XR_001745954.1	LOC105375894	XLOC_116012	6464	coding	noncoding	noncoding	noncoding
MSTRG.115062.1		XLOC_116016	270	coding	noncoding	noncoding	noncoding
MSTRG.115071.1		XLOC_116020	226	coding	noncoding	noncoding	noncoding
MSTRG.115189.1		XLOC_116037	230	coding	noncoding	noncoding	noncoding
XM_017013982.1	KCNB2	XLOC_116042	10233	coding	coding	coding	coding
MSTRG.115215.1	KCNB2	XLOC_116050	298	coding	noncoding	noncoding	noncoding
MSTRG.115218.1	KCNB2	XLOC_116053	226	coding	noncoding	noncoding	noncoding
MSTRG.115221.1	KCNB2	XLOC_116056	265	coding	noncoding	noncoding	noncoding
MSTRG.115226.1		XLOC_116067	258	coding	noncoding	noncoding	noncoding
MSTRG.115253.2	RPL7	XLOC_116072	1285	coding	coding	noncoding	noncoding
MSTRG.115263.1	RDH10-AS1	XLOC_116075	225	coding	noncoding	noncoding	noncoding
MSTRG.115411.4	STAU2	XLOC_116079	2171	coding	coding	coding	noncoding
MSTRG.115307.1		XLOC_116087	266	coding	noncoding	noncoding	noncoding
MSTRG.115466.1	LY96	XLOC_116098	663	coding	noncoding	noncoding	noncoding
MSTRG.115405.1	JPH1	XLOC_116104	274	coding	coding	noncoding	noncoding
MSTRG.115690.1	MIR2052HG	XLOC_116122	310	coding	noncoding	noncoding	noncoding
MSTRG.115694.1		XLOC_116126	304	coding	noncoding	noncoding	noncoding
MSTRG.115695.1		XLOC_116127	315	coding	noncoding	noncoding	noncoding
MSTRG.115724.1	CASC9	XLOC_116135	264	coding	coding	noncoding	noncoding
MSTRG.115741.1		XLOC_116146	450	coding	noncoding	noncoding	noncoding
MSTRG.115751.1		XLOC_116153	257	coding	coding	noncoding	noncoding
MSTRG.115763.1	LOC107986952	XLOC_116157	279	coding	noncoding	noncoding	noncoding
XM_011517594.2	ZFHX4	XLOC_116161	13723	coding	coding	noncoding	coding
MSTRG.115777.1	ZFHX4	XLOC_116162	312	coding	noncoding	noncoding	noncoding
MSTRG.115797.1		XLOC_116183	276	coding	noncoding	noncoding	noncoding
MSTRG.115803.1		XLOC_116186	257	coding	noncoding	noncoding	noncoding
MSTRG.115842.1		XLOC_116198	262	coding	coding	noncoding	noncoding
MSTRG.115858.1		XLOC_116294	222	coding	noncoding	noncoding	noncoding
MSTRG.115864.1		XLOC_116296	340	coding	noncoding	noncoding	noncoding
MSTRG.115874.1		XLOC_116302	206	coding	noncoding	noncoding	noncoding
MSTRG.115898.1	LOC101927040	XLOC_116314	268	coding	noncoding	noncoding	noncoding
MSTRG.115903.1	LOC105375920	XLOC_116325	281	coding	noncoding	noncoding	noncoding
MSTRG.115915.1	LOC105375922	XLOC_116328	305	coding	noncoding	noncoding	noncoding
MSTRG.115918.1	LOC105375922	XLOC_116329	202	coding	noncoding	noncoding	noncoding
MSTRG.115914.1		XLOC_116331	297	coding	noncoding	noncoding	noncoding
MSTRG.115913.1		XLOC_116332	262	coding	noncoding	noncoding	noncoding
MSTRG.115946.1	ZNF704	XLOC_116342	207	coding	noncoding	noncoding	noncoding
MSTRG.116232.1		XLOC_116387	293	coding	noncoding	noncoding	noncoding
MSTRG.116248.1		XLOC_116391	276	coding	noncoding	noncoding	noncoding

MSTRG.116258.1		XLOC_116398	226 coding	noncoding	noncoding	noncoding
MSTRG.116289.1	LOC105375931	XLOC_116410	380 coding	noncoding	noncoding	noncoding
MSTRG.116319.1		XLOC_116428	236 coding	coding	noncoding	noncoding
MSTRG.116324.1		XLOC_116433	311 coding	noncoding	noncoding	noncoding
MSTRG.116380.1	RALYL	XLOC_116457	250 coding	noncoding	noncoding	noncoding
MSTRG.116437.1		XLOC_116480	362 coding	noncoding	noncoding	coding
XM_005251121.2	LOC101929601	XLOC_116481	2084 coding	coding	coding	coding
MSTRG.116448.1		XLOC_116482	998 coding	noncoding	noncoding	noncoding
MSTRG.116459.1		XLOC_116487	217 coding	noncoding	noncoding	noncoding
NM_152565.1	ATP6V0D2	XLOC_116489	2370 coding	coding	coding	coding
MSTRG.116466.1	ATP6V0D2	XLOC_116490	281 coding	noncoding	noncoding	noncoding
MSTRG.116462.1	ATP6V0D2	XLOC_116491	279 coding	noncoding	noncoding	noncoding
MSTRG.116473.1	SLC7A13	XLOC_116494	238 coding	noncoding	noncoding	noncoding
MSTRG.116475.1		XLOC_116495	278 coding	noncoding	noncoding	noncoding
MSTRG.116477.1		XLOC_116496	383 coding	noncoding	noncoding	noncoding
MSTRG.116496.1	CNBD1	XLOC_116529	223 coding	noncoding	noncoding	noncoding
MSTRG.116511.1		XLOC_116542	363 coding	noncoding	coding	noncoding
MSTRG.116512.1		XLOC_116543	292 coding	noncoding	noncoding	noncoding
MSTRG.116522.1	MMP16	XLOC_116549	236 coding	noncoding	noncoding	noncoding
MSTRG.116553.1	LOC105375630	XLOC_116551	297 coding	noncoding	noncoding	noncoding
XM_011517357.2	RIPK2	XLOC_116597	1912 coding	coding	coding	coding
MSTRG.116700.1		XLOC_116688	259 coding	noncoding	noncoding	noncoding
NM_001129890.1	LRRC69	XLOC_116705	1323 coding	coding	coding	coding
NM_001282356.2	SLC26A7	XLOC_116705	5305 coding	coding	coding	coding
MSTRG.116885.1	LRRC69	XLOC_116709	300 coding	noncoding	noncoding	noncoding
MSTRG.116720.1		XLOC_116721	241 coding	noncoding	noncoding	noncoding
MSTRG.116723.1		XLOC_116723	313 coding	noncoding	noncoding	noncoding
MSTRG.116779.1	FLJ46284	XLOC_116735	284 coding	noncoding	noncoding	noncoding
MSTRG.116780.1	FLJ46284	XLOC_116736	307 coding	noncoding	noncoding	noncoding
MSTRG.116969.1	TMEM67	XLOC_116777	241 coding	noncoding	noncoding	noncoding
MSTRG.116985.1		XLOC_116786	294 coding	noncoding	noncoding	noncoding
MSTRG.117040.1		XLOC_116822	262 coding	noncoding	noncoding	noncoding
MSTRG.117046.1	ESRP1	XLOC_116825	294 coding	noncoding	noncoding	noncoding
MSTRG.117044.1		XLOC_116826	282 coding	noncoding	noncoding	noncoding
MSTRG.117050.1		XLOC_116828	299 coding	noncoding	noncoding	noncoding
MSTRG.117052.1		XLOC_116829	318 coding	noncoding	noncoding	noncoding
MSTRG.117132.1	NDUFAF6	XLOC_116838	284 coding	noncoding	noncoding	noncoding
MSTRG.117188.1	C8orf37-AS1	XLOC_116863	253 coding	coding	noncoding	noncoding
MSTRG.117208.1	C8orf37-AS1	XLOC_116867	221 coding	noncoding	noncoding	noncoding
MSTRG.117221.1	C8orf37-AS1	XLOC_116869	290 coding	noncoding	noncoding	noncoding
MSTRG.117089.1		XLOC_116870	270 coding	noncoding	noncoding	noncoding
MSTRG.117102.1		XLOC_116880	318 coding	noncoding	noncoding	noncoding
MSTRG.117237.1		XLOC_116892	266 coding	noncoding	noncoding	noncoding

MSTRG.117225.1		XLOC_116893	294 coding	noncoding	noncoding	noncoding
MSTRG.117685.1	LOC101927066	XLOC_116967	299 coding	noncoding	noncoding	noncoding
MSTRG.117694.1	LOC101927066	XLOC_116974	259 coding	noncoding	noncoding	noncoding
MSTRG.117255.1		XLOC_116977	309 coding	noncoding	noncoding	noncoding
MSTRG.117261.1		XLOC_116981	477 coding	noncoding	noncoding	noncoding
MSTRG.117264.1		XLOC_116991	291 coding	noncoding	noncoding	noncoding
MSTRG.117289.1	MATN2	XLOC_116996	277 coding	noncoding	noncoding	noncoding
MSTRG.117283.1	ERICH5	XLOC_117003	212 coding	noncoding	noncoding	noncoding
MSTRG.117529.1	STK3	XLOC_117023	351 coding	noncoding	noncoding	noncoding
MSTRG.117331.1		XLOC_117031	263 coding	noncoding	noncoding	noncoding
MSTRG.117344.1	RGS22	XLOC_117034	281 coding	coding	noncoding	noncoding
MSTRG.117346.1	RGS22	XLOC_117035	229 coding	noncoding	noncoding	noncoding
MSTRG.117335.1		XLOC_117038	271 coding	noncoding	noncoding	noncoding
MSTRG.117400.1	SNX31	XLOC_117064	370 coding	noncoding	noncoding	noncoding
MSTRG.117422.1		XLOC_117076	559 coding	noncoding	noncoding	noncoding
MSTRG.117445.11	YWHAZ	XLOC_117081	3315 coding	coding	noncoding	noncoding
MSTRG.117445.10	YWHAZ	XLOC_117081	3714 coding	coding	noncoding	noncoding
MSTRG.117430.1		XLOC_117082	266 coding	noncoding	noncoding	noncoding
MSTRG.117436.1		XLOC_117088	263 coding	noncoding	noncoding	noncoding
NR_002182.1	NACA4P	XLOC_117095	703 coding	coding	noncoding	coding
XM_024447286.1	GRHL2	XLOC_117099	3092 coding	noncoding	coding	coding
MSTRG.117551.1		XLOC_117115	459 coding	noncoding	noncoding	noncoding
MSTRG.117554.1		XLOC_117116	240 coding	noncoding	noncoding	noncoding
MSTRG.117562.1		XLOC_117121	212 coding	noncoding	noncoding	noncoding
MSTRG.117563.1		XLOC_117122	263 coding	noncoding	noncoding	noncoding
MSTRG.117804.1	AZIN1	XLOC_117131	4628 coding	coding	noncoding	noncoding
MSTRG.117709.1	LINC01181	XLOC_117150	461 coding	noncoding	noncoding	noncoding
MSTRG.117727.1		XLOC_117186	360 coding	noncoding	noncoding	noncoding
MSTRG.117597.1		XLOC_117188	269 coding	noncoding	noncoding	noncoding
NM_001348484.2	RIMS2	XLOC_117191	9375 coding	coding	coding	coding
MSTRG.117923.1	RIMS2	XLOC_117193	217 coding	noncoding	noncoding	noncoding
XM_011516947.3	ZFPM2	XLOC_117224	11424 coding	noncoding	coding	coding
MSTRG.117999.1	ZFPM2-AS1	XLOC_117233	229 coding	noncoding	noncoding	noncoding
XM_017013590.1	OXR1	XLOC_117239	4211 coding	coding	coding	coding
MSTRG.118160.1	OXR1	XLOC_117264	247 coding	noncoding	noncoding	noncoding
MSTRG.118009.1	ABRA	XLOC_117272	254 coding	noncoding	noncoding	noncoding
MSTRG.118015.1		XLOC_117275	276 coding	noncoding	noncoding	noncoding
MSTRG.118022.1		XLOC_117278	267 coding	noncoding	noncoding	noncoding
MSTRG.118043.1		XLOC_117288	292 coding	noncoding	noncoding	noncoding
MSTRG.118049.1		XLOC_117290	261 coding	noncoding	noncoding	noncoding
MSTRG.118054.1		XLOC_117291	294 coding	noncoding	noncoding	noncoding
MSTRG.118065.1	RSPO2	XLOC_117295	252 coding	noncoding	noncoding	noncoding
MSTRG.118070.1	RSPO2	XLOC_117298	243 coding	noncoding	noncoding	noncoding

MSTRG.118071.1	RSPO2	XLOC_117299	224 coding	noncoding	noncoding	noncoding
MSTRG.118060.1		XLOC_117300	284 coding	coding	noncoding	noncoding
MSTRG.118176.1		XLOC_117320	457 coding	noncoding	noncoding	noncoding
MSTRG.118308.1	PKHD1L1	XLOC_117361	344 coding	noncoding	noncoding	noncoding
MSTRG.118316.1	PKHD1L1	XLOC_117363	289 coding	noncoding	noncoding	noncoding
MSTRG.118273.1		XLOC_117373	251 coding	noncoding	noncoding	noncoding
MSTRG.118324.1		XLOC_117387	296 coding	noncoding	noncoding	noncoding
MSTRG.118383.1		XLOC_117410	268 coding	noncoding	noncoding	noncoding
MSTRG.118385.1		XLOC_117411	221 coding	noncoding	noncoding	noncoding
MSTRG.118410.1	CSMD3	XLOC_117412	318 coding	noncoding	noncoding	noncoding
MSTRG.118431.1	CSMD3	XLOC_117421	256 coding	coding	noncoding	noncoding
MSTRG.118434.1	CSMD3	XLOC_117422	213 coding	noncoding	noncoding	noncoding
MSTRG.118391.1		XLOC_117438	242 coding	noncoding	noncoding	noncoding
MSTRG.118466.1		XLOC_117447	511 coding	noncoding	noncoding	noncoding
MSTRG.118460.1		XLOC_117451	337 coding	noncoding	noncoding	noncoding
MSTRG.118472.1		XLOC_117454	280 coding	noncoding	noncoding	noncoding
MSTRG.118455.1		XLOC_117455	276 coding	noncoding	noncoding	noncoding
MSTRG.118501.1		XLOC_117469	250 coding	noncoding	noncoding	noncoding
MSTRG.118518.1		XLOC_117491	236 coding	noncoding	noncoding	noncoding
XR_002956724.1	LOC112268030	XLOC_117507	3497 coding	coding	noncoding	noncoding
MSTRG.118566.1		XLOC_117509	261 coding	noncoding	noncoding	noncoding
NM_001172811.2	SLC30A8	XLOC_117512	5372 coding	coding	coding	coding
MSTRG.118637.1	LOC105375716	XLOC_117523	359 coding	noncoding	coding	noncoding
MSTRG.118988.1	SAMD12	XLOC_117545	261 coding	noncoding	noncoding	noncoding
MSTRG.118721.1		XLOC_117636	348 coding	noncoding	noncoding	noncoding
MSTRG.118722.1		XLOC_117637	263 coding	noncoding	noncoding	noncoding
MSTRG.118732.1		XLOC_117648	255 coding	noncoding	noncoding	noncoding
MSTRG.118733.1		XLOC_117649	285 coding	noncoding	noncoding	noncoding
MSTRG.118738.1		XLOC_117653	238 coding	noncoding	noncoding	noncoding
MSTRG.118753.1	ENPP2	XLOC_117659	225 coding	noncoding	noncoding	noncoding
MSTRG.118757.1	ENPP2	XLOC_117663	686 coding	noncoding	noncoding	noncoding
MSTRG.118785.4	TAF2	XLOC_117667	478 coding	noncoding	noncoding	noncoding
NM_022783.4	DEPTOR	XLOC_117671	2569 coding	coding	coding	coding
MSTRG.118767.1	DEPTOR	XLOC_117673	346 coding	noncoding	noncoding	noncoding
MSTRG.118771.1	DEPTOR	XLOC_117674	422 coding	noncoding	noncoding	noncoding
XR_928593.3	LOC105375730	XLOC_117675	3227 coding	noncoding	noncoding	noncoding
XM_005251059.4	COL14A1	XLOC_117677	8819 coding	coding	coding	coding
MSTRG.118778.1	COL14A1	XLOC_117679	276 coding	noncoding	noncoding	noncoding
MSTRG.118877.1		XLOC_117706	282 coding	noncoding	noncoding	noncoding
MSTRG.118903.1		XLOC_117718	233 coding	noncoding	noncoding	noncoding
MSTRG.118925.1	HAS2	XLOC_117724	261 coding	noncoding	noncoding	noncoding
MSTRG.118931.1		XLOC_117727	300 coding	noncoding	noncoding	noncoding
MSTRG.119288.1	ZHX2	XLOC_117760	431 coding	noncoding	noncoding	noncoding

MSTRG.119306.1	ZHX2	XLOC_117777	231	coding	noncoding	noncoding	noncoding
MSTRG.119178.4	ZHX1	XLOC_117786	727	coding	coding	noncoding	noncoding
MSTRG.119209.1	KLHL38	XLOC_117798	304	coding	coding	noncoding	noncoding
MSTRG.119245.1	FER1L6-AS1	XLOC_117819	308	coding	noncoding	noncoding	noncoding
MSTRG.119249.1	FER1L6-AS2	XLOC_117822	231	coding	noncoding	noncoding	noncoding
MSTRG.119262.1	LOC101927588	XLOC_117830	221	coding	noncoding	noncoding	noncoding
MSTRG.119390.1	NDUFB9	XLOC_117854	281	coding	noncoding	noncoding	noncoding
MSTRG.119445.1	NSMCE2	XLOC_117892	294	coding	noncoding	noncoding	noncoding
MSTRG.119358.1		XLOC_117896	779	coding	noncoding	noncoding	noncoding
MSTRG.119377.1		XLOC_117936	775	coding	noncoding	coding	noncoding
MSTRG.119414.1		XLOC_117940	261	coding	noncoding	noncoding	noncoding
MSTRG.119411.1		XLOC_117942	272	coding	noncoding	noncoding	noncoding
MSTRG.120461.1	LOC105375751	XLOC_117957	244	coding	noncoding	noncoding	noncoding
MSTRG.119532.1		XLOC_118072	284	coding	noncoding	noncoding	noncoding
MSTRG.119650.1		XLOC_118135	227	coding	noncoding	noncoding	noncoding
MSTRG.119653.1		XLOC_118140	279	coding	noncoding	noncoding	noncoding
MSTRG.119670.1	LOC105375760	XLOC_118156	290	coding	noncoding	noncoding	noncoding
XM_011517076.1	PHF20L1	XLOC_118253	6247	coding	coding	coding	coding
MSTRG.120045.18	SLA	XLOC_118278	3797	coding	coding	coding	noncoding
MSTRG.119934.1		XLOC_118298	288	coding	noncoding	noncoding	noncoding
MSTRG.119970.1		XLOC_118312	225	coding	noncoding	noncoding	noncoding
MSTRG.120008.1		XLOC_118328	267	coding	noncoding	noncoding	noncoding
MSTRG.120009.1		XLOC_118329	293	coding	noncoding	noncoding	noncoding
MSTRG.120102.1		XLOC_118340	223	coding	noncoding	noncoding	noncoding
MSTRG.120118.1		XLOC_118341	248	coding	noncoding	noncoding	noncoding
NM_006558.3	KHDRBS3	XLOC_118347	1978	coding	coding	coding	coding
MSTRG.120237.1	LINC02055	XLOC_118370	231	coding	noncoding	noncoding	noncoding
MSTRG.120259.1	LINC02055	XLOC_118371	298	coding	noncoding	noncoding	noncoding
MSTRG.120265.1	LINC02055	XLOC_118375	271	coding	noncoding	noncoding	noncoding
MSTRG.120195.1		XLOC_118384	246	coding	noncoding	noncoding	noncoding
MSTRG.120200.1		XLOC_118386	323	coding	noncoding	noncoding	noncoding
MSTRG.120214.1	LOC401478	XLOC_118393	257	coding	noncoding	noncoding	noncoding
MSTRG.120215.1	LOC401478	XLOC_118394	308	coding	noncoding	noncoding	noncoding
MSTRG.120286.1	FAM135B	XLOC_118396	314	coding	noncoding	noncoding	noncoding
MSTRG.120345.1	COL22A1	XLOC_118409	237	coding	noncoding	noncoding	noncoding
MSTRG.120307.1		XLOC_118415	276	coding	noncoding	noncoding	noncoding
MSTRG.120310.1		XLOC_118416	261	coding	noncoding	noncoding	noncoding
MSTRG.120319.1		XLOC_118420	315	coding	noncoding	noncoding	noncoding
MSTRG.121056.2	TRAPPC9	XLOC_118429	3249	coding	coding	coding	coding
MSTRG.120718.3	AGO2	XLOC_118446	14316	coding	coding	coding	coding
MSTRG.120745.5	PTK2	XLOC_118449	344	coding	noncoding	noncoding	noncoding
MSTRG.120727.1	LOC105375783	XLOC_118451	247	coding	noncoding	noncoding	noncoding
MSTRG.120945.2	LOC105375787	XLOC_118467	8469	coding	coding	coding	coding

MSTRG.120807.1		XLOC_118522	257	coding	noncoding	noncoding	noncoding
MSTRG.120971.1		XLOC_118577	233	coding	noncoding	noncoding	noncoding
MSTRG.121000.1	ZC3H3	XLOC_118588	448	coding	noncoding	noncoding	noncoding
MSTRG.121115.1	PLEC	XLOC_118623	5695	coding	coding	coding	coding
MSTRG.121115.2	PLEC	XLOC_118623	874	coding	noncoding	coding	noncoding
NM_000837.1	GRINA	XLOC_118625	1968	coding	coding	coding	coding
MSTRG.121099.1	SPATC1	XLOC_118627	299	coding	noncoding	noncoding	noncoding
MSTRG.121105.1	OPLAH	XLOC_118631	333	coding	noncoding	coding	noncoding
MSTRG.121132.2	MAF1	XLOC_118641	2428	coding	coding	coding	noncoding
MSTRG.121135.3	WDR97	XLOC_118642	261	coding	noncoding	noncoding	noncoding
MSTRG.121128.3	HGH1	XLOC_118643	360	coding	noncoding	noncoding	noncoding
NM_032450.2	MROH1	XLOC_118645	5363	coding	coding	coding	coding
MSTRG.121196.8	PPP1R16A	XLOC_118666	285	coding	noncoding	noncoding	noncoding
MSTRG.121218.1	ARHGAP39	XLOC_118675	305	coding	noncoding	noncoding	noncoding
MSTRG.121225.4	RPL8	XLOC_118679	1009	coding	coding	coding	coding
XM_017013384.2	ZNF517	XLOC_118681	6252	coding	coding	coding	coding
XM_017013815.2	ZNF7	XLOC_118682	2801	coding	coding	coding	coding
XM_011534735.3	ERICH1	XLOC_118724	4724	coding	noncoding	coding	coding
MSTRG.110944.2	LOC107986865	XLOC_118769	947	coding	noncoding	noncoding	noncoding
MSTRG.111029.1	LOC105377791	XLOC_118791	311	coding	noncoding	noncoding	noncoding
MSTRG.111031.1	CSMD1	XLOC_118792	273	coding	noncoding	noncoding	noncoding
MSTRG.111036.1	CSMD1	XLOC_118794	297	coding	noncoding	noncoding	noncoding
MSTRG.111059.1	CSMD1	XLOC_118806	299	coding	noncoding	noncoding	noncoding
MSTRG.110964.1		XLOC_118818	243	coding	noncoding	noncoding	noncoding
MSTRG.110980.1		XLOC_118826	238	coding	noncoding	noncoding	noncoding
MSTRG.111009.1		XLOC_118844	371	coding	noncoding	noncoding	noncoding
XM_011534750.3	XKR5	XLOC_118858	3812	coding	coding	coding	coding
MSTRG.111082.1	GS1-24F4.2	XLOC_118859	312	coding	noncoding	coding	noncoding
NR_027000.1	LINC00965	XLOC_118878	2874	coding	noncoding	coding	noncoding
MSTRG.111404.1	GATA4	XLOC_119065	320	coding	noncoding	noncoding	noncoding
NM_147780.4	CTSB	XLOC_119068	3895	coding	coding	coding	coding
MSTRG.111410.1		XLOC_119069	270	coding	noncoding	noncoding	noncoding
XM_017013724.1	DEFB134	XLOC_119072	1806	coding	noncoding	noncoding	noncoding
MSTRG.111421.1	FAM86B1	XLOC_119080	289	coding	noncoding	noncoding	noncoding
MSTRG.111482.1		XLOC_119129	240	coding	noncoding	noncoding	noncoding
MSTRG.111476.1		XLOC_119132	229	coding	noncoding	noncoding	noncoding
MSTRG.111552.1	TUSC3	XLOC_119150	233	coding	coding	noncoding	noncoding
MSTRG.111589.1		XLOC_119176	323	coding	noncoding	noncoding	noncoding
MSTRG.111645.1	PDGFRL	XLOC_119214	251	coding	noncoding	noncoding	noncoding
NM_001001924.2	MTUS1	XLOC_119215	6419	coding	noncoding	coding	coding
MSTRG.111660.1	MTUS1	XLOC_119218	250	coding	noncoding	noncoding	noncoding
MSTRG.111662.1	MTUS1	XLOC_119220	257	coding	noncoding	noncoding	noncoding
MSTRG.111665.1	MTUS1	XLOC_119221	244	coding	noncoding	noncoding	noncoding

MSTRG.111764.12	PCM1	XLOC_119227	6470 coding	coding	coding	noncoding
MSTRG.111764.15	PCM1	XLOC_119227	7123 coding	coding	coding	noncoding
MSTRG.111706.1		XLOC_119245	465 coding	noncoding	noncoding	noncoding
MSTRG.111759.1	PSD3	XLOC_119253	217 coding	noncoding	noncoding	noncoding
MSTRG.111762.1	PSD3	XLOC_119254	271 coding	noncoding	noncoding	noncoding
MSTRG.111728.1	LOC105379300	XLOC_119260	209 coding	noncoding	noncoding	noncoding
MSTRG.111937.1	CSGALNACT1	XLOC_119265	293 coding	noncoding	noncoding	noncoding
MSTRG.111800.1	LOC107986921	XLOC_119304	260 coding	noncoding	noncoding	noncoding
NM_001362884.2	LZTS1	XLOC_119317	6010 coding	coding	coding	coding
MSTRG.111858.1	LOC105379315	XLOC_119326	272 coding	noncoding	noncoding	noncoding
MSTRG.111893.1		XLOC_119340	307 coding	noncoding	noncoding	noncoding
MSTRG.111976.1	NPM2	XLOC_119353	263 coding	noncoding	noncoding	noncoding
MSTRG.112003.1	BMP1	XLOC_119370	286 coding	noncoding	noncoding	noncoding
MSTRG.112004.1	BMP1	XLOC_119371	289 coding	coding	noncoding	noncoding
XM_017014102.2	PHYHIP	XLOC_119374	3476 coding	noncoding	coding	coding
NR_160769.1	LOC107986876	XLOC_119398	1868 coding	coding	coding	noncoding
MSTRG.112099.1	BIN3	XLOC_119405	227 coding	noncoding	noncoding	noncoding
NM_001363233.1	PEBP4	XLOC_119413	931 coding	coding	coding	coding
MSTRG.112077.1	LOC107986925	XLOC_119415	261 coding	noncoding	noncoding	noncoding
MSTRG.112309.1	ADAM7	XLOC_119502	243 coding	noncoding	noncoding	noncoding
MSTRG.112250.1		XLOC_119513	389 coding	noncoding	noncoding	noncoding
MSTRG.112256.1		XLOC_119516	275 coding	noncoding	noncoding	noncoding
MSTRG.112460.8	DOCK5	XLOC_119520	9669 coding	coding	coding	noncoding
MSTRG.112460.9	DOCK5	XLOC_119520	9435 coding	coding	coding	noncoding
MSTRG.112460.23	DOCK5	XLOC_119520	3661 coding	coding	noncoding	coding
MSTRG.112327.1	CDCA2	XLOC_119526	288 coding	noncoding	noncoding	noncoding
MSTRG.112340.1	LOC107986933	XLOC_119532	320 coding	noncoding	noncoding	noncoding
MSTRG.112349.1	EBF2	XLOC_119536	315 coding	noncoding	noncoding	noncoding
MSTRG.112389.1	DPYSL2	XLOC_119577	258 coding	coding	noncoding	noncoding
MSTRG.112379.1		XLOC_119579	262 coding	noncoding	noncoding	noncoding
XM_017013095.1	ADRA1A	XLOC_119580	16509 coding	coding	coding	coding
MSTRG.112395.1		XLOC_119587	521 coding	coding	coding	noncoding
MSTRG.112410.1	LOC105379340	XLOC_119592	248 coding	noncoding	noncoding	noncoding
XM_005273652.4	STMN4	XLOC_119594	1813 coding	coding	coding	coding
MSTRG.112581.1	SCARA3	XLOC_119613	217 coding	noncoding	noncoding	noncoding
MSTRG.112584.1	SCARA3	XLOC_119615	259 coding	noncoding	noncoding	noncoding
MSTRG.112601.1	NUGGC	XLOC_119630	333 coding	noncoding	noncoding	noncoding
MSTRG.112620.1	PNOC	XLOC_119637	273 coding	noncoding	noncoding	noncoding
MSTRG.112642.1	FBXO16	XLOC_119643	277 coding	noncoding	noncoding	noncoding
MSTRG.112859.1	EXTL3	XLOC_119658	305 coding	noncoding	noncoding	noncoding
MSTRG.112863.1	EXTL3	XLOC_119660	274 coding	noncoding	noncoding	noncoding
MSTRG.112866.1	EXTL3	XLOC_119662	314 coding	noncoding	noncoding	noncoding
MSTRG.112887.1	INTS9	XLOC_119677	211 coding	noncoding	noncoding	noncoding

MSTRG.112930.1	KIF13B	XLOC_119692	311	coding	noncoding	noncoding	noncoding
NM_057158.3	DUSP4	XLOC_119695	6436	coding	coding	coding	coding
MSTRG.112673.1		XLOC_119699	245	coding	noncoding	noncoding	noncoding
MSTRG.112690.1		XLOC_119710	445	coding	noncoding	noncoding	noncoding
MSTRG.112743.2	DCTN6	XLOC_119732	1074	coding	coding	coding	noncoding
MSTRG.112743.10	DCTN6	XLOC_119732	902	coding	coding	noncoding	noncoding
MSTRG.112724.1		XLOC_119736	321	coding	noncoding	noncoding	noncoding
MSTRG.112726.1		XLOC_119737	309	coding	noncoding	noncoding	noncoding
MSTRG.112735.1		XLOC_119743	258	coding	noncoding	noncoding	noncoding
MSTRG.112756.1	UBXN8	XLOC_119759	232	coding	coding	noncoding	noncoding
XM_011544593.3	TEX15	XLOC_119764	12140	coding	coding	coding	coding
MSTRG.112772.1		XLOC_119767	511	coding	noncoding	noncoding	noncoding
MSTRG.112806.1	LOC101929492	XLOC_119772	795	coding	noncoding	noncoding	noncoding
MSTRG.112808.1		XLOC_119774	250	coding	noncoding	noncoding	noncoding
MSTRG.112826.1		XLOC_119830	329	coding	noncoding	noncoding	noncoding
MSTRG.112846.1	LOC105379362	XLOC_119841	217	coding	noncoding	noncoding	noncoding
MSTRG.112947.1	FUT10	XLOC_119855	265	coding	noncoding	noncoding	noncoding
MSTRG.112952.1		XLOC_119857	218	coding	noncoding	noncoding	noncoding
MSTRG.112969.1	RNF122	XLOC_119863	404	coding	noncoding	noncoding	noncoding
MSTRG.112961.1		XLOC_119869	231	coding	noncoding	noncoding	noncoding
MSTRG.112972.1		XLOC_119873	282	coding	coding	noncoding	noncoding
MSTRG.112973.1		XLOC_119874	374	coding	noncoding	noncoding	noncoding
MSTRG.112985.1	LOC105379364	XLOC_119876	290	coding	noncoding	noncoding	noncoding
MSTRG.112993.1	LOC105379364	XLOC_119882	259	coding	noncoding	noncoding	noncoding
MSTRG.113002.1		XLOC_119891	305	coding	noncoding	noncoding	noncoding
MSTRG.113139.1	UNC5D	XLOC_119911	274	coding	noncoding	noncoding	noncoding
MSTRG.113145.1	UNC5D	XLOC_119915	233	coding	noncoding	noncoding	noncoding
NR_125819.1	LOC101929550	XLOC_119919	5391	coding	noncoding	coding	noncoding
MSTRG.113158.1	UNC5D	XLOC_119920	223	coding	noncoding	noncoding	noncoding
MSTRG.113116.1		XLOC_119926	314	coding	noncoding	noncoding	noncoding
MSTRG.113167.1		XLOC_119934	226	coding	coding	noncoding	noncoding
MSTRG.113168.1		XLOC_119939	288	coding	noncoding	noncoding	noncoding
MSTRG.113194.1	KCNU1	XLOC_119943	478	coding	noncoding	noncoding	noncoding
MSTRG.113195.1	KCNU1	XLOC_119944	202	coding	noncoding	noncoding	noncoding
MSTRG.113176.1		XLOC_119949	253	coding	noncoding	noncoding	noncoding
MSTRG.113222.1		XLOC_119964	266	coding	noncoding	noncoding	noncoding
MSTRG.113236.1	LINC01605	XLOC_119972	287	coding	noncoding	noncoding	noncoding
MSTRG.113282.1		XLOC_119991	235	coding	noncoding	noncoding	noncoding
MSTRG.113267.1	GOT1L1	XLOC_119999	219	coding	noncoding	noncoding	noncoding
MSTRG.113270.1		XLOC_120003	287	coding	noncoding	noncoding	noncoding
MSTRG.113272.1		XLOC_120004	241	coding	noncoding	noncoding	noncoding
MSTRG.113274.1		XLOC_120005	247	coding	noncoding	noncoding	noncoding
MSTRG.113296.1		XLOC_120009	255	coding	noncoding	noncoding	noncoding

MSTRG.113306.1	LOC105379382	XLOC_120014	251	coding	noncoding	noncoding	noncoding
MSTRG.113310.1		XLOC_120019	375	coding	noncoding	noncoding	noncoding
XM_011544674.2	PLPP5	XLOC_120021	1111	coding	noncoding	coding	coding
MSTRG.113340.1	NSD3	XLOC_120035	228	coding	noncoding	noncoding	noncoding
MSTRG.113344.3	LETM2	XLOC_120038	454	coding	noncoding	noncoding	noncoding
MSTRG.113349.1	FGFR1	XLOC_120042	301	coding	noncoding	noncoding	noncoding
MSTRG.113483.5	TACC1	XLOC_120052	2215	coding	noncoding	coding	coding
MSTRG.113378.1		XLOC_120071	321	coding	noncoding	noncoding	noncoding
MSTRG.113398.1	LOC105379385	XLOC_120075	257	coding	noncoding	noncoding	noncoding
MSTRG.113405.1		XLOC_120083	271	coding	noncoding	noncoding	noncoding
MSTRG.113450.1	ZMAT4	XLOC_120088	313	coding	noncoding	noncoding	noncoding
MSTRG.113464.1	ZMAT4	XLOC_120092	278	coding	noncoding	noncoding	noncoding
MSTRG.113416.1		XLOC_120101	323	coding	noncoding	noncoding	noncoding
MSTRG.113433.1		XLOC_120113	285	coding	coding	noncoding	noncoding
MSTRG.113434.1		XLOC_120114	242	coding	noncoding	noncoding	noncoding
XM_005273476.4	ANK1	XLOC_120123	8590	coding	coding	coding	coding
MSTRG.113543.39	LOC105379392	XLOC_120123	1550	coding	noncoding	noncoding	noncoding
MSTRG.113560.1		XLOC_120154	257	coding	noncoding	noncoding	noncoding
MSTRG.113568.1		XLOC_120155	236	coding	noncoding	noncoding	noncoding
MSTRG.113597.1		XLOC_120168	274	coding	noncoding	noncoding	noncoding
MSTRG.113606.1	CHRNB3	XLOC_120171	323	coding	noncoding	noncoding	noncoding
MSTRG.113601.1	CHRNA6	XLOC_120173	252	coding	noncoding	noncoding	noncoding
MSTRG.113599.1		XLOC_120174	230	coding	noncoding	noncoding	noncoding
MSTRG.113609.1		XLOC_120177	270	coding	noncoding	noncoding	noncoding
MSTRG.113611.1		XLOC_120179	287	coding	noncoding	noncoding	noncoding
NM_199003.1	THAP1	XLOC_120180	1971	coding	noncoding	coding	noncoding
MSTRG.113691.1		XLOC_120202	214	coding	coding	noncoding	noncoding
MSTRG.113740.1	ASNSP1	XLOC_120221	296	coding	coding	coding	noncoding
MSTRG.113754.1	LOC105375815	XLOC_120226	261	coding	noncoding	noncoding	noncoding
MSTRG.113759.1		XLOC_120235	296	coding	noncoding	noncoding	noncoding
MSTRG.113760.1		XLOC_120236	278	coding	noncoding	noncoding	noncoding
MSTRG.113959.4	SPIDR	XLOC_120238	2859	coding	coding	noncoding	noncoding
MSTRG.113782.2	UBE2V2	XLOC_120257	1193	coding	coding	noncoding	noncoding
MSTRG.113766.1		XLOC_120259	240	coding	noncoding	noncoding	noncoding
MSTRG.113801.1	LOC105375821	XLOC_120269	283	coding	noncoding	noncoding	noncoding
MSTRG.113821.1	LOC105375825	XLOC_120279	249	coding	noncoding	noncoding	noncoding
MSTRG.113837.1		XLOC_120285	228	coding	noncoding	noncoding	noncoding
MSTRG.113847.1		XLOC_120291	290	coding	noncoding	noncoding	noncoding
MSTRG.113875.1		XLOC_120299	393	coding	coding	noncoding	noncoding
MSTRG.113866.1		XLOC_120300	302	coding	noncoding	noncoding	noncoding
MSTRG.113890.1		XLOC_120307	232	coding	noncoding	noncoding	noncoding
MSTRG.113896.1		XLOC_120310	257	coding	noncoding	noncoding	noncoding
MSTRG.113926.1	SNTG1	XLOC_120312	369	coding	noncoding	noncoding	noncoding

MSTRG.113934.1	SNTG1	XLOC_120318	210 coding	noncoding	noncoding	noncoding
MSTRG.113937.1	SNTG1	XLOC_120319	435 coding	coding	noncoding	noncoding
MSTRG.113948.1	SNTG1	XLOC_120327	286 coding	noncoding	noncoding	noncoding
MSTRG.113921.1		XLOC_120344	231 coding	noncoding	noncoding	noncoding
MSTRG.114002.1	PCMTD1	XLOC_120358	701 coding	coding	noncoding	coding
MSTRG.114002.7	PCMTD1	XLOC_120358	862 coding	coding	noncoding	coding
MSTRG.113965.1		XLOC_120377	451 coding	noncoding	noncoding	noncoding
MSTRG.114092.1		XLOC_120399	777 coding	noncoding	noncoding	noncoding
MSTRG.114053.1		XLOC_120410	271 coding	noncoding	noncoding	noncoding
MSTRG.114056.1		XLOC_120413	248 coding	noncoding	noncoding	noncoding
NR_149032.1	LOC100507516	XLOC_120415	5813 coding	noncoding	noncoding	noncoding
MSTRG.114066.1		XLOC_120416	271 coding	coding	noncoding	noncoding
MSTRG.114106.1	TCEA1	XLOC_120446	271 coding	noncoding	noncoding	noncoding
MSTRG.114137.1	RP1	XLOC_120462	242 coding	noncoding	noncoding	noncoding
MSTRG.114129.1		XLOC_120464	415 coding	noncoding	noncoding	noncoding
MSTRG.114197.1	XKR4	XLOC_120474	299 coding	noncoding	noncoding	noncoding
MSTRG.114368.1		XLOC_120486	252 coding	noncoding	noncoding	noncoding
MSTRG.114231.1		XLOC_120507	332 coding	noncoding	noncoding	noncoding
NM_001318049.1	SDR16C5	XLOC_120509	3641 coding	coding	coding	coding
MSTRG.114233.1		XLOC_120512	269 coding	coding	noncoding	noncoding
MSTRG.114236.1		XLOC_120513	285 coding	noncoding	noncoding	noncoding
MSTRG.114263.1	LOC101929415	XLOC_120521	273 coding	noncoding	noncoding	noncoding
MSTRG.114258.1		XLOC_120532	419 coding	coding	noncoding	noncoding
MSTRG.114278.1		XLOC_120535	288 coding	noncoding	noncoding	noncoding
NM_017813.5	IMPAD1	XLOC_120539	7224 coding	coding	coding	coding
MSTRG.114290.1	LOC107986888	XLOC_120544	201 coding	noncoding	noncoding	noncoding
MSTRG.114301.1		XLOC_120549	247 coding	noncoding	noncoding	noncoding
MSTRG.114321.1		XLOC_120557	306 coding	noncoding	noncoding	noncoding
MSTRG.114328.1	LOC105375856	XLOC_120560	277 coding	noncoding	noncoding	noncoding
MSTRG.114329.1	LOC105375856	XLOC_120561	288 coding	noncoding	noncoding	noncoding
MSTRG.114402.1		XLOC_120590	225 coding	noncoding	noncoding	noncoding
MSTRG.114439.1		XLOC_120642	293 coding	noncoding	noncoding	noncoding
MSTRG.114461.1		XLOC_120654	264 coding	noncoding	noncoding	noncoding
MSTRG.114484.1	LOC105375864	XLOC_120666	244 coding	noncoding	noncoding	noncoding
MSTRG.114485.1		XLOC_120670	305 coding	noncoding	noncoding	noncoding
MSTRG.115597.1	CLVS1	XLOC_120735	262 coding	noncoding	noncoding	noncoding
MSTRG.115599.1	CLVS1	XLOC_120737	282 coding	noncoding	noncoding	noncoding
MSTRG.115641.1	ASPH	XLOC_120778	268 coding	noncoding	noncoding	noncoding
MSTRG.114580.1		XLOC_120791	260 coding	noncoding	noncoding	noncoding
MSTRG.114631.1	NKAIN3	XLOC_120803	268 coding	noncoding	noncoding	noncoding
MSTRG.114636.1	NKAIN3	XLOC_120805	278 coding	noncoding	noncoding	noncoding
MSTRG.114638.1	NKAIN3	XLOC_120807	276 coding	noncoding	noncoding	noncoding
MSTRG.114589.1	TTPA	XLOC_120813	253 coding	noncoding	noncoding	noncoding

MSTRG.114643.1		XLOC_120822	246	coding	noncoding	noncoding	noncoding
MSTRG.114657.1		XLOC_120830	339	coding	noncoding	noncoding	noncoding
MSTRG.114659.1		XLOC_120832	259	coding	noncoding	noncoding	noncoding
MSTRG.114661.1		XLOC_120833	250	coding	coding	noncoding	noncoding
MSTRG.114701.1	LINC01414	XLOC_120843	375	coding	noncoding	noncoding	noncoding
MSTRG.114704.1	LINC01414	XLOC_120844	277	coding	noncoding	noncoding	noncoding
MSTRG.114708.1	LINC01414	XLOC_120846	272	coding	noncoding	noncoding	noncoding
MSTRG.114693.1		XLOC_120850	224	coding	noncoding	noncoding	noncoding
MSTRG.114732.1		XLOC_120865	258	coding	noncoding	noncoding	noncoding
MSTRG.114739.1		XLOC_120869	261	coding	noncoding	noncoding	noncoding
MSTRG.114744.1		XLOC_120872	600	coding	noncoding	noncoding	noncoding
MSTRG.114804.1		XLOC_120889	219	coding	noncoding	noncoding	noncoding
MSTRG.114820.1	DNAJC5B	XLOC_120894	253	coding	noncoding	noncoding	noncoding
MSTRG.114858.1	LOC102724687	XLOC_120905	360	coding	noncoding	noncoding	noncoding
MSTRG.114882.1	VXN	XLOC_120912	248	coding	noncoding	noncoding	noncoding
MSTRG.114945.1	MCMDC2	XLOC_120937	482	coding	noncoding	noncoding	noncoding
MSTRG.114899.1	PPP1R42	XLOC_120944	258	coding	noncoding	noncoding	noncoding
MSTRG.115101.1	LOC102724708	XLOC_120972	360	coding	noncoding	noncoding	noncoding
MSTRG.114906.1		XLOC_120981	248	coding	noncoding	noncoding	noncoding
MSTRG.114987.1	LINC01603	XLOC_121009	548	coding	noncoding	noncoding	coding
MSTRG.114995.1	SULF1	XLOC_121012	271	coding	noncoding	noncoding	noncoding
MSTRG.115000.1	SULF1	XLOC_121015	226	coding	noncoding	noncoding	noncoding
XM_017013883.1	SLCO5A1	XLOC_121016	9012	coding	coding	coding	coding
MSTRG.115014.1		XLOC_121019	346	coding	noncoding	noncoding	noncoding
MSTRG.115016.1		XLOC_121021	269	coding	noncoding	noncoding	noncoding
MSTRG.115018.1		XLOC_121022	246	coding	noncoding	noncoding	noncoding
XM_017013267.2	TRAM1	XLOC_121117	2908	coding	coding	noncoding	coding
MSTRG.115165.1	XKR9	XLOC_121145	320	coding	noncoding	noncoding	noncoding
MSTRG.115059.1		XLOC_121147	216	coding	noncoding	noncoding	noncoding
MSTRG.115134.1	EYA1	XLOC_121157	311	coding	noncoding	noncoding	noncoding
MSTRG.115136.1	EYA1	XLOC_121159	255	coding	noncoding	noncoding	noncoding
MSTRG.115063.1		XLOC_121161	270	coding	noncoding	noncoding	noncoding
MSTRG.115070.1		XLOC_121166	226	coding	noncoding	noncoding	noncoding
MSTRG.115072.1		XLOC_121167	351	coding	noncoding	noncoding	noncoding
MSTRG.115194.1	LOC392232	XLOC_121218	302	coding	noncoding	noncoding	noncoding
MSTRG.115214.1	KCNB2	XLOC_121227	298	coding	noncoding	noncoding	noncoding
MSTRG.115251.1	C8orf89	XLOC_121242	233	coding	noncoding	noncoding	noncoding
NR_073121.2	UBE2W	XLOC_121274	8275	coding	coding	noncoding	noncoding
MSTRG.115697.1		XLOC_121365	308	coding	noncoding	noncoding	noncoding
MSTRG.115691.1	MIR2052HG	XLOC_121371	310	coding	noncoding	noncoding	noncoding
MSTRG.115720.1		XLOC_121385	253	coding	noncoding	noncoding	noncoding
MSTRG.115723.1	CASC9	XLOC_121387	343	coding	coding	noncoding	noncoding
MSTRG.115738.1		XLOC_121393	853	coding	noncoding	noncoding	noncoding

MSTRG.115744.1		XLOC_121396	307 coding	noncoding	noncoding	noncoding
MSTRG.115756.1	LOC102724858	XLOC_121402	394 coding	noncoding	noncoding	noncoding
MSTRG.115798.1		XLOC_121425	234 coding	noncoding	noncoding	noncoding
MSTRG.115801.1		XLOC_121426	292 coding	noncoding	noncoding	noncoding
MSTRG.115823.1		XLOC_121434	290 coding	noncoding	noncoding	noncoding
MSTRG.115828.1		XLOC_121436	304 coding	coding	noncoding	noncoding
MSTRG.115829.1		XLOC_121438	208 coding	noncoding	noncoding	noncoding
MSTRG.115837.1		XLOC_121442	293 coding	noncoding	noncoding	noncoding
MSTRG.116054.1	LOC105375911	XLOC_121459	259 coding	noncoding	noncoding	noncoding
MSTRG.115863.1		XLOC_121513	340 coding	noncoding	noncoding	noncoding
MSTRG.115865.1		XLOC_121514	319 coding	noncoding	noncoding	noncoding
MSTRG.115873.1		XLOC_121518	512 coding	noncoding	noncoding	noncoding
MSTRG.115899.1	LOC101927040	XLOC_121530	268 coding	noncoding	noncoding	noncoding
MSTRG.115995.1	MRPS28	XLOC_121542	315 coding	noncoding	noncoding	noncoding
MSTRG.115906.1		XLOC_121554	266 coding	noncoding	noncoding	noncoding
MSTRG.115911.1		XLOC_121558	300 coding	noncoding	noncoding	noncoding
MSTRG.115920.1		XLOC_121566	682 coding	noncoding	noncoding	noncoding
MSTRG.115947.1	ZNF704	XLOC_121574	308 coding	noncoding	noncoding	noncoding
MSTRG.116174.1		XLOC_121648	290 coding	noncoding	noncoding	noncoding
MSTRG.116230.1	IMPA1	XLOC_121655	283 coding	noncoding	noncoding	noncoding
MSTRG.116218.1		XLOC_121664	217 coding	noncoding	noncoding	noncoding
MSTRG.116247.1		XLOC_121672	410 coding	noncoding	noncoding	noncoding
MSTRG.116270.1		XLOC_121684	279 coding	noncoding	noncoding	noncoding
MSTRG.116318.1		XLOC_121701	238 coding	coding	noncoding	noncoding
MSTRG.116331.1		XLOC_121706	323 coding	noncoding	noncoding	noncoding
MSTRG.116332.1		XLOC_121707	217 coding	noncoding	noncoding	noncoding
MSTRG.116340.1		XLOC_121712	271 coding	noncoding	noncoding	noncoding
MSTRG.116353.1	RALYL	XLOC_121714	271 coding	noncoding	noncoding	noncoding
MSTRG.116381.1	RALYL	XLOC_121728	273 coding	noncoding	noncoding	noncoding
MSTRG.116350.1		XLOC_121735	301 coding	noncoding	noncoding	noncoding
NM_001738.5	CA1	XLOC_121757	1884 coding	coding	coding	coding
MSTRG.116431.1		XLOC_121776	217 coding	noncoding	noncoding	noncoding
XM_005251122.3	LOC101929627	XLOC_121779	2028 coding	coding	coding	coding
NR_003594.1	REXO1L2P	XLOC_121780	1078 coding	coding	coding	coding
XM_002342862.6	LOC100288562	XLOC_121781	2028 coding	coding	coding	coding
MSTRG.116449.1		XLOC_121782	1473 coding	noncoding	coding	coding
MSTRG.116465.1	ATP6V0D2	XLOC_121789	229 coding	noncoding	noncoding	noncoding
MSTRG.116463.1		XLOC_121791	279 coding	noncoding	noncoding	noncoding
MSTRG.116474.1		XLOC_121795	329 coding	noncoding	noncoding	noncoding
MSTRG.116483.1	CNGB3	XLOC_121805	430 coding	noncoding	noncoding	noncoding
MSTRG.116502.1	CNBD1	XLOC_121813	320 coding	noncoding	noncoding	noncoding
XM_024447154.1	MMP16	XLOC_121820	19061 coding	coding	coding	coding
MSTRG.116523.1	MMP16	XLOC_121823	236 coding	noncoding	noncoding	noncoding

MSTRG.116554.1	LOC105375630	XLOC_121832	297 coding	noncoding	noncoding	noncoding
MSTRG.116568.1	LOC105375631	XLOC_121839	224 coding	noncoding	noncoding	noncoding
MSTRG.116577.1	LOC105375631	XLOC_121846	234 coding	noncoding	noncoding	noncoding
MSTRG.116538.1		XLOC_121859	261 coding	noncoding	noncoding	noncoding
MSTRG.116651.7	DECR1	XLOC_121867	2554 coding	coding	coding	noncoding
NM_001363275.1	C8orf88	XLOC_121902	1002 coding	noncoding	noncoding	noncoding
MSTRG.116717.1		XLOC_121943	314 coding	noncoding	noncoding	noncoding
MSTRG.116721.1		XLOC_121945	241 coding	noncoding	noncoding	noncoding
MSTRG.116733.1		XLOC_121955	340 coding	noncoding	coding	noncoding
MSTRG.116738.1		XLOC_121956	220 coding	noncoding	noncoding	noncoding
MSTRG.116740.1		XLOC_121958	289 coding	noncoding	noncoding	noncoding
MSTRG.116922.1	LOC107986956	XLOC_121984	305 coding	noncoding	noncoding	noncoding
MSTRG.116968.1	TMEM67	XLOC_122007	247 coding	noncoding	noncoding	noncoding
MSTRG.116972.1		XLOC_122009	238 coding	noncoding	noncoding	noncoding
MSTRG.116984.1		XLOC_122021	299 coding	noncoding	noncoding	noncoding
MSTRG.116992.1		XLOC_122034	339 coding	noncoding	noncoding	noncoding
NM_183009.2	VIRMA	XLOC_122044	3668 coding	coding	coding	coding
MSTRG.117047.1	ESRP1	XLOC_122054	297 coding	noncoding	noncoding	noncoding
MSTRG.117048.1	ESRP1	XLOC_122055	263 coding	noncoding	noncoding	noncoding
MSTRG.117049.1		XLOC_122061	299 coding	noncoding	noncoding	noncoding
MSTRG.117131.1	NDUFAF6	XLOC_122079	284 coding	noncoding	noncoding	noncoding
MSTRG.117086.1		XLOC_122100	396 coding	noncoding	noncoding	noncoding
MSTRG.117205.1	C8orf37-AS1	XLOC_122139	263 coding	noncoding	coding	noncoding
MSTRG.117210.1	C8orf37-AS1	XLOC_122142	236 coding	noncoding	noncoding	noncoding
NR_045639.1	UQCRB	XLOC_122163	5109 coding	coding	coding	noncoding
MSTRG.117236.1		XLOC_122170	1244 coding	noncoding	noncoding	noncoding
MSTRG.117256.1		XLOC_122207	309 coding	noncoding	noncoding	noncoding
MSTRG.117265.1		XLOC_122211	279 coding	noncoding	noncoding	noncoding
MSTRG.117266.1		XLOC_122213	224 coding	noncoding	noncoding	noncoding
MSTRG.117290.1	MATN2	XLOC_122216	277 coding	noncoding	noncoding	noncoding
MSTRG.117278.1		XLOC_122223	244 coding	noncoding	noncoding	noncoding
MSTRG.117279.1		XLOC_122224	303 coding	noncoding	noncoding	noncoding
MSTRG.117282.1	ERICH5	XLOC_122226	461 coding	noncoding	noncoding	noncoding
XM_017013756.1	STK3	XLOC_122244	3705 coding	coding	coding	coding
XR_001745585.1	STK3	XLOC_122244	2779 coding	coding	coding	coding
MSTRG.117525.1	STK3	XLOC_122269	248 coding	noncoding	noncoding	noncoding
MSTRG.117329.1		XLOC_122279	213 coding	noncoding	noncoding	noncoding
MSTRG.117579.1	VPS13B-DT	XLOC_122280	14413 coding	coding	noncoding	coding
MSTRG.117330.1		XLOC_122297	263 coding	noncoding	noncoding	noncoding
MSTRG.117345.1	RGS22	XLOC_122301	281 coding	coding	noncoding	noncoding
MSTRG.117347.1	RGS22	XLOC_122302	225 coding	noncoding	noncoding	noncoding
MSTRG.117334.1		XLOC_122303	276 coding	coding	noncoding	noncoding
MSTRG.117350.1		XLOC_122318	237 coding	noncoding	noncoding	noncoding

MSTRG.117356.1	LOC105375670	XLOC_122320	268 coding	noncoding	noncoding	noncoding
MSTRG.117399.1	SNX31	XLOC_122331	303 coding	noncoding	noncoding	noncoding
MSTRG.117404.1	SNX31	XLOC_122333	431 coding	noncoding	noncoding	noncoding
MSTRG.117362.1		XLOC_122334	317 coding	noncoding	noncoding	noncoding
MSTRG.117431.1		XLOC_122361	217 coding	noncoding	noncoding	noncoding
NM_001042510.1	ZNF706	XLOC_122374	2860 coding	coding	noncoding	noncoding
XM_024447304.1	NCALD	XLOC_122391	4763 coding	coding	coding	coding
MSTRG.117552.1		XLOC_122463	285 coding	noncoding	noncoding	noncoding
MSTRG.117553.1		XLOC_122464	250 coding	noncoding	noncoding	noncoding
NM_005655.4	KLF10	XLOC_122471	2915 coding	coding	coding	coding
MSTRG.117865.1		XLOC_122529	271 coding	noncoding	noncoding	noncoding
MSTRG.117728.1		XLOC_122538	214 coding	noncoding	noncoding	noncoding
NR_145697.1	LOC105375690	XLOC_122540	3068 coding	noncoding	coding	noncoding
MSTRG.117924.1	RIMS2	XLOC_122545	217 coding	noncoding	noncoding	noncoding
MSTRG.117931.1	RIMS2	XLOC_122549	299 coding	noncoding	noncoding	noncoding
MSTRG.117883.1		XLOC_122562	207 coding	noncoding	noncoding	noncoding
MSTRG.117899.1		XLOC_122575	224 coding	noncoding	noncoding	noncoding
MSTRG.117974.1		XLOC_122611	857 coding	noncoding	noncoding	noncoding
MSTRG.118005.1		XLOC_122655	271 coding	noncoding	noncoding	noncoding
MSTRG.118017.1		XLOC_122660	262 coding	coding	noncoding	noncoding
MSTRG.118021.1		XLOC_122663	267 coding	noncoding	noncoding	noncoding
MSTRG.118025.1		XLOC_122672	436 coding	noncoding	noncoding	noncoding
MSTRG.118029.1		XLOC_122674	303 coding	noncoding	noncoding	noncoding
MSTRG.118053.1		XLOC_122692	294 coding	noncoding	noncoding	noncoding
MSTRG.118067.1	RSPO2	XLOC_122695	279 coding	noncoding	noncoding	noncoding
MSTRG.118059.1		XLOC_122698	284 coding	coding	noncoding	noncoding
MSTRG.118187.1	LOC105375704	XLOC_122711	247 coding	noncoding	noncoding	noncoding
MSTRG.118212.1	TMEM74	XLOC_122733	213 coding	coding	noncoding	noncoding
MSTRG.118262.1	NUDCD1	XLOC_122753	391 coding	noncoding	noncoding	noncoding
MSTRG.118274.1		XLOC_122780	236 coding	noncoding	noncoding	noncoding
MSTRG.118325.1		XLOC_122794	296 coding	noncoding	noncoding	noncoding
MSTRG.118329.1		XLOC_122795	289 coding	noncoding	noncoding	noncoding
MSTRG.118376.1		XLOC_122821	275 coding	noncoding	noncoding	noncoding
MSTRG.118432.1	CSMD3	XLOC_122839	256 coding	coding	noncoding	noncoding
MSTRG.118440.1	CSMD3	XLOC_122843	317 coding	noncoding	noncoding	noncoding
MSTRG.118402.1		XLOC_122856	314 coding	noncoding	noncoding	noncoding
MSTRG.118660.1	TRPS1	XLOC_122902	302 coding	noncoding	noncoding	noncoding
MSTRG.118536.1		XLOC_122956	236 coding	noncoding	noncoding	noncoding
MSTRG.118580.1		XLOC_123007	214 coding	noncoding	noncoding	noncoding
MSTRG.118603.1		XLOC_123018	218 coding	noncoding	noncoding	noncoding
MSTRG.118702.1		XLOC_123059	203 coding	noncoding	noncoding	noncoding
MSTRG.118739.1		XLOC_123127	238 coding	noncoding	noncoding	noncoding
MSTRG.118741.1		XLOC_123129	255 coding	noncoding	noncoding	noncoding

MSTRG.118747.1		XLOC_123132	207 coding	noncoding	noncoding	noncoding
MSTRG.118769.1	DEPTOR	XLOC_123146	263 coding	noncoding	noncoding	noncoding
MSTRG.118776.1	COL14A1	XLOC_123151	306 coding	noncoding	noncoding	noncoding
MSTRG.118773.1		XLOC_123153	304 coding	noncoding	noncoding	noncoding
MSTRG.119125.1	SNTB1	XLOC_123172	242 coding	noncoding	noncoding	noncoding
MSTRG.118875.1		XLOC_123203	648 coding	coding	noncoding	noncoding
MSTRG.118915.1		XLOC_123226	221 coding	noncoding	noncoding	noncoding
MSTRG.118920.1		XLOC_123229	284 coding	noncoding	noncoding	noncoding
MSTRG.118921.1		XLOC_123230	289 coding	noncoding	noncoding	noncoding
MSTRG.118935.1		XLOC_123237	237 coding	noncoding	noncoding	noncoding
MSTRG.118944.1		XLOC_123243	314 coding	noncoding	noncoding	noncoding
MSTRG.118945.1		XLOC_123244	292 coding	noncoding	noncoding	noncoding
MSTRG.118957.1		XLOC_123262	276 coding	noncoding	noncoding	noncoding
MSTRG.118963.1	LOC107986904	XLOC_123266	315 coding	noncoding	noncoding	noncoding
MSTRG.119156.1		XLOC_123271	307 coding	noncoding	noncoding	noncoding
MSTRG.119191.1	WDYHV1	XLOC_123291	1080 coding	noncoding	noncoding	noncoding
MSTRG.119196.1		XLOC_123304	278 coding	noncoding	noncoding	noncoding
MSTRG.119206.1		XLOC_123305	238 coding	noncoding	noncoding	noncoding
MSTRG.119252.1	FER1L6	XLOC_123320	288 coding	noncoding	noncoding	noncoding
NM_001363295.1	MTSS1	XLOC_123353	4957 coding	coding	coding	coding
MSTRG.119393.1	MTSS1	XLOC_123357	285 coding	noncoding	noncoding	noncoding
XM_017014113.2	WASHC5	XLOC_123384	4366 coding	coding	coding	coding
MSTRG.119451.1	LOC105375746	XLOC_123391	231 coding	noncoding	noncoding	noncoding
MSTRG.119461.1	LOC105375746	XLOC_123396	270 coding	noncoding	noncoding	noncoding
MSTRG.119480.1		XLOC_123409	267 coding	noncoding	noncoding	noncoding
MSTRG.119527.1		XLOC_123702	311 coding	noncoding	noncoding	noncoding
MSTRG.119533.1		XLOC_123709	284 coding	noncoding	noncoding	noncoding
MSTRG.119562.1		XLOC_123723	423 coding	noncoding	noncoding	noncoding
MSTRG.119592.1		XLOC_123729	272 coding	noncoding	noncoding	noncoding
NR_130917.1	CCDC26	XLOC_123731	1718 coding	noncoding	noncoding	noncoding
MSTRG.119597.1	CCDC26	XLOC_123732	241 coding	noncoding	noncoding	noncoding
NM_016623.5	FAM49B	XLOC_123741	3777 coding	coding	coding	coding
NM_001353248.2	FAM49B	XLOC_123741	3900 coding	coding	coding	coding
MSTRG.119651.1		XLOC_123824	303 coding	noncoding	noncoding	noncoding
MSTRG.119654.1		XLOC_123827	348 coding	noncoding	noncoding	noncoding
MSTRG.119669.1		XLOC_123833	276 coding	noncoding	noncoding	noncoding
MSTRG.119691.1	LOC105375760	XLOC_123839	328 coding	noncoding	noncoding	noncoding
MSTRG.119718.1		XLOC_123849	250 coding	noncoding	noncoding	noncoding
MSTRG.119751.1	LOC107986976	XLOC_123855	351 coding	noncoding	noncoding	noncoding
XM_017013400.1	KCNQ3	XLOC_123862	20754 coding	coding	coding	coding
NM_144649.3	TMEM71	XLOC_123883	2000 coding	coding	coding	coding
MSTRG.120012.17	PHF20L1	XLOC_123886	410 coding	noncoding	coding	noncoding
MSTRG.120012.13	PHF20L1	XLOC_123886	2397 coding	coding	coding	noncoding

MSTRG.119935.1		XLOC_123951	288 coding	noncoding	noncoding	noncoding
NM_003033.4	ST3GAL1	XLOC_123953	6918 coding	coding	coding	coding
MSTRG.119969.1		XLOC_123991	301 coding	noncoding	noncoding	noncoding
MSTRG.120122.1	LINC01591	XLOC_124026	293 coding	noncoding	noncoding	noncoding
MSTRG.120144.1		XLOC_124047	242 coding	noncoding	noncoding	noncoding
MSTRG.120148.1		XLOC_124050	255 coding	coding	noncoding	noncoding
MSTRG.120183.1		XLOC_124101	237 coding	noncoding	noncoding	noncoding
MSTRG.120199.1		XLOC_124111	323 coding	noncoding	noncoding	noncoding
NR_161374.1	LOC401478	XLOC_124115	4518 coding	noncoding	coding	coding
NM_015912.4	FAM135B	XLOC_124121	7401 coding	coding	coding	coding
MSTRG.120294.1	FAM135B	XLOC_124127	290 coding	noncoding	noncoding	noncoding
MSTRG.120344.1	COL22A1	XLOC_124136	237 coding	noncoding	noncoding	noncoding
MSTRG.120346.1	COL22A1	XLOC_124137	226 coding	noncoding	noncoding	noncoding
MSTRG.120302.1		XLOC_124141	315 coding	noncoding	noncoding	noncoding
MSTRG.120306.1		XLOC_124144	276 coding	noncoding	noncoding	noncoding
MSTRG.120309.1		XLOC_124145	261 coding	noncoding	noncoding	noncoding
XM_011517102.2	KCNK9	XLOC_124153	5242 coding	coding	coding	coding
MSTRG.121073.1	TRAPPC9	XLOC_124173	287 coding	noncoding	noncoding	noncoding
MSTRG.121093.1	TRAPPC9	XLOC_124190	292 coding	noncoding	noncoding	noncoding
MSTRG.121094.1	TRAPPC9	XLOC_124191	262 coding	noncoding	noncoding	noncoding
XM_011516966.3	AGO2	XLOC_124195	15325 coding	coding	coding	coding
MSTRG.120750.1	PTK2	XLOC_124206	302 coding	noncoding	noncoding	noncoding
MSTRG.120760.1	PTK2	XLOC_124216	277 coding	noncoding	noncoding	noncoding
MSTRG.120729.1		XLOC_124221	320 coding	noncoding	noncoding	noncoding
MSTRG.120731.1		XLOC_124222	247 coding	noncoding	noncoding	noncoding
MSTRG.120740.1		XLOC_124229	240 coding	noncoding	noncoding	noncoding
MSTRG.120937.20	DENND3	XLOC_124231	3931 coding	coding	coding	noncoding
MSTRG.120959.1	SLC45A4	XLOC_124246	257 coding	noncoding	noncoding	noncoding
MSTRG.120795.1		XLOC_124264	373 coding	noncoding	noncoding	noncoding
XR_928307.2	TSNARE1	XLOC_124269	5223 coding	coding	coding	coding
NM_177457.5	LYNX1	XLOC_124297	4594 coding	noncoding	coding	coding
MSTRG.120886.1	LYNX1-SLURP2	XLOC_124298	294 coding	noncoding	noncoding	noncoding
MSTRG.120912.1	LY6E-DT	XLOC_124313	234 coding	noncoding	noncoding	noncoding
MSTRG.120972.1		XLOC_124337	233 coding	noncoding	noncoding	noncoding
XM_011516943.2	ZC3H3	XLOC_124354	2549 coding	coding	coding	coding
NM_001317783.2	TSTA3	XLOC_124368	1391 coding	coding	coding	coding
MSTRG.121102.1	SPATC1	XLOC_124394	338 coding	noncoding	noncoding	noncoding
MSTRG.121161.1	MROH1	XLOC_124412	266 coding	noncoding	noncoding	noncoding
MSTRG.121175.1	MROH1	XLOC_124424	288 coding	noncoding	coding	noncoding
NM_012079.6	DGAT1	XLOC_124430	3653 coding	coding	coding	coding
MSTRG.121141.10	CPSF1	XLOC_124434	270 coding	noncoding	noncoding	noncoding
XM_017013870.2	ARHGAP39	XLOC_124452	6023 coding	coding	coding	coding
MSTRG.121219.1	ARHGAP39	XLOC_124454	322 coding	noncoding	noncoding	noncoding

MSTRG.121220.1	ARHGAP39	XLOC_124455	228	coding	noncoding	noncoding	noncoding
NR_147501.1	LOC100130027	XLOC_124467	4389	coding	noncoding	coding	coding
MSTRG.121236.1		XLOC_124471	279	coding	noncoding	noncoding	noncoding
MSTRG.121237.1		XLOC_124472	404	coding	noncoding	noncoding	noncoding
MSTRG.121262.1	ZNF252P	XLOC_124477	225	coding	noncoding	noncoding	noncoding
MSTRG.121277.1		XLOC_124500	274	coding	noncoding	noncoding	noncoding
XM_017015174.1	DOCK8	XLOC_124507	7388	coding	coding	coding	coding
XM_011518049.2	DOCK8	XLOC_124507	6013	coding	coding	coding	coding
XM_024447462.1	KANK1	XLOC_124512	6589	coding	coding	coding	coding
NM_001354341.1	KANK1	XLOC_124512	5468	coding	coding	coding	coding
MSTRG.121478.1	KANK1	XLOC_124515	319	coding	noncoding	coding	noncoding
MSTRG.121287.1		XLOC_124516	310	coding	noncoding	noncoding	noncoding
MSTRG.121295.1	DMRT1	XLOC_124519	540	coding	noncoding	noncoding	noncoding
MSTRG.121296.1		XLOC_124525	274	coding	coding	noncoding	noncoding
MSTRG.121317.1		XLOC_124533	311	coding	noncoding	noncoding	noncoding
MSTRG.121339.1	LOC105375951	XLOC_124539	244	coding	noncoding	noncoding	noncoding
MSTRG.121346.1		XLOC_124580	321	coding	coding	noncoding	noncoding
MSTRG.121348.1		XLOC_124586	472	coding	noncoding	noncoding	noncoding
MSTRG.121360.1	VLDLR-AS1	XLOC_124588	305	coding	noncoding	noncoding	noncoding
MSTRG.121571.1		XLOC_124733	297	coding	noncoding	noncoding	noncoding
MSTRG.121579.1	SPATA6L	XLOC_124738	282	coding	noncoding	noncoding	noncoding
MSTRG.121699.1	RCL1	XLOC_124766	268	coding	noncoding	noncoding	noncoding
MSTRG.121702.1		XLOC_124769	277	coding	noncoding	noncoding	noncoding
MSTRG.121703.1		XLOC_124770	287	coding	noncoding	noncoding	noncoding
MSTRG.121707.1		XLOC_124774	235	coding	noncoding	noncoding	noncoding
NM_001322194.1	JAK2	XLOC_124780	5386	coding	coding	coding	coding
MSTRG.121717.7	JAK2	XLOC_124780	465	coding	coding	noncoding	noncoding
MSTRG.121710.1		XLOC_124810	235	coding	noncoding	noncoding	noncoding
MSTRG.121941.1	RIC1	XLOC_124858	283	coding	noncoding	noncoding	noncoding
MSTRG.121954.1	RIC1	XLOC_124871	387	coding	coding	noncoding	noncoding
MSTRG.121978.5	MIR4665	XLOC_124886	1572	coding	coding	coding	noncoding
MSTRG.121786.1		XLOC_124888	310	coding	coding	noncoding	noncoding
MSTRG.121787.1		XLOC_124889	290	coding	noncoding	noncoding	noncoding
MSTRG.121795.1	LOC107987046	XLOC_124892	219	coding	coding	noncoding	noncoding
MSTRG.121796.1	LOC107987046	XLOC_124893	256	coding	noncoding	noncoding	noncoding
MSTRG.121811.1	GLDC	XLOC_124908	251	coding	noncoding	noncoding	noncoding
MSTRG.121813.1		XLOC_124912	220	coding	noncoding	noncoding	noncoding
MSTRG.122077.1	KDM4C	XLOC_124916	308	coding	noncoding	noncoding	noncoding
MSTRG.122079.1	KDM4C	XLOC_124917	358	coding	noncoding	noncoding	noncoding
MSTRG.121879.1		XLOC_124964	282	coding	noncoding	noncoding	noncoding
MSTRG.121891.3	DMAC1	XLOC_124967	647	coding	coding	coding	noncoding
MSTRG.121891.2	DMAC1	XLOC_124967	646	coding	coding	coding	noncoding
MSTRG.121903.1		XLOC_124971	238	coding	noncoding	noncoding	noncoding

MSTRG.121908.1		XLOC_124974	281 coding	noncoding	noncoding	noncoding
MSTRG.122242.1	PTPRD	XLOC_124985	220 coding	noncoding	noncoding	noncoding
MSTRG.122252.1	PTPRD	XLOC_124995	206 coding	noncoding	noncoding	noncoding
MSTRG.122261.1	PTPRD	XLOC_124997	310 coding	noncoding	noncoding	noncoding
MSTRG.122266.1	PTPRD	XLOC_124999	228 coding	noncoding	noncoding	noncoding
MSTRG.122279.1	PTPRD	XLOC_125005	265 coding	noncoding	noncoding	noncoding
MSTRG.122291.1	PTPRD	XLOC_125011	295 coding	noncoding	noncoding	noncoding
MSTRG.122293.1	PTPRD	XLOC_125013	276 coding	noncoding	noncoding	noncoding
MSTRG.122314.1	PTPRD	XLOC_125026	297 coding	noncoding	noncoding	noncoding
MSTRG.122318.1	PTPRD	XLOC_125029	238 coding	noncoding	noncoding	noncoding
MSTRG.122005.1		XLOC_125031	202 coding	coding	noncoding	noncoding
MSTRG.122011.1		XLOC_125032	271 coding	noncoding	noncoding	noncoding
MSTRG.122137.1		XLOC_125070	274 coding	noncoding	noncoding	noncoding
MSTRG.122144.1		XLOC_125082	225 coding	noncoding	noncoding	noncoding
MSTRG.122175.1	LOC101929507	XLOC_125086	309 coding	noncoding	noncoding	noncoding
MSTRG.122178.1	LOC101929507	XLOC_125087	318 coding	noncoding	noncoding	noncoding
MSTRG.122322.1		XLOC_125124	261 coding	noncoding	noncoding	noncoding
MSTRG.122324.1		XLOC_125132	312 coding	coding	noncoding	noncoding
MSTRG.122329.1		XLOC_125134	276 coding	noncoding	noncoding	noncoding
MSTRG.122330.1		XLOC_125135	253 coding	noncoding	noncoding	noncoding
MSTRG.122332.1		XLOC_125137	285 coding	noncoding	noncoding	noncoding
MSTRG.122369.1		XLOC_125142	321 coding	noncoding	noncoding	noncoding
MSTRG.122386.1		XLOC_125144	277 coding	noncoding	noncoding	noncoding
XM_017014443.1	CCDC171	XLOC_125146	5662 coding	noncoding	coding	coding
MSTRG.122418.1	CCDC171	XLOC_125148	417 coding	noncoding	noncoding	noncoding
MSTRG.122427.1	CCDC171	XLOC_125150	305 coding	noncoding	noncoding	noncoding
MSTRG.122461.1		XLOC_125201	242 coding	noncoding	noncoding	noncoding
MSTRG.122463.1		XLOC_125203	296 coding	noncoding	noncoding	noncoding
MSTRG.122476.1		XLOC_125224	291 coding	noncoding	noncoding	noncoding
XM_017015310.1	ADAMTSL1	XLOC_125225	14618 coding	coding	coding	coding
MSTRG.122586.1		XLOC_125252	260 coding	noncoding	noncoding	noncoding
XM_017014918.1	DENND4C	XLOC_125253	7503 coding	coding	coding	coding
MSTRG.122611.1	ACER2	XLOC_125255	267 coding	noncoding	noncoding	noncoding
MSTRG.122659.2	MLLT3	XLOC_125278	2793 coding	coding	noncoding	noncoding
MSTRG.122713.1		XLOC_125292	236 coding	noncoding	noncoding	noncoding
MSTRG.122724.1		XLOC_125305	516 coding	noncoding	noncoding	noncoding
MSTRG.122725.1		XLOC_125306	222 coding	noncoding	noncoding	noncoding
MSTRG.122742.1		XLOC_125330	312 coding	noncoding	noncoding	noncoding
MSTRG.122810.1		XLOC_125358	300 coding	coding	noncoding	noncoding
MSTRG.122814.1		XLOC_125360	483 coding	noncoding	noncoding	noncoding
MSTRG.122815.1		XLOC_125361	222 coding	noncoding	noncoding	noncoding
MSTRG.122830.1		XLOC_125376	251 coding	noncoding	noncoding	noncoding
MSTRG.122903.1	LOC105375993	XLOC_125391	274 coding	noncoding	noncoding	noncoding

MSTRG.122900.1		XLOC_125394	221	coding	noncoding	noncoding	noncoding
MSTRG.122912.1		XLOC_125398	308	coding	noncoding	noncoding	noncoding
MSTRG.122926.1		XLOC_125403	247	coding	noncoding	noncoding	noncoding
MSTRG.122935.1		XLOC_125405	265	coding	noncoding	noncoding	noncoding
MSTRG.122941.1		XLOC_125408	218	coding	noncoding	noncoding	noncoding
MSTRG.123001.1	LOC105375999	XLOC_125436	251	coding	noncoding	noncoding	noncoding
MSTRG.123120.16	C9orf72	XLOC_125462	2891	coding	coding	coding	noncoding
MSTRG.123073.1		XLOC_125463	316	coding	noncoding	noncoding	noncoding
MSTRG.123092.1		XLOC_125470	261	coding	noncoding	noncoding	noncoding
MSTRG.123088.1		XLOC_125495	505	coding	noncoding	noncoding	noncoding
MSTRG.124077.1	LINGO2	XLOC_125498	262	coding	noncoding	noncoding	noncoding
MSTRG.124158.1	LINGO2	XLOC_125556	227	coding	noncoding	noncoding	noncoding
MSTRG.123159.1		XLOC_125783	267	coding	noncoding	noncoding	noncoding
MSTRG.123163.1		XLOC_125786	239	coding	noncoding	noncoding	noncoding
MSTRG.123174.1		XLOC_125792	335	coding	noncoding	noncoding	noncoding
MSTRG.123204.1		XLOC_125807	270	coding	coding	noncoding	noncoding
MSTRG.123210.1		XLOC_125812	280	coding	noncoding	noncoding	noncoding
MSTRG.123227.1		XLOC_125820	247	coding	noncoding	noncoding	noncoding
MSTRG.123230.1		XLOC_125823	281	coding	noncoding	noncoding	noncoding
MSTRG.123234.1		XLOC_125824	317	coding	coding	noncoding	noncoding
MSTRG.123235.1		XLOC_125825	229	coding	coding	noncoding	noncoding
MSTRG.123247.1	LOC105376011	XLOC_125828	252	coding	noncoding	noncoding	noncoding
MSTRG.123249.1	LOC105376011	XLOC_125829	208	coding	noncoding	noncoding	noncoding
MSTRG.123238.1		XLOC_125830	215	coding	noncoding	noncoding	noncoding
MSTRG.123252.1		XLOC_125836	275	coding	noncoding	noncoding	noncoding
MSTRG.123264.1		XLOC_125850	260	coding	noncoding	noncoding	noncoding
MSTRG.123281.1		XLOC_125883	228	coding	noncoding	noncoding	noncoding
MSTRG.123322.1	NFX1	XLOC_125891	266	coding	noncoding	noncoding	noncoding
MSTRG.123386.1	LOC107987061	XLOC_125902	270	coding	noncoding	noncoding	noncoding
MSTRG.123425.1	TRBV20OR9-2	XLOC_125911	247	coding	noncoding	noncoding	noncoding
MSTRG.123448.1	TRBV20OR9-2	XLOC_125921	264	coding	noncoding	noncoding	noncoding
MSTRG.123462.11	UBAP2	XLOC_125934	369	coding	coding	noncoding	noncoding
XM_011517900.1	UBAP1	XLOC_125939	2675	coding	noncoding	coding	coding
MSTRG.123402.1	KIF24	XLOC_125945	243	coding	noncoding	noncoding	noncoding
NR_052010.1	IL11RA	XLOC_125971	1776	coding	noncoding	coding	coding
MSTRG.123525.1	PHF24	XLOC_125978	272	coding	coding	noncoding	noncoding
MSTRG.123527.1	PHF24	XLOC_125979	290	coding	noncoding	noncoding	noncoding
XM_005251428.3	DNAJB5	XLOC_125984	2362	coding	coding	coding	coding
MSTRG.123543.1		XLOC_125996	326	coding	noncoding	noncoding	noncoding
MSTRG.123561.1		XLOC_126011	277	coding	noncoding	noncoding	noncoding
MSTRG.123590.1	UNC13B	XLOC_126016	280	coding	noncoding	noncoding	noncoding
MSTRG.123568.1	ATP8B5P	XLOC_126019	260	coding	noncoding	noncoding	noncoding
NM_016446.3	TMEM8B	XLOC_126057	2153	coding	coding	coding	coding

MSTRG.123675.1	RECK	XLOC_126076	258 coding	noncoding	noncoding	noncoding
MSTRG.123684.1	GLIPR2	XLOC_126082	305 coding	noncoding	noncoding	noncoding
MSTRG.123661.1		XLOC_126086	225 coding	noncoding	noncoding	noncoding
MSTRG.123677.1		XLOC_126087	242 coding	noncoding	noncoding	noncoding
MSTRG.123753.1	LOC102724322	XLOC_126095	250 coding	noncoding	noncoding	noncoding
MSTRG.123771.1		XLOC_126101	277 coding	noncoding	noncoding	noncoding
MSTRG.123767.1		XLOC_126102	284 coding	noncoding	noncoding	noncoding
MSTRG.123858.1		XLOC_126122	242 coding	noncoding	noncoding	noncoding
NM_001289119.1	ZCCHC7	XLOC_126124	2690 coding	coding	coding	coding
XR_001746665.1	LOC105376033	XLOC_126132	1608 coding	noncoding	noncoding	noncoding
MSTRG.123841.1	FBXO10	XLOC_126180	260 coding	coding	noncoding	noncoding
XM_017014481.1	FRMPD1	XLOC_126184	5292 coding	coding	coding	coding
MSTRG.123828.1	FRMPD1	XLOC_126188	248 coding	noncoding	noncoding	noncoding
XM_005251577.4	DCAF10	XLOC_126193	2175 coding	coding	coding	coding
XM_017015128.1	DCAF10	XLOC_126193	1976 coding	noncoding	coding	coding
MSTRG.123920.1	SHB	XLOC_126207	693 coding	coding	noncoding	noncoding
MSTRG.123981.1		XLOC_126234	223 coding	noncoding	noncoding	noncoding
MSTRG.123988.1		XLOC_126245	369 coding	noncoding	noncoding	noncoding
MSTRG.123996.1	LOC105376043	XLOC_126249	273 coding	noncoding	noncoding	noncoding
MSTRG.124008.1		XLOC_126253	211 coding	noncoding	noncoding	noncoding
MSTRG.124490.1		XLOC_126326	764 coding	coding	coding	coding
MSTRG.124523.1		XLOC_126332	499 coding	coding	noncoding	noncoding
MSTRG.124534.3	FRG1HP	XLOC_126341	215 coding	noncoding	noncoding	noncoding
MSTRG.124544.1	PGM5P2	XLOC_126351	270 coding	noncoding	noncoding	noncoding
MSTRG.124581.1		XLOC_126386	233 coding	noncoding	noncoding	noncoding
MSTRG.124582.1		XLOC_126387	224 coding	noncoding	noncoding	noncoding
XR_929667.3	LOC105376063	XLOC_126389	4437 coding	noncoding	coding	noncoding
MSTRG.124591.1		XLOC_126391	228 coding	noncoding	noncoding	noncoding
MSTRG.124602.1	CNTNAP3B	XLOC_126394	500 coding	noncoding	noncoding	noncoding
MSTRG.124609.1	CNTNAP3B	XLOC_126399	270 coding	noncoding	noncoding	noncoding
XM_011517871.3	SPATA31A6	XLOC_126404	4215 coding	coding	coding	coding
MSTRG.124658.1		XLOC_126409	308 coding	noncoding	noncoding	noncoding
MSTRG.124698.1		XLOC_126428	216 coding	noncoding	noncoding	noncoding
MSTRG.124752.1		XLOC_126466	280 coding	noncoding	noncoding	noncoding
MSTRG.124757.1		XLOC_126468	550 coding	noncoding	noncoding	noncoding
MSTRG.124767.1	LOC107987008	XLOC_126476	304 coding	noncoding	noncoding	noncoding
MSTRG.124826.1	LINC01189	XLOC_126480	321 coding	noncoding	noncoding	noncoding
MSTRG.124821.1		XLOC_126524	266 coding	noncoding	noncoding	noncoding
MSTRG.124915.1		XLOC_126549	412 coding	noncoding	noncoding	noncoding
MSTRG.124931.1		XLOC_126561	398 coding	noncoding	noncoding	noncoding
NM_199244.3	FOXD4L4	XLOC_126585	1251 coding	coding	coding	coding
XR_950683.2	LOC105379448	XLOC_126603	4252 coding	noncoding	noncoding	noncoding
MSTRG.124988.1		XLOC_126605	275 coding	noncoding	noncoding	noncoding

MSTRG.124989.1		XLOC_126606	238 coding	noncoding	noncoding	noncoding
MSTRG.125000.1		XLOC_126614	276 coding	noncoding	noncoding	noncoding
MSTRG.125004.1		XLOC_126616	383 coding	noncoding	noncoding	noncoding
NR_111893.2	CNTNAP3P2	XLOC_126618	7649 coding	coding	coding	coding
MSTRG.125066.1		XLOC_126626	400 coding	noncoding	noncoding	noncoding
MSTRG.125067.1		XLOC_126627	297 coding	noncoding	noncoding	noncoding
MSTRG.125099.1	CBWD3	XLOC_126646	318 coding	coding	noncoding	noncoding
XM_011519082.2	PIP5K1B	XLOC_126686	5608 coding	coding	coding	coding
MSTRG.125205.1		XLOC_126716	216 coding	noncoding	noncoding	noncoding
MSTRG.125264.2	PTAR1	XLOC_126739	10102 coding	coding	coding	noncoding
MSTRG.125277.1	LOC105376076	XLOC_126741	284 coding	noncoding	noncoding	noncoding
MSTRG.125248.1		XLOC_126746	272 coding	noncoding	noncoding	noncoding
MSTRG.125279.1		XLOC_126768	259 coding	noncoding	noncoding	noncoding
XR_001746711.2	LOC101927086	XLOC_126769	4319 coding	noncoding	coding	noncoding
MSTRG.125343.1	TRPM3	XLOC_126773	315 coding	noncoding	noncoding	noncoding
MSTRG.125348.1	TRPM3	XLOC_126776	325 coding	noncoding	noncoding	noncoding
MSTRG.125378.1	TRPM3	XLOC_126792	230 coding	noncoding	noncoding	noncoding
MSTRG.125285.1		XLOC_126795	448 coding	noncoding	noncoding	noncoding
MSTRG.125288.1		XLOC_126797	291 coding	noncoding	noncoding	noncoding
MSTRG.125289.1		XLOC_126798	250 coding	noncoding	noncoding	noncoding
MSTRG.125483.1	C9orf85	XLOC_126836	545 coding	noncoding	noncoding	noncoding
MSTRG.125432.1	GDA	XLOC_126844	241 coding	noncoding	noncoding	noncoding
MSTRG.125425.1		XLOC_126846	219 coding	noncoding	noncoding	noncoding
MSTRG.125455.1		XLOC_126854	260 coding	noncoding	noncoding	noncoding
NM_138691.2	TMC1	XLOC_126856	3201 coding	coding	coding	coding
MSTRG.125514.1	TMC1	XLOC_126865	300 coding	noncoding	noncoding	noncoding
MSTRG.125458.1		XLOC_126868	324 coding	noncoding	noncoding	noncoding
XM_017014657.1	ANXA1	XLOC_126882	1481 coding	coding	coding	coding
MSTRG.125579.1		XLOC_126899	306 coding	noncoding	noncoding	noncoding
MSTRG.125596.1	LOC101927329	XLOC_126906	262 coding	noncoding	noncoding	noncoding
MSTRG.125707.1		XLOC_126955	278 coding	noncoding	noncoding	noncoding
MSTRG.125709.1		XLOC_126960	284 coding	noncoding	noncoding	noncoding
MSTRG.125839.1	PCSK5	XLOC_126973	260 coding	noncoding	noncoding	noncoding
MSTRG.125841.1	PCSK5	XLOC_126975	218 coding	noncoding	noncoding	noncoding
MSTRG.125870.1	PCSK5	XLOC_126985	301 coding	noncoding	noncoding	noncoding
MSTRG.125814.1	PRUNE2	XLOC_127007	324 coding	noncoding	noncoding	noncoding
MSTRG.125817.1	PRUNE2	XLOC_127009	375 coding	noncoding	noncoding	noncoding
MSTRG.125759.1		XLOC_127010	309 coding	noncoding	noncoding	noncoding
MSTRG.125758.1		XLOC_127011	210 coding	noncoding	noncoding	noncoding
MSTRG.125764.1		XLOC_127014	271 coding	noncoding	noncoding	noncoding
MSTRG.125891.1	VPS13A	XLOC_127029	220 coding	noncoding	noncoding	noncoding
MSTRG.125904.1	GNA14	XLOC_127039	229 coding	noncoding	noncoding	noncoding
MSTRG.126059.9	GNAQ	XLOC_127048	3140 coding	coding	noncoding	noncoding

NM_001330691.3	CEP78	XLOC_127060	11233	coding	coding	coding	coding
MSTRG.125939.1	LOC107987083	XLOC_127070	717	coding	noncoding	noncoding	noncoding
MSTRG.125953.1		XLOC_127078	301	coding	noncoding	noncoding	noncoding
MSTRG.126040.1		XLOC_127089	222	coding	noncoding	noncoding	noncoding
MSTRG.125962.1		XLOC_127095	230	coding	noncoding	noncoding	noncoding
MSTRG.125969.1		XLOC_127097	283	coding	noncoding	noncoding	noncoding
MSTRG.126160.1		XLOC_127158	247	coding	noncoding	noncoding	noncoding
MSTRG.126167.1		XLOC_127169	295	coding	noncoding	noncoding	noncoding
MSTRG.126313.1		XLOC_127200	274	coding	noncoding	noncoding	noncoding
MSTRG.126320.1		XLOC_127204	296	coding	noncoding	noncoding	noncoding
MSTRG.126326.1		XLOC_127205	304	coding	noncoding	noncoding	noncoding
MSTRG.126336.1	LOC101927502	XLOC_127222	215	coding	noncoding	noncoding	noncoding
MSTRG.126341.1	LOC101927502	XLOC_127224	258	coding	noncoding	noncoding	noncoding
MSTRG.126360.1	LOC105376107	XLOC_127236	315	coding	noncoding	noncoding	noncoding
MSTRG.126372.1		XLOC_127257	272	coding	noncoding	noncoding	noncoding
MSTRG.126598.1	FRMD3	XLOC_127290	318	coding	noncoding	noncoding	noncoding
MSTRG.126470.1		XLOC_127300	371	coding	noncoding	noncoding	noncoding
MSTRG.126504.5	RMI1	XLOC_127301	8442	coding	coding	coding	coding
MSTRG.126510.1	RMI1	XLOC_127302	239	coding	noncoding	noncoding	noncoding
MSTRG.126478.1		XLOC_127304	212	coding	noncoding	noncoding	noncoding
MSTRG.126481.1		XLOC_127311	262	coding	noncoding	noncoding	noncoding
MSTRG.126517.1	LOC102724036	XLOC_127314	302	coding	noncoding	noncoding	noncoding
MSTRG.126546.1	NTRK2	XLOC_127318	296	coding	noncoding	noncoding	noncoding
MSTRG.126638.15	AGTPBP1	XLOC_127332	3787	coding	coding	coding	noncoding
MSTRG.126622.1		XLOC_127339	268	coding	noncoding	coding	noncoding
MSTRG.126634.1		XLOC_127346	319	coding	noncoding	noncoding	noncoding
MSTRG.126636.1		XLOC_127347	228	coding	noncoding	noncoding	noncoding
MSTRG.126698.1		XLOC_127364	394	coding	noncoding	noncoding	noncoding
MSTRG.126718.1		XLOC_127375	270	coding	noncoding	noncoding	noncoding
MSTRG.126725.1	LOC440173	XLOC_127378	252	coding	noncoding	noncoding	noncoding
MSTRG.126740.1		XLOC_127390	336	coding	noncoding	noncoding	noncoding
MSTRG.126752.1		XLOC_127392	372	coding	noncoding	noncoding	noncoding
MSTRG.126753.1		XLOC_127393	220	coding	noncoding	noncoding	noncoding
XM_011518702.1	SPATA31C1	XLOC_127420	4336	coding	coding	coding	coding
MSTRG.126776.1		XLOC_127421	289	coding	noncoding	coding	noncoding
MSTRG.126804.1		XLOC_127428	258	coding	noncoding	noncoding	noncoding
MSTRG.126867.1	SHC3	XLOC_127449	296	coding	noncoding	noncoding	noncoding
MSTRG.126869.1	SHC3	XLOC_127450	223	coding	noncoding	noncoding	noncoding
MSTRG.127045.48	SEMA4D	XLOC_127471	5316	coding	coding	coding	coding
MSTRG.127045.49	SEMA4D	XLOC_127471	5861	coding	coding	coding	coding
MSTRG.127045.47	SEMA4D	XLOC_127471	11055	coding	coding	coding	coding
MSTRG.127047.1	SEMA4D	XLOC_127472	9863	coding	noncoding	coding	noncoding
MSTRG.126902.1		XLOC_127478	264	coding	noncoding	noncoding	noncoding

MSTRG.126928.1		XLOC_127497	280	coding	noncoding	noncoding	noncoding
MSTRG.126951.1	DIRAS2	XLOC_127504	239	coding	noncoding	noncoding	noncoding
MSTRG.127012.1		XLOC_127581	245	coding	noncoding	noncoding	noncoding
MSTRG.126999.1		XLOC_127584	453	coding	noncoding	noncoding	noncoding
MSTRG.127035.1	ROR2	XLOC_127597	203	coding	noncoding	noncoding	noncoding
MSTRG.127021.1		XLOC_127599	321	coding	noncoding	noncoding	noncoding
MSTRG.127053.1		XLOC_127609	219	coding	noncoding	noncoding	noncoding
MSTRG.127233.1	LOC107987095	XLOC_127617	295	coding	noncoding	noncoding	noncoding
MSTRG.127072.1		XLOC_127625	204	coding	noncoding	noncoding	noncoding
MSTRG.127240.1	FGD3	XLOC_127634	289	coding	noncoding	noncoding	noncoding
MSTRG.127249.1	FGD3	XLOC_127642	772	coding	coding	noncoding	noncoding
MSTRG.127234.1		XLOC_127657	241	coding	noncoding	noncoding	noncoding
MSTRG.127274.1		XLOC_127667	279	coding	noncoding	noncoding	noncoding
MSTRG.127294.1		XLOC_127709	320	coding	noncoding	noncoding	noncoding
XM_017015403.1	LOC107987099	XLOC_127717	2265	coding	coding	coding	coding
MSTRG.127349.1	LINC02603	XLOC_127727	266	coding	noncoding	noncoding	noncoding
XM_011519114.2	MFSD14B	XLOC_127733	2989	coding	coding	coding	coding
MSTRG.127380.1	MFSD14B	XLOC_127740	225	coding	noncoding	noncoding	noncoding
MSTRG.127381.1	MFSD14B	XLOC_127741	229	coding	noncoding	noncoding	noncoding
MSTRG.127369.1		XLOC_127748	229	coding	noncoding	noncoding	noncoding
MSTRG.127710.1	AOPEP	XLOC_127755	439	coding	noncoding	noncoding	noncoding
MSTRG.127714.1	AOPEP	XLOC_127758	274	coding	noncoding	noncoding	noncoding
MSTRG.127716.1	AOPEP	XLOC_127760	280	coding	noncoding	noncoding	noncoding
MSTRG.127442.1		XLOC_127785	324	coding	noncoding	noncoding	noncoding
MSTRG.127447.1	LOC105376156	XLOC_127787	241	coding	noncoding	noncoding	noncoding
MSTRG.127451.1	LOC105376157	XLOC_127808	290	coding	noncoding	noncoding	noncoding
MSTRG.127561.1	LINC00476	XLOC_127821	258	coding	noncoding	noncoding	noncoding
MSTRG.127500.1		XLOC_127835	268	coding	noncoding	noncoding	noncoding
MSTRG.127538.1	CDC14B	XLOC_127847	247	coding	noncoding	noncoding	noncoding
MSTRG.127527.1		XLOC_127855	219	coding	noncoding	noncoding	noncoding
MSTRG.127529.1		XLOC_127858	261	coding	noncoding	noncoding	noncoding
NR_026792.1	LOC441455	XLOC_127859	1647	coding	coding	coding	coding
MSTRG.127672.1	ZNF782	XLOC_127871	391	coding	noncoding	noncoding	noncoding
MSTRG.127584.1		XLOC_127884	342	coding	noncoding	noncoding	noncoding
MSTRG.127587.1		XLOC_127885	255	coding	noncoding	noncoding	noncoding
MSTRG.127615.1	SUGT1P4-STRA6LP-CCDC180	XLOC_127900	295	coding	noncoding	noncoding	noncoding
MSTRG.127789.1	PTCSC2	XLOC_127926	232	coding	noncoding	noncoding	noncoding
MSTRG.127792.1	PTCSC2	XLOC_127928	245	coding	coding	noncoding	noncoding
MSTRG.127836.2	ANP32B	XLOC_127952	466	coding	noncoding	noncoding	noncoding
MSTRG.127823.1		XLOC_127954	275	coding	noncoding	noncoding	noncoding
MSTRG.127854.3	TBC1D2	XLOC_127966	2031	coding	coding	coding	coding
MSTRG.127866.1	GABBR2	XLOC_127971	310	coding	noncoding	noncoding	noncoding

MSTRG.127867.1	GABBR2	XLOC_127972	225 coding	noncoding	noncoding	noncoding
MSTRG.127871.1	GABBR2	XLOC_127976	256 coding	coding	noncoding	noncoding
NM_001306210.2	TGFBR1	XLOC_127998	6504 coding	coding	coding	coding
MSTRG.127973.1	LOC101928438	XLOC_128029	234 coding	noncoding	noncoding	noncoding
MSTRG.127984.1		XLOC_128039	247 coding	noncoding	noncoding	noncoding
MSTRG.128221.1	INVS	XLOC_128058	255 coding	noncoding	noncoding	noncoding
MSTRG.128002.1	MSANTD3	XLOC_128071	314 coding	noncoding	noncoding	noncoding
MSTRG.128012.1	TMEFF1	XLOC_128076	248 coding	noncoding	noncoding	noncoding
MSTRG.128113.3	RNF20	XLOC_128099	2167 coding	coding	coding	coding
MSTRG.128120.1	RNF20	XLOC_128105	304 coding	noncoding	noncoding	noncoding
MSTRG.128049.1		XLOC_128124	295 coding	noncoding	noncoding	noncoding
MSTRG.128071.1		XLOC_128132	252 coding	noncoding	noncoding	noncoding
MSTRG.128085.1	LINC01492	XLOC_128137	296 coding	coding	noncoding	noncoding
MSTRG.128090.1	LINC01492	XLOC_128139	207 coding	noncoding	noncoding	noncoding
MSTRG.128168.1		XLOC_128161	285 coding	coding	noncoding	noncoding
MSTRG.128169.1		XLOC_128162	301 coding	noncoding	noncoding	noncoding
MSTRG.128181.1		XLOC_128170	229 coding	noncoding	noncoding	noncoding
MSTRG.128183.1		XLOC_128172	413 coding	noncoding	noncoding	noncoding
MSTRG.128187.1		XLOC_128174	261 coding	noncoding	noncoding	noncoding
MSTRG.128272.1	LOC112268038	XLOC_128225	318 coding	noncoding	noncoding	noncoding
MSTRG.128282.1	SLC44A1	XLOC_128234	277 coding	noncoding	noncoding	noncoding
MSTRG.128289.1	SLC44A1	XLOC_128236	273 coding	noncoding	noncoding	noncoding
MSTRG.128290.1	SLC44A1	XLOC_128237	420 coding	noncoding	noncoding	noncoding
MSTRG.128388.1	FKTN	XLOC_128249	278 coding	noncoding	noncoding	noncoding
MSTRG.128508.1		XLOC_128267	352 coding	noncoding	noncoding	noncoding
MSTRG.128519.1		XLOC_128274	216 coding	noncoding	noncoding	noncoding
MSTRG.128550.1		XLOC_128283	251 coding	noncoding	noncoding	noncoding
MSTRG.128553.1		XLOC_128285	291 coding	noncoding	noncoding	noncoding
MSTRG.128559.1		XLOC_128287	338 coding	noncoding	noncoding	noncoding
MSTRG.128416.1		XLOC_128295	272 coding	noncoding	noncoding	noncoding
XM_006717215.4	ZNF462	XLOC_128296	12385 coding	coding	coding	coding
MSTRG.128422.1		XLOC_128301	256 coding	noncoding	noncoding	noncoding
NM_001244724.1	RAD23B	XLOC_128306	3837 coding	coding	coding	coding
MSTRG.128466.1	LINC01509	XLOC_128318	730 coding	noncoding	noncoding	noncoding
MSTRG.128467.1	LINC01509	XLOC_128319	253 coding	noncoding	noncoding	noncoding
MSTRG.128470.1		XLOC_128322	278 coding	noncoding	noncoding	noncoding
MSTRG.128480.1	LOC105376205	XLOC_128326	282 coding	noncoding	noncoding	noncoding
MSTRG.128488.1		XLOC_128334	264 coding	noncoding	noncoding	noncoding
MSTRG.128757.2	TMEM245	XLOC_128422	7667 coding	coding	coding	noncoding
MSTRG.128695.1		XLOC_128424	280 coding	noncoding	noncoding	noncoding
MSTRG.128725.1	PTPN3	XLOC_128432	285 coding	noncoding	noncoding	noncoding
MSTRG.128769.1	PALM2-AKAP2	XLOC_128436	314 coding	noncoding	noncoding	noncoding
MSTRG.128775.1	PALM2-AKAP2	XLOC_128439	216 coding	noncoding	noncoding	noncoding

MSTRG.128800.1		XLOC_128459	262	coding	noncoding	noncoding	noncoding
MSTRG.128817.1	SVEP1	XLOC_128465	235	coding	noncoding	noncoding	noncoding
MSTRG.128996.1		XLOC_128553	316	coding	noncoding	noncoding	noncoding
MSTRG.128936.1	UGCG	XLOC_128562	269	coding	noncoding	noncoding	noncoding
MSTRG.128923.1		XLOC_128565	540	coding	noncoding	noncoding	noncoding
MSTRG.129014.2	PTBP3	XLOC_128573	7114	coding	coding	coding	noncoding
MSTRG.128940.1		XLOC_128574	276	coding	noncoding	noncoding	noncoding
MSTRG.129072.1	HSDL2	XLOC_128590	548	coding	noncoding	noncoding	noncoding
MSTRG.129087.1	KIAA1958	XLOC_128605	231	coding	noncoding	noncoding	noncoding
MSTRG.129113.3	HDHD3	XLOC_128639	1940	coding	coding	coding	noncoding
NM_134427.2	RGS3	XLOC_128644	1714	coding	coding	coding	coding
XM_017014251.2	ZNF618	XLOC_128659	17483	coding	noncoding	coding	coding
MSTRG.129176.1	ZNF618	XLOC_128660	298	coding	noncoding	noncoding	noncoding
MSTRG.129324.20	AKNA	XLOC_128673	1468	coding	noncoding	coding	noncoding
MSTRG.129205.1	LOC105376229	XLOC_128692	258	coding	noncoding	noncoding	noncoding
XM_011518354.1	TMEM268	XLOC_128696	4561	coding	coding	coding	coding
MSTRG.129248.1	TMEM268	XLOC_128698	250	coding	noncoding	noncoding	noncoding
MSTRG.129254.1	TMEM268	XLOC_128701	307	coding	noncoding	noncoding	noncoding
MSTRG.129236.1		XLOC_128706	273	coding	noncoding	noncoding	noncoding
MSTRG.129319.1	1-Dec	XLOC_128728	299	coding	noncoding	noncoding	noncoding
MSTRG.129293.1		XLOC_128732	315	coding	noncoding	noncoding	noncoding
MSTRG.129322.1		XLOC_128738	225	coding	noncoding	noncoding	noncoding
XR_930271.2	LOC105376236	XLOC_128744	6837	coding	noncoding	noncoding	coding
MSTRG.129375.1		XLOC_128748	284	coding	coding	noncoding	noncoding
XR_930276.3	LOC105376239	XLOC_128810	7704	coding	noncoding	noncoding	noncoding
MSTRG.129384.1		XLOC_128827	366	coding	noncoding	noncoding	noncoding
MSTRG.129485.1	LOC105376244	XLOC_128843	265	coding	noncoding	noncoding	noncoding
MSTRG.129420.1		XLOC_128860	382	coding	noncoding	noncoding	noncoding
MSTRG.129426.1		XLOC_128866	214	coding	noncoding	noncoding	noncoding
MSTRG.129452.1	BRINP1	XLOC_128877	267	coding	noncoding	noncoding	noncoding
MSTRG.129504.1	LOC107987122	XLOC_128885	221	coding	noncoding	noncoding	noncoding
MSTRG.129672.1		XLOC_128930	274	coding	coding	noncoding	noncoding
MSTRG.129684.3	FBXW2	XLOC_128937	5464	coding	coding	coding	noncoding
NR_024408.2	CUTALP	XLOC_128942	3765	coding	noncoding	coding	noncoding
XM_011518170.2	CNTRL	XLOC_128959	7650	coding	coding	coding	coding
XM_006716940.3	CNTRL	XLOC_128959	7641	coding	coding	coding	coding
XM_024447392.1	CNTRL	XLOC_128959	6869	coding	coding	coding	coding
XM_011518593.1	GSN	XLOC_128961	2781	coding	coding	coding	coding
XR_001746558.2	LOC102723324	XLOC_128969	1376	coding	noncoding	noncoding	noncoding
XM_017014298.2	DAB2IP	XLOC_128978	8402	coding	coding	coding	coding
MSTRG.129728.1	DAB2IP	XLOC_128981	292	coding	noncoding	noncoding	noncoding
MSTRG.129733.1	DAB2IP	XLOC_128985	289	coding	noncoding	noncoding	noncoding
NM_000962.4	PTGS1	XLOC_129026	5020	coding	coding	coding	coding

NM_001004457.2	OR1N2	XLOC_129033	1103 coding	coding	coding	coding
MSTRG.129822.1	OR1B1	XLOC_129036	203 coding	noncoding	noncoding	noncoding
NM_012197.4	RABGAP1	XLOC_129070	4986 coding	coding	coding	coding
MSTRG.129917.1	RABGAP1	XLOC_129071	234 coding	noncoding	noncoding	noncoding
MSTRG.129863.1	LOC107987037	XLOC_129104	238 coding	noncoding	noncoding	noncoding
MSTRG.129864.1	LOC107987037	XLOC_129105	264 coding	noncoding	noncoding	noncoding
MSTRG.129867.1		XLOC_129108	280 coding	noncoding	noncoding	noncoding
MSTRG.129876.1		XLOC_129115	298 coding	noncoding	noncoding	noncoding
XM_006717348.3	ADGRD2	XLOC_129129	3644 coding	coding	coding	coding
MSTRG.130034.1	OLFML2A	XLOC_129153	218 coding	noncoding	noncoding	noncoding
MSTRG.130030.1		XLOC_129155	479 coding	noncoding	noncoding	noncoding
MSTRG.130101.1	ARPC5L	XLOC_129159	281 coding	noncoding	noncoding	noncoding
MSTRG.130118.1	SCAI	XLOC_129169	270 coding	noncoding	noncoding	noncoding
MSTRG.130181.1		XLOC_129240	239 coding	noncoding	noncoding	noncoding
MSTRG.130263.1		XLOC_129256	251 coding	noncoding	noncoding	noncoding
MSTRG.130258.1		XLOC_129264	468 coding	noncoding	noncoding	noncoding
NM_014007.4	ZBTB43	XLOC_129268	5940 coding	coding	coding	coding
XM_011518699.3	ZBTB34	XLOC_129271	6579 coding	coding	coding	coding
NM_001322321.2	RALGPS1	XLOC_129273	6448 coding	coding	coding	coding
XM_011519235.2	RALGPS1	XLOC_129273	8441 coding	coding	coding	coding
MSTRG.130389.1	RALGPS1	XLOC_129278	230 coding	noncoding	noncoding	noncoding
MSTRG.130429.1	GARNL3	XLOC_129297	241 coding	noncoding	noncoding	noncoding
MSTRG.130430.1	GARNL3	XLOC_129298	262 coding	noncoding	noncoding	noncoding
MSTRG.130360.1		XLOC_129323	358 coding	noncoding	noncoding	noncoding
NR_145961.1	CFAP157	XLOC_129327	1826 coding	noncoding	coding	coding
NM_001018078.2	FPGS	XLOC_129335	2327 coding	noncoding	coding	coding
MSTRG.130491.1	ENG	XLOC_129340	289 coding	noncoding	noncoding	noncoding
MSTRG.130493.1	ENG	XLOC_129341	244 coding	noncoding	noncoding	noncoding
MSTRG.130503.1		XLOC_129348	283 coding	noncoding	noncoding	noncoding
MSTRG.130523.1	SLC25A25	XLOC_129352	224 coding	noncoding	noncoding	noncoding
MSTRG.130574.1	TRUB2	XLOC_129364	289 coding	noncoding	noncoding	noncoding
MSTRG.130554.1	CERCAM	XLOC_129375	290 coding	noncoding	noncoding	noncoding
MSTRG.130556.1	CERCAM	XLOC_129376	238 coding	noncoding	noncoding	noncoding
NM_002540.5	ODF2	XLOC_129377	4041 coding	noncoding	coding	coding
MSTRG.130596.1	LOC100506100	XLOC_129396	395 coding	noncoding	noncoding	noncoding
MSTRG.130597.2	ZER1	XLOC_129397	4281 coding	noncoding	coding	coding
MSTRG.130593.1		XLOC_129399	260 coding	noncoding	noncoding	noncoding
MSTRG.130626.1	KYAT1	XLOC_129402	302 coding	coding	noncoding	noncoding
MSTRG.130651.1	MIGA2	XLOC_129422	331 coding	noncoding	noncoding	noncoding
MSTRG.130709.1		XLOC_129456	243 coding	noncoding	noncoding	noncoding
MSTRG.130710.1		XLOC_129457	237 coding	noncoding	noncoding	noncoding
MSTRG.130711.1		XLOC_129458	212 coding	noncoding	noncoding	noncoding
MSTRG.130714.1		XLOC_129462	320 coding	noncoding	noncoding	noncoding

NM_001286796.1	NTMT1	XLOC_129463	1688	coding	coding	coding	coding
MSTRG.130722.1		XLOC_129467	246	coding	noncoding	noncoding	noncoding
MSTRG.130738.1	PTGES	XLOC_129470	598	coding	noncoding	noncoding	noncoding
MSTRG.130740.1		XLOC_129471	256	coding	noncoding	noncoding	noncoding
MSTRG.130741.1		XLOC_129472	302	coding	noncoding	noncoding	noncoding
XM_011518161.2	USP20	XLOC_129475	4682	coding	coding	coding	coding
MSTRG.130822.1	FNBP1	XLOC_129481	1473	coding	noncoding	noncoding	noncoding
MSTRG.130745.1		XLOC_129490	269	coding	noncoding	noncoding	noncoding
MSTRG.130756.1	NCS1	XLOC_129492	278	coding	noncoding	noncoding	noncoding
NM_001291815.2	HMCN2	XLOC_129499	15789	coding	coding	noncoding	coding
MSTRG.130778.1		XLOC_129510	258	coding	noncoding	noncoding	noncoding
MSTRG.130840.1	ABL1	XLOC_129515	333	coding	noncoding	noncoding	noncoding
MSTRG.130872.1		XLOC_129535	275	coding	noncoding	noncoding	noncoding
MSTRG.130858.1	LOC105376300	XLOC_129543	308	coding	noncoding	noncoding	noncoding
MSTRG.130861.1		XLOC_129545	268	coding	noncoding	noncoding	noncoding
MSTRG.130886.1		XLOC_129549	245	coding	noncoding	noncoding	noncoding
MSTRG.130947.2	RAPGEF1	XLOC_129558	1287	coding	coding	coding	coding
MSTRG.130901.1		XLOC_129559	286	coding	noncoding	noncoding	noncoding
MSTRG.130907.1		XLOC_129566	249	coding	coding	noncoding	noncoding
XM_011519112.2	NTNG2	XLOC_129568	4097	coding	coding	coding	coding
NM_207417.3	CFAP77	XLOC_129574	1833	coding	coding	coding	coding
MSTRG.130935.1	CFAP77	XLOC_129576	215	coding	noncoding	noncoding	noncoding
MSTRG.130936.1	CFAP77	XLOC_129577	401	coding	noncoding	noncoding	noncoding
MSTRG.130938.1	CFAP77	XLOC_129578	286	coding	noncoding	noncoding	noncoding
MSTRG.131046.1	AK8	XLOC_129617	286	coding	noncoding	noncoding	noncoding
MSTRG.131054.1	SPACA9	XLOC_129624	298	coding	noncoding	noncoding	noncoding
MSTRG.131042.1	GTF3C5	XLOC_129636	299	coding	noncoding	noncoding	noncoding
MSTRG.131032.1		XLOC_129639	267	coding	noncoding	noncoding	noncoding
MSTRG.131076.1	RALGDS	XLOC_129644	3428	coding	coding	coding	coding
MSTRG.131066.1		XLOC_129647	248	coding	noncoding	noncoding	noncoding
MSTRG.131130.1	VAV2	XLOC_129684	261	coding	noncoding	noncoding	noncoding
MSTRG.131154.1	VAV2	XLOC_129699	547	coding	noncoding	coding	noncoding
MSTRG.131118.1		XLOC_129702	316	coding	noncoding	noncoding	noncoding
NM_000093.4	COL5A1	XLOC_129733	8440	coding	coding	coding	coding
MSTRG.131242.1		XLOC_129747	264	coding	noncoding	noncoding	noncoding
MSTRG.131259.1		XLOC_129757	236	coding	noncoding	noncoding	noncoding
MSTRG.131261.1	PPP1R26-AS1	XLOC_129758	280	coding	noncoding	noncoding	noncoding
XM_017015360.2	PPP1R26	XLOC_129759	5827	coding	coding	coding	coding
MSTRG.131285.1	SOHLH1	XLOC_129773	225	coding	noncoding	noncoding	noncoding
XM_017014543.2	PMPCA	XLOC_129807	2081	coding	coding	coding	coding
MSTRG.131405.2	NOTCH1	XLOC_129812	9896	coding	coding	noncoding	coding
MSTRG.131405.4	NOTCH1	XLOC_129812	979	coding	noncoding	coding	noncoding
MSTRG.131472.1		XLOC_129858	218	coding	noncoding	noncoding	noncoding

MSTRG.131487.1	ABCA2	XLOC_129859	8317	coding	coding	noncoding	coding
MSTRG.131503.1	DPP7	XLOC_129869	243	coding	noncoding	noncoding	noncoding
XM_005266073.4	GRIN1	XLOC_129872	4507	coding	coding	coding	coding
MSTRG.131515.2	ANAPC2	XLOC_129874	612	coding	noncoding	coding	noncoding
MSTRG.131530.1	FAM166A	XLOC_129885	249	coding	noncoding	noncoding	noncoding
MSTRG.131534.1	NELFB	XLOC_129888	292	coding	noncoding	noncoding	noncoding
MSTRG.131550.1	NSMF	XLOC_129895	2446	coding	noncoding	coding	noncoding
XM_006717322.4	ARRDC1	XLOC_129903	2850	coding	coding	coding	coding
MSTRG.131609.1	ARRDC1	XLOC_129904	274	coding	noncoding	noncoding	noncoding
XM_005266105.5	EHMT1	XLOC_129906	5150	coding	coding	coding	coding
MSTRG.131614.1	EHMT1	XLOC_129907	274	coding	noncoding	noncoding	noncoding
MSTRG.131615.1	EHMT1	XLOC_129908	256	coding	noncoding	noncoding	noncoding
MSTRG.131571.1		XLOC_129927	280	coding	noncoding	noncoding	noncoding
NR_027156.1	TUBBP5	XLOC_129929	2660	coding	coding	coding	coding
MSTRG.131574.1	TUBBP5	XLOC_129930	240	coding	noncoding	noncoding	noncoding
MSTRG.121276.1		XLOC_129943	401	coding	noncoding	noncoding	noncoding
XR_001746591.1	LINC01388	XLOC_129944	6241	coding	noncoding	coding	coding
MSTRG.121436.1	CBWD1	XLOC_129948	340	coding	noncoding	noncoding	noncoding
MSTRG.121441.1		XLOC_129951	280	coding	noncoding	noncoding	noncoding
MSTRG.121477.1	KANK1	XLOC_129977	265	coding	noncoding	noncoding	noncoding
MSTRG.121294.1	DMRT1	XLOC_129984	289	coding	noncoding	noncoding	noncoding
MSTRG.121306.1	DMRT2	XLOC_129987	274	coding	noncoding	noncoding	noncoding
MSTRG.121336.1	LOC105375951	XLOC_130009	275	coding	noncoding	noncoding	noncoding
MSTRG.121550.1		XLOC_130094	339	coding	noncoding	noncoding	noncoding
MSTRG.121551.1		XLOC_130097	277	coding	noncoding	noncoding	noncoding
XM_005251389.5	GLIS3	XLOC_130099	3880	coding	noncoding	coding	coding
XM_024447597.1	SPATA6L	XLOC_130113	4382	coding	coding	coding	coding
MSTRG.121780.1	PLGRKT	XLOC_130148	298	coding	noncoding	noncoding	noncoding
XR_929192.3	KIAA2026	XLOC_130166	7150	coding	noncoding	coding	coding
MSTRG.121987.1	KIAA2026	XLOC_130174	472	coding	noncoding	noncoding	noncoding
MSTRG.121794.1	LOC107987046	XLOC_130187	219	coding	coding	noncoding	noncoding
MSTRG.121806.1		XLOC_130199	263	coding	noncoding	noncoding	noncoding
MSTRG.121814.1		XLOC_130200	220	coding	noncoding	noncoding	noncoding
MSTRG.122080.1	KDM4C	XLOC_130203	277	coding	noncoding	noncoding	noncoding
MSTRG.121880.1		XLOC_130229	273	coding	noncoding	noncoding	noncoding
MSTRG.121896.1		XLOC_130244	230	coding	noncoding	noncoding	noncoding
MSTRG.121902.1		XLOC_130245	404	coding	noncoding	noncoding	noncoding
MSTRG.121907.1		XLOC_130247	265	coding	noncoding	noncoding	noncoding
XM_017014958.2	PTPRD	XLOC_130249	10468	coding	coding	coding	coding
MSTRG.122227.1	PTPRD	XLOC_130252	269	coding	noncoding	noncoding	noncoding
MSTRG.122253.1	PTPRD	XLOC_130262	252	coding	noncoding	noncoding	noncoding
MSTRG.122004.1		XLOC_130297	202	coding	coding	noncoding	noncoding
MSTRG.122010.1		XLOC_130299	268	coding	noncoding	noncoding	noncoding

MSTRG.122014.1		XLOC_130305	303 coding	noncoding	noncoding	noncoding
MSTRG.122029.1	LOC105375974	XLOC_130315	294 coding	noncoding	noncoding	noncoding
MSTRG.122033.1		XLOC_130318	253 coding	noncoding	noncoding	noncoding
MSTRG.122052.1		XLOC_130330	410 coding	noncoding	noncoding	noncoding
MSTRG.122065.1	LURAP1L-AS1	XLOC_130336	311 coding	noncoding	noncoding	noncoding
MSTRG.122159.1		XLOC_130352	298 coding	noncoding	noncoding	noncoding
MSTRG.122161.1		XLOC_130356	272 coding	coding	noncoding	noncoding
MSTRG.122177.1	LOC101929507	XLOC_130362	319 coding	noncoding	noncoding	noncoding
NR_163239.1	FREM1	XLOC_130395	11258 coding	coding	coding	coding
MSTRG.122367.1	FREM1	XLOC_130398	223 coding	noncoding	noncoding	noncoding
MSTRG.122325.1		XLOC_130400	206 coding	noncoding	noncoding	noncoding
MSTRG.122328.1		XLOC_130403	276 coding	noncoding	noncoding	noncoding
MSTRG.122376.1	TTC39B	XLOC_130407	281 coding	noncoding	noncoding	noncoding
MSTRG.122382.1	TTC39B	XLOC_130413	294 coding	noncoding	noncoding	noncoding
MSTRG.122385.1	TTC39B	XLOC_130416	235 coding	noncoding	noncoding	noncoding
MSTRG.122414.1	CCDC171	XLOC_130422	248 coding	noncoding	noncoding	noncoding
MSTRG.122416.1	CCDC171	XLOC_130424	274 coding	noncoding	noncoding	noncoding
MSTRG.122419.1	CCDC171	XLOC_130426	245 coding	noncoding	noncoding	noncoding
MSTRG.122420.1	CCDC171	XLOC_130427	215 coding	noncoding	noncoding	noncoding
MSTRG.122421.1	CCDC171	XLOC_130428	311 coding	noncoding	noncoding	noncoding
MSTRG.122522.1	BNC2	XLOC_130473	297 coding	noncoding	noncoding	noncoding
MSTRG.122460.1		XLOC_130476	242 coding	noncoding	noncoding	noncoding
MSTRG.122462.1		XLOC_130477	312 coding	noncoding	noncoding	noncoding
MSTRG.122464.1		XLOC_130478	230 coding	noncoding	noncoding	noncoding
MSTRG.122469.1		XLOC_130481	241 coding	noncoding	noncoding	noncoding
MSTRG.122577.1	CNTLN	XLOC_130494	298 coding	noncoding	noncoding	noncoding
MSTRG.122553.1	ADAMTSL1	XLOC_130517	261 coding	noncoding	noncoding	noncoding
MSTRG.122650.1	SLC24A2	XLOC_130549	314 coding	noncoding	noncoding	noncoding
MSTRG.122729.1	LOC107987053	XLOC_130628	233 coding	noncoding	noncoding	noncoding
MSTRG.122813.1		XLOC_130697	295 coding	noncoding	noncoding	noncoding
MSTRG.122827.1		XLOC_130701	382 coding	coding	noncoding	noncoding
MSTRG.122845.1	LINC01239	XLOC_130708	322 coding	coding	noncoding	noncoding
MSTRG.122831.1		XLOC_130712	261 coding	coding	noncoding	noncoding
MSTRG.122902.1	LOC105375993	XLOC_130724	274 coding	noncoding	noncoding	noncoding
MSTRG.122933.1		XLOC_130740	239 coding	noncoding	noncoding	noncoding
MSTRG.123000.1	LOC105375999	XLOC_130777	257 coding	noncoding	noncoding	noncoding
XR_001746638.1	LOC105376000	XLOC_130784	6424 coding	noncoding	coding	noncoding
MSTRG.123022.1	CAAP1	XLOC_130792	201 coding	coding	noncoding	noncoding
MSTRG.123041.1	EQTN	XLOC_130813	336 coding	noncoding	noncoding	noncoding
MSTRG.123072.1		XLOC_130841	316 coding	noncoding	noncoding	noncoding
MSTRG.123091.1		XLOC_130843	261 coding	noncoding	noncoding	noncoding
MSTRG.123089.1		XLOC_130852	505 coding	noncoding	noncoding	noncoding
MSTRG.124128.1	LINGO2	XLOC_130870	319 coding	noncoding	noncoding	noncoding

MSTRG.124212.1	LINGO2	XLOC_130884	772 coding	noncoding	noncoding	noncoding
MSTRG.123158.1		XLOC_130920	267 coding	noncoding	noncoding	noncoding
MSTRG.123168.1		XLOC_130924	250 coding	noncoding	noncoding	noncoding
MSTRG.123195.1		XLOC_130938	206 coding	noncoding	noncoding	noncoding
MSTRG.123248.1	LOC105376011	XLOC_130960	252 coding	noncoding	noncoding	noncoding
MSTRG.123237.1		XLOC_130961	214 coding	noncoding	noncoding	noncoding
MSTRG.123239.1		XLOC_130962	215 coding	noncoding	noncoding	noncoding
MSTRG.123250.1		XLOC_130965	237 coding	noncoding	noncoding	noncoding
MSTRG.123269.1	LOC105376017	XLOC_130983	280 coding	noncoding	noncoding	noncoding
MSTRG.123276.1		XLOC_131006	302 coding	noncoding	noncoding	noncoding
MSTRG.123304.1		XLOC_131016	259 coding	noncoding	noncoding	noncoding
MSTRG.123426.1	TRBV20OR9-2	XLOC_131034	266 coding	noncoding	noncoding	noncoding
MSTRG.123447.1	TRBV20OR9-2	XLOC_131046	264 coding	noncoding	noncoding	noncoding
MSTRG.123450.5	UBE2R2	XLOC_131047	3898 coding	coding	noncoding	noncoding
MSTRG.123450.11	UBAP2	XLOC_131047	9840 coding	coding	coding	coding
NM_001370064.1	UBAP2	XLOC_131047	4154 coding	coding	coding	coding
MSTRG.123388.1		XLOC_131049	231 coding	noncoding	noncoding	noncoding
MSTRG.123389.1		XLOC_131051	231 coding	noncoding	noncoding	noncoding
NM_020702.5	MYORG	XLOC_131056	6447 coding	coding	coding	coding
MSTRG.123410.1	DNAI1	XLOC_131066	230 coding	noncoding	noncoding	noncoding
NM_198573.3	ENHO	XLOC_131068	1028 coding	noncoding	coding	noncoding
XM_017014283.1	ARID3C	XLOC_131075	4423 coding	coding	coding	coding
MSTRG.123496.3	GALT	XLOC_131077	719 coding	coding	coding	noncoding
NR_135148.2	LOC730098	XLOC_131083	1347 coding	noncoding	coding	noncoding
MSTRG.123558.1		XLOC_131112	314 coding	noncoding	noncoding	noncoding
MSTRG.123569.1	ATP8B5P	XLOC_131126	260 coding	noncoding	noncoding	noncoding
MSTRG.123633.1		XLOC_131148	273 coding	noncoding	noncoding	noncoding
NM_006289.4	TLN1	XLOC_131153	8623 coding	coding	noncoding	coding
MSTRG.123712.1		XLOC_131166	255 coding	noncoding	noncoding	noncoding
MSTRG.123757.1	LOC102724322	XLOC_131210	252 coding	noncoding	noncoding	noncoding
MSTRG.123772.1		XLOC_131225	277 coding	noncoding	noncoding	noncoding
MSTRG.123768.1		XLOC_131226	284 coding	noncoding	noncoding	noncoding
NR_104000.1	PAX5	XLOC_131235	8836 coding	coding	coding	coding
MSTRG.123793.1	PAX5	XLOC_131236	309 coding	noncoding	noncoding	noncoding
MSTRG.123797.1	PAX5	XLOC_131240	263 coding	noncoding	noncoding	noncoding
XM_017014619.2	FBXO10	XLOC_131269	4892 coding	coding	coding	coding
XR_929230.2	FBXO10	XLOC_131269	2517 coding	coding	coding	coding
MSTRG.123839.1	FBXO10	XLOC_131270	249 coding	noncoding	noncoding	noncoding
MSTRG.123959.1	SLC25A51	XLOC_131289	280 coding	noncoding	noncoding	noncoding
NM_003028.3	SHB	XLOC_131292	6035 coding	coding	coding	coding
MSTRG.123916.1		XLOC_131298	405 coding	noncoding	noncoding	noncoding
MSTRG.123968.1	LOC107987064	XLOC_131303	226 coding	noncoding	noncoding	noncoding
MSTRG.123961.1		XLOC_131304	274 coding	noncoding	noncoding	noncoding

NM_001007563.3	IGFBPL1	XLOC_131309	3566 coding	coding	coding	coding
MSTRG.124001.1	ANKRD18A	XLOC_131313	333 coding	noncoding	noncoding	noncoding
MSTRG.123991.1		XLOC_131317	284 coding	noncoding	noncoding	noncoding
MSTRG.124012.1	LOC101927042	XLOC_131321	305 coding	noncoding	noncoding	noncoding
MSTRG.124446.1	LOC105376050	XLOC_131371	257 coding	noncoding	noncoding	noncoding
MSTRG.124491.1		XLOC_131395	263 coding	noncoding	noncoding	noncoding
NR_147037.1	LOC440896	XLOC_131407	4387 coding	coding	noncoding	noncoding
MSTRG.124575.1		XLOC_131456	653 coding	coding	noncoding	noncoding
NM_001201380.2	CNTNAP3B	XLOC_131467	6379 coding	coding	coding	coding
MSTRG.124606.1	CNTNAP3B	XLOC_131469	210 coding	noncoding	noncoding	noncoding
MSTRG.124692.1		XLOC_131508	235 coding	noncoding	noncoding	noncoding
MSTRG.124749.1		XLOC_131547	409 coding	noncoding	noncoding	noncoding
MSTRG.124753.1		XLOC_131548	280 coding	noncoding	noncoding	noncoding
MSTRG.124829.1	LINC01189	XLOC_131567	231 coding	noncoding	noncoding	noncoding
MSTRG.124863.1	LOC403323	XLOC_131587	299 coding	noncoding	noncoding	noncoding
NM_001126334.1	FOXD4L5	XLOC_131645	3109 coding	coding	coding	coding
MSTRG.125031.1		XLOC_131657	496 coding	noncoding	noncoding	noncoding
MSTRG.125005.1		XLOC_131703	382 coding	noncoding	noncoding	noncoding
NM_001083124.1	SPATA31A3	XLOC_131704	4223 coding	coding	coding	coding
MSTRG.125059.1		XLOC_131719	259 coding	noncoding	noncoding	noncoding
MSTRG.125093.10	CBWD3	XLOC_131732	881 coding	coding	noncoding	noncoding
MSTRG.125204.1		XLOC_131785	302 coding	noncoding	noncoding	noncoding
MSTRG.125180.1	APBA1	XLOC_131795	304 coding	noncoding	noncoding	noncoding
MSTRG.125191.1	APBA1	XLOC_131797	245 coding	coding	noncoding	noncoding
MSTRG.125249.1		XLOC_131815	272 coding	noncoding	noncoding	noncoding
MSTRG.125305.1	MAMDC2	XLOC_131822	258 coding	noncoding	noncoding	noncoding
MSTRG.125318.7	SMC5	XLOC_131828	1486 coding	noncoding	noncoding	noncoding
MSTRG.125340.1	LOC101927086	XLOC_131838	474 coding	noncoding	noncoding	noncoding
MSTRG.125344.1	TRPM3	XLOC_131840	228 coding	noncoding	noncoding	noncoding
MSTRG.125347.1	LOC105376078	XLOC_131841	325 coding	noncoding	noncoding	noncoding
MSTRG.125286.1		XLOC_131861	261 coding	noncoding	noncoding	noncoding
MSTRG.125290.1		XLOC_131862	250 coding	noncoding	noncoding	noncoding
MSTRG.125382.1		XLOC_131879	256 coding	noncoding	noncoding	noncoding
MSTRG.125460.1		XLOC_131884	224 coding	noncoding	noncoding	noncoding
MSTRG.125420.1	LOC101927108	XLOC_131897	227 coding	noncoding	noncoding	noncoding
MSTRG.125423.1		XLOC_131902	240 coding	noncoding	noncoding	noncoding
MSTRG.125574.1		XLOC_131973	287 coding	noncoding	noncoding	noncoding
MSTRG.125595.1	LOC101927329	XLOC_131980	262 coding	noncoding	noncoding	noncoding
MSTRG.125610.1	RORB	XLOC_131992	307 coding	noncoding	noncoding	noncoding
MSTRG.125619.1	RORB	XLOC_131998	254 coding	noncoding	noncoding	noncoding
NM_001177311.1	TRPM6	XLOC_131999	8271 coding	coding	coding	coding
MSTRG.125635.1	TRPM6	XLOC_132008	658 coding	coding	noncoding	noncoding
MSTRG.125621.1		XLOC_132013	298 coding	noncoding	noncoding	noncoding

MSTRG.125670.1		XLOC_132052	263	coding	noncoding	noncoding	noncoding
MSTRG.125700.1		XLOC_132079	302	coding	noncoding	noncoding	noncoding
MSTRG.125710.1		XLOC_132082	284	coding	noncoding	noncoding	noncoding
MSTRG.125708.1		XLOC_132083	244	coding	noncoding	noncoding	noncoding
MSTRG.125748.1		XLOC_132105	273	coding	noncoding	noncoding	noncoding
MSTRG.125755.1		XLOC_132108	252	coding	noncoding	noncoding	noncoding
MSTRG.125756.1		XLOC_132109	270	coding	noncoding	noncoding	noncoding
MSTRG.125808.1	PRUNE2	XLOC_132128	505	coding	noncoding	noncoding	noncoding
MSTRG.125760.1		XLOC_132133	309	coding	noncoding	noncoding	noncoding
MSTRG.125765.1		XLOC_132135	271	coding	noncoding	noncoding	noncoding
MSTRG.125910.1	GNA14	XLOC_132150	248	coding	noncoding	noncoding	noncoding
MSTRG.126122.1	GNAQ	XLOC_132212	333	coding	noncoding	noncoding	noncoding
MSTRG.125877.1		XLOC_132253	258	coding	noncoding	noncoding	noncoding
MSTRG.125952.1		XLOC_132275	301	coding	noncoding	noncoding	noncoding
MSTRG.126174.1	LINC01507	XLOC_132363	236	coding	noncoding	noncoding	noncoding
MSTRG.126316.1		XLOC_132470	299	coding	noncoding	noncoding	noncoding
MSTRG.126342.1	LOC101927502	XLOC_132489	240	coding	noncoding	noncoding	noncoding
MSTRG.126348.1	LOC105376108	XLOC_132493	296	coding	noncoding	noncoding	noncoding
MSTRG.126350.1	LOC105376108	XLOC_132494	297	coding	noncoding	noncoding	noncoding
NM_001244960.1	FRMD3	XLOC_132509	5068	coding	coding	coding	coding
MSTRG.126508.1	HNRNPK	XLOC_132546	737	coding	noncoding	noncoding	noncoding
MSTRG.126475.1		XLOC_132552	301	coding	noncoding	noncoding	noncoding
MSTRG.126515.1	LOC102724036	XLOC_132559	280	coding	coding	noncoding	noncoding
MSTRG.126520.1	LOC102724036	XLOC_132562	266	coding	noncoding	noncoding	noncoding
MSTRG.126539.1		XLOC_132581	312	coding	noncoding	noncoding	noncoding
MSTRG.126628.1		XLOC_132625	239	coding	noncoding	noncoding	noncoding
MSTRG.126631.1		XLOC_132627	277	coding	noncoding	noncoding	noncoding
MSTRG.126637.1		XLOC_132630	228	coding	noncoding	noncoding	noncoding
MSTRG.126677.1	LOC102724080	XLOC_132648	436	coding	noncoding	noncoding	noncoding
MSTRG.126690.1		XLOC_132656	237	coding	noncoding	noncoding	noncoding
MSTRG.126699.1		XLOC_132663	246	coding	noncoding	noncoding	noncoding
MSTRG.126700.1		XLOC_132664	259	coding	noncoding	noncoding	noncoding
MSTRG.126749.1		XLOC_132692	311	coding	noncoding	noncoding	noncoding
MSTRG.126803.1	SPATA31C2	XLOC_132711	314	coding	noncoding	noncoding	noncoding
MSTRG.126805.1		XLOC_132712	259	coding	noncoding	noncoding	noncoding
MSTRG.126807.1		XLOC_132715	293	coding	noncoding	noncoding	noncoding
MSTRG.126810.1		XLOC_132716	324	coding	noncoding	noncoding	noncoding
MSTRG.126885.1		XLOC_132741	269	coding	noncoding	noncoding	noncoding
MSTRG.126901.1		XLOC_132762	381	coding	noncoding	noncoding	noncoding
MSTRG.126930.1		XLOC_132779	259	coding	noncoding	noncoding	noncoding
MSTRG.127026.1	ROR2	XLOC_132846	275	coding	noncoding	noncoding	noncoding
MSTRG.127020.1		XLOC_132852	405	coding	noncoding	noncoding	noncoding
MSTRG.127059.1		XLOC_132853	310	coding	noncoding	noncoding	noncoding

MSTRG.127064.1	SPTLC1	XLOC_132857	220 coding	noncoding	noncoding	noncoding
MSTRG.127067.1	SPTLC1	XLOC_132860	792 coding	coding	coding	noncoding
MSTRG.127232.1	LOC107987095	XLOC_132922	295 coding	noncoding	noncoding	noncoding
NM_001354537.1	ZNF484	XLOC_132932	4841 coding	coding	coding	coding
NM_001007101.3	ZNF484	XLOC_132932	4726 coding	coding	coding	coding
MSTRG.127159.1	ZNF484	XLOC_132933	284 coding	noncoding	noncoding	noncoding
MSTRG.127235.1		XLOC_132952	241 coding	noncoding	noncoding	noncoding
NM_001368813.1	C9orf129	XLOC_132956	6504 coding	noncoding	coding	coding
MSTRG.127295.1		XLOC_132971	320 coding	noncoding	noncoding	noncoding
MSTRG.127370.1		XLOC_133025	318 coding	noncoding	noncoding	noncoding
MSTRG.127707.3	AOPEP	XLOC_133031	418 coding	noncoding	noncoding	noncoding
MSTRG.127439.1		XLOC_133081	279 coding	noncoding	noncoding	noncoding
MSTRG.127448.1	LOC105376156	XLOC_133084	241 coding	noncoding	noncoding	noncoding
MSTRG.127452.1	LOC105376157	XLOC_133091	290 coding	noncoding	noncoding	noncoding
MSTRG.127557.5	LINC00476	XLOC_133098	2139 coding	coding	coding	noncoding
MSTRG.127518.1	HSD17B3	XLOC_133119	222 coding	noncoding	noncoding	noncoding
MSTRG.127506.1		XLOC_133125	299 coding	noncoding	noncoding	noncoding
MSTRG.127545.1	CDC14B	XLOC_133131	243 coding	noncoding	noncoding	noncoding
XM_011518393.2	ZNF510	XLOC_133144	6994 coding	coding	coding	coding
MSTRG.127654.1		XLOC_133145	291 coding	noncoding	noncoding	noncoding
MSTRG.127689.1	NUTM2G	XLOC_133163	271 coding	coding	coding	noncoding
NM_001333.4	CTSV	XLOC_133169	4359 coding	coding	coding	coding
MSTRG.127583.1		XLOC_133172	552 coding	noncoding	noncoding	noncoding
MSTRG.127603.1	ANKRD18CP	XLOC_133179	252 coding	noncoding	noncoding	noncoding
MSTRG.127622.1	SUGT1P4-STRA6LP	XLOC_133188	256 coding	noncoding	noncoding	noncoding
MSTRG.127790.1	PTCSC2	XLOC_133223	232 coding	noncoding	noncoding	noncoding
XM_005252050.4	TRMO	XLOC_133232	4374 coding	coding	coding	coding
MSTRG.127832.1		XLOC_133237	228 coding	noncoding	noncoding	noncoding
NM_005458.8	GABBR2	XLOC_133252	5499 coding	coding	coding	coding
MSTRG.127924.2	TGFBR1	XLOC_133270	7393 coding	coding	noncoding	noncoding
MSTRG.127972.1	LOC101928438	XLOC_133294	230 coding	noncoding	noncoding	noncoding
MSTRG.127981.1		XLOC_133302	267 coding	noncoding	noncoding	noncoding
MSTRG.127992.1		XLOC_133310	477 coding	noncoding	noncoding	noncoding
MSTRG.127998.1		XLOC_133325	269 coding	noncoding	noncoding	noncoding
MSTRG.128020.1	LOC105376179	XLOC_133326	309 coding	noncoding	noncoding	noncoding
MSTRG.128031.1	MRPL50	XLOC_133334	252 coding	noncoding	noncoding	noncoding
MSTRG.128134.1	GRIN3A	XLOC_133354	295 coding	noncoding	noncoding	noncoding
MSTRG.128050.1		XLOC_133367	295 coding	noncoding	noncoding	noncoding
MSTRG.128052.1		XLOC_133368	261 coding	noncoding	noncoding	noncoding
MSTRG.128053.1		XLOC_133369	296 coding	noncoding	noncoding	noncoding
MSTRG.128064.1		XLOC_133377	313 coding	noncoding	noncoding	noncoding
MSTRG.128067.1		XLOC_133379	311 coding	noncoding	noncoding	noncoding
MSTRG.128098.1		XLOC_133397	278 coding	noncoding	noncoding	noncoding

MSTRG.128167.1		XLOC_133412	285 coding	coding	noncoding	noncoding
MSTRG.128182.1		XLOC_133421	318 coding	noncoding	noncoding	noncoding
NM_001004481.1	OR13C2	XLOC_133424	957 coding	coding	coding	coding
NM_001001956.1	OR13C9	XLOC_133425	957 coding	coding	coding	coding
MSTRG.128186.1		XLOC_133426	261 coding	noncoding	noncoding	noncoding
MSTRG.128306.1	LOC107987105	XLOC_133429	221 coding	coding	noncoding	noncoding
MSTRG.128315.1	LOC107987105	XLOC_133432	291 coding	noncoding	noncoding	noncoding
XM_011518342.3	ABCA1	XLOC_133435	10190 coding	coding	coding	coding
MSTRG.128335.1	ABCA1	XLOC_133443	259 coding	noncoding	noncoding	noncoding
MSTRG.128252.1	LOC105376197	XLOC_133456	277 coding	noncoding	noncoding	noncoding
MSTRG.128395.1	TAL2	XLOC_133490	246 coding	coding	noncoding	noncoding
MSTRG.128415.1		XLOC_133559	289 coding	noncoding	noncoding	noncoding
MSTRG.128433.1	ZNF462	XLOC_133564	224 coding	coding	noncoding	noncoding
MSTRG.128448.1		XLOC_133587	309 coding	noncoding	noncoding	noncoding
MSTRG.128481.1	LOC105376205	XLOC_133594	282 coding	noncoding	noncoding	noncoding
MSTRG.128498.1	LOC105376208	XLOC_133606	263 coding	noncoding	noncoding	noncoding
MSTRG.128753.1	CTNNAL1	XLOC_133661	436 coding	coding	noncoding	noncoding
XM_011518452.2	TMEM245	XLOC_133664	7747 coding	coding	coding	coding
NM_014334.3	FRRS1L	XLOC_133672	8197 coding	coding	coding	coding
MSTRG.128702.1	EPB41L4B	XLOC_133675	234 coding	noncoding	noncoding	noncoding
MSTRG.128703.1	EPB41L4B	XLOC_133676	233 coding	noncoding	noncoding	noncoding
MSTRG.128698.1		XLOC_133682	222 coding	noncoding	noncoding	noncoding
MSTRG.128699.1		XLOC_133683	273 coding	noncoding	noncoding	noncoding
MSTRG.128726.1	PTPN3	XLOC_133688	285 coding	noncoding	noncoding	noncoding
MSTRG.128715.1		XLOC_133692	240 coding	noncoding	noncoding	noncoding
MSTRG.128774.1	PALM2-AKAP2	XLOC_133698	216 coding	noncoding	noncoding	noncoding
MSTRG.128779.1	PALM2-AKAP2	XLOC_133700	237 coding	coding	noncoding	noncoding
MSTRG.128819.1	SVEP1	XLOC_133727	270 coding	noncoding	noncoding	noncoding
MSTRG.128820.1	SVEP1	XLOC_133728	233 coding	noncoding	noncoding	noncoding
MSTRG.128795.1		XLOC_133732	270 coding	noncoding	noncoding	noncoding
MSTRG.128898.1	ECPAS	XLOC_133759	246 coding	noncoding	noncoding	noncoding
MSTRG.128908.1	ECPAS	XLOC_133769	257 coding	noncoding	noncoding	noncoding
MSTRG.128893.1	PTGR1	XLOC_133780	298 coding	noncoding	noncoding	noncoding
MSTRG.129013.1	SUSD1	XLOC_133822	204 coding	noncoding	noncoding	noncoding
MSTRG.128939.1		XLOC_133828	314 coding	noncoding	noncoding	noncoding
MSTRG.129025.1	INIP	XLOC_133842	244 coding	noncoding	noncoding	noncoding
MSTRG.129026.1	INIP	XLOC_133843	226 coding	noncoding	noncoding	noncoding
MSTRG.129054.1		XLOC_133858	205 coding	noncoding	noncoding	noncoding
MSTRG.129109.2	PRPF4	XLOC_133865	385 coding	noncoding	noncoding	noncoding
MSTRG.129149.1		XLOC_133890	251 coding	coding	noncoding	noncoding
MSTRG.129200.1	LOC105376224	XLOC_133919	303 coding	noncoding	noncoding	noncoding
XR_929844.3	AKNA	XLOC_133921	5812 coding	coding	coding	coding
XM_011518491.3	WHRN	XLOC_133936	4126 coding	coding	coding	coding

MSTRG.129235.1		XLOC_133953	403	coding	noncoding	noncoding	noncoding
MSTRG.129304.1	1-Dec	XLOC_133998	278	coding	noncoding	noncoding	noncoding
MSTRG.129360.1		XLOC_134016	254	coding	noncoding	noncoding	noncoding
MSTRG.129570.1	ASTN2-AS1	XLOC_134037	233	coding	coding	noncoding	noncoding
MSTRG.129598.1	LOC105376240	XLOC_134044	300	coding	noncoding	noncoding	noncoding
MSTRG.129619.1	ASTN2	XLOC_134055	313	coding	noncoding	noncoding	noncoding
MSTRG.129640.1	ASTN2	XLOC_134065	284	coding	noncoding	noncoding	noncoding
MSTRG.129385.1		XLOC_134068	366	coding	noncoding	noncoding	noncoding
MSTRG.129388.1		XLOC_134070	259	coding	coding	noncoding	noncoding
MSTRG.129472.1	TLR4	XLOC_134071	26225	coding	coding	coding	noncoding
MSTRG.129472.2	TLR4	XLOC_134071	39778	coding	coding	coding	noncoding
MSTRG.129419.1		XLOC_134100	325	coding	noncoding	noncoding	noncoding
MSTRG.129442.1		XLOC_134113	224	coding	noncoding	noncoding	noncoding
MSTRG.129450.1	BRINP1	XLOC_134117	254	coding	noncoding	noncoding	noncoding
MSTRG.129453.1	BRINP1	XLOC_134118	267	coding	noncoding	noncoding	noncoding
MSTRG.129458.1	LOC105376250	XLOC_134121	307	coding	noncoding	noncoding	noncoding
MSTRG.129459.1	LOC105376250	XLOC_134122	237	coding	noncoding	noncoding	noncoding
MSTRG.129702.1	CDK5RAP2	XLOC_134152	244	coding	noncoding	noncoding	noncoding
MSTRG.129703.1	CDK5RAP2	XLOC_134153	295	coding	noncoding	noncoding	noncoding
MSTRG.129692.1	MEGF9	XLOC_134162	4922	coding	coding	coding	coding
MSTRG.130010.6	LOC102723324	XLOC_134200	409	coding	noncoding	noncoding	noncoding
MSTRG.129727.1	DAB2IP	XLOC_134215	367	coding	noncoding	noncoding	noncoding
MSTRG.129732.1	DAB2IP	XLOC_134216	276	coding	noncoding	noncoding	noncoding
MSTRG.129770.1	TTLL11	XLOC_134228	288	coding	noncoding	noncoding	noncoding
MSTRG.129777.1	TTLL11	XLOC_134230	312	coding	noncoding	noncoding	noncoding
MSTRG.129786.1	TTLL11	XLOC_134231	260	coding	noncoding	noncoding	noncoding
MSTRG.129743.1	NDUFA8	XLOC_134235	283	coding	noncoding	noncoding	noncoding
MSTRG.129748.1	MORN5	XLOC_134237	249	coding	noncoding	noncoding	noncoding
MSTRG.129751.1	LHX6	XLOC_134240	315	coding	noncoding	noncoding	noncoding
MSTRG.129847.1		XLOC_134260	264	coding	noncoding	noncoding	noncoding
MSTRG.129948.1	STRBP	XLOC_134281	204	coding	noncoding	noncoding	noncoding
MSTRG.129952.1	STRBP	XLOC_134285	211	coding	noncoding	noncoding	noncoding
MSTRG.129854.1		XLOC_134287	210	coding	noncoding	noncoding	noncoding
XM_011518882.3	DENND1A	XLOC_134289	6039	coding	coding	coding	coding
MSTRG.130202.1	DENND1A	XLOC_134290	318	coding	noncoding	noncoding	noncoding
MSTRG.130234.1	DENND1A	XLOC_134321	306	coding	noncoding	noncoding	noncoding
MSTRG.130257.1	DENND1A	XLOC_134342	409	coding	noncoding	noncoding	noncoding
MSTRG.130058.1	NR6A1	XLOC_134364	250	coding	noncoding	noncoding	noncoding
MSTRG.130098.1	NR6A1	XLOC_134392	261	coding	noncoding	noncoding	noncoding
MSTRG.130033.1	OLFML2A	XLOC_134394	258	coding	noncoding	noncoding	noncoding
MSTRG.130031.1		XLOC_134396	286	coding	noncoding	noncoding	noncoding
MSTRG.130028.1	WDR38	XLOC_134397	261	coding	noncoding	coding	noncoding
MSTRG.130102.1	ARPC5L	XLOC_134399	303	coding	noncoding	noncoding	noncoding

MSTRG.130125.1	SCAI	XLOC_134409	297 coding	noncoding	noncoding	noncoding
MSTRG.130126.1	SCAI	XLOC_134410	295 coding	noncoding	noncoding	noncoding
MSTRG.130127.1	SCAI	XLOC_134411	225 coding	noncoding	noncoding	noncoding
MSTRG.130158.1	MAPKAP1	XLOC_134438	561 coding	noncoding	noncoding	noncoding
MSTRG.130138.1		XLOC_134450	240 coding	noncoding	noncoding	noncoding
MSTRG.130267.1		XLOC_134494	242 coding	noncoding	noncoding	noncoding
MSTRG.130438.1	SLC2A8	XLOC_134525	280 coding	noncoding	coding	noncoding
MSTRG.130441.1	RPL12	XLOC_134527	657 coding	coding	coding	coding
MSTRG.130365.1	NIBAN2	XLOC_134539	254 coding	noncoding	noncoding	noncoding
MSTRG.130490.1	ENG	XLOC_134563	289 coding	noncoding	noncoding	noncoding
MSTRG.130492.1	ENG	XLOC_134564	320 coding	noncoding	noncoding	noncoding
MSTRG.130507.1		XLOC_134576	262 coding	noncoding	noncoding	noncoding
MSTRG.130504.1		XLOC_134577	283 coding	noncoding	noncoding	noncoding
NR_033374.1	SLC25A25-AS1	XLOC_134584	3842 coding	noncoding	coding	noncoding
MSTRG.130532.6	LCN2	XLOC_134587	504 coding	coding	noncoding	noncoding
MSTRG.130565.1	DNM1	XLOC_134594	235 coding	noncoding	noncoding	noncoding
MSTRG.130566.1	DNM1	XLOC_134595	295 coding	noncoding	noncoding	noncoding
MSTRG.130572.1		XLOC_134600	286 coding	noncoding	noncoding	noncoding
MSTRG.130552.1		XLOC_134619	240 coding	noncoding	noncoding	noncoding
XM_017015262.1	KYAT1	XLOC_134633	2782 coding	coding	coding	coding
MSTRG.130620.1	PHYHD1	XLOC_134637	382 coding	noncoding	noncoding	noncoding
MSTRG.130707.1		XLOC_134671	231 coding	noncoding	noncoding	noncoding
NM_016520.3	C9orf78	XLOC_134689	1795 coding	coding	coding	coding
MSTRG.130814.1	FNBP1	XLOC_134696	317 coding	noncoding	noncoding	noncoding
MSTRG.130815.1	FNBP1	XLOC_134697	223 coding	noncoding	noncoding	noncoding
MSTRG.130746.1		XLOC_134707	269 coding	noncoding	noncoding	noncoding
MSTRG.130770.1	HMCN2	XLOC_134715	264 coding	coding	noncoding	noncoding
MSTRG.130777.1		XLOC_134726	334 coding	noncoding	noncoding	noncoding
MSTRG.130837.2	ABL1	XLOC_134728	1851 coding	coding	coding	coding
MSTRG.130785.1		XLOC_134737	276 coding	noncoding	noncoding	noncoding
MSTRG.130879.1		XLOC_134762	240 coding	noncoding	noncoding	noncoding
MSTRG.130946.1		XLOC_134763	281 coding	noncoding	noncoding	noncoding
MSTRG.130956.1	RAPGEF1	XLOC_134772	250 coding	noncoding	noncoding	noncoding
MSTRG.130969.1	RAPGEF1	XLOC_134785	418 coding	noncoding	noncoding	noncoding
MSTRG.130900.1		XLOC_134788	286 coding	noncoding	noncoding	noncoding
MSTRG.130902.1		XLOC_134789	258 coding	noncoding	noncoding	noncoding
MSTRG.130909.1		XLOC_134808	284 coding	noncoding	noncoding	noncoding
MSTRG.130976.1		XLOC_134812	327 coding	noncoding	noncoding	noncoding
MSTRG.130980.1	TTF1	XLOC_134815	255 coding	noncoding	noncoding	noncoding
XM_011518277.2	AK8	XLOC_134828	4825 coding	coding	coding	coding
MSTRG.131059.1	TSC1	XLOC_134833	304 coding	noncoding	noncoding	noncoding
MSTRG.131033.1		XLOC_134841	267 coding	noncoding	noncoding	noncoding
MSTRG.131037.1		XLOC_134843	464 coding	noncoding	noncoding	noncoding

MSTRG.131070.1	GBGT1	XLOC_134847	284	coding	noncoding	noncoding	noncoding
MSTRG.131071.1	GBGT1	XLOC_134848	249	coding	noncoding	noncoding	noncoding
MSTRG.131065.1		XLOC_134849	248	coding	noncoding	noncoding	noncoding
MSTRG.131131.1	VAV2	XLOC_134872	261	coding	noncoding	noncoding	noncoding
MSTRG.131155.1	VAV2	XLOC_134881	269	coding	noncoding	noncoding	noncoding
MSTRG.131163.1	BRD3	XLOC_134885	252	coding	noncoding	noncoding	noncoding
MSTRG.131241.1		XLOC_134927	394	coding	noncoding	noncoding	noncoding
MSTRG.131266.1		XLOC_134943	321	coding	noncoding	noncoding	noncoding
XM_011518698.3	SOHLH1	XLOC_134951	4973	coding	coding	coding	coding
MSTRG.131296.1	KCNT1	XLOC_134957	275	coding	noncoding	noncoding	noncoding
XM_017014301.1	CAMSAP1	XLOC_134960	7912	coding	coding	coding	coding
MSTRG.131315.1	CAMSAP1	XLOC_134961	249	coding	noncoding	noncoding	noncoding
MSTRG.131307.1		XLOC_134971	250	coding	noncoding	noncoding	noncoding
MSTRG.131310.1	UBAC1	XLOC_134973	235	coding	noncoding	noncoding	noncoding
MSTRG.131338.1	NACC2	XLOC_134983	300	coding	noncoding	noncoding	noncoding
NM_001363746.1	LHX3	XLOC_134989	2700	coding	coding	coding	coding
MSTRG.131326.1	LOC101928722	XLOC_134992	268	coding	noncoding	noncoding	noncoding
NR_111982.1	CCDC187	XLOC_134994	4010	coding	noncoding	coding	coding
MSTRG.131328.1	DKFZP434A062	XLOC_134999	201	coding	noncoding	noncoding	noncoding
NR_026964.3	DKFZP434A062	XLOC_135000	5022	coding	noncoding	coding	coding
NM_001080849.3	DNLZ	XLOC_135006	3096	coding	coding	coding	coding
NR_003672.2	SNHG7	XLOC_135034	2176	coding	noncoding	noncoding	noncoding
MSTRG.131415.1		XLOC_135041	225	coding	noncoding	noncoding	noncoding
MSTRG.131488.5	ABCA2	XLOC_135067	351	coding	noncoding	coding	noncoding
MSTRG.131478.1	NPDC1	XLOC_135071	254	coding	noncoding	noncoding	noncoding
MSTRG.131505.1	GRIN1	XLOC_135083	283	coding	noncoding	coding	noncoding
MSTRG.131557.1	PNPLA7	XLOC_135113	361	coding	noncoding	noncoding	noncoding
MSTRG.131558.1	PNPLA7	XLOC_135114	300	coding	noncoding	noncoding	noncoding
MSTRG.131560.1	PNPLA7	XLOC_135116	285	coding	noncoding	noncoding	noncoding
MSTRG.131569.1	DPH7	XLOC_135120	266	coding	noncoding	noncoding	noncoding
MSTRG.131561.1		XLOC_135122	300	coding	noncoding	noncoding	noncoding
MSTRG.131608.3	ARRDC1-AS1	XLOC_135123	3180	coding	coding	coding	coding
MSTRG.131581.1	CACNA1B	XLOC_135132	276	coding	noncoding	noncoding	noncoding
MSTRG.131590.1	CACNA1B	XLOC_135136	297	coding	noncoding	noncoding	noncoding
MSTRG.131575.1		XLOC_135139	249	coding	coding	noncoding	noncoding
MSTRG.131600.4	LOC112267861	XLOC_135142	4276	coding	coding	coding	noncoding
MSTRG.131604.1		XLOC_135143	397	coding	noncoding	noncoding	noncoding
NR_163254.1	ZMYND11	XLOC_135150	3842	coding	coding	coding	coding
MSTRG.131656.1	ZMYND11	XLOC_135163	237	coding	noncoding	noncoding	noncoding
MSTRG.131633.1		XLOC_135196	237	coding	noncoding	noncoding	noncoding
NM_012341.3	GTPBP4	XLOC_135210	4656	coding	coding	coding	coding
NR_024628.1	IDI2-AS1	XLOC_135212	1088	coding	noncoding	coding	coding
MSTRG.131722.1		XLOC_135238	317	coding	noncoding	noncoding	noncoding

MSTRG.131796.1		XLOC_135258	334	coding	coding	noncoding	noncoding
MSTRG.131805.1		XLOC_135265	290	coding	noncoding	noncoding	noncoding
MSTRG.131837.1		XLOC_135272	225	coding	noncoding	noncoding	noncoding
MSTRG.131842.1	LOC105376356	XLOC_135276	295	coding	noncoding	noncoding	noncoding
MSTRG.131852.1	LOC105376360	XLOC_135279	266	coding	noncoding	noncoding	noncoding
MSTRG.131853.1	LOC105376360	XLOC_135280	309	coding	noncoding	noncoding	noncoding
MSTRG.131868.1	LOC105376360	XLOC_135286	253	coding	noncoding	noncoding	noncoding
MSTRG.131872.1	LOC105376360	XLOC_135287	249	coding	noncoding	noncoding	noncoding
MSTRG.131973.28	AKR1E2	XLOC_135336	512	coding	coding	coding	coding
MSTRG.131977.1	AKR1C6P	XLOC_135338	234	coding	noncoding	noncoding	noncoding
MSTRG.132017.1	NET1	XLOC_135397	268	coding	noncoding	noncoding	noncoding
MSTRG.132081.1		XLOC_135410	223	coding	noncoding	noncoding	noncoding
MSTRG.132086.1		XLOC_135412	222	coding	noncoding	noncoding	noncoding
MSTRG.132087.1		XLOC_135413	216	coding	noncoding	noncoding	noncoding
NM_001258453.1	FBH1	XLOC_135422	3688	coding	coding	coding	coding
MSTRG.132507.1		XLOC_135444	296	coding	noncoding	noncoding	noncoding
MSTRG.132510.1		XLOC_135447	313	coding	noncoding	noncoding	noncoding
MSTRG.132550.1	LINC02648	XLOC_135481	351	coding	noncoding	noncoding	noncoding
MSTRG.132212.1	LINC02642	XLOC_135541	473	coding	noncoding	noncoding	noncoding
MSTRG.132228.1	ITIH5	XLOC_135551	342	coding	noncoding	noncoding	noncoding
MSTRG.132323.1	TAF3	XLOC_135567	285	coding	noncoding	noncoding	noncoding
MSTRG.132436.1	LOC101928272	XLOC_135694	265	coding	noncoding	noncoding	noncoding
MSTRG.132437.1		XLOC_135695	284	coding	noncoding	coding	noncoding
MSTRG.132467.1	LINC02663	XLOC_135698	545	coding	noncoding	noncoding	noncoding
MSTRG.132457.1	LOC105376403	XLOC_135704	278	coding	noncoding	noncoding	noncoding
MSTRG.132484.1		XLOC_135712	283	coding	noncoding	noncoding	noncoding
MSTRG.132487.1		XLOC_135713	300	coding	noncoding	noncoding	noncoding
MSTRG.132496.1	CELF2-DT	XLOC_135716	264	coding	noncoding	noncoding	noncoding
XM_017015550.2	CELF2	XLOC_135717	4805	coding	coding	coding	coding
NM_001025076.2	CELF2	XLOC_135717	8044	coding	coding	coding	coding
MSTRG.132654.1	CELF2	XLOC_135719	411	coding	noncoding	noncoding	noncoding
MSTRG.132674.1	CELF2	XLOC_135727	238	coding	noncoding	noncoding	noncoding
MSTRG.132689.1	CELF2	XLOC_135734	324	coding	noncoding	noncoding	noncoding
MSTRG.132603.5	USP6NL	XLOC_135776	1191	coding	coding	noncoding	noncoding
MSTRG.132558.1		XLOC_135784	397	coding	noncoding	noncoding	noncoding
MSTRG.132574.1		XLOC_135787	231	coding	noncoding	noncoding	noncoding
MSTRG.132616.2	UPF2	XLOC_135795	5270	coding	coding	noncoding	noncoding
MSTRG.132597.1		XLOC_135797	283	coding	noncoding	noncoding	noncoding
MSTRG.132634.9	CDC123	XLOC_135800	1470	coding	coding	coding	noncoding
MSTRG.132634.10	CDC123	XLOC_135800	1461	coding	coding	coding	noncoding
XM_011519593.3	CAMK1D	XLOC_135807	1627	coding	coding	coding	coding
MSTRG.132913.1	CAMK1D	XLOC_135837	261	coding	noncoding	noncoding	noncoding
MSTRG.132640.1		XLOC_135882	206	coding	noncoding	noncoding	noncoding

MSTRG.132648.1	MCM10	XLOC_135897	262 coding	noncoding	noncoding	noncoding
MSTRG.132806.1	FRMD4A	XLOC_135928	231 coding	noncoding	noncoding	noncoding
MSTRG.132807.1	FRMD4A	XLOC_135929	205 coding	noncoding	noncoding	noncoding
MSTRG.132832.3	FAM107B	XLOC_135964	4126 coding	coding	noncoding	noncoding
MSTRG.132969.1	LOC105376434	XLOC_135992	276 coding	noncoding	noncoding	noncoding
MSTRG.132984.1	FAM171A1	XLOC_135999	327 coding	noncoding	noncoding	noncoding
MSTRG.132970.1		XLOC_136001	281 coding	noncoding	noncoding	noncoding
MSTRG.132973.1		XLOC_136002	291 coding	noncoding	noncoding	noncoding
MSTRG.133057.1		XLOC_136015	286 coding	noncoding	noncoding	noncoding
MSTRG.133135.1	CUBN	XLOC_136043	273 coding	noncoding	noncoding	noncoding
MSTRG.133171.5	TRDMT1	XLOC_136057	848 coding	noncoding	noncoding	noncoding
MSTRG.133171.6	VIM	XLOC_136057	2611 coding	coding	coding	coding
MSTRG.133159.1		XLOC_136093	297 coding	noncoding	noncoding	noncoding
MSTRG.133164.1	HACD1	XLOC_136095	233 coding	noncoding	noncoding	noncoding
MSTRG.133160.1		XLOC_136097	437 coding	noncoding	noncoding	noncoding
MSTRG.133244.1		XLOC_136109	217 coding	noncoding	noncoding	noncoding
MSTRG.133346.1	CACNB2	XLOC_136124	235 coding	noncoding	noncoding	noncoding
MSTRG.133376.1	NSUN6	XLOC_136142	229 coding	noncoding	noncoding	noncoding
MSTRG.133271.1	LOC105376440	XLOC_136146	405 coding	noncoding	noncoding	noncoding
MSTRG.133273.1	LOC105376440	XLOC_136148	326 coding	noncoding	noncoding	noncoding
MSTRG.133275.1	LOC105376440	XLOC_136149	392 coding	noncoding	noncoding	noncoding
MSTRG.133266.1		XLOC_136155	306 coding	coding	noncoding	noncoding
MSTRG.133326.1		XLOC_136206	281 coding	noncoding	noncoding	noncoding
XR_001747384.2	LOC105376442	XLOC_136208	8519 coding	noncoding	noncoding	noncoding
MSTRG.133422.1	NEBL	XLOC_136223	259 coding	noncoding	noncoding	noncoding
MSTRG.133429.1	NEBL	XLOC_136227	240 coding	noncoding	noncoding	noncoding
MSTRG.133388.1		XLOC_136238	243 coding	noncoding	noncoding	noncoding
MSTRG.133390.1		XLOC_136240	280 coding	noncoding	noncoding	noncoding
MSTRG.133391.1		XLOC_136241	241 coding	noncoding	noncoding	noncoding
MSTRG.133400.1		XLOC_136247	259 coding	noncoding	noncoding	noncoding
XM_024448192.1	MLLT10	XLOC_136251	4593 coding	coding	coding	coding
XM_024448193.1	MLLT10	XLOC_136251	4585 coding	coding	coding	coding
MSTRG.133488.1		XLOC_136278	310 coding	noncoding	noncoding	noncoding
MSTRG.133573.7	PIP4K2A	XLOC_136306	3615 coding	coding	coding	noncoding
MSTRG.133561.1		XLOC_136309	302 coding	noncoding	noncoding	noncoding
MSTRG.133565.1		XLOC_136313	314 coding	noncoding	noncoding	noncoding
MSTRG.133635.1	LOC105376454	XLOC_136342	232 coding	noncoding	noncoding	noncoding
MSTRG.133637.1	LOC105376454	XLOC_136343	270 coding	noncoding	noncoding	noncoding
MSTRG.133670.1	KIAA1217	XLOC_136348	274 coding	noncoding	noncoding	noncoding
MSTRG.133674.1	KIAA1217	XLOC_136351	201 coding	noncoding	noncoding	noncoding
MSTRG.133678.1	KIAA1217	XLOC_136353	225 coding	noncoding	noncoding	noncoding
MSTRG.133680.1	KIAA1217	XLOC_136354	292 coding	noncoding	noncoding	noncoding
MSTRG.133683.1	KIAA1217	XLOC_136355	253 coding	noncoding	noncoding	noncoding

MSTRG.133666.1		XLOC_136374	291	coding	noncoding	noncoding	noncoding
MSTRG.133715.1		XLOC_136381	247	coding	noncoding	noncoding	noncoding
MSTRG.133733.1	GPR158	XLOC_136386	304	coding	noncoding	noncoding	noncoding
MSTRG.133740.1	GPR158	XLOC_136388	223	coding	noncoding	noncoding	noncoding
MSTRG.133784.1	MYO3A	XLOC_136397	319	coding	noncoding	noncoding	noncoding
MSTRG.133789.1	MYO3A	XLOC_136399	246	coding	noncoding	noncoding	noncoding
MSTRG.133824.22	ABI1	XLOC_136418	1025	coding	coding	noncoding	noncoding
MSTRG.133820.1		XLOC_136422	214	coding	noncoding	noncoding	noncoding
MSTRG.133887.1		XLOC_136456	245	coding	noncoding	noncoding	noncoding
MSTRG.133899.1		XLOC_136481	306	coding	noncoding	noncoding	noncoding
MSTRG.133911.1	MKX	XLOC_136484	297	coding	noncoding	noncoding	noncoding
MSTRG.133906.1		XLOC_136488	251	coding	noncoding	noncoding	noncoding
MSTRG.133958.1	ARMC4	XLOC_136491	285	coding	noncoding	noncoding	noncoding
MSTRG.133959.1	ARMC4	XLOC_136492	283	coding	noncoding	noncoding	noncoding
MSTRG.133962.1	ARMC4	XLOC_136494	305	coding	noncoding	noncoding	noncoding
MSTRG.133967.1		XLOC_136508	249	coding	noncoding	noncoding	noncoding
MSTRG.133969.1		XLOC_136509	268	coding	noncoding	noncoding	noncoding
MSTRG.133976.1	LINC02652	XLOC_136511	288	coding	noncoding	noncoding	noncoding
MSTRG.133979.1		XLOC_136524	255	coding	noncoding	noncoding	noncoding
MSTRG.133986.1	LOC107984170	XLOC_136528	234	coding	noncoding	noncoding	noncoding
MSTRG.133984.1		XLOC_136529	278	coding	noncoding	noncoding	noncoding
MSTRG.133988.1		XLOC_136531	259	coding	noncoding	noncoding	noncoding
MSTRG.133994.1	LINC01517	XLOC_136533	320	coding	noncoding	noncoding	noncoding
MSTRG.134006.1		XLOC_136538	248	coding	noncoding	noncoding	noncoding
MSTRG.134007.1		XLOC_136539	318	coding	noncoding	noncoding	noncoding
MSTRG.134008.1		XLOC_136540	244	coding	noncoding	noncoding	noncoding
MSTRG.134012.1		XLOC_136543	411	coding	coding	noncoding	noncoding
MSTRG.134017.1	LOC105376471	XLOC_136548	287	coding	noncoding	noncoding	noncoding
MSTRG.134028.1	LYZL1	XLOC_136554	295	coding	noncoding	noncoding	noncoding
MSTRG.134085.1		XLOC_136569	261	coding	noncoding	noncoding	noncoding
MSTRG.134103.1		XLOC_136578	225	coding	noncoding	noncoding	noncoding
MSTRG.134105.1		XLOC_136580	269	coding	noncoding	noncoding	noncoding
MSTRG.134120.1	GOLGA2P6	XLOC_136592	242	coding	noncoding	noncoding	noncoding
MSTRG.134150.1		XLOC_136604	262	coding	noncoding	noncoding	noncoding
MSTRG.134301.1	LOC105376482	XLOC_136639	467	coding	noncoding	noncoding	noncoding
XM_006717498.2	ZEB1	XLOC_136658	5814	coding	coding	coding	coding
MSTRG.134315.1		XLOC_136670	265	coding	noncoding	noncoding	noncoding
MSTRG.134345.8	LOC107984219	XLOC_136676	5641	coding	coding	coding	noncoding
MSTRG.134323.1		XLOC_136684	306	coding	noncoding	noncoding	noncoding
MSTRG.134726.50	CCDC7	XLOC_136688	508	coding	noncoding	noncoding	noncoding
MSTRG.134733.1	CCDC7	XLOC_136694	227	coding	noncoding	noncoding	noncoding
MSTRG.134775.1	CCDC7	XLOC_136704	232	coding	coding	noncoding	noncoding
MSTRG.134807.1	ITGB1-DT	XLOC_136716	307	coding	noncoding	noncoding	noncoding

MSTRG.134409.1		XLOC_136752	316 coding	noncoding	noncoding	noncoding
MSTRG.134415.1		XLOC_136757	354 coding	noncoding	noncoding	noncoding
MSTRG.134438.1		XLOC_136760	298 coding	noncoding	noncoding	noncoding
MSTRG.134439.1		XLOC_136761	211 coding	coding	noncoding	noncoding
MSTRG.134447.1		XLOC_136782	286 coding	noncoding	noncoding	noncoding
MSTRG.134518.1	CREM	XLOC_136796	345 coding	noncoding	noncoding	noncoding
NM_001282853.1	CCNY	XLOC_136798	4769 coding	coding	coding	coding
NM_001282852.1	CCNY	XLOC_136798	4631 coding	coding	coding	coding
MSTRG.134573.1	CCNY	XLOC_136799	249 coding	noncoding	noncoding	noncoding
MSTRG.134574.1	CCNY	XLOC_136800	314 coding	noncoding	noncoding	noncoding
MSTRG.134533.1		XLOC_136858	316 coding	noncoding	noncoding	noncoding
MSTRG.134535.1		XLOC_136859	313 coding	noncoding	noncoding	noncoding
MSTRG.134537.1	LINC02630	XLOC_136860	266 coding	noncoding	noncoding	noncoding
MSTRG.134564.1		XLOC_136872	2151 coding	coding	noncoding	noncoding
MSTRG.134631.1		XLOC_136882	282 coding	noncoding	noncoding	noncoding
MSTRG.134636.1		XLOC_136888	250 coding	noncoding	noncoding	noncoding
MSTRG.134642.1		XLOC_136890	242 coding	noncoding	noncoding	noncoding
NM_001278178.1	ZNF33A	XLOC_136928	6126 coding	coding	coding	coding
NM_001278171.2	ZNF33A	XLOC_136928	6239 coding	coding	coding	coding
MSTRG.134845.1	ZNF37A	XLOC_136947	207 coding	noncoding	noncoding	noncoding
MSTRG.134719.1	LOC105376500	XLOC_136950	222 coding	noncoding	noncoding	noncoding
NR_024497.2	LINC00999	XLOC_136961	5253 coding	coding	coding	coding
MSTRG.135022.1	LOC105378271	XLOC_137043	285 coding	noncoding	noncoding	noncoding
MSTRG.135030.1		XLOC_137049	352 coding	noncoding	noncoding	noncoding
MSTRG.135048.1	ZNF487	XLOC_137055	284 coding	noncoding	noncoding	noncoding
MSTRG.135082.1		XLOC_137079	452 coding	noncoding	noncoding	noncoding
MSTRG.135096.1		XLOC_137081	278 coding	noncoding	noncoding	noncoding
MSTRG.135135.1	RSU1P2	XLOC_137120	317 coding	noncoding	noncoding	noncoding
MSTRG.135474.1	AGAP10P	XLOC_137136	364 coding	noncoding	noncoding	noncoding
MSTRG.135512.1	LOC107984026	XLOC_137167	267 coding	noncoding	noncoding	noncoding
XR_945930.3	FAM245B	XLOC_137179	6005 coding	noncoding	noncoding	noncoding
MSTRG.135531.2	BMS1P1	XLOC_137180	320 coding	coding	noncoding	noncoding
XM_011539606.3	PTPN20	XLOC_137189	4306 coding	coding	coding	coding
MSTRG.135214.1		XLOC_137207	222 coding	coding	noncoding	noncoding
MSTRG.135245.1	ANXA8	XLOC_137216	220 coding	noncoding	noncoding	noncoding
MSTRG.135310.1	ARHGAP22	XLOC_137267	503 coding	noncoding	noncoding	noncoding
MSTRG.135311.1	ARHGAP22	XLOC_137268	273 coding	noncoding	noncoding	noncoding
MSTRG.135335.1		XLOC_137332	224 coding	noncoding	noncoding	noncoding
MSTRG.135455.3	PARG	XLOC_137357	3404 coding	coding	coding	noncoding
MSTRG.135567.1	WASHC2A	XLOC_137368	353 coding	noncoding	noncoding	noncoding
MSTRG.135568.1	WASHC2A	XLOC_137369	271 coding	noncoding	noncoding	noncoding
MSTRG.135448.1	ASAH2	XLOC_137372	420 coding	coding	noncoding	noncoding
MSTRG.135580.1		XLOC_137390	263 coding	coding	noncoding	noncoding

MSTRG.135673.1	PRKG1	XLOC_137401	296	coding	noncoding	noncoding	noncoding
MSTRG.135678.1	PRKG1	XLOC_137404	247	coding	noncoding	noncoding	noncoding
MSTRG.135696.1	PRKG1	XLOC_137414	291	coding	noncoding	noncoding	noncoding
MSTRG.135598.1		XLOC_137435	246	coding	noncoding	noncoding	noncoding
MSTRG.135613.1		XLOC_137441	268	coding	noncoding	noncoding	noncoding
MSTRG.135615.1		XLOC_137445	212	coding	noncoding	noncoding	noncoding
MSTRG.135616.1		XLOC_137446	323	coding	noncoding	noncoding	noncoding
MSTRG.135638.1	LOC105378308	XLOC_137450	280	coding	coding	noncoding	noncoding
MSTRG.135639.1	LOC105378308	XLOC_137451	278	coding	noncoding	noncoding	noncoding
MSTRG.135652.1		XLOC_137459	233	coding	noncoding	noncoding	noncoding
MSTRG.135653.1		XLOC_137460	210	coding	noncoding	noncoding	noncoding
MSTRG.135841.1	PCDH15	XLOC_137471	292	coding	noncoding	noncoding	noncoding
MSTRG.135849.1	PCDH15	XLOC_137477	254	coding	noncoding	noncoding	noncoding
MSTRG.135859.1	PCDH15	XLOC_137482	302	coding	noncoding	noncoding	noncoding
MSTRG.135879.1	PCDH15	XLOC_137492	214	coding	noncoding	noncoding	noncoding
MSTRG.135735.1		XLOC_137496	267	coding	noncoding	noncoding	noncoding
MSTRG.135772.1		XLOC_137519	273	coding	noncoding	noncoding	noncoding
MSTRG.135789.1		XLOC_137527	325	coding	noncoding	noncoding	noncoding
MSTRG.135790.1		XLOC_137528	262	coding	noncoding	noncoding	noncoding
MSTRG.135796.1		XLOC_137531	317	coding	noncoding	noncoding	noncoding
MSTRG.135798.1		XLOC_137532	232	coding	noncoding	noncoding	noncoding
MSTRG.135804.1		XLOC_137537	461	coding	noncoding	noncoding	noncoding
MSTRG.135890.1	LOC105378314	XLOC_137542	447	coding	noncoding	noncoding	noncoding
MSTRG.135896.1		XLOC_137547	286	coding	noncoding	noncoding	noncoding
NM_018464.5	CISD1	XLOC_137554	2375	coding	coding	coding	noncoding
NM_001270782.1	TFAM	XLOC_137568	5206	coding	coding	noncoding	coding
MSTRG.135928.1	LOC105378316	XLOC_137574	665	coding	noncoding	noncoding	noncoding
MSTRG.135969.1	BICC1	XLOC_137577	218	coding	noncoding	noncoding	noncoding
MSTRG.135972.1	BICC1	XLOC_137578	666	coding	noncoding	noncoding	noncoding
MSTRG.135981.1	BICC1	XLOC_137581	244	coding	noncoding	noncoding	noncoding
MSTRG.135986.1	LOC107984235	XLOC_137595	306	coding	noncoding	noncoding	noncoding
MSTRG.135989.1	LOC107984235	XLOC_137597	282	coding	noncoding	noncoding	noncoding
MSTRG.135984.1		XLOC_137598	277	coding	coding	noncoding	noncoding
MSTRG.135996.1	MRLN	XLOC_137605	432	coding	noncoding	noncoding	noncoding
MSTRG.136100.1		XLOC_137627	285	coding	noncoding	noncoding	noncoding
MSTRG.136109.1		XLOC_137633	248	coding	noncoding	noncoding	noncoding
MSTRG.136209.1	ARID5B	XLOC_137660	284	coding	coding	noncoding	noncoding
MSTRG.136161.1		XLOC_137692	288	coding	noncoding	noncoding	noncoding
MSTRG.136168.1		XLOC_137696	306	coding	noncoding	noncoding	noncoding
MSTRG.136272.1	ZNF365	XLOC_137724	268	coding	noncoding	noncoding	noncoding
MSTRG.136248.1		XLOC_137735	371	coding	noncoding	noncoding	noncoding
MSTRG.136294.1	LOC101929846	XLOC_137758	222	coding	noncoding	noncoding	noncoding
MSTRG.136335.1		XLOC_137776	283	coding	noncoding	noncoding	noncoding

MSTRG.136492.1	CTNNA3	XLOC_137805	306 coding	noncoding	noncoding	noncoding
MSTRG.136493.1	CTNNA3	XLOC_137806	240 coding	noncoding	noncoding	noncoding
MSTRG.136529.1	CTNNA3	XLOC_137828	250 coding	noncoding	noncoding	noncoding
MSTRG.136536.1	CTNNA3	XLOC_137832	259 coding	noncoding	noncoding	noncoding
MSTRG.136545.1	CTNNA3	XLOC_137837	315 coding	coding	noncoding	noncoding
MSTRG.136548.1	CTNNA3	XLOC_137839	249 coding	noncoding	noncoding	noncoding
MSTRG.136551.1		XLOC_137841	311 coding	noncoding	noncoding	noncoding
MSTRG.136477.1		XLOC_137844	280 coding	noncoding	noncoding	noncoding
MSTRG.136474.1		XLOC_137845	299 coding	noncoding	noncoding	noncoding
MSTRG.136472.1		XLOC_137846	303 coding	noncoding	noncoding	noncoding
MSTRG.136563.1	SIRT1	XLOC_137849	362 coding	noncoding	noncoding	noncoding
MSTRG.136593.26	RUFY2	XLOC_137859	2393 coding	coding	noncoding	noncoding
XM_011540205.2	TET1	XLOC_137870	13626 coding	coding	coding	coding
MSTRG.136608.1	TET1	XLOC_137871	317 coding	noncoding	noncoding	noncoding
MSTRG.136613.1	TET1	XLOC_137873	285 coding	noncoding	noncoding	noncoding
MSTRG.136617.1	TET1	XLOC_137874	252 coding	noncoding	noncoding	noncoding
MSTRG.136655.1		XLOC_137882	204 coding	noncoding	noncoding	noncoding
MSTRG.136689.1	SRGN	XLOC_137894	219 coding	noncoding	noncoding	noncoding
MSTRG.136705.1	SUPV3L1	XLOC_137904	243 coding	noncoding	noncoding	noncoding
MSTRG.136710.4	HKDC1	XLOC_137906	335 coding	coding	noncoding	noncoding
MSTRG.136762.1	HK1	XLOC_137908	259 coding	noncoding	noncoding	noncoding
MSTRG.136764.1	HK1	XLOC_137909	280 coding	noncoding	noncoding	noncoding
MSTRG.136767.1	HK1	XLOC_137911	677 coding	noncoding	noncoding	noncoding
MSTRG.136714.1		XLOC_137915	291 coding	noncoding	noncoding	noncoding
MSTRG.136727.1	TSPAN15	XLOC_137920	304 coding	noncoding	noncoding	noncoding
MSTRG.136734.1	TSPAN15	XLOC_137923	309 coding	noncoding	noncoding	noncoding
MSTRG.136718.1		XLOC_137928	292 coding	coding	noncoding	noncoding
MSTRG.136798.1	H2AFY2	XLOC_137950	267 coding	noncoding	noncoding	noncoding
MSTRG.136821.1	LRRC20	XLOC_137963	280 coding	noncoding	noncoding	noncoding
XM_011539301.2	ADAMTS14	XLOC_137982	4563 coding	coding	coding	coding
XR_002957082.1	LOC112268061	XLOC_138008	14684 coding	noncoding	coding	noncoding
MSTRG.136905.1	UNC5B	XLOC_138009	393 coding	noncoding	noncoding	noncoding
MSTRG.136926.1	SLC29A3	XLOC_138016	299 coding	noncoding	noncoding	noncoding
XM_011540369.2	CHST3	XLOC_138024	8897 coding	coding	coding	coding
NM_001242546.1	ANAPC16	XLOC_138032	3569 coding	coding	coding	noncoding
MSTRG.136991.1		XLOC_138041	291 coding	noncoding	noncoding	noncoding
MSTRG.137019.1	OIT3	XLOC_138060	281 coding	noncoding	noncoding	noncoding
MSTRG.137025.1	PLA2G12B	XLOC_138062	394 coding	coding	noncoding	noncoding
MSTRG.137132.1	CFAP70	XLOC_138076	284 coding	noncoding	noncoding	noncoding
MSTRG.137064.1	LOC105378360	XLOC_138102	267 coding	noncoding	noncoding	noncoding
XR_002956964.1	FUT11	XLOC_138109	1647 coding	coding	coding	coding
NM_003373.4	VCL	XLOC_138120	6487 coding	coding	coding	coding
MSTRG.137304.1	ADK	XLOC_138168	302 coding	noncoding	noncoding	noncoding

MSTRG.137157.1		XLOC_138173	261 coding	noncoding	noncoding	noncoding
NM_001256469.1	KAT6B	XLOC_138174	7623 coding	coding	coding	coding
MSTRG.137243.1	VDAC2	XLOC_138187	317 coding	noncoding	noncoding	noncoding
MSTRG.138105.1	LRMDA	XLOC_138255	308 coding	noncoding	noncoding	noncoding
MSTRG.138117.1	LOC112268057	XLOC_138266	300 coding	noncoding	noncoding	noncoding
MSTRG.138123.1	LRMDA	XLOC_138272	262 coding	noncoding	noncoding	noncoding
MSTRG.137321.1		XLOC_138275	276 coding	noncoding	noncoding	noncoding
MSTRG.137374.1	KCNMA1	XLOC_138293	257 coding	noncoding	noncoding	noncoding
MSTRG.137332.1		XLOC_138303	269 coding	noncoding	noncoding	noncoding
NM_001026.4	RPS24	XLOC_138309	634 coding	coding	coding	coding
MSTRG.137474.1		XLOC_138379	215 coding	noncoding	noncoding	noncoding
MSTRG.137484.1		XLOC_138380	227 coding	noncoding	noncoding	noncoding
NM_001099692.1	EIF5AL1	XLOC_138382	3840 coding	coding	coding	coding
MSTRG.137495.1		XLOC_138396	1077 coding	noncoding	noncoding	noncoding
MSTRG.137498.1		XLOC_138398	1952 coding	noncoding	noncoding	noncoding
MSTRG.137503.1	BMS1P21	XLOC_138401	257 coding	noncoding	noncoding	noncoding
MSTRG.137515.1	TMEM254-AS1	XLOC_138410	205 coding	noncoding	noncoding	noncoding
MSTRG.137516.1	TMEM254-AS1	XLOC_138411	232 coding	noncoding	noncoding	noncoding
MSTRG.137518.1	TMEM254-AS1	XLOC_138413	298 coding	noncoding	noncoding	noncoding
NM_001270371.1	TMEM254	XLOC_138414	2155 coding	coding	coding	noncoding
MSTRG.137511.1		XLOC_138415	264 coding	noncoding	noncoding	noncoding
NM_001012973.3	PLAC9	XLOC_138416	776 coding	coding	coding	coding
MSTRG.137598.3	ANXA11	XLOC_138417	11677 coding	coding	coding	noncoding
MSTRG.137581.1		XLOC_138421	278 coding	noncoding	noncoding	noncoding
MSTRG.137620.1	LOC105378387	XLOC_138440	272 coding	noncoding	noncoding	noncoding
MSTRG.137625.1		XLOC_138445	304 coding	noncoding	noncoding	noncoding
MSTRG.137626.1		XLOC_138446	317 coding	noncoding	noncoding	noncoding
MSTRG.137628.1		XLOC_138448	220 coding	noncoding	noncoding	noncoding
MSTRG.137629.1		XLOC_138449	404 coding	noncoding	noncoding	noncoding
MSTRG.137645.1		XLOC_138458	217 coding	noncoding	noncoding	noncoding
MSTRG.137685.1		XLOC_138497	266 coding	noncoding	noncoding	noncoding
MSTRG.137850.1	GRID1	XLOC_138567	257 coding	noncoding	noncoding	noncoding
MSTRG.137852.1	GRID1	XLOC_138568	268 coding	noncoding	noncoding	noncoding
MSTRG.137900.7	WAPL	XLOC_138591	1773 coding	noncoding	coding	noncoding
MSTRG.137890.1		XLOC_138594	249 coding	noncoding	noncoding	noncoding
MSTRG.137899.1	LDB3	XLOC_138599	233 coding	noncoding	noncoding	noncoding
MSTRG.137937.2	SHLD2	XLOC_138614	4484 coding	coding	coding	coding
NM_019054.3	SHLD2	XLOC_138614	3394 coding	coding	coding	coding
NM_001178117.1	MINPP1	XLOC_138639	2665 coding	coding	coding	coding
MSTRG.138039.1		XLOC_138670	234 coding	noncoding	noncoding	noncoding
MSTRG.138229.1	RNLS	XLOC_138693	393 coding	coding	noncoding	noncoding
MSTRG.138486.1		XLOC_138860	229 coding	noncoding	noncoding	noncoding
MSTRG.138492.1		XLOC_138863	236 coding	noncoding	noncoding	noncoding

MSTRG.138519.1		XLOC_138868	238 coding	noncoding	noncoding	noncoding
MSTRG.138577.1	HECTD2-AS1	XLOC_138934	283 coding	noncoding	noncoding	noncoding
MSTRG.138591.1	HECTD2-AS1	XLOC_138941	260 coding	noncoding	noncoding	noncoding
MSTRG.138600.1	HECTD2-AS1	XLOC_138948	284 coding	noncoding	noncoding	noncoding
MSTRG.138566.1		XLOC_138952	317 coding	noncoding	noncoding	noncoding
MSTRG.138573.1		XLOC_138955	272 coding	noncoding	noncoding	noncoding
XM_005270185.4	TNKS2	XLOC_138956	6122 coding	coding	coding	coding
MSTRG.138664.1	BTAF1	XLOC_138972	230 coding	noncoding	noncoding	noncoding
MSTRG.138671.1	BTAF1	XLOC_138979	245 coding	noncoding	noncoding	noncoding
MSTRG.138678.1		XLOC_138993	249 coding	noncoding	noncoding	noncoding
MSTRG.138736.1		XLOC_139040	269 coding	noncoding	noncoding	noncoding
MSTRG.138740.1		XLOC_139043	230 coding	noncoding	noncoding	noncoding
MSTRG.138821.1	TBC1D12	XLOC_139078	261 coding	noncoding	noncoding	noncoding
MSTRG.138891.1	HELLS	XLOC_139081	270 coding	noncoding	noncoding	noncoding
MSTRG.138893.1	HELLS	XLOC_139083	240 coding	noncoding	noncoding	noncoding
MSTRG.138912.1		XLOC_139097	298 coding	noncoding	noncoding	noncoding
MSTRG.138915.1		XLOC_139120	209 coding	noncoding	noncoding	noncoding
MSTRG.138967.1	SORBS1	XLOC_139133	280 coding	noncoding	noncoding	noncoding
XM_011540377.2	ENTPD1	XLOC_139141	12619 coding	coding	coding	coding
MSTRG.139154.1	ENTPD1-AS1	XLOC_139163	245 coding	noncoding	noncoding	noncoding
MSTRG.139173.1	ENTPD1-AS1	XLOC_139182	347 coding	noncoding	noncoding	noncoding
MSTRG.138980.1		XLOC_139234	245 coding	noncoding	noncoding	noncoding
MSTRG.138990.1	OPALIN	XLOC_139243	322 coding	noncoding	noncoding	noncoding
MSTRG.139023.1		XLOC_139250	299 coding	noncoding	noncoding	noncoding
MSTRG.139024.1		XLOC_139251	312 coding	noncoding	noncoding	noncoding
MSTRG.139108.1	SLIT1	XLOC_139281	315 coding	noncoding	noncoding	noncoding
MSTRG.139116.1	ARHGAP19-SLIT1	XLOC_139286	239 coding	noncoding	noncoding	noncoding
MSTRG.139336.1	UBTD1	XLOC_139306	300 coding	noncoding	noncoding	noncoding
MSTRG.139068.1	PI4K2A	XLOC_139312	250 coding	noncoding	noncoding	noncoding
MSTRG.139061.1		XLOC_139315	240 coding	noncoding	noncoding	noncoding
NM_001010917.3	GOLGA7B	XLOC_139329	6843 coding	coding	coding	coding
MSTRG.139310.1	LOC105378449	XLOC_139356	431 coding	noncoding	noncoding	noncoding
MSTRG.139370.1	HPSE2	XLOC_139368	289 coding	noncoding	noncoding	noncoding
MSTRG.139390.1	HPSE2	XLOC_139379	277 coding	noncoding	noncoding	noncoding
MSTRG.139412.1		XLOC_139388	295 coding	noncoding	noncoding	noncoding
MSTRG.139459.1	CUTC	XLOC_139395	364 coding	noncoding	noncoding	noncoding
MSTRG.139447.1	CPN1	XLOC_139415	244 coding	noncoding	noncoding	noncoding
MSTRG.139484.1		XLOC_139426	317 coding	noncoding	noncoding	noncoding
MSTRG.139590.1	PDZD7	XLOC_139459	308 coding	noncoding	noncoding	noncoding
MSTRG.139568.9	KAZALD1	XLOC_139462	312 coding	coding	noncoding	noncoding
MSTRG.139575.1	TLX1NB	XLOC_139465	289 coding	noncoding	noncoding	noncoding
XM_017016872.1	BTRC	XLOC_139473	5503 coding	coding	coding	coding
MSTRG.139608.1	BTRC	XLOC_139486	244 coding	noncoding	noncoding	noncoding

MSTRG.139648.1	NOLC1	XLOC_139516	291	coding	noncoding	noncoding	noncoding
MSTRG.139755.1	SUFU	XLOC_139544	303	coding	noncoding	noncoding	noncoding
MSTRG.139905.1	BORCS7-ASMT	XLOC_139568	317	coding	noncoding	noncoding	noncoding
MSTRG.139789.1		XLOC_139576	241	coding	noncoding	noncoding	noncoding
MSTRG.139793.1		XLOC_139578	265	coding	noncoding	noncoding	noncoding
NM_032727.4	INA	XLOC_139579	3251	coding	coding	coding	coding
MSTRG.139810.1	NEURL1	XLOC_139588	275	coding	noncoding	noncoding	noncoding
MSTRG.139850.1	SH3PXD2A	XLOC_139606	276	coding	noncoding	noncoding	noncoding
MSTRG.139851.1	SH3PXD2A	XLOC_139607	343	coding	noncoding	noncoding	noncoding
MSTRG.139861.1	SH3PXD2A	XLOC_139612	271	coding	noncoding	noncoding	noncoding
MSTRG.139894.1	CFAP43	XLOC_139636	289	coding	noncoding	noncoding	noncoding
MSTRG.139950.1		XLOC_139643	280	coding	noncoding	noncoding	noncoding
NM_014978.3	SORCS3	XLOC_139649	5575	coding	coding	coding	coding
MSTRG.140117.1	SORCS1	XLOC_139687	738	coding	coding	noncoding	noncoding
MSTRG.140087.1		XLOC_139694	266	coding	noncoding	noncoding	noncoding
MSTRG.140102.1		XLOC_139698	263	coding	noncoding	noncoding	noncoding
MSTRG.140216.1		XLOC_139708	323	coding	noncoding	noncoding	noncoding
MSTRG.140228.1		XLOC_139712	759	coding	noncoding	noncoding	noncoding
MSTRG.140133.1	LINC01435	XLOC_139716	231	coding	noncoding	noncoding	noncoding
MSTRG.140144.1	LOC105378477	XLOC_139721	314	coding	noncoding	noncoding	noncoding
MSTRG.140142.1		XLOC_139723	301	coding	noncoding	noncoding	noncoding
MSTRG.140164.1		XLOC_139730	266	coding	coding	noncoding	noncoding
MSTRG.140165.1		XLOC_139731	280	coding	noncoding	noncoding	noncoding
MSTRG.140167.1		XLOC_139732	246	coding	noncoding	noncoding	noncoding
MSTRG.140171.1		XLOC_139733	269	coding	noncoding	noncoding	noncoding
MSTRG.140237.1		XLOC_139734	296	coding	noncoding	noncoding	noncoding
MSTRG.140235.1		XLOC_139736	260	coding	noncoding	noncoding	noncoding
NM_004419.4	DUSP5	XLOC_139795	2477	coding	coding	coding	coding
MSTRG.140353.1	LOC105378483	XLOC_139799	448	coding	noncoding	noncoding	noncoding
MSTRG.140357.1	SMC3	XLOC_139801	292	coding	noncoding	noncoding	noncoding
MSTRG.140331.1		XLOC_139803	483	coding	noncoding	noncoding	noncoding
NM_001324337.1	SHOC2	XLOC_139812	4132	coding	coding	coding	coding
NM_001269039.2	SHOC2	XLOC_139812	3809	coding	coding	coding	coding
MSTRG.140360.1		XLOC_139823	233	coding	noncoding	noncoding	noncoding
MSTRG.140365.1		XLOC_139825	292	coding	noncoding	noncoding	noncoding
MSTRG.140388.1		XLOC_139833	273	coding	noncoding	noncoding	noncoding
MSTRG.140397.1		XLOC_139836	274	coding	coding	noncoding	noncoding
MSTRG.140406.1		XLOC_139842	390	coding	noncoding	coding	noncoding
MSTRG.140421.1	LOC105378486	XLOC_139847	204	coding	noncoding	noncoding	noncoding
MSTRG.140435.1	LOC105378486	XLOC_139855	309	coding	noncoding	coding	noncoding
MSTRG.140437.1		XLOC_139869	486	coding	noncoding	noncoding	noncoding
NM_016234.3	ACSL5	XLOC_139874	3354	coding	coding	coding	coding
MSTRG.140474.1		XLOC_139948	229	coding	noncoding	noncoding	noncoding

MSTRG.140478.1	LOC107984158	XLOC_139950	241 coding	noncoding	noncoding	noncoding
MSTRG.140590.1		XLOC_139975	204 coding	noncoding	noncoding	noncoding
MSTRG.140524.1	NRAP	XLOC_139989	257 coding	coding	noncoding	noncoding
XM_011539769.3	NHLRC2	XLOC_139999	2181 coding	coding	coding	coding
MSTRG.140548.1		XLOC_140011	248 coding	noncoding	noncoding	noncoding
MSTRG.140814.2	ABLIM1	XLOC_140029	8227 coding	coding	noncoding	noncoding
MSTRG.140618.1	FAM160B1	XLOC_140042	209 coding	coding	noncoding	noncoding
MSTRG.140717.1	LOC107984272	XLOC_140052	238 coding	noncoding	noncoding	noncoding
MSTRG.140719.1	LOC107984272	XLOC_140053	313 coding	noncoding	noncoding	noncoding
MSTRG.140721.1	LOC107984272	XLOC_140055	247 coding	noncoding	noncoding	noncoding
MSTRG.140779.1	ATRNL1	XLOC_140061	271 coding	noncoding	noncoding	noncoding
MSTRG.140784.1	ATRNL1	XLOC_140062	298 coding	noncoding	noncoding	noncoding
MSTRG.140786.1	ATRNL1	XLOC_140063	220 coding	noncoding	noncoding	noncoding
MSTRG.140787.1	ATRNL1	XLOC_140064	244 coding	noncoding	noncoding	noncoding
MSTRG.140736.1		XLOC_140078	266 coding	noncoding	noncoding	noncoding
MSTRG.140834.1	C10orf82	XLOC_140097	229 coding	noncoding	noncoding	noncoding
XM_011539797.3	ENO4	XLOC_140104	7224 coding	coding	coding	coding
MSTRG.140858.1	SHTN1	XLOC_140108	206 coding	noncoding	noncoding	noncoding
MSTRG.140901.3	PDZD8	XLOC_140112	3608 coding	coding	coding	noncoding
MSTRG.140868.1		XLOC_140121	273 coding	noncoding	noncoding	noncoding
MSTRG.140883.1		XLOC_140130	264 coding	noncoding	noncoding	noncoding
MSTRG.140888.1		XLOC_140132	280 coding	noncoding	noncoding	noncoding
MSTRG.140985.1		XLOC_140167	309 coding	noncoding	noncoding	noncoding
MSTRG.140986.1		XLOC_140168	286 coding	noncoding	noncoding	noncoding
MSTRG.140997.1		XLOC_140174	252 coding	noncoding	noncoding	noncoding
XM_017016263.1	FAM45A	XLOC_140180	2913 coding	coding	coding	coding
MSTRG.141103.11	INPP5F	XLOC_140209	231 coding	coding	noncoding	noncoding
MSTRG.141066.1		XLOC_140213	234 coding	noncoding	noncoding	noncoding
MSTRG.141068.1		XLOC_140214	319 coding	noncoding	noncoding	noncoding
MSTRG.141075.1		XLOC_140217	310 coding	noncoding	noncoding	noncoding
MSTRG.141083.1	LOC105378515	XLOC_140221	289 coding	noncoding	noncoding	noncoding
NM_001318168.1	PLPP4	XLOC_140225	1250 coding	coding	noncoding	noncoding
XR_428707.3	WDR11	XLOC_140234	4422 coding	noncoding	coding	coding
MSTRG.141214.10	WDR11	XLOC_140234	4156 coding	noncoding	coding	coding
MSTRG.141217.1	WDR11	XLOC_140237	224 coding	coding	noncoding	noncoding
MSTRG.141130.1	LOC105378521	XLOC_140242	438 coding	noncoding	noncoding	noncoding
MSTRG.141133.1	LOC105378522	XLOC_140245	383 coding	coding	noncoding	noncoding
MSTRG.141155.1		XLOC_140257	292 coding	noncoding	noncoding	noncoding
MSTRG.141169.1		XLOC_140276	236 coding	noncoding	noncoding	noncoding
MSTRG.141265.1	ATE1-AS1	XLOC_140284	278 coding	noncoding	noncoding	noncoding
XM_024447757.1	TACC2	XLOC_140289	10173 coding	coding	noncoding	coding
MSTRG.141249.1	HTRA1	XLOC_140306	336 coding	noncoding	noncoding	noncoding
XM_006717660.3	DMBT1	XLOC_140309	8013 coding	coding	coding	coding

MSTRG.141287.1		XLOC_140312	227 coding	noncoding	noncoding	noncoding
MSTRG.141283.1		XLOC_140316	495 coding	noncoding	noncoding	noncoding
NM_153442.4	GPR26	XLOC_140363	10306 coding	coding	coding	coding
MSTRG.141366.1		XLOC_140382	276 coding	noncoding	noncoding	noncoding
XM_011540058.3	LHPP	XLOC_140387	4081 coding	coding	coding	coding
MSTRG.141544.1	FAM53B	XLOC_140392	7700 coding	noncoding	coding	noncoding
XM_017015986.1	ABRAXAS2	XLOC_140401	2870 coding	noncoding	coding	coding
MSTRG.141525.1		XLOC_140403	256 coding	noncoding	noncoding	noncoding
XM_006717907.2	ZRANB1	XLOC_140405	5661 coding	coding	coding	coding
MSTRG.141575.1		XLOC_140408	266 coding	noncoding	noncoding	noncoding
MSTRG.141587.1		XLOC_140414	307 coding	noncoding	noncoding	noncoding
MSTRG.141627.1	MMP21	XLOC_140436	273 coding	coding	noncoding	noncoding
MSTRG.141682.1	ADAM12	XLOC_140450	222 coding	noncoding	noncoding	noncoding
MSTRG.141692.1	ADAM12	XLOC_140459	240 coding	noncoding	noncoding	noncoding
MSTRG.141666.1	C10orf90	XLOC_140494	278 coding	noncoding	noncoding	noncoding
MSTRG.141669.1	C10orf90	XLOC_140496	217 coding	noncoding	noncoding	noncoding
MSTRG.141782.1	DOCK1	XLOC_140516	223 coding	noncoding	noncoding	noncoding
MSTRG.141732.1		XLOC_140517	245 coding	noncoding	noncoding	noncoding
MSTRG.141733.1		XLOC_140518	280 coding	noncoding	noncoding	noncoding
MSTRG.141752.1	CLRN3	XLOC_140533	305 coding	noncoding	noncoding	noncoding
MSTRG.141844.1		XLOC_140566	354 coding	noncoding	noncoding	noncoding
MSTRG.142045.1		XLOC_140667	380 coding	noncoding	noncoding	noncoding
MSTRG.142053.1		XLOC_140675	223 coding	noncoding	noncoding	noncoding
MSTRG.142109.1		XLOC_140700	285 coding	noncoding	noncoding	noncoding
NM_001321042.1	INPP5A	XLOC_140704	3061 coding	coding	coding	coding
MSTRG.142159.1	CFAP46	XLOC_140727	227 coding	noncoding	noncoding	noncoding
NM_001083909.2	ADGRA1	XLOC_140740	4184 coding	coding	coding	coding
NM_152643.8	KNDC1	XLOC_140744	7021 coding	coding	coding	coding
MSTRG.142214.1		XLOC_140755	222 coding	noncoding	noncoding	noncoding
NR_149718.1	SCART1	XLOC_140768	4315 coding	coding	coding	coding
MSTRG.142225.1		XLOC_140774	304 coding	noncoding	noncoding	noncoding
MSTRG.142227.1	FRG2B	XLOC_140775	400 coding	noncoding	noncoding	noncoding
MSTRG.131635.1		XLOC_140795	322 coding	noncoding	noncoding	noncoding
MSTRG.131813.1	LOC107984285	XLOC_140812	244 coding	noncoding	noncoding	noncoding
NM_001317956.2	IDI1	XLOC_140816	3373 coding	coding	coding	coding
MSTRG.131826.14	WDR37	XLOC_140816	1312 coding	coding	noncoding	noncoding
MSTRG.131718.1		XLOC_140839	257 coding	noncoding	noncoding	noncoding
MSTRG.131807.1	LOC105376352	XLOC_140869	310 coding	noncoding	noncoding	noncoding
XR_001747000.1	PITRM1	XLOC_140872	3657 coding	coding	coding	coding
MSTRG.131836.1		XLOC_140880	225 coding	noncoding	noncoding	noncoding
MSTRG.131882.1	LOC105376360	XLOC_140904	263 coding	noncoding	noncoding	noncoding
MSTRG.131899.1		XLOC_140914	270 coding	noncoding	noncoding	noncoding
MSTRG.131990.1		XLOC_140952	288 coding	noncoding	noncoding	noncoding

NR_037164.1	ASB13	XLOC_140991	2808 coding	coding	coding	coding
NM_001115156.1	GDI2	XLOC_140993	2277 coding	coding	coding	coding
MSTRG.132083.1		XLOC_140997	221 coding	noncoding	noncoding	noncoding
MSTRG.132085.1		XLOC_140998	259 coding	noncoding	noncoding	noncoding
NM_019046.3	ANKRD16	XLOC_141000	2734 coding	coding	coding	coding
NM_001351096.1	IL15RA	XLOC_141003	1823 coding	noncoding	coding	coding
MSTRG.132126.1	LOC101928080	XLOC_141013	299 coding	noncoding	noncoding	noncoding
MSTRG.132497.4	PFKFB3	XLOC_141014	2561 coding	coding	coding	coding
NM_001242413.2	PRKCQ	XLOC_141020	3096 coding	coding	coding	coding
MSTRG.132224.1	ITIH5	XLOC_141081	253 coding	noncoding	noncoding	noncoding
MSTRG.132226.1	ITIH5	XLOC_141082	225 coding	noncoding	noncoding	noncoding
MSTRG.132276.1		XLOC_141102	276 coding	coding	noncoding	noncoding
MSTRG.132439.1		XLOC_141130	274 coding	noncoding	noncoding	noncoding
MSTRG.132435.1	LOC101928272	XLOC_141139	255 coding	noncoding	noncoding	noncoding
MSTRG.132438.1		XLOC_141142	284 coding	noncoding	noncoding	noncoding
MSTRG.132464.1	LINC02663	XLOC_141144	209 coding	noncoding	noncoding	noncoding
MSTRG.132469.1	LINC02663	XLOC_141147	232 coding	noncoding	noncoding	noncoding
MSTRG.132460.1		XLOC_141154	243 coding	noncoding	noncoding	noncoding
MSTRG.132488.1		XLOC_141160	234 coding	noncoding	noncoding	noncoding
MSTRG.132495.1	CELF2-DT	XLOC_141166	373 coding	noncoding	noncoding	noncoding
MSTRG.132656.1	CELF2	XLOC_141168	262 coding	noncoding	noncoding	noncoding
MSTRG.132667.1	CELF2	XLOC_141174	273 coding	noncoding	noncoding	noncoding
MSTRG.132684.21	CELF2	XLOC_141185	2226 coding	coding	coding	noncoding
MSTRG.132575.1		XLOC_141210	231 coding	noncoding	noncoding	noncoding
MSTRG.132591.1	PROSER2	XLOC_141219	262 coding	noncoding	noncoding	noncoding
MSTRG.132620.1	UPF2	XLOC_141227	234 coding	noncoding	noncoding	noncoding
MSTRG.132623.1	UPF2	XLOC_141230	256 coding	noncoding	noncoding	noncoding
MSTRG.132596.1		XLOC_141236	283 coding	noncoding	noncoding	noncoding
MSTRG.132876.6	CAMK1D	XLOC_141249	3735 coding	coding	coding	coding
MSTRG.132881.2	CAMK1D	XLOC_141250	2574 coding	noncoding	coding	noncoding
MSTRG.132741.1	CCDC3	XLOC_141268	244 coding	noncoding	noncoding	noncoding
MSTRG.132743.1	CCDC3	XLOC_141269	386 coding	coding	noncoding	noncoding
MSTRG.132749.10	OPTN	XLOC_141271	2891 coding	coding	coding	coding
MSTRG.132749.12	OPTN	XLOC_141271	2176 coding	coding	noncoding	noncoding
MSTRG.132643.1		XLOC_141279	300 coding	noncoding	noncoding	noncoding
MSTRG.132644.1	PHYH	XLOC_141280	244 coding	noncoding	noncoding	noncoding
MSTRG.132728.1	LOC105376419	XLOC_141288	302 coding	noncoding	noncoding	noncoding
MSTRG.132799.1	FRMD4A	XLOC_141300	22715 coding	coding	coding	coding
MSTRG.132862.1	FAM107B	XLOC_141332	291 coding	noncoding	noncoding	noncoding
MSTRG.133044.1	ACBD7-DCLRE1CP1	XLOC_141345	300 coding	noncoding	noncoding	noncoding
MSTRG.132976.1	FAM171A1	XLOC_141361	228 coding	noncoding	noncoding	noncoding
MSTRG.132980.1	FAM171A1	XLOC_141362	302 coding	noncoding	noncoding	noncoding
MSTRG.132972.1		XLOC_141366	346 coding	noncoding	noncoding	noncoding

MSTRG.132991.1	ITGA8	XLOC_141368	310 coding	noncoding	noncoding	noncoding
MSTRG.132988.1		XLOC_141373	414 coding	noncoding	noncoding	noncoding
MSTRG.133065.1		XLOC_141397	214 coding	noncoding	noncoding	noncoding
MSTRG.133056.1		XLOC_141399	237 coding	noncoding	noncoding	noncoding
XM_005252552.4	RSU1	XLOC_141412	3456 coding	coding	coding	coding
MSTRG.133136.1	CUBN	XLOC_141432	273 coding	noncoding	noncoding	noncoding
MSTRG.133148.1	CUBN	XLOC_141436	729 coding	noncoding	noncoding	noncoding
MSTRG.133154.1		XLOC_141448	201 coding	noncoding	noncoding	noncoding
MSTRG.133205.1	ST8SIA6-AS1	XLOC_141456	272 coding	noncoding	noncoding	noncoding
MSTRG.133158.1		XLOC_141462	313 coding	noncoding	noncoding	noncoding
MSTRG.133161.1		XLOC_141464	437 coding	noncoding	noncoding	noncoding
MSTRG.133261.1		XLOC_141481	289 coding	noncoding	noncoding	noncoding
MSTRG.133344.1	CACNB2	XLOC_141490	282 coding	noncoding	noncoding	noncoding
MSTRG.133347.1	CACNB2	XLOC_141491	235 coding	noncoding	noncoding	noncoding
MSTRG.133372.1	NSUN6	XLOC_141499	239 coding	noncoding	noncoding	noncoding
MSTRG.133270.1	LOC105376440	XLOC_141504	360 coding	noncoding	noncoding	noncoding
MSTRG.133274.1	LOC105376440	XLOC_141505	407 coding	noncoding	noncoding	noncoding
MSTRG.133267.1		XLOC_141507	324 coding	coding	noncoding	noncoding
MSTRG.133286.1	MALRD1	XLOC_141510	214 coding	noncoding	noncoding	noncoding
MSTRG.133442.8	PLXDC2	XLOC_141527	3299 coding	noncoding	noncoding	noncoding
MSTRG.133319.1		XLOC_141541	270 coding	noncoding	noncoding	noncoding
MSTRG.133320.1		XLOC_141542	203 coding	coding	noncoding	noncoding
MSTRG.133332.1		XLOC_141547	242 coding	noncoding	noncoding	noncoding
MSTRG.133334.1		XLOC_141548	311 coding	noncoding	noncoding	noncoding
MSTRG.133411.1	NEBL	XLOC_141553	272 coding	noncoding	noncoding	noncoding
MSTRG.133423.1	NEBL	XLOC_141557	259 coding	noncoding	noncoding	noncoding
MSTRG.133384.1		XLOC_141564	246 coding	noncoding	noncoding	noncoding
MSTRG.133386.1		XLOC_141565	301 coding	noncoding	noncoding	noncoding
MSTRG.133392.1		XLOC_141568	289 coding	noncoding	noncoding	noncoding
MSTRG.133401.1		XLOC_141571	259 coding	noncoding	noncoding	noncoding
MSTRG.133406.1		XLOC_141574	243 coding	noncoding	noncoding	noncoding
XM_017016226.2	SKIDA1	XLOC_141577	9225 coding	coding	coding	coding
MSTRG.133510.11	MLLT10	XLOC_141579	4561 coding	coding	noncoding	coding
MSTRG.133651.1	DNAJC1	XLOC_141589	231 coding	noncoding	noncoding	noncoding
MSTRG.133487.1		XLOC_141604	310 coding	noncoding	noncoding	noncoding
MSTRG.133496.1		XLOC_141616	244 coding	noncoding	noncoding	noncoding
MSTRG.133575.7	PIP4K2A	XLOC_141631	1435 coding	coding	coding	coding
MSTRG.133567.1		XLOC_141638	284 coding	noncoding	noncoding	noncoding
MSTRG.133584.1	ARMC3	XLOC_141641	853 coding	noncoding	noncoding	noncoding
MSTRG.133614.1	C10orf67	XLOC_141653	322 coding	noncoding	noncoding	noncoding
MSTRG.133682.1	KIAA1217	XLOC_141675	253 coding	noncoding	noncoding	noncoding
MSTRG.133684.1	KIAA1217	XLOC_141676	272 coding	noncoding	noncoding	noncoding
MSTRG.133687.1	KIAA1217	XLOC_141677	280 coding	noncoding	noncoding	noncoding

MSTRG.133707.1	KIAA1217	XLOC_141687	279 coding	noncoding	noncoding	noncoding
MSTRG.133712.1	ARHGAP21	XLOC_141691	261 coding	noncoding	noncoding	noncoding
NM_145010.4	ENKUR	XLOC_141716	3378 coding	coding	coding	coding
MSTRG.133716.1		XLOC_141724	451 coding	noncoding	noncoding	noncoding
MSTRG.133719.1		XLOC_141726	399 coding	noncoding	noncoding	noncoding
MSTRG.133732.1	GPR158	XLOC_141730	304 coding	noncoding	noncoding	noncoding
MSTRG.133734.1	GPR158	XLOC_141731	391 coding	coding	noncoding	noncoding
MSTRG.133735.1	GPR158	XLOC_141732	283 coding	noncoding	noncoding	noncoding
MSTRG.133723.1		XLOC_141739	259 coding	noncoding	noncoding	noncoding
MSTRG.133783.1	MYO3A	XLOC_141744	236 coding	noncoding	noncoding	noncoding
MSTRG.133785.1	MYO3A	XLOC_141745	219 coding	noncoding	noncoding	noncoding
NM_001178125.1	ABI1	XLOC_141765	3188 coding	coding	coding	coding
NM_001348034.1	ABI1	XLOC_141765	3548 coding	coding	coding	coding
MSTRG.133821.1		XLOC_141777	303 coding	noncoding	noncoding	noncoding
XM_017015930.1	ANKRD26	XLOC_141779	8813 coding	coding	coding	coding
MSTRG.133858.1	ANKRD26	XLOC_141780	460 coding	noncoding	noncoding	noncoding
MSTRG.133900.1		XLOC_141819	309 coding	noncoding	noncoding	noncoding
MSTRG.133910.1	MKX	XLOC_141823	297 coding	noncoding	noncoding	noncoding
MSTRG.133907.1		XLOC_141827	251 coding	noncoding	noncoding	noncoding
MSTRG.133960.1	ARMC4	XLOC_141829	283 coding	noncoding	noncoding	noncoding
MSTRG.134048.1	MPP7	XLOC_141842	251 coding	noncoding	noncoding	noncoding
MSTRG.133966.1		XLOC_141865	249 coding	noncoding	noncoding	noncoding
MSTRG.134077.12	WAC	XLOC_141870	3777 coding	coding	coding	noncoding
MSTRG.133983.1		XLOC_141875	310 coding	noncoding	noncoding	noncoding
MSTRG.133989.1		XLOC_141876	259 coding	noncoding	noncoding	noncoding
MSTRG.133993.1	LINC01517	XLOC_141877	320 coding	noncoding	noncoding	noncoding
MSTRG.133996.1	LINC01517	XLOC_141879	306 coding	noncoding	noncoding	noncoding
MSTRG.134084.1		XLOC_141943	261 coding	noncoding	noncoding	noncoding
MSTRG.134101.1		XLOC_141953	250 coding	noncoding	noncoding	noncoding
MSTRG.134132.1		XLOC_141969	240 coding	noncoding	noncoding	noncoding
MSTRG.134265.1	ZNF438	XLOC_141999	395 coding	noncoding	noncoding	noncoding
MSTRG.134297.1	LOC105376481	XLOC_142017	224 coding	noncoding	noncoding	noncoding
MSTRG.134299.1	LOC105376481	XLOC_142019	277 coding	noncoding	noncoding	noncoding
MSTRG.134330.22	ZEB1	XLOC_142023	4179 coding	coding	noncoding	noncoding
XM_017016224.2	KIF5B	XLOC_142059	5255 coding	coding	coding	coding
MSTRG.134321.1		XLOC_142071	541 coding	noncoding	noncoding	noncoding
MSTRG.134327.1		XLOC_142076	231 coding	noncoding	noncoding	noncoding
MSTRG.134328.1		XLOC_142077	300 coding	noncoding	noncoding	noncoding
MSTRG.134747.1	CCDC7	XLOC_142103	496 coding	noncoding	noncoding	noncoding
MSTRG.134773.1	CCDC7	XLOC_142128	269 coding	noncoding	noncoding	noncoding
MSTRG.134433.1	NRP1	XLOC_142165	271 coding	noncoding	noncoding	noncoding
MSTRG.134437.1		XLOC_142179	298 coding	noncoding	noncoding	noncoding
MSTRG.134449.1		XLOC_142198	230 coding	noncoding	noncoding	noncoding

MSTRG.134492.1	CUL2	XLOC_142203	243	coding	noncoding	noncoding	noncoding
MSTRG.134575.1	CCNY	XLOC_142226	309	coding	noncoding	noncoding	noncoding
MSTRG.134524.1		XLOC_142233	232	coding	noncoding	noncoding	noncoding
MSTRG.134523.1		XLOC_142237	317	coding	noncoding	noncoding	noncoding
MSTRG.134538.1	LINC02630	XLOC_142242	296	coding	noncoding	noncoding	noncoding
MSTRG.134552.1		XLOC_142248	259	coding	noncoding	noncoding	noncoding
MSTRG.134557.1		XLOC_142251	300	coding	noncoding	noncoding	noncoding
MSTRG.134559.1		XLOC_142253	217	coding	noncoding	noncoding	noncoding
MSTRG.134560.1		XLOC_142254	233	coding	noncoding	noncoding	noncoding
MSTRG.134657.1	ANKRD30A	XLOC_142262	261	coding	noncoding	noncoding	noncoding
MSTRG.134641.1		XLOC_142269	274	coding	noncoding	noncoding	noncoding
MSTRG.134662.1		XLOC_142280	273	coding	noncoding	noncoding	noncoding
MSTRG.134969.1	ZNF33B	XLOC_142350	226	coding	noncoding	noncoding	noncoding
MSTRG.134973.1	ZNF33B	XLOC_142353	210	coding	noncoding	noncoding	noncoding
MSTRG.134953.1		XLOC_142356	467	coding	noncoding	noncoding	noncoding
MSTRG.134955.1		XLOC_142359	223	coding	noncoding	noncoding	noncoding
MSTRG.134962.1	LOC105378269	XLOC_142362	312	coding	noncoding	noncoding	noncoding
MSTRG.134975.1		XLOC_142365	501	coding	noncoding	noncoding	noncoding
MSTRG.134994.7	CSGALNACT2	XLOC_142374	3095	coding	coding	coding	noncoding
MSTRG.135005.1		XLOC_142384	285	coding	noncoding	noncoding	noncoding
MSTRG.135026.1		XLOC_142389	312	coding	noncoding	noncoding	noncoding
MSTRG.135031.1		XLOC_142395	301	coding	noncoding	noncoding	noncoding
NM_001324166.1	ZNF32	XLOC_142411	1167	coding	coding	coding	coding
MSTRG.135100.1		XLOC_142432	290	coding	noncoding	noncoding	noncoding
XR_001747171.1	CXCL12	XLOC_142435	5604	coding	coding	coding	noncoding
NM_001282866.2	8-Mar	XLOC_142464	5615	coding	coding	coding	coding
MSTRG.135481.9	PARGP1-AGAP4	XLOC_142480	540	coding	noncoding	coding	noncoding
MSTRG.135530.6	LOC107984026	XLOC_142490	855	coding	coding	noncoding	noncoding
MSTRG.135215.1		XLOC_142519	246	coding	noncoding	noncoding	noncoding
MSTRG.135216.1		XLOC_142520	276	coding	coding	noncoding	noncoding
MSTRG.135305.1	ARHGAP22	XLOC_142551	272	coding	noncoding	noncoding	noncoding
XM_011539829.2	LRRC18	XLOC_142557	2932	coding	coding	coding	coding
MSTRG.135332.1		XLOC_142564	390	coding	noncoding	noncoding	noncoding
XM_017015828.1	VSTM4	XLOC_142566	6781	coding	noncoding	coding	coding
MSTRG.135340.1		XLOC_142578	314	coding	coding	noncoding	noncoding
MSTRG.135385.1		XLOC_142580	238	coding	noncoding	noncoding	noncoding
MSTRG.135379.1		XLOC_142596	626	coding	noncoding	noncoding	noncoding
MSTRG.135380.1		XLOC_142597	295	coding	noncoding	noncoding	noncoding
MSTRG.135577.1		XLOC_142678	635	coding	coding	coding	noncoding
MSTRG.135579.1		XLOC_142679	263	coding	coding	noncoding	noncoding
MSTRG.135668.1	PRKG1	XLOC_142697	289	coding	noncoding	noncoding	noncoding
MSTRG.135670.1	PRKG1	XLOC_142698	248	coding	noncoding	noncoding	noncoding
MSTRG.135677.1	PRKG1	XLOC_142701	323	coding	noncoding	noncoding	noncoding

MSTRG.135697.1	PRKG1	XLOC_142710	291 coding	noncoding	noncoding	noncoding
MSTRG.135612.1		XLOC_142738	268 coding	noncoding	noncoding	noncoding
MSTRG.135617.1	LOC105378307	XLOC_142747	323 coding	noncoding	noncoding	noncoding
MSTRG.135621.1	LOC105378306	XLOC_142749	237 coding	noncoding	noncoding	noncoding
MSTRG.135625.1		XLOC_142753	219 coding	noncoding	noncoding	noncoding
XR_945967.2	LOC105378308	XLOC_142755	8270 coding	coding	coding	coding
MSTRG.135640.1	LOC105378308	XLOC_142756	278 coding	noncoding	noncoding	noncoding
XR_001747192.2	PCDH15	XLOC_142765	15323 coding	coding	coding	coding
MSTRG.135818.1	PCDH15	XLOC_142770	253 coding	noncoding	noncoding	noncoding
MSTRG.135823.1	PCDH15	XLOC_142774	218 coding	noncoding	noncoding	noncoding
MSTRG.135832.1	PCDH15	XLOC_142780	228 coding	noncoding	noncoding	noncoding
MSTRG.135834.1	PCDH15	XLOC_142782	247 coding	noncoding	noncoding	noncoding
MSTRG.135857.1	PCDH15	XLOC_142791	274 coding	noncoding	noncoding	noncoding
MSTRG.135746.1		XLOC_142806	321 coding	noncoding	noncoding	noncoding
MSTRG.135897.1		XLOC_142845	283 coding	noncoding	noncoding	noncoding
MSTRG.135944.3	TFAM	XLOC_142867	4793 coding	coding	noncoding	noncoding
NR_131782.1	CCEPR	XLOC_142889	2502 coding	noncoding	noncoding	noncoding
MSTRG.136006.1	PHYHIPL	XLOC_142890	239 coding	noncoding	noncoding	noncoding
MSTRG.135983.1		XLOC_142896	229 coding	noncoding	noncoding	noncoding
MSTRG.135990.1	LOC107984235	XLOC_142899	316 coding	coding	noncoding	noncoding
MSTRG.135997.1	MRLN	XLOC_142904	432 coding	noncoding	noncoding	noncoding
MSTRG.136021.1		XLOC_142920	259 coding	noncoding	noncoding	noncoding
MSTRG.136108.1		XLOC_143002	248 coding	noncoding	noncoding	noncoding
NR_134508.1	TMEM26	XLOC_143007	2803 coding	coding	coding	coding
MSTRG.136146.1	CABCOCO1	XLOC_143019	566 coding	noncoding	noncoding	noncoding
MSTRG.136167.1		XLOC_143033	306 coding	noncoding	noncoding	noncoding
MSTRG.136170.1		XLOC_143046	285 coding	noncoding	noncoding	noncoding
MSTRG.136249.1		XLOC_143064	246 coding	noncoding	noncoding	noncoding
MSTRG.136263.1		XLOC_143072	218 coding	noncoding	noncoding	noncoding
XM_017015900.1	JMJD1C	XLOC_143078	8771 coding	coding	coding	coding
MSTRG.136635.20	JMJD1C	XLOC_143078	4266 coding	noncoding	coding	coding
MSTRG.136297.1	LOC107984238	XLOC_143086	412 coding	noncoding	noncoding	noncoding
MSTRG.136299.1		XLOC_143087	262 coding	noncoding	noncoding	noncoding
MSTRG.136308.1		XLOC_143094	278 coding	noncoding	noncoding	noncoding
MSTRG.136313.1		XLOC_143096	225 coding	noncoding	noncoding	noncoding
MSTRG.136329.1		XLOC_143104	233 coding	noncoding	noncoding	noncoding
MSTRG.136330.1		XLOC_143105	209 coding	noncoding	noncoding	noncoding
NR_134456.1	LINC02671	XLOC_143111	2572 coding	noncoding	noncoding	noncoding
MSTRG.136351.1		XLOC_143116	266 coding	noncoding	noncoding	noncoding
MSTRG.136381.1	LOC105378338	XLOC_143136	266 coding	noncoding	noncoding	noncoding
MSTRG.136385.1	LOC105378339	XLOC_143138	256 coding	noncoding	noncoding	noncoding
XM_017016152.1	CTNNA3	XLOC_143139	10984 coding	coding	coding	coding
MSTRG.136485.1	LOC105378340	XLOC_143141	315 coding	noncoding	noncoding	noncoding

MSTRG.136488.1	CTNNA3	XLOC_143144	236 coding	coding	noncoding	noncoding
MSTRG.136489.1	CTNNA3	XLOC_143145	264 coding	noncoding	coding	noncoding
MSTRG.136532.1	CTNNA3	XLOC_143161	294 coding	noncoding	noncoding	noncoding
MSTRG.136537.1	CTNNA3	XLOC_143164	273 coding	noncoding	noncoding	noncoding
MSTRG.136544.1	CTNNA3	XLOC_143167	526 coding	coding	noncoding	noncoding
MSTRG.136583.1	HERC4	XLOC_143191	252 coding	noncoding	noncoding	noncoding
MSTRG.136585.1	HERC4	XLOC_143193	221 coding	noncoding	noncoding	noncoding
MSTRG.136554.1		XLOC_143202	273 coding	noncoding	noncoding	noncoding
XR_246097.2	RUFY2	XLOC_143203	4546 coding	coding	coding	coding
MSTRG.136602.1	DNA2	XLOC_143205	292 coding	noncoding	noncoding	noncoding
MSTRG.136603.1		XLOC_143206	279 coding	noncoding	noncoding	noncoding
MSTRG.136612.1	TET1	XLOC_143210	285 coding	noncoding	noncoding	noncoding
MSTRG.136661.1	STOX1	XLOC_143226	235 coding	noncoding	noncoding	noncoding
MSTRG.136664.1	STOX1	XLOC_143229	229 coding	noncoding	noncoding	noncoding
MSTRG.136738.1		XLOC_143272	251 coding	noncoding	noncoding	noncoding
MSTRG.136840.1	PALD1	XLOC_143308	306 coding	noncoding	noncoding	noncoding
MSTRG.136863.1	ADAMTS14	XLOC_143323	273 coding	noncoding	noncoding	noncoding
MSTRG.136879.1		XLOC_143335	405 coding	noncoding	noncoding	noncoding
MSTRG.136906.1	UNC5B	XLOC_143343	392 coding	noncoding	noncoding	noncoding
MSTRG.136907.1	UNC5B	XLOC_143344	235 coding	noncoding	noncoding	noncoding
MSTRG.136932.1		XLOC_143391	263 coding	coding	noncoding	noncoding
MSTRG.136937.1		XLOC_143394	404 coding	noncoding	noncoding	noncoding
MSTRG.136999.41	ASCC1	XLOC_143397	1496 coding	noncoding	noncoding	noncoding
MSTRG.137001.1		XLOC_143398	283 coding	noncoding	noncoding	noncoding
MSTRG.136939.1		XLOC_143399	259 coding	noncoding	noncoding	noncoding
MSTRG.136989.1	DNAJB12	XLOC_143407	247 coding	noncoding	noncoding	noncoding
MSTRG.137005.1	MICU1	XLOC_143410	352 coding	noncoding	noncoding	noncoding
MSTRG.137007.1	MICU1	XLOC_143412	283 coding	noncoding	noncoding	noncoding
MSTRG.137015.1	MICU1	XLOC_143420	255 coding	noncoding	noncoding	noncoding
MSTRG.137017.1	MICU1	XLOC_143422	290 coding	noncoding	noncoding	noncoding
MSTRG.136992.1		XLOC_143424	291 coding	noncoding	noncoding	noncoding
MSTRG.137110.1	P4HA1	XLOC_143439	273 coding	noncoding	noncoding	noncoding
MSTRG.137118.1	ECD	XLOC_143447	733 coding	noncoding	noncoding	noncoding
MSTRG.137129.1	DNAJC9-AS1	XLOC_143456	226 coding	noncoding	noncoding	noncoding
MSTRG.137130.1	DNAJC9-AS1	XLOC_143457	276 coding	noncoding	noncoding	noncoding
MSTRG.137138.1	CFAP70	XLOC_143460	206 coding	noncoding	noncoding	noncoding
XM_017016162.2	ANXA7	XLOC_143463	4475 coding	coding	coding	coding
NM_001142354.2	PPP3CB	XLOC_143469	3497 coding	coding	coding	coding
NM_021132.4	PPP3CB	XLOC_143469	3519 coding	coding	coding	coding
XM_024447832.1	USP54	XLOC_143469	8730 coding	coding	coding	coding
MSTRG.137085.1	PPP3CB	XLOC_143476	306 coding	noncoding	noncoding	noncoding
MSTRG.137097.1	USP54	XLOC_143483	302 coding	noncoding	noncoding	noncoding
MSTRG.137098.1	USP54	XLOC_143484	274 coding	noncoding	noncoding	noncoding

MSTRG.137063.1	LOC105378360	XLOC_143488	280 coding	noncoding	noncoding	noncoding
MSTRG.137177.1		XLOC_143498	203 coding	noncoding	noncoding	noncoding
XM_011540310.3	NDST2	XLOC_143499	4558 coding	coding	coding	coding
MSTRG.137149.1	PLAU	XLOC_143505	256 coding	noncoding	noncoding	noncoding
MSTRG.137254.1	ADK	XLOC_143514	303 coding	noncoding	noncoding	noncoding
MSTRG.137229.1	SAMD8	XLOC_143551	232 coding	noncoding	noncoding	noncoding
MSTRG.137231.2	SAMD8	XLOC_143553	2704 coding	coding	noncoding	noncoding
MSTRG.137231.7	SAMD8	XLOC_143553	2317 coding	coding	noncoding	noncoding
MSTRG.137188.1	ZNF503-AS1	XLOC_143562	299 coding	noncoding	noncoding	noncoding
MSTRG.137316.1		XLOC_143577	293 coding	noncoding	noncoding	noncoding
XM_017016220.2	KCNMA1	XLOC_143583	12705 coding	noncoding	coding	coding
NM_001271520.2	KCNMA1	XLOC_143583	1352 coding	noncoding	coding	coding
MSTRG.137385.1	KCNMA1	XLOC_143603	407 coding	coding	noncoding	noncoding
MSTRG.137330.1		XLOC_143609	282 coding	noncoding	noncoding	noncoding
MSTRG.137487.1		XLOC_143682	433 coding	noncoding	noncoding	noncoding
MSTRG.137492.1		XLOC_143683	261 coding	noncoding	noncoding	noncoding
MSTRG.137502.1	BMS1P21	XLOC_143701	257 coding	noncoding	noncoding	noncoding
NM_003019.5	SFTPD	XLOC_143703	1283 coding	coding	coding	coding
NR_027428.1	TMEM254-AS1	XLOC_143704	4046 coding	noncoding	coding	noncoding
MSTRG.137601.1	ANXA11	XLOC_143707	263 coding	noncoding	noncoding	noncoding
MSTRG.137579.1		XLOC_143717	267 coding	noncoding	noncoding	noncoding
MSTRG.137584.1		XLOC_143720	271 coding	noncoding	noncoding	noncoding
XM_011539334.2	DYDC1	XLOC_143722	3275 coding	coding	coding	coding
MSTRG.137619.1	SH2D4B	XLOC_143736	310 coding	noncoding	noncoding	noncoding
MSTRG.137622.1		XLOC_143738	263 coding	noncoding	noncoding	noncoding
MSTRG.137647.1		XLOC_143747	227 coding	noncoding	noncoding	noncoding
MSTRG.137720.1	NRG3	XLOC_143757	216 coding	noncoding	noncoding	noncoding
MSTRG.137699.1		XLOC_143793	310 coding	noncoding	noncoding	noncoding
MSTRG.137701.1		XLOC_143795	258 coding	noncoding	noncoding	noncoding
MSTRG.137706.1		XLOC_143798	382 coding	noncoding	noncoding	noncoding
MSTRG.137762.1	LOC105378399	XLOC_143806	285 coding	noncoding	noncoding	noncoding
MSTRG.137782.1		XLOC_143823	340 coding	noncoding	noncoding	noncoding
MSTRG.137795.1		XLOC_143831	220 coding	noncoding	noncoding	noncoding
MSTRG.137851.1	GRID1	XLOC_143840	268 coding	noncoding	noncoding	noncoding
MSTRG.137874.1	GRID1	XLOC_143846	225 coding	noncoding	noncoding	noncoding
MSTRG.137839.1		XLOC_143853	251 coding	noncoding	noncoding	noncoding
MSTRG.137886.1		XLOC_143870	280 coding	noncoding	noncoding	noncoding
MSTRG.137919.1	SNCG	XLOC_143879	308 coding	noncoding	noncoding	noncoding
MSTRG.137913.1		XLOC_143880	254 coding	noncoding	noncoding	noncoding
MSTRG.137949.5	MINPP1	XLOC_143904	889 coding	noncoding	noncoding	noncoding
MSTRG.137954.1	PAPSS2	XLOC_143908	243 coding	noncoding	noncoding	noncoding
XR_946175.2	LOC105378415	XLOC_143936	15139 coding	noncoding	coding	noncoding
MSTRG.138134.1		XLOC_143990	294 coding	noncoding	noncoding	noncoding

MSTRG.138363.15		XLOC_144004	4973	coding	noncoding	noncoding	noncoding
MSTRG.138345.1	SLC16A12	XLOC_144038	263	coding	noncoding	noncoding	noncoding
NM_148978.3	PANK1	XLOC_144041	6225	coding	coding	coding	coding
MSTRG.138466.1		XLOC_144111	247	coding	noncoding	noncoding	noncoding
MSTRG.138478.1	LINC02653	XLOC_144116	311	coding	noncoding	noncoding	noncoding
XM_024447973.1	HTR7	XLOC_144130	4711	coding	coding	coding	coding
MSTRG.138576.1	HECTD2-AS1	XLOC_144151	283	coding	noncoding	noncoding	noncoding
MSTRG.138578.1	HECTD2-AS1	XLOC_144152	292	coding	noncoding	noncoding	noncoding
MSTRG.138563.1		XLOC_144161	294	coding	coding	noncoding	noncoding
MSTRG.138564.1		XLOC_144163	236	coding	noncoding	noncoding	noncoding
MSTRG.138572.1		XLOC_144166	316	coding	noncoding	noncoding	noncoding
MSTRG.138675.1		XLOC_144178	269	coding	noncoding	noncoding	noncoding
MSTRG.138679.1		XLOC_144180	249	coding	noncoding	noncoding	noncoding
MSTRG.138682.1		XLOC_144182	229	coding	noncoding	noncoding	noncoding
MSTRG.138715.1		XLOC_144197	314	coding	noncoding	noncoding	noncoding
MSTRG.138732.1		XLOC_144205	306	coding	noncoding	noncoding	noncoding
MSTRG.138733.1		XLOC_144206	238	coding	noncoding	noncoding	noncoding
MSTRG.138735.1		XLOC_144207	296	coding	noncoding	noncoding	noncoding
MSTRG.138734.1		XLOC_144208	302	coding	noncoding	noncoding	noncoding
MSTRG.138737.1		XLOC_144209	237	coding	noncoding	noncoding	noncoding
MSTRG.138741.1		XLOC_144210	230	coding	noncoding	noncoding	noncoding
MSTRG.138744.1		XLOC_144215	251	coding	noncoding	noncoding	noncoding
MSTRG.138872.1	PLCE1	XLOC_144254	273	coding	noncoding	noncoding	noncoding
MSTRG.138817.1	TBC1D12	XLOC_144264	318	coding	noncoding	noncoding	noncoding
MSTRG.138825.1	TBC1D12	XLOC_144270	239	coding	noncoding	noncoding	noncoding
MSTRG.138830.1	CYP2C18	XLOC_144281	297	coding	coding	noncoding	noncoding
MSTRG.138839.1	CYP2C19	XLOC_144285	315	coding	coding	noncoding	noncoding
MSTRG.138840.1	CYP2C19	XLOC_144286	243	coding	noncoding	noncoding	noncoding
MSTRG.138903.1		XLOC_144288	286	coding	noncoding	noncoding	noncoding
MSTRG.138911.1		XLOC_144293	302	coding	coding	noncoding	noncoding
MSTRG.138879.1		XLOC_144294	278	coding	coding	noncoding	noncoding
MSTRG.138909.1		XLOC_144295	226	coding	coding	noncoding	noncoding
MSTRG.138914.1		XLOC_144308	209	coding	noncoding	noncoding	noncoding
XM_011540330.1	PDLIM1	XLOC_144309	1285	coding	coding	coding	coding
MSTRG.138920.1	PDLIM1	XLOC_144310	268	coding	noncoding	noncoding	noncoding
MSTRG.138949.1		XLOC_144321	243	coding	noncoding	noncoding	noncoding
MSTRG.139129.11	ENTPD1-AS1	XLOC_144327	7855	coding	coding	coding	coding
MSTRG.139184.1	ENTPD1-AS1	XLOC_144335	270	coding	noncoding	noncoding	noncoding
MSTRG.138978.1		XLOC_144368	422	coding	noncoding	noncoding	noncoding
MSTRG.138979.1		XLOC_144369	245	coding	noncoding	noncoding	noncoding
MSTRG.139004.1	ZNF518A	XLOC_144378	224	coding	noncoding	noncoding	noncoding
NM_013314.3	BLNK	XLOC_144379	1823	coding	coding	coding	coding
NM_012465.4	TLL2	XLOC_144395	6769	coding	coding	coding	coding

XM_011539249.1	PIK3AP1	XLOC_144402	4647 coding	coding	coding	coding
MSTRG.139044.1	PIK3AP1	XLOC_144409	208 coding	noncoding	noncoding	noncoding
MSTRG.139025.1		XLOC_144416	304 coding	noncoding	noncoding	noncoding
MSTRG.139115.1	LOC105378447	XLOC_144425	239 coding	noncoding	noncoding	noncoding
MSTRG.139120.1	ARHGAP19-SLIT1	XLOC_144427	263 coding	noncoding	noncoding	noncoding
MSTRG.139058.1		XLOC_144455	204 coding	noncoding	noncoding	noncoding
MSTRG.139063.1	MARVELD1	XLOC_144459	472 coding	noncoding	noncoding	noncoding
MSTRG.139264.4	ZFYVE27	XLOC_144461	396 coding	noncoding	noncoding	noncoding
MSTRG.139373.1	HPSE2	XLOC_144505	256 coding	noncoding	noncoding	noncoding
MSTRG.139391.1	HPSE2	XLOC_144513	277 coding	noncoding	noncoding	noncoding
MSTRG.139393.1	HPSE2	XLOC_144515	251 coding	noncoding	noncoding	noncoding
MSTRG.139359.1		XLOC_144522	293 coding	noncoding	noncoding	noncoding
MSTRG.139410.1	LOC101927300	XLOC_144532	270 coding	noncoding	noncoding	noncoding
MSTRG.139413.1		XLOC_144535	205 coding	noncoding	noncoding	noncoding
MSTRG.139439.1	ABCC2	XLOC_144546	226 coding	coding	noncoding	noncoding
MSTRG.139432.1		XLOC_144552	254 coding	noncoding	noncoding	noncoding
NM_015490.4	SEC31B	XLOC_144588	4634 coding	noncoding	coding	coding
MSTRG.139539.2	HIF1AN	XLOC_144590	5909 coding	coding	coding	noncoding
MSTRG.139544.1		XLOC_144598	278 coding	noncoding	noncoding	noncoding
MSTRG.139576.1	TLX1NB	XLOC_144615	289 coding	noncoding	noncoding	noncoding
MSTRG.139571.1	LINC01514	XLOC_144619	323 coding	noncoding	noncoding	noncoding
MSTRG.139595.9	BTRC	XLOC_144623	1396 coding	coding	noncoding	noncoding
MSTRG.139624.1	FBXW4	XLOC_144627	293 coding	noncoding	noncoding	noncoding
MSTRG.139695.1	ARMH3	XLOC_144653	340 coding	noncoding	noncoding	noncoding
MSTRG.139658.1	PPRC1	XLOC_144666	266 coding	noncoding	noncoding	noncoding
MSTRG.139643.1		XLOC_144670	290 coding	noncoding	noncoding	noncoding
MSTRG.139702.5	LOC107984263	XLOC_144673	3436 coding	coding	coding	coding
MSTRG.139667.1	PSD	XLOC_144677	293 coding	noncoding	noncoding	noncoding
MSTRG.139740.1	SUFU	XLOC_144694	258 coding	noncoding	noncoding	noncoding
MSTRG.139752.1	SUFU	XLOC_144704	277 coding	noncoding	noncoding	noncoding
MSTRG.139718.4	LOC105378460	XLOC_144715	3208 coding	noncoding	coding	coding
MSTRG.139716.1		XLOC_144717	275 coding	noncoding	noncoding	noncoding
MSTRG.139767.1		XLOC_144730	322 coding	noncoding	noncoding	noncoding
MSTRG.139790.1		XLOC_144747	852 coding	noncoding	noncoding	noncoding
MSTRG.139792.1		XLOC_144749	265 coding	noncoding	noncoding	noncoding
MSTRG.139795.1	INA	XLOC_144750	231 coding	noncoding	noncoding	noncoding
NM_001011663.2	PCGF6	XLOC_144751	2236 coding	coding	coding	coding
MSTRG.139799.1	PCGF6	XLOC_144752	336 coding	noncoding	noncoding	noncoding
NM_001129742.2	CALHM3	XLOC_144759	1652 coding	coding	coding	coding
MSTRG.139806.1	NEURL1	XLOC_144762	265 coding	noncoding	noncoding	noncoding
MSTRG.139849.1	SH3PXD2A	XLOC_144779	276 coding	noncoding	noncoding	noncoding
MSTRG.139852.1	SH3PXD2A	XLOC_144780	282 coding	noncoding	noncoding	noncoding
MSTRG.139860.1	SH3PXD2A	XLOC_144784	271 coding	noncoding	noncoding	noncoding

MSTRG.139930.1		XLOC_144866	308 coding	coding	noncoding	noncoding
MSTRG.140084.1		XLOC_144920	266 coding	noncoding	noncoding	noncoding
MSTRG.140083.1		XLOC_144922	320 coding	coding	noncoding	noncoding
NM_001206572.2	SORCS1	XLOC_144923	7605 coding	coding	coding	coding
MSTRG.140119.1	SORCS1	XLOC_144931	259 coding	noncoding	noncoding	noncoding
MSTRG.140134.1	LINC01435	XLOC_144988	231 coding	noncoding	noncoding	noncoding
MSTRG.140156.1		XLOC_145004	299 coding	noncoding	noncoding	noncoding
MSTRG.140172.1		XLOC_145011	269 coding	noncoding	noncoding	noncoding
MSTRG.140239.1		XLOC_145019	388 coding	noncoding	noncoding	noncoding
MSTRG.140241.1		XLOC_145021	242 coding	noncoding	noncoding	noncoding
MSTRG.140363.1		XLOC_145073	230 coding	noncoding	noncoding	noncoding
MSTRG.140374.1		XLOC_145081	257 coding	noncoding	noncoding	noncoding
MSTRG.140389.1		XLOC_145088	273 coding	noncoding	noncoding	noncoding
MSTRG.140398.1		XLOC_145095	274 coding	coding	noncoding	noncoding
MSTRG.140553.3	NHLRC2	XLOC_145187	2763 coding	coding	coding	noncoding
MSTRG.140541.1	LOC105378492	XLOC_145192	263 coding	noncoding	noncoding	noncoding
MSTRG.140616.1	AFAP1L2	XLOC_145205	331 coding	noncoding	noncoding	noncoding
MSTRG.140716.1	LOC107984272	XLOC_145216	238 coding	noncoding	noncoding	noncoding
MSTRG.140718.1	LOC107984272	XLOC_145217	317 coding	noncoding	noncoding	noncoding
MSTRG.140777.1	ATRNL1	XLOC_145224	392 coding	noncoding	noncoding	noncoding
MSTRG.140780.1	ATRNL1	XLOC_145226	264 coding	noncoding	noncoding	noncoding
MSTRG.140785.1	ATRNL1	XLOC_145230	384 coding	noncoding	noncoding	noncoding
MSTRG.140788.1	ATRNL1	XLOC_145231	244 coding	noncoding	noncoding	noncoding
MSTRG.140753.1	GFRA1	XLOC_145252	267 coding	noncoding	noncoding	noncoding
MSTRG.140764.1		XLOC_145258	676 coding	coding	noncoding	noncoding
XM_005269673.5	HSPA12A	XLOC_145260	8847 coding	noncoding	coding	coding
MSTRG.140841.1	HSPA12A	XLOC_145262	296 coding	noncoding	noncoding	noncoding
MSTRG.140854.1	SHTN1	XLOC_145270	244 coding	noncoding	noncoding	noncoding
MSTRG.140831.1		XLOC_145274	261 coding	noncoding	noncoding	noncoding
MSTRG.140887.1		XLOC_145317	334 coding	noncoding	noncoding	noncoding
MSTRG.140984.1	CASC2	XLOC_145338	410 coding	noncoding	noncoding	noncoding
MSTRG.140940.1		XLOC_145353	522 coding	noncoding	noncoding	noncoding
NM_004248.3	PRLHR	XLOC_145354	5387 coding	coding	coding	coding
MSTRG.140987.1		XLOC_145367	286 coding	noncoding	noncoding	noncoding
MSTRG.140996.1		XLOC_145370	402 coding	noncoding	noncoding	noncoding
MSTRG.141027.1		XLOC_145381	686 coding	noncoding	noncoding	noncoding
MSTRG.141073.1		XLOC_145429	332 coding	noncoding	noncoding	noncoding
MSTRG.141074.1		XLOC_145430	653 coding	noncoding	noncoding	noncoding
MSTRG.141084.1	LOC105378515	XLOC_145433	289 coding	noncoding	noncoding	noncoding
MSTRG.141092.3	PLPP4	XLOC_145436	2039 coding	coding	noncoding	noncoding
MSTRG.141092.5	PLPP4	XLOC_145436	2332 coding	coding	noncoding	coding
MSTRG.141092.4	PLPP4	XLOC_145436	2460 coding	coding	noncoding	noncoding
MSTRG.141092.2	PLPP4	XLOC_145436	1851 coding	coding	noncoding	noncoding

MSTRG.141092.1	PLPP4	XLOC_145436	2131 coding	coding	noncoding	coding
MSTRG.141094.1	PLPP4	XLOC_145438	225 coding	noncoding	noncoding	noncoding
MSTRG.141209.1	WDR11-AS1	XLOC_145449	200 coding	noncoding	noncoding	noncoding
MSTRG.141131.1	LOC105378521	XLOC_145456	295 coding	noncoding	noncoding	noncoding
MSTRG.141134.1	LOC105378522	XLOC_145458	317 coding	noncoding	noncoding	noncoding
MSTRG.141156.1		XLOC_145467	317 coding	noncoding	noncoding	noncoding
NM_022970.3	FGFR2	XLOC_145468	4643 coding	coding	coding	coding
MSTRG.141178.1	FGFR2	XLOC_145469	278 coding	noncoding	noncoding	noncoding
MSTRG.141184.1	FGFR2	XLOC_145471	415 coding	noncoding	noncoding	noncoding
MSTRG.141188.1	FGFR2	XLOC_145473	268 coding	noncoding	noncoding	noncoding
MSTRG.141160.1	LOC107984183	XLOC_145476	390 coding	noncoding	noncoding	noncoding
MSTRG.141168.1		XLOC_145481	236 coding	noncoding	noncoding	noncoding
MSTRG.141258.1	ATE1	XLOC_145489	396 coding	noncoding	noncoding	noncoding
MSTRG.141264.1	ATE1-AS1	XLOC_145493	278 coding	noncoding	noncoding	noncoding
MSTRG.141233.1	TACC2	XLOC_145498	208 coding	noncoding	noncoding	noncoding
MSTRG.141271.4	PLEKHA1	XLOC_145506	2890 coding	coding	coding	noncoding
MSTRG.141248.1	HTRA1	XLOC_145512	336 coding	noncoding	noncoding	noncoding
MSTRG.141289.1		XLOC_145521	319 coding	noncoding	noncoding	noncoding
NR_037915.1	FAM24B-CUZD1	XLOC_145525	2623 coding	coding	coding	coding
NR_073476.1	IKZF5	XLOC_145541	4584 coding	noncoding	coding	coding
XR_001747625.1	LOC107984128	XLOC_145657	1963 coding	noncoding	noncoding	noncoding
MSTRG.141372.1		XLOC_145675	253 coding	noncoding	noncoding	noncoding
NM_001322966.2	OAT	XLOC_145676	2354 coding	coding	coding	coding
NM_001146340.3	NKX1-2	XLOC_145677	3211 coding	coding	coding	coding
MSTRG.141536.1	FAM53B	XLOC_145685	499 coding	noncoding	noncoding	noncoding
MSTRG.141526.1		XLOC_145720	256 coding	noncoding	noncoding	noncoding
MSTRG.141628.1	CTBP2	XLOC_145721	8919 coding	coding	coding	coding
MSTRG.141576.1		XLOC_145723	266 coding	noncoding	noncoding	noncoding
MSTRG.141577.1		XLOC_145724	234 coding	noncoding	noncoding	noncoding
MSTRG.141588.1		XLOC_145730	307 coding	noncoding	noncoding	noncoding
MSTRG.141598.1	TEX36	XLOC_145735	455 coding	coding	noncoding	noncoding
MSTRG.141618.4	EDRF1-AS1	XLOC_145739	4373 coding	noncoding	coding	coding
MSTRG.141605.1	UROS	XLOC_145744	226 coding	noncoding	noncoding	noncoding
MSTRG.141639.1	FANK1	XLOC_145750	256 coding	coding	noncoding	noncoding
MSTRG.141641.1	FANK1	XLOC_145752	497 coding	noncoding	noncoding	noncoding
MSTRG.141642.1	FANK1	XLOC_145753	629 coding	coding	noncoding	noncoding
MSTRG.141691.1	ADAM12	XLOC_145761	316 coding	noncoding	noncoding	noncoding
MSTRG.141665.1	C10orf90	XLOC_145774	319 coding	noncoding	noncoding	noncoding
MSTRG.141763.1	DOCK1	XLOC_145790	247 coding	noncoding	noncoding	noncoding
MSTRG.141774.1	DOCK1	XLOC_145797	294 coding	noncoding	noncoding	noncoding
MSTRG.141779.1	DOCK1	XLOC_145800	202 coding	noncoding	noncoding	noncoding
NM_152311.5	CLRN3	XLOC_145807	1146 coding	noncoding	coding	coding
MSTRG.141894.3	PTPRE	XLOC_145809	3056 coding	coding	coding	coding

MSTRG.141845.1		XLOC_145857	354 coding	noncoding	noncoding	noncoding
MSTRG.141846.1		XLOC_145858	305 coding	noncoding	noncoding	noncoding
MSTRG.142170.1	ADGRA1-AS1	XLOC_146026	275 coding	noncoding	noncoding	noncoding
MSTRG.142190.1	KNDC1	XLOC_146033	228 coding	noncoding	noncoding	noncoding
MSTRG.142178.1		XLOC_146036	296 coding	noncoding	noncoding	noncoding
XM_011539673.2	FUOM	XLOC_146048	2818 coding	coding	coding	coding
XM_011520235.3	PSMD13	XLOC_146109	1395 coding	noncoding	coding	coding
XM_017018355.1	PGGHG	XLOC_146113	3646 coding	coding	coding	coding
XM_017017654.1	B4GALNT4	XLOC_146119	4766 coding	coding	coding	coding
MSTRG.142387.1	DEAF1	XLOC_146141	232 coding	noncoding	noncoding	noncoding
MSTRG.142390.1	EPS8L2	XLOC_146143	326 coding	noncoding	noncoding	noncoding
MSTRG.142395.1	TALDO1	XLOC_146146	509 coding	noncoding	noncoding	noncoding
NM_001025237.1	TSPAN4	XLOC_146164	1462 coding	coding	coding	coding
MSTRG.142439.1		XLOC_146174	562 coding	noncoding	noncoding	noncoding
MSTRG.142447.1		XLOC_146178	292 coding	noncoding	noncoding	noncoding
MSTRG.142458.1	LINC02689	XLOC_146181	249 coding	noncoding	noncoding	noncoding
MSTRG.142449.1		XLOC_146183	391 coding	noncoding	noncoding	noncoding
MSTRG.142506.1		XLOC_146217	205 coding	noncoding	noncoding	noncoding
MSTRG.142518.1		XLOC_146220	272 coding	noncoding	noncoding	noncoding
MSTRG.142534.2	TSPAN32	XLOC_146228	7516 coding	coding	coding	coding
MSTRG.142534.7	TSPAN32	XLOC_146228	5440 coding	coding	coding	coding
MSTRG.142624.1	KCNQ1	XLOC_146248	317 coding	noncoding	noncoding	noncoding
MSTRG.142557.6	NAP1L4	XLOC_146289	2368 coding	coding	noncoding	noncoding
MSTRG.142595.1		XLOC_146309	323 coding	noncoding	noncoding	noncoding
XM_006718181.3	PGAP2	XLOC_146325	2451 coding	coding	coding	coding
MSTRG.142784.1	STIM1	XLOC_146343	281 coding	coding	noncoding	noncoding
MSTRG.142720.1		XLOC_146357	297 coding	noncoding	noncoding	noncoding
MSTRG.142741.1		XLOC_146403	229 coding	noncoding	noncoding	noncoding
MSTRG.142742.1		XLOC_146407	229 coding	noncoding	noncoding	noncoding
MSTRG.142758.1		XLOC_146420	243 coding	noncoding	noncoding	noncoding
NM_001004489.2	OR2AG1	XLOC_146567	998 coding	coding	coding	coding
MSTRG.143029.1	OR10A4	XLOC_146575	262 coding	noncoding	noncoding	noncoding
MSTRG.143064.1	ZNF215	XLOC_146579	214 coding	coding	noncoding	noncoding
MSTRG.143066.1	ZNF215	XLOC_146580	222 coding	noncoding	noncoding	noncoding
MSTRG.143284.1	ST5	XLOC_146666	273 coding	noncoding	noncoding	noncoding
MSTRG.143290.1	ST5	XLOC_146669	200 coding	noncoding	noncoding	noncoding
MSTRG.143298.1	ST5	XLOC_146674	229 coding	noncoding	noncoding	noncoding
MSTRG.143252.1	LOC105376542	XLOC_146685	305 coding	noncoding	noncoding	noncoding
MSTRG.143310.2	TMEM41B	XLOC_146691	632 coding	coding	noncoding	noncoding
MSTRG.143347.1	ZNF143	XLOC_146702	226 coding	noncoding	noncoding	noncoding
MSTRG.143348.1	ZNF143	XLOC_146703	265 coding	noncoding	noncoding	noncoding
MSTRG.143352.1	ZNF143	XLOC_146707	292 coding	noncoding	noncoding	noncoding
MSTRG.143354.1		XLOC_146708	260 coding	noncoding	noncoding	noncoding

NM_001143976.1	WEE1	XLOC_146709	3219 coding	coding	coding	coding
MSTRG.143662.1	CAND1.11	XLOC_146730	220 coding	coding	noncoding	noncoding
MSTRG.143441.11	MRVI1-AS1	XLOC_146738	2170 coding	coding	noncoding	noncoding
MSTRG.143370.1		XLOC_146743	242 coding	noncoding	noncoding	noncoding
MSTRG.143429.1		XLOC_146787	280 coding	noncoding	noncoding	noncoding
NM_001080521.3	RASSF10	XLOC_146856	2804 coding	noncoding	coding	coding
XM_017017748.1	ARNTL	XLOC_146863	2727 coding	coding	coding	coding
MSTRG.143666.1		XLOC_146864	292 coding	noncoding	noncoding	noncoding
MSTRG.143677.1		XLOC_146866	267 coding	noncoding	noncoding	noncoding
MSTRG.143715.1		XLOC_146872	243 coding	noncoding	noncoding	noncoding
MSTRG.143720.1	LINC02548	XLOC_146894	233 coding	noncoding	noncoding	noncoding
MSTRG.144081.6	PSMA1	XLOC_146921	1971 coding	coding	coding	noncoding
MSTRG.143854.1		XLOC_146939	234 coding	noncoding	noncoding	noncoding
MSTRG.143960.9	SOX6	XLOC_146994	5290 coding	coding	noncoding	noncoding
MSTRG.143960.13	SOX6	XLOC_146994	1812 coding	noncoding	noncoding	coding
MSTRG.143984.1	SOX6	XLOC_147001	295 coding	noncoding	noncoding	noncoding
MSTRG.143941.1	PLEKHA7	XLOC_147015	502 coding	noncoding	noncoding	noncoding
XM_024448533.1	NUCB2	XLOC_147032	2225 coding	coding	coding	coding
MSTRG.144025.1	NUCB2	XLOC_147035	313 coding	noncoding	noncoding	noncoding
XM_011520072.3	NCR3LG1	XLOC_147037	6791 coding	coding	coding	coding
MSTRG.144029.1		XLOC_147048	474 coding	noncoding	noncoding	noncoding
MSTRG.144047.1		XLOC_147050	291 coding	noncoding	noncoding	noncoding
NR_026563.1	SLC25A51P4	XLOC_147076	1884 coding	coding	coding	coding
MSTRG.144157.1		XLOC_147090	233 coding	noncoding	noncoding	noncoding
MSTRG.144167.1	IGSF22	XLOC_147094	270 coding	noncoding	noncoding	noncoding
MSTRG.144168.1	IGSF22	XLOC_147095	510 coding	noncoding	noncoding	noncoding
MSTRG.144170.1		XLOC_147097	313 coding	noncoding	noncoding	noncoding
MSTRG.144169.1		XLOC_147098	259 coding	noncoding	noncoding	noncoding
MSTRG.144175.1		XLOC_147102	286 coding	noncoding	noncoding	noncoding
MSTRG.144231.1	CSRP3-AS1	XLOC_147115	285 coding	noncoding	noncoding	noncoding
MSTRG.144241.1	CSRP3-AS1	XLOC_147119	306 coding	noncoding	noncoding	noncoding
XM_017018522.1	NAV2	XLOC_147120	11241 coding	coding	coding	coding
NM_001244963.2	NAV2	XLOC_147120	11669 coding	coding	coding	coding
MSTRG.144283.1	NAV2	XLOC_147130	262 coding	noncoding	coding	noncoding
MSTRG.144286.1	NAV2	XLOC_147131	293 coding	noncoding	noncoding	noncoding
MSTRG.144306.1	NAV2	XLOC_147136	248 coding	noncoding	noncoding	noncoding
MSTRG.144247.1		XLOC_147146	317 coding	noncoding	noncoding	noncoding
MSTRG.144242.1		XLOC_147149	318 coding	noncoding	noncoding	noncoding
MSTRG.144255.1		XLOC_147153	221 coding	noncoding	noncoding	noncoding
MSTRG.144420.1	HTATIP2	XLOC_147157	244 coding	noncoding	noncoding	noncoding
MSTRG.144423.1	PRMT3	XLOC_147159	314 coding	noncoding	noncoding	noncoding
MSTRG.144339.1	NELL1	XLOC_147180	228 coding	noncoding	noncoding	noncoding
MSTRG.144354.1	NELL1	XLOC_147186	304 coding	noncoding	noncoding	noncoding

MSTRG.144366.1	NELL1	XLOC_147189	243	coding	noncoding	noncoding	noncoding
MSTRG.144375.1	LOC102723370	XLOC_147197	442	coding	noncoding	noncoding	noncoding
MSTRG.144398.1		XLOC_147212	302	coding	coding	noncoding	noncoding
NM_020346.3	SLC17A6	XLOC_147214	3665	coding	coding	coding	coding
MSTRG.144644.1		XLOC_147237	254	coding	noncoding	noncoding	noncoding
MSTRG.144500.1		XLOC_147251	281	coding	noncoding	noncoding	noncoding
MSTRG.144507.1		XLOC_147256	231	coding	noncoding	noncoding	noncoding
MSTRG.144510.1		XLOC_147260	229	coding	noncoding	noncoding	noncoding
MSTRG.144516.1		XLOC_147261	267	coding	noncoding	noncoding	noncoding
XM_024448468.1	LUZP2	XLOC_147266	5775	coding	noncoding	coding	coding
MSTRG.144545.1	LUZP2	XLOC_147269	311	coding	coding	noncoding	noncoding
MSTRG.144549.1	LUZP2	XLOC_147271	261	coding	noncoding	noncoding	noncoding
MSTRG.144526.1		XLOC_147278	234	coding	noncoding	noncoding	noncoding
MSTRG.144567.1		XLOC_147288	231	coding	noncoding	noncoding	noncoding
MSTRG.144661.1	LINC02699	XLOC_147293	355	coding	noncoding	noncoding	noncoding
MSTRG.144676.1		XLOC_147306	204	coding	noncoding	noncoding	noncoding
MSTRG.144680.1		XLOC_147310	235	coding	noncoding	noncoding	noncoding
MSTRG.144709.1	BBOX1-AS1	XLOC_147312	313	coding	coding	noncoding	noncoding
MSTRG.144711.1	BBOX1-AS1	XLOC_147314	239	coding	noncoding	noncoding	noncoding
MSTRG.144703.1		XLOC_147319	243	coding	noncoding	noncoding	noncoding
MSTRG.144755.1	LOC105376671	XLOC_147336	290	coding	noncoding	noncoding	noncoding
MSTRG.144794.1	BDNF	XLOC_147346	305	coding	noncoding	noncoding	noncoding
MSTRG.144726.1		XLOC_147349	200	coding	noncoding	noncoding	noncoding
MSTRG.144811.1	LINC02758	XLOC_147394	274	coding	coding	noncoding	noncoding
MSTRG.144823.1		XLOC_147400	348	coding	noncoding	noncoding	noncoding
MSTRG.144854.1	LOC105376607	XLOC_147413	370	coding	noncoding	noncoding	noncoding
MSTRG.144879.1	LOC101928316	XLOC_147441	226	coding	noncoding	noncoding	noncoding
MSTRG.145157.1	DCDC1	XLOC_147448	745	coding	noncoding	noncoding	noncoding
MSTRG.145159.1	DCDC1	XLOC_147450	254	coding	noncoding	noncoding	noncoding
MSTRG.145162.1	DCDC1	XLOC_147452	202	coding	noncoding	noncoding	noncoding
MSTRG.145226.1	ELP4	XLOC_147504	260	coding	noncoding	noncoding	noncoding
MSTRG.144965.1	LOC107984322	XLOC_147518	246	coding	noncoding	noncoding	noncoding
MSTRG.144997.1	CCDC73	XLOC_147534	267	coding	coding	coding	noncoding
XR_002957197.1	QSER1	XLOC_147545	5175	coding	noncoding	coding	coding
MSTRG.145076.6	HIPK3	XLOC_147557	7393	coding	coding	coding	coding
MSTRG.145066.1	KIAA1549L	XLOC_147576	241	coding	noncoding	noncoding	noncoding
MSTRG.145109.3	LMO2	XLOC_147587	2061	coding	coding	coding	coding
MSTRG.145123.1	ELF5	XLOC_147652	454	coding	coding	noncoding	coding
MSTRG.145127.1	LINC02707	XLOC_147658	304	coding	noncoding	noncoding	noncoding
MSTRG.145390.1	LOC102723568	XLOC_147664	267	coding	noncoding	noncoding	noncoding
MSTRG.145366.1	TRIM44	XLOC_147730	288	coding	noncoding	noncoding	noncoding
MSTRG.145438.1	LDLRAD3	XLOC_147752	283	coding	noncoding	noncoding	noncoding
MSTRG.145692.1	RAG1	XLOC_147791	276	coding	noncoding	noncoding	noncoding

MSTRG.145450.1		XLOC_147800	309 coding	noncoding	noncoding	noncoding
MSTRG.145468.1		XLOC_147804	269 coding	coding	noncoding	noncoding
MSTRG.145470.1		XLOC_147805	276 coding	noncoding	noncoding	noncoding
MSTRG.145476.1		XLOC_147808	288 coding	noncoding	noncoding	noncoding
MSTRG.145492.1		XLOC_147817	262 coding	noncoding	noncoding	noncoding
MSTRG.145493.1		XLOC_147820	278 coding	noncoding	noncoding	noncoding
MSTRG.145502.1		XLOC_147825	237 coding	noncoding	noncoding	noncoding
MSTRG.145517.1		XLOC_147830	232 coding	noncoding	noncoding	noncoding
MSTRG.145520.1		XLOC_147832	253 coding	coding	noncoding	noncoding
MSTRG.145542.1		XLOC_147843	281 coding	noncoding	noncoding	noncoding
MSTRG.145541.1		XLOC_147845	266 coding	noncoding	noncoding	noncoding
MSTRG.145549.1		XLOC_147847	294 coding	noncoding	noncoding	noncoding
MSTRG.145550.1		XLOC_147848	265 coding	noncoding	noncoding	noncoding
MSTRG.145553.1		XLOC_147849	297 coding	noncoding	noncoding	noncoding
MSTRG.145735.1	LRRC4C	XLOC_147888	282 coding	noncoding	noncoding	noncoding
MSTRG.145736.1	LRRC4C	XLOC_147889	275 coding	noncoding	noncoding	noncoding
MSTRG.145763.1	LRRC4C	XLOC_147902	259 coding	noncoding	noncoding	noncoding
MSTRG.145604.1		XLOC_147907	284 coding	noncoding	noncoding	noncoding
MSTRG.145612.1	LINC02741	XLOC_147911	265 coding	noncoding	noncoding	noncoding
MSTRG.145613.1	LINC02741	XLOC_147912	267 coding	noncoding	noncoding	noncoding
MSTRG.145619.1	LINC01499	XLOC_147915	315 coding	noncoding	noncoding	noncoding
MSTRG.145646.1		XLOC_147928	293 coding	noncoding	noncoding	noncoding
MSTRG.145702.1		XLOC_147936	269 coding	noncoding	noncoding	noncoding
MSTRG.145705.1		XLOC_147937	262 coding	noncoding	noncoding	noncoding
MSTRG.145718.1		XLOC_147940	228 coding	coding	noncoding	noncoding
MSTRG.145719.1		XLOC_147941	579 coding	noncoding	noncoding	noncoding
MSTRG.145778.1		XLOC_147973	251 coding	noncoding	noncoding	noncoding
NM_016142.3	HSD17B12	XLOC_147978	2396 coding	coding	coding	coding
MSTRG.145785.1		XLOC_147997	248 coding	noncoding	noncoding	noncoding
XR_001748004.1	ACCS	XLOC_148010	3516 coding	coding	coding	coding
MSTRG.145903.1	EXT2	XLOC_148018	265 coding	noncoding	noncoding	noncoding
MSTRG.145869.1		XLOC_148022	299 coding	noncoding	noncoding	noncoding
MSTRG.145875.1		XLOC_148024	262 coding	noncoding	noncoding	noncoding
MSTRG.145910.1		XLOC_148033	211 coding	noncoding	noncoding	noncoding
MSTRG.145932.1	LINC02687	XLOC_148051	243 coding	noncoding	noncoding	noncoding
XM_017017173.1	LARGE2	XLOC_148071	2651 coding	coding	coding	coding
MSTRG.146000.1		XLOC_148080	261 coding	noncoding	noncoding	noncoding
MSTRG.146005.1		XLOC_148084	262 coding	noncoding	noncoding	noncoding
MSTRG.146025.1	DGKZ	XLOC_148092	222 coding	noncoding	noncoding	noncoding
NM_001346332.1	ATG13	XLOC_148107	6209 coding	coding	coding	coding
MSTRG.146130.4	ARHGAP1	XLOC_148109	3217 coding	coding	coding	coding
MSTRG.146121.1	LRP4	XLOC_148115	223 coding	coding	noncoding	noncoding
MSTRG.146117.1		XLOC_148116	269 coding	noncoding	noncoding	noncoding

MSTRG.146145.20	C11orf49	XLOC_148117	2678 coding	coding	coding	noncoding
MSTRG.146155.1	MYBPC3	XLOC_148128	399 coding	noncoding	coding	noncoding
MSTRG.146160.3	SPI1	XLOC_148130	5203 coding	coding	coding	coding
MSTRG.146184.1	RAPSN	XLOC_148141	304 coding	noncoding	noncoding	noncoding
MSTRG.146213.1		XLOC_148154	410 coding	noncoding	noncoding	noncoding
XR_930883.2	PTPRJ	XLOC_148155	3975 coding	coding	coding	coding
MSTRG.146342.1	PTPRJ	XLOC_148193	203 coding	noncoding	noncoding	noncoding
MSTRG.146229.1		XLOC_148206	272 coding	coding	noncoding	noncoding
MSTRG.146235.1		XLOC_148209	268 coding	noncoding	noncoding	noncoding
MSTRG.146237.1		XLOC_148210	301 coding	noncoding	noncoding	noncoding
MSTRG.146246.1		XLOC_148216	274 coding	noncoding	noncoding	noncoding
MSTRG.146267.1		XLOC_148222	228 coding	noncoding	noncoding	noncoding
MSTRG.146264.1		XLOC_148224	254 coding	noncoding	noncoding	noncoding
MSTRG.146276.1	LOC107984330	XLOC_148231	466 coding	noncoding	noncoding	noncoding
MSTRG.146279.1	LOC441601	XLOC_148232	332 coding	noncoding	noncoding	noncoding
MSTRG.146284.1	LINC02750	XLOC_148234	321 coding	noncoding	noncoding	noncoding
MSTRG.146285.1	LINC02750	XLOC_148235	274 coding	noncoding	noncoding	noncoding
MSTRG.146287.1		XLOC_148240	236 coding	coding	noncoding	noncoding
MSTRG.146395.1		XLOC_148272	226 coding	noncoding	noncoding	noncoding
MSTRG.146397.1		XLOC_148273	260 coding	coding	noncoding	noncoding
MSTRG.146400.1	OR4P4	XLOC_148278	262 coding	noncoding	noncoding	noncoding
NM_001001921.1	OR5AS1	XLOC_148291	975 coding	coding	coding	coding
MSTRG.146420.1		XLOC_148309	271 coding	noncoding	noncoding	noncoding
XM_011545069.2	P2RX3	XLOC_148332	4441 coding	coding	coding	coding
MSTRG.146461.1	P2RX3	XLOC_148333	337 coding	coding	noncoding	noncoding
MSTRG.146476.1		XLOC_148342	298 coding	noncoding	noncoding	noncoding
MSTRG.146475.1		XLOC_148346	319 coding	noncoding	noncoding	noncoding
NM_015457.3	ZDHHC5	XLOC_148355	4443 coding	coding	coding	coding
NM_015959.4	TMX2	XLOC_148358	1645 coding	coding	coding	coding
MSTRG.146555.1	CTNND1	XLOC_148362	221 coding	noncoding	noncoding	noncoding
MSTRG.146556.1	CTNND1	XLOC_148363	241 coding	noncoding	noncoding	noncoding
MSTRG.146516.1		XLOC_148368	636 coding	coding	noncoding	noncoding
MSTRG.146528.1	OR9Q1	XLOC_148373	215 coding	noncoding	noncoding	noncoding
MSTRG.146691.1	OR5A2	XLOC_148470	304 coding	coding	noncoding	noncoding
MSTRG.146766.1		XLOC_148508	286 coding	noncoding	noncoding	noncoding
NM_001031666.2	MS4A3	XLOC_148513	1309 coding	coding	noncoding	noncoding
MSTRG.146822.6	MS4A6A	XLOC_148519	1444 coding	coding	noncoding	noncoding
MSTRG.146825.1		XLOC_148520	215 coding	noncoding	noncoding	noncoding
NM_024021.4	MS4A4A	XLOC_148524	1668 coding	coding	coding	coding
MSTRG.146793.1	MS4A15	XLOC_148543	307 coding	noncoding	noncoding	noncoding
MSTRG.146801.1		XLOC_148547	280 coding	noncoding	noncoding	noncoding
MSTRG.146851.1		XLOC_148585	317 coding	noncoding	noncoding	noncoding
MSTRG.146908.15	TKFC	XLOC_148596	5166 coding	coding	coding	coding

MSTRG.146895.1		XLOC_148599	269 coding	noncoding	noncoding	noncoding
MSTRG.146941.1		XLOC_148607	271 coding	noncoding	noncoding	noncoding
MSTRG.146942.1		XLOC_148608	428 coding	noncoding	noncoding	noncoding
MSTRG.146943.1		XLOC_148609	221 coding	noncoding	noncoding	noncoding
NR_002775.2	RPLP0P2	XLOC_148621	3525 coding	coding	coding	coding
MSTRG.146901.1		XLOC_148622	526 coding	noncoding	noncoding	noncoding
MSTRG.146984.18	BEST1	XLOC_148631	1075 coding	coding	noncoding	noncoding
MSTRG.146980.1		XLOC_148637	228 coding	noncoding	noncoding	noncoding
XM_005274005.3	SCGB2A2	XLOC_148647	465 coding	coding	coding	coding
MSTRG.147010.1		XLOC_148648	239 coding	noncoding	noncoding	noncoding
MSTRG.147022.1	ASRGL1	XLOC_148651	440 coding	noncoding	noncoding	noncoding
MSTRG.147043.32	AHNAK	XLOC_148658	17927 coding	noncoding	coding	coding
NM_001043229.2	CSKMT	XLOC_148667	2034 coding	coding	coding	coding
MSTRG.147141.3	BSCL2	XLOC_148670	1449 coding	coding	coding	noncoding
MSTRG.147066.1		XLOC_148683	305 coding	noncoding	noncoding	noncoding
MSTRG.147068.1		XLOC_148686	346 coding	noncoding	noncoding	noncoding
MSTRG.147078.1	LOC105369333	XLOC_148690	279 coding	noncoding	noncoding	noncoding
MSTRG.147098.1		XLOC_148692	294 coding	noncoding	noncoding	noncoding
MSTRG.147084.1		XLOC_148694	295 coding	noncoding	noncoding	noncoding
MSTRG.147105.1	SLC22A25	XLOC_148695	209 coding	noncoding	noncoding	noncoding
MSTRG.147106.1	SLC22A25	XLOC_148696	262 coding	noncoding	noncoding	noncoding
MSTRG.147108.1	SLC22A25	XLOC_148697	300 coding	noncoding	noncoding	noncoding
MSTRG.147133.1	PLAAT2	XLOC_148713	377 coding	noncoding	noncoding	noncoding
MSTRG.147134.1	PLAAT2	XLOC_148714	285 coding	noncoding	noncoding	noncoding
MSTRG.147200.1	SPINDOC	XLOC_148730	283 coding	noncoding	noncoding	noncoding
MSTRG.147216.1	MACROD1	XLOC_148741	257 coding	noncoding	noncoding	noncoding
MSTRG.147208.4	STIP1	XLOC_148746	275 coding	coding	noncoding	noncoding
NM_018484.4	SLC22A11	XLOC_148775	3545 coding	coding	coding	coding
MSTRG.147280.1		XLOC_148776	213 coding	noncoding	noncoding	noncoding
MSTRG.147289.1	NRXN2	XLOC_148781	320 coding	noncoding	noncoding	noncoding
MSTRG.147290.1	NRXN2	XLOC_148782	320 coding	noncoding	noncoding	noncoding
MSTRG.147308.2	RASGRP2	XLOC_148785	2602 coding	coding	coding	coding
MSTRG.147343.1		XLOC_148810	255 coding	noncoding	noncoding	noncoding
MSTRG.147321.1		XLOC_148812	289 coding	noncoding	noncoding	noncoding
XM_017017452.2	POLA2	XLOC_148828	3037 coding	coding	coding	coding
NR_131012.1	NEAT1	XLOC_148840	22743 coding	noncoding	coding	noncoding
MSTRG.147412.1	LINC02736	XLOC_148858	311 coding	noncoding	noncoding	noncoding
XR_950207.2	RELA-DT	XLOC_148883	969 coding	noncoding	coding	noncoding
MSTRG.147443.1		XLOC_148884	310 coding	noncoding	noncoding	noncoding
MSTRG.147522.1	EFEMP2	XLOC_148893	276 coding	noncoding	noncoding	noncoding
MSTRG.147526.4	FIBP	XLOC_148895	1131 coding	coding	coding	noncoding
MSTRG.147462.1		XLOC_148905	291 coding	noncoding	noncoding	noncoding
MSTRG.147567.1		XLOC_148910	226 coding	noncoding	noncoding	noncoding

MSTRG.147569.1		XLOC_148912	262	coding	noncoding	noncoding	noncoding
MSTRG.147604.1	PACS1	XLOC_148938	297	coding	noncoding	noncoding	noncoding
MSTRG.147611.1		XLOC_148942	266	coding	noncoding	noncoding	noncoding
NR_135761.1	LOC102724064	XLOC_148954	576	coding	coding	noncoding	coding
MSTRG.147562.3	MRPL11	XLOC_148961	669	coding	coding	noncoding	noncoding
NM_130443.4	DPP3	XLOC_148966	2660	coding	coding	coding	coding
MSTRG.147633.1	DPP3	XLOC_148967	237	coding	noncoding	noncoding	noncoding
MSTRG.147645.1	ACTN3	XLOC_148973	271	coding	noncoding	noncoding	noncoding
MSTRG.147653.1	SPTBN2	XLOC_148985	299	coding	noncoding	noncoding	noncoding
MSTRG.147680.1		XLOC_149013	258	coding	noncoding	noncoding	noncoding
MSTRG.147714.1	KDM2A	XLOC_149020	251	coding	noncoding	noncoding	noncoding
MSTRG.147769.1		XLOC_149058	280	coding	noncoding	noncoding	noncoding
XM_024448323.1	TCIRG1	XLOC_149081	3530	coding	coding	coding	coding
MSTRG.147845.1	CHKA	XLOC_149088	363	coding	noncoding	noncoding	noncoding
MSTRG.147846.1	CHKA	XLOC_149089	311	coding	noncoding	noncoding	noncoding
MSTRG.147827.1		XLOC_149093	248	coding	noncoding	noncoding	noncoding
MSTRG.147862.1	LRP5	XLOC_149098	311	coding	noncoding	noncoding	noncoding
MSTRG.147956.1		XLOC_149099	228	coding	noncoding	noncoding	noncoding
XM_024448601.1	PPP6R3	XLOC_149101	5018	coding	coding	coding	coding
NM_001352379.2	PPP6R3	XLOC_149101	5356	coding	coding	coding	coding
XM_024448603.1	PPP6R3	XLOC_149101	4265	coding	coding	coding	coding
MSTRG.147963.1	PPP6R3	XLOC_149105	316	coding	noncoding	noncoding	noncoding
MSTRG.147971.1	PPP6R3	XLOC_149113	235	coding	noncoding	noncoding	noncoding
MSTRG.147865.1		XLOC_149127	296	coding	noncoding	noncoding	noncoding
MSTRG.147866.1		XLOC_149128	407	coding	noncoding	noncoding	noncoding
MSTRG.147905.1	IGHMBP2	XLOC_149146	212	coding	noncoding	noncoding	noncoding
MSTRG.147897.1		XLOC_149151	227	coding	noncoding	noncoding	noncoding
MSTRG.147912.1	LOC338694	XLOC_149161	250	coding	noncoding	noncoding	noncoding
NM_001293294.2	MYEOV	XLOC_149167	2477	coding	noncoding	coding	coding
MSTRG.148027.1	FADD	XLOC_149200	255	coding	noncoding	noncoding	noncoding
MSTRG.148065.1	SHANK2	XLOC_149216	301	coding	noncoding	noncoding	noncoding
MSTRG.148079.1	SHANK2	XLOC_149223	264	coding	noncoding	noncoding	noncoding
MSTRG.148154.1	RNF121	XLOC_149252	218	coding	noncoding	noncoding	noncoding
XM_024448304.1	IL18BP	XLOC_149253	2544	coding	noncoding	coding	coding
MSTRG.148135.1		XLOC_149257	299	coding	coding	noncoding	noncoding
MSTRG.148144.9	INPPL1	XLOC_149260	289	coding	noncoding	noncoding	noncoding
MSTRG.148142.1		XLOC_149263	272	coding	noncoding	noncoding	noncoding
XM_006718734.2	ATG16L2	XLOC_149283	3324	coding	coding	coding	coding
MSTRG.148479.29	FCHSD2	XLOC_149283	1562	coding	coding	noncoding	noncoding
XM_011545077.2	P2RY6	XLOC_149294	3154	coding	coding	coding	coding
NM_176798.2	P2RY6	XLOC_149294	2649	coding	coding	coding	coding
XR_950080.2	RELT	XLOC_149298	3648	coding	coding	coding	coding
MSTRG.148225.1		XLOC_149306	263	coding	noncoding	noncoding	noncoding

NM_001130036.2	PLEKHB1	XLOC_149307	1925 coding	coding	coding	coding
MSTRG.148237.1	MRPL48	XLOC_149311	217 coding	noncoding	noncoding	noncoding
MSTRG.148240.1		XLOC_149318	211 coding	noncoding	noncoding	noncoding
MSTRG.148315.8	KCNE3	XLOC_149332	3413 coding	coding	noncoding	noncoding
MSTRG.148315.14	KCNE3	XLOC_149332	2160 coding	coding	coding	noncoding
XM_011544889.3	RNF169	XLOC_149350	7898 coding	coding	coding	coding
MSTRG.148371.1	SLCO2B1	XLOC_149367	294 coding	noncoding	noncoding	noncoding
MSTRG.148363.1		XLOC_149368	397 coding	noncoding	noncoding	noncoding
MSTRG.148381.1	RPS3	XLOC_149381	220 coding	noncoding	noncoding	noncoding
MSTRG.148382.1	RPS3	XLOC_149382	295 coding	noncoding	noncoding	noncoding
XM_017018227.2	UVRAG	XLOC_149399	4595 coding	coding	coding	coding
MSTRG.148620.1	UVRAG	XLOC_149419	229 coding	noncoding	noncoding	noncoding
MSTRG.148464.3	THAP12	XLOC_149441	2942 coding	coding	noncoding	noncoding
MSTRG.148562.1		XLOC_149478	293 coding	noncoding	noncoding	noncoding
MSTRG.148641.1	ACER3	XLOC_149484	234 coding	noncoding	noncoding	noncoding
MSTRG.148642.1	ACER3	XLOC_149485	236 coding	noncoding	noncoding	noncoding
MSTRG.148696.1	NDUFC2-KCTD14	XLOC_149523	297 coding	noncoding	noncoding	noncoding
MSTRG.148847.1	TENM4	XLOC_149561	285 coding	noncoding	noncoding	noncoding
MSTRG.148714.1		XLOC_149572	236 coding	noncoding	noncoding	noncoding
MSTRG.148725.1		XLOC_149580	226 coding	noncoding	noncoding	noncoding
MSTRG.148732.1		XLOC_149584	247 coding	noncoding	noncoding	noncoding
MSTRG.148748.1		XLOC_149587	207 coding	noncoding	noncoding	noncoding
MSTRG.148757.1		XLOC_149591	282 coding	noncoding	noncoding	noncoding
MSTRG.148769.1		XLOC_149597	263 coding	noncoding	noncoding	noncoding
MSTRG.148780.1		XLOC_149605	218 coding	noncoding	noncoding	noncoding
MSTRG.148781.1		XLOC_149606	204 coding	noncoding	noncoding	noncoding
MSTRG.148787.1		XLOC_149609	258 coding	noncoding	noncoding	noncoding
MSTRG.148790.1		XLOC_149610	234 coding	noncoding	noncoding	noncoding
MSTRG.148803.1		XLOC_149618	221 coding	noncoding	noncoding	noncoding
MSTRG.148880.1		XLOC_149628	299 coding	noncoding	noncoding	noncoding
MSTRG.148956.1	MIR4300HG	XLOC_149637	244 coding	noncoding	noncoding	noncoding
MSTRG.148962.1	MIR4300HG	XLOC_149640	240 coding	coding	noncoding	noncoding
MSTRG.148964.1	MIR4300HG	XLOC_149642	302 coding	noncoding	noncoding	noncoding
MSTRG.148889.1		XLOC_149649	270 coding	noncoding	noncoding	noncoding
MSTRG.148966.1	LINC02734	XLOC_149656	277 coding	noncoding	noncoding	noncoding
MSTRG.148970.1	DDIAS	XLOC_149667	217 coding	noncoding	noncoding	noncoding
MSTRG.149119.1		XLOC_149681	301 coding	noncoding	noncoding	noncoding
NM_001300972.2	ANKRD42	XLOC_149685	2677 coding	coding	coding	coding
MSTRG.149184.1	DLG2	XLOC_149691	257 coding	noncoding	noncoding	noncoding
MSTRG.149199.1	DLG2	XLOC_149699	281 coding	coding	noncoding	noncoding
MSTRG.149203.1	DLG2	XLOC_149702	227 coding	noncoding	noncoding	noncoding
MSTRG.149342.1	DLG2	XLOC_149799	413 coding	coding	noncoding	noncoding
MSTRG.149135.1		XLOC_149805	242 coding	coding	noncoding	noncoding

MSTRG.149148.1		XLOC_149816	288	coding	noncoding	noncoding	noncoding
MSTRG.149386.16	PICALM	XLOC_149833	3446	coding	coding	noncoding	noncoding
MSTRG.149349.1		XLOC_149834	313	coding	noncoding	noncoding	noncoding
NM_001308007.1	EED	XLOC_149839	2532	coding	coding	coding	coding
MSTRG.149357.10	EED	XLOC_149839	408	coding	noncoding	noncoding	noncoding
MSTRG.149373.1		XLOC_149848	253	coding	coding	noncoding	noncoding
MSTRG.149430.1	ME3	XLOC_149854	246	coding	noncoding	noncoding	noncoding
MSTRG.149509.1	LOC107984361	XLOC_149910	248	coding	noncoding	noncoding	noncoding
MSTRG.149510.1	LOC107984361	XLOC_149911	205	coding	noncoding	noncoding	noncoding
MSTRG.149520.1	LOC107984361	XLOC_149917	374	coding	coding	noncoding	noncoding
MSTRG.149666.1	LOC101929174	XLOC_149962	203	coding	noncoding	noncoding	noncoding
MSTRG.149669.1	LOC101929174	XLOC_149963	293	coding	noncoding	noncoding	noncoding
MSTRG.149687.1	LOC101929174	XLOC_149965	410	coding	coding	noncoding	noncoding
MSTRG.149592.1	GRM5	XLOC_149972	265	coding	noncoding	noncoding	noncoding
MSTRG.149603.1	LOC107984363	XLOC_149978	256	coding	noncoding	noncoding	noncoding
MSTRG.149623.1	NOX4	XLOC_149982	280	coding	noncoding	noncoding	noncoding
MSTRG.149624.1	NOX4	XLOC_149983	258	coding	coding	noncoding	noncoding
MSTRG.149629.1	NOX4	XLOC_149986	317	coding	noncoding	noncoding	noncoding
MSTRG.149614.1		XLOC_149989	317	coding	noncoding	noncoding	noncoding
NM_001105522.1	TRIM49D2	XLOC_150001	1651	coding	coding	coding	coding
MSTRG.149694.1	TRIM51EP	XLOC_150004	227	coding	noncoding	noncoding	noncoding
MSTRG.149697.1	TRIM49C	XLOC_150008	463	coding	noncoding	noncoding	noncoding
MSTRG.149779.1	DISC1FP1	XLOC_150013	275	coding	noncoding	noncoding	noncoding
MSTRG.149815.1	DISC1FP1	XLOC_150020	287	coding	coding	noncoding	noncoding
MSTRG.149732.1		XLOC_150030	287	coding	coding	noncoding	noncoding
MSTRG.149758.1		XLOC_150044	310	coding	coding	noncoding	noncoding
MSTRG.149766.1		XLOC_150045	257	coding	noncoding	noncoding	noncoding
XM_017017186.1	FAT3	XLOC_150049	16446	coding	coding	noncoding	coding
MSTRG.149855.1	FAT3	XLOC_150057	270	coding	noncoding	noncoding	noncoding
MSTRG.149836.1	MTNR1B	XLOC_150061	279	coding	noncoding	noncoding	noncoding
MSTRG.149921.1		XLOC_150115	304	coding	noncoding	noncoding	noncoding
MSTRG.149930.1	HEPHL1	XLOC_150119	256	coding	noncoding	noncoding	noncoding
MSTRG.149952.1	LOC105369435	XLOC_150131	248	coding	noncoding	noncoding	noncoding
MSTRG.149961.1	GPR83	XLOC_150139	226	coding	noncoding	noncoding	noncoding
MSTRG.149989.1	C11orf97	XLOC_150150	268	coding	noncoding	noncoding	noncoding
NM_130847.3	AMOTL1	XLOC_150162	8980	coding	coding	coding	coding
MSTRG.150013.1	LOC105369438	XLOC_150163	245	coding	noncoding	noncoding	noncoding
NM_018039.3	KDM4D	XLOC_150182	2951	coding	coding	coding	coding
MSTRG.150124.2	SESN3	XLOC_150199	9796	coding	coding	coding	noncoding
MSTRG.150124.4	SESN3	XLOC_150199	9047	coding	coding	coding	noncoding
MSTRG.150186.14	MTMR2	XLOC_150249	1403	coding	coding	coding	noncoding
MSTRG.150204.1		XLOC_150287	314	coding	noncoding	noncoding	noncoding
MSTRG.150234.1		XLOC_150293	285	coding	noncoding	noncoding	noncoding

MSTRG.150209.1		XLOC_150295	293	coding	noncoding	noncoding	noncoding
MSTRG.150203.1		XLOC_150297	267	coding	coding	noncoding	noncoding
MSTRG.150248.1		XLOC_150303	278	coding	noncoding	coding	noncoding
MSTRG.150257.1		XLOC_150310	255	coding	noncoding	noncoding	noncoding
MSTRG.150259.1		XLOC_150311	216	coding	noncoding	noncoding	noncoding
MSTRG.150263.1		XLOC_150312	584	coding	coding	noncoding	noncoding
MSTRG.150277.1		XLOC_150320	275	coding	noncoding	noncoding	noncoding
MSTRG.150352.1	CNTN5	XLOC_150346	249	coding	coding	noncoding	noncoding
MSTRG.150356.1	CNTN5	XLOC_150348	275	coding	noncoding	noncoding	noncoding
MSTRG.150368.1	CNTN5	XLOC_150356	387	coding	noncoding	noncoding	noncoding
MSTRG.150369.1	CNTN5	XLOC_150357	247	coding	noncoding	noncoding	noncoding
MSTRG.150382.1	CNTN5	XLOC_150364	273	coding	noncoding	noncoding	noncoding
MSTRG.150295.1		XLOC_150366	269	coding	noncoding	noncoding	noncoding
MSTRG.150298.1		XLOC_150368	297	coding	coding	noncoding	noncoding
MSTRG.150407.1	ARHGAP42	XLOC_150377	262	coding	noncoding	noncoding	noncoding
MSTRG.150311.1		XLOC_150389	651	coding	noncoding	noncoding	noncoding
MSTRG.150452.1	TRPC6	XLOC_150398	339	coding	noncoding	noncoding	noncoding
MSTRG.150432.1		XLOC_150401	296	coding	noncoding	noncoding	noncoding
MSTRG.150467.1		XLOC_150418	339	coding	noncoding	noncoding	noncoding
MSTRG.150486.1	YAP1	XLOC_150422	227	coding	noncoding	noncoding	noncoding
MSTRG.150497.1		XLOC_150440	318	coding	noncoding	noncoding	noncoding
MSTRG.150502.1	LOC101928477	XLOC_150443	274	coding	coding	noncoding	noncoding
MSTRG.150528.1	WTAPP1	XLOC_150447	244	coding	coding	noncoding	noncoding
MSTRG.150529.1	WTAPP1	XLOC_150448	307	coding	noncoding	noncoding	noncoding
MSTRG.150525.1		XLOC_150451	240	coding	coding	noncoding	noncoding
MSTRG.150563.1	DYNC2H1	XLOC_150459	291	coding	noncoding	noncoding	noncoding
MSTRG.150555.1		XLOC_150470	300	coding	noncoding	noncoding	noncoding
MSTRG.150591.1		XLOC_150492	237	coding	noncoding	noncoding	noncoding
MSTRG.150916.11	CASP1	XLOC_150526	2818	coding	coding	noncoding	noncoding
MSTRG.150799.1	LOC105369469	XLOC_150534	295	coding	noncoding	noncoding	noncoding
MSTRG.150807.1	LOC105369468	XLOC_150537	240	coding	noncoding	noncoding	noncoding
MSTRG.150809.1	LOC105369468	XLOC_150538	252	coding	noncoding	noncoding	noncoding
XM_017017610.2	GRIA4	XLOC_150541	3696	coding	coding	coding	coding
NR_046356.1	GRIA4	XLOC_150541	5415	coding	coding	coding	coding
XM_011542776.3	GRIA4	XLOC_150541	11052	coding	coding	coding	coding
MSTRG.150815.1	GRIA4	XLOC_150542	302	coding	noncoding	noncoding	noncoding
MSTRG.150897.1	GUCY1A2	XLOC_150583	236	coding	noncoding	noncoding	noncoding
MSTRG.150960.1	SLC35F2	XLOC_150624	286	coding	noncoding	noncoding	noncoding
MSTRG.150947.1		XLOC_150625	299	coding	noncoding	noncoding	noncoding
MSTRG.150967.1		XLOC_150629	231	coding	noncoding	noncoding	noncoding
MSTRG.150968.1		XLOC_150630	233	coding	noncoding	noncoding	noncoding
MSTRG.151028.1	EXPH5	XLOC_150654	388	coding	noncoding	noncoding	noncoding
MSTRG.151021.1		XLOC_150662	246	coding	noncoding	noncoding	noncoding

MSTRG.151187.1	DDX10	XLOC_150677	288 coding	noncoding	noncoding	noncoding
MSTRG.151191.1		XLOC_150681	347 coding	noncoding	noncoding	noncoding
MSTRG.151041.1		XLOC_150683	276 coding	noncoding	noncoding	noncoding
MSTRG.151070.1		XLOC_150688	287 coding	noncoding	noncoding	noncoding
MSTRG.151078.1		XLOC_150693	319 coding	noncoding	noncoding	noncoding
MSTRG.151079.1		XLOC_150694	312 coding	noncoding	noncoding	noncoding
MSTRG.151054.1	LINC02732	XLOC_150713	283 coding	noncoding	noncoding	noncoding
MSTRG.151056.1	LINC02732	XLOC_150714	284 coding	noncoding	noncoding	noncoding
MSTRG.151137.1	LOC105369487	XLOC_150721	259 coding	noncoding	noncoding	noncoding
MSTRG.151143.1		XLOC_150731	309 coding	noncoding	noncoding	noncoding
MSTRG.151159.1		XLOC_150734	298 coding	noncoding	noncoding	noncoding
MSTRG.151305.1	DIXDC1	XLOC_150804	586 coding	noncoding	noncoding	noncoding
MSTRG.151313.1	DIXDC1	XLOC_150807	305 coding	noncoding	noncoding	noncoding
MSTRG.151320.1	PIH1D2	XLOC_150812	264 coding	noncoding	noncoding	noncoding
MSTRG.151284.1		XLOC_150816	404 coding	noncoding	noncoding	noncoding
MSTRG.151301.1	BCO2	XLOC_150821	298 coding	noncoding	noncoding	noncoding
MSTRG.151425.1	LINC02762	XLOC_150829	211 coding	noncoding	noncoding	noncoding
MSTRG.151352.1		XLOC_150837	217 coding	noncoding	noncoding	noncoding
MSTRG.151384.1		XLOC_150848	210 coding	noncoding	noncoding	noncoding
MSTRG.151385.1		XLOC_150849	256 coding	coding	noncoding	noncoding
MSTRG.151497.1	NCAM1-AS1	XLOC_150871	244 coding	noncoding	noncoding	noncoding
MSTRG.151548.6	USP28	XLOC_150892	883 coding	coding	noncoding	noncoding
MSTRG.151535.1	HTR3A	XLOC_150897	262 coding	noncoding	noncoding	noncoding
MSTRG.151537.1		XLOC_150900	317 coding	noncoding	noncoding	noncoding
MSTRG.151562.1		XLOC_150922	303 coding	noncoding	coding	noncoding
MSTRG.151589.1		XLOC_150930	275 coding	noncoding	noncoding	noncoding
MSTRG.151610.1	NXPE4	XLOC_150938	202 coding	noncoding	noncoding	noncoding
MSTRG.151647.1	LOC107987165	XLOC_150960	237 coding	noncoding	noncoding	noncoding
MSTRG.151630.1		XLOC_150967	240 coding	coding	noncoding	noncoding
MSTRG.151669.1		XLOC_150980	280 coding	noncoding	noncoding	noncoding
MSTRG.151711.1		XLOC_150991	308 coding	noncoding	noncoding	noncoding
MSTRG.151883.2	SIK3	XLOC_151003	3282 coding	coding	noncoding	noncoding
XR_001747824.2	RNF214	XLOC_151013	2277 coding	noncoding	coding	coding
MSTRG.151765.1	RNF214	XLOC_151014	366 coding	noncoding	noncoding	noncoding
MSTRG.151819.1	CEP164	XLOC_151020	228 coding	noncoding	noncoding	noncoding
MSTRG.151830.1	CEP164	XLOC_151023	459 coding	noncoding	noncoding	noncoding
MSTRG.151831.1	CEP164	XLOC_151024	232 coding	noncoding	noncoding	noncoding
MSTRG.151778.1	DSCAML1	XLOC_151028	314 coding	noncoding	noncoding	noncoding
MSTRG.151789.1	DSCAML1	XLOC_151035	253 coding	noncoding	noncoding	noncoding
MSTRG.151834.1		XLOC_151092	282 coding	noncoding	noncoding	noncoding
MSTRG.151970.1	UBE4A	XLOC_151105	253 coding	noncoding	noncoding	noncoding
MSTRG.151966.1		XLOC_151110	225 coding	noncoding	noncoding	noncoding
MSTRG.151985.1	PHLDB1	XLOC_151126	218 coding	noncoding	noncoding	noncoding

MSTRG.152005.1	CXCR5	XLOC_151132	10287 coding	coding	coding	coding
MSTRG.152008.8	CCDC84	XLOC_151137	2024 coding	coding	coding	coding
MSTRG.152068.1	USP2	XLOC_151169	208 coding	noncoding	noncoding	noncoding
MSTRG.152110.1		XLOC_151194	318 coding	noncoding	noncoding	noncoding
XM_006718805.3	ARHGEF12	XLOC_151214	9211 coding	coding	coding	coding
MSTRG.152223.1		XLOC_151250	259 coding	noncoding	noncoding	noncoding
MSTRG.152285.1		XLOC_151286	247 coding	noncoding	noncoding	noncoding
MSTRG.152333.1	MIR100HG	XLOC_151302	307 coding	noncoding	noncoding	noncoding
MSTRG.152366.1	JHY	XLOC_151329	288 coding	noncoding	noncoding	noncoding
MSTRG.152373.1		XLOC_151346	249 coding	noncoding	noncoding	noncoding
MSTRG.152518.1	GRAMD1B	XLOC_151352	424 coding	noncoding	noncoding	noncoding
MSTRG.152447.1		XLOC_151356	270 coding	noncoding	noncoding	noncoding
MSTRG.152468.1		XLOC_151374	323 coding	noncoding	noncoding	noncoding
MSTRG.152488.1	VWA5A	XLOC_151377	312 coding	noncoding	noncoding	noncoding
MSTRG.152493.1	OR8B3	XLOC_151388	311 coding	noncoding	noncoding	noncoding
MSTRG.152498.1		XLOC_151394	255 coding	noncoding	noncoding	noncoding
XM_017018350.1	CCDC15	XLOC_151423	2555 coding	noncoding	coding	coding
MSTRG.152563.1		XLOC_151434	205 coding	noncoding	noncoding	noncoding
MSTRG.152622.1	CHEK1	XLOC_151457	295 coding	noncoding	noncoding	noncoding
XR_001748438.1	LOC105369561	XLOC_151504	11178 coding	noncoding	coding	coding
NR_120578.1	LOC101929473	XLOC_151512	2323 coding	noncoding	noncoding	noncoding
MSTRG.152656.1	LOC101929473	XLOC_151514	278 coding	noncoding	noncoding	noncoding
MSTRG.152679.1		XLOC_151528	262 coding	noncoding	noncoding	noncoding
MSTRG.152822.2	ETS1	XLOC_151560	9530 coding	coding	coding	noncoding
MSTRG.152854.1	TP53AIP1	XLOC_151590	388 coding	noncoding	noncoding	noncoding
MSTRG.152990.1	ARHGAP32	XLOC_151622	388 coding	noncoding	noncoding	noncoding
MSTRG.152860.1		XLOC_151672	230 coding	noncoding	noncoding	noncoding
MSTRG.152953.1	TMEM45B	XLOC_151730	232 coding	noncoding	noncoding	noncoding
NM_001328686.2	APLP2	XLOC_151736	3634 coding	coding	coding	coding
MSTRG.153109.5	ZBTB44	XLOC_151747	9070 coding	coding	coding	noncoding
MSTRG.153109.12	ZBTB44	XLOC_151747	1782 coding	coding	noncoding	noncoding
MSTRG.153116.1	ZBTB44-DT	XLOC_151749	286 coding	noncoding	noncoding	noncoding
MSTRG.153094.1		XLOC_151753	288 coding	noncoding	noncoding	noncoding
MSTRG.153102.1		XLOC_151761	219 coding	noncoding	noncoding	noncoding
MSTRG.153141.9	SNX19	XLOC_151767	3678 coding	coding	coding	noncoding
MSTRG.153121.1		XLOC_151768	536 coding	coding	noncoding	noncoding
XM_017017852.2	NTM	XLOC_151775	8139 coding	coding	coding	coding
XM_017017863.1	NTM	XLOC_151775	6446 coding	coding	coding	coding
MSTRG.153159.1	NTM	XLOC_151776	223 coding	noncoding	noncoding	noncoding
MSTRG.153190.1	NTM	XLOC_151789	312 coding	noncoding	noncoding	noncoding
MSTRG.153211.1	OPCML	XLOC_151797	281 coding	noncoding	noncoding	noncoding
MSTRG.153154.1		XLOC_151817	241 coding	noncoding	noncoding	noncoding
MSTRG.153266.2	IGSF9B	XLOC_151828	323 coding	noncoding	noncoding	noncoding

XM_024448438.1	ACAD8	XLOC_151835	2059 coding	coding	coding	coding
MSTRG.153354.1	GLB1L3	XLOC_151838	207 coding	noncoding	noncoding	noncoding
MSTRG.153315.1		XLOC_151864	238 coding	noncoding	noncoding	noncoding
MSTRG.142360.1		XLOC_151884	299 coding	noncoding	noncoding	noncoding
MSTRG.142367.1	PGGHG	XLOC_151889	4580 coding	coding	coding	coding
MSTRG.142369.1		XLOC_151891	287 coding	noncoding	noncoding	noncoding
MSTRG.142370.6	IFITM2	XLOC_151892	1176 coding	coding	coding	noncoding
MSTRG.142391.1	EPS8L2	XLOC_151926	225 coding	coding	noncoding	noncoding
MSTRG.142434.1	AP2A2	XLOC_151948	254 coding	noncoding	noncoding	noncoding
MSTRG.142443.1	MUC5B	XLOC_151955	246 coding	coding	noncoding	noncoding
MSTRG.142446.1	MUC5B	XLOC_151956	216 coding	noncoding	noncoding	noncoding
NM_019009.4	TOLLIP	XLOC_151957	3627 coding	coding	coding	coding
MSTRG.142457.1	LINC02689	XLOC_151961	249 coding	noncoding	noncoding	noncoding
NM_001170820.4	IFITM10	XLOC_151982	3714 coding	coding	coding	coding
MSTRG.142695.1	ART1	XLOC_152076	381 coding	coding	coding	noncoding
MSTRG.142763.3	STIM1	XLOC_152095	931 coding	noncoding	coding	noncoding
MSTRG.142719.1		XLOC_152107	297 coding	noncoding	noncoding	noncoding
NM_001005329.1	OR51A4	XLOC_152137	942 coding	coding	coding	coding
NM_000518.5	HBB	XLOC_152143	628 coding	coding	noncoding	coding
NR_001589.1	HBBP1	XLOC_152146	660 coding	coding	coding	noncoding
NM_000559.2	HBG1	XLOC_152148	584 coding	coding	coding	coding
MSTRG.142808.1	HBG2	XLOC_152148	637 coding	coding	coding	coding
MSTRG.142817.1	OR51B5	XLOC_152157	321 coding	coding	noncoding	noncoding
MSTRG.142823.1	OR51B5	XLOC_152159	206 coding	noncoding	noncoding	noncoding
NM_033092.3	TRIM5	XLOC_152169	3624 coding	coding	coding	coding
NM_001001922.2	OR52N5	XLOC_152188	1034 coding	coding	coding	coding
MSTRG.142916.1	OR52N2	XLOC_152191	253 coding	coding	noncoding	noncoding
MSTRG.142947.1		XLOC_152207	312 coding	coding	noncoding	noncoding
MSTRG.142956.1		XLOC_152212	269 coding	noncoding	noncoding	noncoding
MSTRG.143002.1		XLOC_152227	223 coding	noncoding	noncoding	noncoding
MSTRG.143126.1	RRP8	XLOC_152255	296 coding	noncoding	noncoding	noncoding
MSTRG.143022.1		XLOC_152282	530 coding	noncoding	noncoding	noncoding
MSTRG.143027.1	OR10A5	XLOC_152285	374 coding	noncoding	noncoding	noncoding
MSTRG.143065.1	ZNF215	XLOC_152293	222 coding	noncoding	noncoding	noncoding
MSTRG.143165.1	RIC3	XLOC_152329	681 coding	noncoding	noncoding	noncoding
MSTRG.143285.1	ST5	XLOC_152352	273 coding	noncoding	noncoding	noncoding
MSTRG.143301.1	ST5	XLOC_152359	358 coding	coding	noncoding	noncoding
NM_001286095.1	TMEM9B	XLOC_152363	1733 coding	coding	coding	noncoding
XM_024448608.1	NRIP3	XLOC_152369	3824 coding	coding	coding	coding
MSTRG.143245.1		XLOC_152374	205 coding	noncoding	noncoding	noncoding
MSTRG.143253.1	LOC105376542	XLOC_152377	282 coding	noncoding	noncoding	noncoding
NM_001243254.2	DENND5A	XLOC_152379	4957 coding	coding	coding	coding
MSTRG.143308.1	TMEM41B	XLOC_152399	258 coding	noncoding	noncoding	noncoding

MSTRG.143323.1		XLOC_152407	206 coding	noncoding	noncoding	noncoding
MSTRG.143585.1	LOC101928008	XLOC_152433	473 coding	noncoding	noncoding	noncoding
MSTRG.143663.1	CAND1.11	XLOC_152502	295 coding	noncoding	noncoding	noncoding
NM_001172705.1	EIF4G2	XLOC_152520	4028 coding	coding	coding	coding
NM_001042559.2	EIF4G2	XLOC_152520	3797 coding	coding	coding	coding
MSTRG.143382.1	LOC105379882	XLOC_152533	339 coding	noncoding	noncoding	noncoding
MSTRG.143384.1		XLOC_152534	287 coding	noncoding	noncoding	noncoding
NM_198516.3	GALNT18	XLOC_152543	2503 coding	coding	coding	coding
MSTRG.143484.1	LINC02547	XLOC_152567	259 coding	noncoding	noncoding	noncoding
MSTRG.143690.13	MICAL2	XLOC_152572	4536 coding	noncoding	coding	coding
MSTRG.143526.1	PARVA	XLOC_152579	240 coding	noncoding	noncoding	noncoding
MSTRG.143556.1	TEAD1	XLOC_152590	236 coding	noncoding	noncoding	noncoding
MSTRG.143667.1		XLOC_152603	410 coding	noncoding	noncoding	noncoding
MSTRG.143681.1		XLOC_152615	255 coding	coding	noncoding	noncoding
MSTRG.143684.1		XLOC_152617	253 coding	noncoding	noncoding	noncoding
MSTRG.143726.3	FAR1	XLOC_152621	5285 coding	coding	noncoding	noncoding
MSTRG.143721.1	LINC02548	XLOC_152623	282 coding	noncoding	noncoding	noncoding
MSTRG.143765.1	SPON1	XLOC_152638	260 coding	noncoding	noncoding	noncoding
MSTRG.143852.1		XLOC_152712	324 coding	coding	noncoding	noncoding
MSTRG.143855.1		XLOC_152714	234 coding	noncoding	noncoding	noncoding
MSTRG.143974.1	SOX6	XLOC_152767	220 coding	noncoding	noncoding	noncoding
XM_024448361.1	PLEKHA7	XLOC_152775	7163 coding	coding	coding	coding
NM_001017.3	RPS13	XLOC_152783	527 coding	coding	coding	coding
MSTRG.143998.1	PIK3C2A	XLOC_152787	294 coding	noncoding	noncoding	noncoding
MSTRG.143995.1		XLOC_152798	276 coding	noncoding	noncoding	noncoding
MSTRG.143996.1		XLOC_152799	246 coding	noncoding	noncoding	noncoding
MSTRG.144021.1	NUCB2	XLOC_152800	238 coding	noncoding	noncoding	noncoding
MSTRG.144030.1		XLOC_152819	372 coding	noncoding	noncoding	noncoding
MSTRG.144061.1	MRGPRX3	XLOC_152848	263 coding	coding	noncoding	noncoding
MSTRG.144192.1	LDHAL6A	XLOC_152855	229 coding	noncoding	noncoding	noncoding
MSTRG.144194.1	LDHAL6A	XLOC_152857	278 coding	noncoding	noncoding	noncoding
MSTRG.144205.1	UEVLD	XLOC_152867	214 coding	noncoding	noncoding	noncoding
MSTRG.144208.1	LOC112268073	XLOC_152870	223 coding	noncoding	noncoding	noncoding
XM_011519919.2	SPTY2D1	XLOC_152872	5592 coding	coding	coding	coding
MSTRG.144155.1		XLOC_152874	279 coding	noncoding	noncoding	noncoding
MSTRG.144156.1		XLOC_152875	264 coding	noncoding	noncoding	noncoding
MSTRG.144158.1		XLOC_152876	357 coding	noncoding	noncoding	noncoding
MSTRG.144183.1		XLOC_152890	325 coding	noncoding	noncoding	noncoding
MSTRG.144190.1		XLOC_152893	302 coding	noncoding	noncoding	noncoding
MSTRG.144184.1		XLOC_152894	238 coding	noncoding	noncoding	noncoding
MSTRG.144239.1	CSRP3-AS1	XLOC_152906	227 coding	coding	noncoding	noncoding
MSTRG.144284.1	NAV2	XLOC_152922	222 coding	noncoding	noncoding	noncoding
MSTRG.144299.1	NAV2	XLOC_152934	274 coding	noncoding	noncoding	noncoding

MSTRG.144258.1		XLOC_152960	260 coding	noncoding	noncoding	noncoding
MSTRG.144360.1	NELL1	XLOC_152978	253 coding	noncoding	noncoding	noncoding
MSTRG.144364.1	NELL1	XLOC_152981	279 coding	noncoding	noncoding	noncoding
MSTRG.144389.1	LOC102723370	XLOC_152997	538 coding	noncoding	noncoding	noncoding
MSTRG.144485.1		XLOC_153108	267 coding	noncoding	noncoding	noncoding
MSTRG.144487.1		XLOC_153110	319 coding	noncoding	noncoding	noncoding
MSTRG.144501.1		XLOC_153118	281 coding	noncoding	noncoding	noncoding
MSTRG.144508.1		XLOC_153121	231 coding	noncoding	noncoding	noncoding
MSTRG.144546.1	LUZP2	XLOC_153128	311 coding	coding	noncoding	noncoding
MSTRG.144565.1	LUZP2	XLOC_153140	319 coding	noncoding	noncoding	noncoding
MSTRG.144524.1		XLOC_153143	224 coding	coding	noncoding	noncoding
MSTRG.144647.1		XLOC_153152	277 coding	noncoding	noncoding	noncoding
MSTRG.144653.1		XLOC_153153	370 coding	noncoding	noncoding	noncoding
MSTRG.144664.1	LINC02699	XLOC_153156	265 coding	noncoding	noncoding	noncoding
MSTRG.144649.1		XLOC_153160	222 coding	noncoding	noncoding	noncoding
MSTRG.144670.1		XLOC_153178	208 coding	noncoding	noncoding	noncoding
MSTRG.144674.1		XLOC_153180	280 coding	coding	noncoding	noncoding
MSTRG.144746.1	LGR4	XLOC_153192	267 coding	noncoding	noncoding	noncoding
NM_031217.4	KIF18A	XLOC_153238	3417 coding	coding	coding	coding
MSTRG.144877.1		XLOC_153307	240 coding	noncoding	noncoding	noncoding
MSTRG.145155.1	DCDC1	XLOC_153313	265 coding	noncoding	noncoding	noncoding
MSTRG.145161.1	DCDC1	XLOC_153315	441 coding	noncoding	noncoding	noncoding
MSTRG.144975.1		XLOC_153351	283 coding	noncoding	noncoding	noncoding
NM_001033506.1	CSTF3	XLOC_153371	749 coding	noncoding	noncoding	noncoding
MSTRG.145077.1	HIPK3	XLOC_153393	7929 coding	coding	coding	noncoding
MSTRG.145063.1	KIAA1549L	XLOC_153399	263 coding	noncoding	noncoding	noncoding
MSTRG.145065.1	KIAA1549L	XLOC_153401	241 coding	noncoding	noncoding	noncoding
MSTRG.145071.1	LOC105376617	XLOC_153403	290 coding	noncoding	noncoding	noncoding
NM_012175.4	FBXO3	XLOC_153407	2403 coding	coding	coding	coding
XM_011519981.3	FBXO3	XLOC_153407	3303 coding	coding	coding	coding
MSTRG.145258.1	ABTB2	XLOC_153425	237 coding	noncoding	noncoding	noncoding
MSTRG.145274.1	ABTB2	XLOC_153429	483 coding	noncoding	noncoding	noncoding
NM_198381.2	ELF5	XLOC_153438	2380 coding	coding	coding	coding
MSTRG.145144.1	EHF	XLOC_153445	290 coding	noncoding	noncoding	noncoding
MSTRG.145392.1	LOC102723568	XLOC_153448	256 coding	noncoding	noncoding	noncoding
MSTRG.145413.1	APIP	XLOC_153457	285 coding	noncoding	noncoding	noncoding
MSTRG.145414.2	PDHX	XLOC_153458	1069 coding	coding	coding	noncoding
MSTRG.145500.1		XLOC_153538	271 coding	noncoding	noncoding	noncoding
MSTRG.145538.1		XLOC_153557	207 coding	noncoding	noncoding	noncoding
MSTRG.145543.1		XLOC_153559	281 coding	noncoding	noncoding	noncoding
MSTRG.145551.1		XLOC_153563	265 coding	noncoding	noncoding	noncoding
MSTRG.145554.1		XLOC_153565	311 coding	noncoding	noncoding	noncoding
MSTRG.145565.1		XLOC_153571	277 coding	noncoding	noncoding	noncoding

MSTRG.145584.1	LOC105376637	XLOC_153575	295 coding	noncoding	noncoding	noncoding
MSTRG.145593.1	LOC105376637	XLOC_153579	240 coding	noncoding	noncoding	noncoding
MSTRG.145734.1	LRRC4C	XLOC_153588	282 coding	noncoding	noncoding	noncoding
MSTRG.145737.1	LRRC4C	XLOC_153589	275 coding	noncoding	noncoding	noncoding
MSTRG.145747.1	LRRC4C	XLOC_153594	299 coding	noncoding	noncoding	noncoding
MSTRG.145750.1	LRRC4C	XLOC_153597	286 coding	noncoding	noncoding	noncoding
MSTRG.145752.1	LRRC4C	XLOC_153598	289 coding	noncoding	noncoding	noncoding
MSTRG.145603.1		XLOC_153607	284 coding	noncoding	noncoding	noncoding
MSTRG.145618.1	LINC01499	XLOC_153613	453 coding	noncoding	noncoding	noncoding
MSTRG.145647.1		XLOC_153630	293 coding	noncoding	noncoding	noncoding
MSTRG.145703.1		XLOC_153637	321 coding	noncoding	noncoding	noncoding
MSTRG.145717.1		XLOC_153641	304 coding	coding	noncoding	noncoding
MSTRG.145779.1		XLOC_153646	247 coding	noncoding	noncoding	noncoding
MSTRG.145830.1	HSD17B12	XLOC_153650	268 coding	noncoding	noncoding	noncoding
MSTRG.145857.2	ACCS	XLOC_153665	690 coding	noncoding	noncoding	noncoding
MSTRG.145868.1		XLOC_153683	497 coding	noncoding	noncoding	noncoding
MSTRG.145878.1		XLOC_153697	296 coding	noncoding	noncoding	noncoding
MSTRG.145984.1		XLOC_153751	247 coding	noncoding	noncoding	noncoding
MSTRG.146044.1	AMBRA1	XLOC_153837	234 coding	noncoding	noncoding	noncoding
XM_011520095.2	ARHGAP1	XLOC_153841	4150 coding	coding	coding	coding
MSTRG.146115.1	F2	XLOC_153844	304 coding	coding	coding	noncoding
MSTRG.146116.1	F2	XLOC_153845	242 coding	noncoding	noncoding	noncoding
MSTRG.146118.1		XLOC_153856	269 coding	noncoding	noncoding	noncoding
MSTRG.146183.1	RAPSN	XLOC_153883	261 coding	noncoding	noncoding	noncoding
XM_017017109.1	CELF1	XLOC_153885	7978 coding	coding	coding	coding
NM_001317231.1	MTCH2	XLOC_153888	2628 coding	coding	coding	coding
MSTRG.146212.1		XLOC_153893	250 coding	noncoding	noncoding	noncoding
MSTRG.146305.1	PTPRJ	XLOC_153896	5397 coding	noncoding	noncoding	noncoding
NM_001348223.1	OR4C5	XLOC_153904	1192 coding	coding	coding	coding
MSTRG.146234.1		XLOC_153912	484 coding	noncoding	noncoding	noncoding
MSTRG.146257.1	FOLH1	XLOC_153919	278 coding	noncoding	noncoding	noncoding
NR_003034.2	LOC441601	XLOC_153937	1015 coding	coding	coding	noncoding
MSTRG.146278.1	LOC441601	XLOC_153938	260 coding	noncoding	noncoding	noncoding
MSTRG.146286.1	LINC02750	XLOC_153939	274 coding	noncoding	noncoding	noncoding
MSTRG.146296.1		XLOC_153945	268 coding	noncoding	noncoding	noncoding
MSTRG.146390.1		XLOC_153969	262 coding	noncoding	noncoding	noncoding
MSTRG.146392.1		XLOC_153972	471 coding	noncoding	noncoding	noncoding
MSTRG.146396.1		XLOC_153975	260 coding	coding	noncoding	noncoding
MSTRG.146410.1	OR5AS1	XLOC_153987	303 coding	coding	noncoding	noncoding
MSTRG.146419.1		XLOC_154001	435 coding	noncoding	noncoding	noncoding
MSTRG.146427.1		XLOC_154008	235 coding	noncoding	noncoding	noncoding
MSTRG.146431.1	LOC105369309	XLOC_154010	256 coding	noncoding	noncoding	noncoding
MSTRG.146449.1	LOC105369309	XLOC_154017	233 coding	noncoding	noncoding	noncoding

MSTRG.146434.1		XLOC_154020	241 coding	noncoding	noncoding	noncoding
MSTRG.146474.1	SLC43A1	XLOC_154033	245 coding	noncoding	noncoding	noncoding
MSTRG.146529.1	OR9Q1	XLOC_154062	215 coding	noncoding	noncoding	noncoding
NM_001005211.1	OR9I1	XLOC_154065	945 coding	coding	coding	coding
MSTRG.146535.1	OR9Q1	XLOC_154066	226 coding	noncoding	coding	noncoding
NM_001004471.2	OR10Q1	XLOC_154068	1037 coding	coding	coding	coding
MSTRG.146522.1		XLOC_154070	316 coding	noncoding	noncoding	noncoding
MSTRG.146633.1	LOC283194	XLOC_154113	208 coding	noncoding	noncoding	noncoding
XR_001748223.1	LOC105369315	XLOC_154116	11704 coding	coding	noncoding	noncoding
MSTRG.146693.1		XLOC_154141	250 coding	noncoding	noncoding	noncoding
MSTRG.146707.1		XLOC_154163	252 coding	noncoding	noncoding	noncoding
NM_152851.2	MS4A6A	XLOC_154188	1562 coding	coding	noncoding	coding
XM_024448653.1	MS4A6A	XLOC_154188	1057 coding	coding	coding	coding
MSTRG.146775.1		XLOC_154205	291 coding	noncoding	noncoding	noncoding
MSTRG.146788.1	LINC00301	XLOC_154208	270 coding	noncoding	noncoding	noncoding
MSTRG.146785.1		XLOC_154209	400 coding	noncoding	noncoding	noncoding
MSTRG.146802.1		XLOC_154214	280 coding	noncoding	noncoding	noncoding
NM_001161452.2	CYB561A3	XLOC_154241	2984 coding	coding	coding	coding
MSTRG.146896.1	MIR4488	XLOC_154252	227 coding	noncoding	noncoding	noncoding
XM_005274387.4	SYT7	XLOC_154253	5502 coding	coding	coding	coding
NM_002032.3	FTH1	XLOC_154276	1203 coding	coding	coding	coding
MSTRG.146994.1		XLOC_154300	293 coding	noncoding	coding	noncoding
MSTRG.146997.1		XLOC_154302	300 coding	noncoding	noncoding	noncoding
MSTRG.147011.1		XLOC_154303	239 coding	noncoding	noncoding	noncoding
MSTRG.147015.1	LOC102723765	XLOC_154317	343 coding	noncoding	noncoding	noncoding
XM_017018270.1	AHNAK	XLOC_154318	18556 coding	noncoding	noncoding	coding
MSTRG.147054.1	ROM1	XLOC_154327	215 coding	noncoding	noncoding	noncoding
MSTRG.147148.5	TTC9C	XLOC_154341	1452 coding	noncoding	coding	noncoding
MSTRG.147148.6	TTC9C	XLOC_154341	2278 coding	noncoding	coding	noncoding
MSTRG.147067.1		XLOC_154364	274 coding	noncoding	noncoding	noncoding
MSTRG.147077.1	LOC105369333	XLOC_154369	279 coding	noncoding	noncoding	noncoding
MSTRG.147069.1		XLOC_154370	250 coding	noncoding	noncoding	noncoding
MSTRG.147082.1		XLOC_154374	285 coding	coding	noncoding	noncoding
MSTRG.147083.1		XLOC_154375	313 coding	noncoding	noncoding	noncoding
MSTRG.147097.1		XLOC_154376	294 coding	noncoding	noncoding	noncoding
XM_017017686.1	SLC22A25	XLOC_154379	4809 coding	coding	coding	coding
MSTRG.147107.1	SLC22A25	XLOC_154380	262 coding	noncoding	noncoding	noncoding
MSTRG.147109.1	SLC22A25	XLOC_154381	300 coding	noncoding	noncoding	noncoding
MSTRG.147113.1	SLC22A9	XLOC_154384	253 coding	noncoding	noncoding	noncoding
MSTRG.147172.1	PLAAT3	XLOC_154399	286 coding	noncoding	noncoding	noncoding
MSTRG.147176.1	ATL3	XLOC_154401	308 coding	noncoding	noncoding	noncoding
MSTRG.147179.1	ATL3	XLOC_154404	280 coding	noncoding	noncoding	noncoding
MSTRG.147180.1	ATL3	XLOC_154405	373 coding	noncoding	noncoding	noncoding

MSTRG.147201.1	SPINDOC	XLOC_154410	702 coding	noncoding	noncoding	noncoding
MSTRG.147238.1	KCNK4-TEX40	XLOC_154434	239 coding	noncoding	noncoding	noncoding
MSTRG.147274.1	SLC22A11	XLOC_154452	289 coding	noncoding	noncoding	noncoding
NM_015080.3	NRXN2	XLOC_154454	6623 coding	coding	coding	coding
MSTRG.147314.1		XLOC_154458	286 coding	noncoding	noncoding	noncoding
NM_017525.3	CDC42BPG	XLOC_154465	6161 coding	coding	coding	coding
XM_005273918.2	MAJIN	XLOC_154476	1329 coding	coding	coding	coding
MSTRG.147322.1		XLOC_154479	268 coding	noncoding	noncoding	noncoding
MSTRG.147348.1		XLOC_154494	246 coding	noncoding	noncoding	noncoding
MSTRG.147435.11	NEAT1	XLOC_154510	19320 coding	noncoding	noncoding	coding
MSTRG.147435.9	NEAT1	XLOC_154510	8777 coding	noncoding	noncoding	noncoding
MSTRG.147400.1		XLOC_154512	204 coding	noncoding	noncoding	noncoding
MSTRG.147479.2	EHBP1L1	XLOC_154524	3927 coding	coding	coding	coding
MSTRG.147500.1		XLOC_154536	269 coding	noncoding	noncoding	noncoding
MSTRG.147524.1		XLOC_154553	287 coding	noncoding	noncoding	noncoding
MSTRG.147458.1	LOC105369350	XLOC_154561	283 coding	noncoding	noncoding	noncoding
MSTRG.147463.1		XLOC_154564	291 coding	noncoding	noncoding	noncoding
MSTRG.147577.2	PACS1	XLOC_154573	2043 coding	coding	coding	coding
MSTRG.147530.1	TMEM151A	XLOC_154591	233 coding	coding	coding	noncoding
MSTRG.147563.1		XLOC_154601	240 coding	noncoding	noncoding	noncoding
XR_949860.3	ZDHHC24	XLOC_154611	1775 coding	coding	coding	coding
MSTRG.147646.1	ACTN3	XLOC_154615	452 coding	noncoding	noncoding	noncoding
MSTRG.147612.1		XLOC_154617	287 coding	noncoding	noncoding	noncoding
XM_006718669.3	SPTBN2	XLOC_154623	12969 coding	coding	noncoding	coding
MSTRG.147690.1	PC	XLOC_154636	245 coding	noncoding	noncoding	noncoding
MSTRG.147704.1	SYT12	XLOC_154643	280 coding	noncoding	noncoding	noncoding
MSTRG.147736.1	CARNS1	XLOC_154666	249 coding	noncoding	noncoding	noncoding
MSTRG.147737.1	CARNS1	XLOC_154667	2109 coding	coding	coding	coding
XR_002957210.1	PITPNM1	XLOC_154676	4370 coding	coding	coding	coding
MSTRG.147768.1		XLOC_154683	282 coding	noncoding	noncoding	noncoding
MSTRG.147806.1		XLOC_154694	265 coding	noncoding	noncoding	noncoding
XR_002957254.1	LOC112268076	XLOC_154705	1958 coding	noncoding	coding	coding
MSTRG.147816.1	LOC112268076	XLOC_154706	267 coding	coding	noncoding	noncoding
MSTRG.147855.26	KMT5B	XLOC_154716	651 coding	noncoding	noncoding	noncoding
MSTRG.147828.1		XLOC_154720	248 coding	noncoding	noncoding	noncoding
MSTRG.147867.1		XLOC_154729	466 coding	noncoding	noncoding	noncoding
NM_001876.4	CPT1A	XLOC_154734	5238 coding	coding	coding	coding
MSTRG.147889.1	CPT1A	XLOC_154736	266 coding	noncoding	noncoding	noncoding
MSTRG.147890.1	CPT1A	XLOC_154737	280 coding	noncoding	noncoding	noncoding
MSTRG.147881.1		XLOC_154738	431 coding	noncoding	noncoding	noncoding
MSTRG.147885.1		XLOC_154740	285 coding	noncoding	noncoding	noncoding
MSTRG.148015.1		XLOC_154775	269 coding	noncoding	noncoding	noncoding
XM_017017390.1	SHANK2	XLOC_154802	11610 coding	coding	coding	coding

MSTRG.148060.1	SHANK2	XLOC_154811	268 coding	noncoding	noncoding	noncoding
MSTRG.148078.1	SHANK2	XLOC_154819	222 coding	noncoding	noncoding	noncoding
MSTRG.148002.1		XLOC_154822	243 coding	noncoding	noncoding	noncoding
NR_038862.1	FLJ42102	XLOC_154826	2162 coding	noncoding	noncoding	coding
MSTRG.148085.1		XLOC_154832	328 coding	noncoding	noncoding	noncoding
MSTRG.148124.1		XLOC_154855	246 coding	noncoding	noncoding	noncoding
MSTRG.148128.1	LOC100133315	XLOC_154859	278 coding	noncoding	noncoding	noncoding
MSTRG.148140.1		XLOC_154884	251 coding	noncoding	noncoding	noncoding
MSTRG.148143.1		XLOC_154885	272 coding	noncoding	noncoding	noncoding
MSTRG.148149.1		XLOC_154893	288 coding	noncoding	noncoding	noncoding
NM_014824.3	FCHSD2	XLOC_154917	4710 coding	coding	coding	coding
MSTRG.148496.1	ATG16L2	XLOC_154918	5500 coding	noncoding	coding	noncoding
MSTRG.148274.1	FAM168A	XLOC_154977	214 coding	noncoding	noncoding	noncoding
MSTRG.148277.1	FAM168A	XLOC_154980	269 coding	noncoding	noncoding	noncoding
MSTRG.148286.1	FAM168A	XLOC_154989	212 coding	noncoding	noncoding	noncoding
NM_001243719.1	RAB6A	XLOC_155010	2863 coding	coding	coding	coding
MSTRG.148232.1	RAB6A	XLOC_155012	217 coding	noncoding	noncoding	noncoding
XM_024448674.1	UCP2	XLOC_155021	1830 coding	coding	coding	coding
MSTRG.148329.17	C2CD3	XLOC_155024	744 coding	noncoding	coding	noncoding
MSTRG.148317.1		XLOC_155032	272 coding	noncoding	noncoding	noncoding
MSTRG.148306.1		XLOC_155038	303 coding	coding	noncoding	noncoding
MSTRG.148439.44	XRRA1	XLOC_155056	4772 coding	noncoding	coding	noncoding
XM_017017750.1	ARRB1	XLOC_155078	7478 coding	coding	coding	coding
MSTRG.148396.1	ARRB1	XLOC_155084	492 coding	noncoding	noncoding	noncoding
MSTRG.148412.1	MOGAT2	XLOC_155107	396 coding	coding	noncoding	noncoding
MSTRG.148436.1	LOC105369395	XLOC_155121	230 coding	noncoding	noncoding	noncoding
MSTRG.148437.1	LOC105369395	XLOC_155122	258 coding	noncoding	noncoding	noncoding
XM_011544935.3	LRRC32	XLOC_155136	4602 coding	coding	coding	coding
MSTRG.148658.1	GDPD4	XLOC_155156	215 coding	noncoding	noncoding	noncoding
XM_024448561.1	PAK1	XLOC_155157	3169 coding	coding	coding	coding
MSTRG.148666.1	AQP11	XLOC_155165	287 coding	noncoding	noncoding	noncoding
MSTRG.148896.1	RSF1	XLOC_155176	240 coding	noncoding	noncoding	noncoding
MSTRG.148905.1	RSF1	XLOC_155185	203 coding	noncoding	noncoding	noncoding
MSTRG.148915.1	RSF1	XLOC_155194	382 coding	noncoding	noncoding	noncoding
MSTRG.148932.1	INTS4	XLOC_155208	278 coding	noncoding	noncoding	noncoding
MSTRG.148699.1	NDUFC2-KCTD14	XLOC_155213	270 coding	noncoding	noncoding	noncoding
MSTRG.148700.1	NDUFC2-KCTD14	XLOC_155214	262 coding	noncoding	noncoding	noncoding
MSTRG.148692.1	LOC105369401	XLOC_155216	259 coding	noncoding	noncoding	noncoding
MSTRG.149025.1	ALG8	XLOC_155220	298 coding	noncoding	noncoding	noncoding
XM_006718753.2	GAB2	XLOC_155228	6154 coding	coding	coding	coding
MSTRG.148712.1		XLOC_155301	265 coding	noncoding	noncoding	noncoding
MSTRG.148833.1	TENM4	XLOC_155317	259 coding	noncoding	noncoding	noncoding
MSTRG.148722.1		XLOC_155336	230 coding	noncoding	noncoding	noncoding

MSTRG.148733.1		XLOC_155341	247 coding	noncoding	noncoding	noncoding
MSTRG.148749.1		XLOC_155344	207 coding	noncoding	noncoding	noncoding
MSTRG.148765.1	LOC107984424	XLOC_155352	252 coding	noncoding	noncoding	noncoding
MSTRG.148768.1		XLOC_155354	263 coding	noncoding	noncoding	noncoding
MSTRG.148805.1		XLOC_155370	250 coding	noncoding	noncoding	noncoding
MSTRG.148877.1		XLOC_155378	428 coding	coding	noncoding	noncoding
MSTRG.148888.1		XLOC_155389	277 coding	noncoding	noncoding	noncoding
MSTRG.148939.1		XLOC_155394	313 coding	noncoding	noncoding	noncoding
MSTRG.148967.1		XLOC_155396	277 coding	noncoding	noncoding	noncoding
NM_199418.3	PRCP	XLOC_155398	3721 coding	coding	coding	coding
NM_001286060.2	RAB30	XLOC_155410	9859 coding	coding	coding	coding
MSTRG.149004.1	RAB30	XLOC_155411	602 coding	noncoding	noncoding	noncoding
XM_017017290.1	DLG2	XLOC_155449	6198 coding	coding	coding	coding
MSTRG.149198.1	DLG2	XLOC_155457	281 coding	coding	noncoding	noncoding
MSTRG.149253.1	DLG2	XLOC_155481	246 coding	noncoding	noncoding	noncoding
MSTRG.149271.1	DLG2	XLOC_155482	329 coding	noncoding	noncoding	noncoding
MSTRG.149299.1	DLG2	XLOC_155490	283 coding	coding	noncoding	noncoding
MSTRG.149340.1	DLG2	XLOC_155500	299 coding	coding	noncoding	noncoding
XM_017018087.1	CREBZF	XLOC_155507	4209 coding	noncoding	coding	coding
MSTRG.149172.1	SYTL2	XLOC_155513	284 coding	noncoding	noncoding	noncoding
NM_001206946.2	PICALM	XLOC_155530	4113 coding	coding	coding	coding
XM_005274325.3	PICALM	XLOC_155530	3773 coding	coding	coding	coding
MSTRG.149358.3	EED	XLOC_155564	1271 coding	coding	noncoding	noncoding
MSTRG.149438.1	ME3	XLOC_155584	263 coding	noncoding	noncoding	noncoding
MSTRG.149450.1	ME3	XLOC_155587	238 coding	noncoding	noncoding	noncoding
MSTRG.149464.1	ME3-DT	XLOC_155596	279 coding	noncoding	noncoding	noncoding
MSTRG.149418.1		XLOC_155600	360 coding	noncoding	noncoding	noncoding
MSTRG.149471.1	FZD4-DT	XLOC_155611	315 coding	coding	noncoding	noncoding
MSTRG.149553.1	LOC105369422	XLOC_155614	295 coding	noncoding	noncoding	noncoding
MSTRG.149508.1	LOC107984361	XLOC_155624	248 coding	noncoding	noncoding	noncoding
MSTRG.149512.1	LOC107984361	XLOC_155625	283 coding	noncoding	noncoding	noncoding
MSTRG.149519.1	LOC107984361	XLOC_155628	260 coding	coding	noncoding	noncoding
MSTRG.149521.1	LOC107984361	XLOC_155629	235 coding	noncoding	noncoding	noncoding
MSTRG.149536.1	RAB38	XLOC_155635	357 coding	noncoding	noncoding	noncoding
MSTRG.149581.1		XLOC_155679	269 coding	noncoding	noncoding	noncoding
MSTRG.149597.1	GRM5	XLOC_155689	225 coding	noncoding	noncoding	noncoding
MSTRG.149602.1	LOC107984363	XLOC_155691	256 coding	noncoding	noncoding	noncoding
MSTRG.149583.1		XLOC_155696	345 coding	noncoding	noncoding	noncoding
MSTRG.149708.1		XLOC_155712	228 coding	noncoding	noncoding	noncoding
MSTRG.149711.1		XLOC_155713	227 coding	noncoding	noncoding	noncoding
XM_011542816.3	TRIM49D1	XLOC_155717	1590 coding	coding	coding	coding
NR_146921.1	TRIM51EP	XLOC_155718	1694 coding	coding	coding	coding
MSTRG.149731.1		XLOC_155777	287 coding	coding	noncoding	noncoding

MSTRG.149738.1	LOC107984371	XLOC_155780	226 coding	coding	noncoding	noncoding
MSTRG.149740.1		XLOC_155783	320 coding	noncoding	noncoding	noncoding
MSTRG.149743.1		XLOC_155785	306 coding	noncoding	noncoding	noncoding
MSTRG.149846.1	FAT3	XLOC_155800	242 coding	noncoding	noncoding	noncoding
MSTRG.149900.1	SLC36A4	XLOC_155824	249 coding	noncoding	noncoding	noncoding
MSTRG.149902.1	SLC36A4	XLOC_155825	263 coding	noncoding	noncoding	noncoding
MSTRG.149915.1	DEUP1	XLOC_155830	315 coding	noncoding	noncoding	noncoding
MSTRG.149862.1		XLOC_155834	293 coding	noncoding	noncoding	noncoding
MSTRG.149926.1		XLOC_155854	269 coding	noncoding	noncoding	noncoding
MSTRG.149953.1	LOC105369435	XLOC_155863	573 coding	noncoding	noncoding	noncoding
NM_016540.4	GPR83	XLOC_155867	4277 coding	coding	coding	coding
MSTRG.150154.1		XLOC_155936	303 coding	coding	noncoding	noncoding
MSTRG.150673.1	MAML2	XLOC_155956	308 coding	noncoding	noncoding	noncoding
MSTRG.150689.1	MAML2	XLOC_155969	440 coding	noncoding	noncoding	noncoding
MSTRG.150694.1	MAML2	XLOC_155974	308 coding	noncoding	noncoding	noncoding
MSTRG.150181.1		XLOC_156060	235 coding	noncoding	noncoding	noncoding
MSTRG.150208.1		XLOC_156065	369 coding	noncoding	noncoding	noncoding
MSTRG.150232.1		XLOC_156069	258 coding	noncoding	noncoding	noncoding
MSTRG.150258.1		XLOC_156088	216 coding	noncoding	noncoding	noncoding
MSTRG.150322.1	CNTN5	XLOC_156107	228 coding	noncoding	noncoding	noncoding
MSTRG.150342.1	CNTN5	XLOC_156117	430 coding	noncoding	noncoding	noncoding
MSTRG.150349.1	CNTN5	XLOC_156121	279 coding	noncoding	noncoding	noncoding
MSTRG.150353.1	CNTN5	XLOC_156123	329 coding	coding	noncoding	noncoding
MSTRG.150355.1	CNTN5	XLOC_156124	459 coding	coding	noncoding	noncoding
MSTRG.150362.1	CNTN5	XLOC_156126	235 coding	noncoding	noncoding	noncoding
MSTRG.150363.1	CNTN5	XLOC_156127	283 coding	noncoding	noncoding	noncoding
MSTRG.150375.1	CNTN5	XLOC_156131	458 coding	noncoding	noncoding	noncoding
MSTRG.150376.1	CNTN5	XLOC_156132	206 coding	noncoding	noncoding	noncoding
MSTRG.150383.1	CNTN5	XLOC_156135	273 coding	noncoding	noncoding	noncoding
MSTRG.150294.1		XLOC_156139	269 coding	noncoding	noncoding	noncoding
MSTRG.150305.1		XLOC_156144	532 coding	noncoding	noncoding	noncoding
MSTRG.150400.1	ARHGAP42	XLOC_156150	280 coding	noncoding	noncoding	noncoding
MSTRG.150408.1	ARHGAP42	XLOC_156155	262 coding	noncoding	noncoding	noncoding
MSTRG.150315.1		XLOC_156175	247 coding	noncoding	noncoding	noncoding
MSTRG.150390.1		XLOC_156177	318 coding	noncoding	noncoding	noncoding
MSTRG.150455.1		XLOC_156190	230 coding	coding	noncoding	noncoding
MSTRG.150464.1		XLOC_156197	356 coding	noncoding	noncoding	noncoding
MSTRG.150466.1		XLOC_156198	245 coding	noncoding	noncoding	noncoding
MSTRG.150492.1		XLOC_156209	306 coding	noncoding	noncoding	noncoding
MSTRG.150570.2	DYNC2H1	XLOC_156242	277 coding	coding	noncoding	noncoding
MSTRG.150590.1		XLOC_156276	249 coding	noncoding	noncoding	noncoding
NR_034070.2	CASP12	XLOC_156296	1783 coding	coding	coding	coding
MSTRG.150642.1		XLOC_156298	270 coding	noncoding	noncoding	noncoding

NM_001136109.2	CASP5	XLOC_156303	1280	coding	coding	coding	coding
MSTRG.150923.1	CASP5	XLOC_156307	639	coding	noncoding	noncoding	noncoding
MSTRG.150808.1	LOC105369468	XLOC_156325	252	coding	noncoding	noncoding	noncoding
MSTRG.150816.1	GRIA4	XLOC_156327	302	coding	noncoding	noncoding	noncoding
MSTRG.150834.1	MSANTD4	XLOC_156335	647	coding	noncoding	noncoding	noncoding
MSTRG.150828.1		XLOC_156337	693	coding	noncoding	noncoding	noncoding
MSTRG.150837.1	LINC02719	XLOC_156343	322	coding	noncoding	noncoding	noncoding
MSTRG.150860.1		XLOC_156358	253	coding	noncoding	noncoding	noncoding
NM_001256424.2	GUCY1A2	XLOC_156362	16243	coding	coding	coding	coding
MSTRG.150894.1	LOC112268081	XLOC_156363	254	coding	noncoding	noncoding	noncoding
MSTRG.150898.1	GUCY1A2	XLOC_156365	236	coding	noncoding	noncoding	noncoding
XM_024448768.1	ALKBH8	XLOC_156400	3793	coding	coding	coding	coding
MSTRG.150946.1		XLOC_156409	295	coding	noncoding	noncoding	noncoding
MSTRG.150976.1		XLOC_156414	298	coding	noncoding	noncoding	noncoding
MSTRG.151264.1	C11orf65	XLOC_156422	258	coding	noncoding	noncoding	noncoding
MSTRG.151035.1	EXPH5	XLOC_156427	513	coding	noncoding	noncoding	noncoding
MSTRG.151071.1		XLOC_156439	287	coding	noncoding	noncoding	noncoding
MSTRG.151055.1	LINC02732	XLOC_156478	283	coding	noncoding	noncoding	noncoding
MSTRG.151057.1	LINC02732	XLOC_156479	284	coding	noncoding	noncoding	noncoding
XM_024448600.1	PPP2R1B	XLOC_156531	2461	coding	coding	coding	coding
NM_002716.5	PPP2R1B	XLOC_156531	5553	coding	coding	coding	coding
MSTRG.151267.1	FDXACB1	XLOC_156537	3398	coding	coding	coding	coding
MSTRG.151273.1	HSPB2-C11orf52	XLOC_156540	359	coding	noncoding	noncoding	noncoding
MSTRG.151306.1	DIXDC1	XLOC_156542	260	coding	noncoding	noncoding	noncoding
NR_160400.1	TIMM8B	XLOC_156548	1095	coding	coding	noncoding	noncoding
MSTRG.151370.1		XLOC_156650	313	coding	noncoding	noncoding	noncoding
MSTRG.151389.1		XLOC_156657	258	coding	noncoding	noncoding	noncoding
MSTRG.151501.1	DRD2	XLOC_156674	271	coding	noncoding	noncoding	noncoding
MSTRG.151511.1	LOC107984390	XLOC_156680	255	coding	noncoding	noncoding	noncoding
MSTRG.151521.1	ZW10	XLOC_156683	253	coding	noncoding	noncoding	noncoding
MSTRG.151616.1	NXPE2	XLOC_156742	255	coding	noncoding	noncoding	noncoding
NR_034148.1	LINC00900	XLOC_156770	2245	coding	noncoding	noncoding	noncoding
MSTRG.151648.1	LINC02698	XLOC_156772	237	coding	noncoding	noncoding	noncoding
MSTRG.151670.1		XLOC_156792	269	coding	noncoding	noncoding	noncoding
MSTRG.151683.1		XLOC_156794	302	coding	noncoding	noncoding	noncoding
MSTRG.151858.1	SIK3	XLOC_156821	260	coding	noncoding	noncoding	noncoding
MSTRG.151861.1	SIK3	XLOC_156824	278	coding	noncoding	noncoding	noncoding
MSTRG.151751.1		XLOC_156855	922	coding	coding	coding	noncoding
MSTRG.151944.1	TMPRSS4	XLOC_156893	255	coding	coding	noncoding	noncoding
NM_004588.5	SCN2B	XLOC_156895	4937	coding	coding	coding	coding
MSTRG.151899.1		XLOC_156906	224	coding	noncoding	noncoding	noncoding
MSTRG.151967.2	LOC100131626	XLOC_156912	4321	coding	coding	coding	noncoding
MSTRG.151967.10	LOC100131626	XLOC_156912	6097	coding	coding	noncoding	noncoding

MSTRG.152028.1	TMEM25	XLOC_156915	402 coding	coding	noncoding	noncoding
NM_020153.3	IFT46	XLOC_156917	1875 coding	coding	coding	coding
MSTRG.151991.1		XLOC_156930	297 coding	noncoding	noncoding	noncoding
XR_001748410.1	CCDC84-DT	XLOC_156937	1241 coding	noncoding	noncoding	noncoding
MSTRG.152055.1		XLOC_156961	301 coding	noncoding	noncoding	noncoding
MSTRG.152118.1	NECTIN1	XLOC_156979	321 coding	noncoding	noncoding	noncoding
MSTRG.152112.1		XLOC_156984	555 coding	noncoding	noncoding	noncoding
MSTRG.152162.4	ARHGEF12	XLOC_157004	6285 coding	coding	coding	noncoding
MSTRG.152162.7	ARHGEF12	XLOC_157004	6333 coding	coding	coding	noncoding
MSTRG.152208.9	TBCEL	XLOC_157022	2357 coding	coding	noncoding	noncoding
MSTRG.152418.1	LOC105369536	XLOC_157048	401 coding	noncoding	noncoding	noncoding
MSTRG.152334.1	MIR100HG	XLOC_157119	307 coding	noncoding	noncoding	noncoding
MSTRG.152298.1		XLOC_157120	343 coding	noncoding	noncoding	noncoding
MSTRG.152376.1		XLOC_157123	247 coding	noncoding	noncoding	noncoding
MSTRG.152404.1	CLMP	XLOC_157157	312 coding	noncoding	noncoding	noncoding
MSTRG.152405.1	CLMP	XLOC_157158	240 coding	noncoding	noncoding	noncoding
MSTRG.152374.1		XLOC_157163	299 coding	noncoding	noncoding	noncoding
MSTRG.152513.1	GRAMD1B	XLOC_157170	253 coding	noncoding	noncoding	noncoding
MSTRG.152448.1		XLOC_157176	249 coding	noncoding	noncoding	noncoding
XM_017017716.1	OR8B3	XLOC_157204	2308 coding	coding	coding	coding
MSTRG.152503.1		XLOC_157212	500 coding	noncoding	noncoding	noncoding
MSTRG.152504.1		XLOC_157213	251 coding	noncoding	noncoding	noncoding
NM_212555.3	PATE2	XLOC_157248	1666 coding	noncoding	noncoding	noncoding
MSTRG.152645.1	CDON	XLOC_157255	231 coding	noncoding	noncoding	noncoding
NM_001144827.1	RPUSD4	XLOC_157262	2419 coding	coding	coding	coding
NM_032795.3	RPUSD4	XLOC_157262	2468 coding	coding	coding	coding
MSTRG.152796.5	ST3GAL4	XLOC_157274	741 coding	coding	coding	noncoding
MSTRG.152698.1	KIRREL3	XLOC_157284	273 coding	noncoding	noncoding	noncoding
MSTRG.152680.1		XLOC_157311	262 coding	noncoding	noncoding	noncoding
MSTRG.152683.1		XLOC_157313	302 coding	noncoding	noncoding	noncoding
XM_017018116.1	TP53AIP1	XLOC_157378	5327 coding	coding	coding	coding
MSTRG.152989.1	ARHGAP32	XLOC_157385	344 coding	noncoding	noncoding	noncoding
MSTRG.152997.1	ARHGAP32	XLOC_157387	253 coding	coding	noncoding	noncoding
MSTRG.152998.1	ARHGAP32	XLOC_157388	215 coding	noncoding	noncoding	noncoding
MSTRG.153000.1	ARHGAP32	XLOC_157389	236 coding	coding	noncoding	noncoding
MSTRG.153028.1	ARHGAP32	XLOC_157394	277 coding	noncoding	noncoding	noncoding
MSTRG.152885.1		XLOC_157404	276 coding	coding	noncoding	noncoding
MSTRG.152911.1		XLOC_157410	296 coding	noncoding	noncoding	noncoding
XM_017018023.1	PRDM10	XLOC_157422	8077 coding	coding	coding	coding
MSTRG.153075.1	ST14	XLOC_157441	251 coding	noncoding	noncoding	noncoding
XM_006718826.4	ZBTB44	XLOC_157449	9163 coding	coding	coding	coding
XM_006718825.4	ZBTB44	XLOC_157449	9380 coding	coding	coding	coding
MSTRG.153115.1	ZBTB44-DT	XLOC_157452	275 coding	noncoding	noncoding	noncoding

MSTRG.153095.1		XLOC_157457	254 coding	coding	noncoding	noncoding
MSTRG.153222.1	OPCML	XLOC_157516	227 coding	noncoding	noncoding	noncoding
MSTRG.153223.1	OPCML	XLOC_157517	213 coding	noncoding	noncoding	noncoding
MSTRG.153301.1		XLOC_157565	243 coding	noncoding	noncoding	noncoding
MSTRG.153316.1		XLOC_157578	238 coding	noncoding	noncoding	noncoding
MSTRG.153318.1		XLOC_157579	248 coding	coding	noncoding	noncoding
MSTRG.153324.1		XLOC_157584	319 coding	noncoding	noncoding	noncoding
MSTRG.153326.1		XLOC_157592	298 coding	noncoding	noncoding	noncoding
MSTRG.153327.1		XLOC_157593	263 coding	noncoding	noncoding	noncoding
MSTRG.153366.1		XLOC_157606	287 coding	noncoding	noncoding	noncoding
MSTRG.153382.1	B4GALNT3	XLOC_157609	309 coding	noncoding	noncoding	noncoding
XM_017019835.1	WNK1	XLOC_157619	10234 coding	coding	coding	coding
XM_017019836.1	WNK1	XLOC_157619	10231 coding	coding	coding	coding
XM_017019834.1	WNK1	XLOC_157619	10315 coding	coding	coding	coding
MSTRG.153673.1	RAD52	XLOC_157628	251 coding	noncoding	noncoding	noncoding
MSTRG.153695.1	ERC1	XLOC_157647	290 coding	noncoding	noncoding	noncoding
MSTRG.153702.1	ERC1	XLOC_157654	296 coding	noncoding	noncoding	noncoding
MSTRG.153447.1	CACNA2D4	XLOC_157668	364 coding	coding	noncoding	noncoding
XR_002957360.1	ITFG2	XLOC_157697	3579 coding	coding	coding	coding
MSTRG.153524.1	ITFG2	XLOC_157698	276 coding	noncoding	noncoding	noncoding
MSTRG.153535.1	RHNO1	XLOC_157702	431 coding	noncoding	noncoding	noncoding
MSTRG.153549.1	LOC105369608	XLOC_157733	300 coding	noncoding	noncoding	noncoding
MSTRG.153559.1		XLOC_157738	240 coding	noncoding	noncoding	noncoding
MSTRG.153570.1		XLOC_157741	209 coding	noncoding	noncoding	noncoding
NM_005002.5	NDUFA9	XLOC_157760	8356 coding	coding	coding	coding
NM_000217.3	KCNA1	XLOC_157774	7985 coding	coding	coding	coding
MSTRG.153654.1	LOC105369617	XLOC_157785	295 coding	noncoding	noncoding	noncoding
MSTRG.153792.1	ANO2	XLOC_157798	250 coding	noncoding	noncoding	noncoding
MSTRG.153800.1	ANO2	XLOC_157803	365 coding	noncoding	noncoding	noncoding
MSTRG.153828.13	PLEKHG6	XLOC_157825	3149 coding	coding	coding	coding
MSTRG.153849.17	TAPBPL	XLOC_157833	3396 coding	coding	coding	coding
MSTRG.153845.1		XLOC_157834	238 coding	noncoding	noncoding	noncoding
MSTRG.153847.1		XLOC_157835	283 coding	noncoding	noncoding	noncoding
MSTRG.153875.5	ACRBP	XLOC_157847	825 coding	noncoding	noncoding	noncoding
MSTRG.153880.1		XLOC_157850	350 coding	noncoding	noncoding	noncoding
MSTRG.153907.1	P3H3	XLOC_157862	306 coding	noncoding	noncoding	noncoding
NM_001940.4	ATN1	XLOC_157873	4702 coding	coding	coding	coding
MSTRG.153993.1		XLOC_157901	337 coding	coding	noncoding	noncoding
MSTRG.154006.1		XLOC_157910	240 coding	noncoding	noncoding	noncoding
MSTRG.154024.2	SLC2A3	XLOC_157914	3454 coding	coding	coding	coding
MSTRG.154033.1		XLOC_157915	268 coding	noncoding	noncoding	noncoding
MSTRG.154036.1		XLOC_157917	210 coding	noncoding	noncoding	noncoding
MSTRG.154085.1	LOC112268090	XLOC_157938	226 coding	noncoding	noncoding	noncoding

MSTRG.154154.1		XLOC_157987	246 coding	noncoding	noncoding	noncoding
MSTRG.154163.1		XLOC_157994	233 coding	noncoding	noncoding	noncoding
MSTRG.154269.1	DDX12P	XLOC_158029	237 coding	noncoding	noncoding	noncoding
NR_120305.1	KLRF1	XLOC_158082	949 coding	coding	noncoding	noncoding
NR_147038.1	KLRD1	XLOC_158133	3262 coding	coding	noncoding	noncoding
XM_006719067.4	KLRD1	XLOC_158133	1103 coding	coding	noncoding	coding
MSTRG.154436.1	LINC02617	XLOC_158145	358 coding	noncoding	noncoding	noncoding
MSTRG.154377.1		XLOC_158147	222 coding	noncoding	noncoding	noncoding
MSTRG.154448.1	LOC105369657	XLOC_158160	286 coding	noncoding	noncoding	noncoding
MSTRG.154515.1		XLOC_158233	248 coding	noncoding	noncoding	noncoding
MSTRG.154520.1		XLOC_158235	226 coding	noncoding	noncoding	noncoding
MSTRG.154536.1		XLOC_158296	258 coding	noncoding	noncoding	noncoding
MSTRG.154542.1		XLOC_158300	253 coding	noncoding	noncoding	noncoding
MSTRG.154557.3	LOC107984485	XLOC_158307	5944 coding	coding	coding	noncoding
MSTRG.154700.1		XLOC_158341	270 coding	noncoding	noncoding	noncoding
MSTRG.154698.1		XLOC_158343	228 coding	noncoding	noncoding	noncoding
MSTRG.154709.1	GPRC5A	XLOC_158347	304 coding	noncoding	noncoding	noncoding
MSTRG.154742.1		XLOC_158363	317 coding	noncoding	coding	noncoding
MSTRG.154792.1		XLOC_158372	244 coding	noncoding	noncoding	noncoding
MSTRG.154815.1	GRIN2B	XLOC_158377	387 coding	noncoding	noncoding	noncoding
MSTRG.154831.1		XLOC_158392	290 coding	noncoding	noncoding	noncoding
MSTRG.154832.1		XLOC_158393	306 coding	noncoding	noncoding	noncoding
MSTRG.154839.1		XLOC_158394	299 coding	noncoding	noncoding	noncoding
MSTRG.154840.1		XLOC_158395	308 coding	noncoding	noncoding	noncoding
MSTRG.154842.1		XLOC_158396	228 coding	noncoding	noncoding	noncoding
MSTRG.154846.1		XLOC_158443	289 coding	noncoding	noncoding	noncoding
MSTRG.154900.1	PTPRO	XLOC_158471	295 coding	noncoding	noncoding	noncoding
MSTRG.154903.1	PTPRO	XLOC_158473	379 coding	noncoding	noncoding	noncoding
MSTRG.154956.1	LOC102724146	XLOC_158496	307 coding	noncoding	noncoding	noncoding
NR_036619.1	SKP1P2	XLOC_158533	1882 coding	coding	coding	coding
MSTRG.155089.1		XLOC_158534	326 coding	coding	noncoding	noncoding
MSTRG.155117.1		XLOC_158540	317 coding	noncoding	noncoding	noncoding
MSTRG.155120.1		XLOC_158544	310 coding	noncoding	noncoding	noncoding
MSTRG.155155.1	PIK3C2G	XLOC_158555	276 coding	noncoding	noncoding	noncoding
MSTRG.155138.1		XLOC_158573	251 coding	noncoding	noncoding	noncoding
MSTRG.155183.1		XLOC_158576	270 coding	noncoding	noncoding	noncoding
MSTRG.155196.1	PLEKHA5	XLOC_158586	364 coding	noncoding	noncoding	noncoding
MSTRG.155305.1		XLOC_158597	299 coding	noncoding	noncoding	noncoding
MSTRG.155244.1		XLOC_158619	309 coding	noncoding	noncoding	noncoding
MSTRG.155280.1	SLCO1A2	XLOC_158639	249 coding	noncoding	noncoding	noncoding
NM_001350913.2	PYROXD1	XLOC_158643	4001 coding	coding	coding	coding
NM_001350912.1	PYROXD1	XLOC_158643	4541 coding	coding	coding	coding
NM_030572.4	SPX	XLOC_158645	2280 coding	coding	coding	noncoding

MSTRG.155380.1	ABCC9	XLOC_158656	253	coding	noncoding	noncoding	noncoding
MSTRG.155360.1		XLOC_158658	237	coding	noncoding	noncoding	noncoding
MSTRG.155409.1	ST8SIA1	XLOC_158673	304	coding	noncoding	noncoding	noncoding
MSTRG.155480.1	LOC105369691	XLOC_158701	322	coding	noncoding	noncoding	noncoding
XM_005253420.4	ETNK1	XLOC_158702	1158	coding	coding	coding	coding
MSTRG.155490.1	ETNK1	XLOC_158708	707	coding	noncoding	noncoding	noncoding
MSTRG.155448.1		XLOC_158720	260	coding	noncoding	noncoding	noncoding
MSTRG.155453.1		XLOC_158724	275	coding	noncoding	noncoding	noncoding
MSTRG.155458.1		XLOC_158727	485	coding	noncoding	noncoding	noncoding
MSTRG.155474.1		XLOC_158736	312	coding	noncoding	noncoding	noncoding
MSTRG.155610.1	SOX5	XLOC_158788	322	coding	noncoding	noncoding	noncoding
MSTRG.155533.1	LOC105369698	XLOC_158816	239	coding	noncoding	noncoding	noncoding
MSTRG.155538.1	LOC107984475	XLOC_158825	290	coding	noncoding	noncoding	noncoding
NM_001204126.2	LRMP	XLOC_158828	2329	coding	coding	coding	coding
MSTRG.155711.1	CASC1	XLOC_158844	264	coding	noncoding	noncoding	noncoding
MSTRG.155647.1		XLOC_158847	288	coding	noncoding	noncoding	noncoding
MSTRG.155657.1		XLOC_158855	238	coding	noncoding	coding	noncoding
MSTRG.155665.1		XLOC_158863	270	coding	noncoding	noncoding	noncoding
XM_011520855.2	SSPN	XLOC_158872	4199	coding	coding	coding	noncoding
MSTRG.155896.1		XLOC_158883	292	coding	noncoding	noncoding	noncoding
MSTRG.155905.1	ITPR2	XLOC_158887	14538	coding	coding	coding	noncoding
MSTRG.155905.4	ITPR2	XLOC_158887	11195	coding	coding	coding	noncoding
MSTRG.155738.1		XLOC_158906	259	coding	noncoding	noncoding	noncoding
MSTRG.155739.1		XLOC_158907	256	coding	noncoding	noncoding	noncoding
MSTRG.155740.1		XLOC_158908	316	coding	noncoding	noncoding	noncoding
MSTRG.155783.1	STK38L	XLOC_158928	215	coding	noncoding	noncoding	noncoding
MSTRG.155751.1	ARNTL2	XLOC_158934	272	coding	coding	noncoding	noncoding
MSTRG.155752.1	ARNTL2	XLOC_158935	720	coding	noncoding	noncoding	noncoding
XM_017019780.1	MRPS35	XLOC_158945	685	coding	noncoding	coding	coding
XM_024449036.1	CCDC91	XLOC_158961	2711	coding	noncoding	coding	coding
XM_024449034.1	CCDC91	XLOC_158961	2778	coding	noncoding	coding	coding
MSTRG.156200.13	ERGIC2	XLOC_159015	2443	coding	coding	noncoding	noncoding
MSTRG.156231.1	FAR2	XLOC_159043	317	coding	noncoding	noncoding	noncoding
MSTRG.156065.1	TMTC1	XLOC_159075	237	coding	noncoding	noncoding	noncoding
MSTRG.156039.1	LOC105369715	XLOC_159114	282	coding	noncoding	noncoding	noncoding
MSTRG.156024.1		XLOC_159115	289	coding	noncoding	noncoding	noncoding
MSTRG.156051.1		XLOC_159127	227	coding	noncoding	noncoding	noncoding
MSTRG.156283.1	DDX11-AS1	XLOC_159150	262	coding	noncoding	noncoding	noncoding
MSTRG.156294.1		XLOC_159155	234	coding	noncoding	noncoding	noncoding
MSTRG.156311.1	DENND5B-AS1	XLOC_159164	285	coding	noncoding	noncoding	noncoding
MSTRG.156314.1		XLOC_159170	385	coding	coding	noncoding	noncoding
MSTRG.156364.1	LOC102724618	XLOC_159191	294	coding	noncoding	noncoding	noncoding
MSTRG.156374.1	LOC102724618	XLOC_159194	351	coding	noncoding	noncoding	noncoding

XR_002957369.1	BICD1	XLOC_159197	6585	coding	coding	coding	coding
MSTRG.156379.1	BICD1	XLOC_159198	262	coding	noncoding	noncoding	noncoding
MSTRG.156384.1	BICD1	XLOC_159203	248	coding	noncoding	noncoding	noncoding
MSTRG.156399.1	FGD4	XLOC_159205	264	coding	noncoding	noncoding	noncoding
MSTRG.156414.1		XLOC_159219	266	coding	noncoding	noncoding	noncoding
MSTRG.156420.1	PKP2	XLOC_159221	201	coding	noncoding	noncoding	noncoding
MSTRG.156440.1		XLOC_159235	346	coding	coding	noncoding	noncoding
MSTRG.156456.1		XLOC_159245	349	coding	coding	noncoding	noncoding
MSTRG.156469.1		XLOC_159248	323	coding	noncoding	noncoding	noncoding
MSTRG.156492.1		XLOC_159259	247	coding	coding	noncoding	noncoding
MSTRG.156525.1		XLOC_159277	291	coding	coding	noncoding	noncoding
MSTRG.156567.1		XLOC_159301	262	coding	noncoding	noncoding	noncoding
MSTRG.156721.1	C12orf40	XLOC_159337	311	coding	noncoding	noncoding	noncoding
MSTRG.156623.1		XLOC_159367	320	coding	noncoding	noncoding	noncoding
MSTRG.156889.19	LRRK2	XLOC_159371	2321	coding	coding	noncoding	coding
NM_173600.2	MUC19	XLOC_159375	24727	coding	coding	noncoding	coding
MSTRG.156627.1		XLOC_159377	309	coding	noncoding	noncoding	noncoding
MSTRG.156669.1	CNTN1	XLOC_159383	283	coding	noncoding	noncoding	noncoding
MSTRG.156670.1	CNTN1	XLOC_159384	293	coding	noncoding	noncoding	noncoding
MSTRG.156685.1	CNTN1	XLOC_159393	306	coding	noncoding	noncoding	noncoding
MSTRG.156694.1		XLOC_159412	250	coding	noncoding	noncoding	noncoding
XM_011538459.2	PPHLN1	XLOC_159426	3599	coding	coding	coding	coding
MSTRG.156834.1	PRICKLE1	XLOC_159442	296	coding	noncoding	noncoding	noncoding
MSTRG.156810.1		XLOC_159450	286	coding	noncoding	noncoding	noncoding
MSTRG.156848.1		XLOC_159466	276	coding	noncoding	noncoding	noncoding
MSTRG.156854.1		XLOC_159472	246	coding	noncoding	noncoding	noncoding
MSTRG.156967.3	NELL2	XLOC_159503	3215	coding	coding	coding	noncoding
MSTRG.156905.1		XLOC_159505	232	coding	noncoding	noncoding	noncoding
MSTRG.156911.1		XLOC_159508	295	coding	noncoding	noncoding	noncoding
MSTRG.156939.1		XLOC_159514	266	coding	noncoding	noncoding	noncoding
MSTRG.156922.1		XLOC_159537	275	coding	noncoding	noncoding	noncoding
MSTRG.156925.1		XLOC_159539	311	coding	noncoding	noncoding	noncoding
MSTRG.157010.1		XLOC_159583	310	coding	noncoding	noncoding	noncoding
MSTRG.157012.1		XLOC_159584	301	coding	noncoding	noncoding	noncoding
MSTRG.157585.1	LOC100288798	XLOC_159607	265	coding	noncoding	noncoding	noncoding
MSTRG.157589.1	LOC100288798	XLOC_159611	688	coding	noncoding	noncoding	noncoding
MSTRG.157306.1		XLOC_159664	293	coding	noncoding	noncoding	noncoding
MSTRG.157315.1		XLOC_159674	288	coding	noncoding	noncoding	noncoding
MSTRG.157348.1		XLOC_159696	307	coding	noncoding	noncoding	noncoding
MSTRG.157349.1		XLOC_159697	257	coding	noncoding	noncoding	noncoding
MSTRG.157426.1	RAPGEF3	XLOC_159725	342	coding	noncoding	noncoding	noncoding
MSTRG.157494.1		XLOC_159755	264	coding	noncoding	noncoding	noncoding
MSTRG.157510.1	C12orf54	XLOC_159758	211	coding	noncoding	noncoding	noncoding

MSTRG.157561.1	ARF3	XLOC_159782	4597 coding	coding	coding	noncoding
MSTRG.157736.2	KMT2D	XLOC_159790	2997 coding	noncoding	coding	coding
MSTRG.157737.1	KMT2D	XLOC_159791	1327 coding	noncoding	coding	coding
MSTRG.157738.1	KMT2D	XLOC_159792	403 coding	noncoding	noncoding	noncoding
MSTRG.157739.1	KMT2D	XLOC_159793	469 coding	noncoding	noncoding	noncoding
MSTRG.157740.1	KMT2D	XLOC_159794	294 coding	noncoding	noncoding	noncoding
MSTRG.157741.1	KMT2D	XLOC_159795	884 coding	coding	noncoding	noncoding
MSTRG.157792.1		XLOC_159802	442 coding	noncoding	coding	noncoding
MSTRG.157770.1	SPATS2	XLOC_159817	232 coding	noncoding	noncoding	noncoding
MSTRG.157825.4	TMBIM6	XLOC_159821	2771 coding	coding	coding	coding
MSTRG.157822.1		XLOC_159823	283 coding	noncoding	noncoding	noncoding
MSTRG.157839.1	NCKAP5L	XLOC_159833	315 coding	noncoding	noncoding	noncoding
NM_020039.4	ASIC1	XLOC_159845	4024 coding	coding	coding	coding
MSTRG.157878.1	FAM186A	XLOC_159857	264 coding	noncoding	noncoding	noncoding
XM_024448825.1	LARP4	XLOC_159859	6480 coding	coding	coding	coding
NM_001352312.2	LARP4	XLOC_159859	6490 coding	coding	coding	coding
NM_001352314.1	LARP4	XLOC_159859	6371 coding	coding	coding	coding
MSTRG.157951.1	DIP2B	XLOC_159865	549 coding	noncoding	noncoding	noncoding
MSTRG.157963.1	DIP2B	XLOC_159876	631 coding	noncoding	noncoding	noncoding
MSTRG.157970.1	DIP2B	XLOC_159882	213 coding	noncoding	noncoding	noncoding
MSTRG.157918.1	ATF1	XLOC_159888	276 coding	noncoding	noncoding	noncoding
MSTRG.157913.1	TMPRSS12	XLOC_159890	210 coding	noncoding	noncoding	noncoding
MSTRG.157927.1		XLOC_159904	279 coding	noncoding	noncoding	noncoding
MSTRG.157925.1		XLOC_159905	292 coding	noncoding	noncoding	noncoding
MSTRG.157974.4	TFCP2	XLOC_159907	518 coding	noncoding	noncoding	coding
MSTRG.158042.1	CELA1	XLOC_159916	222 coding	noncoding	noncoding	noncoding
MSTRG.157991.1		XLOC_159927	219 coding	noncoding	noncoding	noncoding
MSTRG.158002.1	SCN8A	XLOC_159931	269 coding	noncoding	noncoding	noncoding
XM_005268824.3	NR4A1	XLOC_159945	2849 coding	coding	coding	coding
MSTRG.158085.1		XLOC_159950	238 coding	noncoding	noncoding	noncoding
MSTRG.158114.1	C12orf80	XLOC_159955	275 coding	noncoding	noncoding	noncoding
MSTRG.158134.1		XLOC_159963	289 coding	noncoding	noncoding	noncoding
MSTRG.158165.1	KRT73	XLOC_159970	1568 coding	coding	noncoding	coding
MSTRG.158145.1	KRT2	XLOC_159974	223 coding	noncoding	noncoding	noncoding
MSTRG.158150.1		XLOC_159978	237 coding	noncoding	noncoding	noncoding
NM_003109.1	SP1	XLOC_160003	7598 coding	coding	coding	coding
NM_018953.3	HOXC5	XLOC_160035	1613 coding	coding	coding	coding
NM_001184976.2	NCKAP1L	XLOC_160068	8898 coding	coding	coding	coding
MSTRG.158341.1	DCD	XLOC_160076	268 coding	coding	noncoding	noncoding
MSTRG.158411.2	LOC107984515	XLOC_160089	5539 coding	coding	noncoding	noncoding
MSTRG.158380.1		XLOC_160096	290 coding	noncoding	noncoding	noncoding
MSTRG.158388.1		XLOC_160101	247 coding	noncoding	noncoding	noncoding
MSTRG.158389.1		XLOC_160102	219 coding	noncoding	noncoding	noncoding

MSTRG.158395.1		XLOC_160110	298 coding	coding	noncoding	noncoding
MSTRG.158403.1		XLOC_160118	300 coding	noncoding	noncoding	noncoding
MSTRG.158407.1		XLOC_160123	230 coding	noncoding	noncoding	noncoding
MSTRG.158440.1		XLOC_160133	301 coding	noncoding	noncoding	noncoding
MSTRG.158471.1		XLOC_160150	430 coding	noncoding	noncoding	noncoding
MSTRG.158515.1	SLC39A5	XLOC_160167	266 coding	noncoding	noncoding	noncoding
NM_144576.4	COQ10A	XLOC_160175	1570 coding	coding	coding	coding
MSTRG.158553.4	STAT2	XLOC_160182	4738 coding	coding	coding	coding
MSTRG.158542.1		XLOC_160184	272 coding	noncoding	noncoding	noncoding
MSTRG.158582.5	PRIM1	XLOC_160197	1463 coding	coding	noncoding	noncoding
MSTRG.158575.1	HSD17B6	XLOC_160199	316 coding	noncoding	noncoding	noncoding
NM_007224.4	NXPH4	XLOC_160227	1805 coding	coding	coding	coding
NR_048562.1	SHMT2	XLOC_160229	2305 coding	coding	coding	coding
NM_005538.4	INHBC	XLOC_160235	3202 coding	coding	coding	coding
MSTRG.158683.1	ARHGAP9	XLOC_160238	372 coding	noncoding	noncoding	noncoding
MSTRG.158642.1	KIF5A	XLOC_160243	308 coding	noncoding	noncoding	noncoding
MSTRG.158679.1		XLOC_160250	211 coding	noncoding	noncoding	noncoding
MSTRG.158676.1		XLOC_160251	495 coding	noncoding	noncoding	noncoding
MSTRG.158768.1	OS9	XLOC_160255	276 coding	noncoding	noncoding	noncoding
MSTRG.158773.1	CTDSP2	XLOC_160258	5660 coding	coding	coding	noncoding
MSTRG.158735.1	LRIG3	XLOC_160288	275 coding	noncoding	noncoding	noncoding
MSTRG.158762.1		XLOC_160303	305 coding	noncoding	noncoding	noncoding
MSTRG.158763.1		XLOC_160304	288 coding	coding	noncoding	noncoding
NR_073055.1	SLC16A7	XLOC_160305	12053 coding	coding	coding	coding
MSTRG.158888.1	SLC16A7	XLOC_160325	271 coding	noncoding	noncoding	noncoding
MSTRG.158792.1		XLOC_160331	304 coding	noncoding	noncoding	noncoding
MSTRG.158817.1		XLOC_160343	232 coding	noncoding	noncoding	noncoding
MSTRG.158820.1		XLOC_160345	281 coding	noncoding	noncoding	noncoding
MSTRG.158840.1		XLOC_160356	318 coding	noncoding	noncoding	noncoding
MSTRG.158851.1		XLOC_160359	267 coding	noncoding	noncoding	noncoding
MSTRG.159007.1	MON2	XLOC_160412	253 coding	noncoding	noncoding	noncoding
MSTRG.158940.1	PPM1H	XLOC_160439	267 coding	noncoding	noncoding	noncoding
MSTRG.158923.1		XLOC_160487	268 coding	noncoding	noncoding	noncoding
MSTRG.158928.1		XLOC_160491	390 coding	coding	noncoding	noncoding
MSTRG.159038.1	SRGAP1	XLOC_160500	325 coding	noncoding	noncoding	noncoding
MSTRG.159069.2	C12orf66	XLOC_160512	306 coding	coding	noncoding	noncoding
MSTRG.159065.1		XLOC_160514	284 coding	noncoding	noncoding	noncoding
MSTRG.159072.1	C12orf56	XLOC_160515	300 coding	noncoding	noncoding	noncoding
MSTRG.159073.1	C12orf56	XLOC_160516	273 coding	noncoding	noncoding	noncoding
MSTRG.159095.1	XPOT	XLOC_160519	237 coding	noncoding	noncoding	noncoding
NM_001330187.1	TBC1D30	XLOC_160548	7706 coding	coding	coding	coding
MSTRG.159168.1	TBC1D30	XLOC_160551	286 coding	noncoding	noncoding	noncoding
MSTRG.159107.1	LINC02389	XLOC_160558	277 coding	noncoding	noncoding	noncoding

MSTRG.159119.1		XLOC_160584	284 coding	noncoding	noncoding	noncoding
MSTRG.159205.1	MSRB3	XLOC_160588	213 coding	noncoding	noncoding	noncoding
MSTRG.159212.1	MSRB3	XLOC_160590	378 coding	noncoding	noncoding	noncoding
MSTRG.159230.1	LOC100507065	XLOC_160599	311 coding	noncoding	noncoding	noncoding
NM_003483.4	HMGA2	XLOC_160605	4140 coding	noncoding	coding	noncoding
MSTRG.159236.3	TMBIM4	XLOC_160618	1068 coding	coding	noncoding	noncoding
MSTRG.159371.1	GRIP1	XLOC_160655	286 coding	noncoding	noncoding	noncoding
MSTRG.159277.1		XLOC_160711	272 coding	noncoding	noncoding	noncoding
MSTRG.159286.1		XLOC_160717	273 coding	noncoding	noncoding	noncoding
MSTRG.159304.1	LINC02408	XLOC_160725	224 coding	noncoding	noncoding	noncoding
MSTRG.159527.1		XLOC_160786	300 coding	noncoding	noncoding	noncoding
MSTRG.159560.1	LOC105369818	XLOC_160797	270 coding	noncoding	noncoding	noncoding
MSTRG.159622.1	RAP1B	XLOC_160829	529 coding	noncoding	noncoding	noncoding
NM_018656.5	SLC35E3	XLOC_160835	17722 coding	coding	coding	coding
MSTRG.159541.1		XLOC_160851	249 coding	noncoding	noncoding	noncoding
MSTRG.159547.1		XLOC_160854	485 coding	noncoding	noncoding	noncoding
XR_001748876.1	YEATS4	XLOC_160869	2864 coding	coding	coding	coding
MSTRG.159721.1		XLOC_160908	278 coding	noncoding	noncoding	noncoding
MSTRG.159725.1		XLOC_160910	211 coding	noncoding	noncoding	noncoding
MSTRG.159723.1		XLOC_160911	327 coding	noncoding	noncoding	noncoding
MSTRG.159726.1		XLOC_160912	442 coding	coding	noncoding	noncoding
MSTRG.159730.1		XLOC_160914	273 coding	noncoding	noncoding	noncoding
MSTRG.159773.1	KCNMB4	XLOC_160939	267 coding	noncoding	noncoding	noncoding
XR_001749196.1	LOC105369828	XLOC_160943	4085 coding	coding	noncoding	noncoding
MSTRG.159817.1	PTPRR	XLOC_160949	224 coding	noncoding	noncoding	noncoding
MSTRG.159822.1	PTPRR	XLOC_160951	354 coding	noncoding	noncoding	noncoding
MSTRG.159829.1		XLOC_160959	270 coding	noncoding	noncoding	noncoding
MSTRG.159833.1	TSPAN8	XLOC_160961	224 coding	noncoding	noncoding	noncoding
MSTRG.159837.1		XLOC_160964	297 coding	noncoding	noncoding	noncoding
MSTRG.159845.1	LOC105369832	XLOC_160966	247 coding	noncoding	noncoding	noncoding
MSTRG.159848.1		XLOC_160971	279 coding	noncoding	noncoding	noncoding
MSTRG.159880.1	LOC105369833	XLOC_160976	282 coding	noncoding	coding	noncoding
MSTRG.159917.1	THAP2	XLOC_160982	218 coding	noncoding	noncoding	noncoding
MSTRG.159886.1	TMEM19	XLOC_160985	307 coding	noncoding	noncoding	noncoding
MSTRG.159891.1		XLOC_160991	284 coding	noncoding	noncoding	noncoding
MSTRG.159929.1		XLOC_161009	264 coding	coding	noncoding	noncoding
MSTRG.159976.1	TRHDE	XLOC_161012	344 coding	coding	noncoding	noncoding
MSTRG.159992.1	TRHDE	XLOC_161019	304 coding	noncoding	noncoding	noncoding
MSTRG.159935.1		XLOC_161024	225 coding	noncoding	noncoding	noncoding
MSTRG.159938.1		XLOC_161026	264 coding	noncoding	noncoding	noncoding
MSTRG.159972.1		XLOC_161034	252 coding	noncoding	noncoding	noncoding
MSTRG.160001.1		XLOC_161037	291 coding	coding	noncoding	noncoding
MSTRG.160052.1		XLOC_161061	324 coding	noncoding	noncoding	noncoding

MSTRG.160157.1	LOC105369844	XLOC_161116	205 coding	noncoding	noncoding	noncoding
MSTRG.160258.2	OSBPL8	XLOC_161134	2636 coding	noncoding	noncoding	noncoding
MSTRG.160202.1		XLOC_161169	259 coding	noncoding	noncoding	noncoding
MSTRG.160207.1		XLOC_161174	263 coding	noncoding	noncoding	noncoding
MSTRG.160221.1		XLOC_161181	382 coding	noncoding	noncoding	noncoding
MSTRG.160284.1		XLOC_161182	366 coding	noncoding	noncoding	noncoding
MSTRG.160394.1	SYT1	XLOC_161220	299 coding	noncoding	noncoding	noncoding
MSTRG.160402.1	SYT1	XLOC_161225	284 coding	noncoding	noncoding	noncoding
MSTRG.160413.1		XLOC_161229	277 coding	coding	coding	noncoding
MSTRG.160415.1		XLOC_161231	286 coding	noncoding	noncoding	noncoding
NM_001368062.1	OTOGL	XLOC_161253	10859 coding	coding	coding	coding
MSTRG.160465.1	OTOGL	XLOC_161256	317 coding	noncoding	noncoding	noncoding
MSTRG.160642.1	ACSS3	XLOC_161285	332 coding	noncoding	noncoding	noncoding
MSTRG.160650.1	PPFIA2	XLOC_161288	314 coding	coding	noncoding	noncoding
MSTRG.160654.1	PPFIA2	XLOC_161291	203 coding	noncoding	noncoding	noncoding
MSTRG.160499.1		XLOC_161297	259 coding	noncoding	noncoding	noncoding
MSTRG.160509.1		XLOC_161301	418 coding	noncoding	noncoding	noncoding
MSTRG.160510.1		XLOC_161302	280 coding	noncoding	noncoding	noncoding
MSTRG.160514.1	LOC105369873	XLOC_161303	279 coding	noncoding	noncoding	noncoding
MSTRG.160525.1		XLOC_161316	299 coding	noncoding	noncoding	noncoding
MSTRG.160790.1	TMTC2	XLOC_161324	221 coding	noncoding	noncoding	noncoding
MSTRG.160793.1	TMTC2	XLOC_161327	220 coding	noncoding	noncoding	noncoding
MSTRG.160799.1	TMTC2	XLOC_161333	272 coding	noncoding	noncoding	noncoding
MSTRG.160540.1		XLOC_161337	260 coding	noncoding	noncoding	noncoding
MSTRG.160553.1		XLOC_161346	257 coding	noncoding	noncoding	noncoding
MSTRG.160574.1	LOC107984536	XLOC_161357	287 coding	noncoding	noncoding	noncoding
MSTRG.160593.1	LOC105369875	XLOC_161368	263 coding	noncoding	noncoding	noncoding
MSTRG.160598.1		XLOC_161370	223 coding	coding	noncoding	noncoding
MSTRG.160610.1	LOC102724680	XLOC_161376	306 coding	noncoding	noncoding	noncoding
XM_011538817.2	LRRIQ1	XLOC_161378	6734 coding	coding	coding	coding
MSTRG.160748.1	MGAT4C	XLOC_161402	576 coding	noncoding	noncoding	noncoding
MSTRG.160757.1	MGAT4C	XLOC_161408	243 coding	noncoding	noncoding	noncoding
MSTRG.160761.1	MGAT4C	XLOC_161410	231 coding	noncoding	noncoding	noncoding
MSTRG.160762.1	MGAT4C	XLOC_161411	307 coding	noncoding	noncoding	noncoding
MSTRG.160765.1	MGAT4C	XLOC_161414	298 coding	noncoding	noncoding	noncoding
MSTRG.160768.1	MGAT4C	XLOC_161416	291 coding	noncoding	noncoding	noncoding
MSTRG.160726.1		XLOC_161427	210 coding	noncoding	noncoding	noncoding
MSTRG.160781.1		XLOC_161437	212 coding	noncoding	noncoding	noncoding
MSTRG.160802.1		XLOC_161452	287 coding	noncoding	noncoding	noncoding
MSTRG.160837.1		XLOC_161472	298 coding	noncoding	noncoding	noncoding
MSTRG.160866.1		XLOC_161508	261 coding	noncoding	noncoding	noncoding
MSTRG.160904.1		XLOC_161529	256 coding	noncoding	noncoding	noncoding
MSTRG.160927.1		XLOC_161543	322 coding	noncoding	noncoding	noncoding

MSTRG.160940.1	DCN	XLOC_161546	207	coding	noncoding	noncoding	noncoding
MSTRG.160957.1	LOC105369898	XLOC_161548	221	coding	noncoding	noncoding	noncoding
MSTRG.160958.1	LOC105369898	XLOC_161549	264	coding	noncoding	noncoding	noncoding
MSTRG.161145.1	LOC643339	XLOC_161641	476	coding	noncoding	noncoding	noncoding
MSTRG.161321.1	CRADD	XLOC_161691	300	coding	noncoding	noncoding	noncoding
MSTRG.161330.1	LOC101928731	XLOC_161700	238	coding	noncoding	noncoding	noncoding
MSTRG.161340.1	CRADD	XLOC_161709	228	coding	noncoding	noncoding	noncoding
MSTRG.161354.1	CRADD	XLOC_161714	392	coding	noncoding	noncoding	noncoding
MSTRG.161361.1	LOC105369911	XLOC_161717	239	coding	noncoding	noncoding	noncoding
MSTRG.161218.1	LOC105369912	XLOC_161722	447	coding	noncoding	noncoding	noncoding
MSTRG.161228.1		XLOC_161732	213	coding	noncoding	noncoding	noncoding
MSTRG.161379.1		XLOC_161760	296	coding	noncoding	noncoding	noncoding
MSTRG.161383.1		XLOC_161807	205	coding	noncoding	noncoding	noncoding
MSTRG.161398.1		XLOC_161813	252	coding	noncoding	noncoding	noncoding
MSTRG.161473.1	LOC105369917	XLOC_161828	234	coding	noncoding	noncoding	noncoding
MSTRG.161483.1		XLOC_161832	282	coding	noncoding	noncoding	noncoding
MSTRG.161508.1	METAP2	XLOC_161836	329	coding	noncoding	noncoding	noncoding
MSTRG.161502.1		XLOC_161842	206	coding	noncoding	noncoding	noncoding
MSTRG.161516.1	NTN4	XLOC_161847	469	coding	noncoding	noncoding	noncoding
MSTRG.161522.1	NTN4	XLOC_161850	200	coding	noncoding	noncoding	noncoding
MSTRG.161539.1	CCDC38	XLOC_161855	217	coding	noncoding	noncoding	noncoding
NM_005230.4	ELK3	XLOC_161867	4201	coding	coding	coding	coding
MSTRG.161710.1		XLOC_161870	323	coding	noncoding	noncoding	noncoding
MSTRG.161586.1		XLOC_161871	229	coding	noncoding	noncoding	noncoding
NR_152618.1	RMST	XLOC_161898	2399	coding	noncoding	noncoding	noncoding
MSTRG.161679.1		XLOC_161915	477	coding	noncoding	noncoding	noncoding
MSTRG.161724.1		XLOC_161928	270	coding	noncoding	noncoding	noncoding
MSTRG.161737.1		XLOC_161938	242	coding	noncoding	noncoding	noncoding
MSTRG.161744.1	TMPO	XLOC_161944	304	coding	noncoding	noncoding	noncoding
MSTRG.161733.1		XLOC_161946	273	coding	noncoding	noncoding	noncoding
MSTRG.161863.1	ANKS1B	XLOC_161968	238	coding	noncoding	noncoding	noncoding
MSTRG.161868.1	ANKS1B	XLOC_161973	247	coding	coding	noncoding	noncoding
MSTRG.161869.1	ANKS1B	XLOC_161974	214	coding	noncoding	noncoding	noncoding
MSTRG.161809.1	DEPDC4	XLOC_162005	275	coding	noncoding	noncoding	noncoding
MSTRG.161812.1	DEPDC4	XLOC_162008	296	coding	noncoding	noncoding	noncoding
MSTRG.161817.1	SCYL2	XLOC_162013	285	coding	noncoding	noncoding	noncoding
MSTRG.161791.1	SLC17A8	XLOC_162015	257	coding	noncoding	noncoding	noncoding
MSTRG.161793.1	SLC17A8	XLOC_162016	281	coding	noncoding	noncoding	noncoding
MSTRG.161795.1	SLC17A8	XLOC_162017	227	coding	noncoding	noncoding	noncoding
MSTRG.161788.1		XLOC_162020	271	coding	noncoding	noncoding	noncoding
MSTRG.161798.1		XLOC_162024	303	coding	coding	noncoding	noncoding
MSTRG.161830.1	ANO4	XLOC_162033	477	coding	noncoding	noncoding	noncoding
MSTRG.161881.1		XLOC_162041	212	coding	noncoding	noncoding	noncoding

MSTRG.161899.1		XLOC_162051	301 coding	noncoding	noncoding	noncoding
MSTRG.161902.1	DRAM1	XLOC_162053	257 coding	noncoding	noncoding	noncoding
MSTRG.161914.1		XLOC_162057	214 coding	noncoding	noncoding	noncoding
MSTRG.161965.1	PARPBP	XLOC_162070	261 coding	noncoding	noncoding	noncoding
MSTRG.161989.1	IGF1	XLOC_162080	209 coding	noncoding	noncoding	noncoding
MSTRG.161997.1	LOC105369944	XLOC_162084	522 coding	noncoding	noncoding	noncoding
MSTRG.161948.1	PAH	XLOC_162092	205 coding	noncoding	noncoding	noncoding
MSTRG.161941.1		XLOC_162096	232 coding	noncoding	noncoding	noncoding
NM_017564.10	STAB2	XLOC_162138	8243 coding	coding	coding	coding
MSTRG.162027.1	NT5DC3	XLOC_162144	317 coding	noncoding	noncoding	noncoding
MSTRG.162035.1	NT5DC3	XLOC_162152	377 coding	coding	noncoding	noncoding
MSTRG.162108.1	TTC41P	XLOC_162160	246 coding	noncoding	noncoding	noncoding
MSTRG.162130.1	GLT8D2	XLOC_162175	390 coding	noncoding	noncoding	noncoding
MSTRG.162386.1	TXNRD1	XLOC_162194	251 coding	noncoding	noncoding	noncoding
MSTRG.162448.1	CHST11	XLOC_162242	277 coding	noncoding	noncoding	noncoding
MSTRG.162209.1		XLOC_162332	214 coding	coding	noncoding	noncoding
NM_001145199.2	C12orf75	XLOC_162344	1311 coding	coding	noncoding	noncoding
MSTRG.162230.1	C12orf75	XLOC_162345	288 coding	noncoding	noncoding	noncoding
MSTRG.162241.1	LOC105369957	XLOC_162351	250 coding	noncoding	noncoding	noncoding
MSTRG.162269.1		XLOC_162358	296 coding	coding	noncoding	noncoding
MSTRG.162338.1		XLOC_162393	405 coding	noncoding	noncoding	noncoding
MSTRG.162345.1	TCP11L2	XLOC_162398	1551 coding	noncoding	noncoding	noncoding
MSTRG.162528.1	POLR3B	XLOC_162400	256 coding	noncoding	noncoding	noncoding
MSTRG.162358.2	MTERF2	XLOC_162430	1383 coding	coding	coding	noncoding
MSTRG.162665.1	BTBD11	XLOC_162452	286 coding	noncoding	noncoding	noncoding
MSTRG.162670.1	BTBD11	XLOC_162453	289 coding	noncoding	coding	noncoding
MSTRG.162555.1		XLOC_162460	238 coding	noncoding	noncoding	noncoding
MSTRG.162556.1		XLOC_162461	393 coding	noncoding	noncoding	noncoding
MSTRG.162560.1		XLOC_162462	251 coding	noncoding	noncoding	noncoding
MSTRG.162566.1		XLOC_162466	279 coding	noncoding	noncoding	noncoding
MSTRG.162595.1		XLOC_162478	289 coding	noncoding	noncoding	noncoding
MSTRG.162620.7	SELPLG	XLOC_162483	3804 coding	noncoding	coding	coding
MSTRG.162624.9	CORO1C	XLOC_162484	3731 coding	coding	coding	coding
MSTRG.162624.8	CORO1C	XLOC_162484	3587 coding	coding	coding	coding
MSTRG.162618.1		XLOC_162492	241 coding	noncoding	noncoding	noncoding
MSTRG.162675.1	SVOP	XLOC_162495	286 coding	noncoding	noncoding	noncoding
XM_017020050.1	USP30	XLOC_162497	3964 coding	coding	coding	coding
MSTRG.162716.1	ACACB	XLOC_162505	262 coding	noncoding	noncoding	noncoding
MSTRG.162717.1	ACACB	XLOC_162506	249 coding	noncoding	noncoding	noncoding
MSTRG.162699.1		XLOC_162510	237 coding	noncoding	noncoding	noncoding
MSTRG.162765.4	MYO1H	XLOC_162511	316 coding	coding	noncoding	noncoding
NM_000431.4	MVK	XLOC_162519	2833 coding	coding	coding	coding
MSTRG.162735.1		XLOC_162524	284 coding	noncoding	noncoding	noncoding

MSTRG.162738.1		XLOC_162526	293	coding	noncoding	noncoding	noncoding
MSTRG.162793.7	GIT2	XLOC_162541	5477	coding	coding	coding	noncoding
MSTRG.162813.1	C12orf76	XLOC_162544	299	coding	noncoding	noncoding	noncoding
MSTRG.162821.1	IFT81	XLOC_162549	236	coding	noncoding	noncoding	noncoding
MSTRG.162822.1	IFT81	XLOC_162550	395	coding	noncoding	noncoding	noncoding
MSTRG.162824.1	IFT81	XLOC_162551	311	coding	noncoding	noncoding	noncoding
MSTRG.162828.1	IFT81	XLOC_162552	285	coding	noncoding	noncoding	noncoding
MSTRG.162816.1	IFT81	XLOC_162553	272	coding	noncoding	noncoding	noncoding
MSTRG.162839.1		XLOC_162558	248	coding	noncoding	noncoding	noncoding
MSTRG.162829.1		XLOC_162560	300	coding	noncoding	noncoding	noncoding
MSTRG.162853.15	RAD9B	XLOC_162567	9911	coding	coding	noncoding	coding
MSTRG.162845.1		XLOC_162569	301	coding	noncoding	noncoding	noncoding
MSTRG.162864.1		XLOC_162574	274	coding	noncoding	noncoding	noncoding
MSTRG.162868.1	CCDC63	XLOC_162576	271	coding	noncoding	noncoding	noncoding
XM_011538061.3	CUX2	XLOC_162578	13706	coding	coding	coding	coding
MSTRG.162901.1	CUX2	XLOC_162586	264	coding	noncoding	noncoding	noncoding
MSTRG.162879.1		XLOC_162594	269	coding	noncoding	noncoding	noncoding
MSTRG.162945.1	SH2B3	XLOC_162598	250	coding	noncoding	noncoding	noncoding
MSTRG.162920.1	ATXN2-AS	XLOC_162607	312	coding	noncoding	noncoding	noncoding
MSTRG.162930.1	ACAD10	XLOC_162610	303	coding	noncoding	noncoding	noncoding
MSTRG.162938.1	ALDH2	XLOC_162613	271	coding	noncoding	noncoding	noncoding
MSTRG.162940.1	ALDH2	XLOC_162615	250	coding	noncoding	noncoding	noncoding
MSTRG.162921.1		XLOC_162616	234	coding	noncoding	noncoding	noncoding
NM_001143906.1	TRAFD1	XLOC_162633	3179	coding	noncoding	coding	coding
XM_011538613.2	PTPN11	XLOC_162643	6109	coding	coding	coding	coding
MSTRG.162957.1		XLOC_162645	272	coding	noncoding	noncoding	noncoding
NM_001320151.2	OAS1	XLOC_162651	2458	coding	coding	coding	coding
MSTRG.163026.1		XLOC_162661	263	coding	noncoding	noncoding	noncoding
MSTRG.163095.2	DDX54	XLOC_162663	1149	coding	coding	coding	noncoding
MSTRG.163100.1	IQCD	XLOC_162665	288	coding	noncoding	noncoding	noncoding
MSTRG.163054.1		XLOC_162678	269	coding	noncoding	noncoding	noncoding
MSTRG.163124.1	LOC107984437	XLOC_162707	245	coding	noncoding	noncoding	noncoding
MSTRG.163128.1		XLOC_162709	318	coding	noncoding	noncoding	noncoding
MSTRG.163177.1	LOC105370003	XLOC_162722	236	coding	noncoding	noncoding	noncoding
MSTRG.163190.1	LOC105370003	XLOC_162728	307	coding	noncoding	noncoding	noncoding
MSTRG.163157.1		XLOC_162738	246	coding	noncoding	noncoding	noncoding
MSTRG.163270.9	MED13L	XLOC_162741	615	coding	noncoding	noncoding	noncoding
MSTRG.163208.1		XLOC_162749	244	coding	noncoding	noncoding	noncoding
MSTRG.163213.1		XLOC_162751	237	coding	noncoding	noncoding	noncoding
MSTRG.163230.1		XLOC_162757	269	coding	noncoding	noncoding	noncoding
MSTRG.163231.1		XLOC_162758	276	coding	noncoding	noncoding	noncoding
MSTRG.163255.1		XLOC_162790	253	coding	noncoding	noncoding	noncoding
MSTRG.163265.1	NOS1	XLOC_162792	270	coding	noncoding	noncoding	noncoding

MSTRG.163268.1	NOS1	XLOC_162793	208	coding	noncoding	noncoding	noncoding
MSTRG.163259.1		XLOC_162795	246	coding	noncoding	noncoding	noncoding
MSTRG.163260.1		XLOC_162796	228	coding	noncoding	noncoding	noncoding
MSTRG.163333.1	KSR2	XLOC_162802	317	coding	noncoding	noncoding	noncoding
MSTRG.163335.1	KSR2	XLOC_162803	214	coding	noncoding	noncoding	noncoding
MSTRG.163339.1	KSR2	XLOC_162805	297	coding	noncoding	noncoding	noncoding
MSTRG.163340.1	KSR2	XLOC_162806	236	coding	noncoding	noncoding	noncoding
NM_001130112.3	RFC5	XLOC_162808	2277	coding	coding	coding	coding
MSTRG.163379.1		XLOC_162820	238	coding	noncoding	noncoding	noncoding
NM_194286.4	SRRM4	XLOC_162831	8431	coding	coding	coding	coding
MSTRG.163410.1	HSPB8	XLOC_162838	303	coding	noncoding	noncoding	noncoding
MSTRG.163439.1	LOC105370027	XLOC_162849	269	coding	noncoding	noncoding	noncoding
XM_005253909.1	PRKAB1	XLOC_162852	2303	coding	coding	coding	coding
MSTRG.163543.7	PXN-AS1	XLOC_162872	4545	coding	coding	coding	coding
MSTRG.163525.1		XLOC_162874	213	coding	noncoding	noncoding	noncoding
MSTRG.163530.1		XLOC_162876	302	coding	noncoding	noncoding	noncoding
NM_176818.3	GATC	XLOC_162879	4238	coding	coding	coding	coding
XM_024449280.1	CABP1	XLOC_162888	1908	coding	coding	coding	coding
MSTRG.163601.4	SPPL3	XLOC_162898	511	coding	coding	noncoding	noncoding
MSTRG.163632.1	LOC105370030	XLOC_162909	301	coding	noncoding	noncoding	noncoding
MSTRG.163639.1	LOC105370032	XLOC_162913	248	coding	noncoding	noncoding	noncoding
MSTRG.163641.1	LOC105370032	XLOC_162915	292	coding	noncoding	noncoding	noncoding
MSTRG.163642.1	LOC105370032	XLOC_162916	268	coding	noncoding	noncoding	noncoding
MSTRG.163619.1		XLOC_162920	260	coding	noncoding	noncoding	noncoding
MSTRG.163627.1		XLOC_162933	265	coding	noncoding	noncoding	noncoding
MSTRG.163656.1	LOC105370034	XLOC_162936	254	coding	noncoding	noncoding	noncoding
MSTRG.163700.1	WDR66	XLOC_162946	280	coding	noncoding	noncoding	noncoding
NM_030765.4	B3GNT4	XLOC_162955	2891	coding	coding	coding	coding
MSTRG.163725.5	CLIP1	XLOC_162958	3251	coding	coding	coding	coding
MSTRG.163725.6	CLIP1	XLOC_162958	1013	coding	coding	coding	noncoding
MSTRG.163765.1	RSRC2	XLOC_162961	6075	coding	coding	coding	noncoding
MSTRG.163765.5	RSRC2	XLOC_162961	1586	coding	coding	coding	noncoding
MSTRG.163774.1	KNTC1	XLOC_162964	265	coding	noncoding	noncoding	noncoding
MSTRG.163733.1		XLOC_162966	305	coding	noncoding	noncoding	noncoding
MSTRG.163734.14	HCAR2	XLOC_162967	2051	coding	coding	coding	coding
MSTRG.163734.16	HCAR2	XLOC_162967	2005	coding	coding	coding	coding
MSTRG.163747.1	CCDC62	XLOC_162970	299	coding	noncoding	noncoding	noncoding
MSTRG.163749.1	CCDC62	XLOC_162971	253	coding	noncoding	noncoding	noncoding
NM_003959.3	HIP1R	XLOC_162972	4509	coding	coding	coding	coding
MSTRG.163780.1	ABCB9	XLOC_162979	200	coding	noncoding	noncoding	noncoding
MSTRG.163782.1	ABCB9	XLOC_162980	281	coding	noncoding	noncoding	noncoding
MSTRG.163791.1	PITPNM2	XLOC_162985	392	coding	coding	noncoding	noncoding
MSTRG.163799.1		XLOC_162998	216	coding	noncoding	noncoding	noncoding

NR_136910.1	KMT5A	XLOC_163000	2905 coding	coding	coding	coding
MSTRG.163829.1		XLOC_163015	320 coding	noncoding	noncoding	noncoding
XM_024449102.1	DDX55	XLOC_163018	2709 coding	coding	coding	coding
XM_017019718.1	DDX55	XLOC_163018	3033 coding	coding	coding	coding
MSTRG.163913.1	ZNF664-RFLNA	XLOC_163040	227 coding	noncoding	noncoding	noncoding
MSTRG.163923.2	NCOR2	XLOC_163044	1509 coding	noncoding	coding	coding
MSTRG.163926.1	NCOR2	XLOC_163046	2472 coding	noncoding	coding	coding
MSTRG.163929.1	NCOR2	XLOC_163048	1034 coding	noncoding	noncoding	coding
MSTRG.163930.1	NCOR2	XLOC_163049	578 coding	noncoding	coding	noncoding
MSTRG.163870.1	SCARB1	XLOC_163100	292 coding	noncoding	noncoding	noncoding
MSTRG.163989.1	LOC105370051	XLOC_163104	351 coding	noncoding	noncoding	noncoding
MSTRG.163990.25	UBC	XLOC_163105	2112 coding	coding	coding	noncoding
MSTRG.164000.1	BRI3BP	XLOC_163116	255 coding	noncoding	noncoding	noncoding
XM_011537854.2	TMEM132B	XLOC_163119	13816 coding	coding	coding	coding
MSTRG.164021.1	TMEM132B	XLOC_163120	306 coding	noncoding	noncoding	noncoding
MSTRG.164029.1	TMEM132B	XLOC_163123	301 coding	noncoding	noncoding	noncoding
MSTRG.164047.1	LOC105370056	XLOC_163134	299 coding	noncoding	noncoding	noncoding
MSTRG.164040.1		XLOC_163135	276 coding	noncoding	noncoding	noncoding
MSTRG.164143.1	LINC00508	XLOC_163187	242 coding	noncoding	noncoding	noncoding
MSTRG.164151.1		XLOC_163194	288 coding	noncoding	noncoding	noncoding
MSTRG.164163.1	TMEM132C	XLOC_163199	242 coding	noncoding	noncoding	noncoding
MSTRG.164165.1	TMEM132C	XLOC_163200	306 coding	noncoding	noncoding	noncoding
NR_159493.1	GLT1D1	XLOC_163211	2929 coding	coding	coding	coding
MSTRG.164385.1	TMEM132D	XLOC_163229	272 coding	noncoding	noncoding	noncoding
MSTRG.164386.1	TMEM132D	XLOC_163230	217 coding	coding	noncoding	noncoding
NM_007197.3	FZD10	XLOC_163240	3282 coding	coding	coding	coding
MSTRG.164330.1	SFSWAP	XLOC_163294	487 coding	noncoding	noncoding	noncoding
MSTRG.164302.1	MMP17	XLOC_163301	240 coding	noncoding	noncoding	noncoding
MSTRG.164304.1		XLOC_163303	325 coding	noncoding	noncoding	noncoding
MSTRG.164491.1		XLOC_163350	297 coding	noncoding	noncoding	noncoding
MSTRG.164502.1		XLOC_163368	257 coding	noncoding	noncoding	noncoding
MSTRG.164548.1	ZNF84	XLOC_163374	253 coding	noncoding	noncoding	noncoding
NM_015394.5	ZNF10	XLOC_163387	4406 coding	coding	coding	coding
MSTRG.153414.1	KDM5A	XLOC_163419	263 coding	noncoding	noncoding	noncoding
MSTRG.153365.1		XLOC_163429	287 coding	noncoding	noncoding	noncoding
MSTRG.153380.1	B4GALNT3	XLOC_163431	313 coding	noncoding	noncoding	noncoding
MSTRG.153383.1	B4GALNT3	XLOC_163433	309 coding	noncoding	noncoding	noncoding
MSTRG.153370.1	LINC02455	XLOC_163441	279 coding	noncoding	noncoding	noncoding
MSTRG.153660.28	WNK1	XLOC_163445	7646 coding	coding	coding	coding
MSTRG.153660.24	WNK1	XLOC_163445	7364 coding	coding	coding	coding
MSTRG.153660.34	RAD52	XLOC_163445	9750 coding	coding	coding	coding
MSTRG.153660.40	RAD52	XLOC_163445	9474 coding	coding	coding	coding
MSTRG.153436.1	WNT5B	XLOC_163460	229 coding	noncoding	noncoding	noncoding

MSTRG.153468.1	CACNA1C	XLOC_163468	279 coding	noncoding	noncoding	noncoding
MSTRG.153482.1		XLOC_163474	316 coding	noncoding	noncoding	noncoding
MSTRG.153530.1	ITFG2	XLOC_163481	287 coding	noncoding	noncoding	noncoding
MSTRG.153454.1		XLOC_163487	303 coding	noncoding	noncoding	noncoding
MSTRG.153489.1	PRMT8	XLOC_163497	274 coding	noncoding	noncoding	noncoding
MSTRG.153760.1	CRACR2A	XLOC_163509	275 coding	noncoding	noncoding	noncoding
MSTRG.153767.1		XLOC_163515	243 coding	noncoding	noncoding	noncoding
MSTRG.153544.1		XLOC_163533	237 coding	noncoding	noncoding	noncoding
MSTRG.153568.1		XLOC_163545	295 coding	noncoding	noncoding	noncoding
MSTRG.153731.1	GALNT8	XLOC_163596	292 coding	noncoding	noncoding	noncoding
XR_001748966.1	LOC105369614	XLOC_163602	2475 coding	noncoding	noncoding	noncoding
MSTRG.153653.1	LOC105369617	XLOC_163616	295 coding	noncoding	noncoding	noncoding
XM_017019672.2	ANO2	XLOC_163624	7377 coding	coding	coding	coding
MSTRG.153751.1	LOC105369623	XLOC_163644	302 coding	noncoding	noncoding	noncoding
MSTRG.153746.1	LOC105369625	XLOC_163650	265 coding	noncoding	noncoding	noncoding
MSTRG.153844.1		XLOC_163664	238 coding	noncoding	noncoding	noncoding
MSTRG.153881.1		XLOC_163686	349 coding	noncoding	noncoding	noncoding
MSTRG.153892.1		XLOC_163692	272 coding	noncoding	noncoding	noncoding
MSTRG.153901.2	CD4	XLOC_163693	3053 coding	coding	noncoding	noncoding
MSTRG.153922.1	LOC105369632	XLOC_163701	293 coding	noncoding	noncoding	noncoding
MSTRG.153928.1		XLOC_163704	231 coding	noncoding	noncoding	noncoding
MSTRG.153948.1		XLOC_163724	230 coding	noncoding	noncoding	noncoding
MSTRG.153970.1	CLSTN3	XLOC_163733	1623 coding	coding	coding	coding
MSTRG.153959.1		XLOC_163738	260 coding	noncoding	noncoding	noncoding
MSTRG.153992.1		XLOC_163745	322 coding	coding	noncoding	noncoding
MSTRG.154010.1	CLEC4C	XLOC_163752	317 coding	noncoding	noncoding	noncoding
MSTRG.154030.1		XLOC_163760	323 coding	noncoding	noncoding	noncoding
MSTRG.154035.1		XLOC_163763	260 coding	noncoding	noncoding	noncoding
MSTRG.154011.1		XLOC_163764	301 coding	noncoding	noncoding	noncoding
MSTRG.154013.1		XLOC_163765	234 coding	noncoding	noncoding	noncoding
MSTRG.154084.1	LOC112268090	XLOC_163800	226 coding	noncoding	noncoding	noncoding
MSTRG.154126.1		XLOC_163807	202 coding	noncoding	noncoding	noncoding
NM_020661.4	AICDA	XLOC_163843	2803 coding	coding	coding	coding
MSTRG.154165.8		XLOC_163849	2244 coding	noncoding	coding	noncoding
MSTRG.154192.1	A2ML1	XLOC_163850	239 coding	coding	noncoding	noncoding
MSTRG.154265.1		XLOC_163867	283 coding	noncoding	noncoding	noncoding
MSTRG.154286.1		XLOC_163881	337 coding	noncoding	noncoding	noncoding
NM_002258.3	KLRB1	XLOC_163885	1478 coding	coding	noncoding	coding
MSTRG.154297.21		XLOC_163888	5218 coding	noncoding	noncoding	noncoding
MSTRG.154341.2	CLEC12A-AS1	XLOC_163911	2278 coding	coding	noncoding	noncoding
MSTRG.154422.1	KLRD1	XLOC_163941	634 coding	noncoding	noncoding	noncoding
MSTRG.154431.10	KLRD1	XLOC_163946	5782 coding	coding	noncoding	noncoding
MSTRG.154471.1		XLOC_163985	239 coding	coding	noncoding	noncoding

MSTRG.154541.1		XLOC_164041	253 coding	noncoding	noncoding	noncoding
MSTRG.154544.1	BCL2L14	XLOC_164042	265 coding	noncoding	noncoding	noncoding
MSTRG.154708.1	GPRC5A	XLOC_164057	238 coding	noncoding	noncoding	noncoding
MSTRG.154777.1		XLOC_164090	576 coding	noncoding	noncoding	noncoding
XM_011520628.2	GRIN2B	XLOC_164097	30873 coding	coding	coding	coding
MSTRG.154814.1	GRIN2B	XLOC_164099	362 coding	noncoding	noncoding	noncoding
MSTRG.154819.1	GRIN2B	XLOC_164102	317 coding	noncoding	noncoding	noncoding
MSTRG.154798.1		XLOC_164107	206 coding	noncoding	noncoding	noncoding
MSTRG.154837.1		XLOC_164116	269 coding	noncoding	noncoding	noncoding
MSTRG.154838.1		XLOC_164117	258 coding	noncoding	noncoding	noncoding
MSTRG.154841.1		XLOC_164118	228 coding	noncoding	noncoding	noncoding
MSTRG.154981.2	PLBD1	XLOC_164119	12819 coding	coding	coding	coding
MSTRG.154845.1		XLOC_164160	289 coding	noncoding	noncoding	noncoding
NM_001190839.3	MGP	XLOC_164163	1725 coding	noncoding	coding	noncoding
MSTRG.154937.1	EPS8	XLOC_164202	241 coding	noncoding	noncoding	noncoding
MSTRG.155073.1		XLOC_164241	263 coding	noncoding	noncoding	noncoding
MSTRG.155110.1		XLOC_164249	304 coding	coding	noncoding	noncoding
MSTRG.155116.1		XLOC_164253	317 coding	noncoding	noncoding	noncoding
MSTRG.155126.1		XLOC_164258	277 coding	noncoding	noncoding	noncoding
MSTRG.155154.1	PIK3C2G	XLOC_164260	291 coding	noncoding	noncoding	noncoding
XM_024449253.1	PLCZ1	XLOC_164269	7578 coding	coding	coding	coding
MSTRG.155133.1		XLOC_164275	392 coding	noncoding	noncoding	noncoding
MSTRG.155139.1		XLOC_164279	251 coding	noncoding	noncoding	noncoding
MSTRG.155143.1	LOC107984527	XLOC_164281	479 coding	coding	noncoding	noncoding
MSTRG.155144.1	LOC107984527	XLOC_164282	280 coding	noncoding	noncoding	noncoding
MSTRG.155182.1		XLOC_164288	270 coding	noncoding	noncoding	noncoding
MSTRG.155278.1	SLCO1C1	XLOC_164384	315 coding	coding	noncoding	noncoding
MSTRG.155245.1		XLOC_164385	250 coding	noncoding	noncoding	noncoding
MSTRG.155251.1	SLCO1B7	XLOC_164387	345 coding	noncoding	noncoding	noncoding
MSTRG.155254.1	SLCO1B1	XLOC_164388	296 coding	noncoding	noncoding	noncoding
NM_134431.3	SLCO1A2	XLOC_164389	7682 coding	coding	noncoding	coding
XM_006719025.4	ABCC9	XLOC_164399	9242 coding	coding	coding	coding
MSTRG.155417.1	ST8SIA1	XLOC_164412	204 coding	noncoding	noncoding	noncoding
MSTRG.155473.1		XLOC_164459	312 coding	noncoding	noncoding	noncoding
MSTRG.155501.1		XLOC_164461	272 coding	coding	noncoding	noncoding
MSTRG.155574.1	SOX5	XLOC_164468	250 coding	noncoding	noncoding	noncoding
MSTRG.155609.1	SOX5	XLOC_164478	322 coding	noncoding	noncoding	noncoding
XR_001749046.1	LOC105369698	XLOC_164491	2781 coding	noncoding	coding	noncoding
MSTRG.155531.1	LOC105369698	XLOC_164495	313 coding	noncoding	noncoding	noncoding
MSTRG.155534.1	LOC105369698	XLOC_164496	239 coding	noncoding	noncoding	noncoding
MSTRG.155539.1	LOC107984475	XLOC_164502	290 coding	noncoding	noncoding	noncoding
MSTRG.155694.1	LOC645177	XLOC_164504	222 coding	noncoding	noncoding	noncoding
MSTRG.155660.1		XLOC_164513	229 coding	noncoding	noncoding	noncoding

MSTRG.155887.1	SSPN	XLOC_164542	255 coding	coding	coding	noncoding
MSTRG.155909.1	ITPR2	XLOC_164558	275 coding	noncoding	noncoding	noncoding
MSTRG.155855.1	LOC105369711	XLOC_164708	257 coding	noncoding	noncoding	noncoding
MSTRG.155859.1		XLOC_164714	250 coding	noncoding	noncoding	noncoding
MSTRG.156201.3	FAR2	XLOC_164718	811 coding	noncoding	noncoding	noncoding
MSTRG.156075.1	TMTC1	XLOC_164760	256 coding	noncoding	noncoding	noncoding
MSTRG.156091.1	TMTC1	XLOC_164764	243 coding	noncoding	noncoding	noncoding
MSTRG.156048.1		XLOC_164777	241 coding	noncoding	noncoding	noncoding
MSTRG.156052.1		XLOC_164779	216 coding	noncoding	noncoding	noncoding
MSTRG.156117.1		XLOC_164799	441 coding	noncoding	noncoding	noncoding
MSTRG.156263.1	CAPRIN2	XLOC_164800	451 coding	noncoding	noncoding	noncoding
NM_032156.4	CAPRIN2	XLOC_164801	4526 coding	coding	coding	coding
XM_006719147.3	CAPRIN2	XLOC_164801	4589 coding	coding	coding	coding
MSTRG.156279.1	DDX11-AS1	XLOC_164812	302 coding	coding	noncoding	noncoding
NR_153414.1	OVOS2	XLOC_164822	5725 coding	coding	coding	coding
MSTRG.156293.1		XLOC_164826	224 coding	noncoding	noncoding	noncoding
MSTRG.156276.1		XLOC_164829	314 coding	noncoding	noncoding	noncoding
MSTRG.156299.1	DENND5B	XLOC_164832	240 coding	noncoding	noncoding	noncoding
MSTRG.156308.1	DENND5B	XLOC_164836	335 coding	noncoding	noncoding	noncoding
MSTRG.156361.1	LOC102724618	XLOC_164858	870 coding	noncoding	noncoding	noncoding
MSTRG.156365.1	LOC102724618	XLOC_164861	945 coding	noncoding	noncoding	noncoding
MSTRG.156372.1	LOC102724618	XLOC_164866	289 coding	noncoding	noncoding	noncoding
MSTRG.156351.1		XLOC_164883	289 coding	noncoding	noncoding	noncoding
MSTRG.156376.1		XLOC_164890	320 coding	noncoding	noncoding	noncoding
MSTRG.156441.1		XLOC_164902	346 coding	coding	noncoding	noncoding
MSTRG.156457.1		XLOC_164909	349 coding	coding	noncoding	noncoding
MSTRG.156466.1		XLOC_164917	234 coding	coding	noncoding	noncoding
MSTRG.156538.1		XLOC_164949	265 coding	coding	noncoding	noncoding
MSTRG.156546.1		XLOC_164959	296 coding	noncoding	noncoding	noncoding
MSTRG.156587.1	CPNE8	XLOC_164966	289 coding	noncoding	noncoding	noncoding
MSTRG.156566.1		XLOC_164974	262 coding	noncoding	noncoding	noncoding
MSTRG.156598.1		XLOC_164990	310 coding	noncoding	noncoding	noncoding
MSTRG.156668.1	CNTN1	XLOC_165045	283 coding	noncoding	noncoding	noncoding
MSTRG.156635.1		XLOC_165057	265 coding	noncoding	noncoding	noncoding
MSTRG.156638.1		XLOC_165069	308 coding	noncoding	noncoding	noncoding
MSTRG.156831.1	PRICKLE1	XLOC_165108	319 coding	noncoding	noncoding	noncoding
MSTRG.156809.1		XLOC_165113	259 coding	noncoding	noncoding	noncoding
MSTRG.156860.1	ADAMTS20	XLOC_165124	252 coding	noncoding	noncoding	noncoding
MSTRG.156852.1		XLOC_165126	290 coding	noncoding	coding	noncoding
MSTRG.156990.1	NELL2	XLOC_165287	263 coding	noncoding	noncoding	noncoding
MSTRG.156910.1		XLOC_165306	482 coding	noncoding	noncoding	noncoding
MSTRG.156943.5	ANO6	XLOC_165310	1409 coding	coding	noncoding	noncoding
XM_011538984.2	SCAF11	XLOC_165329	9115 coding	coding	coding	coding

MSTRG.157009.1		XLOC_165347	342	coding	noncoding	noncoding	noncoding
MSTRG.157041.1	SLC38A1	XLOC_165362	245	coding	noncoding	noncoding	noncoding
MSTRG.157025.1		XLOC_165374	232	coding	noncoding	noncoding	noncoding
MSTRG.157028.1		XLOC_165375	309	coding	noncoding	noncoding	noncoding
MSTRG.157392.15	PCED1B	XLOC_165506	1747	coding	noncoding	coding	coding
MSTRG.157392.20	PCED1B	XLOC_165506	1822	coding	noncoding	coding	coding
MSTRG.157344.1		XLOC_165525	472	coding	noncoding	noncoding	noncoding
MSTRG.157350.1		XLOC_165528	257	coding	noncoding	noncoding	noncoding
MSTRG.157375.1	LINC02354	XLOC_165552	211	coding	noncoding	noncoding	noncoding
MSTRG.157437.1		XLOC_165572	538	coding	noncoding	noncoding	noncoding
MSTRG.157438.1		XLOC_165573	258	coding	noncoding	noncoding	noncoding
MSTRG.157505.1		XLOC_165616	218	coding	noncoding	noncoding	noncoding
MSTRG.157507.1		XLOC_165618	240	coding	noncoding	noncoding	noncoding
MSTRG.157512.1		XLOC_165620	240	coding	noncoding	noncoding	noncoding
MSTRG.157514.1		XLOC_165621	289	coding	noncoding	noncoding	noncoding
MSTRG.157530.1		XLOC_165638	385	coding	noncoding	noncoding	noncoding
MSTRG.157535.1		XLOC_165640	247	coding	noncoding	noncoding	noncoding
MSTRG.157546.1	CCDC65	XLOC_165641	252	coding	noncoding	noncoding	noncoding
MSTRG.157548.1	CCDC65	XLOC_165642	237	coding	noncoding	noncoding	noncoding
MSTRG.157555.1		XLOC_165649	301	coding	noncoding	noncoding	noncoding
XM_017019630.2	LMBR1L	XLOC_165657	4694	coding	coding	coding	coding
MSTRG.157570.1		XLOC_165658	211	coding	noncoding	noncoding	noncoding
MSTRG.157785.1	LOC105369760	XLOC_165660	215	coding	noncoding	noncoding	noncoding
MSTRG.157791.8	TUBA1C	XLOC_165666	1624	coding	coding	coding	coding
MSTRG.157769.1	SPATS2	XLOC_165687	232	coding	noncoding	noncoding	noncoding
MSTRG.157853.1	AQP6	XLOC_165734	286	coding	coding	noncoding	noncoding
MSTRG.157899.1	LIMA1	XLOC_165758	413	coding	noncoding	noncoding	noncoding
MSTRG.157876.1	FAM186A	XLOC_165767	228	coding	noncoding	noncoding	noncoding
MSTRG.157911.1	TMPRSS12	XLOC_165776	279	coding	noncoding	noncoding	noncoding
MSTRG.157939.1	SLC11A2	XLOC_165783	290	coding	noncoding	noncoding	noncoding
MSTRG.157926.1		XLOC_165786	279	coding	noncoding	noncoding	noncoding
MSTRG.157980.1	CSRNP2	XLOC_165789	287	coding	noncoding	noncoding	noncoding
NM_001351088.2	POU6F1	XLOC_165792	3201	coding	noncoding	coding	noncoding
MSTRG.158049.1	GALNT6	XLOC_165800	228	coding	noncoding	noncoding	noncoding
MSTRG.158064.1	SLC4A8	XLOC_165813	290	coding	noncoding	noncoding	noncoding
MSTRG.158066.1	SLC4A8	XLOC_165815	349	coding	noncoding	noncoding	noncoding
MSTRG.158012.1		XLOC_165847	322	coding	noncoding	noncoding	noncoding
MSTRG.158011.1		XLOC_165848	377	coding	noncoding	noncoding	noncoding
MSTRG.158013.1		XLOC_165849	291	coding	noncoding	noncoding	noncoding
NM_002281.4	KRT81	XLOC_165881	1929	coding	coding	coding	coding
MSTRG.158118.1		XLOC_165885	258	coding	coding	noncoding	noncoding
MSTRG.158122.1		XLOC_165888	213	coding	noncoding	noncoding	noncoding
MSTRG.158132.1	KRT6C	XLOC_165895	272	coding	noncoding	noncoding	noncoding

MSTRG.158133.1		XLOC_165897	212	coding	noncoding	noncoding	noncoding
XM_011538257.2	KRT73	XLOC_165906	5890	coding	coding	coding	coding
MSTRG.158149.1		XLOC_165916	237	coding	noncoding	noncoding	noncoding
MSTRG.158176.1		XLOC_165931	263	coding	noncoding	noncoding	noncoding
MSTRG.158192.1	SPRYD3	XLOC_165936	249	coding	noncoding	noncoding	noncoding
MSTRG.158186.1		XLOC_165938	278	coding	noncoding	noncoding	noncoding
MSTRG.158288.2	TARBP2	XLOC_165955	317	coding	coding	noncoding	noncoding
MSTRG.158293.1	ATF7-NPFF	XLOC_165958	257	coding	noncoding	noncoding	noncoding
MSTRG.158299.1	ATF7-NPFF	XLOC_165964	250	coding	noncoding	noncoding	noncoding
MSTRG.158270.1	FLJ12825	XLOC_165992	269	coding	noncoding	noncoding	noncoding
MSTRG.158310.1	NFE2	XLOC_165998	337	coding	noncoding	noncoding	noncoding
MSTRG.158312.7	COPZ1	XLOC_166000	2156	coding	coding	coding	noncoding
MSTRG.158312.10	COPZ1	XLOC_166000	2136	coding	coding	coding	coding
MSTRG.158333.1	LOC102724050	XLOC_166007	297	coding	noncoding	noncoding	noncoding
MSTRG.158335.1	LOC102724050	XLOC_166009	316	coding	noncoding	coding	noncoding
MSTRG.158317.1		XLOC_166011	302	coding	noncoding	noncoding	noncoding
MSTRG.158338.1		XLOC_166031	282	coding	noncoding	noncoding	noncoding
MSTRG.158346.1		XLOC_166033	222	coding	noncoding	noncoding	noncoding
MSTRG.158386.1		XLOC_166051	264	coding	noncoding	noncoding	noncoding
MSTRG.158390.1		XLOC_166054	219	coding	noncoding	noncoding	noncoding
MSTRG.158392.1		XLOC_166055	248	coding	noncoding	noncoding	noncoding
MSTRG.158402.1		XLOC_166059	267	coding	noncoding	noncoding	noncoding
MSTRG.158408.1		XLOC_166060	230	coding	noncoding	noncoding	noncoding
MSTRG.158458.1	SARNP	XLOC_166080	267	coding	noncoding	noncoding	noncoding
MSTRG.158441.1		XLOC_166083	260	coding	noncoding	noncoding	noncoding
MSTRG.158439.1		XLOC_166084	264	coding	noncoding	noncoding	noncoding
MSTRG.158463.1		XLOC_166094	202	coding	noncoding	noncoding	noncoding
MSTRG.158494.4	RPL41	XLOC_166101	507	coding	noncoding	noncoding	noncoding
XM_011538197.2	ANKRD52	XLOC_166110	6730	coding	coding	coding	coding
MSTRG.158534.7	PAN2	XLOC_166117	284	coding	noncoding	noncoding	noncoding
MSTRG.158541.1		XLOC_166123	238	coding	noncoding	noncoding	noncoding
MSTRG.158540.1		XLOC_166124	290	coding	noncoding	noncoding	noncoding
MSTRG.158544.1		XLOC_166127	237	coding	noncoding	noncoding	noncoding
MSTRG.158595.1	RBMS2	XLOC_166131	254	coding	noncoding	noncoding	noncoding
MSTRG.158596.1	RBMS2	XLOC_166132	307	coding	noncoding	noncoding	noncoding
XM_017018740.1	BAZ2A	XLOC_166138	6737	coding	coding	coding	coding
MSTRG.158602.13	BAZ2A	XLOC_166138	321	coding	noncoding	noncoding	noncoding
MSTRG.158603.1	BAZ2A	XLOC_166139	214	coding	noncoding	noncoding	noncoding
MSTRG.158559.1		XLOC_166143	462	coding	noncoding	noncoding	noncoding
XM_011537773.2	PTGES3	XLOC_166144	1497	coding	coding	coding	coding
MSTRG.158566.1		XLOC_166148	534	coding	noncoding	noncoding	noncoding
NM_001256041.2	MYO1A	XLOC_166159	4091	coding	coding	coding	coding
MSTRG.158621.1	NEMP1	XLOC_166162	252	coding	noncoding	noncoding	noncoding

MSTRG.158681.1	GLI1	XLOC_166187	308	coding	noncoding	noncoding	noncoding
XM_011538657.2	ARHGAP9	XLOC_166188	2490	coding	coding	coding	coding
MSTRG.158641.1	KIF5A	XLOC_166192	308	coding	noncoding	noncoding	noncoding
MSTRG.158678.1		XLOC_166201	231	coding	noncoding	noncoding	noncoding
MSTRG.158677.1		XLOC_166202	286	coding	noncoding	noncoding	noncoding
MSTRG.158701.1		XLOC_166225	298	coding	noncoding	noncoding	noncoding
MSTRG.158713.1		XLOC_166233	322	coding	noncoding	noncoding	noncoding
MSTRG.158723.1	LINC02388	XLOC_166239	249	coding	noncoding	noncoding	noncoding
MSTRG.158726.1	LOC100506869	XLOC_166240	285	coding	noncoding	noncoding	noncoding
NM_153377.4	LRIG3	XLOC_166245	4080	coding	coding	coding	coding
MSTRG.158756.1		XLOC_166255	351	coding	noncoding	noncoding	noncoding
MSTRG.158867.3	SLC16A7	XLOC_166259	541	coding	noncoding	noncoding	noncoding
MSTRG.158803.1		XLOC_166271	299	coding	noncoding	noncoding	noncoding
MSTRG.158831.1		XLOC_166285	296	coding	noncoding	noncoding	noncoding
MSTRG.158838.1		XLOC_166288	298	coding	noncoding	noncoding	noncoding
MSTRG.158845.1		XLOC_166292	425	coding	coding	noncoding	noncoding
MSTRG.158846.1		XLOC_166293	255	coding	coding	noncoding	noncoding
MSTRG.158850.1		XLOC_166296	267	coding	noncoding	noncoding	noncoding
MSTRG.158856.1		XLOC_166300	254	coding	noncoding	noncoding	noncoding
MSTRG.158857.1		XLOC_166301	202	coding	noncoding	noncoding	noncoding
MSTRG.158861.1		XLOC_166303	249	coding	coding	noncoding	noncoding
MSTRG.158906.1		XLOC_166324	233	coding	noncoding	noncoding	noncoding
MSTRG.158919.1		XLOC_166328	401	coding	coding	noncoding	noncoding
MSTRG.158924.1		XLOC_166331	268	coding	noncoding	noncoding	noncoding
MSTRG.158930.1		XLOC_166333	269	coding	noncoding	noncoding	noncoding
MSTRG.158931.1		XLOC_166334	289	coding	noncoding	noncoding	noncoding
MSTRG.159066.1		XLOC_166365	284	coding	noncoding	noncoding	noncoding
MSTRG.159067.1	C12orf56	XLOC_166366	256	coding	noncoding	noncoding	noncoding
MSTRG.159075.1	C12orf56	XLOC_166369	301	coding	noncoding	noncoding	noncoding
MSTRG.159070.1		XLOC_166372	261	coding	noncoding	noncoding	noncoding
NR_158985.1	HMGA2-AS1	XLOC_166422	1185	coding	noncoding	coding	coding
MSTRG.159177.1		XLOC_166427	298	coding	noncoding	noncoding	noncoding
MSTRG.159392.1	GRIP1	XLOC_166453	209	coding	noncoding	noncoding	noncoding
MSTRG.159414.1	GRIP1	XLOC_166457	301	coding	noncoding	noncoding	noncoding
MSTRG.159418.1	GRIP1	XLOC_166459	311	coding	noncoding	noncoding	noncoding
MSTRG.159276.1		XLOC_166467	254	coding	noncoding	noncoding	noncoding
MSTRG.159285.1		XLOC_166474	334	coding	noncoding	noncoding	noncoding
MSTRG.159559.1	LOC105369818	XLOC_166524	239	coding	noncoding	noncoding	noncoding
NM_001354969.1	MDM1	XLOC_166525	3032	coding	coding	coding	coding
MSTRG.159608.1	CPM	XLOC_166566	287	coding	noncoding	noncoding	noncoding
MSTRG.159534.1		XLOC_166568	300	coding	noncoding	noncoding	noncoding
MSTRG.159542.1		XLOC_166572	277	coding	noncoding	noncoding	noncoding
MSTRG.159545.1		XLOC_166573	243	coding	noncoding	noncoding	noncoding

MSTRG.159546.1		XLOC_166574	289 coding	noncoding	noncoding	noncoding
MSTRG.159724.1		XLOC_166615	211 coding	noncoding	noncoding	noncoding
MSTRG.159727.1		XLOC_166616	285 coding	noncoding	noncoding	noncoding
MSTRG.159815.1	PTPRR	XLOC_166652	284 coding	noncoding	noncoding	noncoding
MSTRG.159818.1	PTPRR	XLOC_166654	224 coding	noncoding	noncoding	noncoding
MSTRG.159836.1		XLOC_166674	297 coding	noncoding	noncoding	noncoding
MSTRG.159838.1		XLOC_166675	480 coding	noncoding	noncoding	noncoding
MSTRG.159881.1	LGR5	XLOC_166688	217 coding	noncoding	noncoding	noncoding
MSTRG.159890.1		XLOC_166710	284 coding	noncoding	noncoding	noncoding
MSTRG.159928.1		XLOC_166722	388 coding	noncoding	noncoding	coding
MSTRG.159975.1	TRHDE	XLOC_166723	291 coding	noncoding	noncoding	noncoding
MSTRG.159977.1	TRHDE	XLOC_166724	212 coding	coding	noncoding	noncoding
MSTRG.159996.1	TRHDE	XLOC_166735	241 coding	noncoding	noncoding	noncoding
MSTRG.159971.1		XLOC_166749	239 coding	noncoding	noncoding	noncoding
MSTRG.160006.1	LINC02444	XLOC_166752	225 coding	noncoding	noncoding	noncoding
MSTRG.160018.1		XLOC_166758	292 coding	noncoding	noncoding	noncoding
MSTRG.160030.1		XLOC_166764	212 coding	noncoding	noncoding	noncoding
MSTRG.160046.1	LOC100507377	XLOC_166767	292 coding	noncoding	noncoding	noncoding
MSTRG.160062.1		XLOC_166786	296 coding	noncoding	noncoding	noncoding
MSTRG.160108.1	GLIPR1L1	XLOC_166805	288 coding	noncoding	noncoding	noncoding
MSTRG.160116.1	GLIPR1L1	XLOC_166806	269 coding	noncoding	noncoding	noncoding
MSTRG.160147.1	LOC105369844	XLOC_166825	230 coding	noncoding	noncoding	noncoding
MSTRG.160164.1		XLOC_166844	312 coding	noncoding	noncoding	noncoding
MSTRG.160165.1		XLOC_166847	293 coding	noncoding	noncoding	noncoding
MSTRG.160201.1		XLOC_166915	259 coding	noncoding	noncoding	noncoding
MSTRG.160208.1		XLOC_166919	263 coding	noncoding	noncoding	noncoding
MSTRG.160212.1		XLOC_166922	213 coding	noncoding	noncoding	noncoding
MSTRG.160283.1		XLOC_166927	325 coding	noncoding	noncoding	noncoding
MSTRG.160358.1	NAV3	XLOC_166934	320 coding	noncoding	noncoding	noncoding
MSTRG.160403.1	SYT1	XLOC_166970	200 coding	noncoding	noncoding	noncoding
MSTRG.160462.1	OTOGL	XLOC_166994	303 coding	noncoding	noncoding	noncoding
MSTRG.160464.1	OTOGL	XLOC_166995	317 coding	noncoding	noncoding	noncoding
MSTRG.160655.1	PPFIA2	XLOC_167033	203 coding	noncoding	noncoding	noncoding
MSTRG.160661.1	PPFIA2	XLOC_167037	241 coding	noncoding	noncoding	noncoding
MSTRG.160662.1	PPFIA2	XLOC_167038	337 coding	noncoding	noncoding	noncoding
MSTRG.160500.1		XLOC_167040	259 coding	noncoding	noncoding	noncoding
MSTRG.160508.1		XLOC_167045	294 coding	noncoding	noncoding	noncoding
MSTRG.160526.1		XLOC_167056	253 coding	noncoding	noncoding	noncoding
MSTRG.160536.1		XLOC_167059	471 coding	noncoding	noncoding	noncoding
MSTRG.160560.1		XLOC_167067	269 coding	noncoding	noncoding	noncoding
MSTRG.160573.1	LOC107984536	XLOC_167072	289 coding	noncoding	noncoding	noncoding
MSTRG.160588.1		XLOC_167080	221 coding	noncoding	noncoding	noncoding
MSTRG.160611.1	LOC102724680	XLOC_167089	306 coding	noncoding	noncoding	noncoding

MSTRG.160614.1		XLOC_167098	229 coding	noncoding	noncoding	noncoding
MSTRG.160695.1		XLOC_167114	234 coding	noncoding	noncoding	noncoding
NM_001351291.2	MGAT4C	XLOC_167123	26388 coding	coding	coding	coding
MSTRG.160747.1	MGAT4C	XLOC_167125	263 coding	noncoding	noncoding	noncoding
MSTRG.160759.1	MGAT4C	XLOC_167129	286 coding	noncoding	noncoding	noncoding
MSTRG.160704.1		XLOC_167140	236 coding	noncoding	noncoding	noncoding
MSTRG.160805.1		XLOC_167173	765 coding	noncoding	noncoding	noncoding
MSTRG.160812.1		XLOC_167177	293 coding	noncoding	noncoding	noncoding
MSTRG.160813.1		XLOC_167178	309 coding	noncoding	noncoding	noncoding
MSTRG.161251.1		XLOC_167199	237 coding	noncoding	noncoding	noncoding
MSTRG.161276.1		XLOC_167222	261 coding	noncoding	noncoding	noncoding
NR_037660.1	POC1B	XLOC_167223	3048 coding	coding	coding	coding
XM_024448993.1	ATP2B1	XLOC_167241	7106 coding	coding	coding	coding
MSTRG.161310.1	LOC107984543	XLOC_167245	356 coding	noncoding	noncoding	noncoding
MSTRG.160923.1	LOC105369895	XLOC_167279	226 coding	coding	noncoding	noncoding
NM_152638.4	CCER1	XLOC_167280	2962 coding	coding	coding	coding
MSTRG.160930.1		XLOC_167282	379 coding	noncoding	noncoding	noncoding
MSTRG.160956.1	LOC105369898	XLOC_167294	221 coding	noncoding	noncoding	noncoding
MSTRG.160959.1	LOC105369898	XLOC_167295	204 coding	noncoding	noncoding	noncoding
MSTRG.160960.1	LOC105369898	XLOC_167296	227 coding	noncoding	noncoding	noncoding
MSTRG.160984.1		XLOC_167312	286 coding	noncoding	noncoding	noncoding
MSTRG.161073.1	LINC01619	XLOC_167327	250 coding	noncoding	noncoding	noncoding
MSTRG.161006.1	LOC107984467	XLOC_167348	253 coding	noncoding	noncoding	noncoding
MSTRG.161123.1	EEA1	XLOC_167376	403 coding	noncoding	noncoding	noncoding
MSTRG.161125.1	EEA1	XLOC_167378	305 coding	coding	noncoding	noncoding
MSTRG.161152.1	LOC643339	XLOC_167394	256 coding	noncoding	noncoding	noncoding
MSTRG.161176.1	LOC643339	XLOC_167400	311 coding	noncoding	noncoding	noncoding
MSTRG.161204.1	SOCS2	XLOC_167416	227 coding	noncoding	noncoding	noncoding
MSTRG.161316.5	CRADD	XLOC_167421	487 coding	coding	noncoding	noncoding
MSTRG.161570.1	LOC102724960	XLOC_167456	208 coding	noncoding	noncoding	noncoding
MSTRG.161384.1		XLOC_167479	218 coding	noncoding	noncoding	noncoding
MSTRG.161396.1	NDUFA12	XLOC_167482	282 coding	noncoding	noncoding	noncoding
XR_001749267.1	LOC107984545	XLOC_167494	2092 coding	noncoding	noncoding	noncoding
MSTRG.161496.1	USP44	XLOC_167502	415 coding	noncoding	noncoding	noncoding
MSTRG.161492.1		XLOC_167505	239 coding	noncoding	noncoding	noncoding
MSTRG.161525.1		XLOC_167507	202 coding	noncoding	noncoding	noncoding
MSTRG.161509.1		XLOC_167508	307 coding	noncoding	noncoding	noncoding
MSTRG.161531.1	LINC02410	XLOC_167518	246 coding	noncoding	noncoding	noncoding
MSTRG.161538.1	CCDC38	XLOC_167524	217 coding	noncoding	noncoding	noncoding
MSTRG.161575.1		XLOC_167537	309 coding	noncoding	noncoding	noncoding
MSTRG.161711.1		XLOC_167559	227 coding	noncoding	noncoding	noncoding
MSTRG.161712.1		XLOC_167560	257 coding	noncoding	noncoding	noncoding
MSTRG.161615.1	CFAP54	XLOC_167574	358 coding	noncoding	noncoding	noncoding

MSTRG.161598.1		XLOC_167580	287	coding	noncoding	noncoding	noncoding
MSTRG.161633.1	LOC105369928	XLOC_167589	237	coding	noncoding	noncoding	noncoding
MSTRG.161635.1		XLOC_167594	251	coding	noncoding	noncoding	noncoding
MSTRG.161665.1	RMST	XLOC_167604	208	coding	noncoding	noncoding	noncoding
MSTRG.161673.1		XLOC_167611	368	coding	coding	noncoding	noncoding
MSTRG.161734.1	TMPO	XLOC_167621	407	coding	noncoding	noncoding	noncoding
MSTRG.161845.5	ANKS1B	XLOC_167631	11930	coding	coding	coding	coding
MSTRG.161845.9	APAF1	XLOC_167631	5920	coding	coding	noncoding	noncoding
MSTRG.161845.10	ANKS1B	XLOC_167631	9901	coding	coding	coding	coding
XM_017019049.1	UHRF1BP1L	XLOC_167633	3741	coding	coding	coding	coding
NR_036632.1	GOLGA2P5	XLOC_167648	2337	coding	coding	coding	coding
MSTRG.161792.1	SLC17A8	XLOC_167655	281	coding	noncoding	noncoding	noncoding
MSTRG.161794.1	SLC17A8	XLOC_167656	227	coding	noncoding	noncoding	noncoding
MSTRG.162010.6	CHPT1	XLOC_167678	1627	coding	coding	coding	noncoding
MSTRG.161946.1	PAH	XLOC_167735	235	coding	coding	noncoding	noncoding
MSTRG.161950.1	PAH	XLOC_167737	291	coding	noncoding	noncoding	noncoding
MSTRG.161940.1		XLOC_167739	232	coding	noncoding	noncoding	noncoding
MSTRG.162052.1	C12orf42	XLOC_167745	301	coding	coding	noncoding	noncoding
MSTRG.162088.1	C12orf42	XLOC_167754	280	coding	noncoding	noncoding	noncoding
MSTRG.162093.1	C12orf42	XLOC_167756	272	coding	coding	noncoding	noncoding
MSTRG.162372.1	TXNRD1	XLOC_167778	277	coding	noncoding	noncoding	noncoding
MSTRG.162403.14	CHST11	XLOC_167793	13472	coding	noncoding	coding	noncoding
MSTRG.162403.29	CHST11	XLOC_167793	3026	coding	coding	coding	noncoding
MSTRG.162403.32	CHST11	XLOC_167793	2580	coding	noncoding	coding	noncoding
MSTRG.162132.1		XLOC_167838	268	coding	noncoding	noncoding	noncoding
MSTRG.162237.1	LOC105369957	XLOC_167867	245	coding	noncoding	noncoding	noncoding
MSTRG.162298.1	NUAK1	XLOC_167910	204	coding	noncoding	noncoding	noncoding
MSTRG.162316.1		XLOC_167916	265	coding	coding	noncoding	noncoding
MSTRG.162317.1		XLOC_167917	262	coding	noncoding	noncoding	noncoding
MSTRG.162339.1		XLOC_167922	476	coding	noncoding	noncoding	noncoding
MSTRG.162360.1		XLOC_167942	228	coding	noncoding	noncoding	noncoding
MSTRG.162559.1		XLOC_167976	348	coding	noncoding	noncoding	noncoding
MSTRG.162567.1		XLOC_167981	279	coding	noncoding	noncoding	noncoding
XM_017019123.2	CORO1C	XLOC_168014	4300	coding	coding	coding	coding
MSTRG.162633.1		XLOC_168015	229	coding	noncoding	noncoding	noncoding
MSTRG.162617.1		XLOC_168023	241	coding	noncoding	noncoding	noncoding
MSTRG.162635.1	DAO	XLOC_168024	295	coding	noncoding	noncoding	noncoding
MSTRG.162690.1	USP30	XLOC_168029	272	coding	noncoding	noncoding	noncoding
MSTRG.162692.3	USP30	XLOC_168030	800	coding	noncoding	noncoding	noncoding
MSTRG.162711.1	ACACB	XLOC_168038	312	coding	noncoding	noncoding	noncoding
MSTRG.162698.1		XLOC_168059	260	coding	noncoding	noncoding	noncoding
MSTRG.162768.11	UBE3B	XLOC_168062	2956	coding	coding	coding	coding
MSTRG.162739.1		XLOC_168076	293	coding	noncoding	noncoding	noncoding

MSTRG.162740.1		XLOC_168077	311 coding	noncoding	noncoding	noncoding
MSTRG.162786.3	TCHP	XLOC_168087	354 coding	noncoding	noncoding	noncoding
MSTRG.162805.1		XLOC_168107	244 coding	noncoding	noncoding	noncoding
MSTRG.162825.1	IFT81	XLOC_168111	290 coding	noncoding	noncoding	noncoding
MSTRG.162842.1	ANAPC7	XLOC_168117	292 coding	noncoding	noncoding	noncoding
MSTRG.162844.1		XLOC_168131	301 coding	noncoding	noncoding	noncoding
XM_005253948.2	HVCN1	XLOC_168140	1721 coding	coding	coding	coding
MSTRG.162867.1	CCDC63	XLOC_168147	224 coding	noncoding	noncoding	noncoding
MSTRG.162880.1		XLOC_168167	257 coding	noncoding	noncoding	noncoding
NM_002973.4	ATXN2	XLOC_168169	4341 coding	coding	coding	coding
XM_017020192.2	TMEM116	XLOC_168180	3074 coding	noncoding	coding	coding
MSTRG.163019.1	HECTD4	XLOC_168200	309 coding	noncoding	noncoding	noncoding
MSTRG.163022.1	HECTD4	XLOC_168203	286 coding	noncoding	noncoding	noncoding
MSTRG.162955.1		XLOC_168206	326 coding	noncoding	noncoding	noncoding
NM_001320141.2	RPL6	XLOC_168207	1238 coding	coding	coding	coding
MSTRG.162962.4	PTPN11	XLOC_168208	3858 coding	coding	noncoding	noncoding
MSTRG.162971.1	RPH3A	XLOC_168212	300 coding	noncoding	noncoding	noncoding
MSTRG.163096.1	DDX54	XLOC_168228	205 coding	noncoding	noncoding	noncoding
MSTRG.163044.1		XLOC_168241	236 coding	noncoding	noncoding	noncoding
MSTRG.163053.1		XLOC_168245	634 coding	noncoding	noncoding	noncoding
MSTRG.163084.1		XLOC_168265	294 coding	noncoding	noncoding	noncoding
MSTRG.163117.1	LOC105370000	XLOC_168273	231 coding	noncoding	noncoding	noncoding
MSTRG.163123.1	LOC107984437	XLOC_168277	245 coding	noncoding	noncoding	noncoding
MSTRG.163134.1		XLOC_168284	296 coding	coding	noncoding	noncoding
MSTRG.163153.1		XLOC_168293	272 coding	noncoding	noncoding	noncoding
MSTRG.163195.1	LOC105370003	XLOC_168307	292 coding	noncoding	noncoding	noncoding
MSTRG.163206.1		XLOC_168339	242 coding	noncoding	noncoding	noncoding
MSTRG.163209.1		XLOC_168342	244 coding	noncoding	noncoding	noncoding
MSTRG.163210.1		XLOC_168343	263 coding	noncoding	noncoding	noncoding
MSTRG.163212.1		XLOC_168347	237 coding	noncoding	noncoding	noncoding
NM_015002.3	FBXO21	XLOC_168385	5976 coding	coding	coding	coding
MSTRG.163313.1	FBXO21	XLOC_168387	308 coding	noncoding	noncoding	noncoding
MSTRG.163254.1		XLOC_168390	253 coding	noncoding	noncoding	noncoding
NM_001204218.1	NOS1	XLOC_168391	12285 coding	coding	coding	coding
MSTRG.163266.1	NOS1	XLOC_168394	270 coding	noncoding	noncoding	noncoding
MSTRG.163258.1		XLOC_168398	246 coding	noncoding	noncoding	noncoding
XM_011538225.3	KSR2	XLOC_168399	17784 coding	coding	coding	coding
MSTRG.163330.1	KSR2	XLOC_168409	212 coding	noncoding	noncoding	noncoding
MSTRG.163336.1	KSR2	XLOC_168412	214 coding	noncoding	noncoding	noncoding
XM_017019410.2	TAOK3	XLOC_168421	4583 coding	coding	coding	coding
MSTRG.163455.1	TAOK3	XLOC_168423	220 coding	noncoding	noncoding	noncoding
MSTRG.163460.1	TAOK3	XLOC_168428	221 coding	noncoding	noncoding	noncoding
MSTRG.163376.1		XLOC_168449	1032 coding	noncoding	noncoding	noncoding

MSTRG.163381.1		XLOC_168453	326 coding	noncoding	noncoding	noncoding
MSTRG.163383.1		XLOC_168455	238 coding	noncoding	noncoding	noncoding
MSTRG.163391.1	LINC02423	XLOC_168460	264 coding	noncoding	noncoding	noncoding
XR_001749345.1	LOC107984440	XLOC_168476	3331 coding	noncoding	noncoding	noncoding
MSTRG.163478.1	CIT	XLOC_168484	268 coding	noncoding	noncoding	noncoding
MSTRG.163449.1	LOC112268087	XLOC_168492	259 coding	noncoding	noncoding	noncoding
MSTRG.163534.2	GCN1	XLOC_168525	283 coding	noncoding	noncoding	noncoding
MSTRG.163537.1	GCN1	XLOC_168528	223 coding	noncoding	noncoding	noncoding
MSTRG.163529.1	SIRT4	XLOC_168536	289 coding	noncoding	noncoding	noncoding
NM_016399.3	TRIAP1	XLOC_168540	1151 coding	coding	coding	noncoding
MSTRG.163560.1		XLOC_168547	713 coding	noncoding	noncoding	noncoding
MSTRG.163578.1		XLOC_168575	286 coding	noncoding	noncoding	noncoding
MSTRG.163580.1		XLOC_168576	224 coding	noncoding	noncoding	noncoding
MSTRG.163584.1	HNF1A	XLOC_168579	249 coding	noncoding	noncoding	noncoding
MSTRG.163631.1	LOC105370030	XLOC_168585	269 coding	noncoding	noncoding	noncoding
MSTRG.163625.1	ANAPC5	XLOC_168597	205 coding	noncoding	noncoding	noncoding
MSTRG.163657.1	LOC105370034	XLOC_168606	254 coding	noncoding	noncoding	noncoding
MSTRG.163673.1	TMEM120B	XLOC_168607	461 coding	noncoding	noncoding	noncoding
MSTRG.163685.1		XLOC_168622	265 coding	noncoding	noncoding	noncoding
MSTRG.163707.1	LRRC43	XLOC_168631	242 coding	noncoding	noncoding	noncoding
MSTRG.163732.1	CLIP1	XLOC_168643	321 coding	noncoding	noncoding	noncoding
MSTRG.163723.1	ZCCHC8	XLOC_168645	304 coding	noncoding	noncoding	noncoding
MSTRG.163735.1		XLOC_168652	288 coding	noncoding	noncoding	noncoding
MSTRG.163736.1		XLOC_168653	237 coding	noncoding	noncoding	noncoding
MSTRG.163738.1		XLOC_168655	264 coding	noncoding	noncoding	noncoding
MSTRG.163748.1	CCDC62	XLOC_168663	253 coding	noncoding	noncoding	noncoding
MSTRG.163757.1	HIP1R	XLOC_168666	2815 coding	coding	coding	coding
XM_011538098.2	ABCB9	XLOC_168674	4395 coding	coding	coding	coding
MSTRG.163777.1	ABCB9	XLOC_168675	306 coding	noncoding	noncoding	noncoding
MSTRG.163802.21	MPHOSPH9	XLOC_168683	596 coding	noncoding	noncoding	coding
MSTRG.163909.1	ZNF664-RFLNA	XLOC_168720	249 coding	noncoding	noncoding	noncoding
MSTRG.163915.1	ZNF664-RFLNA	XLOC_168724	232 coding	noncoding	noncoding	noncoding
MSTRG.163876.1	SCARB1	XLOC_168749	590 coding	noncoding	noncoding	noncoding
MSTRG.163920.1		XLOC_168760	265 coding	noncoding	noncoding	noncoding
MSTRG.163999.1	BRI3BP	XLOC_168763	296 coding	noncoding	noncoding	noncoding
MSTRG.164005.1	AACS	XLOC_168766	326 coding	noncoding	noncoding	noncoding
MSTRG.164023.1	TMEM132B	XLOC_168773	258 coding	noncoding	noncoding	noncoding
MSTRG.164034.1	TMEM132B	XLOC_168781	228 coding	noncoding	noncoding	noncoding
MSTRG.164048.1	LINC02826	XLOC_168791	299 coding	noncoding	noncoding	noncoding
MSTRG.164019.1		XLOC_168792	227 coding	noncoding	noncoding	noncoding
MSTRG.164082.1	LINC02405	XLOC_168820	358 coding	noncoding	noncoding	noncoding
MSTRG.164065.1	LOC105370064	XLOC_168824	228 coding	noncoding	noncoding	noncoding
MSTRG.164095.1		XLOC_168834	241 coding	noncoding	noncoding	noncoding

MSTRG.164100.1		XLOC_168836	296 coding	noncoding	noncoding	noncoding
MSTRG.164107.1		XLOC_168843	201 coding	noncoding	noncoding	noncoding
MSTRG.164109.1		XLOC_168846	341 coding	noncoding	noncoding	noncoding
MSTRG.164148.1	LINC02369	XLOC_168853	291 coding	noncoding	noncoding	noncoding
MSTRG.164161.1	TMEM132C	XLOC_168857	301 coding	noncoding	noncoding	noncoding
MSTRG.164162.1	TMEM132C	XLOC_168858	242 coding	noncoding	noncoding	noncoding
MSTRG.164387.1	TMEM132D	XLOC_168912	217 coding	coding	noncoding	noncoding
MSTRG.164202.1		XLOC_168922	223 coding	coding	noncoding	noncoding
XR_001749407.2	LOC101929974	XLOC_168970	6144 coding	noncoding	noncoding	noncoding
MSTRG.164320.1	ULK1	XLOC_168992	265 coding	noncoding	noncoding	noncoding
MSTRG.164413.1	LOC105370092	XLOC_169014	226 coding	noncoding	noncoding	noncoding
MSTRG.164483.1	POLE	XLOC_169054	242 coding	noncoding	noncoding	noncoding
MSTRG.164520.1	GOLGA3	XLOC_169066	259 coding	noncoding	noncoding	noncoding
MSTRG.164527.1	CHFR	XLOC_169068	463 coding	noncoding	noncoding	noncoding
MSTRG.164528.1	CHFR	XLOC_169069	267 coding	noncoding	noncoding	noncoding
NM_001164715.2	ZNF605	XLOC_169074	9312 coding	coding	coding	coding
MSTRG.164595.1		XLOC_169118	288 coding	noncoding	noncoding	noncoding
MSTRG.164759.18	PSPC1	XLOC_169127	1826 coding	coding	coding	noncoding
MSTRG.164780.1		XLOC_169135	286 coding	noncoding	noncoding	noncoding
MSTRG.164783.1		XLOC_169136	266 coding	noncoding	noncoding	noncoding
MSTRG.164784.1		XLOC_169137	537 coding	noncoding	noncoding	noncoding
XM_017020733.2	ZMYM2	XLOC_169138	4798 coding	coding	coding	coding
MSTRG.164625.1	LOC105370102	XLOC_169163	212 coding	noncoding	noncoding	noncoding
MSTRG.164668.1		XLOC_169187	234 coding	noncoding	noncoding	noncoding
MSTRG.164675.1	LOC105370104	XLOC_169192	303 coding	noncoding	noncoding	noncoding
MSTRG.164713.4	SKA3	XLOC_169199	1086 coding	noncoding	noncoding	noncoding
MSTRG.164728.12	MIPEPP3	XLOC_169205	4445 coding	coding	coding	noncoding
MSTRG.164728.22	MIPEPP3	XLOC_169205	1335 coding	coding	noncoding	noncoding
MSTRG.164751.1	MIPEPP3	XLOC_169221	232 coding	noncoding	noncoding	noncoding
MSTRG.164842.1		XLOC_169259	376 coding	noncoding	noncoding	noncoding
MSTRG.164844.1		XLOC_169260	243 coding	noncoding	noncoding	noncoding
MSTRG.164858.1	LINC00621	XLOC_169268	247 coding	noncoding	noncoding	noncoding
MSTRG.164861.1		XLOC_169271	278 coding	noncoding	noncoding	noncoding
MSTRG.164864.1		XLOC_169274	226 coding	noncoding	noncoding	noncoding
MSTRG.164890.1	SACS	XLOC_169282	288 coding	noncoding	noncoding	noncoding
NR_038995.1	LINC00327	XLOC_169286	2087 coding	noncoding	coding	noncoding
MSTRG.164880.1		XLOC_169288	245 coding	noncoding	noncoding	noncoding
MSTRG.164937.1	SPATA13	XLOC_169307	240 coding	noncoding	noncoding	noncoding
NR_002815.2	TPTE2P6	XLOC_169329	2716 coding	coding	noncoding	noncoding
MSTRG.165138.1		XLOC_169340	232 coding	noncoding	noncoding	noncoding
MSTRG.165147.1		XLOC_169360	278 coding	noncoding	noncoding	noncoding
MSTRG.165167.1	ATP8A2	XLOC_169365	272 coding	noncoding	noncoding	noncoding
MSTRG.165174.1	ATP8A2	XLOC_169368	286 coding	noncoding	noncoding	noncoding

MSTRG.165184.1	ATP8A2	XLOC_169371	230	coding	noncoding	noncoding	noncoding
MSTRG.165157.1	SHISA2	XLOC_169374	369	coding	noncoding	noncoding	noncoding
MSTRG.165192.1		XLOC_169385	649	coding	noncoding	noncoding	noncoding
MSTRG.165250.1	CDK8	XLOC_169389	503	coding	noncoding	noncoding	noncoding
MSTRG.165255.1	CDK8	XLOC_169393	260	coding	noncoding	noncoding	noncoding
MSTRG.165210.1		XLOC_169396	299	coding	noncoding	noncoding	noncoding
MSTRG.165267.1		XLOC_169429	241	coding	noncoding	noncoding	noncoding
MSTRG.165297.1		XLOC_169437	260	coding	noncoding	noncoding	noncoding
MSTRG.165310.1		XLOC_169439	276	coding	noncoding	noncoding	noncoding
MSTRG.165317.1		XLOC_169444	261	coding	noncoding	noncoding	noncoding
MSTRG.165345.1	URAD	XLOC_169469	207	coding	noncoding	noncoding	noncoding
MSTRG.165369.1		XLOC_169496	343	coding	noncoding	noncoding	noncoding
MSTRG.165409.1		XLOC_169511	243	coding	noncoding	coding	noncoding
MSTRG.165431.1	MTUS2	XLOC_169517	244	coding	coding	noncoding	noncoding
MSTRG.165441.1	MTUS2	XLOC_169522	255	coding	coding	noncoding	noncoding
MSTRG.165454.1	MTUS2	XLOC_169530	341	coding	noncoding	noncoding	noncoding
MSTRG.165492.1	LINC00297	XLOC_169566	297	coding	noncoding	noncoding	noncoding
MSTRG.165563.1		XLOC_169604	276	coding	noncoding	noncoding	noncoding
MSTRG.165582.1	HMGB1	XLOC_169615	290	coding	noncoding	noncoding	noncoding
MSTRG.165574.1		XLOC_169646	307	coding	noncoding	noncoding	noncoding
MSTRG.165658.1		XLOC_169655	255	coding	noncoding	noncoding	noncoding
MSTRG.165663.1		XLOC_169661	213	coding	noncoding	noncoding	noncoding
MSTRG.165733.1	LOC105370148	XLOC_169666	269	coding	noncoding	noncoding	noncoding
MSTRG.166316.1	FRY	XLOC_169776	283	coding	noncoding	noncoding	noncoding
MSTRG.165798.6	N4BP2L1	XLOC_169812	1188	coding	noncoding	noncoding	noncoding
MSTRG.165756.1		XLOC_169815	291	coding	noncoding	noncoding	noncoding
MSTRG.165763.1	LINC00423	XLOC_169840	393	coding	noncoding	noncoding	noncoding
MSTRG.165864.1	STARD13	XLOC_169856	313	coding	noncoding	noncoding	noncoding
MSTRG.165868.1	STARD13	XLOC_169860	227	coding	noncoding	noncoding	noncoding
MSTRG.165815.1		XLOC_169896	249	coding	noncoding	noncoding	noncoding
XM_011535173.3	RFC3	XLOC_169900	3791	coding	coding	coding	coding
MSTRG.165994.1	RFC3	XLOC_169904	362	coding	noncoding	noncoding	noncoding
MSTRG.165949.1	LINC00457	XLOC_169919	277	coding	noncoding	noncoding	noncoding
MSTRG.165930.1		XLOC_169922	260	coding	noncoding	noncoding	noncoding
MSTRG.165957.1		XLOC_169929	311	coding	noncoding	noncoding	noncoding
MSTRG.165961.1		XLOC_169931	209	coding	noncoding	noncoding	noncoding
MSTRG.165964.1		XLOC_169934	228	coding	noncoding	noncoding	noncoding
MSTRG.166135.1	NBEA	XLOC_169947	707	coding	noncoding	noncoding	noncoding
MSTRG.166162.1	NBEA	XLOC_169953	275	coding	noncoding	noncoding	noncoding
MSTRG.166172.1	NBEA	XLOC_169958	431	coding	noncoding	noncoding	noncoding
MSTRG.166030.1	DCLK1	XLOC_169966	201	coding	noncoding	noncoding	noncoding
MSTRG.166087.1	SMAD9	XLOC_169989	228	coding	noncoding	noncoding	noncoding
MSTRG.166089.1	SMAD9	XLOC_169991	280	coding	noncoding	noncoding	noncoding

MSTRG.166185.1		XLOC_170058	217 coding	coding	noncoding	noncoding
MSTRG.166189.1		XLOC_170060	279 coding	noncoding	noncoding	noncoding
NM_207361.6	FREM2	XLOC_170102	16122 coding	coding	noncoding	coding
MSTRG.166413.1		XLOC_170111	238 coding	noncoding	noncoding	noncoding
MSTRG.166425.1		XLOC_170130	241 coding	noncoding	noncoding	noncoding
MSTRG.166561.1	COG6	XLOC_170141	322 coding	noncoding	noncoding	noncoding
MSTRG.166563.1	COG6	XLOC_170142	259 coding	noncoding	noncoding	noncoding
MSTRG.166526.1		XLOC_170145	466 coding	noncoding	noncoding	noncoding
MSTRG.166525.1		XLOC_170146	314 coding	noncoding	noncoding	noncoding
MSTRG.166572.1		XLOC_170192	251 coding	noncoding	noncoding	noncoding
MSTRG.166575.1		XLOC_170194	331 coding	noncoding	noncoding	noncoding
MSTRG.166685.5	TPTE2P5	XLOC_170218	2602 coding	coding	coding	noncoding
MSTRG.166761.1	LOC101929140	XLOC_170246	268 coding	noncoding	noncoding	noncoding
MSTRG.166763.3	MTRF1	XLOC_170247	1478 coding	coding	noncoding	noncoding
MSTRG.166735.1		XLOC_170261	258 coding	noncoding	noncoding	noncoding
NM_001204504.2	DGKH	XLOC_170276	17344 coding	coding	coding	coding
MSTRG.166789.1	LINC02341	XLOC_170295	289 coding	noncoding	noncoding	noncoding
MSTRG.166807.1		XLOC_170308	233 coding	noncoding	noncoding	noncoding
MSTRG.166907.1		XLOC_170352	259 coding	noncoding	noncoding	noncoding
MSTRG.166948.1		XLOC_170361	291 coding	coding	noncoding	noncoding
MSTRG.167002.1		XLOC_170370	231 coding	noncoding	noncoding	noncoding
MSTRG.167003.1		XLOC_170371	232 coding	noncoding	noncoding	noncoding
MSTRG.167010.1	LINC00407	XLOC_170374	281 coding	noncoding	noncoding	noncoding
MSTRG.167017.1		XLOC_170379	317 coding	noncoding	noncoding	noncoding
MSTRG.167027.1	LOC105370187	XLOC_170381	339 coding	noncoding	noncoding	noncoding
XM_011535144.2	GPALPP1	XLOC_170392	4241 coding	coding	coding	coding
MSTRG.167033.1	LOC107984596	XLOC_170395	244 coding	noncoding	noncoding	noncoding
MSTRG.167031.1		XLOC_170397	398 coding	noncoding	noncoding	noncoding
MSTRG.167132.1	TPT1-AS1	XLOC_170415	218 coding	noncoding	noncoding	noncoding
MSTRG.167134.1	TPT1-AS1	XLOC_170417	309 coding	noncoding	noncoding	noncoding
MSTRG.167103.1	COG3	XLOC_170422	237 coding	noncoding	noncoding	noncoding
MSTRG.167076.1		XLOC_170428	317 coding	noncoding	noncoding	noncoding
MSTRG.167077.1		XLOC_170429	246 coding	noncoding	noncoding	noncoding
MSTRG.167294.1	LRCH1	XLOC_170478	262 coding	noncoding	noncoding	noncoding
MSTRG.167236.1		XLOC_170490	309 coding	noncoding	noncoding	noncoding
MSTRG.167269.1		XLOC_170504	257 coding	noncoding	noncoding	noncoding
MSTRG.167370.1	LOC105370202	XLOC_170597	258 coding	noncoding	noncoding	noncoding
MSTRG.167371.1	LOC105370202	XLOC_170598	268 coding	noncoding	noncoding	noncoding
MSTRG.167386.1		XLOC_170610	274 coding	noncoding	noncoding	noncoding
XM_017020442.2	FNDC3A	XLOC_170616	6138 coding	coding	coding	coding
NR_135322.1	PHF11	XLOC_170663	1593 coding	coding	coding	coding
NR_135323.1	PHF11	XLOC_170663	1692 coding	coding	coding	coding
MSTRG.167510.1		XLOC_170676	242 coding	noncoding	noncoding	noncoding

MSTRG.167519.1		XLOC_170682	237 coding	noncoding	noncoding	noncoding
MSTRG.168771.4	TRIM13	XLOC_170686	1923 coding	noncoding	noncoding	noncoding
MSTRG.168771.11	TRIM13	XLOC_170686	4102 coding	noncoding	noncoding	noncoding
MSTRG.168771.14	TRIM13	XLOC_170686	9023 coding	coding	coding	coding
NM_005798.5	TRIM13	XLOC_170686	6720 coding	coding	coding	coding
MSTRG.168771.30	DLEU2	XLOC_170686	749 coding	noncoding	noncoding	noncoding
MSTRG.168853.1	DLEU1	XLOC_170697	315 coding	noncoding	noncoding	noncoding
MSTRG.169000.1	RNASEH2B	XLOC_170733	355 coding	noncoding	noncoding	noncoding
XM_011534978.2	FAM124A	XLOC_170739	4926 coding	coding	coding	coding
MSTRG.167572.1		XLOC_170750	282 coding	noncoding	noncoding	noncoding
MSTRG.167602.1		XLOC_170761	280 coding	noncoding	noncoding	noncoding
MSTRG.167620.1	TPTE2P2	XLOC_170789	241 coding	noncoding	noncoding	noncoding
MSTRG.167616.1		XLOC_170792	254 coding	noncoding	noncoding	noncoding
MSTRG.167617.1		XLOC_170793	277 coding	noncoding	noncoding	noncoding
MSTRG.167655.4	VPS36	XLOC_170795	1189 coding	coding	coding	noncoding
MSTRG.167698.1	HNRNPA1L2	XLOC_170801	269 coding	noncoding	noncoding	noncoding
MSTRG.167690.1		XLOC_170823	259 coding	noncoding	noncoding	noncoding
MSTRG.167721.1		XLOC_170828	488 coding	noncoding	coding	noncoding
MSTRG.167741.1		XLOC_170843	261 coding	noncoding	noncoding	noncoding
MSTRG.167752.1		XLOC_170850	216 coding	noncoding	noncoding	noncoding
MSTRG.167760.1		XLOC_170853	228 coding	noncoding	noncoding	noncoding
MSTRG.167769.1		XLOC_170858	293 coding	coding	noncoding	noncoding
MSTRG.167789.1		XLOC_170868	237 coding	noncoding	noncoding	noncoding
MSTRG.167802.1		XLOC_170877	249 coding	noncoding	noncoding	noncoding
MSTRG.167833.1	LOC105370214	XLOC_170881	289 coding	noncoding	noncoding	noncoding
MSTRG.167838.1	LOC105370214	XLOC_170883	317 coding	noncoding	noncoding	noncoding
MSTRG.167818.1		XLOC_170893	436 coding	noncoding	noncoding	noncoding
MSTRG.167821.1		XLOC_170894	254 coding	noncoding	noncoding	noncoding
MSTRG.167876.1		XLOC_170908	441 coding	coding	noncoding	noncoding
MSTRG.167882.1		XLOC_170913	293 coding	noncoding	noncoding	noncoding
MSTRG.167884.1		XLOC_170914	281 coding	noncoding	noncoding	noncoding
MSTRG.167909.1		XLOC_170925	274 coding	noncoding	noncoding	noncoding
MSTRG.167951.1		XLOC_170954	256 coding	noncoding	noncoding	noncoding
MSTRG.167975.1	LOC107984625	XLOC_170962	226 coding	noncoding	noncoding	noncoding
MSTRG.167977.1	LOC107984625	XLOC_170964	292 coding	noncoding	noncoding	noncoding
MSTRG.167986.1	LINC00434	XLOC_170990	259 coding	noncoding	noncoding	noncoding
MSTRG.167993.1	LOC105370228	XLOC_170994	253 coding	noncoding	noncoding	noncoding
MSTRG.167989.1		XLOC_170996	273 coding	noncoding	noncoding	noncoding
MSTRG.168034.1		XLOC_171009	310 coding	noncoding	noncoding	noncoding
MSTRG.168037.1		XLOC_171011	424 coding	noncoding	noncoding	noncoding
MSTRG.168047.1	LINC01442	XLOC_171014	287 coding	noncoding	noncoding	noncoding
MSTRG.168065.1		XLOC_171026	283 coding	noncoding	noncoding	noncoding
MSTRG.168074.1		XLOC_171030	287 coding	noncoding	noncoding	noncoding

MSTRG.168085.1		XLOC_171037	250 coding	noncoding	noncoding	noncoding
NR_002171.2	OR7E156P	XLOC_171062	2647 coding	coding	noncoding	noncoding
MSTRG.168129.1		XLOC_171064	213 coding	noncoding	noncoding	noncoding
MSTRG.168145.1		XLOC_171069	294 coding	noncoding	noncoding	noncoding
MSTRG.168157.1		XLOC_171074	863 coding	noncoding	noncoding	noncoding
MSTRG.168174.1		XLOC_171083	281 coding	noncoding	noncoding	noncoding
MSTRG.168190.1		XLOC_171091	282 coding	coding	noncoding	noncoding
MSTRG.168191.1		XLOC_171092	296 coding	noncoding	noncoding	noncoding
MSTRG.168211.1	LOC105370245	XLOC_171101	229 coding	noncoding	noncoding	noncoding
MSTRG.168234.1		XLOC_171140	261 coding	coding	noncoding	noncoding
MSTRG.168239.1	LOC105370248	XLOC_171143	252 coding	coding	noncoding	noncoding
MSTRG.168250.1		XLOC_171152	247 coding	noncoding	noncoding	noncoding
MSTRG.168253.1		XLOC_171154	262 coding	noncoding	noncoding	noncoding
MSTRG.168255.1		XLOC_171155	341 coding	coding	coding	noncoding
MSTRG.168284.1		XLOC_171170	235 coding	noncoding	noncoding	noncoding
MSTRG.168295.1		XLOC_171175	238 coding	noncoding	noncoding	noncoding
MSTRG.168304.1		XLOC_171181	270 coding	noncoding	noncoding	noncoding
MSTRG.168312.1		XLOC_171193	256 coding	noncoding	noncoding	noncoding
MSTRG.168376.1		XLOC_171215	310 coding	coding	noncoding	noncoding
MSTRG.168435.1		XLOC_171221	285 coding	coding	noncoding	noncoding
MSTRG.168447.1		XLOC_171308	335 coding	noncoding	noncoding	noncoding
MSTRG.168458.1		XLOC_171317	361 coding	noncoding	noncoding	noncoding
MSTRG.168466.1		XLOC_171321	234 coding	noncoding	noncoding	noncoding
NM_024808.5	BORA	XLOC_171331	2760 coding	coding	coding	coding
MSTRG.168761.1	PIBF1	XLOC_171347	216 coding	noncoding	noncoding	noncoding
MSTRG.168482.1		XLOC_171352	234 coding	noncoding	noncoding	noncoding
MSTRG.168483.1		XLOC_171353	479 coding	noncoding	noncoding	noncoding
MSTRG.168484.1		XLOC_171354	232 coding	noncoding	noncoding	noncoding
MSTRG.168492.1		XLOC_171357	297 coding	noncoding	noncoding	noncoding
MSTRG.168505.1		XLOC_171362	269 coding	noncoding	noncoding	noncoding
MSTRG.169029.9	KLF12	XLOC_171380	2283 coding	noncoding	noncoding	coding
MSTRG.169096.1	KLF12	XLOC_171384	300 coding	noncoding	noncoding	noncoding
MSTRG.168662.1		XLOC_171455	312 coding	noncoding	noncoding	noncoding
MSTRG.169454.4	MYCBP2-AS1	XLOC_171584	15103 coding	coding	coding	coding
MSTRG.169454.19	MYCBP2-AS1	XLOC_171584	7745 coding	coding	noncoding	coding
MSTRG.169277.1	LOC100129307	XLOC_171594	313 coding	noncoding	noncoding	noncoding
MSTRG.169281.1		XLOC_171596	341 coding	noncoding	noncoding	noncoding
MSTRG.169307.1	LOC112268121	XLOC_171605	219 coding	noncoding	noncoding	noncoding
MSTRG.169323.1		XLOC_171618	257 coding	noncoding	noncoding	noncoding
MSTRG.169321.1		XLOC_171619	267 coding	noncoding	noncoding	noncoding
MSTRG.169325.1		XLOC_171620	257 coding	noncoding	noncoding	noncoding
MSTRG.169353.1		XLOC_171625	261 coding	noncoding	noncoding	noncoding
MSTRG.169364.1		XLOC_171641	229 coding	noncoding	noncoding	noncoding

MSTRG.169393.1		XLOC_171657	230 coding	noncoding	noncoding	noncoding
MSTRG.169399.1	LOC101927238	XLOC_171658	307 coding	noncoding	noncoding	noncoding
MSTRG.169401.1		XLOC_171660	256 coding	noncoding	noncoding	noncoding
MSTRG.169412.1		XLOC_171667	291 coding	noncoding	noncoding	noncoding
MSTRG.169590.1		XLOC_171688	243 coding	noncoding	noncoding	noncoding
MSTRG.169608.1		XLOC_171695	269 coding	noncoding	noncoding	noncoding
MSTRG.169625.1	LOC105370284	XLOC_171703	229 coding	noncoding	noncoding	noncoding
MSTRG.169627.1	LOC105370284	XLOC_171704	236 coding	noncoding	noncoding	noncoding
MSTRG.169628.1		XLOC_171706	299 coding	noncoding	noncoding	noncoding
MSTRG.169630.1		XLOC_171708	267 coding	noncoding	noncoding	noncoding
MSTRG.169655.1		XLOC_171719	357 coding	noncoding	noncoding	noncoding
MSTRG.169662.1		XLOC_171724	392 coding	noncoding	noncoding	noncoding
MSTRG.169665.1		XLOC_171726	255 coding	noncoding	noncoding	noncoding
MSTRG.169668.1		XLOC_171728	229 coding	noncoding	noncoding	noncoding
MSTRG.169690.1		XLOC_171740	300 coding	noncoding	noncoding	noncoding
MSTRG.169702.1		XLOC_171746	274 coding	coding	noncoding	noncoding
MSTRG.169727.1		XLOC_171767	239 coding	noncoding	noncoding	noncoding
MSTRG.169737.1	LINC00351	XLOC_171770	309 coding	noncoding	noncoding	noncoding
MSTRG.169732.1		XLOC_171777	407 coding	noncoding	noncoding	noncoding
MSTRG.169795.1		XLOC_171784	308 coding	noncoding	noncoding	noncoding
MSTRG.169805.1	LOC105370299	XLOC_171788	225 coding	coding	noncoding	noncoding
XM_005254038.5	SLITRK5	XLOC_171815	5217 coding	coding	coding	coding
MSTRG.169889.1		XLOC_171819	236 coding	noncoding	noncoding	noncoding
MSTRG.169895.1		XLOC_171832	256 coding	noncoding	noncoding	noncoding
MSTRG.169923.1		XLOC_171842	231 coding	noncoding	noncoding	noncoding
MSTRG.169927.1		XLOC_171843	258 coding	noncoding	noncoding	noncoding
MSTRG.169956.1		XLOC_171863	409 coding	noncoding	noncoding	noncoding
MSTRG.169974.1		XLOC_171871	793 coding	noncoding	noncoding	noncoding
MSTRG.169997.1		XLOC_171884	267 coding	coding	noncoding	noncoding
MSTRG.170068.1	GPC5	XLOC_171910	285 coding	noncoding	noncoding	noncoding
MSTRG.170073.1	GPC5	XLOC_171914	235 coding	noncoding	noncoding	noncoding
MSTRG.170085.1	GPC5	XLOC_171919	355 coding	noncoding	noncoding	noncoding
MSTRG.170040.1		XLOC_171939	250 coding	noncoding	noncoding	noncoding
MSTRG.170043.1		XLOC_171942	322 coding	noncoding	noncoding	noncoding
MSTRG.170140.1	GPC6	XLOC_171950	379 coding	noncoding	noncoding	noncoding
MSTRG.170147.1	GPC6	XLOC_171953	302 coding	noncoding	noncoding	noncoding
MSTRG.170196.1		XLOC_171985	296 coding	noncoding	noncoding	noncoding
MSTRG.170213.1		XLOC_171996	281 coding	noncoding	noncoding	noncoding
MSTRG.170216.1		XLOC_172006	276 coding	noncoding	noncoding	noncoding
XM_011521076.2	HS6ST3	XLOC_172041	4082 coding	coding	coding	coding
XM_017020543.2	HS6ST3	XLOC_172041	11757 coding	coding	coding	coding
MSTRG.170286.1	HS6ST3	XLOC_172044	215 coding	noncoding	noncoding	noncoding
MSTRG.170294.1	HS6ST3	XLOC_172050	296 coding	noncoding	noncoding	noncoding

MSTRG.170300.1	HS6ST3	XLOC_172053	248	coding	noncoding	noncoding	noncoding
MSTRG.170339.1		XLOC_172085	382	coding	noncoding	noncoding	noncoding
MSTRG.170371.1		XLOC_172102	269	coding	noncoding	noncoding	noncoding
MSTRG.170397.1		XLOC_172106	290	coding	noncoding	noncoding	noncoding
MSTRG.170435.1	FARP1	XLOC_172114	10644	coding	coding	coding	coding
MSTRG.170429.1	SLC15A1	XLOC_172124	225	coding	noncoding	noncoding	noncoding
MSTRG.170602.1	LOC101927437	XLOC_172201	300	coding	noncoding	noncoding	noncoding
MSTRG.170604.1	LOC101927437	XLOC_172202	284	coding	noncoding	noncoding	noncoding
NR_047687.1	NALCN-AS1	XLOC_172218	2846	coding	noncoding	noncoding	noncoding
MSTRG.170635.1	NALCN	XLOC_172225	248	coding	noncoding	noncoding	noncoding
MSTRG.170642.1	NALCN	XLOC_172227	243	coding	coding	noncoding	noncoding
MSTRG.170658.1	ITGBL1	XLOC_172232	474	coding	noncoding	noncoding	noncoding
MSTRG.170659.1	ITGBL1	XLOC_172233	262	coding	coding	noncoding	noncoding
MSTRG.170690.1	FGF14	XLOC_172247	591	coding	coding	noncoding	noncoding
MSTRG.170738.1		XLOC_172298	227	coding	noncoding	noncoding	noncoding
MSTRG.170748.1		XLOC_172305	315	coding	noncoding	noncoding	noncoding
MSTRG.170825.1		XLOC_172335	288	coding	noncoding	noncoding	noncoding
MSTRG.170852.1	LINC00343	XLOC_172348	377	coding	noncoding	noncoding	noncoding
MSTRG.170843.1		XLOC_172351	254	coding	noncoding	noncoding	noncoding
MSTRG.170863.1		XLOC_172361	232	coding	noncoding	noncoding	noncoding
MSTRG.170869.1		XLOC_172365	308	coding	noncoding	noncoding	noncoding
MSTRG.170871.1		XLOC_172366	230	coding	noncoding	noncoding	noncoding
MSTRG.170967.1	FAM155A	XLOC_172400	205	coding	noncoding	noncoding	noncoding
MSTRG.170933.1		XLOC_172406	266	coding	noncoding	noncoding	noncoding
MSTRG.170937.1		XLOC_172409	338	coding	noncoding	noncoding	noncoding
NM_001145645.2	TNFSF13B	XLOC_172427	2614	coding	coding	coding	coding
MSTRG.170985.1		XLOC_172442	265	coding	noncoding	noncoding	noncoding
XR_002957496.1	LOC112268112	XLOC_172521	15283	coding	noncoding	noncoding	noncoding
MSTRG.171143.1	LOC101927646	XLOC_172523	435	coding	coding	noncoding	noncoding
MSTRG.171145.1	LOC101927712	XLOC_172527	206	coding	noncoding	noncoding	noncoding
NM_001846.4	COL4A2	XLOC_172531	6446	coding	coding	coding	coding
MSTRG.171163.1	COL4A2-AS1	XLOC_172535	291	coding	noncoding	noncoding	noncoding
NM_001113513.2	ARHGEF7	XLOC_172563	4976	coding	coding	coding	coding
MSTRG.171369.1	ARHGEF7	XLOC_172566	263	coding	noncoding	noncoding	noncoding
MSTRG.171246.1		XLOC_172580	229	coding	noncoding	noncoding	noncoding
MSTRG.171347.1		XLOC_172595	269	coding	noncoding	noncoding	noncoding
XM_005268299.4	ATP11A	XLOC_172611	9066	coding	coding	coding	coding
XM_005268300.4	ATP11A	XLOC_172611	9002	coding	coding	coding	coding
XM_011537476.2	F7	XLOC_172631	3543	coding	coding	coding	coding
MSTRG.171405.1	F10	XLOC_172633	214	coding	noncoding	noncoding	noncoding
NM_001278513.2	CUL4A	XLOC_172638	6073	coding	coding	coding	coding
MSTRG.171494.1	TMCO3	XLOC_172659	403	coding	noncoding	noncoding	noncoding
MSTRG.171554.4	RASA3	XLOC_172691	811	coding	coding	coding	noncoding

MSTRG.164589.1		XLOC_172718	303 coding	noncoding	noncoding	noncoding
MSTRG.164605.1		XLOC_172728	288 coding	noncoding	noncoding	noncoding
NM_001354909.2	PSPC1	XLOC_172737	2435 coding	coding	coding	coding
NM_001142684.2	ZMYM5	XLOC_172739	3283 coding	coding	coding	coding
MSTRG.164779.1		XLOC_172742	286 coding	noncoding	noncoding	noncoding
MSTRG.164781.1		XLOC_172743	293 coding	noncoding	noncoding	noncoding
MSTRG.164782.1		XLOC_172744	289 coding	noncoding	noncoding	noncoding
MSTRG.164610.1	LOC105370101	XLOC_172749	232 coding	noncoding	noncoding	noncoding
NM_001370092.1	GJB6	XLOC_172755	2161 coding	coding	coding	coding
XR_941546.1	ZDHHC20	XLOC_172817	5547 coding	coding	coding	coding
MSTRG.164865.1		XLOC_172981	226 coding	noncoding	noncoding	noncoding
MSTRG.164867.1		XLOC_172984	262 coding	noncoding	noncoding	noncoding
MSTRG.164872.1	SGCG	XLOC_172986	286 coding	noncoding	noncoding	noncoding
XR_001749788.1	LOC105370114	XLOC_172995	6305 coding	noncoding	coding	noncoding
MSTRG.164912.1	LOC105370113	XLOC_173000	227 coding	noncoding	noncoding	noncoding
MSTRG.164936.1	SPATA13	XLOC_173015	240 coding	noncoding	noncoding	noncoding
MSTRG.164928.1		XLOC_173029	297 coding	noncoding	noncoding	noncoding
MSTRG.165118.1	LOC105370117	XLOC_173033	250 coding	noncoding	noncoding	noncoding
MSTRG.165130.1	RNF17	XLOC_173042	245 coding	noncoding	noncoding	noncoding
MSTRG.165137.1	CENPJ	XLOC_173045	259 coding	noncoding	noncoding	noncoding
NR_026730.1	TPTE2P1	XLOC_173046	6477 coding	noncoding	coding	noncoding
MSTRG.165112.1	TPTE2P1	XLOC_173047	280 coding	noncoding	noncoding	noncoding
MSTRG.165139.1		XLOC_173058	310 coding	noncoding	noncoding	noncoding
MSTRG.165183.1	ATP8A2	XLOC_173074	230 coding	noncoding	noncoding	noncoding
NM_001007538.1	SHISA2	XLOC_173080	2889 coding	coding	coding	coding
MSTRG.165196.1	RNF6	XLOC_173084	252 coding	noncoding	noncoding	noncoding
NM_005288.4	GPR12	XLOC_173103	4851 coding	coding	coding	coding
MSTRG.165244.1		XLOC_173111	288 coding	noncoding	noncoding	noncoding
MSTRG.165271.1	LOC105370124	XLOC_173114	234 coding	noncoding	noncoding	noncoding
MSTRG.165296.1		XLOC_173136	260 coding	noncoding	noncoding	noncoding
MSTRG.165311.1		XLOC_173137	424 coding	coding	noncoding	noncoding
MSTRG.165313.1		XLOC_173139	260 coding	noncoding	noncoding	noncoding
MSTRG.165327.3	MTIF3	XLOC_173142	2347 coding	coding	coding	coding
MSTRG.165316.1		XLOC_173143	261 coding	noncoding	noncoding	noncoding
MSTRG.165354.14	LNX2	XLOC_173148	2569 coding	noncoding	coding	coding
MSTRG.165354.10	LNX2	XLOC_173148	2605 coding	noncoding	coding	coding
MSTRG.165330.1		XLOC_173152	235 coding	noncoding	noncoding	noncoding
MSTRG.165334.1	PLUT	XLOC_173154	269 coding	noncoding	noncoding	noncoding
MSTRG.165344.1	LOC105370132	XLOC_173160	526 coding	noncoding	noncoding	noncoding
MSTRG.165408.1		XLOC_173183	249 coding	noncoding	noncoding	noncoding
MSTRG.165430.1	MTUS2	XLOC_173186	338 coding	noncoding	noncoding	noncoding
MSTRG.165443.1	MTUS2	XLOC_173192	226 coding	noncoding	noncoding	noncoding
MSTRG.165448.1	MTUS2	XLOC_173195	252 coding	noncoding	noncoding	noncoding

MSTRG.165471.1	MTUS2	XLOC_173207	356 coding	noncoding	noncoding	noncoding
MSTRG.165475.1		XLOC_173216	249 coding	noncoding	noncoding	noncoding
MSTRG.165533.1		XLOC_173225	280 coding	noncoding	noncoding	noncoding
MSTRG.165537.1	UBL3	XLOC_173228	236 coding	noncoding	noncoding	noncoding
MSTRG.165490.1	LINC00297	XLOC_173248	232 coding	noncoding	noncoding	noncoding
MSTRG.165680.1	KATNAL1	XLOC_173265	316 coding	noncoding	noncoding	noncoding
MSTRG.165566.1		XLOC_173289	205 coding	noncoding	noncoding	noncoding
MSTRG.165573.1		XLOC_173302	307 coding	noncoding	noncoding	noncoding
MSTRG.165656.1		XLOC_173333	290 coding	noncoding	noncoding	noncoding
NM_001349704.2	HSPH1	XLOC_173342	4994 coding	coding	coding	coding
MSTRG.165732.1		XLOC_173348	269 coding	noncoding	noncoding	noncoding
MSTRG.165738.1		XLOC_173356	312 coding	noncoding	noncoding	noncoding
MSTRG.165743.1		XLOC_173357	292 coding	noncoding	noncoding	noncoding
MSTRG.166348.1	ZAR1L	XLOC_173371	318 coding	noncoding	noncoding	noncoding
NR_047020.1	LINC00423	XLOC_173405	1356 coding	noncoding	noncoding	noncoding
MSTRG.165880.1	STARD13	XLOC_173426	262 coding	noncoding	noncoding	noncoding
MSTRG.165905.1	LOC102723406	XLOC_173433	212 coding	noncoding	noncoding	noncoding
MSTRG.165943.1	LINC02343	XLOC_173496	271 coding	noncoding	noncoding	noncoding
MSTRG.165950.1	LINC02343	XLOC_173499	277 coding	noncoding	noncoding	noncoding
NM_004734.5	DCLK1	XLOC_173569	8468 coding	coding	coding	coding
MSTRG.166048.1	CCDC169-SOHLH2	XLOC_173577	374 coding	noncoding	noncoding	noncoding
MSTRG.166086.1	SMAD9	XLOC_173607	218 coding	noncoding	noncoding	noncoding
MSTRG.166178.2	SUPT20H	XLOC_173613	3773 coding	coding	coding	coding
MSTRG.166190.1		XLOC_173627	279 coding	noncoding	noncoding	noncoding
XM_017020356.1	POSTN	XLOC_173630	3363 coding	coding	coding	coding
MSTRG.166416.1	STOML3	XLOC_173664	242 coding	noncoding	noncoding	noncoding
MSTRG.166527.1		XLOC_173695	281 coding	noncoding	noncoding	noncoding
MSTRG.166598.1	LINC00598	XLOC_173709	286 coding	coding	noncoding	noncoding
MSTRG.166571.1		XLOC_173716	251 coding	noncoding	noncoding	noncoding
MSTRG.166574.1		XLOC_173717	218 coding	noncoding	noncoding	noncoding
MSTRG.166627.1		XLOC_173752	287 coding	noncoding	noncoding	noncoding
MSTRG.166694.1	TPTE2P5	XLOC_173767	249 coding	noncoding	noncoding	noncoding
NM_001370332.1	ELF1	XLOC_173773	3986 coding	coding	coding	coding
MSTRG.166756.1	LOC101929140	XLOC_173797	287 coding	noncoding	noncoding	noncoding
MSTRG.166736.1		XLOC_173808	258 coding	noncoding	noncoding	noncoding
MSTRG.166731.1		XLOC_173810	224 coding	noncoding	noncoding	noncoding
MSTRG.166788.1	LINC02341	XLOC_173855	314 coding	noncoding	noncoding	noncoding
MSTRG.166825.1	LINC00428	XLOC_173868	248 coding	noncoding	noncoding	noncoding
XR_001749592.2	ENOX1	XLOC_173900	6188 coding	coding	coding	coding
MSTRG.166865.1	ENOX1	XLOC_173907	217 coding	noncoding	noncoding	noncoding
MSTRG.166852.1		XLOC_173916	353 coding	coding	noncoding	noncoding
MSTRG.166934.2	LOC107984576	XLOC_173927	2485 coding	coding	coding	coding
MSTRG.167001.1		XLOC_173960	231 coding	noncoding	noncoding	noncoding

MSTRG.167011.1	LINC00407	XLOC_173961	230 coding	noncoding	noncoding	noncoding
MSTRG.167006.1		XLOC_173965	293 coding	noncoding	noncoding	noncoding
MSTRG.167016.1		XLOC_173966	217 coding	noncoding	noncoding	noncoding
MSTRG.167026.1	LOC105370187	XLOC_173971	325 coding	noncoding	noncoding	noncoding
MSTRG.167034.1	LOC107984596	XLOC_173981	244 coding	noncoding	noncoding	noncoding
MSTRG.167030.1		XLOC_173982	398 coding	noncoding	noncoding	noncoding
MSTRG.167040.1	LOC101929259	XLOC_173984	417 coding	noncoding	noncoding	noncoding
MSTRG.167127.6	TPT1	XLOC_173988	1765 coding	coding	coding	coding
MSTRG.167127.4	TPT1	XLOC_173988	2073 coding	coding	coding	coding
MSTRG.167127.3	TPT1	XLOC_173988	2072 coding	coding	coding	coding
MSTRG.167127.2	TPT1	XLOC_173988	1781 coding	coding	coding	coding
NM_001286807.2	SLC25A30	XLOC_173996	3721 coding	coding	coding	coding
XM_006719793.4	SLC25A30	XLOC_173996	1702 coding	coding	coding	coding
MSTRG.167078.1		XLOC_174005	246 coding	noncoding	noncoding	noncoding
NM_198849.3	SIAH3	XLOC_174010	7075 coding	coding	coding	coding
MSTRG.167187.1	LRRC63	XLOC_174046	225 coding	noncoding	noncoding	noncoding
MSTRG.167211.1		XLOC_174071	234 coding	noncoding	noncoding	noncoding
MSTRG.167256.1		XLOC_174089	292 coding	noncoding	noncoding	noncoding
MSTRG.167264.1		XLOC_174095	316 coding	noncoding	noncoding	noncoding
MSTRG.167268.1		XLOC_174097	309 coding	noncoding	noncoding	noncoding
MSTRG.167503.1		XLOC_174168	300 coding	noncoding	noncoding	noncoding
MSTRG.167527.1	KPNA3	XLOC_174174	289 coding	noncoding	noncoding	noncoding
MSTRG.167517.1	SPRYD7	XLOC_174185	310 coding	noncoding	noncoding	noncoding
MSTRG.168773.1		XLOC_174192	276 coding	noncoding	noncoding	noncoding
MSTRG.168788.1	DLEU1	XLOC_174207	280 coding	noncoding	noncoding	noncoding
MSTRG.167581.1		XLOC_174410	309 coding	noncoding	noncoding	noncoding
MSTRG.167606.1		XLOC_174426	243 coding	noncoding	noncoding	noncoding
MSTRG.167619.1	TPTE2P2	XLOC_174432	251 coding	noncoding	noncoding	noncoding
MSTRG.167621.1	TPTE2P2	XLOC_174433	296 coding	noncoding	noncoding	noncoding
MSTRG.167615.1		XLOC_174437	269 coding	noncoding	noncoding	noncoding
MSTRG.167626.1	THSD1	XLOC_174439	293 coding	noncoding	noncoding	noncoding
MSTRG.167663.1	TPTE2P3	XLOC_174449	208 coding	noncoding	noncoding	noncoding
MSTRG.167660.1		XLOC_174452	274 coding	noncoding	noncoding	noncoding
MSTRG.167720.1		XLOC_174470	320 coding	noncoding	coding	noncoding
MSTRG.167748.1		XLOC_174485	219 coding	noncoding	noncoding	noncoding
MSTRG.167756.1		XLOC_174490	534 coding	noncoding	noncoding	noncoding
MSTRG.167771.1		XLOC_174498	330 coding	coding	noncoding	noncoding
MSTRG.167774.1		XLOC_174501	232 coding	noncoding	noncoding	noncoding
MSTRG.167790.1		XLOC_174508	272 coding	noncoding	noncoding	noncoding
MSTRG.167831.1	LOC105370214	XLOC_174523	226 coding	noncoding	noncoding	noncoding
MSTRG.167836.1	LOC105370214	XLOC_174525	227 coding	noncoding	noncoding	noncoding
MSTRG.167817.1		XLOC_174534	223 coding	noncoding	noncoding	noncoding
MSTRG.167823.1		XLOC_174537	241 coding	noncoding	noncoding	noncoding

MSTRG.167872.1		XLOC_174544	291 coding	noncoding	noncoding	noncoding
MSTRG.167883.1		XLOC_174550	217 coding	noncoding	noncoding	noncoding
MSTRG.167888.1		XLOC_174558	270 coding	noncoding	noncoding	noncoding
MSTRG.167900.1		XLOC_174560	267 coding	noncoding	noncoding	noncoding
MSTRG.167934.1	LOC105370219	XLOC_174578	281 coding	noncoding	noncoding	noncoding
XM_011535258.2	DIAPH3	XLOC_174595	4021 coding	coding	coding	coding
MSTRG.168005.1	DIAPH3	XLOC_174596	316 coding	noncoding	noncoding	noncoding
MSTRG.167985.1	LINC00434	XLOC_174611	320 coding	noncoding	noncoding	noncoding
MSTRG.168035.1		XLOC_174622	258 coding	noncoding	noncoding	noncoding
MSTRG.168093.1		XLOC_174656	287 coding	noncoding	noncoding	noncoding
MSTRG.168111.1	LINC00448	XLOC_174663	212 coding	noncoding	noncoding	noncoding
MSTRG.168128.1		XLOC_174678	264 coding	coding	noncoding	noncoding
MSTRG.168144.1		XLOC_174687	294 coding	noncoding	noncoding	noncoding
MSTRG.168168.1		XLOC_174700	299 coding	noncoding	noncoding	noncoding
MSTRG.168169.1		XLOC_174701	281 coding	coding	noncoding	noncoding
MSTRG.168186.1		XLOC_174710	243 coding	noncoding	noncoding	noncoding
MSTRG.168189.1		XLOC_174712	300 coding	coding	noncoding	noncoding
MSTRG.168192.1		XLOC_174713	399 coding	noncoding	noncoding	noncoding
NM_203487.3	PCDH9	XLOC_174728	6227 coding	coding	coding	coding
MSTRG.168405.1	PCDH9	XLOC_174736	250 coding	noncoding	noncoding	noncoding
MSTRG.168408.1	PCDH9	XLOC_174738	559 coding	noncoding	noncoding	noncoding
MSTRG.168254.1		XLOC_174757	262 coding	noncoding	noncoding	noncoding
MSTRG.168269.1		XLOC_174765	287 coding	noncoding	noncoding	noncoding
NR_038878.1	LINC00550	XLOC_174774	3434 coding	noncoding	noncoding	noncoding
MSTRG.168305.1		XLOC_174782	360 coding	noncoding	noncoding	noncoding
MSTRG.168309.1		XLOC_174784	251 coding	noncoding	noncoding	noncoding
MSTRG.168327.1	KLHL1	XLOC_174787	284 coding	noncoding	noncoding	noncoding
MSTRG.168311.1		XLOC_174793	256 coding	noncoding	noncoding	noncoding
MSTRG.168348.1		XLOC_174805	201 coding	noncoding	noncoding	noncoding
NM_001366712.1	DACH1	XLOC_174821	5401 coding	coding	coding	coding
MSTRG.168446.1		XLOC_174847	446 coding	noncoding	noncoding	noncoding
MSTRG.168481.1		XLOC_174875	234 coding	noncoding	noncoding	noncoding
MSTRG.168485.1		XLOC_174876	334 coding	noncoding	noncoding	noncoding
MSTRG.168491.1		XLOC_174880	297 coding	noncoding	noncoding	noncoding
MSTRG.168494.1		XLOC_174882	232 coding	noncoding	noncoding	noncoding
XM_017020385.1	KLF12	XLOC_174898	10872 coding	coding	coding	coding
MSTRG.169035.1	KLF12	XLOC_174903	289 coding	noncoding	noncoding	noncoding
MSTRG.168649.1		XLOC_174982	261 coding	coding	noncoding	noncoding
MSTRG.168654.1		XLOC_174985	276 coding	noncoding	noncoding	noncoding
MSTRG.169139.1	COMMD6	XLOC_175000	283 coding	noncoding	noncoding	noncoding
MSTRG.169478.1	MYCBP2	XLOC_175091	259 coding	noncoding	noncoding	noncoding
MSTRG.169279.1		XLOC_175140	247 coding	coding	noncoding	noncoding
MSTRG.169280.1		XLOC_175141	308 coding	noncoding	noncoding	noncoding

MSTRG.169336.1	RNF219-AS1	XLOC_175154	279 coding	noncoding	noncoding	noncoding
MSTRG.169340.1	RNF219-AS1	XLOC_175158	261 coding	noncoding	noncoding	noncoding
NM_006237.4	POU4F1	XLOC_175163	4539 coding	coding	coding	coding
MSTRG.169294.1		XLOC_175168	294 coding	noncoding	noncoding	noncoding
XM_005266497.2	RBM26	XLOC_175182	6382 coding	coding	coding	coding
MSTRG.169358.1		XLOC_175212	245 coding	noncoding	noncoding	noncoding
MSTRG.169390.1	LINC00382	XLOC_175224	452 coding	noncoding	noncoding	noncoding
MSTRG.169381.1		XLOC_175225	218 coding	noncoding	noncoding	noncoding
NM_001318537.1	SPRY2	XLOC_175232	2708 coding	coding	coding	coding
MSTRG.169419.1		XLOC_175240	325 coding	noncoding	noncoding	noncoding
MSTRG.169428.1		XLOC_175248	269 coding	noncoding	noncoding	noncoding
MSTRG.169609.1		XLOC_175292	269 coding	noncoding	noncoding	noncoding
MSTRG.169626.1	LOC105370284	XLOC_175302	229 coding	noncoding	noncoding	noncoding
MSTRG.169618.1		XLOC_175303	210 coding	noncoding	noncoding	noncoding
MSTRG.169635.1		XLOC_175308	306 coding	coding	noncoding	noncoding
MSTRG.169656.1		XLOC_175320	247 coding	noncoding	noncoding	noncoding
MSTRG.169673.1		XLOC_175326	290 coding	noncoding	noncoding	noncoding
MSTRG.169683.1	LOC105370286	XLOC_175329	324 coding	noncoding	noncoding	noncoding
NM_052910.2	SLITRK1	XLOC_175331	5189 coding	coding	coding	coding
MSTRG.169709.1		XLOC_175372	206 coding	noncoding	noncoding	noncoding
MSTRG.169725.1	LOC105370291	XLOC_175379	294 coding	noncoding	noncoding	noncoding
MSTRG.169738.1	LINC00351	XLOC_175385	309 coding	noncoding	noncoding	noncoding
MSTRG.169829.1		XLOC_175434	242 coding	noncoding	noncoding	noncoding
MSTRG.169837.1		XLOC_175437	222 coding	noncoding	noncoding	noncoding
MSTRG.169880.1	MIR4500HG	XLOC_175445	510 coding	noncoding	noncoding	noncoding
MSTRG.169866.1	LOC105370304	XLOC_175458	235 coding	noncoding	noncoding	noncoding
MSTRG.169924.1		XLOC_175473	231 coding	noncoding	noncoding	noncoding
MSTRG.169926.1		XLOC_175475	258 coding	noncoding	noncoding	noncoding
MSTRG.169931.1		XLOC_175476	346 coding	noncoding	noncoding	noncoding
MSTRG.169957.1		XLOC_175493	272 coding	noncoding	noncoding	noncoding
MSTRG.169968.1		XLOC_175500	295 coding	noncoding	noncoding	noncoding
MSTRG.169986.1	LINC01049	XLOC_175507	307 coding	noncoding	noncoding	noncoding
MSTRG.169994.1	LOC107984572	XLOC_175511	293 coding	noncoding	noncoding	noncoding
MSTRG.169998.1		XLOC_175513	280 coding	coding	noncoding	noncoding
MSTRG.170074.1	GPC5	XLOC_175538	309 coding	noncoding	noncoding	noncoding
MSTRG.170076.1	GPC5	XLOC_175539	272 coding	noncoding	noncoding	noncoding
MSTRG.170098.1	GPC5	XLOC_175552	241 coding	noncoding	noncoding	noncoding
MSTRG.170109.1	GPC5	XLOC_175558	355 coding	coding	noncoding	noncoding
MSTRG.170162.1	GPC6	XLOC_175589	207 coding	noncoding	noncoding	noncoding
NM_001922.5	DCT	XLOC_175598	5074 coding	coding	coding	coding
NM_005845.4	ABCC4	XLOC_175626	5856 coding	coding	coding	coding
MSTRG.170238.1	ABCC4	XLOC_175629	283 coding	noncoding	noncoding	noncoding
MSTRG.170214.1		XLOC_175633	303 coding	noncoding	noncoding	noncoding

MSTRG.170222.1	CLDN10	XLOC_175635	251 coding	noncoding	noncoding	noncoding
NM_198968.4	DZIP1	XLOC_175639	7491 coding	coding	coding	coding
MSTRG.170217.1		XLOC_175641	276 coding	noncoding	noncoding	noncoding
MSTRG.170319.1	UGGT2	XLOC_175651	253 coding	noncoding	noncoding	noncoding
MSTRG.170333.1	UGGT2	XLOC_175659	257 coding	noncoding	noncoding	noncoding
MSTRG.170284.1	HS6ST3	XLOC_175666	298 coding	noncoding	noncoding	noncoding
MSTRG.170297.1	HS6ST3	XLOC_175671	317 coding	noncoding	noncoding	noncoding
MSTRG.170298.1	HS6ST3	XLOC_175672	218 coding	noncoding	noncoding	noncoding
MSTRG.170267.1		XLOC_175677	285 coding	noncoding	noncoding	noncoding
MSTRG.170271.1		XLOC_175678	298 coding	noncoding	noncoding	noncoding
MSTRG.170340.1		XLOC_175692	382 coding	noncoding	noncoding	noncoding
MSTRG.170370.1		XLOC_175701	269 coding	noncoding	noncoding	noncoding
XR_931663.2	LOC105370324	XLOC_175705	4233 coding	noncoding	noncoding	noncoding
MSTRG.170433.1	LOC105370328	XLOC_175719	272 coding	noncoding	noncoding	noncoding
MSTRG.170445.1	FARP1	XLOC_175727	289 coding	noncoding	noncoding	noncoding
XM_024449426.1	STK24	XLOC_175735	2588 coding	coding	coding	coding
MSTRG.170427.1	SLC15A1	XLOC_175754	280 coding	noncoding	noncoding	noncoding
XM_017020405.2	GPR183	XLOC_175786	1992 coding	coding	coding	coding
NM_004951.5	GPR183	XLOC_175786	1685 coding	coding	coding	coding
MSTRG.170484.1		XLOC_175790	264 coding	noncoding	noncoding	noncoding
MSTRG.170486.1		XLOC_175791	248 coding	noncoding	noncoding	noncoding
MSTRG.170603.1	LOC101927437	XLOC_175810	300 coding	noncoding	noncoding	noncoding
MSTRG.170540.1		XLOC_175814	266 coding	noncoding	noncoding	noncoding
MSTRG.170614.1	TMTC4	XLOC_175828	279 coding	noncoding	noncoding	noncoding
MSTRG.170622.1	NALCN-AS1	XLOC_175833	432 coding	noncoding	noncoding	noncoding
MSTRG.170634.1	NALCN	XLOC_175841	473 coding	noncoding	noncoding	noncoding
MSTRG.170636.1	NALCN	XLOC_175842	248 coding	noncoding	noncoding	noncoding
MSTRG.170645.1	NALCN	XLOC_175848	259 coding	noncoding	noncoding	noncoding
MSTRG.170660.1	ITGBL1	XLOC_175851	262 coding	coding	noncoding	noncoding
MSTRG.170664.1	ITGBL1	XLOC_175854	433 coding	noncoding	noncoding	noncoding
MSTRG.170682.1	FGF14	XLOC_175862	234 coding	noncoding	noncoding	noncoding
MSTRG.170689.1	FGF14	XLOC_175867	412 coding	coding	noncoding	noncoding
MSTRG.170769.1	POGLUT2	XLOC_175879	475 coding	noncoding	noncoding	noncoding
MSTRG.170729.1		XLOC_175893	265 coding	noncoding	noncoding	noncoding
MSTRG.170737.1		XLOC_175899	227 coding	noncoding	noncoding	noncoding
MSTRG.170803.1		XLOC_175922	318 coding	noncoding	noncoding	noncoding
MSTRG.170883.1	LOC107984626	XLOC_175953	259 coding	noncoding	noncoding	noncoding
MSTRG.170876.1	LOC105370346	XLOC_175957	303 coding	noncoding	noncoding	noncoding
NM_001080396.3	FAM155A	XLOC_175975	9264 coding	noncoding	coding	coding
MSTRG.170943.1	FAM155A	XLOC_175976	280 coding	noncoding	noncoding	noncoding
XR_001750003.2	LOC107984581	XLOC_175993	13330 coding	noncoding	noncoding	noncoding
MSTRG.171079.1	MYO16	XLOC_176041	295 coding	noncoding	noncoding	noncoding
MSTRG.171082.1	MYO16	XLOC_176044	259 coding	noncoding	noncoding	noncoding

MSTRG.171115.1		XLOC_176115	251 coding	noncoding	noncoding	noncoding
XM_011521048.2	COL4A1	XLOC_176124	14378 coding	coding	coding	coding
MSTRG.171167.1		XLOC_176134	444 coding	noncoding	noncoding	noncoding
MSTRG.171184.1		XLOC_176168	317 coding	noncoding	noncoding	noncoding
MSTRG.171391.1	TEX29	XLOC_176199	248 coding	noncoding	noncoding	noncoding
MSTRG.171331.1		XLOC_176208	256 coding	noncoding	noncoding	noncoding
MSTRG.171395.1	TUBGCP3	XLOC_176232	388 coding	noncoding	noncoding	noncoding
MSTRG.171398.1	TUBGCP3	XLOC_176235	253 coding	noncoding	noncoding	noncoding
MSTRG.171538.1		XLOC_176317	400 coding	noncoding	noncoding	noncoding
MSTRG.171586.1		XLOC_176329	265 coding	noncoding	noncoding	noncoding
MSTRG.171589.1		XLOC_176332	315 coding	noncoding	noncoding	noncoding
NM_001145442.1	POTEM	XLOC_176333	6666 coding	coding	coding	coding
NR_122112.1	DUXAP9	XLOC_176335	1401 coding	noncoding	noncoding	noncoding
MSTRG.171622.1		XLOC_176339	304 coding	noncoding	coding	noncoding
MSTRG.171625.1		XLOC_176341	283 coding	noncoding	noncoding	noncoding
MSTRG.171634.1		XLOC_176348	230 coding	noncoding	noncoding	noncoding
MSTRG.171638.1		XLOC_176350	299 coding	coding	noncoding	noncoding
MSTRG.171645.1	OR4N2	XLOC_176356	299 coding	noncoding	noncoding	noncoding
MSTRG.171657.1		XLOC_176367	286 coding	noncoding	noncoding	noncoding
MSTRG.171658.1		XLOC_176368	295 coding	noncoding	noncoding	noncoding
MSTRG.171668.1		XLOC_176389	225 coding	noncoding	noncoding	noncoding
MSTRG.171743.2	TEP1	XLOC_176399	8225 coding	coding	coding	noncoding
MSTRG.171696.1		XLOC_176400	206 coding	noncoding	noncoding	noncoding
MSTRG.171707.1		XLOC_176407	265 coding	noncoding	noncoding	noncoding
MSTRG.171729.1		XLOC_176418	248 coding	noncoding	noncoding	noncoding
MSTRG.171749.1		XLOC_176431	298 coding	noncoding	noncoding	noncoding
MSTRG.171753.1	LOC100507513	XLOC_176433	284 coding	noncoding	noncoding	noncoding
NM_014579.4	SLC39A2	XLOC_176439	1376 coding	coding	coding	coding
MSTRG.171827.9	CHD8	XLOC_176455	1244 coding	noncoding	noncoding	noncoding
MSTRG.171836.2	RAB2B	XLOC_176457	2465 coding	coding	noncoding	coding
MSTRG.171816.1		XLOC_176459	250 coding	noncoding	noncoding	noncoding
MSTRG.171817.1		XLOC_176461	287 coding	noncoding	noncoding	noncoding
TRAV22	TRAV22	XLOC_176462	331 coding	coding	noncoding	noncoding
MSTRG.172178.125	TRAJ9	XLOC_176462	605 coding	coding	coding	coding
MSTRG.172209.1	TRAV1-1	XLOC_176475	257 coding	noncoding	noncoding	noncoding
MSTRG.171865.1	ABHD4	XLOC_176506	233 coding	noncoding	noncoding	noncoding
MSTRG.171861.1		XLOC_176509	231 coding	noncoding	noncoding	noncoding
MSTRG.171914.1	RBM23	XLOC_176524	7899 coding	coding	coding	noncoding
NR_120599.1	PRMT5-AS1	XLOC_176525	2104 coding	coding	coding	noncoding
MSTRG.171941.3	LOC107984665	XLOC_176535	4697 coding	coding	coding	coding
MSTRG.171934.1	SLC7A8	XLOC_176545	417 coding	noncoding	noncoding	coding
MSTRG.171974.1	MYH7	XLOC_176560	265 coding	noncoding	coding	noncoding
MSTRG.172006.1	ZFHX2	XLOC_176565	260 coding	noncoding	noncoding	noncoding

MSTRG.171991.1		XLOC_176570	320 coding	noncoding	noncoding	noncoding
MSTRG.172114.1		XLOC_176621	257 coding	noncoding	noncoding	noncoding
MSTRG.172120.1	LINC02286	XLOC_176633	262 coding	coding	noncoding	noncoding
MSTRG.172171.1		XLOC_176655	290 coding	noncoding	noncoding	noncoding
NR_147061.1	LINC02588	XLOC_176660	2252 coding	noncoding	noncoding	noncoding
MSTRG.172316.1	LOC728755	XLOC_176676	266 coding	noncoding	noncoding	noncoding
MSTRG.172337.1		XLOC_176681	716 coding	noncoding	coding	noncoding
MSTRG.172339.1		XLOC_176689	256 coding	noncoding	noncoding	noncoding
MSTRG.172353.1	LOC107984684	XLOC_176695	304 coding	noncoding	noncoding	noncoding
MSTRG.172356.1		XLOC_176697	298 coding	noncoding	noncoding	noncoding
MSTRG.172395.1	PRKD1	XLOC_176719	314 coding	noncoding	noncoding	noncoding
MSTRG.172391.1		XLOC_176725	510 coding	noncoding	noncoding	noncoding
MSTRG.172416.1		XLOC_176731	348 coding	noncoding	noncoding	noncoding
MSTRG.172426.1		XLOC_176757	515 coding	noncoding	noncoding	noncoding
MSTRG.172575.1	LOC105370438	XLOC_176783	235 coding	noncoding	noncoding	noncoding
MSTRG.172501.1		XLOC_176797	257 coding	noncoding	noncoding	noncoding
NM_001173.3	ARHGAP5	XLOC_176800	9586 coding	coding	coding	coding
MSTRG.172665.1	AKAP6	XLOC_176817	222 coding	noncoding	noncoding	noncoding
MSTRG.172673.1	AKAP6	XLOC_176821	293 coding	noncoding	noncoding	noncoding
MSTRG.172565.1		XLOC_176829	260 coding	noncoding	noncoding	noncoding
MSTRG.172724.1	NPAS3	XLOC_176834	238 coding	noncoding	noncoding	noncoding
MSTRG.172572.1		XLOC_176854	350 coding	noncoding	noncoding	noncoding
MSTRG.172785.1	LOC102724945	XLOC_176880	208 coding	noncoding	noncoding	noncoding
MSTRG.172790.1		XLOC_176888	311 coding	noncoding	noncoding	noncoding
MSTRG.172792.1		XLOC_176890	396 coding	coding	noncoding	noncoding
MSTRG.172808.1		XLOC_176895	406 coding	noncoding	noncoding	noncoding
XM_017021615.2	SRP54	XLOC_176906	2102 coding	coding	coding	coding
NM_001282233.1	PSMA6	XLOC_176909	979 coding	coding	coding	coding
MSTRG.172855.1		XLOC_176915	288 coding	noncoding	noncoding	noncoding
MSTRG.172857.1		XLOC_176916	246 coding	noncoding	noncoding	noncoding
MSTRG.172883.1		XLOC_176935	676 coding	coding	noncoding	noncoding
NM_001195296.2	MIPOL1	XLOC_176973	7351 coding	noncoding	coding	coding
XM_017021019.1	MIPOL1	XLOC_176973	7650 coding	noncoding	coding	coding
MSTRG.172971.1	MIPOL1	XLOC_176974	258 coding	noncoding	noncoding	noncoding
MSTRG.172986.1	MIPOL1	XLOC_176978	298 coding	noncoding	noncoding	noncoding
MSTRG.173005.1	TTC6	XLOC_176987	303 coding	noncoding	noncoding	noncoding
MSTRG.172968.1		XLOC_176992	226 coding	noncoding	noncoding	noncoding
MSTRG.173098.1	LINC00639	XLOC_177044	269 coding	noncoding	noncoding	noncoding
MSTRG.173106.1		XLOC_177060	832 coding	noncoding	noncoding	noncoding
NM_001354154.2	MIA2	XLOC_177066	3961 coding	noncoding	coding	coding
MSTRG.173319.1	MIA2	XLOC_177067	269 coding	noncoding	noncoding	noncoding
MSTRG.173168.1	LINC02315	XLOC_177108	322 coding	noncoding	noncoding	noncoding
MSTRG.173176.1	LINC02315	XLOC_177113	229 coding	noncoding	noncoding	noncoding

MSTRG.173209.1		XLOC_177131	208 coding	noncoding	noncoding	noncoding
MSTRG.173210.1		XLOC_177132	240 coding	noncoding	noncoding	noncoding
MSTRG.173216.1		XLOC_177137	225 coding	noncoding	noncoding	noncoding
MSTRG.173217.1		XLOC_177138	247 coding	noncoding	noncoding	noncoding
MSTRG.173235.1		XLOC_177148	296 coding	coding	noncoding	noncoding
MSTRG.173236.1		XLOC_177149	365 coding	noncoding	noncoding	noncoding
MSTRG.173239.1		XLOC_177151	319 coding	noncoding	noncoding	noncoding
MSTRG.173255.1		XLOC_177159	299 coding	noncoding	noncoding	noncoding
MSTRG.173269.1		XLOC_177166	289 coding	noncoding	noncoding	noncoding
MSTRG.173277.1	LINC02307	XLOC_177168	348 coding	noncoding	noncoding	noncoding
MSTRG.173283.1		XLOC_177171	314 coding	noncoding	noncoding	noncoding
MSTRG.173294.1	LINC02277	XLOC_177174	270 coding	noncoding	noncoding	noncoding
MSTRG.173300.1		XLOC_177180	218 coding	noncoding	noncoding	noncoding
MSTRG.173302.1		XLOC_177181	632 coding	noncoding	noncoding	noncoding
MSTRG.173305.1		XLOC_177183	256 coding	noncoding	noncoding	noncoding
MSTRG.173310.1		XLOC_177184	318 coding	coding	noncoding	noncoding
MSTRG.173312.1		XLOC_177186	236 coding	noncoding	noncoding	noncoding
MSTRG.173422.1		XLOC_177206	224 coding	noncoding	noncoding	noncoding
MSTRG.173427.1	LOC105370476	XLOC_177210	315 coding	noncoding	noncoding	noncoding
MSTRG.173412.1		XLOC_177214	298 coding	noncoding	noncoding	noncoding
MSTRG.173423.1		XLOC_177217	281 coding	noncoding	noncoding	noncoding
MSTRG.173434.1	LOC105370478	XLOC_177219	297 coding	noncoding	noncoding	noncoding
MSTRG.173441.1		XLOC_177224	329 coding	noncoding	noncoding	noncoding
MSTRG.173476.1	LOC105370481	XLOC_177231	448 coding	noncoding	noncoding	noncoding
MSTRG.173515.1		XLOC_177252	315 coding	coding	noncoding	noncoding
MSTRG.173516.1		XLOC_177253	309 coding	coding	noncoding	noncoding
MSTRG.173517.1		XLOC_177254	313 coding	noncoding	noncoding	noncoding
MSTRG.173570.1		XLOC_177268	360 coding	noncoding	noncoding	noncoding
MSTRG.173583.1	LOC105378178	XLOC_177271	283 coding	noncoding	noncoding	noncoding
MSTRG.173599.1	LOC105378178	XLOC_177281	244 coding	noncoding	noncoding	noncoding
MSTRG.173607.1	LOC105378178	XLOC_177287	244 coding	noncoding	noncoding	noncoding
MSTRG.173614.2	RN7SL1	XLOC_177292	269 coding	noncoding	noncoding	noncoding
MSTRG.173614.8	RN7SL1	XLOC_177292	257 coding	noncoding	noncoding	noncoding
MSTRG.173628.5	POLE2	XLOC_177300	719 coding	noncoding	noncoding	noncoding
MSTRG.173678.2	NEMF	XLOC_177306	870 coding	noncoding	noncoding	noncoding
MSTRG.173678.9	NEMF	XLOC_177306	336 coding	noncoding	noncoding	noncoding
MSTRG.173678.18	LOC105378179	XLOC_177306	559 coding	noncoding	noncoding	noncoding
MSTRG.173641.1		XLOC_177324	254 coding	noncoding	noncoding	noncoding
NM_015915.4	ATL1	XLOC_177331	2647 coding	coding	coding	coding
MSTRG.173670.1		XLOC_177335	290 coding	noncoding	noncoding	noncoding
MSTRG.173736.1	LOC400212	XLOC_177349	278 coding	noncoding	noncoding	noncoding
MSTRG.173733.1		XLOC_177350	239 coding	noncoding	noncoding	noncoding
MSTRG.173740.1		XLOC_177359	272 coding	noncoding	noncoding	noncoding

MSTRG.173816.1		XLOC_177382	223 coding	noncoding	noncoding	noncoding
MSTRG.173848.1	FRMD6	XLOC_177385	213 coding	noncoding	noncoding	noncoding
MSTRG.173852.1	FRMD6	XLOC_177387	244 coding	noncoding	noncoding	noncoding
NM_000953.3	PTGDR	XLOC_177436	2957 coding	coding	coding	coding
MSTRG.173955.1	GPR137C	XLOC_177456	223 coding	coding	noncoding	noncoding
MSTRG.173999.1	LOC105370500	XLOC_177469	221 coding	noncoding	noncoding	noncoding
MSTRG.174007.1	FERMT2	XLOC_177471	272 coding	noncoding	noncoding	noncoding
MSTRG.174172.2	DDHD1	XLOC_177474	5259 coding	noncoding	noncoding	noncoding
MSTRG.174252.1	LOC105370504	XLOC_177486	231 coding	noncoding	noncoding	noncoding
MSTRG.174268.1	LOC105370504	XLOC_177488	287 coding	noncoding	noncoding	noncoding
MSTRG.174012.1	LOC107984676	XLOC_177499	240 coding	noncoding	noncoding	noncoding
MSTRG.174036.1	CDKN3	XLOC_177510	230 coding	noncoding	noncoding	noncoding
MSTRG.174142.1		XLOC_177557	303 coding	noncoding	noncoding	noncoding
MSTRG.174165.1	TBPL2	XLOC_177567	266 coding	noncoding	noncoding	noncoding
MSTRG.174170.1	LOC105370512	XLOC_177593	322 coding	noncoding	noncoding	noncoding
MSTRG.174594.1		XLOC_177671	267 coding	noncoding	noncoding	noncoding
MSTRG.174397.1		XLOC_177700	266 coding	noncoding	noncoding	noncoding
MSTRG.174399.1		XLOC_177702	392 coding	noncoding	noncoding	noncoding
MSTRG.174427.1	LOC105370516	XLOC_177710	301 coding	noncoding	noncoding	noncoding
MSTRG.174438.1		XLOC_177715	249 coding	noncoding	noncoding	noncoding
MSTRG.174467.1	SLC35F4	XLOC_177724	269 coding	noncoding	noncoding	noncoding
MSTRG.174469.1	SLC35F4	XLOC_177726	390 coding	coding	noncoding	noncoding
MSTRG.174477.1	ARMH4	XLOC_177731	374 coding	noncoding	noncoding	noncoding
MSTRG.174633.1	ARID4A	XLOC_177745	288 coding	coding	noncoding	noncoding
MSTRG.174634.1	ARID4A	XLOC_177746	250 coding	noncoding	noncoding	noncoding
MSTRG.174641.1	LOC105370522	XLOC_177752	259 coding	noncoding	noncoding	noncoding
MSTRG.174602.1		XLOC_177761	273 coding	noncoding	noncoding	noncoding
MSTRG.174617.1		XLOC_177767	230 coding	noncoding	noncoding	noncoding
NM_016475.5	JKAMP	XLOC_177796	2347 coding	coding	coding	coding
MSTRG.174716.1		XLOC_177826	220 coding	coding	noncoding	noncoding
MSTRG.174739.3	PPM1A	XLOC_177827	1768 coding	coding	coding	coding
XM_005267781.1	PPM1A	XLOC_177827	8309 coding	coding	coding	coding
MSTRG.174720.1		XLOC_177848	308 coding	noncoding	noncoding	noncoding
NM_007374.3	SIX6	XLOC_177852	2592 coding	coding	coding	coding
MSTRG.174870.1	SLC38A6	XLOC_177883	247 coding	noncoding	noncoding	noncoding
MSTRG.174826.1		XLOC_177887	343 coding	noncoding	noncoding	noncoding
XM_024449662.1	PRKCH	XLOC_177889	6492 coding	coding	coding	coding
MSTRG.174887.1	HIF1A	XLOC_177965	3848 coding	coding	coding	coding
MSTRG.174887.4	HIF1A	XLOC_177965	3882 coding	coding	coding	coding
XM_024449721.1	SYT16	XLOC_177972	14515 coding	coding	coding	coding
XM_024449720.1	SYT16	XLOC_177972	14667 coding	coding	coding	coding
MSTRG.174898.1		XLOC_177980	224 coding	noncoding	noncoding	noncoding
MSTRG.174900.1		XLOC_177981	259 coding	noncoding	noncoding	noncoding

MSTRG.174908.1	LOC105370529	XLOC_177986	216 coding	noncoding	noncoding	noncoding
MSTRG.174904.1		XLOC_177989	212 coding	noncoding	noncoding	noncoding
MSTRG.174936.1	KCNH5	XLOC_177995	295 coding	coding	noncoding	noncoding
MSTRG.175054.1		XLOC_178014	349 coding	noncoding	noncoding	noncoding
XM_011536574.1	SYNE2	XLOC_178021	21948 coding	coding	noncoding	coding
MSTRG.175111.3	MTHFD1	XLOC_178034	594 coding	coding	coding	noncoding
NM_021979.3	HSPA2	XLOC_178043	2770 coding	coding	coding	coding
MSTRG.175100.1		XLOC_178049	218 coding	noncoding	noncoding	noncoding
MSTRG.175150.1		XLOC_178113	248 coding	noncoding	noncoding	noncoding
MSTRG.175974.1	RAD51B	XLOC_178320	287 coding	noncoding	noncoding	noncoding
MSTRG.175982.1	RAD51B	XLOC_178327	259 coding	noncoding	noncoding	noncoding
MSTRG.175391.1		XLOC_178396	286 coding	noncoding	noncoding	noncoding
NM_020692.3	GALNT16	XLOC_178399	5523 coding	coding	coding	coding
MSTRG.175440.8	ERH	XLOC_178399	761 coding	coding	noncoding	noncoding
MSTRG.175458.1		XLOC_178421	234 coding	noncoding	noncoding	noncoding
MSTRG.175487.2	SMOC1	XLOC_178432	924 coding	coding	coding	noncoding
NR_135825.1	LOC646548	XLOC_178439	2977 coding	coding	coding	coding
MSTRG.175592.1	LOC101928075	XLOC_178481	217 coding	noncoding	noncoding	noncoding
MSTRG.175612.1		XLOC_178515	324 coding	noncoding	noncoding	noncoding
XR_245673.4	PCNX1	XLOC_178519	5015 coding	noncoding	coding	coding
MSTRG.175664.1	LOC105370557	XLOC_178529	316 coding	noncoding	noncoding	noncoding
NM_001354288.2	SIPA1L1	XLOC_178584	7998 coding	coding	coding	coding
MSTRG.175650.1		XLOC_178646	282 coding	noncoding	noncoding	noncoding
MSTRG.175859.1		XLOC_178648	240 coding	noncoding	noncoding	noncoding
MSTRG.175866.1	RGS6	XLOC_178652	317 coding	noncoding	noncoding	noncoding
MSTRG.175880.1	RGS6	XLOC_178665	285 coding	noncoding	noncoding	noncoding
MSTRG.175851.1	DCAF4	XLOC_178687	290 coding	noncoding	noncoding	noncoding
MSTRG.175852.1	DCAF4	XLOC_178688	291 coding	noncoding	noncoding	noncoding
XM_011536972.2	PSEN1	XLOC_178694	3058 coding	coding	coding	coding
MSTRG.175934.1	HEATR4	XLOC_178709	277 coding	noncoding	noncoding	noncoding
MSTRG.175945.1		XLOC_178715	240 coding	noncoding	noncoding	noncoding
MSTRG.176040.1	DNAL1	XLOC_178720	222 coding	noncoding	noncoding	noncoding
MSTRG.176149.16	COQ6	XLOC_178732	1380 coding	coding	coding	noncoding
XM_017021662.1	BBOF1	XLOC_178733	2664 coding	coding	coding	coding
XM_017021764.1	LIN52	XLOC_178735	942 coding	coding	coding	coding
MSTRG.176127.1		XLOC_178740	212 coding	noncoding	noncoding	noncoding
MSTRG.176201.1	AREL1	XLOC_178766	3222 coding	coding	coding	noncoding
XM_011536966.2	YLPM1	XLOC_178769	6176 coding	coding	coding	coding
NM_001244883.2	DLST	XLOC_178784	1371 coding	coding	coding	coding
MSTRG.176252.1		XLOC_178800	283 coding	noncoding	noncoding	noncoding
MSTRG.176288.1		XLOC_178821	255 coding	noncoding	coding	noncoding
MSTRG.176310.1	IFT43	XLOC_178855	206 coding	noncoding	noncoding	noncoding
XM_011536553.2	ESRRB	XLOC_178870	4943 coding	coding	coding	coding

MSTRG.176354.1	LOC105370577	XLOC_178874	245	coding	noncoding	noncoding	noncoding
NR_110554.1	LINC02288	XLOC_178881	2939	coding	noncoding	coding	noncoding
MSTRG.176423.1		XLOC_178893	303	coding	noncoding	noncoding	noncoding
MSTRG.176480.4	TMED8	XLOC_178905	4708	coding	coding	coding	noncoding
MSTRG.176495.3	VIPAS39	XLOC_178911	2367	coding	coding	noncoding	noncoding
MSTRG.176467.2	SPTLC2	XLOC_178913	8027	coding	coding	coding	noncoding
MSTRG.176464.1		XLOC_178914	257	coding	noncoding	noncoding	noncoding
NR_158973.1	NRXN3	XLOC_178925	13042	coding	coding	coding	coding
MSTRG.176569.1	NRXN3	XLOC_178926	289	coding	noncoding	noncoding	noncoding
MSTRG.176627.1	NRXN3	XLOC_178953	244	coding	noncoding	noncoding	noncoding
MSTRG.176531.1		XLOC_178959	291	coding	noncoding	coding	noncoding
MSTRG.176547.1	DIO2	XLOC_178965	339	coding	noncoding	noncoding	noncoding
MSTRG.176561.1	DIO2-AS1	XLOC_178973	312	coding	noncoding	noncoding	noncoding
MSTRG.176997.1	CEP128	XLOC_178991	243	coding	noncoding	noncoding	noncoding
XM_011537119.2	TSHR	XLOC_179063	4343	coding	coding	coding	coding
MSTRG.177096.1	TSHR	XLOC_179065	226	coding	noncoding	noncoding	noncoding
MSTRG.177124.1	LOC101928462	XLOC_179089	283	coding	noncoding	noncoding	noncoding
MSTRG.177129.1	LOC101928504	XLOC_179093	9126	coding	coding	coding	noncoding
MSTRG.176641.1		XLOC_179100	242	coding	noncoding	noncoding	noncoding
MSTRG.176659.1		XLOC_179108	290	coding	noncoding	noncoding	noncoding
MSTRG.176711.1		XLOC_179124	308	coding	noncoding	noncoding	noncoding
MSTRG.176712.1		XLOC_179125	313	coding	noncoding	noncoding	noncoding
MSTRG.176741.1		XLOC_179137	257	coding	noncoding	noncoding	noncoding
MSTRG.176751.1		XLOC_179142	255	coding	noncoding	noncoding	noncoding
MSTRG.176759.1		XLOC_179147	207	coding	coding	noncoding	noncoding
XM_024449522.1	FLRT2	XLOC_179180	10321	coding	coding	coding	coding
XM_017021130.1	FLRT2	XLOC_179180	17504	coding	coding	coding	coding
MSTRG.176820.1		XLOC_179183	274	coding	noncoding	noncoding	noncoding
MSTRG.176844.1		XLOC_179228	208	coding	noncoding	noncoding	noncoding
MSTRG.176882.1		XLOC_179250	272	coding	noncoding	noncoding	noncoding
XM_017021452.1	SPATA7	XLOC_179282	2178	coding	coding	coding	coding
MSTRG.177151.1	PTPN21	XLOC_179285	279	coding	noncoding	noncoding	noncoding
MSTRG.177152.1	PTPN21	XLOC_179286	249	coding	noncoding	noncoding	noncoding
MSTRG.177182.1	ZC3H14	XLOC_179289	293	coding	noncoding	noncoding	noncoding
MSTRG.177378.1		XLOC_179338	263	coding	noncoding	noncoding	noncoding
MSTRG.177380.1		XLOC_179340	209	coding	noncoding	noncoding	noncoding
MSTRG.177210.1		XLOC_179343	295	coding	noncoding	noncoding	noncoding
MSTRG.177277.1	TDP1	XLOC_179371	313	coding	noncoding	noncoding	noncoding
MSTRG.177298.2	NRDE2	XLOC_179384	424	coding	coding	coding	noncoding
MSTRG.177239.1		XLOC_179386	246	coding	noncoding	noncoding	noncoding
MSTRG.177286.1		XLOC_179394	249	coding	noncoding	noncoding	noncoding
MSTRG.177509.1	DGLUCY	XLOC_179411	255	coding	noncoding	noncoding	noncoding
MSTRG.177411.4	CCDC88C	XLOC_179420	1757	coding	coding	coding	coding

MSTRG.177411.6	CCDC88C	XLOC_179420	2556 coding	noncoding	coding	noncoding
MSTRG.177461.1	CPSF2	XLOC_179447	243 coding	noncoding	noncoding	noncoding
MSTRG.177524.1		XLOC_179496	224 coding	noncoding	noncoding	noncoding
XM_011537026.2	UNC79	XLOC_179515	11858 coding	coding	noncoding	coding
MSTRG.177632.1	SERPINA9	XLOC_179537	264 coding	coding	coding	noncoding
MSTRG.177643.1		XLOC_179547	305 coding	noncoding	noncoding	noncoding
MSTRG.177647.1		XLOC_179551	246 coding	noncoding	noncoding	noncoding
MSTRG.177681.1	LOC107984710	XLOC_179569	274 coding	noncoding	noncoding	noncoding
MSTRG.177682.1	LOC107984710	XLOC_179570	237 coding	noncoding	noncoding	noncoding
NM_001282463.2	C14orf132	XLOC_179599	7726 coding	noncoding	coding	coding
NM_000623.3	BDKRB2	XLOC_179604	4197 coding	coding	coding	coding
MSTRG.177830.6	ATG2B	XLOC_179608	3099 coding	coding	coding	noncoding
MSTRG.177807.1	AK7	XLOC_179612	254 coding	noncoding	noncoding	noncoding
MSTRG.177812.1	AK7	XLOC_179614	239 coding	noncoding	noncoding	noncoding
MSTRG.177870.1		XLOC_179639	238 coding	noncoding	noncoding	noncoding
MSTRG.177879.1		XLOC_179644	321 coding	noncoding	noncoding	noncoding
MSTRG.177902.1	LINC02291	XLOC_179659	522 coding	noncoding	noncoding	noncoding
MSTRG.178040.1	LOC105370655	XLOC_179707	464 coding	noncoding	noncoding	noncoding
MSTRG.178083.1		XLOC_179742	280 coding	noncoding	noncoding	noncoding
MSTRG.178084.1		XLOC_179743	289 coding	noncoding	noncoding	noncoding
MSTRG.178096.1		XLOC_179745	264 coding	coding	noncoding	noncoding
MSTRG.178209.3	BCL11B	XLOC_179812	6704 coding	coding	noncoding	noncoding
MSTRG.178158.1		XLOC_179824	265 coding	noncoding	noncoding	noncoding
XM_011537237.2	HHIPL1	XLOC_179841	2546 coding	coding	coding	coding
MSTRG.178244.1	HHIPL1	XLOC_179843	266 coding	noncoding	noncoding	noncoding
MSTRG.178249.1		XLOC_179851	222 coding	noncoding	noncoding	noncoding
MSTRG.178313.1	EVL	XLOC_179854	300 coding	noncoding	noncoding	noncoding
MSTRG.178284.1		XLOC_179864	281 coding	noncoding	noncoding	noncoding
MSTRG.178456.1	WDR25	XLOC_179891	283 coding	noncoding	noncoding	noncoding
MSTRG.178457.1	WDR25	XLOC_179892	217 coding	noncoding	noncoding	noncoding
MSTRG.178458.1	WDR25	XLOC_179893	230 coding	noncoding	noncoding	noncoding
MSTRG.178337.1	BEGAIN	XLOC_179894	323 coding	noncoding	noncoding	noncoding
MSTRG.178346.1		XLOC_179904	294 coding	noncoding	noncoding	noncoding
NR_046473.1	MEG3	XLOC_179908	9689 coding	noncoding	coding	coding
MSTRG.178375.1		XLOC_180015	232 coding	noncoding	noncoding	noncoding
MSTRG.178440.1		XLOC_180026	291 coding	noncoding	noncoding	noncoding
MSTRG.178484.1		XLOC_180065	280 coding	noncoding	noncoding	noncoding
MSTRG.178516.2	DYNC1H1	XLOC_180066	366 coding	noncoding	noncoding	noncoding
MSTRG.178518.1	DYNC1H1	XLOC_180068	408 coding	noncoding	noncoding	noncoding
MSTRG.178519.1	DYNC1H1	XLOC_180069	242 coding	noncoding	noncoding	noncoding
MSTRG.178520.1	DYNC1H1	XLOC_180070	358 coding	noncoding	coding	noncoding
MSTRG.178528.1	HSP90AA1	XLOC_180074	239 coding	noncoding	noncoding	noncoding
XM_011537347.1	WDR20	XLOC_180076	2838 coding	coding	coding	coding

MSTRG.178576.17	CINP	XLOC_180089	1224 coding	noncoding	coding	noncoding
MSTRG.178579.5	TECPR2	XLOC_180090	229 coding	noncoding	noncoding	noncoding
MSTRG.178591.1	LOC105370680	XLOC_180099	210 coding	noncoding	noncoding	noncoding
XR_943406.3	RCOR1	XLOC_180104	5760 coding	coding	coding	coding
MSTRG.178569.1	RCOR1	XLOC_180110	256 coding	noncoding	noncoding	noncoding
MSTRG.178571.1	RCOR1	XLOC_180112	243 coding	noncoding	noncoding	noncoding
XM_017021619.1	TRAF3	XLOC_180115	9619 coding	coding	coding	coding
XM_024449583.1	MARK3	XLOC_180150	3593 coding	coding	coding	coding
MSTRG.178686.1	MARK3	XLOC_180160	304 coding	noncoding	noncoding	noncoding
XR_002957597.1	LOC112268131	XLOC_180175	3550 coding	noncoding	coding	noncoding
MSTRG.178732.1	TDRD9	XLOC_180197	293 coding	noncoding	noncoding	noncoding
NM_015656.1	KIF26A	XLOC_180203	6757 coding	coding	coding	coding
NR_146611.1	LINC02691	XLOC_180206	3287 coding	noncoding	noncoding	noncoding
MSTRG.178787.1		XLOC_180225	279 coding	noncoding	noncoding	noncoding
MSTRG.178790.1		XLOC_180226	249 coding	noncoding	noncoding	noncoding
XM_017021851.1	LOC107987211	XLOC_180284	3078 coding	noncoding	coding	coding
MSTRG.171590.1		XLOC_180336	309 coding	noncoding	noncoding	noncoding
MSTRG.171621.1		XLOC_180343	390 coding	noncoding	noncoding	noncoding
MSTRG.171624.1		XLOC_180344	283 coding	noncoding	noncoding	noncoding
MSTRG.171636.1	LOC105378174	XLOC_180349	371 coding	noncoding	noncoding	noncoding
MSTRG.171631.1		XLOC_180350	277 coding	noncoding	noncoding	noncoding
MSTRG.171637.1		XLOC_180357	329 coding	noncoding	noncoding	noncoding
MSTRG.171648.1		XLOC_180363	237 coding	noncoding	noncoding	noncoding
MSTRG.171660.1		XLOC_180372	291 coding	noncoding	noncoding	noncoding
MSTRG.171681.1	TTC5	XLOC_180377	475 coding	noncoding	noncoding	noncoding
MSTRG.171667.1		XLOC_180384	225 coding	noncoding	noncoding	noncoding
MSTRG.171704.1		XLOC_180398	294 coding	noncoding	noncoding	noncoding
MSTRG.171706.1		XLOC_180400	265 coding	noncoding	noncoding	noncoding
MSTRG.171780.1		XLOC_180447	269 coding	noncoding	noncoding	noncoding
NM_004500.3	HNRNPC	XLOC_180453	3213 coding	coding	coding	coding
MSTRG.171823.1	RPGRIP1	XLOC_180465	211 coding	noncoding	noncoding	noncoding
MSTRG.171835.1		XLOC_180475	290 coding	noncoding	noncoding	noncoding
MSTRG.172250.33	TRAV1-1	XLOC_180531	2282 coding	coding	noncoding	noncoding
MSTRG.171852.1	DAD1	XLOC_180556	243 coding	noncoding	noncoding	noncoding
MSTRG.171858.1		XLOC_180563	379 coding	noncoding	noncoding	noncoding
MSTRG.171893.1	RBM23	XLOC_180586	318 coding	noncoding	noncoding	noncoding
MSTRG.171918.1	PRMT5	XLOC_180590	430 coding	noncoding	noncoding	noncoding
MSTRG.171919.1	LOC101926933	XLOC_180591	322 coding	noncoding	noncoding	noncoding
MSTRG.171947.1	RNF212B	XLOC_180608	271 coding	noncoding	noncoding	noncoding
MSTRG.171949.1	RNF212B	XLOC_180609	260 coding	noncoding	noncoding	noncoding
MSTRG.171990.1		XLOC_180642	295 coding	noncoding	noncoding	noncoding
XM_011537246.3	ZFHX2	XLOC_180644	11534 coding	coding	coding	coding
MSTRG.172038.18	DCAF11	XLOC_180666	2246 coding	coding	coding	coding

NM_014169.4	CHMP4A	XLOC_180681	1014 coding	coding	coding	coding
MSTRG.172056.2	TGM1	XLOC_180684	230 coding	noncoding	noncoding	noncoding
MSTRG.172069.1	NYNRIN	XLOC_180692	442 coding	noncoding	noncoding	noncoding
MSTRG.172070.1	NYNRIN	XLOC_180693	303 coding	noncoding	noncoding	noncoding
MSTRG.172130.1	STXBP6	XLOC_180717	400 coding	noncoding	noncoding	noncoding
MSTRG.172151.1		XLOC_180729	304 coding	noncoding	noncoding	noncoding
MSTRG.172154.1		XLOC_180731	247 coding	noncoding	noncoding	noncoding
MSTRG.172157.1		XLOC_180732	422 coding	coding	noncoding	noncoding
MSTRG.172172.1		XLOC_180740	290 coding	noncoding	noncoding	noncoding
NM_001366391.2	NOVA1	XLOC_180743	7092 coding	coding	coding	coding
MSTRG.172304.1	NOVA1	XLOC_180744	313 coding	noncoding	noncoding	noncoding
MSTRG.172292.1	LINC02588	XLOC_180746	212 coding	noncoding	noncoding	noncoding
MSTRG.172296.1	LINC02588	XLOC_180748	358 coding	noncoding	noncoding	noncoding
MSTRG.172338.1		XLOC_180771	244 coding	noncoding	noncoding	noncoding
MSTRG.172342.1		XLOC_180773	285 coding	noncoding	noncoding	noncoding
MSTRG.172347.1		XLOC_180776	224 coding	noncoding	noncoding	noncoding
MSTRG.172349.1		XLOC_180778	254 coding	noncoding	noncoding	noncoding
MSTRG.172387.1	LOC107984685	XLOC_180792	535 coding	noncoding	noncoding	noncoding
MSTRG.172370.1		XLOC_180797	213 coding	noncoding	noncoding	noncoding
MSTRG.172400.1	PRKD1	XLOC_180801	234 coding	noncoding	noncoding	noncoding
MSTRG.172417.1		XLOC_180812	224 coding	noncoding	noncoding	noncoding
MSTRG.172531.1	AP4S1	XLOC_180842	277 coding	noncoding	noncoding	noncoding
MSTRG.172470.1	HEATR5A	XLOC_180871	277 coding	noncoding	noncoding	noncoding
MSTRG.172472.1	HEATR5A	XLOC_180873	288 coding	noncoding	noncoding	noncoding
MSTRG.172576.1	LOC105370438	XLOC_180880	266 coding	noncoding	noncoding	noncoding
MSTRG.172579.1	LOC105370438	XLOC_180881	266 coding	noncoding	noncoding	noncoding
MSTRG.172555.1		XLOC_180960	233 coding	noncoding	noncoding	noncoding
MSTRG.172666.1	AKAP6	XLOC_180970	222 coding	noncoding	noncoding	noncoding
MSTRG.172723.1	NPAS3	XLOC_181003	301 coding	noncoding	noncoding	noncoding
MSTRG.172734.1	NPAS3	XLOC_181009	295 coding	noncoding	noncoding	noncoding
MSTRG.172570.1		XLOC_181016	262 coding	noncoding	noncoding	noncoding
MSTRG.172763.1		XLOC_181032	290 coding	noncoding	noncoding	noncoding
MSTRG.172795.1	SPTSSA	XLOC_181034	285 coding	noncoding	noncoding	noncoding
MSTRG.172801.1	EAPP	XLOC_181040	204 coding	noncoding	noncoding	noncoding
MSTRG.172807.1		XLOC_181047	266 coding	noncoding	noncoding	noncoding
MSTRG.172817.1		XLOC_181053	657 coding	noncoding	noncoding	noncoding
MSTRG.172859.3	FAM177A1	XLOC_181060	1507 coding	coding	coding	noncoding
MSTRG.172839.1	PSMA6	XLOC_181064	263 coding	noncoding	noncoding	noncoding
MSTRG.172836.1		XLOC_181071	252 coding	noncoding	noncoding	noncoding
MSTRG.173076.1	RALGAPA1	XLOC_181097	243 coding	noncoding	noncoding	noncoding
MSTRG.173077.1	RALGAPA1	XLOC_181098	279 coding	noncoding	noncoding	noncoding
MSTRG.173078.1	RALGAPA1	XLOC_181099	619 coding	noncoding	noncoding	noncoding
MSTRG.172882.1		XLOC_181116	250 coding	noncoding	noncoding	noncoding

MSTRG.172920.1	SLC25A21	XLOC_181131	244 coding	noncoding	noncoding	noncoding
MSTRG.172931.1	SLC25A21	XLOC_181136	207 coding	noncoding	noncoding	noncoding
MSTRG.172934.1	SLC25A21	XLOC_181138	262 coding	noncoding	noncoding	noncoding
MSTRG.172975.1	MIPOL1	XLOC_181151	261 coding	noncoding	noncoding	noncoding
MSTRG.172982.1	MIPOL1	XLOC_181156	236 coding	noncoding	noncoding	noncoding
MSTRG.172983.1	MIPOL1	XLOC_181157	254 coding	noncoding	noncoding	noncoding
MSTRG.172987.1	MIPOL1	XLOC_181160	298 coding	noncoding	noncoding	noncoding
MSTRG.172998.1	TTC6	XLOC_181165	263 coding	noncoding	noncoding	noncoding
MSTRG.173004.1	TTC6	XLOC_181168	303 coding	noncoding	noncoding	noncoding
MSTRG.173091.1		XLOC_181193	224 coding	noncoding	noncoding	noncoding
MSTRG.173125.1		XLOC_181204	390 coding	noncoding	noncoding	noncoding
MSTRG.173137.1		XLOC_181280	323 coding	noncoding	noncoding	noncoding
MSTRG.173141.1		XLOC_181282	236 coding	noncoding	noncoding	noncoding
MSTRG.173152.1		XLOC_181289	247 coding	noncoding	noncoding	noncoding
XR_001750740.2	LOC105370469	XLOC_181303	9141 coding	noncoding	coding	noncoding
MSTRG.173205.1		XLOC_181310	300 coding	coding	noncoding	noncoding
MSTRG.173208.1		XLOC_181313	303 coding	noncoding	noncoding	noncoding
MSTRG.173211.1		XLOC_181314	255 coding	noncoding	noncoding	noncoding
MSTRG.173228.1		XLOC_181319	203 coding	noncoding	noncoding	noncoding
MSTRG.173234.1		XLOC_181322	202 coding	noncoding	noncoding	noncoding
MSTRG.173256.1		XLOC_181333	598 coding	noncoding	coding	noncoding
MSTRG.173268.1		XLOC_181339	289 coding	noncoding	noncoding	noncoding
MSTRG.173278.1	LINC02307	XLOC_181345	235 coding	noncoding	noncoding	noncoding
MSTRG.173282.1		XLOC_181348	314 coding	noncoding	noncoding	noncoding
MSTRG.173292.1	LINC02277	XLOC_181353	218 coding	noncoding	noncoding	noncoding
MSTRG.173303.1		XLOC_181359	310 coding	noncoding	noncoding	noncoding
MSTRG.173306.1		XLOC_181364	256 coding	noncoding	noncoding	noncoding
MSTRG.173504.3	PRPF39	XLOC_181382	1057 coding	noncoding	noncoding	noncoding
MSTRG.173425.1	LOC105370476	XLOC_181404	298 coding	noncoding	noncoding	noncoding
MSTRG.173437.1		XLOC_181410	207 coding	coding	noncoding	noncoding
MSTRG.173440.1		XLOC_181412	532 coding	noncoding	noncoding	noncoding
MSTRG.173448.1	LINC00871	XLOC_181415	318 coding	noncoding	noncoding	noncoding
MSTRG.173480.1		XLOC_181421	326 coding	noncoding	noncoding	noncoding
MSTRG.173514.1		XLOC_181437	315 coding	coding	noncoding	noncoding
MSTRG.173555.1		XLOC_181439	222 coding	noncoding	noncoding	noncoding
MSTRG.173565.1	LOC101927483	XLOC_181445	276 coding	coding	noncoding	noncoding
MSTRG.173571.1		XLOC_181451	360 coding	noncoding	noncoding	noncoding
MSTRG.173576.1	LOC105378178	XLOC_181453	253 coding	noncoding	noncoding	noncoding
MSTRG.173581.1	LOC105378178	XLOC_181456	353 coding	coding	noncoding	noncoding
MSTRG.173582.1	LOC105378178	XLOC_181457	283 coding	noncoding	noncoding	noncoding
MSTRG.173605.1	LOC105378178	XLOC_181463	256 coding	coding	noncoding	noncoding
MSTRG.173612.5	LOC107987206	XLOC_181466	281 coding	noncoding	noncoding	noncoding
XR_943557.3	NEMF	XLOC_181488	2825 coding	coding	coding	coding

MSTRG.173680.4	LOC105378179	XLOC_181490	467 coding	noncoding	noncoding	noncoding
MSTRG.173680.1	LOC105378179	XLOC_181490	469 coding	noncoding	noncoding	noncoding
MSTRG.173680.3	LOC105378179	XLOC_181490	464 coding	noncoding	noncoding	noncoding
MSTRG.173680.9	LOC105378179	XLOC_181490	273 coding	noncoding	noncoding	noncoding
MSTRG.173680.10	LOC105378179	XLOC_181490	250 coding	noncoding	noncoding	noncoding
XR_943531.2	VCPKMT	XLOC_181493	1810 coding	coding	coding	coding
MSTRG.173642.1		XLOC_181508	277 coding	noncoding	noncoding	noncoding
MSTRG.173697.1	MAP4K5	XLOC_181511	265 coding	noncoding	noncoding	noncoding
XM_024449622.1	NIN	XLOC_181536	10338 coding	noncoding	coding	coding
MSTRG.173748.1	ABHD12B	XLOC_181564	287 coding	noncoding	noncoding	noncoding
MSTRG.173764.1	TRIM9	XLOC_181574	301 coding	noncoding	noncoding	noncoding
MSTRG.173739.1		XLOC_181578	272 coding	noncoding	noncoding	noncoding
MSTRG.173815.1		XLOC_181607	253 coding	noncoding	noncoding	noncoding
MSTRG.173909.1		XLOC_181630	318 coding	noncoding	noncoding	noncoding
MSTRG.173964.1	ERO1A	XLOC_181668	476 coding	noncoding	noncoding	noncoding
MSTRG.174001.1	LOC105370500	XLOC_181678	278 coding	noncoding	noncoding	noncoding
MSTRG.174311.1	LOC105370504	XLOC_181806	241 coding	noncoding	noncoding	noncoding
MSTRG.174315.1	LOC105370504	XLOC_181809	240 coding	noncoding	noncoding	noncoding
MSTRG.174014.1		XLOC_181817	243 coding	noncoding	noncoding	noncoding
NM_001024024.1	GCH1	XLOC_181866	1980 coding	coding	coding	coding
MSTRG.174108.1	GCH1	XLOC_181868	251 coding	coding	noncoding	noncoding
MSTRG.174109.1	GCH1	XLOC_181869	253 coding	noncoding	noncoding	noncoding
MSTRG.174111.1	GCH1	XLOC_181871	237 coding	noncoding	noncoding	noncoding
MSTRG.174113.1		XLOC_181885	231 coding	noncoding	noncoding	noncoding
MSTRG.174148.1		XLOC_181893	281 coding	noncoding	coding	noncoding
MSTRG.174149.1		XLOC_181897	264 coding	noncoding	noncoding	noncoding
MSTRG.174152.1		XLOC_181898	664 coding	noncoding	noncoding	noncoding
MSTRG.174593.1		XLOC_181943	267 coding	noncoding	noncoding	noncoding
MSTRG.174409.11	TMEM260	XLOC_181956	3374 coding	noncoding	noncoding	noncoding
MSTRG.174376.1		XLOC_181962	279 coding	noncoding	noncoding	noncoding
MSTRG.174466.1	SLC35F4	XLOC_182001	269 coding	noncoding	noncoding	noncoding
XM_017021030.2	ARMH4	XLOC_182003	13716 coding	coding	coding	coding
MSTRG.174485.1	ARMH4	XLOC_182011	231 coding	coding	noncoding	noncoding
MSTRG.174642.1	LOC105370522	XLOC_182026	262 coding	noncoding	noncoding	noncoding
MSTRG.174604.1		XLOC_182036	205 coding	noncoding	noncoding	noncoding
MSTRG.174742.12	PPM1A	XLOC_182108	3268 coding	coding	coding	noncoding
MSTRG.174968.9	PRKCH	XLOC_182153	2926 coding	coding	coding	coding
MSTRG.174896.1		XLOC_182182	278 coding	noncoding	noncoding	noncoding
MSTRG.174897.1		XLOC_182183	311 coding	coding	noncoding	noncoding
MSTRG.174899.1		XLOC_182184	411 coding	noncoding	noncoding	coding
MSTRG.174934.1	KCNH5	XLOC_182192	205 coding	noncoding	noncoding	noncoding
MSTRG.174935.1	KCNH5	XLOC_182193	295 coding	coding	noncoding	noncoding
MSTRG.174937.1	KCNH5	XLOC_182194	276 coding	noncoding	noncoding	noncoding

MSTRG.174961.1	RHOJ	XLOC_182206	367	coding	noncoding	noncoding	noncoding
MSTRG.174963.1		XLOC_182211	313	coding	noncoding	noncoding	noncoding
NR_104104.2	PPP2R5E	XLOC_182213	6484	coding	coding	coding	coding
MSTRG.175081.1	PPP2R5E	XLOC_182225	237	coding	noncoding	noncoding	noncoding
MSTRG.175055.1		XLOC_182229	260	coding	noncoding	noncoding	noncoding
MSTRG.175057.1		XLOC_182230	237	coding	noncoding	noncoding	noncoding
NM_030791.4	SGPP1	XLOC_182234	3336	coding	coding	coding	coding
MSTRG.175271.10	SYNE2	XLOC_182235	22053	coding	coding	coding	coding
MSTRG.175089.1		XLOC_182240	234	coding	noncoding	noncoding	noncoding
NR_033777.1	TEX21P	XLOC_182241	1045	coding	noncoding	noncoding	noncoding
MSTRG.175095.1		XLOC_182245	238	coding	noncoding	noncoding	noncoding
NM_145113.3	MAX	XLOC_182256	2111	coding	coding	coding	noncoding
MSTRG.175128.1		XLOC_182264	290	coding	noncoding	noncoding	noncoding
MSTRG.175129.1		XLOC_182266	297	coding	noncoding	noncoding	noncoding
MSTRG.175315.1	FUT8	XLOC_182285	1047	coding	noncoding	noncoding	noncoding
MSTRG.175149.1		XLOC_182297	262	coding	noncoding	noncoding	noncoding
MSTRG.175802.1	GPHN	XLOC_182333	288	coding	noncoding	noncoding	noncoding
NM_001348544.2	TMEM229B	XLOC_182335	4041	coding	coding	coding	coding
MSTRG.175389.1		XLOC_182392	354	coding	noncoding	noncoding	noncoding
XM_011537278.1	DCAF5	XLOC_182393	6356	coding	coding	coding	coding
MSTRG.175444.1	ERH	XLOC_182413	217	coding	noncoding	noncoding	noncoding
MSTRG.175447.4	SLC39A9	XLOC_182415	4852	coding	coding	coding	noncoding
NM_001271507.2	CCDC177	XLOC_182422	4182	coding	coding	coding	coding
MSTRG.175460.1		XLOC_182425	288	coding	noncoding	noncoding	noncoding
NM_033262.4	SLC8A3	XLOC_182446	5262	coding	coding	coding	coding
MSTRG.175528.1		XLOC_182461	239	coding	noncoding	noncoding	noncoding
MSTRG.175546.1	ADAM21	XLOC_182462	247	coding	noncoding	noncoding	noncoding
MSTRG.175574.1	LOC105370556	XLOC_182471	241	coding	noncoding	noncoding	noncoding
MSTRG.175588.1	MED6	XLOC_182479	249	coding	noncoding	coding	noncoding
MSTRG.175626.1	MAP3K9	XLOC_182487	270	coding	noncoding	noncoding	noncoding
MSTRG.175636.1	MAP3K9	XLOC_182490	233	coding	noncoding	noncoding	noncoding
MSTRG.176045.30	LOC102724015	XLOC_182505	5662	coding	coding	coding	noncoding
MSTRG.176045.48	SIPA1L1	XLOC_182505	1301	coding	noncoding	noncoding	coding
MSTRG.176045.51	SIPA1L1	XLOC_182505	1378	coding	noncoding	noncoding	noncoding
MSTRG.175652.1		XLOC_182515	251	coding	noncoding	noncoding	noncoding
MSTRG.175861.1	RGS6	XLOC_182516	254	coding	noncoding	noncoding	noncoding
MSTRG.175864.4	RGS6	XLOC_182518	326	coding	noncoding	noncoding	noncoding
MSTRG.175900.1	DPF3	XLOC_182526	234	coding	noncoding	noncoding	noncoding
MSTRG.175903.1	DPF3	XLOC_182528	506	coding	noncoding	noncoding	noncoding
MSTRG.175856.1	ZFYVE1	XLOC_182537	241	coding	noncoding	noncoding	noncoding
MSTRG.175910.7	LOC105370560	XLOC_182539	2996	coding	coding	noncoding	coding
MSTRG.175910.17	RBM25	XLOC_182539	2501	coding	coding	noncoding	noncoding
XM_006720143.2	HEATR4	XLOC_182551	3509	coding	coding	coding	coding

MSTRG.175933.1	HEATR4	XLOC_182552	277 coding	noncoding	noncoding	noncoding
MSTRG.176152.1	ENTPD5	XLOC_182581	258 coding	noncoding	noncoding	noncoding
MSTRG.176154.5	ALDH6A1	XLOC_182582	1863 coding	coding	coding	noncoding
MSTRG.176154.8	ALDH6A1	XLOC_182582	2091 coding	coding	coding	noncoding
NM_001278594.2	ALDH6A1	XLOC_182582	5625 coding	coding	coding	coding
MSTRG.176175.1	SYNDIG1L	XLOC_182593	286 coding	noncoding	noncoding	noncoding
MSTRG.176184.1	NPC2	XLOC_182599	282 coding	coding	noncoding	noncoding
MSTRG.176139.1		XLOC_182619	255 coding	noncoding	noncoding	noncoding
MSTRG.176266.1	NEK9	XLOC_182638	260 coding	noncoding	noncoding	noncoding
MSTRG.176257.1		XLOC_182643	252 coding	noncoding	noncoding	noncoding
MSTRG.176289.1		XLOC_182655	255 coding	noncoding	noncoding	noncoding
MSTRG.176385.2	BATF	XLOC_182658	648 coding	coding	noncoding	noncoding
MSTRG.176348.1	ESRRB	XLOC_182691	291 coding	noncoding	noncoding	noncoding
MSTRG.176370.1	LRRC74A	XLOC_182707	303 coding	noncoding	coding	noncoding
MSTRG.176426.1		XLOC_182710	311 coding	noncoding	noncoding	noncoding
XM_017021843.2	LOC105370579	XLOC_182714	3532 coding	noncoding	coding	coding
MSTRG.176422.1		XLOC_182725	303 coding	noncoding	noncoding	noncoding
MSTRG.176447.1	TMEM63C	XLOC_182733	273 coding	noncoding	noncoding	noncoding
MSTRG.176482.1	TMED8	XLOC_182744	277 coding	noncoding	noncoding	noncoding
MSTRG.176484.1	TMED8	XLOC_182746	307 coding	noncoding	noncoding	noncoding
MSTRG.176485.1	TMED8	XLOC_182747	235 coding	noncoding	noncoding	noncoding
NM_199296.3	ISM2	XLOC_182755	2940 coding	coding	coding	coding
MSTRG.176471.1	SPTLC2	XLOC_182759	225 coding	noncoding	noncoding	noncoding
MSTRG.176473.1	SPTLC2	XLOC_182761	230 coding	noncoding	noncoding	noncoding
MSTRG.176463.1		XLOC_182767	257 coding	noncoding	noncoding	noncoding
MSTRG.176514.1	ADCK1	XLOC_182775	232 coding	noncoding	noncoding	noncoding
MSTRG.176572.1	NRXN3	XLOC_182782	243 coding	noncoding	noncoding	noncoding
MSTRG.176582.1	NRXN3	XLOC_182789	246 coding	noncoding	noncoding	noncoding
XR_944068.3	LOC105370589	XLOC_182793	2005 coding	coding	coding	coding
XR_001751014.1	LOC107984629	XLOC_182800	8088 coding	coding	noncoding	noncoding
NM_001324462.2	DIO2	XLOC_182832	6383 coding	coding	coding	noncoding
XM_011536495.2	CEP128	XLOC_182842	3950 coding	noncoding	coding	coding
MSTRG.176996.1	CEP128	XLOC_182844	243 coding	noncoding	noncoding	noncoding
MSTRG.177037.1	CEP128	XLOC_182852	323 coding	noncoding	noncoding	noncoding
MSTRG.177087.1	CEP128	XLOC_182864	294 coding	noncoding	noncoding	noncoding
MSTRG.176648.1	STON2	XLOC_182891	238 coding	noncoding	noncoding	noncoding
MSTRG.176692.1	SEL1L	XLOC_182903	612 coding	noncoding	noncoding	noncoding
MSTRG.176642.1		XLOC_182908	242 coding	noncoding	noncoding	noncoding
MSTRG.176670.1		XLOC_182921	249 coding	noncoding	noncoding	noncoding
MSTRG.176713.1		XLOC_182935	313 coding	noncoding	noncoding	noncoding
MSTRG.176744.1		XLOC_182953	248 coding	noncoding	coding	noncoding
MSTRG.176749.1		XLOC_182956	302 coding	coding	noncoding	noncoding
MSTRG.176780.1		XLOC_182970	298 coding	noncoding	noncoding	noncoding

MSTRG.176803.1	LOC105370604	XLOC_182980	318 coding	noncoding	noncoding	noncoding
XR_110231.7	LOC100506731	XLOC_182988	5150 coding	noncoding	noncoding	noncoding
MSTRG.176836.1		XLOC_183004	245 coding	noncoding	noncoding	noncoding
MSTRG.176849.1		XLOC_183011	235 coding	noncoding	noncoding	noncoding
MSTRG.176861.1		XLOC_183018	312 coding	noncoding	noncoding	noncoding
MSTRG.176883.1		XLOC_183028	272 coding	noncoding	noncoding	noncoding
MSTRG.176974.1	LOC105370612	XLOC_183051	320 coding	coding	noncoding	noncoding
MSTRG.177142.2	SPATA7	XLOC_183057	415 coding	coding	noncoding	noncoding
MSTRG.177361.1	FOXN3	XLOC_183117	301 coding	noncoding	noncoding	noncoding
MSTRG.177211.1		XLOC_183129	285 coding	noncoding	noncoding	noncoding
MSTRG.177237.1	KCNK13	XLOC_183155	225 coding	noncoding	noncoding	noncoding
MSTRG.177287.1		XLOC_183164	326 coding	noncoding	noncoding	noncoding
MSTRG.177291.1		XLOC_183165	218 coding	noncoding	noncoding	noncoding
MSTRG.177310.1	TTC7B	XLOC_183172	289 coding	noncoding	noncoding	noncoding
MSTRG.177490.1	RPS6KA5	XLOC_183199	259 coding	noncoding	noncoding	noncoding
MSTRG.177505.14	GPR68	XLOC_183212	12556 coding	coding	coding	coding
MSTRG.177505.18	SNORA11B	XLOC_183212	4256 coding	noncoding	coding	noncoding
MSTRG.177505.28	GPR68	XLOC_183212	7084 coding	coding	coding	noncoding
MSTRG.177416.1	CCDC88C	XLOC_183220	252 coding	noncoding	noncoding	noncoding
MSTRG.177421.1	CCDC88C	XLOC_183224	306 coding	noncoding	noncoding	noncoding
MSTRG.177394.1	PPP4R3A	XLOC_183235	222 coding	noncoding	noncoding	noncoding
NM_001128595.3	TC2N	XLOC_183244	4968 coding	coding	noncoding	coding
MSTRG.177448.1		XLOC_183249	259 coding	noncoding	noncoding	noncoding
MSTRG.177433.1	ATXN3	XLOC_183254	340 coding	noncoding	noncoding	noncoding
MSTRG.177532.1	LGMN	XLOC_183281	232 coding	noncoding	noncoding	noncoding
MSTRG.177642.1		XLOC_183331	536 coding	noncoding	noncoding	noncoding
MSTRG.177646.1		XLOC_183334	308 coding	noncoding	noncoding	noncoding
XM_011536601.3	DICER1	XLOC_183345	10482 coding	coding	coding	coding
MSTRG.177719.1		XLOC_183417	436 coding	noncoding	noncoding	noncoding
MSTRG.177811.1	AK7	XLOC_183442	239 coding	noncoding	noncoding	noncoding
MSTRG.178034.1	LOC105370655	XLOC_183559	280 coding	noncoding	noncoding	noncoding
MSTRG.178111.1		XLOC_183593	216 coding	noncoding	noncoding	noncoding
MSTRG.178314.1	EVL	XLOC_183670	300 coding	noncoding	noncoding	noncoding
MSTRG.178281.1		XLOC_183676	333 coding	noncoding	noncoding	noncoding
XM_024449706.1	WARS	XLOC_183682	2982 coding	coding	coding	coding
MSTRG.178349.1	DLK1	XLOC_183697	335 coding	coding	noncoding	noncoding
MSTRG.178345.1		XLOC_183700	294 coding	noncoding	noncoding	noncoding
MSTRG.178358.1	MEG3	XLOC_183706	261 coding	noncoding	noncoding	noncoding
MSTRG.178363.1		XLOC_183717	306 coding	noncoding	noncoding	noncoding
MSTRG.178438.1		XLOC_183733	305 coding	noncoding	noncoding	noncoding
XR_001750894.1	LOC107984698	XLOC_183734	1471 coding	noncoding	coding	noncoding
MSTRG.178464.1		XLOC_183743	237 coding	noncoding	noncoding	noncoding
MSTRG.178489.10	PPP2R5C	XLOC_183744	8043 coding	coding	coding	noncoding

MSTRG.178487.1		XLOC_183750	291 coding	noncoding	noncoding	noncoding
MSTRG.178515.2	LOC107984661	XLOC_183751	14411 coding	coding	coding	coding
MSTRG.178515.3	LOC107984661	XLOC_183751	12218 coding	coding	coding	coding
XR_943504.2	MOK	XLOC_183759	2111 coding	coding	coding	coding
MSTRG.178546.1	MOK	XLOC_183761	253 coding	noncoding	noncoding	noncoding
MSTRG.178547.1	MOK	XLOC_183762	232 coding	noncoding	noncoding	noncoding
MSTRG.178618.4	TRAF3	XLOC_183782	2463 coding	coding	noncoding	coding
MSTRG.178618.5	TRAF3	XLOC_183782	580 coding	noncoding	noncoding	noncoding
MSTRG.178600.1	CDC42BPB	XLOC_183785	276 coding	noncoding	noncoding	noncoding
MSTRG.178612.1	CDC42BPB	XLOC_183788	255 coding	noncoding	noncoding	noncoding
MSTRG.178627.1		XLOC_183795	227 coding	noncoding	noncoding	noncoding
MSTRG.178672.1		XLOC_183804	244 coding	noncoding	noncoding	noncoding
MSTRG.178689.1	LOC112268130	XLOC_183808	1586 coding	noncoding	coding	coding
MSTRG.178698.2	PPP1R13B	XLOC_183831	970 coding	coding	coding	noncoding
MSTRG.178733.1	TDRD9	XLOC_183840	304 coding	noncoding	noncoding	noncoding
MSTRG.178728.1		XLOC_183852	247 coding	noncoding	noncoding	noncoding
MSTRG.178812.1		XLOC_183894	298 coding	noncoding	noncoding	noncoding
MSTRG.178815.1		XLOC_183896	270 coding	noncoding	noncoding	noncoding
MSTRG.178864.1	BRF1	XLOC_183903	366 coding	noncoding	noncoding	noncoding
MSTRG.178879.1	PACS2	XLOC_183914	287 coding	coding	noncoding	noncoding
MSTRG.178844.1	TEDC1	XLOC_183927	266 coding	noncoding	noncoding	noncoding
MSTRG.178834.1		XLOC_183930	318 coding	noncoding	noncoding	noncoding
IGHG4	IGHG4	XLOC_183931	983 coding	coding	coding	coding
MSTRG.178897.1		XLOC_183948	304 coding	noncoding	noncoding	noncoding
MSTRG.178916.5	HERC2P3	XLOC_183960	2345 coding	coding	noncoding	noncoding
XM_017022654.2	NIPA2	XLOC_184000	3197 coding	coding	coding	coding
MSTRG.178974.1		XLOC_184011	322 coding	noncoding	noncoding	noncoding
MSTRG.179388.32	UBE3A	XLOC_184024	4456 coding	coding	noncoding	noncoding
MSTRG.179491.1	SNHG14	XLOC_184105	249 coding	noncoding	noncoding	noncoding
MSTRG.179074.1		XLOC_184134	293 coding	noncoding	noncoding	noncoding
XM_017022055.1	GABRA5	XLOC_184148	6553 coding	coding	coding	coding
XM_011521431.3	GABRG3	XLOC_184151	9313 coding	coding	coding	coding
MSTRG.179133.1	GABRG3	XLOC_184156	516 coding	noncoding	noncoding	noncoding
MSTRG.179134.1	GABRG3	XLOC_184157	285 coding	noncoding	noncoding	noncoding
MSTRG.179158.1	OCA2	XLOC_184169	280 coding	noncoding	noncoding	noncoding
MSTRG.179208.1		XLOC_184195	498 coding	noncoding	noncoding	noncoding
MSTRG.179291.2	FAN1	XLOC_184229	8437 coding	coding	coding	coding
MSTRG.179291.16	FAN1	XLOC_184229	5465 coding	coding	coding	coding
MSTRG.179286.1	OTUD7A	XLOC_184241	259 coding	noncoding	noncoding	noncoding
MSTRG.179289.1		XLOC_184246	243 coding	noncoding	noncoding	noncoding
MSTRG.179318.1	LINC02256	XLOC_184264	276 coding	noncoding	noncoding	noncoding
NM_199357.2	ARHGAP11A	XLOC_184266	2485 coding	coding	coding	coding
MSTRG.179325.1		XLOC_184304	299 coding	noncoding	noncoding	noncoding

MSTRG.179539.1	RYR3	XLOC_184308	281	coding	noncoding	noncoding	noncoding
MSTRG.179600.13	SLC12A6	XLOC_184337	996	coding	noncoding	noncoding	noncoding
MSTRG.179576.5	LPCAT4	XLOC_184341	225	coding	noncoding	noncoding	noncoding
MSTRG.179582.1		XLOC_184343	227	coding	coding	noncoding	noncoding
MSTRG.179631.1		XLOC_184357	260	coding	noncoding	noncoding	noncoding
MSTRG.179661.1		XLOC_184409	218	coding	noncoding	noncoding	noncoding
MSTRG.179843.1	C15orf41	XLOC_184438	233	coding	noncoding	noncoding	noncoding
MSTRG.179730.1	LOC105370772	XLOC_184469	289	coding	noncoding	noncoding	noncoding
MSTRG.179734.1		XLOC_184473	258	coding	noncoding	noncoding	noncoding
MSTRG.179818.1		XLOC_184477	276	coding	noncoding	noncoding	noncoding
NM_001330255.1	TMCO5A	XLOC_184481	1958	coding	coding	coding	coding
MSTRG.179821.1		XLOC_184482	295	coding	noncoding	noncoding	noncoding
MSTRG.179840.1		XLOC_184490	254	coding	noncoding	noncoding	noncoding
MSTRG.179875.1		XLOC_184497	216	coding	noncoding	noncoding	noncoding
MSTRG.179902.1		XLOC_184499	289	coding	noncoding	noncoding	noncoding
MSTRG.179913.1	LOC107984725	XLOC_184504	247	coding	noncoding	noncoding	noncoding
MSTRG.180058.1		XLOC_184548	234	coding	coding	noncoding	noncoding
MSTRG.180105.1		XLOC_184594	294	coding	noncoding	noncoding	noncoding
MSTRG.180123.1	PAK6	XLOC_184600	233	coding	noncoding	noncoding	noncoding
MSTRG.180154.1	PHGR1	XLOC_184609	227	coding	noncoding	noncoding	noncoding
MSTRG.180164.1		XLOC_184615	245	coding	noncoding	noncoding	noncoding
MSTRG.180175.1		XLOC_184621	262	coding	noncoding	noncoding	noncoding
MSTRG.180230.1		XLOC_184645	206	coding	noncoding	noncoding	noncoding
MSTRG.180231.1		XLOC_184646	250	coding	noncoding	noncoding	noncoding
MSTRG.180256.1	DNAJC17	XLOC_184657	258	coding	noncoding	noncoding	noncoding
MSTRG.180241.1	PPP1R14D	XLOC_184660	224	coding	noncoding	noncoding	noncoding
MSTRG.180295.1	CHP1	XLOC_184674	218	coding	noncoding	noncoding	noncoding
MSTRG.180296.1	CHP1	XLOC_184675	284	coding	noncoding	noncoding	noncoding
MSTRG.180299.1	OIP5-AS1	XLOC_184678	234	coding	noncoding	noncoding	noncoding
MSTRG.180305.1	NUSAP1	XLOC_184681	241	coding	noncoding	noncoding	noncoding
MSTRG.180306.1		XLOC_184682	368	coding	noncoding	noncoding	noncoding
MSTRG.180310.1		XLOC_184684	249	coding	noncoding	noncoding	noncoding
MSTRG.180312.1		XLOC_184685	304	coding	noncoding	noncoding	noncoding
MSTRG.180314.1		XLOC_184686	270	coding	noncoding	noncoding	noncoding
MSTRG.180282.1	LTK	XLOC_184691	460	coding	noncoding	coding	noncoding
NM_006293.4	TYRO3	XLOC_184695	8032	coding	coding	coding	coding
MSTRG.180327.1	MGA	XLOC_184700	238	coding	noncoding	noncoding	noncoding
NM_001114633.1	PLA2G4B	XLOC_184706	2735	coding	coding	coding	coding
MSTRG.180350.1	SPTBN5	XLOC_184708	302	coding	coding	noncoding	noncoding
MSTRG.180360.1	PLA2G4E	XLOC_184712	240	coding	noncoding	noncoding	noncoding
MSTRG.180440.1	VPS39	XLOC_184717	6248	coding	coding	coding	noncoding
MSTRG.180457.1		XLOC_184725	248	coding	noncoding	noncoding	noncoding
XM_011521836.2	STARD9	XLOC_184740	15914	coding	coding	noncoding	coding

MSTRG.180400.1	TGM5	XLOC_184759	309 coding	noncoding	noncoding	noncoding
MSTRG.180509.1	SERF2	XLOC_184788	274 coding	noncoding	noncoding	noncoding
MSTRG.180518.1	WDR76	XLOC_184794	229 coding	noncoding	noncoding	noncoding
NM_177974.3	CASC4	XLOC_184807	3806 coding	noncoding	coding	coding
MSTRG.180581.1		XLOC_184814	331 coding	noncoding	noncoding	noncoding
NM_182985.5	TRIM69	XLOC_184838	1735 coding	coding	coding	coding
MSTRG.180622.1		XLOC_184844	306 coding	noncoding	noncoding	noncoding
MSTRG.180645.1	DUOX2	XLOC_184867	318 coding	coding	noncoding	noncoding
MSTRG.180661.1		XLOC_184886	227 coding	noncoding	noncoding	noncoding
MSTRG.180686.1	LOC107987223	XLOC_184895	253 coding	noncoding	noncoding	noncoding
MSTRG.180770.1	BLOC1S6	XLOC_184905	267 coding	noncoding	noncoding	noncoding
MSTRG.180772.1		XLOC_184907	296 coding	noncoding	noncoding	noncoding
MSTRG.180733.1	LOC105370802	XLOC_184915	450 coding	noncoding	noncoding	noncoding
MSTRG.180727.1		XLOC_184921	300 coding	noncoding	noncoding	noncoding
MSTRG.180752.1		XLOC_184931	281 coding	noncoding	noncoding	noncoding
MSTRG.180753.1		XLOC_184932	417 coding	coding	noncoding	noncoding
MSTRG.180784.1		XLOC_184939	265 coding	noncoding	noncoding	noncoding
MSTRG.180792.1	LINC01491	XLOC_184953	253 coding	noncoding	noncoding	noncoding
MSTRG.180813.1	LOC105370805	XLOC_184964	313 coding	noncoding	noncoding	noncoding
MSTRG.180982.15	SLC12A1	XLOC_184977	9361 coding	coding	noncoding	noncoding
MSTRG.180982.17	SLC12A1	XLOC_184977	2671 coding	coding	noncoding	noncoding
MSTRG.180872.1		XLOC_185024	260 coding	noncoding	noncoding	noncoding
MSTRG.180874.1		XLOC_185025	637 coding	noncoding	noncoding	noncoding
NM_001352047.1	GALK2	XLOC_185035	2923 coding	coding	coding	coding
MSTRG.181076.1	GALK2	XLOC_185040	404 coding	noncoding	noncoding	noncoding
MSTRG.180946.1	SLC27A2	XLOC_185093	237 coding	noncoding	noncoding	noncoding
MSTRG.180947.1	SLC27A2	XLOC_185094	359 coding	noncoding	noncoding	noncoding
MSTRG.180959.1		XLOC_185101	278 coding	noncoding	noncoding	noncoding
MSTRG.180964.1		XLOC_185102	961 coding	coding	coding	coding
MSTRG.181114.1		XLOC_185118	219 coding	noncoding	noncoding	noncoding
MSTRG.181170.1		XLOC_185122	324 coding	noncoding	noncoding	noncoding
NM_001252127.2	AP4E1	XLOC_185127	6766 coding	coding	coding	coding
MSTRG.181158.1	CYP19A1	XLOC_185138	241 coding	noncoding	noncoding	noncoding
XM_011521501.2	GLDN	XLOC_185145	6931 coding	coding	coding	coding
MSTRG.181237.7	DMXL2	XLOC_185152	2426 coding	coding	noncoding	noncoding
MSTRG.181254.1	LOC105370816	XLOC_185156	269 coding	noncoding	noncoding	noncoding
XM_017022088.1	TMOD3	XLOC_185167	4475 coding	coding	coding	coding
MSTRG.181206.1	MAPK6	XLOC_185182	274 coding	noncoding	noncoding	noncoding
MSTRG.181278.1	LOC107983981	XLOC_185213	289 coding	noncoding	noncoding	noncoding
MSTRG.181268.1		XLOC_185220	276 coding	noncoding	noncoding	noncoding
MSTRG.181297.1	LOC105370826	XLOC_185223	249 coding	noncoding	noncoding	noncoding
MSTRG.181366.1	UNC13C	XLOC_185236	215 coding	noncoding	noncoding	noncoding
MSTRG.181367.1	UNC13C	XLOC_185237	305 coding	noncoding	noncoding	noncoding

MSTRG.181313.1		XLOC_185245	251 coding	coding	noncoding	noncoding
MSTRG.181314.1		XLOC_185246	222 coding	noncoding	noncoding	noncoding
MSTRG.181415.1	PRTG	XLOC_185279	250 coding	noncoding	noncoding	noncoding
MSTRG.181425.1		XLOC_185285	250 coding	noncoding	noncoding	noncoding
MSTRG.181718.1	TCF12	XLOC_185356	267 coding	noncoding	noncoding	noncoding
XM_017022685.1	CGNL1	XLOC_185364	7858 coding	coding	coding	coding
MSTRG.181547.1	CGNL1	XLOC_185367	214 coding	noncoding	noncoding	noncoding
MSTRG.181551.1	CGNL1	XLOC_185370	279 coding	noncoding	noncoding	noncoding
MSTRG.181665.1	GCOM1	XLOC_185382	291 coding	noncoding	noncoding	noncoding
MSTRG.181560.1		XLOC_185384	261 coding	noncoding	noncoding	noncoding
XM_017022176.1	LIPC	XLOC_185465	3365 coding	coding	coding	coding
MSTRG.182046.1	LIPC	XLOC_185470	209 coding	noncoding	noncoding	noncoding
XM_011521688.2	MINDY2	XLOC_185476	9345 coding	coding	coding	coding
MSTRG.181756.1	MINDY2	XLOC_185478	234 coding	noncoding	noncoding	noncoding
MSTRG.181759.1	MINDY2	XLOC_185481	263 coding	noncoding	noncoding	noncoding
MSTRG.181761.1		XLOC_185485	271 coding	noncoding	noncoding	noncoding
MSTRG.181765.1		XLOC_185486	279 coding	noncoding	noncoding	noncoding
MSTRG.181854.1	FAM81A	XLOC_185509	379 coding	noncoding	noncoding	noncoding
MSTRG.181856.1	FAM81A	XLOC_185510	251 coding	noncoding	noncoding	noncoding
MSTRG.181778.1		XLOC_185513	257 coding	noncoding	noncoding	noncoding
MSTRG.181813.1		XLOC_185522	282 coding	noncoding	noncoding	noncoding
MSTRG.181815.1		XLOC_185524	271 coding	noncoding	noncoding	noncoding
MSTRG.181818.1		XLOC_185527	701 coding	noncoding	noncoding	noncoding
MSTRG.181831.1		XLOC_185533	216 coding	coding	noncoding	noncoding
MSTRG.182184.7	RORA-AS1	XLOC_185541	13970 coding	coding	noncoding	noncoding
MSTRG.181896.1		XLOC_185595	276 coding	noncoding	noncoding	noncoding
MSTRG.181900.1		XLOC_185596	936 coding	noncoding	noncoding	noncoding
MSTRG.181905.1		XLOC_185601	237 coding	noncoding	noncoding	noncoding
MSTRG.182076.1	LOC105370855	XLOC_185610	253 coding	noncoding	noncoding	noncoding
MSTRG.182093.1	LOC105370854	XLOC_185614	237 coding	noncoding	noncoding	noncoding
MSTRG.182059.1		XLOC_185619	288 coding	noncoding	noncoding	noncoding
XR_429442.2	LACTB	XLOC_185620	1963 coding	coding	coding	coding
MSTRG.182112.1	LOC101928972	XLOC_185627	260 coding	noncoding	noncoding	noncoding
MSTRG.182167.1		XLOC_185645	314 coding	noncoding	noncoding	noncoding
XM_017022765.1	USP3	XLOC_185650	5740 coding	coding	coding	coding
MSTRG.182336.4	HERC1	XLOC_185658	13500 coding	coding	coding	coding
MSTRG.182300.2	LOC101928988	XLOC_185662	1704 coding	coding	noncoding	coding
NM_148955.4	SNX1	XLOC_185665	8019 coding	coding	coding	coding
XM_017022581.1	SNX22	XLOC_185669	3540 coding	coding	coding	coding
MSTRG.182439.5	CSNK1G1	XLOC_185670	1335 coding	coding	noncoding	noncoding
MSTRG.182311.1		XLOC_185695	301 coding	noncoding	noncoding	noncoding
MSTRG.182323.1		XLOC_185701	281 coding	noncoding	noncoding	noncoding
MSTRG.182333.1		XLOC_185707	232 coding	noncoding	noncoding	noncoding

MSTRG.182374.1	KBTBD13	XLOC_185719	348	coding	noncoding	noncoding	noncoding
MSTRG.182502.1	RNU5A-1	XLOC_185741	235	coding	noncoding	coding	noncoding
MSTRG.182427.1		XLOC_185743	399	coding	noncoding	coding	noncoding
MSTRG.182430.1		XLOC_185748	282	coding	noncoding	noncoding	noncoding
MSTRG.182504.9	DPP8	XLOC_185749	2004	coding	coding	noncoding	noncoding
MSTRG.182504.10	DPP8	XLOC_185749	1354	coding	coding	noncoding	noncoding
MSTRG.182597.29	DENND4A	XLOC_185755	243	coding	noncoding	noncoding	noncoding
MSTRG.182597.37	DENND4A	XLOC_185755	4510	coding	coding	noncoding	noncoding
MSTRG.182559.1	MAP2K1	XLOC_185776	303	coding	noncoding	noncoding	noncoding
MSTRG.182546.1		XLOC_185787	267	coding	noncoding	noncoding	noncoding
MSTRG.182547.1		XLOC_185788	315	coding	noncoding	noncoding	noncoding
MSTRG.182571.1		XLOC_185792	264	coding	noncoding	noncoding	noncoding
MSTRG.182572.1		XLOC_185793	302	coding	noncoding	noncoding	noncoding
MSTRG.182582.1	LOC102723493	XLOC_185796	231	coding	noncoding	noncoding	noncoding
MSTRG.182583.1	LOC102723493	XLOC_185797	229	coding	noncoding	noncoding	noncoding
MSTRG.182762.1	MAP2K5	XLOC_185849	268	coding	noncoding	noncoding	noncoding
MSTRG.182632.1		XLOC_185872	267	coding	noncoding	noncoding	noncoding
MSTRG.182639.1		XLOC_185883	269	coding	noncoding	coding	noncoding
NM_001184780.2	NOX5	XLOC_185893	8423	coding	coding	coding	coding
MSTRG.182808.1	GLCE	XLOC_185905	275	coding	noncoding	noncoding	noncoding
MSTRG.182851.1		XLOC_185943	247	coding	noncoding	noncoding	noncoding
MSTRG.182919.1		XLOC_185953	227	coding	noncoding	noncoding	noncoding
MSTRG.182920.1		XLOC_185954	263	coding	noncoding	noncoding	noncoding
MSTRG.182926.1		XLOC_185957	205	coding	noncoding	noncoding	noncoding
MSTRG.182946.1		XLOC_185965	284	coding	noncoding	noncoding	noncoding
MSTRG.182997.1	LRRC49	XLOC_185986	315	coding	noncoding	noncoding	noncoding
MSTRG.183018.1	THSD4	XLOC_185994	395	coding	noncoding	noncoding	noncoding
MSTRG.183019.1	THSD4	XLOC_185995	281	coding	noncoding	coding	noncoding
MSTRG.183023.1	THSD4	XLOC_185999	293	coding	noncoding	noncoding	noncoding
MSTRG.183029.1	THSD4	XLOC_186003	259	coding	noncoding	noncoding	noncoding
MSTRG.183033.1	THSD4	XLOC_186005	218	coding	noncoding	noncoding	noncoding
MSTRG.183109.2	MYO9A	XLOC_186011	5374	coding	coding	noncoding	noncoding
MSTRG.183109.5	MYO9A	XLOC_186011	1085	coding	coding	noncoding	noncoding
MSTRG.183131.1	SENP8	XLOC_186014	226	coding	noncoding	noncoding	noncoding
NR_027262.1	HEXA-AS1	XLOC_186027	2676	coding	noncoding	noncoding	noncoding
MSTRG.183061.1		XLOC_186029	424	coding	noncoding	noncoding	noncoding
MSTRG.183155.9	ADPGK-AS1	XLOC_186055	2497	coding	coding	coding	noncoding
MSTRG.183164.1	HCN4	XLOC_186074	282	coding	noncoding	noncoding	noncoding
MSTRG.183239.1	TBC1D21	XLOC_186103	296	coding	noncoding	noncoding	noncoding
NM_001354988.2	CSK	XLOC_186151	2469	coding	coding	coding	coding
MSTRG.183354.1		XLOC_186162	296	coding	noncoding	noncoding	noncoding
MSTRG.183443.1		XLOC_186195	240	coding	noncoding	noncoding	noncoding
MSTRG.183526.3	SIN3A	XLOC_186199	4989	coding	coding	coding	coding

XR_932489.2	LOC441728	XLOC_186213	5236	coding	coding	coding	coding
MSTRG.183494.1		XLOC_186231	261	coding	noncoding	noncoding	noncoding
NM_002902.3	RCN2	XLOC_186236	6218	coding	coding	coding	coding
NM_018200.3	HMG20A	XLOC_186257	4135	coding	coding	coding	coding
MSTRG.183743.1	HMG20A	XLOC_186261	261	coding	noncoding	noncoding	noncoding
MSTRG.183589.1		XLOC_186285	285	coding	noncoding	noncoding	noncoding
MSTRG.183686.6	DNAJA4	XLOC_186295	1854	coding	noncoding	noncoding	coding
MSTRG.183683.1		XLOC_186304	251	coding	noncoding	noncoding	noncoding
MSTRG.183708.1	IREB2	XLOC_186315	276	coding	noncoding	noncoding	noncoding
MSTRG.183755.1		XLOC_186327	218	coding	noncoding	noncoding	noncoding
MSTRG.183757.1		XLOC_186328	270	coding	noncoding	noncoding	noncoding
MSTRG.183876.1		XLOC_186399	260	coding	noncoding	noncoding	noncoding
NM_001242917.1	ZFAND6	XLOC_186401	1659	coding	coding	noncoding	coding
XM_024449872.1	FAH	XLOC_186415	1522	coding	coding	coding	coding
MSTRG.183888.1	CTXND1	XLOC_186417	260	coding	noncoding	noncoding	noncoding
XM_006720408.2	CFAP161	XLOC_186438	1728	coding	noncoding	coding	coding
NR_148035.1	IL16	XLOC_186440	8176	coding	coding	coding	coding
MSTRG.184099.1	IL16	XLOC_186442	277	coding	noncoding	noncoding	noncoding
MSTRG.184036.1		XLOC_186456	328	coding	noncoding	noncoding	noncoding
MSTRG.184054.1		XLOC_186457	251	coding	noncoding	noncoding	noncoding
MSTRG.184064.1		XLOC_186466	284	coding	noncoding	noncoding	noncoding
MSTRG.184163.1	SAXO2	XLOC_186492	460	coding	noncoding	noncoding	noncoding
MSTRG.184139.4	RPS17	XLOC_186498	504	coding	coding	noncoding	noncoding
NR_046096.1	CPEB1-AS1	XLOC_186501	2138	coding	coding	coding	noncoding
XR_001751643.2	LOC105370928	XLOC_186519	6056	coding	noncoding	noncoding	noncoding
MSTRG.184281.1	C15orf40	XLOC_186522	403	coding	noncoding	coding	noncoding
MSTRG.184198.1	LOC105370932	XLOC_186545	244	coding	noncoding	noncoding	noncoding
MSTRG.184205.1		XLOC_186548	212	coding	noncoding	noncoding	noncoding
XM_024450000.1	ADAMTSL3	XLOC_186554	4805	coding	coding	coding	coding
MSTRG.184256.1	ADAMTSL3	XLOC_186561	336	coding	noncoding	noncoding	noncoding
MSTRG.184257.1	ADAMTSL3	XLOC_186562	305	coding	noncoding	noncoding	noncoding
MSTRG.184298.1	GOLGA6L5P	XLOC_186583	258	coding	noncoding	noncoding	noncoding
MSTRG.184319.1		XLOC_186600	256	coding	noncoding	noncoding	noncoding
MSTRG.184356.8	ZNF592	XLOC_186614	411	coding	noncoding	noncoding	noncoding
MSTRG.184332.1		XLOC_186622	303	coding	noncoding	noncoding	noncoding
NM_020778.4	ALPK3	XLOC_186623	10917	coding	coding	coding	coding
XM_011522204.3	SLC28A1	XLOC_186624	2972	coding	coding	coding	coding
MSTRG.184344.1	SLC28A1	XLOC_186625	344	coding	noncoding	noncoding	noncoding
MSTRG.184346.1	SLC28A1	XLOC_186626	260	coding	noncoding	noncoding	noncoding
MSTRG.184367.1		XLOC_186642	235	coding	noncoding	noncoding	noncoding
MSTRG.184570.1	AKAP13	XLOC_186667	417	coding	noncoding	noncoding	noncoding
MSTRG.184402.1	MIR548AP	XLOC_186685	296	coding	noncoding	noncoding	noncoding
XM_011521226.3	AGBL1	XLOC_186697	12562	coding	coding	coding	coding

MSTRG.184446.1		XLOC_186735	302 coding	noncoding	noncoding	noncoding
MSTRG.184503.1	LOC105370958	XLOC_186750	286 coding	noncoding	noncoding	noncoding
MSTRG.184627.1		XLOC_186783	279 coding	noncoding	noncoding	noncoding
NM_007011.8	ABHD2	XLOC_186784	8822 coding	coding	coding	coding
MSTRG.184656.1	ABHD2	XLOC_186792	430 coding	noncoding	noncoding	noncoding
MSTRG.184665.1		XLOC_186809	262 coding	noncoding	noncoding	noncoding
MSTRG.184682.1	LINC00928	XLOC_186812	254 coding	noncoding	noncoding	noncoding
MSTRG.184686.1	TICRR	XLOC_186814	221 coding	noncoding	noncoding	noncoding
MSTRG.184694.1		XLOC_186819	272 coding	noncoding	noncoding	noncoding
MSTRG.184706.1		XLOC_186821	273 coding	noncoding	noncoding	noncoding
MSTRG.184720.6	AP3S2	XLOC_186824	1177 coding	coding	noncoding	noncoding
MSTRG.184718.1		XLOC_186829	252 coding	noncoding	noncoding	noncoding
NM_198526.4	ZNF710	XLOC_186837	4676 coding	coding	coding	coding
MSTRG.184746.1	SEMA4B	XLOC_186851	274 coding	noncoding	noncoding	noncoding
MSTRG.184739.1		XLOC_186858	211 coding	noncoding	noncoding	noncoding
MSTRG.184784.1		XLOC_186863	293 coding	noncoding	noncoding	noncoding
MSTRG.184786.1		XLOC_186864	232 coding	noncoding	noncoding	noncoding
MSTRG.184811.1	CRTC3	XLOC_186870	317 coding	noncoding	noncoding	noncoding
MSTRG.184831.1	BLM	XLOC_186883	627 coding	noncoding	noncoding	noncoding
MSTRG.184847.1		XLOC_186912	324 coding	noncoding	noncoding	noncoding
MSTRG.184848.1		XLOC_186913	250 coding	noncoding	noncoding	noncoding
MSTRG.185041.1	SLCO3A1	XLOC_186932	293 coding	noncoding	noncoding	noncoding
MSTRG.184914.1	ST8SIA2	XLOC_187004	230 coding	noncoding	noncoding	noncoding
MSTRG.184908.1		XLOC_187006	230 coding	noncoding	noncoding	noncoding
MSTRG.184919.1		XLOC_187019	347 coding	noncoding	noncoding	noncoding
MSTRG.184963.1	RGMA	XLOC_187053	299 coding	noncoding	noncoding	noncoding
MSTRG.184965.1	RGMA	XLOC_187055	214 coding	noncoding	noncoding	noncoding
MSTRG.184969.1	LOC101927025	XLOC_187058	249 coding	noncoding	noncoding	noncoding
MSTRG.184971.1	LOC101927025	XLOC_187059	247 coding	noncoding	noncoding	noncoding
MSTRG.184998.1	LOC105370982	XLOC_187067	218 coding	noncoding	noncoding	noncoding
MSTRG.185006.1		XLOC_187075	387 coding	noncoding	noncoding	noncoding
MSTRG.185385.1	MCTP2	XLOC_187130	296 coding	noncoding	noncoding	noncoding
MSTRG.185117.1		XLOC_187164	262 coding	noncoding	noncoding	noncoding
MSTRG.185149.1		XLOC_187175	243 coding	noncoding	noncoding	noncoding
MSTRG.185211.1	LOC105369212	XLOC_187190	235 coding	noncoding	noncoding	noncoding
MSTRG.185216.1	NR2F2-AS1	XLOC_187191	264 coding	noncoding	noncoding	noncoding
MSTRG.185220.1	NR2F2-AS1	XLOC_187193	282 coding	noncoding	noncoding	noncoding
MSTRG.185223.1	NR2F2-AS1	XLOC_187195	218 coding	noncoding	noncoding	noncoding
MSTRG.185232.1		XLOC_187210	285 coding	noncoding	noncoding	noncoding
MSTRG.185289.1		XLOC_187241	236 coding	noncoding	noncoding	noncoding
NM_001291858.2	IGF1R	XLOC_187264	12232 coding	coding	coding	coding
MSTRG.185323.1		XLOC_187333	233 coding	noncoding	noncoding	noncoding
MSTRG.185325.1		XLOC_187334	291 coding	noncoding	noncoding	noncoding

MSTRG.185435.1	TTC23	XLOC_187341	437 coding	noncoding	noncoding	noncoding
MSTRG.185438.1	TTC23	XLOC_187343	313 coding	noncoding	noncoding	noncoding
MSTRG.185508.1	LOC400464	XLOC_187400	216 coding	noncoding	noncoding	noncoding
MSTRG.185616.1	ADAMTS17	XLOC_187423	248 coding	noncoding	noncoding	noncoding
MSTRG.185709.1		XLOC_187508	231 coding	noncoding	noncoding	noncoding
MSTRG.185714.1		XLOC_187509	266 coding	noncoding	noncoding	noncoding
MSTRG.185735.1		XLOC_187524	272 coding	noncoding	noncoding	noncoding
XR_948846.2	LOC105379208	XLOC_187559	1628 coding	noncoding	noncoding	noncoding
XR_001751434.1	LOC107984786	XLOC_187570	1300 coding	noncoding	coding	noncoding
MSTRG.178943.1	LOC101928039	XLOC_187573	271 coding	noncoding	noncoding	noncoding
MSTRG.178948.1		XLOC_187578	297 coding	coding	noncoding	noncoding
NR_033350.1	GOLGA8EP	XLOC_187579	5269 coding	coding	coding	coding
MSTRG.178949.1		XLOC_187580	555 coding	coding	noncoding	noncoding
MSTRG.178952.1	HERC2P2	XLOC_187583	332 coding	noncoding	noncoding	noncoding
MSTRG.178990.1		XLOC_187620	226 coding	noncoding	noncoding	noncoding
MSTRG.179392.1	SNRPN	XLOC_187626	262 coding	noncoding	noncoding	noncoding
MSTRG.179492.1	SNHG14	XLOC_187645	694 coding	noncoding	coding	noncoding
MSTRG.179066.1	LINC02250	XLOC_187672	245 coding	noncoding	noncoding	noncoding
MSTRG.179069.1	LINC02250	XLOC_187675	271 coding	noncoding	noncoding	noncoding
MSTRG.179058.1		XLOC_187677	298 coding	coding	noncoding	noncoding
MSTRG.179073.1		XLOC_187695	293 coding	noncoding	noncoding	noncoding
MSTRG.179102.1	GABRB3	XLOC_187708	285 coding	noncoding	noncoding	noncoding
XR_002957720.1	LOC112268151	XLOC_187713	6997 coding	noncoding	noncoding	noncoding
MSTRG.179117.1		XLOC_187736	250 coding	noncoding	noncoding	noncoding
NM_001368078.1	GOLGA8G	XLOC_187747	5276 coding	coding	coding	coding
MSTRG.179272.5	KLF13	XLOC_187795	7216 coding	coding	coding	coding
MSTRG.179530.1	RYR3	XLOC_187835	294 coding	noncoding	noncoding	noncoding
MSTRG.179534.1	RYR3	XLOC_187839	216 coding	noncoding	noncoding	noncoding
MSTRG.179545.1	RYR3	XLOC_187846	232 coding	noncoding	noncoding	noncoding
MSTRG.179528.1	KATNBL1	XLOC_187870	282 coding	noncoding	noncoding	noncoding
NR_027410.1	GOLGA8B	XLOC_187883	6519 coding	coding	coding	coding
MSTRG.179581.1		XLOC_187887	298 coding	noncoding	noncoding	noncoding
NM_020660.3	GJD2	XLOC_187892	2922 coding	coding	coding	coding
MSTRG.179667.1		XLOC_187951	236 coding	noncoding	noncoding	noncoding
MSTRG.179674.1		XLOC_187952	316 coding	coding	noncoding	noncoding
MSTRG.179688.1		XLOC_187958	280 coding	coding	noncoding	noncoding
MSTRG.179691.1		XLOC_187961	315 coding	noncoding	noncoding	noncoding
NM_170677.5	MEIS2	XLOC_187997	5582 coding	coding	coding	coding
MSTRG.179806.1	MEIS2	XLOC_188000	284 coding	noncoding	noncoding	noncoding
MSTRG.179724.1		XLOC_188003	270 coding	noncoding	noncoding	noncoding
MSTRG.179727.1		XLOC_188005	273 coding	noncoding	noncoding	noncoding
MSTRG.179820.1		XLOC_188011	269 coding	noncoding	noncoding	noncoding
MSTRG.179824.1		XLOC_188018	281 coding	noncoding	noncoding	noncoding

MSTRG.179874.1		XLOC_188041	265 coding	coding	noncoding	noncoding
XM_011521151.3	RASGRP1	XLOC_188043	4614 coding	coding	coding	coding
NM_005739.4	RASGRP1	XLOC_188043	5031 coding	coding	coding	coding
MSTRG.179901.1		XLOC_188044	545 coding	noncoding	coding	noncoding
MSTRG.179908.1		XLOC_188049	239 coding	noncoding	noncoding	noncoding
MSTRG.180045.1		XLOC_188147	314 coding	noncoding	noncoding	noncoding
MSTRG.180081.1	GPR176	XLOC_188158	281 coding	noncoding	noncoding	noncoding
NM_001080792.3	CCDC32	XLOC_188213	1695 coding	coding	coding	coding
MSTRG.180206.1	RAD51	XLOC_188225	245 coding	noncoding	noncoding	noncoding
MSTRG.180208.1		XLOC_188229	219 coding	noncoding	noncoding	noncoding
MSTRG.180274.1	INO80	XLOC_188245	238 coding	noncoding	noncoding	noncoding
MSTRG.180277.1	INO80	XLOC_188248	339 coding	noncoding	noncoding	noncoding
XM_011521298.2	EXD1	XLOC_188251	3469 coding	coding	coding	coding
MSTRG.180291.1	EXD1	XLOC_188252	227 coding	noncoding	noncoding	noncoding
MSTRG.180293.6	OIP5-AS1	XLOC_188253	8014 coding	noncoding	noncoding	noncoding
MSTRG.180303.4	NUSAP1	XLOC_188256	1345 coding	coding	noncoding	noncoding
MSTRG.180311.1		XLOC_188259	283 coding	noncoding	noncoding	noncoding
MSTRG.180313.1		XLOC_188260	304 coding	noncoding	noncoding	noncoding
MSTRG.180280.1	ITPKA	XLOC_188264	261 coding	noncoding	noncoding	noncoding
XM_017022181.1	LTK	XLOC_188265	3193 coding	coding	coding	coding
MSTRG.180289.1		XLOC_188269	288 coding	noncoding	noncoding	noncoding
MSTRG.180322.12	MGA	XLOC_188270	6414 coding	coding	coding	noncoding
MSTRG.180345.5	PLA2G4F	XLOC_188292	431 coding	noncoding	coding	noncoding
XM_011521404.2	VPS39	XLOC_188293	4929 coding	coding	coding	coding
MSTRG.180443.1	VPS39	XLOC_188295	310 coding	noncoding	noncoding	noncoding
NM_001366846.2	ZNF106	XLOC_188312	10521 coding	coding	coding	coding
MSTRG.180477.1	ZNF106	XLOC_188315	268 coding	noncoding	noncoding	noncoding
MSTRG.180479.1	ZNF106	XLOC_188317	204 coding	noncoding	noncoding	noncoding
MSTRG.180366.1	STARD9	XLOC_188324	255 coding	coding	noncoding	noncoding
MSTRG.180389.9	CDAN1	XLOC_188342	457 coding	noncoding	noncoding	noncoding
XM_024449851.1	TTBK2	XLOC_188344	6798 coding	coding	coding	coding
MSTRG.180413.1	TTBK2	XLOC_188345	375 coding	noncoding	noncoding	noncoding
MSTRG.180427.1	UBR1	XLOC_188358	366 coding	noncoding	noncoding	noncoding
NM_153700.2	STRC	XLOC_188387	5515 coding	noncoding	coding	coding
NM_172095.4	CATSPER2	XLOC_188388	4009 coding	coding	coding	coding
NR_146078.1	STRCP1	XLOC_188392	5415 coding	noncoding	coding	coding
MSTRG.180508.1	SERF2	XLOC_188396	292 coding	noncoding	noncoding	noncoding
MSTRG.180568.1	FRMD5	XLOC_188410	256 coding	noncoding	noncoding	noncoding
MSTRG.180571.1	FRMD5	XLOC_188411	292 coding	noncoding	noncoding	noncoding
MSTRG.180577.1	FRMD5	XLOC_188414	236 coding	noncoding	noncoding	noncoding
MSTRG.180523.1		XLOC_188419	287 coding	noncoding	noncoding	noncoding
MSTRG.180591.2	CTDSPL2	XLOC_188422	1503 coding	noncoding	coding	noncoding
MSTRG.180713.2	EIF3J	XLOC_188425	2183 coding	coding	noncoding	noncoding

MSTRG.180621.1		XLOC_188439	283 coding	noncoding	noncoding	noncoding
MSTRG.180660.1		XLOC_188459	234 coding	noncoding	noncoding	noncoding
MSTRG.180688.1	LOC105376714	XLOC_188466	236 coding	noncoding	noncoding	noncoding
MSTRG.180708.1	HMGN2P46	XLOC_188468	277 coding	noncoding	noncoding	noncoding
MSTRG.180751.1		XLOC_188484	281 coding	noncoding	noncoding	noncoding
MSTRG.180754.1		XLOC_188485	282 coding	noncoding	noncoding	noncoding
MSTRG.180787.1		XLOC_188493	222 coding	noncoding	noncoding	noncoding
MSTRG.180820.1	SEMA6D	XLOC_188498	405 coding	noncoding	noncoding	noncoding
MSTRG.180791.1	LINC01491	XLOC_188509	253 coding	noncoding	noncoding	noncoding
NM_001194998.2	CEP152	XLOC_188560	5560 coding	noncoding	coding	coding
MSTRG.180883.1	CEP152	XLOC_188562	250 coding	noncoding	noncoding	noncoding
MSTRG.180862.1		XLOC_188576	248 coding	noncoding	noncoding	noncoding
MSTRG.180924.1	SECISBP2L	XLOC_188580	256 coding	noncoding	noncoding	noncoding
MSTRG.180871.1		XLOC_188588	260 coding	noncoding	noncoding	noncoding
MSTRG.180918.1		XLOC_188590	208 coding	noncoding	noncoding	noncoding
MSTRG.180958.1		XLOC_188642	278 coding	noncoding	noncoding	noncoding
MSTRG.180965.1		XLOC_188643	283 coding	coding	noncoding	noncoding
MSTRG.181013.1		XLOC_188646	251 coding	noncoding	noncoding	noncoding
XR_001751328.2	TRPM7	XLOC_188648	7196 coding	coding	coding	coding
XM_017022353.2	TRPM7	XLOC_188648	6940 coding	coding	coding	coding
MSTRG.181112.1		XLOC_188662	227 coding	noncoding	noncoding	noncoding
MSTRG.181143.1	GLDN	XLOC_188681	264 coding	noncoding	noncoding	noncoding
MSTRG.181199.1	LOC112268149	XLOC_188713	235 coding	noncoding	noncoding	noncoding
MSTRG.181211.1	LOC112268150	XLOC_188719	282 coding	noncoding	noncoding	noncoding
XM_017021867.2	GNB5	XLOC_188721	4589 coding	coding	coding	coding
MSTRG.181447.1	MYO5C	XLOC_188726	258 coding	noncoding	noncoding	noncoding
MSTRG.181470.1	MYO5A	XLOC_188743	217 coding	noncoding	noncoding	noncoding
NM_001306196.2	ARPP19	XLOC_188757	5423 coding	coding	noncoding	noncoding
MSTRG.181256.1		XLOC_188759	381 coding	noncoding	noncoding	noncoding
MSTRG.181276.1	LOC107983981	XLOC_188797	226 coding	noncoding	noncoding	noncoding
MSTRG.181280.1	LOC107983981	XLOC_188799	284 coding	noncoding	noncoding	noncoding
MSTRG.181298.1	LOC105370826	XLOC_188808	249 coding	noncoding	noncoding	noncoding
MSTRG.181302.1	WDR72	XLOC_188811	292 coding	noncoding	noncoding	noncoding
MSTRG.181390.1		XLOC_188830	255 coding	noncoding	noncoding	noncoding
MSTRG.181389.1		XLOC_188835	210 coding	coding	noncoding	noncoding
MSTRG.181414.1	PRTG	XLOC_188869	313 coding	noncoding	noncoding	noncoding
MSTRG.181417.1	PRTG	XLOC_188871	429 coding	noncoding	noncoding	noncoding
MSTRG.181418.1	PRTG	XLOC_188872	421 coding	noncoding	noncoding	noncoding
MSTRG.181412.1		XLOC_188873	273 coding	noncoding	noncoding	noncoding
MSTRG.181432.1	NEDD4	XLOC_188878	268 coding	noncoding	noncoding	noncoding
MSTRG.181424.1		XLOC_188882	250 coding	noncoding	noncoding	noncoding
MSTRG.181602.3	TEX9	XLOC_188919	601 coding	noncoding	noncoding	noncoding
MSTRG.181626.1		XLOC_188945	241 coding	noncoding	noncoding	noncoding

MSTRG.181685.3	TCF12	XLOC_188953	2395 coding	coding	noncoding	noncoding
MSTRG.181530.1		XLOC_188960	269 coding	noncoding	noncoding	noncoding
MSTRG.181640.1		XLOC_188988	286 coding	coding	noncoding	noncoding
MSTRG.181917.1	ALDH1A2	XLOC_188991	217 coding	noncoding	noncoding	noncoding
MSTRG.181996.1		XLOC_189007	312 coding	noncoding	noncoding	noncoding
MSTRG.181763.1		XLOC_189074	409 coding	noncoding	noncoding	noncoding
MSTRG.181764.1		XLOC_189075	305 coding	noncoding	noncoding	noncoding
MSTRG.181812.1		XLOC_189113	252 coding	noncoding	noncoding	noncoding
NM_001002858.3	ANXA2	XLOC_189124	1626 coding	coding	coding	coding
XR_002957757.1	LOC107984805	XLOC_189185	14997 coding	noncoding	noncoding	noncoding
MSTRG.182273.1	RORA	XLOC_189187	208 coding	noncoding	noncoding	noncoding
NM_017684.5	VPS13C	XLOC_189206	13274 coding	coding	noncoding	coding
MSTRG.182124.10	VPS13C	XLOC_189206	516 coding	noncoding	noncoding	noncoding
MSTRG.181897.1		XLOC_189230	295 coding	noncoding	noncoding	noncoding
MSTRG.181901.1		XLOC_189233	283 coding	noncoding	noncoding	noncoding
MSTRG.181903.1		XLOC_189236	229 coding	noncoding	noncoding	noncoding
MSTRG.181906.1		XLOC_189245	393 coding	noncoding	noncoding	noncoding
MSTRG.181907.1		XLOC_189246	242 coding	noncoding	noncoding	noncoding
MSTRG.182079.1	TLN2	XLOC_189257	245 coding	noncoding	noncoding	noncoding
MSTRG.182052.1		XLOC_189270	255 coding	noncoding	noncoding	noncoding
MSTRG.182061.4	TPM1-AS	XLOC_189275	1339 coding	coding	coding	noncoding
XM_017022052.1	DAPK2	XLOC_189328	4617 coding	noncoding	coding	coding
MSTRG.182437.1	PPIB	XLOC_189340	1440 coding	coding	coding	noncoding
MSTRG.182452.1	CSNK1G1	XLOC_189354	478 coding	noncoding	noncoding	noncoding
MSTRG.182317.1	RBPMS2	XLOC_189375	280 coding	noncoding	noncoding	noncoding
MSTRG.182318.1	RBPMS2	XLOC_189376	276 coding	noncoding	noncoding	noncoding
MSTRG.182310.1		XLOC_189378	331 coding	noncoding	noncoding	noncoding
MSTRG.182322.1		XLOC_189381	281 coding	noncoding	noncoding	noncoding
MSTRG.182328.1		XLOC_189384	227 coding	noncoding	noncoding	noncoding
MSTRG.182330.1		XLOC_189386	253 coding	noncoding	noncoding	noncoding
MSTRG.182380.1		XLOC_189393	258 coding	noncoding	noncoding	noncoding
MSTRG.182397.1		XLOC_189406	234 coding	noncoding	noncoding	noncoding
MSTRG.182369.1		XLOC_189410	264 coding	noncoding	noncoding	noncoding
MSTRG.182496.1	LOC112268147	XLOC_189422	264 coding	noncoding	noncoding	noncoding
NM_001320835.1	DENND4A	XLOC_189444	8837 coding	coding	coding	coding
MSTRG.182604.18	DENND4A	XLOC_189444	4243 coding	coding	coding	coding
MSTRG.182626.1	LOC105370866	XLOC_189461	251 coding	noncoding	noncoding	noncoding
MSTRG.182561.1	RPL4	XLOC_189470	1490 coding	coding	coding	coding
MSTRG.182532.1	LINC01169	XLOC_189478	247 coding	noncoding	noncoding	noncoding
MSTRG.182548.1		XLOC_189485	324 coding	noncoding	noncoding	noncoding
MSTRG.182576.1		XLOC_189492	325 coding	noncoding	noncoding	noncoding
MSTRG.182577.1		XLOC_189495	220 coding	noncoding	noncoding	noncoding
MSTRG.182710.1	IQCH	XLOC_189511	299 coding	noncoding	noncoding	noncoding

NR_040051.1	IQCH-AS1	XLOC_189512	5960 coding	noncoding	coding	noncoding
MSTRG.182636.1		XLOC_189528	314 coding	noncoding	noncoding	noncoding
NR_026808.1	ANP32A-IT1	XLOC_189564	3281 coding	noncoding	coding	noncoding
MSTRG.182758.1		XLOC_189576	233 coding	noncoding	noncoding	noncoding
MSTRG.182793.1	NOX5	XLOC_189578	791 coding	noncoding	noncoding	noncoding
MSTRG.182832.1		XLOC_189597	254 coding	noncoding	noncoding	noncoding
MSTRG.182852.1		XLOC_189641	247 coding	noncoding	noncoding	noncoding
MSTRG.182918.1		XLOC_189664	227 coding	noncoding	noncoding	noncoding
MSTRG.182953.1		XLOC_189679	444 coding	noncoding	noncoding	noncoding
MSTRG.183041.1	THSD4	XLOC_189722	423 coding	noncoding	noncoding	noncoding
NM_005477.3	HCN4	XLOC_189835	6922 coding	coding	coding	coding
MSTRG.183260.1	REC114	XLOC_189840	261 coding	noncoding	noncoding	noncoding
XR_001751216.1	NPTN	XLOC_189844	2606 coding	coding	coding	coding
XM_005254369.4	INSYN1	XLOC_189855	6996 coding	coding	coding	coding
MSTRG.183286.1	GOLGA6A	XLOC_189876	283 coding	noncoding	noncoding	noncoding
MSTRG.183290.1	ISLR2	XLOC_189880	344 coding	noncoding	noncoding	noncoding
NM_001146030.2	SEMA7A	XLOC_189887	3278 coding	coding	coding	coding
MSTRG.183323.1		XLOC_189901	217 coding	noncoding	noncoding	noncoding
MSTRG.183348.1	SCAMP2	XLOC_189913	263 coding	noncoding	noncoding	noncoding
MSTRG.183352.1		XLOC_189918	215 coding	noncoding	noncoding	noncoding
MSTRG.183353.1		XLOC_189921	237 coding	noncoding	noncoding	noncoding
MSTRG.183355.1		XLOC_189922	296 coding	noncoding	noncoding	noncoding
MSTRG.183436.1		XLOC_189945	282 coding	noncoding	noncoding	noncoding
MSTRG.183451.6		XLOC_189963	2045 coding	noncoding	coding	noncoding
MSTRG.183523.1	NEIL1	XLOC_189972	359 coding	noncoding	coding	noncoding
MSTRG.183528.1	SIN3A	XLOC_189976	218 coding	noncoding	noncoding	noncoding
NM_002833.4	PTPN9	XLOC_189981	7839 coding	coding	coding	coding
MSTRG.183540.1	PTPN9	XLOC_189986	671 coding	noncoding	coding	noncoding
MSTRG.183459.1		XLOC_189988	313 coding	noncoding	noncoding	noncoding
NM_001897.5	CSPG4	XLOC_189995	8290 coding	coding	coding	coding
MSTRG.183486.1	LOC441728	XLOC_190002	314 coding	noncoding	noncoding	noncoding
MSTRG.183495.1		XLOC_190024	261 coding	noncoding	noncoding	noncoding
MSTRG.183643.1	SCAPER	XLOC_190035	243 coding	coding	noncoding	noncoding
MSTRG.183669.1	SCAPER	XLOC_190060	258 coding	noncoding	noncoding	noncoding
MSTRG.183564.1		XLOC_190073	271 coding	noncoding	noncoding	noncoding
MSTRG.183581.1		XLOC_190100	239 coding	noncoding	noncoding	noncoding
MSTRG.183582.1		XLOC_190101	283 coding	noncoding	noncoding	noncoding
MSTRG.183590.1		XLOC_190108	285 coding	noncoding	noncoding	noncoding
MSTRG.183617.1		XLOC_190115	303 coding	noncoding	noncoding	noncoding
MSTRG.183677.1		XLOC_190125	288 coding	noncoding	noncoding	noncoding
MSTRG.183710.1	CHRNA5	XLOC_190134	338 coding	noncoding	noncoding	noncoding
MSTRG.183725.1		XLOC_190143	286 coding	noncoding	noncoding	noncoding
MSTRG.183776.1	CTSH	XLOC_190157	235 coding	noncoding	noncoding	noncoding

MSTRG.183966.1		XLOC_190225	556 coding	noncoding	noncoding	noncoding
MSTRG.183889.1	CTXND1	XLOC_190267	260 coding	noncoding	noncoding	noncoding
MSTRG.183897.1	LINC00927	XLOC_190271	253 coding	noncoding	noncoding	noncoding
XR_001751630.2	LOC107984749	XLOC_190300	3691 coding	noncoding	noncoding	noncoding
MSTRG.184016.2	TLNRD1	XLOC_190310	2175 coding	noncoding	coding	noncoding
MSTRG.184098.1	IL16	XLOC_190327	277 coding	noncoding	noncoding	noncoding
MSTRG.184100.5	STARD5	XLOC_190328	8327 coding	coding	coding	noncoding
MSTRG.184100.12	STARD5	XLOC_190328	9384 coding	coding	coding	coding
MSTRG.184075.1		XLOC_190350	215 coding	noncoding	noncoding	noncoding
MSTRG.184126.1	LINC01583	XLOC_190358	220 coding	noncoding	noncoding	noncoding
MSTRG.184148.1	EFL1	XLOC_190365	284 coding	noncoding	noncoding	noncoding
MSTRG.184140.1	RPS17	XLOC_190391	511 coding	coding	coding	coding
MSTRG.184276.1	HOMER2	XLOC_190410	331 coding	noncoding	noncoding	noncoding
MSTRG.184193.1		XLOC_190421	257 coding	noncoding	noncoding	noncoding
MSTRG.184202.1	LOC105370932	XLOC_190427	363 coding	noncoding	noncoding	noncoding
MSTRG.184195.1		XLOC_190428	268 coding	noncoding	noncoding	noncoding
MSTRG.184207.1		XLOC_190431	235 coding	noncoding	noncoding	noncoding
MSTRG.184318.1		XLOC_190457	256 coding	noncoding	noncoding	noncoding
MSTRG.184355.5	ZNF592	XLOC_190465	8608 coding	coding	coding	coding
MSTRG.184345.1	SLC28A1	XLOC_190478	301 coding	noncoding	noncoding	noncoding
MSTRG.184369.1		XLOC_190485	262 coding	noncoding	noncoding	noncoding
MSTRG.184403.1		XLOC_190496	296 coding	noncoding	noncoding	noncoding
MSTRG.184484.1	AGBL1	XLOC_190519	263 coding	noncoding	coding	noncoding
MSTRG.184445.1		XLOC_190539	302 coding	noncoding	noncoding	noncoding
XM_017022254.2	NTRK3	XLOC_190544	20382 coding	coding	coding	coding
XM_024449935.1	NTRK3	XLOC_190544	2316 coding	coding	coding	coding
XM_006720549.4	NTRK3	XLOC_190544	2211 coding	coding	coding	coding
MSTRG.184525.1	NTRK3	XLOC_190550	248 coding	noncoding	noncoding	noncoding
MSTRG.184504.1	LOC105370958	XLOC_190554	286 coding	noncoding	noncoding	noncoding
MSTRG.184617.1	ACAN	XLOC_190595	467 coding	noncoding	noncoding	noncoding
MSTRG.184624.1	HAPLN3	XLOC_190598	223 coding	noncoding	noncoding	noncoding
MSTRG.184625.1	HAPLN3	XLOC_190599	284 coding	noncoding	noncoding	noncoding
MSTRG.184666.1		XLOC_190621	262 coding	noncoding	noncoding	noncoding
MSTRG.184683.1	LINC00928	XLOC_190627	254 coding	noncoding	noncoding	noncoding
MSTRG.184678.1		XLOC_190628	263 coding	noncoding	noncoding	noncoding
MSTRG.184685.1	TICRR	XLOC_190629	221 coding	noncoding	noncoding	noncoding
MSTRG.184709.1	WDR93	XLOC_190637	211 coding	noncoding	noncoding	noncoding
XR_001751354.1	MESP1	XLOC_190638	1823 coding	coding	coding	coding
MSTRG.184703.1		XLOC_190641	486 coding	noncoding	noncoding	noncoding
MSTRG.184717.1		XLOC_190655	252 coding	noncoding	noncoding	noncoding
MSTRG.184755.2	ZNF710	XLOC_190657	1990 coding	noncoding	noncoding	noncoding
MSTRG.184772.1	ZNF710	XLOC_190660	555 coding	noncoding	noncoding	noncoding
MSTRG.184740.1		XLOC_190671	231 coding	noncoding	noncoding	noncoding

MSTRG.184785.1		XLOC_190677	261	coding	noncoding	noncoding	noncoding
MSTRG.184819.1	LINC01585	XLOC_190680	243	coding	noncoding	noncoding	noncoding
MSTRG.184873.1	VPS33B	XLOC_190704	251	coding	noncoding	noncoding	noncoding
XR_001751656.2	LOC105370972	XLOC_190709	11025	coding	noncoding	noncoding	noncoding
MSTRG.184884.1	LOC107984778	XLOC_190716	295	coding	noncoding	noncoding	noncoding
MSTRG.184878.1		XLOC_190732	235	coding	noncoding	noncoding	noncoding
MSTRG.185043.5	SLCO3A1	XLOC_190735	389	coding	noncoding	noncoding	noncoding
MSTRG.184913.1	ST8SIA2	XLOC_190742	230	coding	noncoding	noncoding	noncoding
MSTRG.184915.1	ST8SIA2	XLOC_190743	255	coding	noncoding	noncoding	noncoding
MSTRG.184938.1		XLOC_190755	276	coding	noncoding	noncoding	noncoding
MSTRG.184941.1		XLOC_190756	271	coding	noncoding	noncoding	noncoding
MSTRG.185132.17	CHD2	XLOC_190763	6274	coding	coding	noncoding	coding
MSTRG.185132.27	CHD2	XLOC_190763	3867	coding	coding	noncoding	noncoding
MSTRG.184967.1	LOC101927025	XLOC_190766	229	coding	noncoding	noncoding	noncoding
MSTRG.184970.1	LOC101927025	XLOC_190767	247	coding	noncoding	noncoding	noncoding
MSTRG.184973.1	LOC105370981	XLOC_190768	253	coding	noncoding	noncoding	noncoding
MSTRG.184997.1	LOC105370982	XLOC_190776	307	coding	noncoding	noncoding	noncoding
MSTRG.184985.1		XLOC_190785	280	coding	noncoding	noncoding	noncoding
MSTRG.185005.1		XLOC_190787	324	coding	noncoding	noncoding	noncoding
MSTRG.185014.1		XLOC_190798	256	coding	noncoding	coding	noncoding
MSTRG.185030.1		XLOC_190800	279	coding	noncoding	noncoding	noncoding
MSTRG.185121.1		XLOC_190806	239	coding	noncoding	noncoding	noncoding
MSTRG.185148.1		XLOC_190814	251	coding	noncoding	noncoding	noncoding
MSTRG.185162.1	LINC00924	XLOC_190827	244	coding	noncoding	noncoding	noncoding
MSTRG.185212.1	LOC112268156	XLOC_190846	235	coding	noncoding	noncoding	noncoding
MSTRG.185214.1	LOC112268156	XLOC_190848	317	coding	noncoding	noncoding	noncoding
MSTRG.185215.1	LOC112268156	XLOC_190849	264	coding	noncoding	noncoding	noncoding
MSTRG.185190.1	LOC101927263	XLOC_190855	307	coding	noncoding	noncoding	noncoding
MSTRG.185271.1	LINC00923	XLOC_190888	281	coding	noncoding	noncoding	noncoding
MSTRG.185276.1	LINC02251	XLOC_190895	282	coding	noncoding	noncoding	noncoding
MSTRG.185278.1	LOC105371010	XLOC_190898	262	coding	noncoding	noncoding	noncoding
MSTRG.185299.1	FAM169B	XLOC_190906	278	coding	noncoding	noncoding	noncoding
MSTRG.185301.1	FAM169B	XLOC_190907	295	coding	noncoding	noncoding	noncoding
MSTRG.185520.9	IRAIN	XLOC_190913	5319	coding	coding	coding	coding
MSTRG.185520.10	IRAIN	XLOC_190913	11398	coding	coding	coding	coding
MSTRG.185432.1	TTC23	XLOC_190937	212	coding	noncoding	noncoding	noncoding
MSTRG.185436.1	TTC23	XLOC_190938	302	coding	noncoding	noncoding	noncoding
NR_003260.1	DNM1P46	XLOC_190953	3831	coding	coding	coding	coding
XM_017021978.1	ADAMTS17	XLOC_190965	8814	coding	coding	coding	coding
XM_017021984.1	ADAMTS17	XLOC_190965	11543	coding	coding	coding	coding
MSTRG.185601.1	ADAMTS17	XLOC_190967	277	coding	noncoding	noncoding	noncoding
MSTRG.185673.1	CERS3-AS1	XLOC_190976	249	coding	noncoding	noncoding	noncoding
MSTRG.185681.1	CERS3	XLOC_190980	250	coding	noncoding	noncoding	noncoding

NR_148019.1	LINS1	XLOC_190986	3751 coding	coding	noncoding	coding
NR_148018.1	LINS1	XLOC_190986	2456 coding	coding	noncoding	coding
MSTRG.185710.1		XLOC_191050	246 coding	noncoding	noncoding	noncoding
MSTRG.185713.1		XLOC_191051	266 coding	noncoding	noncoding	noncoding
MSTRG.185785.1	TARSL2	XLOC_191066	241 coding	noncoding	noncoding	noncoding
MSTRG.185736.1		XLOC_191075	272 coding	noncoding	noncoding	noncoding
MSTRG.185816.6	HBA1	XLOC_191103	1056 coding	coding	coding	coding
MSTRG.185816.8	HBA1	XLOC_191103	1082 coding	coding	coding	coding
MSTRG.185816.10	HBA1	XLOC_191103	878 coding	coding	coding	coding
MSTRG.185811.1	LUC7L	XLOC_191106	3968 coding	coding	coding	noncoding
MSTRG.185895.1	LOC105371036	XLOC_191117	207 coding	noncoding	noncoding	noncoding
XM_017022960.2	PRR35	XLOC_191125	2952 coding	coding	coding	coding
XR_243327.2	LOC101929280	XLOC_191131	2219 coding	noncoding	coding	noncoding
NR_138610.1	MCRIP2	XLOC_191136	813 coding	coding	noncoding	noncoding
NR_147953.1	RHOT2	XLOC_191140	2587 coding	coding	coding	coding
MSTRG.185961.1		XLOC_191153	281 coding	noncoding	noncoding	noncoding
MSTRG.185992.1		XLOC_191179	376 coding	noncoding	coding	noncoding
MSTRG.185998.1	C1QTNF8	XLOC_191181	318 coding	noncoding	noncoding	noncoding
NM_021098.3	CACNA1H	XLOC_191183	8219 coding	coding	noncoding	coding
MSTRG.186001.1		XLOC_191188	311 coding	noncoding	noncoding	noncoding
XM_005255541.4	UBE2I	XLOC_191192	1125 coding	coding	coding	coding
MSTRG.186040.1	CLCN7	XLOC_191207	418 coding	coding	noncoding	noncoding
NM_020825.3	CRAMP1	XLOC_191223	7673 coding	noncoding	coding	coding
MSTRG.186088.1	JPT2	XLOC_191227	415 coding	noncoding	noncoding	noncoding
MSTRG.186120.2	EME2	XLOC_191237	2941 coding	coding	coding	coding
NM_001370404.1	TSC2	XLOC_191264	6283 coding	coding	coding	coding
MSTRG.186175.1	TSC2	XLOC_191266	275 coding	noncoding	noncoding	noncoding
MSTRG.186215.1	ABCA3	XLOC_191294	230 coding	noncoding	noncoding	noncoding
MSTRG.186217.1	ABCA3	XLOC_191296	298 coding	noncoding	noncoding	noncoding
MSTRG.186233.1		XLOC_191324	422 coding	noncoding	noncoding	noncoding
XR_932815.2	SRRM2	XLOC_191325	11511 coding	coding	coding	coding
XR_002957796.1	SRRM2	XLOC_191325	11941 coding	coding	coding	coding
MSTRG.186237.1	PRSS41	XLOC_191328	246 coding	noncoding	noncoding	noncoding
MSTRG.186276.1	FLYWCH1	XLOC_191333	295 coding	noncoding	noncoding	noncoding
MSTRG.186270.1		XLOC_191337	252 coding	noncoding	noncoding	noncoding
NR_110947.1	LOC101929613	XLOC_191339	440 coding	noncoding	noncoding	noncoding
XM_017023643.2	ZNF75A	XLOC_191381	1550 coding	coding	coding	coding
MSTRG.186354.1		XLOC_191390	281 coding	coding	noncoding	noncoding
MSTRG.186388.1		XLOC_191398	243 coding	noncoding	noncoding	noncoding
MSTRG.186400.12	NLRC3	XLOC_191399	314 coding	noncoding	coding	noncoding
MSTRG.186443.1	ADCY9	XLOC_191409	228 coding	noncoding	noncoding	noncoding
MSTRG.186447.1	ADCY9	XLOC_191413	222 coding	noncoding	noncoding	noncoding
MSTRG.186453.1	ADCY9	XLOC_191419	413 coding	noncoding	noncoding	noncoding

MSTRG.186435.1	SRL	XLOC_191424	321	coding	noncoding	coding	noncoding
MSTRG.186441.1		XLOC_191429	277	coding	noncoding	noncoding	noncoding
MSTRG.186496.1	C16orf96	XLOC_191441	324	coding	noncoding	noncoding	noncoding
MSTRG.186497.1	C16orf96	XLOC_191442	280	coding	noncoding	noncoding	noncoding
MSTRG.186535.1	SEC14L5	XLOC_191467	297	coding	noncoding	noncoding	noncoding
MSTRG.186552.1	LOC105371067	XLOC_191474	248	coding	noncoding	noncoding	noncoding
MSTRG.186619.1	RBFOX1	XLOC_191486	389	coding	noncoding	noncoding	noncoding
MSTRG.186624.1	RBFOX1	XLOC_191488	304	coding	noncoding	noncoding	noncoding
MSTRG.186630.1	RBFOX1	XLOC_191491	317	coding	noncoding	noncoding	noncoding
MSTRG.186649.1	RBFOX1	XLOC_191502	232	coding	noncoding	noncoding	noncoding
MSTRG.186676.1	RBFOX1	XLOC_191515	314	coding	noncoding	noncoding	noncoding
MSTRG.186569.1		XLOC_191531	295	coding	noncoding	noncoding	noncoding
MSTRG.186575.1	LOC105371070	XLOC_191532	216	coding	noncoding	noncoding	noncoding
XR_001751981.2	METTL22	XLOC_191544	7532	coding	coding	coding	coding
NM_001127448.2	ABAT	XLOC_191548	4906	coding	coding	coding	coding
MSTRG.186709.1		XLOC_191562	360	coding	noncoding	noncoding	noncoding
MSTRG.186705.1		XLOC_191563	269	coding	noncoding	noncoding	noncoding
MSTRG.186707.1		XLOC_191564	550	coding	noncoding	noncoding	noncoding
MSTRG.186732.1		XLOC_191574	253	coding	noncoding	noncoding	noncoding
MSTRG.186734.1		XLOC_191575	314	coding	noncoding	noncoding	noncoding
MSTRG.186737.1		XLOC_191581	244	coding	noncoding	noncoding	noncoding
MSTRG.186790.1	LOC101927026	XLOC_191588	260	coding	noncoding	noncoding	noncoding
MSTRG.186801.1	LOC101927026	XLOC_191591	263	coding	noncoding	noncoding	noncoding
XR_933063.3	LOC105371077	XLOC_191594	4797	coding	noncoding	noncoding	noncoding
MSTRG.186820.1	GRIN2A	XLOC_191598	287	coding	noncoding	noncoding	noncoding
MSTRG.186824.1	GRIN2A	XLOC_191600	271	coding	noncoding	noncoding	noncoding
MSTRG.186773.1		XLOC_191603	347	coding	coding	noncoding	noncoding
MSTRG.186775.1		XLOC_191604	332	coding	noncoding	noncoding	noncoding
MSTRG.186839.1		XLOC_191606	264	coding	noncoding	noncoding	noncoding
MSTRG.186846.1	ATF7IP2	XLOC_191612	252	coding	noncoding	noncoding	noncoding
MSTRG.186849.1	ATF7IP2	XLOC_191614	233	coding	noncoding	noncoding	noncoding
NM_001286403.2	CIITA	XLOC_191621	14309	coding	coding	coding	coding
MSTRG.186855.1		XLOC_191639	228	coding	noncoding	noncoding	noncoding
MSTRG.186934.1	LOC105371082	XLOC_191647	273	coding	noncoding	noncoding	noncoding
MSTRG.186944.1	LOC400499	XLOC_191652	446	coding	noncoding	noncoding	noncoding
MSTRG.186857.1		XLOC_191664	1163	coding	noncoding	noncoding	coding
MSTRG.186859.1		XLOC_191665	609	coding	noncoding	noncoding	noncoding
MSTRG.186889.2	LITAF	XLOC_191669	5428	coding	coding	coding	noncoding
MSTRG.186903.8	TXNDC11	XLOC_191674	1019	coding	coding	noncoding	coding
MSTRG.186904.1		XLOC_191677	230	coding	noncoding	noncoding	noncoding
MSTRG.186960.3	RSL1D1	XLOC_191678	1095	coding	coding	coding	noncoding
MSTRG.187102.1	NPIPB2	XLOC_191683	469	coding	noncoding	noncoding	noncoding
MSTRG.187105.1	NPIPB2	XLOC_191685	250	coding	noncoding	noncoding	noncoding

MSTRG.187112.1	SNX29	XLOC_191690	236	coding	noncoding	noncoding	noncoding
XR_933083.3	LOC105371087	XLOC_191700	2636	coding	noncoding	noncoding	noncoding
MSTRG.187125.1	SNX29	XLOC_191701	218	coding	noncoding	noncoding	noncoding
MSTRG.187132.1	SNX29	XLOC_191708	265	coding	noncoding	noncoding	noncoding
MSTRG.186968.1		XLOC_191710	253	coding	noncoding	noncoding	noncoding
MSTRG.186974.1		XLOC_191720	259	coding	noncoding	noncoding	noncoding
MSTRG.186979.1		XLOC_191723	269	coding	noncoding	noncoding	noncoding
MSTRG.186997.1	SHISA9	XLOC_191726	222	coding	noncoding	noncoding	noncoding
MSTRG.186999.1	SHISA9	XLOC_191727	317	coding	noncoding	noncoding	noncoding
MSTRG.187003.1	SHISA9	XLOC_191729	223	coding	noncoding	noncoding	noncoding
MSTRG.187012.1	LOC105371091	XLOC_191733	268	coding	noncoding	noncoding	noncoding
MSTRG.187016.1	SHISA9	XLOC_191735	300	coding	noncoding	noncoding	noncoding
MSTRG.187020.1	SHISA9	XLOC_191738	236	coding	noncoding	noncoding	noncoding
MSTRG.186985.1		XLOC_191746	278	coding	noncoding	noncoding	noncoding
MSTRG.186990.1		XLOC_191749	318	coding	noncoding	noncoding	noncoding
MSTRG.187080.1		XLOC_191764	236	coding	noncoding	noncoding	noncoding
MSTRG.187091.1	MIR193BHG	XLOC_191771	279	coding	noncoding	noncoding	noncoding
MSTRG.187084.1		XLOC_191774	452	coding	noncoding	noncoding	noncoding
MSTRG.187085.1		XLOC_191775	244	coding	noncoding	noncoding	noncoding
NM_016561.3	BFAR	XLOC_191781	2903	coding	coding	coding	coding
NR_146231.1	PKD1P3-NPIPA1	XLOC_191788	7365	coding	coding	coding	coding
MSTRG.187194.1	PDXDC1	XLOC_191795	346	coding	coding	coding	noncoding
MSTRG.187225.1	ABCC1	XLOC_191810	252	coding	noncoding	noncoding	noncoding
MSTRG.187245.1		XLOC_191825	226	coding	noncoding	noncoding	noncoding
MSTRG.187248.1		XLOC_191828	235	coding	noncoding	noncoding	noncoding
MSTRG.187270.1		XLOC_191852	302	coding	noncoding	noncoding	noncoding
MSTRG.187278.1		XLOC_191858	231	coding	noncoding	noncoding	noncoding
MSTRG.187282.1		XLOC_191867	367	coding	noncoding	noncoding	noncoding
MSTRG.187284.1		XLOC_191868	206	coding	noncoding	noncoding	noncoding
MSTRG.187384.2	SMG1	XLOC_191871	12109	coding	coding	coding	coding
MSTRG.187336.1	TMC7	XLOC_191874	318	coding	noncoding	noncoding	noncoding
MSTRG.187340.1	TMC7	XLOC_191878	278	coding	noncoding	noncoding	noncoding
MSTRG.187359.1		XLOC_191883	246	coding	noncoding	noncoding	noncoding
XM_011545866.3	SYT17	XLOC_191884	3655	coding	coding	coding	coding
MSTRG.187370.1	LOC105371114	XLOC_191886	207	coding	noncoding	noncoding	noncoding
MSTRG.187377.1	LOC105371114	XLOC_191890	444	coding	noncoding	noncoding	noncoding
MSTRG.187397.1	TMC5	XLOC_191892	316	coding	noncoding	noncoding	noncoding
MSTRG.187446.1	IQCK	XLOC_191908	220	coding	noncoding	noncoding	noncoding
MSTRG.187448.1	IQCK	XLOC_191910	288	coding	noncoding	noncoding	noncoding
MSTRG.187452.1	IQCK	XLOC_191911	265	coding	noncoding	noncoding	noncoding
MSTRG.187453.1	IQCK	XLOC_191912	254	coding	noncoding	noncoding	noncoding
MSTRG.187454.1	IQCK	XLOC_191913	320	coding	noncoding	noncoding	noncoding
MSTRG.187408.1		XLOC_191915	572	coding	noncoding	noncoding	noncoding

MSTRG.187417.1		XLOC_191920	602 coding	noncoding	noncoding	noncoding
MSTRG.187419.1		XLOC_191921	268 coding	noncoding	noncoding	noncoding
MSTRG.187430.1	PDILT	XLOC_191927	268 coding	noncoding	noncoding	noncoding
MSTRG.187486.1	LOC105371120	XLOC_191938	306 coding	noncoding	noncoding	noncoding
MSTRG.187489.1	ACSM1	XLOC_191940	383 coding	noncoding	noncoding	noncoding
MSTRG.187466.1	DNAH3	XLOC_191974	253 coding	noncoding	noncoding	noncoding
XM_024450293.1	METTL9	XLOC_191994	2944 coding	coding	coding	coding
MSTRG.187600.3	IGSF6	XLOC_191995	3216 coding	coding	noncoding	noncoding
MSTRG.187621.1		XLOC_192025	350 coding	noncoding	noncoding	noncoding
MSTRG.187640.1	SCNN1B	XLOC_192035	265 coding	noncoding	noncoding	noncoding
MSTRG.187626.1	SCNN1B	XLOC_192038	272 coding	noncoding	noncoding	noncoding
XR_001752007.2	DCTN5	XLOC_192046	7885 coding	coding	coding	noncoding
MSTRG.187692.1	ERN2	XLOC_192049	265 coding	noncoding	coding	noncoding
MSTRG.187662.1		XLOC_192051	378 coding	noncoding	noncoding	noncoding
MSTRG.187714.3	PRKCB	XLOC_192055	201 coding	noncoding	noncoding	noncoding
MSTRG.187695.1		XLOC_192074	271 coding	noncoding	noncoding	noncoding
MSTRG.187699.1		XLOC_192075	284 coding	noncoding	noncoding	noncoding
MSTRG.187738.1	TNRC6A	XLOC_192088	209 coding	noncoding	noncoding	noncoding
MSTRG.187815.1	HS3ST4	XLOC_192126	250 coding	noncoding	noncoding	noncoding
MSTRG.187824.1		XLOC_192137	303 coding	noncoding	noncoding	noncoding
MSTRG.187835.1		XLOC_192141	383 coding	noncoding	noncoding	noncoding
MSTRG.187843.1		XLOC_192146	255 coding	noncoding	noncoding	noncoding
MSTRG.187848.1		XLOC_192150	267 coding	noncoding	noncoding	noncoding
MSTRG.187849.1		XLOC_192151	376 coding	noncoding	noncoding	noncoding
MSTRG.187867.2	NSMCE1	XLOC_192158	549 coding	coding	noncoding	noncoding
MSTRG.187880.1	IL4R	XLOC_192162	285 coding	noncoding	noncoding	noncoding
MSTRG.187882.1	IL4R	XLOC_192164	294 coding	noncoding	noncoding	noncoding
MSTRG.187859.1	IL21R	XLOC_192173	397 coding	noncoding	noncoding	noncoding
MSTRG.187860.1	IL21R-AS1	XLOC_192174	217 coding	noncoding	noncoding	noncoding
MSTRG.187982.1	KIAA0556	XLOC_192181	239 coding	noncoding	noncoding	noncoding
MSTRG.187985.1	KIAA0556	XLOC_192184	233 coding	noncoding	noncoding	noncoding
MSTRG.187909.1	GSG1L	XLOC_192195	291 coding	noncoding	noncoding	noncoding
MSTRG.187920.1	SBK1	XLOC_192208	245 coding	noncoding	noncoding	noncoding
MSTRG.187922.1	SBK1	XLOC_192209	330 coding	noncoding	noncoding	noncoding
MSTRG.188042.2	EIF3CL	XLOC_192214	1448 coding	coding	coding	coding
MSTRG.188054.1		XLOC_192220	292 coding	noncoding	noncoding	noncoding
MSTRG.188057.1		XLOC_192221	213 coding	noncoding	noncoding	noncoding
MSTRG.188059.1	SGF29	XLOC_192223	209 coding	noncoding	noncoding	noncoding
MSTRG.188078.1	NPIPB9	XLOC_192231	294 coding	noncoding	noncoding	noncoding
XM_005255069.1	ATXN2L	XLOC_192236	4433 coding	coding	coding	coding
NM_001308293.1	SH2B1	XLOC_192240	3192 coding	coding	coding	coding
MSTRG.188017.1	RABEP2	XLOC_192244	232 coding	noncoding	noncoding	noncoding
MSTRG.188018.1	RABEP2	XLOC_192245	654 coding	coding	noncoding	noncoding

MSTRG.188027.1	NFATC2IP	XLOC_192251	294 coding	noncoding	noncoding	noncoding
MSTRG.188029.1	LAT	XLOC_192255	212 coding	noncoding	noncoding	noncoding
MSTRG.188030.1		XLOC_192256	273 coding	noncoding	noncoding	noncoding
MSTRG.188036.1		XLOC_192258	236 coding	noncoding	noncoding	noncoding
MSTRG.188248.1		XLOC_192282	238 coding	noncoding	noncoding	noncoding
MSTRG.188087.1		XLOC_192293	278 coding	noncoding	noncoding	noncoding
MSTRG.188095.2	C16orf54	XLOC_192297	2191 coding	coding	coding	noncoding
MSTRG.188092.1	ZG16	XLOC_192300	332 coding	noncoding	noncoding	noncoding
NM_001256270.1	KIF22	XLOC_192304	2257 coding	coding	coding	coding
MSTRG.188120.1	MVP	XLOC_192309	273 coding	noncoding	noncoding	noncoding
XR_001752362.1	LOC107984836	XLOC_192314	2005 coding	noncoding	coding	noncoding
MSTRG.188107.1		XLOC_192316	236 coding	noncoding	noncoding	noncoding
MSTRG.188131.1	TMEM219	XLOC_192318	206 coding	noncoding	noncoding	noncoding
MSTRG.188141.1	INO80E	XLOC_192322	442 coding	noncoding	noncoding	noncoding
MSTRG.188155.1		XLOC_192335	237 coding	noncoding	noncoding	noncoding
NM_001114380.2	ITGAL	XLOC_192356	4877 coding	coding	coding	coding
MSTRG.188296.8	ITGAL	XLOC_192356	358 coding	noncoding	noncoding	noncoding
MSTRG.188291.1		XLOC_192363	312 coding	noncoding	noncoding	noncoding
MSTRG.188301.1	ZNF785	XLOC_192368	261 coding	noncoding	noncoding	noncoding
MSTRG.188303.1	ZNF785	XLOC_192370	599 coding	noncoding	noncoding	noncoding
MSTRG.188312.1		XLOC_192377	318 coding	noncoding	noncoding	noncoding
NM_006662.3	SRCAP	XLOC_192379	11724 coding	coding	noncoding	coding
MSTRG.188365.1	RNF40	XLOC_192386	704 coding	noncoding	noncoding	noncoding
MSTRG.188329.1	BCL7C	XLOC_192389	222 coding	noncoding	noncoding	noncoding
XM_005255712.4	ZNF646	XLOC_192415	6705 coding	coding	coding	coding
MSTRG.188408.1	KAT8	XLOC_192421	265 coding	noncoding	noncoding	noncoding
MSTRG.188378.1		XLOC_192424	214 coding	noncoding	noncoding	noncoding
MSTRG.188383.1		XLOC_192432	437 coding	noncoding	noncoding	noncoding
MSTRG.188468.1		XLOC_192435	236 coding	noncoding	noncoding	noncoding
MSTRG.188470.1		XLOC_192436	267 coding	noncoding	noncoding	noncoding
NM_000887.5	ITGAX	XLOC_192445	4663 coding	coding	coding	coding
MSTRG.188480.6	ITGAX	XLOC_192445	208 coding	noncoding	noncoding	noncoding
MSTRG.188421.1	ZNF843	XLOC_192450	215 coding	noncoding	noncoding	noncoding
NM_001318221.2	AHSP	XLOC_192458	484 coding	coding	coding	coding
MSTRG.188459.1		XLOC_192465	254 coding	noncoding	noncoding	noncoding
MSTRG.188465.1		XLOC_192466	246 coding	coding	noncoding	noncoding
MSTRG.188590.1	ZNF720	XLOC_192475	261 coding	noncoding	noncoding	noncoding
MSTRG.188502.1	LOC102725101	XLOC_192494	249 coding	noncoding	noncoding	noncoding
XM_017023634.2	TP53TG3D	XLOC_192495	3428 coding	noncoding	coding	coding
MSTRG.188517.1	LOC102725101	XLOC_192500	297 coding	noncoding	noncoding	noncoding
MSTRG.188521.1	LOC102725101	XLOC_192501	239 coding	noncoding	noncoding	noncoding
MSTRG.188491.1		XLOC_192508	270 coding	noncoding	noncoding	noncoding
MSTRG.188559.1		XLOC_192533	243 coding	noncoding	noncoding	noncoding

MSTRG.188574.1		XLOC_192545	301 coding	noncoding	noncoding	noncoding
MSTRG.188578.1		XLOC_192550	220 coding	noncoding	noncoding	noncoding
MSTRG.188613.1		XLOC_192553	241 coding	noncoding	noncoding	noncoding
MSTRG.188684.1		XLOC_192590	224 coding	noncoding	noncoding	noncoding
MSTRG.188701.1		XLOC_192599	307 coding	noncoding	noncoding	noncoding
MSTRG.188903.1	PHKB	XLOC_192627	273 coding	noncoding	noncoding	noncoding
MSTRG.188734.1		XLOC_192641	298 coding	noncoding	noncoding	noncoding
MSTRG.188747.1		XLOC_192648	313 coding	noncoding	noncoding	noncoding
MSTRG.188755.1	ABCC12	XLOC_192649	278 coding	noncoding	noncoding	noncoding
MSTRG.188790.1	SIAH1	XLOC_192654	228 coding	noncoding	noncoding	noncoding
MSTRG.188757.1		XLOC_192656	233 coding	noncoding	noncoding	noncoding
MSTRG.188759.14	N4BP1	XLOC_192657	1153 coding	noncoding	coding	noncoding
MSTRG.188812.1		XLOC_192661	211 coding	coding	noncoding	noncoding
MSTRG.188799.1		XLOC_192662	295 coding	coding	noncoding	noncoding
MSTRG.188809.1	LOC105371241	XLOC_192671	315 coding	noncoding	noncoding	noncoding
MSTRG.188810.1		XLOC_192673	273 coding	noncoding	noncoding	noncoding
XR_001752139.2	LOC102724859	XLOC_192674	22318 coding	noncoding	coding	coding
MSTRG.188844.1		XLOC_192691	351 coding	noncoding	noncoding	noncoding
XM_017022895.1	ADCY7	XLOC_192709	6258 coding	coding	coding	coding
NM_015247.2	CYLD	XLOC_192734	8713 coding	coding	coding	coding
MSTRG.189024.1		XLOC_192786	204 coding	noncoding	noncoding	noncoding
MSTRG.189041.1		XLOC_192793	273 coding	noncoding	noncoding	noncoding
MSTRG.189043.1		XLOC_192794	295 coding	noncoding	noncoding	noncoding
XM_005256176.3	CHD9	XLOC_192796	10162 coding	coding	coding	coding
MSTRG.189274.1	CHD9	XLOC_192797	324 coding	noncoding	noncoding	noncoding
MSTRG.189279.1	CHD9	XLOC_192802	231 coding	noncoding	noncoding	noncoding
XM_011523253.2	RBL2	XLOC_192831	4057 coding	coding	coding	coding
MSTRG.189075.1		XLOC_192868	228 coding	noncoding	noncoding	noncoding
MSTRG.189083.1		XLOC_192873	246 coding	noncoding	noncoding	noncoding
MSTRG.189107.1		XLOC_192886	265 coding	noncoding	noncoding	noncoding
MSTRG.189118.1		XLOC_192891	397 coding	noncoding	noncoding	noncoding
MSTRG.189116.1		XLOC_192892	274 coding	noncoding	noncoding	noncoding
MSTRG.189138.1		XLOC_192903	249 coding	noncoding	noncoding	noncoding
MSTRG.189149.1		XLOC_192910	450 coding	noncoding	noncoding	noncoding
NM_004530.6	MMP2	XLOC_192911	3514 coding	coding	coding	coding
MSTRG.189230.1	GNAO1	XLOC_192951	770 coding	noncoding	noncoding	noncoding
MSTRG.189241.1		XLOC_192961	287 coding	noncoding	noncoding	noncoding
MSTRG.189245.1		XLOC_192962	244 coding	noncoding	noncoding	noncoding
MSTRG.189325.1	SLC12A3	XLOC_192994	258 coding	noncoding	noncoding	noncoding
NM_000078.3	CETP	XLOC_193000	1691 coding	coding	coding	coding
XM_011523373.2	NLRC5	XLOC_193004	6928 coding	coding	coding	coding
MSTRG.189432.24	NLRC5	XLOC_193004	477 coding	noncoding	noncoding	noncoding
MSTRG.189423.1	PLLP	XLOC_193029	262 coding	noncoding	noncoding	noncoding

MSTRG.189415.1		XLOC_193032	255 coding	noncoding	noncoding	noncoding
NM_002990.5	CCL22	XLOC_193033	2917 coding	coding	coding	coding
XM_011522950.2	ADGRG5	XLOC_193046	4376 coding	coding	coding	coding
XR_001752213.1	LOC107984889	XLOC_193048	3542 coding	noncoding	noncoding	noncoding
MSTRG.189497.1	KIFC3	XLOC_193057	238 coding	noncoding	noncoding	noncoding
MSTRG.189503.1	KIFC3	XLOC_193060	252 coding	noncoding	noncoding	noncoding
MSTRG.189514.1	CNGB1	XLOC_193065	325 coding	noncoding	noncoding	noncoding
MSTRG.189516.1	CNGB1	XLOC_193067	208 coding	noncoding	noncoding	noncoding
MSTRG.189605.22	CNOT1	XLOC_193087	3624 coding	coding	coding	coding
MSTRG.189581.1		XLOC_193100	226 coding	noncoding	noncoding	noncoding
MSTRG.189588.1		XLOC_193105	280 coding	noncoding	noncoding	noncoding
MSTRG.189598.1		XLOC_193132	406 coding	noncoding	noncoding	noncoding
MSTRG.189653.1		XLOC_193143	232 coding	coding	noncoding	noncoding
MSTRG.189671.1		XLOC_193147	245 coding	noncoding	noncoding	noncoding
MSTRG.189667.1		XLOC_193153	307 coding	noncoding	noncoding	noncoding
MSTRG.189684.1		XLOC_193159	288 coding	noncoding	noncoding	noncoding
MSTRG.189693.1		XLOC_193164	258 coding	noncoding	noncoding	noncoding
MSTRG.189701.1		XLOC_193170	238 coding	noncoding	noncoding	noncoding
MSTRG.189741.1	CDH8	XLOC_193176	296 coding	noncoding	noncoding	noncoding
MSTRG.189719.1		XLOC_193186	269 coding	noncoding	noncoding	noncoding
MSTRG.189753.1		XLOC_193191	201 coding	noncoding	noncoding	noncoding
MSTRG.189804.1	LOC105371308	XLOC_193202	315 coding	coding	noncoding	noncoding
MSTRG.189781.1		XLOC_193220	298 coding	noncoding	noncoding	noncoding
MSTRG.189833.1		XLOC_193234	318 coding	noncoding	noncoding	noncoding
MSTRG.189904.1	LINC00922	XLOC_193261	286 coding	noncoding	noncoding	noncoding
MSTRG.189889.1		XLOC_193266	296 coding	noncoding	noncoding	noncoding
MSTRG.189916.1		XLOC_193271	293 coding	noncoding	noncoding	noncoding
XM_011522892.2	BEAN1	XLOC_193373	3474 coding	noncoding	coding	coding
NM_001363918.1	CMTM3	XLOC_193402	2333 coding	coding	coding	coding
MSTRG.189961.1		XLOC_193414	264 coding	noncoding	noncoding	noncoding
MSTRG.190121.1	TERB1	XLOC_193420	282 coding	coding	noncoding	noncoding
MSTRG.190063.1	CA7	XLOC_193426	282 coding	noncoding	noncoding	noncoding
NM_001185176.1	CES3	XLOC_193433	3179 coding	coding	coding	coding
MSTRG.190128.1		XLOC_193435	211 coding	noncoding	noncoding	noncoding
NM_001040667.3	HSF4	XLOC_193452	2224 coding	coding	coding	coding
MSTRG.190164.3	ELMO3	XLOC_193458	701 coding	coding	coding	noncoding
MSTRG.190170.1	LRRC29	XLOC_193460	1247 coding	noncoding	noncoding	noncoding
MSTRG.190198.1	ZDHHC1	XLOC_193480	246 coding	noncoding	noncoding	noncoding
XM_005255775.4	CTCF	XLOC_193489	3816 coding	coding	coding	coding
MSTRG.190227.1	CTCF	XLOC_193491	231 coding	noncoding	noncoding	noncoding
MSTRG.190272.11	RANBP10	XLOC_193499	1511 coding	coding	coding	coding
NM_001322040.2	NUTF2	XLOC_193505	2332 coding	coding	coding	coding
MSTRG.190258.1	LCAT	XLOC_193514	308 coding	coding	noncoding	noncoding

MSTRG.190295.1	DPEP2	XLOC_193528	1143	coding	noncoding	noncoding	noncoding
MSTRG.190295.17	DPEP2	XLOC_193528	1230	coding	coding	coding	noncoding
MSTRG.190309.1	NFATC3	XLOC_193535	2122	coding	coding	noncoding	coding
MSTRG.190361.1	PRMT7	XLOC_193553	239	coding	noncoding	noncoding	noncoding
MSTRG.190343.1		XLOC_193560	211	coding	noncoding	noncoding	noncoding
MSTRG.190335.1	CDH3	XLOC_193562	309	coding	noncoding	noncoding	noncoding
MSTRG.190337.1	CDH3	XLOC_193563	306	coding	noncoding	noncoding	noncoding
NM_001317185.2	CDH1	XLOC_193564	4878	coding	coding	coding	coding
MSTRG.190346.1	CDH1	XLOC_193565	330	coding	noncoding	noncoding	noncoding
MSTRG.190347.1	CDH1	XLOC_193566	256	coding	noncoding	noncoding	noncoding
MSTRG.190348.1	CDH1	XLOC_193567	241	coding	noncoding	noncoding	noncoding
MSTRG.190357.1	TANGO6	XLOC_193570	362	coding	noncoding	noncoding	noncoding
MSTRG.190378.6	HAS3	XLOC_193573	5848	coding	coding	coding	coding
MSTRG.190375.1	SNTB2	XLOC_193579	262	coding	noncoding	noncoding	noncoding
MSTRG.190377.1	SNTB2	XLOC_193581	267	coding	noncoding	noncoding	noncoding
MSTRG.190392.1	CYB5B	XLOC_193587	204	coding	noncoding	noncoding	noncoding
MSTRG.190428.1	NFAT5	XLOC_193600	361	coding	noncoding	noncoding	noncoding
MSTRG.190439.1	NFAT5	XLOC_193611	539	coding	noncoding	noncoding	noncoding
XM_017022880.1	WWP2	XLOC_193618	4626	coding	coding	coding	coding
XM_011522826.3	WWP2	XLOC_193618	4392	coding	coding	coding	coding
MSTRG.190474.1	WWP2	XLOC_193622	303	coding	noncoding	noncoding	noncoding
MSTRG.190480.1	WWP2	XLOC_193627	291	coding	noncoding	noncoding	noncoding
MSTRG.190567.1	LOC100506083	XLOC_193648	348	coding	noncoding	noncoding	noncoding
MSTRG.190572.5	ST3GAL2	XLOC_193650	3560	coding	coding	coding	coding
MSTRG.190498.1	SF3B3	XLOC_193656	319	coding	noncoding	noncoding	noncoding
MSTRG.190488.1	SF3B3	XLOC_193661	244	coding	noncoding	noncoding	noncoding
MSTRG.190491.1	IL34	XLOC_193664	265	coding	noncoding	noncoding	noncoding
MSTRG.190506.1	MTSS2	XLOC_193666	341	coding	noncoding	noncoding	noncoding
MSTRG.190591.1	ZNF821	XLOC_193702	224	coding	noncoding	noncoding	noncoding
MSTRG.190609.1		XLOC_193704	253	coding	noncoding	noncoding	noncoding
MSTRG.190694.1	C16orf47	XLOC_193851	247	coding	noncoding	noncoding	noncoding
MSTRG.190649.1		XLOC_193855	223	coding	noncoding	noncoding	noncoding
MSTRG.190651.1		XLOC_193856	267	coding	noncoding	noncoding	noncoding
MSTRG.190652.1		XLOC_193857	239	coding	noncoding	noncoding	noncoding
MSTRG.190657.1		XLOC_193861	293	coding	noncoding	noncoding	noncoding
MSTRG.190720.3	LOC283922	XLOC_193872	3434	coding	coding	coding	coding
MSTRG.190701.1	NPIPB15	XLOC_193879	205	coding	noncoding	noncoding	noncoding
MSTRG.190706.1	CLEC18B	XLOC_193881	692	coding	noncoding	noncoding	noncoding
MSTRG.190708.1	LOC107984827	XLOC_193883	414	coding	noncoding	noncoding	noncoding
MSTRG.190748.1		XLOC_193892	237	coding	noncoding	noncoding	noncoding
MSTRG.190955.1	ZNRF1	XLOC_193903	281	coding	noncoding	noncoding	noncoding
MSTRG.190924.1	LDHD	XLOC_193906	249	coding	coding	noncoding	noncoding
MSTRG.190933.1		XLOC_193914	389	coding	noncoding	noncoding	noncoding

NR_136216.1	CNTNAP4	XLOC_193943	6669	coding	coding	coding	coding
MSTRG.191050.1		XLOC_193956	641	coding	noncoding	noncoding	noncoding
MSTRG.191108.1	VAT1L	XLOC_193978	447	coding	noncoding	noncoding	noncoding
MSTRG.192329.1	WWOX	XLOC_194009	201	coding	noncoding	noncoding	noncoding
MSTRG.192450.1	WWOX	XLOC_194032	305	coding	noncoding	noncoding	noncoding
MSTRG.192481.1	WWOX	XLOC_194037	256	coding	noncoding	noncoding	noncoding
MSTRG.192515.1	MAF	XLOC_194043	218	coding	noncoding	noncoding	noncoding
MSTRG.191135.1	MAFTRR	XLOC_194087	283	coding	noncoding	noncoding	noncoding
MSTRG.191140.1	MAFTRR	XLOC_194090	214	coding	noncoding	noncoding	noncoding
MSTRG.191199.1	GCSH	XLOC_194151	262	coding	noncoding	noncoding	noncoding
MSTRG.191241.1	BCO1	XLOC_194153	240	coding	noncoding	noncoding	noncoding
MSTRG.191254.1		XLOC_194166	249	coding	noncoding	noncoding	noncoding
MSTRG.191255.1		XLOC_194167	354	coding	noncoding	noncoding	noncoding
MSTRG.191377.1		XLOC_194204	264	coding	noncoding	noncoding	noncoding
XR_001751898.2	HSD17B2	XLOC_194232	1480	coding	coding	coding	coding
MSTRG.191331.1		XLOC_194259	308	coding	noncoding	noncoding	noncoding
MSTRG.191425.1	CDH13	XLOC_194265	554	coding	noncoding	noncoding	noncoding
MSTRG.191432.1	CDH13	XLOC_194269	258	coding	noncoding	noncoding	noncoding
MSTRG.191445.1	CDH13	XLOC_194275	502	coding	noncoding	noncoding	noncoding
MSTRG.191457.1	CDH13	XLOC_194280	258	coding	noncoding	noncoding	noncoding
XM_017023317.2	NECAB2	XLOC_194297	2152	coding	coding	coding	coding
MSTRG.191519.1		XLOC_194309	364	coding	noncoding	noncoding	noncoding
MSTRG.191552.1		XLOC_194324	264	coding	noncoding	noncoding	noncoding
MSTRG.191580.1	KLHL36	XLOC_194342	241	coding	noncoding	noncoding	noncoding
NR_073578.1	USP10	XLOC_194343	3116	coding	coding	coding	coding
MSTRG.191598.1	CRISPLD2	XLOC_194352	230	coding	noncoding	noncoding	noncoding
MSTRG.191603.1	ZDHHC7	XLOC_194355	4976	coding	coding	coding	noncoding
MSTRG.191624.1	KIAA0513	XLOC_194365	738	coding	noncoding	noncoding	coding
XM_005255863.4	GSE1	XLOC_194371	7363	coding	coding	coding	coding
XR_001752394.2	LOC107984830	XLOC_194375	4752	coding	noncoding	coding	noncoding
MSTRG.191676.1		XLOC_194430	244	coding	noncoding	noncoding	noncoding
MSTRG.191737.1		XLOC_194451	266	coding	noncoding	noncoding	noncoding
MSTRG.191753.1	LOC105371393	XLOC_194455	273	coding	noncoding	noncoding	noncoding
MSTRG.191831.1	JPH3	XLOC_194499	280	coding	noncoding	noncoding	noncoding
MSTRG.191844.1	KLHDC4	XLOC_194509	1885	coding	coding	coding	coding
MSTRG.191849.1	LOC102724467	XLOC_194511	691	coding	noncoding	noncoding	coding
XM_011523172.2	BANP	XLOC_194517	2663	coding	coding	coding	coding
MSTRG.191916.1	BANP	XLOC_194520	211	coding	noncoding	noncoding	noncoding
XM_017023784.1	ZNF469	XLOC_194532	14433	coding	coding	noncoding	coding
NM_153813.3	ZFPM1	XLOC_194533	5432	coding	coding	coding	coding
XM_017022982.1	ZFPM1	XLOC_194533	3030	coding	coding	coding	coding
MSTRG.191966.1	PIEZO1	XLOC_194559	249	coding	noncoding	noncoding	noncoding
MSTRG.191982.1		XLOC_194600	855	coding	noncoding	coding	noncoding

XM_017023022.1	ACSF3	XLOC_194606	1769	coding	coding	coding	coding
NM_004933.3	CDH15	XLOC_194613	2866	coding	coding	coding	coding
MSTRG.192031.1	SLC22A31	XLOC_194615	280	coding	noncoding	noncoding	noncoding
XM_017023016.1	ZNF778	XLOC_194616	7612	coding	coding	coding	coding
MSTRG.192120.1	ZNF778	XLOC_194617	213	coding	noncoding	noncoding	noncoding
MSTRG.192123.1		XLOC_194619	297	coding	noncoding	noncoding	noncoding
NM_001242885.2	LOC100287036	XLOC_194625	1443	coding	noncoding	coding	noncoding
XM_017023009.1	DPEP1	XLOC_194643	2326	coding	coding	coding	coding
XM_017023806.1	CDK10	XLOC_194649	1829	coding	coding	coding	coding
MSTRG.192097.2	VPS9D1-AS1	XLOC_194654	1925	coding	noncoding	coding	coding
NR_110128.1	ZNF276	XLOC_194654	3481	coding	coding	coding	coding
MSTRG.192088.1	SPIRE2	XLOC_194663	285	coding	noncoding	noncoding	noncoding
MSTRG.192079.1	TUBB3	XLOC_194671	287	coding	noncoding	noncoding	noncoding
XR_001752313.1	LOC101927999	XLOC_194684	2177	coding	coding	coding	coding
MSTRG.185763.1	POLR3K	XLOC_194690	254	coding	noncoding	noncoding	noncoding
MSTRG.185766.8	RHBDF1	XLOC_194692	245	coding	coding	coding	noncoding
MSTRG.185807.2	NPRL3	XLOC_194693	2160	coding	coding	coding	coding
MSTRG.185815.4	HBA1	XLOC_194697	1235	coding	coding	coding	noncoding
MSTRG.185815.19	HBA1	XLOC_194697	1154	coding	coding	coding	noncoding
MSTRG.185815.31	HBA1	XLOC_194697	1094	coding	coding	coding	noncoding
MSTRG.185813.1	LUC7L	XLOC_194700	252	coding	noncoding	noncoding	noncoding
MSTRG.185925.1	RAB40C	XLOC_194730	279	coding	noncoding	noncoding	noncoding
NR_136337.1	LOC105371184	XLOC_194746	716	coding	noncoding	noncoding	noncoding
XR_001751838.1	CCDC78	XLOC_194756	2392	coding	coding	coding	coding
MSTRG.185987.1	SOX8	XLOC_194772	449	coding	coding	noncoding	noncoding
MSTRG.186002.1		XLOC_194782	225	coding	noncoding	noncoding	noncoding
MSTRG.186044.1	CLCN7	XLOC_194795	234	coding	noncoding	noncoding	noncoding
MSTRG.186063.11	IFT140	XLOC_194803	1022	coding	noncoding	noncoding	noncoding
MSTRG.186114.1	MAPK8IP3	XLOC_194820	2864	coding	coding	coding	coding
MSTRG.186100.1	MEIOB	XLOC_194832	257	coding	noncoding	noncoding	noncoding
MSTRG.186163.1		XLOC_194858	309	coding	noncoding	noncoding	noncoding
NM_001286625.1	RNPS1	XLOC_194863	2046	coding	coding	coding	coding
NM_001089.3	ABCA3	XLOC_194864	6602	coding	coding	coding	coding
MSTRG.186238.4	PDPK1	XLOC_194884	1045	coding	coding	coding	coding
MSTRG.186324.3	MMP25-AS1	XLOC_194921	6271	coding	coding	coding	noncoding
MSTRG.186324.10	MMP25-AS1	XLOC_194921	1576	coding	noncoding	noncoding	coding
MSTRG.186372.1		XLOC_194945	698	coding	noncoding	noncoding	noncoding
MSTRG.186377.4	ZNF263	XLOC_194948	2536	coding	coding	coding	noncoding
MSTRG.186385.1		XLOC_194952	267	coding	noncoding	noncoding	noncoding
NM_001353382.1	C16orf90	XLOC_194968	1333	coding	coding	coding	coding
MSTRG.186404.1	LOC101929732	XLOC_194973	298	coding	noncoding	noncoding	noncoding
XM_024450471.1	SLX4	XLOC_194975	9178	coding	coding	coding	coding
MSTRG.186410.1	SLX4	XLOC_194976	215	coding	noncoding	noncoding	noncoding

MSTRG.186424.1	TRAP1	XLOC_194987	267 coding	noncoding	noncoding	noncoding
NM_004380.2	CREBBP	XLOC_194989	10197 coding	coding	coding	coding
MSTRG.186478.1	CREBBP	XLOC_195005	220 coding	noncoding	noncoding	noncoding
MSTRG.186426.1		XLOC_195007	510 coding	noncoding	noncoding	noncoding
MSTRG.186427.1		XLOC_195008	200 coding	noncoding	noncoding	noncoding
MSTRG.186457.1	ADCY9	XLOC_195014	243 coding	noncoding	noncoding	noncoding
MSTRG.186430.1		XLOC_195016	219 coding	noncoding	noncoding	noncoding
NM_001323668.1	SRL	XLOC_195018	5592 coding	coding	coding	coding
NM_021646.4	ZNF500	XLOC_195037	5824 coding	coding	coding	coding
MSTRG.186505.1	SEPTIN12	XLOC_195040	236 coding	noncoding	noncoding	noncoding
MSTRG.186536.1	SEC14L5	XLOC_195045	251 coding	noncoding	noncoding	noncoding
MSTRG.186532.1	NAGPA	XLOC_195049	303 coding	noncoding	noncoding	noncoding
MSTRG.186542.1	NAGPA	XLOC_195051	273 coding	noncoding	noncoding	noncoding
MSTRG.186546.1	C16orf89	XLOC_195055	317 coding	noncoding	noncoding	noncoding
MSTRG.186602.1	RBFOX1	XLOC_195060	272 coding	noncoding	noncoding	noncoding
MSTRG.186609.1	RBFOX1	XLOC_195063	229 coding	noncoding	noncoding	noncoding
MSTRG.186625.1	RBFOX1	XLOC_195070	368 coding	noncoding	noncoding	noncoding
MSTRG.186633.1	RBFOX1	XLOC_195073	275 coding	noncoding	noncoding	noncoding
MSTRG.186642.1	RBFOX1	XLOC_195077	302 coding	noncoding	noncoding	noncoding
MSTRG.186648.1	RBFOX1	XLOC_195080	232 coding	noncoding	noncoding	noncoding
MSTRG.186652.1	RBFOX1	XLOC_195082	200 coding	noncoding	noncoding	noncoding
MSTRG.186655.1	RBFOX1	XLOC_195083	306 coding	noncoding	noncoding	noncoding
MSTRG.186660.1	RBFOX1	XLOC_195086	211 coding	noncoding	noncoding	noncoding
MSTRG.186666.1	RBFOX1	XLOC_195090	304 coding	noncoding	noncoding	noncoding
XR_002957901.1	LOC105371068	XLOC_195092	7986 coding	noncoding	noncoding	noncoding
MSTRG.186691.1	RBFOX1	XLOC_195098	317 coding	noncoding	noncoding	noncoding
MSTRG.186558.1		XLOC_195103	289 coding	noncoding	noncoding	noncoding
MSTRG.186581.1		XLOC_195118	436 coding	noncoding	noncoding	noncoding
MSTRG.186587.1		XLOC_195120	271 coding	noncoding	noncoding	noncoding
MSTRG.186742.1	TMEM186	XLOC_195127	241 coding	noncoding	noncoding	noncoding
MSTRG.186746.2	PMM2	XLOC_195129	701 coding	coding	noncoding	noncoding
NM_001321858.1	USP7	XLOC_195137	5207 coding	coding	coding	coding
MSTRG.186764.1	USP7	XLOC_195140	263 coding	noncoding	noncoding	noncoding
MSTRG.186766.1	USP7	XLOC_195142	243 coding	noncoding	noncoding	noncoding
MSTRG.186706.1		XLOC_195145	477 coding	noncoding	noncoding	noncoding
MSTRG.186738.1		XLOC_195158	244 coding	noncoding	noncoding	noncoding
MSTRG.186791.1	LOC101927026	XLOC_195165	260 coding	noncoding	noncoding	noncoding
MSTRG.186802.1	LOC101927026	XLOC_195173	263 coding	noncoding	noncoding	noncoding
NM_001134407.3	GRIN2A	XLOC_195176	14706 coding	coding	coding	coding
MSTRG.186777.1		XLOC_195193	293 coding	noncoding	noncoding	noncoding
MSTRG.186826.1		XLOC_195199	315 coding	noncoding	noncoding	noncoding
MSTRG.186832.1	TEKT5	XLOC_195204	613 coding	coding	noncoding	noncoding
MSTRG.186838.1	TVP23A	XLOC_195207	262 coding	noncoding	noncoding	noncoding

MSTRG.186856.1		XLOC_195216	201 coding	noncoding	noncoding	noncoding
MSTRG.186931.1	LOC105371082	XLOC_195230	244 coding	noncoding	noncoding	noncoding
MSTRG.186933.1	LOC105371082	XLOC_195232	273 coding	noncoding	coding	noncoding
MSTRG.186893.1	LITAF	XLOC_195245	429 coding	noncoding	noncoding	noncoding
MSTRG.186896.1	LITAF	XLOC_195248	279 coding	noncoding	noncoding	noncoding
MSTRG.186866.1		XLOC_195252	239 coding	noncoding	noncoding	noncoding
MSTRG.186907.1	ZC3H7A	XLOC_195255	289 coding	noncoding	noncoding	noncoding
MSTRG.187044.1	CPPED1	XLOC_195284	698 coding	noncoding	noncoding	noncoding
MSTRG.186973.1		XLOC_195303	256 coding	noncoding	noncoding	noncoding
MSTRG.186975.1		XLOC_195304	261 coding	noncoding	noncoding	noncoding
MSTRG.186976.1		XLOC_195305	252 coding	noncoding	noncoding	noncoding
MSTRG.187019.1	SHISA9	XLOC_195317	337 coding	noncoding	noncoding	noncoding
MSTRG.187070.1		XLOC_195331	250 coding	noncoding	noncoding	noncoding
MSTRG.187067.1		XLOC_195340	242 coding	noncoding	noncoding	noncoding
MSTRG.187069.1		XLOC_195341	305 coding	noncoding	noncoding	noncoding
MSTRG.187081.1		XLOC_195355	251 coding	noncoding	noncoding	noncoding
MSTRG.187083.1	LINC02130	XLOC_195356	294 coding	noncoding	noncoding	noncoding
XR_001751906.2	PARN	XLOC_195358	1682 coding	coding	coding	coding
XR_933120.2	LOC105376749	XLOC_195368	334 coding	coding	noncoding	noncoding
MSTRG.187171.2	NOMO1	XLOC_195370	2126 coding	noncoding	coding	coding
MSTRG.187184.2	PDXDC1	XLOC_195374	417 coding	noncoding	noncoding	noncoding
MSTRG.187182.1	MPV17L	XLOC_195384	289 coding	noncoding	noncoding	noncoding
XM_017023904.2	MARF1	XLOC_195387	5567 coding	coding	coding	coding
XM_017023903.2	MARF1	XLOC_195387	5570 coding	coding	coding	coding
MSTRG.187239.1	PKD1P1	XLOC_195398	337 coding	noncoding	noncoding	noncoding
MSTRG.187251.1		XLOC_195411	217 coding	noncoding	noncoding	noncoding
MSTRG.187256.1		XLOC_195415	215 coding	noncoding	noncoding	noncoding
MSTRG.187258.1		XLOC_195416	303 coding	noncoding	noncoding	noncoding
MSTRG.187269.1		XLOC_195423	294 coding	noncoding	noncoding	noncoding
MSTRG.187280.1	ABCC6P1	XLOC_195439	312 coding	noncoding	noncoding	noncoding
MSTRG.187283.1		XLOC_195441	280 coding	noncoding	noncoding	noncoding
MSTRG.187307.1		XLOC_195444	233 coding	noncoding	noncoding	noncoding
MSTRG.187332.1		XLOC_195461	244 coding	noncoding	noncoding	noncoding
MSTRG.187352.1		XLOC_195474	252 coding	noncoding	noncoding	noncoding
MSTRG.187373.1	LOC105371114	XLOC_195488	252 coding	noncoding	noncoding	noncoding
MSTRG.187378.1	LOC105371114	XLOC_195489	227 coding	noncoding	noncoding	noncoding
MSTRG.187396.1	TMC5	XLOC_195490	298 coding	noncoding	noncoding	noncoding
MSTRG.187436.2	VPS35L	XLOC_195496	2569 coding	noncoding	coding	noncoding
MSTRG.187451.1	IQCK	XLOC_195501	265 coding	noncoding	noncoding	noncoding
MSTRG.187484.1	ACSM2B	XLOC_195524	204 coding	noncoding	noncoding	noncoding
MSTRG.187548.3	LYRM1	XLOC_195553	649 coding	coding	noncoding	noncoding
XM_011545882.2	DNAH3	XLOC_195556	12972 coding	coding	noncoding	coding
MSTRG.187465.1	DNAH3	XLOC_195561	217 coding	noncoding	noncoding	noncoding

MSTRG.187470.1	DNAH3	XLOC_195564	240 coding	noncoding	noncoding	noncoding
MSTRG.187641.1	SCNN1B	XLOC_195619	247 coding	noncoding	noncoding	noncoding
MSTRG.187658.1	COG7	XLOC_195623	252 coding	noncoding	noncoding	noncoding
MSTRG.187674.1	EARS2	XLOC_195628	386 coding	noncoding	noncoding	noncoding
MSTRG.187676.1	EARS2	XLOC_195630	293 coding	noncoding	noncoding	noncoding
MSTRG.187689.1	PLK1	XLOC_195638	214 coding	noncoding	noncoding	noncoding
MSTRG.187661.1		XLOC_195641	378 coding	noncoding	noncoding	noncoding
MSTRG.187665.1		XLOC_195643	266 coding	noncoding	noncoding	noncoding
MSTRG.187713.12	PRKCB	XLOC_195645	8649 coding	coding	coding	noncoding
MSTRG.187715.1	PRKCB	XLOC_195646	1840 coding	noncoding	noncoding	noncoding
MSTRG.187697.1	LOC105371143	XLOC_195652	228 coding	noncoding	noncoding	noncoding
MSTRG.187770.1		XLOC_195669	243 coding	noncoding	noncoding	noncoding
MSTRG.187775.2	LCMT1	XLOC_195671	968 coding	coding	noncoding	noncoding
MSTRG.187804.1		XLOC_195690	309 coding	noncoding	noncoding	noncoding
MSTRG.187852.1		XLOC_195712	224 coding	noncoding	noncoding	noncoding
MSTRG.187873.1	NSMCE1-DT	XLOC_195722	218 coding	noncoding	noncoding	noncoding
MSTRG.187908.1	GSG1L	XLOC_195748	291 coding	noncoding	noncoding	noncoding
MSTRG.187891.1		XLOC_195753	511 coding	noncoding	noncoding	noncoding
NM_001270940.2	XPO6	XLOC_195757	4673 coding	coding	coding	coding
MSTRG.187936.10	XPO6	XLOC_195757	575 coding	coding	coding	coding
MSTRG.187921.1	SBK1	XLOC_195792	245 coding	noncoding	noncoding	noncoding
MSTRG.187924.1	SBK1	XLOC_195794	2954 coding	noncoding	coding	coding
NM_001317856.1	EIF3CL	XLOC_195798	3140 coding	coding	coding	coding
MSTRG.188048.1	CLN3	XLOC_195803	238 coding	noncoding	noncoding	noncoding
MSTRG.188013.1	ATP2A1	XLOC_195827	345 coding	coding	noncoding	noncoding
MSTRG.188031.1		XLOC_195835	273 coding	noncoding	noncoding	noncoding
MSTRG.188032.1		XLOC_195836	249 coding	noncoding	noncoding	noncoding
MSTRG.188206.8	RRN3P2	XLOC_195839	1098 coding	coding	noncoding	noncoding
MSTRG.188246.1		XLOC_195857	681 coding	noncoding	noncoding	noncoding
MSTRG.188247.1		XLOC_195858	1181 coding	noncoding	noncoding	noncoding
MSTRG.188279.1	SMG1P2	XLOC_195880	285 coding	noncoding	noncoding	noncoding
MSTRG.188082.1		XLOC_195881	209 coding	noncoding	noncoding	noncoding
MSTRG.188086.5	SPN	XLOC_195882	1173 coding	noncoding	noncoding	noncoding
MSTRG.188091.1	ZG16	XLOC_195887	302 coding	noncoding	noncoding	noncoding
MSTRG.188093.1	ZG16	XLOC_195888	325 coding	noncoding	noncoding	noncoding
MSTRG.188102.1	SEZ6L2	XLOC_195895	241 coding	noncoding	noncoding	noncoding
NM_001040056.3	MAPK3	XLOC_195913	1085 coding	coding	coding	coding
MSTRG.188318.4	LOC730183	XLOC_195959	12628 coding	coding	coding	coding
MSTRG.188328.1	BCL7C	XLOC_195966	222 coding	noncoding	noncoding	noncoding
MSTRG.188326.1		XLOC_195971	205 coding	noncoding	noncoding	noncoding
MSTRG.188343.1	FBXL19	XLOC_195973	680 coding	coding	coding	noncoding
MSTRG.188345.1	FBXL19	XLOC_195975	248 coding	noncoding	noncoding	noncoding
MSTRG.188394.7	ZNF646	XLOC_195988	1958 coding	coding	coding	noncoding

MSTRG.188381.1	PRSS36	XLOC_195994	264	coding	noncoding	noncoding	noncoding
MSTRG.188376.1		XLOC_195997	291	coding	noncoding	noncoding	noncoding
MSTRG.188377.1		XLOC_195998	265	coding	noncoding	noncoding	noncoding
MSTRG.188385.1		XLOC_196004	214	coding	noncoding	noncoding	noncoding
MSTRG.188471.5	ITGAM	XLOC_196009	4632	coding	coding	coding	coding
MSTRG.188466.11	ITGAX	XLOC_196010	709	coding	noncoding	noncoding	noncoding
MSTRG.188419.1	ZNF843	XLOC_196014	273	coding	noncoding	noncoding	noncoding
MSTRG.188445.1		XLOC_196027	319	coding	noncoding	noncoding	noncoding
MSTRG.188436.1		XLOC_196030	241	coding	noncoding	noncoding	noncoding
MSTRG.188460.1		XLOC_196036	254	coding	noncoding	noncoding	noncoding
MSTRG.188458.1		XLOC_196037	205	coding	noncoding	noncoding	noncoding
MSTRG.188585.11	ZNF267	XLOC_196043	3380	coding	coding	coding	noncoding
MSTRG.188510.1	LOC102725101	XLOC_196056	223	coding	noncoding	noncoding	noncoding
MSTRG.188519.1	LOC102725101	XLOC_196062	331	coding	noncoding	noncoding	noncoding
MSTRG.188520.1	LOC102725101	XLOC_196063	239	coding	noncoding	noncoding	noncoding
MSTRG.188490.1		XLOC_196070	270	coding	noncoding	noncoding	noncoding
MSTRG.188525.1		XLOC_196074	320	coding	noncoding	noncoding	noncoding
MSTRG.188608.1	LINC01566	XLOC_196110	296	coding	noncoding	noncoding	noncoding
MSTRG.188577.1		XLOC_196111	220	coding	noncoding	noncoding	noncoding
MSTRG.188614.1		XLOC_196115	241	coding	noncoding	noncoding	noncoding
MSTRG.188615.1		XLOC_196120	476	coding	noncoding	noncoding	noncoding
MSTRG.188669.1	SHCBP1	XLOC_196140	248	coding	noncoding	noncoding	noncoding
NM_182493.3	MYLK3	XLOC_196147	8033	coding	coding	coding	coding
MSTRG.188658.1		XLOC_196150	788	coding	noncoding	noncoding	noncoding
MSTRG.188683.1		XLOC_196154	298	coding	noncoding	noncoding	noncoding
MSTRG.188682.1		XLOC_196155	617	coding	noncoding	noncoding	noncoding
MSTRG.188689.1		XLOC_196158	307	coding	noncoding	noncoding	noncoding
MSTRG.188696.1		XLOC_196159	248	coding	noncoding	noncoding	noncoding
MSTRG.188727.1	NETO2	XLOC_196173	387	coding	noncoding	noncoding	noncoding
MSTRG.188895.17	PHKB	XLOC_196179	1332	coding	coding	noncoding	noncoding
MSTRG.188785.1	ABCC11	XLOC_196201	342	coding	noncoding	noncoding	noncoding
MSTRG.188756.1		XLOC_196203	279	coding	noncoding	noncoding	noncoding
MSTRG.188804.1		XLOC_196245	261	coding	noncoding	noncoding	noncoding
MSTRG.188808.1	LOC105371241	XLOC_196250	271	coding	noncoding	noncoding	noncoding
XR_933534.2	LOC105371244	XLOC_196257	4673	coding	noncoding	coding	noncoding
XM_006721171.4	ZNF423	XLOC_196259	9233	coding	coding	coding	coding
MSTRG.188850.1	ZNF423	XLOC_196261	309	coding	noncoding	noncoding	noncoding
MSTRG.188842.1		XLOC_196266	250	coding	noncoding	noncoding	noncoding
MSTRG.188868.1		XLOC_196272	313	coding	noncoding	noncoding	noncoding
MSTRG.188970.1	BRD7	XLOC_196275	9014	coding	coding	coding	coding
MSTRG.188970.3	ADCY7	XLOC_196275	586	coding	noncoding	coding	noncoding
MSTRG.188970.6	BRD7	XLOC_196275	9944	coding	coding	coding	coding
MSTRG.188923.1		XLOC_196280	446	coding	noncoding	noncoding	noncoding

MSTRG.189047.7	NOD2	XLOC_196294	3245 coding	coding	noncoding	noncoding
MSTRG.188988.1		XLOC_196315	297 coding	noncoding	noncoding	noncoding
MSTRG.188991.1		XLOC_196321	257 coding	noncoding	noncoding	noncoding
MSTRG.188995.1	C16orf97	XLOC_196325	245 coding	noncoding	noncoding	noncoding
MSTRG.189023.1		XLOC_196339	409 coding	noncoding	noncoding	noncoding
MSTRG.189035.1		XLOC_196346	262 coding	noncoding	noncoding	noncoding
MSTRG.189080.1		XLOC_196384	209 coding	noncoding	noncoding	noncoding
MSTRG.189090.1		XLOC_196387	267 coding	noncoding	noncoding	noncoding
MSTRG.189106.1		XLOC_196395	265 coding	noncoding	noncoding	noncoding
MSTRG.189115.1		XLOC_196399	258 coding	noncoding	noncoding	noncoding
MSTRG.189117.1		XLOC_196400	304 coding	noncoding	noncoding	noncoding
MSTRG.189177.1	CES1P2	XLOC_196427	257 coding	noncoding	noncoding	noncoding
MSTRG.189189.1	CES1	XLOC_196430	315 coding	noncoding	noncoding	noncoding
MSTRG.189244.1		XLOC_196476	244 coding	noncoding	noncoding	noncoding
MSTRG.189271.1	LOC105371286	XLOC_196490	240 coding	noncoding	noncoding	noncoding
MSTRG.189328.1	SLC12A3	XLOC_196501	266 coding	noncoding	noncoding	noncoding
MSTRG.189431.16	NLRC5	XLOC_196509	3661 coding	coding	coding	coding
MSTRG.189409.1		XLOC_196517	263 coding	noncoding	noncoding	noncoding
MSTRG.189410.1		XLOC_196518	226 coding	noncoding	noncoding	noncoding
MSTRG.189416.1		XLOC_196522	207 coding	noncoding	noncoding	noncoding
MSTRG.189468.1	CCDC102A	XLOC_196527	313 coding	noncoding	noncoding	noncoding
XM_024450266.1	KIFC3	XLOC_196543	3829 coding	coding	coding	coding
MSTRG.189498.1	KIFC3	XLOC_196544	238 coding	noncoding	noncoding	noncoding
MSTRG.189505.1	KIFC3	XLOC_196547	306 coding	noncoding	noncoding	noncoding
XM_005256069.4	ZNF319	XLOC_196555	4143 coding	coding	coding	coding
MSTRG.189548.1	CCDC113	XLOC_196564	221 coding	noncoding	noncoding	noncoding
MSTRG.189550.1	CCDC113	XLOC_196566	248 coding	noncoding	noncoding	noncoding
MSTRG.189607.6	CNOT1	XLOC_196577	2107 coding	coding	coding	coding
MSTRG.189553.1		XLOC_196587	242 coding	noncoding	noncoding	noncoding
MSTRG.189594.1		XLOC_196610	269 coding	noncoding	noncoding	noncoding
NM_001282301.2	NPAP1L	XLOC_196628	2115 coding	coding	coding	coding
MSTRG.189683.1		XLOC_196637	281 coding	noncoding	noncoding	noncoding
MSTRG.189685.1		XLOC_196638	288 coding	noncoding	noncoding	noncoding
MSTRG.189721.1		XLOC_196666	285 coding	noncoding	noncoding	noncoding
MSTRG.189726.1		XLOC_196671	204 coding	noncoding	noncoding	noncoding
MSTRG.189759.1		XLOC_196678	304 coding	noncoding	noncoding	noncoding
MSTRG.189777.1		XLOC_196708	209 coding	noncoding	noncoding	noncoding
MSTRG.189786.1		XLOC_196711	292 coding	noncoding	noncoding	noncoding
MSTRG.189788.1		XLOC_196713	247 coding	noncoding	noncoding	noncoding
MSTRG.189798.1		XLOC_196718	303 coding	noncoding	noncoding	noncoding
MSTRG.189834.1		XLOC_196722	318 coding	noncoding	noncoding	noncoding
MSTRG.189857.1		XLOC_196737	290 coding	noncoding	noncoding	noncoding
MSTRG.189888.1		XLOC_196760	296 coding	noncoding	noncoding	noncoding

MSTRG.190042.1		XLOC_196780	498 coding	noncoding	noncoding	noncoding
MSTRG.190073.1	BEAN1	XLOC_196792	258 coding	noncoding	noncoding	noncoding
XM_011523005.2	TERB1	XLOC_196807	3048 coding	coding	coding	coding
MSTRG.190123.1	TERB1	XLOC_196808	293 coding	noncoding	noncoding	noncoding
MSTRG.190127.1		XLOC_196821	211 coding	noncoding	noncoding	noncoding
MSTRG.190181.2	TMEM208	XLOC_196836	329 coding	coding	noncoding	noncoding
MSTRG.190169.1	SLC9A5	XLOC_196837	236 coding	noncoding	noncoding	noncoding
MSTRG.190186.1	LRRC36	XLOC_196840	310 coding	noncoding	noncoding	noncoding
MSTRG.190188.1	LRRC36	XLOC_196842	250 coding	noncoding	noncoding	noncoding
MSTRG.190274.1	RANBP10	XLOC_196877	308 coding	noncoding	noncoding	noncoding
MSTRG.190275.1	RANBP10	XLOC_196878	738 coding	noncoding	noncoding	noncoding
MSTRG.190297.1		XLOC_196893	242 coding	noncoding	noncoding	noncoding
XM_005256031.4	SMPD3	XLOC_196905	5716 coding	noncoding	coding	coding
MSTRG.190340.3	ZFP90	XLOC_196915	3169 coding	coding	noncoding	coding
MSTRG.190329.1		XLOC_196916	285 coding	noncoding	noncoding	noncoding
MSTRG.190333.1	CDH3	XLOC_196919	233 coding	noncoding	noncoding	noncoding
MSTRG.190355.2	TANGO6	XLOC_196925	2916 coding	coding	coding	noncoding
MSTRG.190371.1		XLOC_196930	314 coding	noncoding	noncoding	noncoding
XR_001751974.1	TERF2	XLOC_196936	3046 coding	coding	coding	coding
MSTRG.190385.1		XLOC_196938	235 coding	noncoding	noncoding	noncoding
MSTRG.190396.1		XLOC_196944	237 coding	noncoding	noncoding	noncoding
NM_014062.3	NOB1	XLOC_196951	1716 coding	coding	coding	coding
MSTRG.190469.2	WWP2	XLOC_196954	5475 coding	coding	coding	coding
MSTRG.190469.6	WWP2	XLOC_196954	909 coding	coding	noncoding	noncoding
MSTRG.190487.1	CLEC18A	XLOC_196962	207 coding	noncoding	noncoding	noncoding
MSTRG.190454.1	PDXDC2P-NPIPB14P	XLOC_196969	285 coding	noncoding	noncoding	noncoding
MSTRG.190563.1	AARS	XLOC_196982	267 coding	noncoding	noncoding	noncoding
MSTRG.190564.1	AARS	XLOC_196983	239 coding	noncoding	noncoding	noncoding
MSTRG.190576.1	ST3GAL2	XLOC_196991	281 coding	noncoding	noncoding	noncoding
MSTRG.190504.1	MTSS2	XLOC_197001	260 coding	noncoding	noncoding	noncoding
XM_011523148.1	HYDIN	XLOC_197008	21115 coding	coding	noncoding	coding
MSTRG.190517.1	HYDIN	XLOC_197009	233 coding	noncoding	noncoding	noncoding
MSTRG.190521.1	HYDIN	XLOC_197011	353 coding	noncoding	noncoding	noncoding
NM_001304493.2	ZNF23	XLOC_197020	2799 coding	coding	coding	coding
MSTRG.190550.1	CHST4	XLOC_197023	230 coding	noncoding	noncoding	noncoding
MSTRG.190536.1		XLOC_197025	265 coding	noncoding	noncoding	noncoding
MSTRG.190596.1	MARVELD3	XLOC_197028	222 coding	noncoding	noncoding	noncoding
MSTRG.190602.1	AP1G1	XLOC_197033	463 coding	noncoding	noncoding	noncoding
XM_017023419.1	ZNF821	XLOC_197043	1847 coding	noncoding	coding	coding
MSTRG.190610.1		XLOC_197046	253 coding	noncoding	noncoding	noncoding
MSTRG.190613.4	IST1	XLOC_197047	2442 coding	coding	noncoding	coding
MSTRG.190624.1	PKD1L3	XLOC_197050	267 coding	noncoding	noncoding	noncoding
MSTRG.190625.1	PKD1L3	XLOC_197051	242 coding	noncoding	noncoding	noncoding

MSTRG.190897.1	ZFHX3	XLOC_197098	207 coding	noncoding	noncoding	noncoding
MSTRG.190663.1	C16orf47	XLOC_197106	322 coding	noncoding	noncoding	noncoding
MSTRG.190643.1		XLOC_197119	238 coding	noncoding	noncoding	noncoding
MSTRG.190650.1		XLOC_197123	223 coding	noncoding	noncoding	noncoding
MSTRG.190696.1		XLOC_197128	325 coding	noncoding	noncoding	noncoding
MSTRG.190727.1	LOC283922	XLOC_197131	289 coding	noncoding	noncoding	noncoding
XR_002957808.1	CLEC18B	XLOC_197136	2581 coding	coding	coding	coding
MSTRG.190709.1	LOC107984827	XLOC_197137	414 coding	noncoding	noncoding	noncoding
MSTRG.190916.1	MLKL	XLOC_197157	266 coding	noncoding	noncoding	noncoding
MSTRG.190749.1		XLOC_197158	247 coding	noncoding	noncoding	noncoding
MSTRG.190751.1		XLOC_197162	349 coding	noncoding	noncoding	noncoding
MSTRG.190759.1		XLOC_197163	236 coding	noncoding	noncoding	noncoding
MSTRG.190929.1	ZFP1	XLOC_197197	389 coding	noncoding	noncoding	noncoding
MSTRG.191000.1	CFDP1	XLOC_197207	206 coding	noncoding	noncoding	noncoding
NR_074083.2	TMEM231	XLOC_197213	4370 coding	coding	coding	coding
MSTRG.191061.1		XLOC_197256	348 coding	coding	noncoding	noncoding
MSTRG.191088.1	ADAMTS18	XLOC_197266	293 coding	noncoding	noncoding	noncoding
MSTRG.191109.1	VAT1L	XLOC_197277	262 coding	noncoding	noncoding	noncoding
MSTRG.191097.1		XLOC_197283	319 coding	noncoding	noncoding	noncoding
MSTRG.192443.1	WWOX	XLOC_197378	739 coding	noncoding	noncoding	noncoding
XM_017023234.2	MAF	XLOC_197436	6560 coding	coding	coding	coding
MSTRG.192516.1	MAF	XLOC_197439	299 coding	noncoding	noncoding	noncoding
MSTRG.192524.1	MAF	XLOC_197442	271 coding	noncoding	noncoding	noncoding
MSTRG.192536.1	MAF	XLOC_197445	246 coding	noncoding	noncoding	noncoding
MSTRG.191180.1	LOC102724084	XLOC_197486	219 coding	noncoding	noncoding	noncoding
MSTRG.191197.1	GCSH	XLOC_197515	309 coding	noncoding	noncoding	noncoding
MSTRG.191375.7		XLOC_197535	1168 coding	noncoding	noncoding	noncoding
MSTRG.191309.1		XLOC_197556	225 coding	noncoding	noncoding	noncoding
MSTRG.191424.1	CDH13	XLOC_197575	554 coding	noncoding	noncoding	noncoding
MSTRG.191431.1	CDH13	XLOC_197578	295 coding	noncoding	noncoding	noncoding
MSTRG.191433.1	CDH13	XLOC_197579	222 coding	noncoding	noncoding	noncoding
MSTRG.191464.1	CDH13	XLOC_197597	249 coding	noncoding	noncoding	noncoding
MSTRG.191500.1	HSDL1	XLOC_197631	244 coding	noncoding	noncoding	noncoding
MSTRG.191525.15	TAF1C	XLOC_197632	595 coding	noncoding	noncoding	noncoding
MSTRG.191517.1	ADAD2	XLOC_197635	261 coding	coding	noncoding	noncoding
MSTRG.191512.1		XLOC_197636	313 coding	noncoding	noncoding	noncoding
MSTRG.191518.1		XLOC_197638	244 coding	noncoding	noncoding	noncoding
MSTRG.191530.1	ATP2C2	XLOC_197642	284 coding	noncoding	noncoding	noncoding
MSTRG.191553.1		XLOC_197651	240 coding	noncoding	noncoding	noncoding
MSTRG.191608.1	FAM92B	XLOC_197676	245 coding	noncoding	noncoding	noncoding
MSTRG.191680.1	GSE1	XLOC_197681	229 coding	noncoding	coding	noncoding
MSTRG.191682.1	GSE1	XLOC_197682	319 coding	noncoding	noncoding	noncoding
MSTRG.191725.1	FOXL1	XLOC_197719	249 coding	noncoding	noncoding	noncoding

MSTRG.191738.1		XLOC_197727	266 coding	noncoding	noncoding	noncoding
XM_017023082.2	ZCCHC14	XLOC_197751	3874 coding	coding	coding	coding
MSTRG.191832.1	JPH3	XLOC_197755	280 coding	noncoding	noncoding	noncoding
MSTRG.191846.1	KLHDC4	XLOC_197759	287 coding	noncoding	noncoding	noncoding
MSTRG.191850.1	LOC105371399	XLOC_197761	257 coding	noncoding	noncoding	noncoding
MSTRG.191824.1	CA5A	XLOC_197769	255 coding	noncoding	noncoding	noncoding
MSTRG.191883.1		XLOC_197822	1806 coding	noncoding	coding	noncoding
NM_178310.4	SNAI3	XLOC_197834	1740 coding	coding	coding	coding
MSTRG.191957.2	PIEZO1	XLOC_197839	313 coding	noncoding	noncoding	noncoding
MSTRG.191984.1		XLOC_197861	250 coding	noncoding	noncoding	noncoding
XR_933890.2	LOC105371413	XLOC_197872	2546 coding	noncoding	coding	noncoding
MSTRG.192126.19	ANKRD11	XLOC_197873	413 coding	noncoding	coding	noncoding
MSTRG.192145.1	ANKRD11	XLOC_197885	248 coding	noncoding	noncoding	noncoding
MSTRG.192044.4	SPG7	XLOC_197895	1380 coding	coding	coding	noncoding
MSTRG.192087.1	SPIRE2	XLOC_197916	285 coding	noncoding	noncoding	noncoding
MSTRG.192080.1		XLOC_197920	287 coding	noncoding	noncoding	noncoding
MSTRG.192106.4	DEF8	XLOC_197921	1502 coding	coding	coding	coding
MSTRG.192193.1	FAM157C	XLOC_197936	3369 coding	noncoding	noncoding	noncoding
MSTRG.192200.1	FAM157C	XLOC_197937	845 coding	coding	coding	coding
MSTRG.192201.1	FAM157C	XLOC_197938	227 coding	noncoding	noncoding	noncoding
MSTRG.192246.1		XLOC_197987	265 coding	noncoding	noncoding	noncoding
MSTRG.192248.1		XLOC_197988	265 coding	noncoding	noncoding	noncoding
MSTRG.192281.1	SLC43A2	XLOC_198006	846 coding	noncoding	noncoding	noncoding
MSTRG.192577.1	RTN4RL1	XLOC_198029	247 coding	noncoding	noncoding	noncoding
MSTRG.192579.1	RTN4RL1	XLOC_198031	221 coding	noncoding	noncoding	noncoding
MSTRG.192571.1		XLOC_198035	242 coding	noncoding	noncoding	noncoding
XR_002958096.1	SGSM2	XLOC_198041	5734 coding	coding	coding	coding
MSTRG.192608.1	MNT	XLOC_198046	1412 coding	noncoding	noncoding	coding
MSTRG.192600.1		XLOC_198054	243 coding	noncoding	noncoding	noncoding
MSTRG.192602.1		XLOC_198056	238 coding	noncoding	noncoding	noncoding
XM_011523901.2	PAFAH1B1	XLOC_198057	5331 coding	coding	coding	coding
MSTRG.192693.1	RAP1GAP2	XLOC_198074	266 coding	noncoding	noncoding	noncoding
MSTRG.192706.1	TRPV1	XLOC_198098	363 coding	noncoding	noncoding	noncoding
MSTRG.192707.1	TRPV1	XLOC_198099	458 coding	noncoding	noncoding	noncoding
MSTRG.192728.1	ITGAE	XLOC_198109	332 coding	noncoding	noncoding	noncoding
MSTRG.192745.1	ATP2A3	XLOC_198120	5129 coding	coding	coding	coding
NR_146884.1	LINC01975	XLOC_198121	1795 coding	noncoding	coding	noncoding
XM_017024183.2	CYB5D2	XLOC_198122	1300 coding	coding	coding	coding
MSTRG.192750.1		XLOC_198126	239 coding	noncoding	noncoding	noncoding
MSTRG.192751.1		XLOC_198127	250 coding	noncoding	noncoding	noncoding
MSTRG.192753.1		XLOC_198128	280 coding	noncoding	noncoding	noncoding
MSTRG.192801.1	LOC105371499	XLOC_198147	234 coding	noncoding	noncoding	noncoding
XR_002958023.1	PLD2	XLOC_198163	3783 coding	coding	coding	coding

MSTRG.192843.11	PLD2	XLOC_198163	226 coding	noncoding	noncoding	noncoding
MSTRG.192842.1		XLOC_198164	250 coding	noncoding	noncoding	noncoding
MSTRG.192852.1	MINK1	XLOC_198167	217 coding	noncoding	noncoding	noncoding
MSTRG.192854.1	MINK1	XLOC_198169	287 coding	noncoding	noncoding	noncoding
XM_017024427.2	RNF167	XLOC_198172	1777 coding	coding	coding	coding
MSTRG.192876.1	INCA1	XLOC_198178	277 coding	noncoding	noncoding	noncoding
MSTRG.192970.11	NLRP1	XLOC_198204	1619 coding	coding	coding	coding
MSTRG.192913.1	LOC339166	XLOC_198214	269 coding	noncoding	noncoding	noncoding
MSTRG.192930.1		XLOC_198224	236 coding	noncoding	noncoding	noncoding
MSTRG.192943.1	PITPNM3	XLOC_198229	238 coding	noncoding	coding	noncoding
MSTRG.192947.1	PITPNM3	XLOC_198231	298 coding	noncoding	noncoding	noncoding
MSTRG.193035.1	ALOX15P1	XLOC_198239	324 coding	noncoding	noncoding	noncoding
MSTRG.193088.1	ALOX12-AS1	XLOC_198276	292 coding	noncoding	noncoding	noncoding
MSTRG.193081.1		XLOC_198283	457 coding	noncoding	noncoding	noncoding
XR_934022.2	ACADVL	XLOC_198297	2098 coding	coding	coding	coding
NM_001198623.1	TNFSF13	XLOC_198321	2192 coding	coding	coding	coding
NM_020877.4	DNAH2	XLOC_198335	14532 coding	coding	noncoding	coding
XM_006721423.3	CHD3	XLOC_198342	7439 coding	coding	coding	coding
MSTRG.193199.1	CNTROB	XLOC_198351	240 coding	noncoding	noncoding	noncoding
NM_000180.3	GUCY2D	XLOC_198358	3623 coding	coding	coding	coding
MSTRG.193219.1		XLOC_198385	466 coding	noncoding	noncoding	noncoding
XM_024450804.1	PFAS	XLOC_198392	5584 coding	coding	coding	coding
MSTRG.193275.1		XLOC_198401	269 coding	noncoding	noncoding	noncoding
MSTRG.193290.1		XLOC_198402	212 coding	noncoding	noncoding	noncoding
MSTRG.193287.1		XLOC_198403	274 coding	noncoding	noncoding	noncoding
MSTRG.193282.4	RPL26	XLOC_198409	560 coding	coding	noncoding	noncoding
MSTRG.193351.1	MYH10	XLOC_198422	256 coding	noncoding	noncoding	noncoding
MSTRG.193355.1		XLOC_198455	279 coding	noncoding	noncoding	noncoding
MSTRG.193359.1	NTN1	XLOC_198457	284 coding	noncoding	noncoding	noncoding
MSTRG.193361.1	NTN1	XLOC_198459	305 coding	noncoding	noncoding	noncoding
MSTRG.193390.1	USP43	XLOC_198474	277 coding	noncoding	noncoding	noncoding
MSTRG.193391.1	USP43	XLOC_198475	274 coding	noncoding	noncoding	noncoding
MSTRG.193448.1		XLOC_198485	222 coding	noncoding	noncoding	noncoding
MSTRG.193450.1		XLOC_198486	259 coding	noncoding	noncoding	noncoding
MSTRG.193449.1		XLOC_198488	233 coding	noncoding	noncoding	noncoding
MSTRG.193465.1		XLOC_198510	285 coding	noncoding	noncoding	noncoding
NM_207386.4	SHISA6	XLOC_198522	7625 coding	coding	coding	coding
MSTRG.193491.1	SHISA6	XLOC_198523	322 coding	noncoding	noncoding	noncoding
MSTRG.193717.1	DNAH9	XLOC_198536	306 coding	noncoding	noncoding	noncoding
MSTRG.193726.1	LOC101928350	XLOC_198541	237 coding	noncoding	noncoding	noncoding
MSTRG.193727.1	LOC101928350	XLOC_198542	251 coding	noncoding	noncoding	noncoding
XM_005256753.3	MAP2K4	XLOC_198551	3636 coding	coding	coding	coding
MSTRG.193752.1	MAP2K4	XLOC_198562	294 coding	noncoding	noncoding	noncoding

XM_005256864.1	MYOCD	XLOC_198593	8609 coding	coding	coding	coding
MSTRG.193611.1	MYOCD	XLOC_198594	290 coding	noncoding	noncoding	noncoding
MSTRG.193619.1	MYOCD	XLOC_198597	250 coding	noncoding	noncoding	noncoding
MSTRG.193575.1		XLOC_198603	314 coding	noncoding	noncoding	noncoding
MSTRG.193582.1		XLOC_198608	236 coding	noncoding	noncoding	noncoding
MSTRG.193585.1		XLOC_198610	268 coding	noncoding	noncoding	noncoding
MSTRG.193649.1		XLOC_198700	304 coding	noncoding	noncoding	noncoding
MSTRG.193654.1	HS3ST3B1	XLOC_198703	268 coding	noncoding	noncoding	noncoding
MSTRG.193647.1		XLOC_198712	296 coding	noncoding	noncoding	noncoding
MSTRG.193667.1		XLOC_198717	217 coding	noncoding	noncoding	noncoding
MSTRG.193684.1		XLOC_198725	260 coding	noncoding	noncoding	noncoding
MSTRG.193709.1	CDRT1	XLOC_198749	264 coding	noncoding	noncoding	noncoding
MSTRG.193703.1		XLOC_198752	349 coding	noncoding	noncoding	noncoding
MSTRG.193813.1		XLOC_198767	321 coding	noncoding	noncoding	noncoding
XM_011523659.3	ADORA2B	XLOC_198770	1565 coding	coding	coding	coding
MSTRG.194023.1	ADORA2B	XLOC_198771	269 coding	noncoding	noncoding	noncoding
MSTRG.194031.6	TTC19	XLOC_198776	15891 coding	coding	coding	coding
MSTRG.194031.13	TTC19	XLOC_198776	2858 coding	noncoding	noncoding	noncoding
MSTRG.194031.14	NCOR1	XLOC_198776	9397 coding	coding	coding	noncoding
MSTRG.193933.1		XLOC_198795	250 coding	noncoding	noncoding	noncoding
XM_017025429.2	CCDC144A	XLOC_198804	7409 coding	coding	coding	coding
MSTRG.193958.1	TBC1D27P	XLOC_198815	553 coding	noncoding	noncoding	noncoding
MSTRG.193991.1	MPRIP	XLOC_198824	295 coding	noncoding	noncoding	noncoding
MSTRG.193992.1	MPRIP	XLOC_198825	236 coding	noncoding	noncoding	noncoding
MSTRG.193985.1	FLCN	XLOC_198835	285 coding	noncoding	noncoding	noncoding
MSTRG.194006.1		XLOC_198847	313 coding	noncoding	coding	noncoding
MSTRG.194055.1		XLOC_198855	316 coding	noncoding	noncoding	noncoding
NM_030665.4	RAI1	XLOC_198856	7677 coding	coding	coding	coding
MSTRG.194088.1	RAI1	XLOC_198860	236 coding	noncoding	coding	noncoding
MSTRG.194146.1	GID4	XLOC_198888	222 coding	noncoding	noncoding	noncoding
MSTRG.194106.1		XLOC_198889	292 coding	noncoding	noncoding	noncoding
MSTRG.194107.1		XLOC_198890	259 coding	noncoding	noncoding	noncoding
XM_017024715.2	MYO15A	XLOC_198892	12668 coding	coding	noncoding	coding
MSTRG.194196.1	TRIM16L	XLOC_198917	277 coding	noncoding	noncoding	noncoding
NR_051988.1	FBXW10	XLOC_198919	3484 coding	coding	coding	coding
MSTRG.194201.1		XLOC_198925	246 coding	noncoding	noncoding	noncoding
NM_001353100.2	PRPSAP2	XLOC_198926	2041 coding	coding	coding	coding
MSTRG.194238.1	LOC105371574	XLOC_198943	303 coding	noncoding	noncoding	noncoding
MSTRG.194243.1		XLOC_198946	487 coding	noncoding	noncoding	noncoding
MSTRG.194246.1		XLOC_198951	267 coding	noncoding	noncoding	noncoding
MSTRG.194280.1	ALDH3A2	XLOC_198953	301 coding	noncoding	noncoding	noncoding
MSTRG.194293.6	AKAP10	XLOC_198963	3612 coding	coding	noncoding	noncoding
MSTRG.194510.1	SPECC1	XLOC_198968	210 coding	noncoding	noncoding	noncoding

MSTRG.194515.1	SPECC1	XLOC_198972	215 coding	noncoding	noncoding	noncoding
MSTRG.194550.1	LOC100996294	XLOC_198986	296 coding	noncoding	noncoding	noncoding
MSTRG.194558.1	LINC02088	XLOC_198990	212 coding	noncoding	noncoding	noncoding
MSTRG.194321.1	CCDC144NL	XLOC_198996	240 coding	noncoding	noncoding	noncoding
MSTRG.194324.2	USP22	XLOC_198998	4857 coding	coding	coding	noncoding
MSTRG.194338.1		XLOC_199014	242 coding	noncoding	noncoding	noncoding
MSTRG.194374.1		XLOC_199030	325 coding	noncoding	noncoding	noncoding
MSTRG.194406.1		XLOC_199040	291 coding	noncoding	noncoding	noncoding
MSTRG.194413.1		XLOC_199045	270 coding	noncoding	noncoding	noncoding
MSTRG.194476.1		XLOC_199087	264 coding	noncoding	noncoding	noncoding
XM_017025279.1	KSR1	XLOC_199101	2309 coding	coding	coding	coding
XM_017025271.1	KSR1	XLOC_199101	6710 coding	coding	coding	coding
MSTRG.194573.1	NOS2	XLOC_199111	225 coding	coding	noncoding	noncoding
MSTRG.194579.1		XLOC_199117	314 coding	noncoding	noncoding	noncoding
MSTRG.194640.1		XLOC_199128	239 coding	noncoding	noncoding	noncoding
MSTRG.194613.1		XLOC_199165	318 coding	noncoding	noncoding	noncoding
XM_017025228.1	FOXN1	XLOC_199172	3653 coding	coding	coding	coding
MSTRG.194629.1	FOXN1	XLOC_199175	300 coding	noncoding	noncoding	noncoding
XR_001752596.1	SUPT6H	XLOC_199188	4590 coding	coding	coding	coding
MSTRG.194727.1	FAM222B	XLOC_199217	256 coding	noncoding	noncoding	noncoding
MSTRG.194790.1		XLOC_199241	212 coding	noncoding	noncoding	noncoding
MSTRG.194788.1		XLOC_199244	345 coding	noncoding	noncoding	noncoding
MSTRG.194787.1		XLOC_199245	283 coding	noncoding	noncoding	noncoding
MSTRG.194798.1		XLOC_199254	274 coding	noncoding	noncoding	noncoding
MSTRG.194846.1	TAOK1	XLOC_199262	232 coding	noncoding	noncoding	noncoding
MSTRG.194847.1	TAOK1	XLOC_199263	227 coding	noncoding	noncoding	noncoding
MSTRG.194990.1	SSH2	XLOC_199293	303 coding	noncoding	noncoding	noncoding
MSTRG.194878.1	NSRP1	XLOC_199345	291 coding	noncoding	noncoding	noncoding
MSTRG.194895.1	CPD	XLOC_199370	229 coding	noncoding	noncoding	noncoding
MSTRG.194902.1	GOSR1	XLOC_199376	269 coding	noncoding	noncoding	noncoding
MSTRG.194904.1	GOSR1	XLOC_199378	316 coding	coding	noncoding	noncoding
MSTRG.194905.1	GOSR1	XLOC_199379	270 coding	noncoding	noncoding	noncoding
NM_024857.5	ATAD5	XLOC_199392	6873 coding	coding	coding	coding
XM_024450832.1	ADAP2	XLOC_199403	2763 coding	coding	coding	coding
XR_002957953.1	LOC107984974	XLOC_199408	2806 coding	coding	coding	noncoding
MSTRG.195155.1	LOC646030	XLOC_199409	312 coding	coding	noncoding	noncoding
MSTRG.195173.1	NF1	XLOC_199412	276 coding	noncoding	noncoding	noncoding
MSTRG.195089.1	RAB11FIP4	XLOC_199430	238 coding	noncoding	noncoding	noncoding
MSTRG.195091.1	RAB11FIP4	XLOC_199431	209 coding	noncoding	noncoding	noncoding
MSTRG.195063.1		XLOC_199434	325 coding	noncoding	noncoding	noncoding
MSTRG.195075.1		XLOC_199443	403 coding	noncoding	noncoding	noncoding
NM_052888.2	LRRC37B	XLOC_199455	3027 coding	coding	coding	coding
MSTRG.195207.1	RHOT1	XLOC_199465	364 coding	noncoding	noncoding	noncoding

MSTRG.195198.1		XLOC_199467	200	coding	noncoding	noncoding	noncoding
NM_003457.3	ZNF207	XLOC_199473	2275	coding	noncoding	coding	coding
XR_001752613.2	ZNF207	XLOC_199473	4396	coding	noncoding	coding	coding
MSTRG.195215.1		XLOC_199480	263	coding	noncoding	noncoding	noncoding
MSTRG.195221.1	PSMD11	XLOC_199483	329	coding	noncoding	noncoding	noncoding
MSTRG.195396.1	MYO1D	XLOC_199510	272	coding	noncoding	noncoding	noncoding
MSTRG.195240.1		XLOC_199525	258	coding	noncoding	noncoding	noncoding
MSTRG.195299.1	ASIC2	XLOC_199529	410	coding	noncoding	noncoding	noncoding
MSTRG.195304.1	ASIC2	XLOC_199531	303	coding	noncoding	noncoding	noncoding
MSTRG.195319.1	ASIC2	XLOC_199537	263	coding	coding	noncoding	noncoding
MSTRG.195329.1	ASIC2	XLOC_199543	568	coding	noncoding	noncoding	coding
MSTRG.195335.1	ASIC2	XLOC_199547	240	coding	noncoding	noncoding	noncoding
MSTRG.195337.1	ASIC2	XLOC_199548	302	coding	coding	noncoding	noncoding
MSTRG.195342.1	ASIC2	XLOC_199550	264	coding	noncoding	noncoding	noncoding
MSTRG.195344.1	ASIC2	XLOC_199552	258	coding	noncoding	noncoding	noncoding
MSTRG.195244.1	LINC01989	XLOC_199560	295	coding	coding	noncoding	noncoding
MSTRG.195242.1		XLOC_199561	246	coding	noncoding	noncoding	noncoding
MSTRG.195426.1		XLOC_199630	261	coding	noncoding	noncoding	noncoding
MSTRG.195505.12	SLFN12L	XLOC_199644	2644	coding	noncoding	noncoding	noncoding
MSTRG.195527.1	LOC107985033	XLOC_199647	8093	coding	coding	coding	noncoding
MSTRG.195527.3	LOC107985033	XLOC_199647	7086	coding	coding	coding	noncoding
XM_011524448.2	AP2B1	XLOC_199651	5835	coding	coding	coding	coding
XM_005257937.4	AP2B1	XLOC_199651	3976	coding	coding	coding	coding
XM_005276914.3	TBC1D3G	XLOC_199681	3890	coding	coding	coding	coding
XM_006722044.3	TADA2A	XLOC_199709	4365	coding	coding	coding	coding
XM_006722248.2	TBC1D3D	XLOC_199720	3571	coding	coding	coding	coding
XM_006722254.2	TBC1D3E	XLOC_199722	2446	coding	coding	coding	coding
XM_011525074.2	ARHGAP23	XLOC_199727	5962	coding	coding	coding	coding
MSTRG.195685.1	RPL23	XLOC_199735	497	coding	coding	noncoding	noncoding
MSTRG.195685.2	RPL23	XLOC_199735	508	coding	coding	noncoding	noncoding
MSTRG.195671.1		XLOC_199739	287	coding	noncoding	noncoding	noncoding
MSTRG.195675.1	LOC105371767	XLOC_199747	313	coding	noncoding	noncoding	noncoding
MSTRG.195721.1		XLOC_199767	209	coding	noncoding	noncoding	noncoding
XM_024450801.1	CDK12	XLOC_199768	7217	coding	coding	coding	coding
MSTRG.195749.1	CDK12	XLOC_199769	234	coding	noncoding	noncoding	noncoding
MSTRG.195734.1		XLOC_199773	221	coding	noncoding	noncoding	noncoding
MSTRG.195777.9	IKZF3	XLOC_199786	2175	coding	noncoding	coding	coding
MSTRG.195773.7	LRRC3C	XLOC_199792	2554	coding	coding	coding	noncoding
MSTRG.195811.1		XLOC_199810	234	coding	noncoding	noncoding	noncoding
MSTRG.195831.1	WIPF2	XLOC_199812	246	coding	noncoding	noncoding	noncoding
NM_001254.4	CDC6	XLOC_199813	4564	coding	coding	coding	coding
MSTRG.195855.1	LOC105371773	XLOC_199825	216	coding	noncoding	noncoding	noncoding
MSTRG.195869.2	CCR7	XLOC_199828	5320	coding	coding	coding	coding

MSTRG.195866.1		XLOC_199831	238 coding	noncoding	noncoding	noncoding
MSTRG.195917.1	KRT32	XLOC_199858	296 coding	noncoding	noncoding	noncoding
MSTRG.195973.6	ACLY	XLOC_199878	3990 coding	coding	coding	coding
MSTRG.196017.1	STAT5B	XLOC_199895	738 coding	noncoding	noncoding	noncoding
MSTRG.195989.1		XLOC_199899	226 coding	noncoding	noncoding	noncoding
XR_002958025.1	ATP6V0A1	XLOC_199903	4423 coding	coding	coding	coding
MSTRG.196008.1		XLOC_199915	318 coding	noncoding	noncoding	noncoding
MSTRG.196009.1		XLOC_199916	275 coding	coding	coding	noncoding
MSTRG.196043.1	VPS25	XLOC_199925	340 coding	noncoding	noncoding	noncoding
MSTRG.196075.1	LINC00671	XLOC_199935	254 coding	noncoding	noncoding	noncoding
MSTRG.196092.1	IFI35	XLOC_199943	301 coding	noncoding	noncoding	noncoding
XM_011524813.2	NBR1	XLOC_199952	4946 coding	coding	coding	coding
MSTRG.196095.1		XLOC_199961	289 coding	noncoding	noncoding	noncoding
NM_001322216.1	DHX8	XLOC_199986	4392 coding	coding	coding	coding
MSTRG.196177.1	DHX8	XLOC_199987	357 coding	noncoding	noncoding	noncoding
MSTRG.196130.1		XLOC_199995	409 coding	noncoding	noncoding	noncoding
MSTRG.196140.1	MEOX1	XLOC_199997	303 coding	noncoding	noncoding	noncoding
XR_934769.3	LINC02594	XLOC_200000	4527 coding	noncoding	noncoding	noncoding
XM_006721721.3	CD300LG	XLOC_200008	2967 coding	coding	coding	coding
MSTRG.196203.1	PYY	XLOC_200014	212 coding	noncoding	noncoding	noncoding
MSTRG.196228.3	HDAC5	XLOC_200027	2830 coding	coding	coding	coding
NM_024032.5	C17orf53	XLOC_200030	2701 coding	coding	coding	coding
NM_001353191.2	TMUB2	XLOC_200036	2451 coding	coding	coding	coding
XM_017025054.1	TMUB2	XLOC_200036	2302 coding	coding	coding	coding
MSTRG.196260.4	UBTF	XLOC_200040	1091 coding	noncoding	noncoding	noncoding
MSTRG.196260.5	UBTF	XLOC_200040	1092 coding	noncoding	noncoding	noncoding
MSTRG.196260.3	UBTF	XLOC_200040	1090 coding	noncoding	noncoding	noncoding
MSTRG.196236.1		XLOC_200042	319 coding	noncoding	noncoding	noncoding
XR_001752408.2	RUNDC3A	XLOC_200044	2564 coding	coding	coding	coding
MSTRG.196247.1	FAM171A2	XLOC_200047	332 coding	coding	coding	noncoding
MSTRG.196269.1		XLOC_200054	445 coding	noncoding	noncoding	noncoding
MSTRG.196275.1		XLOC_200057	290 coding	noncoding	noncoding	noncoding
XM_017025152.1	DBF4B	XLOC_200059	6223 coding	coding	coding	coding
MSTRG.196297.1		XLOC_200062	260 coding	noncoding	noncoding	noncoding
XR_002958013.1	NMT1	XLOC_200071	5860 coding	coding	coding	coding
MSTRG.196370.1		XLOC_200083	215 coding	noncoding	noncoding	noncoding
MSTRG.196405.6	CDC27	XLOC_200138	5393 coding	coding	noncoding	noncoding
MSTRG.196405.11	CDC27	XLOC_200138	3887 coding	coding	noncoding	noncoding
MSTRG.196410.1		XLOC_200139	277 coding	noncoding	noncoding	noncoding
MSTRG.196537.1		XLOC_200176	225 coding	noncoding	noncoding	noncoding
MSTRG.196550.1	COPZ2	XLOC_200182	303 coding	noncoding	noncoding	noncoding
MSTRG.196570.3	HOXB2	XLOC_200197	1057 coding	coding	coding	noncoding
MSTRG.196595.1	LINC02086	XLOC_200213	318 coding	noncoding	noncoding	noncoding

MSTRG.196600.1	TTLL6	XLOC_200217	308 coding	noncoding	noncoding	noncoding
MSTRG.196601.1	TTLL6	XLOC_200218	252 coding	noncoding	noncoding	noncoding
MSTRG.196602.1	TTLL6	XLOC_200219	360 coding	coding	noncoding	noncoding
MSTRG.196607.1		XLOC_200221	254 coding	noncoding	noncoding	noncoding
MSTRG.196612.1		XLOC_200231	294 coding	noncoding	noncoding	noncoding
MSTRG.196618.1		XLOC_200234	287 coding	noncoding	noncoding	noncoding
MSTRG.196652.4	ZNF652	XLOC_200247	6548 coding	noncoding	noncoding	noncoding
MSTRG.196670.1	LOC100288866	XLOC_200255	274 coding	coding	noncoding	noncoding
MSTRG.196674.1		XLOC_200261	337 coding	noncoding	noncoding	noncoding
MSTRG.196783.1		XLOC_200274	261 coding	noncoding	noncoding	noncoding
MSTRG.196832.1		XLOC_200297	332 coding	noncoding	noncoding	noncoding
MSTRG.196838.1		XLOC_200298	262 coding	noncoding	noncoding	noncoding
MSTRG.196839.1		XLOC_200299	230 coding	noncoding	noncoding	noncoding
MSTRG.196885.1		XLOC_200316	284 coding	noncoding	noncoding	noncoding
MSTRG.196914.1		XLOC_200331	243 coding	noncoding	noncoding	noncoding
MSTRG.196956.1		XLOC_200338	335 coding	noncoding	noncoding	noncoding
MSTRG.196973.1	CA10	XLOC_200341	240 coding	noncoding	noncoding	noncoding
MSTRG.196976.1	CA10	XLOC_200343	266 coding	noncoding	noncoding	noncoding
MSTRG.196993.1	CA10	XLOC_200348	224 coding	noncoding	noncoding	noncoding
MSTRG.196964.1		XLOC_200351	297 coding	noncoding	noncoding	noncoding
MSTRG.196967.1		XLOC_200353	313 coding	noncoding	noncoding	noncoding
MSTRG.197009.1		XLOC_200368	243 coding	noncoding	noncoding	noncoding
MSTRG.197027.1		XLOC_200375	237 coding	noncoding	noncoding	noncoding
MSTRG.197042.1		XLOC_200386	307 coding	noncoding	noncoding	noncoding
MSTRG.197043.1		XLOC_200387	227 coding	noncoding	noncoding	noncoding
MSTRG.197044.1		XLOC_200388	312 coding	noncoding	noncoding	noncoding
MSTRG.197045.1		XLOC_200389	324 coding	noncoding	noncoding	noncoding
MSTRG.197046.1		XLOC_200390	303 coding	noncoding	noncoding	noncoding
MSTRG.197063.1		XLOC_200399	311 coding	noncoding	noncoding	noncoding
MSTRG.197064.1		XLOC_200400	326 coding	noncoding	noncoding	noncoding
MSTRG.197065.1		XLOC_200401	292 coding	noncoding	noncoding	noncoding
MSTRG.197066.1		XLOC_200402	288 coding	noncoding	noncoding	noncoding
MSTRG.197085.1		XLOC_200413	330 coding	noncoding	noncoding	noncoding
XR_002957996.1	HLF	XLOC_200424	4666 coding	noncoding	coding	coding
MSTRG.197107.1	HLF	XLOC_200427	312 coding	noncoding	noncoding	noncoding
MSTRG.197140.1		XLOC_200446	262 coding	noncoding	noncoding	noncoding
MSTRG.197144.1		XLOC_200449	281 coding	noncoding	noncoding	noncoding
MSTRG.197264.1	DGKE	XLOC_200486	266 coding	noncoding	coding	noncoding
MSTRG.197281.1		XLOC_200502	292 coding	noncoding	noncoding	noncoding
MSTRG.197397.1	MSI2	XLOC_200515	290 coding	noncoding	noncoding	noncoding
MSTRG.197355.4	VEZF1	XLOC_200540	5712 coding	coding	coding	coding
MSTRG.197417.1	TSPOAP1	XLOC_200564	5115 coding	coding	coding	coding
MSTRG.197502.1	TEX14	XLOC_200597	292 coding	noncoding	noncoding	noncoding

MSTRG.197508.1	RAD51C	XLOC_200601	278 coding	noncoding	noncoding	noncoding
MSTRG.197511.1	LOC105371843	XLOC_200603	265 coding	noncoding	noncoding	noncoding
MSTRG.197520.1	PPM1E	XLOC_200609	228 coding	noncoding	noncoding	noncoding
MSTRG.197521.1	PPM1E	XLOC_200610	249 coding	noncoding	noncoding	noncoding
MSTRG.197522.1	PPM1E	XLOC_200611	257 coding	noncoding	noncoding	noncoding
MSTRG.197526.1	TRIM37	XLOC_200613	229 coding	noncoding	noncoding	noncoding
MSTRG.197527.3	TRIM37	XLOC_200614	3311 coding	coding	noncoding	noncoding
MSTRG.197470.1		XLOC_200631	284 coding	noncoding	noncoding	noncoding
MSTRG.197482.1	LINC01476	XLOC_200632	286 coding	noncoding	coding	noncoding
MSTRG.197483.1	LINC01476	XLOC_200633	294 coding	noncoding	noncoding	noncoding
MSTRG.197487.1	LINC01476	XLOC_200634	214 coding	noncoding	noncoding	noncoding
NM_001329397.2	VMP1	XLOC_200640	3518 coding	coding	coding	coding
MSTRG.197782.1	WFDC21P	XLOC_200657	295 coding	coding	noncoding	noncoding
MSTRG.197825.1	USP32	XLOC_200664	251 coding	noncoding	noncoding	noncoding
XM_005257036.2	C17orf64	XLOC_200665	999 coding	coding	coding	coding
MSTRG.197535.1		XLOC_200666	203 coding	noncoding	noncoding	noncoding
MSTRG.197541.3	APPBP2	XLOC_200667	4148 coding	coding	noncoding	noncoding
MSTRG.197536.1		XLOC_200669	296 coding	noncoding	noncoding	noncoding
MSTRG.197554.1	PPM1D	XLOC_200676	278 coding	noncoding	noncoding	noncoding
XR_001752547.2	BCAS3	XLOC_200678	2455 coding	coding	coding	coding
MSTRG.197730.1	BCAS3	XLOC_200723	472 coding	noncoding	noncoding	noncoding
MSTRG.197597.7	MED13	XLOC_200747	10316 coding	coding	noncoding	noncoding
MSTRG.197597.8	MED13	XLOC_200747	11015 coding	coding	coding	noncoding
MSTRG.197584.1		XLOC_200751	316 coding	noncoding	noncoding	noncoding
MSTRG.197595.1	EFCAB3	XLOC_200757	261 coding	noncoding	noncoding	noncoding
MSTRG.197616.1	METTL2A	XLOC_200760	272 coding	noncoding	noncoding	noncoding
MSTRG.197617.1	METTL2A	XLOC_200761	656 coding	noncoding	noncoding	noncoding
XR_002958144.1	LOC112268202	XLOC_200773	2663 coding	noncoding	coding	noncoding
MSTRG.197860.1	LOC105371857	XLOC_200800	321 coding	noncoding	noncoding	noncoding
MSTRG.197663.1		XLOC_200809	301 coding	noncoding	noncoding	noncoding
NR_073585.1	DCAF7	XLOC_200812	1414 coding	noncoding	coding	coding
MSTRG.197893.6	DDX42	XLOC_200823	3620 coding	coding	coding	coding
NM_001199163.1	PSMC5	XLOC_200827	1495 coding	coding	coding	coding
MSTRG.197872.1		XLOC_200831	299 coding	noncoding	noncoding	noncoding
MSTRG.197926.1		XLOC_200849	249 coding	noncoding	noncoding	noncoding
MSTRG.197929.1		XLOC_200857	234 coding	noncoding	noncoding	noncoding
MSTRG.197931.1		XLOC_200859	363 coding	noncoding	noncoding	noncoding
MSTRG.197961.13	PLEKHM1P1	XLOC_200863	701 coding	coding	noncoding	noncoding
MSTRG.197967.1	PLEKHM1P1	XLOC_200864	539 coding	noncoding	noncoding	noncoding
MSTRG.197984.1		XLOC_200889	349 coding	noncoding	noncoding	noncoding
MSTRG.198073.1	CEP112	XLOC_200919	294 coding	noncoding	noncoding	noncoding
MSTRG.198044.1	APOH	XLOC_200926	260 coding	noncoding	noncoding	noncoding
MSTRG.198045.1	APOH	XLOC_200927	336 coding	coding	noncoding	noncoding

MSTRG.198143.1	PRKCA	XLOC_200940	377	coding	noncoding	noncoding	noncoding
MSTRG.198168.1	PRKCA	XLOC_200962	225	coding	noncoding	noncoding	noncoding
MSTRG.198171.1	PRKCA	XLOC_200965	248	coding	noncoding	noncoding	noncoding
MSTRG.198100.1		XLOC_200983	248	coding	noncoding	noncoding	noncoding
MSTRG.198187.1	PITPNC1	XLOC_200990	261	coding	coding	noncoding	noncoding
MSTRG.198201.1	PITPNC1	XLOC_201002	231	coding	noncoding	noncoding	noncoding
MSTRG.198128.1		XLOC_201016	240	coding	noncoding	noncoding	noncoding
NM_004459.7	BPTF	XLOC_201018	11024	coding	coding	noncoding	coding
MSTRG.198238.1	BPTF	XLOC_201022	295	coding	noncoding	noncoding	noncoding
MSTRG.198239.1	BPTF	XLOC_201023	476	coding	noncoding	noncoding	noncoding
MSTRG.198259.1	LOC105371870	XLOC_201039	245	coding	noncoding	noncoding	noncoding
NR_144443.1	AMZ2	XLOC_201041	2199	coding	coding	coding	coding
MSTRG.198267.1	AMZ2	XLOC_201044	257	coding	noncoding	noncoding	noncoding
MSTRG.198283.7	SLC16A6	XLOC_201047	2286	coding	coding	coding	coding
XM_011525026.2	MAP2K6	XLOC_201097	13206	coding	coding	coding	coding
MSTRG.198381.1	MAP2K6	XLOC_201102	301	coding	noncoding	noncoding	noncoding
MSTRG.198404.1	LINC01483	XLOC_201113	225	coding	coding	noncoding	noncoding
MSTRG.198310.1		XLOC_201117	218	coding	noncoding	noncoding	noncoding
MSTRG.198430.1		XLOC_201138	279	coding	noncoding	noncoding	noncoding
MSTRG.198435.1		XLOC_201141	243	coding	coding	noncoding	noncoding
NR_110124.1	LINC01152	XLOC_201159	3578	coding	noncoding	noncoding	noncoding
MSTRG.198487.1		XLOC_201167	654	coding	noncoding	noncoding	noncoding
MSTRG.198505.1	LINC00673	XLOC_201180	322	coding	noncoding	noncoding	noncoding
MSTRG.198510.1	LINC00673	XLOC_201183	256	coding	noncoding	noncoding	noncoding
MSTRG.198517.1		XLOC_201204	303	coding	noncoding	noncoding	noncoding
MSTRG.198524.1	CDC42EP4	XLOC_201210	254	coding	noncoding	noncoding	noncoding
MSTRG.198560.1	LOC100134391	XLOC_201229	305	coding	noncoding	noncoding	noncoding
MSTRG.198565.1	LINC00469	XLOC_201232	461	coding	noncoding	noncoding	noncoding
MSTRG.198575.1		XLOC_201242	312	coding	noncoding	noncoding	noncoding
MSTRG.198576.1		XLOC_201243	296	coding	noncoding	noncoding	noncoding
MSTRG.198585.1		XLOC_201252	221	coding	noncoding	noncoding	noncoding
XM_024450879.1	DNAI2	XLOC_201253	2647	coding	noncoding	coding	coding
MSTRG.198594.1	DNAI2	XLOC_201254	291	coding	noncoding	noncoding	noncoding
XM_017024150.1	KIF19	XLOC_201255	3685	coding	coding	coding	coding
MSTRG.198604.1	KIF19	XLOC_201256	244	coding	noncoding	noncoding	noncoding
NM_001331076.1	GPR142	XLOC_201258	1487	coding	coding	coding	coding
MSTRG.198598.1		XLOC_201259	230	coding	noncoding	noncoding	noncoding
NM_001330456.1	CD300A	XLOC_201265	1815	coding	coding	coding	coding
XM_005256990.3	CD300A	XLOC_201265	1521	coding	coding	coding	coding
XM_006721656.3	CD300A	XLOC_201265	1928	coding	coding	coding	coding
MSTRG.198680.1	LOC101928343	XLOC_201278	264	coding	noncoding	noncoding	noncoding
MSTRG.198723.1	RAB37	XLOC_201283	423	coding	noncoding	noncoding	noncoding
MSTRG.198687.1	FADS6	XLOC_201295	252	coding	noncoding	noncoding	noncoding

MSTRG.198741.1	SLC16A5	XLOC_201309	318 coding	noncoding	noncoding	noncoding
MSTRG.198711.1		XLOC_201313	415 coding	noncoding	noncoding	noncoding
MSTRG.198774.3	SLC25A19	XLOC_201318	884 coding	coding	coding	noncoding
MSTRG.198753.1		XLOC_201329	272 coding	noncoding	noncoding	noncoding
MSTRG.198800.1	LLGL2	XLOC_201335	294 coding	noncoding	noncoding	noncoding
NR_028439.1	SMIM5	XLOC_201340	4350 coding	coding	coding	noncoding
MSTRG.198817.1	UNK	XLOC_201350	491 coding	noncoding	noncoding	noncoding
MSTRG.198848.4	ACOX1	XLOC_201360	4441 coding	coding	coding	coding
MSTRG.198880.1		XLOC_201376	234 coding	noncoding	noncoding	noncoding
MSTRG.198883.1		XLOC_201377	212 coding	noncoding	noncoding	noncoding
MSTRG.198904.9	RHBDF2	XLOC_201384	4878 coding	noncoding	coding	noncoding
MSTRG.198896.1		XLOC_201388	220 coding	noncoding	noncoding	noncoding
MSTRG.198925.1		XLOC_201389	231 coding	noncoding	noncoding	noncoding
MSTRG.198928.1	CYGB	XLOC_201391	319 coding	noncoding	noncoding	noncoding
MSTRG.198935.1	SNHG16	XLOC_201396	4731 coding	coding	coding	noncoding
MSTRG.198914.1	ST6GALNAC1	XLOC_201400	286 coding	noncoding	noncoding	noncoding
XM_011524352.2	MGAT5B	XLOC_201417	3550 coding	coding	coding	coding
MSTRG.198952.1		XLOC_201421	306 coding	noncoding	noncoding	noncoding
NM_001204408.2	SEC14L1	XLOC_201422	3320 coding	coding	coding	coding
MSTRG.199044.1	SEPTIN9	XLOC_201434	323 coding	noncoding	noncoding	noncoding
MSTRG.199072.1	SEPTIN9	XLOC_201456	274 coding	noncoding	noncoding	noncoding
MSTRG.198978.1		XLOC_201460	268 coding	noncoding	noncoding	noncoding
XR_001753032.1	LOC107985079	XLOC_201462	3665 coding	noncoding	coding	noncoding
MSTRG.198988.1		XLOC_201466	294 coding	noncoding	noncoding	noncoding
MSTRG.198997.1	LOC105371908	XLOC_201469	319 coding	noncoding	noncoding	noncoding
MSTRG.199102.1		XLOC_201475	256 coding	noncoding	noncoding	noncoding
MSTRG.199105.12	TMC6	XLOC_201478	3879 coding	coding	coding	coding
XM_017024001.1	C17orf99	XLOC_201492	1343 coding	coding	coding	coding
MSTRG.199088.1	C17orf99	XLOC_201493	431 coding	noncoding	noncoding	noncoding
MSTRG.199095.1	AFMID	XLOC_201496	306 coding	noncoding	noncoding	noncoding
MSTRG.199084.1		XLOC_201503	241 coding	noncoding	noncoding	noncoding
MSTRG.199147.1	SCAT1	XLOC_201520	286 coding	noncoding	noncoding	noncoding
MSTRG.199096.1		XLOC_201521	251 coding	noncoding	noncoding	noncoding
MSTRG.199236.14	CYTH1	XLOC_201525	3921 coding	coding	coding	noncoding
MSTRG.199157.1	CANT1	XLOC_201534	3628 coding	coding	coding	coding
MSTRG.199209.1	RBFOX3	XLOC_201554	279 coding	noncoding	noncoding	noncoding
MSTRG.199213.1		XLOC_201571	327 coding	noncoding	noncoding	noncoding
XM_024450821.1	CCDC40	XLOC_201577	4851 coding	coding	coding	coding
MSTRG.199283.1	CCDC40	XLOC_201579	558 coding	noncoding	noncoding	noncoding
MSTRG.199331.1	RNF213	XLOC_201589	242 coding	coding	noncoding	noncoding
NM_001352760.2	ENDOV	XLOC_201592	1406 coding	coding	coding	coding
MSTRG.199434.1	RPTOR	XLOC_201614	246 coding	noncoding	noncoding	noncoding
XM_017024898.2	BAHCC1	XLOC_201656	10816 coding	coding	noncoding	coding

MSTRG.199408.1	FSCN2	XLOC_201661	256	coding	noncoding	noncoding	noncoding
MSTRG.199455.5	NPLOC4	XLOC_201666	648	coding	coding	noncoding	noncoding
NR_110866.1	TSPAN10	XLOC_201668	1905	coding	coding	coding	coding
MSTRG.199470.1	PDE6G	XLOC_201671	313	coding	noncoding	noncoding	noncoding
MSTRG.199471.3	OXLD1	XLOC_201672	869	coding	coding	coding	noncoding
MSTRG.199491.1	MYADML2	XLOC_201702	216	coding	noncoding	noncoding	noncoding
XM_017025038.2	ASPSCR1	XLOC_201703	2212	coding	coding	coding	coding
MSTRG.199526.1	FASN	XLOC_201714	323	coding	noncoding	noncoding	noncoding
MSTRG.199528.1	FASN	XLOC_201716	1219	coding	coding	coding	noncoding
MSTRG.199534.1		XLOC_201740	273	coding	noncoding	noncoding	noncoding
NM_018949.2	UTS2R	XLOC_201747	1310	coding	coding	coding	coding
MSTRG.199598.1	LOC105371942	XLOC_201760	253	coding	noncoding	coding	noncoding
MSTRG.199608.1	RAB40B	XLOC_201768	405	coding	noncoding	noncoding	noncoding
XM_017024523.1	METRNL	XLOC_201775	1957	coding	noncoding	coding	coding
MSTRG.192245.1		XLOC_201812	210	coding	noncoding	noncoding	noncoding
MSTRG.192247.1		XLOC_201813	253	coding	noncoding	noncoding	noncoding
XM_024450768.1	MYO1C	XLOC_201817	5034	coding	coding	coding	coding
MSTRG.192302.1		XLOC_201833	264	coding	noncoding	noncoding	noncoding
NM_178568.4	RTN4RL1	XLOC_201834	3614	coding	coding	coding	coding
MSTRG.192575.1	RTN4RL1	XLOC_201835	284	coding	noncoding	noncoding	noncoding
XM_005256571.5	SMG6	XLOC_201843	4547	coding	coding	coding	coding
MSTRG.192664.1	SMG6	XLOC_201846	313	coding	coding	noncoding	noncoding
MSTRG.192665.1	SMG6	XLOC_201847	279	coding	noncoding	noncoding	noncoding
MSTRG.192667.1	SMG6	XLOC_201849	255	coding	noncoding	noncoding	noncoding
MSTRG.192668.1	SMG6	XLOC_201850	285	coding	noncoding	noncoding	noncoding
MSTRG.192670.1	SMG6	XLOC_201851	334	coding	noncoding	noncoding	noncoding
XM_017024654.2	MNT	XLOC_201862	7273	coding	noncoding	coding	coding
MSTRG.192689.1	RAP1GAP2	XLOC_201877	878	coding	noncoding	coding	noncoding
MSTRG.192695.1	RAP1GAP2	XLOC_201880	2603	coding	noncoding	coding	noncoding
MSTRG.192647.1	OR3A2	XLOC_201892	255	coding	noncoding	noncoding	noncoding
MSTRG.192642.1		XLOC_201897	210	coding	coding	noncoding	noncoding
MSTRG.192658.1	TRPV3	XLOC_201901	207	coding	noncoding	noncoding	noncoding
NM_080706.3	TRPV1	XLOC_201902	4416	coding	coding	coding	coding
XM_024450740.1	ITGAE	XLOC_201909	5744	coding	coding	coding	coding
NM_174956.2	ATP2A3	XLOC_201920	4827	coding	coding	coding	coding
XM_011523882.2	ATP2A3	XLOC_201920	3901	coding	coding	coding	coding
XR_934043.2	ANKFY1	XLOC_201931	2542	coding	coding	coding	coding
MSTRG.192785.1	ANKFY1	XLOC_201933	236	coding	noncoding	noncoding	noncoding
MSTRG.192758.1	UBE2G1	XLOC_201938	248	coding	noncoding	noncoding	noncoding
MSTRG.192762.1	UBE2G1	XLOC_201942	206	coding	noncoding	noncoding	noncoding
MSTRG.192755.1		XLOC_201945	657	coding	noncoding	noncoding	noncoding
MSTRG.192754.1		XLOC_201946	282	coding	noncoding	noncoding	noncoding
MSTRG.192793.1	SPNS2	XLOC_201954	315	coding	noncoding	noncoding	noncoding

MSTRG.192805.1	LINC01996	XLOC_201959	220 coding	noncoding	noncoding	noncoding
MSTRG.192807.1	LINC01996	XLOC_201960	216 coding	noncoding	noncoding	noncoding
MSTRG.192814.1	ALOX15	XLOC_201962	280 coding	noncoding	noncoding	noncoding
MSTRG.192802.1	LOC105371499	XLOC_201965	234 coding	noncoding	noncoding	noncoding
MSTRG.192823.1	PELP1	XLOC_201967	245 coding	noncoding	noncoding	noncoding
MSTRG.192803.1		XLOC_201969	245 coding	noncoding	noncoding	noncoding
MSTRG.192841.1		XLOC_201983	252 coding	noncoding	noncoding	noncoding
MSTRG.192848.1		XLOC_201987	242 coding	noncoding	noncoding	noncoding
MSTRG.192859.1	PFN1	XLOC_201988	791 coding	coding	coding	coding
XM_024450919.1	ZNF232	XLOC_202003	2473 coding	coding	coding	coding
MSTRG.192964.1	LOC100130950	XLOC_202007	276 coding	noncoding	noncoding	noncoding
MSTRG.192887.1		XLOC_202017	273 coding	noncoding	noncoding	noncoding
MSTRG.192910.1	LOC339166	XLOC_202040	244 coding	noncoding	noncoding	noncoding
MSTRG.192907.1		XLOC_202043	336 coding	noncoding	noncoding	noncoding
MSTRG.192929.1		XLOC_202053	286 coding	noncoding	noncoding	noncoding
MSTRG.192933.1		XLOC_202056	215 coding	noncoding	noncoding	noncoding
XM_011524016.3	PITPNM3	XLOC_202060	4473 coding	coding	coding	coding
MSTRG.192996.1		XLOC_202086	277 coding	noncoding	noncoding	noncoding
MSTRG.193080.1		XLOC_202114	239 coding	noncoding	noncoding	noncoding
MSTRG.193101.1	ASGR2	XLOC_202120	309 coding	noncoding	noncoding	noncoding
MSTRG.193103.1	LOC105371512	XLOC_202121	273 coding	noncoding	noncoding	noncoding
NM_001321075.2	DLG4	XLOC_202124	6183 coding	coding	coding	coding
XM_011523972.2	ZBTB4	XLOC_202141	6225 coding	coding	coding	coding
MSTRG.193147.4	POLR2A	XLOC_202142	2237 coding	coding	coding	coding
MSTRG.193142.1		XLOC_202143	237 coding	noncoding	noncoding	noncoding
MSTRG.193168.1	SHBG	XLOC_202151	289 coding	noncoding	noncoding	noncoding
XM_017024073.1	SAT2	XLOC_202152	1030 coding	coding	coding	coding
MSTRG.193241.1	DNAH2	XLOC_202162	214 coding	noncoding	noncoding	noncoding
MSTRG.193242.1	DNAH2	XLOC_202163	273 coding	noncoding	noncoding	noncoding
MSTRG.193179.1		XLOC_202182	251 coding	noncoding	noncoding	noncoding
MSTRG.193185.1	GUCY2D	XLOC_202184	284 coding	coding	noncoding	noncoding
NM_002616.3	PER1	XLOC_202194	4676 coding	coding	coding	coding
XR_001752643.1	CTC1	XLOC_202208	3817 coding	coding	coding	coding
MSTRG.193298.1	CTC1	XLOC_202210	352 coding	noncoding	noncoding	noncoding
MSTRG.193269.4	SLC25A35	XLOC_202216	717 coding	coding	coding	noncoding
MSTRG.193276.1		XLOC_202217	268 coding	noncoding	noncoding	noncoding
MSTRG.193288.1		XLOC_202218	274 coding	noncoding	noncoding	noncoding
XM_017024165.2	KRBA2	XLOC_202220	2974 coding	noncoding	coding	coding
MSTRG.193329.2	NDEL1	XLOC_202225	2236 coding	coding	noncoding	coding
MSTRG.193350.1	MYH10	XLOC_202236	256 coding	noncoding	noncoding	noncoding
MSTRG.193308.1	LOC105371525	XLOC_202238	278 coding	noncoding	noncoding	noncoding
XM_005256633.4	SPDYE4	XLOC_202240	3352 coding	coding	coding	coding
MSTRG.193440.1	LOC107985063	XLOC_202255	250 coding	noncoding	noncoding	noncoding

MSTRG.193411.1	STX8	XLOC_202279	314 coding	noncoding	noncoding	noncoding
MSTRG.193414.1	STX8	XLOC_202282	236 coding	noncoding	noncoding	noncoding
MSTRG.193381.1	CFAP52	XLOC_202286	274 coding	noncoding	noncoding	noncoding
XM_005256833.4	GAS7	XLOC_202294	8038 coding	coding	coding	coding
MSTRG.193545.1	GAS7	XLOC_202304	253 coding	noncoding	noncoding	noncoding
MSTRG.193418.1		XLOC_202311	324 coding	noncoding	noncoding	noncoding
XM_017024675.1	MYH1	XLOC_202319	6196 coding	coding	coding	coding
MSTRG.193460.3	MYH2	XLOC_202320	315 coding	noncoding	noncoding	noncoding
NM_001004313.2	TMEM220	XLOC_202325	3119 coding	coding	coding	coding
MSTRG.193492.1	SHISA6	XLOC_202341	264 coding	noncoding	noncoding	noncoding
MSTRG.193489.1		XLOC_202352	316 coding	noncoding	noncoding	noncoding
MSTRG.193722.1	DNAH9	XLOC_202359	270 coding	noncoding	noncoding	noncoding
MSTRG.193557.1		XLOC_202372	302 coding	noncoding	noncoding	noncoding
MSTRG.193612.1	MYOCD	XLOC_202377	290 coding	noncoding	noncoding	noncoding
MSTRG.193618.1	MYOCD	XLOC_202381	250 coding	noncoding	noncoding	noncoding
MSTRG.193576.1		XLOC_202395	314 coding	noncoding	noncoding	noncoding
MSTRG.193584.1		XLOC_202400	270 coding	noncoding	noncoding	noncoding
MSTRG.193648.1		XLOC_202439	304 coding	noncoding	noncoding	noncoding
MSTRG.193658.1		XLOC_202448	261 coding	noncoding	noncoding	noncoding
MSTRG.193668.1		XLOC_202457	303 coding	noncoding	noncoding	noncoding
MSTRG.193673.1	LINC02096	XLOC_202459	236 coding	noncoding	noncoding	noncoding
MSTRG.193695.1		XLOC_202476	327 coding	noncoding	noncoding	noncoding
MSTRG.193795.1	TVP23C-CDRT4	XLOC_202483	231 coding	coding	noncoding	noncoding
MSTRG.193801.1	TVP23C-CDRT4	XLOC_202486	283 coding	noncoding	noncoding	noncoding
MSTRG.193708.1	CDRT1	XLOC_202489	264 coding	noncoding	noncoding	noncoding
MSTRG.193920.1		XLOC_202507	264 coding	noncoding	noncoding	noncoding
MSTRG.193932.1		XLOC_202511	268 coding	noncoding	noncoding	noncoding
MSTRG.193977.1	TNFRSF13B	XLOC_202530	225 coding	noncoding	noncoding	noncoding
MSTRG.193971.1		XLOC_202531	229 coding	noncoding	noncoding	noncoding
NM_001199125.1	COPS3	XLOC_202538	1816 coding	coding	coding	coding
MSTRG.194011.1	COPS3	XLOC_202539	250 coding	noncoding	noncoding	noncoding
MSTRG.194005.1		XLOC_202544	349 coding	noncoding	noncoding	noncoding
MSTRG.194044.1		XLOC_202552	212 coding	noncoding	noncoding	noncoding
MSTRG.194064.1	RAI1	XLOC_202563	338 coding	noncoding	noncoding	noncoding
NR_024007.1	SMCR5	XLOC_202578	2844 coding	noncoding	coding	noncoding
MSTRG.194142.1	ATPAF2	XLOC_202608	273 coding	noncoding	noncoding	noncoding
MSTRG.194108.1		XLOC_202612	259 coding	noncoding	noncoding	noncoding
MSTRG.194151.2	DRG2	XLOC_202613	561 coding	coding	coding	coding
MSTRG.194153.1	MYO15A	XLOC_202614	219 coding	noncoding	noncoding	noncoding
MSTRG.194131.1		XLOC_202620	300 coding	noncoding	noncoding	noncoding
MSTRG.194182.1	CCDC144B	XLOC_202638	364 coding	noncoding	noncoding	noncoding
MSTRG.194217.1	SLC5A10	XLOC_202655	262 coding	noncoding	noncoding	noncoding
NM_001321215.2	B9D1	XLOC_202673	3479 coding	coding	coding	coding

MSTRG.194275.1	RNF112	XLOC_202682	265 coding	noncoding	noncoding	noncoding
MSTRG.194233.1		XLOC_202687	283 coding	noncoding	noncoding	noncoding
MSTRG.194242.1		XLOC_202688	487 coding	noncoding	noncoding	noncoding
MSTRG.194245.1	LOC112268205	XLOC_202689	219 coding	noncoding	noncoding	noncoding
MSTRG.194276.1		XLOC_202697	231 coding	noncoding	noncoding	noncoding
MSTRG.194507.7	SPECC1	XLOC_202713	2991 coding	noncoding	coding	noncoding
MSTRG.194507.11	SPECC1	XLOC_202713	3104 coding	noncoding	coding	noncoding
MSTRG.194507.14	SPECC1	XLOC_202713	4689 coding	noncoding	coding	coding
MSTRG.194559.1	LINC02088	XLOC_202740	400 coding	noncoding	noncoding	noncoding
MSTRG.194313.1	LOC100287072	XLOC_202745	256 coding	noncoding	noncoding	noncoding
MSTRG.194329.1	USP22	XLOC_202758	262 coding	noncoding	noncoding	noncoding
MSTRG.194337.1		XLOC_202773	242 coding	noncoding	noncoding	noncoding
MSTRG.194407.1		XLOC_202798	291 coding	noncoding	noncoding	noncoding
MSTRG.194452.1		XLOC_202819	276 coding	noncoding	noncoding	noncoding
MSTRG.194473.1		XLOC_202826	234 coding	noncoding	noncoding	noncoding
MSTRG.194477.1		XLOC_202828	264 coding	noncoding	noncoding	noncoding
MSTRG.194479.1	LOC105371703	XLOC_202830	261 coding	coding	noncoding	noncoding
MSTRG.194484.1		XLOC_202833	250 coding	noncoding	noncoding	noncoding
MSTRG.194568.1		XLOC_202852	315 coding	coding	noncoding	noncoding
MSTRG.194569.1		XLOC_202853	258 coding	noncoding	noncoding	noncoding
NM_000625.4	NOS2	XLOC_202855	4206 coding	coding	coding	coding
MSTRG.194575.1	NOS2	XLOC_202856	226 coding	noncoding	noncoding	noncoding
XM_011524491.3	LYRM9	XLOC_202859	4610 coding	coding	coding	noncoding
MSTRG.194589.1		XLOC_202862	270 coding	noncoding	noncoding	noncoding
MSTRG.194642.5	LOC105371709	XLOC_202866	2923 coding	coding	coding	noncoding
XR_001752821.1	LOC101926984	XLOC_202873	8835 coding	coding	coding	coding
MSTRG.194672.1	IFT20	XLOC_202878	260 coding	noncoding	noncoding	noncoding
MSTRG.194611.1		XLOC_202887	312 coding	noncoding	noncoding	noncoding
MSTRG.194617.1		XLOC_202890	228 coding	noncoding	noncoding	noncoding
MSTRG.194628.1	FOXN1	XLOC_202894	300 coding	noncoding	noncoding	noncoding
MSTRG.194631.1	FOXN1	XLOC_202896	269 coding	noncoding	noncoding	noncoding
MSTRG.194754.1	SPAG5-AS1	XLOC_202904	267 coding	noncoding	noncoding	noncoding
MSTRG.194761.8	SUPT6H	XLOC_202908	256 coding	noncoding	noncoding	noncoding
MSTRG.194728.1	FAM222B	XLOC_202922	285 coding	noncoding	noncoding	noncoding
XM_005258016.3	PHF12	XLOC_202931	3787 coding	coding	coding	coding
NM_020889.3	PHF12	XLOC_202931	2985 coding	coding	coding	coding
MSTRG.194735.1	PIPOX	XLOC_202944	241 coding	noncoding	noncoding	noncoding
MSTRG.194789.1		XLOC_202960	345 coding	noncoding	noncoding	noncoding
MSTRG.194799.1		XLOC_202965	490 coding	noncoding	noncoding	noncoding
MSTRG.194808.1		XLOC_202966	235 coding	noncoding	noncoding	noncoding
MSTRG.194840.3	TAOK1	XLOC_202968	10306 coding	coding	noncoding	noncoding
MSTRG.194852.1	GIT1	XLOC_202972	290 coding	noncoding	noncoding	noncoding
MSTRG.194859.1		XLOC_203049	264 coding	noncoding	noncoding	noncoding

MSTRG.195115.1	LOC105371723	XLOC_203068	322	coding	noncoding	noncoding	noncoding
MSTRG.195157.1	LOC107984974	XLOC_203088	286	coding	noncoding	noncoding	noncoding
MSTRG.195190.1		XLOC_203095	246	coding	noncoding	noncoding	noncoding
MSTRG.195098.1		XLOC_203117	265	coding	noncoding	noncoding	noncoding
MSTRG.195106.1	UTP6	XLOC_203121	250	coding	noncoding	noncoding	noncoding
MSTRG.195161.4	SUZ12	XLOC_203123	815	coding	noncoding	noncoding	noncoding
MSTRG.195370.1	MYO1D	XLOC_203155	702	coding	noncoding	noncoding	noncoding
MSTRG.195331.1	ASIC2	XLOC_203189	278	coding	noncoding	noncoding	noncoding
MSTRG.195334.1	ASIC2	XLOC_203190	240	coding	noncoding	noncoding	noncoding
XR_001752829.1	LOC107985035	XLOC_203198	4793	coding	noncoding	coding	noncoding
MSTRG.195290.1	LOC105371742	XLOC_203222	287	coding	noncoding	noncoding	noncoding
MSTRG.195471.1	CCT6B	XLOC_203225	266	coding	noncoding	noncoding	noncoding
MSTRG.195494.1	NLE1	XLOC_203230	207	coding	noncoding	noncoding	noncoding
MSTRG.195427.1		XLOC_203241	261	coding	noncoding	noncoding	noncoding
MSTRG.195495.1		XLOC_203283	275	coding	noncoding	noncoding	noncoding
MSTRG.195503.1	RASL10B	XLOC_203286	292	coding	noncoding	noncoding	noncoding
NM_139285.3	GAS2L2	XLOC_203287	3014	coding	coding	coding	coding
NR_027921.3	CCL15-CCL14	XLOC_203296	1464	coding	coding	coding	noncoding
MSTRG.195686.1	RPL23	XLOC_203376	488	coding	coding	coding	coding
MSTRG.195686.2	RPL23	XLOC_203376	505	coding	coding	coding	coding
MSTRG.195680.1	LRRC37A11P	XLOC_203391	277	coding	noncoding	noncoding	noncoding
MSTRG.195708.1	PLXDC1	XLOC_203394	287	coding	noncoding	noncoding	noncoding
MSTRG.195709.1	PLXDC1	XLOC_203395	290	coding	noncoding	noncoding	noncoding
MSTRG.195725.1	FBXL20	XLOC_203404	238	coding	noncoding	noncoding	noncoding
XM_005257465.3	MED1	XLOC_203411	8022	coding	coding	coding	coding
MSTRG.195762.1	PGAP3	XLOC_203422	202	coding	noncoding	noncoding	noncoding
MSTRG.195764.2	ERBB2	XLOC_203423	1476	coding	coding	coding	noncoding
MSTRG.195788.1	IKZF3	XLOC_203434	320	coding	noncoding	noncoding	noncoding
MSTRG.195810.1		XLOC_203462	234	coding	noncoding	noncoding	noncoding
MSTRG.195842.1	TOP2A	XLOC_203474	280	coding	noncoding	noncoding	noncoding
MSTRG.195863.1		XLOC_203492	277	coding	noncoding	noncoding	noncoding
MSTRG.195865.1		XLOC_203493	238	coding	noncoding	noncoding	noncoding
XM_005257200.5	KRT23	XLOC_203516	1430	coding	coding	coding	coding
XM_017024189.2	KRT40	XLOC_203519	3699	coding	coding	coding	coding
MSTRG.195907.1	KRTAP4-1	XLOC_203544	685	coding	noncoding	noncoding	noncoding
NM_001358661.2	C17orf113	XLOC_203592	3733	coding	noncoding	coding	coding
MSTRG.196024.1	STAT3	XLOC_203609	377	coding	noncoding	noncoding	noncoding
MSTRG.196027.1	STAT3	XLOC_203612	309	coding	noncoding	noncoding	noncoding
MSTRG.195988.1		XLOC_203614	227	coding	noncoding	noncoding	noncoding
MSTRG.195993.1	CAVIN1	XLOC_203616	255	coding	noncoding	noncoding	noncoding
MSTRG.196010.1		XLOC_203625	440	coding	noncoding	noncoding	noncoding
MSTRG.196058.1	WNK4	XLOC_203639	238	coding	noncoding	noncoding	noncoding
MSTRG.196062.1	BECN1	XLOC_203642	259	coding	noncoding	noncoding	noncoding

MSTRG.196065.4	PSME3	XLOC_203644	1784 coding	coding	noncoding	noncoding
MSTRG.196056.1		XLOC_203652	320 coding	noncoding	noncoding	noncoding
MSTRG.196085.1	PTGES3L-AARSD1	XLOC_203655	535 coding	noncoding	noncoding	noncoding
MSTRG.196152.1	BRCA1	XLOC_203662	246 coding	noncoding	noncoding	noncoding
MSTRG.196108.1		XLOC_203685	693 coding	noncoding	noncoding	noncoding
MSTRG.196131.1		XLOC_203687	281 coding	noncoding	noncoding	noncoding
MSTRG.196146.1	LINC02594	XLOC_203694	421 coding	noncoding	noncoding	noncoding
MSTRG.196215.1	LSM12	XLOC_203713	261 coding	noncoding	noncoding	noncoding
MSTRG.196216.1	LSM12	XLOC_203714	212 coding	noncoding	noncoding	noncoding
MSTRG.196230.1	HDAC5	XLOC_203718	303 coding	noncoding	noncoding	noncoding
MSTRG.196264.1		XLOC_203731	275 coding	noncoding	noncoding	noncoding
MSTRG.196235.1		XLOC_203734	319 coding	noncoding	noncoding	noncoding
XM_011524883.3	SLC25A39	XLOC_203738	1538 coding	coding	coding	coding
XM_011524884.1	SLC25A39	XLOC_203738	1530 coding	coding	coding	coding
NM_001366726.1	SLC25A39	XLOC_203738	1489 coding	coding	coding	coding
MSTRG.196270.1		XLOC_203754	202 coding	noncoding	noncoding	noncoding
MSTRG.196278.1		XLOC_203757	217 coding	noncoding	noncoding	noncoding
MSTRG.196276.1		XLOC_203763	326 coding	noncoding	noncoding	noncoding
MSTRG.196296.1		XLOC_203770	260 coding	noncoding	noncoding	noncoding
MSTRG.196313.1	LOC107987243	XLOC_203780	232 coding	noncoding	noncoding	noncoding
MSTRG.196326.1	DCAKD	XLOC_203785	258 coding	noncoding	noncoding	noncoding
MSTRG.196376.3	LOC107985040	XLOC_203794	4199 coding	coding	coding	coding
MSTRG.196389.1	MAP3K14	XLOC_203801	238 coding	noncoding	noncoding	noncoding
MSTRG.196472.1	ARL17B	XLOC_203822	290 coding	noncoding	noncoding	noncoding
MSTRG.196513.1	LOC101927060	XLOC_203845	268 coding	noncoding	noncoding	noncoding
NM_001353047.2	CDC27	XLOC_203846	5763 coding	coding	noncoding	coding
XM_011525549.2	CDC27	XLOC_203846	5622 coding	coding	coding	coding
XM_017025488.1	CDC27	XLOC_203846	5573 coding	coding	coding	coding
XM_017025486.1	CDC27	XLOC_203846	5266 coding	coding	coding	coding
MSTRG.196414.3	ITGB3	XLOC_203850	2893 coding	coding	coding	noncoding
MSTRG.196414.4	ITGB3	XLOC_203850	5541 coding	coding	coding	noncoding
MSTRG.196441.1	MRPL45P2	XLOC_203869	214 coding	noncoding	noncoding	noncoding
MSTRG.196442.1	MRPL45P2	XLOC_203870	264 coding	noncoding	noncoding	noncoding
MSTRG.196413.1		XLOC_203883	319 coding	noncoding	noncoding	noncoding
NM_001258248.2	SP6	XLOC_203891	3824 coding	coding	coding	coding
MSTRG.196554.6	NFE2L1	XLOC_203902	1144 coding	noncoding	coding	noncoding
MSTRG.196711.1	SKAP1	XLOC_203928	226 coding	noncoding	noncoding	noncoding
NM_002144.4	HOXB1	XLOC_203993	2034 coding	coding	coding	coding
MSTRG.196599.1	TTLL6	XLOC_204020	308 coding	noncoding	noncoding	noncoding
MSTRG.196631.3	UBE2Z	XLOC_204029	2695 coding	coding	coding	noncoding
MSTRG.196611.1		XLOC_204032	294 coding	noncoding	noncoding	noncoding
MSTRG.196648.1		XLOC_204049	249 coding	noncoding	noncoding	noncoding
MSTRG.196669.1	LOC100288866	XLOC_204054	271 coding	coding	noncoding	noncoding

NM_001007229.1	SPOP	XLOC_204055	2967 coding	coding	coding	coding
MSTRG.196802.1	FAM117A	XLOC_204069	256 coding	noncoding	noncoding	noncoding
MSTRG.196782.1		XLOC_204086	261 coding	noncoding	noncoding	noncoding
NR_038230.1	PICART1	XLOC_204092	2533 coding	noncoding	noncoding	noncoding
XR_001752932.1	LOC105371818	XLOC_204102	3224 coding	noncoding	coding	noncoding
NM_000088.3	COL1A1	XLOC_204105	5927 coding	coding	coding	coding
MSTRG.196830.1	LOC105371819	XLOC_204109	427 coding	noncoding	noncoding	noncoding
NM_018509.4	LRRC59	XLOC_204117	2880 coding	coding	coding	coding
MSTRG.196853.1		XLOC_204119	240 coding	noncoding	noncoding	noncoding
MSTRG.196883.1	CACNA1G	XLOC_204130	301 coding	noncoding	noncoding	noncoding
XM_017025296.1	ANKRD40	XLOC_204134	3911 coding	noncoding	coding	coding
MSTRG.196892.1		XLOC_204138	460 coding	noncoding	noncoding	noncoding
MSTRG.196895.1		XLOC_204140	294 coding	noncoding	noncoding	noncoding
MSTRG.196898.1	WFIKKN2	XLOC_204142	267 coding	coding	coding	noncoding
MSTRG.196907.1		XLOC_204148	252 coding	noncoding	noncoding	noncoding
MSTRG.196948.1	SPAG9	XLOC_204169	255 coding	noncoding	noncoding	noncoding
MSTRG.196950.1	SPAG9	XLOC_204171	242 coding	noncoding	noncoding	noncoding
MSTRG.196962.1		XLOC_204190	270 coding	coding	noncoding	noncoding
MSTRG.196990.1	CA10	XLOC_204204	280 coding	noncoding	noncoding	noncoding
MSTRG.197036.1		XLOC_204226	268 coding	noncoding	noncoding	noncoding
MSTRG.197041.1		XLOC_204229	264 coding	noncoding	noncoding	noncoding
MSTRG.197050.1		XLOC_204231	267 coding	noncoding	noncoding	noncoding
MSTRG.197051.1		XLOC_204232	233 coding	noncoding	noncoding	noncoding
MSTRG.197077.1		XLOC_204243	720 coding	coding	noncoding	noncoding
MSTRG.197084.1		XLOC_204246	366 coding	noncoding	noncoding	noncoding
MSTRG.197177.1	TOM1L1	XLOC_204249	320 coding	noncoding	noncoding	noncoding
MSTRG.197093.1		XLOC_204289	241 coding	noncoding	noncoding	noncoding
MSTRG.197118.1		XLOC_204305	214 coding	noncoding	noncoding	noncoding
MSTRG.197141.1		XLOC_204331	261 coding	noncoding	noncoding	noncoding
MSTRG.197145.1		XLOC_204332	281 coding	noncoding	noncoding	noncoding
MSTRG.197229.1	ANKFN1	XLOC_204336	229 coding	noncoding	noncoding	noncoding
MSTRG.197232.1	ANKFN1	XLOC_204338	247 coding	noncoding	noncoding	noncoding
NM_004645.3	COIL	XLOC_204358	2634 coding	coding	coding	coding
MSTRG.197294.1		XLOC_204365	362 coding	noncoding	noncoding	noncoding
XR_001752946.1	LOC101927510	XLOC_204368	3667 coding	noncoding	coding	noncoding
MSTRG.197393.5	MSI2	XLOC_204369	507 coding	coding	noncoding	noncoding
MSTRG.197332.1		XLOC_204407	291 coding	noncoding	noncoding	noncoding
MSTRG.197338.1		XLOC_204414	239 coding	noncoding	noncoding	noncoding
MSTRG.197382.1	MPO	XLOC_204423	231 coding	noncoding	noncoding	noncoding
XM_005257784.2	MTMR4	XLOC_204436	5977 coding	coding	coding	coding
MSTRG.197496.1	SEPTIN4-AS1	XLOC_204439	260 coding	noncoding	noncoding	noncoding
XM_011525029.3	TEX14	XLOC_204441	5030 coding	coding	coding	coding
MSTRG.197501.1	LOC107985048	XLOC_204442	292 coding	noncoding	noncoding	noncoding

MSTRG.197519.1	PPM1E	XLOC_204448	254	coding	noncoding	noncoding	noncoding
NM_001005207.5	TRIM37	XLOC_204449	3583	coding	coding	coding	coding
MSTRG.197525.1	PPM1E	XLOC_204450	281	coding	noncoding	noncoding	noncoding
MSTRG.197530.1	TRIM37	XLOC_204453	291	coding	noncoding	noncoding	noncoding
MSTRG.197533.1	TRIM37	XLOC_204456	442	coding	noncoding	noncoding	noncoding
MSTRG.197468.1		XLOC_204470	257	coding	noncoding	noncoding	noncoding
MSTRG.197471.1	LINC01476	XLOC_204471	284	coding	noncoding	noncoding	noncoding
MSTRG.197486.1	LINC01476	XLOC_204475	225	coding	noncoding	noncoding	noncoding
MSTRG.197747.7	PTRH2	XLOC_204478	9280	coding	coding	coding	noncoding
MSTRG.197747.16	VMP1	XLOC_204478	6103	coding	noncoding	coding	noncoding
MSTRG.197794.2	CA4	XLOC_204502	467	coding	coding	noncoding	noncoding
MSTRG.197795.12	USP32	XLOC_204503	1844	coding	coding	coding	coding
MSTRG.197543.1	APPBP2	XLOC_204532	282	coding	noncoding	noncoding	noncoding
MSTRG.197540.1	LINC01999	XLOC_204538	202	coding	noncoding	noncoding	noncoding
MSTRG.197550.2	PPM1D	XLOC_204539	1479	coding	coding	noncoding	noncoding
MSTRG.197566.1		XLOC_204553	273	coding	noncoding	noncoding	noncoding
MSTRG.197567.1		XLOC_204554	330	coding	noncoding	noncoding	noncoding
MSTRG.197575.1	BRIP1	XLOC_204560	284	coding	noncoding	noncoding	noncoding
MSTRG.197601.1	MED13	XLOC_204568	297	coding	noncoding	noncoding	noncoding
MSTRG.197606.1	MED13	XLOC_204572	304	coding	noncoding	noncoding	noncoding
MSTRG.197582.1		XLOC_204577	224	coding	noncoding	noncoding	noncoding
MSTRG.197583.1		XLOC_204578	289	coding	noncoding	noncoding	noncoding
MSTRG.197594.1	EFCAB3	XLOC_204584	261	coding	noncoding	noncoding	noncoding
MSTRG.197644.1	TLK2	XLOC_204591	310	coding	noncoding	noncoding	noncoding
XR_934406.3	10-Mar	XLOC_204602	3811	coding	coding	coding	coding
MSTRG.197640.1	10-Mar	XLOC_204605	218	coding	noncoding	noncoding	noncoding
MSTRG.197624.1	LOC101927877	XLOC_204606	384	coding	noncoding	noncoding	noncoding
MSTRG.197657.1		XLOC_204620	270	coding	noncoding	noncoding	noncoding
MSTRG.197665.1	ACE	XLOC_204621	266	coding	coding	noncoding	noncoding
MSTRG.197838.1	STRADA	XLOC_204636	287	coding	noncoding	noncoding	noncoding
MSTRG.197894.13	FTSJ3	XLOC_204638	1306	coding	coding	coding	coding
NM_000442.5	PECAM1	XLOC_204668	6813	coding	coding	coding	coding
MSTRG.197921.2	PECAM1	XLOC_204668	6764	coding	coding	coding	coding
MSTRG.197968.1	PLEKHM1P1	XLOC_204699	236	coding	noncoding	noncoding	noncoding
MSTRG.197947.1		XLOC_204711	258	coding	noncoding	noncoding	noncoding
MSTRG.197980.1		XLOC_204721	278	coding	noncoding	noncoding	noncoding
XM_011525320.1	AXIN2	XLOC_204729	4434	coding	coding	coding	coding
MSTRG.198029.1	AXIN2	XLOC_204730	270	coding	noncoding	noncoding	noncoding
MSTRG.198055.1	CEP112	XLOC_204738	269	coding	noncoding	noncoding	noncoding
MSTRG.198060.1	CEP112	XLOC_204741	301	coding	noncoding	noncoding	noncoding
MSTRG.198077.1	CEP112	XLOC_204750	248	coding	noncoding	noncoding	noncoding
MSTRG.198083.1	CEP112	XLOC_204753	289	coding	coding	noncoding	noncoding
XR_001752712.2	HELZ	XLOC_204767	3770	coding	coding	coding	coding

MSTRG.198108.1	HELZ	XLOC_204768	295	coding	noncoding	noncoding	noncoding
MSTRG.198094.1		XLOC_204784	217	coding	noncoding	noncoding	noncoding
MSTRG.198097.1		XLOC_204785	238	coding	noncoding	noncoding	noncoding
MSTRG.198099.1		XLOC_204786	282	coding	noncoding	noncoding	noncoding
MSTRG.198176.1		XLOC_204787	346	coding	noncoding	noncoding	noncoding
MSTRG.198126.1		XLOC_204798	394	coding	noncoding	noncoding	noncoding
MSTRG.198127.1		XLOC_204799	321	coding	noncoding	noncoding	noncoding
MSTRG.198232.9	BPTF	XLOC_204800	9543	coding	coding	coding	noncoding
MSTRG.198222.1	LOC105371874	XLOC_204821	307	coding	noncoding	noncoding	noncoding
MSTRG.198392.1	LINC01483	XLOC_204856	289	coding	noncoding	noncoding	noncoding
MSTRG.198359.1	KCNJ16	XLOC_204878	310	coding	coding	noncoding	noncoding
MSTRG.198360.1		XLOC_204888	275	coding	noncoding	noncoding	noncoding
MSTRG.198361.1		XLOC_204889	287	coding	noncoding	noncoding	noncoding
MSTRG.198420.1		XLOC_204892	301	coding	noncoding	noncoding	noncoding
MSTRG.198421.1		XLOC_204893	350	coding	noncoding	noncoding	noncoding
MSTRG.198436.1		XLOC_204899	302	coding	noncoding	noncoding	noncoding
MSTRG.198451.1		XLOC_204906	264	coding	noncoding	noncoding	noncoding
MSTRG.198474.1		XLOC_204920	241	coding	noncoding	noncoding	noncoding
NR_103737.1	SOX9-AS1	XLOC_204923	1739	coding	noncoding	noncoding	noncoding
MSTRG.198514.1		XLOC_204954	229	coding	noncoding	noncoding	noncoding
MSTRG.198523.1	CDC42EP4	XLOC_204961	254	coding	noncoding	noncoding	noncoding
MSTRG.198536.1	SDK2	XLOC_204963	219	coding	noncoding	noncoding	noncoding
MSTRG.198557.1	SDK2	XLOC_204970	299	coding	noncoding	noncoding	noncoding
MSTRG.198529.1		XLOC_204971	269	coding	noncoding	noncoding	noncoding
MSTRG.198568.1	LINC00469	XLOC_204976	308	coding	noncoding	noncoding	noncoding
MSTRG.198586.1		XLOC_204985	221	coding	noncoding	noncoding	noncoding
MSTRG.198601.1		XLOC_204991	309	coding	noncoding	noncoding	noncoding
MSTRG.198657.1		XLOC_204997	263	coding	noncoding	noncoding	noncoding
XM_011525569.2	CD300H	XLOC_205009	1159	coding	coding	coding	coding
MSTRG.198719.1	RAB37	XLOC_205018	273	coding	noncoding	noncoding	noncoding
MSTRG.198721.1	RAB37	XLOC_205020	286	coding	noncoding	noncoding	noncoding
MSTRG.198722.1	RAB37	XLOC_205021	245	coding	noncoding	noncoding	noncoding
MSTRG.198724.1	RAB37	XLOC_205022	252	coding	noncoding	noncoding	noncoding
NM_173477.5	USH1G	XLOC_205033	3558	coding	coding	coding	coding
MSTRG.198709.1	KCTD2	XLOC_205041	365	coding	noncoding	noncoding	noncoding
MSTRG.198739.4	SLC16A5	XLOC_205043	556	coding	coding	coding	noncoding
MSTRG.198746.1		XLOC_205054	348	coding	noncoding	noncoding	noncoding
MSTRG.198752.1		XLOC_205067	272	coding	noncoding	noncoding	noncoding
NM_020753.5	CASKIN2	XLOC_205077	4969	coding	coding	coding	coding
MSTRG.198831.1	MYO15B	XLOC_205083	757	coding	noncoding	coding	noncoding
MSTRG.198834.1	RECQL5	XLOC_205086	10058	coding	coding	coding	coding
MSTRG.198850.1	ACOX1	XLOC_205107	249	coding	noncoding	noncoding	noncoding
MSTRG.198870.1	RNF157	XLOC_205121	266	coding	noncoding	noncoding	noncoding

MSTRG.198871.2	UBALD2	XLOC_205124	1287	coding	coding	coding	noncoding
MSTRG.198887.1	PRPSAP1	XLOC_205126	314	coding	noncoding	noncoding	noncoding
MSTRG.198884.1		XLOC_205129	255	coding	noncoding	noncoding	noncoding
MSTRG.198963.1	JMJD6	XLOC_205154	238	coding	noncoding	noncoding	noncoding
MSTRG.198944.1		XLOC_205163	336	coding	noncoding	noncoding	noncoding
MSTRG.198958.1	MGAT5B	XLOC_205166	337	coding	noncoding	noncoding	noncoding
MSTRG.198947.1		XLOC_205169	306	coding	noncoding	noncoding	noncoding
MSTRG.198951.1		XLOC_205172	306	coding	noncoding	noncoding	noncoding
MSTRG.198953.1		XLOC_205173	291	coding	noncoding	noncoding	noncoding
MSTRG.199026.25	SEC14L1	XLOC_205186	4750	coding	coding	coding	coding
MSTRG.198972.1		XLOC_205189	229	coding	noncoding	noncoding	noncoding
XR_934990.2	LOC105371903	XLOC_205204	2879	coding	noncoding	coding	noncoding
NR_040071.1	TNRC6C-AS1	XLOC_205229	3189	coding	noncoding	coding	noncoding
MSTRG.199119.20	TMC6	XLOC_205229	4494	coding	coding	coding	coding
MSTRG.199093.1	AFMID	XLOC_205235	211	coding	noncoding	noncoding	noncoding
MSTRG.199076.1	BIRC5	XLOC_205239	930	coding	noncoding	noncoding	noncoding
MSTRG.199083.1		XLOC_205242	241	coding	noncoding	noncoding	noncoding
MSTRG.199133.2	DNAH17	XLOC_205246	14929	coding	coding	noncoding	coding
MSTRG.199098.1		XLOC_205250	277	coding	noncoding	noncoding	noncoding
XR_001752573.2	USP36	XLOC_205276	5530	coding	coding	coding	coding
MSTRG.199273.1	TIMP2	XLOC_205282	287	coding	noncoding	noncoding	noncoding
MSTRG.199276.1	TIMP2	XLOC_205285	204	coding	noncoding	noncoding	noncoding
MSTRG.199163.1	ENGASE	XLOC_205296	1051	coding	noncoding	coding	coding
MSTRG.199187.1	RBFOX3	XLOC_205302	252	coding	noncoding	noncoding	noncoding
MSTRG.199192.1	RBFOX3	XLOC_205305	258	coding	noncoding	noncoding	noncoding
MSTRG.199198.1	RBFOX3	XLOC_205308	220	coding	noncoding	noncoding	noncoding
MSTRG.199206.1	RBFOX3	XLOC_205311	277	coding	noncoding	noncoding	noncoding
XM_024450576.1	TBC1D16	XLOC_205325	11134	coding	coding	coding	coding
MSTRG.199225.1	TBC1D16	XLOC_205326	311	coding	noncoding	noncoding	noncoding
MSTRG.199228.1	TBC1D16	XLOC_205327	263	coding	noncoding	noncoding	noncoding
MSTRG.199282.1	CCDC40	XLOC_205328	323	coding	noncoding	noncoding	noncoding
MSTRG.199290.1	CARD14	XLOC_205333	282	coding	noncoding	noncoding	noncoding
MSTRG.199295.1	SLC26A11	XLOC_205337	242	coding	noncoding	noncoding	noncoding
MSTRG.199330.13	LOC100294362	XLOC_205342	20095	coding	coding	coding	coding
NR_026857.1	BAIAP2-DT	XLOC_205366	4421	coding	noncoding	noncoding	coding
NR_038080.1	LINC00482	XLOC_205390	2970	coding	noncoding	coding	coding
MSTRG.199407.1	FSCN2	XLOC_205406	256	coding	noncoding	noncoding	noncoding
MSTRG.199480.2	HGS	XLOC_205422	2857	coding	coding	coding	noncoding
XM_011523538.2	FASN	XLOC_205455	8526	coding	coding	noncoding	coding
XM_017024464.2	CCDC57	XLOC_205457	4548	coding	noncoding	coding	coding
MSTRG.199544.1	CCDC57	XLOC_205461	307	coding	noncoding	noncoding	noncoding
NR_135639.1	LINC01970	XLOC_205463	1810	coding	noncoding	coding	noncoding
MSTRG.199609.1	RAB40B	XLOC_205484	271	coding	noncoding	noncoding	noncoding

MSTRG.199639.1		XLOC_205514	234 coding	noncoding	noncoding	noncoding
MSTRG.199663.1	COLEC12	XLOC_205520	267 coding	noncoding	noncoding	noncoding
MSTRG.199653.1		XLOC_205528	312 coding	noncoding	noncoding	noncoding
MSTRG.199748.1		XLOC_205562	252 coding	noncoding	noncoding	noncoding
MSTRG.199762.1	LOC105371956	XLOC_205570	396 coding	noncoding	noncoding	noncoding
MSTRG.199818.1		XLOC_205609	215 coding	noncoding	noncoding	noncoding
MSTRG.199874.1	MYOM1	XLOC_205611	252 coding	noncoding	noncoding	noncoding
NM_001303047.1	MYL12A	XLOC_205624	1313 coding	coding	coding	coding
NM_173211.1	TGIF1	XLOC_205635	1357 coding	coding	coding	coding
MSTRG.199964.1	DLGAP1	XLOC_205646	275 coding	noncoding	noncoding	noncoding
MSTRG.199967.1	DLGAP1	XLOC_205648	266 coding	noncoding	noncoding	noncoding
MSTRG.200062.1		XLOC_205788	279 coding	noncoding	noncoding	noncoding
NM_001366230.1	ARHGAP28	XLOC_205791	5858 coding	coding	coding	coding
MSTRG.200082.1	ARHGAP28	XLOC_205793	301 coding	noncoding	noncoding	noncoding
MSTRG.200073.1		XLOC_205800	287 coding	noncoding	noncoding	noncoding
MSTRG.200075.1		XLOC_205801	268 coding	coding	noncoding	noncoding
MSTRG.200114.1		XLOC_205810	317 coding	coding	noncoding	noncoding
MSTRG.200116.1		XLOC_205811	310 coding	noncoding	noncoding	noncoding
MSTRG.200343.1	PTPRM	XLOC_205830	248 coding	noncoding	noncoding	noncoding
XM_017025674.2	MTCL1	XLOC_205845	6304 coding	coding	coding	coding
MSTRG.200219.1		XLOC_205850	273 coding	noncoding	noncoding	noncoding
XM_017025670.2	ANKRD12	XLOC_205867	8027 coding	coding	coding	coding
MSTRG.200375.1	ANKRD12	XLOC_205877	407 coding	noncoding	noncoding	noncoding
MSTRG.200305.3	PPP4R1	XLOC_205893	4593 coding	coding	noncoding	noncoding
MSTRG.200305.7	PPP4R1	XLOC_205893	3735 coding	coding	noncoding	noncoding
MSTRG.200305.10	PPP4R1	XLOC_205893	3245 coding	coding	coding	noncoding
XM_017025529.2	RAB31	XLOC_205901	4143 coding	coding	coding	coding
MSTRG.200266.1		XLOC_205922	246 coding	noncoding	noncoding	noncoding
MSTRG.200414.1		XLOC_205949	285 coding	noncoding	noncoding	noncoding
MSTRG.200424.1		XLOC_205956	398 coding	noncoding	noncoding	noncoding
MSTRG.200480.1	PIEZO2	XLOC_205970	313 coding	noncoding	noncoding	noncoding
MSTRG.200484.1	PIEZO2	XLOC_205971	209 coding	noncoding	noncoding	noncoding
MSTRG.200488.1	PIEZO2	XLOC_205973	200 coding	noncoding	noncoding	noncoding
MSTRG.200490.1	PIEZO2	XLOC_205974	259 coding	noncoding	noncoding	noncoding
MSTRG.200459.1		XLOC_205985	238 coding	coding	noncoding	noncoding
MSTRG.200533.1	GNAL	XLOC_205991	289 coding	noncoding	noncoding	noncoding
MSTRG.200503.1	IMPA2	XLOC_206003	380 coding	noncoding	noncoding	noncoding
MSTRG.200506.1		XLOC_206006	302 coding	noncoding	noncoding	noncoding
MSTRG.200519.1	ANKRD62	XLOC_206009	286 coding	noncoding	noncoding	noncoding
NM_001303525.1	TUBB6	XLOC_206019	1192 coding	coding	coding	coding
MSTRG.200578.1	SPIRE1	XLOC_206023	245 coding	noncoding	noncoding	noncoding
MSTRG.200581.1	SPIRE1	XLOC_206025	229 coding	noncoding	noncoding	noncoding
MSTRG.200585.1	SPIRE1	XLOC_206028	272 coding	noncoding	noncoding	noncoding

MSTRG.200597.1	SPIRE1	XLOC_206037	270 coding	noncoding	noncoding	noncoding
MSTRG.200603.1	CEP76	XLOC_206042	314 coding	noncoding	noncoding	noncoding
MSTRG.200555.1		XLOC_206049	284 coding	noncoding	noncoding	noncoding
MSTRG.200632.17	CEP192	XLOC_206068	1976 coding	noncoding	coding	coding
MSTRG.200628.1		XLOC_206080	327 coding	noncoding	noncoding	noncoding
NM_001276251.1	LDLRAD4	XLOC_206081	8682 coding	noncoding	coding	coding
MSTRG.200947.1	LDLRAD4	XLOC_206102	240 coding	noncoding	noncoding	noncoding
MSTRG.200952.1	LDLRAD4	XLOC_206107	266 coding	noncoding	noncoding	noncoding
MSTRG.200656.1		XLOC_206144	280 coding	noncoding	noncoding	noncoding
MSTRG.200742.1		XLOC_206172	293 coding	coding	noncoding	noncoding
NR_026756.1	CYP4F35P	XLOC_206177	2876 coding	noncoding	coding	noncoding
MSTRG.200768.1		XLOC_206180	214 coding	noncoding	noncoding	noncoding
NM_001367607.1	ANKRD30B	XLOC_206185	4927 coding	coding	coding	coding
MSTRG.200784.1		XLOC_206190	282 coding	noncoding	noncoding	noncoding
MSTRG.200800.1		XLOC_206195	372 coding	noncoding	noncoding	noncoding
MSTRG.200834.1		XLOC_206214	308 coding	noncoding	noncoding	noncoding
MSTRG.200858.1	ESCO1	XLOC_206221	5193 coding	coding	coding	noncoding
MSTRG.200840.1		XLOC_206222	234 coding	noncoding	noncoding	noncoding
MSTRG.200846.1	SNRPD1	XLOC_206224	252 coding	noncoding	noncoding	noncoding
MSTRG.200953.8	ABHD3	XLOC_206230	1978 coding	noncoding	noncoding	noncoding
MSTRG.201005.1	MIB1	XLOC_206238	237 coding	noncoding	noncoding	noncoding
MSTRG.200967.1	LINC01900	XLOC_206250	282 coding	noncoding	noncoding	noncoding
MSTRG.200973.1		XLOC_206253	227 coding	noncoding	noncoding	noncoding
MSTRG.200999.1		XLOC_206272	287 coding	noncoding	noncoding	noncoding
MSTRG.201033.1		XLOC_206292	296 coding	noncoding	noncoding	noncoding
MSTRG.201034.1		XLOC_206293	238 coding	noncoding	noncoding	noncoding
MSTRG.201098.1	LAMA3	XLOC_206324	385 coding	noncoding	noncoding	noncoding
XR_935293.2	LOC105372026	XLOC_206368	5014 coding	coding	noncoding	noncoding
MSTRG.201205.1		XLOC_206394	406 coding	noncoding	noncoding	noncoding
MSTRG.201207.1		XLOC_206395	298 coding	noncoding	noncoding	noncoding
MSTRG.201254.1		XLOC_206412	256 coding	noncoding	noncoding	noncoding
MSTRG.201273.1	PSMA8	XLOC_206426	309 coding	noncoding	noncoding	noncoding
MSTRG.201372.1	TAF4B	XLOC_206432	214 coding	noncoding	noncoding	noncoding
MSTRG.201375.1	TAF4B	XLOC_206435	235 coding	noncoding	noncoding	noncoding
MSTRG.201376.1	TAF4B	XLOC_206436	279 coding	noncoding	noncoding	noncoding
MSTRG.201302.1		XLOC_206460	278 coding	noncoding	noncoding	noncoding
MSTRG.201344.1	AQP4-AS1	XLOC_206462	313 coding	noncoding	noncoding	noncoding
MSTRG.201382.1		XLOC_206512	292 coding	noncoding	noncoding	noncoding
MSTRG.201403.1		XLOC_206525	259 coding	noncoding	noncoding	noncoding
MSTRG.201469.1		XLOC_206545	259 coding	noncoding	noncoding	noncoding
MSTRG.201509.1		XLOC_206570	252 coding	noncoding	noncoding	noncoding
NM_001942.4	DSG1	XLOC_206573	7191 coding	coding	coding	coding
NM_001943.5	DSG2	XLOC_206580	5697 coding	coding	coding	coding

NM_000371.3	TTR	XLOC_206582	929	coding	coding	coding	coding
MSTRG.201561.1		XLOC_206599	209	coding	noncoding	noncoding	noncoding
XM_011526045.3	RNF125	XLOC_206601	1532	coding	coding	coding	coding
MSTRG.201601.1	RNF125	XLOC_206606	239	coding	noncoding	noncoding	noncoding
XM_005258285.1	RNF138	XLOC_206607	3347	coding	coding	noncoding	coding
MSTRG.201623.1	GAREM1	XLOC_206616	216	coding	noncoding	noncoding	noncoding
MSTRG.201680.1	ASXL3	XLOC_206653	215	coding	noncoding	noncoding	noncoding
MSTRG.201711.1	NOL4	XLOC_206663	504	coding	noncoding	coding	noncoding
MSTRG.201695.1		XLOC_206664	295	coding	noncoding	noncoding	noncoding
MSTRG.201696.1		XLOC_206665	231	coding	noncoding	noncoding	noncoding
MSTRG.201722.1	DTNA	XLOC_206670	299	coding	noncoding	noncoding	noncoding
MSTRG.201734.1	DTNA	XLOC_206673	437	coding	noncoding	noncoding	noncoding
MSTRG.201713.1		XLOC_206678	364	coding	noncoding	noncoding	noncoding
MSTRG.201786.1		XLOC_206733	292	coding	noncoding	noncoding	noncoding
MSTRG.201791.1		XLOC_206736	268	coding	noncoding	noncoding	noncoding
MSTRG.201817.1		XLOC_206738	270	coding	noncoding	noncoding	noncoding
MSTRG.201845.1		XLOC_206750	231	coding	noncoding	coding	noncoding
MSTRG.201893.1	ELP2	XLOC_206777	259	coding	noncoding	noncoding	noncoding
MSTRG.202290.1	FHOD3	XLOC_206789	254	coding	noncoding	noncoding	noncoding
XM_005258318.5	KIAA1328	XLOC_206799	6420	coding	coding	coding	coding
MSTRG.202335.1	KIAA1328	XLOC_206800	552	coding	noncoding	noncoding	noncoding
MSTRG.201910.1		XLOC_206817	252	coding	noncoding	noncoding	noncoding
MSTRG.201918.1		XLOC_206821	312	coding	noncoding	noncoding	noncoding
MSTRG.201964.1		XLOC_206839	261	coding	noncoding	noncoding	noncoding
MSTRG.201996.1		XLOC_206840	545	coding	noncoding	noncoding	noncoding
MSTRG.202017.1	MIR924HG	XLOC_206848	297	coding	noncoding	noncoding	noncoding
MSTRG.202026.1	MIR924HG	XLOC_206853	247	coding	noncoding	noncoding	noncoding
MSTRG.202000.1		XLOC_206865	417	coding	noncoding	noncoding	noncoding
MSTRG.202075.1		XLOC_206893	221	coding	noncoding	noncoding	noncoding
MSTRG.202085.1		XLOC_206897	308	coding	noncoding	noncoding	noncoding
MSTRG.202099.1		XLOC_206906	208	coding	noncoding	noncoding	noncoding
MSTRG.202223.1		XLOC_206931	266	coding	noncoding	noncoding	noncoding
MSTRG.202264.1	LINC00907	XLOC_206941	303	coding	noncoding	noncoding	noncoding
MSTRG.202130.1		XLOC_206962	318	coding	noncoding	noncoding	noncoding
MSTRG.202149.1		XLOC_206974	212	coding	coding	noncoding	noncoding
XM_024451151.1	SETBP1	XLOC_206999	18382	coding	noncoding	coding	coding
XM_011526144.1	SLC14A1	XLOC_207045	1655	coding	coding	coding	coding
NM_001308278.2	SLC14A1	XLOC_207045	3745	coding	coding	coding	coding
MSTRG.202439.1	HAUS1	XLOC_207077	202	coding	noncoding	noncoding	noncoding
MSTRG.202440.1		XLOC_207078	315	coding	noncoding	noncoding	noncoding
MSTRG.202484.1	ST8SIA5	XLOC_207117	246	coding	noncoding	noncoding	noncoding
MSTRG.202613.28	HDHD2	XLOC_207137	2261	coding	coding	noncoding	noncoding
MSTRG.202643.1	ZBTB7C	XLOC_207176	250	coding	noncoding	noncoding	noncoding

MSTRG.202689.1		XLOC_207181	283 coding	noncoding	noncoding	noncoding
XR_243880.2	LINC02837	XLOC_207201	2260 coding	noncoding	noncoding	noncoding
NM_006033.4	LIPG	XLOC_207203	10418 coding	coding	coding	coding
XR_001753447.2	LOC107984031	XLOC_207207	3627 coding	noncoding	noncoding	noncoding
MSTRG.202747.1	MYO5B	XLOC_207218	256 coding	coding	coding	noncoding
MSTRG.202754.1	MYO5B	XLOC_207224	218 coding	noncoding	noncoding	noncoding
NM_001039535.3	SKA1	XLOC_207230	2894 coding	coding	coding	coding
XM_011526077.2	MAPK4	XLOC_207233	5747 coding	coding	coding	coding
MSTRG.202818.1		XLOC_207238	345 coding	noncoding	noncoding	noncoding
MSTRG.202838.1		XLOC_207246	275 coding	noncoding	noncoding	noncoding
MSTRG.202879.1		XLOC_207262	270 coding	noncoding	noncoding	noncoding
MSTRG.202909.1		XLOC_207275	678 coding	noncoding	noncoding	noncoding
NR_040074.1	LINC01630	XLOC_207276	1799 coding	noncoding	noncoding	noncoding
MSTRG.202972.1	DCC	XLOC_207297	308 coding	noncoding	noncoding	noncoding
MSTRG.202989.1	DCC	XLOC_207303	282 coding	noncoding	noncoding	noncoding
MSTRG.203011.1		XLOC_207327	300 coding	noncoding	noncoding	noncoding
XM_024451081.1	POLI	XLOC_207346	4018 coding	coding	coding	coding
MSTRG.203060.1		XLOC_207351	471 coding	noncoding	noncoding	noncoding
MSTRG.203062.1		XLOC_207357	283 coding	noncoding	noncoding	noncoding
MSTRG.203140.3	TCF4	XLOC_207368	2074 coding	coding	noncoding	coding
MSTRG.203096.1		XLOC_207378	302 coding	noncoding	noncoding	noncoding
MSTRG.203120.1	LOC642484	XLOC_207382	321 coding	noncoding	noncoding	noncoding
MSTRG.203112.1		XLOC_207388	285 coding	noncoding	noncoding	noncoding
XM_006722431.2	WDR7	XLOC_207400	7201 coding	coding	coding	coding
NM_001257964.2	BOD1L2	XLOC_207441	3324 coding	coding	coding	coding
MSTRG.203189.1		XLOC_207455	261 coding	noncoding	noncoding	noncoding
MSTRG.203277.4	FECH	XLOC_207457	3693 coding	coding	noncoding	noncoding
MSTRG.203282.1		XLOC_207460	272 coding	noncoding	noncoding	noncoding
MSTRG.203284.1		XLOC_207461	278 coding	noncoding	noncoding	noncoding
MSTRG.203290.1	ATP8B1	XLOC_207466	230 coding	noncoding	noncoding	noncoding
MSTRG.203302.1	ATP8B1	XLOC_207471	237 coding	noncoding	noncoding	noncoding
MSTRG.203194.1		XLOC_207475	503 coding	noncoding	noncoding	noncoding
MSTRG.203200.1		XLOC_207477	232 coding	noncoding	noncoding	noncoding
NM_001144967.3	NEDD4L	XLOC_207478	8633 coding	coding	coding	coding
MSTRG.203274.1		XLOC_207507	206 coding	noncoding	noncoding	noncoding
MSTRG.203321.1		XLOC_207516	238 coding	noncoding	noncoding	noncoding
MSTRG.203353.1	RAX	XLOC_207523	270 coding	noncoding	coding	noncoding
MSTRG.203380.1	CCBE1	XLOC_207533	311 coding	noncoding	noncoding	noncoding
MSTRG.203392.1	CCBE1	XLOC_207539	277 coding	noncoding	noncoding	noncoding
MSTRG.203372.1		XLOC_207544	283 coding	noncoding	noncoding	noncoding
MSTRG.203374.1		XLOC_207546	352 coding	noncoding	noncoding	noncoding
MSTRG.203400.1		XLOC_207550	248 coding	noncoding	noncoding	noncoding
MSTRG.203413.1		XLOC_207557	210 coding	noncoding	noncoding	noncoding

MSTRG.203418.1	LOC105372152	XLOC_207560	260 coding	noncoding	noncoding	noncoding
MSTRG.203423.1		XLOC_207564	299 coding	noncoding	noncoding	noncoding
MSTRG.203429.1		XLOC_207567	209 coding	noncoding	noncoding	noncoding
MSTRG.203454.1		XLOC_207581	224 coding	noncoding	noncoding	noncoding
MSTRG.203469.1	CDH20	XLOC_207587	304 coding	noncoding	noncoding	noncoding
MSTRG.203515.1		XLOC_207645	250 coding	noncoding	noncoding	noncoding
XM_006722493.3	ZCCHC2	XLOC_207662	5326 coding	coding	coding	coding
MSTRG.203524.1		XLOC_207665	235 coding	noncoding	noncoding	noncoding
MSTRG.203592.1	PHLPP1	XLOC_207672	310 coding	noncoding	noncoding	noncoding
MSTRG.203676.2	VPS4B	XLOC_207696	3137 coding	coding	noncoding	noncoding
MSTRG.203685.1	SERPINB3	XLOC_207708	312 coding	coding	noncoding	noncoding
MSTRG.203693.1		XLOC_207716	234 coding	noncoding	noncoding	noncoding
MSTRG.203744.1	HMSD	XLOC_207722	331 coding	noncoding	noncoding	noncoding
MSTRG.203731.1	LINC01924	XLOC_207736	245 coding	noncoding	noncoding	noncoding
MSTRG.203712.1		XLOC_207739	265 coding	noncoding	noncoding	noncoding
MSTRG.203738.1		XLOC_207745	280 coding	noncoding	noncoding	noncoding
MSTRG.203768.1	LOC107985178	XLOC_207748	292 coding	noncoding	noncoding	noncoding
MSTRG.203773.1	LOC101927404	XLOC_207751	296 coding	noncoding	noncoding	noncoding
MSTRG.203765.1		XLOC_207754	260 coding	noncoding	noncoding	noncoding
MSTRG.203775.1		XLOC_207755	324 coding	noncoding	noncoding	noncoding
MSTRG.203784.1		XLOC_207759	303 coding	noncoding	noncoding	noncoding
NM_001362438.2	CDH7	XLOC_207767	12938 coding	coding	coding	coding
MSTRG.203866.1	LOC643542	XLOC_207799	312 coding	noncoding	noncoding	noncoding
MSTRG.203883.1	LOC643542	XLOC_207806	287 coding	noncoding	noncoding	noncoding
XM_017025975.1	CCDC102B	XLOC_207812	2901 coding	noncoding	coding	coding
MSTRG.203954.1		XLOC_207826	314 coding	coding	noncoding	noncoding
MSTRG.204037.1	DOK6	XLOC_207837	206 coding	noncoding	noncoding	noncoding
MSTRG.204090.1	CD226	XLOC_207868	4232 coding	coding	noncoding	noncoding
MSTRG.204090.11	CD226	XLOC_207868	549 coding	coding	noncoding	noncoding
MSTRG.204014.1		XLOC_207909	265 coding	noncoding	noncoding	noncoding
MSTRG.204111.1		XLOC_207930	324 coding	noncoding	noncoding	noncoding
MSTRG.204195.1		XLOC_207949	281 coding	noncoding	noncoding	noncoding
MSTRG.204280.1	LOC400655	XLOC_207985	347 coding	noncoding	noncoding	noncoding
MSTRG.204319.1		XLOC_207997	263 coding	noncoding	noncoding	noncoding
MSTRG.204331.1		XLOC_208008	335 coding	noncoding	noncoding	noncoding
MSTRG.204342.1	FBXO15	XLOC_208014	247 coding	coding	noncoding	noncoding
MSTRG.204345.1	FBXO15	XLOC_208017	252 coding	noncoding	coding	noncoding
MSTRG.204352.1		XLOC_208028	304 coding	noncoding	noncoding	noncoding
MSTRG.204384.1	DIPK1C	XLOC_208036	312 coding	noncoding	noncoding	noncoding
NM_001370250.1	CNDP2	XLOC_208038	4988 coding	coding	coding	coding
MSTRG.204409.1		XLOC_208146	256 coding	noncoding	noncoding	noncoding
MSTRG.204527.3	ZNF516	XLOC_208180	5713 coding	coding	coding	coding
MSTRG.204523.1		XLOC_208198	273 coding	noncoding	noncoding	noncoding

NM_007345.3	ZNF236	XLOC_208207	8124 coding	coding	coding	coding
MSTRG.204558.1		XLOC_208223	284 coding	noncoding	noncoding	noncoding
MSTRG.204654.1		XLOC_208230	228 coding	noncoding	noncoding	noncoding
NM_001318511.2	CTDP1	XLOC_208289	5699 coding	coding	coding	coding
MSTRG.204819.1	TXNL4A	XLOC_208299	445 coding	noncoding	noncoding	noncoding
MSTRG.204821.1	TXNL4A	XLOC_208300	272 coding	noncoding	noncoding	noncoding
MSTRG.204835.1		XLOC_208318	262 coding	noncoding	noncoding	noncoding
MSTRG.199642.1	TUBB8P12	XLOC_208321	262 coding	noncoding	noncoding	noncoding
XM_017026104.2	THOC1	XLOC_208328	1995 coding	coding	coding	coding
MSTRG.199664.1	COLEC12	XLOC_208331	267 coding	noncoding	noncoding	noncoding
MSTRG.199656.1		XLOC_208335	257 coding	coding	noncoding	noncoding
MSTRG.199694.1	CLUL1	XLOC_208339	246 coding	noncoding	noncoding	noncoding
MSTRG.199696.1	CLUL1	XLOC_208341	228 coding	noncoding	noncoding	noncoding
XM_024451215.1	ENOSF1	XLOC_208342	3625 coding	coding	coding	coding
MSTRG.199743.1		XLOC_208370	336 coding	noncoding	noncoding	noncoding
MSTRG.199789.1		XLOC_208391	227 coding	noncoding	noncoding	noncoding
MSTRG.199798.1	METTL4	XLOC_208395	277 coding	noncoding	noncoding	noncoding
MSTRG.199806.1		XLOC_208403	253 coding	noncoding	noncoding	noncoding
XM_005258178.3	LPIN2	XLOC_208405	6223 coding	coding	coding	coding
MSTRG.199886.1	MYOM1	XLOC_208439	241 coding	noncoding	noncoding	noncoding
MSTRG.199829.1		XLOC_208447	344 coding	noncoding	noncoding	noncoding
XM_024451288.1	DLGAP1	XLOC_208456	7285 coding	coding	coding	coding
MSTRG.199951.1	DLGAP1	XLOC_208458	282 coding	noncoding	noncoding	noncoding
MSTRG.199955.1	DLGAP1	XLOC_208461	274 coding	noncoding	noncoding	noncoding
MSTRG.199965.1	DLGAP1	XLOC_208465	275 coding	noncoding	noncoding	noncoding
MSTRG.200001.1	DLGAP1-AS4	XLOC_208480	269 coding	noncoding	noncoding	noncoding
MSTRG.200017.1	DLGAP1	XLOC_208487	249 coding	noncoding	noncoding	noncoding
MSTRG.199908.1		XLOC_208491	272 coding	noncoding	noncoding	noncoding
MSTRG.199909.1		XLOC_208492	237 coding	noncoding	noncoding	noncoding
MSTRG.199919.1		XLOC_208496	269 coding	coding	noncoding	noncoding
MSTRG.199920.1		XLOC_208497	368 coding	noncoding	noncoding	noncoding
NR_026849.1	LINC00526	XLOC_208501	1306 coding	noncoding	coding	noncoding
MSTRG.200046.1	EPB41L3	XLOC_208518	303 coding	noncoding	noncoding	noncoding
XM_011525729.3	TMEM200C	XLOC_208526	15766 coding	coding	coding	coding
MSTRG.200161.1	L3MBTL4	XLOC_208536	253 coding	noncoding	noncoding	noncoding
MSTRG.200052.1		XLOC_208543	297 coding	noncoding	noncoding	noncoding
MSTRG.200070.1	LOC107985176	XLOC_208551	417 coding	noncoding	noncoding	noncoding
MSTRG.200083.1	ARHGAP28	XLOC_208557	301 coding	noncoding	noncoding	noncoding
MSTRG.200084.1	ARHGAP28	XLOC_208558	228 coding	noncoding	noncoding	noncoding
MSTRG.200090.1	ARHGAP28	XLOC_208561	219 coding	noncoding	noncoding	noncoding
MSTRG.200091.1		XLOC_208568	256 coding	noncoding	noncoding	noncoding
MSTRG.200095.1		XLOC_208569	228 coding	noncoding	noncoding	noncoding
MSTRG.200096.1	LOC105371973	XLOC_208571	288 coding	noncoding	noncoding	noncoding

MSTRG.200115.1		XLOC_208578	310 coding	noncoding	noncoding	noncoding
MSTRG.200207.1		XLOC_208598	361 coding	noncoding	noncoding	noncoding
MSTRG.200209.4	RAB12	XLOC_208599	2483 coding	coding	noncoding	noncoding
MSTRG.200205.1		XLOC_208601	317 coding	noncoding	noncoding	noncoding
MSTRG.200217.1		XLOC_208609	221 coding	noncoding	noncoding	noncoding
MSTRG.200303.1		XLOC_208632	422 coding	noncoding	noncoding	noncoding
XM_011525723.3	PIEZO2	XLOC_208685	10678 coding	coding	noncoding	coding
MSTRG.200481.1	PIEZO2	XLOC_208694	302 coding	noncoding	noncoding	noncoding
MSTRG.200483.1	PIEZO2	XLOC_208696	209 coding	noncoding	noncoding	noncoding
MSTRG.200487.1	PIEZO2	XLOC_208698	221 coding	noncoding	noncoding	noncoding
MSTRG.200489.1	PIEZO2	XLOC_208699	323 coding	noncoding	noncoding	noncoding
MSTRG.200491.1	PIEZO2	XLOC_208700	259 coding	noncoding	noncoding	noncoding
MSTRG.200444.1		XLOC_208703	252 coding	noncoding	noncoding	noncoding
MSTRG.200451.1		XLOC_208711	231 coding	noncoding	noncoding	noncoding
MSTRG.200541.1	GNAL	XLOC_208721	210 coding	noncoding	noncoding	noncoding
MSTRG.200508.1		XLOC_208735	368 coding	noncoding	noncoding	noncoding
MSTRG.200510.1		XLOC_208737	242 coding	noncoding	noncoding	noncoding
MSTRG.200516.1	ANKRD62	XLOC_208741	318 coding	noncoding	noncoding	noncoding
MSTRG.200518.1	ANKRD62	XLOC_208743	244 coding	noncoding	noncoding	noncoding
XM_024451224.1	SPIRE1	XLOC_208746	5654 coding	coding	coding	coding
MSTRG.200579.1	SPIRE1	XLOC_208748	245 coding	noncoding	noncoding	noncoding
MSTRG.200586.1	SPIRE1	XLOC_208750	220 coding	noncoding	noncoding	noncoding
MSTRG.200592.1	SPIRE1	XLOC_208752	270 coding	noncoding	noncoding	noncoding
MSTRG.200558.1	LINC01882	XLOC_208761	331 coding	noncoding	noncoding	noncoding
MSTRG.200629.1		XLOC_208782	296 coding	noncoding	noncoding	noncoding
MSTRG.200706.1		XLOC_208826	337 coding	noncoding	noncoding	noncoding
NR_134878.1	POTEC	XLOC_208862	6571 coding	coding	coding	coding
MSTRG.200776.1		XLOC_208866	340 coding	noncoding	noncoding	noncoding
MSTRG.200835.1		XLOC_208925	308 coding	noncoding	noncoding	noncoding
MSTRG.200866.1		XLOC_208944	202 coding	noncoding	noncoding	noncoding
MSTRG.200966.1	LINC01900	XLOC_208951	337 coding	noncoding	noncoding	noncoding
MSTRG.200963.1		XLOC_208952	263 coding	noncoding	noncoding	noncoding
NM_172241.3	CTAGE1	XLOC_208964	4317 coding	noncoding	coding	coding
MSTRG.201032.1		XLOC_208976	296 coding	noncoding	noncoding	noncoding
MSTRG.201051.1	CABLES1	XLOC_208980	317 coding	noncoding	noncoding	noncoding
MSTRG.201080.2	RIOK3	XLOC_208989	3669 coding	coding	coding	noncoding
MSTRG.201108.1	LAMA3	XLOC_209000	244 coding	noncoding	noncoding	noncoding
MSTRG.201110.1	LAMA3	XLOC_209001	321 coding	noncoding	noncoding	noncoding
MSTRG.201138.1	CABYR	XLOC_209008	245 coding	noncoding	noncoding	noncoding
MSTRG.201150.1	OSBPL1A	XLOC_209014	299 coding	noncoding	noncoding	noncoding
MSTRG.201152.1	OSBPL1A	XLOC_209016	208 coding	noncoding	noncoding	noncoding
MSTRG.201188.1		XLOC_209033	310 coding	noncoding	noncoding	noncoding
MSTRG.201231.1	ZNF521	XLOC_209052	398 coding	noncoding	noncoding	noncoding

MSTRG.201206.1		XLOC_209057	290 coding	noncoding	noncoding	noncoding
MSTRG.201208.1		XLOC_209058	298 coding	noncoding	noncoding	noncoding
MSTRG.201211.1		XLOC_209061	279 coding	noncoding	noncoding	noncoding
MSTRG.201255.1		XLOC_209070	256 coding	noncoding	noncoding	noncoding
MSTRG.201371.3	TAF4B	XLOC_209091	480 coding	noncoding	noncoding	noncoding
MSTRG.201332.1	KCTD1	XLOC_209099	264 coding	noncoding	noncoding	noncoding
NM_031422.6	CHST9	XLOC_209112	11379 coding	coding	coding	coding
MSTRG.201348.1	CHST9	XLOC_209114	241 coding	noncoding	noncoding	noncoding
MSTRG.201310.1	LINC01908	XLOC_209118	230 coding	noncoding	noncoding	noncoding
MSTRG.201369.1		XLOC_209130	321 coding	noncoding	noncoding	noncoding
MSTRG.201387.1		XLOC_209140	213 coding	noncoding	noncoding	noncoding
MSTRG.201467.1		XLOC_209159	370 coding	noncoding	noncoding	noncoding
MSTRG.201468.1		XLOC_209160	259 coding	noncoding	noncoding	noncoding
MSTRG.201470.1		XLOC_209161	259 coding	noncoding	noncoding	noncoding
MSTRG.201479.1		XLOC_209167	488 coding	noncoding	noncoding	noncoding
MSTRG.201480.1		XLOC_209168	241 coding	noncoding	noncoding	noncoding
MSTRG.201481.1		XLOC_209169	246 coding	noncoding	noncoding	noncoding
MSTRG.201494.1		XLOC_209176	231 coding	noncoding	noncoding	noncoding
MSTRG.201508.1		XLOC_209191	252 coding	noncoding	noncoding	noncoding
MSTRG.201524.1	TTR	XLOC_209200	283 coding	noncoding	noncoding	noncoding
MSTRG.201565.1		XLOC_209230	268 coding	noncoding	noncoding	noncoding
MSTRG.201590.1		XLOC_209231	283 coding	noncoding	noncoding	noncoding
MSTRG.201632.1		XLOC_209251	255 coding	noncoding	noncoding	noncoding
MSTRG.201660.1	CCDC178	XLOC_209255	296 coding	noncoding	noncoding	noncoding
MSTRG.201693.1	LOC107985157	XLOC_209274	266 coding	noncoding	noncoding	noncoding
MSTRG.201712.1	NOL4	XLOC_209282	331 coding	coding	noncoding	noncoding
MSTRG.201697.1		XLOC_209283	934 coding	noncoding	noncoding	noncoding
MSTRG.201715.1		XLOC_209303	231 coding	noncoding	noncoding	noncoding
MSTRG.201833.4	GALNT1	XLOC_209328	612 coding	coding	noncoding	noncoding
MSTRG.201862.1	MOCOS	XLOC_209360	206 coding	noncoding	noncoding	noncoding
MSTRG.202286.1	FHOD3	XLOC_209366	295 coding	noncoding	noncoding	noncoding
MSTRG.202298.1	FHOD3	XLOC_209374	294 coding	noncoding	noncoding	noncoding
MSTRG.201901.1		XLOC_209416	215 coding	noncoding	noncoding	noncoding
MSTRG.201905.1		XLOC_209420	294 coding	noncoding	noncoding	noncoding
MSTRG.201907.1		XLOC_209421	201 coding	noncoding	noncoding	noncoding
MSTRG.201909.1		XLOC_209423	406 coding	noncoding	noncoding	noncoding
MSTRG.201949.1		XLOC_209448	262 coding	noncoding	noncoding	noncoding
MSTRG.201961.1		XLOC_209455	202 coding	coding	noncoding	noncoding
MSTRG.201963.1		XLOC_209457	381 coding	noncoding	noncoding	noncoding
MSTRG.201965.1		XLOC_209458	374 coding	noncoding	noncoding	noncoding
MSTRG.201995.1		XLOC_209459	433 coding	noncoding	noncoding	noncoding
MSTRG.201983.1		XLOC_209462	325 coding	noncoding	noncoding	noncoding
MSTRG.202025.1	MIR924HG	XLOC_209468	247 coding	noncoding	noncoding	noncoding

MSTRG.202044.1		XLOC_209489	275 coding	noncoding	noncoding	noncoding
MSTRG.202076.1		XLOC_209503	263 coding	noncoding	noncoding	noncoding
MSTRG.202265.1	LINC00907	XLOC_209565	303 coding	noncoding	noncoding	noncoding
MSTRG.202165.1	LINC01478	XLOC_209588	225 coding	noncoding	noncoding	noncoding
MSTRG.202344.1		XLOC_209604	226 coding	noncoding	noncoding	noncoding
MSTRG.202360.1	SLC14A2	XLOC_209612	309 coding	noncoding	noncoding	noncoding
MSTRG.202372.9	LOC105372093	XLOC_209620	1648 coding	coding	noncoding	noncoding
MSTRG.202377.1	SLC14A2	XLOC_209623	268 coding	noncoding	noncoding	noncoding
XM_017025890.2	EPG5	XLOC_209628	7944 coding	noncoding	coding	coding
MSTRG.202397.1	EPG5	XLOC_209630	277 coding	noncoding	noncoding	noncoding
MSTRG.202485.1	ST8SIA5	XLOC_209690	246 coding	noncoding	noncoding	noncoding
MSTRG.202498.1		XLOC_209691	295 coding	noncoding	noncoding	noncoding
NM_001324053.2	PIAS2	XLOC_209692	5005 coding	coding	coding	coding
XM_017026072.1	PIAS2	XLOC_209692	1785 coding	coding	coding	coding
MSTRG.202611.1	PIAS2	XLOC_209698	261 coding	noncoding	noncoding	noncoding
MSTRG.202616.1	KATNAL2	XLOC_209702	335 coding	noncoding	noncoding	noncoding
MSTRG.202541.1		XLOC_209719	264 coding	noncoding	noncoding	noncoding
MSTRG.202546.1	MIR4527HG	XLOC_209721	278 coding	noncoding	noncoding	noncoding
MSTRG.202652.1	ZBTB7C	XLOC_209760	368 coding	noncoding	noncoding	noncoding
MSTRG.202657.1		XLOC_209765	258 coding	noncoding	noncoding	noncoding
MSTRG.202691.1		XLOC_209775	249 coding	noncoding	noncoding	noncoding
MSTRG.202758.23	DYM	XLOC_209783	278 coding	coding	noncoding	noncoding
NM_000985.5	RPL17	XLOC_209830	863 coding	coding	coding	coding
MSTRG.202719.1	LIPG	XLOC_209834	271 coding	noncoding	noncoding	noncoding
MSTRG.202808.1		XLOC_209856	255 coding	noncoding	noncoding	noncoding
MSTRG.202817.1		XLOC_209861	430 coding	noncoding	noncoding	noncoding
MSTRG.202816.1		XLOC_209862	262 coding	noncoding	noncoding	noncoding
MSTRG.202942.1	LOC105372121	XLOC_209923	271 coding	noncoding	noncoding	noncoding
MSTRG.202990.1	DCC	XLOC_209941	282 coding	noncoding	noncoding	noncoding
MSTRG.203010.1		XLOC_209954	300 coding	noncoding	noncoding	noncoding
MSTRG.203025.1		XLOC_209974	298 coding	noncoding	noncoding	noncoding
MSTRG.203061.1		XLOC_209987	283 coding	noncoding	noncoding	noncoding
MSTRG.203094.1	CCDC68	XLOC_209998	229 coding	noncoding	noncoding	noncoding
MSTRG.203099.1	LINC01416	XLOC_210030	240 coding	coding	noncoding	noncoding
MSTRG.203100.1	LINC01416	XLOC_210031	278 coding	noncoding	noncoding	noncoding
MSTRG.203182.1		XLOC_210062	259 coding	noncoding	noncoding	noncoding
MSTRG.203190.1		XLOC_210064	261 coding	noncoding	noncoding	noncoding
NM_000140.4	FECH	XLOC_210065	7677 coding	coding	coding	coding
MSTRG.203278.1	FECH	XLOC_210066	253 coding	noncoding	noncoding	noncoding
MSTRG.203283.1		XLOC_210068	272 coding	noncoding	noncoding	noncoding
MSTRG.203305.1	ATP8B1	XLOC_210079	288 coding	noncoding	noncoding	noncoding
MSTRG.203205.1		XLOC_210105	258 coding	noncoding	noncoding	noncoding
MSTRG.203275.1		XLOC_210109	206 coding	noncoding	noncoding	noncoding

MSTRG.203336.2	ZNF532	XLOC_210110	1108 coding	noncoding	noncoding	noncoding
MSTRG.203337.1	ZNF532	XLOC_210111	289 coding	noncoding	noncoding	noncoding
MSTRG.203341.1	ZNF532	XLOC_210115	308 coding	noncoding	noncoding	noncoding
MSTRG.203358.1		XLOC_210127	298 coding	noncoding	noncoding	noncoding
MSTRG.203379.1	CCBE1	XLOC_210130	311 coding	noncoding	noncoding	noncoding
MSTRG.203403.1		XLOC_210143	318 coding	noncoding	noncoding	noncoding
MSTRG.203411.1		XLOC_210147	311 coding	noncoding	noncoding	noncoding
MSTRG.203426.1		XLOC_210153	443 coding	noncoding	noncoding	noncoding
MSTRG.203427.1		XLOC_210154	286 coding	noncoding	noncoding	noncoding
MSTRG.203457.1		XLOC_210169	259 coding	noncoding	noncoding	noncoding
MSTRG.203464.1	CDH20	XLOC_210173	450 coding	noncoding	noncoding	noncoding
MSTRG.203468.1	CDH20	XLOC_210176	304 coding	noncoding	noncoding	noncoding
MSTRG.203624.1	PIGN	XLOC_210201	279 coding	noncoding	noncoding	noncoding
MSTRG.203560.7	ZCCHC2	XLOC_210223	4709 coding	coding	coding	noncoding
MSTRG.203518.1		XLOC_210226	300 coding	noncoding	noncoding	noncoding
MSTRG.203519.1		XLOC_210227	272 coding	noncoding	noncoding	noncoding
MSTRG.203523.1		XLOC_210229	235 coding	noncoding	noncoding	noncoding
MSTRG.203527.1		XLOC_210265	272 coding	noncoding	noncoding	noncoding
XM_017025690.2	KDSR	XLOC_210324	5402 coding	coding	coding	coding
MSTRG.203675.5	VPS4B	XLOC_210329	1706 coding	coding	coding	coding
MSTRG.203681.1	SERPINB12	XLOC_210333	246 coding	noncoding	noncoding	noncoding
MSTRG.203697.1		XLOC_210340	279 coding	noncoding	noncoding	noncoding
MSTRG.203743.1	HMSD	XLOC_210346	331 coding	noncoding	noncoding	noncoding
MSTRG.203772.1	LOC101927404	XLOC_210378	296 coding	noncoding	noncoding	noncoding
MSTRG.203779.1	LOC105372168	XLOC_210384	205 coding	noncoding	noncoding	noncoding
MSTRG.203782.1		XLOC_210385	308 coding	noncoding	noncoding	noncoding
MSTRG.203852.1		XLOC_210421	289 coding	noncoding	noncoding	noncoding
MSTRG.203882.1	LOC643542	XLOC_210433	287 coding	noncoding	noncoding	noncoding
MSTRG.204089.1	CD226	XLOC_210501	12132 coding	coding	coding	coding
MSTRG.204261.1	RTTN	XLOC_210515	276 coding	noncoding	noncoding	noncoding
MSTRG.204015.1		XLOC_210536	260 coding	noncoding	noncoding	noncoding
MSTRG.204016.1		XLOC_210537	229 coding	noncoding	noncoding	noncoding
MSTRG.204022.1		XLOC_210539	284 coding	noncoding	noncoding	noncoding
MSTRG.204178.1	LOC107985179	XLOC_210599	285 coding	noncoding	noncoding	noncoding
MSTRG.204194.1		XLOC_210606	281 coding	noncoding	noncoding	noncoding
XM_017025559.1	CBLN2	XLOC_210616	6705 coding	coding	coding	coding
MSTRG.204227.1	CBLN2	XLOC_210618	232 coding	noncoding	noncoding	noncoding
MSTRG.204221.1		XLOC_210620	372 coding	noncoding	noncoding	noncoding
MSTRG.204302.1	NETO1	XLOC_210622	296 coding	noncoding	noncoding	noncoding
MSTRG.204279.1	LOC400655	XLOC_210627	378 coding	noncoding	noncoding	noncoding
MSTRG.204285.1		XLOC_210628	231 coding	noncoding	noncoding	noncoding
MSTRG.204313.1		XLOC_210635	445 coding	noncoding	noncoding	noncoding
MSTRG.204318.1		XLOC_210636	263 coding	noncoding	noncoding	noncoding

MSTRG.204340.1		XLOC_210642	313	coding	noncoding	noncoding	noncoding
MSTRG.204370.1	C18orf63	XLOC_210657	287	coding	noncoding	noncoding	noncoding
MSTRG.204388.1	CNDP1	XLOC_210668	243	coding	noncoding	noncoding	noncoding
MSTRG.204391.1	CNDP1	XLOC_210671	307	coding	noncoding	noncoding	noncoding
MSTRG.204402.1		XLOC_210685	300	coding	noncoding	noncoding	noncoding
NM_001037331.3	SMIM21	XLOC_210693	1834	coding	noncoding	noncoding	noncoding
XM_011525928.1	SMIM21	XLOC_210693	8885	coding	noncoding	coding	noncoding
MSTRG.204439.1		XLOC_210711	206	coding	noncoding	noncoding	noncoding
MSTRG.204731.1	MBP	XLOC_210776	351	coding	noncoding	noncoding	noncoding
XM_017025780.2	MBP	XLOC_210777	4834	coding	coding	coding	coding
MSTRG.204559.1		XLOC_210801	283	coding	noncoding	noncoding	noncoding
MSTRG.204652.1		XLOC_210806	241	coding	noncoding	noncoding	noncoding
MSTRG.204653.1		XLOC_210809	201	coding	noncoding	noncoding	noncoding
MSTRG.204684.1		XLOC_210825	232	coding	noncoding	noncoding	noncoding
MSTRG.204862.7	ATP9B	XLOC_210841	1895	coding	coding	noncoding	noncoding
XR_001753515.1	LOC101927897	XLOC_210852	8202	coding	noncoding	coding	coding
MSTRG.204765.1		XLOC_210860	250	coding	noncoding	noncoding	noncoding
MSTRG.204858.1	PARD6G	XLOC_210898	264	coding	noncoding	noncoding	noncoding
MSTRG.204859.1	PARD6G	XLOC_210899	264	coding	noncoding	noncoding	noncoding
MSTRG.204887.2	LOC101928344	XLOC_210908	5931	coding	coding	coding	noncoding
MSTRG.204947.1	HCN2	XLOC_210944	399	coding	noncoding	noncoding	noncoding
MSTRG.204961.1		XLOC_210955	255	coding	noncoding	noncoding	noncoding
XR_001753829.2	LINC01836	XLOC_210959	954	coding	noncoding	noncoding	noncoding
XM_005259513.5	ARID3A	XLOC_210973	6796	coding	coding	coding	coding
NM_001303499.2	CNN2	XLOC_210980	2116	coding	coding	coding	coding
MSTRG.205046.1	ABCA7	XLOC_210982	231	coding	noncoding	noncoding	noncoding
MSTRG.205000.1	STK11	XLOC_210990	234	coding	noncoding	noncoding	noncoding
NR_023313.2	CIRBP	XLOC_210997	3095	coding	coding	coding	coding
NM_001369792.1	PWWP3A	XLOC_211003	4513	coding	noncoding	coding	coding
MSTRG.205078.1		XLOC_211013	325	coding	noncoding	noncoding	noncoding
MSTRG.205081.1	ADAMTSL5	XLOC_211017	216	coding	noncoding	noncoding	noncoding
MSTRG.205073.2	UQCR11	XLOC_211021	440	coding	coding	noncoding	noncoding
MSTRG.205099.2	LOC100288123	XLOC_211030	1785	coding	noncoding	coding	noncoding
MSTRG.205124.1		XLOC_211037	255	coding	noncoding	noncoding	noncoding
NM_138422.4	ADAT3	XLOC_211038	1599	coding	coding	coding	coding
XM_005259501.2	CSNK1G2	XLOC_211040	3367	coding	coding	coding	coding
MSTRG.205179.8	MOB3A	XLOC_211050	3300	coding	coding	coding	coding
MSTRG.205148.1		XLOC_211054	259	coding	noncoding	noncoding	noncoding
MSTRG.205189.1		XLOC_211071	271	coding	noncoding	noncoding	noncoding
MSTRG.205202.1	TMPRSS9	XLOC_211074	277	coding	noncoding	noncoding	noncoding
MSTRG.205198.1		XLOC_211078	345	coding	noncoding	noncoding	noncoding
MSTRG.205211.1		XLOC_211080	286	coding	noncoding	noncoding	noncoding
MSTRG.205215.1		XLOC_211081	232	coding	noncoding	noncoding	noncoding

MSTRG.205230.1	GNG7	XLOC_211088	258	coding	noncoding	noncoding	noncoding
XM_011528228.2	THOP1	XLOC_211111	3545	coding	coding	coding	coding
MSTRG.205263.1	ZNF554	XLOC_211114	269	coding	noncoding	noncoding	noncoding
MSTRG.205301.4	TLE5	XLOC_211132	1680	coding	coding	coding	noncoding
MSTRG.205301.3	TLE5	XLOC_211132	1796	coding	coding	coding	noncoding
NM_020170.4	NCLN	XLOC_211149	3680	coding	coding	coding	coding
MSTRG.205331.1	NCLN	XLOC_211152	282	coding	noncoding	noncoding	noncoding
NM_001172673.2	CELF5	XLOC_211153	5051	coding	coding	coding	coding
MSTRG.205342.1	CELF5	XLOC_211158	263	coding	noncoding	noncoding	noncoding
XM_017026834.2	NFIC	XLOC_211161	2088	coding	coding	coding	coding
MSTRG.205374.1	FZR1	XLOC_211172	268	coding	noncoding	noncoding	noncoding
MSTRG.205388.1	CACTIN	XLOC_211181	285	coding	noncoding	noncoding	noncoding
MSTRG.205413.1	MATK	XLOC_211192	222	coding	noncoding	noncoding	noncoding
MSTRG.205419.1	ZFR2	XLOC_211193	256	coding	noncoding	noncoding	noncoding
NM_033064.5	ATCAY	XLOC_211196	4971	coding	coding	coding	coding
MSTRG.205416.1	ATCAY	XLOC_211197	314	coding	noncoding	noncoding	noncoding
MSTRG.205446.1		XLOC_211211	300	coding	noncoding	noncoding	noncoding
MSTRG.205455.1	EBI3	XLOC_211217	274	coding	noncoding	noncoding	noncoding
MSTRG.205466.1	YJU2	XLOC_211219	279	coding	noncoding	noncoding	noncoding
MSTRG.205463.1	SHD	XLOC_211221	232	coding	noncoding	noncoding	noncoding
MSTRG.205472.1		XLOC_211232	295	coding	noncoding	noncoding	noncoding
MSTRG.205502.1	PLIN4	XLOC_211237	793	coding	noncoding	coding	noncoding
XM_005259487.3	TNFAIP8L1	XLOC_211242	4493	coding	coding	coding	coding
MSTRG.205584.1	PTPRS	XLOC_211264	487	coding	coding	coding	noncoding
MSTRG.205593.1	PTPRS	XLOC_211271	267	coding	noncoding	noncoding	noncoding
MSTRG.205551.1		XLOC_211274	296	coding	noncoding	noncoding	noncoding
MSTRG.205553.1		XLOC_211275	235	coding	noncoding	noncoding	noncoding
MSTRG.205557.1		XLOC_211278	303	coding	noncoding	noncoding	noncoding
MSTRG.205636.1	CATSPERD	XLOC_211301	321	coding	noncoding	noncoding	noncoding
MSTRG.205637.1	CATSPERD	XLOC_211302	307	coding	noncoding	noncoding	noncoding
MSTRG.205638.1	CATSPERD	XLOC_211303	300	coding	noncoding	noncoding	noncoding
MSTRG.205605.1	FUT6	XLOC_211307	298	coding	noncoding	noncoding	noncoding
MSTRG.205720.1	VMAC	XLOC_211314	251	coding	noncoding	noncoding	noncoding
MSTRG.205737.1	RFX2	XLOC_211322	898	coding	noncoding	coding	noncoding
MSTRG.205751.1	RFX2	XLOC_211334	475	coding	noncoding	noncoding	noncoding
MSTRG.205655.1	MLLT1	XLOC_211348	429	coding	noncoding	noncoding	noncoding
MSTRG.205669.1	ACER1	XLOC_211360	369	coding	coding	noncoding	noncoding
MSTRG.205693.1	TUBB4A	XLOC_211371	250	coding	noncoding	noncoding	noncoding
MSTRG.205689.1		XLOC_211372	270	coding	noncoding	noncoding	noncoding
MSTRG.205691.1		XLOC_211373	281	coding	noncoding	noncoding	noncoding
MSTRG.205714.1	C3	XLOC_211384	202	coding	noncoding	noncoding	noncoding
MSTRG.205716.1	C3	XLOC_211385	292	coding	noncoding	noncoding	noncoding
MSTRG.205782.1	VAV1	XLOC_211392	290	coding	noncoding	noncoding	noncoding

MSTRG.205783.1	VAV1		XLOC_211393	333	coding	noncoding	noncoding	noncoding
MSTRG.205785.1	VAV1		XLOC_211395	375	coding	noncoding	noncoding	noncoding
MSTRG.205778.1			XLOC_211398	253	coding	noncoding	noncoding	noncoding
MSTRG.205792.1			XLOC_211406	285	coding	noncoding	noncoding	noncoding
MSTRG.205803.1			XLOC_211410	414	coding	noncoding	noncoding	noncoding
NM_001367823.1	ARHGEF18		XLOC_211413	6681	coding	coding	coding	coding
MSTRG.205824.1	ARHGEF18		XLOC_211416	240	coding	noncoding	noncoding	noncoding
MSTRG.205827.1	ARHGEF18		XLOC_211419	207	coding	noncoding	noncoding	noncoding
MSTRG.205831.1	ARHGEF18		XLOC_211421	275	coding	noncoding	noncoding	noncoding
NM_001166111.2	PNPLA6		XLOC_211425	4536	coding	coding	coding	coding
MSTRG.205816.1			XLOC_211426	250	coding	noncoding	noncoding	noncoding
NR_002931.2	CLEC4GP1		XLOC_211443	1935	coding	noncoding	coding	coding
MSTRG.205890.1			XLOC_211457	299	coding	noncoding	noncoding	noncoding
MSTRG.205899.1	FBN3		XLOC_211459	299	coding	noncoding	noncoding	noncoding
MSTRG.205906.1	FBN3		XLOC_211464	307	coding	noncoding	noncoding	noncoding
MSTRG.205914.1			XLOC_211465	294	coding	noncoding	noncoding	noncoding
MSTRG.205918.1	CERS4		XLOC_211468	302	coding	noncoding	noncoding	noncoding
MSTRG.205919.1	CERS4		XLOC_211469	264	coding	noncoding	noncoding	noncoding
MSTRG.205925.1			XLOC_211481	253	coding	noncoding	noncoding	noncoding
MSTRG.205941.1	RAB11B		XLOC_211486	224	coding	noncoding	noncoding	noncoding
MSTRG.205942.1	RAB11B		XLOC_211487	294	coding	noncoding	noncoding	noncoding
NM_001369776.1		2-Mar	XLOC_211488	2025	coding	coding	coding	coding
MSTRG.205946.1			XLOC_211500	254	coding	noncoding	noncoding	noncoding
MSTRG.205965.1			XLOC_211510	292	coding	noncoding	noncoding	noncoding
MSTRG.205980.1	ACTL9		XLOC_211513	244	coding	coding	noncoding	noncoding
MSTRG.206009.1	MUC16		XLOC_211521	237	coding	noncoding	noncoding	noncoding
MSTRG.206013.1	MUC16		XLOC_211524	283	coding	noncoding	noncoding	noncoding
MSTRG.206005.1			XLOC_211529	240	coding	noncoding	noncoding	noncoding
MSTRG.206006.1			XLOC_211531	311	coding	noncoding	noncoding	noncoding
MSTRG.206024.1			XLOC_211539	249	coding	noncoding	noncoding	noncoding
MSTRG.206019.1			XLOC_211543	267	coding	noncoding	noncoding	noncoding
MSTRG.206071.1			XLOC_211571	280	coding	noncoding	noncoding	noncoding
MSTRG.206122.1	OLFM2		XLOC_211588	269	coding	noncoding	noncoding	noncoding
MSTRG.206130.1	COL5A3		XLOC_211591	260	coding	noncoding	noncoding	noncoding
MSTRG.206160.1			XLOC_211603	342	coding	noncoding	noncoding	noncoding
MSTRG.206135.1	MIR4322		XLOC_211608	274	coding	noncoding	noncoding	noncoding
MSTRG.206143.1	MRPL4		XLOC_211610	229	coding	noncoding	noncoding	noncoding
MSTRG.206153.1	ICAM1		XLOC_211614	293	coding	noncoding	noncoding	noncoding
MSTRG.206198.1			XLOC_211619	259	coding	noncoding	noncoding	noncoding
MSTRG.206200.1			XLOC_211620	227	coding	noncoding	noncoding	noncoding
MSTRG.206203.5	TYK2		XLOC_211622	4178	coding	coding	coding	coding
MSTRG.206178.1	PDE4A		XLOC_211626	307	coding	noncoding	noncoding	noncoding
MSTRG.206173.1			XLOC_211627	275	coding	noncoding	noncoding	noncoding

MSTRG.206175.1		XLOC_211628	224 coding	noncoding	noncoding	noncoding
MSTRG.206187.1		XLOC_211632	279 coding	noncoding	noncoding	noncoding
MSTRG.206233.1	DNM2	XLOC_211646	317 coding	noncoding	noncoding	noncoding
MSTRG.206234.1	DNM2	XLOC_211647	237 coding	noncoding	noncoding	noncoding
MSTRG.206238.1	DNM2	XLOC_211651	291 coding	noncoding	noncoding	noncoding
MSTRG.206248.1		XLOC_211662	273 coding	noncoding	noncoding	noncoding
MSTRG.206253.1		XLOC_211665	238 coding	noncoding	noncoding	noncoding
MSTRG.206254.1		XLOC_211666	275 coding	noncoding	noncoding	noncoding
NM_000527.4	LDLR	XLOC_211667	5284 coding	coding	coding	coding
MSTRG.206271.1		XLOC_211670	269 coding	noncoding	noncoding	noncoding
MSTRG.206316.1	RGL3	XLOC_211683	308 coding	noncoding	noncoding	noncoding
MSTRG.206321.1	ELAVL3	XLOC_211686	208 coding	noncoding	noncoding	noncoding
MSTRG.206308.4	ELOF1	XLOC_211693	1060 coding	coding	noncoding	noncoding
MSTRG.206308.3	ELOF1	XLOC_211693	1054 coding	coding	noncoding	noncoding
MSTRG.206307.1		XLOC_211696	255 coding	noncoding	noncoding	noncoding
XM_011527780.2	ZNF627	XLOC_211698	2728 coding	coding	coding	coding
MSTRG.206310.1		XLOC_211701	206 coding	noncoding	noncoding	noncoding
MSTRG.206341.1		XLOC_211704	294 coding	noncoding	noncoding	noncoding
MSTRG.206350.1	ZNF441	XLOC_211709	312 coding	noncoding	noncoding	noncoding
XM_005259730.4	ZNF491	XLOC_211710	3828 coding	coding	coding	coding
MSTRG.206392.1		XLOC_211716	275 coding	noncoding	noncoding	noncoding
NM_001012753.2	ZNF763	XLOC_211717	2871 coding	coding	coding	coding
MSTRG.206412.1	ZNF69	XLOC_211725	269 coding	noncoding	noncoding	noncoding
MSTRG.206359.1		XLOC_211732	319 coding	noncoding	noncoding	noncoding
MSTRG.206375.1	LOC105372275	XLOC_211737	249 coding	noncoding	noncoding	noncoding
MSTRG.206436.1	ZNF136	XLOC_211740	321 coding	noncoding	noncoding	noncoding
MSTRG.206380.1		XLOC_211747	291 coding	noncoding	noncoding	noncoding
MSTRG.206454.1	ZNF563	XLOC_211752	420 coding	noncoding	noncoding	noncoding
MSTRG.206456.1	ZNF442	XLOC_211754	277 coding	noncoding	noncoding	noncoding
MSTRG.206457.1	ZNF442	XLOC_211755	242 coding	noncoding	noncoding	noncoding
MSTRG.206473.1	LOC105372278	XLOC_211770	318 coding	noncoding	noncoding	noncoding
XM_017026412.1	ZNF791	XLOC_211774	4524 coding	coding	coding	coding
MSTRG.206508.1	GET3	XLOC_211785	220 coding	noncoding	noncoding	noncoding
MSTRG.206549.1	GADD45GIP1	XLOC_211809	250 coding	noncoding	noncoding	noncoding
MSTRG.206566.1	CACNA1A	XLOC_211822	254 coding	noncoding	noncoding	noncoding
NR_138051.1	MRI1	XLOC_211834	3211 coding	coding	coding	coding
NR_134916.1	LINC01842	XLOC_211858	765 coding	noncoding	noncoding	noncoding
MSTRG.206657.1	DNAJB1	XLOC_211866	339 coding	noncoding	noncoding	noncoding
MSTRG.206701.23	ADGRE3	XLOC_211871	3077 coding	coding	coding	coding
MSTRG.206663.1		XLOC_211874	248 coding	noncoding	noncoding	noncoding
MSTRG.206666.1	OR7A5	XLOC_211875	304 coding	noncoding	noncoding	noncoding
MSTRG.206664.1		XLOC_211876	238 coding	noncoding	noncoding	noncoding
MSTRG.206670.1	OR7A17	XLOC_211877	236 coding	coding	noncoding	noncoding

MSTRG.206682.1		XLOC_211886	282 coding	noncoding	noncoding	noncoding
MSTRG.206695.2	BRD4	XLOC_211887	2220 coding	coding	coding	coding
MSTRG.206738.1	CYP4F22	XLOC_211905	225 coding	noncoding	noncoding	noncoding
MSTRG.206745.1		XLOC_211910	293 coding	noncoding	noncoding	noncoding
MSTRG.206753.1		XLOC_211922	242 coding	noncoding	noncoding	noncoding
MSTRG.206773.1	CYP4F2	XLOC_211933	258 coding	noncoding	noncoding	noncoding
NR_026828.1	LINC00661	XLOC_211936	3342 coding	noncoding	coding	coding
MSTRG.206777.1		XLOC_211939	328 coding	noncoding	noncoding	noncoding
NM_001367837.2	TPM4	XLOC_211942	2216 coding	coding	coding	coding
NR_163150.1	HSH2D	XLOC_211945	2904 coding	noncoding	coding	coding
MSTRG.206816.1	RAB8A	XLOC_211947	220 coding	noncoding	noncoding	noncoding
MSTRG.206820.1	HSH2D	XLOC_211948	267 coding	noncoding	noncoding	noncoding
MSTRG.206810.1	CIB3	XLOC_211950	239 coding	noncoding	noncoding	noncoding
NM_001130524.2	AP1M1	XLOC_211952	12895 coding	coding	coding	coding
MSTRG.206830.1	AP1M1	XLOC_211953	282 coding	noncoding	noncoding	noncoding
MSTRG.206824.1		XLOC_211956	250 coding	noncoding	noncoding	noncoding
MSTRG.206826.1		XLOC_211957	643 coding	noncoding	noncoding	noncoding
MSTRG.206825.1		XLOC_211958	272 coding	noncoding	noncoding	noncoding
MSTRG.206869.1		XLOC_211993	283 coding	noncoding	noncoding	noncoding
MSTRG.206870.1		XLOC_211994	235 coding	noncoding	noncoding	noncoding
MSTRG.206923.1	CPAMD8	XLOC_212003	240 coding	noncoding	noncoding	noncoding
MSTRG.207001.1	LOC100507551	XLOC_212042	227 coding	noncoding	noncoding	noncoding
MSTRG.206984.1		XLOC_212046	283 coding	noncoding	noncoding	noncoding
MSTRG.207005.1		XLOC_212053	337 coding	noncoding	noncoding	noncoding
MSTRG.207014.1		XLOC_212060	302 coding	noncoding	noncoding	noncoding
MSTRG.207029.1	KCNN1	XLOC_212063	262 coding	noncoding	noncoding	noncoding
MSTRG.207078.5	IL12RB1	XLOC_212069	817 coding	noncoding	noncoding	noncoding
XM_017026511.1	MAST3	XLOC_212071	6361 coding	coding	coding	coding
MSTRG.207086.1	MAST3	XLOC_212073	294 coding	noncoding	noncoding	noncoding
MSTRG.207052.1	PDE4C	XLOC_212079	277 coding	noncoding	noncoding	noncoding
MSTRG.207053.1	PDE4C	XLOC_212080	253 coding	noncoding	noncoding	noncoding
MSTRG.207060.1		XLOC_212087	218 coding	noncoding	noncoding	noncoding
XM_017026432.1	SSBP4	XLOC_212093	2783 coding	coding	coding	coding
MSTRG.207100.4	FKBP8	XLOC_212097	1707 coding	coding	coding	coding
MSTRG.207100.3	FKBP8	XLOC_212097	1792 coding	coding	coding	noncoding
XM_011528273.2	KXD1	XLOC_212098	1660 coding	coding	coding	coding
MSTRG.207125.1		XLOC_212107	233 coding	noncoding	noncoding	noncoding
MSTRG.207131.1		XLOC_212114	235 coding	noncoding	noncoding	noncoding
XM_017027105.2	UPF1	XLOC_212116	5932 coding	coding	coding	coding
MSTRG.207153.1		XLOC_212124	236 coding	noncoding	noncoding	noncoding
MSTRG.207235.1	GATAD2A	XLOC_212151	402 coding	noncoding	noncoding	noncoding
MSTRG.207236.1	GATAD2A	XLOC_212152	294 coding	noncoding	noncoding	noncoding
MSTRG.207237.1	GATAD2A	XLOC_212153	267 coding	noncoding	noncoding	noncoding

XM_005259903.4	YJEFN3	XLOC_212162	1364	coding	coding	coding	coding
MSTRG.207258.6	GMIP	XLOC_212179	4971	coding	coding	coding	coding
MSTRG.207282.1	LINC00663	XLOC_212187	283	coding	noncoding	noncoding	noncoding
MSTRG.207294.1		XLOC_212191	233	coding	noncoding	noncoding	noncoding
MSTRG.207269.1		XLOC_212196	276	coding	noncoding	noncoding	noncoding
MSTRG.207277.1		XLOC_212213	256	coding	noncoding	noncoding	noncoding
MSTRG.207337.1	LOC105372310	XLOC_212221	208	coding	noncoding	noncoding	noncoding
MSTRG.207350.1	LOC105372310	XLOC_212229	220	coding	noncoding	noncoding	noncoding
XR_936407.2	LOC105372314	XLOC_212231	2830	coding	coding	coding	noncoding
MSTRG.207380.1		XLOC_212245	230	coding	noncoding	noncoding	noncoding
MSTRG.207394.1		XLOC_212253	270	coding	noncoding	noncoding	noncoding
XR_001753620.1	ZNF431	XLOC_212277	5011	coding	coding	coding	coding
MSTRG.207562.1		XLOC_212281	244	coding	noncoding	noncoding	noncoding
MSTRG.207594.1		XLOC_212298	270	coding	noncoding	noncoding	noncoding
MSTRG.207419.1		XLOC_212304	309	coding	noncoding	noncoding	noncoding
XM_017026752.1	ZNF429	XLOC_212306	5297	coding	coding	coding	coding
MSTRG.207499.1	ZNF257	XLOC_212340	281	coding	noncoding	noncoding	noncoding
NM_001242680.2	ZNF729	XLOC_212343	3844	coding	coding	coding	coding
MSTRG.207527.1		XLOC_212353	240	coding	noncoding	noncoding	noncoding
MSTRG.207529.1		XLOC_212354	282	coding	noncoding	noncoding	noncoding
XM_017026115.2	ZNF730	XLOC_212360	3116	coding	coding	coding	coding
MSTRG.207550.1	ZNF730	XLOC_212362	281	coding	noncoding	noncoding	noncoding
MSTRG.207640.1		XLOC_212367	283	coding	noncoding	noncoding	noncoding
MSTRG.207649.9	ZNF91	XLOC_212374	3837	coding	coding	noncoding	noncoding
MSTRG.207649.10	ZNF91	XLOC_212374	3598	coding	coding	noncoding	noncoding
MSTRG.207600.1		XLOC_212380	235	coding	noncoding	noncoding	noncoding
MSTRG.207706.1	RPSAP58	XLOC_212391	213	coding	noncoding	noncoding	noncoding
MSTRG.207714.1		XLOC_212398	423	coding	noncoding	noncoding	noncoding
MSTRG.207716.1		XLOC_212400	230	coding	coding	noncoding	noncoding
MSTRG.207776.1	LOC100420587	XLOC_212462	232	coding	noncoding	noncoding	noncoding
MSTRG.207830.1		XLOC_212485	285	coding	noncoding	noncoding	noncoding
MSTRG.207855.1	LOC284395	XLOC_212502	299	coding	noncoding	noncoding	noncoding
MSTRG.207865.1	PLEKHF1	XLOC_212508	239	coding	noncoding	noncoding	noncoding
MSTRG.207874.1		XLOC_212514	269	coding	noncoding	noncoding	noncoding
MSTRG.207881.1		XLOC_212517	266	coding	noncoding	noncoding	noncoding
MSTRG.207897.1		XLOC_212541	200	coding	noncoding	noncoding	noncoding
MSTRG.207902.1		XLOC_212555	308	coding	noncoding	noncoding	noncoding
MSTRG.207915.1	LOC105372356	XLOC_212564	445	coding	noncoding	noncoding	noncoding
MSTRG.207911.1	LOC105372358	XLOC_212565	434	coding	noncoding	noncoding	noncoding
MSTRG.207961.1		XLOC_212593	280	coding	noncoding	noncoding	noncoding
MSTRG.207978.1		XLOC_212609	315	coding	noncoding	noncoding	noncoding
MSTRG.208076.1	SLC7A9	XLOC_212637	292	coding	noncoding	noncoding	noncoding
MSTRG.208087.1	CEP89	XLOC_212640	279	coding	noncoding	noncoding	noncoding

MSTRG.208094.1	CEP89	XLOC_212646	220 coding	noncoding	noncoding	noncoding
MSTRG.208105.1	RHPN2	XLOC_212652	303 coding	noncoding	noncoding	noncoding
MSTRG.208129.1		XLOC_212671	242 coding	coding	noncoding	noncoding
MSTRG.208134.1		XLOC_212674	313 coding	noncoding	noncoding	noncoding
MSTRG.208158.1		XLOC_212685	210 coding	noncoding	noncoding	noncoding
NM_001289181.1	ZNF302	XLOC_212734	2778 coding	coding	coding	coding
MSTRG.208202.1		XLOC_212737	235 coding	coding	noncoding	noncoding
NR_024018.1	ZNF30	XLOC_212757	2664 coding	coding	coding	coding
XM_011527155.1	GRAMD1A	XLOC_212763	2915 coding	coding	coding	coding
MSTRG.208287.1	GRAMD1A	XLOC_212765	278 coding	noncoding	noncoding	noncoding
NM_199037.5	SCN1B	XLOC_212766	4780 coding	coding	coding	coding
NM_005303.2	FFAR1	XLOC_212789	923 coding	coding	coding	coding
XM_017026711.1	FFAR2	XLOC_212803	2227 coding	coding	coding	coding
MSTRG.208332.1		XLOC_212806	298 coding	noncoding	noncoding	noncoding
MSTRG.208346.1	SBSN	XLOC_212808	269 coding	noncoding	noncoding	noncoding
MSTRG.208355.1		XLOC_212811	243 coding	noncoding	noncoding	noncoding
MSTRG.208400.1		XLOC_212837	280 coding	noncoding	noncoding	noncoding
MSTRG.208423.15	WDR62	XLOC_212842	256 coding	noncoding	noncoding	noncoding
MSTRG.208424.1	WDR62	XLOC_212843	288 coding	noncoding	noncoding	noncoding
MSTRG.208443.1		XLOC_212851	268 coding	noncoding	noncoding	noncoding
NM_001099638.1	ZNF146	XLOC_212854	3783 coding	coding	coding	coding
MSTRG.208449.1	ZNF565	XLOC_212856	264 coding	noncoding	noncoding	noncoding
NM_001294306.1	LOC728485	XLOC_212888	1885 coding	noncoding	coding	coding
MSTRG.208555.1	ZNF790-AS1	XLOC_212890	256 coding	noncoding	noncoding	noncoding
MSTRG.208598.1	ZNF568	XLOC_212905	244 coding	noncoding	noncoding	noncoding
NM_001345948.2	ZNF383	XLOC_212920	6672 coding	coding	coding	coding
NM_152604.2	ZNF383	XLOC_212920	1946 coding	coding	coding	coding
MSTRG.208603.1	ZNF527	XLOC_212931	302 coding	noncoding	noncoding	noncoding
MSTRG.208683.1	LOC100631378	XLOC_212973	307 coding	noncoding	noncoding	noncoding
NM_021102.4	SPINT2	XLOC_212988	1688 coding	coding	coding	coding
MSTRG.208745.7	RYR1	XLOC_212997	517 coding	noncoding	coding	noncoding
MSTRG.208750.1	RYR1	XLOC_212998	306 coding	noncoding	noncoding	noncoding
MSTRG.208770.1		XLOC_213013	263 coding	noncoding	noncoding	noncoding
MSTRG.208771.1		XLOC_213014	274 coding	noncoding	noncoding	noncoding
MSTRG.208772.1		XLOC_213015	241 coding	noncoding	noncoding	noncoding
MSTRG.208782.1		XLOC_213019	238 coding	noncoding	noncoding	noncoding
XM_011527060.3	SAMD4B	XLOC_213031	3735 coding	coding	coding	coding
MSTRG.208829.1		XLOC_213050	269 coding	noncoding	noncoding	noncoding
MSTRG.208859.1	LGALS17A	XLOC_213055	243 coding	noncoding	noncoding	noncoding
MSTRG.208879.2	FBL	XLOC_213064	1033 coding	coding	coding	coding
MSTRG.208888.1		XLOC_213080	352 coding	noncoding	noncoding	noncoding
MSTRG.208934.1	C19orf47	XLOC_213091	238 coding	noncoding	noncoding	noncoding
MSTRG.208918.4	BLVRB	XLOC_213097	822 coding	coding	coding	noncoding

MSTRG.208918.3	BLVRB	XLOC_213097	823	coding	coding	coding	noncoding
XM_017027049.1	SPTBN4	XLOC_213100	8940	coding	coding	noncoding	coding
MSTRG.208952.1	SPTBN4	XLOC_213102	250	coding	noncoding	noncoding	noncoding
MSTRG.208957.1	SPTBN4	XLOC_213105	216	coding	noncoding	noncoding	noncoding
MSTRG.208958.1	SPTBN4	XLOC_213106	302	coding	noncoding	noncoding	noncoding
MSTRG.208960.8	SHKBP1	XLOC_213107	442	coding	noncoding	coding	noncoding
MSTRG.209025.1	SNRPA	XLOC_213121	273	coding	noncoding	noncoding	noncoding
MSTRG.208981.1		XLOC_213125	299	coding	noncoding	noncoding	noncoding
MSTRG.208962.1		XLOC_213127	268	coding	noncoding	noncoding	noncoding
NR_001278.1	CYP2B7P	XLOC_213129	2910	coding	coding	coding	coding
MSTRG.208971.1		XLOC_213132	277	coding	noncoding	noncoding	noncoding
MSTRG.208987.1		XLOC_213133	252	coding	noncoding	noncoding	noncoding
MSTRG.208993.1		XLOC_213136	310	coding	coding	noncoding	noncoding
MSTRG.208996.1		XLOC_213138	241	coding	noncoding	noncoding	noncoding
XM_017026384.2	CYP2F1	XLOC_213139	2051	coding	coding	coding	coding
NM_001278599.1	AXL	XLOC_213143	4120	coding	coding	coding	coding
XM_005258459.4	HNRNPUL1	XLOC_213144	3789	coding	coding	coding	coding
XM_024451763.1	CEACAM21	XLOC_213170	3032	coding	coding	coding	coding
MSTRG.209138.1	GRIK5	XLOC_213208	546	coding	noncoding	noncoding	noncoding
MSTRG.209139.1	GRIK5	XLOC_213209	291	coding	noncoding	noncoding	noncoding
MSTRG.209141.1	GRIK5	XLOC_213211	293	coding	noncoding	noncoding	noncoding
MSTRG.209143.1	GRIK5	XLOC_213213	246	coding	noncoding	noncoding	noncoding
MSTRG.209149.1		XLOC_213252	299	coding	noncoding	noncoding	noncoding
XM_005258674.2	CIC	XLOC_213253	5558	coding	coding	coding	coding
MSTRG.209184.1	PSG8	XLOC_213277	223	coding	noncoding	noncoding	noncoding
MSTRG.209187.1	PSG8	XLOC_213279	306	coding	noncoding	noncoding	noncoding
MSTRG.209195.1		XLOC_213289	281	coding	noncoding	noncoding	noncoding
MSTRG.209199.1	PSG11	XLOC_213290	296	coding	noncoding	noncoding	noncoding
MSTRG.209252.1	PSG9	XLOC_213298	303	coding	noncoding	noncoding	noncoding
MSTRG.209314.1		XLOC_213301	297	coding	noncoding	noncoding	noncoding
MSTRG.209305.1		XLOC_213306	217	coding	noncoding	noncoding	noncoding
MSTRG.209344.1		XLOC_213328	258	coding	noncoding	noncoding	noncoding
MSTRG.209347.1		XLOC_213329	494	coding	noncoding	noncoding	noncoding
XM_017027562.1	LOC105372412	XLOC_213330	2284	coding	coding	coding	coding
MSTRG.209370.1		XLOC_213339	230	coding	noncoding	noncoding	noncoding
MSTRG.209379.1		XLOC_213343	263	coding	noncoding	noncoding	noncoding
MSTRG.209399.1		XLOC_213348	334	coding	noncoding	noncoding	noncoding
MSTRG.209415.1		XLOC_213356	310	coding	noncoding	noncoding	noncoding
XM_011527286.2	ZNF225	XLOC_213365	5142	coding	coding	coding	coding
XM_017026152.1	ZNF234	XLOC_213369	3037	coding	coding	coding	coding
MSTRG.209405.1	ZNF234	XLOC_213370	218	coding	noncoding	noncoding	noncoding
NM_001289172.1	ZNF227	XLOC_213375	2968	coding	coding	coding	coding
NM_006505.5	PVR	XLOC_213411	5792	coding	coding	coding	coding

MSTRG.209480.1	LOC107985305	XLOC_213412	267 coding	noncoding	noncoding	noncoding
MSTRG.209499.1	BCL3	XLOC_213420	265 coding	noncoding	noncoding	noncoding
MSTRG.209555.1	RELB	XLOC_213435	219 coding	noncoding	noncoding	noncoding
MSTRG.209584.1	BLOC1S3	XLOC_213448	417 coding	noncoding	noncoding	noncoding
NM_031417.4	MARK4	XLOC_213450	5231 coding	coding	coding	coding
MSTRG.209592.1	MARK4	XLOC_213452	252 coding	noncoding	noncoding	noncoding
MSTRG.209593.1	MARK4	XLOC_213453	233 coding	noncoding	noncoding	noncoding
MSTRG.209613.1	PPP1R13L	XLOC_213459	260 coding	noncoding	noncoding	noncoding
XM_017026586.1	GIPR	XLOC_213475	2794 coding	coding	coding	coding
MSTRG.209643.1	BHMG1	XLOC_213482	275 coding	noncoding	noncoding	noncoding
MSTRG.209662.1	DMWD	XLOC_213487	294 coding	noncoding	noncoding	noncoding
MSTRG.209654.1		XLOC_213489	224 coding	noncoding	noncoding	noncoding
MSTRG.209665.1	FOXA3	XLOC_213492	292 coding	noncoding	noncoding	noncoding
MSTRG.209668.1		XLOC_213496	218 coding	noncoding	noncoding	noncoding
MSTRG.209723.1	PNMA8B	XLOC_213509	230 coding	coding	noncoding	noncoding
MSTRG.209725.1	PPP5D1	XLOC_213510	229 coding	noncoding	noncoding	noncoding
MSTRG.209740.1	DACT3	XLOC_213516	234 coding	noncoding	noncoding	noncoding
MSTRG.209755.1	SLC1A5	XLOC_213522	874 coding	coding	coding	coding
MSTRG.209768.1	ARHGAP35	XLOC_213532	226 coding	noncoding	noncoding	noncoding
MSTRG.209785.1	SAE1	XLOC_213541	259 coding	noncoding	noncoding	noncoding
XM_011527550.2	DHX34	XLOC_213556	4972 coding	coding	coding	coding
MSTRG.209810.1	MEIS3	XLOC_213561	266 coding	noncoding	noncoding	noncoding
NM_014601.4	EHD2	XLOC_213574	3506 coding	coding	coding	coding
MSTRG.209840.1		XLOC_213579	254 coding	noncoding	noncoding	noncoding
MSTRG.209846.1		XLOC_213580	319 coding	noncoding	noncoding	noncoding
MSTRG.209847.1		XLOC_213582	315 coding	noncoding	noncoding	noncoding
MSTRG.209851.1	SULT2A1	XLOC_213583	279 coding	noncoding	noncoding	noncoding
NM_022142.5	ELSPBP1	XLOC_213596	1079 coding	coding	coding	coding
MSTRG.209879.2	CABP5	XLOC_213597	547 coding	coding	noncoding	noncoding
MSTRG.209884.1	PLA2G4C	XLOC_213601	225 coding	noncoding	noncoding	noncoding
MSTRG.209963.23	CARD8-AS1	XLOC_213614	3916 coding	coding	coding	noncoding
MSTRG.209963.34	CARD8	XLOC_213614	1740 coding	coding	coding	noncoding
MSTRG.209907.1	CCDC114	XLOC_213617	295 coding	noncoding	noncoding	noncoding
MSTRG.209940.1	GRIN2D	XLOC_213629	248 coding	noncoding	noncoding	noncoding
MSTRG.209945.1	KCNJ14	XLOC_213631	289 coding	noncoding	noncoding	noncoding
XR_001753791.1	CYTH2	XLOC_213632	4534 coding	coding	coding	coding
MSTRG.209925.1	CYTH2	XLOC_213633	246 coding	noncoding	noncoding	noncoding
MSTRG.209934.1	LMTK3	XLOC_213636	229 coding	coding	noncoding	noncoding
MSTRG.209926.1		XLOC_213638	245 coding	noncoding	noncoding	noncoding
NM_004605.2	SULT2B1	XLOC_213640	1253 coding	coding	coding	coding
MSTRG.209977.1	FAM83E	XLOC_213642	343 coding	noncoding	noncoding	noncoding
MSTRG.209959.1	NTN5	XLOC_213648	259 coding	noncoding	noncoding	noncoding
MSTRG.209954.1	FUT2	XLOC_213651	227 coding	noncoding	noncoding	noncoding

MSTRG.209986.1	IZUMO1	XLOC_213652	233 coding	noncoding	noncoding	noncoding
MSTRG.209972.1	PLEKHA4	XLOC_213658	240 coding	noncoding	noncoding	noncoding
XM_017026598.1	DHDH	XLOC_213663	1641 coding	coding	coding	coding
MSTRG.210007.1	RUVBL2	XLOC_213668	268 coding	noncoding	noncoding	noncoding
MSTRG.210009.1	RUVBL2	XLOC_213670	284 coding	noncoding	noncoding	noncoding
NM_001301069.2	SNRNP70	XLOC_213674	1644 coding	coding	coding	coding
NM_017636.4	TRPM4	XLOC_213678	4058 coding	coding	coding	coding
NR_033397.4	PRMT1	XLOC_213727	1568 coding	coding	coding	coding
MSTRG.210144.1	AP2A1	XLOC_213734	236 coding	noncoding	noncoding	noncoding
MSTRG.210157.1	PTOV1	XLOC_213741	3299 coding	coding	coding	noncoding
MSTRG.210130.1		XLOC_213749	218 coding	noncoding	noncoding	noncoding
MSTRG.210165.1	LOC400710	XLOC_213758	496 coding	noncoding	noncoding	noncoding
MSTRG.210163.1		XLOC_213759	240 coding	noncoding	noncoding	noncoding
MSTRG.210170.1		XLOC_213762	252 coding	noncoding	noncoding	noncoding
NM_001145809.2	MYH14	XLOC_213764	6914 coding	coding	coding	coding
MSTRG.210185.1	MYH14	XLOC_213765	217 coding	noncoding	noncoding	noncoding
MSTRG.210186.1	MYH14	XLOC_213766	464 coding	noncoding	noncoding	noncoding
MSTRG.210217.1		XLOC_213786	230 coding	noncoding	noncoding	noncoding
MSTRG.210234.1		XLOC_213798	247 coding	noncoding	noncoding	noncoding
MSTRG.210235.1		XLOC_213799	207 coding	noncoding	noncoding	noncoding
MSTRG.210244.1		XLOC_213801	278 coding	noncoding	noncoding	noncoding
XM_005258799.5	SIGLECL1	XLOC_213822	2005 coding	noncoding	coding	coding
MSTRG.210277.1	VSIG10L	XLOC_213828	262 coding	noncoding	noncoding	noncoding
XM_017027249.2	ZNF175	XLOC_213848	3799 coding	coding	coding	coding
MSTRG.210350.2	SIGLEC5	XLOC_213854	1809 coding	coding	coding	noncoding
MSTRG.210358.1	SPACA6	XLOC_213859	269 coding	noncoding	noncoding	noncoding
MSTRG.210361.1		XLOC_213863	233 coding	noncoding	noncoding	noncoding
MSTRG.210372.1	ZNF577	XLOC_213866	210 coding	noncoding	noncoding	noncoding
MSTRG.210423.3	ZNF841	XLOC_213886	991 coding	coding	noncoding	noncoding
MSTRG.210389.1		XLOC_213893	293 coding	noncoding	noncoding	noncoding
MSTRG.210395.1	LOC105372449	XLOC_213902	257 coding	coding	noncoding	noncoding
XM_017027440.1	ZNF766	XLOC_213903	2846 coding	coding	noncoding	coding
MSTRG.210444.1	ZNF480	XLOC_213906	588 coding	noncoding	noncoding	noncoding
XM_011527394.2	ZNF528	XLOC_213911	4098 coding	coding	coding	coding
XR_935832.3	ZNF808	XLOC_213914	4228 coding	coding	coding	coding
XM_024451508.1	ZNF808	XLOC_213914	3920 coding	coding	coding	coding
MSTRG.210558.9	ZNF611	XLOC_213924	1650 coding	coding	noncoding	noncoding
MSTRG.210569.1		XLOC_213928	271 coding	noncoding	noncoding	noncoding
MSTRG.210577.1	ZNF320	XLOC_213935	220 coding	noncoding	noncoding	noncoding
NM_152473.2	ERVV-1	XLOC_213946	2490 coding	coding	coding	coding
MSTRG.210453.1		XLOC_213963	443 coding	noncoding	noncoding	noncoding
XM_024451583.1	ZNF331	XLOC_213973	6307 coding	coding	coding	coding
MSTRG.210603.1	ZNF331	XLOC_213974	239 coding	noncoding	noncoding	noncoding

MSTRG.210492.1	MIR516B2	XLOC_214012	303 coding	noncoding	noncoding	noncoding
MSTRG.210493.1	MIR519A1	XLOC_214024	275 coding	noncoding	noncoding	noncoding
MSTRG.210514.5	NLRP12	XLOC_214035	4357 coding	coding	coding	coding
NM_001290188.2	MYADM	XLOC_214036	3104 coding	coding	coding	coding
MSTRG.210637.4	LILRB2	XLOC_214058	577 coding	noncoding	noncoding	noncoding
NR_103502.1	LILRA1	XLOC_214068	2162 coding	coding	coding	noncoding
MSTRG.210674.3	PPP6R1	XLOC_214094	367 coding	noncoding	noncoding	noncoding
XM_024451742.1	KMT5C	XLOC_214101	2664 coding	coding	coding	coding
MSTRG.210715.1	LOC105372462	XLOC_214107	277 coding	noncoding	noncoding	noncoding
MSTRG.210720.1	SBK2	XLOC_214114	393 coding	noncoding	noncoding	noncoding
MSTRG.210722.1		XLOC_214115	285 coding	noncoding	noncoding	noncoding
MSTRG.210756.1	NLRP11	XLOC_214136	294 coding	noncoding	noncoding	noncoding
MSTRG.210752.1	NLRP13	XLOC_214139	309 coding	noncoding	noncoding	noncoding
NM_153447.4	NLRP5	XLOC_214142	3885 coding	coding	coding	coding
MSTRG.210763.1	NLRP5	XLOC_214143	244 coding	noncoding	noncoding	noncoding
MSTRG.210892.1	ZIM2	XLOC_214216	393 coding	noncoding	noncoding	noncoding
MSTRG.210882.1		XLOC_214227	202 coding	noncoding	noncoding	noncoding
MSTRG.210898.1	ZNF264	XLOC_214233	434 coding	noncoding	noncoding	noncoding
XM_011526767.3	ZIK1	XLOC_214265	4748 coding	coding	coding	coding
MSTRG.210999.1		XLOC_214281	240 coding	noncoding	noncoding	noncoding
MSTRG.211012.1	C19orf18	XLOC_214310	359 coding	noncoding	noncoding	noncoding
MSTRG.211047.1		XLOC_214333	234 coding	noncoding	noncoding	noncoding
XR_002958439.1	LOC101928344	XLOC_214384	2013 coding	coding	coding	noncoding
MSTRG.204900.1		XLOC_214390	435 coding	noncoding	noncoding	noncoding
MSTRG.204948.1	HCN2	XLOC_214407	399 coding	noncoding	noncoding	noncoding
NM_214710.5	PRSS57	XLOC_214415	1044 coding	coding	coding	coding
MSTRG.204984.5	R3HDM4	XLOC_214427	1566 coding	coding	coding	coding
MSTRG.204984.4	R3HDM4	XLOC_214427	1545 coding	coding	coding	coding
MSTRG.205049.3	POLR2E	XLOC_214439	7263 coding	coding	coding	coding
MSTRG.205010.1		XLOC_214456	257 coding	noncoding	noncoding	noncoding
MSTRG.205061.1	APC2	XLOC_214466	287 coding	noncoding	noncoding	noncoding
XR_002958347.1	TCF3	XLOC_214474	4602 coding	coding	coding	coding
MSTRG.205084.1		XLOC_214479	305 coding	noncoding	noncoding	noncoding
XR_001753839.1	LOC101928543	XLOC_214481	2312 coding	coding	coding	noncoding
XM_006722655.3	ATP8B3	XLOC_214482	5211 coding	coding	coding	coding
MSTRG.205109.1		XLOC_214490	252 coding	noncoding	noncoding	noncoding
MSTRG.205112.1	ABHD17A	XLOC_214491	297 coding	noncoding	noncoding	noncoding
MSTRG.205127.1	SCAMP4	XLOC_214495	1496 coding	coding	coding	noncoding
NR_033400.1	CSNK1G2-AS1	XLOC_214504	1313 coding	noncoding	coding	noncoding
MSTRG.205119.1	BTBD2	XLOC_214505	266 coding	noncoding	noncoding	noncoding
MSTRG.205118.1	MKNK2	XLOC_214510	265 coding	noncoding	noncoding	noncoding
MSTRG.205175.1	LINGO3	XLOC_214533	816 coding	noncoding	noncoding	noncoding
MSTRG.205188.1		XLOC_214541	271 coding	noncoding	noncoding	noncoding

MSTRG.205190.1		XLOC_214542	300 coding	noncoding	noncoding	noncoding
MSTRG.205199.1		XLOC_214548	226 coding	noncoding	noncoding	noncoding
MSTRG.205210.1		XLOC_214550	286 coding	noncoding	noncoding	noncoding
MSTRG.205212.1		XLOC_214551	298 coding	noncoding	noncoding	noncoding
MSTRG.205233.1	GNG7	XLOC_214554	251 coding	noncoding	noncoding	noncoding
MSTRG.205252.1	GNG7	XLOC_214559	263 coding	noncoding	noncoding	noncoding
MSTRG.205257.1		XLOC_214567	232 coding	noncoding	noncoding	noncoding
MSTRG.205264.1		XLOC_214569	298 coding	noncoding	noncoding	noncoding
MSTRG.205282.1		XLOC_214577	252 coding	noncoding	noncoding	noncoding
MSTRG.205288.1	TLE6	XLOC_214579	302 coding	noncoding	noncoding	noncoding
MSTRG.205302.1	TLE2	XLOC_214582	276 coding	noncoding	noncoding	noncoding
MSTRG.205338.1	CELF5	XLOC_214595	252 coding	noncoding	noncoding	noncoding
MSTRG.205366.1		XLOC_214621	257 coding	noncoding	noncoding	noncoding
MSTRG.205389.1	CACTIN	XLOC_214627	285 coding	noncoding	noncoding	noncoding
MSTRG.205420.1	ZFR2	XLOC_214642	256 coding	noncoding	noncoding	noncoding
MSTRG.205414.1		XLOC_214646	242 coding	noncoding	noncoding	noncoding
MSTRG.205449.1	MAP2K2	XLOC_214659	376 coding	noncoding	noncoding	noncoding
MSTRG.205470.1	STAP2	XLOC_214669	340 coding	noncoding	noncoding	noncoding
MSTRG.205497.1	HDGFL2	XLOC_214685	227 coding	noncoding	noncoding	noncoding
XM_011527639.2	SEMA6B	XLOC_214692	4009 coding	coding	coding	coding
MSTRG.205512.1	SEMA6B	XLOC_214693	249 coding	noncoding	noncoding	noncoding
MSTRG.205523.1	MYDGF	XLOC_214701	355 coding	noncoding	noncoding	noncoding
NR_158699.1	DPP9	XLOC_214702	4411 coding	coding	coding	coding
MSTRG.205527.1		XLOC_214705	240 coding	noncoding	noncoding	noncoding
MSTRG.205528.1		XLOC_214706	332 coding	noncoding	noncoding	noncoding
NM_001080523.3	ARRDC5	XLOC_214711	1667 coding	coding	coding	coding
MSTRG.205550.1		XLOC_214725	250 coding	noncoding	noncoding	noncoding
MSTRG.205552.1		XLOC_214726	235 coding	noncoding	noncoding	noncoding
MSTRG.205554.1		XLOC_214727	221 coding	noncoding	noncoding	noncoding
MSTRG.205559.1		XLOC_214730	209 coding	noncoding	noncoding	noncoding
MSTRG.205635.1	CATSPERD	XLOC_214748	321 coding	noncoding	noncoding	noncoding
MSTRG.205639.1	CATSPERD	XLOC_214749	291 coding	noncoding	noncoding	noncoding
MSTRG.205598.1	FUT3	XLOC_214754	234 coding	noncoding	noncoding	noncoding
MSTRG.205752.1	RFX2	XLOC_214770	286 coding	noncoding	noncoding	noncoding
MSTRG.205764.1	RFX2	XLOC_214776	308 coding	noncoding	noncoding	noncoding
MSTRG.205643.1	ACSBG2	XLOC_214783	314 coding	noncoding	noncoding	noncoding
MSTRG.205668.1	ACER1	XLOC_214790	284 coding	noncoding	noncoding	noncoding
MSTRG.205679.1	GTF2F1	XLOC_214795	226 coding	noncoding	noncoding	noncoding
XM_011528281.2	SLC25A23	XLOC_214800	3601 coding	coding	coding	coding
MSTRG.205690.1		XLOC_214803	269 coding	noncoding	noncoding	noncoding
MSTRG.205699.1		XLOC_214807	241 coding	noncoding	noncoding	noncoding
MSTRG.205715.1	C3	XLOC_214812	324 coding	noncoding	noncoding	noncoding
MSTRG.205779.1		XLOC_214819	253 coding	noncoding	noncoding	noncoding

MSTRG.205798.1		XLOC_214831	216	coding	noncoding	noncoding	noncoding
XM_011527988.2	INSR	XLOC_214832	9459	coding	coding	coding	coding
MSTRG.205810.1	INSR	XLOC_214833	247	coding	noncoding	noncoding	noncoding
MSTRG.205811.1	INSR	XLOC_214834	223	coding	noncoding	noncoding	noncoding
MSTRG.205802.1		XLOC_214836	260	coding	noncoding	noncoding	noncoding
XR_001753854.1	LOC107985284	XLOC_214838	347	coding	noncoding	noncoding	noncoding
MSTRG.205832.1	ARHGEF18	XLOC_214842	1050	coding	noncoding	noncoding	noncoding
MSTRG.205815.1	TEX45	XLOC_214846	280	coding	noncoding	noncoding	noncoding
MSTRG.205813.1		XLOC_214847	265	coding	noncoding	noncoding	noncoding
MSTRG.205841.4	PNPLA6	XLOC_214850	459	coding	coding	noncoding	noncoding
MSTRG.205817.1		XLOC_214851	250	coding	noncoding	noncoding	noncoding
MSTRG.205859.1		XLOC_214867	311	coding	noncoding	noncoding	noncoding
MSTRG.205862.1		XLOC_214870	236	coding	noncoding	noncoding	noncoding
MSTRG.205866.1	LRRC8E	XLOC_214880	311	coding	coding	noncoding	noncoding
MSTRG.205885.2	MAP2K7	XLOC_214881	1233	coding	coding	coding	noncoding
MSTRG.205905.1	FBN3	XLOC_214890	294	coding	noncoding	noncoding	noncoding
MSTRG.205907.1	FBN3	XLOC_214891	307	coding	noncoding	noncoding	noncoding
MSTRG.205915.3	CERS4	XLOC_214893	1515	coding	coding	coding	noncoding
MSTRG.205945.1		XLOC_214909	257	coding	noncoding	noncoding	noncoding
MSTRG.205966.1		XLOC_214918	292	coding	noncoding	noncoding	noncoding
NM_178525.5	ACTL9	XLOC_214923	1396	coding	coding	coding	coding
NM_024690.2	MUC16	XLOC_214929	43816	coding	coding	noncoding	coding
MSTRG.205998.1		XLOC_214931	326	coding	noncoding	noncoding	noncoding
[truncated: 261,364 more chars]
