# Supplementary material for: Whole-Transcriptome RNA Sequencing Reveals Significant Differentially Expressed mRNAs, miRNAs, and lncRNAs and Related Regulating Biological Pathways in the Peripheral Blood of COVID-19 Patients
Source: Mediators Inflamm. 2021 Apr 1;2021:6635925. doi: 10.1155/2021/6635925 (PMC8018221; doi:10.1155/2021/6635925)
Supplement: Supplementary Materials — Table S1: the differentially expressed mRNAs of peripheral blood samples in COVID-19 patients. Table S2: the differentially expressed miRNA of peripheral blood samples in COVID-19 patients. Table S3: the differentially expressed lncRNAs of peripheral blood samples in COVID-19 patients. [file 6635925.f1.zip › Table S2.doc]

**Table S2 The differentially expressed miRNA of peripheral blood samples in COVID-19 patients**

| miRNA  hsa-miR-16-2-3p  hsa-miR-5695  hsa-miR-10399-3p  hsa-miR-6501-5p  hsa-miR-4659a-3p  hsa-miR-142-5p  hsa-miR-505-5p  hsa-miR-125b-5p  hsa-miR-618 | logFC  1.562771123  1.017457338  1.332176427  1.739937627  1.036168092  1.019307475  1.060269434  1.114373446  1.620629283 | pvalue  2.16E-04  5.08E-04  5.46E-04  0.002160092  0.004285888  0.00431663  0.015390418  0.019043829  0.019812222 | level | X10(RPM) | X11(RPM) | X13(RPM) | X14(RPM) | X4(RPM) | X5(RPM) | X15(RPM) | X1(RPM) | X2(RPM) | X3(RPM) | X6(RPM) | X7(RPM) | X8(RPM) | X9(RPM) |
| --- | --- | --- | --- | --- | --- | --- | --- | --- | --- | --- | --- | --- | --- | --- | --- | --- | --- |
|  |  |  | Increased | 10.38 | 12.08 | 8.3 | 12.27 | 30.61 | 25.97 | 17.57 | 17.75 | 54.63 | 94.14 | 25.31 | 86.67 | 15.15 | 19.42 |
|  |  |  | Increased | 0.74 | 1.19 | 0.98 | 1.7 | 2.81 | 2.92 | 1.44 | 1.66 | 2.54 | 3.3 | 2.63 | 3.19 | 2.53 | 2.61 |
|  |  |  | Increased | 0.81 | 0.96 | 0.24 | 1 | 2.06 | 1.06 | 1.38 | 2.24 | 2.66 | 2.75 | 2.55 | 2.13 | 2.64 | 1.5 |
|  |  |  | Increased | 0.15 | 0.34 | 0.12 | 0.08 | 0.56 | 1.5 | 0.4 | 0.66 | 0.52 | 1.1 | 0.66 | 0.18 | 0.95 | 0.44 |
|  |  |  | Increased | 0.88 | 1.25 | 1.28 | 1.23 | 1.78 | 2.03 | 0.75 | 2.57 | 3.35 | 3.3 | 4.23 | 3.9 | 2.8 | 2.22 |
|  |  |  | Increased | 552.27 | 693.19 | 664.15 | 1048.46 | 3375.65 | 1437.11 | 445.55 | 1786.53 | 2895.95 | 2865.85 | 1334.26 | 1127.23 | 1773.28 | 685.05 |
|  |  |  | Increased | 0.07 | 0.34 | 0.55 | 0.69 | 0.84 | 0.71 | 0.98 | 1.33 | 0.98 | 2.36 | 0.44 | 0.77 | 0.74 | 0.67 |
|  |  |  | Increased | 61.67 | 27.18 | 34.44 | 45.9 | 31.45 | 77.04 | 68.69 | 147.52 | 223.13 | 58.83 | 95.69 | 61.5 | 136.26 | 186.18 |
|  |  |  | Increased | 0.66 | 0.28 | 0.18 | 0 | 0.84 | 1.06 | 0.23 | 0.17 | 0.87 | 3.07 | 0.73 | 1.95 | 0.79 | 2.05 |
| hsa-miR-183-5p | -1.301202932 | 4.59E-05 | Decreased | 92.57 | 70.92 | 105.52 | 67.27 | 29.86 | 73.77 | 19.64 | 28.19 | 67.28 | 56.71 | 41.94 | 15.95 | 49.98 | 30.69 |
| hsa-miR-627-5p | -2.294946517 | 8.76E-05 | Decreased | 0.44 | 0.11 | 0.79 | 1.16 | 0 | 0.18 | 0 | 0.25 | 0.06 | 0.16 | 0.22 | 0.3 | 0.05 | 0.17 |
| hsa-miR-21-5p | -1.139996598 | 5.98E-04 | Decreased | 134.96 | 123.8 | 116.64 | 61.57 | 44.56 | 105.84 | 24.29 | 57.71 | 85.7 | 91.94 | 60.61 | 17.61 | 66.02 | 61.87 |
| hsa-miR-20a-5p | -1.163995958 | 0.001443502 | Decreased | 68.29 | 53.78 | 69.49 | 44.98 | 29.95 | 59.99 | 11.49 | 26.45 | 56.53 | 37.04 | 25.24 | 5.85 | 36.26 | 35.79 |
| hsa-miR-18a-5p | -1.055509261 | 0.00286504 | Decreased | 8.46 | 7.55 | 7.69 | 4.32 | 5.05 | 10.16 | 2.3 | 3.15 | 5.43 | 5.74 | 1.68 | 1.54 | 3.22 | 3.39 |
| hsa-miR-4521 | -1.207929268 | 0.006723958 | Decreased | 1.1 | 1.48 | 0.31 | 1.08 | 0.75 | 0.18 | 0.23 | 0.83 | 0.81 | 0.47 | 0.07 | 0.41 | 0.53 | 0.55 |
| hsa-miR-144-3p | -1.348840352 | 0.009508389 | Decreased | 746.91 | 593.28 | 1263.76 | 512.89 | 503.78 | 846.35 | 46.29 | 432.18 | 872.73 | 358.63 | 255.5 | 17.66 | 261.91 | 263.25 |
| hsa-miR-199a-3p | -1.210348176 | 0.015517807 | Decreased | 1.55 | 1.42 | 1.95 | 1.54 | 0.19 | 1.41 | 0 | 1.08 | 1.27 | 1.34 | 0.95 | 0.06 | 0.84 | 1.39 |
| hsa-miR-199b-3p | -1.210348176 | 0.015517807 | Decreased | 1.55 | 1.42 | 1.95 | 1.54 | 0.19 | 1.41 | 0 | 1.08 | 1.27 | 1.34 | 0.95 | 0.06 | 0.84 | 1.39 |
| hsa-miR-96-5p | -1.032735881 | 0.017072046 | Decreased | 404.95 | 221.72 | 445.96 | 237.85 | 113.17 | 361.33 | 79.49 | 139.89 | 344.75 | 183.01 | 192.19 | 37.57 | 379.97 | 110.15 |
| hsa-miR-18b-5p | -1.071278826 | 0.018190844 | Decreased | 0.44 | 0.68 | 1.16 | 0.69 | 0.56 | 0.62 | 0.17 | 0.5 | 0.75 | 0.79 | 0.07 | 0 | 0.47 | 0.28 |
| hsa-miR-3613-5p | -1.108270863 | 0.023167595 | Decreased | 2.28 | 2.33 | 3.54 | 2.01 | 1.12 | 2.83 | 0.34 | 1.74 | 3.75 | 0.71 | 1.02 | 0.06 | 1.21 | 1.22 |
| hsa-miR-29b-2-5p | -1.067935525 | 0.025051488 | Decreased | 0.74 | 1.25 | 0.55 | 1 | 0.56 | 1.06 | 0.11 | 0.17 | 0.81 | 0.87 | 0 | 0.77 | 0.37 | 0.22 |
| hsa-miR-32-5p | -1.270769961 | 0.037001462 | Decreased | 2.21 | 3.74 | 6.17 | 1.39 | 1.87 | 2.92 | 0 | 3.73 | 3.23 | 0.39 | 1.17 | 0.12 | 1.58 | 1.5 |
