# Supplementary material for: Whole-Transcriptome RNA Sequencing Reveals Significant Differentially Expressed mRNAs, miRNAs, and lncRNAs and Related Regulating Biological Pathways in the Peripheral Blood of COVID-19 Patients
Source: Mediators Inflamm. 2021 Apr 1;2021:6635925. doi: 10.1155/2021/6635925 (PMC8018221; doi:10.1155/2021/6635925)
Supplement: Supplementary Materials — Table S1: the differentially expressed mRNAs of peripheral blood samples in COVID-19 patients. Table S2: the differentially expressed miRNA of peripheral blood samples in COVID-19 patients. Table S3: the differentially expressed lncRNAs of peripheral blood samples in COVID-19 patients. [file 6635925.f1.zip › Table S3.doc]

**Table S3 The differentially expressed lncRNAs of peripheral blood samples in COVID-19 patients**

| ID | logFC | *P-Value* | type |
| --- | --- | --- | --- |
| MSTRG.72445.6 | 9.69294974 | 3.19E-07 | up |
| MSTRG.136039.10 | 6.903415832 | 1.34E-06 | up |
| MSTRG.142448.1 | 7.975983927 | 3.57E-06 | up |
| MSTRG.60252.1 | 6.949181426 | 2.26E-05 | up |
| MSTRG.45204.1 | 7.2973186 | 8.06E-05 | up |
| MSTRG.71809.3 | 5.30601886 | 0.000140107 | up |
| MSTRG.165354.17 | 9.38798617 | 0.000282381 | up |
| MSTRG.239324.1 | 5.371220137 | 0.000421037 | up |
| MSTRG.71122.1 | 5.344373994 | 0.000489065 | up |
| MSTRG.85826.1 | 3.659854607 | 0.000671458 | up |
| MSTRG.16716.1 | 7.375995349 | 0.000834299 | up |
| MSTRG.178676.2 | 8.512053794 | 0.000887722 | up |
| MSTRG.71809.2 | 8.374138097 | 0.001089835 | up |
| MSTRG.74250.5 | 6.538885068 | 0.001102223 | up |
| MSTRG.28118.19 | 2.684433697 | 0.001161988 | up |
| MSTRG.11372.3 | 10.05975866 | 0.001201585 | up |
| MSTRG.157565.1 | 6.590747721 | 0.001510689 | up |
| MSTRG.105376.5 | 7.273311805 | 0.0015135 | up |
| MSTRG.117579.30 | 9.884883151 | 0.001782037 | up |
| MSTRG.83614.8 | 9.453382764 | 0.002127683 | up |
| MSTRG.210942.1 | 8.864355656 | 0.002848293 | up |
| MSTRG.83624.4 | 3.104523289 | 0.003224455 | up |
| MSTRG.212397.11 | 10.38912653 | 0.003267914 | up |
| MSTRG.5548.2 | 9.534262549 | 0.003396173 | up |
| MSTRG.125444.1 | 7.24751711 | 0.003618007 | up |
| MSTRG.204564.7 | 8.173736482 | 0.003975001 | up |
| MSTRG.157392.9 | 10.67719287 | 0.004018712 | up |
| MSTRG.54290.4 | 2.638140747 | 0.004274498 | up |
| MSTRG.52943.1 | 4.253349022 | 0.004444919 | up |
| MSTRG.200551.1 | 8.028772002 | 0.004521454 | up |
| MSTRG.233788.1 | 12.47943988 | 0.004605464 | up |
| MSTRG.24076.2 | 12.12999217 | 0.005088749 | up |
| MSTRG.90760.1 | 10.1436987 | 0.005474795 | up |
| MSTRG.117588.1 | 1.557183055 | 0.005503896 | up |
| MSTRG.237965.1 | 6.412224769 | 0.006174337 | up |
| MSTRG.205819.6 | 9.423010037 | 0.006363625 | up |
| MSTRG.68494.1 | 2.016038596 | 0.006434774 | up |
| MSTRG.175042.1 | 6.582057192 | 0.006641685 | up |
| MSTRG.48581.17 | 1.732064618 | 0.007507532 | up |
| MSTRG.25036.2 | 8.95664266 | 0.007655136 | up |
| MSTRG.164759.16 | 3.78049816 | 0.008185846 | up |
| MSTRG.72445.4 | 7.421554793 | 0.008538385 | up |
| MSTRG.119845.30 | 13.84642698 | 0.00863788 | up |
| MSTRG.228818.7 | 15.06523673 | 0.008833313 | up |
| MSTRG.38297.2 | 6.927185267 | 0.008993221 | up |
| MSTRG.129011.1 | 4.747002023 | 0.009509185 | up |
| MSTRG.130761.2 | 9.146592957 | 0.009871379 | up |
| MSTRG.129275.1 | 3.898136471 | 0.009876667 | up |
| MSTRG.133364.1 | 3.944683658 | 0.010446567 | up |
| MSTRG.114423.7 | 10.54835483 | 0.010827935 | up |
| MSTRG.228818.4 | 12.29966362 | 0.01083591 | up |
| MSTRG.84403.1 | 1.331956089 | 0.011144432 | up |
| MSTRG.165714.2 | 8.696559195 | 0.011958759 | up |
| MSTRG.177505.26 | 4.804933586 | 0.011991331 | up |
| MSTRG.194772.7 | 10.68619584 | 0.012066542 | up |
| MSTRG.160488.6 | 10.88182924 | 0.012171456 | up |
| MSTRG.43896.1 | 1.695802267 | 0.012244209 | up |
| MSTRG.200259.1 | 7.037657161 | 0.012482574 | up |
| MSTRG.26666.1 | 6.647475822 | 0.01251227 | up |
| MSTRG.184881.2 | 4.289903426 | 0.01303345 | up |
| MSTRG.236993.8 | 1.454279157 | 0.01328293 | up |
| MSTRG.44150.10 | 7.898012335 | 0.013669084 | up |
| MSTRG.182390.2 | 10.18484025 | 0.013956679 | up |
| MSTRG.36340.22 | 3.010328064 | 0.014497593 | up |
| MSTRG.110899.1 | 6.293523907 | 0.015422485 | up |
| MSTRG.119503.8 | 7.591055443 | 0.015720922 | up |
| MSTRG.73343.2 | 2.253363734 | 0.015880722 | up |
| MSTRG.72740.1 | 6.513537784 | 0.016797464 | up |
| MSTRG.100829.5 | 5.314697928 | 0.017203201 | up |
| MSTRG.57624.1 | 3.816994099 | 0.017940883 | up |
| MSTRG.171276.1 | 3.634536895 | 0.018509082 | up |
| MSTRG.37865.9 | 7.784007658 | 0.019556658 | up |
| MSTRG.26589.17 | 3.238881948 | 0.020483457 | up |
| MSTRG.176953.4 | 7.075166809 | 0.020605064 | up |
| MSTRG.123286.1 | 3.460374804 | 0.020913013 | up |
| MSTRG.14715.2 | 2.050104326 | 0.022168585 | up |
| MSTRG.42391.1 | 1.925596257 | 0.022736381 | up |
| MSTRG.196386.4 | 2.333253403 | 0.022830629 | up |
| MSTRG.42448.1 | 1.38449778 | 0.023656706 | up |
| MSTRG.7824.1 | 1.949475903 | 0.023941462 | up |
| MSTRG.106032.14 | 6.830967113 | 0.024081164 | up |
| MSTRG.64491.8 | 12.14568871 | 0.024108505 | up |
| MSTRG.130310.2 | 8.680722304 | 0.024837699 | up |
| MSTRG.87509.4 | 11.32555727 | 0.025641241 | up |
| MSTRG.234117.2 | 6.7632205 | 0.025985997 | up |
| MSTRG.208588.1 | 4.207320656 | 0.026005364 | up |
| MSTRG.63755.15 | 11.58622237 | 0.026282306 | up |
| MSTRG.58567.1 | 1.374882188 | 0.026430823 | up |
| MSTRG.7164.1 | 1.815340467 | 0.026605996 | up |
| MSTRG.41715.1 | 2.918876353 | 0.027955276 | up |
| MSTRG.205155.2 | 8.440906256 | 0.028418934 | up |
| MSTRG.180483.4 | 6.636382743 | 0.028669941 | up |
| MSTRG.64588.2 | 8.622133549 | 0.028814235 | up |
| MSTRG.136700.1 | 2.098891982 | 0.029026265 | up |
| MSTRG.4784.7 | 8.249236475 | 0.029497183 | up |
| MSTRG.189052.24 | 10.8893311 | 0.029801051 | up |
| MSTRG.190761.1 | 6.710203486 | 0.029922128 | up |
| MSTRG.225394.1 | 5.253287103 | 0.029964027 | up |
| MSTRG.183932.12 | 7.043353091 | 0.030484161 | up |
| MSTRG.233758.7 | 10.40544388 | 0.03053877 | up |
| MSTRG.118643.8 | 2.452291876 | 0.030813639 | up |
| MSTRG.82766.18 | 11.75682275 | 0.031404133 | up |
| MSTRG.239428.5 | 2.64177309 | 0.031937683 | up |
| MSTRG.68689.5 | 2.652321622 | 0.03211474 | up |
| MSTRG.154981.13 | 1.826726266 | 0.033456116 | up |
| MSTRG.76888.15 | 7.582797123 | 0.033498754 | up |
| MSTRG.77875.1 | 6.257384423 | 0.034024409 | up |
| MSTRG.162418.3 | 9.075343146 | 0.0365617 | up |
| MSTRG.64491.5 | 8.299635072 | 0.037785564 | up |
| MSTRG.201378.1 | 5.757463941 | 0.038785638 | up |
| MSTRG.42136.1 | 5.630680672 | 0.038900023 | up |
| MSTRG.110681.2 | 4.846412449 | 0.041648653 | up |
| MSTRG.155979.3 | 4.173023911 | 0.041828215 | up |
| MSTRG.59979.8 | 9.100299486 | 0.042698219 | up |
| MSTRG.101489.3 | 9.468569815 | 0.042841083 | up |
| MSTRG.240434.1 | 1.400440586 | 0.043143747 | up |
| MSTRG.158214.4 | 8.775884099 | 0.04418983 | up |
| MSTRG.220220.27 | 5.811443976 | 0.044943465 | up |
| MSTRG.37645.1 | 2.216693351 | 0.045526765 | up |
| MSTRG.53656.11 | 1.182438183 | 0.045708136 | up |
| MSTRG.71487.1 | 7.529785854 | 0.046219847 | up |
| MSTRG.203229.1 | 1.638712769 | 0.046579824 | up |
| MSTRG.233827.14 | 8.364878403 | 0.047211568 | up |
| MSTRG.11366.29 | 1.024034333 | 0.048274157 | up |
| MSTRG.26621.1 | 5.936150208 | 0.048277486 | up |
| MSTRG.216965.33 | 9.169830293 | 0.048712668 | up |
| MSTRG.60253.1 | 1.109629066 | 0.048892699 | up |
| MSTRG.95193.2 | 8.484680324 | 0.049068157 | up |
| MSTRG.47649.1 | 5.54771015 | 0.049316172 | up |

| MSTRG.173874.3 | -8.364320644 | 4.46E-07 | down |
| --- | --- | --- | --- |
| MSTRG.219679.5 | -10.96847566 | 1.06E-06 | down |
| MSTRG.26260.17 | -4.879807287 | 1.21E-06 | down |
| MSTRG.163364.1 | -8.190409858 | 7.61E-06 | down |
| MSTRG.228818.11 | -16.21556648 | 1.68E-05 | down |
| MSTRG.109232.2 | -13.02059906 | 3.14E-05 | down |
| MSTRG.150651.8 | -12.3481204 | 4.78E-05 | down |
| MSTRG.185341.23 | -12.24895701 | 5.10E-05 | down |
| MSTRG.185043.8 | -12.24110949 | 5.13E-05 | down |
| MSTRG.151872.3 | -10.34867342 | 6.47E-05 | down |
| MSTRG.2118.13 | -11.73417892 | 9.23E-05 | down |
| MSTRG.87742.12 | -2.964412847 | 0.000105352 | down |
| MSTRG.177.1 | -5.392423142 | 0.00012988 | down |
| MSTRG.92873.1 | -2.810200167 | 0.000144316 | down |
| MSTRG.236150.1 | -7.355801664 | 0.000145577 | down |
| MSTRG.83899.1 | -10.98108413 | 0.000184097 | down |
| MSTRG.157825.11 | -10.85435772 | 0.000214337 | down |
| MSTRG.26589.15 | -10.74221331 | 0.000237891 | down |
| MSTRG.42962.13 | -10.56097785 | 0.000251588 | down |
| MSTRG.64265.35 | -10.52411113 | 0.000263973 | down |
| MSTRG.108951.5 | -9.683808851 | 0.000277787 | down |
| MSTRG.77897.3 | -8.227122553 | 0.00039548 | down |
| MSTRG.126969.7 | -10.13307293 | 0.000439767 | down |
| MSTRG.105154.2 | -10.18394125 | 0.000490326 | down |
| MSTRG.142219.1 | -8.086665666 | 0.000514658 | down |
| MSTRG.224690.12 | -10.07416638 | 0.000576138 | down |
| MSTRG.100805.1 | -8.336016118 | 0.000579907 | down |
| MSTRG.16080.50 | -9.995620521 | 0.000627228 | down |
| MSTRG.81026.1 | -5.813114425 | 0.000658774 | down |
| MSTRG.187775.4 | -7.783363528 | 0.000747074 | down |
| MSTRG.12141.1 | -2.591033585 | 0.000786295 | down |
| MSTRG.162403.12 | -9.854819338 | 0.000795153 | down |
| MSTRG.216018.5 | -5.210315706 | 0.000922857 | down |
| MSTRG.161300.10 | -9.671162796 | 0.001025723 | down |
| MSTRG.112189.1 | -6.807533679 | 0.001135055 | down |
| MSTRG.85299.16 | -9.569723714 | 0.001193338 | down |
| MSTRG.105664.3 | -9.406140874 | 0.001289479 | down |
| MSTRG.85459.2 | -9.529763074 | 0.001294251 | down |
| MSTRG.98299.5 | -6.956942084 | 0.001357725 | down |
| MSTRG.73380.2 | -9.369581783 | 0.001361194 | down |
| MSTRG.131183.1 | -7.020847741 | 0.001396625 | down |
| MSTRG.221998.1 | -2.334536873 | 0.001402761 | down |
| MSTRG.54550.3 | -9.348863523 | 0.001403484 | down |
| MSTRG.40193.1 | -8.055053654 | 0.001426325 | down |
| MSTRG.218952.21 | -9.323602295 | 0.001456717 | down |
| MSTRG.17861.5 | -9.442383604 | 0.001472328 | down |
| MSTRG.177551.1 | -2.351191107 | 0.001476922 | down |
| MSTRG.192038.1 | -7.746189774 | 0.001562004 | down |
| MSTRG.64260.1 | -5.099889102 | 0.001593809 | down |
| MSTRG.12143.7 | -9.258443206 | 0.001602908 | down |
| MSTRG.2270.1 | -7.462756953 | 0.00161663 | down |
| MSTRG.84685.5 | -9.351324743 | 0.001646341 | down |
| MSTRG.143330.1 | -7.491628 | 0.00192833 | down |
| MSTRG.49993.37 | -9.223569704 | 0.001996732 | down |
| MSTRG.134581.9 | -9.123448839 | 0.00202379 | down |
| MSTRG.191344.1 | -1.751150248 | 0.002074226 | down |
| MSTRG.41375.17 | -3.099913686 | 0.002083127 | down |
| MSTRG.212977.1 | -2.479601185 | 0.00229114 | down |
| MSTRG.236144.1 | -9.154208185 | 0.002315659 | down |
| MSTRG.98408.1 | -5.973851899 | 0.002326984 | down |
| MSTRG.177550.1 | -1.662464186 | 0.002470963 | down |
| MSTRG.56234.1 | -7.5797025 | 0.002489493 | down |
| MSTRG.221202.6 | -6.021910702 | 0.002553383 | down |
| MSTRG.134038.6 | -9.072849735 | 0.002618618 | down |
| MSTRG.106112.2 | -9.075091628 | 0.002667972 | down |
| MSTRG.236485.1 | -2.031353427 | 0.002676723 | down |
| MSTRG.4240.1 | -7.424973578 | 0.00283226 | down |
| MSTRG.176214.1 | -2.208588069 | 0.002858873 | down |
| MSTRG.128565.1 | -5.381905738 | 0.003044292 | down |
| MSTRG.24253.3 | -8.93980243 | 0.003313972 | down |
| MSTRG.13052.1 | -8.941278187 | 0.003380344 | down |
| MSTRG.23199.6 | -5.976678131 | 0.003405144 | down |
| MSTRG.117826.3 | -4.686610677 | 0.00363372 | down |
| MSTRG.189047.9 | -4.019954232 | 0.003901097 | down |
| MSTRG.132883.1 | -3.372379103 | 0.00390179 | down |
| MSTRG.223839.3 | -5.036769354 | 0.004053889 | down |
| MSTRG.98723.1 | -8.8217416 | 0.004163266 | down |
| MSTRG.49801.2 | -3.717982816 | 0.004422363 | down |
| MSTRG.78814.8 | -8.767286732 | 0.004473056 | down |
| MSTRG.50536.5 | -8.672182937 | 0.004503192 | down |
| MSTRG.71484.4 | -8.647997288 | 0.004691751 | down |
| MSTRG.39051.8 | -8.606698883 | 0.005035325 | down |
| MSTRG.224610.3 | -5.641592226 | 0.005083478 | down |
| MSTRG.185823.16 | -8.686532646 | 0.005136316 | down |
| MSTRG.15792.3 | -2.571925074 | 0.005246565 | down |
| MSTRG.51997.3 | -8.676166627 | 0.005344296 | down |
| MSTRG.82736.1 | -2.316666648 | 0.005460098 | down |
| MSTRG.215949.2 | -8.660767783 | 0.005485947 | down |
| MSTRG.20862.2 | -5.61851107 | 0.00572246 | down |
| MSTRG.239627.36 | -8.509442127 | 0.005941266 | down |
| MSTRG.87754.1 | -1.668509222 | 0.00611976 | down |
| MSTRG.154149.1 | -3.613377341 | 0.006175313 | down |
| MSTRG.147315.18 | -3.485142938 | 0.006455155 | down |
| MSTRG.205821.2 | -3.833251913 | 0.006968871 | down |
| MSTRG.175653.26 | -8.408624014 | 0.007032994 | down |
| MSTRG.53824.5 | -6.897605219 | 0.00706725 | down |
| MSTRG.119134.2 | -8.347308313 | 0.007622564 | down |
| MSTRG.116420.1 | -8.444021401 | 0.007886698 | down |
| MSTRG.12663.5 | -8.413003013 | 0.008070004 | down |
| MSTRG.142081.6 | -8.425917175 | 0.008097872 | down |
| MSTRG.234628.1 | -8.40758104 | 0.008278553 | down |
| MSTRG.32248.14 | -8.270061943 | 0.008360997 | down |
| MSTRG.14634.1 | -8.370194707 | 0.008658515 | down |
| MSTRG.8662.7 | -8.206983896 | 0.009011734 | down |
| MSTRG.223296.5 | -5.006904947 | 0.009075756 | down |
| MSTRG.69562.1 | -8.19240883 | 0.009172801 | down |
| MSTRG.218562.5 | -8.19240883 | 0.009172801 | down |
| MSTRG.234627.1 | -8.31223404 | 0.009279401 | down |
| MSTRG.187871.2 | -8.31223404 | 0.009279401 | down |
| MSTRG.21343.1 | -1.847150818 | 0.00928606 | down |
| MSTRG.15891.3 | -8.290838755 | 0.009339293 | down |
| MSTRG.191981.1 | -6.070431059 | 0.009346029 | down |
| MSTRG.36405.1 | -8.292384732 | 0.009501256 | down |
| MSTRG.215363.1 | -3.779188874 | 0.009613382 | down |
| MSTRG.205734.6 | -6.533374155 | 0.009664041 | down |
| MSTRG.29708.1 | -1.677438274 | 0.009807679 | down |
| MSTRG.129542.1 | -8.251847854 | 0.009969515 | down |
| MSTRG.59023.3 | -8.101926541 | 0.010198312 | down |
| MSTRG.207708.1 | -8.23114429 | 0.010216735 | down |
| MSTRG.221686.1 | -1.602965938 | 0.010331248 | down |
| MSTRG.199350.1 | -8.210139399 | 0.010473269 | down |
| MSTRG.131283.1 | -8.210139399 | 0.010473269 | down |
| MSTRG.76700.6 | -8.188824281 | 0.010739593 | down |
| MSTRG.220123.1 | -8.188824281 | 0.010739593 | down |
| MSTRG.141057.1 | -4.906719425 | 0.010745179 | down |
| MSTRG.57033.1 | -8.145225744 | 0.011303673 | down |
| MSTRG.233384.1 | -2.66632604 | 0.011389702 | down |
| MSTRG.63846.1 | -3.274411798 | 0.01175881 | down |
| MSTRG.153141.5 | -8.100269029 | 0.011913448 | down |
| MSTRG.155287.6 | -8.077254391 | 0.012237037 | down |
| MSTRG.132914.1 | -2.048626621 | 0.012330731 | down |
| MSTRG.438.6 | -5.450534492 | 0.01277511 | down |
| MSTRG.236197.1 | -8.030093987 | 0.01292514 | down |
| MSTRG.199125.1 | -1.713865106 | 0.013026677 | down |
| MSTRG.71957.1 | -3.312911747 | 0.013266257 | down |
| MSTRG.130420.1 | -8.005923036 | 0.013291215 | down |
| MSTRG.191885.3 | -5.331911994 | 0.013298013 | down |
| MSTRG.32654.2 | -6.367100623 | 0.013828704 | down |
| MSTRG.2012.11 | -7.956331791 | 0.014071746 | down |
| MSTRG.41765.1 | -7.956331791 | 0.014071746 | down |
| MSTRG.129716.1 | -7.930882211 | 0.014488132 | down |
| MSTRG.96173.1 | -2.108406912 | 0.014638483 | down |
| MSTRG.150123.8 | -2.142450796 | 0.014834175 | down |
| MSTRG.234618.1 | -7.904975826 | 0.014923343 | down |
| MSTRG.55470.1 | -7.904975826 | 0.014923343 | down |
| MSTRG.181997.2 | -7.769098996 | 0.014943435 | down |
| MSTRG.17212.1 | -6.12602915 | 0.01495002 | down |
| MSTRG.166745.2 | -3.773594942 | 0.014986902 | down |
| MSTRG.66325.4 | -1.555292006 | 0.015233962 | down |
| MSTRG.220382.1 | -7.87859594 | 0.015378536 | down |
| MSTRG.236149.1 | -7.87859594 | 0.015378536 | down |
| MSTRG.69906.1 | -7.87859594 | 0.015378536 | down |
| MSTRG.118182.1 | -7.851724924 | 0.015854964 | down |
| MSTRG.161979.2 | -2.912799103 | 0.016043559 | down |
| MSTRG.18799.2 | -7.821492573 | 0.01609275 | down |
| MSTRG.159571.9 | -3.406771705 | 0.01622199 | down |
| MSTRG.44663.1 | -7.824344149 | 0.016353977 | down |
| MSTRG.36406.1 | -7.824344149 | 0.016353977 | down |
| MSTRG.236642.1 | -7.824344149 | 0.016353977 | down |
| MSTRG.54738.2 | -5.487259402 | 0.016483181 | down |
| MSTRG.148395.1 | -2.249174825 | 0.016668483 | down |
| MSTRG.183810.1 | -7.796433902 | 0.016877037 | down |
| MSTRG.64265.25 | -7.642534848 | 0.017216701 | down |
| MSTRG.130345.1 | -7.767973305 | 0.017425726 | down |
| MSTRG.234617.2 | -7.738940223 | 0.01800176 | down |
| MSTRG.20533.1 | -7.709311156 | 0.018607001 | down |
| MSTRG.234624.1 | -7.709311156 | 0.018607001 | down |
| MSTRG.198180.2 | -7.709311156 | 0.018607001 | down |
| MSTRG.15773.2 | -3.760259712 | 0.019005165 | down |
| MSTRG.57032.1 | -6.849587376 | 0.019090538 | down |
| MSTRG.97485.1 | -7.679061126 | 0.019243475 | down |
| MSTRG.130010.5 | -3.08938504 | 0.019297614 | down |
| MSTRG.186323.7 | -2.86130434 | 0.019496133 | down |
| MSTRG.215377.1 | -1.318602517 | 0.01951207 | down |
| MSTRG.214553.2 | -2.321958144 | 0.020070361 | down |
| MSTRG.207089.2 | -7.48384169 | 0.020440007 | down |
| MSTRG.23141.1 | -7.616590112 | 0.020619145 | down |
| MSTRG.124862.1 | -7.616590112 | 0.020619145 | down |
| MSTRG.129251.4 | -7.616590112 | 0.020619145 | down |
| MSTRG.180741.1 | -7.616590112 | 0.020619145 | down |
| MSTRG.50536.3 | -5.042059046 | 0.021450775 | down |
| MSTRG.110880.1 | -2.048660564 | 0.021841409 | down |
| MSTRG.109549.1 | -2.396999864 | 0.02237557 | down |
| MSTRG.123487.2 | -6.080892375 | 0.022431916 | down |
| MSTRG.57017.1 | -7.482863611 | 0.022813956 | down |
| MSTRG.80623.1 | -7.482863611 | 0.022813956 | down |
| MSTRG.137131.1 | -7.482863611 | 0.022813956 | down |
| MSTRG.164222.1 | -7.482863611 | 0.022813956 | down |
| MSTRG.234617.1 | -7.447392756 | 0.02317986 | down |
| MSTRG.212049.1 | -7.411027796 | 0.023559648 | down |
| MSTRG.102242.2 | -7.160550219 | 0.023564638 | down |
| MSTRG.74587.1 | -4.686961893 | 0.024298652 | down |
| MSTRG.90245.1 | -7.335426903 | 0.024364355 | down |
| MSTRG.149705.1 | -7.335426903 | 0.024364355 | down |
| MSTRG.129717.1 | -7.335426903 | 0.024364355 | down |
| MSTRG.2870.7 | -7.296087029 | 0.02479121 | down |
| MSTRG.106669.1 | -2.941129709 | 0.025231947 | down |
| MSTRG.9621.11 | -7.255644309 | 0.025235837 | down |
| MSTRG.136851.1 | -7.200991769 | 0.025509085 | down |
| MSTRG.169904.1 | -7.200991769 | 0.025509085 | down |
| MSTRG.154636.12 | -7.214035119 | 0.025699438 | down |
| MSTRG.199351.1 | -7.214035119 | 0.025699438 | down |
| MSTRG.98065.1 | -2.759858284 | 0.025857592 | down |
| MSTRG.222711.2 | -1.68598757 | 0.026116315 | down |
| MSTRG.219493.6 | -6.884559568 | 0.026491691 | down |
| MSTRG.203891.9 | -7.12703379 | 0.02668898 | down |
| MSTRG.34856.3 | -7.12703379 | 0.02668898 | down |
| MSTRG.157434.2 | -6.861343928 | 0.026749996 | down |
| MSTRG.161231.2 | -4.175247054 | 0.02677258 | down |
| MSTRG.185007.2 | -7.079718613 | 0.026882307 | down |
| MSTRG.175533.1 | -4.28783674 | 0.027143588 | down |
| MSTRG.117872.6 | -6.789367678 | 0.027562098 | down |
| MSTRG.59536.17 | -3.469282008 | 0.028705865 | down |
| MSTRG.236147.1 | -7.672163497 | 0.028926508 | down |
| MSTRG.23196.1 | -3.884525619 | 0.028959829 | down |
| MSTRG.33081.46 | -6.935509199 | 0.028963697 | down |
| MSTRG.108170.1 | -3.240504705 | 0.029277684 | down |
| MSTRG.126216.2 | -1.352368754 | 0.029633383 | down |
| MSTRG.66325.29 | -2.14663263 | 0.029956385 | down |
| MSTRG.63729.5 | -1.615157288 | 0.02997384 | down |
| MSTRG.121830.30 | -6.577778297 | 0.030048222 | down |
| MSTRG.190374.3 | -6.577778297 | 0.030048222 | down |
| MSTRG.174006.1 | -6.577778297 | 0.030048222 | down |
| MSTRG.46971.1 | -6.549006789 | 0.030397669 | down |
| MSTRG.132526.16 | -3.668864486 | 0.030502865 | down |
| MSTRG.98413.1 | -1.379805442 | 0.0306593 | down |
| MSTRG.129798.1 | -6.523568746 | 0.030719609 | down |
| MSTRG.72468.1 | -6.519649822 | 0.030757036 | down |
| MSTRG.99834.1 | -6.519649822 | 0.030757036 | down |
| MSTRG.98582.2 | -6.489683076 | 0.031126794 | down |
| MSTRG.192677.20 | -6.489683076 | 0.031126794 | down |
| MSTRG.111794.1 | -6.459080686 | 0.031507446 | down |
| MSTRG.22242.2 | -6.639292779 | 0.032310648 | down |
| MSTRG.130660.3 | -6.363186755 | 0.032507598 | down |
| MSTRG.194951.1 | -6.363186755 | 0.032507598 | down |
| MSTRG.12021.1 | -6.363186755 | 0.032507598 | down |
| MSTRG.194357.1 | -6.295529812 | 0.033117926 | down |
| MSTRG.231789.4 | -4.206664335 | 0.033239475 | down |
| MSTRG.148912.1 | -3.165791147 | 0.033439998 | down |
| MSTRG.33609.1 | -1.812128172 | 0.03398907 | down |
| MSTRG.195505.19 | -6.153782642 | 0.034442516 | down |
| MSTRG.101666.47 | -2.4665447 | 0.034467889 | down |
| MSTRG.77593.3 | -6.149885409 | 0.03446818 | down |
| MSTRG.80915.1 | -6.149885409 | 0.03446818 | down |
| MSTRG.103255.13 | -6.456494881 | 0.034559061 | down |
| MSTRG.36120.1 | -5.453087137 | 0.034683366 | down |
| MSTRG.46171.1 | -6.142388623 | 0.034774227 | down |
| MSTRG.107917.5 | -6.453644638 | 0.034981375 | down |
| MSTRG.42616.1 | -1.75380828 | 0.035452807 | down |
| MSTRG.5767.1 | -2.547863908 | 0.035986377 | down |
| MSTRG.21514.4 | -5.987875365 | 0.0360314 | down |
| MSTRG.142361.2 | -6.302988444 | 0.036476794 | down |
| MSTRG.125168.2 | -4.424896333 | 0.036572775 | down |
| MSTRG.197393.10 | -5.899497035 | 0.036912831 | down |
| MSTRG.161141.2 | -2.539761658 | 0.036998349 | down |
| MSTRG.19028.1 | -3.568649835 | 0.037310181 | down |
| MSTRG.197393.9 | -5.853191921 | 0.037383082 | down |
| MSTRG.69765.1 | -6.335357502 | 0.03740822 | down |
| MSTRG.137167.2 | -6.169949361 | 0.03741583 | down |
| MSTRG.109908.11 | -1.953774147 | 0.037997715 | down |
| MSTRG.85299.17 | -5.759739026 | 0.038362956 | down |
| MSTRG.13611.8 | -1.111541695 | 0.040072535 | down |
| MSTRG.175125.1 | -1.153280077 | 0.040114962 | down |
| MSTRG.222651.1 | -1.281577849 | 0.040318548 | down |
| MSTRG.191934.3 | -7.248347412 | 0.040929899 | down |
| MSTRG.137258.5 | -5.351897444 | 0.0428858 | down |
| MSTRG.237262.1 | -2.035178133 | 0.043380709 | down |
| MSTRG.228818.16 | -5.28371736 | 0.043699117 | down |
| MSTRG.185691.1 | -3.384795964 | 0.044079797 | down |
| MSTRG.84186.1 | -3.621638147 | 0.044420109 | down |
| MSTRG.177508.1 | -1.568326773 | 0.045439852 | down |
| MSTRG.142208.1 | -4.265524501 | 0.045609893 | down |
| MSTRG.132526.20 | -2.278407397 | 0.045921904 | down |
| MSTRG.191604.5 | -5.297762318 | 0.046072453 | down |
| MSTRG.133442.12 | -5.33477648 | 0.047151973 | down |
| MSTRG.205137.1 | -1.785467661 | 0.047372447 | down |
| MSTRG.45087.1 | -3.138450538 | 0.047604289 | down |
| MSTRG.58479.6 | -1.704028761 | 0.048386253 | down |
| MSTRG.134214.1 | -4.108228396 | 0.048625591 | down |
| MSTRG.177693.3 | -4.887862311 | 0.048808659 | down |
| MSTRG.233871.1 | -5.874320116 | 0.049492097 | down |
